# A species-level taxonomic review and host associations of *Glyptapanteles* (Hymenoptera, Braconidae, Microgastrinae) with an emphasis on 136 new reared species from Costa Rica and Ecuador

**DOI:** 10.3897/zookeys.890.35786

**Published:** 2019-11-20

**Authors:** Diana Carolina Arias-Penna, James B. Whitfield, Daniel H. Janzen, Winifred Hallwachs, Lee A. Dyer, M. Alex Smith, Paul D.N. Hebert, José L. Fernández-Triana

**Affiliations:** 1 Department of Entomology, University of Illinois, 320 Morrill Hall, 505 S. Goodwin Ave., Urbana, IL 61801, USA; 2 Department of Biology, University of Pennsylvania, 102 Leidy Laboratories, 433 S. University Ave., Philadelphia, PA 19104, USA; 3 Department of Biology, University of Pennsylvania, 3400 Chestnut St, Philadelphia, PA 19104, USA; 4 Department of Biology, University of Nevada, 1664 N. Virginia Street, Reno, NV 89557, USA; 5 Department of Integrative Biology, University of Guelph, 50 Stone Road East, Guelph, Ontario, N1G 2W1, Canada; 6 Biodiversity Institute of Ontario, University of Guelph, 579 Gordon St., Guelph, Ontario, N1G 1Y2, Canada; 7 Canadian National Collection of Insects, Agriculture and Agri-Food Canada, 960 Carling Avenue, Ottawa, Ontario, K1A 0C6, Canada

**Keywords:** Central America, diversity, mtDNA, natural history, parasitoid wasps, South America

## Abstract

The descriptive taxonomic study reported here is focused on *Glyptapanteles*, a species-rich genus of hymenopteran parasitoid wasps. The species were found within the framework of two independent long-term Neotropical caterpillar rearing projects: northwestern Costa Rica (Área de Conservación Guanacaste, ACG) and eastern Andes, Ecuador (centered on Yanayacu Biological Station, YBS). One hundred thirty-six new species of *Glyptapanteles* Ashmead are described and all of them are authored by Arias-Penna. None of them was recorded in both countries; thus, 78 are from Costa Rica and the remaining 58 from Ecuador. Before this revision, the number of Neotropical described *Glyptapanteles* did not reach double digits. Reasonable boundaries among species were generated by integrating three datasets: Cytochrome Oxidase I (COI) gene sequencing data, natural history (host records), and external morphological characters. Each species description is accompanied by images and known geographical distribution. Characteristics such as shape, ornamentation, and location of spun *Glyptapanteles* cocoons were imaged as well. Host-parasitoid associations and food plants are also here published for the first time. A total of 88 species within 84 genera in 15 Lepidoptera families was encountered as hosts in the field. With respect to food plants, these wild-caught parasitized caterpillars were reared on leaves of 147 species within 118 genera in 60 families. The majority of *Glyptapanteles* species appeared to be relatively specialized on one family of Lepidoptera or even on some much lower level of taxonomic refinement. Those herbivores in turn are highly food-plant specialized, and once caterpillars were collected, early instars (1–3) yielded more parasitoids than later instars. *Glyptapantelesjimmilleri* Arias-Penna, ** sp. nov.** is the first egg-larval parasitoid recorded within the genus, though there may be many more since such natural history requires a more focused collection of eggs. The rate of hyperparasitoidism within the genus was approximately 4% and was represented by *Mesochorus* spp. (Ichneumonidae). A single case of multiparasitoidism was reported, *Copidosomafloridanum* Ashmead (Encyrtidae) and *Glyptapantelesilarisaaksjarvi* Arias-Penna, ** sp. nov.** both parasitoid species emerged from the caterpillar of Noctuidae: *Condicacupienta* (Cramer). Bodyguard behavior was observed in two *Glyptapanteles* species: *G.howelldalyi* Arias-Penna, ** sp. nov.** and *G.paulhansoni* Arias-Penna, ** sp. nov.** A dichotomous key for all the new species is provided. The numerous species described here, and an equal number already reared but not formally described, signal a far greater *Glyptapanteles* species richness in the Neotropics than suggested by the few described previously.

## Introduction

Bees, wasps, and ants are the most widely recognized insects among the Hymenoptera, one of the four largest (megadiverse) insect orders. In terms of species richness, parasitoid wasps numerically dominate the order (Quicke 1997). Within hymenopteran parasitoids, Microgastrinae is among the most commonly collected groups and one of the two most species-rich subfamilies of Braconidae (the other being Braconinae; Jones et al. 2009, Rodriguez et al. 2012, Quicke 2015). The subfamily, worldwide, contains 81 extant genera comprising nearly 2,600 to 2,700 described species (Fernández-Triana and Ward 2018, Fernández-Triana and Boudreault 2018, Whitfield et al. 2018). Although some major geographical areas have not yet been heavily explored, recent extrapolations based on both field studies and known faunas estimate Microgastrinae species richness worldwide to be 17,000 to 46,000+ species (Rodriguez et al. 2012). All known microgastrines are obligate endoparasitoids of larval Lepidoptera (henceforth, caterpillars) (Quicke 2015), and practically every Lepidoptera higher taxon is used as a host, making the subfamily the single most important group of Hymenoptera parasitoids specialized on attacking caterpillars. Around 100 species of Microgastrinae have been used in biological control of Lepidoptera pests (Whitfield 1995).

During the 21^st^ Century, significant progress has been made with the taxonomy and biology of the subfamily, mainly in the Neotropics. Over the last decade, some Microgastrinae genera have been targets of taxonomic revisions, descriptions of new genera, and new species descriptions. Taxonomic revisions have included *Apanteles* sensu stricto Förster (Fernández-Triana et al. 2014a), *Distatrix* Mason (Grinter et al. 2009), *Exoryza* Mason (Fernández-Triana et al. 2016a), *Hypomicrogaster* Ashmead (Valerio and Whitfield 2015), *Iconella* Mason (Fernández-Triana et al. 2013), *Microplitis* Förster and *Snellenius* Westwood (Fernández-Triana et al. 2015), *Parapanteles* Ashmead (Valerio et al. 2009), *Prasmodon* Nixon (Fernández-Triana et al. 2014b), *Promicrogaster* (Fernández-Triana et al. 2016b), *Sendaphne* Nixon (Fernández-Triana et al. 2014d), *Venanus* Mason (Whitfield et al. 2011, Fernández-Triana et al. 2014c), and *Wilkinsonellus* Mason (Arias-Penna et al. 2013). Descriptions of new genera have included *Mariapanteles* Whitfield & Fernández-Triana (Whitfield et al. 2012) and *Janhalacaste* Fernández-Triana (Fernández-Triana and Boudreault 2018). Descriptions of new species have included *Austrocotesia* Austin & Dangerfield (Valerio and Whitfield 2005), *Glyptapanteles* Ashmead (Whitfield et al. 2002a), *Janhalacaste* Fernández-Triana (Fernández-Triana and Boudreault 2018), and *Prasmodon* Nixon (Valerio et al. 2005). Notwithstanding these efforts, the extreme species richness harbored in the Neotropics means that both taxonomic and biological records are still highly incomplete (Whitfield et al. 2002a and the two inventories referenced here).

*Glyptapanteles* are small parasitoid wasps that occur in all faunal regions, and recent field sampling from Neotropical countries suggests that the genus is among the three most diverse within Microgastrinae (the other two being *Apanteles* Förster and *Diolcogaster* Ashmead; Whitfield et al. 2018). Worldwide *Glyptapanteles* species descriptions correspond to specimens scattered around the world but have not been part of place-based revisions [e.g., Australia (Austin and Dangerfield 1992), Ecuador (Whitfield et al. 2002a), China (Chen and Song 2004), Greece (Papp 2007), Croatia, Bosnia and Macedonia (Papp 2009), India (Gupta and Pereira 2012, Gupta and Fernández-Triana 2014, Gupta et al. 2016b)]. In tropical and subtropical regions, *Glyptapanteles* is one of the omnipresent genera (Whitfield 1995). Approximately 1,000 or more species have been estimated for the tropics (Mason 1981). In the Palearctic, the genus is abundant mainly in cool, humid and, warm ecosystems and few species have been reported from dry ecosystems (Mason 1981). In the Neotropics, *Glyptapanteles* is especially species-rich in lowland regions (Whitfield et al. 2009).

Currently, more than 122 species are described worldwide (Yu et al. 2016, Gupta et al. 2016b), of which six, prior to this revision, are reported from the Neotropics: *G.agrotivorus* Whitfield, *G.bourquini* (Blanchard), *G.ecuadorius* Whitfield, *G.herbertii* (Ashmead), *G.militaris* (Walsh), and *G.muesebecki* (Blanchard). They are primarily agricultural species (some of them probably not native), that attack mainly noctuids. Whitfield et al. (2002a) offer a key, species notes, and a plate of cocoon photos, which should help discriminate these six species. This small number has made it possible to describe an enormous number of Neotropical *Glyptapanteles* without fear of creating synonyms. Before this study, a comprehensive revision for a portion of the Neotropics had not been attempted. It is clear that hundreds if not thousands of species of *Glyptapanteles* remain undescribed.

Some character-states and distribution data that might be able to help with the identification of these six Neotropical species are specified below. *Glyptapantelesagrotivorus* has the petiole on T1 distally with lateral margins curved (convex); the tegula is dark brown; the cocoons are white, loosely spun in a cluster with much loose silk (Whitfield et al. 2002a); and it is so far known to be distributed in Ecuador (Chimborazo province: San Pedro de Riobamba, San Antonio at 2,770 m, Whitfield et al. 2002a). *Glyptapantelesbourquini* has the fore wing with outer side of junction of r and 2RS veins forming a distinct stub, the r vein is much longer than the 2Rs vein; the petiole on T1 is somewhat sculptured and rounded distally; the median area on T2 often is not well demarcated from the third medioapically; the cocoon mass is typically tightly spun together, occasionally looser, cocoons woolly, the coloration varies from yellowish brown to orangish or pinkish (Whitfield et al. 2002a); and it is distributed in Argentina (Buenos Aires province: Alberdi [Juan Bautista], Blanchard 1936; and La Pampa province: departments of Capital, Maracó and Trenel, Baudino 2005); Ecuador (Chimborazo province: San Pedro de Riobamba, San Antonio at 2,770 m); Chile and Uruguay (Whitfield et al. 2002a). Probably it is found throughout South America (Whitfield et al. 2002a). *Glyptapantelesecuadorius* has the petiole on T1 smooth and relatively polished throughout; the median area on T2 is approximately twice as broad distally as long medially, often the central part is raised slightly, so that it may superficially appear less broad; the cocoons of this species are unknown; and it is distributed in Ecuador (Chimborazo province: San Pedro de Riobamba, Bilbao at 2,000 m; Whitfield et al. 2002a). *Glyptapantelesherbertii* has the petiole on T1 usually more evenly narrowing from base to apex and with some distinct, but often very fine punctation in distal half, not highly polished apically; the cocoon mass coloration is yellowish brown, elongate, and with cocoons arranged and stacked like two rows of cordwood; and its distribution appears to be circum-Caribbean (Belize, Colombia, Cuba, Florida, Mexico, Nicaragua, and Venezuela), but is also found as far south in South America as Peru and Argentina (Whitfield et al. 2002a). *Glyptapantelesmilitaris* has the hind coxa predominantly, typically entirely bright yellowish in color, as is the tegula; the cocoons are loosely spun together or near the host caterpillar’s body, and the coloration is white to light yellowish beige; and it is widely distributed throughout North America, but in the neotropics it is at least found in the Caribbean Region (Whitfield et al. 2002a). *Glyptapantelesmuesebecki* has the petiole on T1 as least 1.5× as long as anteriorly broad, and evenly narrowing posteriorly, with lateral margins relatively straight; the tegula is pale yellowish brown; the cocoons are yellowish brown, spun in a loose mass; and it is distributed in Argentina and Paraguay (Whitfield et al. 2002a).

### Biology

Like other microgastrines, adult *Glyptapanteles* are free-living wasps that feed primarily on nectar, pollen, or secretions from scales and aphids (Landis et al. 2000), while larvae develop inside caterpillars. The female wasp penetrates the host cuticle with her ovipositor and oviposits eggs, which float freely in the hemolymph. Independent of the number of eggs deposited, in solitary parasitoid species only one larva completes development, whereas, in gregarious parasitoid species, more than one offspring successfully completes development (Hanson and Gauld 2006). All *Glyptapanteles* are endoparasitoid koinobionts, meaning that their hosts continue to develop after being attacked. Inside the host, the egg absorbs proteins through a specialized extra-embryonic membrane before hatching (Jervis et al. 2001, 2008). The wasp larvae develop by consuming only the hemolymph and fat body of the host (Shaw and Huddleston 1991). The host eventually dies, although usually not until the parasitoid larva or larvae have emerged through the host cuticle. At the end its second larval instar, each parasitoid larva emerges by burrowing through its host’s cuticle (Godfray 1994). Outside the host, the parasitoids molt to their third and final larval instar, then spin individual cocoons and pupate in the cocoon, on the outer surface of the host or nearby (Fig. 4; Calkins and Sutter 1976).

This ancient physiological interaction of microgastrine endoparasitoids with their hosts is in part mediated by a fascinating mutualistic association with polydnaviruses (PDVs), an alliance that arose 73±11 mya (Whitfield 2002, though an older origin, ~100 mya, is proposed by Murphy et al. 2008). These double-stranded DNA viruses are integrated into the genome (proviral DNA) of both male and female wasps. However, the largest fragment is excised from the female wasp genome only during her later pupal and adult stages. Thus, PDVs are only “free-living” during reproduction in wasp ovarian calyx tissue or after injection into host hemocoel’s (D’Amico and Slavicek 2012). Once in the larval host, PDVs, in concert with other injected material such as toxins and ovarian fluids, play a crucial role in the survival of the developing egg (Lapointe et al. 2007) by suppressing or misdirecting host immune systems and arresting host development.

Biologically, prior to the two inventories referenced here, *Glyptapanteles* was still poorly known in the Neotropics. Those host records were mainly restricted to Noctuidae, Geometridae, Pieridae, Notodontidae, and Megalopygidae. Noctuid hosts included *Agrotisdeprivata* Walker feeding on *Brassicaoleracea* [wild cabbage, Brassicaceae], *Medicagosativa* [Alfalfa, Fabaceae], *Viciavillosa* [hairy vetch, Fabaceae], and *Zeamays* [corn, Poaceae]; *A.gypaetina* Guenée and *A.ipsilon* (Hufnagel) feeding on *Brassicaoleracea*, *Daucuscarota* [wild carrot, Apiaceae], *Helianthusannuus* [sunflower, Asteraceae], *Lactucasativa* [lettuce, Asteraceae], *Medicagosativa*, and *Trifoliumrepens* [white clover, Fabaceae]; *Peridromasaucia* (Hübner) feeding on *Trifoliumrepens* and *Medicagosativa*; *Helicoverpazea* (Boddie) feeding on *Zeamays* (Corn, Poaceae); and *Anticarsiagemmatalis* (Hübner), *Mythimnaunipuncta* (Haworth), *Peridromamargaritasa* (Haworth), *Pseudoplusiaincludens* (Walker), and *Trichoplusiani* (Hübner) for which the food plants were not reported (Blanchard 1936, Whitfield et al. 2002a, Baudino 2005). Geometridae hosts included *Thyrinteinaleucocerae* (Rindge) feeding on *Psidiumguajava* [guava, Myrtaceae] and *Eucalyptusgrandisand* [eucalyptus, Myrtaceae] (Grosman et al. 2008); *Cyclomiamopsaria* Guenée, *Glena* sp., and *Physocleora* sp. feeding on *Erythroxylummicrophyllum* (Erythroxylaceae) (Marconato et al. 2008). Pieridae host was *Dismorphiacrisialubina* (Blater) feeding on *Ingamortoniana* (Fabacae, Koptur 1989). Notodontidae host includes *Nystaleanyseus* (Cramer) (Whitfield et al. 2002a). One host genus from Megalopygidae has been reported (*Plodia* Guenée, sp.?) feeding on *Ingadensiflora* (Koptur 1989). Six additional lepidopteran host families also have been reported: Apatelodidae, Erebidae, Limacodidae, Nymphalidae, Pyralidae, and Saturniidae, although no species names were referenced (Whitfield et al. 2009).

### History of *Glyptapanteles* classification

*Glyptapanteles* was described in 1904 (Ashmead 1904), but its legitimacy as a distinct genus has only been accepted since 1981. Ashmead segregated it from *Apanteles* and erected the genus based on two females and one male collected from Manila, Philippines (Ashmead 1904). The description of *Glyptapanteles* coincided with an epoch of proliferation of descriptions of new genera within Microgastrinae in an attempt to subdivide the gigantic genus *Apanteles*. The advent of these newly described taxa only increased taxonomic confusion within the subfamily, due in large part to the fact that the worldwide fauna was both taxonomically not well-studied and the species descriptions were inadequate to enable taxonomists to categorize morphospecies among the plethora of new species encountered. Consequently, contemporary colleagues found it difficult to interpret those classifications and opted to ignore them.

In an attempt to stop this rapid increase in the production of generic names while also stabilizing the nomenclature, Muesebeck (1920, 1922) proposed a conservative approach and synonymized many genera, subsuming *Glyptapanteles* again into *Apanteles* (Whitfield et al. 2002b). Later, Nixon (1965, 1973) offered a reclassification of Microgastrinae, recognizing only one tribe (Microgasterini) with 19 genera, and *Glyptapanteles* was not considered to be a valid genus. Nixon kept a broad definition of *Apanteles*, dividing it into 44 species groups and suggesting that the genus ultimately should be split into more genera. Sixteen years later, Mason (1981) succeeded in subdividing the gigantic genus *Apanteles* and proposed a radical generic classification of the subfamily based on re-grouping of existing species groups, in which *Glyptapanteles*, one of the larger segregates of the *Apanteles*, was recognized as a valid genus in the tribe Cotesiini (Nixon’s Microgasterini therefore, was discarded). Mason divided Microgastrinae into five tribes (Apantelini, Cotesiini, Forniciini, Microgastrini, and Microplitini) based mainly on the association between female genitalic structures and host use. In his system, females that attack Microlepidoptera larvae possess long ovipositors, presumably an adaptation for reaching the small and cryptic Microlepidoptera caterpillars that feed in semi-concealed situations such as leaf rolls or silk webs. In contrast, females with short ovipositors attack Macrolepidoptera larvae, which are large caterpillars and live fully exposed on vegetation throughout their larval stages (Shaw and Huddleston 1991). In his reclassification, Mason assigned *Glyptapanteles* to the “Macrolepidoptera-attacking” Microgastrinae. However, host data now known for many genera have not substantiated Mason’s hypothesized associations, at least at the generic level; as a result, the tribal classification for the subfamily has been largely abandoned and the current classification is now based on genera and not tribes (Valerio et al. 2009, Whitfield et al. 2018).

Issues concerning *Glyptapanteles* taxonomy have partly contributed to the poor documentation of its natural history. Characteristics such as high incidence of morphological convergence and lack of obvious discrete morphological variation (character reduction) within both the genus and subfamily, together hinder or virtually preclude straightforward morphological identification of specimens (Shaw and Huddleston 1991). Other factors that possibly have contributed to the poor taxonomic state and its avoidance by taxonomists include its previously mentioned astonishing diversity, total lack of striking coloration (e.g., mainly black or dark brown), and the minute body size of its specimens (2–3 mm long). Taxonomic groups with small body sizes tend to be described much later than taxa with large body sizes (Jones et al. 2009). The poor understanding of *Glyptapanteles* diversity in the tropics could also be a consequence of other factors such as specialized ecological niches, relatively small population sizes, and the concealed parasitoid life history (Stireman et al. 2009).

### Demarcation of *Glyptapanteles*

As currently delineated, *Glyptapanteles* is the result of the fusion of several *Apanteles* species groups. Mason (1981) transferred seven *Apanteles* species groups from Nixon’s classification (1965, 1973) to the newly established *Glyptapanteles*. Those species groups were: the *octonarius* group, *siderion* group, *vitripennis* group, and the four monotypics *demeter* group, *fraternus* group, *pallipes* group, and *triangulator* group. The first thing that stands out in this classification is the pattern of reliance on the external morphological characters used. Those species groups were characterized not by any exclusive feature, but by the combination of features. Thus, the high level of homoplasy makes *Glyptapanteles* a genus with boundaries poorly defined and taxonomically difficult among Microgastrinae. Additionally, these species groups were based mainly on the north-western European fauna instead of the Neotropical fauna. The traits-per-group proposed by Nixon are listed below.

The *octonarius* group was distinguished mainly by the weak and even curvature at the junction of the r and 2RS veins on fore wing (e.g., *G.ronaldzunigai*, Figs 193L, 194K), whereas the remaining groups have that junction distinctly angled (e.g., *G.diegocamposi*, Fig. 70K), it is distributed worldwide (Nixon 1973), and the vast majority of species reassigned by Mason to *Glyptapanteles* belong mainly to here. In the *siderion* group, the petiole shows a wide base and a very narrow apex, the propodeum is short and has a well-defined median longitudinal carina, the phragma of the scutellum is hidden, the inner side of the distal margin of the hind tibia has a dense silky fringe of setae that is entirely differentiated from the normal tibial setae, and it is distributed in Java and the Philippines (Nixon 1965). In the *vitripennis* group, the phragma of the scutellum is always visible, the metanotum is always without a lateral, forward-pointing projection, proximally the median area on T2 is extensively polished, otherwise usually with at least some minute striae or punctation laterally, the inner spur of the hind tibia is longer than the outer spur, reaching to middle of hind basitarsus, and it is distributed worldwide (Nixon 1965, 1973). Specimens belonging to the *demeter* group have a very short and thick antenna, only the proximal and the distal antennal flagellomeres are longer than wide, the phragma of the scutellum is concealed, the propodeum is coarsely rugose, and it is distributed in New Zealand (Nixon 1965). In the *fraternus* group, the median area on T2 is polished, the inner spur of the hind tibia is slightly longer than the outer one, not reaching beyond middle of hind basitarsus, and it is distributed widely in the northwest of Europe (Nixon 1973). In the *pallipes* group, the propodeum is rugose all over and possesses a strong median longitudinal carina, the pronotum lacks a dorsal furrow, the petiole is twice as long as wide, the phragma of the scutellum is widely visible, and it is distributed in the northwest of Europe and widely in North America (Nixon 1965, 1973). In the *triangulator* group, the median area on T2 can be polished or with minute punctation, the edges on the median area lose definition distally, but have weak longitudinal sculpture, the petiole has parallel sides, but is distally rounded, the phragma of scutellum is narrowly visible, the propodeum is strongly shining, almost unsculptured, except toward distal corners, both spurs of the hind tibia are short and subequal in length, the outer side of hind tibia has dense strong spines, and it is distributed in France, Germany, and England (Nixon 1973). As mentioned previously, it became clear that the overlap of external morphological characters among species group makes the identification of the *Glyptapanteles* species more doubtful and problematic.

### *Glyptapanteles* and its resemblance to five genera within the Microgastrinae subfamily

Complications in the definition of *Glyptapanteles* also arise because a suite of external morphological characters that had been used for distinguishing them from the rest of Microgastrinae was later found to be shared with other genera. Currently, five genera within the subfamily have often been confused with *Glyptapanteles*; these are *Cotesia* Cameron, *Distatrix* Mason, *Lathrapanteles* Williams, *Protapanteles* Ashmead, and *Sathon* Mason. All five genera share: the hypopygium is evenly sclerotized from side to side and the fore wing with second r-m vein absent, so that the small areolet (second submarginal cell) is open distally (Mason 1981, Whitfield 1997). The less than fully diagnostic characters that complicate the separation of *Glyptapanteles* with those five genera are outlined here below.

***Glyptapanteles* and *Cotesia*.** In *Cotesia* the petiole is virtually never narrower at its apex, the usual shape is a little longer than wide and broadened distally, but occasionally it can be wider than long or somewhat barrel-shaped (Mason 1981) or unusual narrowing at midlength (Gupta et al. 2016a); frequently the petiole on T1 is smooth proximally, but distally always with sculpture; the shape of the median area on T2 is variable, usually subrectangular, but it can be truncated trapezoidal or semicircular; and the propodeum is rugose, usually with a median longitudinal carina that may be partially obscured by rugosity and with an incomplete transverse carina laterally separating the rugose declivity from a smoother proximal area (Mason 1981). *Cotesia* tends to be a more dominant faunal component in temperate regions worldwide (Whitfield et al. 2009), but in the tropics, *Cotesia* is displaced ecologically by *Glyptapanteles* (Mason 1981). Some Neotropical *Glyptapanteles* collected at high elevations (> 1,000 m) seem to share morphological similarities present in Holarctic *Cotesia* [e.g., propodeum rugose (as in *G.erictepei*, Figs 80G, 81F; *G.felipesotoi*, Figs 82F, 83C) and petiole barrel-shape (as in *G.marcpolleti*, Fig. 151G, H)]. Some *Glyptapanteles* in the Australasian region exhibit coarsely rugose tergites, and sometimes they are confused with *Cotesia*. However, both genera can be separated by the shape of petiole on T1 and the median area on T2 (Austin and Dangerfield 1992).

***Glyptapanteles* and *Distatrix*.***Distatrix* is an unusual genus, with coloration partly xanthic (brownish yellow), large eyes (sometimes only in one sex), pedunculate cocoons, and is relatively rare in collections (Whitfield and Scaccia 1996, Whitfield et al. 2009). *Distatrix* possesses the following characteristics: the propodeum is smooth and weakly curved, sometimes with enlarged spiracles; the pronotum only with the ventral furrow; the inner spur on the hind tibia is much longer than half length of the hind basitarsus; the females of some species have an enlarged seta on the fore telotarsus (fifth tarsomere, as *Protapanteles*); the vannal lobe on the hind wing with the margin straight or concave; the petiole parallel-sided and rounded distally or weakly narrow distally, the petiole can be smooth or with weak sculpture; and the median area on T2 smooth with lateral grooves poorly defined distally. *Glyptapanteles* differs from *Distatrix* in having the petiole more strongly narrowed distally, the margins of the median area on T2 are better defined, and the ovipositor sheath exhibits normal setae distally (Whitfield et al. 2009). Again, these characteristics are also found in some Neotropical *Glyptapanteles*.

***Glyptapanteles* and *Lathrapanteles*.***Lathrapanteles* was separated from *Sathon* by Williams (1985), who placed four species of *Sathon* in this new genus: three from the Northeastern United States and one from South America. He was aware that *Lathrapanteles* was not a natural group and he realized the difficulties of separating both genera by one or a few external morphological characters. Some of the characteristics that define the genus, but are also shared with *Glyptapanteles* are: the pronotum with both dorsal and ventral furrows (e.g., *G.andysuarezi*, Fig. 19A, E), the metanotum reduced and without sublateral setose lobes, the phragma of the scutellum exposed (e.g., *G.ianyarrowi*, Fig. 107B, C), the propodeum weakly or strongly convex with median longitudinal carina or groove, the petiole evenly narrowed to apex or parallel-sided for the proximal 0.75 or slightly barrel-shaped, and the edges of the median area on T2 defined by grooves or obscured by rugosity (e.g., *G.linghsiuae*, Fig. 141D, G) (Williams 1985).

***Glyptapanteles* and *Protapanteles*.***Protapanteles* shares with *Glyptapanteles* a weakly sculptured propodeum and relatively trapezoidal median area on T2 (Whitfield et al. 2009). However, the most frequent characteristic used to distinguish the two genera is the petiole shape: parallel-sided (3/4 proximal or more) and then strongly narrowing at the apex, or sides gradually converging distally for *Glyptapanteles*, while for *Protapanteles* the petiole is parallel-sided throughout except for a strongly rounded apex (Mason 1981). Additional traits ostensibly exclusive to *Protapanteles* are: the pronotum with two distinctive furrows, one dorsal and other ventral; in females, the fore telotarsus usually with a conspicuous lateroventral curved seta and a weak distal excavation; the larval mandible with a row of 12 or fewer large teeth concentrated distally on the blade; and its distribution which is almost completely confined to the Holarctic Region (Mason 1981). However, Neotropical *Glyptapanteles* here described exhibit petioles with an extensive array of shapes ranging from barrel-shaped with apex rounded/truncate (e.g., *G.phildevriesi*, Fig. 187G, H; *G.rafamanitioi*, Fig. 190H, I) to petioles with broad base to a very narrow apex (e.g., *G.pamitchellae*, Fig. 178G, H; *G.scottshawi*, Fig. 199G, H). Some *Glyptapanteles* species also have a pronotum with both dorsal and ventral furrows (e.g., *G.andywarreni*, Fig. 20A, C, I; *G.markshawi*, Fig. 154A, E), and in some females a fore telotarsus with a curved seta can be found (e.g., *G.boharti*, Fig. 37A, E), although sometimes it is difficult to see the seta due to the small body size of some specimens. Recently, an earlier perspective has been resurrected that *Glyptapanteles* and *Sathon* should be part of an expanded *Protapanteles* (van Achterberg 2003, Fernández-Triana 2010). The three genera, *Glyptapanteles*, *Protapanteles* and *Sathon*, share the following traits: median area on T2 clearly delimited by a pair of submedial grooves, females with hypopygium evenly sclerotized and ovipositor sheath usually short (van Achterberg 2003), the anterior furrow of metanotum is glabrous and flattened (without sublateral lobe), and the phragma of scutellum exposed (Mason 1981). On the other hand, *Protapanteles* is in many respects intermediate morphologically between *Cotesia* and *Glyptapanteles*. It shares a quadrate petiole with *Cotesia*, and a weakly sculptured propodeum and more trapezoidal median area on T2 with *Glyptapanteles* (Whitfield et al. 2009).

***Glyptapanteles* and *Sathon*.** As currently defined, *Sathon* resembles *Glyptapanteles* in nearly all features except ovipositor length: short for *Glyptapanteles* and long for *Sathon*. However, Neotropical *Glyptapanteles* exhibit ovipositors with a broad length spectrum, ranging from short (e.g., *G.sydneycameronae*, Fig. 212A, J, and *G.victoriapookae*, Fig. 219A, J) to long (e.g., *G.alejandrovalerioi*, Fig. 5A, G and *G.alvarowillei*, Fig. 10A, G, I). Additionally, Mason (1981) proposed *Sathon* as a new genus based upon the distinctive large external genitalia in males; yet again some Neotropical *Glyptapanteles* males also bear prominent genitalia (e.g., *G.andybennetti*, Fig. 15A, F, K and *G.andydeansi*, Fig. 17A, E, J). It has been suggested that *Sathon* probably should be subsumed within *Glyptapanteles* in the near future (Whitfield et al. 2009).

The two basic philosophical approaches to classification have always generated substantial controversy in taxonomy. The question is whether to divide (“split”) or to merge (“lump”) specific taxa. On the one hand, there are those who prefer a large number of small taxa, stressing diagnostic differences but on the other, there are those who support that it is better to recognize a relatively small number of taxa, emphasizing broader relationships. As it has been pointed out, the subfamily Microgastrinae has experienced taxonomic chaos during its history due to varied decisions taken in the past. Several genera within Microgastrinae are confused with *Glyptapanteles*, so it would be a premature decision to deal with potential synonyms here. A reclassification now would seem untimely and thus, a generic reclassification of *Glyptapanteles* must wait until more data have been accumulated. Currently, Hybrid Anchored Enrichment and Ultraconserved Elements are being used in order to revise the phylogeny of Microgastrinae. Those two approaches may hopefully provide a better understanding and more justified arguments to the synonymy of some genera in Microgastrinae.

The main objective of this paper is to describe for the first time a large array of Neotropical *Glyptapanteles* species based on the extensive material that is available from two large-scale rearing projects, one in Costa Rica and the other in Ecuador. This taxonomic revision incorporates morphology, DNA sequences (COI gene), and an extensive base of natural history knowledge. Additionally, a morphological image library and a dichotomous key are provided to facilitate species identification. This is not meant to be a full revision of the genus but instead is intended as a significant starting point for understanding their Neotropical biodiversity and as a guide for future research.

## Materials and methods

### Sampling

A robust intraspecific analysis benefits from a large quantity of specimens collected across its distributional range. The primary taxon sampling of *Glyptapanteles* for this study derives from two independent long-term rearing projects: the Caterpillar and Parasitoid Inventory of the Área de Conservación Guanacaste (ACG) in northwestern Costa Rica (http://www.acguanacaste.ac.cr, http://janzen.sas.upenn.edu) and the project Caterpillars and Parasitoids of the Eastern Andes (CAPEA) in Ecuador (www.caterpillars.org). These two Neotropical countries have high species richness and what appears to be endemism in the face of their neighboring countries being poorly studied for their Microgastrinae biodiversity.

The ACG project began in 1978; initially, samples were collected exclusively in dry forest on the small area of Santa Rosa National Park (SRNP). By the end of the 1980’s the sampling was expanded eastward and upward into the rain forest and cloud forest. Currently, the sampling covers a wide altitudinal range from 90 m to 2,000 m (Janzen et al. 2009, Janzen and Hallwachs 2016b). In 2003, the project incorporated DNA barcoding, a microgenomic identification system, by which species can be identified and usually discriminated through the analysis of a small segment of the genome: the mitochondrial gene cytochrome *c* oxidase I (COI). The CAPEA project was more recently started in 2001 and participants have collected and reared caterpillars at Yanayacu (black river in the Kichwa language), a biological station in the Quijos Valley, Napo Province, in the Andes of northeastern Ecuador (Miller and Dyer 2009). A variety of ecosystems have been sampled such as paramo, montane wet forest, cloud forest, and mid-elevation rain forest ranging from 3,800 m down to 100 m (Dyer et al. 2012).

In both projects, caterpillars were collected directly in the field and subsequently reared in “laboratory” conditions (partly enclosed rearing barn). Voucher specimens of the food plants also were collected for taxonomic identification. Plant vouchers for the CAPEA project were deposited at the Museo Ecuatoriano de Ciencias Naturales (Quito, Ecuador). Rearing took place individually for each host caterpillar in clear plastic bags, bottles, jars, or plastic cups in an open-air shelter with ambient temperature, humidity, and natural day length. Larvae were fed with fresh excised foliage of the food-plant species on which the caterpillar was collected and placed in containers as needed. Larvae were inspected daily to record stage of development, parasitoid emergence, or simply to remove frass. Each caterpillar of ACG was tagged with a voucher code which refers to the event-based record of finding the caterpillar and rearing it: yy-SRNP-xxxxxxx e.g., 90-SRNP-1146. The prefix refers to the last two digits of the year that caterpillar was discovered in the field. The acronym SRNP stands for Santa Rosa National Park, and the suffix is a unique number assigned within the year. When a solitary parasitoid emerged from its host, the same caterpillar voucher code was assigned at that time, but also a unique DNA wasp voucher code later was assigned for any further study of that specimen: DHJPARxxxxxxx (e.g., DHJPAR0001443, DHJ = Daniel Hunt Janzen and PAR = parasitoid) (Janzen et al. 2009, Janzen and Hallwachs 2016a, Janzen and Hallwachs 2016b). In gregarious samples only one wasp was selected to DNA barcoded and received a unique DHJPARxxxxxx code; however, the yy-SRNP-xxxxx code is retained by the unique one as well as all specimens reared from the same caterpillar sample. In the CAPEA project, the voucher code for each caterpillar collected was labeled as ECxxxx. EC stands for Ecuador and the suffix is a unique number assigned to each sample according to a list of consecutive numbers. The DNA wasp voucher code for each parasitoid wasp was presented as YY-Axxxxxxx (YY = Yanayacu, A = first author code).

The caterpillars collected in the field had already been parasitized (or not). Thus, parasitoid identification was based on adult wasps just after their emergence (Dyer et al. 2007) in the rearing containers, or by later study by taxonomists using all information available. In contrast, caterpillar identifications were done at the time of caterpillar collection based on larval morphology (using photographs and previous rearings), since the caterpillar host is destroyed when the parasitoid emerges. Alternatively, lepidopteran identifications were based on adult morphology when a presumably conspecific sample contained more than one caterpillar, including at least one survivor. After emerging, all parasitoid wasps were preserved in 95% to 100% ethanol, facilitating their future use in molecular systematic work. All the Microgastrinae samples were initially sent to the James B. Whitfield lab, [Systematics of Parasitic Hymenoptera at the University of Illinois, Urbana-Champaign (UIUC), Illinois, USA] and later to the Canadian National Collection (CNC) of Insects, Arachnids and Nematodes, Ottawa, Ontario, Canada or the Pontificia Universidad Católica del Ecuador, Colección Entomológica, Quito, Ecuador. Adults collected using Malaise traps were available (for Costa Rica) supplementing the reared material. Information and pictures about herbivore hosts (Lepidoptera) and food plants were provided by DH Janzen and LA Dyer and are accessible in searchable databases (http://janzen.sas.upenn.edu/ and http://caterpillars.unr.edu/lsacat/ecuador/).

### Insect collection

In gregarious samples (some with more than 100 individuals) between six (three females and three males) to ten (five females and five males) specimens were selected for point mounting, while the remainder were kept in ethanol (100%) and refrigerated at -20 °C. Specimens selected to be point-mounted were previously treated with hexamethyldisilazane, [(CH_3_)_3_Si]_2_NH –HMDS, to permit easy manipulation and avoid specimen fragmentation during handling (Heraty and Hawks 1998). Specimens were placed into a small vial (2 ml) permitting the evaporation of ethanol and then were completely soaked in HMDS. A small amount of reagent was used, not only due to the small size of the specimens, but also to avoid gas buildup in the vial. Containers were capped and left in a fume hood for 1 hour at room temperature. After this time, the excess HMDS was eliminated by transferring the specimens into a small Petri dish and permit to air dry for 30 minutes. Additionally, Petri dishes were covered with glass microscope slides to prevent loss of specimens. The impact of this treatment on DNA is unknown, but in the study, a specimen or a leg from a specimen was taken from an ethanol-preserved specimen for DNA barcoding, before being treated as above. If further DNA analysis is to be performed, the ethanol-preserved specimens will be used.

### Wing slides

Wing slides were prepared for each species, and where feasible, for each sex. The right set of wings (fore and hind) was selected and placed between two glass microscope slides. Wings were detached from the body with the help of a #2 insect pin and thin forceps. Both wings were placed on the middle section of one of the two slides and soaked with ethanol, facilitating the spread of the wings on the slide. Wings were straightened out with the tip of a thin forceps through gentle movements to avoid tearing the wings. Afterward, excess ethanol was allowed to evaporate for a few seconds and only then was the second slide put on the top. For fastening the two slides together, cellophane tape was wrapped at both ends of the slide. Labels (identification and codes for sampling) were affixed to the right edge of the slide.

### Morphological image library

High-resolution images were obtained by two sources: scanning electron microscope (SEM) and digital photography. Each species description has images of full habitus, head, mesosoma, and metasoma in both lateral and dorsal views. In some cases, both male and female of each species were photographed. The species plates are presented in alphabetical order.

**Scanning electron microscopy (SEM).** All specimens used for SEM had their wings removed. No pre-cleaning procedure was done before SEM. The entire wasp was either directly affixed to or point-mounted on a metal stub with carbon adhesive tabs. Stub-mounted specimens were sputter coated using a Desk-1 TSC (Denton vacuum LLC, Moorestown, NJ, USA) with a gold-palladium alloy from at least three different angles while rotating the stub to ensure complete coverage. Then, images were taken with a Philips XL30 ESEM-FEG (FEI Company, Hillsboro, OR, USA) at the Imaging Technology Group (ITG) at Beckman Institute, UIUC. Each image was subsequently cropped and incorporated into the plates using Adobe Photoshop® CS v5 and saved as .jpg files.

**Digital imaging.** Digital photos were taken with a Leica® DFC425 digital microscope camera affixed to a Leica M205 stereomicroscope (Wetzlar®, Germany) with white LED (light-emitting diode) ring lights and dome. Specimens for imaging were held in place under the Leica by gray play dough mounted on a gray background. The LAS (Leica Application Suite) Multifocus module integrated within the Leica microscope was used to create a series of partially focused images. The acquisition of a composite focused image from Z-stack images at different focus positions was obtained with Zerene Stacker™ version 1.04 (http://zerenesystems.com/cms/stacker). The final image was post-processed with Adobe Photoshop CS v5 and saved as .jpg files.

### Barcode of Life Data library [mitochondrial cytochrome oxidase subunit I (COI) gene]

All COI sequences of Costa Rican *Glyptapanteles* were generated at the Biodiversity Institute of Ontario (BIO, University of Guelph, Ontario, Canada), by methods described in other ACG inventory barcode inventory and taxonomic papers by Smith et al. (2007, 2008) and Fernández-Triana et al. (2014a), and deposited in the Barcode of Life Data System (BOLD, http://www.boldsystems.org; Ratnasingham and Hebert 2007). In contrast, the Ecuadorian sequences were generated in the facilities at UIUC, and then deposited in BOLD for analysis (https://doi.org/10.5883/DS-ASGLYP). At UIUC, an entire wasp (head and wings were the only body parts discarded) was selected for maceration in samples with multiple specimens (gregarious) while one hind leg of the pair was plucked in samples with a unique specimen (solitary), preserving the body for taxonomic work. In both situations, specimens were ground in a mini-pestle. Genomic DNA extractions were carried out using DNeasy tissue extraction kits (QIAGEN, Valencia, CA, USA). COI Primers used are listed in Table 1.

**Table 1.** Mitochondrial cytochrome oxidase subunit I (COI) primers used in this study.

Gene	Primer name	Sequence	Annealing T °C	Fragment length	Reference
COI					
Forward	LepF	5’-TAT CAA CCA ATC ATA AAG ATA TTGG-3’	52 °C	648 bp	Hajibabaei et al. 2006
Reverse	LepR	5’-TAA ACT TCT GGA TGT CCA AAA AAT CA-3’			
Forward	LCO1490	5’-GGT CAA CAA ATC ATA AAG ATA TTG G-3’	53 °C	658 bp	Folmer et al. 1994
Reverse	Ben3r	5’-GCW ACW ACR TAA TAK GTA TCA TG-3’			

At UIUC, Polymerase chain reactions (PCR) for all primer pairs were carried out in 25 µl reaction volumes consisting of molecular biology grade H_2_O=15.38 μl; 10*Ex* Taq buffer=2.5 μl; forward primer (10 μM)=1 μl; reverse primer (10 μM)=1μl; dNTPs mixture (10 mM)=2 μl; Takara *Ex* Taq DNA (5 U/µl)=0.125 μl and DNA template=3 μl. All amplifications were carried out using a Thermal Cycler (PCR Machine) C1000 Touch™ (Bio-Rad Laboratories, Hercules, CA, USA). The thermocycling program consisted of an initial denaturation step of 94 °C for 2 minutes, followed by 34 cycles [30 seconds at 94°, 25 seconds at 45 °C or 53 °C and 1 minute at 72 °C], and a final extension step of 72 °C for 4 minutes. A negative control was included in each round of amplifications that contained dH_2_O instead of DNA template. All PCR products were visualized on 1.5% agarose gels (Fisher Scientific, Pittsburgh, PA, USA), stained with red dye (Phenix Research, Candler, NC, USA) and visualized using UV light to measure PCR success. Amplicons were purified with QIAquick PCR purification kits (QIAGEN, Valencia, CA, USA) according to the manufacturer’s protocol. Sequences were generated via Sanger cycle-sequencing using amplification primers (forward and reverse directions) and visualized on ABI 3730 Capillary Electrophoresis Genetic Analyzer (Applied BioSystems) at the W.M. Keck Center for Comparative and Functional Genomics, UIUC. The bioinformatics software Geneious ProTM 5.3.4 (Biomatters Ltd., Newark, NJ, USA) was used to visualize chromatograms, edit sequences, and assemble both forward and reverse sequences in contigs. Additionally, all the sequences were translated to amino acids (invertebrate mitochondrial or standard genetic code) to assist in manual adjustments and proof-reading.

### Species boundaries

Three different datasets (COI sequences, natural history (host records), and external morphological characters) were integrated in order to generate realistic discrimination of species. While provisional species hypotheses were associated with an approximate 2% sequence divergence (Jones et al. 2011, Smith et al. 2013, Ratnasingham and Hebert 2013), we do not consider this a strict rule or criterion. For example, if we observed clear and consistent morphological differences and/or obvious biological differences between specimens with high sequence similarity we “split” these genetically distinct units into two names based on the weight of evidence. Using the same weight of evidence criteria, we split morphologically similar individuals if they were characterized by both genetic and biological differences.

A tree of COI DNA sequences was constructed in MEGA6 (Tamura et al. 2013) using the Maximum Likelihood (ML) method based on the General Time Reversible model (+G, parameter = 0.3431) (Nei and Kumar 2000). Samples selected for this representative tree were the holotypes for each species except for 5 cases (indicated with an * on the tree) where the holotype was not successfully sequenced (*G.boharti*, *G.alvarowillei*, and *G.alejandrovalerioi*) or having sequence but with insufficient overlap to permit tree construction (*G.mikeschauffi* and *G.sondrawardae*). In these cases, we substituted other high-quality sequences from the same species. All COI sequences and their specimen information are available on BOLD: https://doi.org/10.5883/DS-ASGLYP

### Species descriptions and taxonomic terminology

Descriptions are based on adult female/male holotypes. When additional specimens were available, notable intraspecific variation was reported. Base on a dataset of 126 characters and 484 character-states, a uniform format for species descriptions was generated with LucID 3.5 software (www.lucidcentral.com) using the Lucid3 Builder tool. The species descriptions are presented in alphabetical order.

Each examined sample that included type material has information about the country, province, region, sector, site, type of forest, elevation, latitude, longitude, collection date, collector, instar of caterpillar collected, date of formation of wasp cocoon (often), and emergence date of the adult parasitoid. The codes for sampling and DNA are also provided. For Costa Rican those codes are yy-SRNP-xxxxxx and DHJPARxxxxxx and for Ecuador EC-xxx and YY-Axxxx. Geographical coordinates are given in decimal degrees (DD). Latitude is expressed before longitude. Positive latitudes are north of the equator, negative latitudes are south of the equator. Positive longitudes are east of the prime meridian, negative longitudes are west of the prime meridian. The conversion of degree minutes seconds to decimal were obtained using the Federal Communication Commission (FCC) converter (http://transition.fcc.gov/mb/audio/bickel/DDDMMSS-decimal.html).

The total number of specimens examined as well as numbers of females and males are specified for each sample. At the beginning of each sample examined a series of numbers are presented [e.g., 8 (3♀, 3♂) (2♀, 0♂)]. The first number (8) indicates the total of specimens found in the sample followed by the number of female(s) and male(s) that were point-mounted (first parenthesis), and then by the quantity of females and males left in ethanol (second parentheses).

In the section of etymology, each species is named in honor of a person who has, during the past 55 years, helped Daniel H. Janzen, Winifred Hallwachs, and Lee A. Dyer, as well as many others identify and understand tropical fauna and flora. Those people constitute a diverse and far-flung team, without which this paper and many others like it would not exist. Mentors, colleagues, friends, and relatives of the first author are also included. Each person is mentioned after the word Etymology in each species account with a brief description of their interests.

**Character sampling.** Only characters derived from the external morphology were used. Most of the species descriptions are based on females; males were used only when females were absent.

**Measurements.** All specimens were examined using a Leica M125 stereomicroscope (Wetzlar, Germany). Holotype measurements were taken using a micrometer mounted in the microscope. Body length, antenna length, and fore wing length were taken in 2.0×, while remaining measurements in 10.0×. Body length was measured from the anterior margin of the head to the posterior margin of metasoma, excluding ovipositor and ovipositor sheath; and fore wing length from first axillary sclerite to the edge of the wing. All measurements are expressed in mm.

**Taxonomic characters.** All samples were identified to genus at UIUC using a key to New World genera of Microgastrinae (Whitfield 1997). Types for the previously described Neotropical species were examined: Blanchard collection, Buenos Aires, Argentina; Natural History Museum, London, UK (NHMUK); and United States National Museum USNM (now National Museum of Natural History, Smithsonian Institution, Washington, DC). Additional material examined: portions of Blanchard’s material, extensive reared material in USNM; Illinois Natural History Survey, Champaign, IL, USA; and Whitfield’s personal reared collection, Urbana, IL, USA. All illustrated and discussed in Whitfield et al. 2002a. Terminology for surface sculpturing follows Harris (1979), for wing venation follows Sharkey and Wharton (1997) and for morphology follows Mason (1981), Austin and Dangerfield (1992), Sharkey and Wharton (1997) and Whitfield (1997).

The antenna is described as it is resting above the body. The body coloration is defined as pale and dark. However, in the section’s coloration in adult wasps and species descriptions, the body coloration is treated in more detail. On the metasoma, the metasomal tergum 1 (T1) and the metasomal tergum 2 (T2) are divide into one mediotergite (medial chitinous portion) and two lateral tergites (two membranous lateral areas or laterotergites). Here, petiole (pe, Fig. 3C) on T1 and median area (ma, Fig. 3C) on T2 are the terms preferred over mediotergite on T1 and mediotergite on T2 respectively. Also, the sublateral area (sa, Fig. 3C) on T1 is the preferred terminology for the lateral tergite on T1. On T2, the terms adjacent area (ada, colored area next to the median area) and lateral end (le) are used to highlight the different colors of these two areas (Fig. 3C). Additionally, some morphological terms are used for the first time and refer to structures mainly in the scutellum and the metanotum. For the scutellum these are: axillary trough of scutellum (**ATS**) and medioposterior band of scutellum (**BS**). For the metanotum the terms are: anterior furrow of metanotum (**AFM**), the posterior furrow of metanotum (**PFM**), an axillary trough of metanotum (**ATM**), medioposterior band of metanotum (**BM**), and medioanterior pit of metanotum (**MPM**) (Fig. 2F). These external morphological terms, as well as others used in the descriptions, are illustrated in the Figures 2, 3.

The following acronyms are used to denote the depositories:

**CNC** Canadian National Collection of Insects, Arachnids and Nematodes, Ottawa, Ontario, Canada.

**PUCE** Pontificia Universidad Católica del Ecuador, Colección Entomológica, Quito, Ecuador.

Morphological terms and their abbreviations used in the text and figures are:

**ada** adjacent area,

**AFM** anterior furrow of metanotum,

**ATM** axillary trough of metanotum,

**ATS** axillary trough of scutellum,

**BM** medioposterior band of metanotum,

**BS** medioposterior band of scutellum,

**cl** clypeus,

**CR** Costa Rica,

**e** eye,

**EC** Ecuador,

**er** epicnemial ridge,

**f** female,

**fa** face,

**fc** fore coxa,

**fr** frons,

**G** gregarious,

**ge** gena,

**gusaneros** parataxonomists or paraecologists who find and rear the caterpillars (Janzen and Hallwachs 2011),

**hc** hind coxa,

**l** lunule of scutellum,

**la** labrum,

**le** lateral end,

**lp** labial palp,

**m** male,

**ma** median area,

**mc** middle coxa,

**md** mandible,

**me** mesopleuron,

**mls** malar suture,

**mp** maxillary palp,

**ms** mesoscutum,

**mtn** metanotum,

**MT** Malaise trap,

**MPM** medioanterior pit of metanotum,

**mtp** metapleuron,

**n** nucha,

**o** ocellus,

**OOL** ocular ocellar line (the shortest distance between lateral ocellus and adjacent compound eye margin),

**pe** petiole,

**pd** pedicel,

**pg** precoxal groove,

**ph** phragma of scutellum;

**pn** pronotum (1, dorsal furrow; 2, central area; 3 ventral furrow),

**POL** posterior ocellar line (the shortest distance between the lateral ocelli),

**pp** propodeum,

**ppl** propleuron,

**PFM** posterior furrow of metanotum;

**S** metasomal sternum:

**S4** sternum 4 or antepenultimate sternum,

**S5** sternum 5 or penultimate sternum,

**S6** sternum 6 or hypopygium,

**sa** sublateral area,

**sc** scape,

**scl** scutellum,

**so** solitary,

**T** metasomal tergum:

**T1** tergum 1,

**T2** tergum 2 and so on,

**te** temple,

**v** vertex,

**YPT** yellow-pan trap.

## Results

### Species

This *Glyptapanteles* species revision resulted in the recognition and description of 136 new species, none of them are shared between the two countries. Thus, 78 are from Costa Rica and the remaining 58 from Ecuador. The Costa Rican species are *alejandrovalerioi*, *alexborisenkoi*, *alvarowillei*, *andybennetti*, *andydeansi*, *annettewalkerae*, *barneyburksi*, *billbrowni*, *bobhanneri*, *bobkulai*, *bobwhartoni*, *boharti*, *brianestjaquesae*, *carlhuffakeri*, *carlossarmientoi*, *carlrettenmeyeri*, *charlesmicheneri*, *charlesporteri*, *chrisdarlingi*, *chrisgrinteri*, *christerhanssoni*, *corriemoreauae*, *daveroubiki*, *daveschindeli*, *davesmithi*, *davidwahli*, *donquickei*, *eowilsoni*, *garygibsoni*, *gavinbroadi*, *gerarddelvarei*, *henrytownesi*, *howelldalyi*, *hugokonsi*, *iangauldi*, *ianyarrowi*, *ilarisaaksjarvi*, *jacklonginoi*, *jamesrobertsoni*, *jeremydewaardi*, *jesusugaldei*, *jjrodriguezae*, *johnburnsi*, *johnheratyi*, *johnlasallei*, *johnnoyesi*, *lubomasneri*, *malloryvanwyngaardenae*, *marjorietownesae*, *markshawi*, *meganmiltonae*, *mehrdadhajibabaei*, *mikegatesi*, *mikeschauffi*, *mikesharkeyi*, *nataliaivanovae*, *nealweberi*, *ninazitaniae*, *pamitchellae*, *paulhansoni*, *paulheberti*, *paulhurdi*, *philwardi*, *robbinthorpi*, *ronaldzunigai*, *roysnellingi*, *scottmilleri*, *scottshawi*, *shelbystedenfeldae*, *sondrawardae*, *stephaniecluttsae*, *stephaniekirkae*, *sujeevanratnasinghami*, *sureshnaiki*, *sydneycameronae*, *tanyadapkeyae*, *victoriapookae*, and *wonyoungchoi*. The Ecuadorian species are *alexwildi*, *andrewdebeveci*, *andysuarezi*, *andywarreni*, *ankitaguptae*, *betogarciai*, *carinachicaizae*, *celsoazevedoi*, *claudiamartinezae*, *diegocamposi*, *dorislagosae*, *edgardpalacioi*, *edwinnarvaezi*, *erictepei*, *felipesotoi*, *ferfernandezi*, *genorodriguezae*, *grantgentryi*, *gunnarbrehmi*, *haroldgreeneyi*, *helmuthaguirrei*, *henryhespenheidei*, *jaquioconnorae*, *jerrypowelli*, *jimmilleri*, *johnstiremani*, *josesimbanai*, *juanvargasi*, *jumamuturii*, *keithwillmotti*, *kevinjohnsoni*, *kyleparksi*, *linghsiuae*, *luchosalagajei*, *malleyneae*, *mamiae*, *marcelotavaresi*, *marcepsteini*, *marcpolleti*, *marshawheelerae*, *mayberenbaumae*, *michelleduennesae*, *mikepoguei*, *montywoodi*, *pachopinasi*, *petermarzi*, *phildevriesi*, *rafamanitioi*, *suniae*, *suzannegreenae*, *taniaariasae*, *thibautdelsinnei*, *thomaspapei*, *toluagunbiadeae*, *tomwallai*, *wilmersimbanai*, *yalizhangae*, and *yanayacuensis.* Before this study, only six species had been described for the Neotropics. A total of 16,663 specimens was examined, of which 13,542 are preserved in 100% ethanol and 3,121 point-mounted.

The samples reviewed from Costa Rica are the result of 30 years of continuous collecting, from 1982 to 2012, and are accompanied by more than 100 undescribed sympatric species. In contrast, material examined from Ecuador covers a period of only five years (the oldest samples were caught in 2005 while the most recent in 2010). It is important to note that both inventories of the caterpillars and their food plants and parasitoids are still running and will continue for a number of years, which means even more species in this genus could be added to those described here and the 100+ undescribed species already collected. Indeed, material recently collected (2010–2019) for both projects, including reared specimens and Malaise-trapped specimens, was also available at the time of this revision, but it was not possible to include them. It is expected that those remaining species will be described in the near future.

### Identifying *Glyptapanteles* species using DNA barcoding (Fig. 1)

Within *Glyptapanteles*COI, intraspecific variation is much less than interspecific variation (on average 0.09% vs. 10.1 % when estimated using the BOLD distance summary tool on sequences longer than 400 bp (i.e., overlapping)).

**Figure 1.**
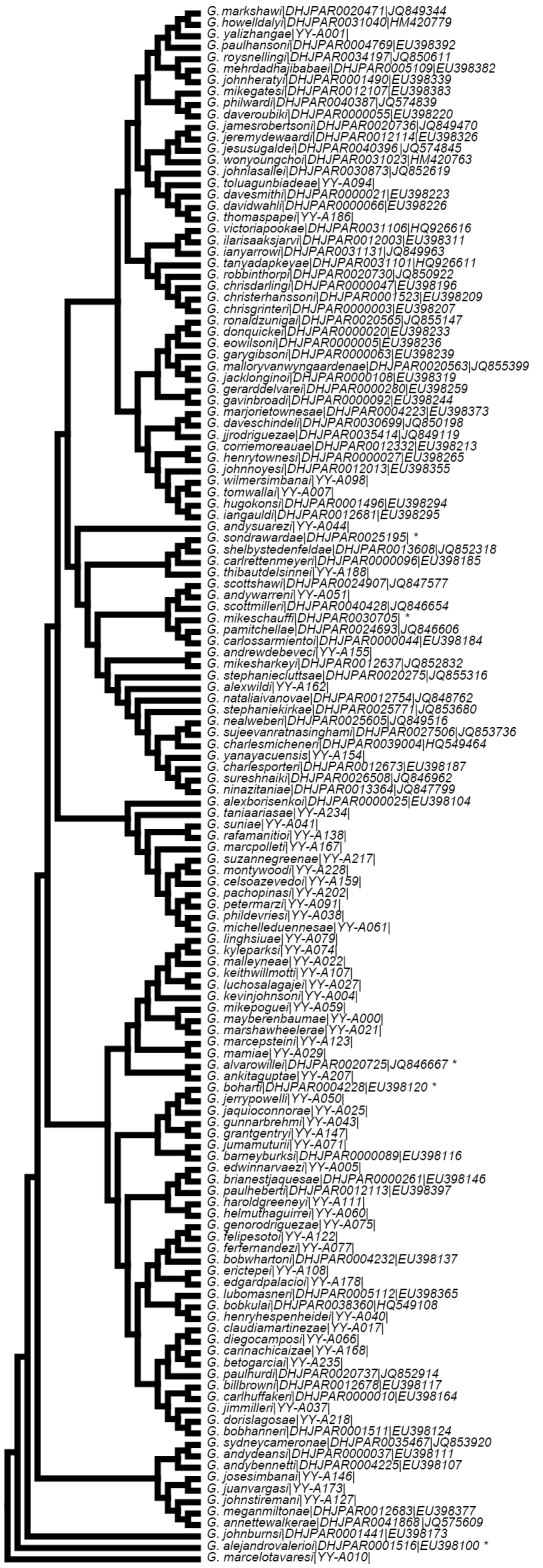
A tree of COI gene sequences from the new 136 species of *Glyptapanteles* is described here. The tree with the highest log likelihood (-12901.1226) is shown. Initial tree(s) for the heuristic search were obtained by applying the Neighbor-Joining method to a matrix of pairwise distances estimated using the Maximum Composite Likelihood (MCL) approach. A discrete Gamma distribution was used to model evolutionary rate differences among sites (5 categories (+G, parameter = 0.3431)). The rate variation model allowed for some sites to be evolutionarily invariable ([+I], 0.0000% sites). The tree is drawn to scale, with branch lengths measured by the number of substitutions per site. The analysis involved nucleotide sequences from 136 specimens using a total of 926 positions in the final dataset. Samples selected for this representative tree were the holotypes for each species except for five cases (indicated with an * on the tree) where the holotype was not successfully sequenced (*G.boharti*, *G.alvarowillei*, and *G.alejandrovalerioi*) or having sequence but with insufficient overlap to permit tree construction (*G.mikeschauffi* and *G sondrawardae*). In these cases, we substituted other high-quality sequences from the same species.

### Distribution

*Glyptapanteles* has a wide range of ecological distribution. These wasps can be found from 90 m to 2,800 m elevation. Elevation was not reported for only one species, *G.toluagunbiadeae*. Forty percent of the species (55 of 136 spp.) were reported above 1,500 m. All the species from Ecuador are found at or above an elevation of 1,000 m whereas Costa Rican species are found from 90 m to 1,460 m because that is the range available for sampling; there is no doubt that their distributions range down to sea level, and they will be found up to the highest available point (2,000 m in ACG).

### Body length

Most Neotropical *Glyptapanteles* (89%) are between 2 and 3.4 mm in length, whereas a low percentage is below (5%) or above (6%) that range. The seven species with body length less than 2 mm are *G.daveroubiki* (1.67), *G.carlossarmientoi* and *G.philwardi* (each with 1.81), *G.ronaldzunigai* (1.86), *G.carlrettenmeyeri* and *G.wonyoungchoi* (each with 1.91), and *G.chrisgrinteri* (1.96). The eight species more than 3.5 mm are: *G.charlesporteri* (3.53), *G.pachopinasi* (3.58), *G.sureshnaiki* (3.63), *G.ferfernandezi* (3.68), *G.ninazitaniae* (3.78), *G.alvarowillei* (3.81), *G.andydeansi* (3.85), and *G.malleyneae* (3.88).

### Coloration in adult wasps

In most species here described, the body coloration is generally dark, ranging from dark brown to black. Pale coloration (light or dark yellow) is mostly limited to legs, first segments of the metasoma, and, rarely, the antenna and the mesosoma. In species with a yellow metasoma, that pale coloration is limited exclusively to the first terga and first sterna when the specimen is seen in lateral view. However, twelve species displayed an uncommon pattern: the yellow coloration extends beyond including all the terga and all the sterna. Thus, in lateral view the metasoma appears to be completely yellow in females: *G.andybennetti* (Fig. 14A), *G.andydeansi* (Fig. 16A), *G.annettewalkerae* (Fig. 23A, K), *G.billbrowni* (Fig. 29A), *G.bobhanneri* (Fig. 31A), *G.brianestjaquesae* (Fig. 39A), *G.claudiamartinezae* (Fig. 58A, J), *G.gavinbroadi* (Fig. 88A), *G.mayberenbaumae* (Fig. 157A, J), *G.paulhurdi* (Fig. 184A, J), *G.suzannegreenae* (Fig. 211A, K), and *G.sydneycameronae* (Fig. 212A, J).

The six Neotropical *Glyptapanteles* species described previously to this study show bodies exclusively tinted with dark colors. Here, *G.stephaniecluttsae* (Figs 204–205) is the first Neotropical species with extensive yellow coloration on the mesosoma although all the mesoscutum, small portions on the scutellum, and the metanotum are dark brown. The head in this species is black. A closer look at the metasoma shows that in lateral view, the predominant coloration is pale (yellow) though dark brown areas are present and limited by a small area in the dorsal part of the T4 and beyond. It must be noted, however, that in dorsal view the dark areas are more extensive than pale areas. Thus, the T3 and the remaining terga are completely brown. The yellow coloration is limited to 3/4 proximally of the petiole and sublateral areas on T1, the median area but not in the male (Fig. 205D), and the lateral ends on T2 (Fig. 204H, I). This Costa Rican gregarious species was collected at 730 m in rain forest. Pale coloration or “xanthic coloration” along with enlarged compound eyes are features generally associated with species that are nocturnally active (Whitfield and Scaccia 1996).

Taking advantage of the large number of new species here described, the variation in the body coloration has been stressed in detail. These minute wasps are not wholly black as might be thought initially. The color variation in those species is focused on the antenna (five types), the propleuron (two types), the petiole (three types), the median area on T2 (three types), and the legs (13 types). In the legs, the greatest color variation occurs in the hind ones and includes mainly the segments of the coxa, the femora, and the tibia. It is worth mentioning that coloration was not taken into consideration in the key for species separation.

**Antennal coloration.** At least five types of antennal coloration were observed: all the antennal flagellomeres have the same color in both sides, only the dorsal part of all the antennal flagellomeres or only the dorsal part of the first proximal flagellomeres or only the last distal antennal flagellomeres are light-colored with the remaining areas dark-colored, and the antenna tricolored.

***All the antennal flagellomeres have the same color throughout.*** This is the most common color pattern. The antenna is completely dark brown throughout and occurs on most *Glyptapanteles* species (83 spp.). However, there are two variations: all the flagellomeres are stained darker with a black tint, this shade is present in only three species (*G.michelleduennesae*, *G.montywoodi*, and *G.petermarzi*) or all the flagellomeres can be completely yellow-brown, which was recorded in only one species (*G.johnburnsi*).

***The dorsal part of all the antennal flagellomeres is lighter than the ventral part.*** In this category, the dorsal coloration of the antenna (resting above the body) can be yellow-brown (only in *G.donquickei*) or light brown (as in *G.alejandrovalerioi*, *G.eowilsoni*, *G.marcelotavaresi*, *G.paulhansoni*, *G.phildevriesi*, and *G.sydneycameronae*). In both cases, the ventral coloration is (dark) brown.

***Only the dorsal part of the most proximal flagellomeres is lighter than the ventral part.*** In 27 species, the coloration of the dorsal part of the most proximal flagellomeres is light brown. The species that repeat this pattern are *G.daveschindeli*, *G.garygibsoni*, *G.gavinbroadi*, *G.hugokonsi*, *G.ilarisaaksjarvi*, *G.johnlasallei*, *G.keithwillmotti*, *G.kyleparksi*, *G.luchosalagajei*, *G.markshawi*, *G.meganmiltonae*, *G.mehrdadhajibabaei*, *G.mikeschauffi*, *G.nataliaivanovae*, *G.robbinthorpi*, *G.roysnellingi*, *G.scottmilleri*, *G.shelbystedenfeldae*, *G.sondrawardae*, *G.sujeevanratnasinghami*, *G.suniae*, *G.tanyadapkeyae*, *G.thibautdelsinnei*, *G.thomaspapei*, *G.toluagunbiadeae*, *G.yalizhangae*, and *G.yanayacuensis*.

In other species, the dorsal coloration of the most proximal flagellomeres is yellow-brown and it was recorded in ten species: *G.charlesmicheneri*, *G.christerhanssoni*, *G.davidwahli*, *G.howelldalyi*, *G.jamesrobertsoni*, *G.jeremydewaardi*, *G.jesusugaldei*, *G.jjrodriguezae*, *G.malloryvanwyngaardenae*, and *G.marjorietownesae*. In one species, *G.davesmithi*, the first five proximal flagellomeres are dorsally yellow. In all instances, the ventral coloration of those proximal flagellomeres is dark brown. However, there is a slight modification to this pattern: in *G.scottshawi*, the first seven-eight proximal antennal flagellomeres are yellow on both sides. On all the examples cited, the remaining flagellomeres are brown or dark brown.

***The last distal antennal flagellomeres are lighter than the remaining flagellomeres.*** Only four species fit in this category. The last distal antennal flagellomeres can be completely yellow (*G.ninazitaniae* and *G.sureshnaiki* only in females), yellow-brown (*G.bobwhartoni*) or light brown (*G.mikegatesi*). In all these species, the remaining flagellomeres are brown or dark brown.

***Antenna tricolored.*** Only one species, *G.wonyoungchoi*, has the antenna with three colors: the first four proximal antennal flagellomeres are completely yellow, the following five to seven are totally yellow-brown, and the remaining flagellomeres are brown on both sides.

**Propleuron coloration**. With respect to propleuron two types of coloration were observed: propleuron matches the mesosoma coloration and propleuron coloration differs partially or completely from mesosoma coloration.

***Propleuron matches the mesosoma coloration.*** In the vast majority of the species (102 spp.) the propleuron is dark as the mesosoma. Only in one species, *G.stephaniecluttsae*, the propleuron is as pale (yellow) as mesosoma.

***Propleuron coloration differs partially or completely from mesosoma coloration.*** In five species the propleuron coloration (entirely yellow or entirely light brown) deviates from mesosoma coloration (brown or brown-black): *G.alexwildi* (Fig. 9H), *G.barneyburksi* (Fig. 25A), *G.bobhanneri* (Fig. 31A), *G.jaquioconnorae* (Fig. 115I), and *G.mikesharkeyi* (Fig. 168A). In 28 species, the propleuron has some pale areas (yellow-brown or light brown) that contrasts with the dark color of the mesosoma: *G.andrewdebeveci* (Fig. 12J), *G.annettewalkerae* (Fig. 23J), *G.brianestjaquesae* (Fig. 39A), *G.charlesporteri* (Fig. 50A), *G.daveschindeli* (Fig. 64A), *G.grantgentryi* (Fig. 94I), *G.gunnarbrehmi* (Fig. 95J), *G.haroldgreeneyi*, *G.helmuthaguirrei* (Fig. 97I), *G.jesusugaldei*, *G.josesimbanai*, *G.mayberenbaumae*, *G.meganmiltonae* (Fig. 158A), *G.nataliaivanovae* (Fig. 171I), *G.nealweberi* (Fig. 173G), *G.ninazitaniae* (Fig. 175I), *G.pamitchellae* (Fig. 178I), *G.paulheberti*, *G.paulhurdi* (Fig. 184A), *G.scottmilleri*, *G.scottshawi* (Fig. 199I), *G.shelbystedenfeldae*, *G.sondrawardae* (Fig. 203I), *G.stephaniekirkae* (Fig. 206C), *G.sujeevanratnasinghami* (Fig. 208I), *G.sureshnaiki* (Fig. 210I), *G.sydneycameronae* (Fig. 212I), and *G.yanayacuensis* (Fig. 223I).

**Petiole coloration.** Three types of coloration were observed: petiole entirely dark, petiole entirely pale, and petiole with two colors.

***Petiole entirely dark.*** This is the category that contains the largest number of species. In 107 species the petiole is dark brown or brown-black (same coloration as metasoma) and the contours of the petiole can or can not be darkened.

***Petiole entirely pale.*** This is an unusual case. Only in *G.stephaniecluttsae*, the petiole is yellow with contours yellow-brown.

***Petiole with two colors.*** In two species, the pale coloration dominates over the dark one. *Glyptapantelesalexwildi* (Fig. 9H) has the petiole yellow-brown with the entire inner edge dark brown. *Glyptapantelessuzannegreenae* (Fig. 211H) has it dark yellow; however, the lateral parts on the distal half have light yellow-brown tints. In both species the petiole contours are darkened. In some species, the pale coloration on the petiole covers more area than the dark one. Thus, in six species 3/4 of the proximal part is pale and the distal 1/4 is dark. The pale coloration varies from yellow, yellow-brown, brown-orange, and reddish brown while the dark coloration is dark brown or brown-black. Those species are: *G.charlesmicheneri*, *G.corriemoreauae*, *G.davidwahli*, *G.ilarisaaksjarvi* (Fig. 109D), *G.malloryvanwyngaardenae* (Fig. 146F), and *G.pamitchellae* (Fig. 178G). In one species, *G.eowilsoni*, proximal 2/3 is reddish brown and the distal 1/3 is black. In some species the dark coloration on the petiole dominates the pale coloration. Thus, in three species 3/4 of the distal part is dark and the proximal 1/4 is pale. The pale coloration changes from yellow-brown and reddish and the dark coloration from dark brown and black. These species are *G.mehrdadhajibabaei*, *G.scottshawi* (Fig. 199G), and *G.sondrawardae* (Fig. 203G). In seven species the dark and the pale colorations cover the petiole in equal proportions. The pale coloration is variegated, and can be yellow, brown-orange, brown-red, reddish or light brown. The dark coloration can be brown or dark brown. Those species are: *G.daveschindeli*, *G.jamesrobertsoni*, *G.jesusugaldei*, *G.jumamuturii*, *G.kyleparksi*, *G.mayberenbaumae*, and *G.tanyadapkeyae* (Fig. 214G).

In seven species the petiole is mostly dark but with a central pale area. The pale coloration can be yellow, yellow-brown or light brown, and the dark coloration fluctuates between brown and brown-black. These species are *G.donquickei*, *G.gerarddelvarei*, *G.henrytownesi*, *G.howelldalyi*, *G.jeremydewaardi*, *G.johnheratyi*, and *G.robbinthorpi*.

***Petiole with three colors.*** In two species the petiole coloration intensifies from proximal to distal, in both species their contours are darkened. In *Glyptapantelesmarjorietownesae* proximally the petiole is yellow, medially reddish/yellow-brown and distally brown and in *G.markshawi* the petiole proximally is yellow-brown, medially light brown and distally dark brown.

**Coloration in the median area on T2.** In most of the species here described (109 spp.) the median area (chitinous portion) is dark (dark brown or brown-black) and next to it there is an obviously defined colored area (membranous portion), the adjacent area (ada, Fig. 3C), which is also dark, and the lateral ends pale.

In twelve species the median area is dark and the lateral ends are pale; this means the adjacent area is missing on T2: *G.alexborisenkoi* (Fig. 8G), *G.bobhanneri*, *G.brianestjaquesae*, *G.carlhuffakeri* (Fig. 42G), *G.christerhanssoni*, *G.daveroubiki*, *G.daveschindeli*, *G.gavinbroadi*, *G.howelldalyi*, *G.marjorietownesae*, *G.mikeschauffi* (Fig. 166F), and *G.paulhurdi* (Fig. 184H).

In twelve species both the median area and lateral ends are dark (the adjacent area is absent): *G.alejandrovalerioi*, *G.ankitaguptae* (Fig. 22H), *G.carlossarmientoi*, *G.celsoazevedoi* (Fig. 47H), *G.haroldgreeneyi* (Fig. 96H), *G.jerrypowelli* (Fig. 118H), *G.johnburnsi*, *G.johnnoyesi* (Fig. 131F), *G.marcelotavaresi* (Fig. 149G), *G.marcepsteini* (Fig. 150H), *G.marcpolleti* (Fig. 151G), and *G.toluagunbiadeae* (Fig. 217G).

Four species show unusual color patterns. First, both the median area and the lateral ends are pale, and the adjacent area is dark: *G.stephaniecluttsae* (Fig. 204H) and *G.stephaniekirkae* (Fig. 206G). Second, the median area as well as the lateral ends are pale, the adjacent area is missing, *G.wonyoungchoi* (Fig. 221G). Third, the median area has two colors (proximal 1/3 yellow, distal 2/3 brown), the lateral ends are yellow, and the adjacent area is absent in *G.suzannegreenae* (Fig. 211H).

**Coxal coloration.** Taking into account the coloration of all the coxae compared with the body coloration, six types were recorded: the body and all the coxae yellow; the body dark and all the coxae yellow; the body dark, the fore and the middle coxae yellow, and the hind coxae with two colors; the body and the hind coxae dark, and the fore and the middle coxae yellow; the body, the middle and the hind coxae dark, and the fore coxae yellow; and the body and all the coxae dark.

***The body and all the coxae yellow*** (Figs 204A, 205A). This is an unusual pattern of coloration, only was reported in one species, *G.stephaniecluttsae*.

***The body dark and all the coxae yellow.*** Only four species have the hind coxae completely yellow [*G.alexwildi* (Fig. 9K), *G.andrewdebeveci* (Fig. 12K), *G.mayberenbaumae* (Fig. 157J), and *G.yanayacuensis* (Fig. 223J)].

***The body dark, the fore and the middle coxae yellow, and the hind coxae with two colors.*** It is worth mentioning that of the three pairs of coxae are the hind ones which can exhibit at the same time two colors which vary in quantity and location. In this category, the fore and the middle coxae always are completely yellow, thus the differences are based on hind coxae coloration. This is the largest size category with a total of 51 species. First, it will start with the species whose hind coxae coloration is mostly yellow with a little of brown or brown-black and it will end with the species showing the opposite pattern, brown or black coloration dominates over the yellow coloration.

In one species (*G.meganmiltonae*, Fig. 158A) the hind coxae are yellow, but proximally with an irregular area brown, although in the male they are completely light brown (Fig. 159A); in one species (*G.ankitaguptae*, Fig. 22K) the yellow hind coxae have a tiny black spot both in proximal and distal edges; and in one species (*G.marcelotavaresi*, Fig. 149J) has the hind coxae both proximally and distally with an irregular narrow brown area, thus the yellow coloration is in the middle part.

Another dark mark detected in the yellow hind coxae is a dorsal elongated brown-black spot on the proximal part, this pattern was observed in three species (*G.ninazitaniae*, Fig. 175J; *G.sureshnaiki*, Fig. 210J; and *G.taniaariasae*, Fig. 213A), and in one species (*G.mikesharkeyi*, Fig. 168J) the size of the spot was smaller.

In three species, the dark coloration is located in the same place, covering the proximal third of the hind coxae, the remaining area is yellow. *Glyptapantelesnataliaivanovae* female exhibits unevenly dark brown blotches (Fig. 171J); however, in the male those marks are more extensive, covering until the proximal half (Fig. 172I); in *G.stephaniekirkae* (Fig. 206J) both sexes have the brown-black coloration uniform, but in male the area is more extensive covering until the proximal half (Fig. 207A); and in *G.thibautdelsinnei* male (Fig. 215K) the brown-black coloration also is homogeneous; however, its expansion is unknown in the female.

In four species the dark coloration on hind coxae is more extensive, covering the half or more than half the length of the hind coxae. *Glyptapantelescarlossarmientoi* female (Fig. 44A) has the proximal half light brown (distal half yellow), but in the male (Fig. 45A) the hind coxae are completely light brown; in both sexes of *G.nealweberi* (Figs 173H, 174J) the proximal half is brown-black and distal half is yellow; and in two species (*G.pamitchellae*, Fig. 178A and *G.charlesmicheneri*, Fig. 48A) the brown-black coloration covers the proximal 3/4 and only the distal 1/4 is yellow.

In three species (*G.charlesporteri* both sexes, Fig. 50A; *G.scottshawi* only female, Fig. 199A; and *G.sydneycameronae* only female, Fig. 212J) the distal third has a yellow area which is more extensive ventrally, reaching almost the distal half. Thus, brown-black is the dominant color on hind coxae.

In 25 species the hind coxae are brown or brown-black but distally have a yellow area which is distinctively tiny: *G.annettewalkerae*, *G.betogarciai*, *G.billbrowni*, *G.bobkulai*, *G.bobwhartoni*, *G.boharti*, *G.brianestjaquesae*, *G.carlhuffakeri*, *G.claudiamartinezae*, *G.davesmithi* (male with fore and middle coxae yellow-brown instead of light yellow), *G.diegocamposi*, *G.edwinnarvaezi*, *G.felipesotoi*, *G.ferfernandezi*, *G.genorodriguezae*, *G.haroldgreeneyi*, *G.helmuthaguirrei*, *G.henryhespenheidei*, *G.jaquioconnorae* (dorso-distally takes on a somewhat lighter color, yellow-brown), *G.keithwillmotti*, *G.kevinjohnsoni*, *G.kyleparksi*, *G.linghsiuae G*. *marcepsteini*, and *G.wonyoungchoi*. However, in four species (*G.bobhanneri*, *G.erictepei*, *G.grantgentryi*, and *G.gunnarbrehmi*) the distal tiny area is lighter (yellow-brown) than the remaining brown-black area.

In one species (*G.sujeevanratnasinghami*) the yellow-brown coloration covers the distal half of the hind coxae, the proximal half is brown-black; in two species (*G.edgardpalacioi* and *G.johnstiremani*) the distal third is yellow-brown (proximal 2/3 is brown-black), and in two species (G. *andydeansi* and G. *jeremydewaardi*) the yellow-brown coloration is restricted to ventro-distal part and covers a small area, the remaining area is brown-black.

***The body and the hind coxae dark, and the fore and the middle coxae pale.*** In total, 29 species exhibits hind coxae completely dark, that coloration coincides with the dark body coloration; the fore and the middle coxae are completely yellow. Those species are: *G.alexborisenkoi*, *G.alvarowillei*, *G.carinachicaizae*, *G.dorislagosae*, *G.jerrypowelli*, *G.jesusugaldei*, *G.jimmilleri*, *G.josesimbanai*, *G.juanvargasi*, *G.jumamuturii*, *G.luchosalagajei*, *G.malleyneae*, *G.mamiae*, *G.markshawi*, *G.marshawheelerae*, *G.michelleduennesae*, *G.mikegatesi*, *G.mikepoguei*, *G.montywoodi*, *G.pachopinasi*, *G.paulheberti*, *G.paulhurdi*, *G.petermarzi*, *G.phildevriesi*, *G.rafamanitioi*, *G.shelbystedenfeldae*, *G.sondrawardae*, *G.suniae*, and *G.suzannegreenae.* However, in three species the hind coxae are lighter (yellow-brown/light brown) than body coloration (*G.andybennetti*, Figs 14A, 15A; *G.barneyburksi*, Figs 25A, 26A; and *G.lubomasneri*, Figs 142A, 143A).

***The body, the middle and the hind coxae dark, and the fore coxae yellow.*** In four species (*G.carlrettenmeyeri*, Fig. 46A; *G.daveschindeli*, Fig. 64A; *G.johnheratyi*, Fig. 127A; and *G.toluagunbiadeae*, Fig. 217A) the coloration of the coxae changes from light to dark. Thus, the fore coxae are yellow, the middle coxae yellow-brown, and the hind coxae brown-black. In all of them, the body coloration corresponds with the hind coxae coloration, brown-black.

***The body and all the coxae dark.*** In 24 species the dark coloration (dark brown or brown-black) of all the coxae matches with the dark body coloration. Those species are: *G.alejandrovalerioi*, *G.andysuarezi*, *G.andywarreni*, *G.celsoazevedoi*, *G.daveroubiki*, *G.davidwahli*, *G.garygibsoni*, *G.gavinbroadi*, *G.gerarddelvarei*, *G.henrytownesi*, *G.jjrodriguezae*, *G.johnburnsi*, *G.johnnoyesi*, *G.malloryvanwyngaardenae*, *G.marcpolleti*, *G.paulhansoni*, *G.ronaldzunigai*, *G.roysnellingi*, *G.scottmilleri*, *G.tanyadapkeyae*, *G.thomaspapei*, *G.tomwallai*, *G.wilmersimbanai*, and *G.yalizhangae.* However, in five species all the coxae exhibit a coloration lighter than body: *G.chrisgrinteri*, *G.eowilsoni*, *G.howelldalyi*, *G.hugokonsi*, and *G.marjorietownesae*. However, in five species all the coxae are lighter than body coloration: *G.chrisgrinteri*, *G.eowilsoni*, *G.howelldalyi*, *G.hugokonsi*, and *G.marjorietownesae*.

When comparing the coxae coloration, some species exhibit the fore and middle coxae slightly light or lighter (yellow-brown or light brown) than hind coxae (brown-black). In all of them, the hind coxae coloration matches the body coloration. The 15 species with this pattern are *G.chrisdarlingi*, *G.christerhanssoni*, *G.corriemoreauae*, *G.donquickei*, *G.iangauldi*, *G.ianyarrowi*, *G.ilarisaaksjarvi*, *G.jacklonginoi*, *G.jamesrobertsoni*, *G.johnlasallei*, *G.mehrdadhajibabaei*, *G.mikeschauffi*, *G.philwardi*, *G.robbinthorpi*, and *G.victoriapookae*.

**Femoral coloration.** Five types of femoral coloration were recorded: all the femora pale; the fore femora with two colorations, and the middle and the hind ones dark; the fore and the middle femora with two colorations, and the hind ones dark; the fore and the middle femora pale, and the hind ones dark; and the fore and the middle femora pale, and the hind ones with two colorations.

***All the femora pale.*** In nine species, the femora in all the three pairs of legs are completely pale: *G.alexborisenkoi*, *G.ankitaguptae*, *G.boharti*, *G.charlesporteri*, *G.gavinbroadi*, *G.haroldgreeneyi*, *G.mayberenbaumae*, *G.paulheberti*, and *G.stephaniecluttsae*. A variation was also registered in species with all the femora yellow. A narrow dorsal dark strip from top to bottom can be present in all of them (*G.celsoazevedoi*, *G.johnstiremani*, *G.marcepsteini*, and *G.petermarzi*) or only in the fore and the middle femora (*G.phildevriesi*) or only in the middle and the hind femora (*G.luchosalagajei* and *G.mamiae*) or only in the hind femora (*G.andybennetti* and *G.johnnoyesi*).

***The fore femora with two colorations, and the middle and the hind ones dark.*** Only one species fits in this category, *G.johnburnsi*.

***The fore and the middle femora with two colorations, and the hind ones dark.*** Two species registered this pattern: *G.alejandrovalerioi* and *G.shelbystedenfeldae*.

***The fore and the middle femora pale, and the hind ones dark.*** Only *G.mikegatesi* is in this category.

***The fore and the middle femora pale, and the hind ones with two colorations.*** The vast majority of species (114 spp.) are in this type of coloration. The variation in the dark coloration on hind femora mainly concerns quantity. The dark coloration can cover most of the apex, a tiny dot at the tip, the half of the length of the femora, or most to the entire structure.

**Tibial coloration.** Two types of tibial coloration were observed: all the tibiae pale, and the fore and the middle tibiae pale, and the hind tibiae with two colorations.

***All the tibiae pale.*** Six species matches this pattern: *G.mayberenbaumae*, *G.mikesharkeyi*, *G.nealweberi*, *G.ninazitaniae*, *G.stephaniekirkae*, and *G.sureshnaiki*. Additionally, in two species, *G.ankitaguptae* and *G.johnstiremani*, all the pale tibiae have a narrow dorsal brown strip from top to bottom.

***The fore and the middle tibiae pale, and the hind tibiae with two colorations.*** The largest variation in the tibiae coloration is focused mainly on the hind tibiae. Most of the species here described (128 spp.) are included in this category. The variation in the dark coloration on hind tibiae includes the location (distally or at both ends) and quantity (mostly to entirely). In two species, *G.marcelotavaresi* and *G.marcpolleti*, the fore and middle tibiae are pale, but additionally, they have a narrow dorsal dark strip from top to bottom.

More details in the body parts which coloration varies are specified in the species description under the coloration section.

### Morphological image library

One problem that many taxonomists face during the identification of a specific taxon is the absence of drawings, images, or visual aids. Here, a detailed compilation of body part images was undertaken instead of a minimalist descriptive prose approach. It is expected that this high-resolution material will be of great help for future species identification and also serve as a source that facilitates the search of new morphological characters.

Approximately 2,300 original figures populate the morphological image library, which was used to create 222 plates. All of the 136 species described in this work were photographed, of which 84 species have plates for both sexes; for 39 species, only females were considered for the plates even though males were unknown for only ten species. In contrast, females of 13 species were unknown so digital images were taken from males in those cases. Most of the species (90%) were described based on females. A small fraction (10%, 13 spp.) of holotypes correspond to males, due to females not having been caught: *G.alexwildi*, *G.ankitaguptae*, *G.celsoazevedoi*, *G.dorislagosae*, *G.josesimbanai*, *G.juanvargasi*, *G.malleyneae*, *G.marcpolleti*, *G.montywoodi*, *G.pachopinasi*, *G.shelbystedenfeldae*, *G.tanyadapkeyae*, and *G.thomaspapei.*

**Figure 2.**
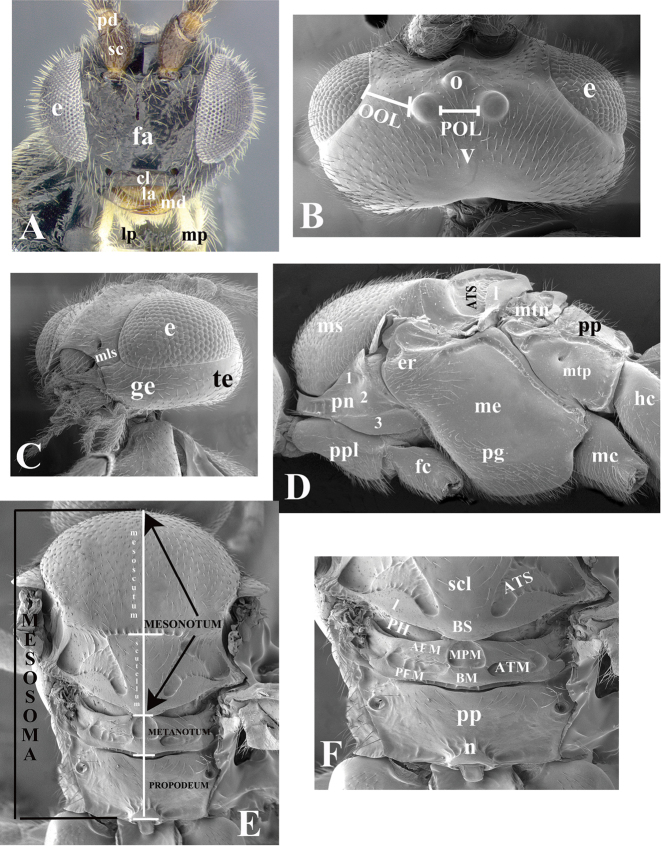
Head and mesosoma structures *Glyptapanteles* spp., females **A–C** Head **A** Frontal view, *G.felipesotoi* sp. nov. **B** Dorsal view, *G.alvarowillei* sp. nov. **C** Lateroventral view, *G.barneyburksi* sp. nov. **D, E** Mesosoma **D** Lateral view, *G.andybennetti* sp. nov. **E** Dorsal view, *G.ianyarrowi* sp. nov. **F** Scutellum, metanotum, propodeum, dorsal view, *G.ianyarrowi* sp. nov. Abbreviations: cl = clypeus, e = eye, er = epicnemial ridge, fa = face, fc = fore coxa, fr = frons, ge = gena, hc = hind coxa, l = lunule, la = labrum, lp = labial palp, md = mandible, mc = middle coxa, me = mesopleuron, mls = malar suture, mp = maxillary palp, ms = mesoscutum, mtn = metanotum (AFM = anterior furrow of metanotum; ATM = axillary trough of metanotum; BM = medioposterior band of metanotum; MPM = medioanterior pit of metanotum, PFM = Posterior furrow of metanotum), mtp = metapleuron, n = nucha, o = ocellus, OOL = ocular ocelar line (the shortest distance between lateral ocellus and adjacent compound eye margin), POL = posterior ocelar line (the shortest distance between the lateral ocelli), pd = pedicel, pg = precoxal groove, pn = pronotum (1, dorsal rim; 2, central area; 3 ventral rim), pp = propodeum, ppl = propleuron, sc = scape, scl = scutellum (ATS = axillary trough of scutellum; BS = medioposterior band of scutellum, PH = phragma of scutellum), t = temple, v = vertex.

**Figure 3.**
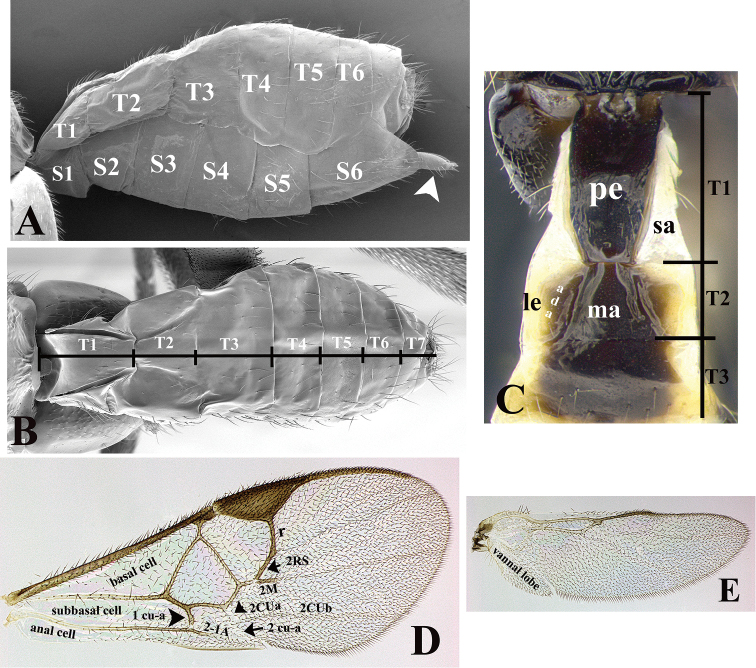
Metasoma and wings *Glyptapanteles* spp. **A, B** Metasoma **A** Lateral view, *G.barneyburksi* sp. nov., female **B** Dorsal view, *G.jeremydewaardi* sp. nov., female **C**T1–3, dorsal view, *G.sujeevanratnasinghami* sp. nov. **D, E** Wings, *G.mehrdadhajibabaei* sp. nov., male **E** Fore F Hind. Abbreviations: S4 = sternum 4 or antepenultimate sternum, S5 = sternum 5 or penultimate sternum, S6 = sternum 6 or hypopygium, T1 = tergum 1 (pe, petiole; sa, sublateral area), T2 = tergum 2 (ada, adjacent area; le, lateral end; ma, median area).

### Malaise-trapped specimens

In this revision, the specimens collected by Malaise traps came only from Costa Rica (material from Ecuador was also available but was not included in this revision) and constitute 27% (21 spp.) of the ACG species described here from that country (out of 78 spp.). Twelve species, of those 21 caught by Malaise traps, were also obtained from reared material. Thus, it was possible to assign two species as solitary (*G.nealweberi* and *G.sujeevanratnasinghami*) and the other ten species as gregarious (*G.andybennetti*, *G.charlesporteri*, *G.daveschindeli*, *G.eowilsoni*, *G.henrytownesi*, *G.hugokonsi*, *G.ilarisaaksjarvi* [also collected with YPT], *G.jamesrobertsoni*, *G.jesusugaldei*, and *G.philwardi*). Lifestyle, herbivore hosts, and host food plants for nine species continue to be unknown: *G.mikesharkeyi*, *G.nataliaivanovae*, *G.ninazitaniae*, *G.pamitchellae*, *G.scottshawi*, *G.shelbystedenfeldae*, *G.sondrawardae*, *G.stephaniekirkae*, and *G.sureshnaiki*. It is worth emphasizing that the project to date has reared more than 650,000 wild-caught caterpillars and 1,182 *Glyptapanteles*-attacked caterpillars, yet those nine species have not yet been reared. This gap between sampling methods emphasizes the importance of using diverse methods to assess biodiversity in a specific place. In addition, abundance of those species caught by Malaise traps and yet not recovered from wild caterpillars suggests that probably they are locally common: *G.mikesharkeyi* (133 specimens), *G.pamitchellae* (95), *G.scottshawi* (126), *G.sondrawardae* (148), *G.stephaniekirkae* (84), *G.sureshnaiki* (59), and to a lesser extent *G.nataliaivanovae* (11), *G.ninazitaniae* (5), and *G.shelbystedenfeldae* (13). It is unknown as to whether those species are solitary or gregarious, but that is irrelevant to their abundance in a Malaise trap. Since tens of thousands of exposed caterpillars living on herbs, small trees and bushes, and a very large number of leaf-rolling microlepidoptera have been well-sampled in both projects, the absence of these species in reared material suggests that these species may selectively parasitize either very cryptic caterpillars (leaf rolls, grass moths) or caterpillars that feed higher up in the canopy.

Considering the whole body of examined specimens, females were more abundant (12,609) than males (4,054). Female-biased sex ratios are common under the conditions of local mate competition (LMC, Hamilton 1967). LMC predicts that in environments consisting of several isolated patches where the number of mated females is small, it is advantageous for inseminated females to lay female-biased clutches. This is achievable because in Hymenoptera the act of fertilization is under the voluntary control of the egg-laying female. Adult females can alter progeny sex ratios by producing unfertilized male eggs and fertilized female eggs. Other factors such as host size, host quality, and superparasitism (Tagawa 2000) also play an important role when controlling the progeny of sex ratios, but their significance needs to be assessed in Neotropical *Glyptapanteles*.

A close look at Malaise-trapped specimens shows that the vast majority of samples exhibit the opposite pattern, with males exceeding females: *G.nataliaivanovae* (1♀, 10♂), *G.pamitchellae* (29♀, 66♂), *G.scottshawi* (17♀, 109♂), *G.shelbystedenfeldae* (1♀, 11♂), *G.sondrawardae* (8♀, 140♂), *G.stephaniekirkae* (10♀, 74♂), and *G.sureshnaiki* (13♀, 46♂). Males seem to be common near the forest floor where Malaise traps usually are set up while females are comparatively rare at the same place. It is also perhaps males are the dispersing-sex, moving around to look for females. As a result, males are easier to capture.

### Reared specimens (Table 2)

A total of 127 species is described here from the reared material, and nine species were collected only by Malaise traps. From the reared specimens, 92 *Glyptapanteles* species seem completely gregarious while 26 seem exclusively solitary (Table 2). It is worth noting that nine species reared showed both solitary and gregarious lifestyles: *G.alexborisenkoi* (with one sample gregarious of three specimens, and only one solitary), *G.bobkulai* (seven samples solitary, only one gregarious of two specimens), *G.boharti* (ten samples gregarious with two, three, five or ten specimens, and only one solitary), *G.bobwhartoni* (ten samples gregarious with no more than five specimens per sample, only one solitary), *G.carlhuffakeri* (16 samples gregarious with maximum of six or eleven specimens in few vials, and only one solitary), *G.haroldgreeneyi* (eleven samples gregarious with maximum three specimens, only one solitary), *G.jimmilleri* (five samples gregarious with two to four, seven specimens, only one solitary), *G.mamiae* (eight samples gregarious some with only two specimens, and only one solitary) and *G.meganmiltonae* ten samples gregarious, some of them with two, five, nine, maximum 13 specimens, and only one solitary). The presence of two specimens in a sample automatically qualifies it as “gregarious” (as obviously a misnomer, but as used traditionally in Hymenoptera natural history). The occurrence of a single solitary sample in a species which a majority of samples are gregarious can be explained by human error from transferring specimens from rearing containers to vials with ethanol or during handling of the material in the laboratory, or that simply one wasp larva survived the arduous trip from egg to emerging adult. Loss of specimens during these procedures is feasible due to the small size of the adult parasitoids. Almost all of the species mentioned above have gregarious samples with the minimum number of siblings (one) that categorizes a sample as gregarious, a fact that supports the idea of a technical artifact rather than a real biological phenomenon. However, it has been reported in other Microgastrinae genera that females of some species laid one to three eggs per host, but usually, only one offspring survived to adulthood. One example comes from *Microplitisdemolitor* Wilkinson that parasitizes the caterpillar *Heliothisvirescens* (F.) (Noctuidae) (Strand et al. 1988).

**Table 2.** Number of *Glyptapanteles* species described here, specifying its lifestyle. Abbreviations: G = gregarious; MT = Malaise trap, So = solitary.

	MT	So	G
**MT**	9	2	10
**So**	–	24	9
**G**	–	–	82

In the majority of gregarious species, the number of wasps emerging from one host caterpillar does not exceed one hundred individuals. Only seven species overstep this amount. Such is the case of *G.howelldalyi* (from 100 to 161 adults), *G.donquickei* (106), *G.andydeansi* (from 108 to 190), *G.iangauldi* (from 114 to 196), *G.andybennetti* (138), *G.billbrowni* (185), and *G.sydneycameronae* (212). It is assumed that offspring is the result of a single ovipositing female, but need not be. The hosts of these species belong to the Lepidoptera families Sphingidae, Noctuidae, and Apatelodidae (Table 4) with body sizes relatively large.

### Lepidoptera hosts (Tables 3, 4)

Fifteen lepidopteran families were reported as hosts of *Glyptapanteles* of which five families are reported for the first time as hosts from Neotropical *Glyptapanteles*: Crambidae, Depressariidae, Euteliidae, Hesperiidae, and Sphingidae (Table 3). However, in the older literature, almost any record of Elachistidae would be reported as Depressariidae today. A total of 88 species within 84 genera was identified as hosts. *Glyptapanteles* attacks mainly members of the family Noctuidae, followed by Erebidae, and then distantly by Geometridae (Table 3). In contrast, *Apanteles*, another of the “super genera” within Microgastrinae, parasitizes (in the same region) principally species of Hesperiidae, Elachistidae, and Crambidae (Fernández-Triana et al. 2014a). As for the very species-rich microgastrine genus, *Cotesia*, the most frequent hosts are Nymphalidae, Saturniidae, and Hesperiidae (O’Connor 2011). None of these host families used by *Apanteles* and *Cotesia* is the core target of *Glyptapanteles*. Putative *Glyptapanteles* species waiting for description and which belong to both projects have emerged from different Lepidoptera families from those reported here. This is the case for hosts from Bombycidae, Dalceridae, Gelechiidae, Nolidae, Riodinidae, and Tortricidae (Janzen and Dyer pers. obs.), demonstrating a wider breadth of host range within *Glyptapanteles*.

**Table 3.** Lepidoptera families reported as hosts of *Glyptapanteles*, numbers of newly described *Glyptapanteles* species parasitizing each family of Lepidoptera, and numbers of plant families that are consumed by the caterpillars that *Glyptapanteles* use as hosts. Key: * = newly reported lepidopteran family hosts. N.B. Pyralidae and Crambidae are generally confused with each other in the literature; Euteliidae has generally been reported as a member of Noctuidae, as is the case with Erebidae.

	Apatelodidae	*Crambridae	*Depressariidae	Erebidae	*Euteliidae	Geometridae	*Hesperiidae	Noctuidae	Notodontidae	Nymphalidae	Pantheidae	Pieridae	Pyralidae	Saturniidae	*Sphingidae
Number of *Glyptapanteles* spp.	8	2	1	26	1	19	1	30	11	15	1	2	7	4	4
Number of plant families	15	3	1	21	1	16	2	20	10	7	1	1	5	6	2

As mentioned before, 127 *Glyptapanteles* species out of 136 were obtained from reared material of which 74 species (58%) were recovered multiple times. Thus, the remaining 53 species had a unique rearing occurrence. In total, 45 *Glyptapanteles* species have registered only family or subfamily hosts determinations. Two species, *G.josesimbanai* and *G.marshawheelerae*, lack of any level of information about lepidopteran host affiliations (Table 4).

**Table 4.** List of new *Glyptapanteles* species successfully reared from caterpillars and the food plant species used by those caterpillars.

*Glyptapanteles* species	Lepidoptera host species	Lepidopteran host family/subfamily	Food plant species	Food plant family/subfamily
*G.agrotivorus* Whitfield	*Agrotisipsilon* (Hufnagel)	Noctuidae: Noctuinae	*Brassicaoleracea*	Brassicaceae
*G.alejandrovalerioi* sp. nov.	*Periphobaarcaei* (Druce)	Saturniidae: Hemileucinae	*Hymenaeacourbaril*	Fabaceae
			*Combretumfarinosum*	Combretaceae
*G.alexborisenkoi* sp. nov.	*Cynea* sp.	Hesperiidae: Hesperiinae	*Renealmiaalpinia*	Zingiberaceae
	*Salianaplacens* (Butler)	Hesperiidae: Hesperiinae	*Costusscaber*	Costaceae
*G.alexwildi* sp. nov.	Undetermined	Noctuidae	Diplaziumcostalevar.robustum	Dryopteridaceae
*G.alvarowillei* sp. nov.	*Pachydotadrucei* Rothschild	Erebidae: Arctiinae	*Ocoteawhitei*	Lauraceae
*G.andrewdebeveci* sp. nov.	Undetermined	Noctuidae	Diplaziumcostalevar.robustum	Dryopteridaceae
	Undetermined	Pyralidae	Diplaziumcostalevar.robustum	Dryopteridaceae
*G.andybennetti* sp. nov.	*Unzelajapix* (Cramer)	Sphingidae: Macroglossinae	*Davillakunthii*	Dilleniaceae
			*Davillanitida*	Dilleniaceae
		Sphingidae: Macroglossinae	*Doliocarpusmultiflorus*	Dilleniaceae
		Sphingidae: Macroglossinae	*Tetraceravolubilis*	Dilleniaceae
*G.andydeansi* sp. nov.	*Enyoocypete* (Linnaeus)	Sphingidae: Macroglossinae	*Doliocarpusmultiflorus*	Dilleniaceae
	*Pachygonidiadrucei* (Rothschild & Jordan)	Sphingidae: Macroglossinae	*Doliocarpusmultiflorus*	Dilleniaceae
	*Aleuroncarinata* (Walker)	Sphingidae: Macroglossinae	*Doliocarpusmultiflorus*	Dilleniaceae
*G.andysuarezi* sp. nov.	*Bertholdiapartita* Rawlins	Erebidae: Arctiinae	*Renealmiafragilis*	Zingiberaceae
*G.andywarreni* sp. nov.	Undetermined	Noctuidae	*Evodianthusfunifer*	Cyclanthaceae
*G.ankitaguptae* sp. nov.	Undetermined	Geometridae	Undetermined	Pteridophyta
*G.annettewalkerae* sp. nov.	*Sylleptenitidalis* Dognin	Crambidae: Spilomelinae	*Malvaviscusarboreus*	Malvaceae
	*Trichaeapilicornis* Herrich-Schäffer	Crambidae: Spilomelinae	*Psychotriapanamensis*	Rubiaceae
*G.barneyburksi* sp. nov.	*Smicropusintercepta* Walker	Geometridae: Sterrhinae	*Tetrapterysdiscolor*	Malpighiaceae
			*Mascagniasinemariensis*	Malpighiaceae
*G.betogarciai* sp. nov.	Undetermined	Geometridae	Undetermined	Pteridophyta
*G.billbrowni* sp. nov.	*Xylophanesporcus* (Hübner)	Sphingidae: Macroglossinae	*Psychotriaberteriana*	Rubiaceae
			*Hameliapatens*	Rubiaceae
*G.bobhanneri* sp. nov.	*Scoturaleucophleps* Warren	Notodontidae: Dioptinae	*Rinoreadeflexiflora*	Violaceae
			*Rinoreasylvatica*	Violaceae
*G.bobkulai* sp. nov.	*Eois* sp.	Geometridae: Larentiinae	*Piperaugustum*	Piperaceae
			*Piperglabrescens*	Piperaceae
	*Hagnagoramortipax* Butler	Geometridae: Larentiinae	*Clethramexicana*	Clethraceae
	*Semaeopusillimitata* Warren	Geometridae: Sterrhinae	*Abutapanamensis*	Menispermaceae
	Undetermined	Geometridae	*Tremamicrantha*	Cannabaceae
*G.bobwhartoni* sp. nov.	*Ochrodotamarina* Schaus	Erebidae: Arctiinae	*Ocotealeucoxylon*	Lauraceae
	*Symphlebiatessellata* (Schaus)	Erebidae: Arctiinae	*Pouteriaviridis*	Sapotaceae
	*Perigacluacina* Druce	Saturniidae: Hemileucinae	*Carapaguianensis*	Meliaceae
*G.boharti* sp. nov.	*Anomisluridula* Guenée	Noctuidae: Catocalinae	*Hampeaappendiculata*	Malvaceae
*G.bourquini* (Blanchard)	*Agrotisdeprivata* Walker	Noctuidae: Noctuinae	*Brassicaoleracea*	Brassicaceae
			*Medicagosativa*	Fabaceae
			*Viciavillosa*	Fabaceae
			*Zeamays*	Poaceae
			*Triticum* sp.	Poaceae
	*Agrotisgypaetina* Guenée	Noctuidae: Noctuinae	*Brassicaoleracea*	Brassicaceae
			*Medicagosativa*	Fabaceae
*G.bourquini* (Blanchard)	*Agrotisipsilon* (Hufnagel)	Noctuidae: Noctuinae	*Daucuscarota*	Apiaceae
			*Helianthusannuus*	Asteraceae
			*Lactucasativa*	Asteraceae
			*Medicagosativa*	Fabaceae
	*Helicoverpazea* (Boddie)	Noctuidae: Heliothinae	*Trifoliumrepens*	Fabaceae
	*Mythimnaunipunctata* (Haworth)	Noctuidae: Noctuinae		
	*Peridromamargaritasa* (Haworth)	Noctuidae: Noctuinae		
	*Peridromasaucia* (Hübner)	Noctuidae: Noctuinae	*Trifoliumrepens*	Fabaceae
			*Medicagosativa*	Fabaceae
*G.brianestjaquesae* sp. nov.	*Drugeramorona* Druce	Notodontidae: Heterocampinae	*Ossaeamicrantha*	Melastomataceae
			*Conostegiaxalapensis*	Melastomataceae
	*Rhudadifficilis* Schaus	Notodontidae: Heterocampinae	*Conostegiamicrantha*	Melastomataceae
*G.carinachicaizae* sp. nov.	Undetermined	Noctuidae	*Chusqueascandens*	Poaceae
*G.carlhuffakeri* sp. nov.	*Leucotmemisnexa* (Herrich-Schäffer)	Erebidae: Arctiinae	*Serjaniaatrolineata*	Sapindaceae
*G.carlossarmientoi* sp. nov.	*Aniclaignicans* (Guenée)	Noctuidae: Noctuinae	*Cynodonnlemfuensis* (introduced)	Poaceae
*G.carlrettenmeyeri* sp. nov.	*Isogonanatatrix* Guenée	Noctuidae: Catocalinae	*Celtisiguanaea*	Ulmaceae
*G.celsoazevedoi* sp. nov.	Undetermined	Geometridae	*Chusqueascandens*	Poaceae
*G.charlesmicheneri* sp. nov.	*Phyprosopusparthenope* Schaus	Noctuidae: Catocalinae	*Celtisiguanaea*	Ulmaceae
*G.charlesporteri* sp. nov.	*Apatelodes* sp.	Apatelodidae	*Philodendronrhodoaxis*	Araceae
	*Tarchonfelderi* Druce	Apatelodidae	*Chamaedoreatepejilote*	Araceae
			*Acalyphadiversifolia*	Euphorbiaceae
			*Heliconiairrasa*	Heliconiaceae
			*Pavoniaschiedeana*	Malvaceae
			*Psychotriaberteriana*	Rubiaceae
			*Lycianthespauciflora*	Solanaceae
*G.chrisdarlingi* sp. nov.	*Concana* sp.	Noctuidae: Bagisarinae	*Bunchosiacornifolia*	Malpighiaceae
*G.chrisgrinteri* sp. nov.	*Lesmoneaemylia* (Druce)	Noctuidae: Catocalinae	*Mimosadormiens*	Fabaceae
*G.christerhanssoni* sp. nov.	*Lepidodesgallopavo* Druce	Noctuidae: Catocalinae	*Bunchosiapolystachia*	Malpighiaceae
*G.claudiamartinezae* sp. nov.	Undetermined	Geometridae	Iiexaff.yurumanguinis	Aquifoliaceae
			Undetermined	Celastraceae
*G.corriemoreauae* sp. nov.	*Euphyiacrispa* Druce	Geometridae: Larentiinae	*Pleuropetalumsprucei*	Amaranthaceae
*G.daveroubiki* sp. nov.	Undetermined	Noctuidae	Undetermined	Undetermined
*G.daveschindeli* sp. nov.	*Oxydia* sp.	Geometridae: Ennominae	Undetermined	Undetermined
	*Oxydiaapidania* Cramer	Geometridae: Ennominae	*Ingapunctata*	Fabaceae
	*Oxydiavesulia* (Cramer)	Geometridae: Ennominae	*Spondiaspurpurea* (introduced)	Anacardiaceae
*G.davesmithi* sp. nov.	*Antiblemma* sp.	Erebidae: Eulepidotinae	*Henrietteatuberculosa*	Melastomataceae
	*Antiblemmaleucocyma* Hampson	Erebidae: Eulepidotinae	*Conostegiaxalapensis*	Melastomataceae
			*Miconiabrenesii*	Melastomataceae
			*Ossaeabrenesii*	Melastomataceae
*G.davidwahli* sp. nov.	*Parachaboraabydas* (Herrich-Schäffer)	Noctuidae: Catocalinae	*Tephrosiamultifolia*	Fabaceae
*G.diegocamposi* sp. nov.	Undetermined	Nymphalidae: Ithomiinae	*Cestrum* sp.	Solanaceae
			*Cestrummegalophyllum*	Solanaceae
*G.donquickei* sp. nov.	*Condicacupienta* (Cramer)	Noctuidae: Amphipyrinae	*Neurolaenalobata*	Asteraceae
			*Plucheacarolinensis*	Asteraceae
	*Condicafunerea* (Schaus)	Noctuidae: Amphipyrinae	*Neurolaenalobata*	Asteraceae
*G.dorislagosae* sp. nov.	*Nebulosayanayacu* Miller	Notodontidae: Dioptinae	*Tibouchinalepidota*	Melastomataceae
*G.edgardpalacioi* sp. nov.	Undetermined	Saturniidae	*Psammisia* sp.	Ericaceae
*G.edwinnarvaezi* sp. nov.	Undetermined	Apatelodidae	*Columnea* sp.	Gesneriaceae
			*Columneaericae*	Gesneriaceae
	Undetermined	Apatelodidae	*Alloplectustetragonoides*	Gesneriaceae
	Undetermined	Noctuidae	*Salviatortuosa*	Lamiaceae
	Undetermined	Nymphalidae: Ithomiinae	*Cestrummegalophyllum*	Solanaceae
*G.ecuadorius* Whitfield	*Helicoverpazea* (Boddie)	Noctuidae: Heliothinae	*Zeamays*	Poaceae
*G.eowilsoni* sp. nov.	*Calledemaplusia* Felder	Notodontidae: Nystaleinae	*Hirtellaamericana*	Chrysobalanaceae
			*Hirtellaguatemalensis*	Chrysobalanaceae
			*Hirtellaracemosa*	Chrysobalanaceae
			*Hirtellatriandra*	Chrysobalanaceae
			*Licaniaarborea*	Chrysobalanaceae
*G.erictepei* sp. nov.	*Actinotestratonice* Latreille	Nymphalidae: Acraeinae	*Eratopolymnioides*	Asteraceae
*G.felipesotoi* sp. nov.	Memphisnr.lorna (Druce)	Nymphalidae: Charaxinae	*Nectandra* sp.	Lauraceae
*G.ferfernandezi* sp. nov.	Memphisnr.lorna (Druce)	Nymphalidae: Charaxinae	*Nectandra* sp.	Lauraceae
*G.garygibsoni* sp. nov.	*Nystaleacollaris* Schaus	Notodontidae: Nystaleinae	*Psidiumguineense*	Myrtaceae
			*Eugeniasalamensis*	Myrtaceae
	*Nystaleaguzmani* Schaus	Notodontidae: Nystaleinae	*Calyptrantheschytraculia*	Myrtaceae
*G.gavinbroadi* sp. nov.	*Pararcteschneideriana* Stoll	Noctuidae: Catocalinae	*Cecropiapeltata*	Urticaceae
*G.genorodriguezae* sp. nov.	Memphisnr.lorna (Druce)	Nymphalidae: Charaxinae	*Nectandra* sp.	Lauraceae
*G.gerarddelvarei* sp. nov.	*Macrocnemecabimensis* Dyar	Erebidae: Arctiinae	*Fischeriapanamensis*	Apocynaceae
			*Mandevillahirsuta*	Apocynaceae
*G.grantgentryi* sp. nov.	Undetermined	Notodontidae	*Myriocarpa* sp.	Urticaceae
*G.gunnarbrehmi* sp. nov.	*Pantherodescolubrariaviperaria* Thierry-Mieg	Geometridae: Ennominae	*Boehmeriacaudata*	Urticaceae
	Undetermined	Undetermined	*Myriocarpa* sp.	Urticaceae
*G.haroldgreeneyi* sp. nov.	*Actinotestratonice* Latreille	Nymphalidae: Acraeinae	*Eratopolymnioides*	Asteraceae
			*Munnoziahastifolia*	Asteraceae
*G.helmuthaguirrei* sp. nov.	Undetermined	Pieridae	*Inga* sp.	Fabaceae
*G.henryhespenheidei* sp. nov.	Undetermined	Pieridae	*Inga* sp.	Fabaceae
*G.henrytownesi* sp. nov.	*Heterochromasarepta* (Druce)	Noctuidae: Amphipyrinae	*Smilaxmollis*	Smilacaceae
			*Smilaxspinosa*	Smilacaceae
*G.herbertii* (Ashmead)	*Anticarsiagemmatalis* (Hübner)	Noctuidae: Eulepidotinae		
	*Pseudoplusiaincludens* (Walker)	Noctuidae: Plusiinae		
	*Trichoplusiani* (Hübner)	Noctuidae: Plusiinae		
	*Nystaleanyseus* Cramer	Notodontidae: Nystaleinae	*Psidiumguajava*	Myrtaceae
*G.howelldalyi* sp. nov.	*Dyopschromatophila* Walker	Noctuidae: Catocalinae	*Cecropiapeltata*	Urticaceae
			*Coussapoanymphaeifolia*	Urticaceae
*G.hugokonsi* sp. nov.	*Olceclosteraamoria* Druce	Apatelodidae	*Amphilophiumpaniculatum*	Bignoniaceae
			*Pleonotomavariabilis*	Bignoniaceae
			*Gmelinaarborea* (introduced)	Verbenaceae
*G.iangauldi* sp. nov.	*Zanolaverago* Cramer	Apatelodidae	*Iresinediffusa*	Amaranthaceae
			*Philodendron* sp.	Araceae
			*Ingaoerstediana*	Fabaceae
			*Ingasamanensis*	Fabaceae
			*Hameliapatens*	Rubiaceae
			*Psychotriaberteriana*	Rubiaceae
*G.iangauldi* sp. nov.	*Zanolaverago* Cramer	Apatelodidae	*Spermacoceocymifolia*	Rubiaceae
			*Solanumcircinatum*	Solanaceae
*G.ianyarrowi* sp. nov.	*Episcepsishypoleuca* (Hampson)	Erebidae: Arctiinae	*Ochromapyramidale*	Malvaceae
	*Eucereonaurantiaca* Draudt	Erebidae: Arctiinae	*Ficuscitrifolia*	Moraceae
			*Ficuscolubrinae*	Moraceae
	*Hyaleucereamorosa* Schaus	Erebidae: Arctiinae	*Pouroumabicolor*	Urticaceae
	*Napataflaviceps* Hampson	Erebidae: Arctiinae	*Cespedesiaspathulata*	Ochnaceae
*G.ilarisaaksjarvi* sp. nov.	Undetermined	Noctuidae	*Stachytarphetajamaicensis*	Verbenaceae
	*Condicacupienta* (Cramer)	Noctuidae: Amphipyrinae	*Mikaniacordifolia*	Asteraceae
			*Mikaniamicrantha*	Asteraceae
	*Condicasutor* (Guenée)	Noctuidae: Amphipyrinae	*Eryngiumfoetidum*	Apiaceae
			*Elephantopusmollis*	Asteraceae
			*Lepidaploacinera*	Asteraceae
	*Agraphaoxygramma* (Geyer)	Noctuidae: Plusiinae	*Baccharistrinervis*	Asteraceae
	*Argyrogrammabasigera* (Walker)	Noctuidae: Plusiinae	*Hydrocotyleumbellate*	Araliaceae
	*Argyrogrammaverruca* (Fabricius)	Noctuidae: Plusiinae	*Echinodorussubalatus*	Alismataceae
	*Pseudoplusiaincludens* (Walker)	Noctuidae: Plusiinae	*Milleriaquinqueflora*	Asteraceae
*G.jacklonginoi* sp. nov.	*Gonodontapulverea* Schaus	Erebidae: Calpinae	Undetermined	Undetermined
*G.jamesrobertsoni* sp. nov.	*Antiblemma* sp.	Erebidae: Eulepidotinae	*Psychotriachagrensis*	Rubiaceae
			*Psychotriagraciliflora*	Rubiaceae
			*Psychotriapanamensis*	Rubiaceae
*G.jaquioconnorae* sp. nov.	Undetermined	Nymphalidae: Ithomiinae	Undetermined	Solanaceae
*G.jeremydewaardi* sp. nov.	*Antiblemma* sp.	Erebidae: Eulepidotinae	*Psychotriahorizontalis*	Rubiaceae
*G.jerrypowelli* sp. nov.	Undetermined	Nymphalidae: Ithomiinae	Schoenobibluscf.peruvianus	Thymeliaceae
*G.jesusugaldei* sp. nov.	*Antiblemma* sp.	Erebidae: Eulepidotinae	*Psychotriamicrodon*	Rubiaceae
			*Psychotrianervosa*	Rubiaceae
*G.jimmilleri* sp. nov.	Undetermined	Notodontidae	*Passiflora* sp.	Passifloraceae
			*Passifloraligularis*	Passifloraceae
	*Josialigata* Walker	Notodontidae: Dioptinae	*Passiflora* sp.	Passifloraceae
	*Lyces* sp.	Notodontidae: Dioptinae	*Passiflora* sp.	Passifloraceae
	*Lycesfornax* Druce	Notodontidae: Dioptinae	*Passifloraligularis*	Passifloraceae
*G.jjrodriguezae* sp. nov.	*Nagaravitrea* (Guenée)	Noctuidae: Stictopterinae	*Clusiacylindrical*	Clusiaceae
			*Garciniaintermedia*	Clusiaceae
*G.johnburnsi* sp. nov.	*Eunica* sp.	Nymphalidae: Biblidinae	*Mabeaoccidentalis*	Euphorbiaceae
	*Eunicacaresa* Hewitson	Nymphalidae: Biblidinae	*Mabeaoccidentalis*	Euphorbiaceae
	*Eunicamalvina* Bates	Nymphalidae: Biblidinae	*Mabeaoccidentalis*	Euphorbiaceae
*G.johnheratyi* sp. nov.	*Scaptiusvinasia* (Schaus)	Erebidae: Arctiinae	*Eugeniabasilaris*	Myrtaceae
*G.johnlasallei* sp. nov.	*Sericochroa* sp.	Notodontidae: Heterocampinae	*Vochysiaferruginea*	Vochysiaceae
			*Vochysiaguatemalensis*	Vochysiaceae
*G.johnnoyesi* sp. nov.	*Deinopabiligula* Guenée	Erebidae: Calpinae	*Pterocarpushayesii*	Fabaceae
	*Deinopasigniplena* Walker	Erebidae: Calpinae	*Swartziacostaricensis*	Fabaceae
*G.johnstiremani* sp. nov.	Undetermined	Undetermined	Undetermined	Urticaceae
	Undetermined	Pyralidae	Undetermined	Apiaceae
			*Urtica* sp.	Urticaceae
*G.josesimbanai* sp. nov.	Undetermined	Undetermined	*Rubus* sp.	Rosaceae
*G.juanvargasi* sp. nov.	Undetermined	Pyralidae	*Boehmeria* sp.	Urticaceae
*G.jumamuturii* sp. nov.	Undetermined	Pyralidae	*Oreopanax* sp.	Araliaceae
*G.keithwillmotti* sp. nov.	Undetermined	Noctuidae	*Dendrophorbiumlloense*	Asteraceae
			*Salviatortuosa*	Lamiaceae
*G.kevinjohnsoni* sp. nov.	Undetermined	Erebidae: Arctiinae	*Rubus* sp.	Rosaceae
*G.kyleparksi* sp. nov.	Undetermined	Nymphalidae	Undetermined	Undetermined
*G.linghsiuae* sp. nov.	*Hypanartia* sp.	Nymphalidae: Nymphalinae	*Boehmeria* sp.	Urticaceae
*G.lubomasneri* sp. nov.	*Ithomiahippocrenis* Bates	Nymphalidae: Ithomiinae	*Witheringiasolanacea*	Solanaceae
	*Mechanitisisthmia* Bates	Nymphalidae: Ithomiinae	*Solanumhayesii*	Solanaceae
*G.luchosalagajei* sp. nov.	Undetermined	Nymphalidae	*Myriocarpa* sp.	Urticaceae
			*Boehmeriacaudate*	Urticaceae
	*Hypanartia* sp.	Nymphalidae: Nymphalinae	*Myriocarpa* sp.	Urticaceae
			Undetermined	Urticaceae
	Undetermined	Saturniidae	*Boehmeriacaudate*	Urticaceae
	*Pseudautomerisyourii* Lemaire	Saturniidae: Hemileucinae	Undetermined	Melastomataceae
*G.malleyneae* sp. nov.	Undetermined	Pyralidae	Undetermined	Melastomataceae
*G.malloryvanwyngaardenae* sp. nov.	*Rifargiaelgiva* Schaus	Notodontidae: Heterocampinae	*Styraxargenteus*	Styracaceae
*G.mamiae* sp. nov.	Undetermined	Erebidae: Arctiinae	*Miconia* sp.	Melastomataceae
			*Chusqueascandens*	Poaceae
*G.marcelotavaresi* sp. nov.	Undetermined	Erebidae: Arctiinae	*Monninasubspeciosa*	Polygalaceae
*G.marcepsteini* sp. nov.	Undetermined	Pyralidae	Diplaziumcostalevar.robustum	Dryopteridaceae
*G.marcpolleti* sp. nov.	Undetermined	Apatelodidae	*Miconia* sp.	Melastomataceae
*G.marjorietownesae* sp. nov.	*Azetaceramina* Hübner	Noctuidae: Catocalinae	Undetermined	Undetermined
			*Acosmiumpanamense*	Fabaceae
*G.markshawi* sp. nov.	*Ethmiascythropa* Walsingham	Depressaridae: Ethmiinae	*Bourreriacostaricensis*	Boraginaceae
			*Bourreriaoxyphylla*	Boraginaceae
*G.marshawheelerae* sp. nov.	Undetermined	Undetermined	*Vismia* sp.	Clusiaceae
*G.mayberenbaumae* sp. nov.	Undetermined	Noctuidae	*Burmeisteraborgensis*	Campanulaceae
*G.meganmiltonae* sp. nov.	*Herpetogramma* sp.	Crambidae: Spilomelinae	*Achyranthesaspera*	Amaranthaceae
			*Achyranthesindica*	Amaranthaceae
			*Alternantherapubiflora*	Amaranthaceae
*G.mehrdadhajibabaei* sp. nov.	*Carathisseptentrionalis* Becker	Erebidae: Arctiinae	*Nectandramartinicensis*	Lauraceae
*G.michelleduennesae* sp. nov.	Undetermined	Pantheidae	*Rubus* sp.	Rosaceae
*G.mikegatesi* sp. nov.	*Pero* sp.	Geometridae: Ennominae	Undetermined	Undetermined
			*Cyathulaachyranthoides*	Amaranthaceae
*G.mikepoguei* sp. nov.	Undetermined	Erebidae: Arctiinae	*Saurauia* sp.	Actinidiaceae
*G.mikeschauffi* sp. nov.	*Bertholdiaalbipuncta* Schaus	Erebidae: Arctiinae	*Drymoniamacrophylla*	Gesneriaceae
	*Bertholdiaspecularis* (Herrich-Schäffer)	Erebidae: Arctiinae	*Sabiceavillosa*	Rubiaceae
*G.mikesharkeyi* sp. nov.	Undetermined	Undetermined	Undetermined	Undetermined
*G.militaris* (Walsh)	*Mythimnaunipunctata* (Haworth)	Noctuidae: Noctuinae	*Zeamays*	Poaceae
		Noctuidae	*Poa* sp.	Poaceae
*G.montywoodi* sp. nov.	Undetermined	Erebidae: Arctiinae	*Chusqueascandens*	Poaceae
*G.muesebecki* (Blanchard)	*Mythimnaunipunctata* (Haworth)	Noctuidae: Noctuinae		
*G.nataliaivanovae* sp. nov.	Undetermined	Undetermined	Undetermined	Undetermined
*G.nealweberi* sp. nov.	*Rejectaria* sp.	Erebidae: Herminiinae	*Alsophilafirma*	Cyatheaceae
			*Cyatheamultiflora*	Cyatheaceae
			*Cyatheatrichiata*	Cyatheaceae
			*Serpocaulonmaritimum*	Polypodiaceae
	*Scopiferaantelia* Druce	Erebidae: Herminiinae	*Cyatheamultiflora*	Cyatheaceae
			*Cyatheatrichiata*	Cyatheaceae
*G.ninazitaniae* sp. nov.	Undetermined	Undetermined	Undetermined	Undetermined
*G.pachopinasi* sp. nov.	Undetermined	Noctuidae	*Acalypha* sp.	Euphorbiaceae
*G.pamitchellae* sp. nov.	Undetermined	Undetermined	Undetermined	Undetermined
*G.paulhansoni* sp. nov.	*Yidalptaauragalis* Guenée	Noctuidae: Catocalinae	*Securidacadiversifolia*	Polygalaceae
			*Securidacasylvestris*	Polygalaceae
*G.paulheberti* sp. nov.	*Disphragisproba* Schaus	Notodontidae: Heterocampinae	*Nectandrasalicifolia*	Lauraceae
			*Ocotealeucoxylon*	Lauraceae
*G.paulhurdi* sp. nov.	*Rosemaattenuata* (Dognin)	Notodontidae: Phalerinae	*Ingaoerstediana*	Fabaceae
*G.petermarzi* sp. nov.	Undetermined	Geometridae	Undetermined	Undetermined
*G.phildevriesi* sp. nov.	*Daedalmadinias* Hewitson	Nymphalidae: Satyrinae	*Chusqueascandens*	Poaceae
*G.philwardi* sp. nov.	Undetermined	Geometridae	*Pisoniaaculeata*	Nyctaginaceae
*G.rafamanitioi* sp. nov.	Undetermined	Noctuidae	*Chusqueascandens*	Poaceae
*G.robbinthorpi* sp. nov.	*Letismycerina* (Cramer)	Erebidae: Erebiinae	*Ingaoerstediana*	Fabaceae
			*Ingapunctata*	Fabaceae
*G.ronaldzunigai* sp. nov.	*Macarianundinata* Guenée	Geometridae: Ennominae	*Daleacarthagenensis*	Fabaceae
*G.roysnellingi* sp. nov.	Undetermined	Geometridae	*Bunchosiapolystachia*	Malpighiaceae
*G.scottmilleri* sp. nov.	*Metalectra* sp.	Noctuidae: Boletobiinae	Epiphytic microplants	Epiphytic microplants
*G.scottshawi* sp. nov.	Undetermined	Undetermined	Undetermined	Undetermined
*G.shelbystedenfeldae* sp. nov.	Undetermined	Undetermined	Undetermined	Undetermined
*G.sondrawardae* sp. nov.	Undetermined	Undetermined	Undetermined	Undetermined
*G.stephaniecluttsae* sp. nov.	*Bertholdiaalbipuncta* Schaus	Erebidae: Arctiinae	*Guazumaulmifolia*	Malvaceae
*G.stephaniekirkae* sp. nov.	Undetermined	Undetermined	Undetermined	Undetermined
*G.sujeevanratnasinghami* sp. nov.	*Psaliodes* sp.	Geometridae: Larentiinae	*Cyatheamultiflora*	Cyatheaceae
*G.suniae* sp. nov.	Undetermined	Erebidae: Arctiinae	Undetermined	Undetermined
*G.sureshnaiki* sp. nov.	Undetermined	Undetermined	Undetermined	Undetermined
*G.suzannegreenae* sp. nov.	Undetermined	Pyralidae	*Miconia* sp.	Melastomataceae
*G.sydneycameronae* sp. nov.	*Aleuroncarinate* (Walker)	Sphingidae: Macroglossinae	*Doliocarpusmultiflorus*	Dilleniaceae
	*Enyoocypete* (Linnaeus)	Sphingidae: Macroglossinae	*Doliocarpusmultiflorus*	Dilleniaceae
	*Pachygonidiadrucei* (Rothschild & Jordan)	Sphingidae: Macroglossinae	*Doliocarpusmultiflorus*	Dilleniaceae
*G.taniaariasae* sp. nov.	*Pantherodesunciaria* Guenée	Geometridae: Ennominae	*Boehmeriabullata*	Urticaceae
*G.tanyadapkeyae* sp. nov.	*Perochapela* Poole	Geometridae: Ennominae	*Anemopaegmaorbiculatum*	Bignoniaceae
*G.thibautdelsinnei* sp. nov.	Undetermined	Geometridae	*Chusqueascandens*	Poaceae
*G.thomaspapei* sp. nov.	Undetermined	Noctuidae	*Munnoziapinnatipartita*	Asteraceae
*G.toluagunbiadeae* sp. nov.	Undetermined	Noctuidae	*Miconia* sp.	Melastomataceae
*G.tomwallai* sp. nov.	Undetermined	Apatelodidae	*Dendrophorbiumlloense*	Asteraceae
	Undetermined	Erebidae: Arctiinae	*Baccharislatifolia*	Asteraceae
*G.victoriapookae* sp. nov.	*Paecteslunodes* Guenée	Euteliidae: Euteliinae	*Ocoteaveraguensis*	Lauraceae
*G.wilmersimbanai* sp. nov.	Undetermined	Apatelodidae	*Dendrophorbiumlloense*	Asteraceae
*G.wonyoungchoi* sp. nov.	*Antiblemmaceras* Druce	Erebidae: Eulepidotinae	*Conostegiaxalapensis*	Melastomataceae
*G.yalizhangae* sp. nov.	*Zanola* sp.	Apatelodidae	Undetermined	Asteraceae
			*Psammisiapauciflora*	Ericaceae
*G.yanayacuensis* sp. nov.	Undetermined	Noctuidae	Diplaziumcostalevar.robustum	Dryopteridaceae

Approximately 96% of the *Glyptapanteles* species with known host records parasitize a defined group of Lepidoptera, just a single host family or a narrower group, while a very small number (five species) use a slightly broader taxonomic range, parasitizing more than one Lepidoptera family [e.g., *G.andrewdebeveci* (Noctuidae and Pyralidae), *G.bobwhartoni* (Erebiidae and Saturniidae), *G.edwinnarvaezi* (Apatelodidae, Noctuidae, and Nymphalidae), *G.luchosalagaje* (Nymphalidae and Saturniidae), and *G.tomwallai* (Apatelodidae and Erebidae)]. All of these supposedly broader host ranges require more study before concluding that they are accurate, owing to potential errors in host caterpillar identification. However, misidentifications at the family level seem to have a low probability.

In total, 16 *Glyptapanteles* species were reared from more than one Lepidoptera species that belong to the same caterpillar family as well as the same subfamily: *G.alexborisenkoi* (2), *G.andydeansi* (3), *G.annettewalkerae* (2), *G.brianestjaquesae* (2), *G.charlesporteri* (2), *G.daveschindeli* (3), *G.donquickei* (2), *G.garygibsoni* (2), *G.ianyarrowi* (4), *G.jimmilleri* (3), *G.johnburnsi* (3), *G.johnnoyesi* (2), *G.lubomasneri* (2), *G.mikeschauffi* (2), *G.nealweberi* (2), and *G.sydneycameronae* (3). Only two parasitoid species emerged from hosts from different subfamilies within the same family: *G.bobkulai* attacks members of Sterrhinae and Larentiinae (Geometridae) and *G.ilarisaaksjarvi* specialized in Amphipyrinae and Plusiinae (Noctuidae) (Table 4). With six Noctuidae species hosts, *G.ilarisaaksjarvi* is the species with the greatest number of hosts recorded in this study.

Four duos and two trios of *Glyptapanteles* species share the same Lepidoptera host(s). Thus, *G.erictepei* and *G.haroldgreeneyi* have been reared from *Actinotestratonice* Latreille (Nymphalidae), *G.donquickei* and *G.ilarisaaksjarvi* have been reared from *Condicacupienta* (Cramer) (Noctuidae), *G.linghsiuae* and *G.luchosalagajei* have been reared from *Hypanartia* sp. Hübner (Nymphalidae), *G.sydneycameronae* and *G.andydeansi* have been reared from *Aleuroncarinata* (Walker), *Enyoocypete* (Linnaeus), and *Pachygonidiadrucei* (Rothschild & Jordan) (Sphingidae). *Glyptapantelesfelipesotoi*, *G.ferfernandezi*, and *G.genorodriguezae* have been reared from Memphisnr.lorna (Druce) (Nymphalidae), and *G.davesmithi*, *G.jamesrobertsoni*, and *G.jesusugaldei* have been reared from *Antiblemma* sp. Hübner (Erebidae). All these host records (and for every caterpillar) require more replications and additional scrutiny of host caterpillar identifications to be certain that they represent actual host affiliations.

It is worth mentioning that none of the species previously reported as Lepidoptera hosts in the scientific literature from the Neotropics were obtained by the rearing projects. Some plausible explanations for the lack of those records include: some Lepidoptera species occur naturally at low densities at study sites, time of foraging (early or late in the season) does not coincide with the collecting time, and larvae are well-camouflaged or semi-concealed (leafrollers, leaf tiers, shelter-building, grass moth, twig-like pose of some Geometridae) are even more difficult to spot in the field and, to a certain extent, never collected during censuses. Moreover, previous studies seem to be mostly restricted to field crops, not natural ecosystems. Additionally, some species are not reared successfully because natural conditions are difficult to replicate in the laboratory; caterpillars need special conditions or succumb to pathogens or other sources of mortality.

### Instars of Lepidoptera hosts

Lepidoptera hosts collected include caterpillars caught from eggs to last larval instar, and once caterpillars were collected, early instars (1–3) yielded more parasitoids than later instars. For an analysis of when oviposition occurs, and why, a totally different kind of study would need to be conducted.

### Egg-larval parasitoidism

Microgastrinae is a subfamily of specialized parasitoids because larval parasitism is assumed to be the dominant life history strategy. However, there are some exceptions. *Cotesiamarginiventris* (Cresson) (Ruberson and Whitfield 1996), *C.hyphantriae* (Riley) and *Diolcogaster* have the capacity to oviposit in both eggs and larvae, and emerge from the latter as last instar wasp larvae; this behavior may be facultative (Shaw and Huddleston 1991). In addition, morphological modifications found in the distal part of the ovipositor of *Rasivalva* Mason, another genus in the same subfamily (and related to *Diolcogaster*), suggest that parasitism of eggs is usual in that case (Shaw and Huddleston 1991).

Here, one species of *Glyptapanteles* was reared from oviposition in eggs. *Glyptapantelesjimmilleri* emerged from an undetermined species of dioptine Notodontidae whose eggs were collected on *Passifloraligularis* (Passifloraceae) leaves. The wasp species has been also reared from *Lycesfornax* Druce and *Josialigata* Walker (Notodontidae: Dioptinae) collected as eggs and as larvae in first, second, and fifth instars, and also feeding on *Passiflora* (Table 4). Thus, *G.jimmilleri* is the first *Glyptapanteles* species reported to be an egg-larval parasitoid. As noted above, almost all the Microgastrinae are endoparasitoids that feed and develop exclusively inside caterpillars. In this case of facultative egg-larval parasitism, the parasitoid waits in its egg until the host itself ecloses as a larva and only then begins to feed (Whitfield et al. 2018).

The subfamily Dioptinae is almost entirely Neotropical; only one of the 456 species described occurs on the USA, while the remaining taxa are found from Mexico south to northern Argentina and Uruguay. None is known from the Old World (Miller 2009). Unlike their relatives in other notodontid subfamilies, most dioptine adults are diurnal with aposematic color patterns and some of their larval food plants are toxic, such as nightshades (*Solanum* spp.) and passionflowers (*Passiflora* spp.) (Miller 2009). We note that *G.jimmilleri* is facultatively attacking eggs that are poorly defended immunologically in comparison to the larvae that will hatch from them, though obviously, the caterpillar must deal with the parasitoid, no matter how it gets into it. In addition, the dioptines oviposit aggregated eggs that may be easier to find than larvae that disperse in search of food (Shaw and Huddleston 1991).

### Hyperparasitoidism and multiparasitoidism

The frequency of hyperparasitoidism for *Glyptapanteles* was 4% (six of 127 species); hyperparasitoids were from one family of Hymenoptera: Ichneumonidae (*Mesochorus* Gravenhorst, Mesochorinae). Specimens of *Mesochorus* were reported as hyperparasitoids in six gregarious species: *G.ianyarrowi*, *G.jesusugaldei*, *G.jjrodriguezae*, *G.luchosalagajei*, *G.marcpolleti*, and *G.sydneycameronae*.

*Mesochorus* is a large genus of Ichenumonidae that attacks a broad range of hosts, including many species of Lepidoptera, Coleoptera (Yeargan and Braman 1989) and Hymenoptera, including Microgastrinae and Ichneumonidae. Besides *Glyptapanteles*, seven other Microgastrinae genera are hyperparasitized by *Mesochorus*: *Alphomelon* Mason, *Apanteles*, *Cotesia*, *Diolcogaster*, *Hypomicrogaster* Ashmead, *Microplitis* Förster, and *Parapanteles* Ashmead (Whitfield et al. 2009 and both of the current Neotropical inventories, unpublished data).

A case of multiparasitoidism was reported. A single lepidopteran host was attacked by more than one species of parasitoid. *Copidosomafloridanum* Ashmead (Chalcidoidea: Encyrtidae, Encyrtinae) and the gregarious species *G.ilarisaaksjarvi* emerged from a caterpillar of *Condicacupienta* (Cramer), a Noctuidae, Amphipyrinae feeding on *Mikaniamicranth* (Asteraceae). *Copidosomafloridanum*, is one of the few species of Hymenoptera that is polyembryonic (clonal production of multiple embryos from a single fertilized egg) and at the same time has evolved a caste system (Smith et al. 2017). The two castes that produces this species are know as reproductive larvae and soldier larvae. Reproductive caste larvae synchronously emerge during the host’s last instar, consume the host, and metamorphose into adult wasps (Gordon and Strand 2009). In contrast, soldier caste larvae defense against other parasitoids that attempt to develop in the same host, never molt, and die when their reproductive caste siblings consume the host and pupate (Grbic et al. 1992). The female of this polyembryonic species lays its eggs in the egg stage of *Trichoplusiani* (Hübner) (Noctuidae: Plusiinae). In contrast, larva stage of *T.ni* is parasitized by a microgastrine, *Microplitisdemolitor* (Smith et al. 2017).

Here, four other species of Noctuidae in the subfamily Plusiinae were reported as hosts of *G.ilarisaaksjarvi*: *Agraphaoxygramma* (Geyer) feeding on *Baccharistrinervis* (Asteraceae), *Argyrogrammabasigera* (Walker) feeding on *Hydrocotyleumbellate* (Araliaceae), *Argyrogrammaverruca* (F.) feeding on *Echinodorussubalatus* (Alismataceae), and soybean looper *Pseudoplusiaincludens* (Walker) feeding on *Milleriaquinqueflora* (Asteraceae).

### Behavior

Endoparasitoid wasp larvae live inside the caterpillars until they are ready to emerge. After leaving their hosts, the parasitoid larvae pupate in their own cocoons. During their larval development, some endoparasitoids consume most or all tissues of the host (after spending most instars only consuming hemolymph and fat body), whereas others consume a small fraction of host resources and either ensure that the host moves away from the pupation site or allow the host to remain close to the parasitoid cocoon(s) (Harvey et al. 2011).

For a few species of *Microplitis* (Microgastrinae) the host may carry on moving and feeding for up to weeks following parasitoid larval emergence (Strand et al. 1988, Quicke 1997). In four gregarious species here described, the caterpillar hosts continued living even after parasitoid emergence and when the caterpillars were disturbed (pinched) they did not try to bite the investigator’s fingers. Neither time of death after the emergence of parasitoids nor ability to continue feeding was recorded. These species are: *G.johnburnsi* attacks two species of Nymphalidae, *Eunicamalvina* (Bates) and *E.caresa* Hewitson; *G.chrisgrinteri* parasitizes one species of Noctuidae, *Lesmoneaemylia* (Druce); *G.corriemoreauae* attacks one species of Geometridae, *Euphyiacrispa* (Druce), and *G.garygibsoni* parasitizes two species of Notodontidae, *Nystaleacollaris* Schaus and *N.guzmani* Schaus.

### Usurpation hypothesis

The invariable consequence of parasitoidism for the host is to be killed by the parasitoid wasp of many higher taxa, as well as other kinds of parasitoids (fungi, flies, tapeworms, etc., Poulin and Randhawa 2015). However, it has been observed that some parasitoids modify the behaviour of their hosts, and that modification in the behavior is in place by the time the wasp larvae emerge from the caterpillar. A hypothesis known as the “usurpation hypothesis” was developed (Brodeur and Vet 1994) and describes a very commonly observed and frequently documented phenomenon of host exploitation strategies by the parasitoid. The parasitoid manipulates the host in such a way that the host guards the wasp larvae from hyperparasitoids, or from predators of the parasitoids (Harvey et al. 2008); thus, parasitoids may benefit from the retained defensive reflexes of the host. Following parasitoid emergence, the host caterpillar positions itself near the cocoons and undergoes a particular repertoire including a) ceasing feeding and walking, b) becoming the bodyguard defending the parasitoid cocoons by producing violent head swings against approaching predators or upon disturbance, c) regurgitating fluid from the gut, d) spinning protective thick silk webs over parasitoid cocoons, and e) dying before reaching adulthood (Grosman et al. 2008, Harvey et al. 2008). A few studies on wasp-caterpillar systems have revealed that parasitoid larvae can interfere with levels of juvenile hormone, ecdysteroids, and neurotransmitters (e.g., octopamine); the titer of these hormones increases shortly before the parasitoid(s) emerges from the host (Miles and Booker 2000), and continues to increase after parasitoid larval emergence. An elevated level of octopamine has been associated with the decline of caterpillar activity together with the decrease in the ability to digest food due to the absence or decrease of peristaltic activity in the foregut (Miles and Booker 2000), and they simply do not feed anymore.

This bodyguard behavior posited by the ‘usurpation hypothesis’ was frequently observed in many microgastrine genera by both inventories (e.g., *Apanteles*, *Cotesia*, *Microplitis*, *Snellenius*, *Xanthomicrogaster* Cameron, and others). Additionally, this has been reported for an undescribed species of *Glyptapanteles* from Brazil that attacks *Thyrinteinaleucocerae* (Rindge) (Geometridae) that feed on two Myrtaceae species: *Psidiumguajava* (guava), and *Eucalyptusgrandis* (eucalyptus) (Grosman et al. 2008) and *Cotesiaglomerata* (L.) attacking *Pierisbrassicae* (L.) (Pieridae) (Brodeur and Vet 1994, Harvey et al. 2008) or the tobacco hornworm *Manducasexta* (L.) (Sphingiidae) (Miles and Booker 2000). The behavior was observed in two gregarious *Glyptapanteles* species here described: *G.howelldalyi* that attacks caterpillar of *Dyopschromatophila* (Walker) (Noctuidae) that feed on *Coussapoanymphaeifolia* (Urticaceae) and *G.paulhansoni* that parasitizes caterpillars of *Yidalptaauragalis* Guenée (Noctuidae) that feed on two species of Polygalaceae: *Securidacadiversifolia* and *S.sylvestris.* The wild caterpillars were collected while still alive taking care of cocoons. When the caterpillars were touched by anything, they bit and attacked violently, eventually spitting up red gut contents on the attacker. The long setae of the caterpillars also impeded access to the cocoons.

### Cocoons (Fig. 4)

**Figure 4.**
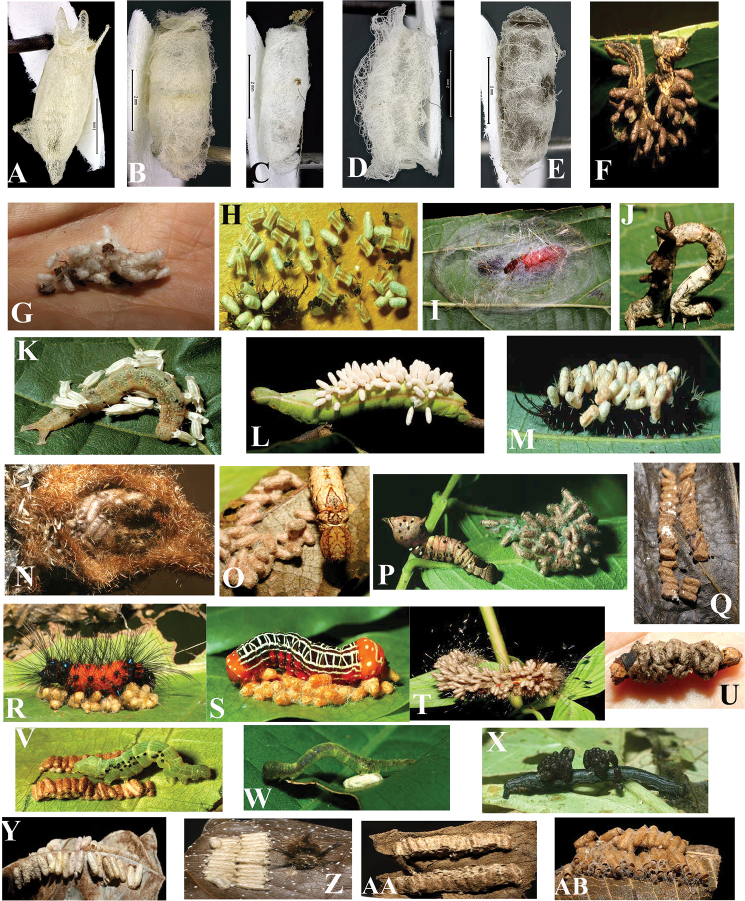
**J***G.tanyadapkeyae* sp. nov. parasitoid of *Perochapela* Poole: Geometridae, 08-SRNP-31435, photo DHJ440487 **K***G.bobboharti* sp. nov. parasitoid of *Anomisluridula* Guenée: Noctuidae, 01-SRNP-21185, photo DHJ62275 **L***G.andybennetti* sp. nov. parasitoid of *Aleuroniphis* (Walker): Sphingidae, 08-SRNP-32177, photo DHJ445890 **N***G.charlesporteri* sp. nov. parasitoid of *Tarchonfelderi* Druce: Apatelodinae, 04-SRNP-3328, photo DHJ420376 **O***G.eowilsoni* sp. nov. parasitoid of *Calledemaplusia* Felder: Noctuidae, 09-SRNP-71063, photo DHJ461044 **P***G.garygibsoni* sp. nov. parasitoid of *Nystaleacollaris* Schaus: Noctuidae, 82-SRNP-418, photo DHJ4186 **Q***G.chrisdarlingi* sp. nov. parasitoid of *Concana* sp. Walker: Noctuidae, 06-SRNP-4972, photo DHJ436488 **R***G.gerarddelvarei* sp. nov. parasitoid of *Macrocnemecabimensis* Dyar: Erebidae, 07-SRNP-32365, photo DHJ421485 **S***G.henrytownesi* sp. nov. parasitoid of *Heterochromasarepta* (Druce): Noctuidae, 97-SRNP-990, photo DHJ40659 **T** G. iangauldi sp. nov. parasitoid of *Zanolaverago* Cramer: Apatelodinae, 06-SRNP-9671, photo DHJ424751 U *G.daveschindeli* sp. nov. parasitoid of *Oxydiavesulia* (Cramer): Geometridae, 08-SRNP-16708, photo DHJ452329 **V***G.ilarisaaksjarvi* sp. nov. parasitoid of *Argyrogrammaverruca* (F.): Noctuidae, 92-SRNP-6132, photo DHJ16975 **W***G.johnnoyesi* sp. nov. parasitoid of *Deinopasigniplena* Walker: Erebidae, 05-SRNP-31619, photo DHJ404219 **X***G.corriemoreauae* sp. nov. parasitoid of *Euphyiacrispa* Druce: Geometridae, 03-SRNP-23245, photo DHJ78940 **Y***G.jeremydewaardi* sp. nov. parasitoid of *Antiblemmasp*. Hübner: Erebidae, 06-SRNP-35622, photo DHJ467720 **Z***G.mehrdadhajibabaei* sp. nov. parasitoid of *Carathisseptentrionalis* Becker: Erebidae, 06-SRNP-3399, photo DHJ436471 **AA***G.donquickei* sp. nov. parasitoid of *Condicafunereal* (Schaus): Noctuidae, 09-SRNP-43316, photo DHJ476509 **AB***G.howelldalyi* sp. nov. parasitoid of *Dyopschromatophila* Walker: Noctuidae, 05-SRNP-6986, photo DHJ424017.

The cocoons recorded here commonly exhibit pale coloration (white and beige) although dark coloration (dark gray and black) is recorded for some species and white cocoons with dark spots throughout are infrequent (e.g., *G.thomaspapei*, Fig. 4E). *Glyptapanteles* species exhibit a diverse set of behaviors to spin cocoons. Shape, ornamentation, and location are highly distinctive and often species-specific. The function of those cocoons seems to be to protect the pupae from the weather (desiccation, water damage, and rapid temperature shifts) and reduce the risk of attack by natural enemies (Harvey et al. 2011). Some species of parasitoid wasps (Ichneumonidae) are able to adjust the investment in cocoon silk according to the environmental conditions. The thickness of the cocoon wall varies from thin in summer generations to thick in overwintering wasps from temperate regions (Tagawa and Sato 2009). The cocoons are made of dense silk threads produced by the labial glands. The strands are twisted together making them difficult to penetrate. In this study, at least seven major kinds of cocoons and cocoon masses were observed: oval, rings, lace-shaped, bud-like, drum-shaped, single row of cordwood, and two rows of cordwood.

**Shapes**

***Oval*** (Fig. 4B, C, E). This is the simplest cocoon shape. Those cocoons lack any kind of remarkable ornament. Sometimes the silk fibers are compact and neatly arranged and at other times the silk threads look disordered and fluffy.

***Rings*** (Fig. 4U). An unusual disposition, only observed in *G.daveschindeli*, where cocoons were adhered laterally against the caterpillar’s body in rings.

***Lace-shaped cocoon*** (Fig. 4D). A strange cocoon design which was observed in two species. In cocoons of *G.montywoodi* and *G.rafamanitioi* the exit hole, as well as the opposite end, are surrounded by a wavy lacy fringe, and although the body of the cocoon also displays the same kind of ornament, it is arranged in such a way that it forms three parallel lines running from the exit hole to the opposite end.

***Bud-like cocoon*** (Fig. 4A, K). A peculiar architecture which was observed in some cocoons. Appendages in both ends of cocoons appear whereas the cocooned body lacks any kind of ornamentation. The exit hole is surrounded by a crown of elongated lobes while the opposite end is embellished with one elongate lobe at each side. Fifteen species exhibited those adornments: *G.betogarciai*, *G.bobhanneri*, *G.bobkulai*, *G.bobwhartoni*, *G.boharti*, *G.brianestjaquesae*, *G.carinachicaizae*, *G.carlhuffakeri*, *G.dorislagosae*, *G.edgardpalacioi*, *G.erictepei*, *G.haroldgreeneyi*, *G.lubomasneri*, *G.paulheberti*, and *G.paulhurdi*. The purpose of those bud-like cocoons (Koptur 1989) is to avoid perhaps visually oriented vertebrate predators as foliage-gleaning birds (Greenberg and Gradwohl 1980) or crawling predators. Falling away from the dead host, associated with white coloration as well as mimicry a flower could help parasitoid cocoons to be unnoticed or seen as fallen flower buds by birds (Koptur 1989). The hosts’ food plants of the fifteen species above mentioned encompassing a wide variety of families: Asteraceae, Ericaceae, Fabaceae, Lauraceae, Malvaceae, Melastomataceae, Piperaceae, Poaceae, Sapindaceae, Solanaceae, and Violaceae (Table 4).

***Drum-shaped cocoon*** (Fig. 4H, M). Two kinds of cocoons were observed in *G.johnburnsi*. In this gregarious species, single oval cocoons are somewhat separate from one another and individually adhered to the larval cuticle. However, among them, a few cocoons exhibit a drum-shaped form which never eclose because there is no pupa inside and it is unclear as to whom they belong or why these sterile cocoons are formed.

***Single row of cordwood*** (Fig. 4Q, Y, Z). In this arrangement, the long chain of irregularly oval cocoons is located along the side of the cadaver of the caterpillar, but not forming double cordwood, so cocoons are adhered to the leaf substrate (e.g., *G.charlesmichener*, *G.chrisgrinteri*).

***Two rows of cordwood*** (Fig. 4R, S, V, AA, AB). Some species form two parallel rows of cordwood cocoons strongly adhered to each other or sometimes well apart. The caterpillar cadaver always is located in the middle and cocoons adhered to the leaf substrate. This arrangement was observed in *G.carlossarmientoi*, *G.carlrettenmeyeri*, *G.chrisdarlingi*, *G.davesmithi*, *G.donquickei*, and *G.eowilsoni*.

The characteristics of the common mass of cocoons clearly may serve as a potential tool for identifying reared species. For example, *Cotesiaphobetri* (Rohwer) and *C.halisidotae* (Muesebeck) are species morphologically similar as adults, both attack Arctiinae in the eastern United States; however, their cocoon masses associated with host remains are distinct. *Cotesiaphobetri* kills the host larva before it reaches the last instar and forms the cocoon in an irregular mass on the back of the caterpillar. In contrast, *C.halisidotae* waits until the host spins its dark cocoon and then emerge, forming their cocoon inside the host cocoon. Discarding cocoons of reared parasitoids or dissecting the cocoon mass to put one cocoon with each point-mounted parasitoid causes an unfortunate loss of useful information (Mason 1981).

Another variation occurring is in the places where the cocoons are woven. *Glyptapanteles* species can weave cocoons either on the host, hosts’ food plants or in the soil after larval emergence. Some *Glyptapanteles* construct a mass of somewhat separate white cocoons completely filling the caterpillar cocoon (e.g., *G.alvarowillei* and *G.charlesporteri*, Fig. 4N). In contrast, other cocoons were found in the soil or litter or attached to the leaf substrate (Fig. 4I, O–S, V–W, Y–AB) (e.g., *G.bobkulai*, *G.bobwhartoni*, *G.boharti*, and *G.carlhuffakeri*). In many species, as in other genera of Microgastrinae (e.g., *Apanteles*, *Cotesia*, *Hypomicrogaster*, *Microplitis*, and others), the cocoons have tightly adhered to the host larval cuticle (Fig. 4F, L, M, T, U, X).

### Food plants (Table 4)

Lepidopteran hosts were reared from 60 families of Angiosperms (147 species within 118 genera) according to the rearing databases. In the case of 14 *Glyptapanteles* species, the data do not clearly associate the food plants (neither species nor family) on which the caterpillar(s) feed. Of those, nine species correspond to the above-mentioned Malaise-trapped specimens: *G.mikesharkeyi*, *G.nataliaivanovae*, *G.ninazitaniae*, *G.pamitchellae*, *G.scottshawi*, *G.shelbystedenfeldae*, *G.sondrawardae*, *G.stephaniekirkae*, and *G.sureshnaiki*. For the other five species, although they were collected from reared material, plant names were not recorded: *G.daveroubiki*, *G.jacklonginoi*, *G.petermarzi*, *G.philwardi*, and *G.suniae*. Two species, *G.ankitaguptae* and *G.betogarciai*, were reared on undetermined Geometridae larvae collected from undetermined Pteridophyta. Additionally, *G.scottmilleri* was reared from *Metalectra* Hübner (Noctuidae: Boletobiinae), the caterpillars of which were collected feeding on undetermined epiphytic microplants. When comparing the quantity of food plants with herbivore hosts, the number of families, genera, and species is higher in the former. The extensive list of plants, plant-feeding insects and their associated parasitoids here provided is a basis for the future understanding of one of the most complex and species-rich food webs.

## Taxonomic accounts

### Glyptapanteles

Taxon classificationAnimaliaHymenopteraBraconidae

Ashmead, 1904

#### Type species.

*Glyptapantelesmanilae*Ashmead 1904 = *Apantelesashmeadi* Wilkinson 1928.

#### Diagnosis.

Hypopygium of female evenly sclerotized from side to side, never with a series of parallel longitudinal creases. Ovipositor sheath short and mostly concealed by hypopygium, its length not more than half of the hind tibia (rarely longer, but if so hypopygium is large and acutely pointed, concealing most of the ovipositor), sheaths dagger-shaped with only a few setae concentrated near the apex. Petiole on T1 never wider at the apex, the sides either gradually converging distally or parallel and strongly rounded to the apex. The median area on T2 broadening distally and often subtrapezoidal or truncate-trapezoidal, sometimes lateral grooves delimiting the median area are lost among many diverging aciculations and sometimes do not reach the proximal edge of T3; T3 always smooth. Propodeum usually completely or mostly smooth, but often with coarsely sculpture covering all or part of the surface; rarely with a median longitudinal carina, but never with even a trace of the areola. Fore wing with r-m vein absent, so that the small areolet is open distally. Distal half of margin of vannal lobe of hind wing convex or flattened, with or without a fringe of setae. The anterior furrow of metanotum flattened (without sublateral setiferous projections) and glabrous; scutellar phragma exposed or concealed (Mason 1981).

A dataset of 126 characters and 484 character-states was evaluated for each species and was used to provide uniformity for all species descriptions. In total, 20 character-states were found in all the species and they have therefore not been taken into account in the descriptions. They are: *Head*: pubescence on the head long and dense, placodes of proximal antennal flagellomeres arranged in two ranks, apex of distal antennal flagellomere pointed, antennal scrobes-frons shallow, toruli more than half total eye length, carina surrounding antennal scrobes and fronto-clypeal suture both absent, vertex medially convex and slightly dented, and occiput concave. Although the length of the three proximal antennal flagellomeres was always longer than wide it was conserved in the descriptions and their length was provided. *Mesosoma*: mesosoma dorso-ventrally convex, mesoscutum relatively setose, notauli absent, lunules smooth and semicircular, pleural suture in the metapleuron absent although a dark groove is visible, and median fovea in metapleuron present. *Legs*: antennal cleaner apparatus with a forked spur at the fore tibial apex and proximal weak emargination on fore basitarsus. *Wings*: hind wing with spectral junction among 2RS, 2M and (RS+M)b veins. *Metasoma*: Antero-median depression on petiole present, and pubescence adjacent to spiracle on T1 dense.

##### Key to *Glyptapanteles* species from Costa Rica and Ecuador

**Table:** 

1	Fore wing with outer side of junction of r and 2RS veins forming a slight or distinct stub (Figs 16N, 22L, 96L, 139K, 150L)	**2**
–	Fore wing with outer side of junction of r and 2RS veins not forming a stub (Figs 92J, 136L, 168L, 186K, 222K)	**75**
2	Fore wing with r vein straight (Figs 44G, 70K, 81C, 137K, 216K)	**3**
–	Fore wing with r vein slightly curved or curved (Figs 75L, 115K, 140K, 213L, 215L)	**20**
3(2)	Lateral grooves delimiting the median area on T2 clearly defined and reaching the distal edge of T2 (Fig. 7G)	**4**
–	Lateral grooves delimiting the median area on T2 distally losing definition on T2	**5**
4(3)	Scutellar punctation distinct throughout (Figs 7F, 8F); area just behind transscutal articulation with same kind of sculpture as mesoscutum (Figs 7E, 8E); inner margin of eyes diverging slightly at antennal sockets (Figs 7B, 8B); vertex in dorsal view wide (Figs 7D, 8D)	***G* . *alexborisenkoi* Arias-Penna, sp. nov.**
–	Scutellar punctation distinct peripherally, absent centrally (Fig. 16G); area just behind transscutal articulation smooth and shiny (Figs 16G, H, 17F); inner margin of eyes straight throughout (Fig. 16B); vertex in dorsal view narrow (Figs 16D, 17C)	***G* . *andydeansi* Arias-Penna, sp. nov.**
5(3)	Petiole variously sculptured (finely sculptured, with one type of sculpture or with a mix of sculptures)	**6**
–	Petiole completely smooth and polished, with faint, satin-like sheen (Figs 44D, 45D, G)	***G* . *carlossarmientoi* Arias-Penna, sp. nov.**
6(5)	Petiole with one type of sculpture: finely sculptured or with rugae	**7**
–	Petiole with a mix of sculptures: finely rugulate and punctate (Fig. 137G, H)	***G* . *jumamuturii* Arias-Penna, sp. nov.**
7(6)	Petiole finely sculptured (Figs 80H, 81G, 142D, 143G)	**8**
–	Petiole with rugae (Figs 138G, H)	***G* . *keithwillmotti* Arias-Penna, sp. nov.**
8(7)	Propodeum with a median longitudinal dent (Figs 80G, 81F)	**9**
–	Propodeum without a median longitudinal dent	**10**
9(8)	Precoxal groove smooth and shiny (Figs 80I, 81H); scutellar punctation scattered throughout (Figs 80F, 81E); vertex in dorsal view wide (Fig. 80C); mesoscutum punctation distinct throughout (Figs 80F, 81E); T3 as long as T2 (Figs 80K, 81G)	***G* . *erictepei* Arias-Penna, sp. nov.**
–	Precoxal groove with transverse lineate sculpture (Fig. 217A, I); scutellar punctation distinct peripherally, absent centrally (Fig. 217F); vertex in dorsal view quite wide; mesoscutum punctation distinct proximally but absent/dispersed distally (Fig. 217E); T3 longer than T2 (Fig. 217H)	***G* . *toluagunbiadeae* Arias-Penna, sp. nov.**
10(8)	Petiole parallel-sided in proximal half or 3/4 (Figs 90G, 94G, 216H)	**11**
–	Petiole evenly narrowing distally (wide base to a narrow apex, Figs 70G, 119G, 212G)	**14**
11(10)	Petiole on T1 parallel-sided in proximal half, then narrowing (gradually or not, Fig. 90G)	**12**
–	Petiole on T1 virtually parallel-sided over most of length but narrowing over distal 1/3 (Fig. 216H) or apex	**13**
12(11)	Vertex in lateral view rounded (Fig. 90C); frons punctate; scutellar punctation scattered throughout (Figs 90E, 91B); in lateral view, metasoma curved (Figs 90A, 91A); median area on T2 as broad as long (Figs 90G, 91F)	***G* . *genorodriguezae* Arias-Penna, sp. nov.**
–	Vertex in lateral view pointed (Fig. 94C); frons smooth; scutellar punctation indistinct throughout (Fig. 94E); in lateral view, metasoma laterally compressed (Fig. 94A); median area on T2 broader than long (Fig. 94G)	***G* . *grantgentryi* Arias-Penna, sp. nov.**
13(11)	Inner margin of eyes straight throughout; medioanterior pit of metanotum circular without median longitudinal carina (Fig. 40D); mesoscutum punctation distinct throughout (Figs 39B, 40B); phragma of the scutellum partially exposed (Figs 39E, 40D)	***G* . *brianestjaquesae* Arias-Penna, sp. nov.**
–	Inner margin of eyes diverging slightly at antennal sockets (Fig. 216B); medioanterior pit of metanotum circular with a short proximal carina (Fig. 216G); mesoscutum punctation proximally distinct, but distally absent/dispersed (Fig. 216F); phragma of the scutellum widely visible (Fig. 216G)	***G* . *thomaspapei* Arias-Penna, sp. nov.**
14(10)	In lateral view, metasoma laterally compressed; T3 longer than T2; inner margin of eyes diverging slightly at antennal sockets	**15**
–	In lateral view, metasoma curved (Figs 70A, 71A); T3 as long as T2 (Figs 70H, 71D); inner margin of eyes straight throughout (Fig. 70B)	***G* . *diegocamposi* Arias-Penna, sp. nov.**
15(14)	Edges of median area on T2 obscured by weak longitudinal stripes	**16**
–	Edges of median area on T2 polished and followed by a deep groove (Fig. 212G)	***G.sydneycameronae* Arias-Penna, sp. nov.**
16(15)	Median area on T2 broader than long; vertex in dorsal view wide	**17**
–	Median area on T2 as broad as long (Figs 118H, 119G); vertex in dorsal view quite wide (Figs 118D, 119D)	***G.jerrypowelli* Arias-Penna, sp. nov.**
17(16)	Fore wing with vein 2 cu-a absent	**18**
–	Fore wing with vein 2 cu-a present as spectral vein, sometimes difficult to see	**19**
18(17)	Fore telotarsus almost same width throughout, ventral margin without seta; medioposterior band of scutellum only very partially overlapping the medioanterior pit of metanotum (Figs 142C, 143F); phragma of the scutellum partially exposed (Figs 142B, 143F)	***G* . *lubomasneri* Arias-Penna, sp. nov.**
–	Fore telotarsus basally narrow, apically wide, ventral margin with a tiny curved seta; medioposterior band of scutellum not overlapping the medioanterior pit of metanotum (Fig. 159B, C); phragma of the scutellum widely visible (Figs 158B, 159C)	***G.meganmiltonae* Arias-Penna, sp. nov.**
19(17)	Hind coxa with dorsal half sparsely punctate, ventral half densely punctate (Fig. 35J); antenna shorter than body; distal antennal flagellomere subequal in length with penultimate; scutellar punctation distinct peripherally, absent centrally (Figs 35I, 36E)	***G.bobwhartoni* Arias-Penna, sp. nov.**
–	Hind coxa finely punctate throughout (Fig. 95J); antenna longer than body; distal antennal flagellomere longer than penultimate; scutellar punctation scattered throughout (Fig. 95E, F)	***G.gunnarbrehmi* Arias-Penna, sp. nov.**
20(2)	Edges of median area on T2 obscured by sculpture (longitudinal stripes, coarse sculpture or finely sculptured, Figs 37I, 75H, 141G)	**21**
–	Edges of median area on T2 polished and followed by a deep groove (Figs 20G, 56D, 218G)	**49**
21(20)	Dorsal outer depression on hind coxa absent (Figs 37D, 139J, 141I)	**22**
–	Dorsal outer depression on hind coxa present (Figs 135K, 144J, 162J)	**33**
22(21)	Edges of median area on T2 obscured by sculptures (Figs 37I, 141G)	**23**
–	Edges of median area on T2 obscured by weak longitudinal stripes (Figs 33I, 42G, 140G)	**24**
23(22)	Medioanterior pit of metanotum circular without median longitudinal carina (Fig. 37G); edges of median area on T2 with little sculpture (Figs 37I, 38I); scutellar punctation distinct peripherally, absent centrally (Figs 37G, 38G); in lateral view, metasoma laterally compressed (Figs 37A, 38A)	***G* . *boharti* Arias-Penna, sp. nov.**
–	Medioanterior pit of metanotum circular and bisected by a median longitudinal carina (Fig. 141F); edges of median area on T2 obscured by coarse sculpture (Fig. 141G); scutellar punctation scattered throughout (Fig. 141E); in lateral view, metasoma curved (Fig. 141A)	***G.linghsiuae* Arias-Penna, sp. nov.**
24(22)	Surface of metasternum convex (Fig. 5F)	**25**
–	Surface of metasternum flat or nearly so (Figs 31H, 32H)	**28**
25(24)	Propleuron with fine punctations throughout; longitudinal median carina on face present	**26**
–	Propleuron finely sculptured only ventrally (Fig. 139A, C); longitudinal median carina on face absent (Fig. 139B)	***G* . *kevinjohnsoni* Arias-Penna, sp. nov.**
26(25)	Ventral margin of fore telotarsus entire without seta; anteroventral contour of mesopleuron straight/angulate or nearly so	**27**
–	Ventral margin of fore telotarsus slightly excavated and with a tiny curved seta; anteroventral contour of mesopleuron convex (Fig. 165A, I)	***G* . *mikepoguei* Arias-Penna, sp. nov.**
27(26)	Medioanterior pit of metanotum elongated with some sculpture inside and not covered by medioposterior band of scutellum (Fig. 74G); transscutal articulation with small homogeneous carinated foveae (Fig. 74F); inner margin of eyes straight throughout; median area on T2 as broad as long (Fig. 74H, K)	***G* . *dorislagosae* Arias-Penna, sp. nov.**
–	Medioanterior pit of metanotum circular without median longitudinal carina and very partially covered by medioposterior band of scutellum (Fig. 140F); transscutal articulation with tiny homogeneous foveae without carina (Fig. 140E); inner margin of eyes diverging slightly at antennal sockets (Fig. 140B); median area on T2 broader than long (Fig. 140G, H)	***G* . *kyleparksi* Arias-Penna, sp. nov.**
28(24)	Petiole on T1 virtually parallel-sided but narrowing over distal 1/3 or at apex	**29**
–	Petiole on T1 evenly narrowing along its length (wide base to a narrow apex)	**30**
29(28)	In lateral view scutellum slightly higher than mesoscutum (Figs 10A, 11A); T3 as longer as T2; longitudinal median carina on face absent (Fig. 11B); antenna shorter than body; distal antennal flagellomere longer than penultimate	***G* . *alvarowillei* Arias-Penna, sp. nov.**
–	In lateral view scutellum on same plane as mesoscutum (Fig. 42A); T3 longer than T2 (Fig. 42H); longitudinal median carina on face present (Fig. 42B); antenna longer than body; distal antennal flagellomere subequal in length with penultimate	***G* . *carlhuffakeri* Arias-Penna, sp. nov.**
30(28)	Scutellum sculptured; medioposterior band of scutellum not overlapping or only very partially overlapping the medioanterior pit of metanotum	**31**
–	Scutellum shiny smooth (Fig. 96F); medioposterior band of scutellum mostly overlapping the medioanterior pit of metanotum (Fig. 96G)	***G* . *haroldgreeneyi* Arias-Penna, sp. nov.**
31(30)	Scutellar punctation distinct throughout (Figs 23G, 26D); fore wing with tubular vein 1 cu-a incomplete/broken, not reaching the edge of 1-1A vein (Figs 23L, 26K)	**32**
–	Scutellar punctation distinct peripherally, absent centrally (Figs 33G, 34F); fore wing with tubular vein 1 cu-a complete, touching the edge of 1-1A vein (Figs 33L, 34K)	***G* . *bobkulai* Arias-Penna, sp. nov.**
32(31)	Propleuron with fine punctations throughout (Fig. 23C, J); axillary trough of metanotum with undulate carinae throughout (Figs 23G, 24C); medioposterior band of scutellum not overlapping the medioanterior pit of metanotum (Figs 23F, 24C); longitudinal median carina on face absent (Fig. 23B); inner margin of eyes diverging slightly at antennal sockets (Fig. 23B)	***G* . *annettewalkerae* Arias-Penna, sp. nov.**
–	Propleuron with fine punctations only ventrally (Figs 25A, 26A, B); axillary trough of metanotum proximally with undulate carina, distally smooth (Figs 25F, 26D); medioposterior band of scutellum only very partially overlapping the medioanterior pit of metanotum (Figs 25F, 26D); longitudinal median carina on face present (Figs 25B, 26A); inner margin of eyes straight throughout	***G* . *barneyburksi* Arias-Penna, sp. nov.**
33(21)	Petiole parallel-sided in proximal half, or over distal 1/3 or at apex then narrowing (gradually or not)	**34**
–	Petiole evenly narrowing distally (wide base to a narrow apex)	**43**
34(33)	Edges of median area on T2 with little sculpture	**35**
–	Edges of median area on T2 obscured by longitudinal stripes	**36**
35(34)	In lateral view, metasoma curved (Fig. 144A, J); hind coxa medium-size punctate throughout (Fig. 144A, J); antenna longer than body; scutellar punctation distinct peripherally, absent centrally (Fig. 144E, F)	***G* . *luchosalagajei* Arias-Penna, sp. nov.**
–	In lateral view, metasoma laterally compressed (Fig. 145A, J); hind coxa very finely punctate throughout (Fig. 145A, J); antenna shorter than body; scutellar punctation scattered throughout (Fig. 145E, F)	***G* . *malleyneae* Arias-Penna, sp. nov.**
36(34)	Edges of median area on T2 obscured by strong longitudinal stripes (Figs 135H, 149G)	**37**
–	Edges of median area on T2 obscured by weak longitudinal stripes (Figs 84G, 162G)	**38**
37(36)	Petiole virtually parallel-sided, but narrowing over distal 1/3 (Fig. 135H, I); distal edge on T2 straight (Fig. 135H, I); lateral grooves delimiting the median area on T2 clearly defined and reaching the distal edge of T2 (Fig. 135H, I); T3 longer than T2 (Fig. 135I); distal antennal flagellomere longer than penultimate; mesoscutum punctation distinct throughout (Fig. 135F); in lateral view, metasoma curved (Fig. 135A, K)	***G* . *josesimbanai* Arias-Penna, sp. nov.**
–	Petiole proximal half straight and distal half convex (Fig. 149G); distal edge on T2 slightly convex (Fig. 149G, H); lateral grooves delimiting the median area on T2 distally losing definition (Fig. 149G); T3 as long as T2 (Fig. 149H); distal antennal flagellomere subequal in length with penultimate; mesoscutum punctation proximally distinct, but distally absent/dispersed (Fig. 149E); in lateral view, metasoma laterally compressed (Fig. 149A, J)	***G* . *marcelotavaresi* Arias-Penna, sp. nov.**
38(36)	Petiole virtually parallel-sided, but narrowing over distal 1/3 or at apex	**39**
–	Petiole parallel-sided in proximal half, then narrowing (gradually or not)	**40**
39(38)	In lateral view, metasoma curved (Fig. 148A, J); hind coxa very finely punctate throughout (Fig. 148A, J); propodeum without a transverse discontinuous carina (Fig. 148F); petiole virtually parallel-sided, but narrowing over distal 1/3 (Fig. 148G, H); scutellar punctation scattered throughout (Fig. 148E)	***G.mamiae* Arias-Penna, sp. nov.**
–	In lateral view, metasoma cylindrical (Fig. 162A, J); hind coxa punctate only on ventral surface (Fig. 162A, J); propodeum with a transverse discontinuous carina only present laterally (Fig. 162F); petiole virtually parallel-sided, but narrowing at apex (Fig. 162G, H); scutellar punctation indistinct throughout (Fig. 162E, F)	***G.michelleduennesae* Arias-Penna, sp. nov.**
40(38)	Anterior furrow of metanotum without setiferous lobes (Fig. 84F)	**41**
–	Anterior furrow of metanotum with a small lobe (without setae, Fig. 150G)	**42**
41(40)	Distal half of propodeum rugose (Figs 82F, 83C); precoxal groove indistinct (Figs 82I, 83E); on pronotum central area smooth, but both dorsal and ventral furrows with short parallel carinae (Figs 82C, 83E)	***G* . *felipesotoi* Arias-Penna, sp. nov.**
–	Distal half of propodeum with a mix of coarse sculpture and rugae (Figs 84F, 85C); precoxal groove deep (Figs 84H, 85D); on pronotum central area and dorsal furrow smooth, but ventral furrow with short parallel carinae (Figs 84C, 85D)	***G* . *ferfernandezi* Arias-Penna, sp. nov.**
42(40)	Malar suture present (Fig. 97B); median area between lateral ocelli without depression (Fig. 97D); propodeum medially rhomboid-shaped with transverse rugae (Fig. 97F); scutellar punctation indistinct throughout (Fig. 97E, F); axillary trough of metanotum proximally with a groove with some sculpturing, distally with rugae (Fig. 97E, F)	***G* . *helmuthaguirrei* Arias-Penna, sp. nov.**
–	Malar suture absent or difficult to see (Fig. 150B); median area between lateral ocelli slightly depressed (Fig. 150D); propodeum with a median longitudinal dent, but no trace of median longitudinal carina (Fig. 150G); scutellar punctation distinct throughout (Fig. 150F, G); axillary trough of metanotum proximally with sculpture, but dorsally without a well delimited smooth area (Fig. 150F, G)	***G.marcepsteini* Arias-Penna, sp. nov.**
43(33)	In dorsal view, proximal half of propodeum weakly curved	**44**
–	In dorsal view, proximal half of propodeum more strongly curved	**46**
44(43)	Antenna longer than body; scutellum in profile flat and on same plane as mesoscutum	**45**
–	Antenna as same length as body; scutellum in profile slightly convex, but on same plane as mesoscutum (Figs 29I, 30G)	***G* . *billbrowni* Arias-Penna, sp. nov.**
45(44)	Vertex in lateral view rounded (Fig. 41C); dorsal carina delimiting a dorsal furrow on propleuron absent (Fig. 41A, J); inner margin of eyes straight throughout (Fig. 41B); fore wing with vein 2-1A tubular throughout (Fig. 41L); median area on T2 broader than long (Fig. 41H, I)	***G* . *carinachicaizae* Arias-Penna, sp. nov.**
–	Vertex in lateral view pointed or nearly so (Fig. 184C); dorsal carina delimiting a dorsal furrow on propleuron present; inner margin of eyes diverging slightly at antennal sockets (Fig. 184B); fore wing with vein 2-1A proximally tubular, distally spectral (Fig. 184K); median area on T2 as broad as long (Figs 184H, 185D)	***G* . *paulhurdi* Arias-Penna, sp. nov.**
46(43)	Distal antennal flagellomere longer than penultimate; median area between lateral ocelli without depression	**47**
–	Distal antennal flagellomere subequal in length with penultimate; median area between lateral ocelli slightly depressed (Fig. 182C)	***G* . *paulheberti* Arias-Penna, sp. nov.**
47(46)	Propodeal spiracle distally framed by faintly concave/wavy carina; inner margin of eyes diverging slightly at antennal sockets	**48**
–	Propodeal spiracle without distal carina (Fig. 75G); inner margin of eyes straight throughout (Fig. 75B)	***G*** . ***edgardpalacioi* Arias-Penna, sp. nov.**
48(47)	Surface of metasternum flat or nearly so; nucha surrounded by very short radiating carinae (Figs 76F, 77C); median area on T2 broader than long (Figs 76G, 77D)	***G* . *edwinnarvaezi* Arias-Penna, sp. nov.**
–	Surface of metasternum convex; nucha surrounded by long radiating carinae (Figs 58F, 59G); median area on T2 as broad as long (Figs 58G, 59E)	***G.claudiamartinezae* Arias-Penna, sp. nov.**
49(20)	Anteroventral contour of mesopleuron straight/angulate or nearly so (Figs 12J, 22J, 204J)	**50**
–	Anteroventral contour of mesopleuron convex (Figs 151I, 156I)	**64**
50(49)	Precoxal groove shallow, but visible (Figs 20I, 204J)	**51**
–	Precoxal groove deep (Figs 12J, 210I)	**53**
51(50)	Lateral grooves delimiting the median area on T2 clearly defined and reaching the distal edge of T2; axillary trough of metanotum proximally with semircular/undulate carina, distally smooth	**52**
–	Lateral grooves delimiting the median area on T2 distally losing definition (Figs 204H, 205D); axillary trough of metanotum completely smooth (Figs 204G, 205C)	***G* . *stephaniecluttsae* Arias-Penna, sp. nov.**
52(51)	Distal antennal flagellomere longer than penultimate; posterior ocelar line shorter than ocular ocelar line; mesoscutum punctation distinct throughout (Figs 20E, 21B)	***G* . *andywarreni* Arias-Penna, sp. nov.**
–	Distal antennal flagellomere subequal in length with penultimate; posterior ocelar line broader than ocular ocelar line (Fig. 22D); mesoscutum punctation proximally distinct, but distally absent/dispersed (Fig. 22F)	***G* . *ankitaguptae* Arias-Penna, sp. nov.**
53(50)	Petiole parallel-sided in proximal half then narrowing or parallel-sided, but narrowing over distal 1/3 or at apex	**54**
–	Petiole evenly narrowing throughout length (Figs 12H, I, 13F)	***G* . *andrewdebeveci* Arias-Penna, sp. nov.**
54(53)	Dorsal carina delimiting a dorsal furrow on propleuron present (Figs 56E, 210C, 218C)	**55**
–	Dorsal carina delimiting a dorsal furrow on propleuron absent (Figs 211C, 215C)	**60**
55(54)	Nucha surrounded by long radiating carinae; propodeum without median longitudinal carina or medially rhomboid-shaped with transverse rugae	**56**
–	Nucha surrounded by very short radiating carinae (Fig. 210F); propodeum with a median longitudinal dent, but no trace of median longitudinal carina (Fig. 210F)	***G* . *sureshnaiki* Arias-Penna, sp. nov.**
56(55)	Propodeal spiracle distally framed by faintly concave/wavy carina (Figs 218F, 220F); phragma of the scutellum widely visible (Figs 218F, 220F)	**57**
–	Propodeal spiracle without distal carina (Figs 56C, 99C); phragma of the scutellum partially exposed (Fig. 99C) or completely concealed (Fig. 56C)	**58**
57(56)	Area just behind transscutal articulation with a sloped transverse strip (Fig. 218E); dorsal furrow of pronotum with a defined smooth band only proximally (Fig. 218A); entire surface of hind tibia with numerous strong spines	***G* . *tomwallai* Arias-Penna, sp. nov.**
–	Area just behind transscutal articulation nearly at the same level as mesoscutum (flat, Fig. 220E); dorsal furrow of pronotum with a well-defined smooth band throughout (Fig. 220C); surface of hind tibia with strong spines only on distal half	***G* . *wilmersimbanai* Arias-Penna, sp. nov.**
58(56)	Medioanterior pit of metanotum bisected by a median longitudinal carina; propodeum without a median longitudinal carina, without medially rhomboid-shaped; scutellum in profile flat and on same plane as mesoscutum	**59**
–	Medioanterior pit of metanotum without median longitudinal carina (Figs 105C, 106E); propodeum medially rhomboid-shaped with transverse rugae (Figs 105C, 106E); scutellum in profile convex and slightly higher than mesoscutum (Figs 105E, 106G)	***G* . *iangauldi* Arias-Penna, sp. nov.**
59(58)	Proximal half of propodeum weakly curved (Figs 56B, 57B); propleuron with fine punctations throughout throughout (Figs 56E, 57E); distal antennal flagellomere longer than penultimate; mesoscutum punctation distinct proximally ranging to satiny distally (Figs 56B, 57B)	***G* . *christerhanssoni* Arias-Penna, sp. nov.**
–	Proximal half of propodeum curved (Figs 99B, 100B); propleuron with fine rugae (Figs 99E, 100E); distal antennal flagellomere subequal in length with penultimate; mesoscutum punctation distinct throughout (Figs 99B, 100B)	***G* . *henrytownesi* Arias-Penna, sp. nov.**
60(54)	Precoxal groove with faintly lineate sculpture (Figs 54E, 191J)	**61**
–	Precoxal groove smooth and shiny (Figs 213J, 215J)	**62**
61(60)	In lateral view, metasoma laterally compressed (Figs 54A, G, 55A); fore wing with 1 cu-a vein straight, complete, touching the edge of 1-1A vein (Figs 54I, 55H); inner margin of eyes straight throughout; scutellum in profile flat and on same plane as mesoscutum (Figs 54E, 55E)	***G* . *chrisgrinteri* Arias-Penna, sp. nov.**
–	In lateral view, metasoma curved (Figs 191K, 192A); fore wing with 1 cu-a vein curved, incomplete/broken, not reaching the edge of 1-1A vein (Fig. 191L); inner margin of eyes diverging slightly at antennal sockets (Figs 191C, 192B); scutellum in profile convex and slightly higher than mesoscutum (Figs 191J, 192I)	***G* . *robbinthorpi* Arias-Penna, sp. nov.**
62(60)	Petiole on T1 distally with lateral margins curved (convex, Figs 211H, 213H); mesoscutum punctation proximally distinct, but distally absent/dispersed; dorsal furrow of pronotum with a well-defined smooth band	**63**
–	Petiole on T1 distally with lateral margins relatively straight (Fig. 215H, I); mesoscutum punctation distinct throughout (Fig. 215F); dorsal furrow of pronotum without a smooth band (Fig. 215C, J)	***G* . *thibautdelsinnei* Arias-Penna, sp. nov.**
63(62)	Face punctate-lacunose (Fig. 211B); distal antennal flagellomere longer than penultimate; scutellum in profile slightly convex, but on same plane as mesoscutum (Fig. 211J)	***G.suzannegreenae* Arias-Penna, sp. nov.**
–	Face with dense fine punctations (Fig. 213B); distal antennal flagellomere subequal in length with penultimate; scutellum in profile convex and slightly higher than mesoscutum (Fig. 213A)	***G* . *taniaariasae* Arias-Penna, sp. nov.**
64(49)	Propodeum medially rhomboid-shaped with transverse rugae, but no trace of median longitudinal carina	**65**
–	Propodeum without medially rhomboid-shaped or without median longitudinal carina	**66**
65(64)	Hind coxa with medium-size punctate throughout (Fig. 151A, J); hind telotarsus as equal in length as fourth tarsomere; distal antennal flagellomere subequal in length with penultimate; phragma of the scutellum widely visible (Fig. 151F)	***G* . *marcpolleti* Arias-Penna, sp. nov.**
–	Hind coxa punctate only ventrally (Fig. 208A, J); hind telotarsus longer than fourth tarsomere; distal antennal flagellomere longer than penultimate; phragma of the scutellum completely concealed (Fig. 208F)	***G* . *sujeevanratnasinghami* Arias-Penna, sp. nov.**
66(64)	Lateral grooves delimiting the median area on T2 distally losing definition (Figs 15I, 128D, 156G)	**67**
–	Lateral grooves delimiting the median area on T2 clearly defined and reaching the distal edge of T2 (Figs 157G, 170H, 223G)	**69**
67(66)	Shape of proximal half of propodeum weakly curved in dorsal view; longitudinal median carina on face present	**68**
–	Shape of proximal half of propodeum more strongly curved in dorsal view (Fig. 156F); longitudinal median carina on face absent (Fig. 156B)	***G* . *marshawheelerae* Arias-Penna, sp. nov.**
68(67)	Dorsal outer depression on hind coxa absent (Figs 14D, 15E); fore telotarsus longer than fourth tarsomere; antenna shorter than body; distal antennal flagellomere shorter than penultimate; vertex in dorsal view narrow (Figs 14C, 15C); scutellar punctation distinct throughout (Figs 14F, 15G)	***G* . *andybennetti* Arias-Penna, sp. nov.**
–	Dorsal outer depression on hind coxa present (Figs 127A, 128A); fore telotarsus as equal as fourth tarsomere; antenna slightly longer than body; distal antennal flagellomere longer than penultimate; vertex in dorsal view wide (Figs 127B, 128B); scutellar punctation indistinct throughout (Figs 127B, 128B)	***G* . *johnheratyi* Arias-Penna, sp. nov.**
69(66)	Anterior furrow of metanotum with a small lobe (without setae, Figs 122F, 223F); axillary trough of scutellum almost smooth (Figs 122F, 223F)	**70**
–	Anterior furrow of metanotum without setiferous lobes (Figs 115G, 177F); axillary trough of scutellum with sculpture (Figs 115G, 177F)	**71**
70(69)	Petiole finely sculptured only distally (Fig. 122G, H); vertex in lateral view rounded (Fig. 122C); scutellar punctation indistinct throughout (Fig. 122E); phragma of the scutellum widely visible (Fig. 122F); median area on T2 as broad as long (Fig. 122G)	***G* . *jimmilleri* Arias-Penna, sp. nov.**
–	Petiole completely smooth and polished, with faint, satin-like sheen (Fig. 223G, H); vertex in lateral view pointed or nearly so (Fig. 223C); scutellar punctation scattered throughout (Fig. 223F); phragma of the scutellum partially exposed (Fig. 223F); median area on T2 broader than long (Fig. 223G, H)	***G* . *yanayacuensis* Arias-Penna, sp. nov.**
71(69)	Dorsal carina delimiting a dorsal furrow on propleuron present (Figs 157C, 177C)	**72**
–	Dorsal carina delimiting a dorsal furrow on propleuron absent (Fig. 115C)	**73**
72(71)	Medioanterior pit of metanotum circular or oval with a short proximal carina (Fig. 157E, F); vertex in dorsal view wide (Fig. 157D); scutellar punctation indistinct throughout (Fig. 157E, F); dorsal furrow of pronotum without a smooth band (Fig. 157A, C, I)	***G* . *mayberenbaumae* Arias-Penna, sp. nov.**
–	Medioanterior pit of metanotum semicircular without median longitudinal carina (Fig. 177E, F); vertex in dorsal view narrow (Fig. 177D); scutellar punctation scattered throughout (Fig. 177E, F); dorsal furrow of pronotum with a well-defined smooth band (Fig. 177A, C, I)	***G* . *pachopinasi* Arias-Penna, sp. nov.**
73(71)	Scutellum in profile flat; fore wing with vein 2-1A tubular throughout; median area on T2 distally with lateral margins relatively straight	**74**
–	Scutellum in profile slightly convex (Fig. 170A, J); fore wing with vein 2-1A proximally tubular, distally spectral (Fig. 170L); median area on T2 distally with lateral margins curved (concave, Fig. 170H, I)	***G.montywoodi* Arias-Penna, sp. nov.**
74(73)	Distal 1/4 of mesoscutum with a central dent (Fig. 115F); medioposterior band of scutellum only very partially overlapping the medioanterior pit of metanotum (Fig. 115F, G); median area on T2 slightly longer than broad (Fig. 115E, H)	***G* . *jaquioconnorae* Arias-Penna, sp. nov.**
–	Distal 1/3 of mesoscutum with lateral margin slightly dented (Figs 133G, 134F); medioposterior band of scutellum mostly overlapping the medioanterior pit of metanotum (Figs 133G, H, 134F, G); median area on T2 broader than long (Figs 133I, 134H)	***G* . *johnstiremani* Arias-Penna, sp. nov.**
75(1)	Lateral grooves delimiting the median area on T2 clearly defined and reaching the distal edge of T2 (Figs 32I, 116D)	**76**
–	Lateral grooves delimiting the median area on T2 distally losing definition (Figs 48D, 109D)	**122**
76(75)	Propodeum with a clearly visible median longitudinal carina (Fig. 201F)	**77**
–	Propodeum without median longitudinal carina or with a median longitudinal dent, or medially rhomboid-shaped with transverse rugae, but no trace of median longitudinal carina	**78**
77(76)	Precoxal groove smooth and shiny (Fig. 31A, G, H); medioanterior pit of metanotum circular without median longitudinal carina (Figs 31G, 32G); inner margin of eyes straight throughout; scutellar punctation indistinct throughout (Figs 31G, 32G); fore wing with 1 cu-a vein complete, touching the edge of 1-1A vein (Figs 31L, 32L)	***G* . *bobhanneri* Arias-Penna, sp. nov.**
–	Precoxal groove with transverse lineate sculpture (Figs 201A, I, 202E); medioanterior pit of metanotum circular and bisected by a median longitudinal carina (Figs 201F, 202C); inner margin of eyes diverging slightly at antennal sockets (Fig. 201B); scutellar punctation distinct throughout (Figs 201E, 202B); fore wing with 1 cu-a vein complete, but junction with 1-1A vein spectral (Fig. 202D)	***G* . *shelbystedenfeldae* Arias-Penna, sp. nov.**
78(76)	Petiole on T1 completely smooth and polished, with faint, satin-like sheen (Figs 116D, 123D, 166F)	**79**
–	Petiole on T1 finely sculptured or with rugae (Figs 98G, 171E) or with a mix of sculpture (Figs 186G, 209G)	**90**
79(78)	Petiole on T1 evenly narrowing distally (wide base to a narrow apex, Figs 116D, 123D)	**80**
–	Petiole on T1 parallel-sided in proximal half (gradually or not), then narrowing or petiole parallel-sided, but narrowing over distal 1/3 or at apex (Figs 9H, 125D, 131F)	**84**
80(79)	Fore wing with r vein slightly curved or curved; distal antennal flagellomere longer than penultimate	**81**
–	Fore wing with r vein straight (Figs 116I, 117I); distal antennal flagellomere subequal in length with penultimate	***G* . *jeremydewaardi* Arias-Penna, sp. nov.**
81(80)	Surface of metasternum flat or nearly so (as in Figs 37H, 38H)	**82**
–	Surface of metasternum convex (as in Fig. 5F)	***G* . *mikeschauffi* Arias-Penna, sp. nov.**
82(81)	Antenna longer than body; longitudinal median carina on face absent	***G* . *jesusugaldei* Arias-Penna, sp. nov.**
–	Antenna shorter than body; longitudinal median carina on face present	**83**
83(82)	Vertex laterally pointed or nearly so; contour of mesopleuron straight/angulate or nearly so; area just behind transscutal articulation with a sloped transverse strip	***G* . *victoriapookae* Arias-Penna, sp. nov.**
–	Vertex laterally rounded; contour of mesopleuron convex; area just behind transscutal articulation depressed centrally	***G* . *jjrodriguezae* Arias-Penna, sp. nov.**
84(79)	Inner margin of eyes straight throughout	**85**
–	Inner margin of eyes diverging slightly at antennal sockets (Fig. 9B)	**86**
85(84)	Precoxal groove shallow, but visible, smooth and shiny (Figs 92A, E, 93A, E); distal antennal flagellomere longer than penultimate; median area and adjacent area on T2 dark, but lateral ends pale	***G* . *gerarddelvarei* Arias-Penna, sp. nov.**
–	Precoxal groove deep with transverse lineate sculpture (Figs 131D, 132G); distal antennal flagellomere subequal in length with penultimate; median area and lateral ends on T2 dark (Figs 131F, 132F)	***G.johnnoyesi* Arias-Penna, sp. nov.**
86(83)	Petiole virtually parallel-sided, but narrowing over distal 1/3 or at apex (Figs 9H, I, 125D, H)	**87**
–	Petiole parallel-sided in proximal half, then narrowing (gradually or not, Figs 62F, 152G)	**88**
87(86)	Nucha surrounded by very short radiating carinae (Fig. 9G); proximal half of propodeum weakly curved (Fig. 9G); antenna longer than body; mesoscutum punctation proximally distinct, but distally absent/dispersed (Fig. 9F); axillary trough of metanotum with small punctation throughout (Fig. 9G)	***G* . *alexwildi* Arias-Penna, sp. nov.**
–	Nucha without distinct short radiating carinae (Figs 125C, 126C); proximal half of propodeum straight or nearly so (Fig. 125B, C); antenna as same length as body length; mesoscutum distinctly punctate throughout (Figs 125B, 126B); axillary trough of metanotum proximally with semircular/undulate carina, distally smooth (Figs 125C, 126C)	***G* . *johnburnsi* Arias-Penna, sp. nov.**
88(86)	Median area between lateral ocelli without depression; distal antennal flagellomere longer than penultimate	**89**
–	Median area between lateral ocelli slightly depressed (Figs 62B, 63A); distal antennal flagellomere subequal in length with penultimate	***G* . *daveroubiki* Arias-Penna, sp. nov.**
89(88)	Vertex in lateral view rounded; anterior furrow of metanotum without setiferous lobes and not as well delineated as posterior furrow of metanotum (Figs 66B, C, 67C); mesoscutum punctation distinct throughout (Figs 66B, 67B); fore wing with vein 2-1A proximally tubular, distally spectral although sometimes difficult to see (Figs 66I, 67I)	***G* . *davesmithi* Arias-Penna, sp. nov.**
–	Vertex in lateral view pointed or nearly so (Fig. 153A); anterior furrow of metanotum with a small lobe (without setae) and not as well delineated as posterior furrow of metanotum (Figs 152C, 153C); mesoscutum proximally distinctly punctate, distally with a polished area (Figs 152B, 153B); fore wing with vein 2-1A absent (Figs 152I, 153I)	***G.marjorietownesae* Arias-Penna, sp. nov.**
90(78)	Inner margin of eyes straight throughout (Fig. 136B)	**91**
–	Inner margin of eyes diverging slightly at antennal sockets (Figs 168B, 186B)	**92**
91(90)	Edges of median area on T2 polished and followed by a deep groove (Figs 129G, 130E); scutellar punctation only on distal half (Fig. 129D, F); in lateral view, metasoma laterally compressed (Figs 129H, 130G)	***G* . *johnlasallei* Arias-Penna, sp. nov.**
–	Edges of median area on T2 obscured by weak longitudinal stripes (Fig. 136H, I); scutellar punctation scattered throughout (Fig. 136F, G); in lateral view, metasoma curved (Fig. 136A, K)	***G* . *juanvargasi* Arias-Penna, sp. nov.**
92(90)	Fore wing with r vein straight (Figs 6I, 98K)	**93**
–	Fore wing with r vein slightly curved or curved (Figs 18K, 27L)	**94**
93(90)	Mesoscutum proximally convex distally flat with punctation distinct proximally ranging to satiny distally (Figs 5D, 6D); scutellar punctation peripherally distinct, absent centrally (Figs 5D, 6D); antenna shorter than body; phragma of the scutellum widely visible (Figs 5B, D, 6D); T3 longer than T2 (Fig. 5E)	***G* . *alejandrovalerioi* Arias-Penna, sp. nov.**
–	Distal 1/3 of mesoscutum with lateral margin slightly dented, punctation distinct throughout (Fig. 98E); scutellar punctation scattered throughout (Fig. 98E, F); antenna longer than body; phragma of the scutellum completely concealed (Fig. 98E, F); T3 as long as T2 (Fig. 98H)	***G* . *henryhespenheidei* Arias-Penna, sp. nov.**
94(92)	Dorsal outer depression on hind coxa absent (Figs 18J, 27K)	**95**
–	Dorsal outer depression on hind coxa present (Figs 186J, 209J)	**96**
95(94)	Propleuron with fine rugae (Figs 18I, 19E); anteroventral contour of mesopleuron straight/angulate or nearly so (Figs 18A, 19A); mesoscutum punctation proximally distinct, but distally absent/dispersed (Figs 18E, 19B); T3 longer than T2 (Figs 18H, 19D)	***G* . *andysuarezi* Arias-Penna, sp. nov.**
–	Propleuron with fine punctations throughout (Figs 27J, 28F); anteroventral contour of mesopleuron convex (Figs 27A, 28A); mesoscutum proximally with distinct punctation distally with a polished area (Figs 27F, 28C); T3 as long as T2 (Fig. 27I)	***G* . *betogarciai* Arias-Penna, sp. nov.**
96(94)	Fore wing with vein 1 cu-a straight (Figs 172J, 175K, 222K)	**97**
–	Fore wing with vein 1 cu-a curved (Figs 72I, 193L, 207B)	**103**
97(96)	Petiole on T1 with a mix of fine rugae and coarse sculpture (Figs 186G, 209G); propodeum medially rhomboid-shaped with transverse rugae, but no trace of median longitudinal carina (Figs 186F, 209F)	**98**
–	Petiole on T1 finely sculptured (Figs 171E, 222G); propodeum without median longitudinal carina or with a median longitudinal dent (Figs 171G, 222F)	**99**
98(97)	Petiole on T1 with lateral margin straight throughout (Fig. 186G, H); fore telotarsus proximally narrow, distally wide; dorsal furrow of pronotum without a smooth band (Fig. 186A, I)	***G* . *petermarzi* Arias-Penna, sp. nov.**
–	Petiole on T1 with lateral margin relatively straight in proximal half, but distal half curved (convex, Fig. 209G, H); fore telotarsus almost same width throughout; dorsal furrow of pronotum with a well-defined smooth band (Fig. 209A, I)	***G* . *suniae* Arias-Penna, sp. nov.**
99(97)	Petiole on T1 virtually parallel-sided for most of length but narrowing for distal 1/3 or apex (Figs 190H, 222G)	**100**
–	Petiole on T1 parallel-sided in proximal half, then narrowing (Figs 171E, H, 172F)	***G* . *nataliaivanovae* Arias-Penna, sp. nov.**
100(99)	Temple punctate-lacunose; propodeum with a median longitudinal dent, but no trace of median longitudinal carina	**101**
–	Temple punctate; propodeum without median longitudinal carina	**102**
101(100)	Fore wing with vein 2 cu-a present as spectral vein, sometimes difficult to see (Figs 175K, 176H); dorsal groove on axillary trough of scutellum with parallel carinae (Figs 175F, 176C); propodeum with a median longitudinal dent (Figs 175F, 176C); mesoscutum proximally distinctly punctate, distally with a polished area (Figs 175E, 176B)	***G* . *ninazitaniae* Arias-Penna, sp. nov.**
–	Fore wing with vein 2 cu-a absent (Fig. 222K); dorsal groove on axillary trough of scutellum smooth (Fig. 222E, F); propodeum with a shallow median longitudinal dent with rugae (Fig. 222F); mesoscutum punctation proximally distinct, but distally absent/dispersed (Fig. 222E)	***G* . *yalizhangae* Arias-Penna, sp. nov.**
102(100)	Vertex in lateral view rounded (Fig. 46A); dorsal groove on axillary trough of scutellum with semicircular/parallel carinae (Fig. 46B, C); distal antennal flagellomere subequal in length with penultimate; mesoscutum distinctly punctate throughout (Fig. 46B)	***G* . *carlrettenmeyeri* Arias-Penna, sp. nov.**
–	Vertex in lateral view pointed or nearly so (Fig. 190C); dorsal groove on axillary trough of scutellum with carinae only proximally (Fig. 190F, G); distal antennal flagellomere longer than penultimate; mesoscutum punctation proximally distinct, but distally absent/dispersed (Fig. 190F)	***G* . *rafamanitioi* Arias-Penna, sp. nov.**
103(96)	Precoxal groove shallow, but visible (Figs 47I, 187I, 206I)	**104**
–	Precoxal groove deep (Figs 168I, 221I)	**106**
104(103)	Petiole on T1 with rugae (Figs 187G, 206G)	**105**
–	Petiole on T1with a mix of fine rugae and coarse sculpture for most of the surface (Fig. 47H)	***G.celsoazevedoi* Arias-Penna, sp. nov.**
105(104)	Petiole on T1 with lateral margin in proximal half straight and distal half curved (convex, Fig. 187G, H); propodeum with a median longitudinal dent, but no trace of median longitudinal carina (Fig. 187F); hind coxa medially smooth, dorsally sparsely punctate, ventrally densely punctate (Fig. 187A, J); mesoscutum punctation proximally distinct, but distally absent/dispersed (Fig. 187E)	***G* . *phildevriesi* Arias-Penna, sp. nov.**
–	Petiole on T1 with lateral margins straight throughout (Fig. 206G, H); propodeum medially rhomboid-shaped with transverse rugae, but no trace of median longitudinal carina (Fig. 206F); hind coxa punctate only ventrally (Figs 206A, J, 207A); mesoscutum proximally distinctly punctate, distally with a polished area (Fig. 206E)	***G* . *stephaniekirkae* Arias-Penna, sp. nov.**
106(103)	Precoxal groove smooth and shiny (Figs 72E, 99I, 219I)	**107**
–	Precoxal groove with lineate sculpture (Figs 163F, 178I, 188I)	**108**
107(106)	Petiole on T1 virtually parallel-sided for most of length, but narrowing along distal 1/3 (Fig. 72D, G); medioposterior band of scutellum only very partially overlapping the medioanterior pit of metanotum (Fig. 72B, C); pronotum virtually without trace of dorsal furrow (Fig. 72A, E)	***G* . *donquickei* Arias-Penna, sp. nov.**
–	Petiole on T1 evenly narrowing distally (Figs 199G, H, 200E, G); medioposterior band of scutellum mostly overlapping the medioanterior pit of metanotum (Figs 199F, 220F); pronotum with a distinct dorsal furrow (Figs 199I, 200H)	***G* . *scottshawi* Arias-Penna, sp. nov.**
108(106)	Surface of metasternum convex (as in Fig. 5F)	**109**
–	Surface of metasternum flat or nearly so (as in Figs 17G, 25G)	**113**
109(108)	Dorsal carina delimiting a dorsal furrow on propleuron present (Figs 188A, 203C)	**110**
–	Dorsal carina delimiting a dorsal furrow on propleuron absent (Figs 193J, 194I)	***G* . *ronaldzunigai* Arias-Penna, sp. nov.**
110(109)	Antenna longer than body; anterior furrow of metanotum with a small lobe (without setae, Figs 174G, 203F); distal antennal flagellomere longer than penultimate	**111**
–	Antenna as same length as body; anterior furrow of metanotum without setiferous lobes (Fig. 188F); distal antennal flagellomere subequal in length with penultimate	***G* . *philwardi* Arias-Penna, sp. nov.**
111(110)	Propleuron with fine rugae (Figs 178C, 179G)	**112**
–	Propleuron with a mix of rugae and fine punctation (Fig. 203A, C, I)	***G* . *sondrawardae* Arias-Penna, sp. nov.**
112(111)	Ventral margin of fore telotarsus slightly excavated and with a tiny curved seta (as in Fig. 37E); medioanterior pit of metanotum semicircular and bisected by a median longitudinal carina (Figs 173F, 174G)	***G* . *nealweberi* Arias-Penna, sp. nov.**
–	Ventral margin of fore telotarsus entire without seta; medioanterior pit of metanotum circular without median longitudinal carina (Figs 178F, 179B)	***G* . *pamitchellae* Arias-Penna, sp. nov.**
113(108)	Petiole on T1 distally with lateral margins curved (convex, Figs 163E, 195H, 221G)	**114**
–	Petiole on T1 distally with lateral margins relatively straight (Figs 53G, 64D, 160D)	**116**
114(113)	Fore telotarsus almost same width throughout; medioposterior band of scutellum only very partially overlapping the medioanterior pit of metanotum (Figs 195G, 221E)	**115**
–	Fore telotarsus proximally narrow, distally wide; medioposterior band of scutellum mostly overlapping the medioanterior pit of metanotum (Figs 163D, 164C)	***G* . *mikegatesi* Arias-Penna, sp. nov.**
115(114)	Ventral margin of fore telotarsus excavated with conspicuous curved seta over this excavation; mesoscutum distinctly punctate throughout (Figs 195F, 196E); fore wing with vein 2-1A present only proximally as tubular vein (Figs 195L, 196K)	***G* . *roysnellingi* Arias-Penna, sp. nov.**
–	Ventral margin of fore telotarsus apex excavated, but without seta; mesoscutum punctation distinct proximally ranging to satiny distally (Fig. 221E); fore wing with vein 2-1A absent (Fig. 221K)	***G* . *wonyoungchoi* Arias-Penna, sp. nov.**
116(113)	Propodeal spiracle without distal carina (Figs 52C, 64C, 101C)	**117**
–	Propodeal spiracle distally framed by a short concave carina (Figs 109C, 111B, 160C)	**119**
117(116)	Ventral margin of fore telotarsus slightly excavated; scutellar punctation indistinct throughout (Figs 65B, 101B)	**118**
–	Ventral margin of fore telotarsus entire; scutellar punctation distinct throughout (Figs 52B, 53B)	***G* . *chrisdarlingi* Arias-Penna, sp. nov.**
118(117)	Mesoscutum punctation proximally distinct, but distally absent/dispersed (Figs 64B, 65B); phragma of the scutellum completely concealed (Figs 64C, 65C); antenna longer than body	***G* . *daveschindeli* Arias-Penna, sp. nov.**
–	Mesoscutum punctate throughout (Figs 101B, 102B); phragma of the scutellum partially exposed (Figs 101C, 102C); antenna shorter than body	***G* . *howelldalyi* Arias-Penna, sp. nov.**
119(116)	Inner spur of hind tibia much longer than outer spur; median area on T2 broader than long (Figs 111C, 113D, 168H)	**120**
–	Inner spur of hind tibia slightly longer than outer spur; median area on T2 as broad as long (Fig. 160D, G)	***G* . *mehrdadhajibabaei* Arias-Penna, sp. nov.**
120(119)	Ventral margin of fore telotarsus slightly excavated and with a tiny curved seta; distal antennal flagellomere longer than penultimate	**121**
–	Ventral margin of fore telotarsus entire without seta; distal antennal flagellomere subequal in length with penultimate	***G* . *mikesharkeyi* Arias-Penna, sp. nov.**
121(120)	Face convex (Fig. 112C); area just behind transscutal articulation nearly at the same level as mesoscutum (Fig. 112F)	***G* . *jacklonginoi* Arias-Penna, sp. nov.**
–	Face flat or nearly so (Fig. 113A); area just behind transscutal articulation with a sloped transverse strip (Figs 113B, 114B)	***G* . *jamesrobertsoni* Arias-Penna, sp. nov.**
122(75)	Fore wing with 2RS vein straight (Figs 48G, 86I, 107I)	**123**
–	Fore wing with 2RS slightly convex to convex (Figs 104I, 146J)	**132**
123(122)	Nucha surrounded by very short radiating carinae (Figs 107C, 180G); propodeum without median longitudinal carina; antenna longer than body	**124**
–	Nucha surrounded by long radiating carinae (Figs 109C, 110D); propodeum medially rhomboid-shaped with transverse rugae, but no trace of median longitudinal carina (Figs 109B, 110B); antenna shorter than body	***G* . *ilarisaaksjarvi* Arias-Penna, sp. nov.**
124(123)	Propodeal spiracle without distal carina (Figs 86C, 156F)	**125**
–	Propodeal spiracle distally framed by a short concave carina (Figs 48B, C, 49C)	***G* . *charlesmicheneri* Arias-Penna, sp. nov.**
125(124)	Petiole on T1 distally with lateral margins curved (convex, Figs 87D, 107D)	**126**
–	Petiole on T1 distally with lateral margins relatively straight (Figs 154D, 214G)	**127**
126(125)	Phragma of the scutellum partially exposed (Fig. 86B, C); longitudinal median carina on face absent; inner margin of eyes straight throughout; scutellar punctation scattered throughout (Figs 86B, 87C)	***G* . *garygibsoni* Arias-Penna, sp. nov.**
–	Phragma of the scutellum widely visible (Figs 107C, 108C); longitudinal median carina on face present; inner margin of eyes diverging slightly at antennal sockets; scutellar punctation indistinct throughout (Figs 107B, 108B)	***G* . *ianyarrowi* Arias-Penna, sp. nov.**
127(125)	Anteroventral contour of mesopleuron convex	**128**
–	Anteroventral contour of mesopleuron straight/angulate or nearly so	**130**
128(127)	Medioanterior pit of metanotum semicircular without median longitudinal carina (Figs 154C, 214F)	**129**
–	Medioanterior pit of metanotum circular and bisected by a median longitudinal carina (Figs 180G, 181C)	***G* . *paulhansoni* Arias-Penna, sp. nov.**
129(128)	Medioposterior band of scutellum mostly overlapping the medioanterior pit of metanotum (Figs 154C, 155C); fore wing with vein 2-1A present only proximally as tubular vein (Figs 154I, 155I)	***G* . *markshawi* Arias-Penna, sp. nov.**
–	Medioposterior band of scutellum only very partially overlapping the medioanterior pit of metanotum (Fig. 214E, F); fore wing with vein 2-1A absent (Fig. 214K)	***G* . *tanyadapkeyae* Arias-Penna, sp. nov.**
130(127)	Propleuron with fine rugae (Figs 60E, 88E); mesoscutum punctate throughout (Fig. 60B)	**131**
–	Propleuron with fine punctations throughout (Figs 68E, 69E); mesoscutum punctation proximally distinct, but distally absent/dispersed (Figs 68B, 69B)	***G* . *davidwahli* Arias-Penna, sp. nov.**
131(130)	Scutellar punctation distinctly throughout (Figs 60B, 61B); distal antennal flagellomere subequal in length with penultimate; inner margin of eyes diverging slightly at antennal sockets; phragma of the scutellum partially exposed (Figs 60C, 61C); fore wing with vein 2-1A proximally tubular, distally spectral (Figs 60J, 61I)	***G* . *corriemoreauae* Arias-Penna, sp. nov.**
–	Scutellar punctation indistinct throughout (Fig. 89B, C); distal antennal flagellomere longer than penultimate; inner margin of eyes straight throughout; phragma of the scutellum completely concealed (Figs 88C, 89C); fore wing with vein 2-1A absent (Figs 88I, 89I)	***G* . *gavinbroadi* Arias-Penna, sp. nov.**
132(122)	Propodeal spiracle distally framed by a short concave carina (Figs 50C, 51C); scutellum in profile convex and slightly higher than mesoscutum (Figs 50E, 51E)	**133**
–	Propodeal spiracle without distal carina (Figs 78C, 79C); scutellum in profile otherwise	**134**
133(132)	Propleuron with fine rugae (Figs 50E, 51E); dorsal carina delimiting a dorsal furrow on propleuron absent (Figs 50E, 51E); distal antennal flagellomere subequal in length with penultimate; mesoscutum punctation distinct proximally ranging to satiny distally (Figs 50B, 51B); median area on T2 as broad as long (Figs 50D, 51D)	***G* . *charlesporteri* Arias-Penna, sp. nov.**
–	Propleuron with fine punctation throughout (Figs 197C, 198D); dorsal carina delimiting a dorsal furrow on propleuron present (Figs 197I, 198D); distal antennal flagellomere longer than penultimate; mesoscutum proximally distinctly punctate, distally with a polished area (Figs 197E, 198B); median area on T2 broader than long (Figs 197G, 198F)	***G* . *scottmilleri* Arias-Penna, sp. nov.**
134(132)	Anteroventral contour of mesopleuron straight/angulate or nearly so (Fig. 146A, G); distal antennal flagellomere subequal in length with penultimate	**135**
–	Anteroventral contour of mesopleuron convex (Figs 78A, 79A); distal antennal flagellomere longer than penultimate	***G* . *eowilsoni* Arias-Penna, sp. nov.**
135(134)	Vertex in lateral view rounded; scutellum in profile flat and on same plane as mesoscutum (Fig. 104E); scutellar punctation indistinct throughout (Figs 103B, 104B)	***G* . *hugokonsi* Arias-Penna, sp. nov.**
–	Vertex in lateral view pointed or nearly so (Figs 146C, 147C); scutellum in profile slightly convex, but on same plane as mesoscutum (Figs 146G, 147H); scutellar punctation distinct throughout (Figs 146E, 147E)	***G* . *malloryvanwyngaardenae* Arias-Penna, sp. nov.**

##### Species descriptions

### Glyptapanteles alejandrovalerioi

Taxon classificationAnimaliaHymenopteraBraconidae

Arias-Penna, sp. nov.

http://zoobank.org/DCF330F4-656A-41AA-A7F5-D4FD49B5AC1B

Figs 5 , 6

#### Female.

Body length 2.72 mm, antennal length 2.17 mm, fore wing length 2.55 mm.

#### Type material.

**Holotype**: COSTA RICA • 1♀; 89-SRNP-670A DHJPAR0000057; Área de Conservación Guanacaste, Guanacaste, Sector Santa Rosa, Cafetal; 280 m; 10.85827, -85.61089; 15.vii.1989; gusaneros leg.; cocoons formed on 28.vii.1989 and adhered to the larval cuticle; adult parasitoids emerged on 05.viii.1989; (CNC). **Paratypes.** • 21 (4♀ + 2♂) (9♀ + 6♂); 89-SRNP-670A DHJPAR0000057; same data as for holotype; (CNC).

#### Other material.

**Reared material.** COSTA RICA: *Área de Conservación Guanacaste*, *Guanacaste*, *Sector Santa Rosa*, *Bosque Humedo*: • 26 (0♀, 2♂) (0♀, 24♂); 90-SRNP-1146, DHJPAR0001443; dry forest; 290 m, 10.85145, -85.60801; 29.vi.1990; Daniel H Janzen leg.; caterpillar collected in second instar; cocoons formed on 13.vii.1990 and adhered to the larval cuticle; adult parasitoids emerged on 19.vii.1990. • 23 (0♀, 2♂) (0♀, 21♂); 90-SRNP-1146A, DHJPAR0001500; same data as for preceding except: 29.vi.1990; gusaneros leg.; white cocoons separated but tightly attached and adhered to the larval cuticle; adult parasitoids emerged on 18.vii.1990. • 30 (0♀, 2♂) (0♀, 28♂); 90-SRNP-1146B, DHJPAR0000058; same data as for preceding except: 29.vi.1990; gusaneros leg.; cocoons white and separate and tightly attached to larval cuticle. • 17 (0♀, 2♂) (0♀, 15♂); 04-SRNP-12126.2, DHJPAR0001516; same data as for preceding except: 20.vi.2004, Ruth Franco leg.; cocoons formed on 06.vii.2004 and adhered to the larval cuticle; adult parasitoids emerged on 14.vii.2004. • 13 (0♀, 3♂) (0♀, 10♂); 04-SRNP-12126.3, DHJPAR0001526; same data as for preceding except: 20.vi.2004, Ruth Franco leg.; caterpillar collected in third instar; cocoons massed among the scoli of the larva, formed on 06.vii.2004 and adhered to the larval cuticle; adult parasitoids emerged on 14.vii.2004. • 12 (0♀, 2♂) (0♀, 10♂); 04-SRNP-12126.1, DHJPAR0000286; same data as for preceding except: 20.vi.2004, Ruth Franco leg.; parasitoid cocoons formed on 06.vii.2004 and adhered to the larval cuticle; adult parasitoids emerged on 15.vii.2004.

*Área de Conservación Guanacaste*, *Guanacaste*, *Sector Santa Rosa*, *Cafetal*: • 17 (3♀ + 1♂), (11♀ + 2♂); 93-SRNP-2506, DHJPAR0000072; 280 m; 10.85827, -85.61089; 12.vi.1993; gusaneros leg.; caterpillar collected in second instar; white fluffy cocoons formed on 26.vi.1993 and adhered to the larval cuticle; adult parasitoids emerged on 02.vii.1993. • 11 (3♀ + 2♂) (4♀ + 2♂); 93-SRNP-2507, DHJPAR0000073; same data as for preceding except: white fluffy cocoons adhered to the larval cuticle; date of cocoons not reported; adult parasitoids emerged on 05.vii.1993.

#### Diagnosis.

**Figure 5.**
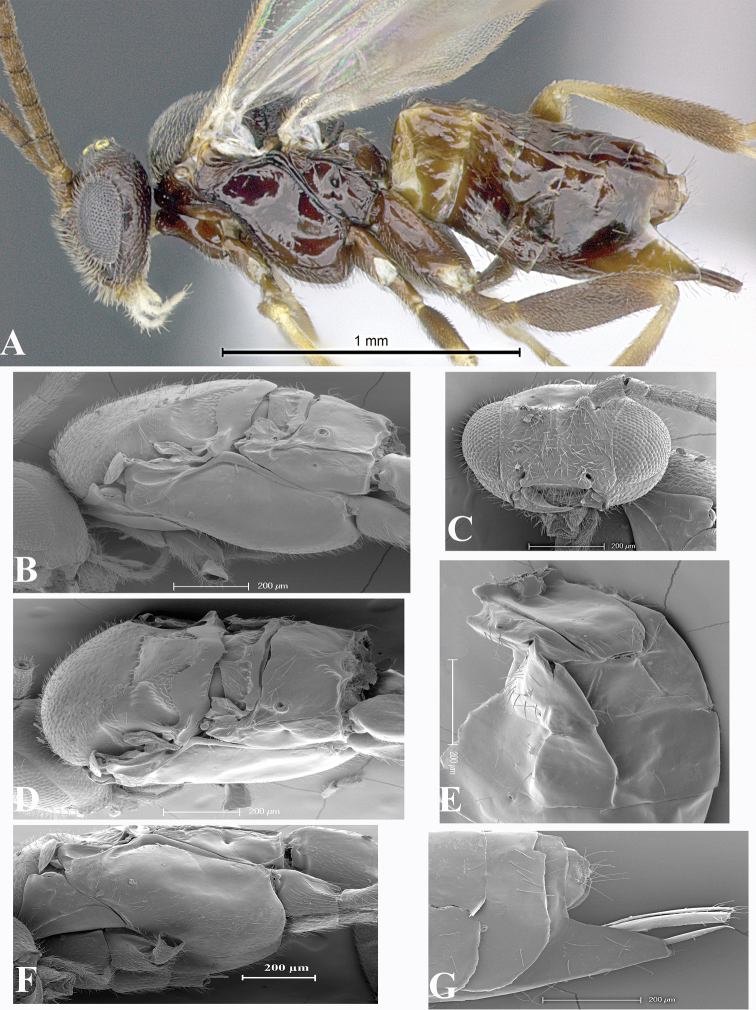
*Glyptapantelesalejandrovalerioi* sp. nov. female 89-SRNP-670A DHJPAR0000057 **A** Habitus **B** Mesosoma, lateral view **C** Head, frontal view **D, F** Mesosoma **D** Dorsolateral view **F** Ventrolateral view **E**T1–3, dorsolateral view **G** Genitalia: hypopygium, ovipositor, ovipositor sheaths, lateral view.

**Figure 6.**
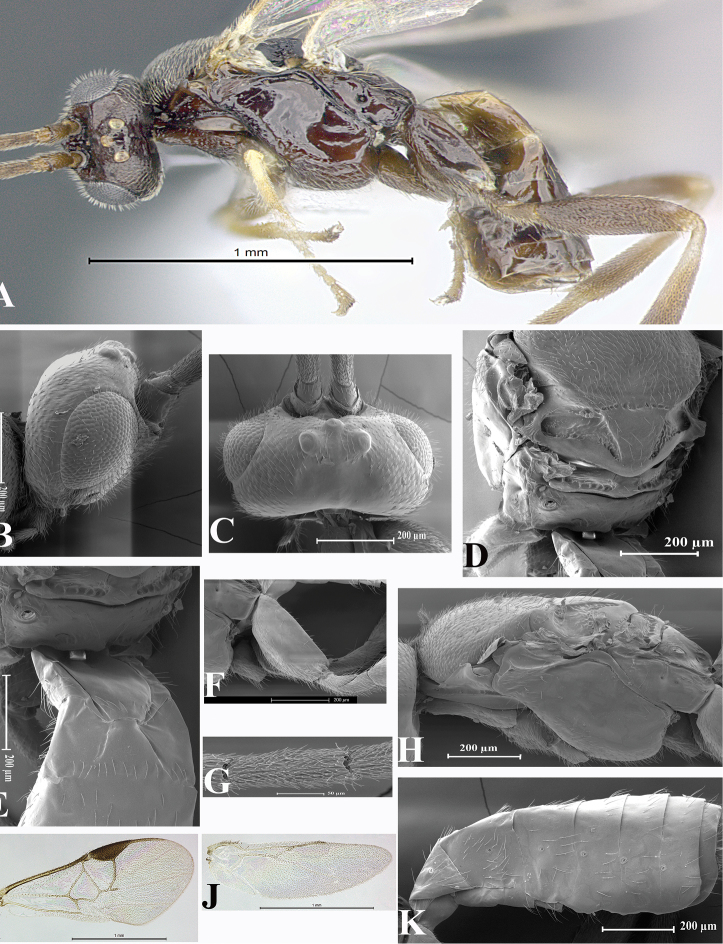
*Glyptapantelesalejandrovalerioi* sp. nov. male 89-SRNP-670A DHJPAR0000057 **A** Habitus **B, C** Head **B** Lateral view **C** Dorsal view **D, H** Mesosoma **D** Dorsolateral view **H** Lateral view **E** Propodeum, T1–3, dorsolateral view **F** Hind coxa, lateral view **G** Flagellomeres **I, J** Wings **I** Fore **J** Hind **K** Metasoma, lateral view.

Mesoscutum proximally convex distally flat with punctation distinct proximally ranging to satiny distally (Figs 5D, 6D), scutellar punctation peripherally distinct, absent centrally (Figs 5D, 6D), antenna shorter than body, phragma of the scutellum widely visible (Figs 5B, D, 6D), T3 longer than T2 (Fig. 5E), fore wing with r vein straight, outer side of junction of r and 2RS veins not forming a stub (Fig. 6I), inner margin of eyes diverging slightly at antennal sockets (Fig. 5C), petiole on T1 finely sculptured only distally (Figs 5E, 6E), propodeum without median longitudinal carina (Figs 5D, 6D), and lateral grooves delimiting the median area on T2 clearly defined and reaching the distal edge of T2 (Figs 5E, 6E).

#### Coloration

(Fig. 5A). General body coloration dark brown, except clypeus, labrum and mandibles yellow-brown; glossa, labial and maxillary palps yellow; all antennal flagellomeres dorsally lighter (light brown) than ventrally (dark brown); propleuron, pronotum, proximal middle area and distal corners of mesoscutum, scutellum, BS, lunules, BM and propodeum lighter than body coloration. Eyes dark gray and ocelli silver. Fore and middle legs brown, except apex of femur, tibiae and the four tarsomeres yellow, telotarsus with yellow-brown tints and claws brown; hind leg brown except proximal 1/3 of tibiae yellow-brown, tibial spurs yellow and tarsi yellow-brown. Petiole on T1 yellow-red/reddish with contours darkened, sublateral areas yellow-brown; T2 with median area and lateral ends brown; T3 and beyond completely brown. In lateral view, T1–2 yellow-brown; T3 and beyond brown. S1–2 yellow-brown, S3 and beyond brown, although hypopygium medially yellow-brown.

#### Description.

**Head** (Fig. 5C). Head rounded with pubescence long and dense. Proximal three antennal flagellomeres longer than wide (0.11:0.06; 0.13:0.06; 0.14:0.06), distal antennal flagellomere longer than penultimate (0.09:0.05, 0.07:0.05), antenna shorter than body (2.17, 2.72); antennal scrobes-frons shallow. Face with punctate-lacunose sculpture, interspaces wavy, laterally with depression and longitudinal median carina present. Fronto-clypeal suture absent or at least indicated by a groove/dark coloration. Frons smooth. Temple wide, punctate-lacunose and interspaces wavy. Inner margin of eyes diverging slightly at antennal sockets; in lateral view, eye anteriorly convex and posteriorly straight. POL shorter than OOL (0.09:0.11). Malar suture present. Median area between lateral ocelli without depression. Vertex laterally pointed or nearly so and dorsally wide.

**Mesosoma** (Fig. 5B, D, F). Mesosoma dorsoventrally convex. Mesoscutum proximally convex and distally flat with punctation distinct proximally ranging to satiny distally and interspaces wavy/lacunose. Scutellum long and slender, distally sloped and fused with BS, scutellar punctation distinct peripherally, absent centrally, in profile scutellum flat and on same plane as mesoscutum, phragma of the scutellum widely visible; BS only very partially overlapping the MPM; ATS demilune proximally with undulate carinae and distally smooth; dorsal ATS groove smooth. Transscutal articulation with small and heterogeneous foveae; area just behind transscutal articulation smooth, shiny and nearly at the same level as mesoscutum (flat). Metanotum with BM wider than PFM (clearly differentiated); MPM circular without median longitudinal carina; AFM without setiferous lobes and not as well delineated as PFM; PFM thick and smooth; ATM with little and incomplete parallel carinae proximally. Propodeum without median longitudinal carina; proximal half relatively polished and weakly curved and distal half relatively polished; distal edge of propodeum with a flange at each side and without stubs; propodeal spiracle without distal carina; nucha surrounded by very short radiating carinae. Pronotum with a distinct dorsal furrow, dorsally with a defined smooth band only proximally; central area of pronotum and dorsal furrow smooth, but ventral furrow with short parallel carinae. Propleuron finely sculptured only ventrally and dorsally without a carina. Metasternum convex. Contour of mesopleuron convex; precoxal groove smooth, shiny, shallow, but visible. Epicnemial ridge convex and teardrop-shaped.

**Legs.** Ventral margin of fore telotarsus entire without seta, proximally narrow and distally wide; fore telotarsus longer than fourth tarsomere (0.10, 0.05). Hind coxa medially smooth, dorsally with scattered punctation, ventrally with dense punctation; dorsal outer depression on hind coxa present. Inner spur of hind tibia slightly longer than outer spur (0.16, 0.12); surface of hind tibia with strong spines only on distal half; hind telotarsus longer than fourth tarsomere (0.10, 0.08).

**Wings.** Fore wing with r vein straight; 2RS vein straight; r and 2RS veins forming an angle at their junction and outer side of junction not forming a stub; shape of 2M vein straight; distally fore wing [where spectral veins are] with microtrichiae more densely concentrated than the rest of the wing; anal cell 1/3 proximally lacking microtrichiae; subbasal cell proximal half smooth; veins 2CUa absent and 2CUb spectral; vein 2 cu-a absent; vein 2-1A present only proximally as spectral vein; tubular vein 1 cu-a straight, incomplete/broken, not reaching the edge of 1-1A vein. Hind wing with vannal lobe narrow, subdistally and subproximally straightened, and setae present only proximally.

**Metasoma** (Fig. 5E, C). Metasoma laterally compressed. Petiole on T1 fine-sculptured only distally; virtually parallel-sided over most of length but narrowing over distal 1/3 (length 0.25; maximum width 0.17; minimum width 0.07) with scattered pubescence concentrated in the first distal third and apex truncate. Lateral grooves delimiting the median area on T2 clearly defined and reaching the dorsal edge (length median area 0.14, length T2 0.14); edges of median area polished, median area broader than long (length 0.14, maximum width 0.16; minimum width 0.07); T2 with a distinctive row of pubescence only at the distal margin. T3 longer than T2 (0.20, 0.14) and with a distinctive row of pubescence only at the distal margin. Pubescence on hypopygium scattered.

**Cocoons.** White oval cocoons with silk fibers messy/disordered/fluffy. Fluffy cocoons separated, but tightly attached and adhered to the larval cuticle.

#### Comments.

The mesopleuron is elongated and rectangle-shaped, the precoxal groove is shallow, the telotarsus on fore leg is twice the length of fourth tarsomere (0.10, 0.05) and with a comb in the claw, the fifth tarsomere proximally is narrow, but it expands distally, the head in dorsal view is rectangular, and the petiole and the median area with the edges clearly distinct.

#### Male

(Fig. 6A–K). The body coloration is darker than females and the antennal flagellomeres are shorter than females.

#### Etymology.

Alejandro A. Valerio is a Costa Rican entomologist; as a graduate student at UIUC, IL, USA he worked with *Parapanteles* and *Hypomicrogaster* (Microgastrinae) from ACG. Currently, he works at the Central American Institute of Biological Research and Conservation (CIBRC), Costa Rica.

#### Distribution.

Parasitized caterpillars were collected in Costa Rica, ACG, Sector Santa Rosa (Cafetal and Bosque Humedo), during June-July 1990, June 1993, and June 2004, at 280–290 m in dry forest and coffee plantations.

#### Biology.

The lifestyle of this parasitoid species is gregarious.

#### Host.

*Periphobaarcaei* (Druce) (Saturniidae, Hemileucinae) feeding on *Hymenaeacourbaril* (Fabaceae) and *Combretumfarinosum* (Combretaceae). Caterpillars were collected at second and third instar.

### Glyptapanteles alexborisenkoi

Taxon classificationAnimaliaHymenopteraBraconidae

Arias-Penna, sp. nov.

http://zoobank.org/83FD8966-FC89-4858-8039-40383F966B1E

Figs 7 , 8

#### Female.

Body length 3.37 mm, antenna length 3.75 mm, fore wing length 3.57 mm.

#### Type material.

**Holotype**: COSTA RICA • 1♀; 02-SRNP-23217, DHJPAR0000025; Área de Conservación Guanacaste, Guanacaste, Sector Cacao, Sendero Toma Agua; cloud forest; 1,140 m; 10.92847, -85.46680; 15.vii.2002; Freddy Quesada leg.; caterpillar collected in third instar; white cocoons somewhat scattered, adhered on leaf surface and formed on 03.viii.2002; adult parasitoid emerged on 17.viii.2002; (CNC). **Paratypes.** • 2 (0♀, 1♂), (0♀, 1♂); 02-SRNP-23217, DHJPAR0000025; same data as for holotype; (CNC).

#### Other material.

**Reared material.** COSTA RICA: *Área de Conservación Guanacaste*, *Guanacaste*, *Sector Cacao*, *SenderoDerrumbe*: • 1 (0♀, 0♂) (1♀, 0♂); 06-SRNP-36373 DHJPAR0012668; cloud forest; 1,220 m; 10.92918, -85.46426; 13.x.2006, Manuel Pereira leg.; caterpillar collected in fifth instar; cocoons formed on 26.x.2006 and adhered to the leaf substrate; adult parasitoid emerged on 04.xi.2006.

#### Diagnosis.

**Figure 7.**
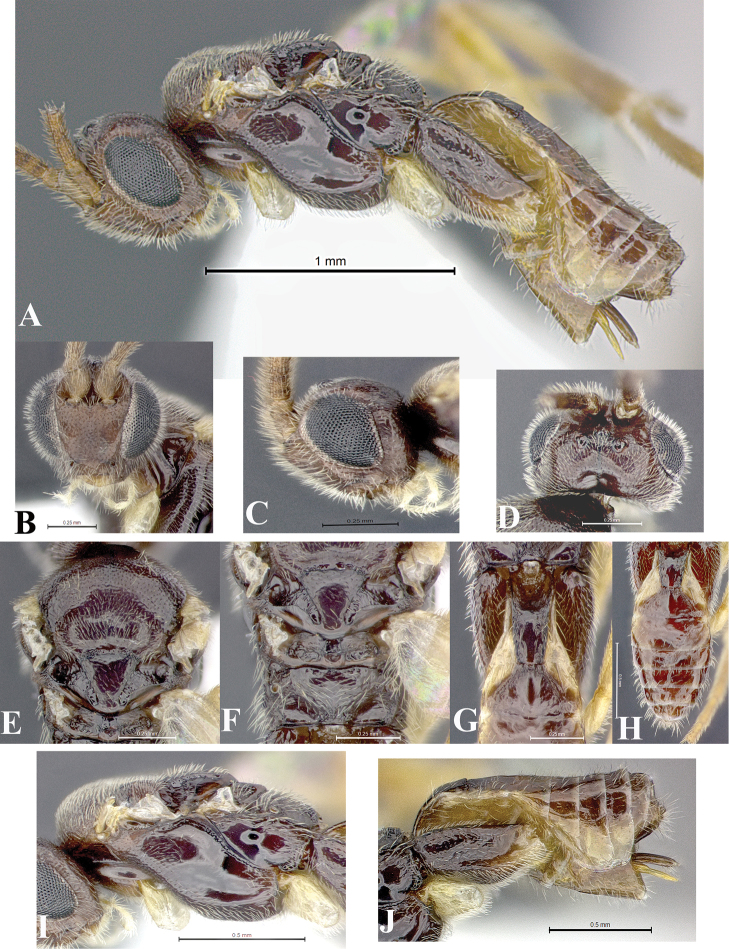
*Glyptapantelesalexborisenkoi* sp. nov. female 02-SRNP-23217 DHJPAR0000025 **A** Habitus **B–D** Head **B** frontal view **C** lateral view **D** dorsal view **E, I** Mesosoma **E** Dorsal view **I** Lateral view **F** Scutellum, metanotum, propodeum, dorsal view **G**T1–2, dorsal view **H, J** Metasoma **H** Dorsal view **J** Lateral view.

**Figure 8.**
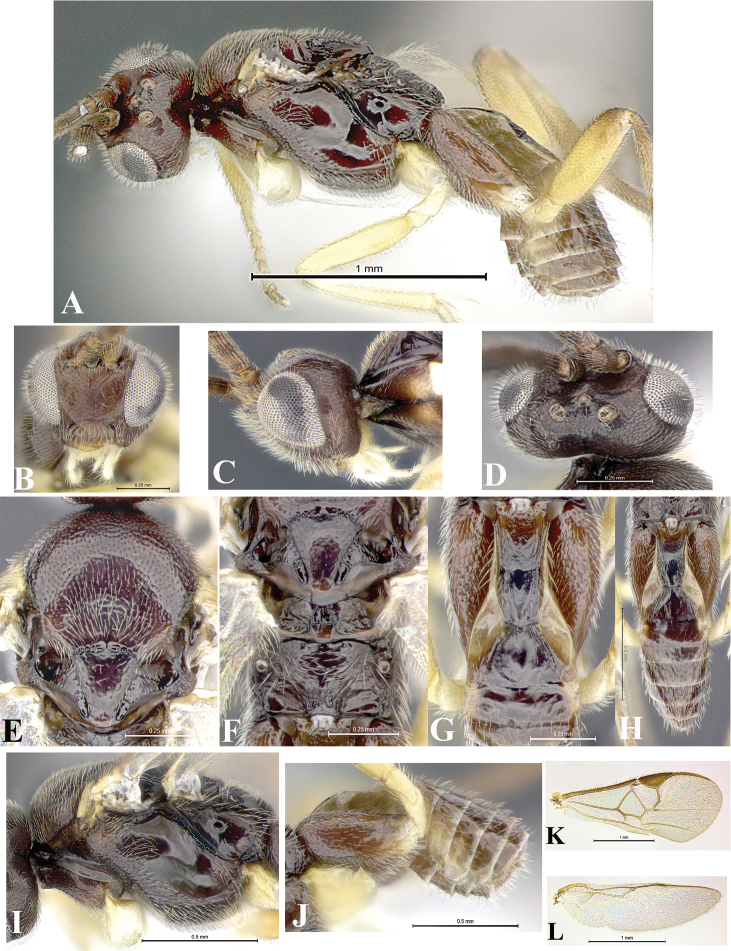
*Glyptapantelesalexborisenkoi* sp. nov. male 02-SRNP-23217 DHJPAR0000025 **A** Habitus **B–D** Head **B** frontal view **C** lateral view **D** dorsal view **E, I** Mesosoma **E** Dorsal view **I** Lateral view **F** Scutellum, metanotum, propodeum, dorsal view, **G**T1–3, dorsal view **H, J** Metasoma **H** Dorsal view **J** Lateral view **K, L** Wings **K** Fore **L** Hind.

Scutellar punctation distinct throughout (Figs 7F, 8F), area just behind transscutal articulation with same kind of sculpture as mesoscutum (Figs 7E, 8E), inner margin of eyes diverging slightly at antennal sockets (Figs 7B, 8B), vertex in dorsal view wide (Figs 7D, 8D), lateral grooves delimiting the median area on T2 clearly defined and reaching the distal edge of T2 (Fig. 7G, H), and fore wing with r vein straight, outer side of junction of r and 2RS veins forming a stub (Fig. 8K).

#### Coloration

(Fig. 7A–J). General body coloration dark reddish brown except glossa, labial and maxillary palps, and tegulae yellow-brown; apex of propleuron, lunules, BS, PMR and BM with yellow tints. Eyes dark gray and ocelli reddish. Fore legs with coxae yellow remaining parts missing; middle legs yellow although tarsomeres with brown tints; hind leg yellow-brown except coxae brown with yellow apex, apex of femora, distal half of tibia and tarsomeres brown. Petiole on T1 brown, although proximally slight lighter, contours darkened, sublateral areas light yellow-brown; T2 with median area brown, lateral ends light yellow-brown; T3 and beyond completely brown, distally each tergum with a narrow translucent band. In lateral view, T1–2 light yellow-brown; T3 and beyond with half dorsal brown, half ventral light yellow-brown. S1–4 light yellow-brown; penultimate sternum and hypopygium brown.

#### Description.

**Head** (Fig. 7B–D). Head rounded with long and dense pubescence. Proximal three antennal flagellomeres longer than wide (0.23:0.06, 0.23:0.06, 0.23:0.05), distal antennal flagellomere longer than penultimate (0.12:0.05, 0.10:0.05), antenna longer than body (3.75, 3.37); antennal scrobes-frons shallow. Face with lateral depression, scattered and finely punctate, interspaces smooth and longitudinal median carina present. Frons punctate. Temple wide, punctations barely noticeable and interspaces smooth. Inner margin of eyes diverging slightly at antennal sockets; in lateral view, eye anteriorly convex and posteriorly straight. POL shorter than OOL (0.10, 0.12). Malar suture present. Median area between lateral ocelli without depression. Vertex laterally rounded and dorsally wide.

**Mesosoma** (Fig. 7E, F, I). Mesosoma dorsoventrally convex. Mesoscutum with punctation distinct throughout, interspaces wavy/lacunose, distal half with a central dent. Scutellum triangular, apex sloped and fused with BS, scutellar punctation distinct throughout, in profile slightly convex, but on same plane as mesoscutum, phragma of the scutellum partially exposed; BS not overlapping the MPM; ATS demilune with a little and incomplete parallel carinae only distally; dorsal ATS groove with carinae only proximally. Transscutal articulation with small and heterogeneous foveae, area just behind transscutal articulation with a sloped transverse strip and with same kind of sculpture as mesoscutum. Metanotum with BM wider than PFM (clearly differentiated); MPM circular and without median longitudinal carina; AFM without setiferous lobes and not as well delineated as PFM; PFM thick and smooth; ATM proximally with semicircular/undulate carina and distally smooth. Propodeum without median longitudinal carina, with fine sculpture throughout and with a shallow dent at each side of nucha; proximal half curved; distal edge with a flange at each side and without stubs; propodeal spiracle distally framed by faintly concave/wavy carina; nucha surrounded by very short radiating carinae. Pronotum with a distinct dorsal furrow, dorsally with a well-defined smooth band; central area of pronotum and both dorsal and ventral furrows smooth. Propleuron with fine punctations throughout and dorsally without a carina. Metasternum flat or nearly so. Contour of mesopleuron convex; precoxal groove smooth, shiny and shallow, but visible; epicnemial ridge convex and teardrop-shaped.

**Legs.** Ventral margin of fore telotarsus entire without seta, shape of fore telotarsus proximally narrow and distally wide. Hind coxa finely punctate throughout, and dorsal outer depression present. Inner spur of hind tibia slightly longer than outer spur (0.21, 0.19); surface of hind tibia with strong spines only on distal half. Hind telotarsus slightly longer than fourth tarsomere (0.15, 0.12).

**Wings.** Fore wing with r vein straight; 2RS vein slightly convex to convex; r and 2RS veins forming a weak, even curve at their junction and outer side of junction forming a slight stub; 2M vein slightly curved/swollen; distally fore wing [where spectral veins are] with microtrichiae more densely concentrated than the rest of the wing; anal cell 1/3 proximally lacking microtrichiae; subbasal cell with microtrichiae virtually throughout; veins 2CUa and 2CUb completely spectral; vein 2 cu-a present as spectral vein, sometimes difficult to see; vein 2-1A proximally tubular and distally spectral, although sometimes difficult to see; tubular vein 1 cu-a curved, incomplete/broken and not reaching the edge of 1-1A vein. Hind wing with vannal lobe narrow, subdistally evenly convex, subproximally evenly convex, and setae evenly scattered in the margin.

**Metasoma** (Fig. 7G, H, J). Metasoma cylindrical; petiole finely sculptured only laterally, evenly narrowing distally with apex truncate (length 0.40, maximum width 0.17, minimum width 0.12), with scattered pubescence on distal half only laterally. Lateral grooves delimiting the median area on T2 distally losing definition (length median area 0.10, length T2 0.17), edges of median area with little sculpture, median area broader than long (length median area 0.10, maximum width 0.25, minimum width 0.10); T2 with scattered pubescence only distally. T3 longer than T2 (0.22, 0.17) and with scattered pubescence throughout. Pubescence on hypopygium dense.

**Cocoons.** White oval cocoons with silk fibers messy/disordered/fluffy. Cocoons somewhat scattered and adhered on leaf surface.

#### Comments.

Both fore legs are missing. The third distal of mesoscutum is concave [with a dent]. The fronto-clypeal suture is present and dark delineated. The longitudinal median carina on the face is very short.

#### Male

(Fig. 8A–L). Similar in coloration to female, although in lateral view, all terga and sterna are brown (in females those are yellow). The fore legs are yellow with claws brown; both the dorsal and the ventral furrows of pronotum and epicnemial ridge with yellow coloration.

#### Etymology.

Alex V. Borisenko is a research associate, Director of International Programs, at the Biodiversity Institute of Ontario (BIO), University of Guelph, Ontario, Canada.

#### Distribution.

The parasitized caterpillars were collected in Costa Rica, ACG, Sector Cacao (Sendero Toma Agua and SenderoDerrumbe), during July 2002 and October 2006 at 1,140 m and 1,220 m in cloud forest.

#### Biology.

The lifestyle of this parasitoid species is solitary/gregarious.

#### Host.

*Cynea* sp. Evans (Hesperiidae, Hesperinae, skipper butterflies) feeding on *Renealmiaalpinia* (Zingiberaceae). *Salianaplacens* (Butler) (Hesperiidae, Hesperinae) feeding on *Costusscaber* (Costaceae). Caterpillars were collected in third and fifth instar.

### Glyptapanteles alexwildi

Taxon classificationAnimaliaHymenopteraBraconidae

Arias-Penna, sp. nov.

http://zoobank.org/0CB66A58-5817-48E8-96E4-9FDAF92ED1C8

Fig. 9

#### Male.

Body length 3.23 mm, antenna length 4.69 mm, fore wing length 3.53 mm.

#### Type material.

**Holotype**: ECUADOR • 1♀; EC-37439, YY-A162; Napo, Yanayacu Biological Station, Río Pumayacu, Plot 424; cloud forest; 2163 m; -0.5833, -77.8833; 06.iii.2009; CAPEA leg.; caterpillar collected in third instar; cocoons formed on 12.iii.2009; adult parasitoid emerged on 01.iv.2009; (PUCE).

#### Diagnosis.

**Figure 9.**
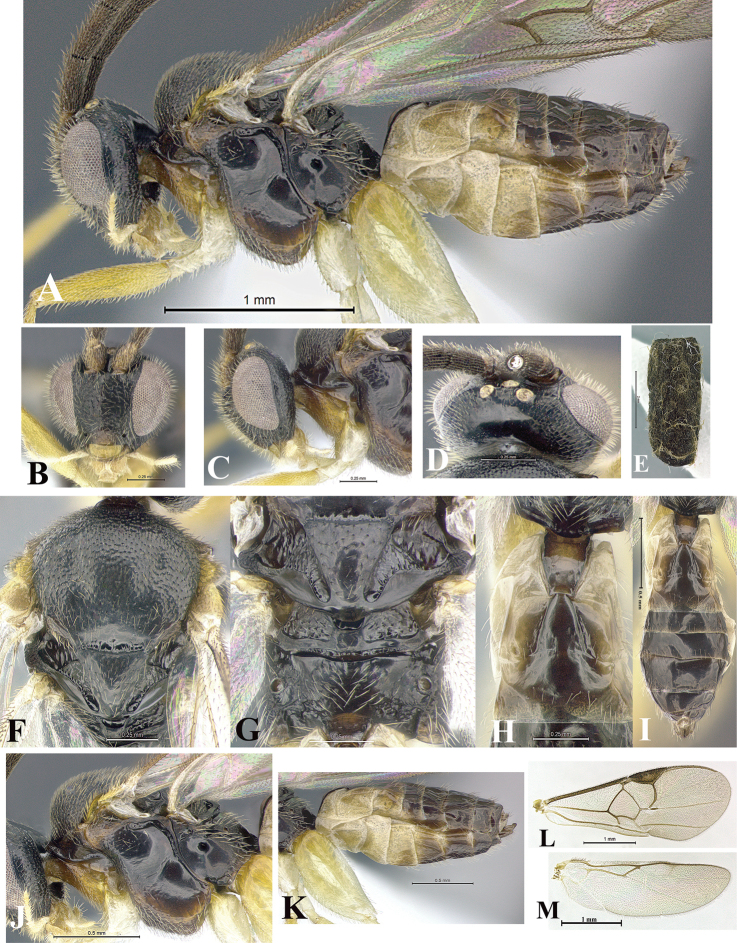
*Glyptapantelesalexwildi* sp. nov. male EC-37439 YY-A162 **A** Habitus **B, D** Head **B** Frontal view **D** Dorsal view **C** Head, pronotum, propleuron, lateral view **E** Cocoon **F** Mesonotum, dorsal view **G** Scutellum, metanotum, propodeum, dorsal view **H**T1–3, dorsal view **I, K** Metasoma **I** Dorsal view **K** Lateral view **J** Mesosoma, lateral view **L, M** Wings **L** Fore **M** Hind.

Nucha surrounded by very short radiating carinae (Fig. 9G), proximal half of propodeum weakly curved (Fig. 9G), antenna longer than body, mesoscutum punctation proximally distinct, but distally absent/dispersed (Fig. 9F), axillary trough of metanotum with small punctation throughout (Fig. 9G), inner margin of eyes diverging slightly at antennal sockets (Fig. 9B), petiole on T1 virtually parallel-sided, but narrowing over distal 1/3, completely smooth and polished, with faint, satin-like sheen (Fig. 9H, I), propodeum without median longitudinal carina (Fig. 9G), lateral grooves delimiting the median area on T2 clearly defined and reaching the distal edge of T2 (Fig. 9H, I), and fore wing with outer side of junction of r and 2RS veins not forming a stub (Fig. 9L).

#### Coloration

(Fig. 9A–M). General body coloration dark brown except clypeus, scape and pedicel (with some areas brown), propleuron, ventral edge from proximal to distal of mesopleuron, parts of dorsal and ventral furrows of pronotum, and epicnemial ridge yellow-brown; labrum, maxillae, maxillary and labial palps yellow. Eyes gray and ocelli yellowish. Fore and middle legs yellow except claws brown; hind legs yellow except a tiny brown dot at the apex of femora, tibiae medially yellow-brown and both ends brown, all tarsomeres brown. Petiole on T1 yellow-brown with the entire inner edge dark brown; T2 with median and wide adjacent areas light brown, and lateral ends yellow-brown; T3 completely light brown and distally with a narow whitish transparent band; T4 and beyond dark brown; distally each tergum with a narrow whitish transparent band. In lateral view, T1–3 completely yellow; T4 and beyond completely light brown. S1–3 completely yellow; S4 yellow, but medial brown; penultimate sternum and hypopygium completely brown.

#### Description.

**Head** (Fig. 9B–D). Head triangular with pubescence long and dense. Proximal three antennal flagellomeres longer than wide (0.27:0.11, 0.29:0.11, 0.29:0.11), distal antennal flagellomere longer than penultimate (0.19:0.07, 0.16:0.07), antenna longer than body (4.69, 3.23); antennal scrobes-frons shallow. Face flat or nearly so, with dense fine punctations, interspaces wavy and longitudinal median carina present. Frons punctate. Temple wide, punctate and interspaces wavy. Inner margin of eyes diverging slightly at antennal sockets; in lateral view, eye anteriorly convex and posteriorly straight. POL shorter than OOL (0.10, 0.13). Malar suture present. Median area between lateral ocelli without depression. Vertex laterally rounded and dorsally wide.

**Mesosoma** (Fig. 9F–G, J). Mesosoma dorsoventrally convex. Mesoscutum with narrow grooves/dents taking the place of notauli, punctation distinct proximally, but absent/dispersed distally, and interspaces wavy/lacunose. Scutellum triangular, apex sloped and fused with BS, scutellar punctation distinct throughout, in profile scutellum slightly convex, but on same plane as mesoscutum, phragma of the scutellum completely concealed; BS only very partially overlapping the MPM; ATS demilune with short stubs delineating the area; dorsal ATS groove with carinae only proximally. Transscutal articulation with small and homogeneous foveae, area just behind transscutal articulation depressed centrally, sculpture on area just behind transscutal articulation smooth and shiny. Metanotum with BM wider than PFM (clearly differentiated); MPM semicircular without median longitudinal carina; AFM without setiferous lobes and not as well delineated as PFM; PFM thick and smooth; ATM small punctate throughout. Propodeum without median longitudinal carina, proximal half weakly curved with medium-sized sculpture and distal half with a shallow dent at each side of nucha; distal edge of propodeum with a flange at each side and without stubs; propodeal spiracle without distal carina; nucha surrounded by very short radiating carinae. Pronotum with a distinct dorsal furrow, dorsally with a defined smooth band only proximally; central area of pronotum and dorsal furrow smooth, but ventral furrow with short parallel carinae. Propleuron with fine rugae and dorsally without a carina. Metasternum flat or nearly so. Contour of mesopleuron straight/angulate or nearly so; precoxal groove deep and with faintly lineate sculpture; epicnemial ridge elongated, more fusiform (tapering at both ends).

**Legs.** Ventral margin of fore telotarsus entire without seta, fore telotarsus almost same width throughout and longer than fourth tarsomere (0.15, 0.08). Hind coxa with punctation only on ventral surface, dorsal outer depression present. Inner spur of hind tibia much longer than outer spur (0.42, 0.31). Entire surface of hind tibia with dense strong spines clearly differentiated by color and length. Hind telotarsus longer than fourth tarsomere (0.20, 0.16).

**Wings** (Fig. 9L, M). Fore wing with r vein curved; 2RS vein straight; r and 2RS veins forming a weak, even curve at their junction and outer side of junction not forming a stub; 2M vein slightly curved/swollen; distally fore wing [where spectral veins are] with microtrichiae more densely concentrated than the rest of the wing; anal cell 1/3 proximally lacking microtrichiae; subbasal cell with a small smooth area; veins 2CUa and 2CUb completely spectral; vein 2 cu-a present as spectral vein, sometimes difficult to see; vein 2-1A proximally tubular and distally spectral, although sometimes difficult to see; tubular vein 1 cu-a curved and complete, but junction with 1-1A vein spectral. Hind wing with vannal lobe narrow, subdistally and subproximally straightened, and setae evenly scattered in the margin.

**Metasoma** (Fig. 9H, I, K). Metasoma laterally compressed. Petiole on T1 completely smooth and polished, with faint, satin-like sheen, virtually parallel-sided over most of length, but narrowing over distal 1/3 (length 0.40, maximum width 0.19, minimum width 0.10) and with scattered pubescence concentrated in the first distal third. Lateral grooves delimiting the median area on T2 clearly defined and reaching the distal edge of T2 (length median area 0.21, length T2 0.21), edges of median area polished, lateral grooves deep, median area as broad as long (length 0.21, maximum width 0.20, minimum width 0.07); T2 with scattered pubescence only distally. T3 longer than T2 (0.27, 0.21) and with scattered pubescence only distally.

**Cocoon** (Fig. 8E). Dark oval cocoon with silk fibers messy/disordered/fluffy.

#### Comments.

The pronotum is elevated in the middle part (convex) and distally at different level that mesopleuron, there forming a deep hollow.

#### Female.

Unknown.

#### Etymology.

Alexander (Alex) L. Wild, is an American entomologist and photographer who worked on ant evolution. His photographs appear in numerous natural history museums, magazines, books, television programs, and other media. Currently, he works at the University of Texas, Austin, USA.

#### Distribution.

Parasitized caterpillar was collected in Ecuador, Napo, Yanayacu Biological Station (Río Pumayacu), during March 2009 at 2,163 m in cloud forest.

#### Biology.

The lifestyle of this parasitoid species is solitary.

#### Host.

Undetermined species of Noctuidae feeding on Diplaziumcostalevar.robustum (Dryopteridaceae). Caterpillar was collected at third instar.

### Glyptapanteles alvarowillei

Taxon classificationAnimaliaHymenopteraBraconidae

Arias-Penna, sp. nov.

http://zoobank.org/BB106A5B-D964-4E46-897E-DF5DF9729597

Figs 10 , 11

#### Female.

Body length 3.81 mm, antenna length 3.33 mm, fore wing length 3.17 mm.

#### Type material.

**Holotype**: COSTA RICA • 1♀; 02-SRNP-8901, DHJPAR0000031; Área de Conservación Guanacaste, Guanacaste, Sector Cacao, Sendero Maritza; cloud forest; 760 m; 10.93644, -85.47764; 14.iv.2002; Freddy Quesada leg.; caterpillar collected in fourth instar; mass of somewhat separate white cocoons completely filling the caterpillar cocoon formed on 02.v.2002; adult parasitoids emerged on 16.v.2002; (CNC). **Paratypes.** • 40 (4♀, 5♂) (24♀, 5♂); 02-SRNP-8901, DHJPAR0000031; same data as for holotype; (CNC).

#### Other material.

**Reared material.** COSTA RICA: *Área de Conservación Guanacaste*, *Guanacaste*, *Sector Cacao*, *SenderoDerrumbe*: • 30 (7♀, 1♂) (22♀, 0♂); 00-SRNP-9564, DHJPAR0000004; cloud forest; 1,220 m; 10.92918, -85.46426; 29.v.2000; Mariano Pereira leg.; caterpillar collected in fourth instar; elongate white cocoons, separate and formed on 07.vi.2000; adult parasitoids emerged on 22.vi.2000. • 41 (3♀, 3♂) (28♀, 7♂); 08-SRNP-35029, DHJPAR0020725; same data as for preceding except: 05.ii.2008; Dunia Garcia leg.; caterpillar collected in fifth instar; mass of white fluffy cocoons barely adhered together; date of cocoons not reported; adult parasitoids emerged on 23.ii.2008.

*Área de Conservación Guanacaste*, *Guanacaste*, *Sector Cacao*, *Sendero Circular*: • 17 (6♀, 1♂) (9♀, 1♂); 02-SRNP-9369, DHJPAR0000032; cloud forest; 1,185 m; 10.92714, -85.46683; 15.v.2002; Freddy Quesada leg.; caterpillar collected in fourth instar; white cocoons not glued together; adult parasitoids emerged on 05.vi.2002. • 52 (5♀, 5♂) (36♀, 6♂); 02-SRNP-23276, DHJPAR0000026; same data as for preceding except: 19.vii.2002; Mariano Pereira leg.; characteristics of cocoon not reported; adult parasitoids emerged on 10.viii.2002.

#### Diagnosis.

**Figure 10.**
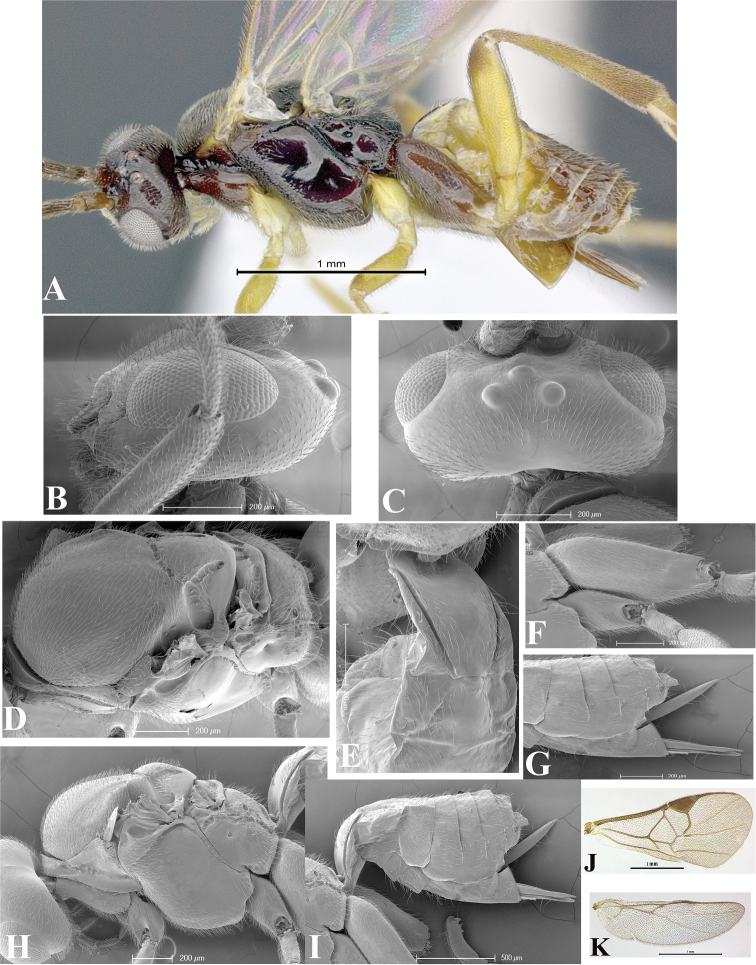
*Glyptapantelesalvarowillei* sp. nov. female 02-SRNP-8901 DHJPAR0000031 **A** Habitus **B, C** Head **B** Lateral view **C** Dorsal view **D, H** Mesosoma **D** Dorsolateral view **H** lateral view **E**T1–2, dorsolateral **F** Hind coxa, lateral view **G** Genitalia: hypopygium, ovipositor, ovipositor sheaths, lateral view **I** Metasoma, lateral view **J, K** Wings **J** Fore **K** Hind.

**Figure 11.**
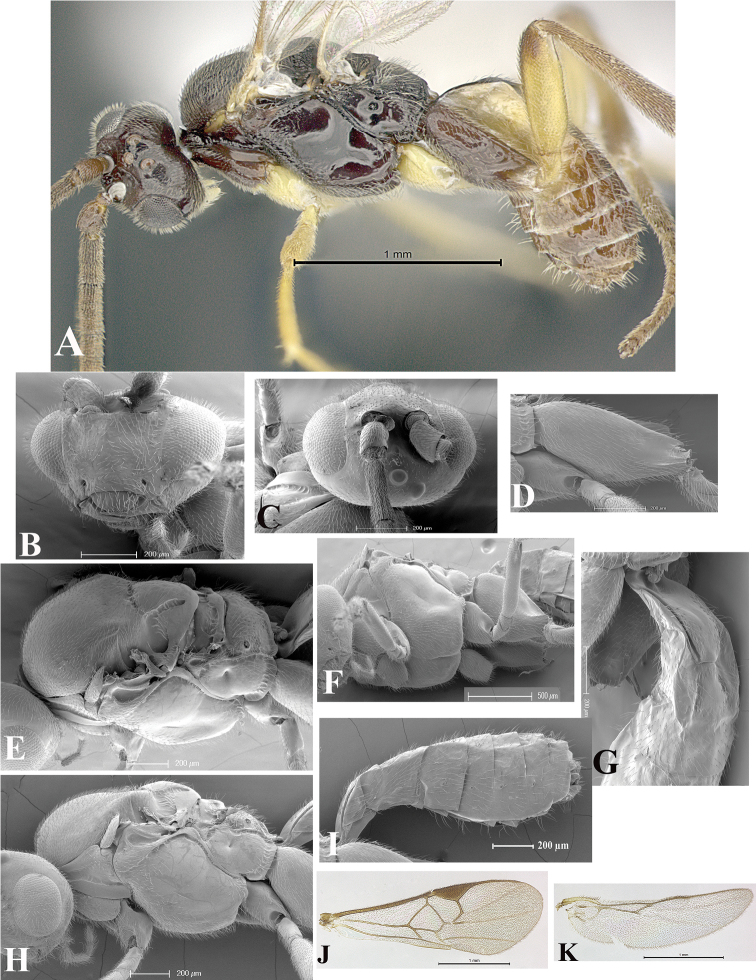
*Glyptapantelesalvarowillei* sp. nov. male 02-SRNP-8901 DHJPAR0000031 **A** Habitus **B, C** Head **B** Frontal view **C** Dorsal view **D** Hind coxa, lateral view **E, F, H** Mesosoma **E** Dorsolateral view **F** ventrolateral view **H** Lateral view **G**T1–3, dorsolateral view **I** Metasoma, lateral view **J, K** Wings **J** Fore **K** Hind.

In lateral view scutellum slightly higher than mesoscutum (Figs 10H, 11H), T3 as longer as T2 (Fig. 11G), longitudinal median carina on face absent (Fig. 11B), antenna shorter than body, distal antennal flagellomere longer than penultimate, petiole on T1 virtually parallel-sided but narrowing over distal 1/3 (Figs 10E, 11G), surface of metasternum flat or nearly so, edges of median area on T2 obscured by weak longitudinal stripes (Figs 10E, 11G), dorsal outer depression on hind coxa absent (Figs 10F, 11D), and fore wing with r vein slightly curved, outer side of junction of r and 2RS veins forming a slight stub (Figs 10J, 11J).

#### Coloration

(Fig. 10A). General body coloration brown-red/reddish except labrum and maxillae yellow-brown; glossa, maxillary and labial palps yellow; propleuron, pronotum, epicnemial ridge, ventral edge of mesopleuron, distal edge of mesoscutum, lunules, BS, PFM, BM, and proximal-medial part of propodeum with yellow-brown tints. Eyes and median ocellus silver, lateral ocelli brown. Fore and middle legs yellow although middle tarsomeres with brown tints; hind legs yellow except coxae completely brown-red/reddish, distally femora with a tiny dot, tibiae and tarsi brown. Petiole on T1 brown-red/reddish coloration intensifying at the edges, thus contours darkened, sublateral areas light yellow-brown; T2 with median and adjacent areas brown, and lateral ends yellow-brown; T3 extended brown coloration, but lateral areas yellow-brown; T4 and beyond completely brown and distally each tergum with a narrow whitish translucent band. In lateral view, T1–3 completely yellow, T4 and beyond dorsally brown and ventrally yellow, extent of brown coloration intensity increasing from proximal to distal. S1–3 completely yellow; S4 yellow, but medially brown; penultimate sternum and hypopygium brown.

#### Description.

**Head** (Fig. 10B, C). Head triangular with long and dense pubescence. Proximal three antennal flagellomeres longer than wide (0.27:0.08, 0.25:0.08, 0.25:0.08); distal antennal flagellomere longer than penultimate (0.15:0.05, 0.11:0.06); antenna shorter than body (3.33, 3.81); antennal scrobes-frons shallow. Face with scattered and finely punctate, interspaces smooth, distal half dented, but only laterally and longitudinal median carina absent. Frons smooth. Temple wide, punctate and interspaces clearly smooth. Inner margin of eyes straight throughout; in lateral view, eye anteriorly convex and posteriorly straight. POL shorter than OOL length (0.07, 0.13). Malar suture present. Median area between lateral ocelli without depression. Vertex laterally pointed or nearly so and dorsally wide.

**Mesosoma** (Fig. 10D, H). Mesosoma dorsoventrally convex. Mesoscutum with narrow grooves laterally, punctation distinct throughout and interspaces smooth. Scutellum triangular with punctation distinct throughout, apex sloped and fused with BS; in profile scutellum convex and slightly higher than mesoscutum; phragma of the scutellum partially exposed; BS only very partially overlapping the MPM; ATS demilune proximally with undulate carinae and distally smooth; dorsal ATS groove with carinae only proximally. Transscutal articulation with small and heterogeneous foveae; area just behind transscutal articulation with a smooth and shiny sloped transverse strip. Metanotum with BM wider than PFM (clearly differentiated); MPM circular without median longitudinal carina; AFM without setiferous lobes and not as well delineated as PFM; PFM thick and smooth; ATM proximally with semircular/undulate carina and distally smooth. Propodeum without median longitudinal carina, entirely with fine sculpture, proximal half of propodeum curved and distal half with a shallow dent at each side of nucha; distal edge of propodeum with a flange at each side and without stubs; propodeal spiracle without distal carina; nucha surrounded by very short radiating carinae. Pronotum with a distinct dorsal furrow, dorsally with a well-defined smooth band; central area of pronotum smooth, but both dorsal and ventral furrows with short parallel carinae. Propleuron finely sculpture throughout and dorsally without a carina. Metasternum flat or nearly so. Contour of mesopleuron convex; precoxal groove smooth, shiny and shallow, but visible; epicnemial ridge convex, teardrop-shaped.

**Legs** (Fig. 10A, F). Fore telotarsus proximally narrow and distally wide and longer than fourth tarsomere (0.10, 0.05). Hind coxa finely punctate throughout, and dorsal outer depression absent. Inner spur of hind tibia longer then outer spur (0.26, 0.21); entire surface of hind tibia with dense strong spines clearly differentiated by color and length. Hind telotarsus shorter than fourth tarsomere (0.12, 0.15).

**Wings** (Fig. 10J, K). Fore wing with r vein slightly curved; 2RS vein slightly convex to convex; r and 2RS veins forming a weak, even curve at their junction and outer side of junction forming a slight stub; shape of 2M vein slightly curved/swollen; distally fore wing [where spectral veins are] with microtrichiae more densely concentrated than the rest of the wing; anal cell with 1/3 proximal lacking microtrichiae; subbasal cell with microtrichiae virtually throughout; veins 2CUa and 2CUb completely spectral; vein 2 cu-a present as spectral vein, sometimes difficult to see; vein 2-1A proximally tubular and distally spectral, although sometimes difficult to see; tubular vein 1 cu-a straight, incomplete/broken, not reaching the edge of 1-1A vein. Hind wing with vannal lobe narrow, subdistally evenly convex, subproximally straightened, and setae evenly scattered in the margin.

**Metasoma** (Fig. 10E, G, I). Metasoma laterally compressed. Petiole on T1 finely sculptured only laterally, virtually parallel-sided over most of length, but narrowing over distal 1/3, apex truncate (length 0.45, maximum width 0.26, minimum width 0.11), and with scattered pubescence on distal half, but only laterally. Lateral grooves delimiting the median area on T2 distally losing definition (length median area 0.15, length T2 0.22), edges of median area obscured by weak longitudinal stripes, median area broader than long (length 0.15, maximum width 0.20, minimum width 0.10); T2 with scarce pubescence throughout. T3 slightly longer than T2 (0.24, 022) and with scattered pubescence throughout. Pubescence on hypopygium scattered.

**Cocoons** (Fig. 4G). White oval cocoons with silk fibers messy/disordered/fluffy. Mass of elongate fluffy, white cocoons somewhat separate and completely filling the caterpillar cocoon.

#### Comments.

In both sexes, laterally the mesoscutum with a narrow dent extending throughout almost all of its length (Fig. 10D, H). In some females, the body coloration is brown, thus the tints in the body are brown-red/reddish.

#### Male

(Fig. 11A–K). Similar in coloration to female.

#### Etymology.

Álvaro Wille Trejos (May 17, 1928-June 11, 2006) was a well-known Costa Rican entomologist. Most of his publications were on bees, especially the phylogeny, behavior, and systematics of stingless bees (Meliponini).

#### Distribution.

The parasitized caterpillars were collected in Costa Rica, ACG, Sector Cacao (Sendero Circular, SenderoDerrumbe, and Sendero Maritza), during May 2000, April-May and July 2002, and February 2008 at 760 m, 1,185 m, and 1,220 m in cloud forest.

#### Biology.

The lifestyle of this parasitoid species is gregarious.

#### Host.

*Pachydotadrucei* Rothschild (Erebidae: Arctiinae) (Fig. 4G) feeding on *Ocoteawhitei* (Lauraceae). Caterpillars were collected in fourth and fifth instar.

### Glyptapanteles andrewdebeveci

Taxon classificationAnimaliaHymenopteraBraconidae

Arias-Penna, sp. nov.

http://zoobank.org/96052F6B-C379-48FA-9446-D25B58D7D189

Figs 12 , 13

#### Female.

Body length 2.53 mm, antenna length 3.38 mm, fore wing length 3.18 mm.

#### Type material.

**Holotype**: ECUADOR • 1♀; EC-43507, YY-A155; Napo, Yanayacu Biological Station, Stream trail, Plot 451; 2,006 m; cloud forest; -0.596722, -77.895556; 19.xi.2009; Wilmer Simbaña leg.; caterpillar collected in second instar; cocoons formed on 08.xii.2009; adult parasitoid emerged on 05.i.2010; (PUCE). **Paratype**. • 1 (0♀, 1♂) (0♀, 0♂); EC-41691, YY-A161; same data as for holotype except: Plot 439; 2,114 m; -0.594444, -77.888333; 18.viii.2009; Lee Dyer leg.; caterpillar collected in third instar; cocoons formed on 28.viii.2009; adult parasitoid emerged on 19.ix.2009; (PUCE).

#### Other material.

**Reared material.** ECUADOR: *Napo*, *Yanayacu Biological Station*, *Yanayacu Road*, *Plot 360*: • 1 (1♀, 0♂) (0♀, 1♂); EC-26062, YY-A222; 1,998 m; cloud forest; -0.566667, -77.866667; 10.ix.2007; Lauren Loe leg.; caterpillar collected in second instar; cocoons formed on 09.x.2007; adult parasitoid emerged on 26.xii.2007.

*Napo*, *Yanayacu Biological Station*, *Sendero de las Lágrimas*, *Plot 365*: • 1 (1♀, 0♂) (0♀, 0♂); EC-26559, YY-A223; 2,075 m; cloud forest; -0.598333, -77.882778; 24.ix.2007; Lauren Loe leg.; caterpillar collected in second instar; cocoons formed on 09.x.2007; adult parasitoid emerged on 03.xi.2007.

#### Diagnosis.

**Figure 12.**
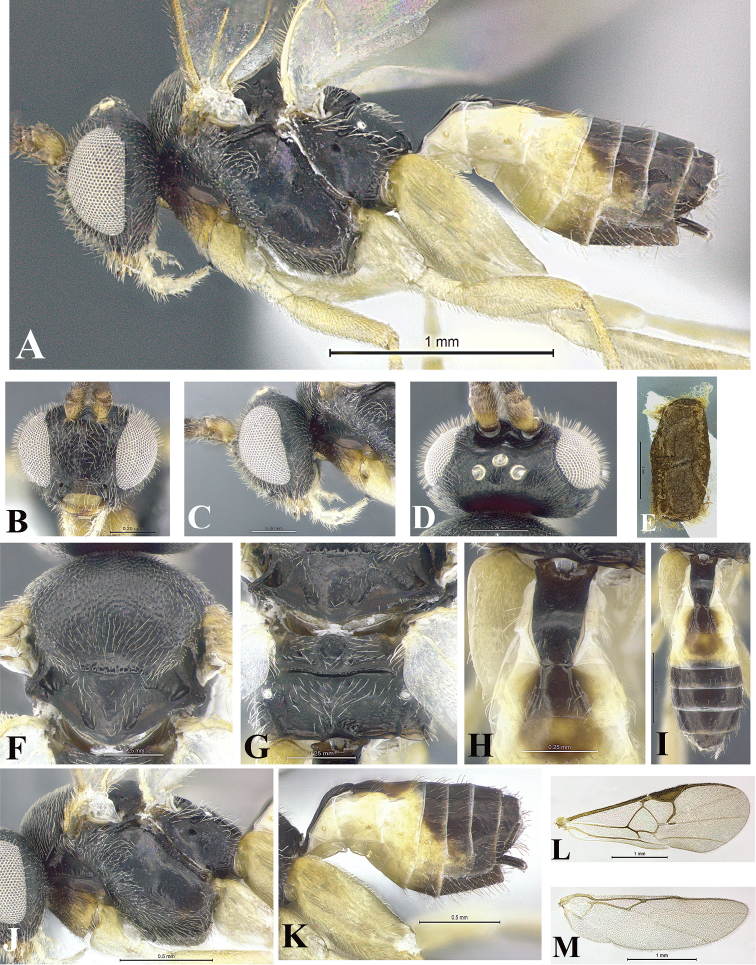
*Glyptapantelesandrewdebeveci* sp. nov. female EC-26559 YY-A223 **A** Habitus **B, D** Head **B** Frontal view **D** Dorsal view **C** Head, pronotum, propleuron, lateral view **E** Cocoon **F** Mesonotum, dorsal view **G** Scutellum, metanotum, propodeum, dorsal view **H**T1–2, dorsal view **I, K** Metasoma **I** Dorsal view **K** Lateral view **J** Mesosoma, lateral view **L, M** Wings **L** Fore **M** Hind.

**Figure 13.**
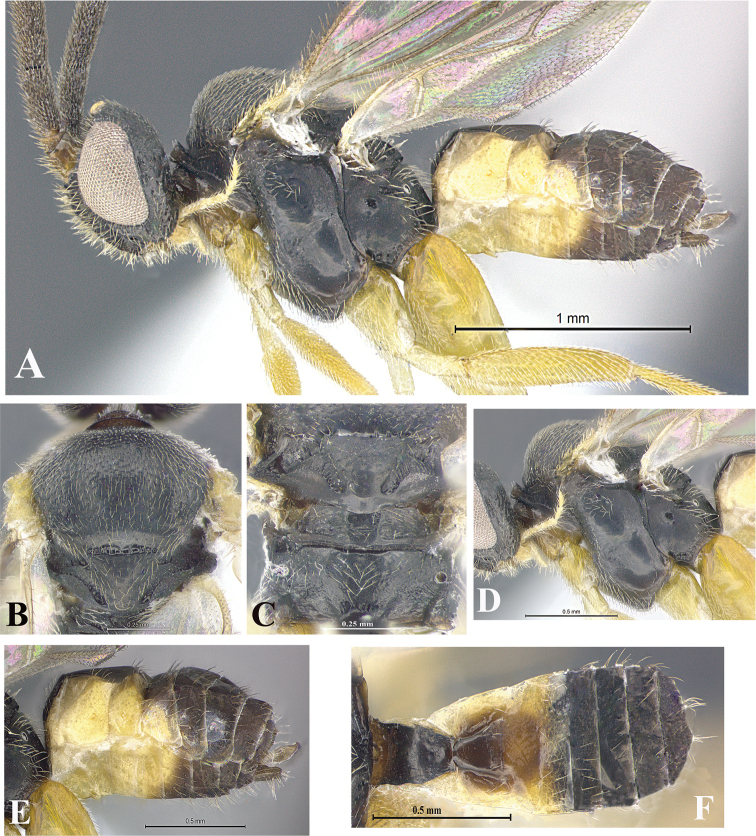
*Glyptapantelesandrewdebeveci* sp. nov. male EC-41691 YY-A161 **A** Habitus **B** Mesonotum, dorsal view **C** Scutellum, metanotum, propodeum, dorsal view **D** Mesosoma, lateral view **E, F** Metasoma **E** Lateral view **F** Dorsal view.

Petiole on T1 evenly narrowing throughout length (Figs 12H, I, 13F), precoxal groove deep (Figs 12A, J, 13A, D), anteroventral contour of mesopleuron straight/angulate or nearly so (Figs 12A, 13A), edges of median area on T2 polished and followed by a deep groove (Figs 12H, I, 13F), and fore wing with r vein curved, outer side of junction of r and 2RS veins forming a slight stub (Fig. 12L).

#### Coloration

(Fig. 12A–M). General body coloration dark brown except mandibles, scape and pedicel (both with lateral brown strip) yellow-brown; propleuron mostly brown with apex yellow; metasternum yellow; ventral edge of mesopleuron lighter than mesosoma coloration; labrum, maxillae, maxillary and labial palps, and tegulae yellow. Fore and middle legs yellow (in tarsomeres, yellow coloration intensity increasing from proximal to distal) except claws brown; hind legs yellow except a tiny brown dot at the apex of femora, tibiae with apex brown, all tarsomeres brown. Petiole on T1 dark brown with contours darkened and sublateral areas yellow; T2 with median and adjacent areas dark brown, and lateral ends yellow; T3 with an extended brown area which proximally coincides with width of dark area on T2, but distally narrow; T4 and beyond black. In lateral view, T1–3 completely yellow, T4–5 yellow, but dorsally brown; following tergites completely dark brown. S1–4 completely yellow, penultimate sternite with proximal half yellow and distal half brown; hypopygium completely brown.

#### Description.

**Head** (Fig. 12B–D). Head rhomboid with pubescence long and dense. Proximal three antennal flagellomeres longer than wide (0.25:0.08, 0.26:0.08, 0.25:0.08), distal antennal flagellomere longer than penultimate (0.14:0.05, 0.11:0.05), antenna longer than body (3.38, 2.53); antennal scrobes-frons sloped and forming a shelf. Face flat or nearly so, with dense and fine punctations, interspaces smooth and longitudinal median carina present. Frons smooth. Temple wide, punctate and interspaces clearly smooth. Inner margin of eyes diverging slightly at antennal sockets; in lateral view, eye anteriorly convex and posteriorly straight. POL shorter than OOL (0.09, 0.13). Malar suture present. Median area between lateral ocelli slightly depressed. Vertex laterally pointed or nearly so and dorsally wide.

**Mesosoma** (Fig. 12F, G, J). Mesosoma dorsoventrally convex. Distal 1/3 of mesoscutum with lateral margin slightly dented, punctation distinct throughout, and interspaces smooth. Scutellum triangular, apex sloped and fused with BS, scutellar punctation scattered throughout, in profile scutellum flat and on same plane as mesoscutum, phragma of the scutellum partially exposed; BS only very partially overlapping the MPM; ATS demilune with a little, complete parallel carinae, dorsal ATS groove with carinae only proximally. Transscutal articulation with small and heterogeneous foveae, area just behind transscutal articulation with a smooth and shiny sloped transverse strip. Metanotum with BM wider than PFM (clearly differentiated); MPM circular without median longitudinal carina; AFM with a small lobe and not as well delineated as PFM; PFM thick and smooth; ATM proximally with a groove with some sculpturing and distally smooth. Propodeum relatively polished without median longitudinal carina, proximal half weakly curved; distal edge of propodeum with a flange at each side and without stubs; propodeal spiracle without distal carina; nucha surrounded by very short radiating carinae. Pronotum with a distinct dorsal furrow, dorsally with a well-defined smooth band; central area of pronotum smooth, but both dorsal and ventral furrows with short parallel carinae. Propleuron with fine rugae and dorsally with a carina. Metasternum flat or nearly so. Contour of mesopleuron straight/angulate or nearly so; precoxal groove deep and with transverse lineate sculpture; epicnemial ridge elongated, more fusiform (tapering at both ends).

**Legs** (Fig. 12A). Ventral margin of fore telotarsus slightly excavated and with a tiny curved seta, fore telotarsus almost same width throughout and longer than fourth tarsomere (0.15, 0.07). Hind coxa with punctation only on ventral surface, dorsal outer depression present, inner spur of hind tibia much longer than outer spur (0.35, 0.28), entire surface of hind tibia with dense strong spines clearly differentiated by color and length. Hind telotarsus longer than fourth tarsomere (0.21, 0.15).

**Wings** (Fig. 12L, M). Fore wing with r vein curved; r and 2RS veins forming a weak, even curve at their junction and outer side of junction forming a slight stub; 2M vein straight; distally fore wing [where spectral veins are] with microtrichiae more densely concentrated than the rest of the wing; anal cell 1/3 proximally lacking microtrichiae; subbasal cell with microtrichiae virtually throughout; veins 2CUa and 2CUb completely spectral; vein 2 cu-a present as spectral vein, sometimes difficult to see; vein 2-1A proximally tubular and distally spectral, although sometimes difficult to see; tubular vein 1 cu-a curved and complete, but junction with 1-1A vein spectral. Hind wing with vannal lobe wide, subdistally evenly convex, subproximally straightened, and setae evenly scattered in the margin.

**Metasoma** (Fig. 12H, I, K). Metasoma laterally compressed. Petiole on T1 finely sculptured only laterally, evenly narrowing distally (length 0.40, maximum width 0.15, minimum width 0.10) and with scattered pubescence concentrated in the first distal third. Lateral grooves delimiting the median area on T2 clearly defined and reaching the distal edge of T2 (length median area 0.16, length T2 0.16), edges of median area polished and lateral grooves deep, median area as broad as long (length 0.16, maximum width 0.17, minimum width 0.08); T2 with scattered pubescence only distally. T3 longer than T2 (0.23, 0.16) and with scattered pubescence only distally. Pubescence on hypopygium dense.

**Cocoon** (Fig. 12E). Dark oval cocoon with silk fibers messy/disordered/fluffy.

#### Comments.

Some females have both the dorsal and the ventral furrows of pronotum lighter than mesosoma coloration. The coloration on T3 varies dorsally, some females with T3 mostly yellow, but with a central proximal yellow-brown spot that occupies 2/3 proximal and not touching the boundaries between T3–4. The lunules and PFM are lighter than mesosoma coloration.

#### Male

(Fig. 13A–F). Like other females, the T3 coloration varies dorsally, some males with T3 mostly yellow, but with a central proximal yellow-brown spot that occupies 2/3 proximal and not touching the boundaries between T3–4.

#### Etymology.

Andrew Henry Debevec is an American entomologist. As a graduate student at the UIUC, IL, USA, he was interested in Microgastrinae, mainly the genus *Xanthomicrogaster*. Now, he works as an IT specialist in the School of Integrative Biology at UIUC, IL, USA.

#### Distribution.

Parasitized caterpillars were collected in Ecuador, Napo, Yanayacu Biological Station (Stream trail), during September 2007, and August and November 2009 at 1,998 m, 2,006 m, 2,075 m, and 2,114 m in cloud forest.

#### Biology.

The lifestyle of this parasitoid species is solitary.

#### Host.

Undetermined species of Pyralidae and Noctuidae feeding on Diplaziumcostalevar.robustum (Dryopteridaceae). Caterpillars were collected in second and third instar.

### Glyptapanteles andybennetti

Taxon classificationAnimaliaHymenopteraBraconidae

Arias-Penna, sp. nov.

http://zoobank.org/96BADEC2-477E-4477-A22F-222D5584CB02

Figs 14 , 15

#### Female.

Body length 2.83 mm, antenna length 2.78 mm, fore wing length 2.73 mm.

#### Type material.

**Holotype**: COSTA RICA • 1♀; 05-SRNP-32118, DHJPAR0004225; Área de Conservación Guanacaste, Guanacaste, Sector Pitilla, Loaiciga; rain forest; 445 m; 11.01983, -85.41342; 15.vi.2005; Manuel Rios leg.; caterpillar collected on second instar; single beige-white cocoons formed on 07.vii.2005 and adhered to the larval cuticle; adult parasitoids emerged on 14.vii.2005; (CNC). **Paratypes.** • 71 (4♀, 5♂) (42♀, 20♂); 05-SRNP-32118, DHJPAR0004225; same data as for holotype; (CNC).

#### Other material.

**Reared material.** COSTA RICA: *Área de Conservación Guanacaste*, *Guanacaste*, *Sector Santa Rosa*, *Bosque Humedo*: • 17 (3♀, 0♂) (14♀, 0♂); 95-SRNP-11077, DHJPAR0000090; dry forest; 290 m; 10.85145, -85.60801; 14.xi.1995; gusaneros leg.; caterpillar collected in fifth instar; each separate white elongate oval cocoons adhered tightly to larval cuticle; adult parasitoids emerged on 04.xii.1995.

*Área de Conservación Guanacaste*, *Guanacaste*, *Sector El Hacha*, *Finca Araya*: • 138 (6♀, 5♂) (122♀, 5♂); 02-SRNP-4475, DHJPAR0000030; dry forest; 295 m; 11.01541, -85.51125; 22.i.2002; gusaneros leg.; caterpillar collected in fifth instar; cadaver of caterpillar covered with tightly packed separate white cocoons; cocoons at right angles adhered to the larval cuticle; adult parasitoids emerged on 07.ii.2002.

*Área de Conservación Guanacaste*, *Alajuela*, *Sector Rincón Rain Forest*, *Sendero Anonás*: • 56 (3♀, 3♂) (44♀, 6♂); 03-SRNP-10052, DHJPAR0001474; 405 m; 10.90528, -85.27882; 10.i.2003; José Perez leg.; caterpillar collected in fifth instar; elongate small white cocoons, adhered individually but in groups on back of caterpillar; adult parasitoids emerged on 28.i.2003.

#### Malaise-trapped material.

COSTA RICA: *Área de Conservación Guanacaste*, *Guanacaste*, *Sector Santa Rosa*, *Bosque Humedo*: • 1 (1♀, 0♂) (0♀, 0♂); 98-SRNP-16105, DHJPAR0013357; dry forest; 290 m; 10.85145, -85.60801; 05.i.1998; Malaise trap; DH Janzen & W Hallwachs leg.

#### Diagnosis.

**Figure 14.**
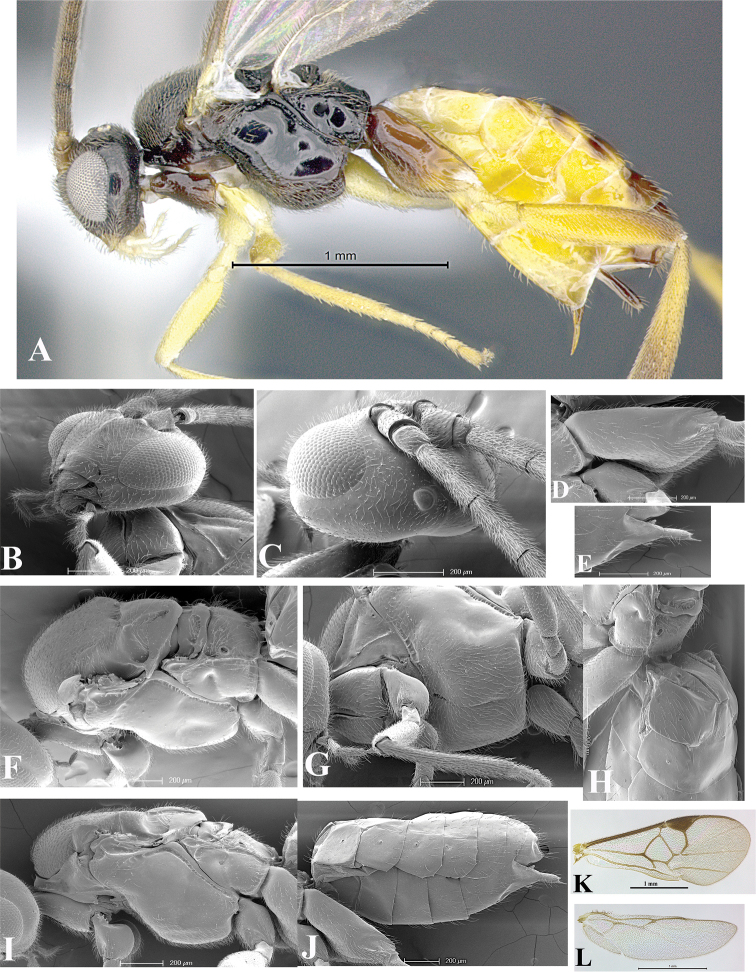
*Glyptapantelesandybennetti* sp. nov. female 05-SRNP-32118 DHJPAR0004225 **A** Habitus **B, C** Head **B** Laterofrontal view **C** Dorsal view **D** Hind coxa, lateral view **E** Genitalia: hypopygium, ovipositor sheaths, lateral view **F, G, I** Mesosoma **F** Dorsolateral view **G** Ventrolateral view **I** Lateral view **H** Propodeum, T1–3, laterodorsal view **J** Metasoma, lateral view **K, L** Wings **K** Fore **L** Hind.

**Figure 15.**
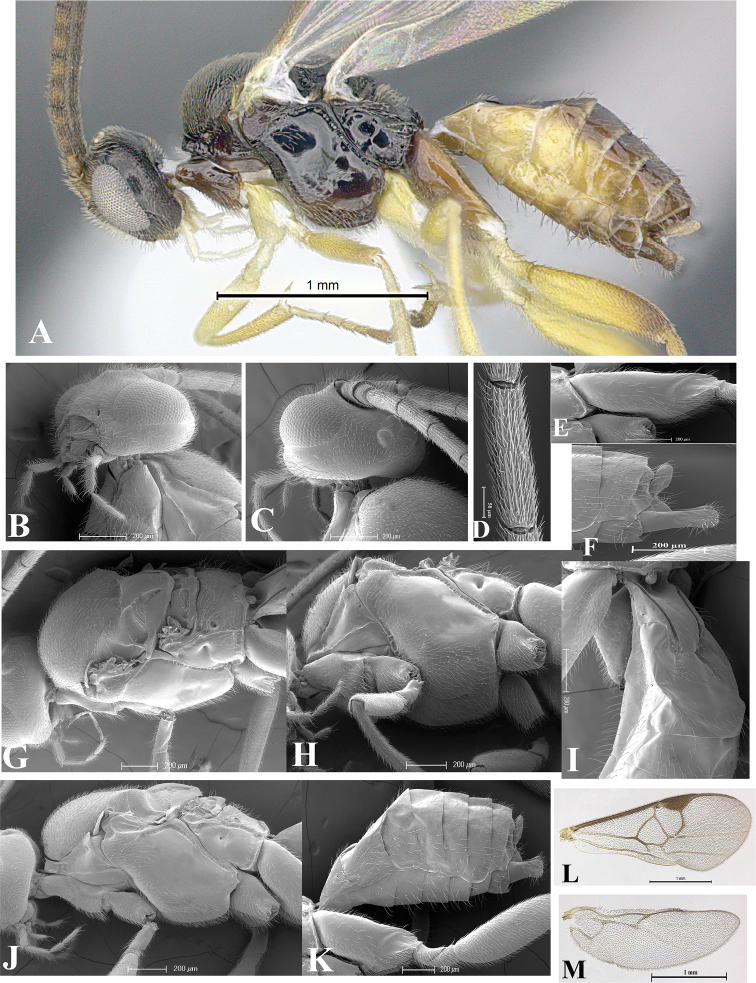
*Glyptapantelesandybennetti* sp. nov. male 05-SRNP-32118 DHJPAR0004225 **A** Habitus **B, C** Head **B** Laterofrontal view **C** Dorsal view **D** Flagellomeres **E** Hind coxa, lateral view **F** Genitalia: parameres, lateral view **G, H, J** Mesosoma **G** Dorsolateral view **H** Ventrolateral view **J** Lateral view **I**T1–3, laterodorsal view **K** Metasoma, lateral view **L, M** Wings **L** Fore **M** Hind.

Dorsal outer depression on hind coxa absent (Figs 14D, 15E), fore telotarsus longer than fourth tarsomere, antenna shorter than body, distal antennal flagellomere shorter than penultimate, vertex in dorsal view narrow (Figs 14C, 15C), scutellar punctation distinct throughout (Figs 14F, 15G), shape of proximal half of propodeum weakly curved in dorsal view, longitudinal median carina on face present (Figs 14B, 15B), lateral grooves delimiting the median area on T2 distally losing definition (Fig. 15I), propodeum without median longitudinal carina (Figs 14F, 15G), anteroventral contour of mesopleuron convex (Figs 14A, F, 15A, J), edges of median area on T2 polished and followed by a deep groove (Figs 14H, 15I), and fore wing with r vein curved, outer side of junction of r and 2RS veins forming a distinct stub (Figs 14K, 15L).

#### Coloration

(Fig. 14A). General body coloration dark-brown, except scape, pedicel, labrum, mandibles, and tegulae dark yellow; glossa, maxillary and labial palps pale yellow/ivory; low face, labrum, propleuron, pronotum, epicnemial ridge, ventral edge of mesopleuron, and metasternum with brown-reddish tints. Eyes and ocelli silver. Fore and middle legs yellow, claws brown; hind legs yellow except coxae light yellow-brown, apex of femora brown, 3/4 distal of tibiae and all tarsomeres yellow-brown. Petiole on T1 brown and sublateral areas yellow; T2 with median and narrow adjacent areas brown, and lateral ends yellow; T3–5 brown over most of the middle surface and with a narrow strip brown only proximally, but laterally with yellow-brown area; T6 and beyond completely brown. In lateral view, T1–2 completely yellow, T3 and beyond yellow, but dorsally with a small brown area. Each sternite completely yellow.

#### Description.

**Head** (Fig. 14B, C). Head triangular with pubescence long and dense. Proximal three antennal flagellomeres longer than wide (0.22:0.07, 0.22:007, 0.22:0.07); distal antennal flagellomere shorter than penultimate (0.14:0.06, 0.11:0.06), antenna shorter than body (2.78, 2.83); antennal scrobes-frons shallow. Face finely punctate-lacunose, interspaces wavy, middle with lateral depression and longitudinal median carina present. Frons smooth. Temple wide, punctations barely noticeable and interspaces clearly smooth. Inner margin of eyes straight throughout; in lateral view, eye anteriorly convex and posteriorly straight. POL shorter than OOL (0.08, 0.15). Malar suture present. Median area between lateral ocelli without depression. Vertex laterally rounded and dorsally narrow.

**Mesosoma** (Fig. 14F, G, I). Mesosoma dorsoventrally convex. Mesoscutum convex with punctation distinct throughout, interspaces smooth, and 1/3 distally with slightly dented lateral margins. Scutellum triangular, apex sloped and fused with BS, in profile flat and on same plane as mesoscutum, scutellar punctation distinct throughout, phragma of the scutellum partially exposed; BS only very partially overlapping the MPM; ATS demilune with complete undulate/reticulate carinae; dorsal ATS groove smooth. Transscutal articulation with small and heterogeneous foveae; area just behind transscutal articulation with a smooth and shiny sloped transverse strip. Metanotum with BM wider than PFM (clearly differentiated); MPM circular without median longitudinal carina; AFM without setiferous lobes and not as well delineated as PFM; PFM thick and smooth; ATM proximally with semircular/undulate carina and distally smooth. Propodeum without median longitudinal carina, proximal half weakly curved and with fine sculpture, and distal half relatively polished, distal edge of propodeum with a flange at each side and without stubs; propodeal spiracle without distal carina; nucha surrounded by very short radiating carinae. Pronotum with a distinct dorsal furrow, dorsally with a well-defined smooth band; central area of pronotum and dorsal furrow smooth, but ventral furrow with short parallel carinae. Propleuron finely sculptured only ventrally and dorsally without a carina. Metasternum flat or nearly so. Contour of mesopleuron convex; precoxal groove smooth, shiny and shallow, but visible; epicnemial ridge convex, teardrop-shaped.

**Legs** (Fig. 14A, D). Ventral margin of fore telotarsus entire without seta, fore telotarsus proximally narrow and distally wide and longer than fourth tarsomere (0.13, 0.06). Hind coxa finely punctate throughout, and dorsal outer depression absent. Inner spur of hind tibia longer than outer spur (0.22, 0.18); entire surface of hind tibia with dense strong spines clearly differentiated by color and length. Hind telotarsus as longer than fourth tarsomere (0.15, 0.11).

**Wings** (Fig. 14K, L). Fore wing r vein slightly curved; 2RS vein straight; r and 2RS veins forming a weak, even curve at their junction and outer side of junction forming a slight stub; 2M vein slightly curved/swollen. Distally fore wing [where spectral veins are] with microtrichiae more densely concentrated than the rest of the wing; anal cell 1/3 proximally lacking microtrichiae; subbasal cell with a small smooth area; veins 2CUa and 2CUb completely spectral; vein 2 cu-a absent, vein 2-1A proximally tubular and distally spectral, although sometimes difficult to see; tubular vein 1 cu-a straight, incomplete/broken, not reaching the edge of 1-1A vein. Hind wing with vannal lobe very narrow subdistally and subproximally straightened; and setae evenly scattered in the margin.

**Metasoma** (Fig. 14E, H, J). Metasoma laterally compressed. Petiole on T1 completely smooth and polished, with faint, satin-like sheen, petiole evenly narrowing distally with apex truncate (length 0.40, maximum width 0.17, minimum width 0.10) and pubescence absent. Lateral grooves delimiting the median area on T2 distally losing definition (length median area 0.08, length T2 0.16), edges of median area polished, median area broader than long (length 0.08, maximum width 0.15, minimum width 0.10); T2 with a distinctive row of pubescence only at the distal margin. T3 longer than T2 (0.25, 0.16), T3 with a distinctive row of pubescence only at the distal margin. Pubescence on hypopygium dense.

**Cocoons** (Fig. 4L). White/beige oval cocoons with silk fibers evenly smooth. Tightly packed separate elongate oval cocoons adhered tightly to larval cuticle.

#### Comments.

The coloration on metasoma is different in some specimens (e.g., 95-SRNP-11077): in lateral view, all terga and all sterna are yellow-brown; in dorsal view T3 and beyond are completely brown.

#### Male

(Fig. 15A–M). Similar in coloration to female but darkened. However, in lateral view, T1–2 completely yellow; T3 and beyond yellow/yellow-brown and dorsally brown. S1–3 yellow, but beyond all sterna medial brown.

#### Etymology.

Named after the noted ichneumonid wasp specialist Andrew M.R. Bennett, of the Canadian National Collection (CNC) of Insects, Arachnids and Nematodes, Ottawa, Ontario, Canada.

#### Distribution.

Parasitized caterpillars were collected in Costa Rica, ACG, Sector El Hacha (Finca Araya), Sector Pitilla (Loaiciga), Sector Rincón Rain Forest (Sendero Anonás), and Sector Santa Rosa (Bosque Humedo), during November 1995, January 2002, 2003, and June 2005 at 290 m, 295 m, 405 m, and 445 m in dry forest and rain forest.

Adult parasitoid was collected in Costa Rica, ACG, Sector Santa Rosa (Bosque Humedo), during January 1998 at 290 m in dry forest.

#### Biology.

The lifestyle of this parasitoid species is gregarious.

#### Host.

*Unzelajapix* (Cramer) (Sphingidae: Macroglossinae, hawkmoths) feeding on *Davillakunthii*, *D.nitida*, *Doliocarpusmultiflorus*, and *Tetraceravolubilis* (Dilleniaceae). Caterpillars were collected in second and fifth instars (dead).

### Glyptapanteles andydeansi

Taxon classificationAnimaliaHymenopteraBraconidae

Arias-Penna, sp. nov.

http://zoobank.org/BF5A7B01-6515-461E-AA64-9F8B42F44D2C

Figs 16 , 17

#### Female.

Body length 3.5 mm, antenna length 3.85 mm, fore wing length 3.7 mm.

#### Type material.

**Holotype**: COSTA RICA • 1♀; 03-SRNP-20108, DHJPAR0000037; Área de Conservación Guanacaste, Guanacaste, Sector Pitilla, Estación Pitilla; rain forest; 675 m; 10.98931, -85.42581; 11.vii.2003; Petrona Rios leg.; caterpillar collected in fourth instar; mass of cocoons adhered to the larval cuticle, but not to each other, and fall off easily, formed on 26.vii.2003; adult parasitoids emerged on 01.viii.2003; (CNC). **Paratypes.** • 30 (4♀, 5♂) (20♀, 1♂); 03-SRNP-20108, DHJPAR0000037; same data as for holotype; (CNC).

#### Other material.

**Reared material.** COSTA RICA: *Área de Conservación Guanacaste*, *Guanacaste*, *Sector Pitilla*, *Estación Pitilla*: • 1 (1♀, 0♂) (0♀, 0♂ but many pieces in alcohol, website said 58 in total emerged); 03-SRNP-20107, DHJPAR0000036; rain forest; 675 m; 10.98931, -85.42581; 11.vii.2003; Petrona Rios leg.; caterpillar collected in fifth instar; cocoons adhered to the larval cuticle; adult parasitoids emerged on 24.vii.2003. • 26 (5♀, 1♂) (20♀, 0♂); 03-SRNP-20109, DHJPAR0000038; same data as for preceding except: caterpillar collected in second instar; large number of single white cocoons not fluffy, but very close together that fall easily off the living larva and formed on 28.vii.2003 and adhered to the larval cuticle; adult parasitoids emerged on 01.viii.2003. • 7 (2♀, 4♂) (1♀, 0♂); 03-SRNP-20132, DHJPAR0000039; same data as for preceding except: 12.vii.2003; caterpillar collected in fourth instar; isolated white tight elongated cylinders that stick to the back of the caterpillar in the last instar; adult parasitoids emerged on 21.xii.2003.

*Área de Conservación Guanacaste*, *Guanacaste*, *Sector Pitilla*, *Ingas*: • 190 (5♀, 5♂) (180♀, 0♂); 11-SRNP-31470, DHJPAR0042952; rain forest; 580 m; 11.00311, -85.42041; 23.v.2011; Freddy Quesada leg.; caterpillar collected in fourth instar; cocoons formed on 06.vi.2011 and adhered to the larval cuticle; adult parasitoids emerged on 11.vi.2011.

*Área de Conservación Guanacaste*, *Guanacaste*, *Sector Pitilla*, *Sendero Orosilito*: • 108 (5♀, 0♂) (103♀, 0♂); 11-SRNP-31486, DHJPAR0045148; rain forest; 900 m; 10.98332, -85.43623; 25.v.2011; Manuel Rios leg.; caterpillar collected in third instar; cocoons formed on 19.vi.2011 and adhered to the larval cuticle; adult parasitoids emerged on 23.vi.2011.

*Área de Conservación Guanacaste*, *Alajuela*, *Sector Rincón Rain Forest*, *Sendero Anonás*: • 41 (3♀, 3♂) (29♀, 6♂); 03-SRNP-11971, DHJPAR0000269; 405 m; 10.90528, -85.27882; 01.viii.2003; Osvaldo Espinoza leg.; caterpillar collected in fifth instar; many white cocoons adhered lightly to the back of the caterpillar and bunched, formed on 03.viii.2003; adult parasitoids emerged on 11.viii.2003.

#### Diagnosis.

**Figure 16.**
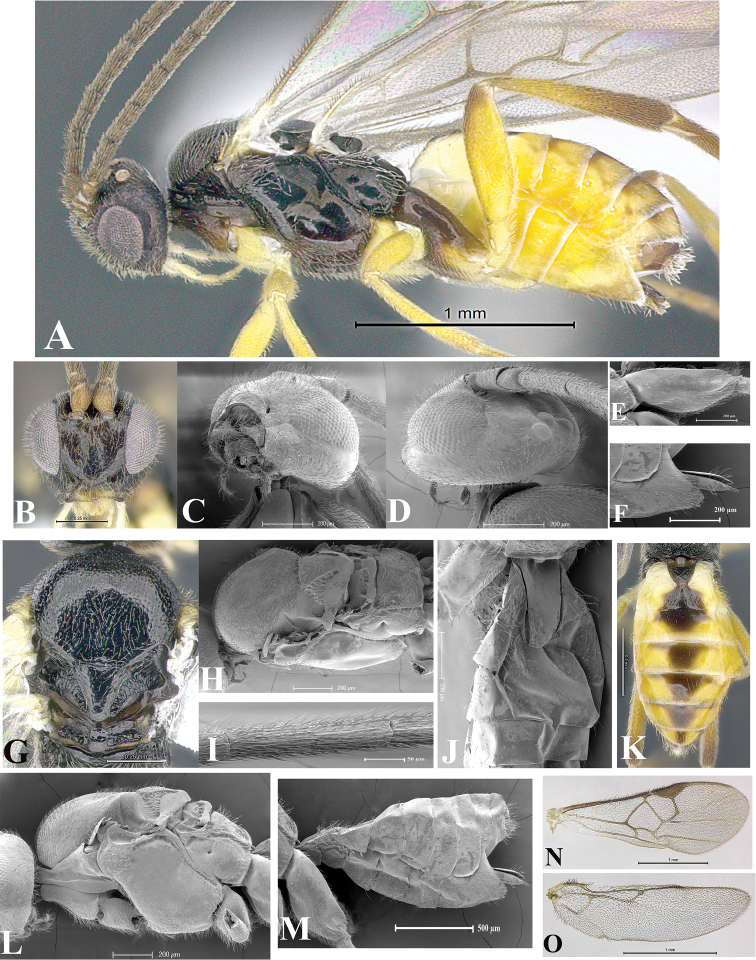
*Glyptapantelesandydeansi* sp. nov. female 03-SRNP-20108 DHJPAR0000037 **A** Habitus **B–D** Head **B** Frontal view **C** Ventrolateral view **D** Dorsolateral view **E** Hind coxa, lateral view **F** Genitalia: hypopygium, ovipositor, ovipositor sheaths, lateral view **G** Mesonotum, dorsal view **H, L** Metasoma **H** Dorsolateral view **L** Lateral view **I** Flagellomeres **J**T1–3 dorsolateral **K, M** Metasoma **K** Dorsal view **M** Lateral view **N, O** Wings **N** Fore **O** Hind.

**Figure 17.**
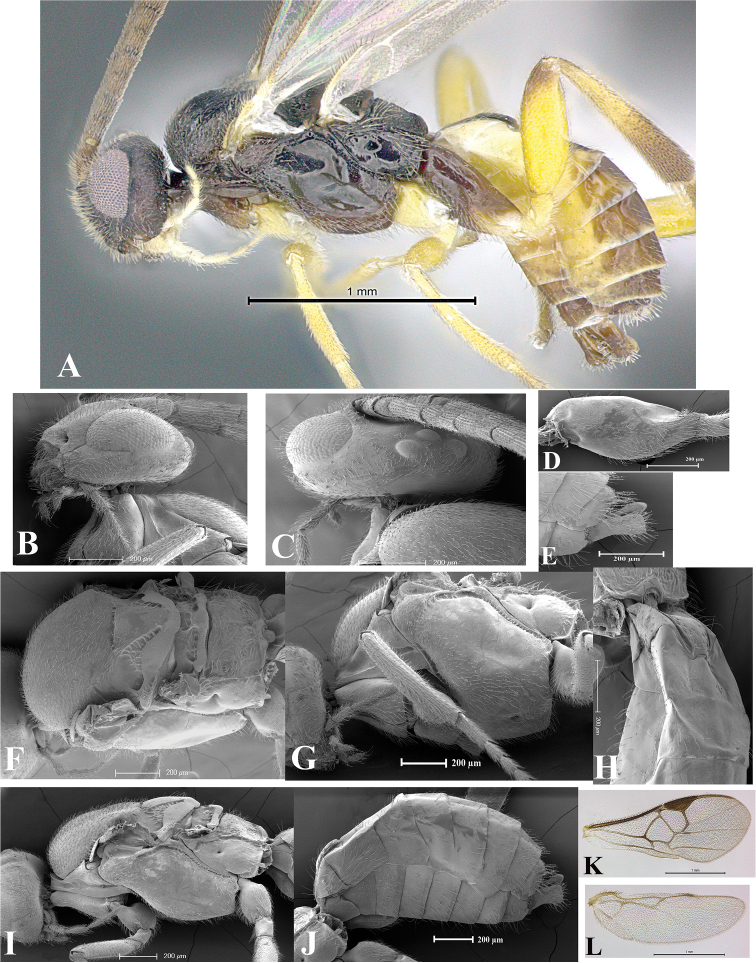
*Glyptapantelesandydeansi* sp. nov. male 03-SRNP-20108 DHJPAR0000037 **A** Habitus **B, C** Head **B** Lateral view **C** Dorsal view **D** Hind coxa, lateral view **E** Genitalia: parameres, lateral view **F, G, I** Mesosoma **F** Dorsolateral view **G** Ventrolateral view **I** Lateral view **H**T1–3 laterodorsal view **J** Metasoma, lateral view **K, L** Wings **K** Fore **L** Hind.

Scutellar punctation distinct peripherally, absent centrally (Fig. 16G), area just behind transscutal articulation smooth and shiny (Figs 16G, H, 17F), inner margin of eyes straight throughout (Fig. 16B), vertex in dorsal view narrow (Figs 16D, 17C), lateral grooves delimiting the median area on T2 clearly defined and reaching the distal edge of T2 (Fig. 16J, K), and fore wing with r vein straight, outer side of junction of r and 2RS veins forming a stub (Fig. 16N).

#### Coloration

(Fig. 16A, B, K). General body coloration brown-black, except scape, pedicel, mandibles, propleuron, BS, lunules, PFM and BM with light brown tints; ventral edge of mesopleuron, metasternum, and distal edge of mesoscutum reddish brown; maxillary and labial palps, and tegulae yellow. Eyes purple (in preserved specimen) and ocelli yellowish. Fore legs yellow except brown claw; middle legs yellow although tarsomeres yellow-brown; hind legs yellow except coxae brown (distally yellow-brown and proximally light brown), small area on the apex of the femora brown, distal half of tibiae and all tarsomeres brown. Petiole on T1 brown and sublateral areas yellow; T2 with median and adjacent areas brown, adjacent area wide and with contours well-defined, and lateral ends brown; T3 and remaining terga medially with an area dark brown wider proximally than distally, and sublateral areas yellow; however on T4 and beyond that brown area is narrower than T3; T3 and beyond distally with a narrow yellowish transparent band. In lateral view, all terga yellow. All sterna yellow, although hypopygium medially brown.

#### Description.

**Head** (Fig. 16B–D, I). Head triangular with pubescence long and dense. Proximal three antennal flagellomeres longer than wide (0.23:0.07, 0.22:0.07, 0.23:0.07), distal antennal flagellomere longer than penultimate (0.14:0.05, 0.12:0.05), antenna longer than body (3.85, 3.50); antennal scrobes-frons shallow. Face flat or nearly so, with scattered and fine sculpture, interspaces smooth and longitudinal median carina present. Frons smooth. Temple wide with punctate sculpture and interspaces clearly smooth. Inner margin of eyes straight throughout; in lateral view, eye anteriorly convex and posteriorly straight. POL shorter than OOL (0.10, 0.14). Malar suture present. Median area between lateral ocelli without depression. Vertex laterally rounded and dorsally narrow.

**Mesosoma** (Fig. 16G, H, L). Mesosoma dorsoventrally convex. Distal 1/3 of mesoscutum with lateral margin slightly dented, punctation distinct throughout, and interspaces smooth. Scutellum triangular, apex sloped and fused with BS, in profile flat and on same plane as mesoscutum, scutellar punctation distinct peripherally, absent centrally, phragma of the scutellum partially exposed; BS only very partially overlapping the MPM; ATS demilune with a little, complete parallel carinae; dorsal ATS groove with carinae only proximally. Transscutal articulation with small and heterogeneous foveae; area just behind transscutal articulation with a smooth and shiny sloped transverse strip. Metanotum with BM wider than PFM (clearly differentiated); MPM circular without median longitudinal carina; AFM without setiferous lobes and not as well delineated as PFM; PFM thick and smooth; ATM proximally with semircular/undulate carina and distally smooth. Propodeum without median longitudinal carina, proximal half curved with fine sculpture and distal half rugose and with a shallow dent at each side of nucha; distal edge of propodeum with a flange at each side and without stubs; propodeal spiracle without distal carina; nucha without distinct short radiating carinae. Pronotum virtually without trace of dorsal furrow, dorsally with a well-defined smooth band; central area of pronotum and dorsal furrow smooth, but ventral furrow with short parallel carinae. Propleuron finely sculptured only ventrally and dorsally without a carina. Metasternum flat or nearly so. Contour of mesopleuron convex; precoxal groove smooth, shiny and shallow, but visible; epicnemial ridge convex, teardrop-shaped.

**Legs** (Fig. 16A, E). Ventral margin of fore telotarsus entire without seta, proximally narrow and distally wide, and longer than fourth tarsomere (0.10, 0.06). Hind coxa with punctation only on ventral surface, dorsal outer depression absent. Inner spur of hind tibia much longer than outer spur (0.24, 0.17); entire surface of hind tibia with dense strong spines clearly differentiated by color and length. Hind telotarsus as equal as fourth tarsomere (0.12, 0.11).

**Wings** (Fig. 15N, O). Fore wing with r vein straight; 2RS vein slightly convex to convex; r and 2RS veins forming an angle at their junction and outer side of junction forming a slight stub; 2M vein slightly curved/swollen; distally fore wing [where spectral veins are] with microtrichiae more densely concentrated than the rest of the wing; anal cell 1/3 proximally lacking microtrichiae; subbasal cell with microtrichiae virtually throughout; veins 2CUa and 2CUb completely spectral; vein 2 cu-a present as spectral vein, sometimes difficult to see; vein 2-1A present only proximally as spectral vein; tubular vein 1 cu-a curved, incomplete/broken, and not reaching the edge of 1-1A vein. Hind wing with vannal lobe very narrow subdistally and subproximally evenly convex, and setae evenly scattered in the margin.

**Metasoma** (Fig. 16J, K, M). Metasoma laterally compressed. Petiole on T1 finely sculptured only distally, evenly narrowing distally and apex truncate (length 0.35, maximum width 0.20, minimum width 0.09), petiole with scattered pubescence concentrated in the first distal third. Lateral grooves delimiting the median area on T2 distally losing definition (length median area 0.10, length T2 0.18), edges of median area polished, median area broader than long (length 0.10, maximum width 0.20, minimum width 0.08); T2 with scattered pubescence only distally. T3 longer than T2 (0.25, 0.18) and with scattered pubescence throughout. Pubescence on hypopygium dense.

**Cocoons.** Light (white, beige, or yellow) oval cocoons with silk fibers evenly smooth. Mass of tight elongate cylindrical cocoons adhered to the larval cuticle, but not to each other; cocoons fall easily off the living larva.

#### Comments.

Some females (e.g., 09-SRNP-11971) with additional obvious reddish brown tints in both dorsal and ventral furrows of the pronotum, the epicnemial ridge and the mesopleuron. In lateral view, T4 and beyond with a narrow medial brown area.

#### Male

(Fig. 17A–L). Similar in coloration to female; however, genitalia and the two last distal sterna partly brown. Dorsally, T4 and beyond with brown medial areas more extensive than in females, also sublateral and lateral areas yellow-brown. In other males, T4 and beyond the medial brown area is not well delimited or even inexistent.

#### Etymology.

Andrew (Andy) Robert Deans’ research has been focused largely on Evaniidae but includes the microgastrine genus *Alphomelon.* Currently, he is a professor and director of the Frost Entomological Museum at the Pennsylvania State University, PA, USA.

#### Distribution.

The parasitized caterpillars were collected in Costa Rica, ACG, Sector Pitilla (Estación Pitilla, Ingas, and Sendero Orosilito) and Sector Rincón Rain Forest (Sendero Anonás), during July-August 2003 and May 2011 at 405 m, 580 m, 675 m, and 900 m in rain forest.

#### Biology.

The lifestyle of this parasitoid species is gregarious.

#### Host.

*Enyoocypete* (L.), *Pachygonidiadrucei* Rothschild & Jordan and *Aleuroncarinata* (Walker) (Sphingidae: Macroglossinae) feeding on *Doliocarpusmultiflorus* (Dilleniaceae). Caterpillars were collected in second, third, fourth and fifth instar.

### Glyptapanteles andysuarezi

Taxon classificationAnimaliaHymenopteraBraconidae

Arias-Penna, sp. nov.

http://zoobank.org/26501038-2CA8-4FE4-9A7B-CCA16591A4FD

Figs 18 , 19

#### Female.

Body length 2.22 mm, antenna length 2.68 mm, fore wing length 2.53 mm.

#### Type material.

**Holotype**: ECUADOR • 1♀; EC-14335, YY-A044; Napo, Yanayacu Biological Station, Baeza Granja Integral, Plot 215; 1,896 m; cloud forest; -0.45, -77.883333; 05.v.2006; Rafael Granizo leg.; caterpillar collected in second instar; cocoons formed on 22.v.2006; (PUCE). **Paratypes**. • 8 (2♀, 2♂) (4♀ in pieces, 0♂); EC-14335, YY-A044; same data as for holotype; (PUCE).

#### Diagnosis.

**Figure 18.**
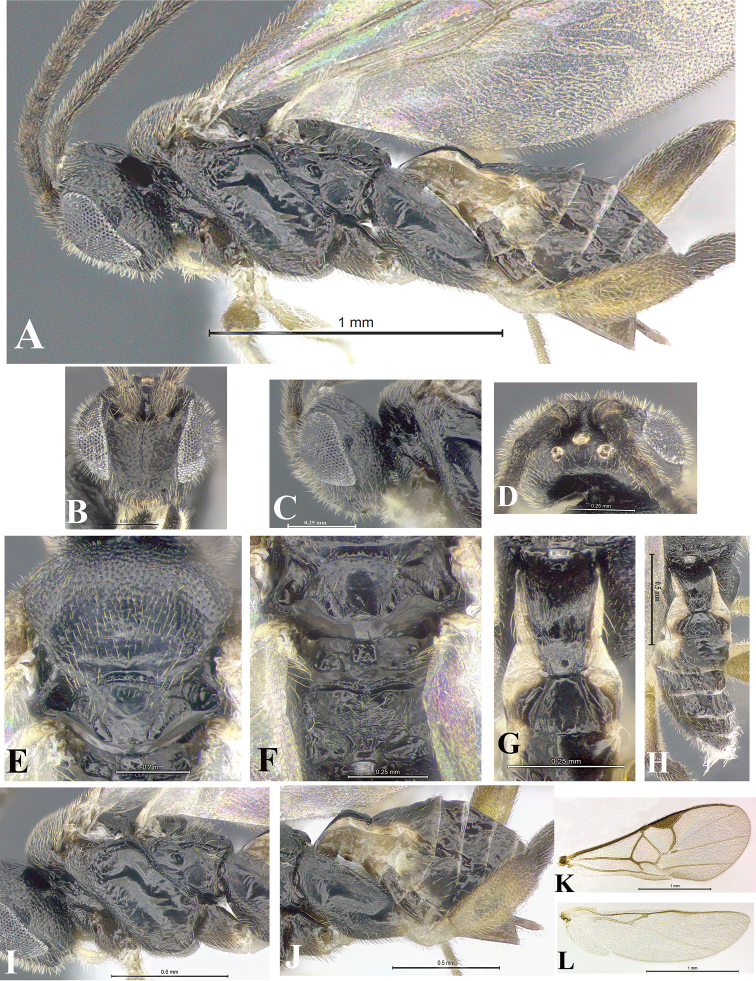
*Glyptapantelesandysuarezi* sp. nov. female EC-14335 YY-A044 **A** Habitus **B, D** Head **B** Frontal view **D** Dorsal view **C** Head, pronotum, propleuron, lateral view **E** Mesonotum, dorsal view **F** Scutellum, metanotum, propodeum, dorsal view **G**T1–2, dorsal view **H, J** Metasoma **H** Dorsal view **J** Lateral view **I** Mesosoma, lateral view **K, L** Wings **K** Fore **L** Hind.

**Figure 19.**
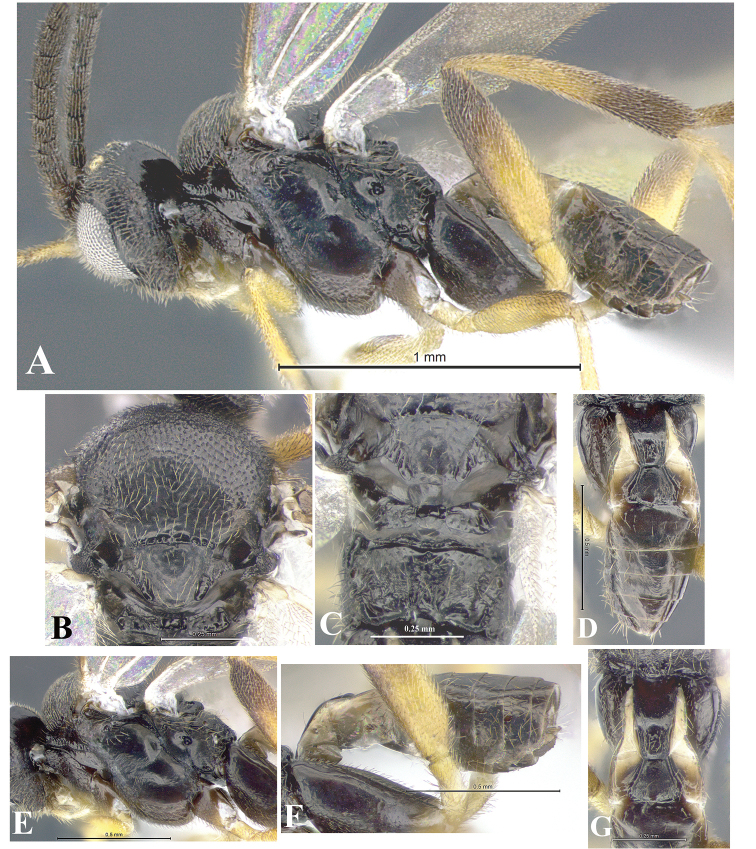
*Glyptapantelesandysuarezi* sp. nov. male EC-14335 YY-A044 **A** Habitus **B** Mesonotum, dorsal view **C** Scutellum, metanotum, propodeum, dorsal view **D, F** Metasoma **D** Dorsal view **F** Lateral view **E** Mesosoma, lateral view **G**T1–2, dorsal view.

Propleuron with fine rugae (Figs 18I, 19E), anteroventral contour of mesopleuron straight/angulate or nearly so (Figs 18A, I, 19A, E), mesoscutum punctation proximally distinct, but distally absent/dispersed (Figs 18E, 19B), T3 longer than T2 (Figs 18H, 19D), dorsal outer depression on hind coxa absent (Figs 18A, 19A, E), fore wing with r vein curved, outer side of junction of r and 2RS veins not forming a stub (Fig. 18K), inner margin of eyes diverging slightly at antennal sockets (Fig. 18B), petiole on T1 finely sculptured on 3/4 proximal (Figs 18G, H, 19D, G), propodeum without median longitudinal carina (Figs 18F, 19C), and lateral grooves delimiting the median area on T2 clearly defined and reaching the distal edge of T2 (Figs 18G, H, 19D, G).

#### Coloration

(Fig. 18A–L). General body coloration black except scape, pedicel, all antennal flagellomeres and tegulae dark brown; labrum, mandibles, maxillary and labial palps yellow-brown. Eyes gray/black and ocelli yellowish. Fore and middle legs yellow-brown except coxae light brown, claws black-brown; hind legs yellow-brown except black coxae with yellow-brown apex, 1/4 distal of femora, 1/4 distal of tibiae and tarsomeres brown, although proximal half of basitarsus yellow-brown. Petiole on T1 black with contours slightly darkened and sublateral areas yellow-brown; T2 with median area black, wide adjacent area brown forming a irregular shape, and lateral ends yellow-brown; T3 black, but proximal corners yellow-brown with diagonal inner edges; T4 and beyond completely black-brown; distally each tergum with a narrow whitish transparent band. In lateral view, T1–2 completely yellow-brown; T3 proximal half yellow-brown and distal half brown; T4 and beyond completely brown. S1–3 yellow, but medial brown; S4 and beyond completely brown.

#### Description.

**Head** (Fig. 18B–D). Head rounded with pubescence long and dense. Proximal three antennal flagellomeres longer than wide (0.17:0.06, 20.18:0.06, 0.20:0.06), distal antennal flagellomere longer than penultimate (0.12:0.03, 0.10:0.03), antenna longer than body (2.68, 2.22); antennal scrobes-frons shallow. Face with dense fine punctations, interspaces with microsculpture, face with depression only laterally and longitudinal median carina present. Frons punctate. Temple wide, punctate and interspaces wavy. Inner margin of eyes diverging slightly at antennal sockets; in lateral view, eye anteriorly convex and posteriorly straight. POL shorter than OOL (0.10, 0.12). Malar suture present. Median area between lateral ocelli without depression. Vertex laterally rounded and dorsally wide.

**Mesosoma** (Fig. 18E, F, I). Mesosoma dorsoventrally convex. Mesoscutum proximally convex and distally flat, punctation distinct proximally, but absent/dispersed distally, and interspaces with microsculpture. Scutellum short and broad, apex sloped and fused with BS, scutellar punctation distinct throughout, in profile scutellum slightly convex, but on same plane as mesoscutum, phragma of the scutellum partially exposed; BS not overlapping the MPM; ATS demilune with short stubs delineating the area; dorsal ATS groove smooth. Transscutal articulation with small and heterogeneous foveae, area just behind transscutal articulation smooth, shiny and nearly at the same level as mesoscutum (flat). Metanotum with BM wider than PFM (clearly differentiated); MPM circular without median longitudinal carina; AFM with a small lobe and not as well delineated as PFM; PFM thick and smooth; ATM proximally with semircular/undulate carina and distally smooth. Propodeum without median longitudinal carina, proximal half weakly curved with medium-sized sculpture and distal half with a shallow dent at each side of nucha; distal edge of propodeum with a flange at each side and without stubs; propodeal spiracle distally framed by faintly concave/wavy carina; nucha surrounded by very short radiating carinae. Pronotum with a distinct dorsal furrow, dorsally with a narrow band; central area of pronotum smooth, but both dorsal and ventral furrows with short parallel carinae. Propleuron with fine rugae and dorsally without a carina. Metasternum flat or nearly so. Contour of mesopleuron straight/angulate or nearly so; precoxal groove shallow, but visible and with faintly lineate sculpture; epicnemial ridge elongated more fusiform (tapering at both ends).

**Legs** (Fig. 18A). Ventral margin of fore telotarsus entire, but with a tiny curved seta, fore telotarsus almost same width throughout and longer than fourth tarsomere (0.12, 0.10). Hind coxa with punctation only on ventral surface, dorsal outer depression absent. Inner spur of hind tibia much longer than outer spur (0.20, 0.12), entire surface of hind tibia with dende strong spines clearly differentiated by color and length. Hind telotarsus as equal as fourth tarsomere (0.10, 0.11).

**Wings** (Fig. 18K, L). Fore wing with r vein curved; 2RS vein slightly convex to convex; r and 2RS veins forming a weak, even curve at their junction and outer side of junction not forming a stub; 2M vein slightly curved/swollen; distally fore wing [where spectral veins are] with microtrichiae almost homogeneously distributed as the rest of the wing; anal cell 1/3 proximally lacking microtrichiae; subbasal cell with microtrichiae virtually throughout; veins 2CUa and 2CUb completely spectral; vein 2 cu-a absent; vein 2-1A proximally tubular and distally spectral, although sometimes difficult to see; tubular vein 1 cu-a straight, incomplete/broken and not reaching the edge of 1-1A vein. Hind wing with vannal lobe narrow, subdistally and subproximally straightened, and setae evenly scattered in the margin.

**Metasoma** (Fig. 18G, H, J). Metasoma cylindrical. Petiole on T1 finely sculptured on 3/4 proximal, parallel-sided in proximal half and then narrowing (length 0.30, maximum width 0.14, minimum width 0.08) and pubescence on distal half. Lateral grooves delimiting the median area on T2 clearly defined and reaching the distal edge of T2 (length median area 0.15, length T2 0.15), edges of median area obscured by weak longitudinal stripes, median area broader than long (length 0.15, maximum width 0.18, minimum width 0.07); T2 with scattered pubescence throughout. T3 longer than T2 (0.20, 0.15) and with scattered pubescence throughout. Pubescence on hypopygium dense.

**Cocoons.** Unknown.

#### Comments.

The sculpture on the body is rough. The junction between the placodes on flagellomeres is darker than flagellomere itself. On the face, the median longitudinal carina extends from the scrobes to the clypeus. The median area on the propodeum has a transversal fine rugae. Some females from the same sample have both dorsal and ventral furrows of pronotum and distally the propleuron with reddish/brown tints.

#### Male

(Fig. 19A–G). Coloration similar to that of female.

#### Etymology.

Andrew (Andy) Suarez’s research is focused upon knowing the causes and consequences of biological invasions, mainly ants, and how polymorphism and complex societies contribute to their ecological success. Currently, he is head of Department of Animal Biology at UIUC, USA.

#### Distribution.

Parasitized caterpillar was collected in Ecuador, Napo, Yanayacu Biological Station (Baeza Granja Integral), during May 2006 at 1,896 m in cloud forest.

#### Biology.

The lifestyle of this parasitoid species is gregarious.

#### Host.

*Bertholdiapartita* Rawlins (Erebidae: Arctiinae) feeding on *Renealmiafragilis* (Zingiberaceae). Caterpillar was collected in second instar. In Ecuador, *B.partita* has been reported as host for three families of Hymenoptera: two groups of Eulophidae, one group of Braconidae and one group of Ichneumonidae; and one family of Diptera: Tachinidae.

### Glyptapanteles andywarreni

Taxon classificationAnimaliaHymenopteraBraconidae

Arias-Penna, sp. nov.

http://zoobank.org/321AEC30-BD0A-476A-8C4D-0DCA96A7D187

Figs 20 , 21

#### Female.

Body length 2.88 mm, antenna length 3.03 mm, fore wing length 3.13 mm.

#### Type material.

**Holotype**: ECUADOR • 1♀; EC-26009, YY-A051; Napo, Yanayacu Biological Station, Sendero Macuculoma, Plot 358; cloud forest; 2,091 m; -0.6, -77.883333; 07.ix.2007; Rafael Granizo leg.; caterpillar collected in fourth instar; loose groups of brown cocoons formed on 18.ix.2007; adult parasitoids emerged on 05.x.2007; (PUCE). **Paratypes.** • 17 (4♀, 1♂) (12♀, 0♂); EC-26009, YY-A051; same data as for holotype; (PUCE).

#### Diagnosis.

**Figure 20.**
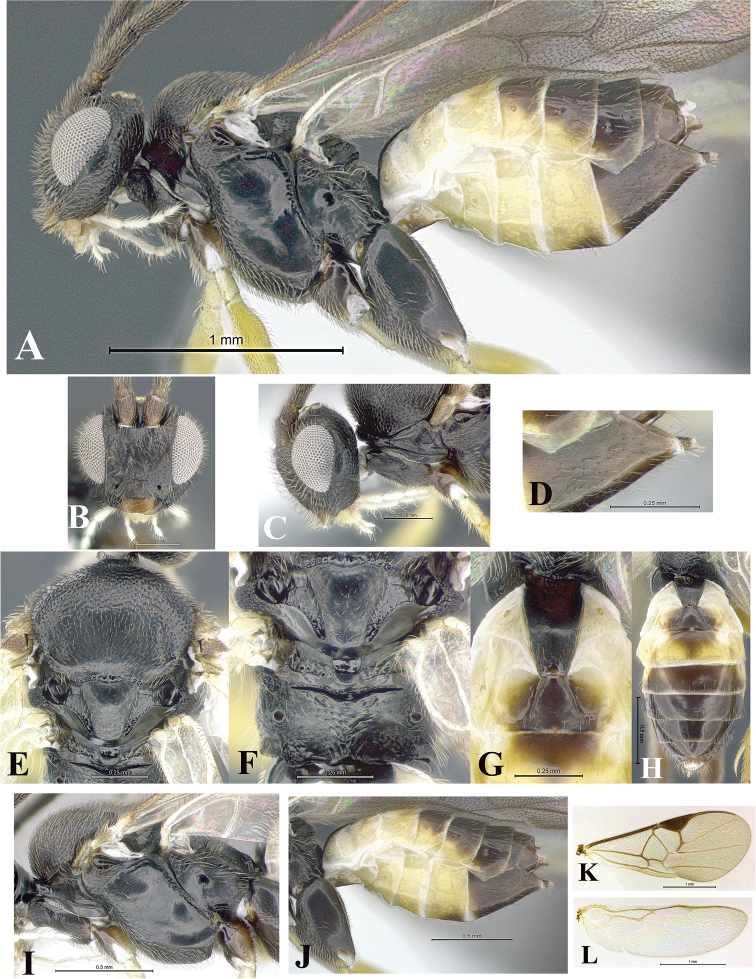
*Glyptapantelesandywarreni* sp. nov. female EC-26009 YY-A051 **A** Habitus **B** Head, frontal view **C** Head, pronotum, propleuron, lateral view **D** Genitalia: hypopygium, ovipositor, ovipositor sheaths, lateral view **E** Mesonotum, dorsal view **F** Scutellum, metanotum, propodeum, dorsal view **G**T1–2, dorsal view **H, J** Metasoma **H** Dorsal view **J** Lateral view **I** Mesosoma, lateral view **K, L** Wings **K** Fore **L** Hind.

**Figure 21.**
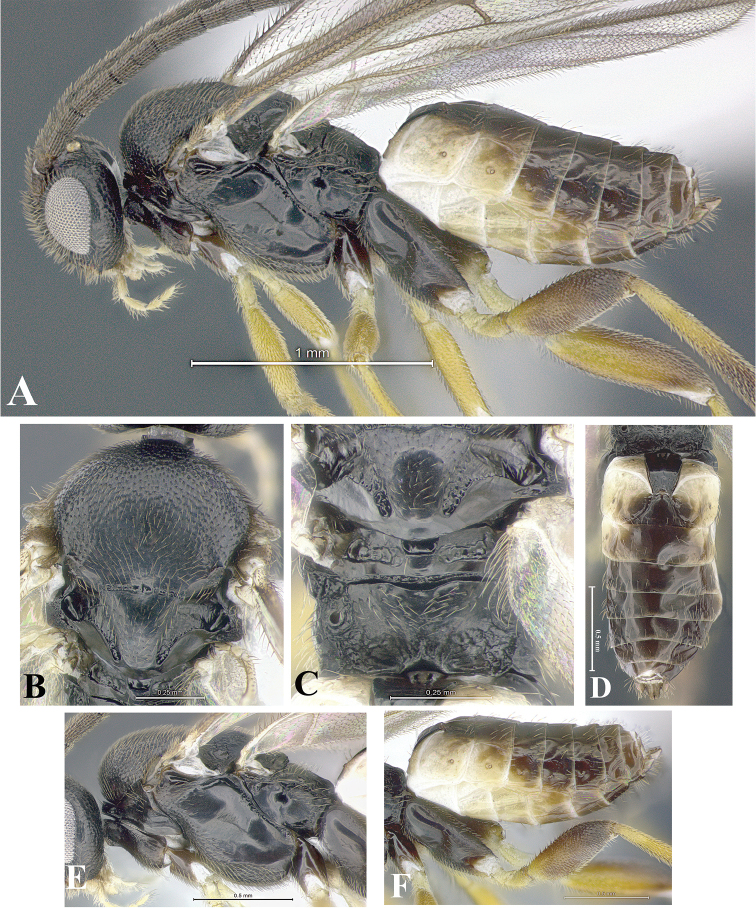
*Glyptapantelesandywarreni* sp. nov. male EC-26009 YY-A051 **A** Habitus **B** Mesonotum, dorsal view **C** Scutellum, metanotum, propodeum, dorsal view **D, F** Metasoma **D** Dorsal view **F** Lateral view **E** Mesosoma, lateral view.

Distal antennal flagellomere longer than penultimate, posterior ocelar line shorter than ocular ocelar line, mesoscutum punctation distinct throughout (Figs 20E, 21B), lateral grooves delimiting the median area on T2 clearly defined and reaching the distal edge of T2, edges of median area on T2 polished and followed by a deep groove (Figs 20G, 21D), axillary trough of metanotum proximally with semircular/undulate carina, distally smooth (Figs 20F, 21C), precoxal groove shallow, but visible (Figs 20I, 21E), anteroventral contour of mesopleuron straight/angulate or nearly so (Figs 20I, 21E), and fore wing with r vein curved, outer side of junction of r and 2RS veins forming a distinct stub (Fig. 20K).

#### Coloration

(Fig. 20A–L). General body coloration brown-black except clypeus and mandibles with yellow-brown coloration; glossa, maxillary and labial palps, and tegulae yellow; both dorsal and ventral furrows of pronotum, ventral edge of mesopleuron, epicnemial ridge, and lunules with reddish brown tints. Eyes silver and ocelli yellowish. Fore and middle legs yellow except coxae brown-black and claws brown; hind legs yellow except coxae black, 1/4 distal of femora, 3/4 proximal of tibia and tarsomeres brown. Petiole on T1 black, contours darkened and sublateral areas yellow; T2 with median area dark brown, contours darkened, wide adjacent area light yellow-brown, and lateral ends yellow; T3 yellow, but medially with an inverted triangle yellow-brown, proximal edges of inverted triangle area coincides with the width of median plus adjacent areas on T2; T4 yellow-brown/light brown with proximal corners yellow; T5 and beyond completely dark brown; distally each tergum with a narrow whitish/yellowish transparent band. In lateral view, T1–3 completely yellow; T4 and beyond dorsally brown and ventrally yellow, extent of brown area increasing from proximal to distal. S1–4 completely yellow; penultimate sternum yellow, ventrally with a brown spot; hypopygium completely brown.

#### Description.

**Head** (Fig. 20A–C). Head triangular with pubescence long and dense. Proximal three antennal flagellomeres longer than wide (0.25:0.07, 0.25:0.07, 0.25:0.07), distal antennal flagellomere longer than penultimate (0.14:0.07, 0.10:0.07), antenna longer than body (3.03, 2.88); antennal scrobes-frons shallow. Face convex with dense fine punctations, interspaces smooth, and longitudinal median carina present. Frons punctate. Temple wide, punctate and interspaces wavy. Inner margin of eyes diverging slightly at antennal sockets; in lateral view, eye anteriorly convex and posteriorly straight. POL shorter than OOL (0.10, 0.14). Malar suture present. Median area between lateral ocelli without depression. Vertex laterally rounded and dorsally wide.

**Mesosoma** (Fig. 20E, F, I). Mesosoma dorsoventrally convex. Mesoscutum with narrow grooves/dents taking the place of notauli, punctation distinct throughout, and interspaces wavy/lacunose. Scutellum triangular, apex sloped and fused with BS, scutellar punctation distinct throughout, in profile scutellum slightly convex, but on same plane as mesoscutum, phragma of the scutellum partially exposed; BS only very partially overlapping the MPM; ATS demilune with a little and incomplete parallel carinae only proximally; dorsal ATS groove smooth. Transscutal articulation with small and heterogeneous foveae, area just behind transscutal articulation with a smooth and shiny sloped transverse strip. Metanotum with BM wider than PFM (clearly differentiated); MPM circular without median longitudinal carina; AFM without setiferous lobes and not as well delineated as PFM; PFM thick and smooth; ATM proximally with semircular/undulate carina and distally smooth. Propodeum without median longitudinal carina, proximal half weakly curved with medium-sized sculpture and distal half with a shallow dent at each side of nucha; distal edge of propodeum with a flange at each side and without stubs; propodeal spiracle distally framed by a short concave carina; nucha surrounded by very short radiating carinae. Pronotum all smooth with a distinct dorsal furrow, dorsally with a well-defined smooth band. Propleuron with fine punctations throughout and dorsally with a carina. Metasternum flat or nearly so. Contour of mesopleuron straight/angulate or nearly so; precoxal groove smooth, shiny and shallow, but visible; epicnemial ridge convex, teardrop-shaped.

**Legs** (Fig. 20A). Ventral margin of fore telotarsus entire, but with a tiny curved seta, fore telotarsus almost same width throughout and longer than fourth tarsomere (0.15, 0.07). Hind coxa with punctation only on ventral surface, dorsal outer depression present, inner spur of hind tibia much longer than outer spur (0.25, 0.17), entire surface of hind tibia with dense strong spines clearly differentiated by color and length. Hind telotarsus longer than fourth tarsomere (0.16, 0.13).

**Wings** (Fig. 20K, L). Fore wing with r vein slightly curved; 2RS vein straight; r and 2RS veins forming a weak, even curve at their junction and outer side of junction forming a distinct stub; 2M vein straight or slightly curved/swollen; distally fore wing [where spectral veins are] with microtrichiae more densely concentrated than the rest of the wing; anal cell 1/3 proximally lacking microtrichiae; subbasal cell with microtrichiae virtually throughout; veins 2CUa and 2CUb completely spectral; vein 2 cu-a present as spectral vein, sometimes difficult to see; vein 2-1A proximally tubular and distally spectral, although sometimes difficult to see; tubular vein 1 cu-a curved, complete, but junction with 1-1A vein spectral. Hind wing with vannal lobe narrow, subdistally and subproximally straightened, and setae evenly scattered in the margin.

**Metasoma** (Fig. 20D, G, H, J). Metasoma laterally compressed. Petiole on T1 completely smooth and polished, with faint, satin-like sheen, petiole evenly narrowing distally (length 0.40, maximum width 0.20, minimum width 0.10) and with scattered pubescence concentrated in the first distal third. Lateral grooves delimiting the median area on T2 clearly defined and reaching the distal edge of T2 (length median area 0.18, length T2 0.18), edges of median area polished and lateral grooves deep, median area broader than long (length 0.18, maximum width 0.30, minimum width 0.08); T2 with scattered pubescence only distally. T3 longer than T2 (0.27, 0.18) and with scattered pubescence only distally. Pubescence on hypopygium dense.

**Cocoons.** Light brown oval cocoons with messy/disordered/fluffy silk fibers.

#### Comments.

Distally the pronotum at different level than mesopleuron and forming a deep hollow. The lateral margins of the median area on T2 are delicately curved (concave, Figs 20G, H, 21D) resembling the median area on T2 of *G.bourquini* (Blanchard) and *G.ecuadorius* (Whitfield et al. 2002a, Figs 2, 14).

#### Male

(Fig. 21A–F). Coloration similar to females but darkened. Dorsally, T3 brown with lateral ends yellow-brown rather than yellow and with a brown inverted-triangle area.

#### Etymology.

Andrew (Andy) D. Warren is an American lepidopterist, specialized on Hesperiidae. He is working as Senior Collections Manager at McGuire Center for Lepidoptera and Biodiversity, Florida Museum of Natural History, University of Florida, Gainesville, FL, USA.

#### Distribution.

Parasitized caterpillar was collected in Ecuador, Napo, Yanayacu Biological Station (Sendero Macuculoma), during September 2007 at 2,091 m in cloud forest.

#### Biology.

The lifestyle of this parasitoid species is gregarious.

#### Host.

Undetermined species of Noctuidae feeding on *Evodianthusfunifer* (Cyclanthaceae). Caterpillar was collected in fourth instar.

### Glyptapanteles ankitaguptae

Taxon classificationAnimaliaHymenopteraBraconidae

Arias-Penna, sp. nov.

http://zoobank.org/016A0E5D-2C58-4B02-B774-38EF9BFC9111

Fig. 22

#### Male.

Body length 2.99 mm, antenna length 4.04 mm, fore wing length 3.18 mm.

#### Type material.

**Holotype**: ECUADOR • 1♀; EC-12625, YY-A207; Napo, Yanayacu Biological Station, Ruben trail, Plot 186; cloud forest; 2,105 m, -0.6, -77.883333; 24.ii.2006; María de los Ángeles Simbaña leg.; caterpillar collected in first instar; cocoons formed on 14.iii.2006; adult parasitoids emerged on 09.iv.2006; (PUCE).

#### Diagnosis.

**Figure 22.**
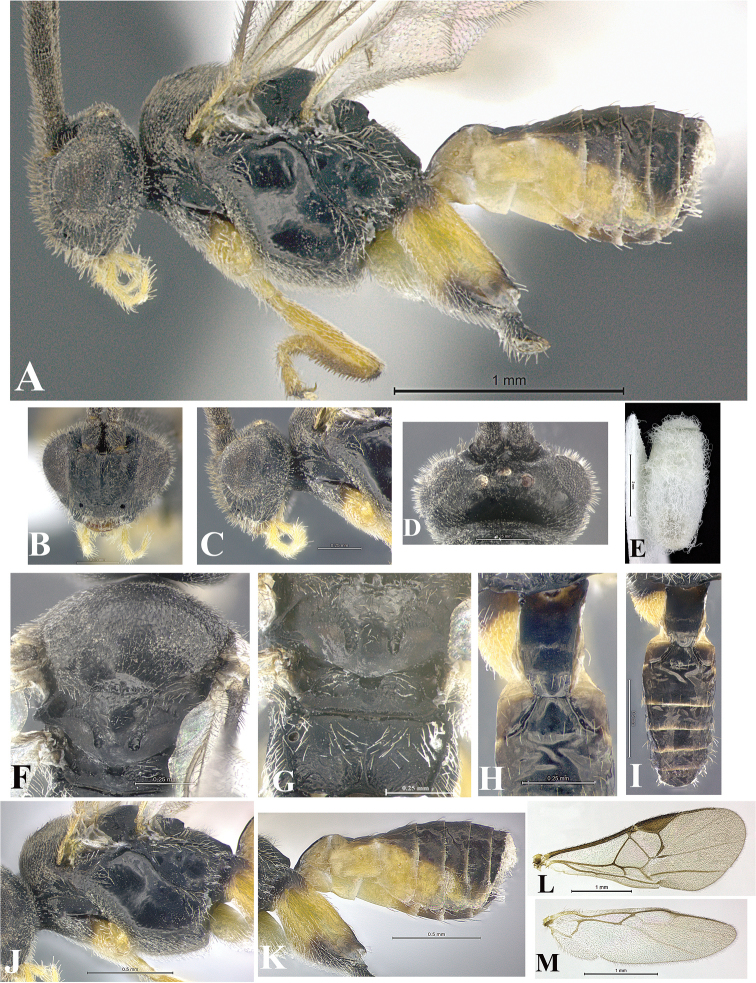
*Glyptapantelesankitaguptae* sp. nov. male EC-12625 YY-A207 **A** Habitus **B, D** Head **B** Frontal view **D** Dorsal view **C** Head, pronotum, propleuron, lateral view **E** Cocoon **F** Mesonotum, dorsal view **G** Scutellum, metanotum, propodeum, dorsal view **H**T1–2, dorsal view **I, K** Metasoma **I** Dorsal view **K** Lateral view **J** Mesosoma, lateral view **L, M** Wings **L** Fore **M** Hind.

Distal antennal flagellomere subequal in length with penultimate, posterior ocelar line broader than ocular ocelar line (Fig. 22D), mesoscutum punctation proximally distinct, but distally absent/dispersed (Fig. 22F), lateral grooves delimiting the median area on T2 clearly defined and reaching the distal edge of T2 (Fig. 22H, I), axillary trough of metanotum proximally with semircular/undulate carina, distally smooth (Fig. 22G), precoxal groove shallow, but visible (Fig. 22A, J), anteroventral contour of mesopleuron straight/angulate or nearly so (Fig. 22A, J), edges of median area on T2 polished and followed by a deep groove (Fig. 22H, I), and fore wing with r vein curved, outer side of junction of r and 2RS veins forming a slight stub (Fig. 22L).

#### Coloration

(Fig. 22A–M). General body coloration black except labrum, mandibles, and tegulae yellow-brown; glossa, maxillary and labial palps yellow. Eyes gray/black and ocelli whitish/reddish. Fore and middle legs yellow or light yellow-brown, except tibiae with a dorsal narrow brown strip from top to bottom, middle tarsomeres light brown, claws brown; hind legs yellow except a tiny brown area in both ends of coxae, tibiae with a dorsal narrow brown strip from top to bottom, and tarsi and claws brown. Petiole on T1 black and sublateral areas yellow; T2 with median area black-brown and lateral ends brown; T3 and beyond black-brown; distally each tergum with a narrow yellowish transparent band. In lateral view, T1–2 completely yellow; T3 and beyond yellow, but dorsally brown, the extent of brown area increasing from proximal to distal. S1–3 completely yellow; S4 yellow-brown; penultimate sternum and hypopygium completely brown.

#### Description.

**Head** (Fig. 22A–D). Head triangular with pubescence long and dense. Proximal three antennal flagellomeres longer than wide (0.28:0.10, 0.29:0.10, 0.31:0.10), distal antennal flagellomere subequal in length with penultimate (0.14:0.06, 0.14:0.06), antenna longer than body (4.04, 2.99); antennal scrobes-frons shallow. Face with lateral depression with scattered finely punctate, interspaces smooth, and longitudinal median carina present. Frons punctate. Temple wide, punctate and interspaces clearly smooth. Inner margin of eyes diverging slightly at antennal sockets; in lateral view, eye anteriorly convex and posteriorly straight. POL broader than OOL (0.14, 0.11). Malar suture present. Median area between lateral ocelli without depression. Vertex laterally rounded and dorsally wide.

**Mesosoma** (Fig. 22A, F–H, J). Mesosoma dorsoventrally convex. Distal 1/3 of mesoscutum with lateral margin slightly dented, punctation proximally distinct, but distally absent/dispersed, and interspaces wavy/lacunose. Scutellum triangular, apex sloped and fused with BS, scutellar punctation scattered throughout, in profile scutellum slightly convex, but on same plane as mesoscutum, phragma of the scutellum partially exposed; BS only very partially overlapping the MPM; ATS demilune with complete undulate/reticulate carinae; dorsal ATS groove with carinae only proximally. Transscutal articulation with small and heterogeneous foveae, area just behind transscutal articulation smooth, shiny and depressed centrally. Metanotum with BM wider than PFM (clearly differentiated); MPM semicircular without median longitudinal carina; AFM without setiferous lobes and not as well delineated as PFM; PFM thick and smooth; ATM proximally with semircular/undulate carina and distally smooth. Propodeum relatively polished without median longitudinal carina, proximal half straight or nearly so; distal edge of propodeum with a flange at each side and without stubs; propodeal spiracle distally framed by faintly concave/wavy carina; nucha surrounded by very short radiating carinae. Pronotum with a distinct dorsal furrow, dorsally with a well-defined smooth band; central area of pronotum and dorsal furrow smooth, but ventral furrow with short parallel carinae. Propleuron with fine punctations throughout and dorsally without a carina. Metasternum flat or nearly so. Contour of mesopleuron straight/angulate or nearly so; precoxal groove smooth, shiny, and shallow, but visible; epicnemial ridge elongated more fusiform (tapering at both ends).

**Legs.** Ventral margin of fore telotarsus entire without seta, fore telotarsus almost same width throughout and longer than fourth tarsomere (0.15, 0.08). Hind coxa with punctation only on ventral surface, dorsal outer depression absent, entire surface of hind tibia with dense strong spines clearly differentiated by color and length. Hind telotarsus longer than fourth tarsomere (0.14, 0.09).

**Wings** (Fig. 22L, M). Fore wing with r vein slightly curved; 2RS vein straight; r and 2RS veins forming a weak, even curve at their junction and outer side of junction forming a slight stub; 2M vein slightly curved/swollen; distally fore wing [where spectral veins are] with microtrichiae more densely concentrated than the rest of the wing; anal cell 1/3 proximally lacking microtrichiae; subbasal cell with microtrichiae virtually throughout; veins 2CUa and 2CUb completely spectral; vein 2 cu-a present as spectral vein, sometimes difficult to see; vein 2-1A proximally tubular and distally spectral, although sometimes difficult to see; tubular vein 1 cu-a curved, complete, but junction with 1-1A vein spectral. Hind wing with vannal lobe very narrow, subdistally and subproximally straightened, and setae evenly scattered in the margin.

**Metasoma** (Fig. 22A, H, I, K). Metasoma laterally compressed. Petiole on T1 completely smooth and polished, with faint, satin-like sheen, virtually parallel-sided over most of length, but narrowing over distal 1/3 (length 0.42, maximum width 0.20, minimum width 0.11), with scattered pubescence concentrated in the first distal third. Lateral grooves delimiting the median area on T2 clearly defined and reaching the distal edge of T2 (length median area 0.12, length T2 0.12), edges of median area polished, median area broader than long (length 0.12, maximum width 0.20, minimum width 0.08); T2 with scattered pubescence throughout. T3 longer than T2 (0.25, 0.12) and with scattered pubescence throughout.

**Cocoon** (Fig. 22E). White oval cocoon with silk fibers messy/disordered/fluffy.

#### Comments.

The inner spur in hind tibiae is missing. Length of the inner hind tibial spur is 0.24 mm, the outer spur is glued to pointed card, so it is difficult to see and measured.

#### Female.

Unknown

#### Etymology.

Ankita Gupta is an Indian entomologist who research is focused on parasitic Hymenoptera. She works at the Indian Council of Agricultural Research (ICAR), National Bureau of Agricultural Insect Resources, Bangalore, Karnataka, India.

#### Distribution.

Parasitized caterpillar was collected in Ecuador, Napo, Yanayacu Biological Station (Ruben trail), during February 2006 at 2,105 m in cloud forest.

#### Biology.

The lifestyle of this parasitoid species is solitary.

#### Host.

Undetermined species of Geometridae feeding on undetermined species of Pteridophyta. Caterpillar was collected in first instar.

### Glyptapanteles annettewalkerae

Taxon classificationAnimaliaHymenopteraBraconidae

Arias-Penna, sp. nov.

http://zoobank.org/8AE84FCD-A507-4796-B0F6-CB392FA31835

Figs 23 , 24

#### Female.

Body length 3.18 mm, antenna length 3.28 mm, fore wing length 3.13 mm.

#### Type material.

**Holotype**: COSTA RICA • 1♀; 10-SRNP-35889, DHJPAR0041868; Área de Conservación Guanacaste, Guanacaste, Sector Cacao, Sendero Nayo; cloud forest; 1,090 m; 10.92446, -85.46953; 24.viii.2010; Dunia Garcia leg.; caterpillar collected in third instar; cocoon formed on 01.ix.2010; adult parasitoids emerged on 09.ix.2010; (CNC). **Paratypes.** • 1 (0♀, 0♂) (0♀, 1♂); 10-SRNP-35885, DHJPAR0041861; same data as for holotype except: single white cocoon formed on 01.ix.2010; adult parasitoids emerged on 08.ix.2010; (CNC). • 1 (0♀, 1♂) (0♀, 0♂); 10-SRNP-35883, DHJPAR0041865; same data as for holotype except: single white cocoon (not white bud-like cocoon) adhered to the leaf substrate; adult parasitoids emerged on 10.ix.2010; (CNC). • 1 (0♀, 0♂) (0♀, 1♂); 10-SRNP-35888, DHJPAR0041866; same data as for holotype except: adult parasitoids emerged on 10.ix.2010; (CNC).

#### Other material.

**Reared material.** COSTA RICA: *Área de Conservación Guanacaste*, *Alajuela*, *Sector Rincón Rain Forest*, *Sendero Albergue Crater*: • 1 (0♀, 0) (1♀, 0♂); 10-SRNP-1390, DHJPAR0039020; 980 m; 10.84886, -85.3281; 14.iii.2010; Carolina Cano leg.; caterpillar collected in third instar; cocoon adhered to the leaf substrate and formed on 16.iii.2010; adult parasitoids emerged on 26.iii.2010.

#### Diagnosis.

**Figure 23.**
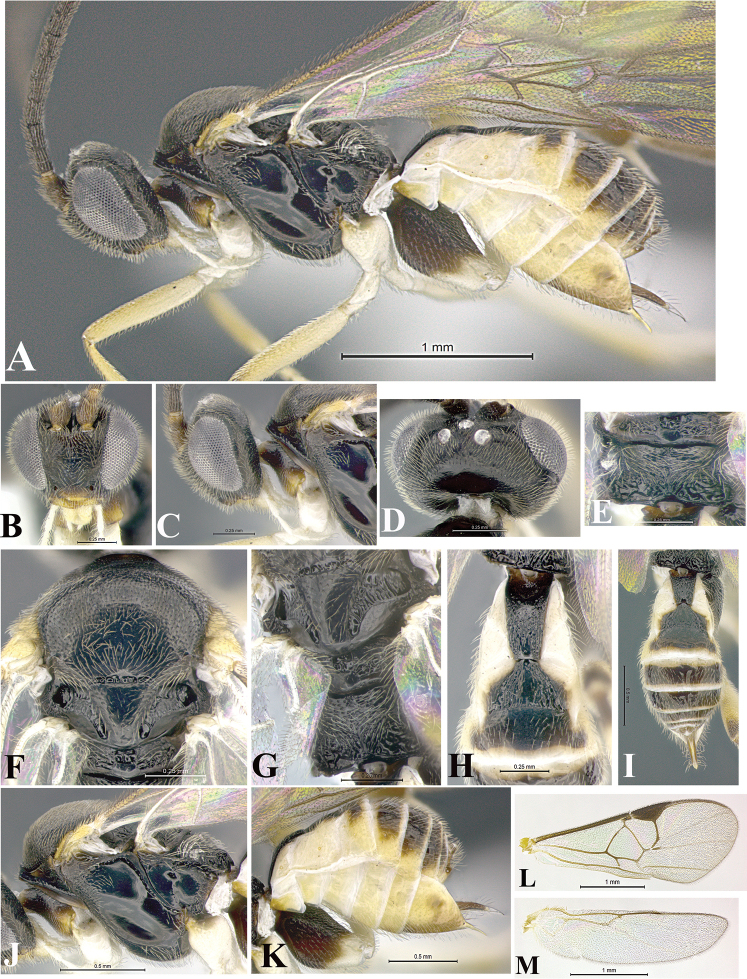
*Glyptapantelesannettewalkerae* sp. nov. female 10-SRNP-35889 DHJPAR0041868 **A** Habitus **B–D** Head **B** Frontal view **C** Lateral view **D** Dorsal view **E** Propodeum, dorsal view **F** Mesonotum, dorsal view **G** Scutellum, metanotum, propodeum, dorsolateral view **H**T1–3, dorsal view **I, K** Metasoma **I** Dorsal view **K** Lateral view **J** Mesosoma, lateral view **L, M** Wings **L** Fore **M** Hind.

**Figure 24.**
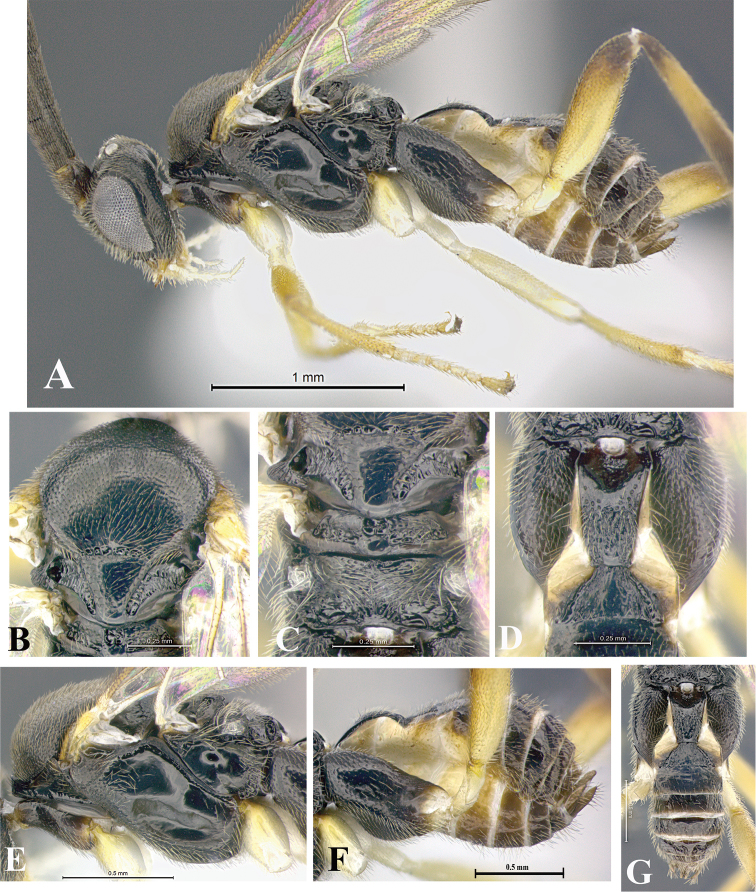
*Glyptapantelesannettewalkerae* sp. nov. male 10-SRNP-35883 DHJPAR0041865 **A** Habitus **B** Mesonotum, dorsal view **C** Scutellum, metanotum, propodeum, dorsolateral view **D**T1–2, dorsal view **E** Mesosoma, lateral view **F, G** metasoma **F** Lateral view **G** Dorsal view.

Propleuron with fine punctations throughout (Fig. 23C, J), axillary trough of metanotum with undulate carinae throughout (Figs 23G, 24C), medioposterior band of scutellum not overlapping the medioanterior pit of metanotum (Figs 23F, 24C), longitudinal median carina on face absent (Fig. 23B), inner margin of eyes diverging slightly at antennal sockets (Fig. 23B), scutellar punctation distinct throughout (Figs 23F, 24B), fore wing with tubular vein 1 cu-a incomplete/broken, not reaching the edge of 1-1A vein, r vein curved, outer side of junction of r and 2RS veins forming a slight stub (Fig. 23L), petiole on T1 evenly narrowing over its length (Figs 23H, 24D), surface of metasternum flat or nearly so, edges of median area on T2 obscured by weak longitudinal stripes (Figs 23H, I, 24D, G), and dorsal outer depression on hind coxa absent (Fig. 24A, F).

#### Coloration

(Fig. 23A–M). General body coloration black except apex of pedicel and clypeus yellow-brown; labrum, mandibles, and tegulae yellow; glossa, maxillary and labial palps ivory; distal 1/3 of propleuron and some spots on dorsal and ventral edge of pronotum yellow-brown. Eyes purple (in preserved specimen) and ocelli silver. Fore and middle legs light yellow or ivory although edges from femora to tarsomeres yellow, and claws brown; hind legs with trochanter and trochantellus ivory, coxae proximally black with apex ivory, femora ivory and with a tiny distal brown spot, tibiae yellow with both ends brown, and tarsomeres brown. Petiole on T1 black and sublateral areas pale yellow; T2 with median and adjacent areas black, and narrow lateral ends yellow; T3 with a medial brown area that coincides with the width of median and adjacent areas on T2; T4 and beyond brown; distally each tergum with a narrow yellowish transparent band. In lateral view, T1–3 completely light yellow or ivory; T4 and beyond yellow, but dorsally brown, extent of brown area increasing from proximal to distal. All sterna light yellow or ivory, although hypopygium medially yellow-brown; ovipositor sheaths brown.

#### Description.

**Head** (Fig. 23A–D). Head triangular with long and dense pubescence. Proximal three antennal flagellomeres longer than wide (0.29:0.08, 0.28:0.08, 0.26:0.08); antenna longer than body (3.28, 3.18); antennal scrobes-frons shallow. Face convex with scattered and finely punctate, interspaces smooth, and longitudinal median carina absent. Frons smooth. Temple wide, punctate and interspaces clearly smooth. Inner margin of eyes diverging slightly at antennal sockets; in lateral view, eye anteriorly convex and posteriorly straight. POL shorter than OOL (0.10, 0.14). Malar suture present. Median area between lateral ocelli without depression. Vertex laterally pointed or nearly so and dorsally wide.

**Mesosoma** (Fig. 23A, E–G, J). Mesosoma dorsoventrally convex. Mesoscutum proximally convex and distally flat, punctation distinct throughout, interspaces smooth. Scutellum triangular, apex sloped and fused with BS, in profile scutellum flat and on same plane as mesoscutum, scutellar punctation distinct throughout, phragma of the scutellum partially exposed; BS not overlapping the MPM; ATS demilune with a little, complete parallel carinae; dorsal ATS groove with carinae only proximally. Transscutal articulation with small and homogeneous foveae; area just behind transscutal articulation with same kind of sculpture as mesoscutum and with a sloped transverse strip. Metanotum with BM wider than PFM (clearly differentiated); MPM circular with some sculpture inside; AFM without setiferous lobes and not as well delineated as PFM; PFM thick and smooth; ATM with undulate carinae throughout. Propodeum without median longitudinal carina, proximal half curved and with rather coarse sculpture and distal half rugose with a shallow dent at each side of nucha; distal edge of propodeum with a flange at each side and short stubs; propodeal spiracle without distal carina; nucha surrounded by very short radiating carinae. Pronotum with a distinct dorsal furrow, dorsally with a well-defined smooth band; central area of pronotum and dorsal furrow smooth, but ventral furrow with short parallel carinae. Propleuron with fine punctations throughout and dorsally without a carina. Metasternum flat or nearly so. Contour of mesopleuron convex; precoxal groove smooth, shiny and shallow, but visible; epicnemial ridge convex, teardrop-shaped.

**Legs.** Ventral margin of fore telotarsus entire without seta; fore telotarsus proximally narrow and distally wide and longer than fourth tarsomere (0.14, 0.07). Hind coxa finely punctate throughout, and dorsal outer depression absent. Inner spur of hind tibia longer than outer spur (0.30, 0.25), entire surface of hind tibia with dense strong spines clearly differentiated by color and length. Hind telotarsus longer than fourth tarsomere (0.20, 0.15).

**Wings** (Fig. 23L, M). Fore wing with r vein slightly curved; 2RS vein straight; r and 2RS veins forming a weak, even curve at their junction and outer side of junction forming a slight stub; 2M vein slightly curved/swollen; distally fore wing [where spectral veins are] with microtrichiae more densely concentrated than the rest of the wing; anal cell 1/3 proximally lacking microtrichiae; subbasal cell with microtrichiae virtually throughout; veins 2CUa and 2CUb completely spectral; vein 2 cu-a present as spectral vein, sometimes difficult to see; vein 2-1A present only proximally as spectral vein; tubular vein 1 cu-a straight and incomplete/broken, not reaching the edge of 1-1A vein. Hind wing with vannal lobe very narrow, subdistally evenly convex, subproximally evenly convex, and setae evenly scattered in the margin.

**Metasoma** (Fig. 23A, H, I, K). Metasoma cylindrical. Petiole on T1 with rugae all over except antero-median depression, petiole evenly narrowing distally (length 0.45, maximum width 0.20, minimum width 0.14) with apex truncate, and with scattered pubescence concentrated in the first distal third. Lateral grooves delimiting the median area on T2 clearly defined and reaching the distal edge of T2 (length median area 0.21, length T2 0.21); edges of median area obscured by weak longitudinal stripes, median area broader than long (length 0.21, maximum width 0.40, minimum width 0.10); T2 with scattered pubescence only distally. T3 longer than T2 (0.26, 0.21), T3 with scattered pubescence throughout. Pubescence on hypopygium dense.

**Cocoon.** White cocoon with silk fibers messy/disordered/fluffy. Single cocoon adhered to the leaf substrate.

#### Comments.

The antenna is broken, only with 11 flagellomeres.

#### Male

(Fig. 24A–G). The body coloration is darker than in female: light yellow-brown rather than light yellow/ivory. The sterna are completely yellow-brown with arthrodial membranes yellow; the hind coxa is black with apex yellow. The lateral margins of the median area on T2 are slightly curved (convex, Fig. 24D, G).

#### Etymology.

Named after Annette K. Walker, now in New Zealand but previously in London specializing in Microgastrinae as part of the Commonwealth Institute of Entomology.

#### Distribution.

Parasitized caterpillars were collected in Costa Rica, ACG, Sector Cacao (Sendero Nayo) and Sector Rincón Rain Forest (Sendero Albergue Crater), during March and August 2010 at 980 m and 1,090 m in cloud forests.

#### Biology.

The lifestyle of this parasitoid species is solitary.

#### Host.

*Sylleptenitidalis* Dognin (Crambidae: Spilomelinae) feeding on *Malvaviscusarboreus* (Malvaceae) and *Trichaeapilicornis* Herrich-Schäffer (Crambidae: Spilomelinae) feeding on *Psychotriapanamensis* (Rubiaceae). Caterpillars were collected in third instar.

### Glyptapanteles barneyburksi

Taxon classificationAnimaliaHymenopteraBraconidae

Arias-Penna, sp. nov.

http://zoobank.org/898C7DEF-C709-4A70-9EDC-0A7307A93509

Figs 25 , 26

#### Female.

Body length 2.37 mm, antenna length 2.73 mm, fore wing length 2.37 mm.

#### Type material.

**Holotype**: COSTA RICA • 1♀; 95-SRNP-10048, DHJPAR0000089; Área de Conservación Guanacaste, Guanacaste, Sector Santa Rosa, Bosque Humedo; dry forest; 290 m; 10.85145, -85.60801; 16.x.1995; gusaneros leg.; caterpillar collected in fifth instar; white cocoons separate and individually adhered to back of larval cuticle, formed on 16.x.1995; adult parasitoids emerged on 24.x.1995; (CNC). **Paratypes.** • 16 (4♀, 3♂) (8♀, 1♂); 95-SRNP-10048, DHJPAR0000089; same data as for holotype; (CNC).

#### Other material.

**Reared material.** COSTA RICA: *Área de Conservación Guanacaste*, *Guanacaste*, *Sector Santa Rosa*, *Bosque Humedo*: • 6 (2♀, 0♂) (4♀ + 0♂); 82-SRNP-854.1, DHJPAR0000051; dry forest; 290 m; 10.85145, -85.60801; 12.xi.1982; gusaneros leg.; caterpillar collected in fifth instar; white cocoons adhered to the larval cuticle and formed on 18.xi.1982; adult parasitoids emerged on 24.xi.1982.

*Área de Conservación Guanacaste*, *Guanacaste*, *Sector Pitilla*, *Casa Roberto*: • 20 (3♀, 3♂) (14♀ + 0♂); 04-SRNP-34906, DHJPAR0001509; rain forest; 520 m; 11.01095, -85.42094; 30.viii.2004; Calixto Moraga leg.; caterpillar collected in third instar; cocoons adhered to the larval cuticle and formed on 16.ix.2004; adult parasitoids emerged on 25.ix.2004.

#### Diagnosis.

**Figure 25.**
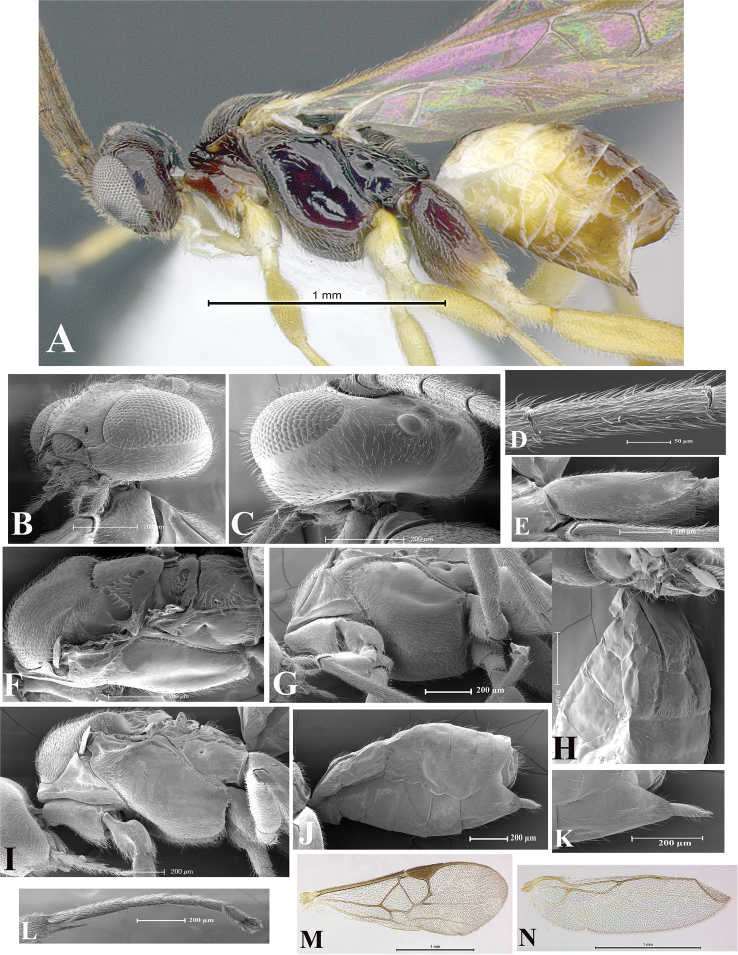
*Glyptapantelesbarneyburksi* sp. nov. female 95-SRNP-10048 DHJPAR0000089 **A** Habitus **B, C** Head **B** Ventrolateral view **C** Dorsal view **D** Flagellomeres **E** Hind coxa, lateral view **F, G, I** Mesosoma **F** Dorsolateral view **G** Ventrolateral view **I** Lateral view **H**T1–3, dorsolateral view **J** Metasoma, lateral view **K** Genitalia: hypopygium, ovipositor, ovipositor sheaths, lateral view **L** Fore tarsus **M, N** Wings **M** Fore **N** Hind.

**Figure 26.**
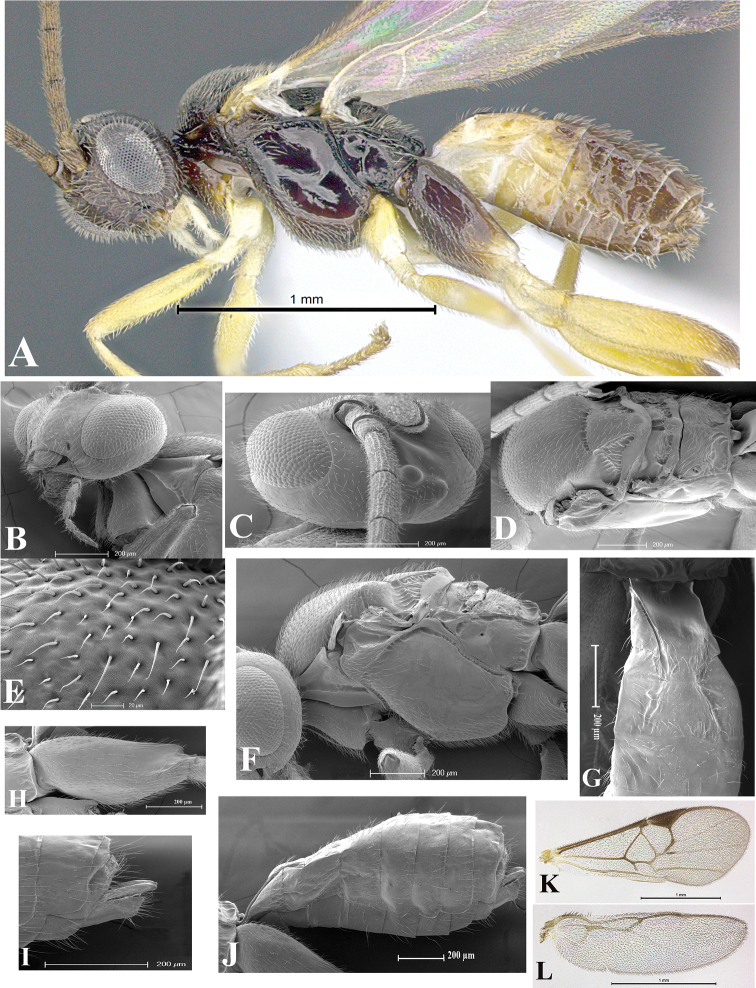
*Glyptapantelesbarneyburksi* sp. nov. male 95-SRNP-10048 DHJPAR0000089 **A** Habitus **B, C** Head **B** Ventrolateral view **C** Dorsal view **D, F** Mesosoma **D** Dorsolateral view **F** Lateral view **E** Microsculptures mesoscutum **G**T1–3, dorsal view **H** Hind coxa, lateral view **I** Genitalia: parameres, lateral view **J** Metasoma, lateral view **K, L** Wings **K** Fore **L** Hind.

Propleuron with fine punctations only ventrally (Figs 25A, 26B, F), axillary trough of metanotum proximally with undulate carina, distally smooth (Figs 25F, 26D), medioposterior band of scutellum only very partially overlapping the medioanterior pit of metanotum (Figs 25F, 26D), longitudinal median carina on face present (Figs 25B, 26A), inner margin of eyes straight throughout, scutellar punctation distinct throughout (Figs 25F, 26D), fore wing with tubular vein 1 cu-a incomplete/broken, not reaching the edge of 1-1A vein, r vein slightly curved, outer side of junction of r and 2RS veins forming a slight stub (Figs 25M, 26K), petiole on T1 evenly narrowing over its length (Figs 25H, 26G), surface of metasternum flat or nearly so (Fig. 25G), edges of median area on T2 obscured by weak longitudinal stripes (Figs 25H, 26G), and dorsal outer depression on hind coxa absent (Figs 25E, 26H).

#### Coloration

(Fig. 25A). General body coloration brown-black except scape, labrum, and mandibles yellow-brown; glossa, maxillary and labial palps, and tegulae yellow; propleuron, ventral furrow of pronotum and small area on dorsal furrow of pronotum brown-yellow. Eyes and ocelli silver. Fore and middle legs yellow with claws brown; hind legs yellow except coxa brown with yellow apex, distally femora brown, tibiae with apex brown, and tarsomeres brown. Petiole on T1 brown with sublateral areas yellow; T2 with median and adjacent areas brown, and lateral ends yellow; T3 and beyond completely brown; distally each tergum with a narrow yellowish transparent band. In lateral view, T1–2 completely yellow; T3 and beyond yellow-brown, but dorsally brown, extent of brown area increasing from proximal to distal. S1–3 completely yellow; S4–5 yellow-brown, medially brown; hypopygium completely brown.

#### Description.

**Head** (Fig. 25A–D). Head rhomboid with pubescence long and dense. Proximal three antennal flagellomeres longer than wide (0.21:0.07, 0.21:0.07, 0.21:0.07), distal antennal flagellomere longer than penultimate (0.12:0.06, 0.09:0.06), antenna longer than body (2.73, 2.37), antennal scrobes-frons shallow. Face finely punctate-lacunose, interspaces smooth, face with depression only laterally and longitudinal median carina present. Frons smooth. Temple wide, punctate and interspaces clearly smooth. Inner margin of eyes straight throughout; in lateral view, eye anteriorly convex and posteriorly straight. POL shorter than OOL (0.09, 0.14). Malar suture present. Median area between lateral ocelli without depression. Vertex laterally rounded and dorsally wide.

**Mesosoma** (Fig. 25A, F, G, I). Mesosoma dorsoventrally convex. Mesoscutum proximally convex and distally flat, with punctation distinct throughout, and interspaces wavy/lacunose. Scutellum triangular, apex sloped and fused with BS, in profile scutellum flat and on same plane as mesoscutum, scutellar punctation distinct throughout, phragma of the scutellum partially exposed; BS only very partially overlapping the MPM; ATS demilune with a little, complete parallel carinae; dorsal ATS groove with carinae only proximally. Transscutal articulation with small and heterogeneous foveae; area just behind transscutal articulation smooth and shiny with a sloped transverse strip. Metanotum with BM wider than PFM (clearly differentiated); MPM circular and bisected by a median longitudinal carina; AFM without setiferous lobes and not as well delineated as PFM; PFM thick and smooth; ATM proximally with semircular/undulate carina and distally smooth. Propodeum without median longitudinal carina, proximal half weakly curved with fine sculpture and distal half rugose and with a shallow dent at each side of nucha; distal edge of propodeum with a flange at each side and without stubs; propodeal spiracle distally framed by faintly concave/wavy carina; nucha surrounded by very short radiating carinae. Pronotum with a distinct dorsal furrow, dorsally with a well-defined smooth band; central area of pronotum smooth, but both dorsal and ventral furrows with short parallel carinae. Propleuron finely sculptured only ventrally and dorsally without a carina. Metasternum flat or nearly so. Contour of mesopleuron convex; precoxal groove smooth, shiny and shallow, but visible; epicnemial ridge convex, teardrop-shaped.

**Legs** (Fig. 25A, E, L). Ventral margin of fore telotarsus slightly excavated and with a tiny curved seta, fore telotarsus proximally narrow and distally wide, and longer than fourth tarsomere (0.14, 0.06). Hind coxa with dorsal half sparsely punctate, ventral half densely punctate, and dorsal outer depression absent. Inner spur of hind tibia longer than outer spur (0.20, 0.17), entire surface hind tibia with dense strong spines clearly differentiated by color and length. Hind telotarsus longer than fourth tarsomere (0.15, 0.11).

**Wings** (Fig. 25M, N). Fore wing with r vein slightly curved; 2RS vein straight; r and 2RS veins forming an angle at their junction and outer side of junction forming a slight stub; 2M vein slightly curved/swollen; distally fore wing [where spectral veins are] with microtrichiae more densely concentrated than the rest of the wing; anal cell 1/3 proximally lacking microtrichiae; subbasal cell with microtrichiae virtually throughout; veins 2CUa and 2CUb completely spectral; vein 2 cu-a absent; vein 2-1A proximally tubular and distally spectral, although sometimes difficult to see; tubular vein 1 cu-a curved, incomplete/broken, and not reaching the edge of 1-1A vein. Hind wing with vannal lobe narrow, subdistally evenly convex, subproximally straightened, and setae evenly scattered in the margin.

**Metasoma** (Fig. 25A, H–J, K). Metasoma laterally compressed. Petiole on T1 finely sculptured on distal half, evenly narrowing distally and apex truncate (length 0.32, maximum width 0.18, minimum width 0.14), petiole with scattered pubescence and concentrated in the first distal third. Lateral grooves delimiting the median area on T2 clearly defined and reaching the distal edge of T2 (length median area 0.18, length T2 0.18), edges of median area obscured by weak longitudinal stripes, median area broader than long (length 0.18, maximum width 0.20, minimum width 0.10); T2 with scarce pubescence throughout. T3 longer than T2 (0.20, 0.18), T3 with scattered pubescence throughout. Pubescence on hypopygium dense.

**Cocoons.** White oval cocoons with silk fibers messy/disordered/fluffy. Cocoons separate and individually adhered to back of larval cuticle.

#### Male

(Fig. 26A–L). Males tends to be thinner and darker than females.

#### Etymology.

Barnard (Barney) D. Burks (November 12, 1909-December 15, 1990) was a well-known American entomologist (especially active with Chalcidoidea) who studied (B.A., M.A., and Ph.D.) at UIUC, IL, USA.

#### Distribution.

The parasitized caterpillars were collected in Costa Rica, ACG; Sector Pitilla (Casa Roberto) and Sector Santa Rosa (Bosque Humedo), during November 1982, October 1995, and August 2004 at 290 m and 520 m in dry forest and rain forest.

#### Biology.

The lifestyle of this parasitoid species is gregarious.

#### Host.

*Smicropusintercepta* Walker (Geometridae, Sterrhinae) feeding on *Mascagniasinemariensis* and *Tetrapterysdiscolor* (Malpighiaceae). Caterpillars were collected in third and fifth instars.

### Glyptapanteles betogarciai

Taxon classificationAnimaliaHymenopteraBraconidae

Arias-Penna, sp. nov.

http://zoobank.org/13C39CE5-70FC-4CA2-96A7-63E047BC7B14

Figs 27 , 28

#### Female.

Body length 2.63 mm, antenna length 3.53 mm, fore wing length 3.23 mm.

#### Type material.

**Holotype**: ECUADOR • 1♀; EC-34000, YY-A235; Napo, Yanayacu Biological Station, Yanayacu Road; cloud forest; 2,100 m; -0.566667, -77.866667; 25.vi.2008; Earthwatch volunteers leg.; caterpillar collected in third instar; white bud-like cocoons formed on 13.vii.2008; adult parasitoid emerged on 14.viii.2008; (PUCE). **Paratype.** 1 (0♀, 1♂) (0♀, 0♂); EC-43164, YY-A175; same data as for holotype except: 05.xi.2009; CAPEA leg.; caterpillar collected in third instar; cocoons formed on 17.xi.2009; adult parasitoid emerged on 21.xii.2009; (PUCE).

#### Diagnosis.

**Figure 27.**
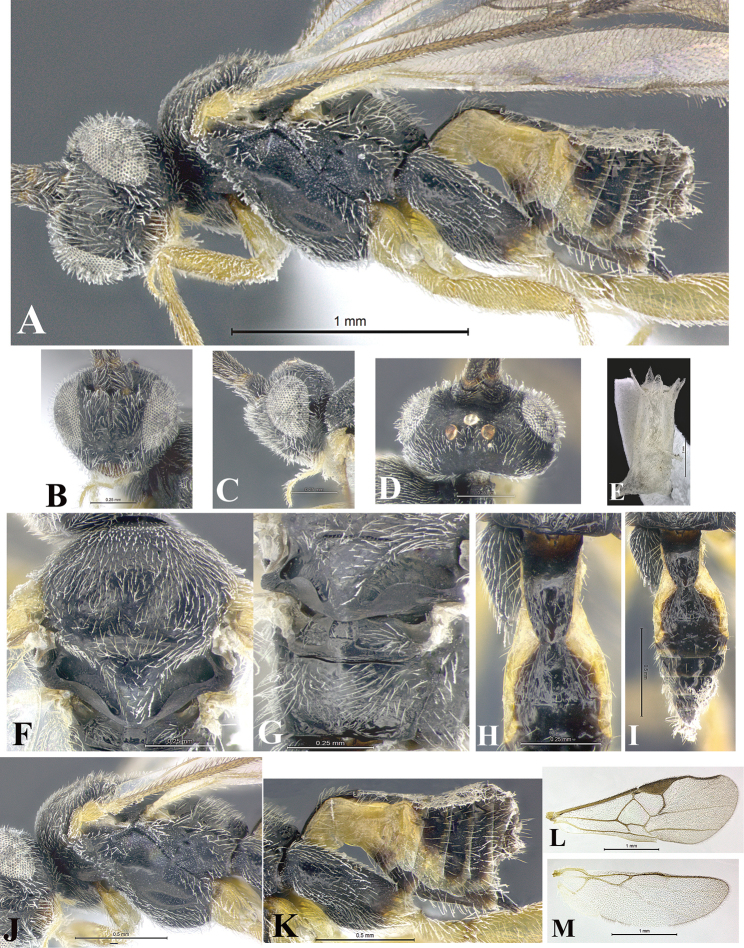
*Glyptapantelesbetogarciai* sp. nov. female EC-34000 YY-A235 **A** Habitus **B, D** Head **B** Frontal view **D** Dorsal view **C** Head, pronotum, propleuron, lateral view **E** Cocoon **F** Mesonotum, dorsal view **G** Scutellum, metanotum, propodeum, dorsal view **H**T1–2, dorsal view **I, K** Metasoma **I** Dorsal view **K** Lateral view **J** Mesosoma, lateral view **L, M** Wings **L** Fore **M** Hind.

**Figure 28.**
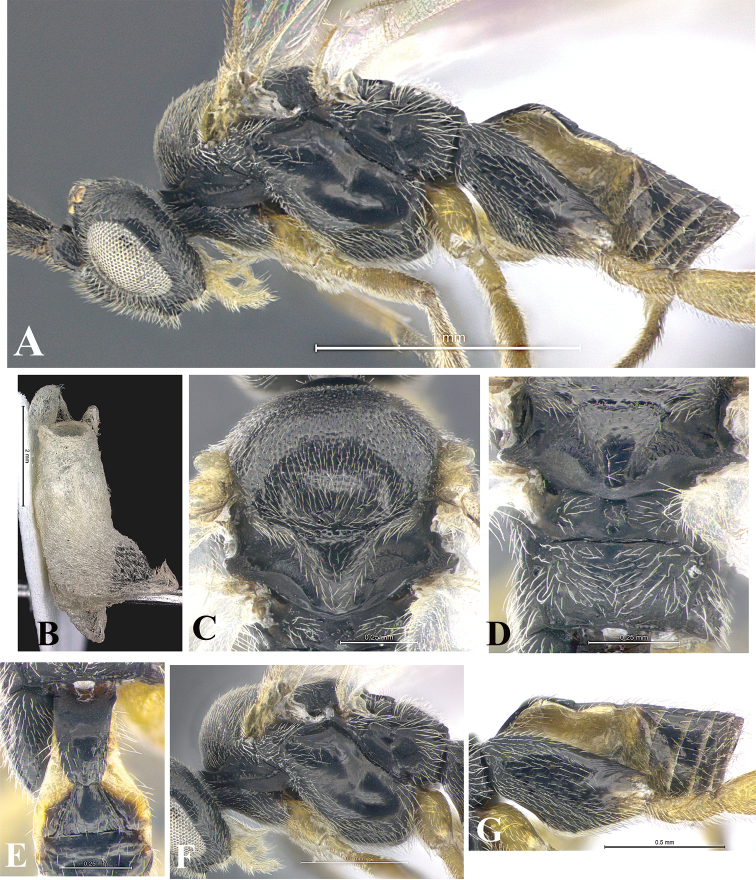
*Glyptapantelesbetogarciai* sp. nov. male EC-43164 YY-A175 **A** Habitus **B** Cocoon **C** Mesonotum, dorsal view **D** Scutellum, metanotum, propodeum, dorsal view **E**T1–2, dorsal view **F** Mesosoma, lateral view **G** Metasoma, lateral view.

Propleuron with fine punctations throughout (Figs 27J, 28F), anteroventral contour of mesopleuron convex (Figs 27A, 28A, F), mesoscutum proximally with distinct punctation distally with a polished area (Figs 27F, 28C), T3 as long as T2 (Fig. 27I), dorsal outer depression on hind coxa absent (Figs 27A, K, 28A, G), fore wing with r vein curved, outer side of junction of r and 2RS veins not forming a stub (Fig. 27L), inner margin of eyes diverging slightly at antennal sockets (Fig. 27B), petiole on T1 finely sculptured on distal half (Figs 27H, 28E), propodeum without median longitudinal carina (Figs 27G, 28D), and lateral grooves delimiting the median area on T2 clearly defined and reaching the distal edge of T2 (Figs 27H, I, 28E).

#### Coloration

(Fig. 27A–M). General body coloration black except labrum, mandibles and distally pedicel with a ring reddish brown tints; glossa, maxillary and labial palps, and tegulae yellow. Eyes silver, but black mottled and ocelli whitish/reddish. Fore and middle legs yellow although tibiae and tarsomeres dark yellow/light or yellow-brown, claws dark brown; hind legs yellow except coxae black with apex yellow, tiny brown dot in apex of femora, apex of tibiae brown, and tarsomeres brown. Petiole on T1 black and sublateral areas yellow; T2 with media and wide adjacent areas black-brown, and lateral ends yellow; T3 mostly black-brown with lateral ends yellow; T4 and beyond completely black-brown; distally each tergum with a narrow whitish transparent band. In lateral view, T1–2 completely yellow; T3–4 yellow, but dorsally brown, brown area larger on T4 than T3; T5 and beyond completely brown. S1–3 completely yellow; S4–5 brown, distally with a wide yellow-brown band; hypopygium completely brown.

#### Description.

**Head** (Fig. 27A–D). Head triangular with pubescence long and dense. Proximal three antennal flagellomeres longer than wide (0.25:0.08, 0.30:0.08, 0.27:0.08), distal antennal flagellomere longer than penultimate (0.15:0.06, 0.10:0.06), antenna longer than body (3.53, 2.63); antennal scrobes-frons shallow. Face flat or nearly so, with dense fine punctations, interspaces smooth, and longitudinal median carina present. Frons smooth. Temple wide punctate and interspaces clearly smooth. Inner margin of eyes diverging slightly at antennal sockets; in lateral view, eye anteriorly convex and posteriorly straight. POL shorter than OOL (0.10, 0.13). Malar suture present. Median area between lateral ocelli without depression. Vertex laterally rounded and dorsally wide.

**Mesosoma** (Fig. 27A, F, G, J). Mesosoma dorsoventrally convex. Mesoscutum with narrow grooves/dents taking the place of notauli, punctation distinct proximally with polished area distally, and interspaces wavy/lacunose. Scutellum triangular, apex sloped and fused with BS, scutellar punctation scattered throughout, in profile scutellum flat and on same plane as mesoscutum, phragma of the scutellum partially exposed; BS only very partially overlapping the MPM; ATS demilune with a little and complete parallel carinae; dorsal ATS groove smooth. Transscutal articulation with small and heterogeneous foveae, area just behind transscutal articulation with a smooth, shiny and sloped transverse strip. Metanotum with BM wider than PFM (clearly differentiated); MPM semicircular without median longitudinal carina; AFM with a small lobe and not as well delineated as PFM; PFM thick and smooth; ATM proximally with semircular/undulate carina and distally smooth. Propodeum without median longitudinal carina, proximal half weakly curved with medium-sized sculpture and distal half with a shallow dent at each side of nucha; distal edge of propodeum with a flange at each side and without stubs; propodeal spiracle distally framed by faintly concave/wavy carina; nucha surrounded by very short radiating carinae. Pronotum with a distinct dorsal furrow, dorsally with a well-defined smooth band; central area of pronotum smooth, but both dorsal and ventral furrows with short parallel carinae. Propleuron with fine punctations throughout and dorsally without a carina. Metasternum flat or nearly so. Contour of mesopleuron convex; precoxal groove smooth, shiny and shallow, but visible; epicnemial ridge elongated more fusiform (tapering at both ends).

**Legs.** Ventral margin of fore telotarsus entire, but with a tiny curved seta, fore telotarsus almost same width throughout and longer than fourth tarsomere (0.14, 0.09). Hind coxa with dorsal half sparsely punctate, ventral half densely punctate, and dorsal outer depression absent. Inner spur of hind tibia longer than outer spur (0.27, 0.21), entire surface of hind tibia with dense strong spines clearly differentiated by color and length. Hind telotarsus and fourth tarsomere missing.

**Wings** (Fig. 27L, M). Fore wing with r vein slightly curved; 2RS vein straight; r and 2RS veins forming a weak, even curve at their junction and outer side of junction not forming a stub; 2M vein straight; distally fore wing [where spectral veins are] with microtrichiae more densely concentrated than the rest of the wing; anal cell 1/3 proximally lacking microtrichiae; subbasal cell with microtrichiae virtually throughout; veins 2CUa and 2CUb completely spectral; vein 2 cu-a present as spectral vein, sometimes difficult to see; vein 2-1A proximally tubular and distally spectral, although sometimes difficult to see; tubular vein 1 cu-a straight, incomplete/broken, and not reaching the edge of 1-1A vein. Hind wing with vannal lobe very narrow, subdistally and subproximally straightened, and setae evenly scattered in the margin.

**Metasoma** (Fig. 27A, H, I, K). Metasoma laterally compressed. Petiole on T1 finely sculptured on distal half, parallel-sided in proximal half, then narrowing (gradually or not), with scattered pubescence on distal half. Lateral grooves delimiting the median area on T2 clearly defined and reaching the distal edge of T2 (length median area 0.20, length T3 0.20), edges of median area obscured by weak longitudinal stripes, median area longer than broad (length 0.20, maximum width 0.18, minimum width 0.08); T2 with scarce pubescence throughout. T3 as long as T2 (0.20, 0.20) and with scattered pubescence throughout. Pubescence on hypopygium dense.

**Cocoon** (Fig. 27E). White bud-like cocoon with silk fibers evenly smooth.

#### Comments.

The body is completely covered with dense white pubescence. The mesosoma is more robust in the females than males.

#### Male

(Fig. 28A–G). Similar in coloration to female.

#### Etymology.

Humberto (Beto) García López is a Costa Rican research assistant who works at La Selva Biological Station, Puerto Viejo de Sarapiquí, Heredia, Costa Rica. Currently, he is involved in the project Orugas (Caterpillars) and the project Latex (focus in *Piper* plants).

#### Distribution.

Parasitized caterpillars were collected in Ecuador, Napo, Yanayacu Biological Station (Yanayacu Road), during June 2008 and November 2009 at 2,100 m in cloud forest.

#### Biology.

The lifestyle of this parasitoid species is solitary.

#### Host.

Undetermined species of Geometridae feeding on undetermined species of Pteridophyta. Caterpillars were collected in third instar.

### Glyptapanteles billbrowni

Taxon classificationAnimaliaHymenopteraBraconidae

Arias-Penna, sp. nov.

http://zoobank.org/92723F0C-47DF-4451-9057-41B891191D87

Figs 29 , 30

#### Female.

Body length 2.78 mm, antenna length 2.78 mm, fore wing length 2.53 mm.

#### Type material.

**Holotype**: COSTA RICA • 1♀; 06-SRNP-65722, DHJPAR0012678; Área de Conservación Guanacaste, Guanacaste, Sector Pitilla, Sendero Carica; rain forest; 660 m; 10.99284, -85.42936; 20.xii.2006; Manuel Rios leg.; caterpillar collected in fourth instar; single cocoons not adhered to each other, adhered to the larval cuticle and formed on 21.xii.2006; adult parasitoids emerged on 30.xii.2006; (CNC). **Paratypes.** • 185 (5♀, 5♂) (160♀, 15♂); 06-SRNP-65722, DHJPAR0012678; same data as for holotype; (CNC).

#### Other material.

**Reared material.** COSTA RICA: *Área de Conservación Guanacaste*, *Guanacaste*, *Sector Cacao*, *Estación Cacao*: • 44 (4♀, 3♂) (28♀, 9♂); 99-SRNP-669, DHJPAR0001484; cloud forest; 1,150 m; 10.92691, -85.46822; 21.v.1999; Harry Ramirez leg.; caterpillar collected in third instar; single white ovoid small cocoons not adhered together, adhered to the larval cuticle and formed on 27.v.1999; adult parasitoids emerged on 06.vi.1999.

*Área de Conservación Guanacaste*, *Guanacaste*, *Sector Del Oro*, *Guacimos*: • 8 (2♀, 2♂) (4♀, 0♂); 08-SRNP-21695, DHJPAR0031029; rain forest; 380 m; 11.01454, -85.47492; 21.vi.2008; Roster Moraga leg.; caterpillar collected in fourth instar; white scattered cocoons adhered to the leaf substrate.

#### Diagnosis.

**Figure 29.**
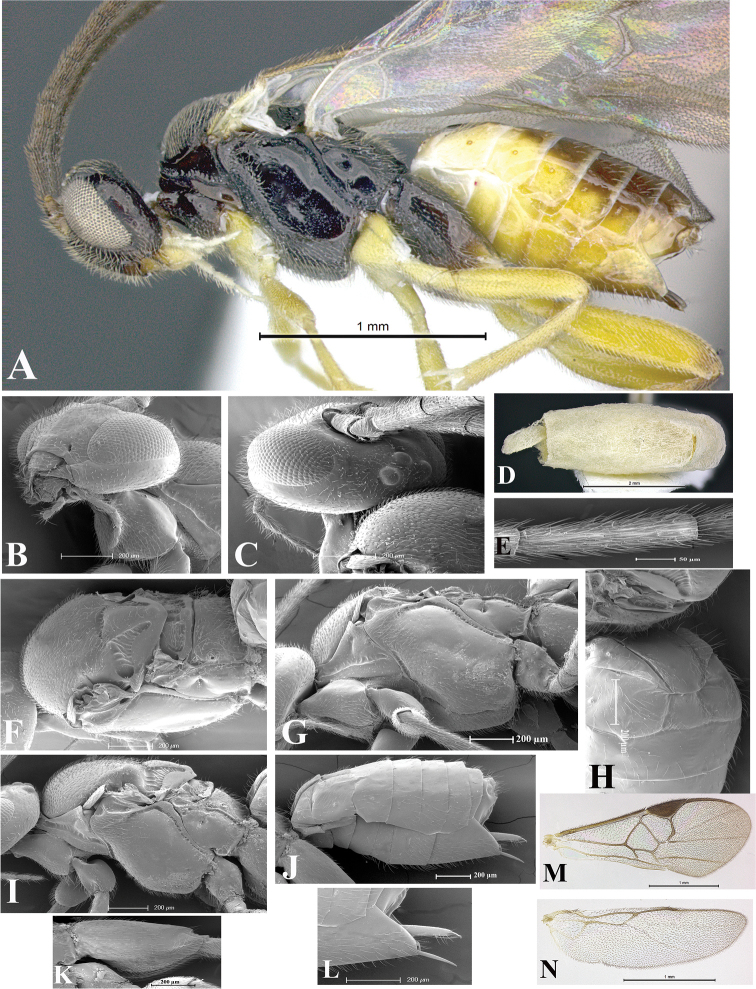
*Glyptapantelesbillbrowni* sp. nov. female 06-SRNP-65722, DHJPAR0012678 **A** Habitus **B, C** Head **B** Ventrolateral view **C** Dorsal view **D** Cocoon **E** Flagellomeres **F, G, I** Mesosoma **F** Dorsolateral view **G** Ventrolateral view **I** Lateral view **H**T1–3, dorsolateral view **J** Metasoma, lateral view **K** Hind coxa, lateral view **L** Genitalia: hypopygium, ovipositor, ovipositor sheaths, lateral view **M, N** Wings **M** Fore **N** Hind.

**Figure 30.**
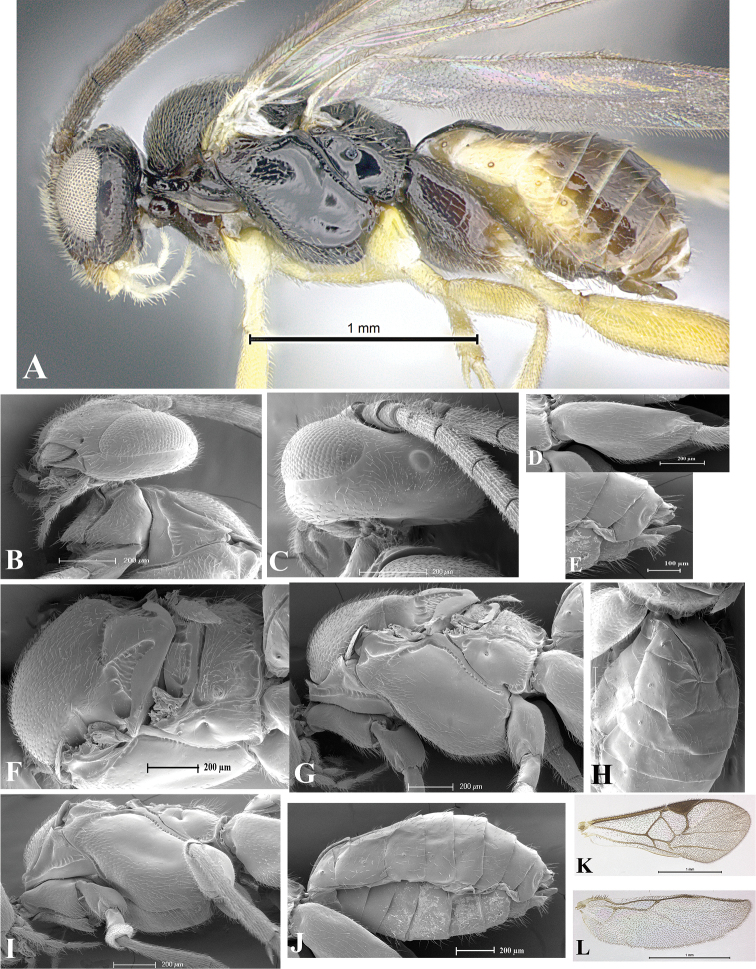
*Glyptapantelesbillbrowni* sp. nov. male 06-SRNP-65722 DHJPAR0012678 **A** Habitus **B** Head, pronotum, propleuron, lateral view **C** Head, dorsal view **D** Hind coxa, lateral view **E** Genitalia: Parameres, lateral view **F, G, I** Mesosoma **F** Dorsolateral view **G** Lateral view **I** Ventrolateral view **H**T1–4, dorsolateral view **J** Metasoma, lateral view **K, L** Wings **K** Fore **L** Hind.

Antenna as same length as body, scutellum in profile slightly convex, but on same plane as mesoscutum (Figs 29I, 30G), in dorsal view, proximal half of propodeum weakly curved (Figs 29F, 30F). petiole on T1 evenly narrowing distally (Figs 29H, 30H), dorsal outer depression on hind coxa present (Figs 29A, 30A), edges of median area on T2 obscured by little sculpture (Figs 29H, 30H), and fore wing with r vein curved, outer side of junction of r and 2RS veins forming a distinct stub (Figs 29M, 30K).

#### Coloration

(Fig. 29A). General body coloration brown-black except labrum, glossa, mandibles, maxillary and labial palps, and tegulae yellow; scape, pedicel and clypeus yellow-brown. Eyes and ocelli silver. Fore and middle legs yellow with claws brown; hind legs yellow except coxae brown-black with apex yellow/yellow-brown, ventrally yellow-brown coloration covers the distal half, femora with a brown dot at the tip, tibiae with both ends brown, and tarsomeres brown. Petiole on T1 brown and sublateral areas yellow-brown; T2 with median and adjacent areas brown, and lateral ends narrow and yellow-brown; T3 and beyond completely brown; distally each tergum with a wide yellowish transparent band. In lateral view, T1–3 completely yellow; T4 and beyond yellow-brown, but dorsally brown, extent of brown area remains constant. All sterna completely yellow, although hypopygium medially brown; ovipositor sheaths brown.

#### Description.

**Head** (Fig. 29A–C, E). Head rounded with pubescence long and dense. Proximal three antennal flagellomeres longer than wide (0.22:0.07, 0.24:0.07, 0.23:0.07), distal antennal flagellomere longer than penultimate (0.13:0.06, 0.11:0.06), antenna as same length as body (2.78, 2.78); antennal scrobes-frons shallow. Face flat or nearly so, finely punctate-lacunose, interspaces smooth and longitudinal median carina present. Frons smooth. Temple wide, punctate and interspaces clearly smooth. Inner margin of eyes straight throughout; in lateral view, eye anteriorly convex and posteriorly straight. POL shorter than OOL (0.10, 0.13). Malar suture present. Median area between lateral ocelli without depression. Vertex laterally rounded and dorsally wide.

**Mesosoma** (Fig. 29A, F, G, I). Mesosoma dorsoventrally convex. Mesoscutum proximally convex and distally flat with punctation distinct throughout, and interspaces wavy/lacunose. Scutellum triangular, apex sloped and fused with BS, in profile slightly convex, but on same plane as mesoscutum, phragma of the scutellum completely concealed, scutellar punctation distinct throughout; BS only very partially overlapping the MPM; ATS demilune with a little, complete parallel carinae; dorsal ATS groove with semicircular/parallel carinae. Transscutal articulation with small and heterogeneous foveae; area just behind transscutal articulation with a smooth, shiny and sloped transverse strip. Metanotum with BM wider than PFM (clearly differentiated); MPM circular without median longitudinal carina; AFM without setiferous lobes and not as well delineated as PFM; PFM thick and smooth; ATM proximally with semircular/undulate carina and distally smooth. Propodeum without median longitudinal carina, proximal half weakly curved with fine sculpture and distal half relatively polished and with a shallow dent at each side of nucha; distal edge of propodeum with a flange at each side and short stubs; propodeal spiracle without distal carina; nucha surrounded by very short radiating carinae. Pronotum with a distinct dorsal furrow, dorsally with a well-defined smooth band; central area of pronotum and dorsal furrow smooth, but ventral furrow with short parallel carinae. Propleuron finely sculptured only ventrally and dorsally without a carina. Metasternum flat or nearly so. Contour of mesopleuron convex; precoxal groove smooth, shiny and shallow, but visible; epicnemial ridge elongated more fusiform (tapering at both ends).

**Legs** (Fig. 29A, K). Ventral margin of fore telotarsus entire without seta, fore telotarsus proximally narrow and distally wide and longer than fourth tarsomere (0.12, 0.06). Hind coxa with dorsal half sparsely punctate, ventral half densely punctate, and dorsal outer depression present. Inner spur of hind tibia longer than outer spur (0.25, 0.19). Entire surface of hind tibia with dense strong spines clearly differentiated by color and length. Hind telotarsus longer than fourth tarsomere (0.14, 0.11).

**Wings** (Fig. 29M, N). Fore wing with r vein slightly curved; 2RS vein straight; r and 2RS veins forming a weak, even curve at their junction and outer side of junction forming a slight stub; 2M vein slightly curved/swollen; distally fore wing [where spectral veins are] with microtrichiae more densely concentrated than the rest of the wing; anal cell 1/3 proximally lacking microtrichiae; subbasal cell proximal half smooth; veins 2CUa and 2CUb completely spectral; vein 2 cu-a present as spectral vein, sometimes difficult to see; vein 2-1A proximally tubular and distally spectral, although sometimes difficult to see; tubular vein 1 cu-a curved, incomplete/broken, and not reaching the edge of 1-1A vein. Hind wing with vannal lobe narrow, subdistally evenly convex, subproximally straightened, and setae evenly scattered in the margin.

**Metasoma** (Fig. 29A, H, J, L). Metasoma cylindrical. Petiole on T1 finely sculptured only laterally, petiole evenly narrowing distally, apex truncate (length 0.34, maximum width 0.21, minimum width 0.12), with scattered pubescence concentrated in the first distal third. Lateral grooves delimiting the median area on T2 clearly defined and reaching the distal edge of T2 (length median area 0.15, length T2 0.15), edges of median area with little sculpture, median area broader than long (length 0.15, maximum width 0.17, minimum width 0.07); T2 with a distinctive row of pubescence only at the distal margin. T3 longer than T2 (0.23, 0.15) and with scattered pubescence throughout. Pubescence on hypopygium dense.

**Cocoons** (Fig. 29D). White oval cocoons with silk fibers evenly smooth. Single small cocoons not adhered to each other, adhered to the larval cuticle or to the leaf substrate.

#### Comments.

The AFM on metanotum has a small lobe, but without setae. The flange at each distal side of propodeum with a distinctive curvature and long stubs.

#### Male

(Fig. 30A–L). The coloration on terga and sterna is darker than in females.

#### Etymology.

William (Bill) L. Brown, Jr. (1 June 1922–30 March 1997) was a well-known American myrmecologist; his research was focused mainly on the ant subfamily Ponerinae.

#### Distribution.

The parasitized caterpillars were collected in Costa Rica, ACG, Sector Cacao (Estación Cacao), Sector Del Oro (Guacimos), and Sector Pitilla (Sendero Carica), during May 1995, June 2008, and December 2006 at 380 m, 660 m, and 1,150 m in rain forest and cloud forest.

#### Biology.

The lifestyle of this parasitoid species is gregarious.

#### Host.

*Xylophanesporcus* (Hübner) (Sphingidae: Macroglossinae) feeding on *Hameliapatens* and *Psychotriaberteriana* (Rubiaceae). Caterpillars were collected in third and fourth instar.

### Glyptapanteles bobhanneri

Taxon classificationAnimaliaHymenopteraBraconidae

Arias-Penna, sp. nov.

http://zoobank.org/E9D71D65-3B00-4687-B4AE-7D599EF89F4E

Figs 31 , 32

#### Female.

Body length 2.53 mm, antenna length 2.98 mm, fore wing length 2.78 mm.

#### Type material.

**Holotype**: COSTA RICA • 1♀; 04-SRNP-33819, DHJPAR0001511]; Área de Conservación Guanacaste, Guanacaste, Sector Pitilla, Sendero Mismo; rain forest; 680 m; 10.98758, -85.41967; 01.vii.2004; Manuel Rios leg.; caterpillar collected in fourth instar; bud-like cocoons, scattered loose, adhered to the leaf substrate and formed on 11.vii.2004; adult parasitoids emerged on 17.vii.2004; (CNC). **Paratypes.** • 3 (1♀, 1♂) (1♀, 0♂); 04-SRNP-33819, DHJPAR0001511; same data as for holotype; (CNC).

#### Other material.

**Reared material.** COSTA RICA: *Área de Conservación Guanacaste*, *Guanacaste*, *Sector Pitilla*, *Sendero Mismo*: • 3 (2♀, 0♂) (1♀, 0♂); 04-SRNP-33811, DHJPAR0001473; rain forest; 680 m; 10.98758, -85.41967; 01.vii.2004; Petrona Rios leg.; caterpillar collected in fourth instar; white ridged bud-like cocoons adhered to the leaf substrate and formed on 09.vii.2004; adult parasitoids emerged on 17.vii.2004. • 5 (2♀, 0♂) (3♀, 0♂); 04-SRNP-33810, DHJPAR0001502; same data as for preceding except: scattered white bud-like cocoons adhered to the leaf substrate and formed on 06.vii.2004; adult parasitoids emerged on 14.vii.2004. • 3 (1♀, 1♂) (1♀, 0♂); 04-SRNP-33813, DHJPAR0001515; same data as for preceding except: caterpillar collected in third instar; white bud-like cocoons formed on 06.vii.2004; adult parasitoids emerged on 14.vii.2004. • 2 (0♀, 1♂) (0♀, 1♂); 04-SRNP-33808, DHJPAR0001518; same data as for preceding except: white bud-like cocoons formed on 06.vii.2004; adult parasitoids emerged on 14.vii.2004. • 2 (1♀, 0♂) (1♀, 0♂); 04-SRNP-33648, DHJPAR0001525; same data as for preceding except: 23.vi.2004; Manuel Rios leg.; caterpillar collected in third instar; white bud-like cocoons formed on 22.vii.2004 and adhered to the leaf substrate; adult parasitoids emerged on 28.vii.2004.

*Área de Conservación Guanacaste*, *Guanacaste*, *Sector Pitilla*, *Sendero Laguna*; • 3 (2♀, 0♂) (1♀, 0♂); 04-SRNP-33351, DHJPAR0001531; rain forest; 680 m; 10.9888, -85.42336; 15.vi.2004; Calixto Moraga leg.; caterpillar collected in third instar; white bud-like cocoons adhered to the leaf substrate and formed on 27.vi.2004; adult parasitoids emerged on 09.vii.2004.

*Área de Conservación Guanacaste*, *Guanacaste*, *Sector Pitilla*, *Estación Quica*: • 2 (1♀, 0♂) (1♀, 0♂); 08-SRNP-71182, DHJPAR0031102; rain forest; 470 m; 10.99697, -85.39666; 07.vii.2008; Oscar Siezar leg.; caterpillar collected in third instar; white bud-like cocoons adhered to the leaf substrate and formed on 29.vii.2008; adult parasitoids emerged on 06.viii.2008.

#### Diagnosis.

**Figure 31.**
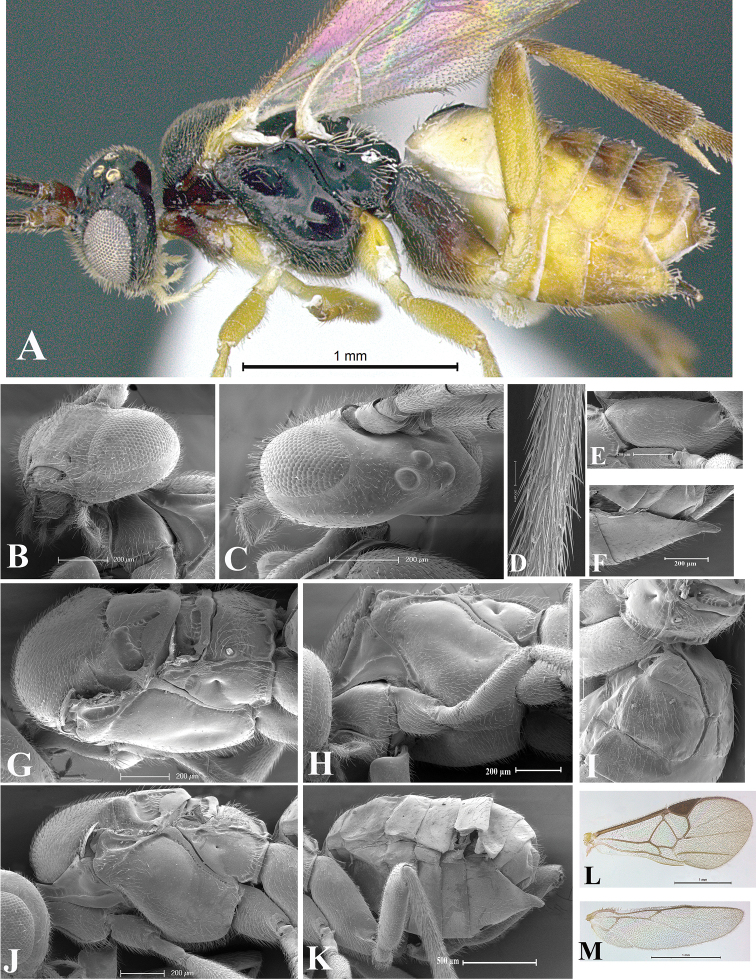
*Glyptapantelesbobhanneri* sp. nov. female 04-SRNP-33819 DHJPAR0001511 **A** Habitus **B, C** Head **B** Laterofrontal view **C** Dorsal view **D** Flagellomeres **E** Hind coxa, lateral view **F** Genitalia: hypopygium, ovipositor sheaths, lateral view **G, H, J** Mesosoma **G** Dorsolateral view **H** Ventrolateral view **J** Lateral view **I**T1–2, dorsolateral view **K** Metasoma, lateral view **L, M** Wings **L** Fore **M** Hind.

**Figure 32.**
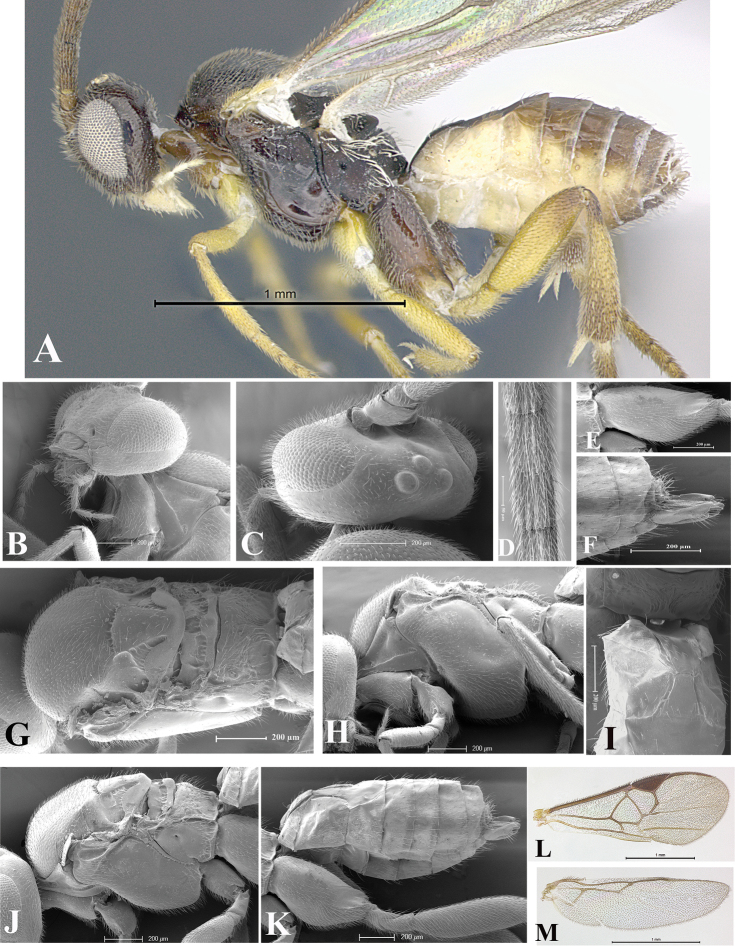
*Glyptapantelesbobhanneri* sp. nov. male 04-SRNP-33819 DHJPAR0001511 **A** Habitus **B, C** Head **B** Laterofrontal view **C** Dorsal view **D** Flagellomeres **E** Hind coxa, lateral view **F** Genitalia: parameres, lateral view **G, H, J** Mesosoma **G** Dorsolateral view **H** Ventrolateral view **J** Lateral view **I**T1–2, dorsal view **K** Metasoma, lateral view **L, M** Wings **L** Fore **M** Hind.

Precoxal groove smooth and shiny (Figs 31A, G, H, 32A), medioanterior pit of metanotum circular without median longitudinal carina (Figs 31G, 32G), inner margin of eyes straight throughout, scutellar punctation indistinct throughout (Figs 31G, 32G), fore wing with 1 cu-a vein complete, touching the edge of 1-1A vein, outer side of junction of r and 2RS veins not forming a stub (Figs 31L, 32L), propodeum with a median longitudinal carina (Figs 31G, 32G), and lateral grooves delimiting the median area on T2 clearly defined and reaching the distal edge of T2 (Figs 31H, 32K).

#### Coloration

(Fig. 31A). General body coloration dark brown black except labrum, mandibles, glossa, maxillary and labial palps, and tegulae yellow-brown. Eyes silver and ocelli silver/yellowish. Fore and middle legs yellow-brown, fore claws brown; hind legs yellow-brown except coxae brown-black with apex yellow-brown, apex of femora, both ends of tibiae brown and tarsomeres brown although tarsomeres 1–3 with a proximal yellow-brown ring. Petiole on T1 black-brown and sublateral areas yellow-brown; T2 with median area brown with contours darkened, adjacent area narrow and brown, and lateral ends yellow-brown; T3 and beyond yellow-brown/brown; distally each tergum with a narrow whitish band. In lateral view, T1–2 completely yellow; T3 and beyond yellow, but dorsally brown, extent of brown area remains constant in each tergum. All sterna yellow, but hypopygium medially brown and some lateral parts too; ovipositor sheaths brown.

#### Description.

**Head** (Fig. 31A–D). Head triangular with pubescence long and dense. Proximal three antennal flagellomeres longer than wide (0.25:0.08, 0.25:0.08, 0.24:0.08), distal antennal flagellomere longer than penultimate (0.12:0.06, 0.10:0.05), antenna longer than body (2.98, 2.53); antennal scrobes-frons shallow. Face convex with scattered finely punctate, interspaces smooth, and longitudinal median carina present. Frons smooth. Temple wide, punctate, and interspaces clearly smooth. Inner margin of eyes straight throughout; in lateral view, eye anteriorly convex and posteriorly straight. POL shorter than OOL (0.09, 0.12). Malar suture present. Median area between lateral ocelli without depression. Vertex laterally rounded and dorsally wide.

**Mesosoma** (Fig. 31A, G, H, J). Mesosoma dorsoventrally convex. Mesoscutum convex, punctation distinct throughout, and interspaces smooth. Scutellum long and slender, apex sloped and fused with BS, scutellar punctation indistinct throughout, scutellum in profile flat and on same plane as mesoscutum, phragma of the scutellum partially exposed; BS only very partially overlapping the MPM; ATS demilune with a little and complete parallel carinae; dorsal ATS groove with carinae only proximally. Transscutal articulation with small and heterogeneous foveae; area just behind transscutal articulation smooth, shiny and depressed centrally. Metanotum with BM upward; MPM circular without median longitudinal carina; AFM without setiferous lobes and not as well delineated as PFM; PFM thick and smooth; ATM proximally with little and incomplete parallel carinae. Propodeum with a clearly visible median longitudinal carina, proximal half straight or nearly so, with fine sculpture and distal half rugose with a shallow dent at each side of nucha; distal edge of propodeum with a flange at each side and short stubs; propodeal spiracle without distal carina; nucha surrounded by very short radiating carinae. Pronotum with a distinct dorsal furrow, dorsally with a well-defined smooth band; central area of pronotum smooth, but both dorsal and ventral furrows with short parallel carinae. Propleuron finely sculptured only ventrally and dorsally without a carina. Metasternum flat or nearly so. Contour of mesopleuron convex; precoxal groove smooth, shiny and shallow, but visible; epicnemial ridge convex, teardrop-shaped.

**Legs** (Fig. 31A, E). Ventral margin of fore telotarsus slightly excavated and with a tiny curved seta, fore telotarsus proximally narrow and distally wide, and longer than fourth tarsomere (0.12, 0.06). Hind coxa finely punctate throughout, and dorsal outer depression absent. Inner spur of hind tibia longer than outer spur (0.24, 0.15); entire surface of hind tibia with dense strong spines clearly differentiated by color and length. Hind telotarsus as equal as fourth tarsomere (0.11, 0.10).

**Wings** (Fig. 31L, M). Fore wing with r vein curved; 2RS vein straight; r and 2RS veins forming a weak, even curve at their junction and outer side of junction not forming a stub; 2M vein slightly curved/swollen; distally fore wing [where spectral veins are] with microtrichiae more densely concentrated than the rest of the wing; anal cell 1/3 proximally lacking microtrichiae; subbasal cell with microtrichiae virtually throughout; veins 2CUa and 2CUb completely spectral; vein 2 cu-a present as spectral vein, sometimes difficult to see; vein 2-1A proximally tubular and distally spectral, although sometimes difficult to see; tubular vein 1 cu-a curved, complete, and touching the edge of 1-1A vein. Hind wing with vannal lobe very narrow, subdistally and subproximally evenly convex, and setae evenly scattered in the margin.

**Metasoma** (Fig. 31A, F, I, K). Metasoma laterally compressed. Petiole on T1 finely sculptured only laterally, evenly narrowing distally and apex truncate (length 0.39, maximum with 0.22, minimum width 0.08) with pubescence on distal half. Lateral grooves delimiting the median area on T2 clearly defined and reaching the distal edge of T2 (length median area 0.22, length T2 0.22), edges of median area polished and lateral grooves deep, median area longer than broad (length 0.22 mm, maximum width 0.18, minimum width 0.08); T2 scarce pubescence throughout. T3 longer than T2 (0.25, 0.22) and with scattered pubescence throughout. Pubescence on hypopygium dense.

**Cocoons.** White bud-like cocoon with body ridge-shaped and silk fibers evenly smooth. Cocoons scattered loose and adhered to the leaf substrate.

#### Comments.

The shape of pronotum is characteristic, the distal half is convex instead of concave. The propodeum with an incomplete median longitudinal carina.

#### Male

(Fig. 32A–M). Similar in coloration to females. Males tend to be slenderer than females.

#### Etymology.

Robert (Bob) Hanner is working as Associate Director at Canadian Barcode of Life Network at the Biodiversity Institute of Ontario (BIO), University of Guelph, Ontario, Canada.

#### Distribution.

The parasitized caterpillars were collected in Costa Rica, ACG, Sector Pitilla (Estación Quica, Sendero Laguna, and Sendero Mismo), during June-July 2004 and July 2008 at 680 m in rain forest.

#### Biology.

The lifestyle of this parasitoid species is gregarious.

#### Host.

*Scoturaleucophleps* Warren (Notodontidae: Dioptinae) feeding on *Rinoreadeflexiflora* and *R. sylvatica* (Violaceae). Caterpillars were collected in third and fourth instar.

### Glyptapanteles bobkulai

Taxon classificationAnimaliaHymenopteraBraconidae

Arias-Penna, sp. nov.

http://zoobank.org/51AA25B7-D7B3-4090-83EB-910D1240828A

Figs 33 , 34

#### Female.

Body length 2.32 mm, antenna length 3.23 mm, fore wing length 2.78 mm.

#### Type material.

**Holotype**: COSTA RICA • 1♀; 10-SRNP-30219, DHJPAR0038360; Área de Conservación Guanacaste, Guanacaste, Sector Pitilla, Sendero Mismo; rain forest; 680 m; 10.98758, -85.41967; 14.i.2010; Petrona Rios leg.; caterpillar collected in fourth instar; white bud-like cocoon adhered to the leaf substrate and formed on 22.i.2010; adult parasitoid emerged on 01.ii.2010; (CNC). **Paratype.** 1 (0♀, 0♂) (1♀, 0♂); 10-SRNP-30221, DHJPAR0038328; same data as for holotype except: caterpillar collected in third instar; cocoons formed on 27.i.2010 in litter or soil; (CNC).

#### Other material.

**Reared material.** COSTA RICA: *Área de Conservación Guanacaste*, *Alajuela*, *Sector San Cristobal*, *Sendero Pinyal*: • 1 (0♀, 1♂) (0♀, 0♂); 03-SRNP-7211, DHJPAR0004082; rain forest; 630 m; 10.87161, -85.39333; 14.vii.2003; Carolina Cano leg.; caterpillar collected in third instar; single white elongate bud-like cocoon adhered to the leaf substrate; adult parasitoid emerged on 25.vii.2003.

*Área de Conservación Guanacaste*, *Guanacaste*, *Sector Cacao*, *Sendero a Maritza*: • 1 (1♀, 0♂) (0♀, 0♂); 06-SRNP-35967, DHJPAR0012328; 570 m; 10.95727, -85.49514; 17.viii.2006; Dunia Garcia leg.; caterpillar collected in third instar; single white bud-like cocoon in litter or soil and formed on 22.viii.2006; adult parasitoid emerged on 01.ix.2006. • 1 (0♀, 0♂) (0♀, 1♂); 06-SRNP-35969, DHJPAR0012331; same data as for preceding.

*Área de Conservación Guanacaste*, *Guanacaste*, *Sector Cacao*, *Estación Cacao*: • 1 (0♀, 0♂) (1♀, 0♂); 08-SRNP-37409, DHJPAR0030700; cloud forest; 1,150 m; 10.92691, -85.46822; 29.x.2008; Dunia Garcia leg.; caterpillar collected in third instar; slightly beige bud-like cocoon adhered to the leaf substrate and formed on 03.xi.2008; adult parasitoid emerged on 03.xi.2008. • 1 (0♀, 0♂) (1♀, 0♂); 10-SRNP-35622, DHJPAR0040452; same data as for preceding except: 30.vii.2010; Harry Ramirez leg.; caterpillar collected in first instar; cocoon formed on 11.viii.2010; adult parasitoid emerged on 22.viii.2010.

*Área de Conservación Guanacaste*, *Guanacaste*, *Sector Cacao*, *Sendero Ponderosa*: • 2 (0♀, 1♂) (0♀, 1♂); 09-SRNP-35753, DHJPAR0035424; cloud forest; 1,060 m; 10.91460, -85.46262; 06.v.2009; Manuel Pereira leg.; caterpillar collected in third instar; white bud-like cocoons in litter or soil formed on 10.v.2009; adult parasitoids emerged on 15.v.2009.

#### Diagnosis.

**Figure 33.**
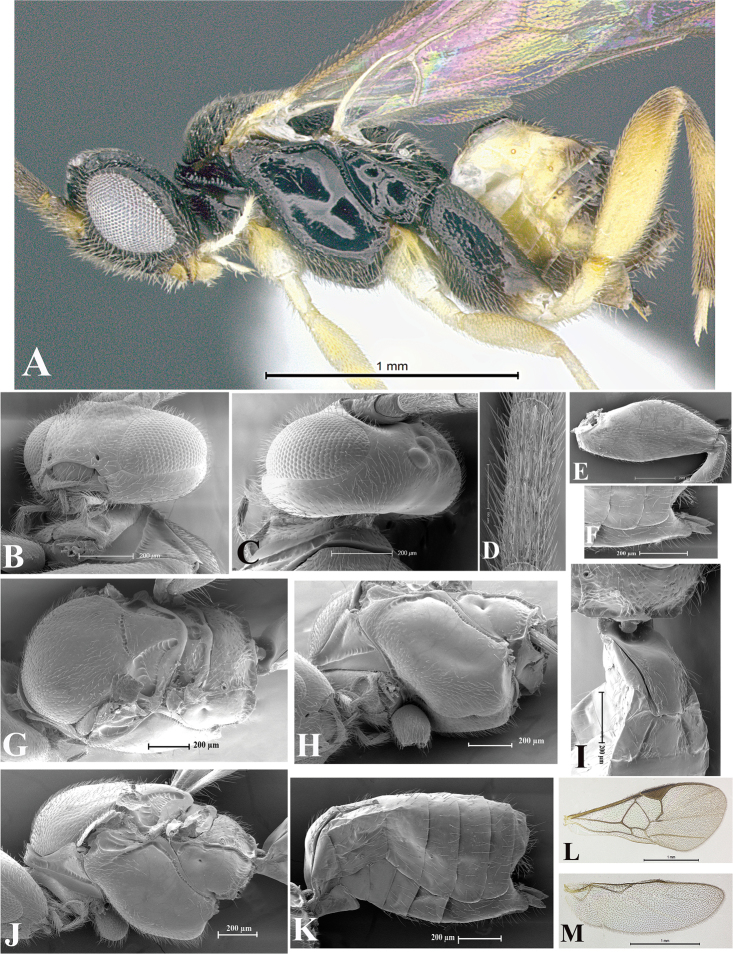
*Glyptapantelesbobkulai* sp. nov. female 06-SRNP-35967 DHJPAR0012328 10-SRNP-30219 DHJPAR0038360 **A** Habitus **B, C** Head **B** Laterofrontal view **C** Dorsolateral view **D** Flagellomeres **E** Hind coxa, lateral view **F** Genitalia: hypopygium, ovipositor sheaths, lateral view **G, H, J** Mesosoma **G** Dorsolateral view **H** Ventrolateral view **J** Lateral view **I**T1–2, dorsolateral view **K** Metasoma, lateral view **L, M** Wings **L** Fore **M** Hind.

**Figure 34.**
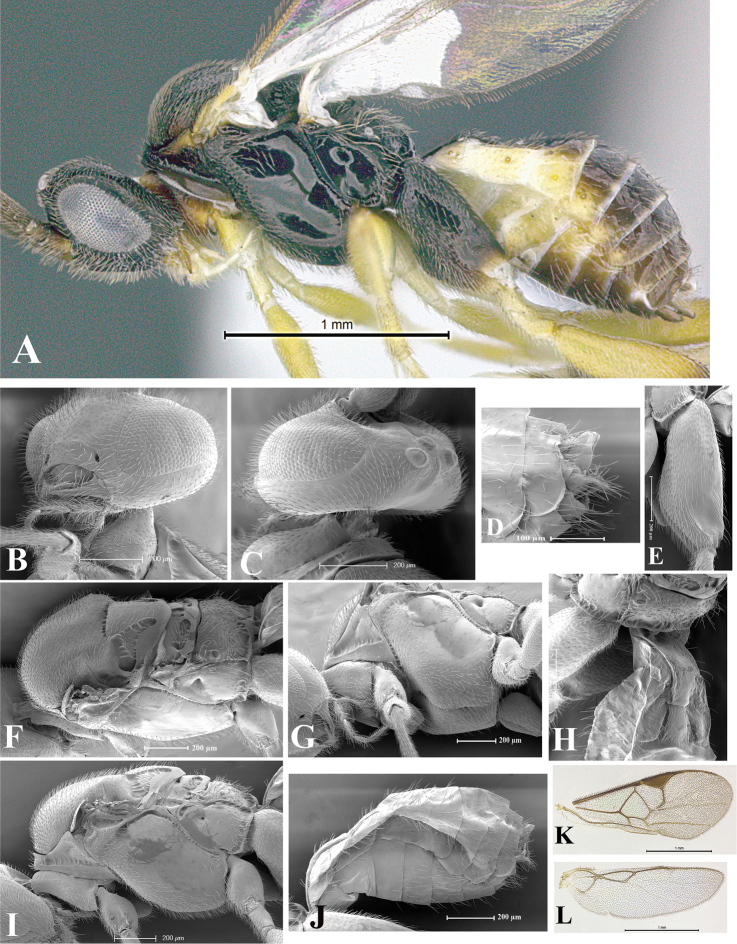
*Glyptapantelesbobkulai* sp. nov. male 03-SRNP-7211 DHJPAR0004082, 09-SRNP-35753 DHJPAR0035424 **A** Habitus **B, C** Head **B** Laterofrontal view **C** Dorsolateral view **D** Genitalia: parameres, lateral view **E** Hind coxa, lateral view **F, G, I** Mesosoma **F** Dorsolateral view **G** Ventrolateral view **I** Lateral view **H**T1–2, dorsolateral view **J** Metasoma, lateral view **K, L** Wings **K** Fore **L** Hind.

Scutellar punctation distinct peripherally, absent centrally (Figs 33G, 34F), fore wing with tubular vein 1 cu-a complete, touching the edge of 1-1A vein, r vein slightly curved, outer side of junction of r and 2RS veins forming a slight stub (Figs 33L, 34K), medioposterior band of scutellum only very partially overlapping the medioanterior pit of metanotum (Figs 33G, 34F), petiole on T1 evenly narrowing over its length (Figs 33I, 34H), surface of metasternum flat or nearly so (Figs 33H, 34G), edges of median area on T2 obscured by weak longitudinal stripes (Figs 33I, 34H), and dorsal outer depression on hind coxa absent (Figs 33A, 34E).

#### Coloration

(Fig. 33A). General body coloration brown-black except proximal half of scape, distal half of pedicel, labrum, mandibles, glossa, and maxillary and labial palps yellow; clypeus yellow-brown. Eyes and ocelli silver. Fore and middle legs yellow except fore claws brown (tarsomeres of mid legs are lost); hind legs yellow except coxae, apex of femora, both ends of tibiae, and tarsomeres brown. Petiole on T1 brown-black, and sublateral areas yellow; T2 with median and narrow adjacent areas brown, and lateral ends yellow; T3 and beyond dark brown; distally each tergum with a yellowish transparent band. In lateral view, T1–3 completely pale yellow; T4 and beyond yellow, but dorsally dark-brown, this dark coloration covering most of the area of each tergum. S1–4 pale yellow, penultimate sternum yellow, medially with a brown spot; hypopygium completely brown.

#### Description.

**Head** (Fig. 33A–D). Head triangular with pubescence long and dense. Proximal three antennal flagellomeres longer than wide (0.25:0.08, 0.23:0.08, 0.23:0.08), distal antennal flagellomere longer than penultimate (0.14:0.07, 0.10:0.07), antenna longer than body (3.23, 2.32); antennal scrobes-frons shallow. Face flat or nearly so, with punctate-lacunose punctation, interspaces smooth, and longitudinal median carina absent. Frons smooth. Temple wide with punctate and interspaces clearly smooth. Inner margin of eyes diverging slightly at antennal sockets; in lateral view, eye anteriorly convex and posteriorly straight. POL shorter than OOL (0.11, 0.13). Malar suture present. Median area between lateral ocelli without depression. Vertex laterally rounded and dorsally wide.

**Mesosoma** (Fig. 33A, G, H, J). Mesosoma dorsoventrally convex. Distal 1/3 of mesoscutum with lateral margin slightly dented, punctation distinct throughout, interspaces wavy/lacunose. Scutellum shape triangular, apex sloped and fused with BS, scutellar punctation distinct peripherally and absent centrally, scutellum in profile flat and on same plane as mesoscutum, phragma of the scutellum completely concealed; BS only very partially overlapping the MPM; ATS demilune with short stubs delineating the area; dorsal ATS groove with semicircular/parallel carinae. Transscutal articulation with small and heterogeneous foveae; area just behind transscutal articulation smooth, shiny nearly at the same level as mesoscutum (flat). Metanotum with BM wider than PFM (clearly differentiated); MPM circular and bisected by a median longitudinal carina; AFM without setiferous lobes and not as well delineated as PFM; PFM thick and smooth; ATM proximally with semircular/undulate carina and distally smooth. Propodeum without median longitudinal carina, proximal half weakly curved with medium-sized sculpture and distal half rugose with a shallow dent at each side of nucha; distal edge of propodeum with a flange at each side and short stubs; propodeal spiracle without distal carina; nucha surrounded by very short radiating carinae. Pronotum with a distinct dorsal furrow, dorsally with a well-defined smooth band; central area of pronotum and dorsal furrow smooth, but ventral furrow with short parallel carinae. Propleuron with fine punctations throughout and dorsally without a carina. Metasternum flat or nearly so. Contour of mesopleuron convex; precoxal groove smooth, shiny and shallow, but visible; epicnemial ridge convex, teardrop-shaped.

**Legs** (Fig. 33A, E). Ventral margin of fore telotarsus slightly excavated and with a tiny curved seta, fore telotarsus proximally narrow and distally wide, and longer than fourth tarsomere (0.12, 0.06). Hind coxa finely punctate throughout, and dorsal outer depression absent. Inner spur of hind tibia longer than outer spur (0.23, 0.19); entire surface of hind tibia with dense strong spines clearly differentiated by color and length. Hind telotarsus longer than fourth tarsomere (0.15, 0.13).

**Wings** (Fig. 33L, M). Fore wing with r vein slightly curved; 2RS vein straight; r and 2RS veins forming an angle at their junction and outer side of junction forming a slight stub; 2M vein slightly curved/swollen; distally fore wing [where spectral veins are] with microtrichiae more densely concentrated than the rest of the wing; anal cell 1/3 proximally lacking microtrichiae; subbasal cell with microtrichiae virtually throughout; veins 2CUa and 2CUb completely spectral; vein 2 cu-a present as spectral vein, sometimes difficult to see; vein 2-1A proximally tubular and distally spectral, although sometimes difficult to see; tubular vein 1 cu-a straight, complete, and touching the edge of 1-1A vein. Hind wing with vannal lobe narrow, subdistally evenly convex, subproximally straightened, and setae evenly scattered in the margin.

**Metasoma** (Fig. 33A, F, I, K). Metasoma laterally compressed. Petiole on T1 finely sculptured only laterally, petiole evenly narrowing distally, apex truncate (length 0.35, maximum width 0.20, minimum width 0.11), petiole with scattered pubescence on distal half only laterally. Lateral grooves delimiting the median area on T2 clearly defined and reaching the distal edge of T2 (length median area 0.18, length T2 0.18), edges of median area obscured by weak longitudinal stripes, median area as broad as long (length 0.18, maximum width 0.20, minimum width 0.11); T2 with scarce pubescence throughout. T3 longer than T2 (0.22, 0.18) and with scattered pubescence throughout. Pubescence on hypopygium dense.

**Cocoon.** White, beige bud-like cocoon with body ridge-shaped and silk fibers evenly smooth. Cocoon adhered to the leaf substrate, in litter or soil.

#### Male

(Fig. 34A–L). The propleuron has a yellow-brown coloration; the dorsal furrow of pronotum is yellow; the S1–3 are yellow, the S4 and beyond are brown.

#### Etymology.

Robert (Bob) Kula is a research entomologist with the Systematic Entomology Laboratory, Agricultural Research Service, United States Department of Agriculture (USDA), Washington, DC. He is an adjunct scientist with the Smithsonian Institution and is curator of Ichneumonoidea at the National Museum of Natural History, Washington, DC. His research focuses on classification, evolution, and biodiversity of parasitoid wasps in Braconidae.

#### Distribution.

Parasitized caterpillars were collected in Costa Rica, ACG, Sector Cacao (Estación Cacao, Sendero a Maritza, and Sendero Ponderosa), Sector San Cristobal (Sendero Pinyal), and Sector Pitilla (Sendero Mismo), during July 2003, August 2006, October 2008, May 2009, and January and July 2010 at 570 m, 630 m, 680 m, 1,060 m, and 1,150 m in rain and cloud forests.

#### Biology.

The lifestyle of this parasitoid species is solitary/gregarious.

#### Host.

*Eois* sp. Hübner (Geometridae: Larentiinae) feeding on *Piperaugustum*, *P.glabrescens* (Piperaceae); *Hagnagoramortipax* Butler (Geometridae: Larentiinae) feeding on *Clethramexicana* (Clethraceae); *Semaeopusillimitata* Warren (Geometridae: Sterrhinae) feeding on *Abutapanamensis* (Menispermaceae); undetermined species of Geometridae feeding on *Tremamicrantha* (Cannabaceae). Caterpillars were collected in first, third, and fourth instar.

### Glyptapanteles bobwhartoni

Taxon classificationAnimaliaHymenopteraBraconidae

Arias-Penna, sp. nov.

http://zoobank.org/A832338C-0C91-42E7-A8C0-5689B00F3CF4

Figs 35 , 36

#### Female.

Body length 2.93 mm, antenna length 2.63 mm, fore wing length 2.83 mm.

#### Type material.

**Holotype**: COSTA RICA • 1♀; 04-SRNP-55913, DHJPAR0004232; Área de Conservación Guanacaste, Guanacaste, Sector Pitilla, Sendero Mismo; rain forest; 680 m; 10.98758, -85.41967; 10.x.2004; Manuel Rios leg.; caterpillar collected in second instar; white bud-like elongate cocoon adhered to the leaf substrate and formed on 02.xi.2004; adult parasitoid emerged on 11.xi.2004; (CNC). **Paratype.** 1 (0♀, 0♂) (1♀, 0♂); 04-SRNP-55913, DHJPAR0004232; same data as for holotype; (CNC).

#### Other material.

**Reared material.** COSTA RICA: *Área de Conservación Guanacaste*, *Alajuela*, *Sector San Cristóbal*, *Sendero Perdido*: • 5 (3♀, 1♂) (1♀, 0♂); 03-SRNP-5375, DHJPAR0000046; rain forest; 620 m; 10.8794, -85.38607, 10.ii.2003; Carolina Cano leg.; caterpillar collected in fourth instar; white bud-like cocoons adhered to the leaf substrate and formed on 13.ii.2003; adult parasitoids emerged on 23.ii.2003.

*Área de Conservación Guanacaste*, *Guanacaste*, *Sector Pitilla*, *Sendero Mismo*: • 2 (1♀, 0♂) (0♀, 1♂); 04-SRNP-55910, DHJPAR0002900; rain forest; 680 m; 10.98758, -85.41967;10.x.2004; Manuel Rios leg.; caterpillar collected in second instar; white elongate ridged bud-like cocoons in litter or soil and formed on 02.xi.2004; adult parasitoids emerged on 07.xi.2004. • 2 (1♀, 0♂) (1♀, 0♂); 04-SRNP-55980, DHJPAR0002901; same data as for preceding except: white bud-like cocoons adhered to the leaf substrate and formed on 28.x.2004. • 2 (1♀, 0♂) (1♀, 0♂); 04-SRNP-55922, DHJPAR0002902; same data as for preceding except: white bud-like cocoons adhered to the leaf substrate and formed on 28.x.2004; adult parasitoids emerged on 06.xi.2004. • 5 (2♀, 2♂) (1♀, 0♂); 04-SRNP-55912, DHJPAR0002903; same data as for preceding except: white ridged elongate solitary bud-like cocoons formed on 07.xi.2004 adhered to the leaf substrate; adult parasitoids emerged on 14.xi.2004. • 1 (0♀, 0♂) (1♀, 0♂, 4 died before emerged); 04-SRNP-55916, DHJPAR0002905; same data as for preceding except: white ridged elongate bud-like cocoons adhered to the leaf substrate and formed on 02.xi.2004; adult parasitoids emerged on 07.xi.2004. • 2 (1♀, 0♂) (1♀, 0♂); 04-SRNP-55909, DHJPAR0004222; same data as for preceding except: white bud-like cocoons adhered to the leaf substrate and formed on 28.x.2004. • 4 (3♀, 0♂) (1♀, 0♂); 04-SRNP-55911, DHJPAR0004235; same data as for preceding except: white bud-like cocoons adhered to the leaf substrate and formed on 06.xi.2004; adult parasitoids emerged on 16.xi.2004. • 2 (1♀, 0♂) (1♀, 0♂); 04-SRNP-55920, DHJPAR0004236; same data as for preceding except: adult parasitoids emerged on 06.xi.2004.

*Área de Conservación Guanacaste*, *Alajuela*, *Sector Rincón Rain Forest*, *Sendero Rincón*: • 1 (0♀, 0♂) (1♀, 0♂); 05-SRNP-40591, DHJPAR0004243; 430 m; 10.8962, -85.27769; 19.ii.2005; José Pérez leg.; caterpillar collected in third instar; white bud-like cocoon adhered to the leaf substrate; adult parasitoid emerged on 28.ii.2005.

#### Diagnosis.

**Figure 35.**
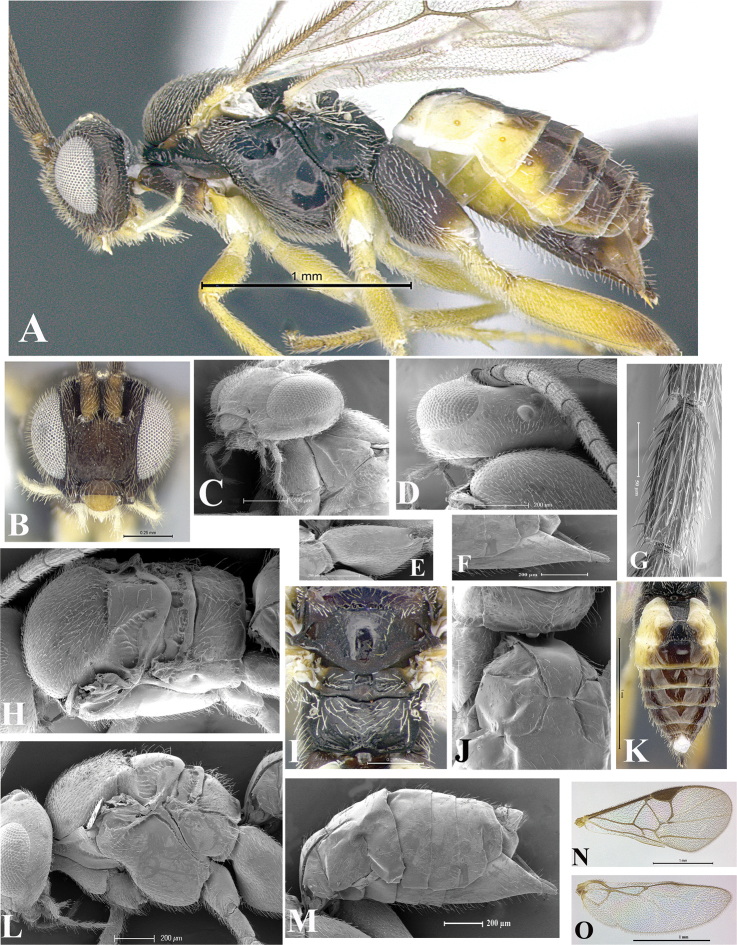
*Glyptapantelesbobwhartoni* sp. nov. female 04-SRNP-55912 DHJPAR0002903, 04-SRNP-55913 DHJPAR0004232 **A** Habitus **B, D** Head **B** Frontal view **C** Laterofrontal view **D** Dorsolateral view **E** Hind coxa, lateral view **F** Genitalia: hypopygium, ovipositor sheaths, lateral view **G** Flagellomeres **H** Mesosoma, dorsolateral view **I** Scutellum, metanotum, propodeum, dorsal view **J**T1–2, dorsal view **K, M** Metasoma **K** Dorsal view **M** Lateral view **L** Mesosoma, lateral view **N, O** Wings **N** Fore **O** Hind.

**Figure 36.**
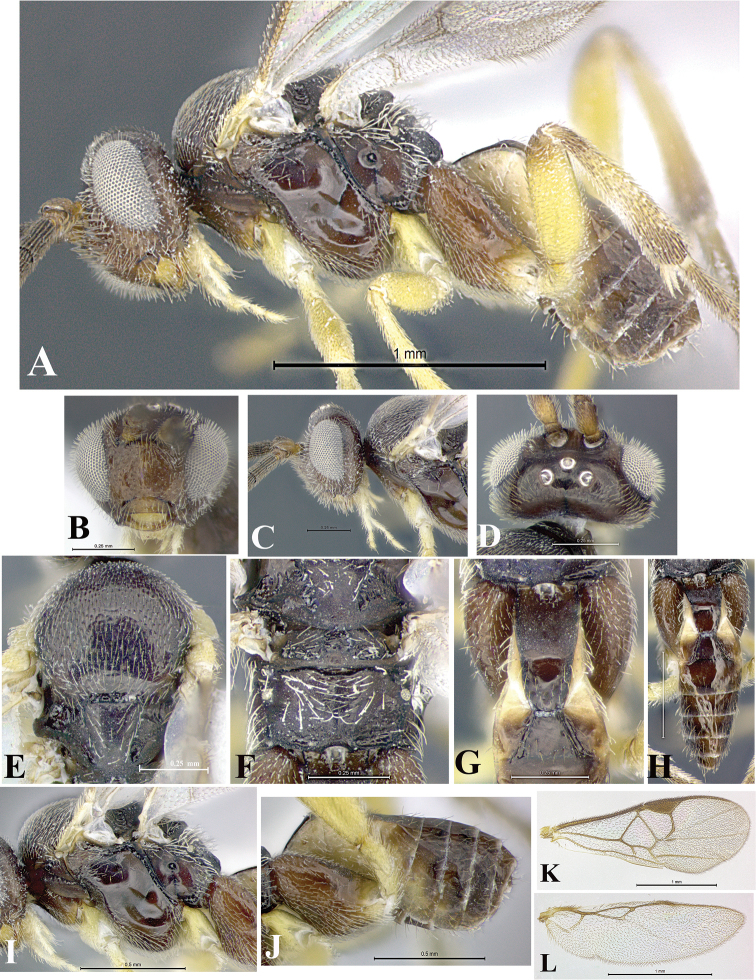
*Glyptapantelesbobwhartoni* sp. nov. male 04-SRNP-55912 DHJPAR0002903 **A** Habitus **B, D** Head **B** Frontal view **C** Lateral view **D** Dorsal view **E** Mesonotum, dorsal view **F** Scutellum, metanotum, propodeum, dorsal view **G**T1–2, dorsal view **H, J** Metasoma **H** Dorsal view **J** Lateral view **I** Mesosoma, lateral view **K, L** Wings **K** Fore **L** Hind.

Hind coxa with dorsal half sparsely punctate, ventral half densely punctate (Figs 35A, 36J), antenna shorter than body, distal antennal flagellomere subequal in length with penultimate, scutellar punctation distinct peripherally, absent centrally (Figs 35I, 36E), fore wing with vein 2 cu-a present as spectral vein, sometimes difficult to see, r vein straight, outer side of junction of r and 2RS veins forming a stub (Figs 35N, 36K), median area on T2 broader than long, edges of median area on T2 obscured by weak longitudinal stripes, and lateral grooves delimiting the median area on T2 distally losing definition on T2 (Figs 35J, K, 36G, H), vertex in dorsal view wide (Fig. 36D), in lateral view, metasoma laterally compressed (Figs 35M, 36J), T3 longer than T2 (Figs 35K, 36H), inner margin of eyes diverging slightly at antennal sockets (Figs 35B, 36B), petiole on T1 evenly narrowing distally (wide base to a narrow apex) and finely sculptured (Figs 35J, 36G), and propodeum without a median longitudinal dent (Figs 35H, I, 36F).

#### Coloration

(Fig. 35A, B, I, K). General body coloration brown-black except scape, pedicel, clypeus, mandibles, apex of propleuron, and dorsal furrow of pronotum yellow-brown; last five distal antennal flagellomeres yellow-brown, remaining flagellomeres brown on both sides; labrum, glossa, maxillary and labial palps yellow. Eyes and ocelli silver. Fore and middle legs yellow, except fore claw brown; hind legs yellow except dark brown coxae with apex yellow, apex of femora, both ends of tibiae, and tarsomeres brown. Petiole on T1 brown-black and sublateral areas yellow; T2 with median and wide adjacent areas brown-black, and lateral ends yellow; T3 with a medial brown area that coincides with the width of dark median and adjacent areas on T2, and lateral ends yellow; T4 and beyond brown; distally each tergum with a yellowish transparent band. In lateral view, T1–2 completely yellow; T3 and beyond yellow, but dorsally brown, extent of brown area increasing from proximal to distal, thus distal terga completely brown. S1–3 completely yellow; S4–5 yellow, medially brown; hypopygium completely brown.

#### Description.

**Head** (Fig. 35A–D, G). Head rounded with pubescence long and dense. Proximal three antennal flagellomeres longer than wide (0.24:0.08, 0.22:0.08, 0.23:0.08), distal antennal flagellomere subequal in length with penultimate (0.10:0.06, 0.09:0.06), antenna shorter than body (2.63, 2.93); antennal scrobes-frons shallow. Face flat or nearly so, with scattered finely punctate, and interspaces smooth and longitudinal median carina absent. Frons smooth. Temple wide, punctate and interspaces wavy. Inner margin of eyes diverging slightly at antennal sockets; in lateral view, eye anteriorly convex and posteriorly straight. POL shorter than OOL (0.11, 0.15). Malar suture present. Median area between lateral ocelli without depression. Vertex laterally rounded and dorsally wide.

**Mesosoma** (Fig. 35A, H, I, L). Mesosoma dorsoventrally convex. Mesoscutum proximally convex and distally flat, with punctation distinct throughout and interspaces wavy/lacunose. Scutellum shape triangular, apex sloped and fused with BS, scutellar punctation distinct peripherally and absent centrally, scutellum in profile flat and on same plane as mesoscutum, phragma of the scutellum partially exposed; BS only very partially overlapping the MPM; ATS demilune with short stubs delineating the area; dorsal ATS groove with semicircular/parallel carinae. Transscutal articulation with small and heterogeneous foveae; area just behind transscutal articulation smooth, shiny, and depressed centrally. Metanotum with BM wider than PFM (clearly differentiated); MPM circular without median longitudinal carina; AFM with a small lobe and not as well delineated as PFM; PFM thick and smooth; ATM proximally with semircular/undulate carina and distally smooth. Propodeum without median longitudinal carina, finely sculptured, proximal half curved; distal edge of propodeum with a flange at each side and without stubs; propodeal spiracle without distal carina; nucha surrounded by very short radiating carinae. Pronotum with a distinct dorsal furrow, dorsally with a well-defined smooth band; central area of pronotum and dorsal furrow smooth, but ventral furrow with short parallel carinae. Propleuron finely sculptured only ventrally and dorsally without a carina. Metasternum flat or nearly so. Contour of mesopleuron convex; precoxal groove smooth, shiny and shallow, but visible; epicnemial ridge elongated more fusiform (tapering at both ends).

**Legs** (Fig. 35A, E). Ventral margin of fore telotarsus entire, but with a tiny curved seta, fore telotarsus proximally narrow and distally wide, and longer than fourth tarsomere (0.14, 0.06). Hind coxa with dorsal half sparsely punctate, ventral half densely punctate, and dorsal outer depression absent. Inner spur of hind tibia longer than outer spur (0.25, 0.19); entire surface of hind tibia with dense strong spines clearly differentiated by color and length. Hind telotarsus longer than fourth tarsomere (0.15, 0.12).

**Wings** (Fig. 35N, O). Fore wing with r vein straight; 2RS vein straight; r and 2RS veins forming an angle at their junction and outer side of junction forming a slight stub; 2M vein slightly curved/swollen; distally fore wing [where spectral veins are] with microtrichiae more densely concentrated than the rest of the wing; anal cell 1/3 proximally lacking microtrichiae; subbasal cell with microtrichiae virtually throughout; veins 2CUa and 2CUb completely spectral; vein 2 cu-a present as spectral vein, sometimes difficult to see; vein 2-1A proximally tubular and distally spectral, although sometimes difficult to see; tubular vein 1 cu-a straight, incomplete/broken and not reaching the edge of 1-1A vein. Hind wing with annal lobe very narrow, subdistally and subproximally evenly convex, and setae evenly scattered in the margin.

**Metasoma** (Fig. 35A, F, J, K, M). Metasoma laterally compressed. Petiole on T1 finely sculptured only laterally, evenly narrowing distally (length 0.43, maximum width 0.25, minimum width 0.11), petiole with scattered pubescence on distal half and only laterally. Lateral grooves delimiting the median area on T2 clearly defined and reaching the distal edge of T2 (length median area 0.18, length T2 0.18), edges of median area obscured by weak longitudinal stripes, median area broader than long (length 0.18, maximum width 0.25, minimum width 0.11); T2 with scarce pubescence throughout. T3 longer than T2 (0.24, 0.18) and with scattered pubescence throughout. Pubescence on hypopygium dense.

**Cocoons.** White bud-like cocoons with body ridge-shaped and silk fibers evenly smooth. Cocoons adhered to the leaf substrate or in litter or soil.

#### Male

(Fig. 36A–L). The body coloration is lighter in males than in females.

#### Etymology.

Robert (Bob) A. Wharton is an American entomologist whose research is focused upon the evolution of behavior and life histories in parasitoids, especially Opiinae and Alysiinae (Braconidae). He is an emeritus professor at the Department of Entomology, Texas A&M University, College Station, TX, USA.

#### Distribution.

Parasitized caterpillars were collected in Costa Rica, ACG, Sector Pitilla (Sendero Mismo), Sector Rincón Rain Forest (Sendero Rincón), and Sector San Cristóbal (Sendero Perdido), during February 2003, October 2004, and February 2005 at 430 m, 620 m, and 680 m in rain forests.

#### Biology.

The lifestyle of this parasitoid species is solitary/gregarious.

#### Host.

*Ochrodotamarina* Schaus (Erebidae: Arctiinae) feeding on *Ocotealeucoxylon* (Lauraceae); *Symphlebiatessellata* (Schaus) (Erebidae: Arctiinae) feeding on *Pouteriaviridis* (Sapotaceae); *Perigacluacina* Druce (Saturniidae: Hemileucinae) feeding on *Carapaguianensis* (Meliaceae). Caterpillars were collected in second and third instar.

### Glyptapanteles boharti

Taxon classificationAnimaliaHymenopteraBraconidae

Arias-Penna, sp. nov.

http://zoobank.org/4D4FB863-CF09-4079-9616-50EB95862EC2

Figs 37 , 38

#### Female.

Body length 2.63 mm, antenna length 2.83 mm, fore wing 2.78 mm.

#### Type material.

**Holotype**: COSTA RICA • 1♀; 01-SRNP-21185, DHJPAR0000016; Área de Conservación Guanacaste, Guanacaste, Sector Cacao, Estación Cacao; cloud forest; 1,150 m; 10.92691, -85.46822; 26.x.2001; Harry Ramirez leg.; caterpillar collected in fourth instar; elongate white cocoons with the exploded star at each end, each cocoon loose and only very lightly adhered to the larval cuticle, cocoons formed on 26.x.2001; adult parasitoids emerged on 10.xi.2001; (CNC). **Paratypes.** • 37 (3♀, 4♂) (28♀, 2♂); 01-SRNP-21185, DHJPAR0000016; same data as for holotype; (CNC).

#### Other material.

**Reared material.** COSTA RICA: *Área de Conservación Guanacaste*, *Guanacaste*, *Sector Cacao*, *Sendero Toma Agua*: • 31 (4♀, 3♂) (18♀, 6♂); 98-SRNP-3354, DHJPAR0000109; cloud forest; 1,140 m; 10.92847, -85.46680; 14.viii.1998; Mariano Pereira leg.; caterpillar collected in fifth instar; beige white ridged bud-like cocoons adhered to the leaf substrate; adult parasitoids emerged on 26.viii.1988. • 9 (3♀, 0♂) (9♀, 0♂); 99-SRNP-17047, DHJPAR0001514; same data as for preceding except: 13.xi.1999; elongate white separate cocoons glued lightly to the leaf, with conspicuous tail fins at each end, cocoons adhered to the leaf substrate; adult parasitoids emerged on 28.xi.1999.

*Área de Conservación Guanacaste*, *Guanacaste*, *Sector Cacao*, *Sendero Nayo*: • 2 (1♀, 0♂) (0♀, 1♂); 03-SRNP-22411, DHJPAR0000041; cloud forest; 1,090 m; 10.92446, -85.46953; 23viii.2003; Harry Ramirez leg.; caterpillar collected in fifth instar; elongate white bud-like cocoons only very lightly adhered to the leaf substrate, cocoons formed on 25.viii.2003; adult parasitoids emerged on 04.ix.2003.

*Área de Conservación Guanacaste*, *Guanacaste*, *Sector Cacao*, *Sendero Circular*: • 32 (5♀, 5♂) (20♀, 2♂); 05-SRNP-35705, DHJPAR0004228; cloud forest; 1,185 m; 10.92714, -85.46683; 07.vii.2005; Dunia Garcia leg.; caterpillar collected in fifth instar; single, scattered and ridged white bud-like cocoons adhered to the leaf substrate; adult parasitoids emerged on 19.vii.2005.

*Área de Conservación Guanacaste*, *Guanacaste*, *Sector Cacao*, *Estación Cacao*: • 29 (4♀, 4♂) (20♀, 1♂); 06-SRNP-36826, DHJPAR0012677; cloud forest; 1,150 m; 10.92691, -85.46822; 21.xi.2006; Harry Ramirez leg.; caterpillar collected in fourth instar; white bud-like cocoons in litter or soil, formed on 29.xi.2006; adult parasitoids emerged on 11-12.xii.2006. • 14 (5♀, 5♂) (4♀, 0♂); 09-SRNP-36318, DHJPAR0039957; same data as for preceding except: 17.vi.2009; Dunia Garcia leg.; caterpillar collected in fifth instar; cocoons formed on 26.vi.2009; adult parasitoids emerged on 02.vii.2009. • 33 (5♀, 5♂) (20♀, 3♂); 10-SRNP-35361, DHJPAR0040397; same data as for preceding except: 06.vi.2010; caterpillar collected in fifth instar; cocoons formed on 19.vi.2010; adult parasitoids emerged on 29.vi.2010. • 8 (3♀, 2♂) (3♀, 0♂); 10-SRNP-35697, DHJPAR0040388; same data as for preceding except: 11.viii.2010; cocoons formed on 19.viii.2010; adult parasitoids emerged on 27.viii.2010.

*Área de Conservación Guanacaste*, *Guanacaste*, *Sector Cacao*, *Sendero Segundo*: • 20 (5♀, 0♂) (15♀, 0♂); 07-SRNP-36296, DHJPAR0020266; cloud forest; 1,180 m; 10.92679, -85.45332; 30.vii.2007; Dunia Garcia leg.; caterpillar collected in fifth instar; white bud-like cocoons in litter or soil formed on 08.viii.2007; adult parasitoids emerged on 14.viii.2007.

*Área de Conservación Guanacaste*, *Guanacaste*, *Sector Pitilla*, *Estación Pitilla*: • 1 (0♀, 0♂) (1♀, 0♂); 06-SRNP-31943, DHJPAR0005107; rain forest; 675 m; 10.98931, -85.42581; 19.v.2006; Calixto Moraga leg.; caterpillar collected in second instar; single gray cocoon formed on 25.v.2006 and adhered to the leaf substrate; adult parasitoids emerged on 01.vi.2006.

#### Diagnosis.

**Figure 37.**
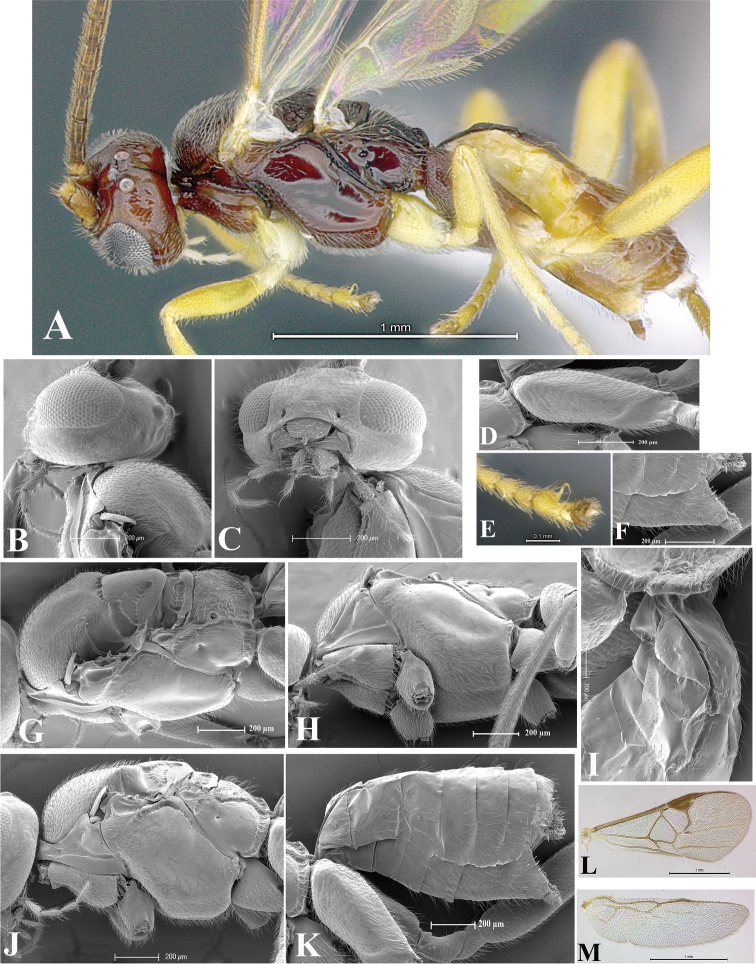
*Glyptapantelesboharti* sp. nov. female 01-SRNP-21185 DHJPAR0000016 **A** Habitus **B, C** Head **B** Laterodorsal view **C** ventrofrontal view **D** Hind coxa, lateral view **E** Fore tarsomeres 2–5 **F** Genitalia: hypopygium, ovipositor, ovipositor sheaths, lateral view **G, H, J** Mesosoma **G** Dorsolateral view **H** Ventrolateral view **J** Lateral view **I**T1–2, dorsolateral view **K** Metasoma, lateral view **L, M** Wings **L** Fore **M** Hind.

**Figure 38.**
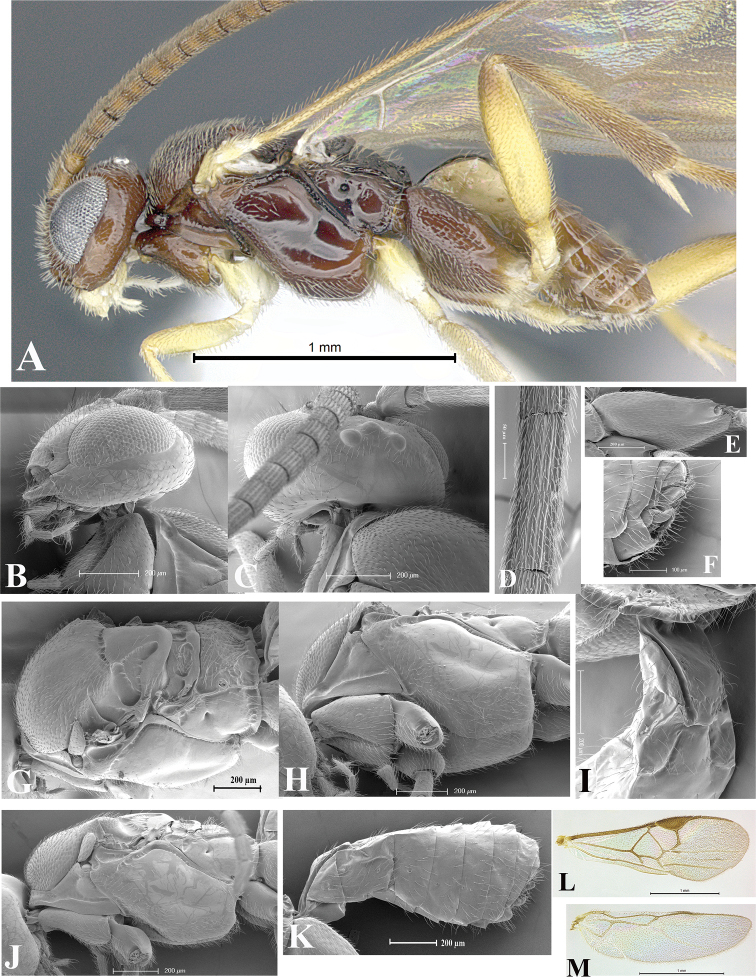
*Glyptapantelesboharti* sp. nov. male 01-SRNP-21185 DHJPAR0000016 **A** Habitus **B, C** Head **B** Lateral view **C** Dorsal view **D** Flagellomeres **E** Hind coxa, lateral view **F** Genitalia: Parameres, lateral view **G, H, J** Mesosoma **G** Dorsolateral view **H** Ventrolateral view **J** Lateral view **I**T1–2, dorsolateral view **K** Metasoma, lateral view **L, M** Wings **L** Fore **M** Hind.

Medioanterior pit of metanotum circular without median longitudinal carina (Figs 37G, 38G), edges of median area on T2 with little sculpture (Figs 37I, 38I), scutellar punctation distinct peripherally, absent centrally (Figs 37G, 38G), in lateral view, metasoma laterally compressed (Figs 37A, 38A), dorsal outer depression on hind coxa absent (Fig. 37K), and fore wing with r vein slightly curved or curved, outer side of junction of r and 2RS veins forming a stub (Figs 37L, 38L).

#### Coloration

(Fig. 37A). General body coloration dark reddish brown except labrum and mandibles yellow-brown; glossa, maxillary and labial palps, and tegulae yellow. Eyes gray/silver and ocelli silver. Fore and middle legs yellow; hind legs yellow except coxae dark brown with apex yellow-brown, tibiae and tarsomeres brown. Petiole on T1 dark brown with contours darkened and sublateral areas yellow; T2 with median and narrow adjacent areas brown, and lateral ends yellow; T3 and beyond completely brown; distally each tergum with a narrow whitish transparent band. In lateral view T1–2 completely yellow; T3–4 yellow, but dorsally light brown; T5 and beyond completely brown. S–-4 completely yellow; penultimate sternum and hypopygium yellow-brown.

#### Description.

**Head** (Fig. 37A–C). Head triangular with pubescence long and dense. Proximal three antennal flagellomeres longer than wide (0.25:0.06, 0.24:0.06, 0.24:0.06), distal antennal flagellomere longer than penultimate (0.15:0.05, 0.11:0.05), antenna longer than body (2.83, 2.63); antennal scrobes-frons shallow. Face flat or nearly so, finely punctate-lacunose, interspaces smooth and longitudinal median carina absent. Frons smooth. Temple wide, punctate and interspaces clearly smooth. Inner margin of eyes diverging slightly at antennal sockets; in lateral view, eye anteriorly convex and posteriorly straight. POL shorter than OOL (0.10, 0.13). Malar suture present. Median area between lateral ocelli without depression. Vertex laterally rounded and dorsally wide.

**Mesosoma** (Fig. 37A, G, H, J). Mesosoma dorsoventrally convex. Distal 1/3 of mesoscutum with lateral margin slightly dented, punctation distinct throughout and interspaces smooth. Scutellum shape triangular, apex sloped and fused with BS, scutellar punctation distinct peripherally, absent centrally, in profile scutellum flat and on same plane as mesoscutum, phragma of the scutellum partially exposed; BS only very partially overlapping the MPM; ATS demilune with short stubs delineating the area; dorsal ATS groove with carinae only proximally. Transscutal articulation with small and heterogeneous foveae; area just behind transscutal articulation smooth, shiny, and depressed centrally. Metanotum with BM wider than PFM (clearly differentiated); MPM circular without median longitudinal carina; AFM with a small lobe and not as well delineated as PFM; PFM thick and smooth; ATM proximally with semircular/undulate carina and distally smooth. Propodeum without median longitudinal carina, proximal half weakly curved with medium-sized sculpture and distal half with fine sculpture and with a shallow dent at each side of nucha; distal edge of propodeum with a flange at each side and short stubs; propodeal spiracle without distal carina; nucha surrounded by very short radiating carinae. Pronotum with a distinct dorsal furrow, dorsally with a well-defined smooth band; central area of pronotum and dorsal furrow smooth, but ventral furrow with short parallel carinae. Propleuron finely sculptured only ventrally and dorsally without a carina. Metasternum flat or nearly so. Contour of mesopleuron convex; precoxal groove smooth, shiny and shallow, but visible; epicnemial ridge convex, teardrop-shaped.

**Legs** (Fig. 37A, D, E). Ventral margin of fore telotarsus excavated with a conspicuous curved seta over this excavation, fore telotarsus proximally narrow and distally wide, and longer than fourth tarsomere (0.15, 0.07). Hind coxa finely punctate throughout, and dorsal outer depression absent. Inner spur of hind tibia longer than outer spur (0.21, 0.17), entire surface of hind tibia with dense strong spines clearly differentiated by color and length. Hind telotarsus longer than fourth tarsomere (0.20, 0.11).

**Wings** (Fig. 37L, M). Fore wing with r vein slightly curved; 2RS vein straight; r and 2RS veins forming a weak, even curve at their junction and outer side of junction forming a slight stub; 2M vein slightly curved/swollen; distally fore wing [where spectral veins are] with microtrichiae more densely concentrated than the rest of the wing; anal cell 1/3 proximally lacking microtrichiae; subbasal cell with a small smooth area; veins 2CUa and 2CUb completely spectral; vein 2 cu-a present as spectral vein, sometimes difficult to see; vein 2-1A proximally tubular and distally spectral, although sometimes difficult to see; tubular vein 1 cu-a curved, complete, and touching the edge of 1-1A vein. Hind wing with vannal lobe narrow, subdistally evenly convex, subproximally straightened, and setae evenly scattered in the margin.

**Metasoma** (Fig. 37A, F, I, K). Metasoma laterally compressed. Petiole on T1 finely sculptured only laterally, petiole evenly narrowing distally, apex truncate (length 0.35, maximum width 0.20, minimum width 0.08), petiole with scattered pubescence on distal half. Lateral grooves delimiting the median area on T2 clearly defined and reaching the distal edge of T2 (length median area 0.16, length T2 0.16), edges of median area with little sculpture, median area broader than long (length 0.16, maximum width 0.19, minimum width 0.07); T2 with scattered pubescence throughout. T3 longer than T2 (0.22, 0.16) and with scattered pubescence throughout. Pubescence on hypopygium dense.

**Cocoons** (Fig. 4K). White or gray bud-like cocoon with body ridge-shaped and silk fibers evenly smooth. Each cocoon is loose and only very lightly adhered to the larval cuticle or very lightly adhered to the leaf substrate or in litter or soil.

#### Comments.

The reddish brown body coloration is characteristic for this species.

#### Male

(Fig. 38A–M). The coloration on metasoma is slightly darker than in females.

#### Etymology.

Richard (Dick) M. Bohart (Sept. 28, 1913-Feb. 1, 2007) was a professor at the University of California (UC), Davis, CA, USA. He was one of the world’s leading experts on wasps and mosquitoes during a 32-year career at the UC, Davis. He identified more than 1 million of these insects, many of which are in the Bohart Museum of Entomology (UC, Davis), and published 230 articles, as well as six books on wasps and mosquitoes.

#### Distribution.

The parasitized caterpillars were collected in Costa Rica, ACG, Sector Cacao (Estación Cacao, Sendero Circular, Sendero Nayo, Sendero Segundo, and Sendero Toma Agua), and Sector Pitilla (Estación Pitilla), during August 1998, November 1999, October 2001, August 2003, July 2005, May and November 2006, August 2007, June 2009, and June and August 2010 at 675 m, 1,090 m, 1,140 m, 1,150 m, 1,180 m, and 1,185 m in rain and cloud forests.

#### Biology.

The lifestyle of this parasitoid species is solitary/gregarious.

#### Host.

*Anomisluridula* Guenée (Noctuidae: Catocalinae) (Fig. 4K) feeding on *Hampeaappendiculata* (Malvaceae). Caterpillars were collected in second, fourth and fifth instar.

### Glyptapanteles brianestjaquesae

Taxon classificationAnimaliaHymenopteraBraconidae

Arias-Penna, sp. nov.

http://zoobank.org/161BF4AC-B650-48D4-B54A-A9A5B75B8BF2

Figs 39 , 40

#### Female.

Body length 3.03 mm, antenna length 2.98 mm, fore wing length 2.63 mm.

#### Type material.

**Holotype**: COSTA RICA •1♀; 02-SRNP-2950, DHJPAR0000261; Área de Conservación Guanacaste, Alajuela, Sector El Ensayo, Camino Ensayo; rain forest; 500 m; 10.95152, -85.37388; 01.v.2002; Carolina Cano leg.; caterpillar collected in third instar; white single bud-like cocoons adhered to the leaf substrate; adult parasitoids emerged on 10.v.2002; (CNC). **Paratypes.** • 18 (3♀, 3♂) (8♀, 4♂); 02-SRNP-2950, DHJPAR0000261; same data as for holotype; (CNC).

#### Other material.

**Reared material.** COSTA RICA: *Área de Conservación Guanacaste*, *Alajuela*, *Sector Rincón Rain Forest*, *Sendero Rincón*: • 10 (3♀, 3♂) (2♀, 2♂); 02-SRNP-7651, DHJPAR0000263; 430 m; 10.8962, -85.27769; 04.vii.2002; Freyci Vargas leg.; caterpillar collected in fourth instar; white single bud-like cocoons adhered to the leaf substrate and formed on 06.vii.2002; adult parasitoids emerged on 14.vii.2002.

*Área de Conservación Guanacaste*, *Alajuela*, *Sector San Cristóbal*, *Río Blanco Abajo*: 14 (3♀, 3♂) (8♀, 0♂); 07-SRNP-2053, DHJPAR0030994; rain forest; 500 m; 10.90037, -85.37254; 21.v.2007; Anabelle Cordoba leg.; caterpillar collected in fourth instar; white bud-like cocoons adhered very lightly to the leaf and formed on 14.v.2007; adult parasitoids emerged on 21.v.2007.

#### Diagnosis.

**Figure 39.**
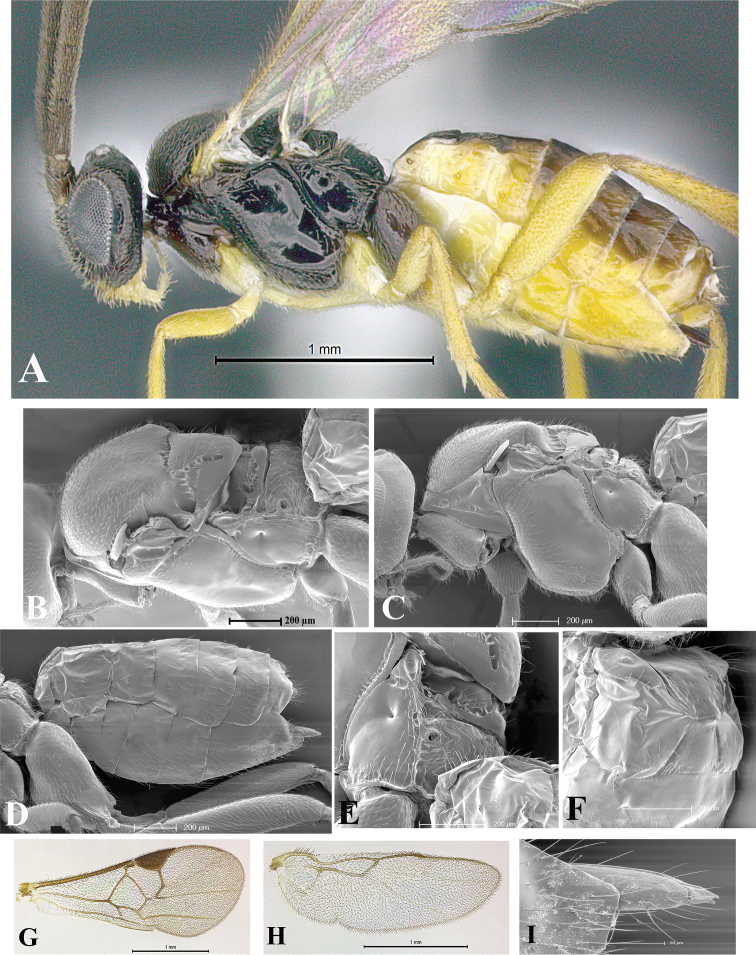
*Glyptapantelesbrianestjaquesae* sp. nov. female 02-SRNP-2950 DHJPAR0000261 **A** Habitus **B, C** Mesosoma, **B** dorsolateral view **C** Lateral view **D** Metasoma, lateral view **E** Metanotum, propodeum, laterodorsal view **F**T1–3, dorsolateral view **G, H** Wings **G** Fore **H** Hind **I** Genitalia: hypopygium, ovipositor, ovipositor sheaths, lateral view.

**Figure 40.**
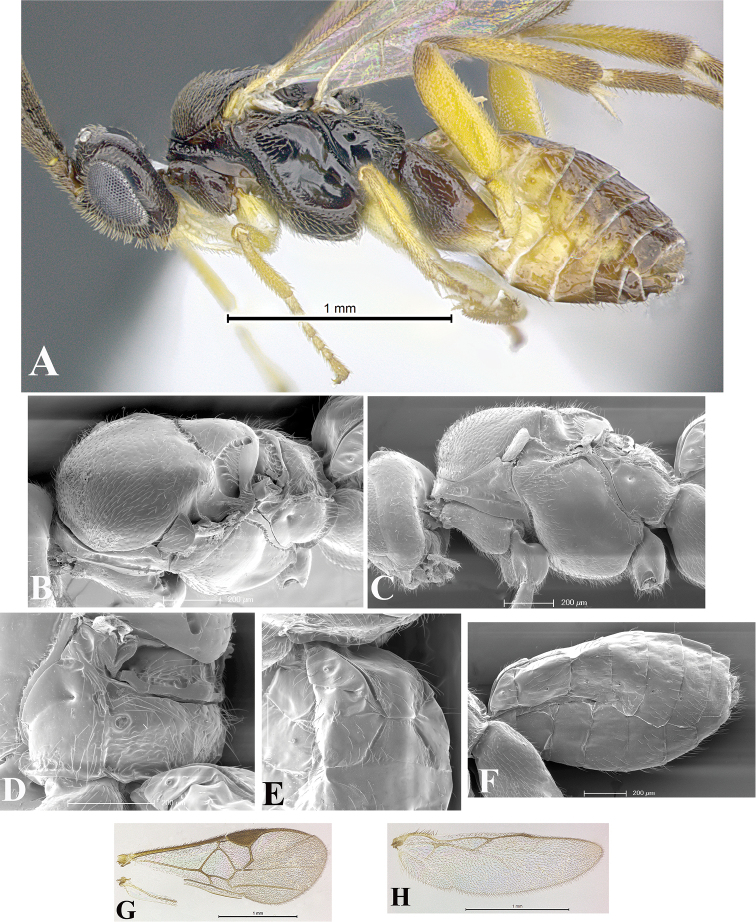
*Glyptapantelesbrianestjaquesae* sp. nov. male 02-SRNP-2950 DHJPAR0000261 **A** Habitus **B, C** Mesosoma, **B** dorsolateral view **C** Lateral view **D** Metanotum, propodeum, laterodorsal view **E**T1–3, dorsolateral view **F** Metasoma, lateral view **G, H** Wings **G** Fore **H** Hind.

Inner margin of eyes straight throughout, medioanterior pit of metanotum circular without median longitudinal carina (Fig. 40D), mesoscutum punctation distinct throughout (Figs 39B, 40B), phragma of the scutellum partially exposed (Figs 39E, 40D), petiole on T1 virtually parallel-sided over most of length but narrowing over distal 1/3 and finely sculptured (Figs 39F, 40E), propodeum without a median longitudinal dent (Fig. 40D), lateral grooves delimiting the median area on T2 distally losing definition on T2 (Figs 39F, 40E), and fore wing with r vein straight, outer side of junction of r and 2RS veins forming a stub (Figs 39G, 40G).

#### Coloration

(Fig. 39A). General body coloration brown-black although some areas on body are light brown/reddish as propleuron, parts of both dorsal and ventral furrows of pronotum, epicnemial ridge, distal-ventral corner of mesopleuron, distal corners of mesoscutum, lunules and lateral ends of dorsal ATS groove; proximal part of scape and apex of pedicel yellow-brown; labrum, mandibles, glossa, maxillary and labial palps, and tegulae yellow. Eyes gray/black and ocelli silver. Fore and middle legs yellow except fore claws brown; hind legs yellow except 3/4 coxae brown/reddish with 1/4 distal yellow (in the inner side, the yellow coloration expanded until covers the distal half), femora with a tiny brown spot at the apex, apex of tibiae brown and tarsomeres brown although basitarsus with a proximal yellow ring. Petiole on T1 brown-black and sublateral areas yellow; T2 with median and adjacent areas brown, limits of adjacent area smeary, and lateral ends yellow-brown; T3 medially brown without defined shape, remaining area on T3 yellow-brown; T4 and beyond yellow-brown/brown; distally each tergum with a narrow yellowish transparent band. In lateral view, T1–3 yellow with little spots brown; T4 and beyond yellow, but dorsally light brown, extent of brown area remains constant in each tergum. All sterna yellow; ovipositor sheaths brown.

#### Description.

**Head** (Fig. 39A). Head rounded with pubescence long and dense. Proximal three antennal flagellomeres longer than wide (0.25:0.08, 0.24:0.08, 0.24:0.08), distal antennal flagellomere longer than penultimate (0.13:0.06, 0.11:0.06), antenna shorter than body (2.98, 3.03); antennal scrobes-frons shallow. Face shape flat or nearly so, finely punctate-lacunose, interspaces smooth, and longitudinal median carina present. Frons smooth. Temple wide, punctuate, and interspaces clearly smooth. Inner margin of eyes straight throughout; in lateral view, eye anteriorly convex and posteriorly straight. POL shorter than OOL (0.07, 0.11). Malar suture present. Median area between lateral ocelli without depression. Vertex laterally rounded and dorsally wide.

**Mesosoma** (Fig. 39A–C, E). Mesosoma dorsoventrally convex. Mesoscutum proximally convex and distally flat, punctation distinct throughout and interspaces smooth. Scutellum triangular, apex sloped and fused with BS, scutellar punctation distinct throughout, in profile scutellum flat and on same plane as mesoscutum, phragma of the scutellum partially exposed; BS only very partially overlapping the MPM; ATS demilune inner side with a row of foveae; dorsal ATS groove with carinae only proximally. Transscutal articulation with small and heterogeneous foveae, area just behind transscutal articulation with same kind of sculpture as mesoscutum and nearly at the same level as mesoscutum (flat) and depressed centrally. Metanotum with BM wider than PFM (clearly differentiated); MPM circular without median longitudinal carina; AFM with a small lobe and not as well delineated as PFM; PFM thick and smooth; ATM proximally with semircular/undulate carina and distally smooth. Propodeum without median longitudinal carina, proximal half weakly curved with medium-sized sculpture and distal half rugose with a shallow dent at each side of nucha; distal edge of propodeum with a flange at each side and without stubs; propodeal spiracle distally framed by faintly concave/wavy carina; nucha surrounded by at most three short carinae. Pronotum with a distinct dorsal furrow, dorsally with a well-defined smooth band; central area of pronotum and dorsal furrow smooth, but ventral furrow with short parallel carinae. Propleuron with fine punctations throughout and dorsally without a carina. Metasternum flat or nearly so. Contour of mesopleuron convex; precoxal groove smooth, shiny and shallow, but visible; epicnemial ridge elongated more fusiform (tapering at both ends).

**Legs** (Fig. 39A). Ventral margin of fore telotarsus entire, but with a tiny curved seta, fore telotarsus proximally narrow and distally wide, and longer than fourth tarsomere (0.11, 0.06). Medially hind coxa smooth, dorsally with scattered punctation and ventrally with dense punctation, dorsal outer depression present. Inner spur of hind tibia longer than outer spur (0.22, 0.15); entire surface of hind tibia with dense strong spines clearly differentiated by color and length. Hind telotarsus as equal in length as fourth tarsomere (0.13, 0.12).

**Wings** (Fig. 39G, H). Fore wing with r vein straight; 2RS vein straight; r and 2RS veins forming an angle at their junction and outer side of junction forming a slight stub; 2M vein slightly curved/swollen; distally fore wing [where spectral veins are] with microtrichiae more densely concentrated than the rest of the wing; anal cell 1/3 proximally lacking microtrichiae; subbasal cell with microtrichiae virtually throughout; veins 2CUa and 2CUb completely spectral; vein 2 cu-a present as spectral vein, sometimes difficult to see; vein 2-1A proximally tubular and distally spectral, although sometimes difficult to see; tubular vein 1 cu-a straight, incomplete/broken and not reaching the edge of 1-1A vein. Hind wing with vannal lobe very narrow, subdistally and subproximally evenly convex, and setae evenly scattered in the margin.

**Metasoma** (Fig. 38A, D, F, I). Metasoma laterally compressed. Petiole on T1 finely sculptured on distal half, virtually parallel-sided over most of length, but narrowing over distal 1/3 and apex truncate (length 0.39, maximum width 0.23, minimum width 0.11), petiole with scattered pubescence on distal half. Lateral grooves delimiting the median area on T2 clearly defined and reaching the distal edge of T2 (length median area 0.15, length T2 0.15), edges of median area obscured by weak longitudinal stripes, median area broader than long (length 0.15, maximum width 0.25, minimum width 0.10); T2 with scarce pubescence throughout. T3 longer than T2 (0.22, 0.15) and with scattered pubescence throughout. Pubescence on hypopygium dense.

**Cocoons.** White or beige bud-like cocoons with body ridge-shaped and silk fibers evenly smooth. Single cocoons adhered very lightly to the leaf substrate.

#### Male

(Fig. 40A–H). The sterna are darker in coloration than females.

#### Etymology.

Briane St. Jaques is collections data manager at the Biodiversity Institute of Ontario (BIO), University of Guelph, Ontario, CA, since 2011.

#### Distribution.

Parasitized caterpillars were collected in Costa Rica, ACG, Sector El Ensayo (Camino Ensayo), Sector Rincón Rain Forest (Sendero Rincón), and Sector San Cristóbal (Río Blanco Abajo), during May and July 2002, and May 2007 at 430 m and 500 m in rain forest.

#### Biology.

The lifestyle of this parasitoid species is gregarious.

#### Host.

*Drugeramorona* Druce (Notodontidae: Heterocampinae) feeding on *Ossaeamicrantha* and *Conostegiamicrantha* (Melastomataceae). *Rhudadifficilis* Schaus (Notodontidae: Heterocampinae) feeding on *Conostegiamicrantha* (Melastomataceae). Caterpillars were collected in third and fourth instar.

### Glyptapanteles carinachicaizae

Taxon classificationAnimaliaHymenopteraBraconidae

Arias-Penna, sp. nov.

http://zoobank.org/9B810151-95A5-496B-8563-CD180E57A362

Fig. 41

#### Female.

Body length 2.63 mm, antenna length 3.53 mm, fore wing length 3.08 mm.

#### Type material.

**Holotype**: ECUADOR • 1♀; EC-37323, YY-A168; Napo, Yanayacu Biological Station, Sendero Macuculoma, Plot 423; cloud forest; 2,108 m; -0.597778, -77.8875; 28.ii.2009; Wilmer Simbaña leg.; caterpillar collected in second instar; cocoon formed on 16.iii.2009; adult parasitoid emerged on 01.iv.2009; (PUCE).

#### Diagnosis.

**Figure 41.**
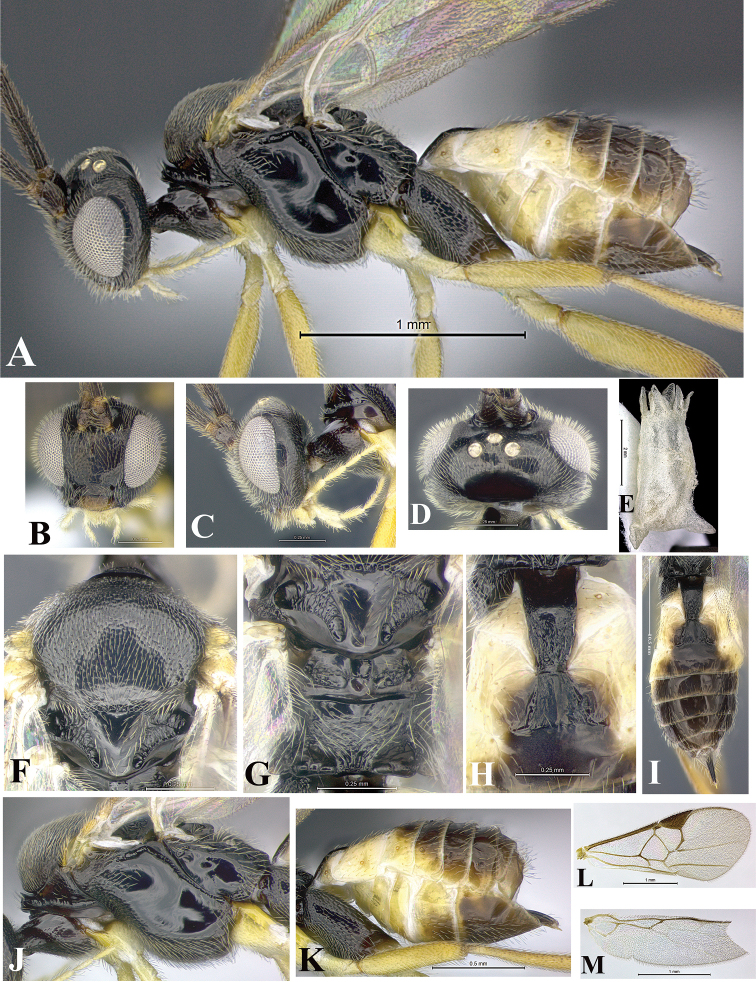
*Glyptapantelescarinachicaizae* sp. nov. female EC-37323 YY-A168 **A** Habitus **B, D** Head **B** Frontal view **D** Dorsal view **C** Head, pronotum, propleuron, lateral view **E** Cocoon **F** Mesonotum, dorsal view **G** Scutellum, metanotum, propodeum, dorsal view **H**T1–2, dorsal view **I, K** Metasoma **I** Dorsal view **K** Lateral view **J** Mesosoma, lateral view **L, M** Wings **L** Fore **M** Hind.

Vertex in lateral view rounded (Fig. 41C), dorsal carina delimiting a dorsal furrow on propleuron absent (Fig. 41A, J), inner margin of eyes straight throughout (Fig. 41B), fore wing with vein 2-1A tubular throughout, r vein curved, outer side of junction of r and 2RS veins forming a distinct stub (Fig. 41L), median area on T2 broader than long, edges of median area on T2 obscured by weak longitudinal stripes (Fig. 41H, I), antenna longer than body, scutellum in profile flat and on same plane as mesoscutum, in dorsal view, proximal half of propodeum weakly curved (Fig. 41G), petiole on T1 evenly narrowing distally (Fig. 41H, I), and dorsal outer depression on hind coxa present (Fig. 41A, K).

#### Coloration

(Fig. 41A–M). General body coloration black except proximally scape, distally pedicel, labrum, and mandibles yellow-brown; all antennal flagellomeres brown on both sides; glossa, maxillary and labial palps, and tegulae yellow. Eyes silver and ocelli yellowish. Fore and middle legs yellow with brown claws; hind legs yellow, but a dot at the apex of femora, distal half of tibiae and tarsomeres brown. Petiole on T1 black and sublateral areas yellow; T2 with median and adjacent areas black, and lateral ends yellow/yellow-brown; T3 medially brown, but distal half with tints yellow/yellow-brown; T4 and beyond completely brown; distally each tergum with a narrow yellow transparent band. In lateral view, T1–2 completely yellow; T3–4 yellow, but dorsally brown; T5 and beyond completely brown. S1–3 completely yellow; S4 yellow, but medial with a brown spot; penultimate sternum yellow, medially brown; hypopygium completely brown.

#### Description.

**Head** (Fig. 41A–D). Head rounded with pubescence long and dense. Proximal three antennal flagellomeres longer than wide (0.24:0.07, 0.24:0.07, 0.24:0.07), distal antennal flagellomere longer than penultimate (0.15:0.06, 0.12:0.06), antenna longer than body (3.53, 2.63); antennal scrobes-frons shallow. Face flat or nearly so, with dense fine punctations, interspaces smooth, and longitudinal median carina present. Frons smooth. Temple wide, punctate and interspaces clearly smooth. Inner margin of eyes straight throughout; in lateral view, eye anteriorly convex and posteriorly straight. POL shorter than OOL (0.09, 0.12). Malar suture present. Median area between lateral ocelli without depression. Vertex laterally rounded and dorsally wide.

**Mesosoma** (Fig. 41A, F, G, J). Mesosoma dorsoventrally convex. Mesoscutum distal half with a central dent, punctation distinct throughout, and interspaces wavy/lacunose. Scutellum triangular, apex sloped and fused with BS, scutellar punctation scattered throughout, in profile scutellum flat and on same plane as mesoscutum, phragma of the scutellum partially exposed; BS only very partially overlapping the MPM; ATS demilune with quite a little complete parallel carinae; dorsal ATS groove with semicircular/parallel carinae. Transscutal articulation with small and heterogeneous foveae, area just behind transscutal articulation with a smooth and shiny sloped transverse strip. Metanotum with BM wider than PFM (clearly differentiated); MPM semicircular without median longitudinal carina; AFM with a small lobe and not as well delineated as PFM; PFM thick and smooth; ATM proximally with semircular/undulate carina and distally smooth. Propodeum with medium-sized sculpture without median longitudinal carina, proximal half weakly curved and distal half with a shallow dent at each side of nucha; distal edge of propodeum with a flange at each side and without stubs; propodeal spiracle distally framed by faintly concave/wavy carina; nucha surrounded by very short radiating carinae. Pronotum with a distinct dorsal furrow, dorsally with a well-defined smooth band; central area of pronotum smooth, but both dorsal and ventral furrows with short parallel carina. Propleuron with fine punctations throughout and dorsally without a carina. Metasternum convex. Contour of mesopleuron convex; precoxal groove smooth, shiny and shallow, but visible; epicnemial ridge elongated more fusiform (tapering at both ends).

**Legs.** Ventral margin of fore telotarsus entire, but with a tiny curved seta, fore telotarsus proximally narrow and distally wide, and longer than fourth tarsomere (0.14, 0.09). Hind coxa with medium-size punctate throughout, and dorsal outer depression present. Inner spur of hind tibia longer than outer spur (0.25, 0.19), entire surface of hind tibia with dense strong spines clearly differentiated by color and length. Hind telotarsus longer than fourth tarsomere (0.16, 0.12).

**Wings** (Fig. 41L, M). Fore wing with r vein slightly curved; 2RS vein straight; r and 2RS veins forming a weak, even curve at their junction and outer side of junction forming a slight stub; 2M vein slightly curved/swollen; distally fore wing [where spectral veins are] with microtrichiae more densely concentrated than the rest of the wing; anal cell 1/3 proximally lacking microtrichiae; subbasal cell with microtrichiae virtually throughout; veins 2CUa and 2CUb completely spectral; vein 2 cu-a present as spectral vein, sometimes difficult to see; vein 2-1A tubular throughout; tubular vein 1 cu-a straight, incomplete/broken, and not reaching the edge of 1-1A vein. Hind wing with vannal lobe narrow, subdistally straightened, subproximally straightened, and setae absent proximally, but scattered distally.

**Metasoma** (Fig. 41A, H, I, K). Metasoma cylindrical. Petiole on T1 finely sculptured on distal half, evenly narrowing distally (length 0.33, maximum width 0.27, minimum width 0.10), petiole with scattered pubescence on distal half. Lateral grooves delimiting the median area on T2 clearly defined and reaching the distal edge of T2 (length median area 0.18, length T2 0.18), edges of median area obscured by weak longitudinal stripes, median area broader than long (length 0.18, maximum width 0.20, minimum width 0.10); T2 with scattered pubescence throughout. T3 longer than T2 (0.21, 0.18) and with scattered pubescence throughout. Pubescence on hypopygium dense.

**Cocoon** (Fig. 41E). White bud-like cocoon with silk fibers evenly smooth.

#### Comments.

The female with body slender.

#### Male.

Unknown.

#### Etymology.

Carina Chicaiza is an Ecuadorian biologist who has helped in the identification of the food plants of the most common lepidopteran species collected at the Yanayacu Biological Station.

#### Distribution.

Parasitized caterpillar was collected in Ecuador, Napo, Yanayacu Biological Station (Sendero Macuculoma), during February 2009 at 2,108 m in cloud forest.

#### Biology.

The lifestyle of this parasitoid species is solitary.

#### Host.

Undetermined species of Noctuidae feeding on *Chusqueascandens* (Poaceae). Caterpillar was collected in second instar.

### Glyptapanteles carlhuffakeri

Taxon classificationAnimaliaHymenopteraBraconidae

Arias-Penna, sp. nov.

http://zoobank.org/3F37BBC6-3AD2-477C-B5E3-F4BB0D644CB7

Figs 42 , 43

#### Female.

Body length 2.37 mm, antenna length 2.88 mm, fore wing length 2.48 mm.

#### Type material.

**Holotype**: COSTA RICA • 1♀; 01-SRNP-11339, DHJPAR0000010; Área de Conservación Guanacaste, Guanacaste, Sector El Hacha, Sendero Bejuquilla; dry-rain intergrade forest; 280 m; 11.03004, -85.52699; 01.x.2001; Lucia Ríos leg.; caterpillar collected in fourth instar; white bud-like cocoons adhered to the leaf substrate; adult parasitoids emerged on 12.x.2001; (CNC). **Paratypes.** • 10 (3♀, 2♂) (5♀, 0♂); 01-SRNP-11339, DHJPAR0000010; same data as for holotype; (CNC).

#### Other material.

**Reared material**. COSTA RICA: *Área de Conservación Guanacaste*, *Guanacaste*, *Sector El Hacha*, *Sendero Bejuquilla*: • 3 (1♀, 1♂) (1♀, 0♂); 01-SRNP-11329, DHJPAR0001489; dry-rain intergrade forest; 280 m; 11.03004, -85.52699; 01.x.2001; Lucia Ríos leg.; caterpillar collected in fourth instar; white bud-like cocoons adhered to the leaf substrate and formed on 04.x.2001; adult parasitoids emerged on 14.x.2001. • 2 (1♀, 1♂) (0♀, 0♂); 01-SRNP-11333, DHJPAR0001499; same data as for preceding except: adult parasitoids emerged on 12.x.2001. • 1 (1♀, 0♂) (0♀, 0♂); 01-SRNP-11335, DHJPAR0001494; same data as for preceding except: cocoons formed on 02.x.2001; adult parasitoids emerged on 12.x.2001. • 2 (1♀, 0♂) (1♀, 0♂); 01-SRNP-11337, DHJPAR0000007; same data as for preceding except: cocoons formed on 04.x.2001; adult parasitoids emerged on 12.x.2001. • 2 (0♀, 1♂) (0♀, 1♂ broken); 01-SRNP-11338, DHJPAR0000009; same data as for preceding except: white elongate square bud-like cocoons; adult parasitoids emerged on 12.x.2001. • 1 (0♀, 1♂) (0♀, 0♂); 01-SRNP-11340, DHJPAR0000011; same data as for preceding except: cocoons white, elongate, square bud-like formed on 02.x.2001; adult parasitoids emerged on 12.x.2001. • 8 (3♀, 0♂) (5♀, 0♂); 01-SRNP-11346, DHJPAR0001498; same data as for preceding except: white bud-like cocoons adhered to the leaf substrate; adult parasitoids emerged on 17.x.2001.

*Área de Conservación Guanacaste*, *Guanacaste*, *Sector El Hacha*, *Sendero Tigre*: • 2 (1♀, 0♂) (1♀, 0♂); 01-SRNP-11361, DHJPAR0001491; dry-rain intergrade forest; 280 m; 11.03172, -85.52615; 05.x.2001; Lucia Ríos leg.; caterpillar collected in fourth instar; parasitoid cocoons adhered to the leaf substrate and formed on 09.x.2001; adult parasitoids emerged on 17.x.2001. • 4 (2♀, 0♂) (3♀, 0♂); 01-SRNP-11362, DHJPAR0001488; same data as for preceding except: white bud-like cocoons. • 6 (2♀, 2♂) (1♀, 1♂); 01-SRNP-11365, DHJPAR0001476; same data as for preceding except: white bud-like cocoons formed on 08.x.2001. • 2 (1♀, 0♂) (1♀, 0♂); 01-SRNP-11374, DHJPAR0001497; same data as for preceding except: white bud-like cocoon adhered to the leaf surface and formed on 08.x.2001. • 4 (2♀, 0♂) (2♀, 0♂); 01-SRNP-11376, DHJPAR0001513; white bud-like cocoons adhered to the leaf substrate. • 3 (1♀, 0♂) (2♀, 0♂); 01-SRNP-11377, DHJPAR0001512; same data as for preceding except: white bud-like cocoon which are loose rather than adhered to the leaf and formed on 08.x.2001. • 1 (0♀, 0♂) (0♀, 1♂); 01-SRNP-11378, DHJPAR0001492; same data as for preceding except: white hard cylindrical bud-like cocoons only very slightly adhered together and to leaf surface and formed on 08.x.2001; adult parasitoids emerged on 15.x.2001.

*Área de Conservación Guanacaste*, *Guanacaste*, *Sector Del Oro*, *Quebrada Raíz*: • 11 (3♀, 3♂) (3♀, 2♂); 06-SRNP-21947, DHJPAR0012015; dry-rain intergrade forest; 280 m; 11.02865, -85.48669; 29.vi.2006; Roster Moraga leg.; caterpillar collected in fourth instar; white bud-like cocoons in litter or soil and formed on 06.vii.2006; adult parasitoids emerged on 15.vii.2006. • 2 (0♀, 1♂) (0♀, 1♂); 06-SRNP-21942, DHJPAR0012020; same data as for preceding except: adult parasitoids emerged on 14.vii.2006.

#### Diagnosis.

**Figure 42.**
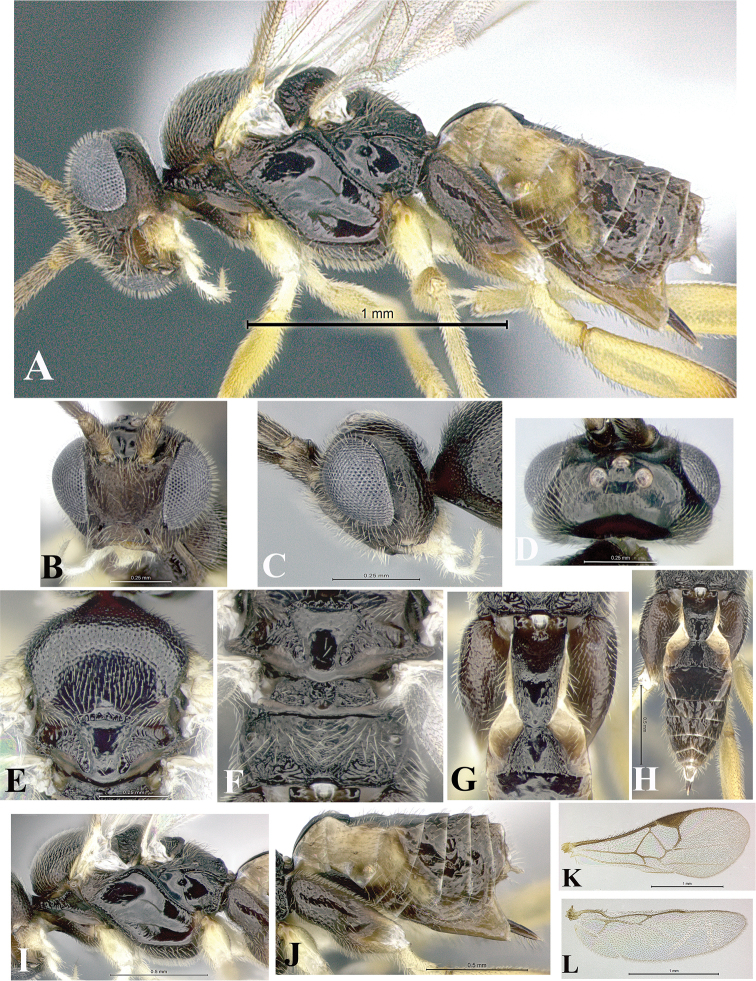
*Glyptapantelescarlhuffakeri* sp. nov. female 01-SRNP-11339 DHJPAR0000010 **A** Habitus **B–D** Head **B** Frontal view **C** Lateral view **D** Dorsal view **E** Mesonotum, dorsal view **F** Scutellum, metanotum, propodeum, dorsal view **G**T1–2, dorsal view **H, J** Metasoma **H** Dorsal view **J** Lateral view **I** Mesosoma, lateral view **K, L** Wings **K** Fore **L** Hind.

**Figure 43.**
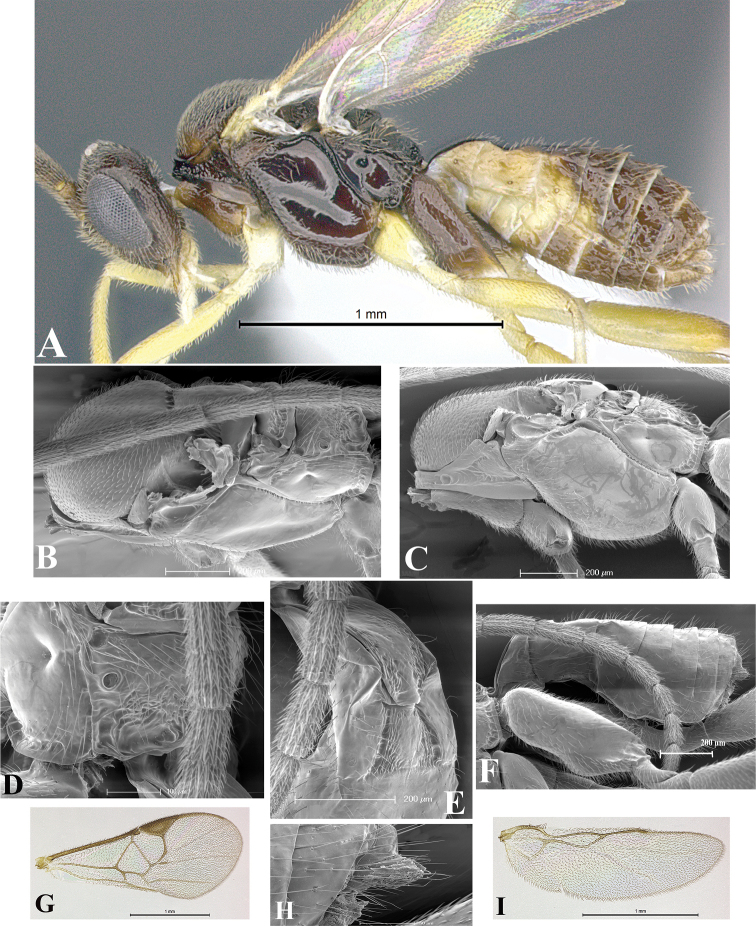
*Glyptapantelescarlhuffakeri* sp. nov. male 01-SRNP-11339 DHJPAR0000010 **A** Habitus **B, C** Mesonotum **B** Dorsolateral view **C** Lateral view **D** Propodeum dorsolateral view **E**T1–2, dorsolateral view **F** Metasoma, lateral view **G, I** Wings **G** Fore **I** Hind **H** Genitalia: parameres, lateral view.

In lateral view scutellum on same plane as mesoscutum (Figs 42A, 43C), T3 longer than T2 (Fig. 42H), longitudinal median carina on face present (Fig. 42B), antenna longer than body, distal antennal flagellomere subequal in length with penultimate, petiole on T1 virtually parallel-sided but narrowing over distal 1/3 (Figs 42G, 43E), surface of metasternum flat or nearly so, edges of median area on T2 obscured by weak longitudinal stripes (Figs 42G, 43E), dorsal outer depression on hind coxa absent (Figs 42J, 43F), and fore wing with r vein slightly curved, outer side of junction of r and 2RS veins forming a slight stub (Figs 42K, 43G).

#### Coloration

(Fig. 42A–L). General body coloration brown-black except proximal part of scape, pedicel, dorsal and ventral furrows of pronotum, and epicnemial ridge with yellow-brown tints; glossa, maxillary and labial palps, and tegulae yellow. Eyes dark gray and ocelli silver. Fore and middle legs yellow except claws brown; hind legs yellow except dark brown coxae with apex yellow, apex of femora, apex of tibiae and tarsomeres brown. Petiole on T1 dark brown and sublateral areas yellow-brown; T2 with median area dark brown and lateral ends light brown; T3 and beyond completely brown; distally each tergum with a narrow whitish/yellowish band. In lateral view, T1–2 completely yellow-brown; T3 yellow-brown, but dorsally brown; T4 and beyond brown. S1–3 completely yellow-brown; S4 and beyond brown.

#### Description.

**Head** (Fig. 42A–D). Head rounded with pubescence long and dense. Proximal three antennal flagellomeres longer than wide (0.24:0.06, 0.22:0.06, 0.22:0.06), distal antennal flagellomere subequal in length with penultimate (0.11:0.06, 0.10:0.06), antenna longer than body (2.88, 2.37); antennal scrobes-frons shallow. Face with scattered finely punctate, interspaces on face smooth with lateral depression and longitudinal median carina present. Frons smooth. Temple wide with punctate sculpture and interspaces with microsculpture. Inner margin of eyes diverging slightly at antennal sockets; in lateral view, eye anteriorly convex and posteriorly straight. POL shorter than OOL (0.09, 0.14). Malar suture present. Median area between lateral ocelli without depression. Vertex laterally rounded and dorsally wide.

**Mesosoma** (Fig. 42A, E, F, I). Mesosoma dorsoventrally convex. Distal 1/3 of mesoscutum with lateral margin slightly dented, punctation distinct throughout and interspaces wavy/lacunose. Scutellum triangular, apex sloped and fused with BS, scutellar punctation distinct throughout, scutellum in profile slightly convex, but on same plane as mesoscutum, phragma of the scutellum partially exposed; BS not overlapping the MPM; ATS demilune with a little, complete parallel carinae; dorsal ATS groove with semicircular/parallel carinae. Transscutal articulation with small and heterogeneous foveae; area just behind transscutal articulation with same kind of sculpture as mesoscutum and nearly at the same level as mesoscutum (flat). Metanotum with BM wider than PFM (clearly differentiated); MPM circular and bisected by a median longitudinal carina; AFM with a small lobe and not as well delineated as PFM; PFM thick and smooth; ATM with undulate carinae throughout. Propodeum without median longitudinal carina, proximal half weakly curved with medium-sized sculpture and distal half rugose with a shallow dent at each side of nucha; distal edge of propodeum with a flange at each side and without stubs; propodeal spiracle distally framed by faintly concave/wavy carina; nucha surrounded by very short radiating carinae. Pronotum with a distinct dorsal furrow, dorsally with a well-defined smooth band; central area of pronotum smooth, but both dorsal and ventral furrows with short parallel carinae. Propleuron with fine punctations throughout and dorsally without a carina. Metasternum flat or nearly so. Contour of mesopleuron straight/angulate or nearly so; precoxal groove smooth, shiny and shallow, but visible; epicnemial ridge elongated more fusiform (tapering at both ends).

**Legs.** Ventral margin of fore telotarsus entire, but with a tiny curved seta, fore telotarsus almost same width throughout and longer than fourth tarsomere (0.14, 0.09). Hind coxa finely punctate throughout, and dorsal outer depression absent. Inner spur of hind tibia longer that outer spur (0.20, 0.16); entire surface of hind tibia with dense strong spines clearly differentiated by color and length. Hind telotarsus as equal in length as fourth tarsomere (0.24, 0.23).

**Wings** (Fig. 42K, L). Fore wing with r vein slightly curved; 2RS vein straight; r and 2RS veins forming an angle at their junction and outer side of junction forming a slight stub; 2M vein slightly curved/swollen; distally fore wing [where spectral veins are] with microtrichiae more densely concentrated than the rest of the wing; anal cell 1/3 proximally lacking microtrichiae; subbasal cell with microtrichiae virtually throughout; veins 2CUa and 2CUb completely spectral; vein 2 cu-a absent; vein 2-1A proximally tubular and distally spectral, although sometimes difficult to see; tubular vein 1 cu-a straight, incomplete/broken, and not reaching the edge of 1-1A vein. Hind wing with vannal lobe narrow, subdistally and subproximally evenly convex, and setae evenly scattered in the margin.

**Metasoma** (Fig. 42A, G, H, J). Metasoma laterally compressed. Petiole on T1 finely sculptured only laterally, virtually parallel-sided over most of length, but narrowing over distal 1/3, apex truncate (length 0.36, maximum width 0.29, minimum width 0.08), petiole with little and concentrated pubescence in the first distal third. Lateral grooves delimiting the median area on T2 clearly defined and reaching the distal edge of T2 (length median area 0.16, length T2 0.16), edges of median area obscured by weak longitudinal stripes, median area broader than long (length 0.16, maximum width 0.22, minimum width 0.08); T2 with scattered pubescence throughout. T3 longer than T2 (0.21, 0.16) and with scattered pubescence throughout. Pubescence on hypopygium dense.

**Cocoons.** White bud-like cocoons with body ridge-shape and silk fibers evenly smooth. Cocoons elongate, square in cross section, only very slightly adhered together and to adhered to the leaf substrate or in litter or soil.

#### Comments.

The sculpture on the petiole are located laterally, but differ in shape: proximally with longitudinal stripes and distally with punctation.

#### Male

(Fig. 43A–I). The body is slender than females.

#### Etymology.

Carl Barton Huffaker (September 30, 1914-October 10, 1995) was an eminent American biologist, ecologist, and agricultural entomologist at the University of California, Berkeley, USA.

#### Distribution.

Parasitized caterpillars were collected in Costa Rica, ACG, Sector El Hacha (Sendero Bejuquilla and Sendero Tigre) and Sector Del Oro (Quebrada Raíz), during October 2001 and June 2006 at 280 m in dry-rain intergrade forest.

#### Biology.

The lifestyle of this parasitoid species is solitary/gregarious.

#### Host.

*Leucotmemisnexa* (Herrich-Schäffer) (Erebidae, Arctiinae) feeding on *Serjaniaatrolineata* (Sapindaceae). Caterpillars were collected in fourth instar.

### Glyptapanteles carlossarmientoi

Taxon classificationAnimaliaHymenopteraBraconidae

Arias-Penna, sp. nov.

http://zoobank.org/FE8E5F6D-B4EC-44DB-83D8-5A8734D6C813

Figs 44 , 45

#### Female.

Body length 1.81 mm, antenna length 2.22 mm, fore wing length 2.02 mm.

#### Type material.

**Holotype**: COSTA RICA • 1♀; 03-SRNP-3824, DHJPAR0000044; Área de Conservación Guanacaste, Guanacaste, Sector Cacao, Sendero Nayo; cloud forest; 1,090 m; 10.92446, -85.46953; 12.iv.2003; Freddy Quesada leg.; caterpillar collected in fifth instar; cocoons formed on 15.iv.2003, tight stack of white cocoons standing on end tightly glued together at right angles to the leaf, forming two rows of parallel cordwood to the long axis of the cadaver, next to it on one side; adult parasitoids emerged on 24.iv.2003; (CNC). **Paratypes.** • 86 (5♀, 4♂) (77, 0♂); 03-SRNP-3824, DHJPAR0000044; same data as for holotype; (CNC).

#### Diagnosis.

**Figure 44.**
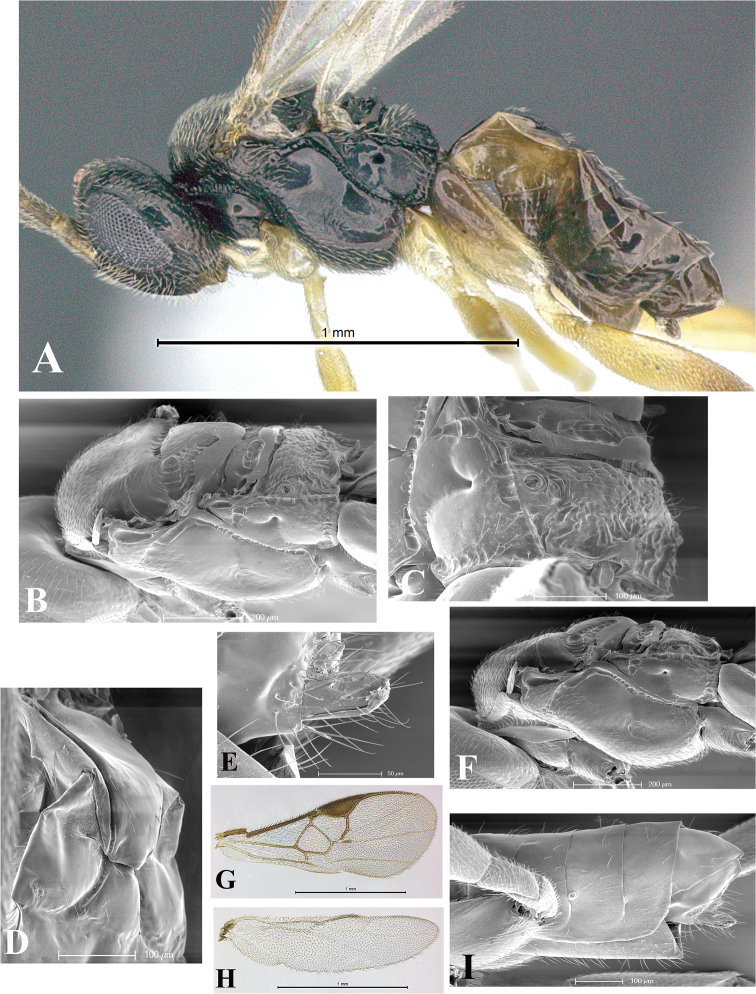
*Glyptapantelescarlossarmientoi* sp. nov. female 03-SRNP-3824 DHJPAR0000044 **A** Habitus **B, F** Mesosoma **B** Dorsolateral view **F** Lateral view **C** Metanotum, propodeum, laterodorsal view **D**T1–2, laterodorsal view **E** Genitalia: ovipositor sheaths, lateral view **G, H** Wings **G** Fore **H** Hind **I** Metasoma, lateral view.

**Figure 45.**
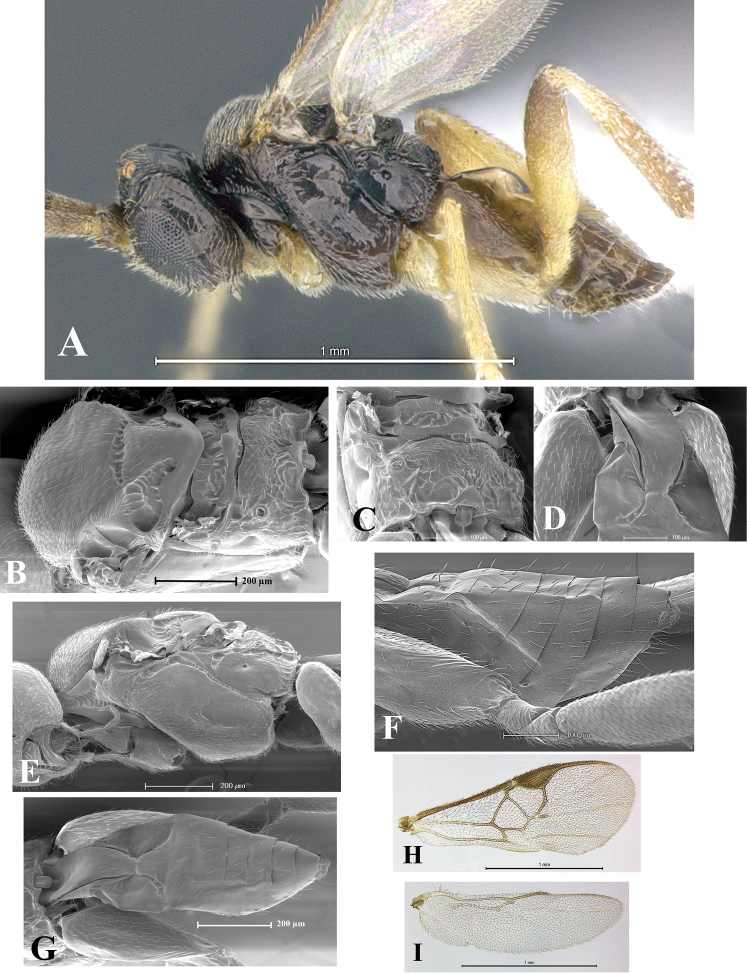
*Glyptapantelescarlossarmientoi* sp. nov. male 03-SRNP-3824 DHJPAR0000044 **A** Habitus **B, E** Mesosoma **B** Dorsolateral view **E** Lateral view **C** Metanotum, propodeum, dorsal view **D**T1–2, dorsal view **F, G** Metasoma **F** Lateral view **G** Dorsal view **H, I** Wings **H** Fore **I** Hind.

Petiole on T1 completely smooth and polished, with faint, satin-like sheen (Figs 44D, 45D), lateral grooves delimiting the median area on T2 distally losing definition on T2, and fore wing with r vein straight, outer side of junction of r and 2RS veins forming a stub (Fig. 44G).

#### Coloration

(Fig. 44A). General body coloration brown-black except some parts of both dorsal and ventral furrows of pronotum, epicnemial ridge, ventral edge of mesopleuron (coloration more conspicuous distally), distal corners of mesoscutum, lunules, BS, PFM and BM with light brown/reddish tints; scape, apex of pedicel, labrum, mandible and tegulae yellow-brown; maxillary and labial palps yellow; all antennal flagellomeres brown on both sides. Eyes gray/black and ocelli reddish (in preserved specimen). Fore and middle legs yellow-brown except brown claws; hind legs yellow-brown except proximal half of coxae, apex of femora, apex of tibiae and tarsomeres brown. Petiole on T1 yellow-brown/reddish, contours darkened, and sublateral areas yellow; T2 with median area and lateral ends brown, median area with darkened contours; T3 and beyond completely brown; distally each tergum with a narrow whitish transparent band. In lateral view, T1–2 completely yellow-brown; T3 and beyond completely brown. S1–3 yellow-brown; S4 and beyond completely brown.

#### Description.

**Head** (Fig. 44A). Head rounded with pubescence long and dense. Proximal three antennal flagellomeres longer than wide (0.15:0.05, 0.17:0.05, 0.15:0.05), distal antennal flagellomere longer than penultimate (0.12:0.05, 0.09:0.05), antenna longer than body (2.22, 1.81); antennal scrobes-frons shallow. Face with scattered finely punctate, interspaces smooth with a lateral depression at each side, and longitudinal median carina present. Frons smooth. Temple wide with punctate sculpture and interspaces wavy. Inner margin of eyes diverging slightly at antennal sockets; in lateral view, eye anteriorly convex and posteriorly straight. POL shorter than OOL (0.10, 0.12). Malar suture absent or difficult to see. Median area between lateral ocelli without depression. Vertex laterally rounded and dorsally wide.

**Mesosoma** (Fig. 44A–C, F). Mesosoma dorsoventrally convex. Distal 1/3 of mesoscutum with lateral margin slightly dented, proximally with distinctive punctation distally with a polished area, interspaces wavy/lacunose. Scutellum triangular, apex sloped and fused with BS, scutellar punctation distinct throughout, in profile scutellum flat and on same plane as mesoscutum, phragma of the scutellum completely concealed; BS only very partially overlapping the MPM; ATS demilune with complete undulate/reticulate carinae; dorsal ATS groove with semicircular/parallel carinae. Transscutal articulation with small and heterogeneous foveae, area just behind transscutal articulation with a smooth and shiny sloped transverse strip. Metanotum with BM wider than PFM (clearly differentiated); MPM circular and bisected by a median longitudinal carina; AFM without setiferous lobes and not as well delineated as PFM; PFM thick and smooth; ATM proximally with semircular/undulate carina and distally smooth. Propodeum without median longitudinal carina, proximal half weakly curved with medium-sized sculpture and distal half rugose with a shallow dent at each side of nucha; distal edge of propodeum with a flange at each side and without stubs; propodeal spiracle distally framed by a short concave carina; nucha surrounded by very short radiating carinae. Pronotum with a distinct dorsal furrow, dorsally with a well-defined smooth band; central area of pronotum smooth, but both dorsal and ventral furrows with short parallel carinae. Propleuron with fine punctations throughout and dorsally without a carina. Metasternum flat or nearly so. Contour of mesopleuron convex; precoxal groove shallow, but visible and with transverse lineate sculpture; epicnemial ridge elongated more fusiform (tapering at both ends).

**Legs** (Fig. 44A). Ventral margin of fore telotarsus entire without seta, fore telotarsus almost same width throughout and longer than fourth tarsomere (0.07, 0.05). Hind coxa with very finely punctate throughout, and dorsal outer depression absent. Inner spur of hind tibia longer than outer spur (0.16, 0.14), entire surface of hind tibia with dense strong spines clearly differentiated by color and length. Hind telotarsus longer than fourth tarsomere (0.11, 0.09).

**Wings** (Fig. 44G, H). Fore wing with r vein straight; 2RS vein slightly convex to convex; r and 2RS veins forming an angle at their junction and outer side of junction forming a slight stub; 2M vein slightly curved/swollen; distally fore wing [where spectral veins are] with microtrichiae more densely concentrated than the rest of the wing; anal cell 1/3 proximally lacking microtrichiae; subbasal cell with a small smooth area; veins 2CUa and 2CUb completely spectral; vein 2 cu-a absent; vein 2-1A proximally tubular and distally spectral, although sometimes difficult to see; tubular vein 1 cu-a straight, incomplete/broken and not reaching the edge of 1-1A vein. Hind wing with vannal lobe very narrow, subdistally and subproximally evenly convex, and setae evenly scattered in the margin.

**Metasoma** (Fig. 44A, D, E, J). Metasoma laterally compressed. Petiole on T1 completely smooth and polished, with faint, satin-like sheen, virtually parallel-sided over most of length, but narrowing over distal 1/3, apex truncate (length 0.29, maximum width 0.17, minimum width 0.06), petiole with scattered pubescence concentrated in the first distal third. Lateral grooves delimiting the median area on T2 clearly defined and reaching the distal edge of T2 (length median area 0.10, length T2 0.10), edges of median area polished and lateral grooves deep, median area broader than long (length 0.10, maximum width 0.15, minimum width 0.06); T2 with a distinctive row of pubescence only at the distal margin. T3 longer than T2 (0.18, 0.10) and with a distinctive row of pubescence only at the distal margin. Pubescence on hypopygium dense.

**Cocoons.** White oval cocoons with silk fibers evenly smooth. Two rows of cordwood cocoons tightly glued together at right angles to the leaf, running parallel to the long axis of the cadaver, next to it on one side.

#### Comments.

The propodeum is rugose in both sexes. The lateral margins of the median area on T2 are slightly curved (concave, Figs 44D, 45G) resembling the median area on T2 of *G.bourquini* (Blanchard) and *G.ecuadorius* (Whitfield et al. 2002a).

#### Male

(Fig. 45A–I). Hind coxae are completely brown, but in general, the coloration is similar to that of the female.

#### Etymology.

This species is named in honor of Carlos Eduardo Sarmiento Monroy, a Colombian entomologist, whose research is focused on Vespidae and Braconidae. Currently, he is a professor at the Universidad Nacional de Colombia, Bogotá, Colombia.

#### Distribution.

Parasitized caterpillar was collected in Costa Rica, ACG, Sector Cacao (Sendero Nayo), during April 2003 at 1,090 m in cloud forest.

#### Biology.

The lifestyle of this parasitoid species is gregarious.

#### Host.

*Aniclaignicans* (Guenée) (Noctuidae, Noctuinae) feeding on *Cynodonnlemfuensis*, introduced species, (Poaceae). Caterpillar was collected in fifth instar.

### Glyptapanteles carlrettenmeyeri

Taxon classificationAnimaliaHymenopteraBraconidae

Arias-Penna, sp. nov.

http://zoobank.org/27470263-23AE-48C8-8F55-B9E626C470ED

Fig. 46

#### Female.

Body length 1.91 mm, antenna length 2.20 mm, fore wing length 1.91 mm.

#### Type material.

**Holotype**: COSTA RICA • 1♀; 97-SRNP-9592, DHJPAR0000096; Área de Conservación Guanacaste, Guanacaste, Sector Horizontes, Quebrada San Pancho; 90 m; 10.74769, -85.58577; 09.x.1997; gusaneros leg.; caterpillar collected in fifth instar; long lines of parallel cordwood cocoons on each side of the larva, somewhat separate; adult parasitoids emerged on 25.x.1997; (CNC). **Paratypes.** • 49 (4♀, 0♂) (45♀, 0♂); 97-SRNP-9592, DHJPAR0000096; same data as for holotype; (CNC).

#### Diagnosis.

**Figure 46.**
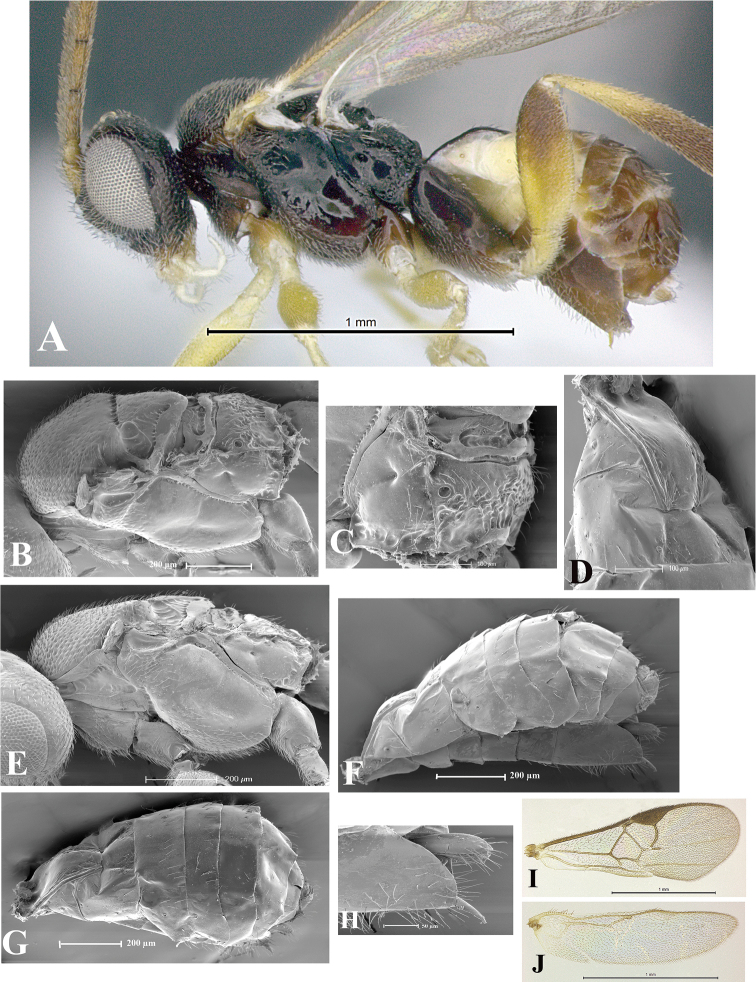
*Glyptapantelescarlrettenmeyeri* sp. nov. female 97-SRNP-9592 DHJPAR0000096 **A** Habitus **B, E** Mesosoma **B** Dorsolateral view **E** Lateral view **C** Metanotum, propodeum, laterodorsal view **D**T1–2, laterodorsal view **F, G** Metasoma **F** Lateral view **G** Dorsolateral view **H** Genitalia: hypopygium, ovipositor, ovipositor sheaths, lateral view **I, J** Wings **I** Fore **J** Hind.

Vertex in lateral view rounded (Fig. 46A), dorsal groove on axillary trough of scutellum with semicircular/parallel carinae (Fig. 46B, C), distal antennal flagellomere subequal in length with penultimate, mesoscutum distinctly punctate throughout (Fig. 46B), temple punctate, propodeum without median longitudinal carina (Fig. 46C), petiole on T1 virtually parallel-sided over most of length, but narrowing over distal 1/3, finely sculptured (Fig. 46D, G), fore wing with vein 1 cu-a straight, r vein curved, outer side of junction of r and 2RS veins not forming a stub (Fig. 46I), dorsal outer depression on hind coxa present (Fig. 46A), inner margin of eyes diverging slightly at antennal sockets, and lateral grooves delimiting the median area on T2 clearly defined and reaching the distal edge of T2 (Fig. 46D, G).

#### Coloration

(Fig. 46A). General body coloration brown-black except scape, pedicel, clypeus, mandibles, tegulae, dorsal and ventral furrows on pronotum, both ends on propleuron, epicnemial ridge, ventral edge of mesopleuron, mesosternum, distal corners on mesoscutum and lateral ends on PFM with yellow-brown/reddish brown tints; glossa, maxillary and labial palps yellow. Eyes and ocelli silver. Fore and middle legs yellow, except fore yellow-brown coxae, brown middle coxae and brown claws; hind legs yellow-brown except coxae, distal half of femora, mostly distal of tibiae and tarsomeres brown. Petiole on T1 brown, contours darkened and sublateral areas ivory/pale yellow; T2 with median area brown, contours darkened, adjacent area wide and together with the median area forming a rectangle-shape, and lateral ends ivory/pale yellow; T3 brown with proximal corners ivory/pale yellow, distally with a yellow/whitish band; T4 and beyond completely brown; distally each tergum with a narrow whitish transparent band. In lateral view, T1–3 completely pale yellow/ivory; T4 and beyond brown. S1–3 completely pale yellow/ivory; S4 and beyond completely brown.

#### Description.

**Head** (Fig. 46A). Head rounded with pubescence long and dense. Proximal three antennal flagellomeres longer than wide (0.18:0.04, 0.18:0.04, 0.15:0.04), distal antennal flagellomere subequal in length with penultimate (0.08:0.06, 0.07:0.06), antenna longer than body (2.20, 1.91); antennal scrobes-frons shallow. Face convex with scattered finely punctate and interspaces wavy, and longitudinal median carina present. Frons with punctuate sculpture. Temple wide with punctate sculpture and interspaces wavy. Inner margin of eyes diverging slightly at antennal sockets; in lateral view, eye anteriorly convex and posteriorly straight. POL shorter than OOL (0.08, 0.12). Malar suture present. Median area between lateral ocelli without depression. Vertex laterally rounded and dorsally wide.

**Mesosoma** (Fig. 46A–C, E). Mesosoma dorsoventrally convex. Mesoscutum proximally convex and distally flat, punctation distinct throughout, interspaces wavy/lacunose. Scutellum triangular, apex sloped and fused with BS, scutellar punctation distinct throughout, in profile scutellum flat and on same plane as mesoscutum, phragma of the scutellum partially exposed; BS only very partially overlapping the MPM; ATS demilune with short stubs delineating the area; dorsal ATS groove with semicircular/parallel carinae. Transscutal articulation smooth and shiny with small and homogeneous foveae, area just behind transscutal articulation nearly at the same level as mesoscutum (flat). Metanotum with BM wider than PFM (clearly differentiated); MPM circular and bisected by a median longitudinal carina; AFM without setiferous lobes and not as well delineated as PFM; PFM thick and smooth; ATM proximally with semircular/undulate carina and distally smooth. Propodeum without median longitudinal carina, proximal half weakly curved with rather coarse sculpture and distal half with a shallow dent at each side of nucha; distal edge of propodeum with a flange at each side and without stubs; propodeal spiracle distally framed by faintly concave/wavy carina; nucha surrounded by very short radiating carinae. Pronotum with a distinct dorsal furrow, dorsally with a well-defined smooth band; central area of pronotum smooth, but both dorsal and ventral furrows with short parallel carinae. Propleuron with fine punctations throughout and dorsally with a carina. Metasternum flat or nearly so. Contour of mesopleuron convex; precoxal groove smooth, shiny and shallow, but visible; epicnemial ridge elongated more fusiform (tapering at both ends).

**Legs.** Ventral margin of fore telotarsus entire, but with a tiny curved seta, fore telotarsus almost same width thought and longer than fourth tarsomere (0.10, 0.05). Hind coxa with punctation only on ventral surface and dorsal outer depression present. Inner spur of hind tibia longer than outer spur (0.11, 0.07), entire surface of hind tibia with dense strong spines clearly differentiated by color and length. Hind telotarsus as equal in length as fourth tarsomere (0.10, 0.09).

**Wings** (Fig. 46I, J). Fore wing with r vein curved; 2RS vein straight; r and 2RS veins forming a weak, even curve at their junction and outer side of junction not forming a stub; 2M vein slightly curved/swollen; distally fore wing [where spectral veins are] with microtrichiae more densely concentrated than the rest of the wing; anal cell 1/3 proximally lacking microtrichiae; subbasal cell proximal half smooth; veins 2CUa and 2CUb completely spectral; vein 2 cu-a absent; vein 2-1A proximally tubular and distally spectral, although sometimes difficult to see; tubular vein 1 cu-a straight, incomplete/broken and not reaching the edge of 1-1A vein. Hind wing with vannal lobe very narrow, subdistally and subproximally straightened, and setae absent proximally, but scattered distally.

**Metasoma** (Fig. 46A, F–H). Metasoma laterally compressed. Petiole on T1 finely sculptured only laterally, virtually parallel-sided over most of length, but narrowing over distal 1/3 (length 0.29, maximum width 0.15, minimum width 0.09), petiole with scattered pubescence and concentrated in the first distal third. Lateral grooves delimiting the median area on T2 clearly defined and reaching the distal edge of T2 (length median area 0.12, length T2 0.12), edges of median area polished and lateral grooves deep, median area broader than long (length 0.12, maximum width 0.19, minimum width 0.08), T2 with a distinctive row of pubescence only at the distal margin. T3 longer than T2 (0.17, 0.12) and with a distinctive row of pubescence only at the distal margin. Pubescence on hypopygium dense.

**Cocoons.** White or beige oval cocoons with silk fibers evenly smooth. Two parallel cordwood cocoons on each side of the larva.

#### Comments.

The ventral furrow of pronotum is wide, the intersection between dorsal and ventral furrows is wide, there with long parallel carinae.

#### Male.

unknown

#### Etymology.

Carl W. Rettenmeyer (February 10, 1931-April 9, 2009) was an American biologist who specialized in army ants (Ecitoninae).

#### Distribution.

Parasitized caterpillar was collected in Costa Rica, ACG, Sector Horizontes (Quebrada San Pancho), during October 1997 at 90 m.

#### Biology.

The lifestyle of this parasitoid species is gregarious.

#### Host.

*Isogonanatatrix* Guenée (Noctuidae, Catocalinae) feeding on *Celtisiguanaea* (Ulmaceae). Caterpillar was collected in fifth instar.

### Glyptapanteles celsoazevedoi

Taxon classificationAnimaliaHymenopteraBraconidae

Arias-Penna, sp. nov.

http://zoobank.org/B4038ABA-8DDF-4F69-BE87-5A14026442B3

Fig. 47

#### Male.

Body length 2.88 mm, antenna length 3.78 mm, fore wing length 3.08 mm.

#### Type material.

**Holotype**: ECUADOR • 1♀; EC-40395, YY-A159; Napo, Yanayacu Biological Station, Yanayacu Road, Beat 383; cloud forest; 2,100 m; -0.566667, -77.866667; 21.vii.2009; CAPEA leg.; caterpillar collected in third instar; cocoon formed on 26.vii.2009; adult parasitoid emerged on 14.viii.2009; (PUCE).

#### Diagnosis.

**Figure 47.**
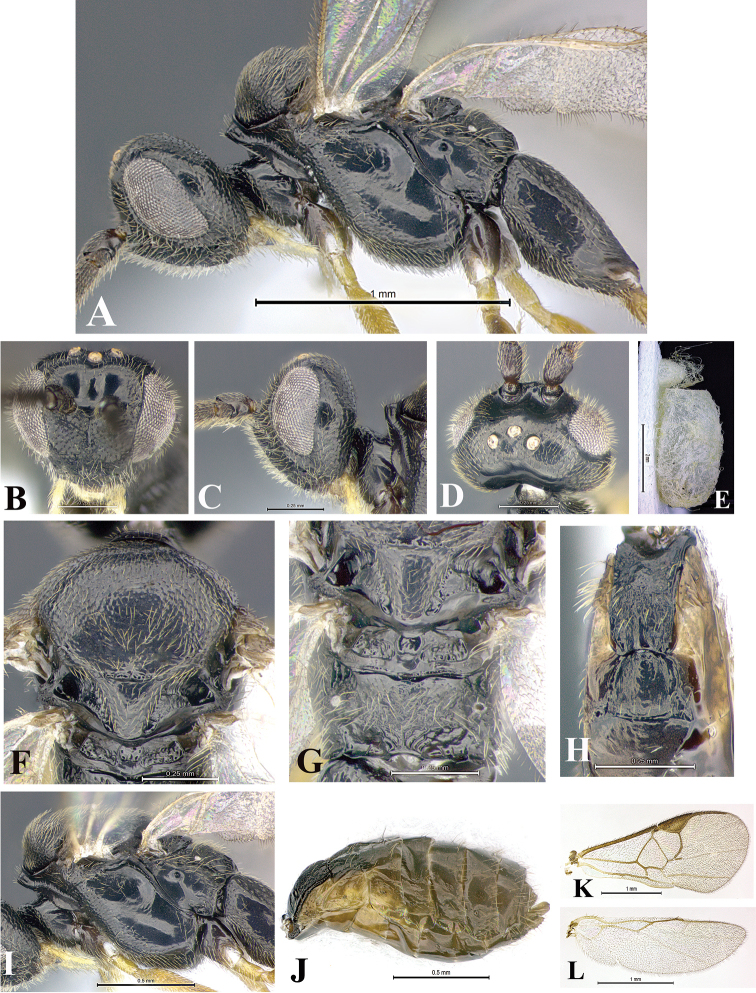
*Glyptapantelescelsoazevedoi* sp. nov. male EC-40395 YY-A159 **A** Habitus **B, D** Head **B** Frontal view **D** Dorsal view **C** Head, propleuron, lateral view **E** Cocoon **F** Mesonotum, dorsal view **G** Scutellum, metanotum, propodeum, dorsal view **H**T1–2, dorsal view **I** Mesosoma, lateral view **J** Metasoma, lateral view **K, L** Wings **K** Fore **L** Hind.

Petiole on T1with a mix of fine rugae and coarse sculpture over most of the surface (Fig. 47H), precoxal groove shallow, but visible (Fig. 47A, I), fore wing with vein 1 cu-a curved, r vein curved, outer side of junction of r and 2RS veins not forming a stub (Fig. 47K), dorsal outer depression on hind coxa present (Fig. 47A), inner margin of eyes diverging slightly at antennal sockets (Fig. 47B), propodeum without median longitudinal carina (Fig. 47G), and lateral grooves delimiting the median area on T2 clearly defined and reaching the distal edge of T2 (Fig. 47H).

#### Coloration

(Fig. 47A–L). General body coloration polished black except scape, pedicel, all antennal flagellomeres (on both sides) and tegulae brown; glossa, maxillary and labial palps light yellow-brown; labrum, mandibles, dorsal furrow of pronotum, lunules and BS brown-red/reddish. Eyes gray and ocelli reddish (in preserved specimen). Fore and middle legs light yellow-brown, except brown/brown-reddish coxae, additionally femora, tibiae and tarsomeres with a narrow dorsal brown strip from top to bottom, and claws brown; hind legs light yellow-brown except black coxae, femora with a small brown area on the apex, tibia and tarsomeres brown, additionally femora, tibiae, and tarsomeres with a narrow dorsal brown strip from top to bottom. Petiole on T1 black and sublateral areas yellow-brown; T2 with median area black and lateral ends brown; T3 and beyond completely brown; distally each tergum with a narrow yellowish translucent band. In lateral view, T1–2 completely yellow-brown; T3 yellow-brown, but dorsally brown; T4 and beyond completely brown. S1–3 completely yellow-brown; S4 and beyond completely brown.

#### Description.

**Head** (Fig. 47A–D). Head rounded with pubescence long and dense. Proximal three antennal flagellomeres longer than wide (0.25:0.10, 0.25:0.10, 0.25:0.10), distal antennal flagellomere longer than penultimate (0.15:0.06, 0.12:0.06), antenna longer than body (3.78, 2.88); antennal scrobes-frons sloped and forming a shelf. Distal half of face dented, laterally with punctations barely noticeable, interspaces smooth and longitudinal median carina present. Frons smooth. Temple wide, punctate-lacunose and interspaces wavy. Inner margin of eyes diverging slightly at antennal sockets; in lateral view, eye anteriorly convex and posteriorly straight. POL subequal in length with OOL (0.11, 0.12). Malar suture faint. Median area between lateral ocelli slightly depressed. Vertex laterally rounded and dorsally wide.

**Mesosoma** (Fig. 47A, F, G, I). Mesosoma dorsoventrally convex. Mesoscutum proximally convex and distally flat, punctation distinct throughout, and interspaces wavy/lacunose. Scutellum triangular, apex sloped and fused with BS, but not in the same plane, scutellar punctation distinct throughout, in profile scutellum flat and on same plane as mesoscutum, phragma of the scutellum partially exposed; BS only very partially overlapping the MPM; ATS demilune with short stubs delineating the area; dorsal ATS groove with semicircular/parallel carinae. Transscutal articulation with small and heterogeneous foveae, area just behind transscutal articulation nearly at the same level as mesoscutum (flat) and with same kind of sculpture as mesoscutum. Metanotum with BM convex; MPM circular without median longitudinal carina; AFM with a small lobe and not as well delineated as PFM; PFM thick and smooth with lateral ends rounded; ATM proximally with a groove with some sculpturing and distally smooth. Propodeum without median longitudinal carina, proximal half curved with rather coarse sculpture and distal half with a shallow dent at each side of nucha; distal edge of propodeum with a flange at each side and short stubs; propodeal spiracle distally framed by a short transverse carina; nucha surrounded by long radiating carinae. Pronotum with a distinct dorsal furrow, dorsally with a well-defined smooth band; central area of pronotum and dorsal furrow smooth, but ventral furrow with short parallel carinae. Propleuron finely sculptured only ventrally and dorsally without a carina. Metasternum convex. Contour of mesopleuron convex; precoxal groove smooth, shiny and shallow, but visible; epicnemial ridge convex, teardrop-shaped.

**Legs.** Ventral margin of fore telotarsus entire without seta, fore telotarsus almost same width throughout and longer than fourth tarsomere (0.12, 0.08). Dorsally hind coxa with scattered punctation, medially smooth and ventrally with dense punctation, and dorsal outer depression present. Inner spur of hind tibia longer than outer spur (0.31, 0.20), entire surface of hind tibia with dense strong spines clearly differentiated by color and length. Hind telotarsus as equal in length as fourth tarsomere (0.14, 0.14).

**Wings** (Fig. 47K, L). Fore wing with r vein slightly curved; 2RS vein slightly concave; r and 2RS veins forming a weak, even curve at their junction and outer side of junction not forming a stub; 2M vein straight; distally fore wing [where spectral veins are] with microtrichiae more densely concentrated than the rest of the wing; anal cell 1/3 proximally lacking microtrichiae; subbasal cell with microtrichiae virtually throughout; vein 2 cu-a absent; vein 2-1A proximally tubular and distally spectral, although sometimes difficult to see; tubular vein 1 cu-a curved, incomplete/broken and not reaching the edge of 1-1A vein. Hind wing with vannal lobe very narrow, subdistally and subproximally straightened, and setae evenly scattered in the margin.

**Metasoma** (Fig. 47H–J). Metasoma cylindrical. Petiole on T1 with a mix of fine rugae and coarse sculpture over most of the surface, virtually parallel-sided over most of length, but barely narrowing at apex, apex truncate (length 0.34, maximum width 0.17, minimum width 0.14), and with scattered pubescence on distal half. Lateral grooves delimiting the median area on T2 clearly defined and reaching the distal edge of T2 (length median area 0.17, length T2 0.17), edges of median area polished and followed by a deep groove, median area broader than long (length 0.17, maximum width 0.20, minimum width 0.10); T2 with pubescence only distally. T3 longer than T2 (0.23, 0.17) and with scattered pubescence only distally.

**Cocoon** (Fig. 47E). White or beige oval cocoon with silk fibers messy/disordered/fluffy.

#### Comments.

The distal half of petiole with contours convex. The distal half of propodeum with a transverse discontinuous carinae present only laterally, proximally with a dent in each lateral side, proximal half with coarse sculpture. The limit between mesopleuron and metasternum with a flattened area. The middle part of petiole is elevated, at different level that the remaining portion of the structure.

#### Female.

Unknown.

#### Etymology.

Celso Oliviera Azevedo is a Brazilian entomologist whose research is focused upon the systematics and taxonomy of Bethylidae (Hymenoptera). Currently, he works at the Universidade Federal do Espirito Santo (UFES), Vitória, Brazil.

#### Distribution.

Parasitized caterpillar was collected in Ecuador, Napo, Yanayacu Biological Station (Yanayacu Road), during July 2009 at 2,100 m in cloud forest.

#### Biology.

The lifestyle of this parasitoid species is solitary.

#### Host.

Undetermined species of Geometridae feeding on *Chusqueascandens* (Poaceae). Caterpillar was collected in third instar.

### Glyptapanteles charlesmicheneri

Taxon classificationAnimaliaHymenopteraBraconidae

Arias-Penna, sp. nov.

http://zoobank.org/6BEB63F9-EE73-4D81-9C68-DBE4F8696A0B

Figs 48 , 49

#### Female.

Body length 2.12 mm, antenna length 2.58 mm, fore wing length 2.27 mm.Type material. Holotype: COSTA RICA • 1♀; 10-SRNP-1546, DHJPAR0039004; Área de Conservación Guanacaste, Alajuela, Sector Rincón Rain Forest, Sendero Albergue Crater; 980 m; 10.84886, -85.3281; 16.iii.2001; Gloria Sihezar leg.; caterpillar collected in fifth instar; cocoons adhered to the leaf substrate and formed on 22.iii.2010; adult parasitoids emerged on 27.iii.2001; (CNC). Paratypes. • 8 (3♀, 3♂) (2♀, 0♂); 10-SRNP-1546, DHJPAR0039004; same data as for holotype; (CNC).

#### Other material.

**Reared material.** COSTA RICA: *Área de Conservación Guanacaste*, *Alajuela*, *Sector San Cristóbal*, *Melina Bufalo*: • 33 (6♀, 1♂) (26♀, 0♂); 01-SRNP-1416, DHJPAR0000015; rain forest; 560 m; 10.88400, -85.38600; 22.iv.2001; Gloria Sihezar leg.; caterpillar collected in fifth instar; long chain of irregularly pointed nearly black cocoons, alongside of what was the cadaver; not double cordwood; adult parasitoids emerged on 02.v.2001.

#### Diagnosis.

**Figure 48.**
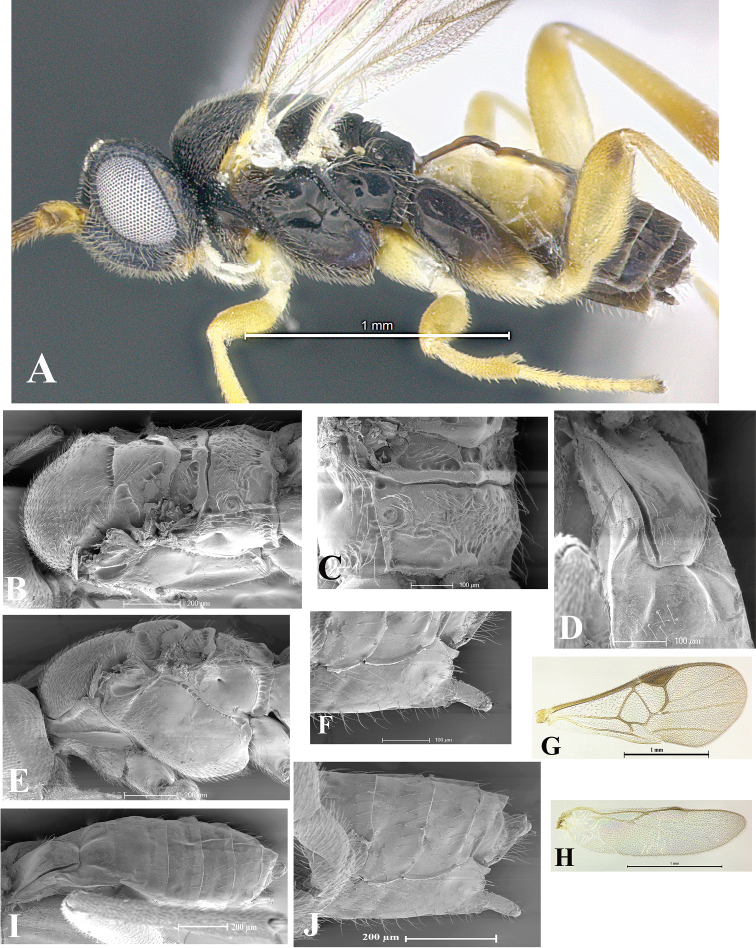
*Glyptapantelescharlesmicheneri* sp. nov. female 10-SRNP-1546 DHJPAR0039004 **A** Habitus **B, E** Mesosoma **B** Dorsolateral view **E** Lateral view **C** Metanotum, propodeum, dorsolateral view **D**T1–2, dorsolateral view **F** Genitalia: hypopygium, ovipositor, ovipositor sheaths, lateral view **G, H** Wings **G** Fore **H** Hind **I, J** Metasoma **I** Dorsolateral view **J** Lateral view.

**Figure 49.**
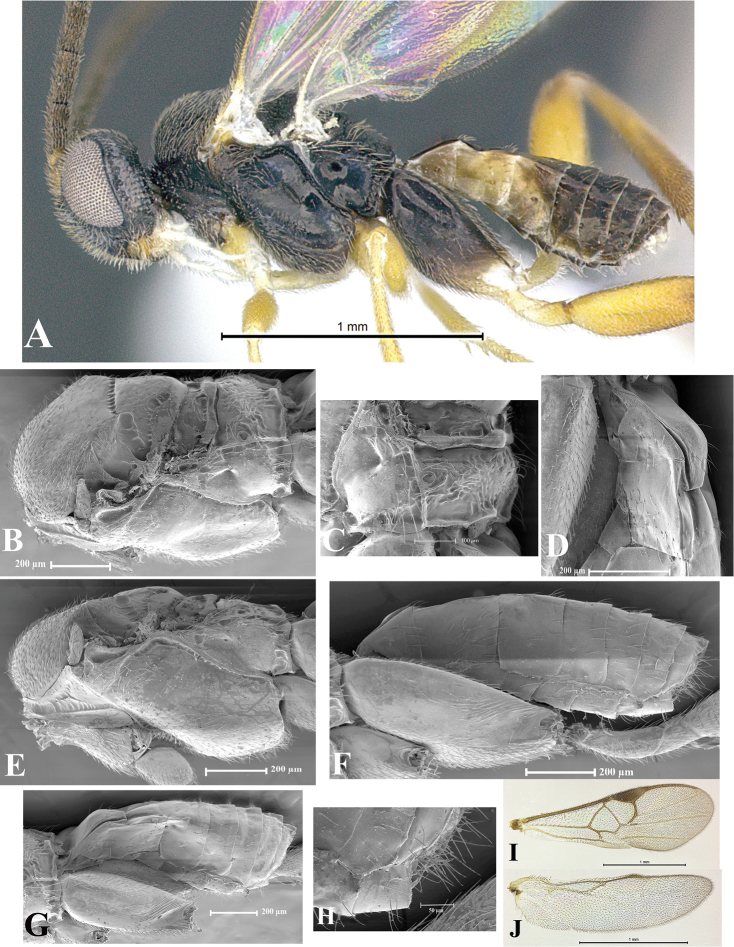
*Glyptapantelescharlesmicheneri* sp. nov. male 10-SRNP-1546 DHJPAR0039004 **A** Habitus **B, E** Mesosoma **B** Laterodorsal view **E** Lateral view **C** Metanotum, propodeum, laterodorsal view **D**T1–2, laterodorsal view **F, G** Metasoma **F** Lateral view **G** Laterodorsal view **H** Genitalia: parameres, lateral view **I, J** Wings **I** Fore **J** Hind.

Propodeal spiracle distally framed by a short concave carina (Figs 48B, C, 49B, C). Nucha surrounded by very short radiating carinae (Figs 48B, C, 49B, C). Propodeum without median longitudinal carina (Figs 48B, C, 49B, C). Antenna longer than body. Fore wing with 2RS vein straight, outer side of junction of r and 2RS veins not forming a stub (Fig. 48G, I). Lateral grooves delimiting the median area on T2 distally losing definition (Figs 48D, I, 49D, G).

#### Coloration

(Fig. 48A). General body coloration brown-black except scape and pedicel with yellow-brown with a lateral strip brown; first three proximal antennal flagellomeres dorsally lighter (yellow-brown) than ventrally (brown), remaining flagellomeres brown on both sides; labrum and mandibles yellow-brown; glossa, maxillary and labial palps yellow. Eyes and ocelli silver. Fore and middle legs yellow, but claws brown; hind legs yellow except black-brown coxae with yellow apex, distally femora with a brown dot, apex of the tibiae and tarsomeres brown. Petiole on T1 yellow, but distal 1/3 brown, contours brown, and sublateral areas light yellow; median area on T2 with proximal half yellow-brown and distal half brown, and lateral ends light yellow; T3 medially with an extended brown area with a central yellow-brown spot, and lateral ends yellow; T4 and beyond completely brown-black; distally each tergum with a narrow yellowish transparent band. In lateral view, T1–3 completely yellow; T4 and beyond brown. S1–4 yellow; penultimate sternum yellow-brown; hypopygium completely brown.

#### Description.

**Head** (Fig. 48A). Head rounded with pubescence long and dense. Proximal three antennal flagellomeres longer than wide (0.21:007, 0.21:007, 0.21:0.07), distal antennal flagellomere longer than penultimate (0.11:0.05, 0.09:0.05), antenna longer than body (2.58, 2.12); antennal scrobes-frons shallow. Face convex with scattered finely punctate, interspaces with microsculpture and longitudinal median carina present. Frons punctate. Temple wide with punctate sculpture and interspaces with microsculpture. Inner margin of eyes diverging slightly at antennal sockets; in lateral view, eye anteriorly convex and posteriorly straight. POL shorter than OOL (0.09, 0.11). Malar suture present. Median area between lateral ocelli without depression. Vertex laterally rounded and dorsally wide.

**Mesosoma** (Fig. 48A–C, E). Mesosoma dorsoventrally convex. Mesoscutum proximally convex and distally flat, punctation distinct proximally ranging to satiny distally, and interspaces wavy/lacunose. Scutellum triangular, apex sloped and fused with BS, scutellar punctation distinct peripherally, absent centrally, in profile scutellum flat and on same plane as mesoscutum, phragma of the scutellum completely concealed; BS only very partially overlapping the MPM; ATS demilune with complete undulate/reticulate carinae; dorsal ATS groove with semicircular/parallel carinae. Transscutal articulation with small and homogeneous foveae, area just behind transscutal articulation smooth, shiny and nearly at the same level as mesoscutum (flat). Metanotum with BM wider than PFM (clearly differentiated); MPM circular and bisected by a median longitudinal carina; AFM without setiferous lobes and not as well delineated as PFM; PFM thick and smooth; ATM with little and incomplete parallel carinae proximally. Propodeum without median longitudinal carina, proximal half weakly curved with rather coarse sculpture and distal half rugose with a shallow dent at each side of nucha; distal edge of propodeum with a flange at each side and without stubs; propodeal spiracle distally framed by a short concave carina; nucha surrounded by very short radiating carinae. Pronotum with a distinct dorsal furrow, dorsally with a well-defined smooth band; central area of pronotum smooth, but both dorsal and ventral furrows with short parallel carinae. Propleuron with fine rugae and dorsally without a carina. Metasternum flat or nearly so. Contour of mesopleuron straight/angulate or nearly so; precoxal groove shallow, but visible and with transverse lineate sculpture; epicnemial ridge convex, teardrop-shaped.

**Legs.** Ventral margin of fore telotarsus entire, but with a tiny curved seta, fore telotarsus almost same width throughout and longer than fourth tarsomere (0.12, 0.06). Hind coxa with punctation only on ventral surface and dorsal outer depression present. Inner spur of hind tibia longer than outer spur (0.24, 0.19), entire surface of hind tibia with dense strong spines clearly differentiated by color and length. Hind telotarsus longer than fourth tarsomere (0.13, 0.10).

**Wings** (Fig. 48G, H). Fore wing with r vein curved; 2RS vein straight; r and 2RS veins forming an angle at their junction and outer side of junction not forming a stub; 2M vein straight; distally fore wing [where spectral veins are] with microtrichiae more densely concentrated than the rest of the wing; anal cell 1/3 proximally lacking microtrichiae; subbasal cell with a small smooth area; vein 2CUa absent and vein 2CUb spectral; vein 2 cu-a absent; vein 2-1A proximally tubular and distally spectral, although sometimes difficult to see; tubular vein 1 cu-a curved, incomplete/broken and not reaching the edge of 1-1A vein. Hind wing with vannal lobe narrow, subdistally and subproximally straightened, and setae present only proximally.

**Metasoma** (Fig. 48A, D, F, I, J). Metasoma laterally compressed. Petiole on T1 completely smooth and polished, with faint, satin-like sheen, virtually parallel-sided over most of length, but narrowing over distal 1/3, apex truncate (length 0.34, maximum width 0.14, minimum width 0.09), petiole with scattered pubescence concentrated in the first distal third. Lateral grooves delimiting the median area on T2 distally losing definition (length median area 0.10, length T2 0.14), edges of median area polished and lateral grooves deep, median area broader than long (length 0.10, maximum width 0.12, minimum width 0.05), T2 with scattered pubescence only distally. T3 longer than T2 (0.21, 0.14) and with scattered pubescence only distally. Pubescence on hypopygium dense.

**Cocoons.** Black oval cocoons with silk fibers evenly smooth. Single row of cordwood cocoons forming a long chain of irregularly cocoons alongside the caterpillar cadaver and adhered to the leaf substrate.

#### Comments.

The propodeum in both sexes is rugose.

#### Male

(Fig. 49A–J). The males are slenderer than females. The hind coxa is completely brown.

#### Etymology.

Charles Duncan Michener (22 September 1918-1 November 2015) was an American entomologist who devoted his entire distinguished career to the systematics and natural history of bees.

#### Distribution.

The parasitized caterpillar was collected in Costa Rica, ACG, Sector Rincón Rain Forest (Sendero Albergue Crater) and Sector San Cristóbal (Melina Bufalo), during March 2001 at 560 m and 980 m in rain forest.

#### Biology.

The lifestyle of this parasitoid species is gregarious.

#### Host.

*Phyprosopusparthenope* Schaus (Noctuidae, Catocalinae) on *Celtisiguanaea* (Ulmaceae). Caterpillar was collected in fifth instar.

### Glyptapanteles charlesporteri

Taxon classificationAnimaliaHymenopteraBraconidae

Arias-Penna, sp. nov.

http://zoobank.org/10FD1E1E-597B-4F9F-B60F-6AE6881F68A8

Figs 50 , 51

#### Female.

Body length 3.53 mm, antenna length 4.03 mm, fore wing length 3.18 mm.Type material. Holotype COSTA RICA • 1♀; 06-SRNP-9500, DHJPAR0012673; Área de Conservación Guanacaste, Alajuela, Sector San Cristóbal, Potrero Argentina; pastures; 520 m; 10.89021, -85.38803; 21.xi.2006; Carolina Cano leg.; caterpillar collected in fourth instar; brown dull gray cocoons adhered to the leaf substrate and formed on 03.xii.2006; adult parasitoids emerged on 12.xii.2006; (CNC). Paratypes. • 23 (4♀, 4♂) (15♀, 0♂); 06-SRNP-9500, DHJPAR0012673; same data as for holotype; (CNC).

#### Other material.

**Reared material.** COSTA RICA: *Área de Conservación Guanacaste*, *Alajuela*, *Sector San Cristóbal*, *Río Blanco Abajo*: • 61 (3♀, 3♂) (46♀, 9♂); 02-SRNP-704, DHJPAR0000276; rain forest; 500 m; 10.90037, -85.37254; 05.ii.2002; Carolina Cano; caterpillar collected in fourth instar; cocoons in host cocoon; adult parasitoids emerged on 23.ii.2002. • 32 (3♀, 3♂) (21♀, 5♂); 05-SRNP-7307, DHJPAR0005108; same data as for preceding except: 23.xi.2005, Gloria Sihezar; caterpillar collected in third instar; dark gray cocoons in host cocoon; adult parasitoids emerged on 05.i.2006.

*Área de Conservación Guanacaste*, *Alajuela*, *Sector San Cristóbal*, *Sendero Corredor*: • 44 (3♀, 3♂) (37♀, 1♂); 03-SRNP-35034, DHJPAR0000266, DHJPAR0001522; 620 m; 10.87868, -85.38963; 22.xii.2003; Elda Araya; caterpillar collected in fourth instar; beige solitary ovoid cocoons in host cocoon; adult parasitoids emerged on 10.i.2003.

*Área de Conservación Guanacaste*, *Alajuela*, *Sector San Cristóbal*, *Sendero Palo Alto*: 39 (3♀, 3♂) (30♀, 3♂); 05-SRNP-2592, DHJPAR0004233; 570 m; 10.88186, -85.38221; 09.v.2005; Carolina Cano; caterpillar collected in fourth instar; dark cocoons in host cocoon; adult parasitoids emerged on 03.vi.2005.

*Área de Conservación Guanacaste*, *Alajuela*, *Sector San Cristóbal*, *Vado Río Cucaracho*: 42 (3♀, 3♂) (32♀, 4♂); 06-SRNP-4410, DHJPAR0012010; 640 m; 10.8702, -85.39153; 06.vi.2006; Yessenia Mendoza; caterpillar collected in fourth instar; cocoons adhered to the larval cuticle; adult parasitoids emerged on 27.vi.2006.

*Área de Conservación Guanacaste*, *Alajuela*, *Sector San Cristóbal*, *Sendero Tepiscuintle*: • 32 (3♀, 3♂) (24♀, 2♂); 07-SRNP-1274, DHJPAR0030908; rain forest; 14.iii.2007; Carolina Cano; caterpillar collected in third instar; cocoons in host cocoon; adult parasitoids emerged on 12.iv.2007.

*Área de Conservación Guanacaste*, *Alajuela*, *Sector Rincón Rain Forest*, *Sendero Anonás*: • 60 (5♀, 1♂) (54♀, 0♂); 09-SRNP-40118, DHJPAR0034264; 405 m; 10.90528, -85.27882, 20.i.2009; Jorge Hernández; caterpillar collected in fourth instar; cocoons adhered to larva and substrate; adult parasitoids emerged on 20.ii.2009.

#### Malaise-trapped material.

COSTA RICA: *Área de Conservación Guanacaste*, *Alajuela*, *Sector San Cristóbal*, *Río Blanco Abajo*: • 1 (1♀, 0♂) (0♀, 0♂); 07-SRNP-66569, DHJPAR0025107; rain forest; 500 m; 10.90037, -85.37254; Malaise trap; 13.xii.2007; DH Janzen & W Hallwachs. • 1 (1♀, 0♂) (0♀, 0♂); 07-SRNP-66768, DHJPAR0025306; same data as for preceding except: 02.ix.2007.

*Área de Conservación Guanacaste*, *Alajuela*, *Sector San Cristóbal*, *Bosque Trampa Malaise*: • 1 (0♀, 0♂) (1♀, 0♂); 07-SRNP-67843, DHJPAR0027639; rain forest; 815 m; 10.86280, -85.38460; 01.xi.2007; DH Janzen & W Hallwachs.

*Área de Conservación Guanacaste*, *Alajuela*, *Sector Rincón Rain Forest*, *Vado Río Francia*: • 1 (1♀, 0♂) (0♀, 0♂); 07-SRNP-66943, DHJPAR0025481; 400 m; 10.90093, -85.28915; Malaise trap; 09.x.2007; DH Janzen & W Hallwachs. • 1 (0♀, 0♂) (1♀, 0♂); 07-SRNP-66965, DHJPAR0025503; same data as for preceding except: 20.xii.2007. • 1 (0♀, 0♂) (1♀, 0♂); 08-SRNP-41681, DHJPAR0026124; same data as for preceding except: 01.i.2008.

#### Diagnosis.

**Figure 50.**
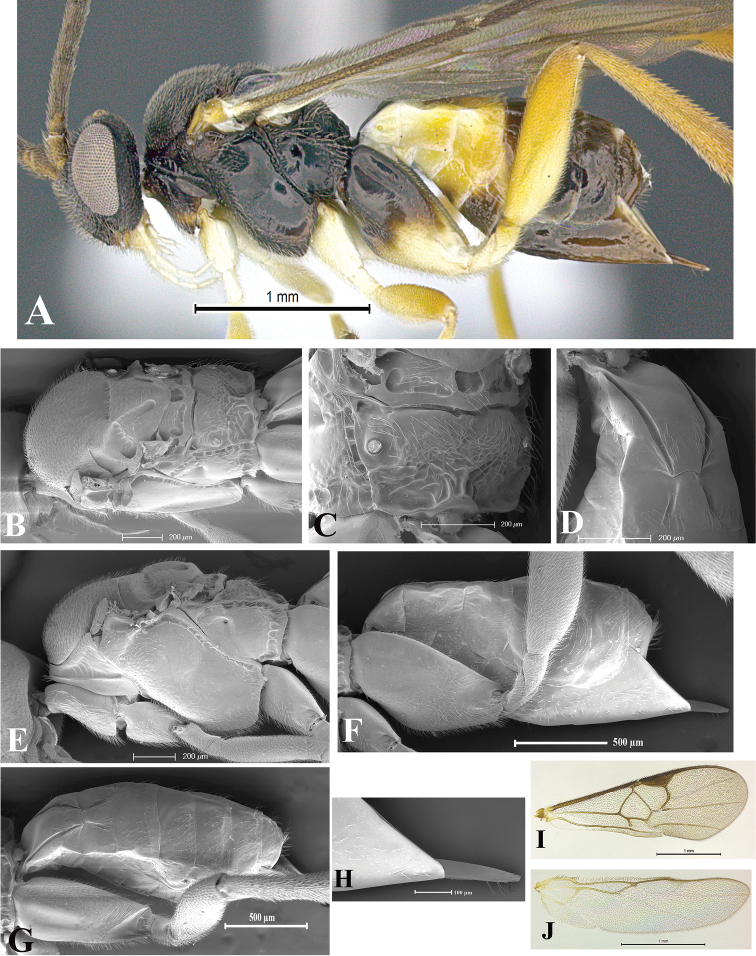
*Glyptapantelescharlesporteri* sp. nov. female 06-SRNP-9500 DHJPAR0012673 **A** Habitus **B, E** Mesosoma **B** Dorsolateral view **E** Lateral view **C** Metanotum, propodeum, dorsal view **D**T1–2, dorsolateral view **F, G** Metasoma **F** Lateral view **G** Laterodorsal view **H** Genitalia: hypopygium, ovipositor sheaths, lateral view **I, J** Wings **I** Fore **J** Hind.

**Figure 51.**
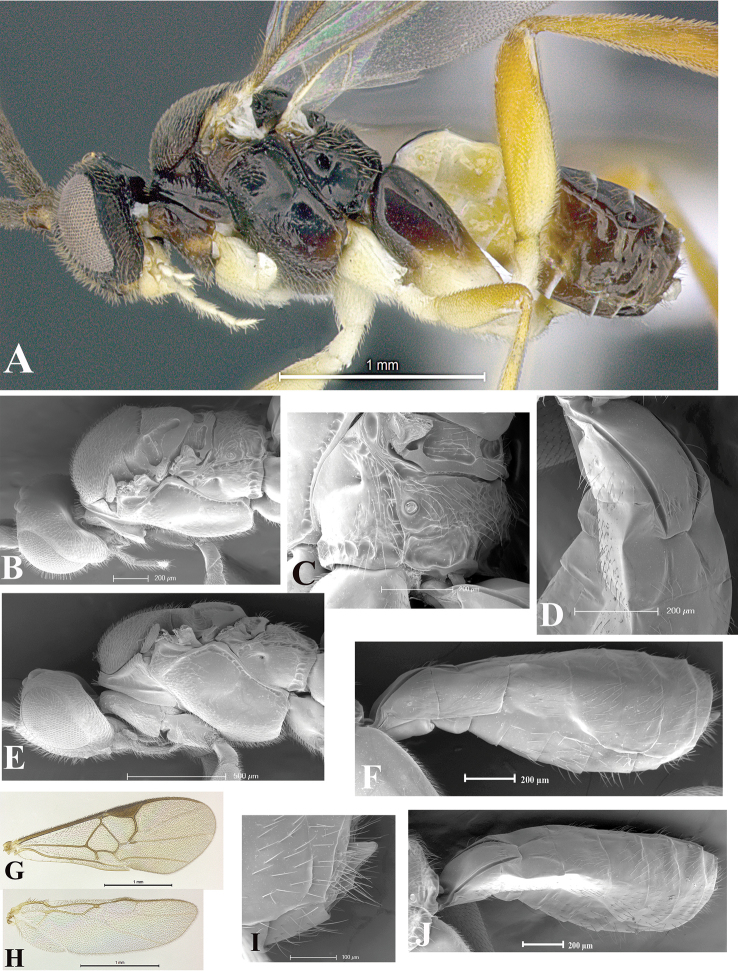
*Glyptapantelescharlesporteri* sp. nov. male 06-SRNP-9500 DHJPAR0012673 **A** Habitus **B, E** Mesosoma **B** Laterodorsal view **E** Lateral view **C** Metanotum, propodeum, laterodorsal view **D**T1–2, dorsolateral view **F, J** Metasoma **F** Lateral view **J** Laterodorsal view **G, H** Wings **G** Fore **H** Hind **I** Genitalia: parameres, lateral view.

Propleuron with fine rugae, dorsal carina delimiting a dorsal furrow absent (Figs 50A, E, 51A, B, E), distal antennal flagellomere subequal in length with penultimate, mesoscutum punctation distinct proximally ranging to satiny distally (Figs 50B, 51B), median area on T2 as broad as long, lateral grooves delimiting the median area distally losing definition (Figs 50D, G, 51D, J), propodeal spiracle distally framed by a short concave carina (Figs 50B, C, 51B, C), scutellum in profile convex and slightly higher than mesoscutum (Figs 50E, 51E), and fore wing with 2RS convex, outer side of junction of r and 2RS veins not forming a stub (Figs 50I, 51G).

#### Coloration

(Fig. 50A). General body coloration brown-black except scape, pedicel, labrum, mandibles, a narrow ventral strip of propleuron, distal-lateral of mesopleuron and epicemial ridge with brown-yellow tints; glossa, maxillary and labial palps, and tegulae yellow. Eyes and ocelli silver. Fore and middle legs yellow (coloration intensity increasing towards the apex); hind legs yellow except coxae with proximal half dark brown, apex of tibiae and basitarsus yellow-brown, remaining tarsomeres brown. Petiole on T1 brown-black and sublateral areas light yellow; median area on T2 with yellow-brown, contours darkened, adjacent area and lateral ends yellow; T3 completely dark yellow; T4 and beyond dark brown; distally each tergum with a narrow whitish/yellow-brown transparent band. In lateral view, T1–3 completely yellow; T4 and beyond brown. S1–3 yellow; S4 yellow, medially with a small brown spot; penultimate sternum and hypopygium completely brown.

#### Description.

**Head** (Fig. 50A). Head triangular with pubescence long and dense. Proximal three antennal flagellomeres longer than wide (0.32:0.10, 0.30:0.10, 0.30:0.10), distal antennal flagellomere subequal in length with penultimate (0.18:0.10, 0.17:0.10), antenna longer than body (4.03, 3.53); antennal scrobes-frons shallow. Face convex with scattered finely punctate, interspaces wavy and longitudinal median carina present. Frons smooth. Temple wide with punctate sculpture and interspaces wavy. Inner margin of eyes diverging slightly at antennal sockets; in lateral view, eye anteriorly convex and posteriorly straight. POL shorter than OOL (0.10, 0.13). Malar suture present. Median area between lateral ocelli without depression. Vertex laterally rounded and dorsally wide.

**Mesosoma** (Fig. 50A–C, E). Mesosoma dorsoventrally convex. Distal 1/3 of mesoscutum with lateral margin slightly dented, punctation distinct proximally ranging to satiny distally, interspaces wavy/lacunose. Scutellum triangular, apex sloped and fused with BS, scutellar punctation distinct throughout, in profile scutellum convex and slightly higher than mesoscutum, phragma of the scutellum partially exposed; BS only very partially overlapping the MPM; ATS demilune with short stubs delineating the area; dorsal ATS groove with semicircular/parallel carinae. Transscutal articulation with small and heterogeneous foveae, area just behind transscutal articulation smooth, shiny and nearly at the same level as mesoscutum (flat). Metanotum with BM wider than PFM (clearly differentiated); MPM circular without median longitudinal carina; AFM without setiferous lobes and not as well delineated as PFM; PFM thick and smooth; ATM proximally with semircular/undulate carina and distally smooth. Propodeum without median longitudinal carina, proximal half weakly curved with medium-sized sculpture and distal half rugose with a shallow dent at each side of nucha; distal edge of propodeum with a flange at each side and without stubs; propodeal spiracle distally framed by a short concave carina; nucha surrounded by very short radiating carinae. Pronotum with a distinct dorsal furrow, dorsally with a well-defined smooth band; central area of pronotum and dorsal furrow smooth, but ventral furrow with short parallel carinae. Propleuron with fine rugae and dorsally without a carina. Metasternum flat or nearly so. Contour of mesopleuron straight/angulate or nearly so; precoxal groove with faintly transverse lineate sculpture and shallow, but visible; epicnemial ridge convex, teardrop-shaped.

**Legs** (Fig. 50A). Ventral margin of fore telotarsus entire without seta, fore telotarsus almost same width throughout and longer than fourth tarsomere (0.14, 0.09). Hind coxa with punctation only on ventral surface and dorsal outer depression present. Inner spur of hind tibia longer than outer spur (0.41, 0.25), entire surface of hind tibia with dense strong spines clearly differentiated by color and length. Hind telotarsus as equal in length as fourth tarsomere (0.15, 0.16).

**Wings** (Fig. 50I, J). Fore wing with r vein curved; 2RS vein slightly convex to convex; r and 2RS veins forming an angle at their junction and outer side of junction not forming a stub; 2M vein slightly curved/swollen; distally fore wing [where spectral veins are] with microtrichiae more densely concentrated than the rest of the wing; anal cell 1/3 proximally lacking microtrichiae; subbasal cell with a small smooth area; veins 2CUa and 2CUb completely spectral; vein 2 cu-a absent; vein 2-1A proximally tubular and distally spectral, although sometimes difficult to see; tubular vein 1 cu-a curved, incomplete/broken and not reaching the edge of 1-1A vein. Hind wing with vannal lobe narrow, subdistally and subproximally straightened, and setae present only proximally.

**Metasoma** (Fig. 50A, D, F–H). Metasoma laterally compressed. Petiole on T1 finely sculptured only distally, virtually parallel-sided over most of length, but narrowing over distal 1/3, apex truncate (length 0.46, maximum width 0.20, minimum width 0.10), petiole with scattered pubescence concentrated in the first distal third. Lateral grooves delimiting the median area on T2 distally losing definition (length median area 0.15, length T2 0.20 mm), edges of median area polished and lateral grooves deep, median area longer than broad (length 0.15, maximum width 0.12, minimum width 0.06), T2 with scattered pubescence throughout. T3 longer than T2 (0.25, 0.20) and with scattered pubescence only distally. Pubescence on hypopygium dense.

**Cocoons** (Fig. 4N). Beige, gray or brown oval cocoons with silk fibers ordered, but covered by a net. Cocoons adhered to the leaf substrate or formed in host cocoon or adhered to the larval cuticle.

#### Comments.

The distal half of the propodeum, at each side of the nucha, with strong wavy carinae. The propodeal spiracle framed with a strong distal concave carina. Dorsally, the propleuron without a carina, but the limit among smooth area with rugae area is distinctive. Female with the ovipositor sheath protruding beyond the hypopygium.

#### Male

(Fig. 51A–J). In some males (e.g., 06-SRNP-4410, 05-SRNP-7307) the coloration on T1–3 is darker than females.

#### Etymology.

Charles C. Porter is an ichneumonidologist at Florida State Collection of Arthropods, Florida Department of Agriculture and Consumer Services Gainesville, FL, USA.

#### Distribution.

The parasitized caterpillars were collected in Costa Rica, ACG, Sector Rincón Rain Forest (Sendero Anonás) and Sector San Cristóbal (Potrero Argentina, Río Blanco Abajo, Sendero Corredor, Sendero Palo Alto, Sendero Tepiscuintle, and Vado Río Cucaracho), during February 2002, December 2003, May and November 2005, June and November 2006, March 2007, and January 2009 at 405 m, 500 m, 520 m, 570 m, 620 m,and 640 m on pasture and rain forest.

Adult parasitoids were collected in Costa Rica, ACG, Sector Rincón Rain Forest (Vado Río Francia) and Sector San Cristóbal (Bosque Trampa Malaise and Río Blanco Abajo), during September-December 2007 and January 2008 at 400 m, 500 m, and 815 m in rain forest.

#### Biology.

The lifestyle of this parasitoid species is gregarious.

#### Host.

Shag-carpet moth *Tarchonfelderi* Druce (Apatelodidae) (Fig. 4N) feeding on *Acalyphadiversifolia* (Euphorbiaceae), *Chamaedoreatepejilote* (Arecaceae), *Heliconiairrasa* (Heliconiaceae), *Lycianthespauciflora* (Solanaceae), *Pavoniaschiedeana* (Malvaceae) and *Psychotriaberteriana* (Rubiaceae). *Apatelodes* sp. Packard (Apatelodidae) feeding on *Philodendronrhodoaxis* (Araceae). Caterpillars were collected in third and fourth instar.

### Glyptapanteles chrisdarlingi

Taxon classificationAnimaliaHymenopteraBraconidae

Arias-Penna, sp. nov.

http://zoobank.org/D9CE0EE6-E60C-4522-84DE-4B9792651738

Figs 52 , 53

#### Female.

Body length 2.17 mm, antenna length 2.27 mm, fore wing length 2.27 mm.

#### Type material.

**Holotype**: COSTA RICA • 1♀; 03-SRNP-7181, DHJPAR0000047; Área de Conservación Guanacaste, Alajuela, Sector San Cristóbal, Sendero Pinyal; 630 m; 10.87161, -85.39333; 14.vii.2003; Carolina Cano leg.; caterpillar collected in fifth instar; two rows of dark gray cordwood on each side of the cadaver, parallel to long axis; adult parasitoids emerged on 25.vii.2003; (CNC). **Paratypes.** • 43 (3♀, 3♂) (37♀, 0♂); 03-SRNP-7181, DHJPAR0000047; same data as for holotype; (CNC).

#### Other material.

**Reared material.** COSTA RICA: *Área de Conservación Guanacaste*, *Alajuela*, *Sector San Cristóbal*, *Vado Río Cucaracho*: • 49 (5♀, 5♂) (28♀, 11♂); 06-SRNP-4972, DHJPAR0012008; rain forest; 640 m; 10.8702, -85.39153; 20.vi.2006; Carolina Cano leg.; caterpillar collected in fifth instar; brown cocoons forming two rows of cordwood on each side of the cadaver; adult parasitoids emerged on 28.vi.2006.

#### Diagnosis.

**Figure 52.**
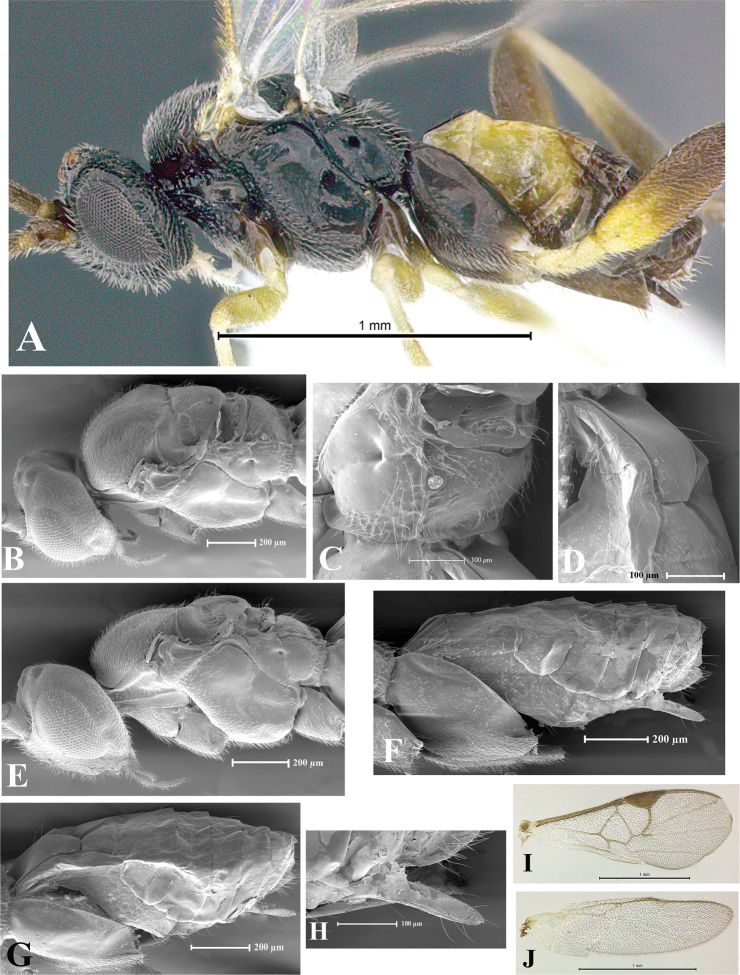
*Glyptapanteleschrisdarlingi* sp. nov. female 03-SRNP-7181 DHJPAR0000047 **A** Habitus **B, E** Mesosoma **B** Laterodorsal view **E** Lateral view **C** Metanotum, propodeum, laterodorsal view **D**T1–2, dorsolateral view **F, G** Metasoma **F** Lateral view **G** Laterodorsal view **H** Genitalia: hypopygium, ovipositor sheaths, lateral view **I, J** Wings **I** Fore **J** Hind.

**Figure 53.**
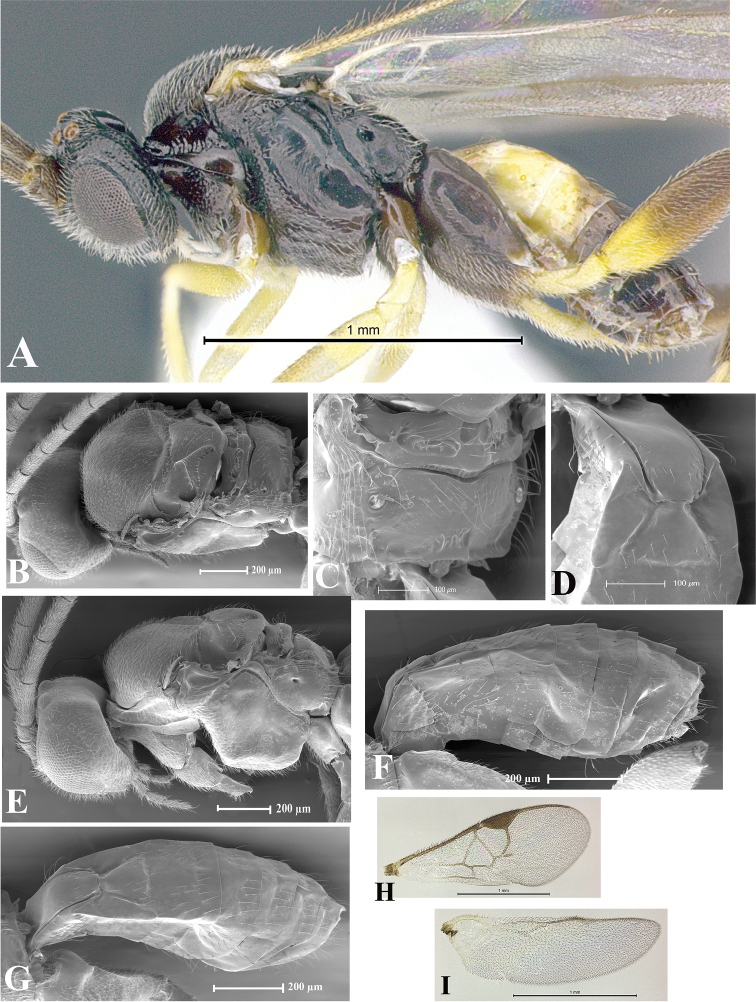
*Glyptapanteleschrisdarlingi* sp. nov. male 03-SRNP-7181 DHJPAR0000047 **A** Habitus **B, E** Mesosoma **B** Dorsolateral view **E** Lateral view **C** Metanotum, propodeum, dorsal view **D**T1–2, dorsal view **F, G** Metasoma **F** Lateral view **G** Dorsolateral view **H, I** Wings **H** Fore **I** Hind.

Ventral margin of fore telotarsus entire, scutellar punctation distinct throughout (Figs 52B, 53B), propodeal spiracle without distal carina (Figs 52C, 53C), petiole on T1 distally with lateral margins relatively straight, finely sculptured only laterally (Figs 52D, G, 53D, G), surface of metasternum flat or nearly, precoxal groove deep with lineate sculpture (Figs 52A, E, 53A, E), fore wing with vein 1 cu-a curved, r vein curved, outer side of junction of r and 2RS veins not forming a stub (Figs 52I, 53H), dorsal outer depression on hind coxa present (Figs 52A, F, 53A), inner margin of eyes diverging slightly at antennal sockets, propodeum without median longitudinal (Figs 52C, 53C), and lateral grooves delimiting the median area on T2 clearly defined and reaching the distal edge of T2 (Figs 52D, G, 53D, G).

#### Coloration

(Fig. 52A). General body coloration brown-black except scape, pedicel, labrum, mandibles and tegulae yellow-brown; glossa, maxillary and labial palps yellow. Eyes dark gray and ocelli reddish (in preserved specimen). Fore and middle legs yellow except coxae and claws; hind legs yellow except coxae, femora, 2/3 distal of tibiae and tarsomeres brown. Petiole on T1 brown and sublateral areas yellow; T2 with median and adjacent areas brown, and narrow lateral ends yellow; T3 almost completely brown except proximal corners yellow; T4 and beyond completely brown; distally each tergum with a narrow whitish/yellowish transparent band. In lateral view, T1–3 completely yellow; T4 and beyond brown. S1–3 yellow; S4 yellow-brown; penultimate sternum and hypopygium brown; ovipositor sheath brown.

#### Description.

**Head** (Fig. 52A, B, E). Head rounded with pubescence long and dense. Proximal three antennal flagellomeres longer than wide (0.17:0.06, 0.17:0.06, 0.16:0.06), distal antennal flagellomere longer than penultimate (0.11:0.05, 0.09:0.05), antenna longer than body (2.27, 2.17); antennal scrobes-frons shallow. Face punctate-lacunose, interspaces with microsculpture, distal half dented only laterally, and longitudinal median carina present. Frons smooth. Temple wide, punctate, and interspaces wavy. Inner margin of eyes diverging slightly at antennal sockets; in lateral view, eye anteriorly convex and posteriorly straight. POL shorter than OOL (0.09, 0,11). Malar suture present. Median area between lateral ocelli slightly depressed. Vertex laterally rounded and dorsally wide.

**Mesosoma** (Fig. 52A–C, E). Mesosoma dorsoventrally convex. Distal 1/3 of mesoscutum with lateral margin slightly dented, punctation distinct proximally and with polished area distally, and interspaces wavy/lacunose. Scutellum triangular, apex sloped and fused with BS, scutellar punctation distinct throughout, in profile scutellum slightly convex, but on same plane as mesoscutum, phragma of the scutellum partially exposed; BS only very partially overlapping the MPM; ATS demilune with short stubs delineating the area; dorsal ATS groove with carinae only proximally. Transscutal articulation with small and heterogeneous foveae, area just behind transscutal articulation with a smooth and shiny sloped transverse strip. Metanotum with BM wider than PFM (clearly differentiated); MPM circular without median longitudinal carina; AFM without setiferous lobes and not as well delineated as PFM; PFM thick and smooth; ATM proximally with semircular/undulate carina and distally smooth. Propodeum without median longitudinal carina, proximal half weakly curved with medium-sized sculpture and distal half relatively polished; distal edge of propodeum with a flange at each side and without stubs; propodeal spiracle without distal carina; nucha surrounded by very short radiating carinae. Pronotum with a distinct dorsal furrow, dorsally with a well-defined smooth band; central area of pronotum smooth, but both dorsal and ventral furrows with short parallel carinae. Propleuron with fine rugae and dorsally without a carina. Metasternum flat or nearly so. Contour of mesopleuron straight/angulate or nearly so; precoxal groove deep with faintly transverse lineate sculpture; epicnemial ridge convex, teardrop-shaped.

**Legs.** Ventral margin of fore telotarsus entire, but with a tiny curved seta, fore telotarsus almost same width throughout and longer than fourth tarsomere (0.11, 0.06). Hind coxa with punctation only on ventral surface and dorsal outer depression present. Inner spur of hind tibia longer than outer spur (0.20, 0.16), entire surface of hind tibia with dense strong spines clearly differentiated by color and length. Hind telotarsus longer than fourth tarsomere (0.14, 0.11).

**Wings** (Fig. 52I, J). Fore wing with r vein slightly curved; 2RS vein straight; r and 2RS veins forming an angle at their junction and outer side of junction not forming a stub; 2M vein straight; distally fore wing [where spectral veins are] with microtrichiae more densely concentrated than the rest of the wing; anal cell 1/3 proximally lacking microtrichiae; subbasal cell with a small smooth area; vein 2CUa absent and vein 2CUb spectral; vein 2 cu-a absent; vein 2-1A proximally tubular and distally spectral, although sometimes difficult to see; tubular vein 1 cu-a curved, incomplete/broken and not reaching the edge of 1-1A vein. Hind wing with vannal lobe narrow, subdistally and subproximally straightened, and setae present only proximally.

**Metasoma** (Fig. 52A, D, F–H). Metasoma laterally compressed. Petiole on T1 finely sculptured only laterally, virtually parallel-sided over most of length, but narrowing over distal 1/3, apex truncate (length 0.25, maximum width 0.13, minimum width 0.05), petiole with scattered pubescence concentrated in the first distal third. Lateral grooves delimiting the median area on T2 clearly defined and reaching the distal edge of T2 (length median area 0.15, length T2 0.15 mm), edges of median area polished and lateral grooves deep, median area broader than long (length 0.15, maximum width 0.17, minimum width 0.05), T2 with scattered pubescence only distally. T3 longer than T2 (0.19 0.15) and with scattered pubescence only distally. Pubescence on hypopygium dense.

**Cocoons** (Fig. 4Q). Brown or gray oval cocoons with silk fibers evenly smooth. Cocoons arranged in two rows of cordwood on each side of the caterpillar cadaver.

#### Comments.

The minimum wide on median area on T2 has a fold, at first glance looks like the edges are not well-defined.

#### Male

(Fig. 53A–I). The coloration similar to that of females and the metasoma is thinner than in females.

#### Etymology.

D. Christopher (Chris) Darling is a senior curator of Entomology in the Department of Natural History at the Royal Ontario Museum (ROM), Toronto, Ontario, Canada. He is a world authority on the taxonomy of the Perilampidae (Chalcidoidea) and he has focused upon understanding the diversity and evolutionary relationships of these wasps.

#### Distribution.

The parasitized caterpillars were collected in Costa Rica, ACG, Sector San Cristóbal (Sendero Pinyal and Vado Río Cucaracho), during June of 2003 and 2006 at 630 m and 640 m in rain forest.

#### Biology.

The lifestyle of this parasitoid species is gregarious.

#### Host.

*Concana* sp. Walker (Noctuidae: Bagisarinae) (Fig. 4Q) feeding on *Bunchosiacornifolia* (Malpighiaceae). Caterpillars were collected in fifth instar.

### Glyptapanteles chrisgrinteri

Taxon classificationAnimaliaHymenopteraBraconidae

Arias-Penna, sp. nov.

http://zoobank.org/3B5CC15C-95A9-4E3E-9A2A-BE7011E3C27E

Figs 54 , 55

#### Female.

Body length 1.96 mm, antenna length 2.32 mm, fore wing length 2.07 mm.

#### Type material.

**Holotype**: COSTA RICA • 1♀; 00-SRNP-21162, DHJPAR0000003; Área de Conservación Guanacaste, Alajuela, Sector Rincón Rain Forest, Sendero Rincón; 430 m; 10.8962, -85.27769; 23.xii.2000; José Pérez leg.; caterpillar collected in second instar; dark gray cocoons are glued hard side by side on a stem forming a single row of cordwood, cocoons formed on 07.i.2001; adult parasitoids emerged on 16.i.2001; (CNC). **Paratypes.** • 59 (3♀, 4♂) (48♀, 2♂); 00-SRNP-21162, DHJPAR0000003; same data as for holotype; (CNC).

#### Other material.

**Reared material.** COSTA RICA: *Área de Conservación Guanacaste*, *Alajuela*, *Sector San Cristóbal*, *Río Blanco Abajo*: • 9 (4♀, 2♂) (3♀, 0♂); 01-SRNP-3283, DHJPAR0000019; rain forest; 500 m; 10.90037, -85.37254; 30.viii.2001; Freddy Quesada leg.; caterpillar collected in third instar; dark elongate cocoons adhered lightly to each other in no particular pattern and adhered to the leaf substrate; adult parasitoids emerged on 11.ix.2001. • 46 (3♀, 0♂) (43♀, 0♂); 05-SRNP-6549, DHJPAR0004773; same data as for preceding except: 18.x.2005; Elda Araya; tightly packed dark cocoons adhered on midrib of leaf; adult parasitoids emerged on 05.xi.2005. • 19 (3♀, 3♂) (13♀, 0♂); 05-SRNP-6550, DHJPAR0004781; same data as for preceding except: 18.x.2005; Elda Araya leg.; caterpillar collected in second instar; tightly packed dark cocoons, on a midrib; adult parasitoids emerged on 09.xi.2005. • 46 (3♀, 3♂) (39♀, 1♂); 05-SRNP-6551, DHJPAR0004782; same data as for preceding except: 18.x.2005; Elda Araya leg.; adult parasitoids emerged on 01.xi.2005. • 52 (3♀, 3♂) (43♀, 3♂); 07-SRNP-2878, DHJPAR0020263; same data as for preceding except: 21.vi.2007; Anabelle Cordoba leg.; cocoons adhered in tight clusters on the rachis of the leaf; adult parasitoids emerged on 01.xi.2005.

*Área de Conservación Guanacaste*, *Alajuela*, *Sector San Cristóbal*, *Vado Río Cucaracho*: • 46 (3♀, 3♂) (10♀, 30♂); 05-SRNP-6415, DHJPAR0004771; rain forest; 640 m; 10.8702, -85.39153; 14.x.2005; Elda Araya leg.; caterpillar collected in fifth instar; dark stack of cocoons along a midrib; adult parasitoids emerged on 26.x.2005. • 29 (3♀, 3♂) (18♀, 5♂); 05-SRNP-6416, DHJPAR0004785; same data as for preceding except: cocoons adhered to the leaf substrate; adult parasitoids emerged on 28.x.2005. • 25 (3♀, 3♂) (13♀, 6♂); 05-SRNP-6417, DHJPAR0004775; same data as for preceding except: cocoons adhered to the leaf substrate; adult parasitoids emerged on 28.x.2005. • 46 (3♀, 3♂) (5♀, 35♂); 05-SRNP-6418, DHJPAR0004776; same data as for preceding except: cocoons adhered to the leaf substrate.

*Área de Conservación Guanacaste*, *Alajuela*, *Sector San Cristóbal*, *Sendero Huerta*: • 37 (5♀, 5♂) (19♀, 8♂); 07-SRNP-289, DHJPAR0012679; rain forest; 527 m; 10.9305, -85.37223; 16.i.2007; Anabelle Cordoba leg.; caterpillar collected in fourth instar; cocoons adhered to the leaf substrate and formed on 20.i.2007; adult parasitoids emerged on 02.ii.2007. • 58 (5♀, 3♂) (50♀, 0♂); 07-SRNP-290, DHJPAR0012682; same data as for preceding except: dark brown cocoons; date of cocoons not reported; adult parasitoids emerged on 26.i.2007.

*Área de Conservación Guanacaste*, *Alajuela*, *Sector San Cristóbal*, *1 km al Este Nueva Zelandia*: • 42(3♀, 3♂) (29♀, 7♂); 05-SRNP-6172, DHJPAR0002895; rain forest; 675 m; 10.86564, -85.39561; 03.x.2005; Anabelle Cordoba leg.; caterpillar collected in fifth instar; cocoons adhered to the leaf substrate and formed on 08.x.2005; adult parasitoids emerged on 17.x.2005 and caterpillar still alive.

*Área de Conservación Guanacaste*, *Alajuela*, *Sector San Cristóbal*, *Puente Palma*: • 29 (3♀, 0♂) (26♀, 0♂); 05-SRNP-6692, DHJPAR0004777; rain forest; 460 m; 10.9163, -85.37869; 23.x.2005; Gloria Sihezar leg.; caterpillar collected in fourth instar; cocoons adhered to the leaf substrate; adult parasitoids emerged on 10.xi.2005.

#### Diagnosis.

**Figure 54.**
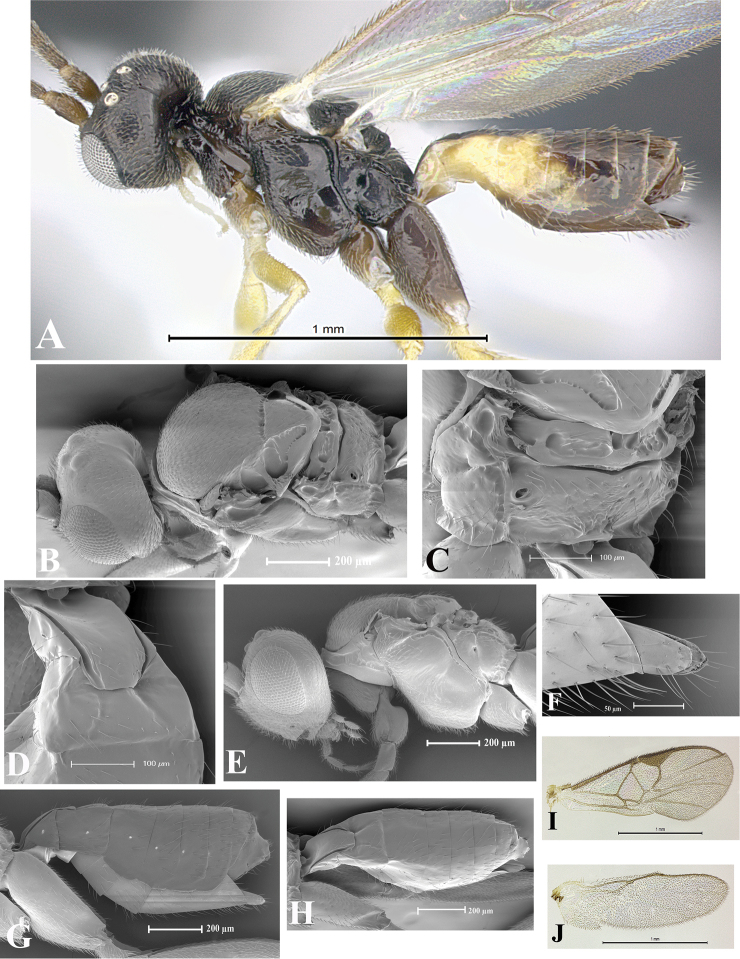
*Glyptapanteleschrisgrinteri* sp. nov. female 00-SRNP-21162 DHJPAR0000003 **A** Habitus **B, E** Head, mesosoma **B** Dorsolateral view **E** Lateral view **C** Metanotum, propodeum, dorsolateral view **D**T1–2, dorsolateral view **F** Genitalia: hypopygium, ovipositor sheaths, lateral view **G, H** Metasoma **G** Lateral view **H** Dorsolateral view **I, J** Wings **I** Fore **J** Hind.

**Figure 55.**
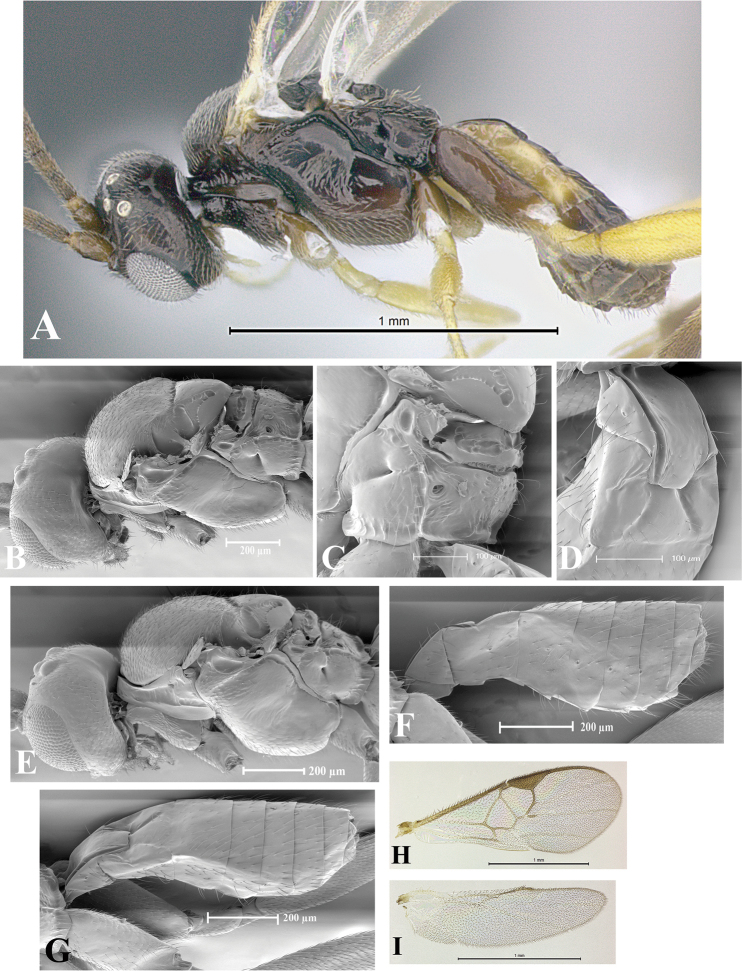
*Glyptapanteleschrisgrinteri* sp. nov. male 00-SRNP-21162 DHJPAR0000003 **A** Habitus **B, E** Head, mesosoma **B** Laterodorsal view **E** Lateral view **C** Metanotum, propodeum, laterodorsal view **D**T1–2, dorsolateral view **F, G** Metasoma **F** Lateral view **G** Dorsolateral view **H, I** Wings **H** Fore **I** Hind.

In lateral view metasoma laterally compressed (Figs 54A, G, 55A), fore wing with 1 cu-a vein straight, complete, touching the edge of 1-1A vein, vein curved, outer side of junction of r and 2RS veins forming a slight stub (Figs 54I, 55H), inner margin of eyes straight throughout, scutellum in profile flat and on same plane as mesoscutum (Figs 54E, 55E), precoxal groove with faintly lineate sculpture (Figs 54A, E, 55A, E), dorsal carina delimiting a dorsal furrow on propleuron absent (Figs 54E, 55E), petiole on T1 parallel-sided, but narrowing over distal 1/3 (Figs 54D, 55D), precoxal groove deep (Figs 54A, 55A), anteroventral contour of mesopleuron straight/angulate or nearly so (Figs 54E, 55E), and edges of median area on T2 polished and followed by a deep groove (Figs 54D, 55D).

#### Coloration

(Fig. 54A). General body coloration brown-black except scape, pedicel, mandibles and labrum yellow-brown; glossa, maxillary and labial palps, and tegulae yellow. Eyes and ocelli silver. Fore and middle legs yellow except dark yellow-brown coxae and brown claws; hind legs yellow except coxae, a small dot on the apex of femora, distal half of tibiae, and tarsomeres brown. Petiole on T1 brown and sublateral areas yellow-brown; T2 with median and adjacent areas brown, and lateral ends yellow-brown; T3 brown with lateral ends narrow and yellow-brown; T4 and beyond completely brown; distally each tergum with a narrow whitish transparent band. In lateral view, T1–3 completely yellow; T4 yellow, but dorsally brown; T5 and beyond brown. S1–3 completely yellow; S4 proximal half yellow, distal half brown; penultimate sternum and hypopygium completely brown.

#### Description.

**Head** (Fig. 54A, B, E). Head rounded with pubescence long and dense. Proximal three antennal flagellomeres longer than wide (0.17:0.05, 0.18:0.05, 0.18:0.05), distal antennal flagellomere longer than penultimate (0.11:0.05, 0.09:0.05), antenna longer than body (2.32, 1.96); antennal scrobes-frons shallow. Face convex with scattered finely punctate, interspaces on face wavy and longitudinal median carina present. Frons with punctuate sculpture. Temple wide with punctate sculpture and interspaces wavy. Inner margin of eyes straight throughout; in lateral view, eye anteriorly convex and posteriorly straight. POL shorter than OOL (0.08, 0.11). Malar suture absent or difficult to see. Median area between lateral ocelli without depression. Vertex laterally rounded and dorsally wide.

**Mesosoma** (Fig. 54A–C, E). Mesosoma dorsoventrally convex. Mesoscutum 1/4 distal with a central dent, punctation distinct throughout and interspaces wavy/lacunose. Scutellum triangular, apex sloped and fused with BS, scutellar punctation distinct throughout, in profile scutellum flat and on same plane as mesoscutum, phragma of the scutellum partially exposed; BS only very partially overlapping the MPM; ATS demilune with short stubs delineating the area; dorsal ATS groove smooth. Transscutal articulation with small and heterogeneous foveae, area just behind transscutal articulation with same kind of sculpture as mesoscutum and nearly at the same level as mesoscutum (flat). Metanotum with BM wider than PFM (clearly differentiated); MPM circular and bisected by a median longitudinal carina; AFM with a small lobe and not as well delineated as PFM; PFM thick and smooth; ATM proximally with semircular/undulate carina and distally smooth. Propodeum without median longitudinal carina, proximal half weakly curved with medium-sized sculpture and distal half relatively polished and with a shallow dent at each side of nucha; distal edge of propodeum with a flange at each side and without stubs; propodeal spiracle without distal carina; nucha surrounded by very short radiating carinae. Pronotum with a distinct dorsal furrow, dorsally with a well-defined smooth band; central area of pronotum and dorsal furrow smooth, but ventral furrow with short parallel carinae. Propleuron with fine rugae and dorsally without a carina. Metasternum flat or nearly so. Contour of mesopleuron straight/angulate or nearly so; precoxal groove deep with faintly lineate sculpture; epicnemial ridge elongated more fusiform (tapering at both ends).

**Legs.** Ventral margin of fore telotarsus entire, but with a tiny curved seta, fore telotarsus almost same width throughout and longer than fourth tarsomere (0.09, 0.06). Hind coxa with punctation only on ventral surface and dorsal outer depression absent. Inner spur of hind tibia longer than outer spur (0.19, 0.13), entire surface of hind tibia with dense strong spines clearly differentiated by color and length. Hind telotarsus longer than fourth tarsomere (0.11, 0.09).

**Wings** (Fig. 54I, J). Fore wing with r vein slightly curved; 2RS vein slightly convex to convex; r and 2RS veins forming an angle at their junction and outer side of junction forming a slight stub; 2M vein slightly curved/swollen; distally fore wing [where spectral veins are] with microtrichiae more densely concentrated than the rest of the wing; anal cell 1/3 proximally lacking microtrichiae; subbasal cell with a small smooth area; vein 2CUa absent and vein 2CUb spectral; vein 2 cu-a absent; vein 2-1A proximally tubular and distally spectral, although sometimes difficult to see; tubular vein 1 cu-a straight, complete and touching the edge of 1-1A vein. Hind wing with vannal lobe narrow, subdistally evenly convex and subproximally straightened, and setae present only proximally.

**Metasoma** (Fig. 54A, D, F–H). Metasoma laterally compressed. Petiole on T1 completely smooth and polished, with faint, satin-like sheen, virtually parallel-sided over most of length, but narrowing over distal 1/3 and apex truncate (length 0.21, maximum width 0.11, minimum width 0.05), and with scattered pubescence concentrated in the first distal third. Lateral grooves delimiting the median area on T2 clearly defined and reaching the distal edge of T2 (length median area 0.12, length T2 0.12), edges of median area polished and lateral grooves deep, median area as broad as long (length 0.12, maximum width 0.13, minimum width 0.05); T2 with scattered pubescence only distally. T3 longer than T2 (0.17, 0.12) and with scattered pubescence throughout. Pubescence on hypopygium dense.

**Cocoons.** Gray or brown oval cocoons with silk fibers evenly smooth. Tightly packed cocoons arranged in a single row of cordwood and adhered to the leaf substrate.

#### Comments.

The propodeum has a series of delicate rugae that appear to follow the median longitudinal carinae. Distally, the pronotum is at a different level than the mesopleuron, thus forming a deep hollow. The specimens have slim bodies.

#### Male

(Fig. 55A–I). Similar in coloration to female.

#### Etymology.

Christopher (Chris) C. Grinter is an American lepidopterist working as Collection Manager of Entomology at California Academy of Sciences, San Francisco, CA, USA, and is the assistant secretary/treasurer of The Lepidopterists’ Society.

#### Distribution.

The parasitized caterpillars were collected in Costa Rica, ACG, Sector Rincón Rain Forest (Sendero Rincón) and Sector San Cristóbal (Este Nueva Zelandia, Puente Palma, Río Blanco Abajo, Sendero Huerta, and Vado Río Cucaracho), during December 2000, August 2001, October 2005, and January and June 2007 at 430 m, 460 m, 500 m, 527 m, 640 m, and 675 m in rain forest.

#### Biology.

The lifestyle of this parasitoid species is gregarious.

#### Host.

*Lesmoneaemylia* (Druce) (Noctuidae: Catocalinae) feeding on *Mimosadormiens* (Fabaceae). Caterpillars were collected in second, third, fourth, and fifth instar. Caterpillars were still alive after the adult parasitoids emerged.

### Glyptapanteles christerhanssoni

Taxon classificationAnimaliaHymenopteraBraconidae

Arias-Penna, sp. nov.

http://zoobank.org/F162090F-C764-4593-A3AB-1A86B0F0E299

Figs 56 , 57

#### Female.

Body length 2.02 mm, antenna length 2.63 mm, fore wing length 2.28 mm.

#### Type material.

**Holotype**: COSTA RICA • 1♀; 99-SRNP-3045, DHJPAR0001523; Área de Conservación Guanacaste, Guanacaste, Sector El Hacha, Sendero Tigre; intergrade dry-rain forest; 280 m; 11.03172, -85.52615; 01.vii.1999; Roster Moraga leg.; caterpillar collected in fifth instar; grayish white cocoons forming two parallel rows of cordwood with the caterpillar in the middle; adult parasitoids emerged on 11.vii.1999; (CNC). **Paratypes.** • 41 (3♀, 4♂) (32♀, 2♂); 99-SRNP-3045, DHJPAR0001523; same data as for holotype; (CNC).

#### Other material.

**Reared material.** COSTA RICA: *Área de Conservación Guanacaste*, *Guanacaste*, *Sector El Hacha*, *Finca Araya*: • 38 (6♀, 3♂) (29♀, 0♂); 01-SRNP-24114, DHJPAR0000018; intergrade dry-rain forest; 295 m; 11.01541, -85.51125; 20.xi.2001; Roster Moraga leg.; caterpillar collected in fourth instar; cocoons adhered to the leaf substrate forming a cordwood on each side of cadaver and formed on 22.xi.2001; adult parasitoids emerged on 01.xii.2001.

*Área de Conservación Guanacaste*, *Guanacaste*, *Sector El Hacha*, *Sendero Tigre*: • 34 (5♀, 5♂) (23♀, 1♂); 01-SRNP-11314, DHJPAR0000006; intergrade dry-rain forest; 280 m; 11.03172, -85.52615; 01.x.2001, Lucia Ríos leg.; caterpillar collected in fourth instar; single row of beige cordwood cocoons on each side of the caterpillar; adult parasitoids emerged on 06.x.2001.

#### Diagnosis.

**Figure 56.**
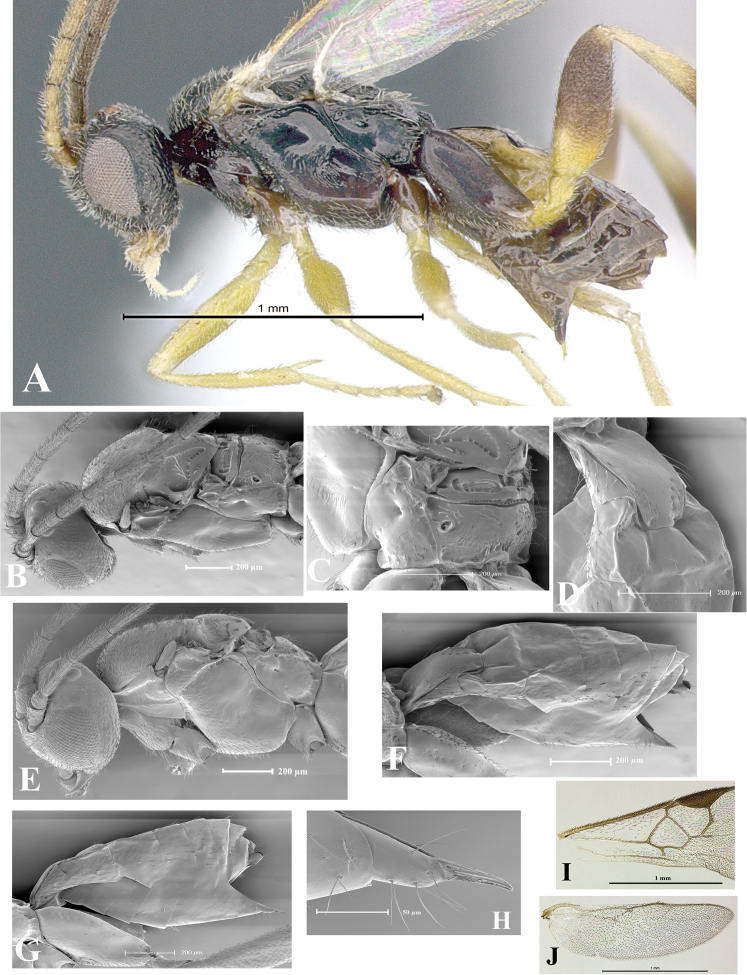
*Glyptapanteleschristerhanssoni* sp. nov. female 99-SRNP-3045 DHJPAR0001523 **A** Habitus **B, E** Head, mesosoma **B** Dorsolateral view **E** Lateral view **C** Metanotum, propodeum, laterodorsal view **D**T1–2, dorsolateral view **F, G** Metasoma **F** Dorsolateral view **G** Lateral view **H** Genitalia: hypopygium, ovipositor, ovipositor sheaths, lateral view **I, J** Wings **I** Fore **J** Hind.

**Figure 57.**
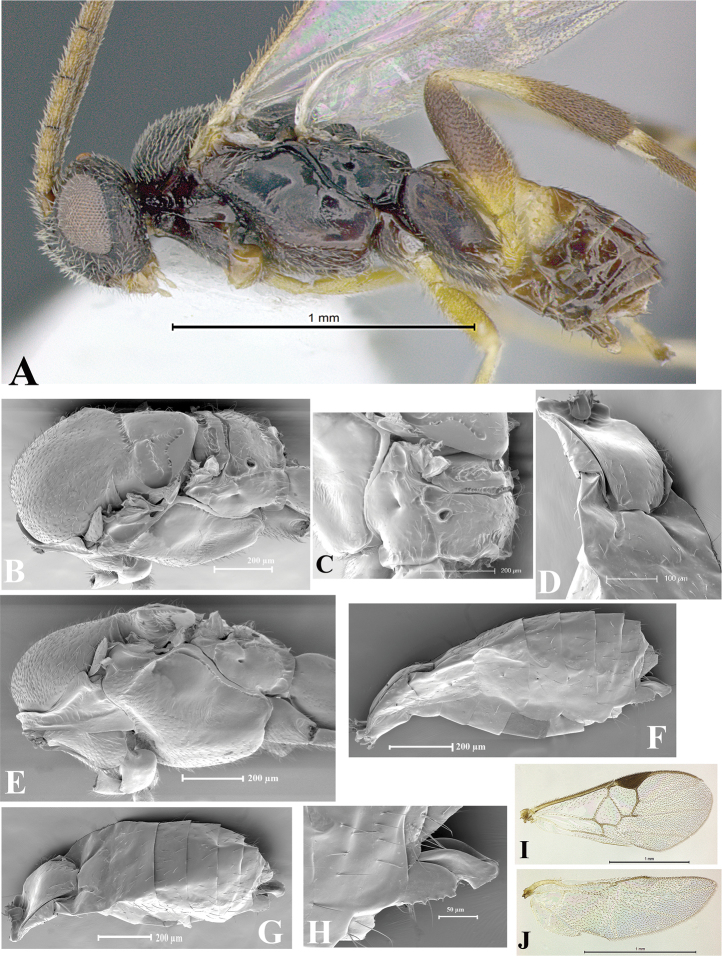
*Glyptapanteleschristerhanssoni* sp. nov. male 99-SRNP-3045 DHJPAR0001523 **A** Habitus **B, E** Mesosoma **B** Dorsolateral view **E** Lateral view **C** Metanotum, propodeum, laterodorsal view **D**T1–2, dorsolateral view **F, G** Metasoma **F** Lateral view **G** Dorsolateral view **H** Genitalia: parameres, lateral view **I, J** Wings **I** Fore **J** Hind.

Proximal half of propodeum weakly curved (Figs 56C, 57C, D), propleuron with fine punctations throughout (Figs 56E, 57E), distal antennal flagellomere longer than penultimate, mesoscutum punctation distinct proximally ranging to satiny distally (Figs 56B, 57B), medioanterior pit of metanotum bisected by a median longitudinal carina (Figs 56C, 57B), propodeum without a median longitudinal carina (Figs 56C, 57C), scutellum in profile flat and on same plane as mesoscutum (Figs 56E, 57E), propodeal spiracle without distal carina (Figs 56C, 57C), phragma of the scutellum completely concealed (Figs 56B, 57B), nucha surrounded by long radiating carinae (Figs 56C, 57C), dorsal carina delimiting a dorsal furrow on propleuron present (Figs 56E, 57E), petiole on T1 parallel-sided, but narrowing over distal 1/3 (Figs 56D, 57D), precoxal groove deep (Figs 56A, 57A), anteroventral contour of mesopleuron straight/angulate or nearly so (Figs 56E, 57E), edges of median area on T2 polished and followed by a deep groove (Figs 56D, 57D), and fore wing with r vein curved, outer side of junction of r and 2RS veins forming a distinct stub (Figs 56I, 57I).

#### Coloration

(Fig. 56A). General body coloration black-brown except scape, pedicel, labrum, mandibles, and tegulae; five-six proximal antennal flagellomeres dorsally lighter (yellow-brown) than ventrally (brown), remaining flagellomeres brown on both sides; glossa, maxillary and labial palps yellow. Eyes and ocelli reddish (in preserved specimens). Fore and middle legs yellow except coxae and claws brown; hind legs yellow except coxae, distal 3/4 of femora, distal 3/4 of tibiae and tarsomeres brown. Petiole on T1 brown and sublateral areas yellow-brown; T2 with median area brown, and adjacent area and lateral ends yellow-brown; T3 and beyond brown; distally each tergum with a narrow whitish transparent band. In lateral view, T1–3 yellow-brown; T4 and beyond brown. S1–3 yellow-brown; S4 and beyond brown; ovipositor sheaths brown.

#### Description.

**Head** (Fig. 56A, B, E). Head triangular with pubescence long and dense. Proximal three antennal flagellomeres longer than wide (0.17:0.05, 0.18:0.05, 0.18:0.05), distal antennal flagellomere longer than penultimate (0.12:0.05, 0.8:0.05), antenna longer than body (2.63, 2.02); antennal scrobes-frons shallow. Face with scattered finely punctate, interspaces wavy, distal half dented laterally, and longitudinal median carina present. Frons punctate. Temple wide, punctate and interspaces wavy. Inner margin of eyes diverging slightly at antennal sockets; in lateral view, eye anteriorly convex and posteriorly straight. POL shorter than OOL (0.09, 0.12). Malar suture present. Median area between lateral ocelli without depression. Vertex laterally rounded and dorsally wide.

**Mesosoma** (Fig. 56A–C, E). Mesosoma dorsoventrally convex. Mesoscutum proximally convex and distally flat, punctation distinct proximally ranging to satiny distally and interspaces wavy/lacunose. Scutellum triangular, apex sloped and fused with BS, scutellar punctation distinct throughout, in profile scutellum flat and on same plane as mesoscutum, phragma of the scutellum completely concealed; BS only very partially overlapping the MPM; ATS demilune with complete undulate/reticulate carinae; dorsal ATS groove with carinae only proximally. Transscutal articulation with small and heterogeneous foveae, area just behind transscutal articulation smooth, shiny and nearly at the same level as mesoscutum (flat). Metanotum with BM wider than PFM (clearly differentiated); MPM circular and bisected by a median longitudinal carina; AFM without setiferous lobes and not as well delineated as PFM; PFM thick and smooth; ATM proximally with semircular/undulate carina and distally smooth. Propodeum without median longitudinal carina, proximal half weakly curved with medium-sized sculpture and distal half with fine sculpture; distal edge with a flange at each side and without stubs; propodeal spiracle without distal carina; nucha surrounded by very short radiating carinae. Pronotum virtually without trace of dorsal furrow, dorsally with a well-defined smooth band; central area of pronotum smooth, but both dorsal and ventral furrows with short parallel carinae. Propleuron with fine punctations throughout and dorsally with a carina. Metasternum flat or nearly so. Contour of mesopleuron straight/angulate or nearly so; precoxal groove deep faintly lineate sculpture; epicnemial ridge elongated more fusiform (tapering at both ends).

**Legs.** Ventral margin of fore telotarsus entire, but with a tiny curved seta, fore telotarsus almost same width throughout and longer than fourth tarsomere (0.11, 0.06). Hind coxa with punctation only on ventral surface and dorsal outer depression present. Inner spur of hind tibia longer than outer spur (0.20, 0.15), entire surface of hind tibia with dense strong spines clearly differentiated by color and length. Hind telotarsus longer than fourth tarsomere (0.11, 0.09).

**Wings** (Fig. 56I, J). Fore wing with r vein slightly curved; 2RS vein straight; r and 2RS veins forming an angle at their junction and outer side of junction forming a slight stub; 2M vein slightly curved/swollen; distally fore wing [where spectral veins are] with microtrichiae more densely concentrated than the rest of the wing; anal cell 1/3 proximally lacking microtrichiae; subbasal cell proximal half smooth; vein 2CUa absent and vein 2CUb spectral; vein 2 cu-a absent; vein 2-1A proximally tubular and distally spectral, although sometimes difficult to see; tubular vein 1 cu-a curved and complete, but junction with 1-1A vein spectral. Hind wing with vannal lobe wide, subdistally and subproximally straightened, and setae present only proximally.

**Metasoma** (Fig. 56A, D, F–H). Metasoma laterally compressed. Petiole on T1 finely sculptured only laterally, virtually parallel-sided over most of length, but narrowing over distal 1/3 and apex truncate (length 0.29, maximum width 0.14, minimum width 0.10), petiole with little pubescence on distal half. Lateral grooves delimiting the median area on T2 distally losing definition (length median area 0.10, length T2 0.15), edges of median area polished and lateral grooves deep, median area broader than long (length 0.10, maximum width 0.15, minimum width 0.06); T2 with scattered pubescence throughout. T3 longer than T2 (0.20, 0.15) and with scattered pubescence only distally. Pubescence on hypopygium dense.

**Cocoons.** Beige or gray-white oval cocoons with silk fibers evenly smooth. Cocoons forming two parallel rows of cordwood with the caterpillar in the middle.

#### Comments.

The specimens are slim.

#### Male

(Fig. 57A–J). Similar in shape and coloration to the female.

#### Etymology.

Christer Hansson is the curator Museum of Zoology, faculty of Science at Lund University, Sweden. He is interested in some Chalcidoidea families.

#### Distribution.

The parasitized caterpillars were collected in Costa Rica, ACG, Sector El Hacha (Finca Araya and Sendero Tigre), during July 1999 and October-November 2001 at 280 m and 295 m.

#### Biology.

The lifestyle of this parasitoid species is gregarious.

#### Host.

*Lepidodesgallopavo* Druce (Noctuidae: Catocalinae) feeding on *Bunchosiapolystachia* (Malpighiaceae). Caterpillars were collected in fourth and fifth instar.

### Glyptapanteles claudiamartinezae

Taxon classificationAnimaliaHymenopteraBraconidae

Arias-Penna, sp. nov.

http://zoobank.org/440368B0-4F35-426F-BCFD-8550D9785B17

Figs 58 , 59

#### Female.

Body length 3.03 mm, antenna length 3.84 mm, fore wing length 3.49 mm.

#### Type material.

**Holotype**: ECUADOR • 1♀; EC-37562, YY-A017; Napo, Yanayacu Biological Station, San Benjamin forest, Plot 425; cloud forest; 1,934 m; -0.598889, -77.889722; 10.iii.2009; Wilmer Simbaña leg.; caterpillar collected in fourth instar; cocoons formed on 23.iii.2009; adult parasitoids emerged on 09.iv.2009; (PUCE). **Paratypes.** • 9 (4♀, 3♂) (2♀, 0♂); EC-37562, YY-A017, same data as for holotype; (PUCE).

#### Other material.

**Reared material.** ECUADOR: *Napo*, *Yanayacu Biological Station*, *San Benjamin Forest*, *Plot 425*: • 14 (5♀, 2♂) (7♀, 0♂); EC-37564, YY-A016; cloud forest; 1,934 m; -0.598889, -77.889722; 10.iii.2009; Wilmer Simbaña leg.; caterpillar collected in fourth instar; cocoons formed on 30.iii.2009; adult parasitoids emerged on 13.iv.2009.

*Napo*, *Yanayacu Biological Station*, *Sendero Macuculoma*, *Plot 392*: • 6 (2♀, 2♂) (2♀, 0♂); EC-30623, YY-A114; cloud forest; 2,155 m; -0.6, -77.883333; 17.iv.2008; Lee Dyer leg.; caterpillar collected in third instar; cocoons formed on 20.v.2008; adult parasitoids emerged on 05.vi.2008. • 4 (1♀, 1♂) (2♀, 0♂); EC-30624, YY-A115; same data as for preceding except: adult parasitoids emerged on 02.vi.2008. • 10 (3♀, 1♂) (6♀, 0♂); EC-30627, YY-A116, same data as for preceding except: cocoons formed on 12.v.2008. • 12 (2♀, 2♂) (6♀, 2♂); EC-30628, YY-A117; same data as for preceding except: cocoons formed on 18.v.2008.

*Napo*, *Yanayacu Biological Station*, *Sendero Stream Trail*: • 6 (1♀, 1♂) (3♀, 1♂); EC-37648, YY-A121; cloud forest; 2,444 m; -0.601472, -77.886444; 20.iii.2009; CAPEA; caterpillar collected in first instar; cocoons formed on 05.v.2009; adult parasitoids emerged on 19.v.2009.

*Napo*, *Yanayacu Biological Station*, *Yanayacu Road*: • 3 (1♀, 1♂) (1♀, 0♂); EC-38485, YY-A011; cloud forest; 2,100 m; -0.566667, -77.866667; 30.iv.2009; CAPEA leg.; caterpillar collected in third instar; cocoons formed on 27.v.2009; adult parasitoids emerged on 14.vi.2009. • 8 (2♀, 2♂) (4♀, 0♂); [EC-38486, YY-A100]; same data as for preceding.

#### Diagnosis.

**Figure 58.**
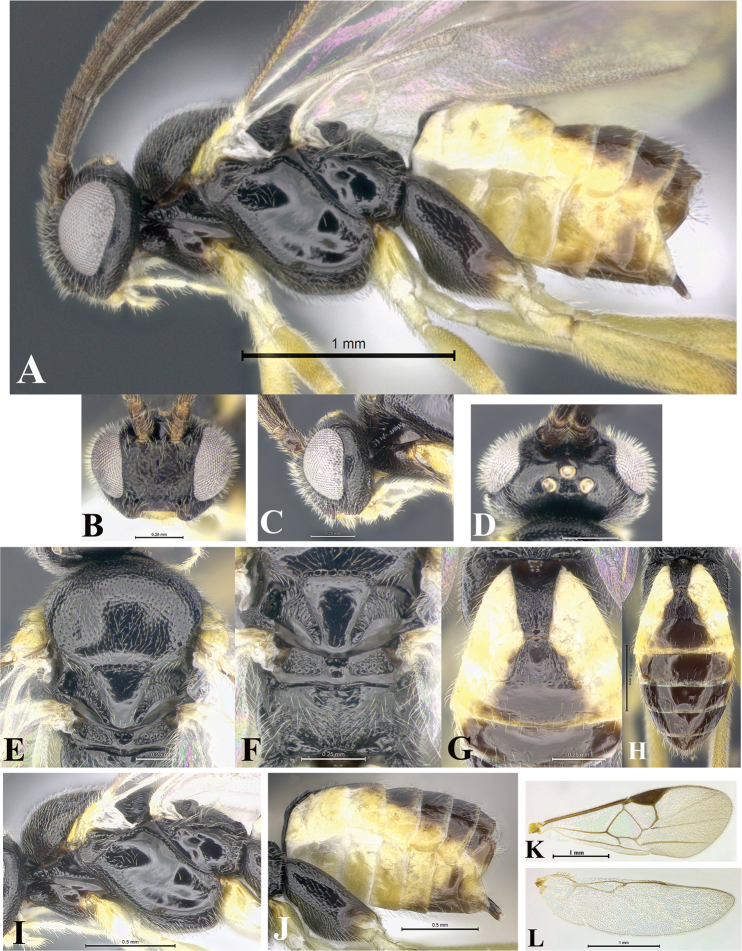
*Glyptapantelesclaudiamartinezae* sp. nov. female EC-37562 YY-A017 **A** Habitus **B–D** Head **B** Frontal view **C** Lateral view **D** Dorsal view **E** Mesonotum, dorsal view **F** Scutellum, metanotum, propodeum, dorsal view **G**T1–3, dorsal view **H, J** Metasoma **H** Dorsal view **J** Lateral view **I** Mesosoma, lateral view **K, L** Wings **K** Fore **L** Hind.

**Figure 59.**
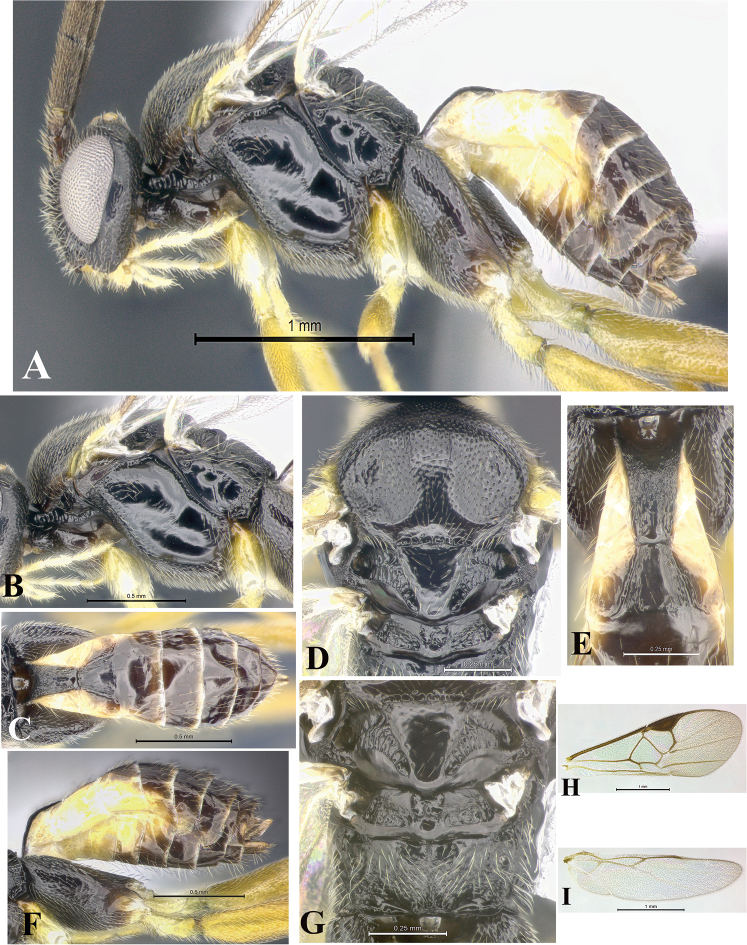
*Glyptapantelesclaudiamartinezae* sp. nov. male EC-37562 YY-A017 **A** Habitus **B** Mesosoma, lateral view **C, F** Metasoma **C** Dorsal view **F** Lateral view **D** Mesonotum, dorsal view **E**T1–2, dorsal view **G** Scutellum, metanotum, propodeum, dorsal view **H, I** Wings **H** Fore **I** Hind.

Surface of metasternum convex, nucha surrounded by long radiating carinae (Figs 58F, 59G), median area on T2 as broad as long, edges of median area on T2 obscured by strong longitudinal stripes (Figs 58G, H, 59C, E), propodeal spiracle distally framed by faintly concave/wavy carina, inner margin of eyes diverging slightly at antennal sockets (Fig. 58B), distal antennal flagellomere longer than penultimate, median area between lateral ocelli without depression (Fig. 58D), in dorsal view, proximal half of propodeum more strongly curved (Figs 58F, 59G), petiole on T1 evenly narrowing distally (Figs 58G, 59E), dorsal outer depression on hind coxa present (Figs 58A, J, 59A, F), and fore wing with r vein curved, outer side of junction of r and 2RS veins forming a distinct stub (Figs 58K, 59H).

#### Coloration

(Fig. 58A–L). General body coloration shiny black except clypeus, mandibles, proximal half of scape, and distal half of pedicel yellow-brown; all antennal flagellomeres brown on both sides; glossa, maxillary and labial palps ivory; dorsally furrow of pronotum, distal corner of propleuron and tegulae yellow. Eyes and ocelli silver. Fore and middle legs yellow except fore claws brown and middle tarsomeres with yellow-brown tints; hind legs yellow except black coxae with apex yellow-brown (coxae with extensive yellow-brown coloration in the inner side), femora with a tiny brown area on the apex, distal half of tibiae brown and distally with a brown band; tarsomeres brown although basitarsus proximally with a yellow ring. Petiole on T1 black although proximal 1/3 yellow-brown/reddish, contours darkened, and sublateral areas yellow; T2 with median area dark brown, adjacent area narrow and brown, and lateral ends yellow; T3 with a trapezoidal brown area which proximal width coincides with the distal width of median plus adjacent areas on T2, and lateral ends yellow; T4 and beyond completely dark brown; distally each tergum with a yellowish transparent band. In lateral view, T1–3 completely yellow; T4 yellow, but dorsally brown, extent of brown area remains almost constant in each tergum. S1-4 completely yellow; penultimate sternum and hypopygium yellow, medially brown/yellow-brown.

#### Description.

**Head** (Fig. 58A–D). Head rounded with pubescence long and dense. Proximal three antennal flagellomeres longer than wide (0.27:0.09, 0.27:0.09, 0.28:0.09), distal antennal flagellomere longer than penultimate (0.16:0.06, 0.12:0.06), antenna longer than body (3.84, 3.03); antennal scrobes-frons shallow. Face convex, finely punctate-lacunose, interspaces wavy and longitudinal median carina present. Frons smooth. Temple wide with punctate sculpture and interspaces wavy. Inner margin of eyes diverging slightly at antennal sockets; in lateral view, eye anteriorly convex and posteriorly straight. POL shorter than OOL (0.11, 0.13). Malar suture absent or difficult to see. Median area between lateral ocelli without depression. Vertex laterally pointed or nearly so and dorsally wide.

**Mesosoma** (Fig. 58A, E, F, I). Mesosoma dorsoventrally convex. Distal 1/3 of mesoscutum with lateral margin slightly dented, punctation distinct throughout, and interspaces wavy/lacunose. Scutellum long and slender, apex sloped and fused with BS, scutellar punctation scattered throughout, in profile scutellum flat and on same plane as mesoscutum, but not in the same plane; phragma of the scutellum partially exposed; BS only very partially overlapping the MPM; ATS demilune entirely covered by parallel carinae; dorsal ATS groove with semicircular/parallel carinae. Transscutal articulation with small and homogeneous foveae, area just behind transscutal articulation depressed centrally and with same kind of sculpture as mesoscutum. Metanotum with BM convex; MPM oval/circular with a short proximal carina; AFM without setiferous lobes and not as well delineated as PFM; PFM thick, smooth and with lateral ends rounded; ATM proximally with sculpture distally without a well delimited smooth area. Propodeum without median longitudinal carina, proximal half curved with fine sculpture and distal half with a mix of coarse sculpture and rugae; distal edge of propodeum with a flange at each side and without stubs; propodeal spiracle distally framed by faintly concave/wavy carina; nucha surrounded by long radiating carinae. Pronotum with a distinct dorsal furrow, dorsally with a well-defined smooth band; central area of pronotum and dorsal furrow smooth, but ventral furrow with short parallel carinae. Propleuron with fine punctations throughout and dorsally without a carina. Metasternum convex. Contour of mesopleuron convex; precoxal groove deep, smooth and shiny; epicnemial ridge convex, teardrop-shaped.

**Legs** (Fig. 58A). Ventral margin of fore telotarsus excavated with conspicuous curved seta over this excavation, fore telotarsus almost same width throughout and longer than fourth tarsomere (0.17, 0.09). Dorsal half of hind coxa with scattered punctation, ventral half with dense punctation and dorsal outer depression present. Inner spur of hind tibia longer than outer spur (0.27, 0.22), entire surface of hind tibia with dense strong spines clearly differentiated by color and length. Hind telotarsus longer than fourth tarsomere (0.20, 0.15).

**Wings** (Fig. 58K, L). Fore wing with r vein slightly curved; 2RS vein slightly convex to convex; r and 2RS veins forming a weak, even curve at their junction and outer side of junction forming a slight stub; 2M vein slightly curved/swollen; distally fore wing [where spectral veins are] with microtrichiae more densely concentrated than the rest of the wing; anal cell 1/3 proximally lacking microtrichiae; subbasal cell with microtrichiae virtually throughout; veins 2CUa and 2CUb completely spectral; vein 2 cu-a absent; vein 2-1A proximally tubular and distally spectral, although sometimes difficult to see; tubular vein 1 cu-a straight, incomplete/broken and not reaching the edge of 1-1A vein. Hind wing with vannal lobe narrow, subdistally and subproximally evenly convex, and setae evenly scattered in the margin.

**Metasoma** (Fig. 58A, G, H, J). Metasoma laterally compressed. Petiole on T1 with a mix of fine rugae and coarse sculpture over most of the surface, evenly narrowing distally (length 0.40, maximum width 0.21, minimum width 0.10) and with scattered pubescence on distal half. Lateral grooves clearly defined and reaching the distal edge of T2 (length median area 0.20, length T2 0.20), edges of median area obscured by strong longitudinal stripes, median area as broad as long (length 0.20, maximum width 0.21, minimum width 0.10); T2 with scattered pubescence throughout. T3 longer than T2 (0.23, 0.20) and with pubescence more notorious in distal half. Pubescence on hypopygium dense.

**Cocoons.** Unknown.

#### Male

(Fig. 59A–I). Similar in coloration and shape to female.

#### Etymology.

Claudia Martinez is a Colombian entomologist whose research was focused on Carabidae (Coleoptera).

#### Distribution.

Parasitized caterpillars were collected in Ecuador, Napo, Yanayacu Biological Station (San Benjamin Forest, Sendero Macuculoma, Sendero Stream Trail, and Yanayacu Road), during April and December 2008 and March and April 2009 at 2,163 m in cloud forest.

#### Biology.

The lifestyle of this parasitoid species is gregarious.

#### Host.

Undetermined species of Geometridae feed on Iiexaffyurumanguinis (Aquifoliaceae) and undetermined species of Celastraceae. Caterpillars were collected in first, third, and fourth instar.

### Glyptapanteles corriemoreauae

Taxon classificationAnimaliaHymenopteraBraconidae

Arias-Penna, sp. nov.

http://zoobank.org/CF8A4CA6-35EC-4AC0-81E5-028C1C566AFF

Figs 60 , 61

#### Female.

Body length 2.27 mm, antenna length 2.73 mm, fore wing length 2.58 mm.

#### Type material.

**Holotype**: COSTA RICA • 1♀; 06-SRNP-36358, DHJPAR0012332; Área de Conservación Guanacaste, Guanacaste, Sector Cacao, Sendero Nayo; cloud forest; 1,090 m; 10.92446, -85.46953; 13.x.2006; Harry Ramirez leg.; caterpillar collected in fifth instar; black elongate non-fuzzy cocoons adhered to caterpillar back formed on 20.x.2006; adult parasitoids emerged on 30.x.2006; (CNC). **Paratypes.** • 14 (4♀, 3♂) (7♀, 0♂); 06-SRNP-36358, DHJPAR0012332; same data as for holotype; (CNC).

#### Other material.

**Reared material.** COSTA RICA: *Área de Conservación Guanacaste*, *Guanacaste*, *Sector Cacao*, *Sendero Arenales*: • 32 (4♀, 1♂) (27♀, 0♂); 03-SRNP-23247, DHJPAR0001471; cloud forest; 1,080 m; 10.92471, -85.46738; 09.x.2003; Harry Ramirez leg.; caterpillar collected in fourth instar; black cylindrical cocoons, clustered in two groups, no threads, adhered to the leaf substrate together, cocoons formed on 16.x.2003; adult parasitoids emerged on 26.x.2003. • 32 (4♀, 1♂) (27♀, 0♂); 03-SRNP-23247, DHJPAR0001471; same data as for preceding. • 17 (4♀, 4♂) (7♀, 2♂); 03-SRNP-23206, DHJPAR0000042; same data as for preceding except: 07.x.2003; caterpillar collected in fifth instar; cocoons formed on 11.x.2003; adult parasitoids emerged on 18.x.2003. • 26 (4♀, 1♂) (21♀, 0♂); 03-SRNP-23245, DHJPAR0001450; same data as for preceding except: caterpillar collected in fifth instar; caterpillar still very much alive, but when pinched, did not try to bit fingers; cocoons formed on 18.x.2003; adult parasitoids emerged on 28.x.2003. • 19 (3♀, 0♂) (16♀, 0♂); 03-SRNP-23341, DHJPAR0001449; same data as for preceding except: 10.x.2003; Mariano Pereira leg.; caterpillar collected in second instar; black cocoons formed on 29.x.2003; adult parasitoids emerged on 07.xi.2003; a yellow ant with the cocoons. • 19 (3♀, 1♂) (15♀, 0♂); 03-SRNP-23342, DHJPAR0001462; same data as for preceding except: 10.x.2003; Mariano Pereira leg.; caterpillar collected in second instar; cocoons formed on 29.x.2003; adult parasitoids emerged on 05.xi.2003. • 17 (4♀, 2♂) (11♀, 0♂); 03-SRNP-23344, DHJPAR0000267; same data as for preceding except: 10.x.2003; Mariano Pereira leg.; caterpillar collected in second instar; black cocoons formed on 27.x.2003; adult parasitoids emerged on 05.xi.2003. • 15 (3♀, 3♂) (9♀, 0♂); 07-SRNP-36191, DHJPAR0020265; same data as for preceding except: 06.vii.2007; black cocoons adhered to the larval cuticle and formed on 20.vii.2007; adult parasitoids emerged on 31.vii.2007. • 6 (1♀, 1♂) (3♀, 1♂); 10-SRNP-35602, DHJPAR0040420, same data as for preceding except: 29.vii.2010, caterpillar collected in third instar; dark cocoons formed on 20.viii.2010; adult parasitoids emerged on 25.viii.2010.

*Área de Conservación Guanacaste*, *Guanacaste*, *Sector Cacao*, *Sendero Nayo*: • 36 (0♀, 5♂) (0♀, 31♂); 06-SRNP-36009, DHJPAR0012330; cloud forest; 1,090 m; 10.92446, -85.46953; 19.viii.2006; Dunia García leg.; black cocoons adhered together to the larval cuticle and maybe the leaf next to it, formed on 05.ix.2006; adult parasitoids emerged on 11.ix.2006.

#### Diagnosis.

**Figure 60.**
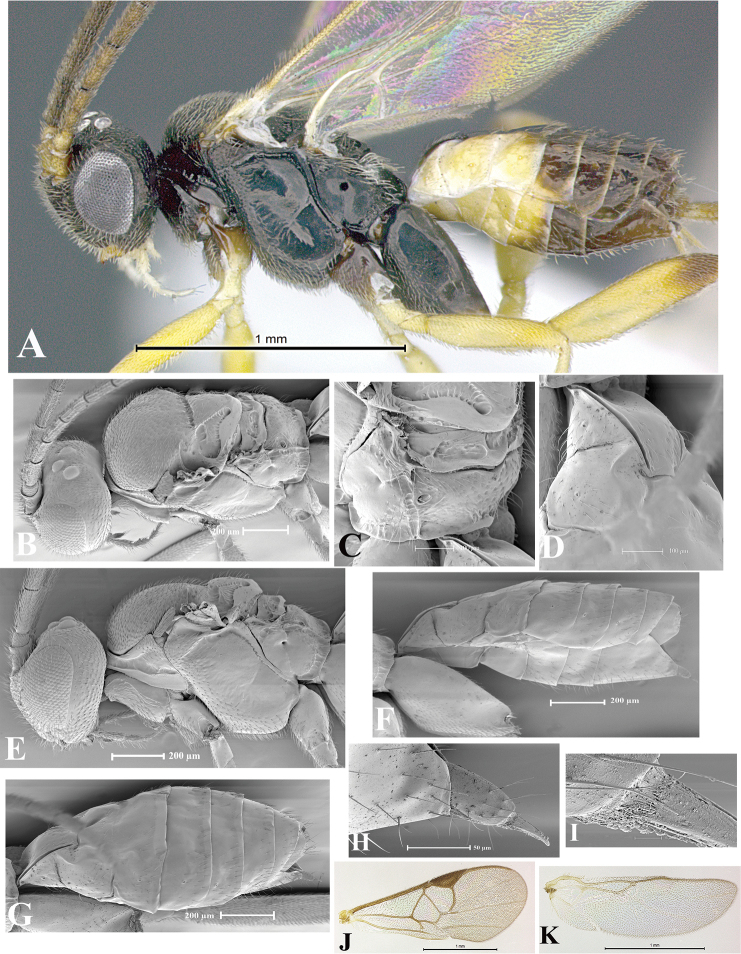
*Glyptapantelescorriemoreauae* sp. nov. female 03-SRNP-23206 DHJPAR0000042, 06-SRNP-36358 DHJPAR0012332 **A** Habitus **B, E** Head, mesosoma **B** Dorsolateral view **E** Lateral view **C** Metanotum, propodeum, dorsolateral view **D**T1–2, laterodorsal view **F, G** Metasoma **F** Lateral view **G** Dorsolateral view **H, I** Genitalia **H** Genitalia: hypopygium, ovipositor, ovipositor sheaths, lateral view **I** Ovipositor sheaths detail **J, K** Wings **J** Fore **K** Hind.

**Figure 61.**
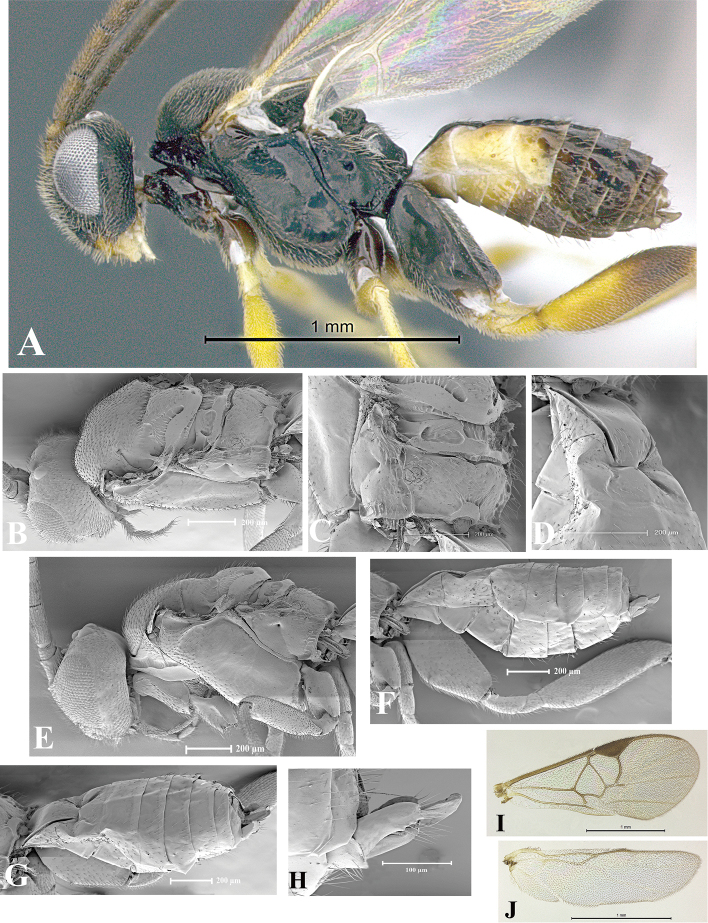
*Glyptapantelescorriemoreauae* sp. nov. male 03-SRNP-23206 DHJPAR0000042, 06-SRNP-36358 DHJPAR0012332 **A** Habitus **B, E** Head, Mesosoma **B** Laterodorsal view **E** Lateral view **C** Metanotum, propodeum, dorsolateral view **D**T1–2, dorsolateral view **F, G** Metasoma **F** Lateral view **G** Dorsolateral view **H** Genitalia: parameres, lateral view **I, J** Wings **I** Fore **J** Hind.

Scutellar punctation distinctly throughout (Figs 60B, 61B), distal antennal flagellomere subequal in length with penultimate, inner margin of eyes diverging slightly at antennal sockets, phragma of the scutellum partially exposed (Figs 60B, C, 61B, C), fore wing with vein 2-1A proximally tubular, distally spectral, 2RS vein straight, outer side of junction of r and 2RS veins not forming a stub (Figs 60J, 61I), propleuron with fine rugae (Figs 60A, E, 61A, E), mesoscutum punctate throughout (Figs 60B, 61B), anteroventral contour of mesopleuron straight/angulate or nearly so (Figs 60A, E, 61A, E), petiole on T1 distally with lateral margins relatively straight (Figs 60D, G, 61D, G), propodeum without median longitudinal carina, propodeal spiracle without distal carina (Figs 60B, C, 61B, C), nucha surrounded by very short radiating carinae (Figs 60B, C, 61B, C), antenna longer than body, and lateral grooves delimiting the median area on T2 distally losing definition (Figs 60D, G, 61D, G).

#### Coloration

(Fig. 60A). General body coloration brown-black except scape, pedicel, labrum and mandibles yellow-brown; glossa, maxillary and labial palps, and tegulae yellow. Eyes gray/silver and ocelli silver. Fore and middle legs yellow except coxae and claws brown; hind legs yellow except coxae, apex of femorae and tibiae, and tarsomeres brown. Petiole on T1 with two colorations: proximal 3/4 yellow-brown with edges distinctively brown and distal 1/4 brown-black, and sublateral areas light yellow; T2 with median area brown-black, adjacent area yellow-brown and lateral ends light yellow; T3 mostly brown, but lateral ends light yellow; T4 and beyond brown; distally each tergum with a narrow whitish transparent band. In lateral view, T1–3 completely yellow; T4 and beyond brown. S1–3 completely yellow; S4 yellow, medially brown; penultimate sternum and hypopygium completely brown; ovipositor sheath brown.

#### Description.

**Head** (Fig. 60A, B, E). Head triangular with pubescence long and dense. Proximal three antennal flagellomeres longer than wide (0.21:0.05, 0.20:0.0, 0.20:0.05), distal antennal flagellomere subequal in length with penultimate (0.11:0.05, 0.10:0.05), antenna longer than body (2.73, 2.27); antennal scrobes-frons shallow. Face with scattered finely punctate, interspaces wavy, distal half dented only laterally, and longitudinal median carina present. Frons punctate. Temple wide, punctate and interspaces wavy. Inner margin of eyes diverging slightly at antennal sockets; in lateral view, eye anteriorly convex and posteriorly straight. POL shorter than OOL (0.08, 0.12). Malar suture present. Median area between lateral ocelli slightly depressed. Vertex laterally rounded and dorsally wide.

**Mesosoma** (Fig. 60A–C, E). Mesosoma dorsoventrally convex. Mesoscutum 1/4 distal with a central dent, punctation distinct throughout and interspaces wavy/lacunose. Scutellum triangular, apex sloped and fused with BS, scutellar punctation distinct throughout, in profile scutellum slightly convex, but on same plane as mesoscutum; phragma of the scutellum partially exposed; BS only very partially overlapping the MPM; ATS demilune with complete undulate/reticulate carinae; dorsal ATS groove with carinae only proximally. Transscutal articulation with small and heterogeneous foveae, area just behind transscutal articulation depressed centrally with same kind of sculpture as mesoscutum. Metanotum with BM wider than PFM (clearly differentiated); MPM circular without median longitudinal carina; AFM without setiferous lobes and not as well delineated as PFM; PFM thick and smooth; ATM proximally with semircular/undulate carina and distally smooth. Propodeum without median longitudinal carina, proximal half weakly curved with medium-sized sculpture and distal half with medium-sized punctation; distal edge of propodeum with a flange at each side and without stubs; propodeal spiracle without distal carina; nucha surrounded by very short radiating carinae. Pronotum with a distinct dorsal furrow, dorsally with a well-defined smooth band; central area of pronotum and dorsal furrow smooth, but ventral furrow with short parallel carinae. Propleuron with fine rugae and dorsally with a carina. Metasternum flat or nearly so. Contour of mesopleuron straight/angulate or nearly so; precoxal groove deep with faintly transverse lineate sculpture; epicnemial ridge convex, teardrop-shaped.

**Legs.** Ventral margin of fore telotarsus entire without seta, fore telotarsus almost same width throughout and longer than fourth tarsomere (0.10, 0.08). Hind coxa with punctation only on ventral surface and dorsal outer depression present. Inner spur of hind tibia longer than outer spur (0.25, 0.18); entire surface of hind tibia with dense strong spines clearly differentiated by color and length. Hind telotarsus as equal in length as fourth tarsomere (0.11, 0.11).

**Wings** (Fig. 60J, K). Fore wing with r vein slightly curved; 2RS vein straight; r and 2RS veins forming a weak, even curve at their junction and outer side of junction not forming a stub; 2M vein slightly curved/swollen; distally fore wing [where spectral veins are] with microtrichiae more densely concentrated than the rest of the wing; anal cell 1/3 proximally lacking microtrichiae; subbasal cell with microtrichiae virtually throughout; veins 2CUa and 2CUb completely spectral; vein 2 cu-a absent; vein 2-1A proximally tubular and distally spectral, although sometimes difficult to see; tubular vein 1 cu-a curved, incomplete/broken and not reaching the edge of 1-1A vein. Hind wing with vannal lobe narrow, subdistally evenly convex, subproximally straightened, and setae present only proximally.

**Metasoma** (Fig. 60A, D, F–I). Metasoma laterally compressed. Petiole on T1 finely sculptured only distally, evenly narrowing distally (length 0.33, maximum width 0.17, minimum width 0.08), and with scattered pubescence concentrated in the first distal third. Lateral grooves delimiting the median area on T2 distally losing definition (length median area 0.10, length T2 0.15), edges of median area polished and lateral grooves deep, median area broader than long (length 0.10, maximum width 0.20, minimum width 0.08); T2 with scattered pubescence only distally. T3 longer than T2 (0.21, 0.15) and with scattered pubescence only distally. Pubescence on hypopygium dense.

**Cocoons** (Fig. 4X). Black oval cocoons with evenly smooth silk fibers. Cocoons, clustered in two groups, without threads, adhered to the leaf substrate or to the larval cuticle.

#### Male

(Fig. 61A–J). Similar in coloration and shape to female.

#### Etymology.

Corrie S. Moreau’s research is focuses on the factors that drive evolutionary diversification and how these factors have facilitated the ecological dominance of ants in almost all terrestrial ecosystems. Currently, she is the Curator of Entomology at Cornell University, Ithaca, NY, USA.

#### Distribution.

The parasitized caterpillars were collected in Costa Rica, ACG, Sector Cacao (Sendero Arenales and Sendero Nayo), during September-October 2003, August and October 2006, and July 2007 and 2010 at 760 m, 1,080 m, and 1,090 m in cloud forest.

#### Biology.

The lifestyle of this parasitoid species is gregarious.

#### Host.

*Euphyiacrispa* Druce (Geometridae: Larentiinae) (Fig. 4X) feeding on *Pleuropetalumsprucei* (Amaranthaceae). Caterpillars were collected in second, third, fourth, and fifth instar. Caterpillar still very much alive after parasitoid emerged, but when pinched, did not try to bite fingers.

### Glyptapanteles daveroubiki

Taxon classificationAnimaliaHymenopteraBraconidae

Arias-Penna, sp. nov.

http://zoobank.org/549FBD83-5318-44FC-8DE3-4906E0A95770

Figs 62 , 63

#### Female.

Body length 1.67 mm, antenna length 1.86 mm, fore wing length 1.81 mm.

#### Type material.

**Holotype**: COSTA RICA • 1♀; 88-SRNP-385, DHJPAR0000055; Área de Conservación Guanacaste, Guanacaste, Sector Santa Rosa, Bosque Humedo; dry forest; 290 m; 10.85145, -85.60801; 21.vi.1988; gusaneros leg.; caterpillar collected in fifth instar; beige, elongate cocoons glued side-by-side (like tettigoniid eggs), tight spun silk; adult parasitoids emerged on 26.vi.1998; (CNC). **Paratypes.** • 49 (4♀, 4♂) (34♀, 7♂); 88-SRNP-385, DHJPAR0000055; same data as for holotype; (CNC).

#### Diagnosis.

**Figure 62.**
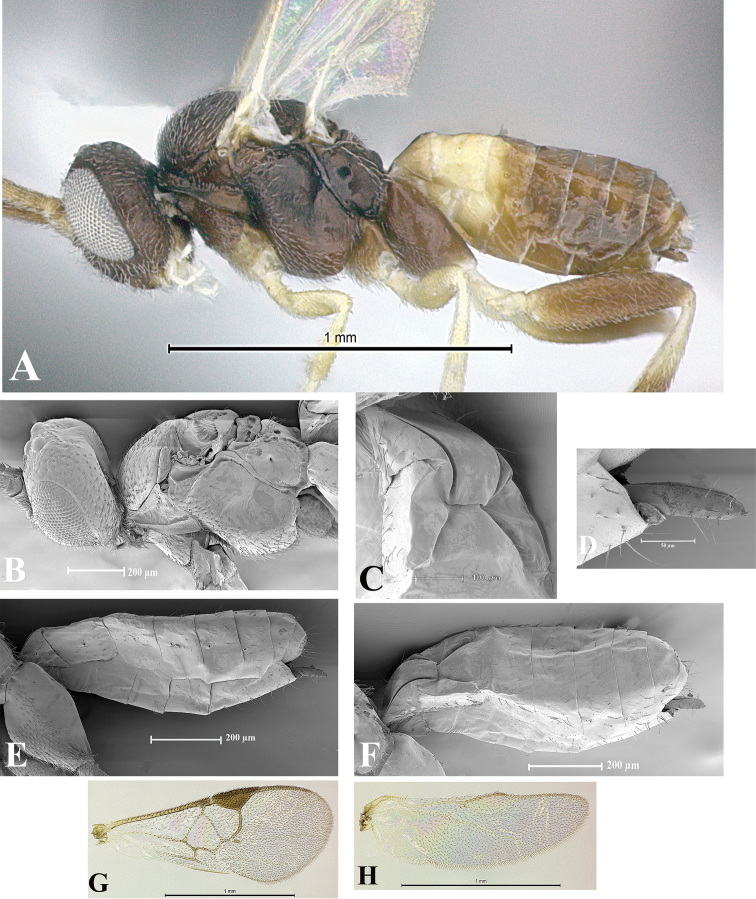
*Glyptapantelesdaveroubiki* sp. nov. female 88-SRNP-385 DHJPAR0000055 **A** Habitus **B** Head, mesosoma, lateral view **C**T1–2, dorsolateral view **D** Genitalia: hypopygium, ovipositor sheaths, lateral view **E, F** Metasoma **E** Lateral view **F** Dorsolateral view **G, H** Wings **G** Fore **H** Hind.

**Figure 63.**
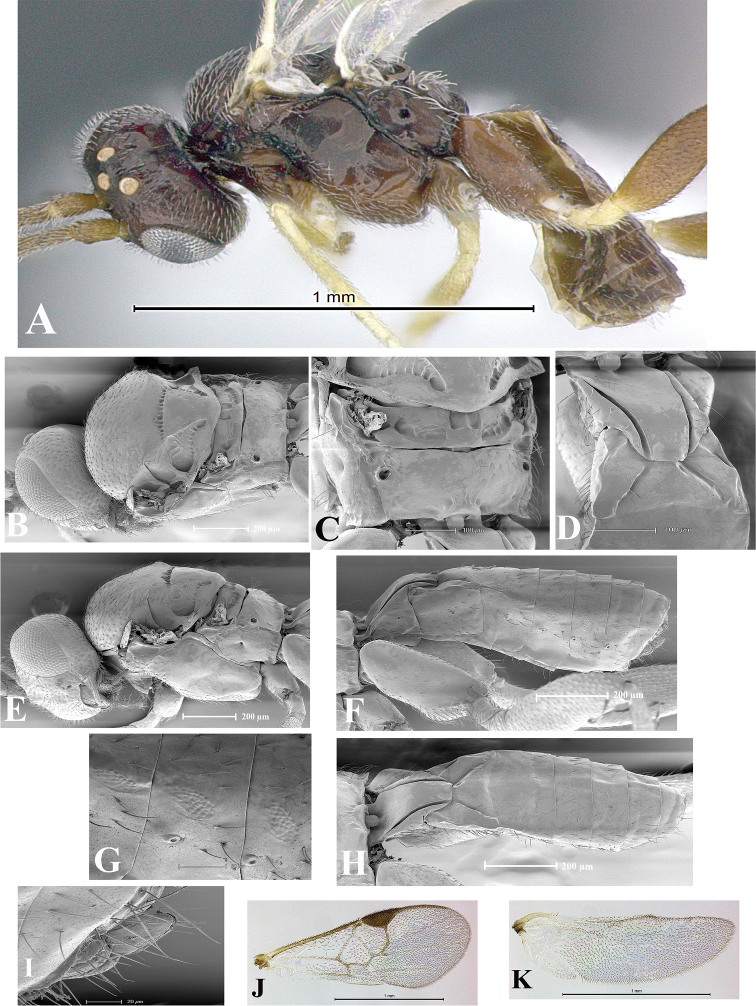
*Glyptapantelesdaveroubiki* sp. nov. male 88-SRNP-385 DHJPAR0000055 **A** Habitus **B, E** Head, mesosoma **B** dorsalateral view **E** Lateral view **C** Metanotum, propodeum, dorsal view **D**T1–2, dorsal view **F–H** Metasoma **F** Lateral view **G** Metasoma glands **H** Dorsal view **I** Genitalia: parameres, lateral view **J, K** Wings **J** Fore **K** Hind.

Median area between lateral ocelli slightly depressed (Figs 62B, 63A), distal antennal flagellomere subequal in length with penultimate, petiole on T1 completely smooth and polished, with faint, satin-like sheen, parallel-sided in proximal half, then narrowing (Figs 62C, F, 63D, H), inner margin of eyes diverging slightly at antennal sockets, propodeum without median longitudinal carina (Figs 62C, 63C), lateral grooves delimiting the median area on T2 clearly defined and reaching the distal edge of T2 (Figs 62C, F, 63D, H), and fore wing with outer side of junction of r and 2RS veins not forming a stub (Figs 62G, 63J).

#### Coloration

(Fig. 62A). General body coloration light brown although some areas on body are light brown/reddish as propleuron, both dorsal and ventral furrows of pronotum, ventral edge of mesopleuron, mesosternum, epicnemial ridge, metepimeron, distal corners of mesoscutum, lateral edges of scutellum, lunules, BS, PFM, BM and propleuron; scape, pedicel, labrum and mandibles yellow-brown; maxillary and labial palps, and glossa yellow. Eyes gray/silver and ocelli yellowish. Fore and middle legs yellow except yellow-brown coxae and brown claws; hind legs light brown/brown except the following yellow areas: apex of coxae, trochanter, trochantellus, 1/4 proximal of tibiae, both tibial spurs and basitarsus with a proximal ring. Petiole on T1 light brown, contours darkened, and sublateral areas yellow; T2 with median area light brown, and lateral ends yellow; T3 light brown with proximal corners yellow; T4 and beyond completely light brown; distally each tergum with a narrow whitish/yellow transparent band. In lateral view, T1–2 completely yellow; T3 proximal half yellow, distal half light brown; T4 and beyond completely light brown. S1–3 yellow, medially with some light brown spots; S4 and beyond completely light brown.

#### Description.

**Head** (Fig. 62A, B). Head rounded with pubescence long and dense. Proximal three antennal flagellomeres longer than wide (0.15:0.05, 0.15:0.05, 0.14:0.05), distal antennal flagellomere subequal in length with penultimate (0.08:0.05, 0.07:0.05), antenna longer than body (1.86, 1.67); antennal scrobes-frons shallow. Face finely punctate, interspaces smooth, distal half dented only laterally and longitudinal median carina present. Frons smooth. Temple wide, punctate and interspaces with microsculpture. Inner margin of eyes diverging slightly at antennal sockets; in lateral view, eye anteriorly convex and posteriorly straight. POL shorter than OOL (0.07, 0.11). Malar suture present. Median area between lateral ocelli slightly depressed. Vertex laterally rounded and dorsally wide.

**Mesosoma** (Fig. 62A, B). Mesosoma dorsoventrally convex. Mesoscutum 1/4 distal with a central dent, punctation distinct proximally, but absent/dispersed distally, interspaces with microsculpture. Scutellum triangular, apex sloped and fused with BS, scutellar punctation distinct peripherally, absent centrally, in profile scutellum flat and on same plane as mesoscutum, phragma of the scutellum partially exposed; BS only very partially overlapping the MPM; ATS demilune with complete undulate/reticulate carinae; dorsal ATS groove with semicircular/parallel carinae. Transscutal articulation with small and homogeneous foveae, area just behind transscutal articulation smooth, shiny and depressed centrally. Metanotum with BM wider than PFM (clearly differentiated); MPM circular and bisected by a median longitudinal carina; AFM without setiferous lobes and not as well delineated as PFM; PFM thick and smooth; ATM proximally with semircular/undulate carina and distally smooth. Propodeum without median longitudinal carina, relatively polished, proximal half weakly curved; distal edge of propodeum with a flange at each side and without stubs; propodeal spiracle without distal carina; nucha surrounded by very short radiating carinae. Pronotum with a distinct dorsal furrow, dorsally with a well-defined smooth band; central area of pronotum and dorsal furrow smooth, but ventral furrow with short parallel carinae. Propleuron with fine rugae and dorsally without a carina. Metasternum flat or nearly so. Contour of mesopleuron straight/angulate or nearly so; pecoxal groove distinct with faintly transverse lineate sculpture; epicnemial ridge convex, teardrop-shaped.

**Legs.** Ventral margin of fore telotarsus entire, but with a tiny curved seta, fore telotarsus almost same width throughout and longer than fourth tarsomere (0.10, 0.05). Hind coxa with punctation only on ventral surface and dorsal outer depression present. Inner spur of hind tibia longer than outer spur (0.15, 0.12), entire surface of hind tibia with dense strong spines clearly differentiated by color and length. Hind telotarsus longer than fourth tarsomere (0.10, 0.07).

**Wings** (Fig. 62G, H). Fore wing with r vein slightly curved; 2RS vein slightly convex to convex; r and 2RS veins forming a weak, even curve at their junction and outer side of junction not forming a stub; 2M vein slightly curved/swollen; distally fore wing [where spectral veins are] with microtrichiae more densely concentrated than the rest of the wing; anal cell 1/3 proximally lacking microtrichiae; subbasal cell with a small smooth area, vein 2CUa absent and vein 2CUb spectral; vein 2 cu-a absent; vein 2-1A present only proximally as tubular vein; tubular vein 1 cu-a curved, complete and touching the edge of 1-1A vein. Hind wing with vannal lobe narrow, subdistally evenly convex, subproximally straightened, and setae present only proximally.

**Metasoma** (Fig. 62A, C–F). Metasoma laterally compressed. Petiole on T1 completely smooth and polished, with faint, satin-like sheen, parallel-sided in proximal half and then narrowing (length 0.24, maximum width 0.10, minimum width 0.05), and with scattered pubescence concentrated in the first distal third. Lateral grooves delimiting the median area on T2 clearly defined and reaching the distal edge of T2 (length median area 0.09, length T2 0.09), edges of median area polished and lateral grooves deep, median area broader than long (length 0.09, maximum width 0.15, minimum width 0.05); T2 with scattered pubescence only distally. T3 longer than T2 (0.16, 0.09) and with scattered pubescence only distally. Pubescence on hypopygium dense.

**Cocoons.** Beige oval cocoons with silk fibers evenly smooth. Cocoons glued side-by-side.

#### Male

(Fig. 63A–K). Coloration and shape similar to that of female.

#### Etymology.

David (Dave) Ward Roubik works at the Smithsonian Tropical Research Institute, Panama City, Panama. He is interested in understanding how tropical bee communities change through monitoring populations of bees, plants, animals, and even microbes and molecules.

#### Distribution.

The parasitized caterpillar was collected in Costa Rica, ACG, Sector Santa Rosa (Bosque Humedo), during July 1998 at 290 m in dry forest.

#### Biology.

The lifestyle of this parasitoid species is gregarious.

#### Host.

Undetermined species of Noctuidae,, food plant was not reported. Caterpillar was collected in fifth instar.

### Glyptapanteles daveschindeli

Taxon classificationAnimaliaHymenopteraBraconidae

Arias-Penna, sp. nov.

http://zoobank.org/35E15D31-8CB5-47AF-9C6E-C2EA06921126

Figs 64 , 65

#### Female.

Body length 2.02 mm, antenna length 2.22 mm, fore wing length 1.96 mm.

#### Type material.

**Holotype**: COSTA RICA • 1♀; 08-SRNP-16708, DHJPAR0030699; Área de Conservación Guanacaste, Guanacaste, Sector Santa Rosa, Área administrativa; dry forest; 295 m; 10.83764, -85.61871; 14.xi.2008; Lucia Vargas leg.; caterpillar collected in fifth instar; cocoons adhered to caterpillar body in rings; adult parasitoids emerged on 26.xi.2008; (CNC). **Paratypes.** • 28 (2♀, 2♂) (25♀, 0♂); 08-SRNP-16708, DHJPAR0030699; same data as for holotype; (CNC).

#### Other material.

**Reared material.** COSTA RICA: *Área de Conservación Guanacaste*, *Guanacaste*, *Sector Santa Rosa*, *Bosque Humedo*: • 49 (6♀, 1♂) (42♀, 0♂); 93-SRNP-2251, DHJPAR0000071; dry forest; 290 m; 10.85145, -85.60801; 14.vi.1993; gusaneros leg.; caterpillar collected in fifth instar; brown/gray cocoons adhered laterally against caterpillar body in rings; adult parasitoids emerged on 16.vi.1993.

*Área de Conservación Guanacaste*, *Guanacaste*, *Sector Cacao*, *Sendero Guayabal*: • 8 (3♀, 0♂) (5♀, 0♂); 08-SRNP-45004, DHJPAR0020724; cloud forest; 500 m; 10.88571, -85.48184; 03.i.2008; Manuel Pereira leg.; caterpillar collected in third instar; brown cocoons adhered to the larval cuticle and formed on 24.i.2008; adult parasitoids emerged on 04.ii.2008.

#### Malaise-trapped material.

COSTA RICA: *Área de Conservación Guanacaste*, *Guanacaste*, *Sector Santa Rosa*, *Bosque San Emilio*: • 1 (1♀, 0♂) (0♀, 0♂); 99-SRNP-19008, DHJPAR0013385; dry forest; 300 m; 10.84389, -85.61384; Malaise trap; 10.v.1999; DH Janzen & W Hallwachs leg.

#### Diagnosis.

**Figure 64.**
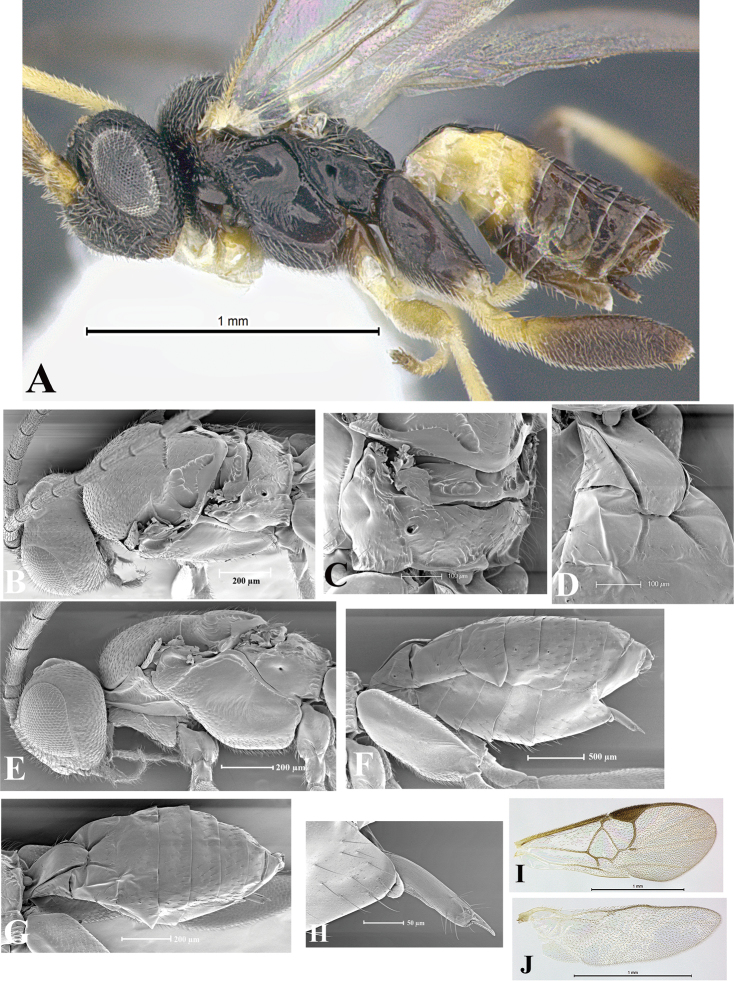
*Glyptapantelesdaveschindeli* sp. nov. female 93-SRNP-2251 DHJPAR0000071, 08-SRNP-16708 DHJPAR0030699 **A** Habitus **B, E** Head, mesosoma **B** Dorsolateral view **E** lateral view **C** Metanotum, propodeum, dorsolateral view **D**T1–2, dorsolateral view **F, G** Metasoma **F** Lateral view **G** Dorsolateral view **H** Genitalia: hypopygium, ovipositor, ovipositor sheaths, lateral view **I, J** Wings **I** Fore **J** Hind.

**Figure 65.**
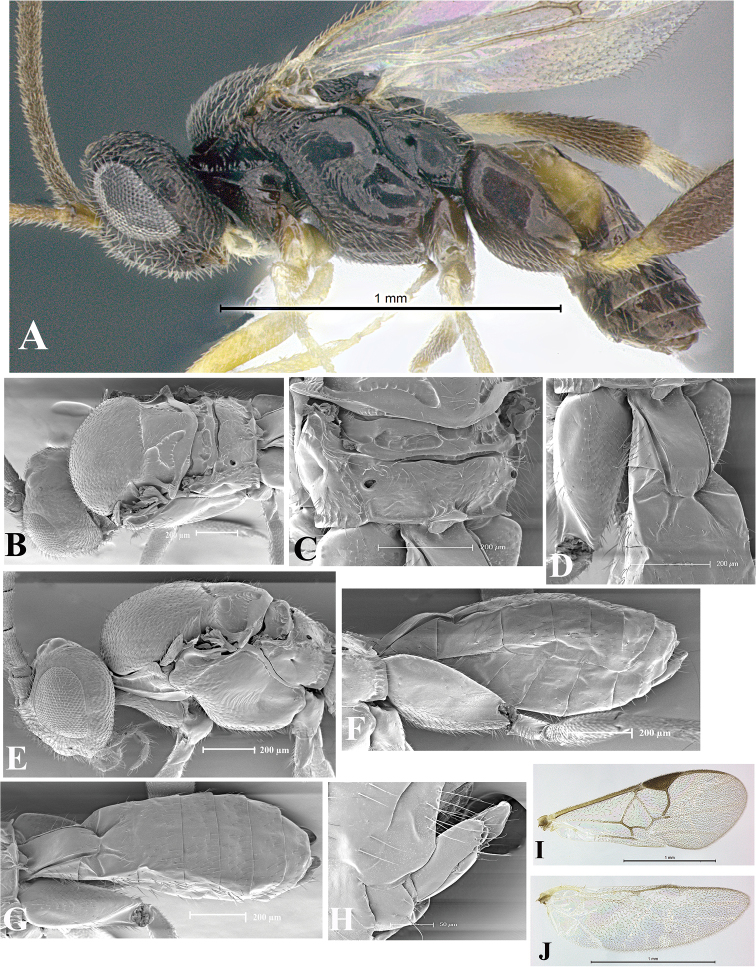
*Glyptapantelesdaveschindeli* sp. nov. male 93-SRNP-2251 DHJPAR0000071, 08-SRNP-16708 DHJPAR0030699 **A** Habitus **B, E** Head, mesosoma **B** Dorsolateral view **E** lateral view **C** Metanotum, propodeum, dorsolateral view **D**T1–2, dorsolateral view **F, G** Metasoma **F** Lateral view **G** Dorsolateral view **H** Genitalia: parameres, lateral view **I, J** Wings **I** Fore **J** Hind.

Mesoscutum punctation proximally distinct, but distally absent/dispersed (Figs 64B, 65B), phragma of the scutellum completely concealed (Figs 64C, 65C), antenna longer than body, ventral margin of fore telotarsus slightly excavated, scutellar punctation indistinct throughout (Figs 64B, 65B), propodeal spiracle without distal carina (Figs 64C, 65C), petiole on T1 distally with lateral margins relatively straight and finely sculptured only laterally (Figs 64D, G, 65D, G), surface of metasternum flat or nearly so, precoxal groove deep with lineate sculpture (Figs 64A, E, 65A, E), fore wing with vein 1 cu-a curved, r vein curved, outer side of junction of r and 2RS veins not forming a stub (Figs 64I, 65I), dorsal outer depression on hind coxa present (Figs 64A, F, 65A, F), inner margin of eyes diverging slightly at antennal sockets, propodeum without median longitudinal carina (Figs 64C, 65C), and lateral grooves delimiting the median area on T2 clearly defined and reaching the distal edge of T2 (Figs 64D, G, 65D, G).

#### Coloration

(Fig. 64A). General body coloration brown-black although some areas on body are light brown/reddish as clypeus, propleuron, both dorsal and ventral furrows of pronotum, epicnemial ridge, ventral edge of mesopleuron, distal corner of mesoscutum, lunules, BS, and PFM; scape, pedicel, labrum and mandibles yellow-brown; tegulae yellow; first five-six proximal antennal flagellomeres dorsally lighter (light brown) than ventrally (dark brown), remaining flagellomeres dark brown on both sides; glossa, maxillary and labial palps, and tegulae yellow. Fore legs yellow except brown claws; middle legs yellow except coxae and claws brown; hind legs brown except trochanter, trochantellus, proximal half of tibiae, and tibial spurs yellow, basitarsus with a proximal yellow ring, last two distal tarsomeres lost. Petiole on T1 with two colorations: proximal half brown-orange and distal half brown, contours darkened, and sublateral areas yellow-brown; T2 with median area brown, adjacent area yellow-brown and very narrow although proximal wide, and lateral ends yellow-brown; T3 extended brown, lateral ends narrow and yellow-brown; T4 and beyond completely brown; distally each tergum with a narrow whitish transparent band. In lateral view, T1–3 completely yellow; T4 and beyond completely brown. S1–3 completely yellow; S4 proximal half yellow, distal half brown; penultimate sternum and hypopygium completely brown.

#### Description.

**Head** (Fig. 64A, B) Head rounded with pubescence long and dense. Proximal three antennal flagellomeres longer than wide (0.16:0.05, 0.16:0.0, 0.16:0.05), distal antennal flagellomeres longer than penultimate (0.10:0.05, 0.08:0.05), antenna longer than body (2.22, 2.02); antennal scrobes-frons shallow. Face with dense fine punctations, interspaces wavy, distal half dented only laterally, and longitudinal median carina present. Frons smooth. Temple narrow, punctate and interspaces wavy. Inner margin of eyes diverging slightly at antennal sockets; in lateral view, eye anteriorly convex and posteriorly straight. POL shorter than OOL (0.09, 0.13). Malar suture present. Median area between lateral ocelli without depression. Vertex laterally rounded and dorsally wide.

**Mesosoma** (Fig. 64A–C, E). Mesosoma dorsoventrally convex. Distal 1/3 of mesoscutum with lateral margin slightly dented, punctation proximally distinct, but distally absent/dispersed, and interspaces with microsculpture. Scutellum triangular, apex sloped and fused with BS, scutellar punctation indistinct throughout, in profile scutellum flat and on same plane as mesoscutum, phragma of the scutellum completely concealed; BS only very partially overlapping the MPM; ATS demilune with short stubs delineating the area; dorsal ATS groove with semicircular/parallel carinae. Transscutal articulation with small and heterogeneous foveae, area just behind transscutal articulation with a smooth and shiny sloped transverse strip. Metanotum with BM wider than PFM (clearly differentiated); MPM circular without median longitudinal carina; AFM without setiferous lobes and not as well delineated as PFM; PFM thick and smooth; ATM proximally with semircular/undulate carina and distally smooth. Propodeum without median longitudinal carina, proximal half weakly curved with medium-sized sculpture and distal half with a shallow dent at each side of nucha or rugose; distal edge of propodeum with a flange at each side and without stubs; propodeal spiracle without distal carina; nucha surrounded by very short radiating carinae. Pronotum virtually without trace of dorsal furrow, dorsally with a well-defined smooth band; central area of pronotum smooth, but both dorsal and ventral furrows with short parallel carinae. Propleuron with fine punctations throughout and dorsally with a carina. Metasternum flat or nearly so. Contour of mesopleuron straight/angulate or nearly so; precoxal groove deep with faintly transverse lineate sculpture; epicnemial ridge convex, teardrop-shaped.

**Legs.** Ventral margin of fore telotarsus slightly excavated and with a tiny curved seta, fore telotarsus almost same width throughout and longer than fourth tarsomere (0.10, 0.06). Hind coxa with punctation only on ventral surface and dorsal outer depression present. Inner spur of hind tibia longer than outer spur (0.20, 0.12), entire surface of hind tibia with dense strong spines clearly differentiated by color and length.

**Wings** (Fig. 64I, J). Fore wing with r vein curved; 2RS vein straight; r and 2RS veins forming a weak, even curve at their junction and outer side of junction not forming a stub; 2M vein slightly curved/swollen; distally fore wing [where spectral veins are] with microtrichiae more densely concentrated than the rest of the wing; anal cell 1/3 proximally lacking microtrichiae; subbasal cell proximal half smooth; vein 2CUa absent and vein 2CUb spectral; vein 2 cu-a absent; vein 2-1A present only proximally as tubular vein; tubular vein 1 cu-a curved and complete, but junction with 1-1A vein spectral. Hind wing with vannal lobe wide, subdistally and subproximally straightened, and setae present only proximally.

**Metasoma** (Fig. 64A, D, F–H). Metasoma laterally compressed. Petiole on T1 finely sculptured only laterally, virtually parallel-sided over most of length, but narrowing over distal 1/3 (length 0.27, maximum width 0.14, minimum width 0.06), and with scattered pubescence concentrated in the first distal third. Lateral grooves delimiting the median area on T2 clearly defined and reaching the distal edge of T2 (length median area 0.12, length T2 0.12), edges of median area polished and lateral grooves deep, median area broader than long (length 0.12, maximum width 0.18, minimum width 0.06); T2 with a distinctive row of pubescence only at the distal margin. T3 longer than T2 (0.17, 0.12) and with scattered pubescence throughout. Pubescence on hypopygium dense.

**Cocoons** (Fig. 4U). Brown or gray oval cocoon with evenly smooth silk fibers. Cocoons were adhered laterally against the caterpillar’s body in rings.

#### Comments.

Some females from the same sample as holotype do not exhibit brown-orange petiole at the proximal part; instead the petiole is completely brown. The proximal half of propodeum with rugae at each side of the nucha.

#### Male

(Fig. 65A–J). Similar in coloration to female, although the mesosoma is stouter and more robust than female.

#### Etymology.

David (Dave) Schindel is the executive secretary of the Consortium for the Barcode of Life (CBOL). He works at the National Museum of Natural History, Washington, DC, USA.

#### Distribution.

The parasitized caterpillars were collected in Costa Rica, ACG, Sector Cacao (Sendero Guayabal) and Sector Santa Rosa (Área administrative and Bosque Humedo), during June 1993 and January and November 2008 at 290 m, 295 m, and 500 m in dry forest and cloud forest. The adult parasitoids were Malaise-trapped in Costa Rica, ACG, Sector Santa Rosa (Bosque San Emilio), during May 1999 at 300 m in dry forest.

#### Biology.

The lifestyle of this parasitoid species is gregarious.

#### Host.

*Oxydiaapidania* Cramer (Geometridae: Ennominae) feeding on *Ingapunctata* (Fabaceae), *O.vesulia* (Cramer) (Fig. 4U) feeding on *Spondiaspurpurea*, introduced species, (Anacardiaceae), and *Oxydia* sp. although food plant was not reported. Caterpillars were collected in third and fifth instar.

### Glyptapanteles davesmithi

Taxon classificationAnimaliaHymenopteraBraconidae

Arias-Penna, sp. nov.

http://zoobank.org/A71C1EF7-7CF7-489E-A68F-96B8B88243E7

Figs 66 , 67

#### Female.

Body length 2.42 mm, antenna length 2.63 mm, fore wing length 2.27 mm.

#### Type material.

**Holotype**: COSTA RICA • 1♀; 01-SRNP-6841, DHJPAR0000021; Área de Conservación Guanacaste, Guanacaste, Sector Cacao, Estación Cacao; cloud forest; 1,150 m; 10.92691, -85.46822; 22.v.2001; Harry Ramirez leg.; a row of brown cordwood cocoons on each side of the caterpillar and adhered to the leaf substrate, cocoons formed on 24.v.2001; adult parasitoids emerged on 04.vi.2001; (CNC). **Paratypes.** • 17 (4♀, 1♂) (12♀, 0♂); 01-SRNP-6841, DHJPAR0000021; same data as for holotype; (CNC).

#### Other material.

**Reared material**. COSTA RICA: *Área de Conservación Guanacaste*, *Guanacaste*, *Sector Cacao*, *Senderoderrumbe*: • 11 (5♀, 4♂) (2♀, 0♂); 02-SRNP-23076, DHJPAR0000023; cloud forest; 1,220 m; 10.92918, -85.46426; 08.vii.2002; Freddy Quesada leg.; caterpillar collected in fourth instar; white small cocoons forming two rows of cordwood on each side of cadaver, cocoons adhered to the leaf substrate; adult parasitoids emerged on 24.vii.2002. • 7 (5♀, 1♂) (1♀, 0♂); 02-SRNP-23078, DHJPAR0000024; same data as for preceding except: scattered small white cocoons adhered to the leaf substrate; adult parasitoids emerged on 21.vii.2002. • 6 (2♀, 0♂) (4♀, 0♂); 02-SRNP-23079, DHJPAR0001468; same data as for preceding except: small white cocoons separate from each other and adhered to the leaf substrate, cocoons formed on 17.vii.2002; adult parasitoids emerged on 24.vii.2002. • 7 (3♀, 3♂) (1♀, 0♂); 02-SRNP-9987, DHJPAR0000034; same data as for preceding except: 05.vii.2002; Harry Ramírez leg.; single row of cordwood cocoons on each side of caterpillar and adhered to the leaf substrate; adult parasitoids emerged on 22.vii.2002.

*Área de Conservación Guanacaste*, *Guanacaste*, *Sector Pitilla*, *Sendero Orosilito*: • 11 (3♀, 2♂) (6♀, 0♂); 03-SRNP-37399, DHJPAR0000268; rain forest; 900 m; 10.98332, -85.43623; 11.xii.2003; Calixto Moraga leg.; each one cocoon width apart in a single row cordwood parallel to the body on each side of the caterpillar, each cocoon at right angles to the long axis of the caterpillar and adhered to the leaf substrate, cocoons formed on 29.xii.2003; adult parasitoids emerged on 04.i.2004.

*Área de Conservación Guanacaste*, *Guanacaste*, *Sector Pitilla*, *Sendero Evangelista*: • 8 (3♀, 1♂) (4♀, 0♂); 11-SRNP-32121, DHJPAR0045123; rain forest; 660 m; 10.98680, -85.42083; 03.viii.2011; Freddy Quesada leg.; caterpillar collected in fifth instar; cocoons adhered to larva and leaf substrate and formed on 09.viii.2011; adult parasitoids emerged on 13.viii.2011.

#### Diagnosis.

**Figure 66.**
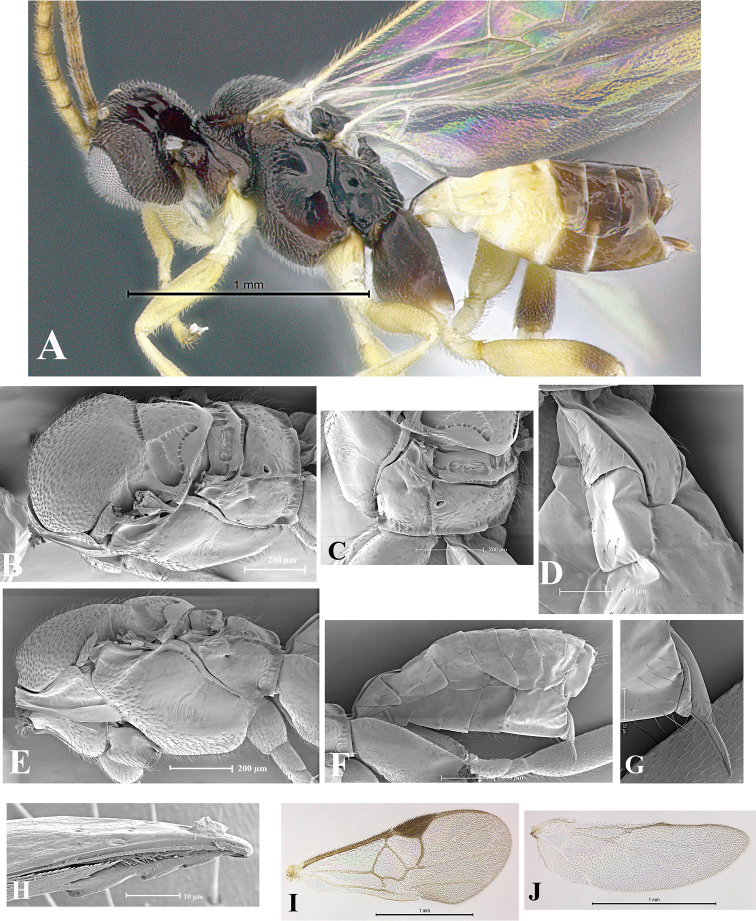
*Glyptapantelesdavesmithi* sp. nov. female 01-SRNP-6841 DHJPAR0000021, 02-SRNP-9987 DHJPAR0000034 **A** Habitus **B, E** Mesosoma **B** Dorsolateral view **E** lateral view **C** Metanotum, propodeum, laterodorsal view **D**T1–3, laterodorsal view **F** Metasoma, lateral view **G, H** Genitalia **G** Hypopygium, ovipositor, ovipositor sheaths, lateral view **H** Ovipositor detail **I, J** Wings **I** Fore **J** Hind.

**Figure 67.**
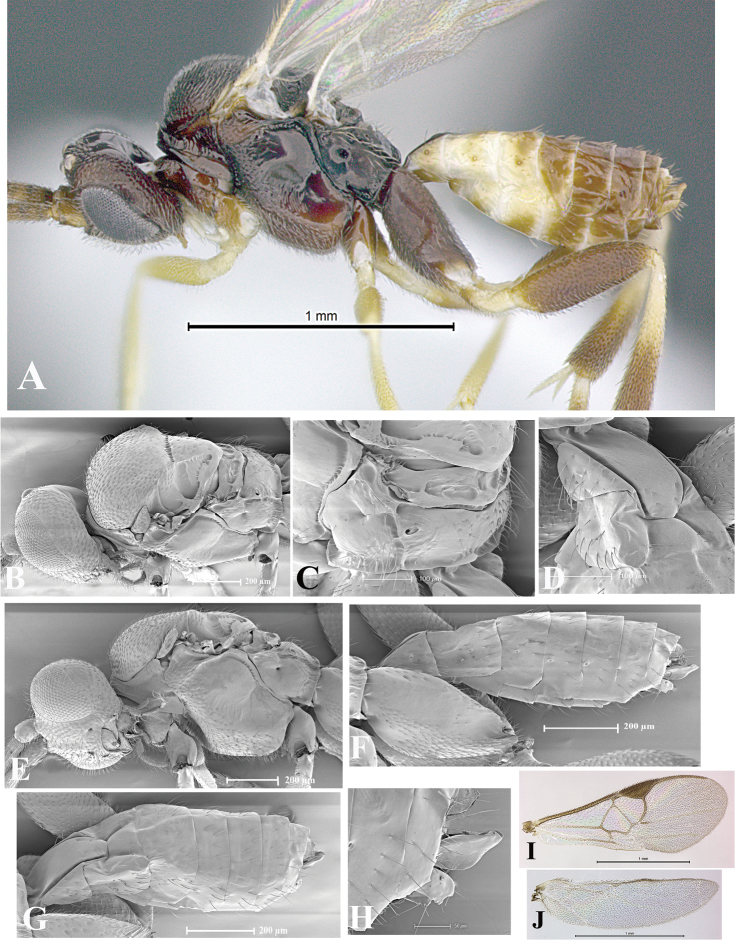
*Glyptapantelesdavesmithi* sp. nov. male 01-SRNP-6841 DHJPAR0000021, 02-SRNP-9987 DHJPAR0000034 **A** Habitus **B, E** Head, mesosoma **B** Dorsolateral view **E** lateral view **C** Metanotum, propodeum, dorsolateral view **D**T1–2, dorsolateral view **F, G** Metasoma **F** Lateral view **G** Dorsolateral view **H** Genitalia: parameres, lateral view **I, J** Wings **I** Fore **J** Hind.

Vertex in lateral view rounded, anterior furrow of metanotum without setiferous lobes and not as well delineated as posterior furrow of metanotum (Figs 66B, C, 67C), mesoscutum punctation distinct throughout (Figs 66B, 67B), and fore wing with vein 2-1A proximally tubular, distally spectral although sometimes difficult to see, outer side of junction of r and 2RS veins not forming a stub (Figs 66I, 67I).

Median area between lateral ocelli without depression. Distal antennal flagellomere longer than penultimate. Petiole on T1 parallel-sided in proximal half, then narrowing, completely smooth and polished, with faint, satin-like sheen (Figs 66D, 67D, G). Inner margin of eyes diverging slightly at antennal sockets. Propodeum without median longitudinal carina (Figs 66B, C, 67B, C). Lateral grooves delimiting the median area on T2 clearly defined and reaching the distal edge of T2 (Figs 66D, 67D, G).

#### Coloration

(Fig. 66A). General body coloration dark brown except scape, pedicel, labrum, mandibles, glossa, maxillary and labial palps yellow; ventro-lateral corners of mesopleuron, epicnemial ridge, dorsal edge of pronotum, distal edge of mesoscutum, and distal half of scutellum reddish brown; first five proximal antennal flagellomeres dorsally lighter (yellow) than ventrally (brown), remaining flagellomeres brown on both sides. Eyes and ocelli silver. Fore and middle legs yellow with claws brown; hind legs yellow, except coxae, apexes of femora and tibiae, most of basitarsus and remaining tarsomeres brown. Petiole on T1 brown with contour darkened, and sublateral areas yellow; T2 with median and adjacent areas brown, and lateral ends yellow; T3 mostly brown, but with a small yellow area on distal corners; T4 and beyond brown; distally each tergum with a narrow whitish transparent band. In lateral view, T1–3 completely yellow; T4 and beyond brown. S1–4 yellow, medially with a brown spot; penultimate sternum and hypopygium completely brown.

#### Description.

**Head** (Fig. 66A). Head triangular with pubescence long and dense. Proximal three antennal flagellomeres longer than wide (0.19:0.05, 0.19:0.05, 0.20:0.05), distal antennal flagellomere longer than penultimate (0.11:0.05: 0.09:0.05), antenna longer than body (2.63, 2.42); antennal scrobes-frons shallow. Face convex, with dense fine punctations, interspaces with microsculpture, and longitudinal median carina present. Frons punctate. Temple wide, punctate and interspaces wavy. Inner margin of eyes diverging slightly at antennal sockets; in lateral view, eye anteriorly convex and posteriorly straight. POL shorter than OOL (0.08, 0.10). Malar suture present. Median area between lateral ocelli without depression. Vertex laterally rounded and dorsally wide.

**Mesosoma** (Fig. 66A–C, E). Mesosoma dorsoventrally convex. Mesoscutum convex, punctation distinct throughout, and interspaces wavy/lacunose. Scutellum triangular, apex sloped and fused with BS, scutellar punctation scattered throughout, in profile scutellum convex and slightly higher than mesoscutum, phragma of the scutellum partially exposed; BS only very partially overlapping the MPM; ATS demilune with short stubs delineating the area; dorsal ATS groove with carinae only proximally. Transscutal articulation with small and heterogeneous foveae, area just behind transscutal articulation with a smooth and shiny sloped transverse strip. Metanotum with BM wider than PFM (clearly differentiated); MPM circular and bisected by a median longitudinal carina; AFM without setiferous lobes and not as well delineated as PFM; PFM thick and smooth; ATM proximally with semircular/undulate carina and distally smooth. Propodeum without median longitudinal carina, proximal half weakly curved with medium-sized sculpture and distal half rugose with a shallow dent at each side of nucha; distal edge of propodeum with a flange at each side and without stubs; propodeal spiracle distally framed by a short concave carina; nucha surrounded by very short radiating carinae. Pronotum virtually without trace of dorsal furrow, dorsally with a well-defined smooth band; central area of pronotum smooth, but both dorsal and ventral furrows with short parallel carinae. Propleuron with fine rugae and dorsally with a carina. Metasternum flat or nearly so. Contour of mesopleuron straight/angulate or nearly so; precoxal groove deep with transverse lineate sculpture; epicnemial ridge convex, teardrop-shaped.

**Legs.** Ventral margin of fore telotarsus slightly excavated and with a tiny curved seta, fore telotarsus almost same width throughout and longer than fourth tarsomere (0.12, 0.06). Hind coxa with punctation only on ventral surface and dorsal outer depression present. Inner spur of hind tibia longer than outer spur (0.20, 0.15), entire surface of hind tibia with dense strong spines clearly differentiated by color and length. Hind telotarsus longer than fourth tarsomere (0.13, 0.10).

**Wings** (Fig. 66I, J). Fore wing with r vein curved; 2RS vein straight; r and 2RS veins forming a weak, even curve at their junction and outer side of junction not forming a stub; 2M vein slightly curved/swollen; distally fore wing [where spectral veins are] with microtrichiae more densely concentrated than the rest of the wing; anal cell 1/3 proximally lacking microtrichiae; subbasal cell with a small smooth area, vein 2CUa absent and vein 2CUb spectral; vein 2 cu-a absent; vein 2-1A proximally tubular and distally spectral, although sometimes difficult to see; tubular vein 1 cu-a curved and complete, but junction with 1-1A vein spectral. Hind wing with vannal lobe narrow, subdistally and subproximally straightened, and setae present only proximally.

**Metasoma** (Fig. 66A, D, F–H). Metasoma laterally compressed. Petiole on T1 completely smooth and polished, with faint, satin-like sheen, parallel-sided in proximal half and then narrowing (length 0.33, maximum width 0.17, minimum width 0.08), and with scattered pubescence concentrated in the first distal third. Lateral grooves delimiting the median area on T2 clearly defined and reaching the distal edge of T2 (length median area 0.11, length T2 0.11), edges of median area polished and lateral grooves deep, median area broader than long (length 0.11, maximum width 0.17, minimum width 0.08); T2 with scattered pubescence only distally. T3 longer than T2 (0.18, 0.11) and with scattered pubescence only distally. Pubescence on hypopygium dense.

**Cocoons.** White or brown oval cocoons with evenly smooth silk fibers. Each cocoon was one width apart, arranged in two rows of cordwood on each side of caterpillar cadaver and adhered to the leaf substrate.

#### Comments.

Both sexes with body slim.

#### Male

(Fig. 67A–J). Similar in coloration and shape to female. As well as female, male has the first five proximal antennal flagellomeres with two colorations: dorsally lighter (yellow) than ventrally (brown), remaining flagellomeres brown on both sides.

#### Etymology.

David (Dave) R. Smith is interested in the systematics and biology of world sawflies (Hymenoptera: Symphyta) and parasitoid wasps (Hymenoptera: Evanioidea, Trigonalyidae). Currently, he is an emeritus research entomologist at the United States Department of Agriculture Department of Agriculture (USDA), the Systematic Entomology Laboratory (SEL), Washington, D.C., USA.

#### Distribution.

Parasitized caterpillars were collected in Costa Rica, ACG, Sector Cacao (Estación Cacao and Senderoderrumbe) and Sector Pitilla (Sendero Evangelista and Sendero Orosilito) during May 2001, July 2002, December 2003, and August 2011 at 660 m, 900 m, 1,150 m, and 1,220 m in rain and cloud forests.

#### Biology.

The lifestyle of this parasitoid species is gregarious.

#### Host.

*Antiblemmaleucocyma* Hampson (Erebidae: Eulepidotinae) feeding on *Conostegiaxalapensis*, *Miconiabrenesii*, and *Ossaeabrenesiior* (Melastomataceae) and *Antiblemma* sp. Hübner feeding on *Henrietteatuberculosa* (Melastomataceae). Caterpillars were collected in fourth and fifth instar.

### Glyptapanteles davidwahli

Taxon classificationAnimaliaHymenopteraBraconidae

Arias-Penna, sp. nov.

http://zoobank.org/4C5C0E66-7263-49FC-831D-31F1EA871F00

Figs 68 , 69

#### Female.

Body length 2.07 mm, antenna length 2.12 mm, fore wing length 2.12 mm.

#### Type material.

**Holotype**: COSTA RICA • 1♀; 92-SRNP-5068, DHJPAR0000066; Área de Conservación Guanacaste, Guanacaste, Sector Santa Rosa, Cafetal; 280 m; 10.85827, -85.61089; 22.viii.1992; gusaneros leg.; caterpillar collected in fifth instar and already with a brown/gray a single row of cordwood cocoons on each side of caterpillar; adult parasitoids emerged on 26.viii.1992; (CNC). **Paratypes.** • 9 (2♀, 2♂) (4♀, 1♂); 92-SRNP-5068, DHJPAR0000066; same data as for holotype; (CNC).

#### Other material.

**Reared material.** COSTA RICA: *Área de Conservación Guanacaste*, *Guanacaste*, *Sector Santa Rosa*, *Cafetal*: • 6 (1♀, 2♂) (1♀, 2♂); 92-SRNP-5067, DHJPAR0000067; 280 m; 10.85827, -85.61089; 22.viii.1992; gusaneros leg.; caterpillar collected in fifth instar and already with a single brown/gray row of cordwood cocoons on each side of caterpillar; adult parasitoids emerged on 27.viii.1992.

#### Diagnosis.

**Figure 68.**
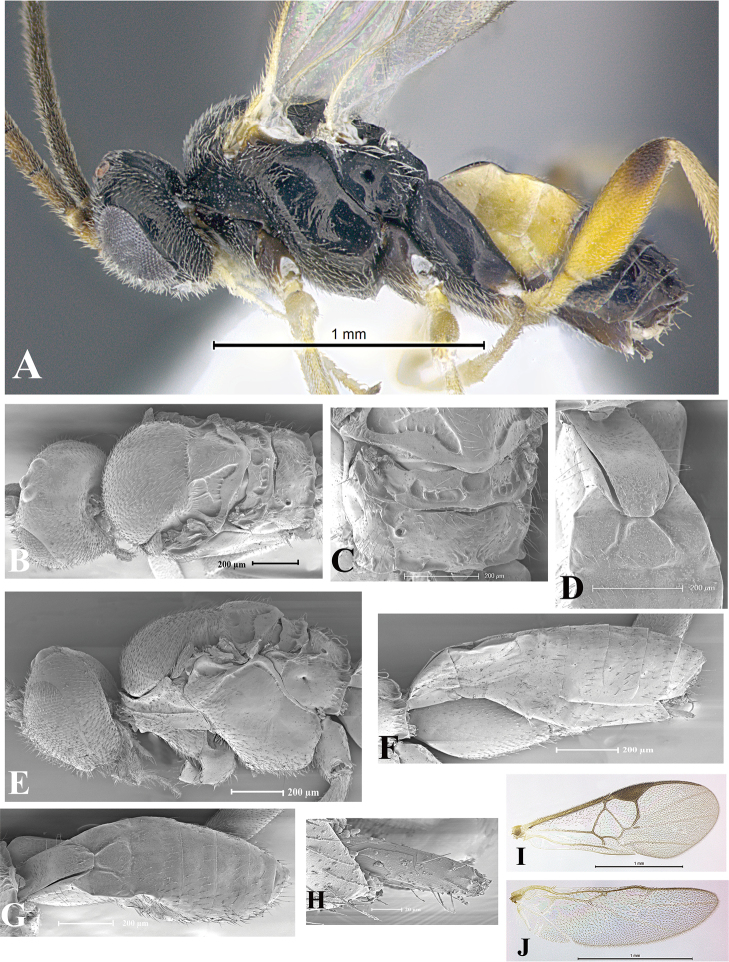
*Glyptapantelesdavidwahli* sp. nov. female 92-SRNP-5068 DHJPAR0000066 **A** Habitus **B, E** Head, mesosoma **B** Dorsolateral view **E** lateral view **C** Metanotum, propodeum, dorsal view **D**T1–2, dorsal view **F, G** Metasoma **F** Lateral view **G** Dorsolateral view **H** Genitalia: hypopygium, ovipositor, ovipositor sheaths, lateral view **I, J** Wings **I** Fore **J** Hind.

**Figure 69.**
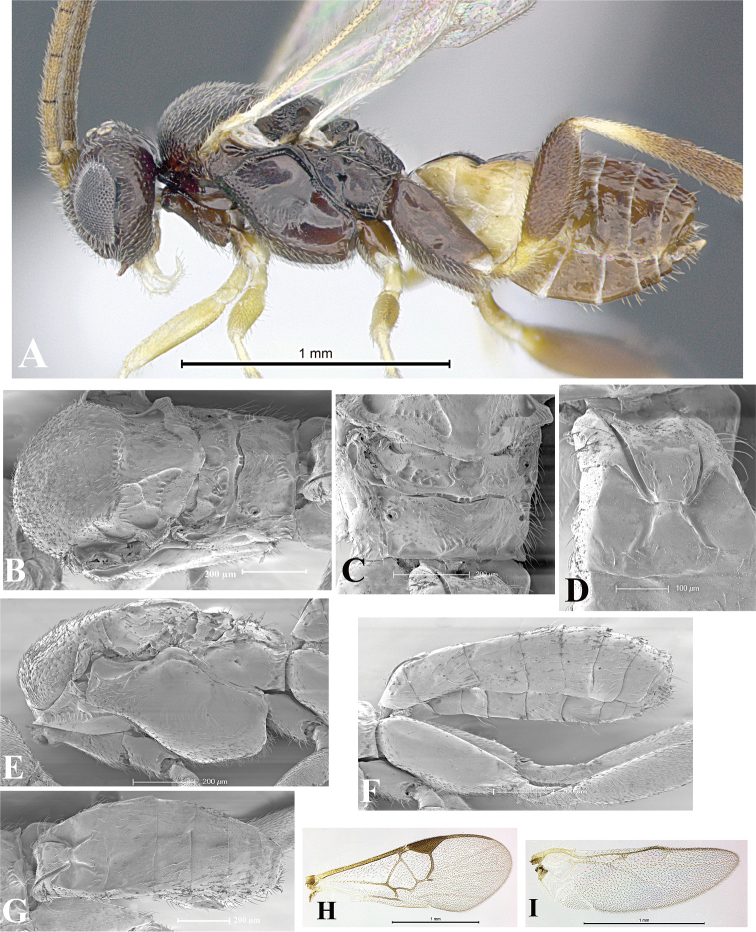
*Glyptapantelesdavidwahli* sp. nov. male 92-SRNP-5068 DHJPAR0000066 **A** Habitus **B, E** Mesosoma **B** Dorsal view **E** lateral view **C** Metanotum, propodeum, dorsal view **D**T1–2, dorsal view **F, G** Metasoma **F** Lateral view **G** Dorsal view **H, I** Wings **H** Fore **I** Hind.

Propleuron with fine punctations throughout (Figs 68A, E, 69A, E), mesoscutum punctation proximally distinct, but distally absent/dispersed (Figs 68B, 69B), anteroventral contour of mesopleuron straight/angulate or nearly so (Figs 68A, E, 69A, E), petiole on T1 distally with lateral margins relatively straight (Figs 68D, G, 69D, G), propodeum without median longitudinal carina, propodeal spiracle without distal carina (Figs 68B, C, 69B, C), nucha surrounded by very short radiating carinae (Figs 68B, C, 69B, C), antenna longer than body, fore wing with 2RS vein straight, outer side of junction of r and 2RS veins not forming a stub (Figs 68I, 69H), and lateral grooves delimiting the median area on T2 distally losing definition (Figs 68D, G, 69D, G).

#### Coloration

(Fig. 68A). General body coloration black except first four proximal antennal flagellomeres dorsally lighter (yellow-brown) than ventrally (brown), remaining flagellomeres brown on both sides; scape, labrum, mandibles, and tegulae yellow-brown; pedicel with some brown hue; maxillary and labial palps yellow. Eyes gray and ocelli silver. Fore and middle legs yellow except dark brown coxae and brown claws; hind legs yellow except black coxae, distal dot in femora, distal half tibiae, distal 3/4 of basitarsus and rest of tarsomeres brown. Petiole on T1 with two colorations: proximal 3/4 yellow and distal 1/4 black, contours darkened, and sublateral areas yellow; T2 with median and adjacent areas black, adjacent area wide with limits well-defined and forming together with the median area a rectangle shape, and lateral ends yellow/yellow-brown; T3 medially with a black area which width coincides with the distal width of median plus adjacent areas on T2, black area on T3 reaches the distal edge of T3, and lateral ends yellow; T4 and beyond black; distally each tergum with a narrow whitish transparent band. In lateral view, T1–3 completely yellow; T4 and beyond completely brown/black. S1–3 yellow; S4 proximal half yellow, distal half brown/black; penultimate sternum and hypopygium completely brown/black.

#### Description.

**Head** (Fig. 68A, B, E) Head rounded with pubescence long and dense. Proximal three antennal flagellomeres length longer than wide (0.19:0.05, 0.17:0.05, 0.17:0.05), distal antennal flagellomere longer than penultimate (0.11:0.05, 0.08:0.05), antenna longer than body (2.12, 2.07); antennal scrobes-frons shallow. Face with dense fine punctations, laterally with depressions, interspaces wavy and longitudinal median carina present. Frons punctate. Temple wide, punctate and interspaces wavy. Inner margin of eyes diverging slightly at antennal sockets; in lateral view, eye anteriorly convex and posteriorly straight. POL shorter than OOL (0.10, 0.13). Malar suture present. Median area between lateral ocelli slightly depressed. Vertex laterally rounded and dorsally wide.

**Mesosoma** (Fig. 68A–C, E). Mesosoma dorsoventrally convex. Mesoscutum shape proximally convex distally flat, punctation distinct proximally, but absent/dispersed distally and interspaces wavy/lacunose. Scutellum triangular, apex sloped and fused with BS, scutellar punctation distinct peripherally, absent centrally, in profile scutellum flat and on same plane as mesoscutum, phragma of the scutellum widely visible; BS only very partially overlapping the MPM; ATS demilune with short stubs delineating the area; dorsal ATS groove with semicircular/parallel carinae. Transscutal articulation with small and heterogeneous foveae, area just behind transscutal articulation smooth, shiny and depressed centrally. Metanotum with BM wider than PFM (clearly differentiated); MPM circular without median longitudinal carina; AFM with a small lobe and not as well delineated as PFM; PFM thick, smooth and with lateral ends rounded; ATM with complete parallel carinae. Propodeum without median longitudinal carina, proximal half weakly curved relatively polished and distal half with a shallow dent at each side of nucha; distal edge of propodeum with a flange at each side and without stubs; propodeal spiracle without distal carina; nucha surrounded by very short radiating carinae. Pronotum with a distinct dorsal furrow, dorsally with a well-defined smooth band; central area of pronotum smooth, but both dorsal and ventral furrows with short parallel carinae. Propleuron with fine punctations throughout and dorsally without a carina. Metasternum flat or nearly so. Contour of mesopleuron straight/angulate or nearly so, precoxal groove deep with faintly transverse lineate sculpture; epicnemial ridge elongated more fusiform (tapering at both ends).

**Legs.** Ventral margin of fore telotarsus excavated with conspicuous curved seta over this excavation, fore telotarsus almost same width throughout and longer than fourth tarsomere (0.12, 0.06). Hind coxa with punctation only on ventral surface and dorsal outer depression present. Inner spur of hind tibia longer than outer spur (0.25, 0.14), entire surface of hind tibia with dense strong spines clearly differentiated by color and length. Hind telotarsus as equal in length as fourth tarsomere (0.09, 0.09).

**Wings** (Fig. 68I, J). Fore wing with r vein slightly curved; 2RS vein straight; r and 2RS veins forming a weak, even curve at their junction and outer side of junction not forming a stub; 2M vein slightly curved/swollen; distally fore wing [where spectral veins are] with microtrichiae more densely concentrated than the rest of the wing; anal cell 1/3 proximally lacking microtrichiae; subbasal cell with a small smooth area; veins 2CUa and 2CUb completely spectral; vein 2 cu-a present as spectral vein, sometimes difficult to see; vein 2-1A proximally tubular and distally spectral, although sometimes difficult to see; tubular vein 1 cu-a curved, incomplete/broken and not reaching the edge of 1-1A vein. Hind wing with vannal lobe narrow, subdistally straightened, subproximally straightened, and setae present only proximally.

**Metasoma** (Fig. 68A, D, F–H). Metasoma laterally compressed. Petiole on T1 completely smooth and polished, with faint, satin-like sheen, virtually parallel-sided over most of length, but narrowing over distal 1/3 (length 0.31, maximum width 0.14, minimum width 0.06), with little pubescence concentrated in the first distal third. Lateral grooves delimiting the median area on T2 distally losing definition (length median area 0.10, length T2 0.14), edges of median area polished and lateral grooves deep, median area broader than long length (length 0.10, maximum width 0.18, minimum width 0.06); T2 with a distinctive row of pubescence only at the distal margin. T3 longer than T2 (0.18, 0.14) and with scattered pubescence throughout. Pubescence on hypopygium dense.

**Cocoons.** Brown or gray oval cocoons with evenly smooth silk fibers. A single row of cordwood cocoons on each side of caterpillar.

#### Comments.

Medially, the pronotum is at a different level to the remaining portion of the structure. The propodeal spiracle is small.

#### Male

(Fig. 69A–I). Similar in shape to female, but the general coloration is lighter than female.

#### Etymology.

David B. Wahl works at the American Entomological Institute (AEI) that is part of Utah State University (USU), Logan, UT, USA. His main focus has been the family Ichneumonidae.

#### Distribution.

Parasitized caterpillar was collected in Costa Rica, ACG, Sector Santa Rosa (Cafetal), during August 1992 at 280 m on coffee plantation.

#### Biology.

The lifestyle of this parasitoid species is gregarious.

#### Host.

*Parachaboraabydas* (Herrich-Schäffer) (Noctuidae: Catocalinae) feeding on *Tephrosiamultifolia* (Fabaceae). Caterpillar was collected in fifth instar.

### Glyptapanteles diegocamposi

Taxon classificationAnimaliaHymenopteraBraconidae

Arias-Penna, sp. nov.

http://zoobank.org/A2E37A6C-A66E-429B-9B1E-DDE20164649E

Figs 70 , 71

#### Female.

Body length 2.78 mm, antenna length 3.28 mm, fore wing length 3.03 mm.

#### Type material.

**Holotype**: ECUADOR • 1♀; EC-36329, YY-A066; Napo, Yanayacu Biological Station, Sendero Macuculoma, Plot 413; cloud forest; 2,120 m; -0.6, -77.883333; 12.xii.2008; Wilmer Simbaña leg.; caterpillar collected in third instar; cocoons formed on 03.i.2009; adult parasitoids emerged on 20.i.2009; (PUCE). **Paratypes.** • 8 (3♀, 4♂) (1♀, 0♂); EC-36329, YY-A066; same data as for holotype; (PUCE).

#### Other material.

**Reared material.** ECUADOR: *Napo*, *Yanayacu Biological Station*, *San Isidro Forest*, *Plot 191*: • 11 (5♀, 1♂) (5♀, 0♂); EC-12997, YY-A090; cloud forest; 2,208 m; -0.6, -77.883333; 10.iii.2006; Rafael Granizo leg.; caterpillar collected in second instar; cocoons formed on 07.iv.2006; adult parasitoids emerged on 03.v.2006.

*Napo*, *Yanayacu Biological Station*, *Sendero Macuculoma*, *Plot 417*: • 6 (1♀, 2♂) (1♀, 2♂); EC-36604, YY-A099; cloud forest; 2,120 m; -0.6, -77.883333; 10.i.2009; Earthwatch volunteers leg.; caterpillar collected in second instar; cocoons formed on 29.i.2009; adult parasitoids emerged on 12.ii.2009.

#### Diagnosis.

**Figure 70.**
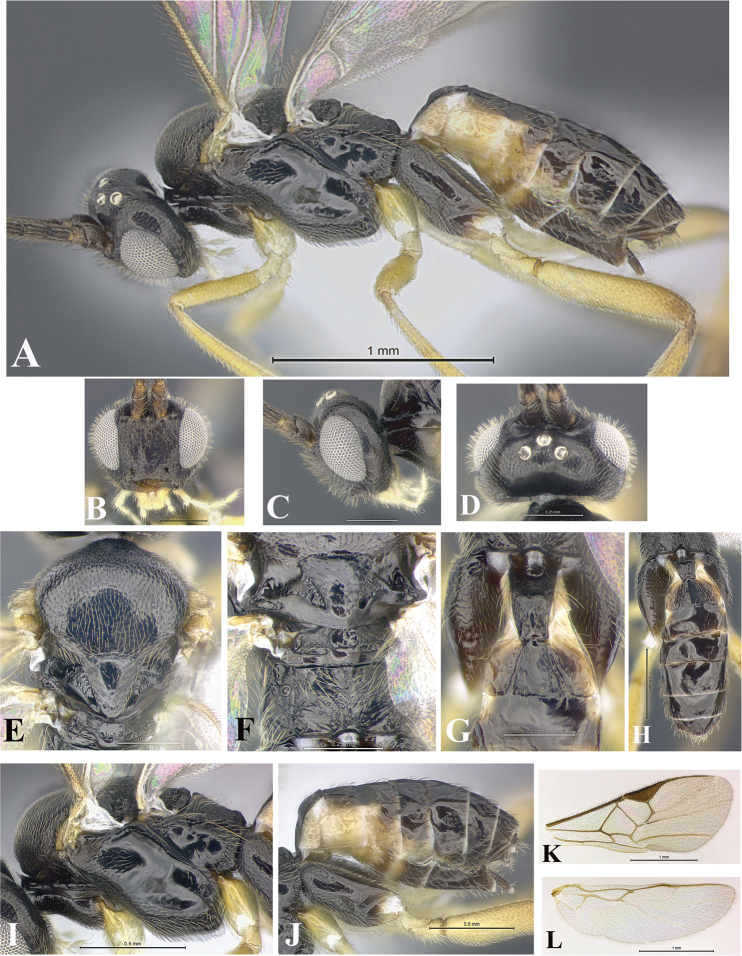
*Glyptapantelesdiegocamposi* sp. nov. female EC-36329 YY-A066 **A** Habitus **B, D** Head **B** Frontal view **D** Dorsal view **C** Head, pronotum, propleuron, lateral view **E** Mesonotum, dorsal view **F** Scutellum, metanotum, propodeum, dorsal view **G**T1–2, dorsal view **H, J** Metasoma **H** Dorsal view **J** Lateral view **I** Mesosoma, lateral view **K, L** Wings **K** Fore **L** Hind.

**Figure 71.**
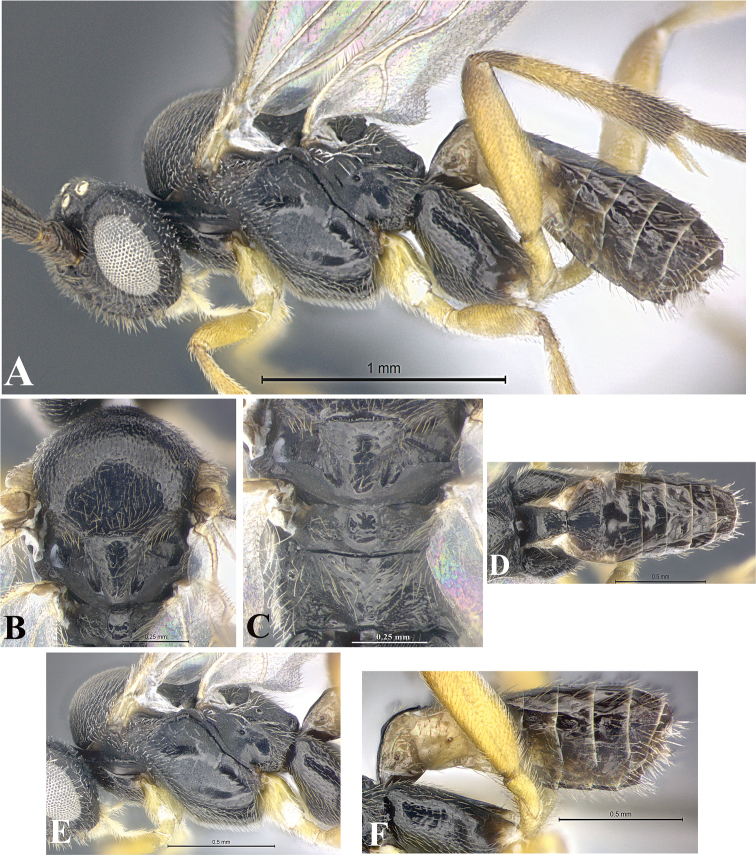
*Glyptapantelesdiegocamposi* sp. nov. male EC-36329 YY-A066 **A** Habitus **B** Mesonotum, dorsal view **C** Scutellum, metanotum, propodeum, dorsal view **D, F** Metasoma **D** Dorsal view **F** Lateral view **E** Mesosoma, lateral view.

In lateral view, metasoma curved (Figs 70A, 71A), T3 as long as T2 (Figs 70H, 71D), inner margin of eyes straight throughout (Fig. 70B), petiole on T1 evenly narrowing distally (wide base to a narrow apex) and finely sculptured (Figs 70G, 71D), propodeum without a median longitudinal dent (Figs 70F, 71C), lateral grooves delimiting the median area on T2 distally losing definition on T2 (Figs 70H, 71D), and fore wing with r vein straight, outer side of junction of r and 2RS veins forming a stub (Fig. 70K).

#### Coloration

(Fig. 70A–L). General body coloration black except labrum, mandibles, scape and pedicel yellow-brown; all antennal flagellomeres brown on both sides; glossa, maxillary and labial palps, and tegulae yellow. Eyes and ocelli silver. Fore and middle legs yellow except brown claws; hind legs yellow except black-brown coxae with apex yellow, femora with a tiny brown area on the apex, tibiae with 1/4 distal brown, and tarsomeres brown. Petiole on T1 black and sublateral areas yellow; T2 with median area black with contours darkened and lateral ends yellow-brown; T3 brown, but proximal corners yellow-brown; T4 and beyond completely dark brown; distally each tergum with a narrow yellowish transparent band. In lateral view, T1–2 completely yellow; T3–4 yellow-brown, but dorsally brown, extent of brown area increasing from proximal to distal; T5 and beyond completely brown. S1–3 completely yellow; S4–5 yellow-brown; hypopygium completely brown.

#### Description.

**Head** (Fig. 70A–D). Head rounded with pubescence long and dense. Proximal three antennal flagellomeres longer than wide (0.23:0.08, 0.24:0.08, 0.22:0.08), distal antennal flagellomere longer than penultimate (0.15:0.06, 0.11:0.06), antenna longer than body (3.28, 2.78); antennal scrobes-frons shallow. Face flat or nearly so, with dense fine punctations, interspaces smooth and longitudinal median carina present. Frons smooth. Temple wide, punctate sculpture and interspaces clearly smooth. Inner margin of eyes straight throughout; in lateral view, eye anteriorly convex and posteriorly straight. POL shorter than OOL (0.09, 0.13). Malar suture present. Median area between lateral ocelli without depression. Vertex laterally rounded and dorsally wide.

**Mesosoma** (Fig. 70A, E, F, I). Mesosoma dorsoventrally convex. Mesoscutum proximally convex and distally flat, punctation distinct throughout, interspaces smooth. Scutellum triangular, apex sloped and fused with BS, scutellar punctation scattered throughout, in profile scutellum flat and on same plane as mesoscutum, phragma of the scutellum partially exposed; BS only very partially overlapping the MPM; ATS demilune with short stubs delineating the area, dorsal ATS groove smooth. Transscutal articulation with small and heterogeneous foveae, area just behind transscutal articulation smooth, shiny and nearly at the same level as mesoscutum (flat). Metanotum with BM wider than PFM (clearly differentiated); MPM circular without median longitudinal carina; AFM with a small lobe and not as well delineated as PFM; PFM thick and smooth; ATM proximally with semircular/undulate carina and distally smooth. Propodeum without median longitudinal carina, proximal half straight or nearly so and with medium-sized sculpture and distal half with a shallow dent at each side of nucha; distal edge of propodeum with a flange at each side and without stubs; propodeal spiracle distally framed by faintly concave/wavy carina; nucha surrounded by very short radiating carinae. Pronotum with a distinct dorsal furrow, dorsally with a well-defined smooth band; central area of pronotum and dorsal furrow smooth, but ventral furrow with short parallel carina. Propleuron with fine punctations throughout and dorsally without a carina. Metasternum flat or nearly so. Contour of mesopleuron straight/angulate or nearly so; precoxal groove smooth, shiny and shallow, but visible; epicnemial ridge elongated more fusiform (tapering at both ends).

**Legs** (Fig. 70A). Ventral margin of fore telotarsus entire without seta, fore telotarsus proximally narrow and distally wide, and longer than fourth tarsomere (0.12, 0.08). Hind coxa with very finely punctate throughout, and dorsal outer depression absent. Inner spur of hind tibia longer than outer spur (0.22, 0.17), entire surface of hind tibia with dense strong spines clearly differentiated by color and length. Hind telotarsus longer than fourth tarsomere (0.13, 0.11).

**Wings** (Fig. 70K, L). Fore wing with r vein straight; 2RS vein slightly concave; r and 2RS veins forming an angle at their junction and outer side of junction forming a slight stub; 2M vein straight; distally fore wing [where spectral veins are] with microtrichiae more densely concentrated than the rest of the wing; anal cell 1/3 proximally lacking microtrichiae; subbasal cell with microtrichiae virtually throughout; veins 2CUa and 2CUb completely spectral; vein 2 cu-a present as spectral vein, sometimes difficult to see; vein 2-1A proximally tubular and distally spectral, although sometimes difficult to see; tubular vein 1 cu-a curved and complete, but junction with 1-1A vein spectral. Hind wing with vannal lobe very narrow, subdistally and subproximally evenly convex, and setae evenly scattered in the margin.

**Metasoma** (Fig. 70A, G, H, J). Metasoma curved. Petiole on T1 finely sculptured on distal half, evenly narrowing distally (length 0.36, maximum width 0.17, minimum width 0.10), and with scattered pubescence concentrated in the first distal third. Lateral grooves delimiting the median area on T2 clearly defined and reaching the distal edge of T2 (length median area 0.19, length T2 0.19), edges of median area with little sculpture, median area as broad as long (length 0.19, maximum width 0.20, minimum width 0.08); T2 with scattered pubescence throughout. T3 as long as T2 (0.20, 0.19) and with scattered pubescence throughout. Pubescence on hypopygium dense.

**Cocoons.** Unknown.

#### Comments.

In lateral view, body is curved.

#### Male

(Fig. 71A–F). Similar in coloration and shape to female.

#### Etymology.

Diego Fernando Campos Moreno is a Colombian entomologist whose research has been focused on Braconidae. Currently, he is a Ph.D. student at El Colegio de la Frontera Sur (ECOSUR), Chetumal, Quintana Roo, México.

#### Distribution.

Parasitized caterpillars were collected in Ecuador, Napo, Yanayacu Biological Station (Sendero Macuculoma and San Isidro Forest), during March 2006, December 2008, and January 2009 at 2,120 m and 2,208 m in cloud forest.

#### Biology.

The lifestyle of this parasitoid species is gregarious.

#### Host.

Undetermined species of Nymphalidae (Ithomiinae) feeding on *Cestrummegalophyllum* Dunal and *Cestrum* sp. (Solanaceae). Caterpillars were collected in second and third instar.

### Glyptapanteles donquickei

Taxon classificationAnimaliaHymenopteraBraconidae

Arias-Penna, sp. nov.

http://zoobank.orgAF20239B-7A33-4B2F-9EED-4F5EF32B7F12

Figs 72 , 73

#### Female.

Body length 2.02 mm, antenna length 2.37 mm, fore wing length 2.22 mm.

#### Type material.

**Holotype**: COSTA RICA • 1♀; 01-SRNP-5776, DHJPAR0000020; Área de Conservación Guanacaste, Alajuela, Sector Rincón Rain Forest, Vado Río Francia; 400 m, 10.90093, -85.28915, 13.ix.2001; Freyci Vargas leg.; caterpillar collected in second instar; single row of gray cordwood cocoons on each side of cadaver caterpillar, each cocoon at right angles to the long axis of the caterpillar, cocoons formed on 23.ix.2001; adult parasitoids emerged on 02.x.2001; (CNC). **Paratypes.** • 82 (3♀, 4♂) (68♀, 7♂); 01-SRNP-5776, DHJPAR0000020; same data as for holotype; (CNC).

#### Other material.

**Reared material.** COSTA RICA: *Área de Conservación Guanacaste*, *Guanacaste*, *Sector Santa Rosa*, *Cafetal*: • 73 (4♀, 3♂) (55♀, 11♂); 93-SRNP-7203, DHJPAR0000076; 280 m, 10.85827, -85.61089; 26.x.1993; gusaneros leg.; brown cordwood cocoons in neat rows on each side of larva; adult parasitoids emerged on 08.xi.1993. • 67 (3♀, 3♂) (59♀, 2♂); 93-SRNP-7204, DHJPAR0000077; same data as for preceding except: two rows of neatly brown cordwood cocoons; adult parasitoids emerged on 09.xi.1993. • 70 (4♀, 4♂) (6♀, 0♂); 93-SRNP-7205, DHJPAR0000078; same data as for preceding except: neat brown rows of cocoons stacked on each side of live larva. • 71 (3♀, 5♂) (63♀, 0♂); 93-SRNP-7206, DHJPAR0000079; same data as for preceding except: cocoon characteristics not reported; adult parasitoids emerged on 06.xi.1993. • 95 (3♀, 3♂) (79♀, 10♂); 93-SRNP-7207, DHJPAR0000080; same data as for preceding except: neat rows of brown cordwood cocoons on each side of larva; adult parasitoids emerged on 05.xi.1993.

*Área de Conservación Guanacaste*, *Alajuela*, *Sector Rincón Rain Forest*, *Estación Caribe*: • 81 (5♀, 4♂) (72♀, 0♂); 09-SRNP-43316, DHJPAR0038060; 415 m, 10.90187, -85.27495; 30.xi.2009; José Pérez leg.; caterpillar collected in fifth instar; cocoons adhered to the leaf substrate and formed on 08.xii.2009; adult parasitoids emerged on 17.xii.2009.

*Área de Conservación Guanacaste*, *Alajuela*, *Sector Rincón Rain Forest*, *Sendero Albergue Crater*: • 78 (5♀, 5♂) (65♀, 3♂); 10-SRNP-2700, DHJPAR0040422; rain forest; 980 m; 10.84886, -85.3281; 31.v.2010; Elda Araya leg.; caterpillar collected in fifth instar; cocoons adhered to the leaf substrate; adult parasitoids emerged on 15.vi.2010.

*Área de Conservación Guanacaste*, *Guanacaste*, *Sector Pitilla*, *Loaiciga*: • 97 (7♀, 7♂) (83♀, 0♂); 06-SRNP-65171, DHJPAR0012671; rain forest, 445 m; 11.01983, -85.41342; 07.xi.2006; Petrona Rios leg.; caterpillar collected in third instar; neat row of cordwood cocoons on the leaf on each side of larva and formed on 19.xi.2006; adult parasitoids emerged on 29.xi.2006.

*Área de Conservación Guanacaste*, *Guanacaste*, *Sector Pitilla*, *Estación Pitilla*: • 41 (5♀, 1♂) (35♀, 0♂); 06-SRNP-65610, DHJPAR0012676; rain forest; 675 m; 10.98931, -85.42581; 15.xii.2006; Petrona Rios leg.; caterpillar collected in third instar; orderly single row of cordwood cocoons on each side of cadaver, cocoons formed on 29.xii.2006; adult parasitoids emerged on 08.i.2007.

*Área de Conservación Guanacaste*, *Guanacaste*, *Sector Pitilla*, *Pasmompa*: • 76 (5♀, 5♂) (66♀, 0♂); 10-SRNP-31937, DHJPAR0041808; rain forest; 440 m; 11.01926, -85.40997; 04.ix.2010; Calixto Moraga leg.; caterpillar collected in fifth instar; stacked cordwood cocoons adhered to the leaf substrate, cocoons formed on 12.ix.2010; adult parasitoids emerged on 20.ix.2010. • 106 (5♀, 5♂) (94♀, 2♂); 10-SRNP-31938, DHJPAR0041751; same data as for preceding except: caterpillar collected in fourth instar; single row of brown cordwood cocoons on each side of the larva and adhered to the leaf substrate, cocoons emerged on 16.ix.2010; adult parasitoids emerged on 23.ix.2010. • 61 (5♀, 5♂) (50♀, 1♂); 10-SRNP-31939, DHJPAR0041747; same data as for preceding except: caterpillar collected in third instar; cocoons emerged on 19.ix.2010 and adhered to the leaf substrate, cocoon characteristics not reported; adult parasitoids emerged on 23.ix.2010.

*Área de Conservación Guanacaste*, *Guanacaste*, *Sector Del Oro*, *Sendero Puertas*: • 10 (3♀, 0♂) (7♀, 0♂); 10-SRNP-21656, DHJPAR0040439; intergrade dry-rain forest; 400 m; 11.01087, -85.48817; 23.vii.2010; Roster Moraga leg.; caterpillar collected in fifth instar; single row of brown gray cordwood cocoons adhered to the leaf substrate and formed on 26.vii.2010; adult parasitoids emerged on 05.viii.2010.

#### Diagnosis.

**Figure 72.**
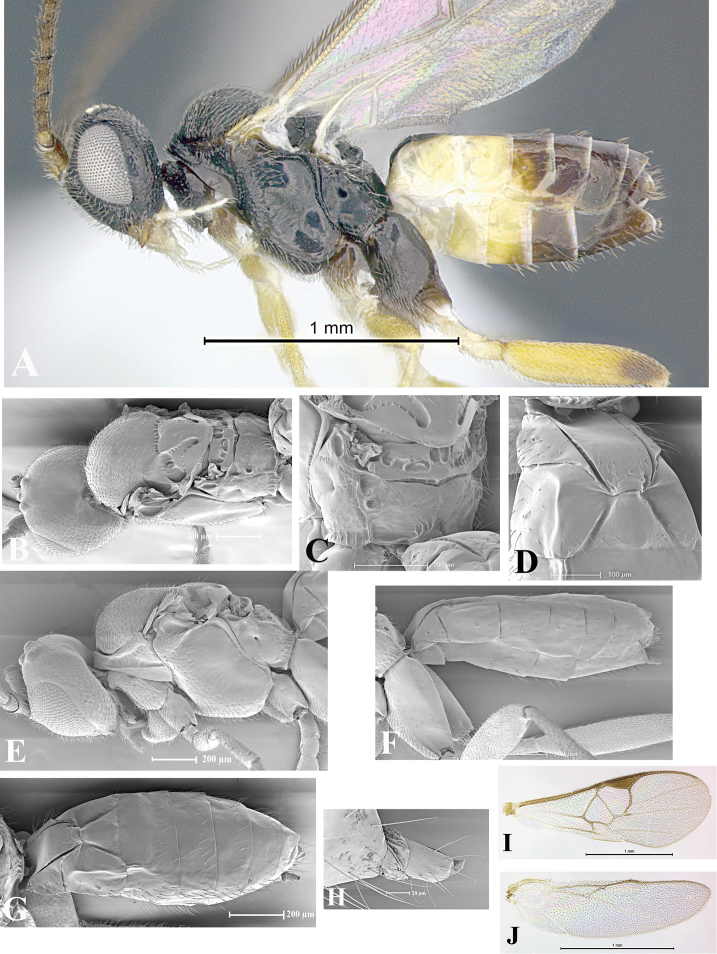
*Glyptapantelesdonquickei* sp. nov. female 01-SRNP-5776 DHJPAR0000020, 10-SRNP-31939 DHJPAR0041747 **A** Habitus **B, E** Head, mesosoma **B** Dorsolateral view **E** lateral view **C** Metanotum, propodeum, dorsolateral view **D**T1–2, dorsal view **F, G** Metasoma **F** Lateral view **G** Dorsolateral view **H** Genitalia: hypopygium, ovipositor, ovipositor sheaths, lateral view **I, J** Wings **I** Fore **J** Hind.

Petiole on T1 virtually parallel-sided over most of length, but narrowing over distal 1/3, finely sculptured only distally (Fig. 72D, G), medioposterior band of scutellum only very partially overlapping the medioanterior pit of metanotum (Fig. 72B, C), pronotum virtually without trace of dorsal furrow (Fig. 72A, E), precoxal groove deep, smooth, and shiny (Fig. 72A, E), fore wing with vein 1 cu-a curved, r vein curved, outer side of junction of r and 2RS veins not forming a stub (Fig. 72I), dorsal outer depression on hind coxa present (Fig. 72A, F), inner margin of eyes diverging slightly at antennal sockets, propodeum without median longitudinal carina (Fig. 72C), and lateral grooves delimiting the median area on T2 clearly defined and reaching the distal edge of T2 (Fig. 72D, G).

#### Coloration

(Fig. 72A). General body coloration black-brown except labrum, mandibles, scape and pedicel with yellow-brown tints; all antennal flagellomeres dorsally lighter (yellow-brown) than ventrally (brown); glossa, maxillary and labial palps yellow. Fore and middle legs yellow except brown coxae and claws; hind legs yellow except black-brown coxae, brown apex of femora and distal half of tibiae and tarsomeres brown. Petiole on T1 brown, but proximally with a median area yellow-brown, contours darkened, and sublateral areas yellow; T2 with median and adjacent areas brown, and lateral ends yellow; T3 mostly dark, but with narrow lateral ends yellow; T4 and beyond completely brown; distally each tergum with a narrow whitish transparent band. In lateral view, T1–3 completely yellow; T4 yellow-brown; T5 and beyond completely dark brown. S1–3 completely yellow; S4 yellow, medially with a small brown spot; penultimate sternum and hypopygium completely dark brown.

#### Description.

**Head** (Fig. 72A, B, E). Head rounded with pubescence long and dense. Proximal three antennal flagellomeres longer than wide (0.29:0.05, 0.28:0.05, 0.27:0.05), distal antennal flagellomere longer than penultimate (0.11:0.05, 0.08:0.05), antenna longer than body (2.37, 2.02); antennal scrobes-frons shallow. Face shape flat or nearly so, with dense fine punctations, interspaces with microsculpture and longitudinal median carina present. Frons punctate. Temple wide, punctate and interspaces wavy. Inner margin of eyes diverging slightly at antennal sockets; in lateral view, eye anteriorly convex and posteriorly straight. POL shorter than OOL (0.09, 0.11). Malar suture present. Median area between lateral ocelli slightly depressed. Vertex laterally rounded and dorsally wide.

**Mesosoma** (Fig. 72A–C, E). Mesosoma dorsoventrally convex. Mesoscutum 1/4 distal with a central dent, punctation distinct proximally with polished area distally, interspaces wavy/lacunose. Scutellum triangular, apex sloped and fused with BS, scutellar punctation scattered throughout, in profile scutellum flat and on same plane as mesoscutum, phragma of the scutellum partially exposed; BS only very partially overlapping the MPM; ATS demilune with short stubs delineating the area; dorsal ATS groove with semicircular/parallel carinae. Transscutal articulation with small and heterogeneous foveae, area just behind transscutal articulation smooth, shiny and depressed centrally. Metanotum with BM wider than PFM (clearly differentiated); MPM circular and bisected by a median longitudinal carina; AFM without setiferous lobes and not as well delineated as PFM; PFM thick and smooth; ATM proximally with semircular/undulate carina and distally smooth. Propodeum without median longitudinal carina, proximal half curved with medium-sized sculpture and distal half relatively polished or with a shallow dent at each side of nucha; distal edge of propodeum with a flange at each side and without stubs; propodeal spiracle without distal carina; nucha surrounded by very short radiating carinae. Pronotum virtually without trace of dorsal furrow, dorsally with a well-defined smooth band; central area of pronotum and dorsal furrow smooth, but ventral furrow with short parallel carinae. Propleuron with fine punctations throughout and dorsally with a carina. Metasternumflat or nearly so. Contour of mesopleuron straight/angulate or nearly so; precoxal groove smooth, shiny and distinct; epicnemial ridge convex, teardrop-shaped.

**Legs** (Fig. 72A). Ventral margin of fore telotarsus slightly excavated and with a tiny curved seta, fore telotarsus almost same width throughout and longer than fourth tarsomere (0.12, 0.06). Hind coxa with punctation only on ventral surface, dorsal outer depression present. Inner spur of hind tibia longer than outer spur (0.21, 0.15), entire surface of hind tibia with dense strong spines clearly differentiated by color and length. Hind telotarsus as equal in length as fourth tarsomere (0.11, 0.11).

**Wings** (Fig. 72I, J). Fore wing with r vein slightly curved; 2RS vein slightly convex to convex; r and 2RS veins forming a weak, even curve at their junction and outer side of junction not forming a stub; 2M vein slightly curved/swollen; distally fore wing [where spectral veins are] with microtrichiae more densely concentrated than the rest of the wing; anal cell 1/3 proximally lacking microtrichiae; subbasal cell with a small smooth area; vein 2CUa absent and vein 2CUb spectral; vein 2 cu-a absent; vein 2-1A present only proximally as tubular vein; tubular vein 1 cu-a curved and complete, but junction with 1-1A vein spectral. Hind wing with vannal lobe narrow, subdistally straightened, subproximally straightened, and setae present only proximally.

**Metasoma** (Fig. 72A, D, F–H). Metasoma laterally compressed. Petiole on T1 finely sculptured only distally, virtually parallel-sided over most of length, but narrowing over distal 1/3 (length 0.28, maximum width 0.14, minimum width 0.08), and with scattered pubescence concentrated in the first distal third. Lateral grooves delimiting the median area on T2 clearly defined and reaching the distal edge of T2 (length median area 0.14, length T2 0.14), edges of median area polished and lateral grooves deep, median area broader than long (length 0.14, maximum width 0.20, minimum width 0.07); T2 with a distinctive row of pubescence only at the distal margin. T3 longer than T2 (0.10, 0.14) and with scattered pubescence only distally. Pubescence on hypopygium dense.

**Cocoons** (Fig. 4AA). Brown or gray oval cocoon with evenly smooth silk fibers. Two rows of neat cordwood cocoons on the leaf on each side of live larvae.

#### Comments.

Both sexes with slim bodies.

#### Male

**Figure 73.**
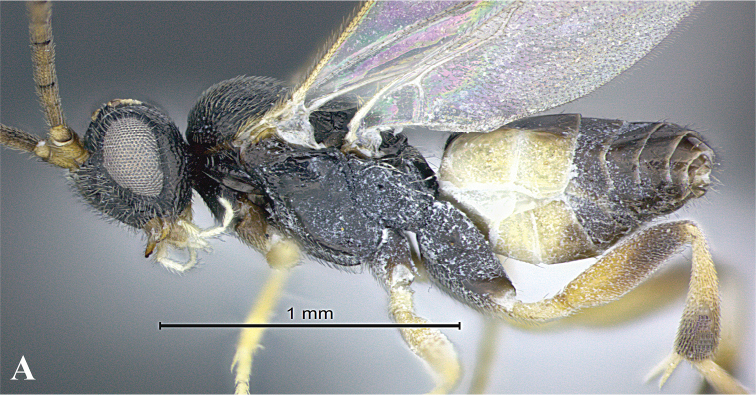
Habitus of *Glyptapantelesdonquickei* sp. nov. male 10-SRNP-31939 DHJPAR0041747.

(Fig. 73A). Similar in coloration and shape to female.

#### Etymology.

Donald (Don) L. J. Quicke is a braconidologist and hymenopterist with a long-standing interest in many diverse aspects of parasitoid biology and evolution and he is also a book writer. Currently, he is at the Chulalongkorn University, Bangkok, Thailand.

#### Distribution.

Parasitized caterpillars were collected in Costa Rica, ACG, Sector Del Oro (Sendero Puertas), Sector Pitilla (Estación Pitilla and Pasmompa), Sector Rincón Rain Forest (Estación Caribe, Sendero Albergue Crater, and Vado Río Francia), and Sector Santa Rosa (Cafetal), during October 1993, September 2001, November and December 2006, September 2009, and May, July, and September 2010 at 280 m, 400 m, 415 m, 440 m, 445 m, and 675 m in coffee plantation, intergrade dry-rain and rain forests.

#### Biology.

The lifestyle of this parasitoid species is gregarious.

#### Host.

*Condicacupienta* (Cramer) (Noctuidae: Amphipyrinae) feeding on *Neurolaenalobata* and *Plucheacarolinensis* (Asteraceae) and *C.funerea* (Schaus) (Noctuidae: Amphipyrinae) (Fig. 4AA) feeding on *Neurolaenalobata* (Asteraceae). Caterpillars were collected in second, third, fourth, and fifth instar.

### Glyptapanteles dorislagosae

Taxon classificationAnimaliaHymenopteraBraconidae

Arias-Penna, sp. nov.

http://zoobank.org/4ED63427-1493-4DA5-983C-DC4D69F325B2

Fig. 74

#### Male.

Body length 2.68 mm, antenna length 3.68 mm, fore wing length 3.13 mm.

#### Type material.

**Holotype.** ECUADOR • 1♀; EC-25275, YY-A218; Napo, Yanayacu Biological Station, Yanayacu Road; cloud forest; 2,100 m; -0.583333, -77.866667; 13.viii.2007; Rafael Granizo leg.; caterpillar collected in third instar; white bud-like cocoon formed on 06.ix.2007; adult parasitoid emerged on 20.x.2007; (PUCE).

#### Diagnosis.

**Figure 74.**
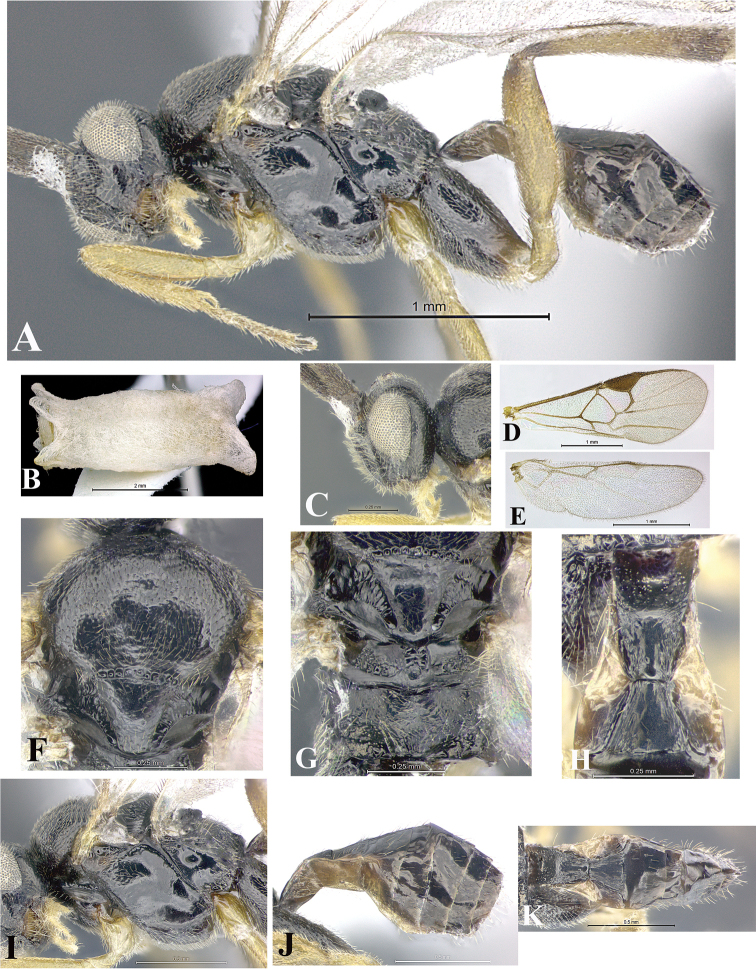
*Glyptapantelesdorislagosae* sp. nov. male EC-25275 YY-A218 **A** Habitus **B** Cocoon **C** Head, lateral view **D, E** Wings **D** Fore **E** Hind **F** Mesonotum, dorsal view **G** Scutellum, metanotum, propodeum, dorsal view **H**T1–2, dorsal view **I** Mesosoma, lateral view **J, K** Metasoma **J** Lateral view **K** Dorsal view.

Medioanterior pit of metanotum elongated with some sculpture inside and not covered by medioposterior band of scutellum (Fig. 74G), transscutal articulation with small homogeneous carinated foveae (Fig. 74F), inner margin of eyes straight throughout, median area on T2 as broad as long, edges of median area on T2 obscured by weak longitudinal stripes (Fig. 74H, K), ventral margin of fore telotarsus entire without seta, anteroventral contour of mesopleuron straight/angulate or nearly so (Fig. 74A, I), propleuron with fine punctations throughout (Fig. 74A, I), longitudinal median carina on face present, surface of metasternum convex, dorsal outer depression on hind coxa absent (Fig. 74A), and fore wing with r vein slightly curved, outer side of junction of r and 2RS veins forming a stub (Fig. 74D).

#### Coloration

(Fig. 74A–K). General body coloration polished black, except labrum, mandibles, maxillary and labial palps, and tegulae with yellow-brown tints; all antennal flagellomeres brown on both sides. Eyes silver and ocelli reddish (in preserved specimen). Fore and middle legs yellow-brown except brown claws; hind legs yellow-brown except black-brown coxae, femora with a brown spot at the apex, both ends of tibiae brown, and tarsomeres brown. Petiole on T1 black, and sublateral areas yellow-brown; T2 with median area black, adjacent area brown, and lateral ends narrow and yellow-brown; T3 and beyond black; distally each tergum with a narrow whitish transparent band. In lateral view, T1–3 yellow-brown; T4 and beyond black-brown. S1–3 yellow-brown; S4 and beyond brown.

#### Description.

**Head** (Fig. 74A, C). Head triangular with pubescence long and dense. Proximal three antennal flagellomeres longer than wide (0.26:0.06, 0.26:0.06, 0.25:0.06), distal antennal flagellomere longer than penultimate (0.16:0.06, 0.11:0.06), antenna longer than body (3.68, 2.68); antennal scrobes-frons shallow. Face with lateral depression with scattered finely punctate, interspaces smooth and longitudinal median carina present. Frons punctate. Temple wide, punctate and interspaces clearly smooth. Inner margin of eyes straight throughout, in lateral view eye anteriorly convex and posteriorly straight. POL shorter than OOL (0.09, 0.13). Malar suture absent or difficult to see. Median area between lateral ocelli without depression. Vertex laterally rounded and dorsally wide.

**Mesosoma** (Fig. 74A, F, G, I). Mesosoma dorsoventrally convex. Mesoscutum 1/4 distal with a central dent, punctation distinct throughout, interspaces wavy/lacunose. Scutellum triangular, apex sloped and fused with BS, scutellar punctation scattered throughout, in profile scutellum flat and on same plane as mesoscutum, phragma of the scutellum partially exposed; BS only very partially overlapping the MPM; ATS demilune with a little, complete parallel carinae; dorsal ATS groove with semicircular/parallel carinae. Transscutal articulation with small and homogeneous foveae, area just behind transscutal articulation nearly at the same level as mesoscutum (flat) and with same kind of sculpture as mesoscutum. Metanotum with BM wider than PFM (clearly differentiated); MPM circular with some sculpture inside; AFM with a small lobe and not as well delineated as PFM; PFM thick and smooth; ATM proximally with semircular/undulate carina and distally smooth. Propodeum without median longitudinal carina, proximal half weakly curved with medium-sized sculpture and distal half with a shallow dent at each side of nucha; distal edge of propodeum with a flange at each side and without stubs; propodeal spiracle distally framed by faintly concave/wavy carina; nucha surrounded by very short radiating carinae. Pronotum with a distinct dorsal furrow, dorsally with a well-defined smooth band; central area of pronotum and dorsal furrow smooth, but ventral furrow with short parallel carinae. Propleuron with fine punctations throughout and dorsally without a carina. Metasternum convex. Contour of mesopleuron straight/angulate or nearly so; precoxal groove smooth, shiny and shallow, but visible; epicnemial ridge elongated more fusiform (tapering at both ends).

**Legs.** Ventral margin of fore telotarsus entire without seta, fore telotarsus proximally narrow and distally wide slightly, and longer than fourth tarsomere (0.10, 0.08). Hind coxa finely punctate throughout, and dorsal outer depression absent. Inner spur of hind tibia longer than outer spur (0.23, 0.17), entire surface of hind tibia with dense strong spines clearly differentiated by color and length. Hind telotarsus slightly longer than fourth tarsomere (0.14, 0.12).

**Wings** (Fig. 74D, E). Fore wing with r vein slightly curved; 2RS vein slightly concave; r and 2RS veins forming a weak, even curve at their junction and outer side of junction forming a slight stub; 2M vein slightly curved/swollen; distally fore wing [where spectral veins are] with microtrichiae more densely concentrated than the rest of the wing; anal cell 1/3 proximally lacking microtrichiae; subbasal cell with microtrichiae virtually throughout; veins 2CUa and 2CUb completely spectral; vein 2 cu-a absent; vein 2-1A proximally tubular and distally spectral, although sometimes difficult to see; tubular vein 1 cu-a curved, incomplete/broken and not reaching the edge of 1-1A vein. Hind wing with vannal lobe very narrow, subdistally and subproximally straightened, setae evenly scattered in the margin.

**Metasoma** (Fig. 74A, H, J, K). Metasoma laterally compressed. Petiole on T1 finely sculptured only laterally, parallel-sided in proximal half and then narrowing (length 0.38, maximum width 0.20, minimum width 0.10), and with scattered pubescence on distal half. Lateral grooves delimiting the median area on T2 clearly defined and reaching the distal edge of T2 (length median area 0.20, length T2 0.20), edges of median area obscured by weak longitudinal stripes; median area as broad as long (length 0.20, maximum width 0.21, minimum width 0.10); T2 scarce pubescence throughout. T3 longer than T2 (0.22, 0.20) and with scattered pubescence throughout.

**Cocoon** (Fig. 74B). White or beige bud-like cocoon with evenly smooth silk fibers.

#### Comments.

The metasomal segments are desiccated and shrunken. The mesosoma is stout.

#### Female.

Unknown.

#### Etymology.

Doris Lagos-Kutz, Peruvian-American entomologist, who carries out research on aphid systematics and soybean host plant resistance.

#### Distribution.

Parasitized caterpillar was collected in Ecuador, Napo, Yanayacu Biological Station (Yanayacu Road), during August 2007 at 2,100 m in cloud forest.

#### Biology.

The lifestyle of this parasitoid species is solitary.

#### Host.

*Nebulosayanayacu* Miller (Notodontidae: Dioptinae) feeding on *Tibouchinalepidota* (Melastomataceae). Caterpillar was collected in third instar.

### Glyptapanteles edgardpalacioi

Taxon classificationAnimaliaHymenopteraBraconidae

Arias-Penna, sp. nov.

http://zoobank.org/186ECAF5-9694-4DE2-A3EC-2F77E736586F

Fig. 75

#### Female.

Body length 3.94 mm, antenna length 4.04 mm, fore wing length 3.89 mm.

#### Type material.

**Holotype**: ECUADOR • 1♀; EC-37411, YY-A178; Napo, Yanayacu Biological Station, Río Pumayacu, Plot 424; cloud forest; 2,095 m; -0.604722, 77.880833; 06.iii.2009; Drew Townsend leg.; caterpillar collected in third instar; cocoon formed on 23.iii.2009; adult parasitoid emerged on 08.iv.2009; (PUCE).

#### Diagnosis.

**Figure 75.**
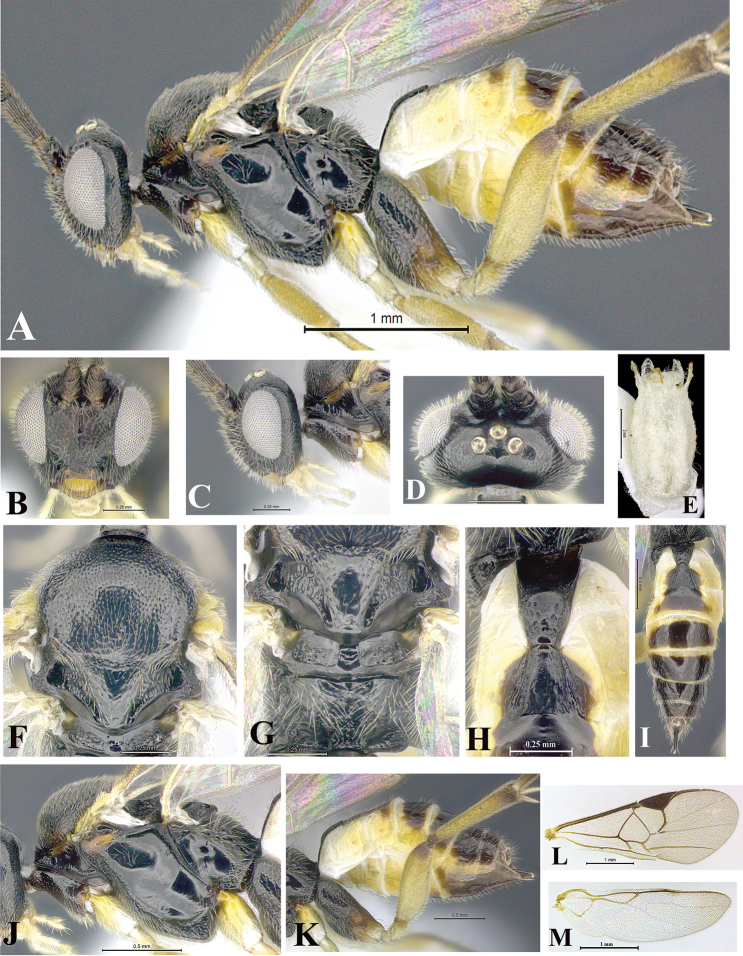
*Glyptapantelesedgardpalacioi* sp. nov. female EC-37411 YY-A178 **A** Habitus **B, D** Head **B** Frontal view **D** Dorsal view **C** Head, pronotum, propleuron, lateral view **E** Cocoon **F** Mesonotum, dorsal view **G** Scutellum, metanotum, propodeum, dorsal view **H**T1–2, dorsal view **I, K** Metasoma **I** Dorsal view **K** Lateral view **J** Mesosoma, lateral view **L, M** Wings **L** Fore **M** Hind.

Propodeal spiracle without distal carina, inner margin of eyes straight throughout (Fig. 75B), distal antennal flagellomere longer than penultimate, median area between lateral ocelli without depression (Fig. 75D), in dorsal view, proximal half of propodeum more strongly curved (Fig. 75G), petiole on T1 evenly narrowing distally (Fig. 75H, I), dorsal outer depression on hind coxa present (Fig. 75A, K), edges of median area on T2 obscured by weak longitudinal stripes (Fig. 75H, I), and ore wing with r vein curved, outer side of junction of r and 2RS veins forming a slight stub (Fig. 75L).

#### Coloration

(Fig. 75A–M). General body coloration polished black except proximally scape, distally pedicel, laterally clypeus, dorsal furrow of pronotum, epicnemial ridge, and wall just above of dorsal ATS groove with light brown/yellow-brown tints; all antennal flagellomeres brown on both sides; labrum and tegula yellow; glossa, maxillary and labial palps pale yellow/ivory. Eyes and ocelli silver. Fore and middle legs yellow except brown claws; hind legs yellow except coxae black with apex yellow, tiny brown area at the apex of the femora, apex of the tibiae, and tarsomeres brown. Petiole on T1 black and sublateral areas yellow; T2 with median area black, wide adjacent area brown and lateral ends yellow; T3 brown, dark area coincides with the width of dark area formed by both median and adjacent areas on T2; however dark area on T3 not reaching the distal edge of T3, distally with a wide yellow-brown band and proximal corners of lateral ends yellow; T4 and beyond brown; distally each tergum with a narrow transparent yellow band. In lateral view, T1–2 completely yellow; T3–4 yellow, but dorsally brown, brown area larger on T4 than T3; T5 and beyond completely brown. S1–4 completely yellow; penultimate sternum yellow, distally with a medial brown spot; hypopygium completely brown.

#### Description.

**Head** (Fig. 75A–D). Head triangular with pubescence long and dense. Proximal three antennal flagellomeres longer than wide (0.29:0.10, 0.29:0.10, 0.30:0.10), distal antennal flagellomere longer than penultimate (0.16:0.07, 0.13:0.07), antenna longer than body (4.04, 3.93); antennal scrobes-frons shallow. Face flat or nearly so with dense fine punctations, interspaces smooth and longitudinal median carina present. Frons punctate. Temple wide, punctate and interspaces clearly smooth. Inner margin of eyes straight throughout, in lateral view, eye anteriorly convex and posteriorly straight. POL shorter than OOL (0.10, 0.15). Malar suture present. Median area between lateral ocelli without depression. Vertex laterally rounded and dorsally wide.

**Mesosoma** (Fig. 75A, F, G, H). Mesosoma dorsoventrally convex. Mesoscutum proximally convex and distally flat, punctation distinct throughout and interspaces wavy/lacunose. Scutellum triangular, apex sloped and fused with BS, scutellar punctation scattered throughout, in profile scutellum flat and on same plane as mesoscutum, phragma of the scutellum partially exposed; BS only very partially overlapping the MPM; ATS demilune with short stubs delineating the area; dorsal ATS groove with semicircular/parallel carinae. Transscutal articulation with small and heterogeneous foveae, area just behind transscutal articulation depressed centrally and with same kind of sculpture as mesoscutum. Metanotum with BM wider than PFM (clearly differentiated); MPM semicircular and without median longitudinal carina; AFM without setiferous lobes and not as well delineated as PFM; PFM thick and smooth; ATM proximally with semicircular/undulate carina and distally smooth. Propodeum without median longitudinal carina, proximal half curved with medium-sized sculpture and distal half with a shallow dent at each side of nucha; distal edge of propodeum with a flange at each side and without stubs; propodeal spiracle without distal carina; nucha surrounded by very short radiating carinae. Pronotum with a distinct dorsal furrow, dorsally with a well-defined smooth band; central area of pronotum and dorsal furrow smooth, but ventral furrow with short parallel carinae. Propleuron with fine punctations throughout and dorsally without a carina. Metasternum flat or nearly so. Contour of mesopleuron straight/angulate or nearly so; precoxal groove smooth, shiny and shallow, but visible; epicnemial ridge elongated more fusiform (tapering at both ends).

**Legs** (Fig. 75A). Ventral margin of fore telotarsus entire, but with a tiny curved seta, fore telotarsus almost same width throughout and longer than fourth tarsomere (0.17, 0.10). Hind coxa finely punctate throughout, and dorsal outer depression present. Inner spur of hind tibia longer than outer spur (0.30, 0.25), entire surface of hind tibia with dense strong spines clearly differentiated by color and length. Hind telotarsus longer than fourth tarsomere (0.20, 0.17).

**Wings** (Fig. 75L, M). Fore wing with r vein slightly curved; 2RS vein straight; r and 2RS veins forming a weak, even curve at their junction and outer side of junction forming a slight stub; 2M vein slightly curved/swollen; distally fore wing [where spectral veins are] with microtrichiae more densely concentrated than the rest of the wing; anal cell 1/3 proximally lacking microtrichiae; subbasal cell with microtrichiae virtually throughout; veins 2CUa and 2CUb completely spectral; vein 2 cu-a present as spectral vein, sometimes difficult to see; vein 2-1A proximally tubular and distally spectral, although sometimes difficult to see; tubular vein 1 cu-a curved and complete, but junction with 1-1A vein spectral. Hind wing with vannal lobe narrow, subdistally and subproximally straightened, and setae evenly scattered in the margin.

**Metasoma** (Fig. 75A, H, I, K). Metasoma laterally compressed. Petiole on T1 finely sculptured only laterally, evenly narrowing distally (length 0.50, maximum width 0.25, minimum width 0.11) and with scattered pubescence concentrated in the first distal third. Lateral grooves delimiting the median area on T2 clearly defined and reaching the distal edge of T2 (length median area 0.25, length T2 0.25), edges of median area obscured by weak longitudinal stripes, median area as broad as long (length 0.25, maximum width 0.25, minimum width 0.10); T2 with scarce pubescence throughout. T3 longer than T2 (0.31, 0.25) and with scattered pubescence throughout. Pubescence on hypopygium dense.

**Cocoons** (Fig. 75E). White or beige bud-like cocoon with body ridge-shaped and evenly smooth silk fibers.

#### Male.

Unknown.

#### Etymology.

Edgard Enrique Palacio Goenaga is a Colombian entomologist. He has contributed to the knowledge of Hymenoptera, especially in ants and Ichneumonidae (mainly subfamily Pimplinae). Currently, he works at the Instituto Colombiano Agropecuario (ICA), Colombia.

#### Distribution.

Parasitized caterpillar was collected in Ecuador, Napo, Yanayacu Biological Station (Río Pumayacu), during March 2009 at 2095 m in cloud forest.

#### Biology.

The lifestyle of this parasitoid species is solitary.

#### Host.

Undetermined species of Saturniidae feeding on *Psammisia* sp. (Ericaceae). Caterpillar was collected in third instar.

### Glyptapanteles edwinnarvaezi

Taxon classificationAnimaliaHymenopteraBraconidae

Arias-Penna, sp. nov.

http://zoobank.org/0ABC155F-FCA1-432E-95EB-3BBC259CAB6F

Figs 76 , 77

#### Female.

Body length 2.73 mm, antenna length 3.78 mm, fore wing length 3.33 mm.

#### Type material.

**Holotype**: ECUADOR • 1♀; EC-42168A, YY-A005; Napo, Yanayacu Biological Station, Sendero Macuculoma, Plot 443; cloud forest; 2,014 m; -0.6, -77.883333; 11.ix.2009; Luis Salagaje leg.; caterpillar collected in third instar; cocoons formed on 08.x.2009; adult parasitoids emerged on 17.x.2009; (PUCE). **Paratypes.** • 28 (5♀, 6♂) (17♀, 0♂); EC-42168A, YY-A005; same data as for holotype; (PUCE).

#### Other material.

**Reared material**. ECUADOR: *Napo*, *Yanayacu Biological Station*, *Miraflores-Cosanga Forest*, *Plot 185*: • 1 (1♀, 0♂) (0♀, 0♂); EC-12570, YY-A045; cloud forest; 1,973 m; -0.583333, -77.866667; 21.ii.2006; Rafael Granizo leg.; caterpillar collected in second instar; cocoons on larval cuticle and formed on 21.iii.2006; adult parasitoids emerged on 30.iii.2006.

*Napo*, *Yanayacu Biological Station*, *Sendero Macuculoma*, *Plot 417*: • 5 (3♀, 1♂) (1♀, 0♂); EC-36601, YY-A067; cloud forest; 2,120 m; -0.596944, -77.869722; 10.i.2009; Earthwatch volunteers leg.; caterpillar collected in second instar; cocoons formed on 09.ii.2009; adult parasitoids emerged on 18.ii.2009.

*Napo*, *Yanayacu Biological Station*, *YanayacuForest*, *Plot 428*: • 48 (7♀, 2♂) (39♀, 0♂); EC-38137, YY-A069; cloud forest; 2,144 m; -0.596944, -77.869722; 09.iv.2009; Wilmer Simbaña leg.; caterpillar collected in third instar; cocoons formed on 01.vi.2009; adult parasitoids emerged on 12.vi.2009.

*Napo*, *Yanayacu Biological Station*, *Isla de Palmas*, *Plot 434*: • 38 (5♀, 3♂) (30♀, 0♂); EC-38988, YY-A009; cloud forest; 1,863 m, -0.541111, -77.874722; 29.v.2009; Wilmer Simbaña leg.; caterpillar collected in second instar; cocoons formed on 29.vii.2009; adult parasitoids emerged on 15.viii.2009.

#### Diagnosis.

**Figure 76.**
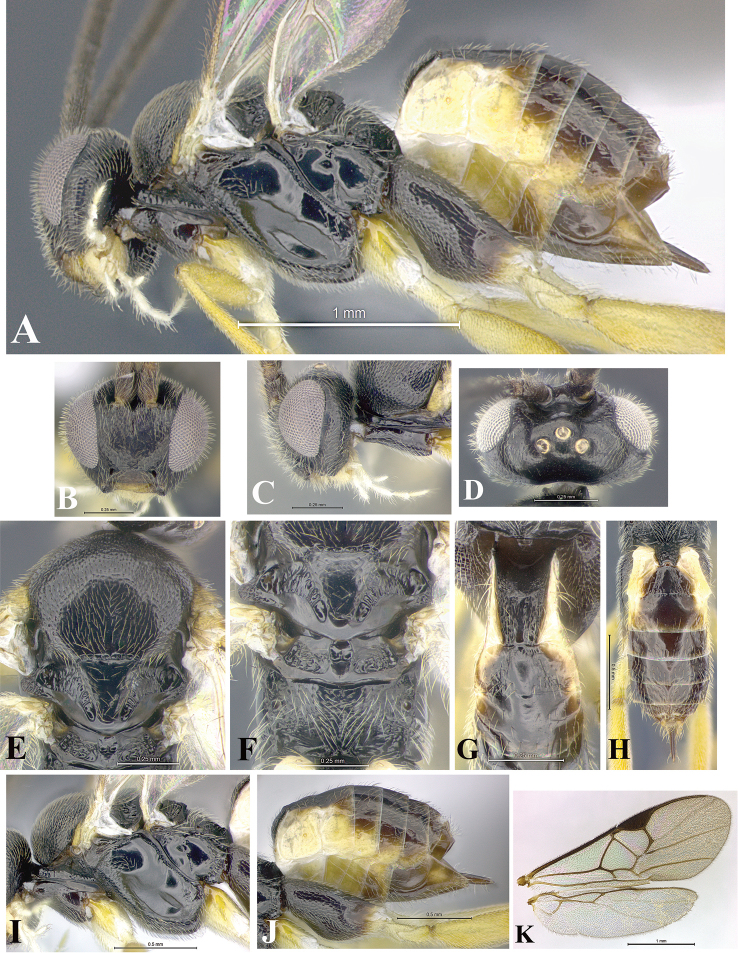
*Glyptapantelesedwinnarvaezi* sp. nov. female EC-42168A YY-A005 **A** Habitus **B, D** Head **B** Frontal view **D** Dorsal view **C** Head, pronotum, propleuron, lateral view **E** Mesonotum, dorsal view **F** Scutellum, metanotum, propodeum, dorsal view **G**T1–2, dorsal view **H, J** Metasoma **H** Dorsal view **J** Lateral view **I** Mesosoma, lateral view **K** Fore and hind wings.

**Figure 77.**
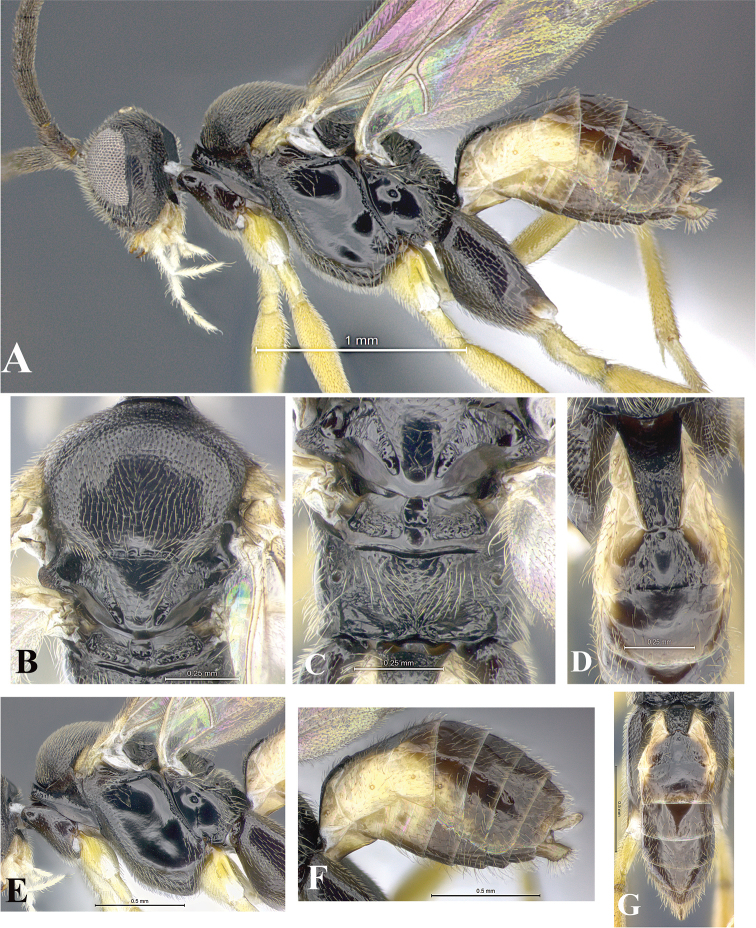
*Glyptapantelesedwinnarvaezi* sp. nov. male EC-42168A YY-A005 **A** Habitus **B** Mesonotum, dorsal view **C** Scutellum, metanotum, propodeum, dorsal view **D**T1–3, dorsal view **E** Mesosoma, lateral view **F, G** Metasoma **F** Lateral view **G** Dorsal view.

Surface of metasternum flat or nearly so, nucha surrounded by very short radiating carinae (Figs 76F, 77C), median area on T2 broader than long, edges of median area on T2 obscured by weak longitudinal stripes (Figs 76G, 77D), propodeal spiracle distally framed by faintly concave/wavy carina. Inner margin of eyes diverging slightly at antennal sockets (Fig. 76B), distal antennal flagellomere longer than penultimate, median area between lateral ocelli without depression (Fig. 76D), in dorsal view, proximal half of propodeum more strongly curved (Figs 76F, 77C), petiole on T1 evenly narrowing distally (Figs 76G, 77D), dorsal outer depression on hind coxa present (Figs 76A, J, 77A), and fore wing with r vein slightly curved, outer side of junction of r and 2RS veins forming a distinct stub (Fig. 76K).

#### Coloration

(Fig. 76A–K). General body coloration polished black except labrum and mandibles with yellow-brown tints; glossa, maxillary and labial palps, and tegulae yellow; all antennal flagellomeres brown on both sides. Eyes and ocelli silver. Fore and middle legs yellow except brown claws; hind legs yellow except black-brown coxae with apex yellow, femora with small brown spot at the apex, tibiae with both ends brown, and tarsomeres brown. Petiole on T1 dark brown and sublateral areas yellow; T2 with median and adjacent areas dark brown, and lateral ends yellow-brown; T3 mostly brown, but proximal corners yellow; T4 and beyond complete dark brown; distally each tergum with a narrow whitish transparent band. In lateral view, T1–3 completely yellow; T4 and beyond yellow, but dorsally brown, extent of brown area increasing from proximal to distal. S1–4 completely yellow; penultimate sternum and hypopygium dark brown with some spots yellow-brown.

#### Description.

**Head** (Fig. 76A–D). Head triangular with pubescence long and dense. Proximal three antennal flagellomeres longer than wide (0.28:0.09, 0.28:0.09, 0.28:0.09), distal antennal flagellomere longer than penultimate (0.15:0.06, 0.13:0.06), antenna longer than body (3.78, 2.73); antennal scrobes-frons shallow. Face flat or nearly so, with dense fine punctations, interspaces smooth and longitudinal median carina present. Frons punctate. Temple wide with punctate sculpture and interspaces clearly smooth. Inner margin of eyes diverging slightly at antennal sockets; in lateral view, eye anteriorly convex and posteriorly straight. POL shorter than OOL (0.10, 0.12). Malar suture present. Median area between lateral ocelli without depression. Vertex laterally rounded and dorsally wide.

**Mesosoma** (Fig. 76A, E, F, I). Mesosoma dorsoventrally convex. Mesoscutum proximally convex and distally flat, punctation distinct throughout and interspaces wavy/lacunose. Scutellum triangular, apex sloped and fused with BS, scutellar punctation scattered throughout, in profile scutellum flat and on same plane as mesoscutum, phragma of the scutellum partially exposed; BS only very partially overlapping the MPM; ATS demilune with a little and complete parallel carinae; dorsal ATS groove with carinae only proximally. Transscutal articulation with small and heterogeneous foveae, area just behind transscutal articulation nearly at the same level as mesoscutum (flat) and with same kind of sculpture as mesoscutum. Metanotum with BM wider than PFM (clearly differentiated); MPM circular and bisected by a median longitudinal carina; AFM with a small lobe and not as well delineated as PFM; PFM thick and smooth; ATM proximally with semircular/undulate carina and distally smooth. Propodeum with medium-sized sculpture without median longitudinal carina, proximal half curved and distal half with a shallow dent at each side of nucha; distal edge of propodeum with a flange at each side and without stubs; propodeal spiracle distally framed by faintly concave/wavy carina; nucha surrounded by very short radiating carinae. Pronotum with a distinct dorsal furrow, dorsally with a well-defined smooth band; central area of pronotum and dorsal furrow smooth, but ventral furrow with short parallel carinae. Propleuron with fine rugae and dorsally without a carina. Metasternum flat or nearly so. Contour of mesopleuron straight/angulate or nearly so; precoxal groove smooth, shiny and shallow, but visible; epicnemial ridge elongated more fusiform (tapering at both ends).

**Legs.** Ventral margin of fore telotarsus entire, but with a tiny curved seta, fore telotarsus proximally narrow and distally wide, and longer than fourth tarsomere (0.15, 0.09). Hind coxa finely punctate throughout, and dorsal outer depression present. Inner spur of hind tibia longer than outer spur (0.25, 0.17), entire surface of hind tibia with dense strong spines clearly differentiated by color and length. Hind telotarsus as equal in length as fourth tarsomere (0.17, 0.17).

**Wings** (Fig. 76K). Fore wing with r vein slightly curved; 2RS vein straight; r and 2RS veins forming a weak, even curve at their junction and outer side of junction forming a slight stub; 2M vein slightly curved/swollen; distally fore wing [where spectral veins are] with microtrichiae more densely concentrated than the rest of the wing; anal cell 1/3 proximally lacking microtrichiae; subbasal cell with microtrichiae virtually throughout; veins 2CUa and 2CUb completely spectral; vein 2 cu-a present as spectral vein, sometimes difficult to see; vein 2-1A proximally tubular and distally spectral, although sometimes difficult to see; tubular vein 1 cu-a straight and complete, but junction with 1-1A vein spectral. Hind wing with vannal lobe very narrow, subdistally and subproximally straightened, and setae evenly scattered in the margin.

**Metasoma** (Fig. 76A, G, H, J). Metasoma laterally compressed. Petiole on T1 finely sculptured throughout, evenly narrowing distally (length 0.40, maximum width 0.22, minimum width 0.10), and with scattered pubescence on distal half. Lateral grooves delimiting the median area on T2 clearly defined and reaching the distal edge of T2 (length median area 0.20, length T2 0.20), edges of median area obscured by weak longitudinal stripes, median area broader than long (length 0.20, maximum width 0.25, minimum width 0.10); T2 with scarce pubescence throughout. T3 longer than T2 (0.25, 0.20) and with scattered pubescence throughout. Pubescence on hypopygium dense.

**Cocoons.** Stack on larval cuticle.

#### Male

(Fig. 77A–G). Similar in coloration to female, but slimmer than female.

#### Etymology.

Edwin Narvaez is a botanist who has helped in the identification of food plants at Yanayacu Biological Station. He works at Herbario Nacional del Ecuador (QCNE), Quito, Ecuador.

#### Distribution.

Parasitized caterpillars were collected in Ecuador, Napo, Yanayacu Biological Station (Isla de Palmas, Sendero Macuculoma, Miraflores-Cosanga Forest, and YanayacuForest), during February 2006; and January, April, May, and September 2009 at 1,863 m, 1,973 m, 2,014 m, 2,144 m, and 2,120 m in cloud forest.

#### Biology.

The lifestyle of this parasitoid species is gregarious.

#### Host.

Undetermined species of Nymphalidae: Ithomiinae feeding on *Cestrummegalophyllum* (Solanaceae). Undetermined species of Apatelodidae feeding on *Columnea* sp., *C.ericae* and *Alloplectustetragonoides* (Gesneriaceae). Caterpillars were collected in second and third instar.

### Glyptapanteles eowilsoni

Taxon classificationAnimaliaHymenopteraBraconidae

Arias-Penna, sp. nov.

http://zoobank.org/3BC39D82-795F-4FF5-935E-F417334B20CD

Figs 78 , 79

#### Female.

Body length 2.53 mm, antenna length 2.68 mm, fore wing length 2.47 mm.

#### Type material.

**Holotype**: COSTA RICA • 1♀; 01-SRNP-1148, DHJPAR0000005; Área de Conservación Guanacaste, Guanacaste, Sector El Hacha, Sendero Tigre; 280 m; 11.03172, -85.52615; 18.ix.2001; Lucia Ríos leg.; caterpillar collected in fourth instar; beige short single cocoons arranged in two rows of cordwood on each side of cadaver, at right angles to the cadaver axis, cocoons adhered to the leaf; adult parasitoids emerged on 23.ix.2006; (CNC). **Paratypes.** • 17 (5♀, 2♂) (0♀, 10♂); 01-SRNP-1148, DHJPAR0000005; same data as for holotype; (CNC).

#### Other material.

**Reared material.** COSTA RICA: *Área de Conservación Guanacaste*, *Guanacaste*, *Sector El Hacha*, *Sendero Bejuquilla*: • 35 (3♀, 1♂) (31♀, 0♂); 98-SRNP-13786, DHJPAR0000106; intergrade dry-rain forest; 280 m; 11.03004, -85.52699; 13.x.1998; Lucia Ríos leg.; small cylindrical cocoons somewhat adhered together and adhered to the leaf substrate; adult parasitoids emerged on 18.x.1998.

*Área de Conservación Guanacaste*, *Guanacaste*, *Sector Santa Rosa*, *Sendero Natural*: • 2 (2♀, 0♂) (0♀, 0♂); 03-SRNP-27829, DHJPAR0000271; dry forest; 290 m; 10.83575, -85.61253; 05.xii.2003; Freddy Quesada leg.; caterpillar collected in third instar; cocoons adhered to the leaf substrate; adult parasitoids emerged on 22.xii.2003; this is apparently a case where we got both a tachinid and braconids out of the same caterpillar; the single tachinid puparium was in the litter and the braconid cocoons adhered to the leaf.

*Área de Conservación Guanacaste*, *Guanacaste*, *Sector Del Oro*, *Quebrada Trigal*: • 1 (1♀, 0♂) (0♀, 0♂); 03-SRNP-28397, DHJPAR0000043; intergrade dry-rain forest; 290 m; 11.02681, -85.49547; 01.ix.2003; Roster Moraga leg.; caterpillar collected in fourth instar; small white cocoons irregularly adhered to each other on the leaf substrate, no common spinning web; adult parasitoids emerged on 12.ix.2003.

*Área de Conservación Guanacaste*, *Guanacaste*, *Sector Del Oro*, *Sendero Puertas*: • 25 (5♀, 0♂) (20♀, 0♂); 10-SRNP-22472, DHJPAR0041695; intergrade dry-rain forest; 400 m; 11.01087, -85.48817; 10.xi.2010; Roster Moraga leg.; caterpillar collected in fourth instar; cocoons adhered to the leaf substrate; adult parasitoids emerged on 02.xii.2010.

*Área de Conservación Guanacaste*, *Guanacaste*, *Sector Pitilla*, *Sendero Trocha*: • 27 (3♀, 3♂) (18♀, 3♂); 09-SRNP-71062, DHJPAR0039969; rain forest; 540 m; 10.9971, -85.40315; 26.vi.2009; Ricardo Calero leg.; caterpillar collected in fourth instar; cocoons adhered to the leaf substrate and formed on 04.vii.2009; adult parasitoids emerged on 12.vii.2009. • 30 (5♀, 5♂) (18♀, 2♂); 09-SRNP-71063, DHJPAR0039966; same data as for preceding.

#### Malaise-trapped material.

COSTA RICA: *Área de Conservación Guanacaste*, *Guanacaste*, *Sector El Hacha*, *Sendero Bejuquilla*: • 1 (1♀, 0♂) (0♀, 0♂); 99-SRNP-18935, DHJPAR0012634; intergrade dry-rain forest; 280 m; 11.03004, -85.52699; Malaise trap; 08.iii.1999; DH Janzen & W Hallwachs leg.

#### Diagnosis.

**Figure 78.**
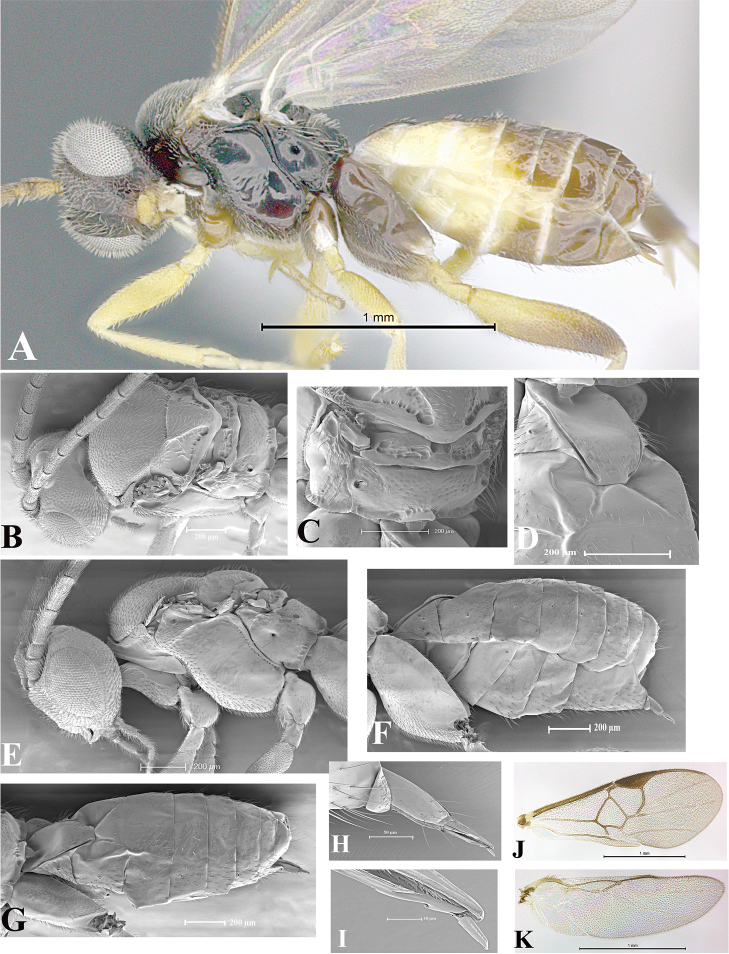
*Glyptapanteleseowilsoni* sp. nov. female 98-SRNP-13786 DHJPAR0000106, 01-SRNP-11148 DHJPAR0000005 **A** Habitus **B, E** Head, mesosoma **B** Dorsolateral view **E** lateral view **C** Metanotum, propodeum, dorsal view **D**T1–2, dorsolateral view **F, G** Metasoma **F** lateral view **G** Dorsolateral view **H, I** Genitalia **H** Genitalia: hypopygium, ovipositor, ovipositor sheaths, lateral view **I** Ovipositor detail **J, K** Wings **J** Fore **K** Hind.

**Figure 79.**
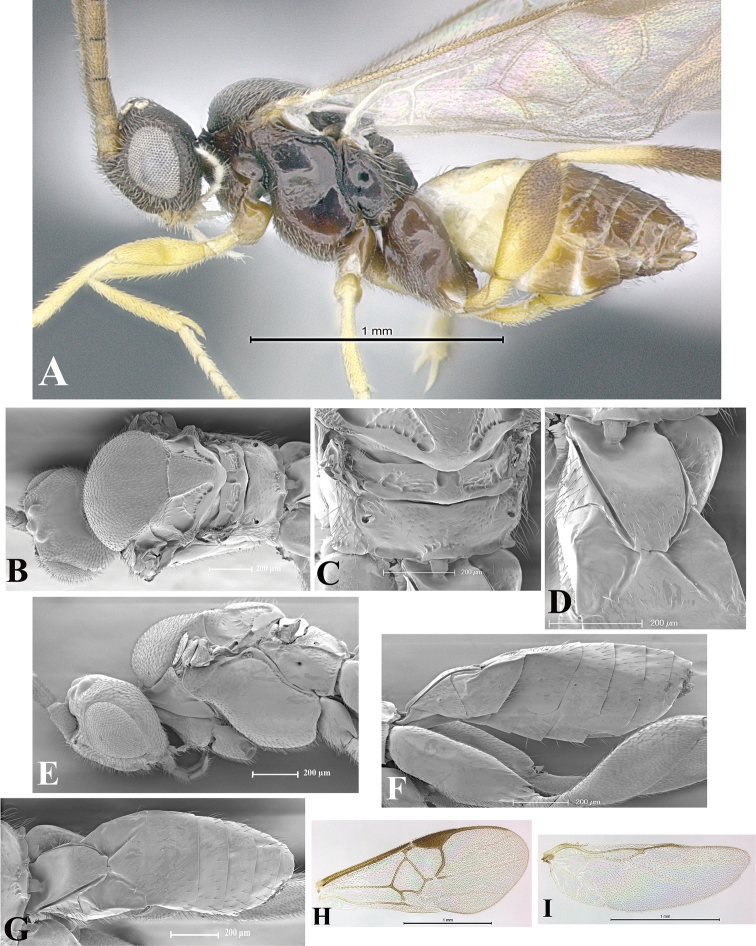
*Glyptapanteleseowilsoni* sp. nov. male 98-SRNP-13786 DHJPAR0000106, 01-SRNP-11148 DHJPAR0000005 **A** Habitus **B, E** Head, mesosoma **B** Dorsolateral view **E** lateral view **C** Metanotum, propodeum, dorsal view **D**T1–2, dorsal view **F, G** Metasoma **F** lateral view **G** Dorsolateral view **H, I** Wings **H** Fore **I** Hind.

Anteroventral contour of mesopleuron convex (Figs 78A, E, 79A, E), distal antennal flagellomere longer than penultimate, propodeal spiracle without distal carina (Figs 78B, C, 79B, C), scutellum in profile flat and on same plane as mesoscutum (Figs 78E, 79E), fore wing with 2RS slightly convex, outer side of junction of r and 2RS veins not forming a stub (Figs 78J, 79H), and lateral grooves delimiting the median area on T2 distally losing definition (Figs 78D, G, 79D, G).

#### Coloration

(Fig. 78A). Body coloration polished dark brown; all antennal flagellomeres dorsally lighter (light brown) than ventrally (dark brown); labrum, mandibles, scape, pedicel, and tegulae yellow-brown; glossa, maxillary and labial palps yellow. Eyes silver and ocelli yellowish. Fore and middle legs yellow except brown coxae and claws; hind legs yellow except dark brown coxae, most of the femora, tibiae and tarsomeres brown. Petiole on T1 with 2/3 proximal reddish brown, 1/3 distal black, contours black, and sublateral areas yellow; T2 with median and adjacent areas brown, and lateral ends yellow; T3 and beyond completely dark brown; distally each tergum with a narrow whitish transparent band. In lateral view, T1–2 completely yellow; T3–4 dorsally brown, but ventrally yellow; T5 and beyond brown. S1–3 yellow; S4–5 yellow, medially with a small brown area, which is more extended in S5 than S4; hypopygium completely brown; ovipositor sheath brown.

#### Description.

**Head** (Fig. 78A, B, E) Head rounded with pubescence short and dense. Proximal three antennal flagellomeres longer than wide (0.19:0.07, 0.18:0.07, 0.18:0.07), distal antennal flagellomere longer than penultimate (0.12:0.05, 0.09:0.06), antenna longer than body (2.68, 2.53); antennal scrobes-frons shallow. Face with dense fine punctations, interspaces wavy, distal half dented only laterally and longitudinal median carina present. Frons punctate. Temple wide, punctate and interspaces wavy. Inner margin of eyes diverging slightly at antennal sockets; in lateral view, eye anteriorly convex and posteriorly straight. POL shorter than OOL (0.09, 0.14). Malar suture present. Median area between lateral ocelli slightly depressed. Vertex laterally rounded and dorsally wide.

**Mesosoma** (Fig. 78A–D). Mesosoma dorsoventrally convex. Mesoscutum proximally convex and distally flat, punctation distinct throughout and interspaces wavy/lacunose. Scutellum triangular, apex sloped and fused with BS, but not in the same plane, scutellar punctation distinct throughout, in profile scutellum flat and on same plane as mesoscutum, phragma of the scutellum partially exposed; BS only very partially overlapping the MPM; ATS demilune with short stubs delineating the area; dorsal ATS groove with carinae only proximally. Transscutal articulation with small and heterogeneous foveae, area just behind transscutal articulation depressed centrally and with same kind of sculpture as mesoscutum. Metanotum with BM wider than PFM (clearly differentiated); MPM circular and bisected by a median longitudinal carina; AFM without setiferous lobes and not as well delineated as PFM; PFM thick and smooth; ATM proximally with semircular/undulate carina and distally smooth. Propodeum without median longitudinal carina, proximal half curved with medium-sized sculpture and distal half relatively polished and with a shallow dent at each side of nucha; distal edge of propodeum with a flange at each side and without stubs; propodeal spiracle without distal carina; nucha surrounded by very short radiating carinae. Pronotum with a distinct dorsal furrow, dorsally with a well-defined smooth band; central area of pronotum smooth, but both dorsal and ventral furrows with short parallel carinae. Propleuron with fine punctations throughout and dorsally with a carina. Metasternum flat or nearly so. Contour of mesopleuron convex; precoxal groove deep with faintly transverse lineate sculpture; epicnemial ridge convex, teardrop-shaped.

**Legs.** Ventral margin of fore telotarsus slightly excavated and with a tiny curved seta, fore telotarsus almost same width throughout and longer than fourth tarsomere (0.10, 0.06). Hind coxa with punctation only on ventral surface and dorsal outer depression present. Inner spur of hind tibia longer than outer spur (0.27, 0.16), entire surface of hind tibia with dense strong spines clearly differentiated by color and length. Hind telotarsus as equal in length as fourth tarsomere (0.11, 0.10).

**Wings** (Fig. 78J, K). Fore wing with r vein slightly curved; 2RS vein slightly convex to convex; r and 2RS veins forming an angle at their junction and outer side of junction not forming a stub; 2M vein slightly curved/swollen; distally fore wing [where spectral veins are] with microtrichiae more densely concentrated than the rest of the wing; anal cell 1/3 proximally lacking microtrichiae; subbasal cell with a small smooth area; vein 2CUa absent and 2CUb spectral; vein 2 cu-a absent; vein 2-1A proximally tubular and distally spectral, although sometimes difficult to see; tubular vein 1 cu-a curved, incomplete/broken and not reaching the edge of 1-1A vein. Hind wing with vannal lobe wide, subdistally straightened, subproximally straightened, and setae present only proximally.

**Metasoma** (Fig. 78A, D, F–I). Metasoma laterally compressed. Petiole on T1 finely sculptured only distally, virtually parallel-sided over most of length, but narrowing over distal 1/3 (length 0.35, maximum width 0.20, minimum width 0.08), and with scattered pubescence concentrated in the first distal third. Lateral grooves delimiting the median area on T2 distally losing definition (length median area 0.14, length T2 0.17), edges of median area polished and lateral grooves deep, median area broader than long (length 0.14, maximum width 0.21, minimum width 0.08); T2 with scattered pubescence only distally. T3 longer than T2 (0.22, 0.17) and with scattered pubescence throughout. Pubescence on hypopygium dense.

**Cocoons** (Fig. 4O). White, beige or light brown oval cocoons with ordered silk fibers and covered by a net. Single oval cocoons somewhat adhered together and arranged in two rows of cordwood on each side of cadaver and adhered to the leaf substrate.

#### Comments.

Both sexes with slim bodies.

#### Male

(Fig. 79A–I). Similar in coloration and shape to female.

#### Etymology.

Edward Osborne Wilson is considered to be the world’s leading living authority in myrmecology. He has been called “the father of sociobiology” and “the father of biodiversity”.

#### Distribution.

Parasitized caterpillars were collected in Costa Rica, ACG, Sector Del Oro (Quebrada Trigal and Sendero Puertas), Sector El Hacha (Sendero Bejuquilla and Sendero Tigre), Sector Pitilla (Sendero Trocha), Sector Santa Rosa (Sendero Natural), during September 1992, October 1998, September and December 2003, June 2009, and November 2010 at 280 m, 290 m, 400 m, and 540 m in intergrade dry-rain forest and rain forest. Adult parasitoid was collected in Costa Rica, ACG, Sector El Hacha (Sendero Bejuquilla) during, March 1999 at 280 m in intergrade dry-rain forest.

#### Biology.

The lifestyle of this parasitoid species is gregarious.

#### Host.

*Calledemaplusia* Felder (Noctuidae: Nystaleinae) (Fig. 4O) feeding on *Hirtellaamericana*, *H.guatemalensis*, *H.racemosa*, *H.triandra* and *Licaniaarborea* (Chrysobalanaceae). Caterpillars were collected in third and fourth instar.

### Glyptapanteles erictepei

Taxon classificationAnimaliaHymenopteraBraconidae

Arias-Penna, sp. nov.

http://zoobank.org/BE0D1B09-CCCF-44B1-89CD-FA0EFC191AD5

Figs 80 , 81

#### Female.

Body length 2.87 mm, antenna length 3.73 mm, fore wing length 3.38 mm.

#### Type material.

**Holotype**: ECUADOR • 1♀; EC-29576, YY-A108; Napo, Yanayacu Biological Station, Río Aliso, Isla del Río Aliso; cloud forest; 2,100 m; -0.633333, -77.9, 23.i.2008; CAPEA leg.; caterpillar collected in third instar; cocoon formed on 09.ii.2008; adult parasitoid emerged on 10.iii.2008; (PUCE). **Paratypes.** • 1 (1♀, 0♂) (0♀, 0♂); EC-29352, YY-A169; same data as for holotype except: cocoon formed on 19.ii.2008; adult parasitoid emerged on 21.iii.2008; (PUCE). • 1 (1♀, 0♂) (0♀, 0♂); EC-29355, YY-A172; same data as for holotype except: cocoon formed on 20.ii.2008; adult parasitoid emerged on 24.iii.2008; (PUCE). • 1 (1♀, 0♂) (0♀, 0♂); EC-29357, YY-A110; same data as for holotype except: cocoon formed on 20.ii.2008; adult parasitoid emerged on 21.iii.2008; (PUCE). • 1 (1♀, 0♂) (0♀, 0♂); EC-29380, YY-A018; same data as for holotype except: cocoon formed on 20.ii.2008; adult parasitoid emerged on 21.iii.2008; (PUCE). • 1 (0♀, 1♂) (0♀ + 0♂); EC-29575, YY-A119; same data as for holotype except: adult parasitoid emerged on 12.iii.2008; (PUCE). • 1 (0♀ + 1♂) (0♀ + 0♂); EC-29582, YY-A181; same data as for holotype except: cocoon formed on 08.ii.2008; adult parasitoid emerged on 21.iii.2008; (PUCE). • 1 (1♀, 0♂) (0♀, 0♂); EC-29586, YY-A174; same data as for holotype except: cocoon formed on 08.ii.2008; adult parasitoid emerged on 20.iii.2008; (PUCE).

#### Diagnosis.

**Figure 80.**
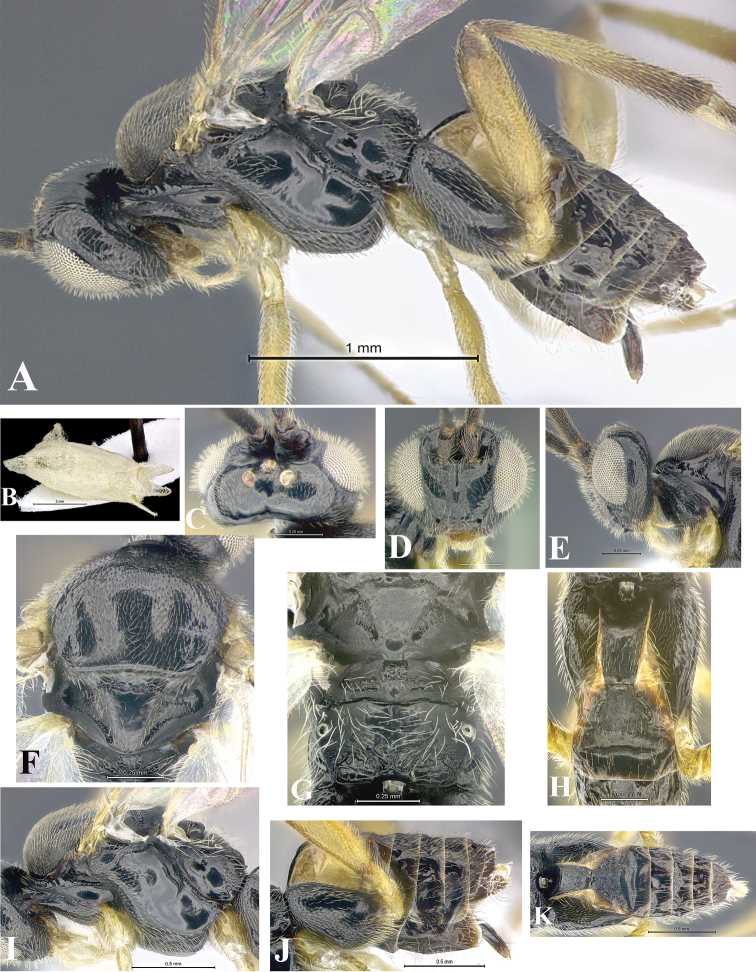
*Glyptapanteleserictepei* sp. nov. female EC-29355 YY-A172, EC-29576 YY-A108 **A** Habitus **B** Cocoon **C, D** Head **C** Dorsal view **D** Frontal view **E** Head, pronotum, propleuron, lateral view **F** Mesonotum, dorsal view **G** Scutellum, metanotum, propodeum, dorsal view **H**T1–3, dorsal view **I** Mesosoma, lateral view **J, K** Metasoma **J** Lateral view **K** Dorsal view.

**Figure 81.**
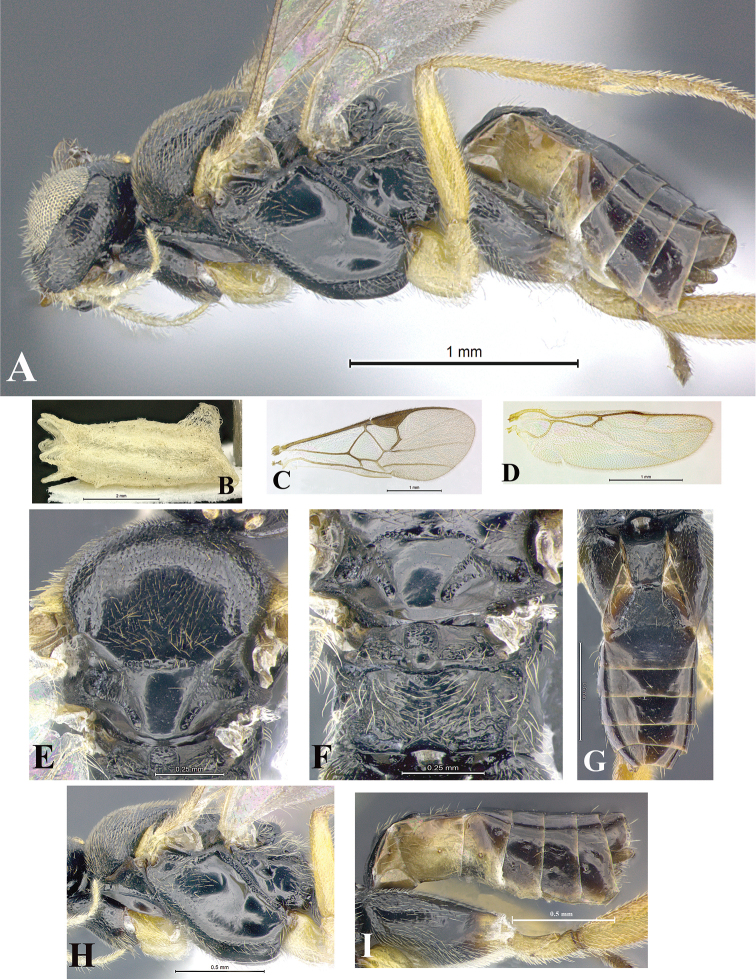
*Glyptapanteleserictepei* sp. nov. male EC-29575 YY-A119, EC-29582 YY-A181 **A** Habitus **B** Cocoon **C, D** Wings **C** Fore **D** Hind **E** Mesonotum, dorsal view **F** Scutellum, metanotum, propodeum, dorsal view **G, I** Metasoma **G** Dorsal view **I** Lateral view **H** Mesosoma, lateral view.

Precoxal groove smooth and shiny (Figs 80I, 81H), scutellar punctation scattered throughout (Figs 80F, 81E), vertex in dorsal view wide (Fig. 80C), mesoscutum punctation distinct throughout (Figs 80F, 81E), T3 as long as T2 (Figs 80K, 81G), propodeum with a median longitudinal dent (Figs 80G, 81F), petiole on T1 finely sculptured (Figs 80H, 81G), lateral grooves delimiting the median area on T2 distally losing definition on T2 (Figs 80H, 81G), and fore wing with r vein straight, outer side of junction of r and 2RS veins forming a stub (Fig. 81C).

#### Coloration

(Fig. 80A–K). General body coloration polished black except labrum, and mandibles yellow-brown; glossa, maxillary and labial palps, and tegulae yellow; proximal ring on scape and distal ring in pedicel reddish brown; all antennal flagellomeres brown on both sides. Eyes silver and ocelli reddish (in preserved specimen). Fore and middle legs yellow except tarsomeres proximally yellow-brown and distally brown, and claws brown; hind legs yellow except black coxae, brown apex of femora, both ends of tibiae brown, and brown tarsomeres. Petiole on T1 black and sublateral areas yellow; T2 with median and adjacent areas black, and narrow lateral ends yellow-brown; T3 and beyond black; distally each tergum with a narrow whitish transparent band. In lateral view, T1–2 completely yellow; T3 yellow, but dorsally brown; T4 and beyond completely brown-black. S1–3 completely yellow; S4 and beyond completely brown-black; ovipositor sheaths brown-black.

#### Description.

**Head** (Fig. 80A, C–E). Head rounded with pubescence long and dense. Proximal three antennal flagellomeres longer than wide (0.27:0.08, 0.29:0.08, 0.29:0.08), distal antennal flagellomere longer than penultimate (0.12:0.05, 0.10:0.05), antenna longer than body (3.73, 2.87); antennal scrobes-frons shallow. Face with depression only laterally and dense fine punctations, interspaces smooth and longitudinal median carina present. Frons smooth. Temple wide, punctate and interspaces clearly smooth. Inner margin of eyes diverging slightly at antennal sockets; in lateral view, eye anteriorly convex and posteriorly straight. POL shorter than OOL (0.10, 0.14). Malar suture present. Median area between lateral ocelli without depression. Vertex laterally rounded and dorsally wide.

**Mesosoma** (Fig. 80A, F, G, I). Mesosoma dorsoventrally convex. Mesoscutum with narrow grooves laterally, punctation distinct throughout and interspaces smooth. Scutellum triangular, apex sloped and fused with BS, scutellar punctation scattered throughout, in profile scutellum flat and on same plane as mesoscutum, phragma of the scutellum partially exposed; BS only very partially overlapping the MPM; ATS demilune with short stubs delineating the area; dorsal ATS groove with carinae only proximally. Transscutal articulation with small and heterogeneous foveae, area just behind transscutal articulation with a smooth and shiny sloped transverse strip. Metanotum with BM upward; MPM circular without median longitudinal carina; AFM without setiferous lobes and not as well delineated as PFM; PFM thick and smooth; ATM proximally with semircular/undulate carina and distally smooth. Propodeum with a median longitudinal dent, but no trace of median longitudinal carina, proximal half curved with medium-sized sculpture and distal half with a shallow dent at each side of nucha or rugose; distal edge of propodeum with a flange at each side and without stubs; propodeal spiracle distally framed by faintly concave/wavy carina; nucha surrounded by very short radiating carinae. Pronotum with a distinct dorsal furrow, dorsally with a well-defined smooth band; central area of pronotum and dorsal furrow smooth, but ventral furrow with short parallel carinae. Propleuron with fine punctations throughout and dorsally without a carina. Metasternum flat or nearly so. Contour of mesopleuron straight/angulate or nearly so; precoxal groove smooth, shiny and shallow, but visible; epicnemial ridge elongated more fusiform (tapering at both ends).

**Legs.** Ventral margin of fore telotarsus entire without seta, fore telotarsus proximally narrow and distally wide, and longer than fourth tarsomere (0.17, 0.09). Hind coxa finely punctate throughout, and dorsal outer depression present. Inner spur of hind tibia longer than outer spur (0.25, 0.20), entire surface of hind tibia with dense strong spines clearly differentiated by color and length. Hind telotarsus longer than fourth tarsomere (0.20, 0.15).

**Wings** (Fig. 81C, D). Fore wing with r vein straight; r and 2RS veins forming a weak, even curve at their junction and outer side of junction forming a distinct stub; 2M vein slightly curved/swollen; distally fore wing [where spectral veins are] with microtrichiae more densely concentrated than the rest of the wing; anal cell 1/3 proximally lacking microtrichiae; subbasal cell with microtrichiae virtually throughout; veins 2CUa and 2CUb completely spectral; vein 2 cu-a present as spectral vein, sometimes difficult to see; vein 2-1A proximally tubular and distally spectral, although sometimes difficult to see; tubular vein 1 cu-a curved and complete, but junction with 1-1A vein spectral. Hind wing with vannal lobe very narrow, subdistally and subproximally straightened, and setae absent proximally, but scattered distally.

**Metasoma** (Fig. 80A, H, J, K). Metasoma laterally compressed. Petiole on T1 finely sculptured on distal half, evenly narrowing distally (length 0.45, maximum width 0.25, minimum width 0.15), and with scattered pubescence on distal half. Lateral grooves delimiting the median area on T2 clearly defined and reaching the distal edge of T2 (length median area 0.20, length T2 0.20), edges of median area obscured by weak longitudinal stripes, median area broader than long (length 0.20, maximum width 0.33, minimum width 0.10); T2 with scarce pubescence throughout. T3 as long as T2 (0.21, 0.20) and with scattered pubescence throughout. Pubescence on hypopygium dense.

**Cocoon** (Figs 4A, 80B, 81B). White or beige bud-like cocoon with body ridge-shaped and evenly smooth silk fibers.

#### Comments.

The ovipositor sheath is thick and curved. Both sexes have stout bodies.

#### Male

(Fig. 81A–I). Similar in coloration and shape to female.

#### Etymology.

Eric J. Tepe is an American botanist who studies wild potatoes and the relatives of black pepper (*Piper* spp., Piperaceae). Currently, he works at the University of Cincinnati, OH, USA.

#### Distribution.

Parasitized caterpillars were collected in Ecuador, Napo, Yanayacu Biological Station (Río Aliso), during January 2008 at 2,100 m in cloud forest.

#### Biology.

The lifestyle of this parasitoid species is solitary.

#### Host.

*Actinotestratonice* Latreille (Nymphalidae: Acraeinae) feeding on *Eratopolymnioides* (Asteraceae). Caterpillars were collected in third instar.

### Glyptapanteles felipesotoi

Taxon classificationAnimaliaHymenopteraBraconidae

Arias-Penna, sp. nov.

http://zoobank.org/FDEE9605-D037-45B0-9D78-53E40CF6B2FE

Figs 82 , 83

#### Female.

Body length 2.92 mm, antenna length 3.43 mm, fore wing length 3.38 mm.

#### Type material.

**Holotype**: ECUADOR • 1♀; EC-12321, YY-A122; Napo, Yanayacu Biological Station, Forest Aguilar, Plot 181; cloud forest; 2,241 m; -0.616667, -77.9; 17.ii.2006; Aaron Fox leg.; caterpillar collected in prepupa; cocoons formed on 07.iv.2006; adult parasitoids emerged on 12.iv.2006; (PUCE). **Paratypes.** • 69 (5♀, 6♂) (46♀, 12♂); EC-12321, YY-A122; same data as for holotype; (PUCE).

#### Diagnosis.

**Figure 82.**
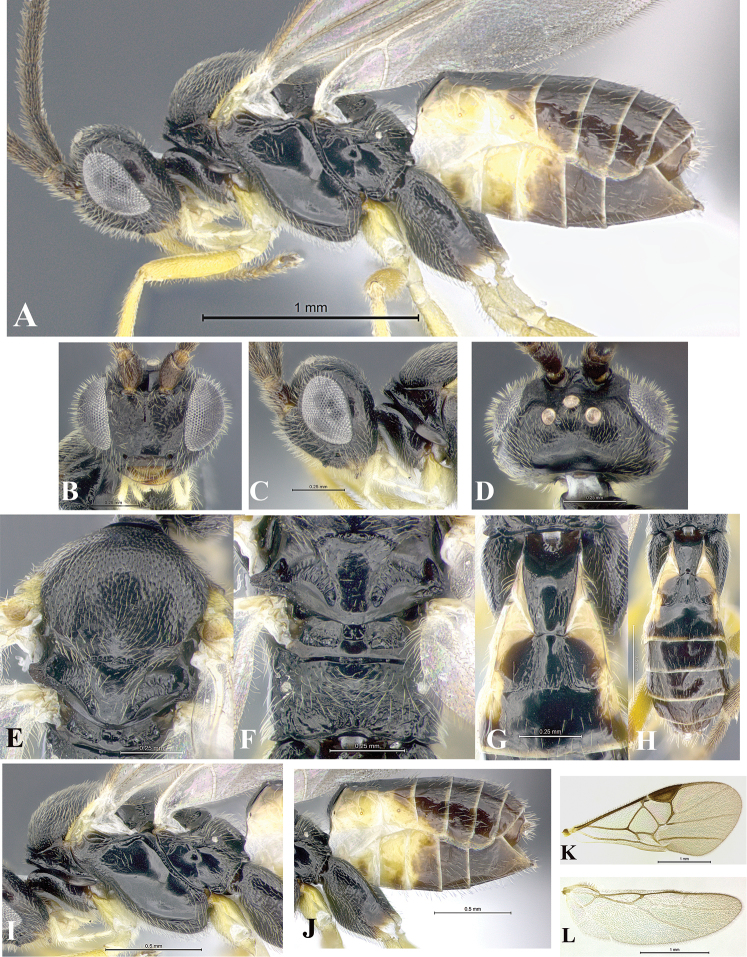
*Glyptapantelesfelipesotoi* sp. nov. female EC-12321 YY-A122 **A** Habitus **B, D** Head **B** Frontal view **D** Dorsal view **C** Head, pronotum, propleuron, lateral view **E** Mesonotum, dorsal view **F** Scutellum, metanotum, propodeum, dorsal view **G**T1–3, dorsal view **H, J** Metasoma **H** Dorsal view **J** Lateral view **I** Mesosoma, lateral view **K, L** Wings **K** Fore **L** Hind.

**Figure 83.**
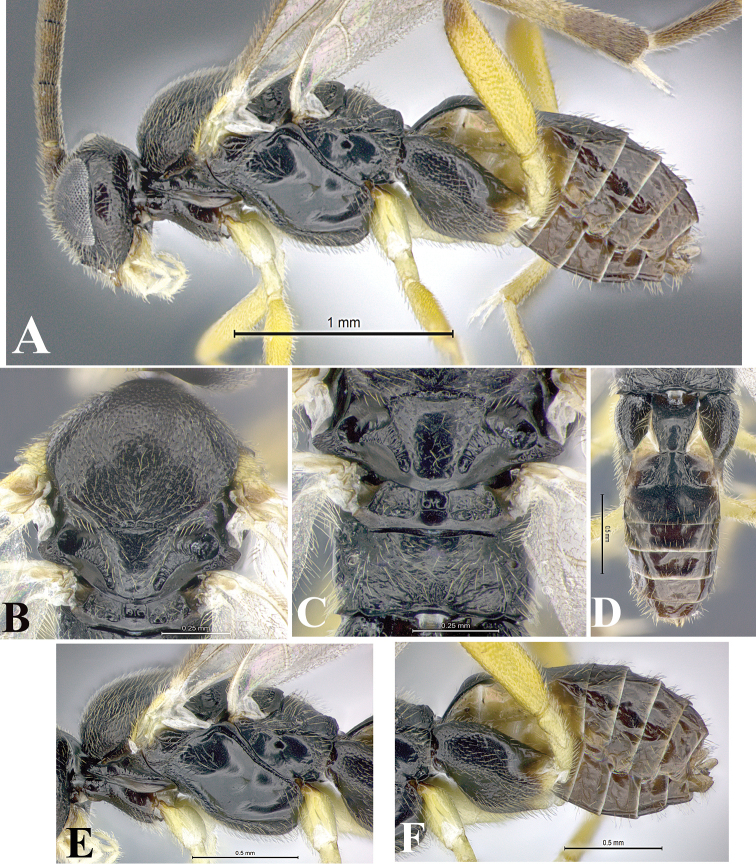
*Glyptapantelesfelipesotoi* sp. nov. male EC-12321 YY-A122 **A** Habitus **B** Mesonotum, dorsal view **C** Scutellum, metanotum, propodeum, dorsal view **D, F** Metasoma **D** Dorsal view **F** Lateral view **E** Mesosoma, lateral view.

Distal half of propodeum rugose (Figs 82F, 83C), precoxal groove indistinct (Figs 82I, 83E), on pronotum central area smooth, but both dorsal and ventral furrows with short parallel carinae (Figs 82C, 83E), anterior furrow of metanotum without setiferous lobes (Figs 82F, 83C), petiole on T1 parallel-sided in proximal half, then narrowing (Figs 82G, 83D), edges of median area on T2 obscured by weak longitudinal stripes (Figs 82G, H, 83D), dorsal outer depression on hind coxa present (Figs 82A, J, 83A, F), and fore wing with r vein curved, outer side of junction of r and 2RS veins forming a stub (Fig. 82K).

#### Coloration

(Fig. 82A–L). General body coloration polished black except labrum and mandibles yellow-brown; glossa, maxillary and labial palps, and tegulae yellow; proximal ring on scape, and distal ring on pedicel reddish brown; all antennal flagellomeres brown on both sides. Eyes silver and ocelli reddish (in preserved specimen). Fore and middle legs yellow except tarsomeres, which coloration intensity increasing from proximal (yellow-brown) to distal (brown), and claws brown; hind legs yellow except black coxae, a tiny brown spot of femora, both ends of tibiae brown, and tarsomeres brown. Petiole on T1 black and sublateral areas yellow; T2 with median and adjacent areas black, and lateral ends yellow-brown; T3 mostly black, but with proximal corners yellow-brown; T4 and beyond completely black; distally each tergum with a narrow yellowish band. In lateral view, T1–2 completely yellow; T3 yellow, but dorsally brown; T4 and beyond brown. S1–4 yellow, medially brown, brown area increasing from proximal to distal; penultimate sternum and hypopygium completely brown.

#### Description.

**Head** (Fig. 82A–D). Head rounded with pubescence long and dense. Proximal three antennal flagellomeres longer than wide (0.27:0.08, 0.29:0.08, 0.29:0.08), distal antennal flagellomere longer than penultimate (0.12:0.05, 0.10:0.05), antenna longer than body (3.43, 2.92); antennal scrobes-frons shallow. Face flat or nearly so, with dense fine punctations, interspaces smooth and longitudinal median carina present. Frons smooth. Temple wide, punctate and interspaces clearly smooth. Inner margin of eyes diverging slightly at antennal sockets; in lateral view, eye anteriorly convex and posteriorly straight. POL shorter than OOL (0.10, 0.14). Malar suture present. Median area between lateral ocelli slightly depressed. Vertex laterally rounded and dorsally wide.

**Mesosoma** (Fig. 82A, E, F, I). Mesosoma dorsoventrally convex. Mesoscutum with narrow grooves/dents taking the place of notauli, punctation distinct throughout and interspaces smooth. Scutellum long and slender, apex of scutellum sloped and fused with BS, scutellar punctation distinct peripherally and absent centrally, in profile scutellum flat and on same plane as mesoscutum, phragma of the scutellum partially exposed; BS only very partially overlapping the MPM; ATS demilune entirely covered by parallel carinae; dorsal ATS groove with carinae only proximally. Transscutal articulation with small and heterogeneous foveae, area just behind transscutal articulation smooth, shiny and nearly at the same level as mesoscutum (flat). Metanotum with BM wider than PFM (clearly differentiated); MPM semicircular and bisected by a median longitudinal carina; AFM without setiferous lobes and not as well delineated as PFM; PFM thick, smooth and with a distal flat flange; ATM proximally with a groove with some sculpturing and distally smooth. Propodeum without median longitudinal carina, proximal half weakly curved with medium-sized sculpture and distal half rugose; distal edge of propodeum with a flange at each side and without stubs; propodeal spiracle without distal carina; nucha surrounded by very short radiating carinae. Pronotum with a distinct dorsal furrow, dorsally with a well-defined smooth band; central area smooth, but both dorsal and ventral furrows with short parallel carinae. Propleuron with fine punctations throughout and dorsally without a carina. Metasternum flat or nearly so. Contour of mesopleuron straight/angulate or nearly so; precoxal groove indistinct, smooth and shiny; epicnemial ridge elongated more fusiform (tapering at both ends).

**Legs.** Ventral margin of fore telotarsus entire without seta, fore telotarsus almost same width throughout and longer than fourth tarsomere (0.17, 0.09). Hind coxa with dorsal half sparsely punctate, ventral half densely punctate, and dorsal outer depression present. Inner spur of hind tibia longer than outer spur (0.25, 0.20), entire surface of hind tibia with dense strong spines clearly differentiated by color and length. Hind telotarsus longer than fourth tarsomere (0.20, 0.15).

**Wings** (Fig. 82K, L). Fore wing with r vein slightly curved; 2RS vein straight; r and 2RS veins forming an angle at their junction and outer side of junction forming a slight stub; 2M vein slightly curved/swollen; distally fore wing [where spectral veins are] with microtrichiae more densely concentrated than the rest of the wing; anal cell 1/3 proximally lacking microtrichiae; subbasal cell with microtrichiae virtually throughout; veins 2CUa and 2CUb completely spectral; vein 2 cu-a present as spectral vein, sometimes difficult to see; vein 2-1A proximally tubular and distally spectral, although sometimes difficult to see; tubular vein 1 cu-a curved, incomplete/broken and not reaching the edge of 1-1A vein. Hind wing with vannal lobe wide, subdistally and subproximally straightened, and setae present only proximally.

**Metasoma** (Fig. 82A, G, H, J). Metasoma laterally compressed. Petiole on T1 finely sculptured only laterally, parallel-sided in proximal half and then narrow (length 0.45, maximum width 0.25, minimum width 0.15), and with scattered pubescence concentrated in the first distal third. Lateral grooves delimiting the median area on T2 clearly defined and reaching the distal edge of T2 (length median area 0.20, length T2 0.20), edges of median area obscured by weak longitudinal stripes, median area broader than long (length 0.20, maximum width 0.23, minimum width 0.12); T2 with scattered pubescence only distally. T3 longer than T2 (0.23, 0.20) and with scattered pubescence only distally. Pubescence on hypopygium dense.

**Cocoons.** Unknown.

#### Male

(Fig. 83A–F). The coloration on metasoma is darker than in females, but the shape is similar to female.

#### Etymology.

Felipe N. Soto-Adames is a Puerto Rican collembolan systematist. His interests are focused on insect systematics, phylogeny and evolution of Collembola, and evolution of arthropod muscle proteins. Currently, he is curator of Thysanoptera, Collembola, and non-Insect arthropods at the Florida State Collection of Arthropods in Gainesville, Florida, USA.

#### Distribution.

Parasitized caterpillar was collected in Ecuador, Napo, Yanayacu Biological Station (Forest Aguilar), during February 2006 at 2,241 m in cloud forest.

#### Biology.

The lifestyle of this parasitoid species is gregarious.

#### Host.

Memphisnr.lorna (Druce) (Nymphalidae: Charaxinae) feeding on *Nectandra* sp. (Lauraceae). Caterpillar was collected in prepupa.

### Glyptapanteles ferfernandezi

Taxon classificationAnimaliaHymenopteraBraconidae

Arias-Penna, sp. nov.

http://zoobank.org/80E4D2B6-991C-4AC3-A3CC-34338E677CD8

Figs 84 , 85

#### Female.

Body length 3.68 mm, antenna length 3.98 mm, fore wing length 3.88 mm.

#### Type material.

**Holotype**: ECUADOR • 1♀; EC-1932, YY-A077; Napo, Yanayacu Biological Station, Yanayacu Road; cloud forest; 2,100 m; -0.566667, -77.866667; 12.ii.2005; Heidi Connahs leg.; adult parasitoids emerged on 22.iv.2006; (PUCE). **Paratypes.** • 26 (4♀, 5♂) (14♀, 3♂); EC-1932, YY-A077; same data as for holotype; (PUCE).

#### Diagnosis.

**Figure 84.**
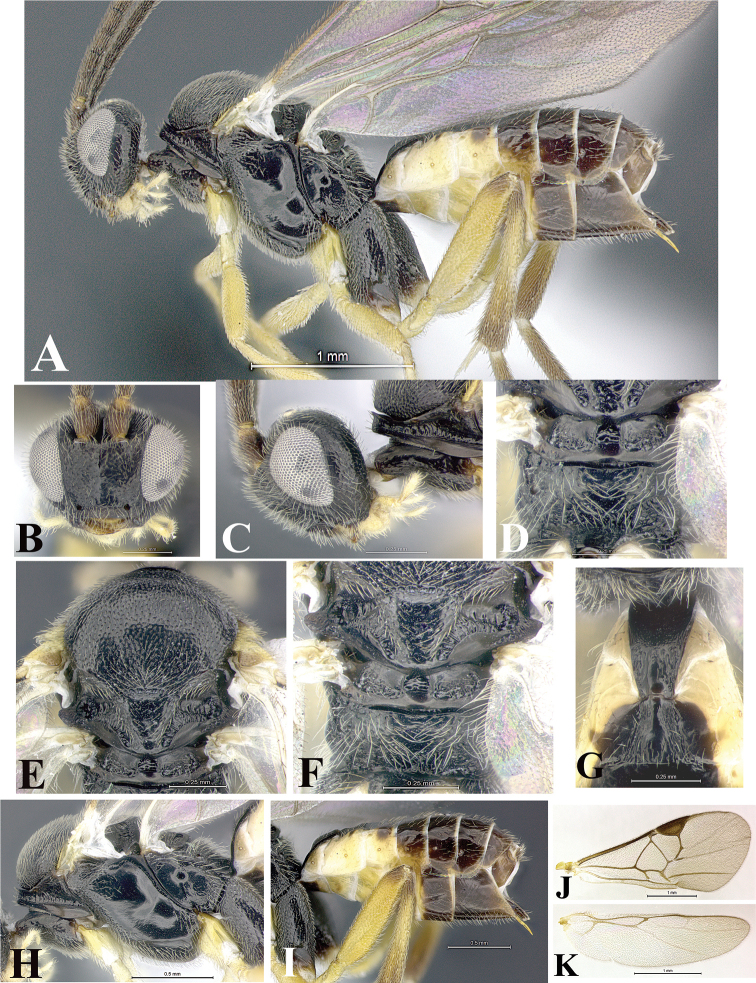
*Glyptapantelesferfernandezi* sp. nov. female EC-1932 YY-A077 **A** Habitus **B** Head, frontal view **C** Head, pronotum, propleuron, lateral view **D** Metanotum, Propodeum, dorsal view **E** Mesonotum, dorsal view **F** Scutellum, metanotum, propodeum, dorsal view **G**T1–2, dorsal view **H** Mesosoma, lateral view **I** Metasoma, lateral view **J, K** Wings **J** Fore **K** Hind.

**Figure 85.**
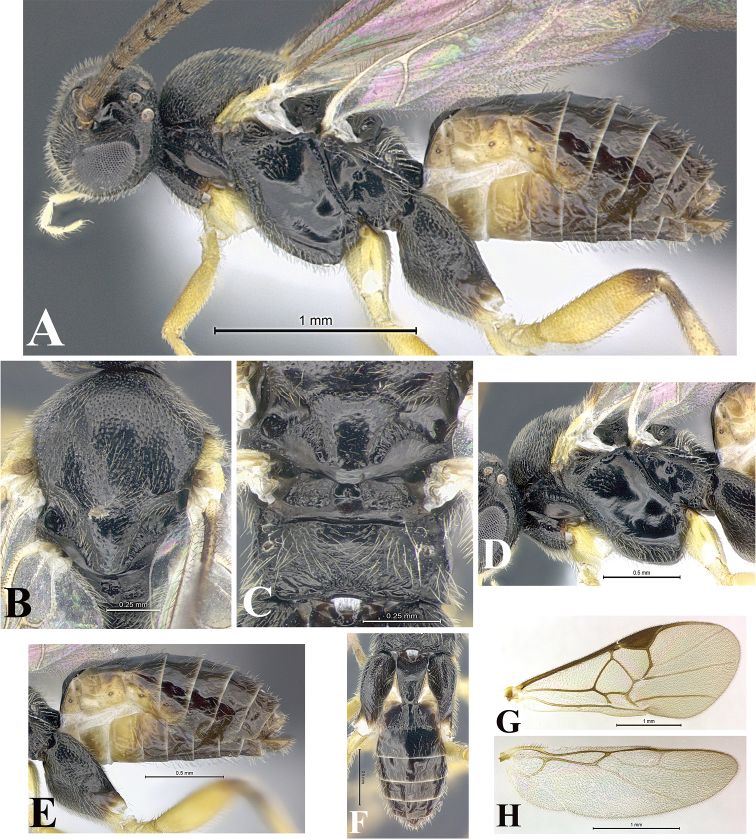
*Glyptapantelesferfernandezi* sp. nov. male EC-1932 YY-A077 **A** Habitus **B** Mesonotum, dorsal view **C** Scutellum, metanotum, propodeum, dorsal view **D** Mesosoma, lateral view **E, F** Metasoma **E** Lateral view **F** Dorsal view **G, H** Wings **G** Fore **H** Hind.

Distal half of propodeum with a mix of coarse sculpture and rugae (Figs 84F, 85C), precoxal groove deep (Figs 84H, 85D), on pronotum central area and dorsal furrow smooth, but ventral furrow with short parallel carinae (Figs 84C, 85D), anterior furrow of metanotum without setiferous lobes (Figs 84F, 85C), petiole on T1 parallel-sided in proximal half, then narrowing (Figs 84G, 85F), edges of median area on T2 obscured by weak longitudinal stripes (Figs 84G, 85F), dorsal outer depression on hind coxa present (Figs 84A, 85A, E), and fore wing with r vein curved, outer side of junction of r and 2RS veins forming a distinct stub (Figs 84J, 85G).

#### Coloration

(Fig. 84A–K). General body coloration polished black except scape with brown tints; all antennal flagellomeres brown in both sides; labrum, mandible and pedicel yellow-brown; glossa, maxillary and labial palps, and tegulae yellow. Eyes and ocelli silver. Fore and middle legs yellow except brown claws and tarsomeres (yellow coloration intensifying from proximal to distal); hind legs yellow except black coxae with yellow apex, a tiny brown dot at the apex of femora, tibiae with apex brown, and tarsomeres brown, although telotarsus with proximal yellow ring. Petiole on T1 black and sublateral areas yellow; T2 with median and adjacent areas (with contours well-defined) brown-black, and lateral ends yellow; T3 with an extended brown area which proximally coincides with width of median and adjacent areas on T2, and proximal corners of lateral ends yellow; T4 and beyond black; distally each tergum with a narrow yellow band. In lateral view, T1–2 completely yellow; T3 yellow, but dorsally brown; T4 and beyond completely black. S1–3 yellow; S4 proximal half yellow, distal half brown; penultimate sternum and hypopygium completely brown.

#### Description.

**Head** (Fig. 84A–C). Head rhomboid with pubescence long and dense. Proximal three antennal flagellomeres longer than wide (0.28:0.08, 0.28:0.08, 0.28:0.08), distal antennal flagellomere longer than penultimate (0.14:0.07, 0.12:0.07), antenna longer than body (3.98, 3.68); antennal scrobes-frons shallow. Distal half of face dented only laterally, with dense fine punctations, interspaces smooth and longitudinal median carina present. Frons smooth. Temple wide, punctate and interspaces clearly smooth. Inner margin of eyes diverging slightly at antennal sockets; in lateral view, eye anteriorly convex and posteriorly straight. POL shorter than OOL (0.12, 0.15). Malar suture present. Median area between lateral ocelli slightly depressed. Vertex laterally rounded and dorsally wide.

**Mesosoma** (Fig. 84A, D–F, H) Mesosoma dorsoventrally convex. Mesoscutum with narrow grooves/dents taking the place of notauli, punctation distinct throughout, interspaces wavy/lacunose. Scutellum shield-shaped, apex sloped and fused with BS, but not in the same plane, scutellar punctation scattered throughout, in profile scutellum flat and on same plane as mesoscutum, phragma of the scutellum completely concealed; BS only very partially overlapping the MPM; ATS demilune entirely covered by parallel carinae; dorsal ATS groove with carinae only proximally. Transscutal articulation with small and heterogeneous foveae, area just behind transscutal articulation depressed centrally and with same kind of sculpture as mesoscutum. Metanotum with BM convex; MPM oval/circular with a short proximal carina; AFM without setiferous lobes and not as well delineated as PFM; PFM thick, smooth and with a proximal flat flange; ATM proximally with semircular/undulate carina and distally smooth. Propodeum without median longitudinal carina, proximal half weakly curved with medium-sized sculpture and distal half with a mix of coarse sculpture and rugae; distal edge of propodeum with a flange at each side and without stubs; propodeal spiracle without distal carina; nucha surrounded by very short radiating carinae. Pronotum with a distinct dorsal furrow, dorsally with a well-defined smooth band; central area of pronotum and dorsal furrow smooth, but ventral furrow with short parallel carinae. Propleuron finely sculptured only ventrally and dorsally without a carina. Metasternum flat or nearly so. Contour of mesopleuron straight/angulate or nearly so; precoxal groove deep, smooth and shiny; epicnemial ridge widen.

**Legs.** Ventral margin of fore telotarsus entire without seta, fore telotarsus proximally narrow and distally wide, and longer than fourth tarsomere (0.11, 0.09). Hind coxa with medium-size punctate throughout, and dorsal outer depression present. Inner spur of hind tibia longer than outer spur (0.31, 0.24), entire surface of hind tibia with dense strong spines clearly differentiated by color and length. Hind telotarsus as equal in length as fourth tarsomere (0.16, 0.16).

**Wings** (Fig. 84J, K). Fore wing with r vein slightly curved; 2RS vein straight; r and 2RS veins forming a weak, even curve at their junction and outer side of junction forming a distinct stub; 2M vein slightly curved/swollen; distally fore wing [where spectral veins are] with microtrichiae more densely concentrated than the rest of the wing; anal cell 1/3 proximally lacking microtrichiae; subbasal cell with microtrichiae virtually throughout; veins 2CUa and 2CUb completely spectral; vein 2 cu-a present as spectral vein, sometimes difficult to see; vein 2-1A proximally tubular and distally spectral, although sometimes difficult to see; tubular vein 1 cu-a straight, incomplete/broken and not reaching the edge of 1-1A vein. Hind wing with vannal lobe very narrow, subdistally and subproximally straightened, and setae present only proximally.

**Metasoma** (Fig. 84A, G, I). Metasoma laterally compressed. Petiole on T1 finely sculptured throughout, parallel-sided in proximal half and then narrowing (length 0.41, maximum width 0.22, minimum width 0.12), and with scattered pubescence concentrated in the first distal third. Lateral grooves delimiting the median area on T2 clearly defined and reaching the distal edge of T2 (length median area 0.21, length T2 0.21), edges of median area obscured by weak longitudinal stripes, median area broader than long (length 0.21, maximum width 0.27, minimum width 0.12); T2 with scattered pubescence only distally. T3 longer than T2 (0.29, 0.21) and with scattered pubescence only distally. Pubescence on hypopygium dense.

**Cocoons.** Unknown.

#### Comments.

The area replacing the notauli with a depression.

#### Male

(Fig. 85A–H). The body coloration is darker than females. The males are stouter than females.

#### Etymology.

Fernando (Fer) Fernández is a Colombian entomologist; his work is focused on taxonomy and systematics of Hymenoptera, mainly Formicidae. He is a professor at the Universidad Nacional de Colombia, Bogotá, Colombia.

#### Distribution.

Parasitized caterpillars were collected in Ecuador, Napo, Yanayacu Biological Station (Yanayacu Road), during February 2006 at 2,100 m in cloud forest.

#### Biology.

The lifestyle of this parasitoid species is gregarious.

#### Host.

Memphisnr.lorna (Druce) (Nymphalidae: Charaxinae) feeding on *Nectandra* sp. (Lauraceae). Caterpillar instar was not reported.

### Glyptapanteles garygibsoni

Taxon classificationAnimaliaHymenopteraBraconidae

Arias-Penna, sp. nov.

http://zoobank.org/1C217DC8-0F36-469F-9B79-AC15DEBC8734

Figs 86 , 87

#### Female.

Body length 2.22 mm, antenna length 2.53 mm, fore wing length 2.42 mm.

#### Type material.

**Holotype**: COSTA RICA • 1♀; 91-SRNP-1820, DHJPAR0000063; Área de Conservación Guanacaste, Guanacaste, Sector Santa Rosa, Cafetal; 280 m; 10.85827, -85.61089; 16.vii.1991; gusaneros leg.; separate, light brown cocoons on back of caterpillar and formed on 25.vii.1991; adult parasitoids emerged on 01.viii.1991; (CNC). **Paratypes.** • 60 (2♀, 3♂) (44♀, 11♂); 91-SRNP-1820, DHJPAR0000063; same data as for holotype; (CNC).

#### Other material.

**Reared material.** COSTA RICA: *Área de Conservación Guanacaste*, *Guanacaste*, *Sector Santa Rosa*, *Área Administrativa*: • 57 (3♀, 3♂) (43♀, 8♂); 82-SRNP-418, DHJPAR0000052; dry forest; 295 m; 10.83764, -85.61871; 01.vii.1982; DH Janzen leg.; caterpillar collected in fourth instar, found with the cocoons already out of the caterpillar; cocoons adhered to the leaf substrate; adult parasitoids emerged on 03.vii.1982 and caterpillar was still alive when the wasps eclosed.

*Área de Conservación Guanacaste*, *Guanacaste*, *Sector Santa Rosa*, *Cafetal*: • 13 (3♀, 3♂) (15♀, 2♂); 91-SRNP-1814, DHJPAR0000061; 280 m; 10.85827, -85.61089; 16.vii.1991; gusaneros leg.; caterpillar collected in fourth instar; cocoons adhered to the leaf substrate and formed on 25.vii.1991; adult parasitoids emerged on 01.viii.1991. • 8 (2♀, 2♂) (4♀, 0♂); 91-SRNP-1816, DHJPAR0000062; same data as for preceding except: hard dorsal cocoons adhered to the larval cuticle; adult parasitoids emerged on 24.viii.1991.

*Área de Conservación Guanacaste*, *Guanacaste*, *Sector El Hacha*, *Sendero Bejuquilla*: • 3 (1♀, 1♂) (0♀, 1♂); 98-SRNP-5332, DHJPAR0000113; intergrade dry-rain forest; 280 m; 11.03004, -85.52699; 03.vii.1998; Roster Moraga leg.; caterpillar collected in fourth instar; small white-gray somewhat separate cocoon adhered to the leaf substrate and formed on 11.vii.1998; adult parasitoids emerged on 21.vii.1998.

#### Diagnosis.

**Figure 86.**
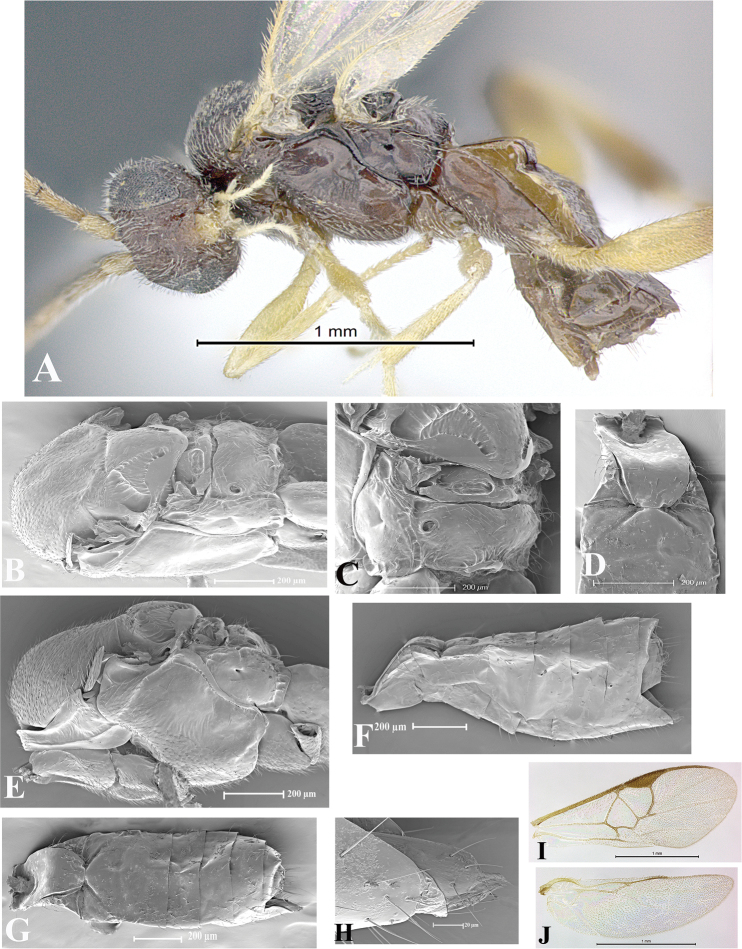
*Glyptapantelesgarygibsoni* sp. nov. female 91-SRNP-1820 DHJPAR0000063 **A** Habitus **B, E** Mesosoma **B** Dorsolateral view **E** Lateral view **C** Metanotum, propodeum, dorsolateral view **D**T1–2, dorsal view **F, G** Metasoma **F** lateral view **G** Dorsal view **H** Genitalia: hypopygium, ovipositor, ovipositor sheaths, lateral view **I, J** Wings **I** Fore **J** Hind.

**Figure 87.**
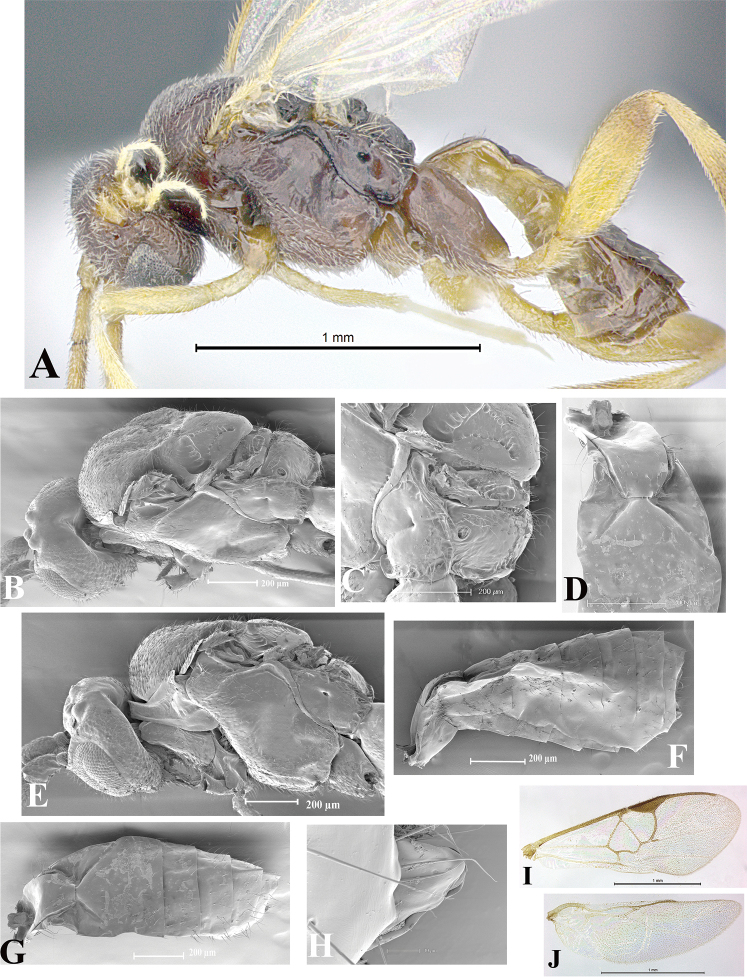
*Glyptapantelesgarygibsoni* sp. nov. male 91-SRNP-1820 DHJPAR0000063 **A** Habitus **B, E** Head, mesosoma **B** Dorsolateral view **E** lateral view **C** Metanotum, propodeum, laterodorsal view **D**T1–3, dorsal view **F, G** Metasoma **F** lateral view **G** Dorsal view **H** Genitalia: parameres, lateral view **I, J** Wings **I** Fore **J** Hind.

Phragma of the scutellum partially exposed (Figs 86B, C, 87B, C), longitudinal median carina on face absent, inner margin of eyes straight throughout, scutellar punctation scattered throughout (Figs 86B, C, 87B, C), petiole on T1 distally with lateral margins curved (convex, Figs 86D, G, 87D, G), propodeal spiracle without distal carina (Figs 86B, C, 87B, C), nucha surrounded by very short radiating carinae (Figs 86B, C, 87B, C), propodeum without median longitudinal carina (Figs 86B, C, 87B, C), antenna longer than body, fore wing with 2RS vein straight, outer side of junction of r and 2RS veins not forming a stub (Figs 86I, 87I), and lateral grooves delimiting the median area on T2 distally losing definition (Figs 86D, G, 87D, G).

#### Coloration

(Fig. 86A). General body coloration light brown except labrum, mandibles, glossa, maxillary and labial palps, scape, pedicel, and tegulae yellow; three-four most proximal antennal flagellomeres dorsally lighter (light brown) than ventrally (dark brown), following flagellomeres dark brown on both sides. Eyes gray and ocelli silver. Fore and middle legs yellow except coxae and claws brown; hind legs yellow except coxae, apex of femora, distal 3/4 of tibiae and tarsomeres brown. Petiole on T1 yellow-brown/light brown, contours darkened and sublateral areas yellow-brown; T2 with median and wide adjacent areas light brown, and lateral ends yellow-brown; T3 and beyond light brown; distally each tergum with a very narrow transparent band. In lateral view, T1–3 and S1–3 completely yellow-brown remaining terga and sterna brown.

#### Description.

**Head** (Fig. 86A). Head rounded with pubescence short and dense. Proximal three antennal flagellomeres longer than wide (0.16:0.05, 0.18:0.05, 0.18:0.05), distal antennal flagellomere longer than penultimate (0.10:0.05, 0.08:0.05), antenna longer than body (2.53, 2.22); antennal scrobes-frons shallow. Face flat or nearly so, with dense fine punctations, interspaces with microsculpture and longitudinal median carina absent. Frons smooth. Temple wide, punctate and interspaces with microsculpture. Inner margin of eyes straight throughout; in lateral view, eye anteriorly convex and posteriorly straight. POL shorter than OOL (0.08, 0.10). Malar suture present. Median area between lateral ocelli without depression. Vertex laterally pointed or nearly so and dorsally wide.

**Mesosoma** (Fig. 86A–C, E). Mesosoma dorsoventrally convex. Distal 1/3 of mesoscutum with lateral margin slightly dented, punctation distinct throughout, interspaces with microsculpture. Scutellum triangular, apex sloped and fused with BS, scutellar punctation scattered throughout, in profile scutellum flat and on same plane as mesoscutum, phragma of the scutellum partially exposed; BS only very partially overlapping the MPM; ATS demilune with short stubs delineating the area and inner side with a row of foveae; dorsal ATS groove with semicircular/parallel carinae. Transscutal articulation with small and heterogeneous foveae, area just behind transscutal articulation smooth, shiny and depressed centrally. Metanotum with BM wider than PFM (clearly differentiated); MPM circular without median longitudinal carina; AFM without setiferous lobes and not as well delineated as PFM; PFM thick and smooth; ATM proximally with semircular/undulate carina and distally smooth. Propodeum without median longitudinal carina, proximal half curved and relatively polished and distal half relatively polished; distal edge of propodeum with a flange at each side and without stubs; propodeal spiracle without distal carina; nucha surrounded by very short radiating carinae. Pronotum with a distinct dorsal furrow, dorsally with a well-defined smooth band; central area of pronotum smooth, but both dorsal and ventral furrows with short parallel carinae. Propleuron finely sculptured only ventrally and dorsally with a carina. Metasternum flat or nearly so. Contour of mesopleuron straight/angulate or nearly so; precoxal groove shallow, but visible and with faintly transverse lineate sculpture; epicnemial ridge convex, teardrop-shaped.

**Legs.** Ventral margin of fore telotarsus entire, but with a tiny curved seta, fore telotarsus almost same width throughout and longer than fourth tarsomere (0.09, 0.06). Hind coxa with punctation only on ventral surface and dorsal outer depression present. Inner spur of hind tibia longer than outer spur (0.23, 0.15), entire surface of hind tibia with dense strong spines clearly differentiated by color and length. Hind telotarsus as equal in length as fourth tarsomere (0.10, 0.10).

**Wings** (Fig. 86I, J). Fore wing with r vein curved, 2RS vein straight; r and 2RS veins forming a weak, even curve at their junction and outer side of junction not forming a stub; 2M vein straight; distally fore wing [where spectral veins are] with microtrichiae more densely concentrated than the rest of the wing; anal cell 1/3 proximally lacking microtrichiae; subbasal cell with a small smooth area, vein 2CUa absent and vein 2CUb spectral; vein 2 cu-a absent; vein 2-1A present only proximally as tubular vein; tubular vein 1 cu-a curved, incomplete/broken and not reaching the edge of 1-1A vein. Hind wing with vannal lobe wide, subdistally straightened and subproximally straightened, and setae present proximally, but absent distally.

**Metasoma** (Fig. 86A, D, F–H). Metasoma laterally compressed. Petiole on T1 completely smooth and polished, with faint, satin-like sheen, virtually parallel-sided over most of length, but narrowing over distal 1/3, apex truncate (length 0.32, maximum width 0.19, minimum width 0.11), and with scattered pubescence concentrated in the first distal third. Lateral grooves delimiting the median area on T2 distally losing definition (length median area 0.09, length T2 0.14), edges of median area polished and lateral grooves deep, median area broader than long (length 0.09, maximum width 0.20, minimum width 0.04); T2 with scattered pubescence only distally. T3 longer than T2 (0.20, 0.14) and with scattered pubescence throughout. Pubescence on hypopygium dense.

**Cocoons** (Fig. 4P). Light brown or gray oval cocoons with ordered silk fibers, but covered by a net. Cocoons on back of caterpillar or attached to the leaf substrate.

#### Comments.

This species looks like *Distatrix*, the lateral grooves delimiting the median area on T2 are far from the proximal edge of T3.

#### Male

(Fig. 87A–J). The body coloration and the body shape similar to female.

#### Etymology.

Gary A. P. Gibson is a research scientist at Agriculture and Agri-Food Canada, Ottawa, Ontario, Canada. His expertise is focused upon systematics of chalcid parasitoid wasps (Chalcidoidea), especially the families Eupelmidae and Pteromalidae and functional and comparative morphology of Chalcidoidea and Hymenoptera.

#### Distribution.

Parasitized caterpillars were collected in Costa Rica, ACG, Sector El Hacha (Sendero Bejuquilla) and Sector Santa Rosa (Área Administrativa and Cafetal), during July of 1982, 1991, and 1998 at 280 m and 295 m in coffee plantations, dry forest, and intergrade dry-rain forest.

#### Biology.

The lifestyle of this parasitoid species is gregarious.

#### Host.

*Nystaleacollaris* Schaus (Fig. 4P) (Noctuidae: Nystaleinae) feeding on *Psidiumguineense* and *Eugeniasalamensis* (Myrtaceae) and *N.guzmani* Schaus feeding on *Calyptrantheschytraculia* (Myrtaceae). Caterpillar were collected in fourth instar and cocoons were already out of the caterpillar.

### Glyptapanteles gavinbroadi

Taxon classificationAnimaliaHymenopteraBraconidae

Arias-Penna, sp. nov.

http://zoobank.org/96B6F473-5BA2-43A5-8DE3-0F98D1BBF947

Figs 88 , 89

#### Female.

Body length 2.53 mm, antenna length 2.63 mm, fore wing length 2.42 mm.

#### Type material.

**Holotype**: COSTA RICA • 1♀; 95-SRNP-8935, DHJPAR0000092; Área de Conservación Guanacaste, Guanacaste, Sector Santa Rosa, Área administrativa; dry forest; 295 m; 10.83764, -85.61871; 05.ix.1995; gusaneros leg.; caterpillar collected in fifth instar; cocoons in two rows of white cordwood stack on each side of caterpillar and adhered to the leaf substrate; adult parasitoids emerged on 17.ix.1995; (CNC). **Paratypes.** • 32 (5♀, 6♂) (21♀, 0♂); 95-SRNP-8935, DHJPAR0000092; same data as for holotype; (CNC).

#### Diagnosis.

**Figure 88.**
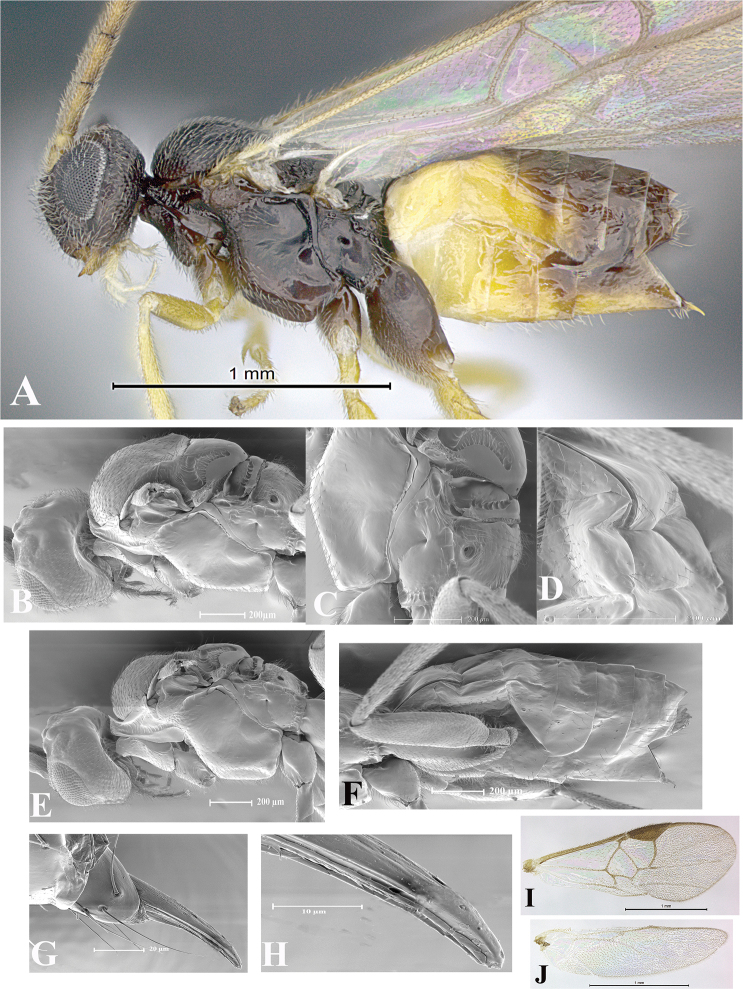
*Glyptapantelesgavinbroadi* sp. nov. female 95-SRNP-8935 DHJPAR0000092 **A** Habitus **B, E** Head, mesosoma **B** Laterodorsal view **E** lateral view **C** Metanotum, propodeum, laterodorsal view **D**T1–2, laterodorsal view **F** Metasoma, lateral view **G, H** Genitalia **G** Hypopygium, ovipositor, ovipositor sheaths, lateral view **H** Ovipositor detail **I, J** Wings **I** Fore **J** Hind.

**Figure 89.**
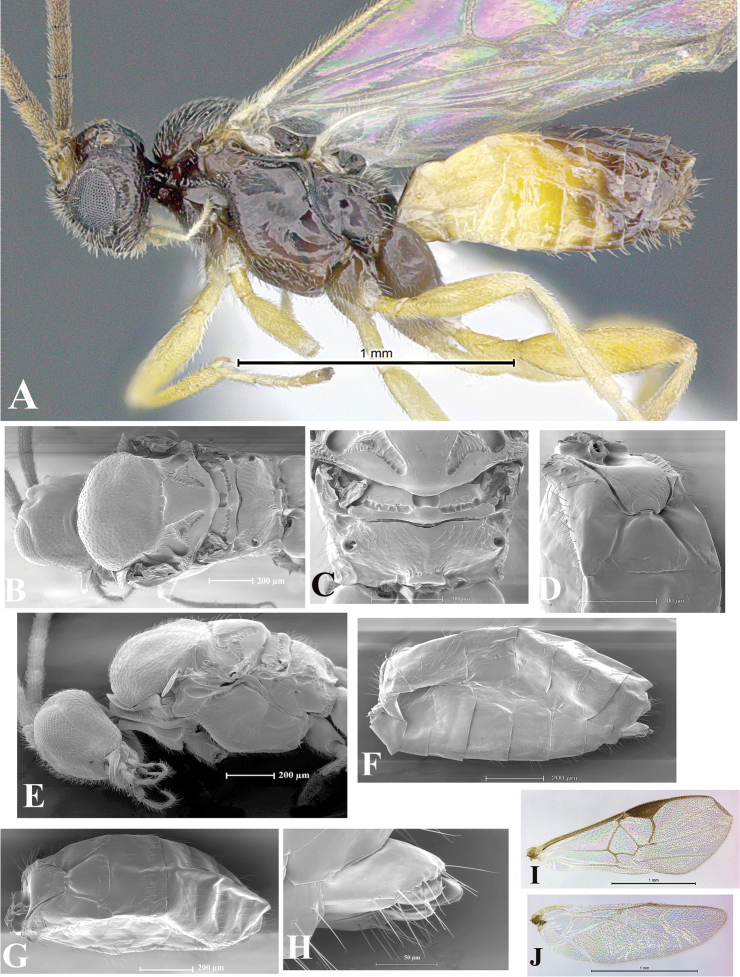
*Glyptapantelesgavinbroadi* sp. nov. male 95-SRNP-8935 DHJPAR0000092 **A** Habitus **B, E** Head, mesosoma **B** Dorsal view **E** lateral view **C** Metanotum, propodeum, dorsal view **D**T1–2, dorsal view **F, G** Metasoma **F** lateral view **G** Dorsolateral view **H** Genitalia: parameres, lateral view **I, J** Wings **I** Fore **J** Hind.

Scutellar punctation indistinct throughout (Fig. 89B, C), distal antennal flagellomere longer than penultimate, inner margin of eyes straight throughout, phragma of the scutellum completely concealed (Figs 88B, C, 89B, C), fore wing with vein 2-1A absent, 2RS vein straight, outer side of junction of r and 2RS veins not forming a stub (Figs 88I, 89I), propleuron with fine rugae (Figs 88A, B, E, 89A, E), mesoscutum punctate throughout (Fig. 89B), anteroventral contour of mesopleuron straight/angulate or nearly so (Figs 88A, B, E, 89A, E), petiole on T1 distally with lateral margins relatively straight (Figs 88D, F, 89D, G), propodeum without median longitudinal carina, propodeal spiracle without distal carina (Figs 88B, C, 89B, C), nucha surrounded by very short radiating carinae (Figs 88B, C, 89B, C), antenna longer than body, and lateral grooves delimiting the median area on T2 distally losing definition (Figs 88D, F, 89D, G).

#### Coloration

(Fig. 88A). General body coloration dark brown except labrum, mandibles, scape, and pedicel yellow-brown; glossa, maxillary and labial palps, and tegulae yellow; three most proximal antennal flagellomeres dorsally lighter (light brown) than ventrally (dark brown), following flagellomeres dark brown on both sides. Eyes gray/black and ocelli reddish. Fore and middle legs yellow except brown coxae (inner side yellow-brown) and fore claws brown; hind legs yellow-brown except coxae and distal 3/4 of tibiae brown, and tarsomeres brown except proximal 1/3 of basitarsus yellow-brown. Petiole on T1 brown, contour darkened and sublateral areas yellow; T2 with median area dark, adjacent area and lateral ends yellow; T3 medially with a inverted triangular brown area and remaining area yellow-brown; T4 and beyond brown; distally each tergum with a narrow whitish transparent band. In lateral view, T1–2 completely yellow; T3 yellow, dorsally brown; T4 and beyond completely brown. S1–4 completely yellow; penultimate sternum and hypopygium brown, medially yellow.

#### Description.

**Head** (Fig. 88A, B, E). Head triangular with pubescence short and dense. Proximal three antennal flagellomeres longer than wide (0.22:0.06, 0.20:0.06, 0.18:0.06), distal antennal flagellomere longer than penultimate (0.11:0.05, 0.09:0.05), antenna longer than body (2.63, 2.53); antennal scrobes-frons shallow. Face convex, with dense fine punctations, interspaces smooth and longitudinal median carina present. Frons smooth. Temple wide, punctate and interspaces wavy. Inner margin of eyes straight throughout; in lateral view, eye anteriorly convex and posteriorly straight. POL shorter than OOL (0.09, 0.11). Malar suture absent or difficult to see. Median area between lateral ocelli slightly depressed. Vertex laterally rounded and dorsally wide.

**Mesosoma** (Fig. 88A–C, E). Mesosoma dorsoventrally convex. Mesoscutum with narrow grooves laterally, punctation distinct throughout, interspaces with microsculpture. Scutellum triangular, apex sloped and fused with BS, scutellar punctation indistinct throughout, in profile scutellum flat and on same plane as mesoscutum, phragma of the scutellum completely concealed; BS only very partially overlapping the MPM; ATS demilune with complete undulate/reticulate carinae; dorsal ATS groove smooth. Transscutal articulation with small and heterogeneous foveae, area just behind transscutal articulation with a smooth and shiny sloped transverse strip. Metanotum with BM wider than PFM (clearly differentiated); MPM circular without median longitudinal carina; AFM without setiferous lobes and not as well delineated as PFM; PFM thick, smooth and with lateral ends rounded; ATM proximally with a well-defined row of foveae and distally smooth. Propodeum without median longitudinal carina, proximal half curved with medium-sized sculpture and distal half relatively polished; distal edge of propodeum without flange; propodeal spiracle without distal carina; nucha surrounded by very short radiating carinae. Pronotum with a distinct dorsal furrow, dorsally with a well-defined smooth band; central area of pronotum smooth, but both furrows dorsal and ventral with short parallel carinae. Propleuron with fine rugae and dorsally with a carina. Metasternum flat or nearly so. Contour of mesopleuron straight/angulate or nearly so; precoxal groove deep with faintly lineate sculpture; epicnemial ridge convex, teardrop-shaped.

**Legs.** Ventral margin of fore telotarsus entire, but with a tiny curved seta, fore telotarsus almost same width throughout and longer than fourth tarsomere (0.12, 0.07). Hind coxa with punctation only on ventral surface and dorsal outer depression present. Inner spur of hind tibia longer than outer spur (0.20, 015), entire surface of hind tibia with dense strong spines clearly differentiated by color and length. Hind telotarsus longer than fourth tarsomere (0.15, 0.10).

**Wings** (Fig. 88I, J). Fore wing with r vein slightly curved; 2RS vein straight; r and 2RS veins forming a weak, even curve at their junction and outer side of junction not forming a stub; 2M vein slightly curved/swollen; distally fore wing [where spectral veins are] with microtrichiae more densely concentrated than the rest of the wing; anal cell 1/3 proximally lacking microtrichiae; subbasal cell with a small smooth area; vein 2CUa absent and vein 2CUb spectral; vein 2 cu-a absent; vein 2-1A absent; tubular vein 1 cu-a straight, incomplete/broken and not reaching the edge of 1-1A vein. Hind wing with vannal lobe wide, subdistally straightened, subproximally straightened, and setae present only proximally.

**Metasoma** (Fig. 88A, D, F–H). Metasoma laterally compressed. Petiole on T1 completely smooth and polished, with faint, satin-like sheen, virtually parallel-sided over most of length, but narrowing over distal 1/3 (length 0.32, maximum width 0.17, minimum width 0.09), and with scattered pubescence concentrated in the first distal third. Lateral grooves delimiting the median area on T2 distally losing definition (length median area 0.11, length T2 0.14), edges of median area polished and lateral grooves deep, median area broader than long (length 0.11, maximum width 0.20, minimum width 0.08); T2 with scattered pubescence only distally. T3 longer than T2 (0.21, 0.14) and with scattered pubescence only distally. Pubescence on hypopygium dense.

**Cocoons.** White oval cocoons with evenly smooth silk fibers. Cocoons arranged in two rows of cordwood stack on each side of caterpillar and adhered to the leaf substrate.

#### Comments.

This species shares a character with *Distatrix*, that the lateral grooves delimiting the median area on T2 do not reach the proximal part of T3.

#### Male

(Fig. 89A–J). Similar in coloration and shape to female.

#### Etymology.

Gavin R. Broad is Senior Curator in the Entomology Department at the Natural History Museum, London, UK, and an expert on Ichneumonoidea and Vespoidea (except ants).

#### Distribution.

Parasitized caterpillar was collected in Costa Rica, ACG, Sector Santa Rosa (Área administrativa), during September 1995 at 295 m in dry forest.

#### Biology.

The lifestyle of this parasitoid species is gregarious.

#### Host.

*Pararcteschneideriana* Stoll (Noctuidae: Catocalinae) feeding on *Cecropiapeltata* (Urticaceae). Caterpillar was collected in fifth instar.

### Glyptapanteles genorodriguezae

Taxon classificationAnimaliaHymenopteraBraconidae

Arias-Penna, sp. nov.

http://zoobank.org/F05CF281-3EF8-4528-865B-87203EEE27C9

Figs 90 , 91

#### Female.

Body length 3.38 mm, antenna length 3.78 mm, fore wing length 3.38 mm.

#### Type material.

**Holotype**: ECUADOR • 1♀; EC-1933, YY-A075; Napo, Yanayacu Biological Station, Yanayacu Road; cloud forest; 2,100 m; -0.566667, -77.866667; 12.ii.2005; Heidi Connahs leg.; cocoons formed on 15.iv.2005; adult parasitoids emerged on 24.iv.2005; (PUCE). **Paratypes.** • 13 (4♀, 2♂) (7♀, 0♂); EC-1933, YY-A075; same data as for holotype; (PUCE).

#### Diagnosis.

**Figure 90.**
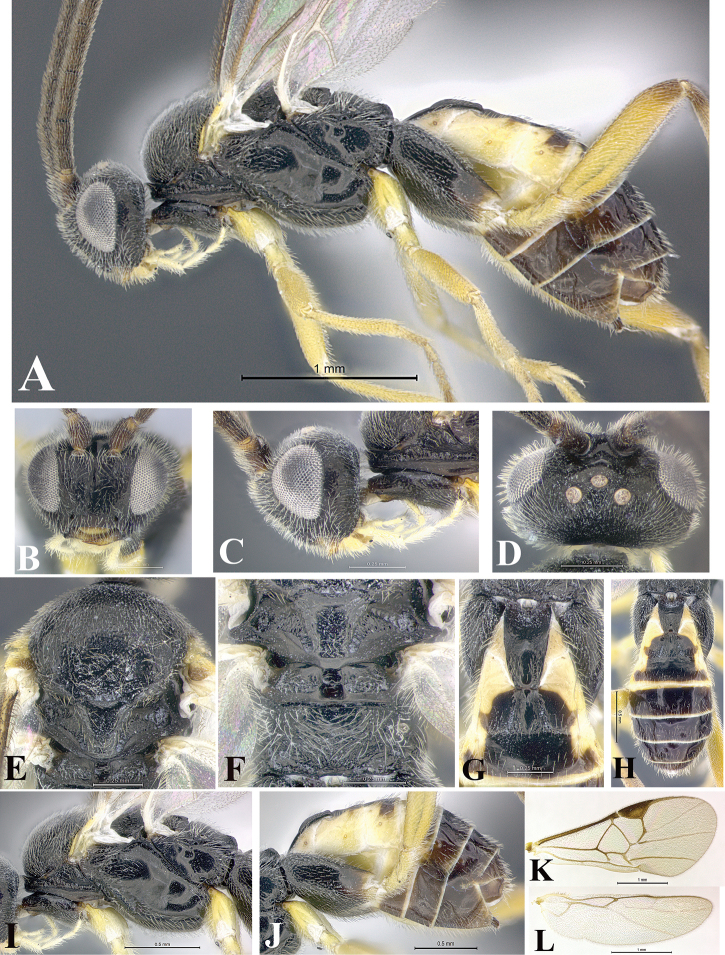
*Glyptapantelesgenorodriguezae* sp. nov. female EC-1933 YY-A075 **A** Habitus **B, D** Head **B** Frontal view **D** Dorsal view **C** Head, pronotum, propleuron, lateral view **E** Mesonotum, dorsal view **F** Scutellum, metanotum, propodeum, dorsal view **G**T1–3, dorsal view **H, J** Metasoma **H** Dorsal view **J** Lateral view **I** Mesosoma, lateral view **K, L** Wings **K** Fore **L** Hind.

**Figure 91.**
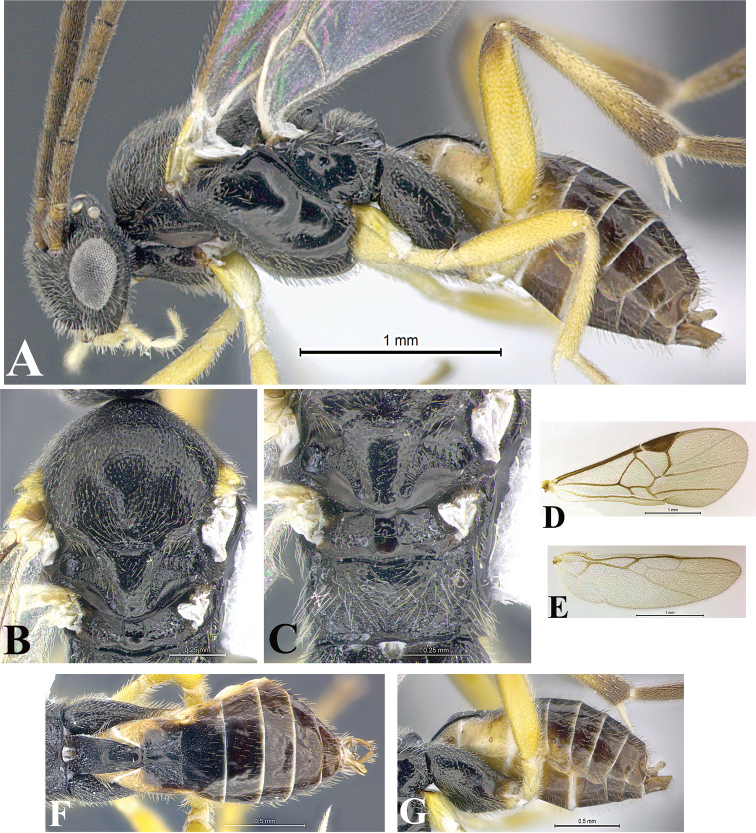
*Glyptapantelesgenorodriguezae* sp. nov. male EC-1933 YY-A075 **A** Habitus **B** Mesonotum, dorsal view **C** Scutellum, metanotum, propodeum, dorsal view **D, E** Wings **D** Fore **E** Hind **F, G** Metasoma **F** Dorsal view **G** Lateral view.

Vertex in lateral view rounded (Fig. 90C), frons punctate, scutellar punctation scattered throughout (Figs 90E, 91B), in lateral view, metasoma curved (Figs 90A, 91A), median area on T2 as broad as long (Figs 90G, 91F) and lateral grooves delimiting the median area on T2 distally losing definition on T2 (Fig. 90G, H), petiole on T1 parallel-sided in proximal half, then narrowing (Fig. 90G) and finely sculptured (Figs 90G, 91F), propodeum without a median longitudinal dent (Figs 90F, 91C), and fore wing with r vein straight, outer side of junction of r and 2RS veins forming a stub (Figs 90K, 91D).

#### Coloration

(Fig. 90A–L). General body coloration polished black except scape with brown tints; all antennal flagellomeres brown on both sides; labrum, mandible and pedicel yellow-brown; glossa, maxillary and labial palps, and tegulae yellow. Fore and middle legs yellow except brown claws, and tarsomeres 3–5 coloration intensity increasing from proximal (yellow) to distal (light brown); hind legs yellow except black coxae with apex yellow, femora with a tiny brown dot at the apex, tibiae with both ends brown, tarsomeres brown, although basitarsus with a proximal yellow ring. Petiole on T1 black and sublateral areas yellow; T2 with median and adjacent areas black, adjacent area with contours well-defined and with two unevenly shaped blotches, and lateral ends yellow; T3 with a extensive brown area which proximally with the same width of median and adjacent areas on T2, but distally it extents along the width of T3, and proximal half of corners yellow; T4 and beyond completely black; distally each tergum with a narrow yellowish transparent band. In lateral view, T1–3 completely yellow; T4 yellow, but dorsally brown; T5 and beyond completely black. S1–3 completely yellow; S4 proximal half yellow, distal half brown; penultimate sternum and hypopygium completely brown.

#### Description.

**Head** (Fig. 90A–D). Head rhomboid and pubescence long and dense. Proximal three antennal flagellomeres longer than wide (0.28:0.07, 0.31:0.07, 0.29:0.07), distal antennal flagellomere longer than penultimate (0.16:0.06, 0.12:0.06), antenna longer than body (3.78, 3.38); antennal scrobes-frons shallow. Face flat or nearly so, with dense fine punctations, interspaces smooth and longitudinal median carina present. Frons punctate. Temple narrow, punctate and interspaces clearly smooth. Inner margin of eyes diverging slightly at antennal sockets; in lateral view, eye anteriorly convex and posteriorly straight. POL shorter than OOL (0.12, 0.14). Malar suture present. Median area between lateral ocelli slightly depressed. Vertex laterally rounded and dorsally wide.

**Mesosoma** (Fig. 90A, E, F, I). Mesosoma dorsoventrally convex. Mesoscutum proximally convex and distally flat, punctation distinct throughout and interspaces wavy/lacunose. Scutellum long and slender, apex sloped and fused with BS, scutellar punctation scattered throughout, in profile scutellum flat and on same plane as mesoscutum, phragma of the scutellum partially exposed; BS only very partially overlapping the MPM; ATS demilune inner side with a row of foveae; dorsal ATS groove with semicircular/parallel carinae. Transscutal articulation with small and heterogeneous foveae, area just behind transscutal articulation smooth, shiny and nearly at the same level as mesoscutum (flat). Metanotum with BM wider than PFM (clearly differentiated); MPM semicircular without median longitudinal carina; AFM without setiferous lobes and not as well delineated as PFM; PFM thick, smooth and with lateral ends rounded; ATM proximally with a groove with some sculpturing and distally smooth. Propodeum without median longitudinal carina, proximal half weakly curved with medium-sized sculpture and distal half with a shallow dent at each side of nucha and slightly rugose; distal edge of propodeum with a flange at each side and without stubs; propodeal spiracle without distal carina; nucha surrounded by very short radiating carinae. Pronotum with a distinct dorsal furrow, dorsally with a well-defined smooth band; central area of pronotum smooth, but both dorsal and ventral furrows with short parallel carinae. Propleuron with fine punctations throughout and dorsally without a carina. Metasternum convex. Contour of mesopleuron straight/angulate or nearly so; precoxal groove smooth, shiny and shallow, but visible; epicnemial ridge elongated more fusiform (tapering at both ends).

**Legs.** Ventral margin of fore telotarsus entire and without seta, fore telotarsus almost same width throughout, and longer than fourth tarsomere (0.15, 0.10). Dorsal half of hind coxa with scattered punctation and ventral half with dense punctation and dorsal outer depression present. Inner spur of hind tibia longer than outer spur (0.31, 0.24), entire surface of hind tibia with dense strong spines clearly differentiated by color and length. Hind telotarsus as equal in length as fourth tarsomere (0.17, 0.16).

**Wings** (Fig. 90K, L). Fore wing with r vein straight; 2RS vein straight; r and 2RS veins forming a weak, even curve at their junction and outer side of junction forming a slight stub; 2M vein slightly curved/swollen; distally fore wing [where spectral veins are] with microtrichiae more densely concentrated than the rest of the wing; anal cell 1/3 proximally lacking microtrichiae; subbasal cell with microtrichiae virtually throughout; veins 2CUa and 2CUb completely spectral; vein 2 cu-a present as spectral vein, sometimes difficult to see; vein 2-1A proximally tubular and distally spectral, although sometimes difficult to see; tubular vein 1 cu-a straight, incomplete/broken and not reaching the edge of 1-1A vein. Hind wing with vannal lobe very narrow, subdistally and subproximally straightened, and setae evenly scattered in the margin.

**Metasoma** (Fig. 90A, G, H, J). Metasoma curved. Petiole on T1 finely sculptured completely parallel-sided in proximal half and then narrowing (length 0.40, maximum width 0.19, minimum width 0.12) and with scattered pubescence concentrated in the first distal third. Lateral grooves delimiting the median area on T2 clearly defined and reaching the distal edge of T2 (length median area 0.16, length T2 0.16), edges of median area obscured by weak longitudinal stripes, median area as broad as long (length 0.16, maximum width 0.16, minimum width 0.12); T2 with scattered pubescence only distally. T3 longer than T2 (0.20, 0.16) and with scattered pubescence only distally. Pubescence on hypopygium dense.

**Cocoons.** Unknown.

#### Comments.

In both sexes the body is curved.

#### Male

(Fig. 91A–G). Coloration and shape similar to females.

#### Etymology.

Genoveva (Geno) Rodriguez Castañeda is a Guatemalan biologist. Her research interests are centered on how biotic interactions (herbivory and predation) change across climatic gradients and what causes rates of speciation and ranges of species distributions to vary along environmental gradients. She works at Beta Hatch, Insect Entrepeneurs, Seattle, WA, USA.

#### Distribution.

Parasitized caterpillar was collected in Ecuador, Napo, Yanayacu Biological Station (Yanayacu Road), during February 2005 at 2,100 m in cloud forest.

#### Biology.

The lifestyle of this parasitoid species is gregarious.

#### Host.

Memphisnr.lorna (Druce) (Nymphalidae: Charaxinae) feeding on *Nectandra* sp. (Lauraceae). Caterpillar instar was not reported.

### Glyptapanteles gerarddelvarei

Taxon classificationAnimaliaHymenopteraBraconidae

Arias-Penna, sp. nov.

http://zoobank.org/EC41FA76-95D5-46A9-9806-1EAD968F03AC

Figs 92 , 93

#### Female.

Body length 2.53 mm, antenna length 2.97 mm, fore wing length 2.77 mm.

#### Type material.

**Holotype**: COSTA RICA • 1♀; 04-SRNP-34445, DHJPAR0000280; Área de Conservación Guanacaste, Guanacaste, Sector Pitilla, Loaiciga; rain forest; 445 m; 11.01983, -85.41342; 11.viii.2004; Calixto Moraga leg.; caterpillar collected in fourth instar; beige cocoons scattered under the cadaver with the cadaver adhered on top, cocoons formed on 27.viii.2004; adult parasitoids emerged on 04.ix.2004; (CNC). **Paratypes.** • 33 (2♀, 3♂) (26♀, 2♂); 04-SRNP-34445, DHJPAR0000280; same data as for holotype; (CNC).

#### Other material.

**Reared material.** COSTA RICA: *Área de Conservación Guanacaste*, *Alajuela*, *Sector Rincón Rain Forest*, *Camino Río Francia*: • 25 (3♀, 1♂) (21♀, 0♂); 03-SRNP-11431, DHJPAR0001481; 410 m; 10.90425, -85.28651; 24.vi.2003; Minor Carmona leg.; caterpillar collected in fourth instar; brown cocoons tacked lightly together in a jumbled group, adhered to the leaf substrate and formed on 01.vii.2003; adult parasitoids emerged on 11.vii.2003.

*Área de Conservación Guanacaste*, *Alajuela*, *Sector Rincón Rain Forest*, *Finca Hugo*: • 8 (1♀, 1♂) (6♀, 0♂); 04-SRNP-42148, DHJPAR0001457; 540 m; 10.88068, -85.26968; 17.viii.2004; José Pérez leg.; caterpillar collected in fifth instar; brown cocoons adhered to the leaf substrate formed on 21.viii.2004; adult parasitoids emerged on 30.viii.2004. • 27 (3♀, 3♂) (19♀, 2♂); 06-SRNP-41881, DHJPAR0012005; same data as for preceding except: 25.v.2006, Minor Carmona leg.; brown cocoons below the cadaver and adhered to the leaf substrate; adult parasitoids emerged on 04.vi.2006.

*Área de Conservación Guanacaste*, *Alajuela*, *Sector Rincón Rain Forest*, *Camino Porvenir*: • 11 (3♀, 1♂) (7♀, 0♂); 05-SRNP-40922, DHJPAR0004240; 383 m; 10.90383, -85.25964; 06.iv.2005; Minor Carmona; caterpillar collected in fifth instar; cocoons forming an irregular cordwood adhered to the leaf substrate and formed on 17.iv.2004; adult parasitoids emerged on 24.iv.2005.

*Área de Conservación Guanacaste*, *Alajuela*, *Sector Rincón Rain Forest*, *Sendero Anonás*: • 32 (0♀, 5♂) (0♀, 28♂); 07-SRNP-41122, DHJPAR0030749; 405 m; 10.90528, -85.27882; 30.iv.2007; Minor Carmona leg.; caterpillar collected in third instar; two rows of parallel brown cordwood cocoons adhered to the leaf substrate, cocoons formed on 10.v.2007; adult parasitoids emerged on 17.v.2007.

*Área de Conservación Guanacaste*, *Alajuela*, *Sector San Cristóbal*, *Corrales Viejos*: • 30 (3♀, 4♂) (23♀, 0♂); 04-SRNP-3180, DHJPAR0000278; rain forest; 495 m; 10.89974, -85.38085; 28.vi.2004; Elda Araya leg.; caterpillar collected in second instar; brown/beige cocoons with cadaver adhered on top; adult parasitoids emerged 13.vii.2004.

*Área de Conservación Guanacaste*, *Guanacaste*, *Sector Pitilla*, *Casa Roberto*: • 25 (7♀, 3♂) (15♀, 0♂); 03-SRNP-20660, DHJPAR0000040, DHJPAR0000272; rain forest; 520 m; 11.01095, -85.42094; 12.viii.2003; Petrona Rios leg.; caterpillar collected in third instar; beige cocoons were not adhered to the larva cuticle among the setae as per usual with setose species, but lightly aggregated on leaf, cocoons formed on 24.viii.2003; adult parasitoids emerged on 02.ix.2003.

*Área de Conservación Guanacaste*, *Guanacaste*, *Sector Pitilla*, *Pasmompa*: • 5 (2♀, 2♂) (1♀, 0♂); 04-SRNP-33427, DHJPAR0001503; rain forest; 440 m; 11.01926, -85.40997; 17.vi.2004; Calixto Moraga leg.; caterpillar collected in fourth instar; brown cocoons under the spiny cadaver and adhered to the leaf substrate, cocoons formed on 04.vii.2004; adult parasitoids emerged on 14.vii.2004.

*Área de Conservación Guanacaste*, *Guanacaste*, *Sector Pitilla*, *Sendero Rótulo*: • 20 (3♀, 3♂) (14♀, 0♂); 04-SRNP-33922, DHJPAR0000281; rain forest; 510 m; 11.01355, -85.42406; 17.vii.2004; Manuel Rios leg.; caterpillar collected in third instar; beige cocoons adhered to larva and substrate; adult parasitoids emerged on 03.viii.2004.

*Área de Conservación Guanacaste*, *Guanacaste*, *Sector Pitilla*, *Sendero Naciente*: • 13 (2♀, 2♂) (8♀, 1♂); 04-SRNP-34502, DHJPAR0001524; rain forest; 700 m; 10.98705, -85.42816; 13.viii.2004; Calixto Moraga leg.; caterpillar collected in third instar; beige cocoons lightly adhered together and on the leaf substrate, cocoons formed on 24.viii.2004; adult parasitoids emerged on 02.ix.2004.

*Área de Conservación Guanacaste*, *Guanacaste*, *Sector Pitilla*, *Cabrera*: • 6 (3♀, 3♂) (14♀, 2♂); 04-SRNP-55037, DHJPAR0000287; rain forest; 500 m; 11.00891, -85.40977; 05.ix.2004; Calixto Moraga leg.; caterpillar collected in fifth instar; brown cocoons adhered to the leaf and to each other, with the black spined caterpillar on top of them, cocoons adhered to the leaf substrate and formed on 15.ix.2004; adult parasitoids emerged on 21.ix.2004.

*Área de Conservación Guanacaste*, *Guanacaste*, *Sector Pitilla*, *Coneja*: • 2 (1♀, 0♂) (1♀, 0♂); [05-SRNP-34214, DHJPAR0004786; rain forest; 415 m; 11.01525, -85.39766; 05.x.2005; Calixto Moraga leg.; caterpillar collected in third instar; cocoons adhered to larva and substrate; adult parasitoids emerged on 04.xi.2005.

*Área de Conservación Guanacaste*, *Guanacaste*, *Sector Pitilla*, *Sendero Laguna*: • 25 (3♀, 3♂) (17♀, 2♂); 07-SRNP-32365, DHJPAR0030818; rain forest; 680 m; 10.9888, -85.42336; 24.v.2007; Calixto Moraga leg.; caterpillar collected in fifth instar and already with cocoons, brown parallel cordwood cocoons below the cadaver caterpillar, cocoons adhered to larva and leaf substrate, cocoons formed on 24.v.2007; adult parasitoids emerged on 30.v.2007.

*Área de Conservación Guanacaste*, *Guanacaste*, *Sector Pitilla*, *Molina*: • 16 (3♀, 2♂) (11♀, 0♂); 08-SRNP-70408, DHJPAR0030842; rain forest; 465 m; 11.00054, -85.39341; 19.v.2008; Virginia Siezar leg.; caterpillar collected in fifth instar; cocoons adhered to the leaf substrate and formed on 21.v.2008; adult parasitoids emerged on 30.v.2008.

*Área de Conservación Guanacaste*, *Guanacaste*, *Sector Pitilla*, *Sendero Memos*: • 37 (3♀, 3♂) (26♀, 5♂); 11-SRNP-31105, DHJPAR0042941; rain forest; 740 m; 10.98171, -85.42785; 16.iv.2011; Freddy Quesada leg.; caterpillar collected in third instar; cocoons adhered to the leaf substrate and formed on 03.v.2011; adult parasitoids emerged on 09.v.2011.

*Área de Conservación Guanacaste*, *Guanacaste*, *Sector Pitilla*, *Sendero Carica*: • 15 (3♀, 3♂) (8♀, 1♂); 11-SRNP-31541, DHJPAR0045221; rain forest; 660 m; 10.99284, -85.42936; 03.vi.2011; Freddy Quesada leg.; caterpillar collected in fourth instar; cocoons adhered to larva and leaf substrate, cocoons formed on 04.vi.2011; adult parasitoids emerged on 14.vi.2011.

*Área de Conservación Guanacaste*, *Guanacaste*, *Sector Pitilla*, *Sendero Nacho*: • 7 (2♀, 2♂) (2♀, 1♂); 04-SRNP-34517, DHJPAR0001530; rain forest; 710 m, 10.98445, -85.42481; 18.viii.2004; Manuel Rios leg.; caterpillar collected in third instar; dumpy lumpy brown cocoons adhered together on leaf substrate, cocoons formed on 09.ix.2004; adult parasitoids emerged on 16.ix.2004. • 27 (3♀, 2♂) (22♀, 0♂); 04-SRNP-34518, DHJPAR0001448; same data as for preceding except: caterpillar collected in fourth instar; cocoons are mostly adhered to the leaf below the cadaver, with some parts of some of them glued to the spines of the caterpillar, cocoons formed on 22.viii.2004; adult parasitoids emerged on 30.viii.2004. • 6 (2♀, 0♂) (4♀, 0♂); 04-SRNP-34974, DHJPAR0001459; same data as for preceding except: 01.ix.2004; Calixto Moragua leg.; caterpillar collected in fourth instar; brown cocoons adhered to the leaf, with cadaver of black-spined caterpillar on top, cocoons formed on 09.ix.2004; adult parasitoids emerged on 17.ix.2004. • 20 (3♀, 1♂) (16♀, 0♂); 09-SRNP-31929, DHJPAR0039961; same data as for preceding except: 14.vi.2009; Wady Obando leg.; caterpillar collected in fifth instar; mass of cocoons below cadaver, adhered to larva and substrate, cocoons formed on 15.vi.2009; adult parasitoids emerged on 24.vi.2009.

*Área de Conservación Guanacaste*, *Guanacaste*, *Sector Pitilla*, *Estación Quica*: • 22 (3♀, 3♂) (15♀, 1♂); 08-SRNP-70945, DHJPAR0031103; rain forest; 470 m; 10.99697, -85.39666; 24.vi.2008; Leonel Siezar leg.; caterpillar collected in fifth instar; brown cocoons adhered to the leaf substrate, below the cadaver of caterpillar and formed on 28.vi.2008; adult parasitoids emerged on 04.vii.2008. • 2 (1♀, 0♂) (1♀, 0♂); 08-SRNP-71014, DHJPAR0031120; same data as for preceding except: 27.vi.2008; cocoon formed on 03.vii.2008; adult parasitoids emerged on 08.vii.2008 and 12.vii.2008. • 13 (3♀, 3♂) (5♀, 2♂); 08-SRNP-71306, DHJPAR0031095; same data as for preceding except: 07.vii.2008; Oscar Siezar leg.; caterpillar collected in third instar; cocoons adhered to larva and the leaf substrate and formed on 24.vii.2008; adult parasitoids emerged on 01.viii.2008.

*Área de Conservación Guanacaste*, *Guanacaste*, *Sector Pitilla*, *Quebradona*: • 14 (5♀, 2♂) (7♀, 0♂); 09-SRNP-70186, DHJPAR0035438; rain forest; 475 m; 10.99102, -85.39539; 03.v.2009; Dinia Martinez leg.; caterpillar collected in third instar; cocoons adhered to the leaf substrate formed on 16.v.2009; adult parasitoids emerged on 24.v.2009. • 29 (5♀, 3♂) (20♀, 1♂); 09-SRNP-70652, DHJPAR0035329; same data as for preceding except: 31.v.2009; Ricardo Calero leg.

*Área de Conservación Guanacaste*, *Guanacaste*, *Sector Pitilla*, *Canita*: • 22 (5♀, 3♂) (14♀, 0♂); 09-SRNP-70653, DHJPAR0035332; rain forest; 480 m; 11.00006, -85.40195; 03.vi.2009; Dinia Martinez leg.; caterpillar collected in fifth instar; brown somewhat ordered mass of cocoons below cadaver, adhered to the leaf substrate and formed on 07.vi.2009; adult parasitoids emerged on 16.vi.2009. • 6 (5♀, 0♂) (1♀, 0♂); 09-SRNP-70655, DHJPAR0035426; same data as for preceding except: Ricardo Calero.

#### Diagnosis.

**Figure 92.**
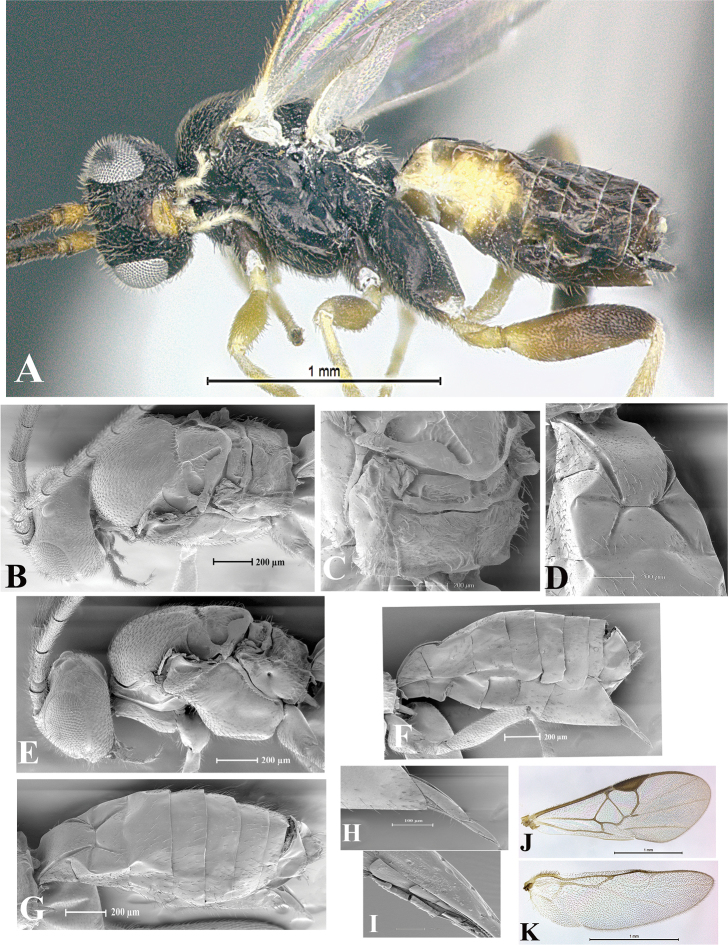
*Glyptapantelesgerarddelvarei* sp. nov. female 04-SRNP-34445 DHJPAR0000280, 08-SRNP-70408 DHJPAR0030842 **A** Habitus **B, E** Head, mesosoma **B** Dorsolateral view **E** lateral view **C** Metanotum, propodeum, dorsolateral view **D**T1–2, dorsal view **F, G** Metasoma **F** lateral view **G** Dorsolateral view **H, I** Genitalia **H** Hypopygium, ovipositor, ovipositor sheaths, lateral view **I** Ovipositor detail **J, K** Wings **J** Fore **K** Hind.

**Figure 93.**
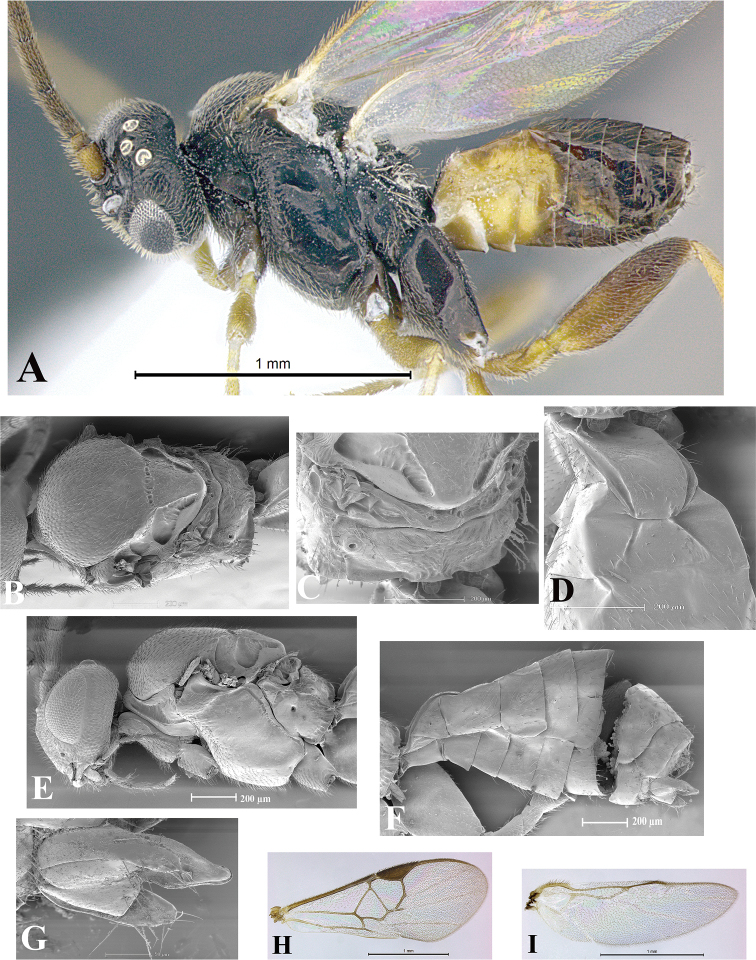
*Glyptapantelesgerarddelvarei* sp. nov. male 04-SRNP-34445 DHJPAR0000280, 08-SRNP-70408 DHJPAR0030842 **A** Habitus **B** Mesosoma, dorsal view, **C** Metanotum, propodeum, dorsal view **D**T1–2, dorsal view **E** Head, mesosoma, lateral view **F** Metasoma, lateral view **G** Genitalia: parameres, lateral view **H, I** Wings **H** Fore **I** Hind.

Precoxal groove shallow, but visible, smooth, and shiny (Figs 92A, E, 93A, E), distal antennal flagellomere longer than penultimate, median area and adjacent area on T2 dark, but lateral ends pale, inner margin of eyes straight throughout, petiole parallel-sided, but narrowing over distal 1/3, completely smooth and polished, with faint, satin-like sheen (Figs 92D, G, 93D), propodeum medially rhomboid-shaped with transverse rugae (Figs 92C, 93C), lateral grooves delimiting the median area on T2 clearly defined and reaching the distal edge of T2 (Figs 92D, 93D), and fore wing with outer side of junction of r and 2RS veins not forming a stub (Figs 92J, 93H).

#### Coloration

(Fig. 92A). General body coloration black except scape, pedicel, labrum, and mandibles yellow-brown; all antennal flagellomeres brown on both sides. Eyes and ocelli silver. Fore and middle legs yellow except coxae and claws brown; hind legs yellow except black coxae, brown femora, distal 3/4 of tibiae black and tarsomeres brown except proximal tarsomeres with apex yellow. Petiole on T1 brown with a median yellow-brown spot, contours darkened, and sublateral areas yellow-brown; T2 with median area brown, adjacent area and lateral ends light brown; T3 and beyond completely brown; distally each tergum with a narrow whitish transparent band. In lateral view, T1–3 yellow, dorsally brown; T4 and beyond completely brown. S1–2 yellow-brown; S3 proximally yellow, distally brown; S4 and beyond completely brown; ovipositor sheaths brown.

#### Description.

**Head** (Fig. 92A, B, E). Head triangular with pubescence short and dense. Proximal three antennal flagellomeres longer than wide (0.21:0.06, 0.22:0.06, 0.21:0.06), distal antennal flagellomere longer than penultimate (0.12:0.05, 0.10:0.05), antenna longer than body (2.97, 2.53); antennal scrobes-frons shallow. Face convex with dense fine punctations, interspaces wavy and longitudinal median carina present. Frons smooth. Temple wide, punctate and interspaces with microsculpture. Inner margin of eyes straight throughout; in lateral view, eye anteriorly convex and posteriorly straight. POL shorter than OOL (0.09, 0.14). Malar suture present. Median area between lateral ocelli without depression. Vertex laterally rounded and dorsally wide.

**Mesosoma** (Fig. 92A–C, E). Mesosoma dorsoventrally convex. Mesoscutum proximally convex and distallyly flat, punctation distinct throughout, interspaces wavy/lacunose. Scutellum triangular, apex sloped and fused with BS, scutellar punctation distinct throughout, in profile scutellum flat and on same plane as mesoscutum, phragma of the scutellum partially exposed; BS only very partially overlapping the MPM; ATS demilune with complete undulate/reticulate carinae; dorsal ATS groove smooth. Transscutal articulation with small and heterogeneous foveae, area just behind transscutal articulation smooth, shiny and nearly at the same level as mesoscutum (flat). Metanotum with BM wider than PFM (clearly differentiated); MPM circular without median longitudinal carina; AFM without setiferous lobes and not as well delineated as PFM; PFM thick and smooth; ATM proximally with semircular/undulate carina and distally smooth. Propodeum medially romboid-shaped with rugae, proximal half weakly curved and relatively polished, and distal half relatively polished; distal edge of propodeum with a flange at each side and without stubs; propodeal spiracle without distal carina; nucha surrounded by very short radiating carinae. Pronotum with a distinct dorsal furrow, dorsally with a well-defined smooth band; central area of pronotum and dorsal furrow smooth, but ventral groove with short parallel carinae. Propleuron with fine punctations throughout and dorsally with a carina. Metasternum flat or nearly so. Contour of mesopleuron straight/angulate or nearly so; precoxal groove smooth, shiny and shallow, but visible; epicnemial ridge elongated more fusiform (tapering at both ends).

**Legs.** Ventral margin of fore telotarsus entire, but with a tiny curved seta, fore telotarsus almost same width throughout and longer than fourth tarsomere (0.13, 0.07). Hind coxa with punctation only on ventral surface and dorsal outer depression present. Inner spur of hind tibia longer than outer spur (0.26, 0.16), entire surface of hind tibia with dense strong spines clearly differentiated by color and length. Hind telotarsus longer than fourth tarsomere (0.15, 0.13).

**Wings** (Fig. 92J, K). Fore wing with r vein curved; 2RS vein straight; r and 2RS veins forming a weak, even curve at their junction and outer side of junction not forming a stub; 2M vein straight; distally fore wing [where spectral veins are] with microtrichiae more densely concentrated than the rest of the wing; anal cell 1/3 proximally lacking microtrichiae; subbasal cell with a small smooth area; vein 2CUa absent and vein 2CUb spectral; vein 2 cu-a absent; vein 2-1A present only proximally as spectral vein; tubular vein 1 cu-a straight, incomplete/broken and not reaching the edge of 1-1A vein. Hind wing with vannal lobe very narrow, subdistally and subproximally evenly convex, and setae evenly scattered in the margin.

**Metasoma** (Fig. 92A, D, F–I). Metasoma laterally compressed. Petiole on T1 completely smooth and polished, with faint, satin-like sheen, virtually parallel-sided over most of length, but narrowing over distal 1/3 (length 0.34, maximum width 0.20, minimum width 0.13 mm), and with scattered pubescence concentrated in the first distal third. Lateral grooves delimiting the median area on T2 clearly defined and reaching the distal edge of T2 (length median area 0.15, length T2 0.15), edges of median area polished and lateral grooves deep, median area broader than long (length 0.15, maximum width 0.22, minimum width 0.08); T2 with scattered pubescence only distally. T3 longer than T2 (0.22, 0.15) and with scattered pubescence throughout. Pubescence on hypopygium dense.

**Cocoons** (Fig. 4R). Beige or brown oval cocoons with ordered silk fibers, but covered by a net. Mass of cocoons tacked lightly together in a jumbled group or forming an irregular cordwood, below the cadaver and adhered to the leaf substrate.

#### Comments.

Although the punctation in the head are fine, they looklike grains, due to the interspaces with microsculpture. The propodeum medially with a rhomboid-shaped and with rugae inside.

#### Male

(Fig. 93A–I). The male is stouter than female. The coloration on metasoma is lighter than on that of the female.

#### Etymology.

Gérard Delvare is a French entomologist interested in the systematics and phylogeny of Chalcididae. He works at the Centre de Biologie et de Gestion des Populations (CBGP), Montpellier, France.

#### Distribution.

Parasitized caterpillars were collected in Costa Rica, ACG, Sector Pitilla (Cabrera, Casa Roberto, Canita, Coneja, Estación Quica, Loaiciga, Molina, Pasmompa, Quebradona, Sendero Carica, Sendero Laguna, Sendero Memos, Sendero Naciente, Sendero Nacho, and Sendero Rótulo), Sector Rincón Rain Forest (Camino Porvenir, Camino Río Francia, Finca Hugo, and Sendero Anonás), and Sector San Cristóbal (Corrales Viejos), during June and August 2003, June-September 2004, April and October 2005, May 2006, April-May 2007, May-July 2008, May-June 2009, and April and June 2011 at 383 m, 405 m, 410 m, 415 m, 440 m, 445 m, 465 m, 470 m, 475 m, 480 m, 495 m, 500 m, 510 m, 520 m, 540 m, 660 m, 680 m, 700 m, 710 m, and 740 m in rain forest.

#### Biology.

The lifestyle of this parasitoid species is gregarious.

#### Host.

*Macrocnemecabimensis* Dyar (Erebidae: Arctiinae) (Fig. 4R) feeding on *Mandevillahirsute* and *Fischeriapanamensis* (Apocynaceae). Caterpillars were collected in second, third, fourth, and fifth instar.

### Glyptapanteles grantgentryi

Taxon classificationAnimaliaHymenopteraBraconidae

Arias-Penna, sp. nov.

http://zoobank.org/A4B9813C-BC1A-4623-BF38-1B571143ECDC

Fig. 94

#### Female.

Body length 2.88 mm, antenna length 3.58 mm, fore wing length 3.33 mm.

#### Type material.

**Holotype**: ECUADOR • 1♀; EC-2645, YY-A147; Napo, Yanayacu Biological Station, Yanayacu Road; cloud forest; 2,100 m; -0.566667, -77.866667; 03.v.2005; Harold Greeney leg.; cocoon formed on 16.v.2005; adult parasitoids emerged on 27.v.2005; (PUCE). **Paratypes.** • 1 (0♀, 1♂) (0♀, 0♂); EC-2645, YY-A147; same data as for holotype; (PUCE). 3 (1♀, 1♂) (0♀, 1♂); EC-2644, YY-A073; same data as for holotype except: cocoons formed on 26.v.2005; (PUCE).

#### Diagnosis.

**Figure 94.**
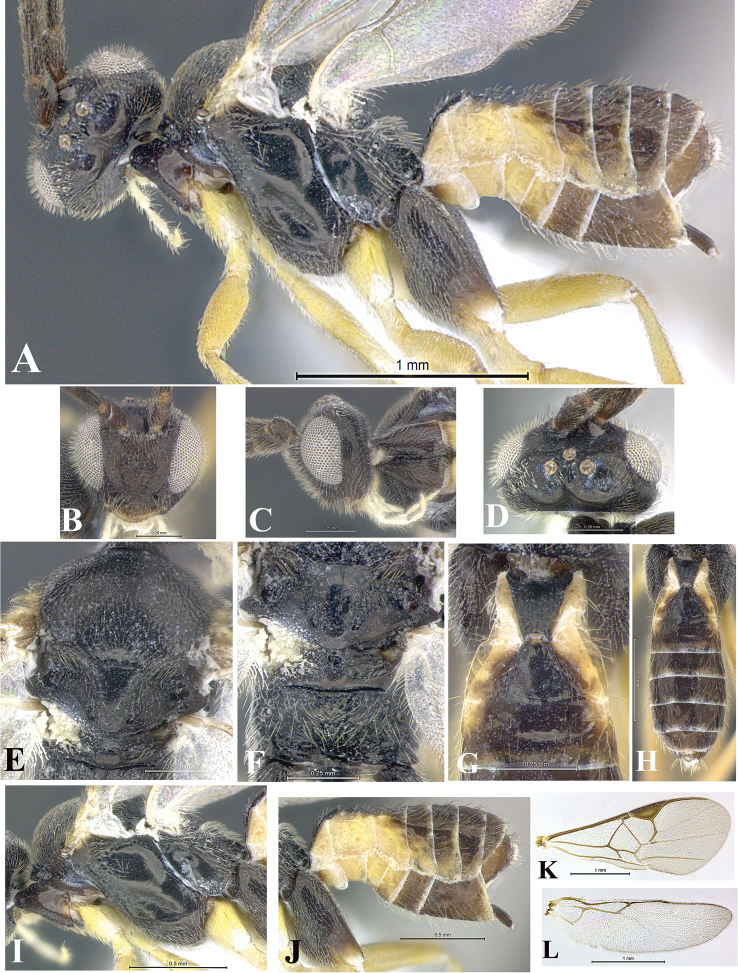
*Glyptapantelesgrantgentryi* sp. nov. female EC-2645 YY-A147 **A** Habitus **B, D** Head **B** Frontal view **D** Dorsal view **C** Head, pronotum, propleuron, lateral view **E** Mesonotum, dorsal view **F** Scutellum, metanotum, propodeum, dorsal view **G**T1–3, dorsal view **H, J** Metasoma **H** Dorsal view **J** Lateral view **I** Mesosoma, lateral view **K, L** Wings **K** Fore **L** Hind.

Vertex in lateral view pointed (Fig. 94C), frons smooth, scutellar punctation indistinct throughout (Fig. 94E), in lateral view, metasoma laterally compressed (Fig. 94A), median area on T2 broader than long (Fig. 94G), lateral grooves delimiting the median area on T2 distally losing definition on T2 (Fig. 94G), petiole on T1 parallel-sided in proximal half, then narrowing (Fig. 94G, H) and finely sculptured (Fig. 94G), propodeum without a median longitudinal dent (Fig. 94F), and fore wing with r vein straight, outer side of junction of r and 2RS veins forming a stub (Fig. 94K).

#### Coloration

(Fig. 94A–L). General body coloration brown-black, scape and all antennal flagellomeres (on both sides) brown; pedicel brown with an distal narrow yellow-brown ring; labrum light brown; mandible yellow-brown; glossa, maxillary and labial palps, and tegulae yellow; propleuron light brown with a tiny yellow-brown distal-ventral spot; dorsal and ventral furrows of pronotum somewhat lighter than mesosoma coloration. Eyes and ocelli silver. Fore and middle legs yellow except brown claws; hind legs yellow except black coxae with apex yellow, femora with a tiny brown area at the apex, tibiae with both ends brown, and tarsomeres brown, although basitarsus with a proximal yellow ring. Petiole on T1 black and sublateral ends yellow; T2 with median area black, adjacent area brown and lateral ends yellow with two elongate spots one on each side; T3 mostly brown although lateral ends with proximal half yellow/yellow-brown; T4 and beyond completely black; distally each tergum with a narrow yellow/whitish transparent band. In lateral view, T1–2 completely yellow; T3–4 yellow, but dorsally brown; T5 and beyond completely dark brown. S1 proximal half brown, distal half yellow; S2–3 completely yellow; S4 yellow, medially brown; penultimate sternum and hypopygium completely brown.

#### Description.

**Head** (Fig. 94A–D). Head rounded with pubescence long and dense. Proximal three antennal flagellomeres longer than wide (0.24:0.06, 0.26:0.06, 0.25:0.06), distal antennal flagellomere longer than penultimate (0.15:0,05, 0.10:0.05), antenna longer than body (3.58, 2.88); antennal scrobes-frons shallow. Face flat or nearly so with dense fine punctations, interspaces smooth and longitudinal median carina present. Frons smooth. Temple wide, punctate, interspaces clearly smooth. Inner margin of eyes diverging slightly at antennal sockets; in lateral view, eye anteriorly convex and posteriorly straight. POL shorter than OOL (0.09, 0.12). Malar suture present. Median area between lateral ocelli slightly depressed. Vertex laterally pointed or nearly so and dorsally wide.

**Mesosoma** (Fig. 94A, E, F, I). Mesosoma dorsoventrally convex. Mesoscutum proximally convex and distally flat, punctation distinct throughout, interspaces smooth. Scutellum triangular, apex sloped and fused with BS, scutellar punctation indistinct throughout, in profile scutellum flat and on same plane as mesoscutum, phragma of the scutellum partially exposed; BS not overlapping the MPM; ATS demilune with quite a little complete parallel carinae; dorsal ATS groove with semicircular/parallel carinae. Transscutal articulation wih large and heterogeneous foveae, area just behind transscutal articulation smooth, shiny and nearly at the same level as mesoscutum (flat). Metanotum with BM wider than PFM (clearly differentiated); MPM circular without median longitudinal carina; AFM with a small lobe and not as well delineated as PFM; PFM thick and smooth; ATM proximally with semircular/undulate carina and distally smooth. Propodeum without median longitudinal carina, proximal half weakly curved with medium-sized sculpture and distal half slightly rugose; distal edge of propodeum with a flange at each side and without stubs; propodeal spiracle distally framed by a short concave carina; nucha surrounded by very short radiating carinae. Pronotum with a distinct dorsal furrow, dorsally with a well-defined smooth band; central area of pronotum smooth, but both dorsal and ventral furrows with short parallel carinae. Propleuron finely sculptured only ventrally and dorsally without a carina. Metasternum flat or nearly so. Contour of mesopleuron straight/angulate or nearly so; precoxal groove smooth, shiny and distinct; epicnemial ridge convex, teardrop-shaped.

**Legs.** Ventral margin of fore telotarsus excavated with conspicuous curved seta over this excavation, fore telotarsus almost same width throughout and longer than fourth tarsomere (0.15, 0.09). Hind coxa with very finely punctate throughout, and dorsal outer depression present. Inner spur of hind tibia longer than outer spur (0.25, 0.21), entire surface of hind tibia with dense strong spines clearly differentiated by color and length. Hind telotarsus longer than fourth tarsomere (0.23, 0.12).

**Wings** (Fig. 94K, L). Fore wing with r vein straight; 2RS vein straight; r and 2RS veins forming an angle at their junction and outer side of junction forming a slight stub; 2M vein slightly curved/swollen; distally fore wing [where spectral veins are] with microtrichiae more densely concentrated than the rest of the wing; anal cell 1/3 proximally lacking microtrichiae; subbasal cell with microtrichiae virtually throughout; veins 2CUa and 2CUb completely spectral; vein 2 cu-a present as spectral vein, sometimes difficult to see; vein 2-1A proximally tubular and distally spectral, although sometimes difficult to see; tubular vein 1 cu-a straight, incomplete/broken and not reaching the edge of 1-1A vein. Hind wing with vannal lobe very narrow, subdistally and subproximally straightened, and setae evenly scattered in the margin.

**Metasoma** (Fig. 94A, G, H, J). Metasoma laterally compressed. Petiole on T1 finely sculptured throughout, parallel-sided in proximal half and then narrowing (length 0.37, maximum width 0.19, minimum width 0.09), and with scattered pubescence concentrated in the first distal third. Lateral grooves delimiting the median area on T2 clearly defined and reaching the distal edge of T2 (length median area 0.18, length T2 0.18), edges of median area with little sculpture, median area broader than long (length 0.18, maximum width 0.25, minimum width 0.07); T2 with scattered pubescence only distally. T3 longer than T2 (0.20, 0.18) and with scattered pubescence throughout. Pubescence on hypopygium dense.

**Cocoons.** Unknown.

#### Comments.

In females the hypopygium (S6) is the only sternum that is completely dark, penultimate sternum (S5) is yellow-brown.

#### Male.

The coloration is similar to female, except that colored adjacent area on T2 is not extensive and the two elongate spots are not noticeable, and besides T3 and beyond are completely brown. The body coloration is slightly darker than females and the hind tibia looks completely dark.

#### Etymology.

Grant Gentry is an American biologist with interests in tritrophic interactions, tropical caterpillars, and efficacies of lepidopteran larval defenses against parasitoids, with an emphasis on chemical defenses derived from food plants. He works at Samford University, Birmingham, AL, USA.

#### Distribution.

Parasitized caterpillar was collected in Ecuador, Napo, Yanayacu Biological Station (Yanayacu Road), during May 2005 at 2,100 m in cloud forest.

#### Biology.

The lifestyle of this parasitoid species is gregarious.

#### Host.

Undetermined species of Notodontidae feeding on *Myriocarpa* sp. (Urticaceae). Caterpillar instar was not reported.

### Glyptapanteles gunnarbrehmi

Taxon classificationAnimaliaHymenopteraBraconidae

Arias-Penna, sp. nov.

http://zoobank.org/7D57AE5E-7B8A-4307-B016-D89E04B86258

Fig. 95

#### Female.

Body length 3.03 mm, antenna length 3.48 mm, fore wing length 3.43 mm.

#### Type material.

**Holotype**: ECUADOR • 1♀; EC-15124, YY-A043; Napo, Yanayacu Biological Station, Yanayacu Road; cloud forest; 2,100 m; -0.566667, -77.866667; 02.vi.2006; Wilmer Simbaña leg.; caterpillar collected in second instar; cocoons formed on 13.vi.2006; adult parasitoids emerged on 04.vii.2006; (PUCE). **Paratypes.** • 4 (2♀, 1♂) (0♀, 1♂); EC-15124, YY-A043; same data as for holotype; (PUCE).

#### Other material.

**Reared material.** ECUADOR: *Napo*, *Yanayacu Biological Station*, *Yanayacu forest*: • 7 (6♀, 0♂) (1♀, 0♂); EC-1406, YY-A072; cloud forest; 2,100 m; -0.6, -77.883333; 21.i.2005; Lee Dyer leg.; caterpillar collected in second instar; cocoons formed on 23.ii.2006; adult parasitoids emerged on 25.ii.2005.

#### Diagnosis.

**Figure 95.**
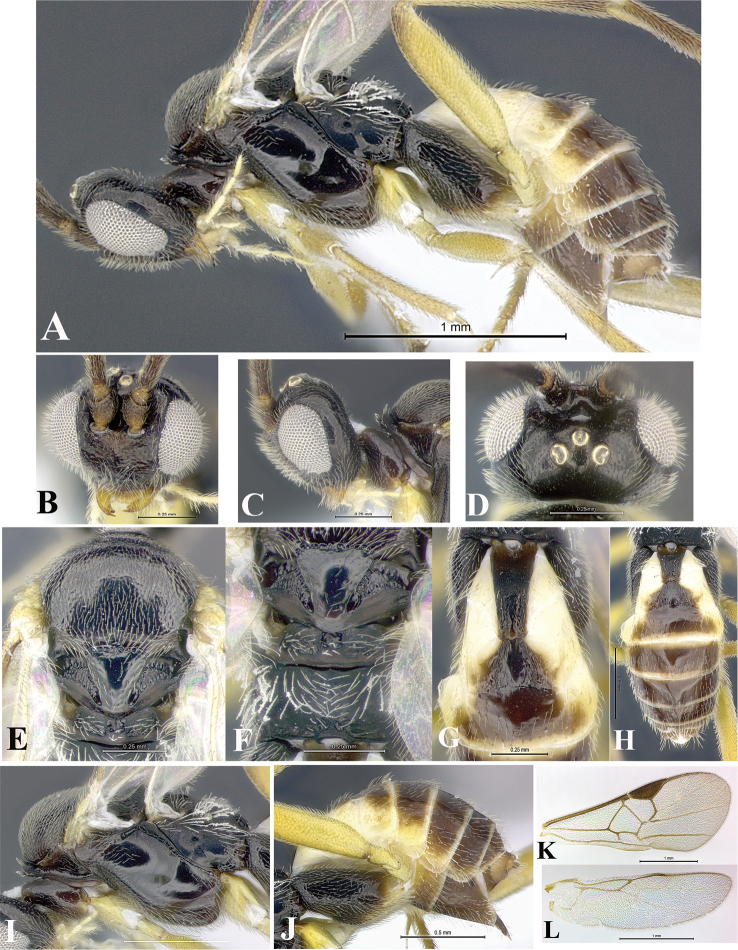
*Glyptapantelesgunnarbrehmi* sp. nov. female EC-1406 YY-A072, EC-15124 YY-A043 **A** Habitus **B, D** Head **B** Frontal view **D** Dorsal view **C** Head, pronotum, propleuron, lateral view **E** Mesonotum, dorsal view **F** Scutellum, metanotum, propodeum, dorsal view **G**T1–3, dorsal view **H, J** Metasoma **H** Dorsal view **J** Lateral view **I** Mesosoma, lateral view **K, L** Wings **K** Fore **L** Hind.

Hind coxa finely punctate throughout (Fig. 95J), antenna longer than body, distal antennal flagellomere longer than penultimate, scutellar punctation scattered throughout (Fig. 95E, F), fore wing with vein 2 cu-a present as spectral vein, sometimes difficult to see, r vein straight, and outer side of junction of r and 2RS veins forming a stub (Fig. 95K), median area on T2 broader than long, edges of median area on T2 obscured by weak longitudinal stripes, and lateral grooves delimiting the median area on T2 distally losing definition on T2 (Fig. 95G, H), vertex in dorsal view wide (Fig. 95D), in lateral view, metasoma laterally compressed (Fig. 95A, J), T3 longer than T2 (Fig. 95H), inner margin of eyes diverging slightly at antennal sockets (Fig. 95B), petiole on T1 evenly narrowing distally (wide base to a very narrow apex) and finely sculptured (Fig. 95G, H), and propodeum without a median longitudinal dent (Fig. 95F).

#### Coloration

(Fig. 95A–L). General body coloration polished black except proximally scape and apically pedicel yellow-brown; all antennal flagellomeres dark brown on both sides; labrum and mandibles light brown; glossa, maxillary and labial palps, and tegulae yellow; propleuron light brown with a tiny yellow-brown distal-ventral spot; both dorsal and ventral furrows of pronotum and ventrally mesosoma with coloration lighter than rest of mesosoma. Eyes silver and ocelli yellowish. Fore and middle legs yellow except brown claws; hind legs yellow except black coxae with apex yellow, femora with a tiny brown area at the apex, tibiae with apex brown, and tarsomeres brown. Petiole on T1 black and sublateral areas light yellow; T2 with median area black, adjacent area brown and lateral ends yellow; T3 mostly brown, lateral ends with proximal half yellow/yellow-brown and distally with a yellow band; T4 and beyond completely brown; distally each tergum with a narrow yellow/whitish transparent band. In lateral view, T1–2 completely ivory; T3–4 yellow, but dorsally brown; T5 and beyond completely dark brown. S1-2 completely yellow; S3–4 yellow, medially brown; penultimate sternum and hypopygium completely brown.

#### Description.

**Head** (Fig. 95A–D). Head rhomboid with pubescence long and dense. Proximal three antennal flagellomeres longer than wide (0.26:0.09, 0.27:0.09, 0.25:0.09), distal antennal flagellomere longer than penultimate (0.15:0.06, 0.10:0.06), antenna longer than body (3.48, 3.03); antennal scrobes-frons sloped and forming a shelf. Face flat or nearly so, with dense fine punctations, interspaces wavy and longitudinal median carina present. Frons smooth. Temple wide, punctate and interspaces clearly smooth. Inner margin of eyes diverging slightly at antennal sockets; in lateral view, eye anteriorly convex and posteriorly straight. POL shorter than OOL (0.09, 0.14). Malar suture present. Median area between lateral ocelli slightly depressed. Vertex laterally pointed or nearly so and dorsally wide.

**Mesosoma** (Fig. 95A, E, F, I). Mesosoma dorsoventrally convex. Mesoscutum proximally convex and distally flat, punctation distinct throughout, interspaces smooth. Scutellum long and slender, apex sloped and fused with BS, scutellar punctation scattered throughout, in profile scutellum flat and on same plane as mesoscutum, phragma of the scutellum partially exposed; BS only very partially overlapping the MPM; ATS demilune with short stubs delineating the area; dorsal ATS groove with semicircular/parallel carinae. Transscutal articulation with small and heterogeneous foveae, area just behind transscutal articulation with a smooth and shiny sloped transverse strip. Metanotum with BM wider than PFM (clearly differentiated); MPM circular and bisected by a median longitudinal carina; AFM with a small lobe and not as well delineated as PFM; PFM thick, smooth and with a distal flat flange; ATM proximally with semircular/undulate carina and distally smooth. Propodeum without median longitudinal carina, proximal half weakly curved with medium-sized sculpture and distal half relatively polished; distal edge of propodeum with a flange at each side and without stubs; propodeal spiracle without distal carina; nucha surrounded by very short radiating carinae. Pronotum with a distinct dorsal furrow, dorsally with a well-defined smooth band; central area of pronotum smooth, but both dorsal and ventral furrows with short parallel carinae. Propleuron finely sculptured only ventrally and dorsally without a carina. Metasternum convex. Contour of mesopleuron convex; precoxal groove deep, smooth and shiny; epicnemial ridge elongated more fusiform (tapering at both ends).

**Legs.** Ventral margin of fore telotarsus slightly excavated and with a tiny curved seta, fore telotarsus almost same width throughout and longer than fourth tarsomere (0.15, 0.09). Hind coxa with very finely punctate throughout and dorsal outer depression present. Inner spur of hind tibia longer than outer spur (0.25, 0.20), entire surface of hind tibia with dense strong spines clearly differentiated by color and length. Hind telotarsus as equal in length as fourth tarsomere (0.13, 0.14).

**Wings** (Fig. 95K, L). Fore wing with r vein straight; 2RS vein slightly concave; r and 2RS veins forming a weak, even curve at their junction and outer side of junction forming a slight stub; 2M vein slightly curved/swollen; distally fore wing [where spectral veins are] with microtrichiae more densely concentrated than the rest of the wing; anal cell 1/3 proximally lacking microtrichiae; subbasal cell with microtrichiae virtually throughout; veins 2CUa and 2CUb completely spectral; vein 2 cu-a present as spectral vein, sometimes difficult to see; vein 2-1A proximally tubular and distally spectral, although sometimes difficult to see; tubular vein 1 cu-a straight, incomplete/broken and not reaching the edge of 1-1A vein. Hind wing with vannal lobe narrow, subdistally and subproximally straightened, and setae evenly scattered in the margin.

**Metasoma** (Fig. 95A, G, H, J). Metasoma laterally compressed. Petiole on T1 finely sculptured throughout, evenly narrowing distally (length 0.39, maximum width 0.19, minimum width 0.10) and with scattered pubescence concentrated in the first distal third. Lateral grooves delimiting the median area on T2 clearly defined and reaching the distal edge of T2 (length median area 0.18, length T2 0.18), edges of median area obscured by weak longitudinal stripes, median area broader than long (length 0.18, maximum width 0.25, minimum width 0.08), T2 with scattered pubescence only distally. T3 longer than T2 (0.24, 0.18) and with scattered pubescence only distally. Pubescence on hypopygium dense.

**Cocoons.** Unknown.

#### Male.

Similar in coloration to female.

#### Etymology.

Gunnar Brehm is a German ecologist. His research focuses on macroecology, biogeography, and systematics of species-rich moth communities in Ecuador and Costa Rica. He works at Phyletisches Museum, Jena, Germany.

#### Distribution.

Parasitized caterpillars were collected in Ecuador, Napo, Yanayacu Biological Station (Yanayacu Road), during January 2005 and June 2006 at 2,100 m in cloud forest.

#### Biology.

The lifestyle of this parasitoid species is gregarious.

#### Host.

*Pantherodescolubrariaviperaria* Thierry-Mieg (Geometriidae: Ennominae) feeding on *Boehmeriacaudata* (Urticaceae). Undetermined species of Lepidoptera feeding on *Miriocarpa* sp. (Urticaceae). Caterpillars were collected in second instar.

### Glyptapanteles haroldgreeneyi

Taxon classificationAnimaliaHymenopteraBraconidae

Arias-Penna, sp. nov.

http://zoobank.org/720000EA-D353-4572-A860-77E2ED3F866F

Fig. 96

#### Female.

Body length 2.97 mm, antenna length 3.78 mm, fore wing length 3.88 mm.

#### Type material.

**Holotype**: ECUADOR • 1♀; EC-29376, YY-A111; Napo, Yanayacu Biological Station, Río Aliso, Isla del río Aliso; cloud forest; 2,100 m; -0.633333, -77.9; 23.i.2008; CAPEA leg.; caterpillar collected in third instar; cocoons formed on 19.ii.2008; adult parasitoids emerged on 21.iii.2008; (PUCE). **Paratypes**. • 2 (1♀, 0♂) (1♀, 0♂); EC-29350, YY-A014; same data as for holotype except: adult parasitoids emerged on 10.iii.2008; (PUCE). • 1 (0♀, 1♂) (0♀, 0♂); EC-29351, YY-A015; same data as for holotype except: adult parasitoids emerged on 10.iii.200; (PUCE). • 1 (1♀, 0♂) (0♀, 0♂); EC-29353, YY-A012; same data as for holotype, (PUCE). • 3 (1♀, 1♂) (1♀, 0♂); EC-29354, YY-A112; same data as for holotype except: adult parasitoids emerged on 10.iii.2008; (PUCE). • 1 (0♀, 1♂) (0♀, 0♂); EC-29358, YY-A013; same data as for holotype except: cocoons formed on 20.ii.2008; adult parasitoids emerged on 10.iii.2008; (PUCE). • 1 (1♀, 0♂) (0♀, 0♂); EC-29381, YY-A120; same data as for holotype except: cocoons formed on 20.ii.2008; (PUCE).

#### Other material.

**Reared material.** ECUADOR: *Napo*, *Yanayacu Biological Station*, *Yanayacu Road*: • 1 (1♀, 0♂) (0♀, 0♂); EC-28946, YY-A097; cloud forest; 2,100 m; -0.566667, -77.866667; 10.xii.2007; CAPEA leg.; caterpillar collected in second instar; cocoons formed on 01.ii.2008; adult parasitoids emerged on 25.ii.2008. • 1 (1♀, 0♂) (0♀, 0♂); EC-28947, YY-A056; same data as for preceding except: adult parasitoids emerged on 19.ii.2008.

*Napo*, *Yanayacu Biological Station*, *Road Río Aliso*: • 1 (1♀, 0♂) (0♀, 0♂); EC-29059, YY-A166; -0.633333, -77.9; 26.xii.2007; CAPEA leg.; caterpillar collected in second instar; cocoons formed on 13.ii.2008; adult parasitoids emerged on 10.iii.2008. • 2 (1♀, 0♂) (1♀, 0♂); EC-29060, YY-A118; same data as for preceding except: cocoons formed on 26.ii.2008; adult parasitoids emerged on 22.iii.2008. • 1 (1♀, 0♂) (0♀, 0♂); EC-29061, YY-A019; same data as for preceding except: cocoons formed on 26.ii.2008; adult parasitoids emerged on 22.iii.2008.

#### Diagnosis.

**Figure 96.**
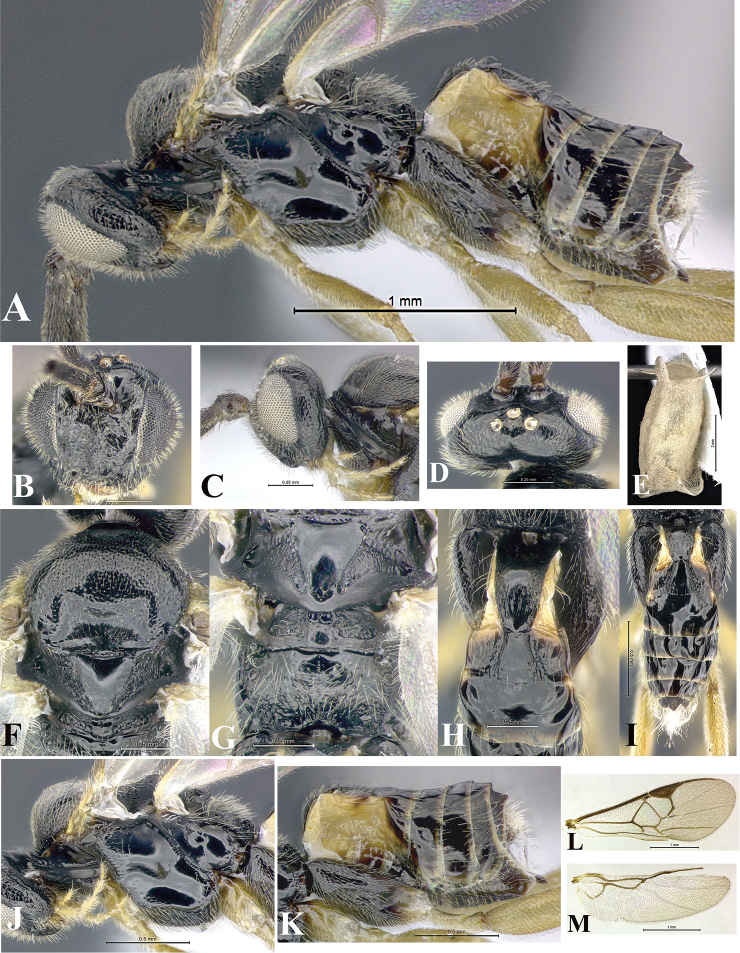
*Glyptapantelesharoldgreeneyi* sp. nov. female EC-29059 YY-A166, EC-29061 YY-A019, EC-29376 YY-A111 **A** Habitus **B, D** Head **B** Frontal view **D** Dorsal view **C** Head, pronotum, propleuron, lateral view **E** Cocoon **F** Mesonotum, dorsal view **G** Scutellum, metanotum, propodeum, dorsal view **H**T1–3, dorsal view **I, K** Metasoma **I** Dorsal view **K** Lateral view **J** Mesosoma, lateral view **L, M** Wings **L** Fore **M** Hind.

Scutellum shiny smooth (Fig. 96F, G), medioposterior band of scutellum mostly overlapping the medioanterior pit of metanotum (Fig. 96G), petiole on T1 evenly narrowing over its length (Fig. 96H, I), surface of metasternum flat or nearly so, edges of median area on T2 obscured by weak longitudinal stripes (Fig. 96H, I), dorsal outer depression on hind coxa absent (Fig. 96A, K), and fore wing with r vein slightly curved, outer side of junction of r and 2RS veins forming a stub (Fig. 96L).

#### Coloration

(Fig. 96A–M). General body coloration polished satin black except all antennal flagellomeres (on both sides) brown; proximally scape, apex of pedicel, dorsal furrow of pronotum, and spot ventro-distal of propleuron with yellow-brown/reddish tints; labrum, mandible and glossa light brown; maxillary and labial palps, and tegulae yellow. Eyes and ocelli silver. Fore and middle legs yellow-brown except brown claws and yellow-brown tarsomeres (intensity of yellow-brown coloration increasing from proximal to distal); hind legs yellow-brown except black coxae with yellow apex (coloration extensive in the inner side), tibiae with apex brown, tarsomeres brown, although telotarsus proximally with yellow ring. Petiole on T1 satin black and sublateral areas yellow; T2 with median area and lateral ends black; T3 and beyond black; distally each tergum with a narrow yellowish transparent band. In lateral view, T1–2 completely yellow; T3 yellow, dorsally brown; T4 and beyond completely black. S1 completely yellow; S–4 yellow, medially brown, area covered by brown coloration increasing from proximal to distal; penultimate sternum and hypopygium completely brown.

#### Description.

**Head** (Fig. 96A–D). Head rhomboid with pubescence long and dense. Proximal three antennal flagellomeres longer than wide (0.29:0.08, 0.29:0.08, 0.30:0.08), distal antennal flagellomere longer than penultimate (0.15:0.05, 0.12:0.06), antenna longer than body (3.78, 2.97); antennal scrobes-frons sloped and forming a shelf. Face with depression only laterally, face with dense and finely punctate, interspaces wavy and longitudinal median carina present. Frons smooth. Temple wide, punctate and interspaces wavy. Inner margin of eyes diverging slightly at antennal sockets; in lateral view, eye anteriorly convex and posteriorly straight. POL shorter than OOL (0.10, 0.17). Malar suture present. Median area between lateral ocelli without depression. Vertex laterally pointed or nearly so and dorsally wide.

**Mesosoma** (Fig. 96A, F, G, J). Mesosoma dorsoventrally convex. Mesoscutum proximally convex and distally flat, punctation distinct throughout, interspaces smooth. Scutellum long and slender, apex sloped and fused with BS, scutellar punctation indistinct throughout, in profile scutellum slightly convex, but on same plane as mesoscutum, phragma of the scutellum partially exposed; BS mostly overlapping the MPM; ATS demilune with short stubs delineating the area; dorsal ATS groove with semicircular/parallel carinae. Transscutal articulation with small and heterogeneous foveae, area just behind transscutal articulation smooth, shiny and depressed centrally although sometimes with a sloped transverse strip. Metanotum with BM wider than PFM (clearly differentiated); MPM semicircular without median longitudinal carina; AFM with a small lobe and not as well delineated as PFM; PFM thick, smooth and with a proximal flat flange; ATM proximally with a groove with some sculpturing and distally with rugae. Propodeum relatively polished without median longitudinal carina and proximal half curved; distal edge of propodeum with a flange at each side and without stubs; propodeal spiracle without distal carina; nucha surrounded by very short radiating carinae. Pronotum with a distinct dorsal furrow, dorsally with a well-defined smooth band; central area of pronotum smooth, but both dorsal and ventral furrows with short parallel carinae. Propleuron with fine punctations throughout and dorsally without a carina. Metasternum flat or nearly so. Contour of mesopleuron straight/angulate or nearly so; precoxal groove deep, smooth and shiny; epicnemial ridge widen.

**Legs.** Ventral margin of fore telotarsus entire without seta, fore telotarsus proximally narrow and distally wide, and as equal in length as fourth tarsomere (0.11, 0.11). Hind coxa with medium-size punctate throughout, and dorsal outer depression absent. Inner spur of hind tibia longer than outer spur (0.25, 0.20), entire surface of hind tibia with dense strong spines clearly differentiated by color and length. Hind telotarsus longer than fourth tarsomere (0.17, 0.15).

**Wings** (Fig. 96L, M). Fore wing with r vein slightly curved; 2RS vein straight; r and 2RS veins forming a weak, even curve at their junction and outer side of junction forming a distinct stub; 2M vein slightly curved/swollen; distally fore wing [where spectral veins are] with microtrichiae more densely concentrated than the rest of the wing; anal cell 1/3 proximally lacking microtrichiae; subbasal cell with microtrichiae virtually throughout; veins 2CUa and 2CUb completely spectral; vein 2 cu-a present as spectral vein, sometimes difficult to see; vein 2-1A proximally tubular and distally spectral, although sometimes difficult to see; tubular vein 1 cu-a curved and complete, but junction with 1-1A vein spectral. Hind wing with vannal lobe narrow, subdistally and subproximally evenly convex, and setae evenly scattered in the margin.

**Metasoma** (Fig. 96A, H, I, K). Metasoma laterally compressed. Petiole on T1 finely sculptured throughout, evenly narrowing distally (length 0.48, maximum width 0.29, minimum width 0.13), and with scattered pubescence concentrated in the first distal third. Lateral grooves delimiting the median area on T2 clearly defined and reaching the distal edge of T2 (length median area 0.23, length T2 0.23), edges of median area obscured by weak longitudinal stripes, median area broader than long (length 0.23, maximum width 0.25, minimum width 0.11); T2 with scattered pubescence only distally. T3 longer than T2 (0.27, 0.23) and with scattered pubescence throughout. Pubescence on hypopygium dense.

**Cocoons** (Fig. 96E). White or beige bud-like cocoons with body ridge-shaped and silk fibers evenly smooth.

#### Comments.

Some females with a transverse strip just behind transscutal articulation. In lateral view, the mesosoma is slightly flat.

#### Male.

Similar in coloration to female.

#### Etymology.

Harold Francis Greeney III is a biologist, the founder and director of Yanayacu Biological Station, Ecuador.

#### Distribution.

Parasitized caterpillars were collected in Ecuador, Napo, Yanayacu Biological Station (Río Aliso, Yanayacu Road, and Road Río Aliso), during December 2007 and January 2008 at 2,100 m in cloud forest.

#### Biology.

The lifestyle of this parasitoid species is solitary/gregarious.

#### Host.

*Actinotestratonice* Latreille (Nymphalidae: Acraeinae) feeding on *Eratopolymnioides* and *Munnoziahastifolia* (Asteraceae). Caterpillars were collected in second and third instar.

### Glyptapanteles helmuthaguirrei

Taxon classificationAnimaliaHymenopteraBraconidae

Arias-Penna, sp. nov.

http://zoobank.org/2F8BEFA4-EA26-4B04-96BF-105E8C74C66F

Fig. 97

#### Female.

Body length 3.08 mm, antenna length 3.43 mm, fore wing length 3.38 mm.

#### Type material.

**Holotype**: ECUADOR • 1♀; EC-26313, YY-A060; Napo, Yanayacu Biological Station, Granja Integral Baeza, Baeza sendero granja; cloud forest; 1,700 m; -0.5833, -77.8833; 17.ix.2007; Rafael Granizo leg.; caterpillar collected in fourth instar; white bud-like cocoons formed on 22.ix.2008; adult parasitoids emerged on 12.x.2007; (PUCE). **Paratypes.** • 3 (2♀, 0♂) (1♀, 0♂); EC-26313, YY-A060; same data as for holotype; (PUCE).

#### Diagnosis.

**Figure 97.**
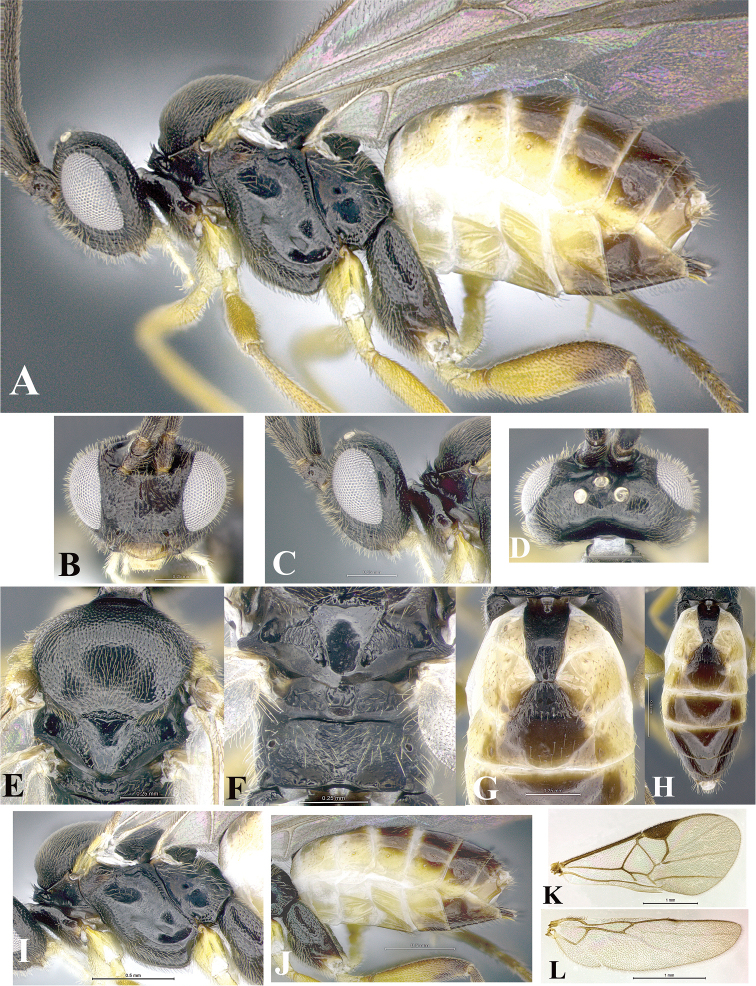
*Glyptapanteleshelmuthaguirrei* sp. nov. female EC-26313 YY-A060 **A** Habitus **B, D** Head **B** Frontal view **D** Dorsal view **C** Head, pronotum, propleuron, lateral view **E** Mesonotum, dorsal view **F** Scutellum, metanotum, propodeum, dorsal view **G**T1–3, dorsal view **H, J** Metasoma **H** Dorsal view **J** Lateral view **I** Mesosoma, lateral view **K, L** Wings **K** Fore **L** Hind.

Malar suture present (Fig. 97B), median area between lateral ocelli without depression (Fig. 97D), propodeum medially rhomboid-shaped with transverse rugae (Fig. 97F), scutellar punctation indistinct throughout (Fig. 97E, F), axillary trough of metanotum proximally with a groove with some sculpturing, distally with rugae (Fig. 97E, F), anterior furrow of metanotum with a small lobe without setae (Fig. 97F), petiole on T1 parallel-sided in proximal half, then narrowing (Fig. 97G, H), edges of median area on T2 obscured by weak longitudinal stripes (Fig. 97G), dorsal outer depression on hind coxa present (Fig. 97A, J), and fore wing with r vein curved, outer side of junction of r and 2RS veins forming a slight stub (Fig. 97K).

#### Coloration

(Fig. 97A–L). General body coloration black except all antennal flagellomeres brown on both sides; scape brown with a yellow-brown/reddish ring; pedicel brown distally with yellow-brown ring; labrum and mandible yellow-brown; glossa, maxillary and labial palps, and tegulae yellow; propleuron with a small yellow spot ventro-distal; dorsal furrow of pronotum lighter than mesosoma coloration. Eyes and ocelli silver. Fore and middle legs yellow except brown claws, and tarsomeres brown (intensity of brown coloration increasing from proximal to distal); hind legs yellow except black coxae only distally yellow, femora distally brown, which ventrally with a distinctive dark spot, tibiae with both ends brown (shape of the brown coloration in distal half is particular: proximally narrow and distally wide, however outer side yellow), and tarsomeres brown, although basitarsus proximally with a yellow ring. Petiole on T1 black and sublateral ends yellow; T2 with median area black, adjacent area brown, and lateral ends yellow with two elongate brown spots one on each distal edge; T3 with a extended brown area which width proximally coincides with the width of median and adjacent areas on T2; however distally T3 with a yellow-brown band that extent along the width of T3, distally T3 also with two elongate spots; T4 and beyond completely brown; distally each tergum with a narrow yellow/yellow-brown transparent band. In lateral view, T1–3 completely yellow; T4 and beyond yellow, dorsally brown, extent of brown area remains constant. S1–4 completely yellow; penultimate sternum yellow, medially brown; hypopygium completely brown.

#### Description.

**Head** (Fig. 97A–D). Head rhomboid with pubescence long and dense. Proximal three antennal flagellomeres longer than wide (0.24:0.09, 0.25:0.09, 0.23:0.09), distal antennal flagellomere longer than penultimate (0.13:0.06, 0.11:0.06), antenna longer than body (3.43, 3.08); antennal scrobes-frons shallow. Face flat or nearly so, with dense and fine punctations, interspaces wavy and longitudinal median carina present. Frons smooth. Temple wide, punctate and interspaces clearly smooth. Inner margin of eyes diverging slightly at antennal sockets; in lateral view, eye anteriorly convex and posteriorly straight. POL shorter than OOL (0.11, 0.14). Malar suture present. Median area between lateral ocelli without depression. Vertex laterally rounded and dorsally wide.

**Mesosoma** (Fig. 97A, E, F, I). Mesosoma dorsoventrally convex. Mesoscutum proximally convex and distally flat, punctation distinct throughout, interspaces smooth. Scutellum long and slender, apex sloped and fused with BS, scutellar punctation indistinct throughout, in profile scutellum flat and on same plane as mesoscutum, phragma of the scutellum partially exposed; BS only very partially overlapping the MPM; ATS demilune with complete undulate/reticulate carinae; dorsal ATS groove with semicircular/parallel carinae. Transscutal articulation with small and heterogeneous foveae, area just behind transscutal articulation depressed centrally, smooth and shiny. Metanotum with BM wider than PFM (clearly differentiated); MPM circular and bisected by a median longitudinal carina; AFM with a small lobe and not as well delineated as PFM; PFM thick, smooth and with lateral ends rounded; ATM proximally with a groove with some sculpturing and distally with rugae. Propodeum with transverse rugae, proximal half curved with medium-sized sculpture and distal half relatively polished; distal edge of propodeum with a flange at each side and without stubs; propodeal spiracle without distal carina; nucha surrounded by very short radiating carinae. Pronotum with a distinct dorsal furrow, dorsally with a well-defined smooth band; central area of pronotum smooth, but both dorsal and ventral furrows with short parallel carinae. Propleuron with fine punctations throughout and dorsally without a carina. Metasternum flat or nearly so. Contour of mesopleuron straight/angulate or nearly so; precoxal groove smooth, shiny and shallow, but visible; epicnemial ridge elongated more fusiform (tapering at both ends).

**Legs.** Ventral margin of fore telotarsus entire without seta, fore telotarsus almost same width throughout and longer than fourth tarsomere (0.12, 0.08). Dorsal half of hind coxa with scattered punctation and ventral half with dense punctation, and dorsal outer depression present. Inner spur of hind tibia longer than outer spur (0.22, 0.20), entire surface of hind tibia with dense strong spines clearly differentiated by color and length. Hind telotarsus longer than fourth tarsomere (0.13, 0.11).

**Wings** (Fig. 97K, L). Fore wing with r vein slightly curved; 2RS vein slightly convex to convex; r and 2RS veins forming a weak, even curve at their junction and outer side of junction forming a slight stub; 2M vein slightly curved/swollen; distally fore wing [where spectral veins are] with microtrichiae more densely concentrated than the rest of the wing; anal cell 1/3 proximally lacking microtrichiae; subbasal cell with microtrichiae virtually throughout; veins 2CUa and 2CUb completely spectral; vein 2 cu-a present as spectral vein, sometimes difficult to see; vein 2-1A proximally tubular and distally spectral, although sometimes difficult to see; tubular vein 1 cu-a straight, incomplete/broken and not reaching the edge of 1-1A vein. Hind wing with vannal lobe very narrow, subdistally and subproximally straightened, and setae evenly scattered in the margin.

**Metasoma** (Fig. 97A, G, H, J). Metasoma laterally compressed. Petiole on T1 finely sculptured throughout, parallel-sided in proximal half and then narrowing (length 0.42, maximum width 0.20, minimum width 0.10), and with scattered pubescence concentrated in the first distal third. Lateral grooves delimiting the median area on T2 clearly defined and reaching the distal edge of T2 (length median area 0.20, length T2 0.20), edges of median area obscured by weak longitudinal stripes, median area broader than long (length 0.20, maximum width 0.27, minimum width 0.10); T2 with scattered pubescence only distally. T3 longer than T2 (0.26, 0.20) and with scattered pubescence throughout. Pubescence on hypopygium dense.

**Cocoons.** White bud-like cocoons.

#### Comments.

The mesopleuron is so convex that it looks rounded. Distally the pronotum is higher (convex) than proximally (concave).

#### Male.

Unknown.

#### Etymology.

Helmuth Aguirre Fernández is a Colombian entomologist who studies taxonomy and systematics of *Meteorus* (Meteorinae, Braconidae) in the Neotropics. He earned his Ph.D. at the University of Wyoming, Laramie, WY, USA.

#### Distribution.

Parasitized caterpillar was collected in Ecuador, Napo, Yanayacu Biological Station (Granja Integral Baeza), during September 2007 at 1,700 m in cloud forest.

#### Biology.

The lifestyle of this parasitoid species is gregarious.

#### Host.

Undetermined species of Pieridae feeding on *Inga* sp. (Fabaceae). Caterpillar was collected in fourth instar.

### Glyptapanteles henryhespenheidei

Taxon classificationAnimaliaHymenopteraBraconidae

Arias-Penna, sp. nov.

http://zoobank.org/664964ED-ABC2-486D-9F7C-335923867AFF

Fig. 98

#### Female.

Body length 3.23 mm, antenna length 3.73 mm, fore wing length 3.53 mm.

#### Type material.

**Holotype**: ECUADOR • 1♀; EC-11240, YY-A040; Napo, Yanayacu Biological Station, Sierra Azul Camino Cascadas, Plot 150; cloud forest; 2,280 m; -0.7, -77.933333; 22.xii.2005; Aaron Fox leg.; caterpillar collected in second instar; cocoons formed on 22.i.2006; adult parasitoids emerged on 01.ii.2006; (PUCE). **Paratypes.** • 3 (2♀, 0♂) (1♀, 0♂); EC-11240, YY-A040; same data as for holotype; (PUCE).

#### Diagnosis.

**Figure 98.**
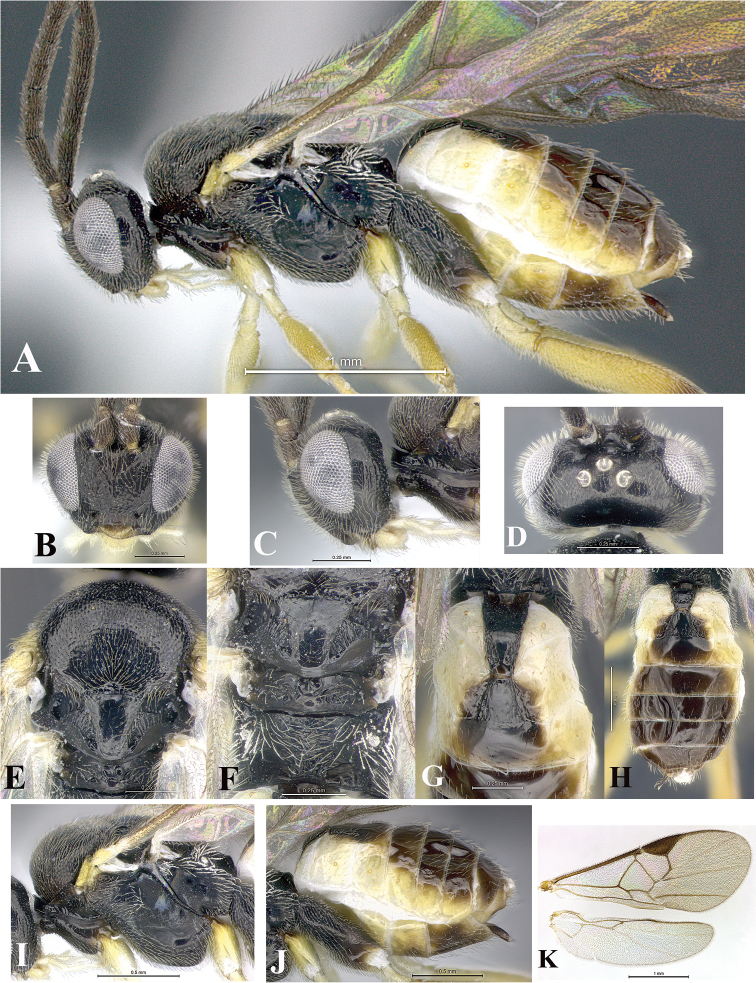
*Glyptapanteleshenryhespenheidei* sp. nov. female EC-11240 YY-A040 **A** Habitus **B, D** Head **B** Frontal view **D** Dorsal view **C** Head, pronotum, propleuron, lateral view **E** Mesonotum, dorsal view **F** Scutellum, metanotum, propodeum, dorsal view **G**T1–3, dorsal view **H, J** Metasoma **H** Dorsal view **J** Lateral view **I** Mesosoma, lateral view **K** Fore and hind wings.

Distal 1/3 of mesoscutum with lateral margin slightly dented, punctation distinct throughout (Fig. 98E), scutellar punctation scattered throughout (Fig. 98E, F), antenna longer than body, phragma of the scutellum completely concealed (Fig. 98E, F), T3 as long as T2 (Fig. 98H), fore wing with r vein straight, outer side of junction of r and 2RS veins not forming a stub (Fig. 98K), inner margin of eyes diverging slightly at antennal sockets (Fig. 98B), petiole on T1 finely sculptured only laterally (Fig. 98G, H), propodeum without median longitudinal carina (Fig. 98F), and lateral grooves delimiting the median area on T2 clearly defined and reaching the distal edge of T2 (Figs 98G, H).

#### Coloration

(Fig. 98A–K). General body coloration polished black except a small area on postero-ventral in propleuron, and both dorsal and ventral furrows of pronotum with brown-red/reddish tints; labrum and mandibles yellow-brown; glossa, maxillary and labial palps, and tegulae yellow; proximal ring on scape and distal ring in pedicel yellow-brown/reddish; all antennal flagellomeres brown on both sides. Eyes and ocelli silver. Fore and middle legs yellow except brown claws; hind legs yellow except black coxae with apex yellow (coloration extensive in the inner side), femora with a small brown area in the apex, both ends of tibiae brown, and tarsomeres brown. Petiole on T1 black and sublateral areas yellow; T2 with median and adjacent areas brown, and lateral ends yellow; T3 medially mostly with an elongate brown area that not touching the distal margin of T3 and lateral ends yellow-brown; T4 and beyond brown; distally each tergum with a narrow yellow-brown transparent band. In lateral view, T1–2 completely yellow; T3 and beyond yellow, dorsally brown, extent of brown area remains constant. S1–4 completely yellow; penultimate sternum yellow with a longitudinal median brown band; hypopygium brown with some small yellow areas.

#### Description.

**Head** (Fig. 98A–D). Head rhomboid with pubescence long and dense. Proximal three antennal flagellomeres longer than wide (0.28:0.09, 0.28:0.09, 0.27:0.09), distal antennal flagellomere longer than penultimate (0.15:0.07, 0.13:0.07), antenna longer than body (3.73, 3.23); antennal scrobes-frons shallow. Face flat or nearly so, with dense and fine punctations, interspaces wavy and longitudinal median carina present. Frons smooth. Temple wide, punctate and interspaces wavy. Inner margin of eyes diverging slightly at antennal sockets; in lateral view, eye anteriorly convex and posteriorly straight. POL shorter than OOL (0.10, 0.15). Malar suture present. Median area between lateral ocelli without depression. Vertex laterally rounded and dorsally wide.

**Mesosoma** (Fig. 98A, E, F, I). Mesosoma dorsoventrally convex. Distal 1/3 of mesoscutum with lateral margin slightly dented, punctation distinct throughout, interspaces smooth. Scutellum long and slender, apex sloped and fused with BS, scutellar punctation scattered throughout, in profile scutellum flat and on same plane as mesoscutum, phragma of the scutellum completely concealed; BS only very partially overlapping the MPM; ATS demilune with quite a little complete parallel carinae; dorsal ATS groove with semicircular/parallel carinae. Transscutal articulation with small and homogeneous foveae, area just behind transscutal articulation nearly at the same level as mesoscutum (flat), smooth and shiny. Metanotum with BM wider than PFM (clearly differentiated); MPM circular and bisected by a median longitudinal carina; AFM with a small lobe and not as well delineated as PFM; PFM thick, smooth and with lateral ends rounded; ATM proximally with a groove with some sculpturing and distally smooth. Propodeum without median longitudinal carina, proximal half curved with medium-sized sculpture and distal half with indistinct sculpture and with a shallow dent at each side of nucha; distal edge of propodeum with a flange at each side and short stubs; propodeal spiracle without distal carina; nucha surrounded by very short radiating carinae. Pronotum with a distinct dorsal furrow, dorsally with a well-defined smooth band; central area of pronotum smooth, but both dorsal and ventral furrows with short parallel carinae. Propleuron with fine punctations throughout and dorsally without a carina. Metasternum flat or nearly so. Contour of mesopleuron straight/angulate or nearly so; precoxal groove deep, smooth and shiny; epicnemial ridge elongated more fusiform (tapering at both ends).

**Legs.** Ventral margin of fore telotarsus entire without seta, fore telotarsus almost same width throughout and longer than fourth tarsomere (0.13, 0.08). Hind coxa with medium-size punctate throughout, and dorsal outer depression present. Inner spur of hind tibia longer than outer spur (0.26, 0.22), entire surface of hind tibia with dense strong spines clearly differentiated by color and length. Hind telotarsus as equal in length as fourth tarsomere (0.14, 0.14).

**Wings** (Fig. 98K). Fore wing with r vein straight; 2RS vein straight; r and 2RS veins forming an angle at their junction and outer side of junction forming a slight stub; 2M vein slightly curved/swollen; distally fore wing [where spectral veins are] with microtrichiae more densely concentrated than the rest of the wing; anal cell 1/3 proximally lacking microtrichiae; subbasal cell with microtrichiae virtually throughout; veins 2CUa and 2CUb completely spectral; vein 2 cu-a present as spectral vein, sometimes difficult to see; vein 2-1A proximally tubular and distally spectral, although sometimes difficult to see; tubular vein 1 cu-a curved and complete, but junction with 1-1A vein spectral. Hind wing with vannal lobe very narrow, subdistally evenly convex, subproximally straightened, and setae evenly scattered in the margin.

**Metasoma** (Fig. 98A, G, H, J). Metasoma laterally compressed. Petiole on T1 finely sculptured only laterally, parallel-sided in proximal half and then narrowing (length 0.45, maximum width 0.20, minimum width 0.11), and with scattered pubescence concentrated in the first distal third. Lateral grooves delimiting the median area on T2 clearly defined and reaching the distal edge of T2 (length median area 0.22, length T2 0.22), edges of median area obscured by weak longitudinal stripes, median area broader than long (length 0.22, maximum width 0.27, minimum width 0.10); T2 with scattered pubescence only distally. T3 as long as T2 (0.23, 0.22) and with scattered pubescence throughout. Pubescence on hypopygium dense.

**Cocoons.** Unknown.

#### Etymology.

Henry Hespenheide is an American entomologist and ecologist. He is interested in how many species live in a particular area, what evolutionary pressures they face and predator-prey interactions. Most current field work is in La Selva Biological Station, Costa Rica. He is a professor emeritus at University of California, Los Angeles (UCLA), CA, USA.

#### Distribution.

Parasitized caterpillar was collected in Ecuador, Napo, Yanayacu Biological Station (Sierra Azul Camino Cascadas), during December 2005 at 2,280 m in cloud forest.

#### Biology.

The lifestyle of this parasitoid species is gregarious.

#### Host.

Undetermined species of Pieridae feeding on *Inga* sp. (Fabaceae). Caterpillar was collected in second instar.

### Glyptapanteles henrytownesi

Taxon classificationAnimaliaHymenopteraBraconidae

Arias-Penna, sp. nov.

http://zoobank.org/F1BA5815-5B50-4A8A-B592-FA75E8362858

Figs 99 , 100

#### Female.

Body length 2.12 mm, antenna length 2.47 mm, fore wing length 2.53 mm.

#### Type material.

**Holotype**: COSTA RICA • 1♀; 02-SRNP-23728, DHJPAR0000027; Área de Conservación Guanacaste, Guanacaste, Sector Cacao, Sendero Toma Agua; cloud forest; 1,140 m; 10.92847, -85.46680; 16.ix.2002; Mariano Pereira leg.; caterpillar collected in third instar; a disorderly (oriented in all directions) jumbled row of light brown cocoons on each side of the caterpillar, cocoons formed on 30.ix.2002; adult parasitoids emerged on 09.x.2002; (CNC). **Paratypes.** • 44 (3♀, 4♂) (30♀, 7♂); 02-SRNP-23728, DHJPAR0000027; same data as for holotype; (CNC).

#### Other material.

**Reared material.** COSTA RICA: *Área de Conservación Guanacaste*, *Guanacaste*, *Sector Cacao*, *Sendero Toma Agua*: • 17 (4♀, 3♂) (10♀, 0♂); 98-SRNP-2191, DHJPAR0000107; cloud forest; 1,140 m; 10.92847, -85.46680; 04.ii.1998; Fredy Moraga leg.; caterpillar collected in fifth instar; golden brown tough cocoons, elongated cylinders, adhered together and to the leaf forming irregular cordwood and formed on 07.ii.1998; adult parasitoids emerged on 15.ii.1998. • 63 (3♀, 3♂) (42♀, 15♂); 98-SRNP-3335, DHJPAR0001456; same data as for preceding except: 09.viii.1998; Mariano Pereira leg.; caterpillar was collected dead; cylindrical cocoons adhered to the leaf substrate; date of cocoons not reported; adult parasitoids emerged on 25.viii.1998. • 18 (5♀, 3♂) (0♀, 10♂); 02-SRNP-23730, DHJPAR0000028; same data as for preceding except: 16.ix.2002; Mariano Pereira leg.; caterpillar collected in third instar; two somewhat sloppy rows of brown cordwood cocoons on each side of the caterpillar, cocoons formed on 30.ix.2002; adult parasitoids emerged on 08.x.2002.

*Área de Conservación Guanacaste*, *Guanacaste*, *Sector Cacao*, *Sendero Nayo*: • 51 (5♀, 5♂) (38♀, 3♂); 03-SRNP-3971, DHJPAR0000045; cloud forest; 1,090 m; 10.92446, -85.46953; 27.iv.2003; Dunia Garcia leg.; caterpillar collected in fifth instar; beige cocoons forming two rows of cordwood on each side of the caterpillar, snuggled up against both sides so that larva is in a groove between them, cocoons at right angles to the long axis of the body, cocoons formed on 30.iv.2003; adult parasitoids emerged on 09.v.2003.

*Área de Conservación Guanacaste*, *Guanacaste*, *Sector Mundo Nuevo*, *Sendero Melón*: • 14 (3♀, 3♂) (6♀, 2♂); 07-SRNP-57287, DHJPAR0020269; intergrade dry-rain forest; 361 m; 10.76820, -85.43504; 12.vi.2007; José Alberto Sanchez leg.; caterpillar collected in third instar; cream adhered together hard cocoons, jumbled on both sides of cadaver, not attached to it, cocoons formed on 23.vi.2007; adult parasitoids emerged on 27.vi.2007. • 33 (3♀, 2♂) (28♀, 0♂); 07-SRNP-57289, DHJPAR0020268; same data as for preceding except: caterpillar collected in second instar; cocoons adhered to the leaf substrate; adult parasitoids emerged on 28.vi.2007.

*Área de Conservación Guanacaste*, *Guanacaste*, *Sector Del Oro*, *Metereológico*: • 32 (3♀, 1♂) (2♀, 0♂); 10-SRNP-22294, DHJPAR0045168; intergrade dry-rain forest; 590 m; 11.00199, -85.46166; 05.x.2010; Lucia Ríos leg.; caterpillar collected in fourth instar; irregular cordwood brown cocoons adhered to the leaf substrate and formed on 13.x.2010; adult parasitoids emerged on 01.xi.2010.

*Área de Conservación Guanacaste*, *Guanacaste*, *Sector Pitilla*, *Sendero Evangelista*: • 41 (5♀, 2♂) (34♀, 0♂); 10-SRNP-30348, DHJPAR0038273; rain forest; 660 m; 10.98680, -85.42083; 14.i.2010; Petrona Rios leg.; caterpillar collected in third instar; cocoons adhered to the leaf substrate and formed on 31.i.2010; adult parasitoids emerged on 06.ii.2010. • 40 (5♀, 1♂) (34♀, 0♂); 10-SRNP-30427, DHJPAR0038288; same data as for preceding except: adult parasitoids emerged on 05.ii.2010. • 31 (5♀, 1♂) (25♀, 0♂); 10-SRNP-30428, DHJPAR0038277; same data as for preceding except: adult parasitoids emerged on 05.ii.2010. • 32 (5♀, 0♂) (27♀, 0♂); 10-SRNP-30429, DHJPAR0038279; same data as for preceding except: adult parasitoids emerged on 04.ii.2010. • 36 (5♀, 4♂) (27♀, 0♂); 10-SRNP-30430, DHJPAR0038259; same data as for preceding except: two rows of cordwood cocoons adhered to the leaf substrate. • 36 (5♀, 3♂) (28♀, 0♂); 10-SRNP-30431, DHJPAR0038285; same data as for preceding except: caterpillar collected in third instar; double cordwood cocoons adhered to the leaf substrate; adult parasitoids emerged on 05.ii.2010.

#### Malaise-trapped material.

COSTA RICA: *Área de Conservación Guanacaste*, *Alajuela*, *Sector San Cristóbal*, *Bosque Trampa Malaise*: • 1♀; 07-SRNP-67371, DHJPAR0025909; rain forest; 815 m; 10.86280, -85.38460; Malaise; 22.vii.2007; DH Janzen & W Hallwachs leg.

#### Diagnosis.

**Figure 99.**
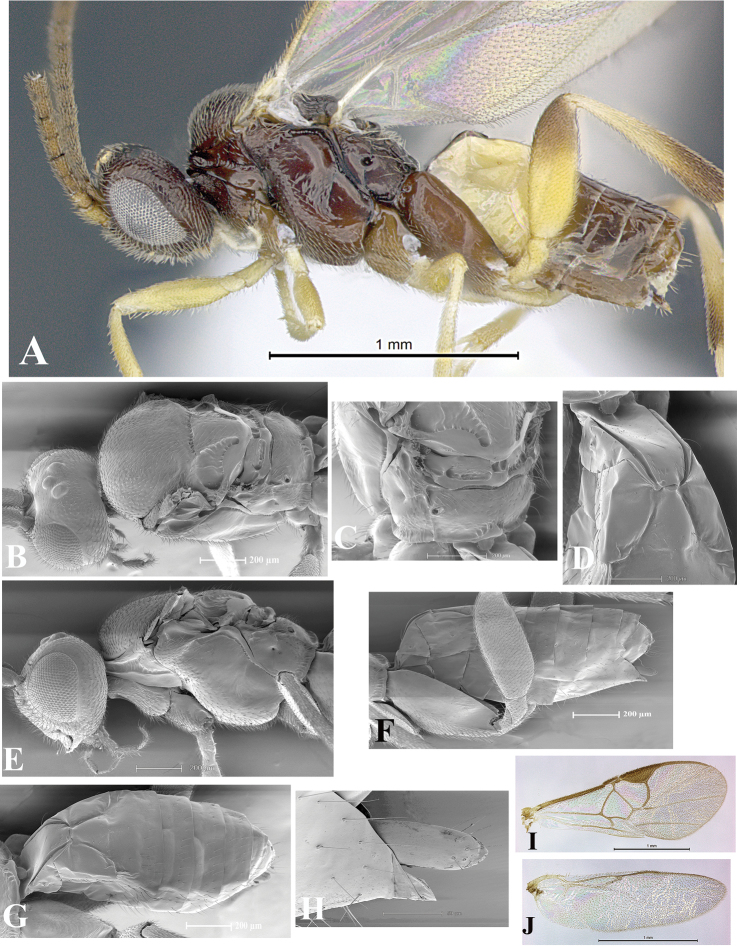
*Glyptapanteleshenrytownesi* sp. nov. female 02-SRNP-23728 DHJPAR0000027, 02-SRNP-23730 DHJPAR0000028 **A** Habitus **B, E** Head, mesosoma **B** Dorsolateral view **E** lateral view **C** Metanotum, propodeum, dorsolateral view **D**T1–2, dorsolateral view **F, G** Metasoma **F** lateral view **G** Dorsolateral view **H** Genitalia: hypopygium, ovipositor, ovipositor sheaths, lateral view **I, J** Wings **I** Fore **J** Hind.

**Figure 100.**
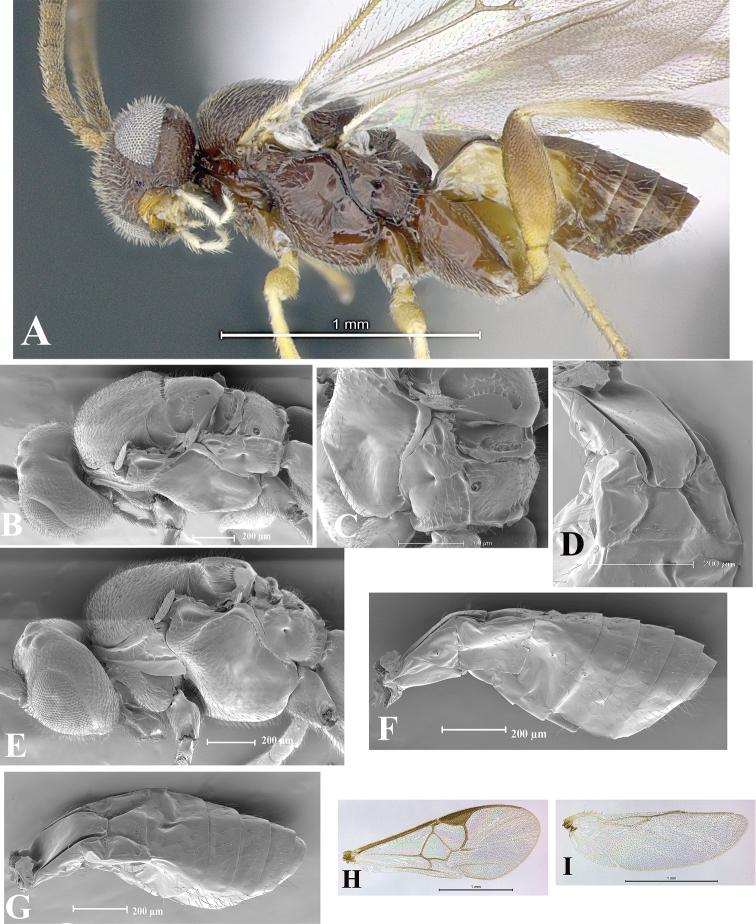
*Glyptapanteleshenrytownesi* sp. nov. male 02-SRNP-23728 DHJPAR0000027, 02-SRNP-23730 DHJPAR0000028 **A** Habitus **B, E** Head, mesosoma **B** Laterodorsal view **E** Lateral view **C** Metanotum, propodeum, laterodorsal view **D**T1–2, dorsolateral view **F, G** Metasoma **F** lateral view **G** Dorsolateral view **H, I** Wings **H** Fore **I** Hind.

Proximal half of propodeum curved, without a median longitudinal carina, propodeal spiracle without distal carina (Figs 99C, 100C), propleuron with fine rugae (Figs 99E, 100E), distal antennal flagellomere subequal in length with penultimate, mesoscutum punctation distinct throughout (Figs 99B, 100B), medioanterior pit of metanotum bisected by a median longitudinal carina (Fig. 99B, C), scutellum in profile flat and on same plane as mesoscutum (Figs 99E, 100E), phragma of the scutellum partially exposed (Figs 99C, 100C), nucha surrounded by long radiating carinae (Figs 99C, 100C), dorsal carina delimiting a dorsal furrow on propleuron present (Figs 99E, 100E), petiole on T1 parallel-sided in proximal half then narrowing (Figs 99D, 100D), precoxal groove deep (Figs 99A, 100A), anteroventral contour of mesopleuron straight/angulate or nearly so (Figs 99E, 100E), edges of median area on T2 polished and followed by a deep groove (Figs 99D, 100D), and fore wing with r vein curved, outer side of junction of r and 2RS veins forming a distinct stub (Figs 99I, 100H).

#### Coloration

(Fig. 99A). General body coloration light brown except scape, pedicel, labrum, mandibles, maxillary and labial palps, and tegulae pale yellow; all antennal flagellomeres dark brown on both sides. Eyes and ocelli silver. Fore and middle legs yellow except brown coxae; hind legs yellow except coxae, apex of femora and tibiae brown, tarsomeres brown except first tarsomere proximally yellow. Petiole on T1 brown, but medially light brown, and sublateral areas yellow; T2 with median and adjacent areas brown, and lateral ends yellow; T3 and beyond completely brown; distally each tergum with a narrow yellowish transparent band. In lateral view, T1–3 yellow; T4 and beyond completely brown. S1–3 yellow; S4 and beyond completely brown.

#### Description.

**Head** (Fig. 99A, B, E). Head triangular with pubescence short and dense. Proximal three antennal flagellomeres longer than wide (0.18:0.06, 0.19:0.06, 0.18:0.06), distal antennal flagellomere subequal in length with penultimate (0.11:0.05, 0.10:0.05), antenna longer than body (2.47, 2.12); antennal scrobes-frons shallow. Face convex, dense fine punctations, interspaces with microsculpture and longitudinal median carina present. Frons smooth. Temple wide, punctate and interspaces with microsculpture. Inner margin of eyes diverging slightly at antennal sockets; in lateral view, eye anteriorly convex and posteriorly straight. POL shorter than OOL (0.10, 0.13). Malar suture present. Median area between lateral ocelli without depression. Vertex laterally rounded and dorsally wide.

**Mesosoma** (Fig. 99A–C, E). Mesosoma dorsoventrally convex. Mesoscutum proximally convex and distally flat, punctation distinct throughout, interspaces wavy/lacunose. Scutellum triangular, apex sloped and fused with BS, scutellar punctation distinct throughout, in profile scutellum flat and on same plane as mesoscutum, phragma of the scutellum partially exposed; BS only very partially overlapping the MPM; ATS demilune with short stubs delineating the area; dorsal ATS groove smooth. Transscutal articulation with small and heterogeneous foveae, area just behind transscutal articulation with a smooth and shiny sloped transverse strip. Metanotum with BM wider than PFM (clearly differentiated); MPM circular and bisected by a median longitudinal carina; AFM without setiferous lobes and not as well delineated as PFM; PFM thick and smooth; ATM proximally with semircular/undulate carina and distally smooth. Propodeum relatively polished without median longitudinal carina, proximal half curved; distal edge of propodeum without flange; propodeal spiracle without distal carina; nucha surrounded by very short radiating carinae. Pronotum with a distinct dorsal furrow, dorsally with a well-defined smooth band; central area of pronotum smooth, but both dorsal and ventral furrows with short parallel carinae. Propleuron with fine rugae and dorsally wit h a carina. Metasternum flat or nearly so. Contour of mesopleuron straight/angulate or nearly so; precoxal groove deep with faintly transverse lineate sculpture; epicnemial ridge convex, teardrop-shaped.

**Legs.** Ventral margin of fore telotarsus entire, but with a tiny curved seta, fore telotarsus almost same width throughout and longer than fourth tarsomere (0.20, 0.06). Hind coxa with punctation only on ventral surface and dorsal outer depression present. Inner spur of hind tibia longer than outer spur (0.25, 0.16), entire surface of hind tibia with dense strong spines clearly differentiated by color and length. Hind telotarsus as equal in length as fourth tarsomere (0.11, 0.10).

**Wings** (Fig. 99I, J). Fore wing with r vein slightly curved; 2RS vein straight; r and 2RS veins forming an angle at their junction and outer side of junction not forming a stub; 2M vein slightly curved/swollen; distally fore wing [where spectral veins are] with microtrichiae more densely concentrated than the rest of the wing; anal cell 1/3 proximally lacking microtrichiae; subbasal cell with a small smooth area; vein 2CUa absent and vein 2CUb spectral; vein 2 cu-a absent; vein 2-1A proximally tubular and distally spectral, although sometimes difficult to see; tubular vein 1 cu-a curved, incomplete/broken, not reaching the edge of 1-1A vein. Hind wing with vannal lobe wide, subbassally evenly convex and subdistally straightened, and setae present only proximally.

**Metasoma** (Fig. 99A, D, F–H). Metasoma laterally compressed. Petiole on T1 completely smooth and polished, with faint, satin-like sheen, parallel-sided in proximal half and then narrowing (length 0.27, maximum width 0.15, minimum width 0.08), and with scattered pubescence and concentrated in the first distal third. Lateral grooves delimiting the median area on T2 clearly defined and reaching the distal edge of T2 (length median area 0.16, length T2 0.16), edges of median area polished and lateral grooves deep, median area broader than long (length 0.16, maximum width 0.18, minimum width 0.05 mm); T2 with scattered pubescence only distally. T3 longer than T2 (0.23, 0.16) and with pubescence more notorious in distal half. Pubescence on hypopygium dense.

**Cocoons** (Fig. 4S). Brown oval cocoons with ordered silk fibers, but covered by a net. Cocoons disorderly, irregular and oriented in all directions forming two rows of cordwood and located on each side of the caterpillar.

#### Comments.

In some specimens, the general body coloration is polished and black instead of light brown and in lateral view the T3 is brown only ventrally.

#### Male

(Fig. 100A–I). Similar in coloration and shape to female.

#### Etymology.

Henry Keith Townes Jr. (20 January 1913-2 May 1990) was widely known for his work on hymenopteran systematics, particularly the large and difficult family Ichneumonidae.

#### Distribution.

Parasitized caterpillars were collected in Costa Rica, ACG, Sector Cacao (Sendero Nayo and Sendero Toma Agua), Sector Del Oro (Metereológico), Sector Mundo Nuevo (Sendero Melón), and Sector Pitilla (Sendero Evangelista), during February and August 1998, September 2002;,April 2003, June 2007, and January and October 2010 at 361 m, 590 m, 660 m, 1,090 m, and 1,140 m in intergrade dry-rain, rain and cloud forests. The adult parasitoid was collected in Costa Rica, ACG, Sector San Cristóbal (Bosque Trampa Malaise), during July 2007 at 815 m in rain forest.

#### Biology.

The lifestyle of this parasitoid species is gregarious.

#### Host.

*Heterochromasarepta* (Druce) (Noctuidae: Amphipyrinae) (Fig. 4S) feeding on *Smilaxmollis* and *S.spinosa* (Smilacaceae). Caterpillars were collected in second, third, fourth and fifth instar.

### Glyptapanteles howelldalyi

Taxon classificationAnimaliaHymenopteraBraconidae

Arias-Penna, sp. nov.

http://zoobank.org/AC4249AF-8AD9-4A20-BDCE-8C1FA4205D3F

Figs 101 , 102

#### Female.

Body length 2.68 mm, antenna length 2.63 mm, fore wing length 2.53 mm.

#### Type material.

**Holotype**: COSTA RICA • 1♀; 08-SRNP-72188, DHJPAR0031040; Área de Conservación Guanacaste, Alajuela, Sector Pitilla, Medrano; rain forest; 380 m; 11.01602, -85.38053; 26.viii.2008; Walter Siezar leg.; caterpillar collected in fourth instar; two rows of cordwood cocoons adhered to larva and substrate, cocoons formed on 02.ix.2008; adult parasitoids emerged on 09.ix.2008; (CNC). **Paratypes.** • 69 (1♀, 2♂) (37♀, 29♂); 08-SRNP-72188, DHJPAR0031040; same data as for holotype; (CNC).

#### Other material.

**Reared material.** COSTA RICA: *Área de Conservación Guanacaste*, *Alajuela*, *Sector San Cristóbal*, *Potrero Argentina*: • 59 (3♀, 3♂) (44♀, 9♂); 03-SRNP-9142, DHJPAR0000048; pastures; 520 m; 10.89021, -85.38803; 11.x.2003; Elda Araya leg.; caterpillar collected in fourth instar; mass of brown cocoons tightly glued to each other and to the leaf, forming two rows of cordwood, a bit irregular, caterpillar must has been on the side; adult parasitoids emerged on 14.x.2003.

*Área de Conservación Guanacaste*, *Alajuela*, *Sector San Cristóbal*, *Estación San Cristobal*: • 38 (3♀, 3♂) (27♀, 5♂); 04-SRNP-3377, DHJPAR0000284; rain forest; 640 m; 10.87097, -85.39144; 10.vii.2004; Elda Araya leg.; caterpillar collected in fourth instar; two rows of cordwood cocoons adhered to the leaf substrate; adult parasitoids emerged on 17.vii.2004.

*Área de Conservación Guanacaste*, *Alajuela*, *Sector San Cristóbal*, *Corrales viejos*: • 78 (3♀, 3♂) (40♀, 32♂); 04-SRNP-4458, DHJPAR0000277; rain forest; 495 m; 10.89974, -85.38085; 31.viii.2004; Osvaldo Espinoza leg.; caterpillar collected in fifth instar; two rows of cordwood cocoons adhered to the leaf substrate; adult parasitoids emerged on 12.ix.2004.

*Área de Conservación Guanacaste*, *Alajuela*, *Sector San Cristóbal*, *Quebrada Cementerio*: • 38 (3♀, 3♂) (23♀, 9♂); 06-SRNP-5536, DHJPAR0012096; rain forest; 700 m; 10.87124, -85.38749; 08.vii.2006; Elda Araya leg.; caterpillar collected in fourth instar; two somewhat irregular rows of cordwood on each side of the cadaver, cocoons form on 19.vii.2006; adult parasitoids emerged on 26.vii.2006.

*Alajuela*, *Dos Ríos*, *Sector San Cristóbal*, *Finca San Gabriel*: • 51 (4♀, 3♂) (32♀, 12♂); 03-SRNP-34135, DHJPAR0000275, DHJPAR0001529; rain forest; 645 m; 10.87766, -85.39343; 10.xi.2003; Carolina Cano leg.; caterpillar collected in fifth instar; very dense two rows of cordwood brown cocoons, but not on each side of the caterpillar but along the midrib of the leaf; adult parasitoids emerged on 18.xi.2003. • 24 (3♀, 3♂) (15, 3♂); 04-SRNP-4174, DHJPAR0001479; same data as for preceding except: 20.viii.2004; Yessenia Mendoza leg.; caterpillar collected in third instar; cocoons adhered to the leaf substrate; adult parasitoid emerged on 14.ix.2004. • 26 (3♀, 3♂) (19♀, 1♂); 07-SRNP-2795, DHJPAR0020267; same data as for preceding except: 20.vi.2007, Minor Carmona leg.; caterpillar collected in third instar; cocoons adhered to the leaf substrate; adult parasitoid emerged on 06.vii.2007.

*Área de Conservación Guanacaste*, *Alajuela*, *Sector San Cristóbal*, *Río Blanco Abajo*: • 103 (3♀, 3♂) (89♀, 8♂); 04-SRNP-1182, DHJPAR0020497; rain forest; 500 m; 10.90037, -85.37254; 04.iv.2004; Osvaldo Espinoza leg.; caterpillar collected in fifth instar; two rows of cordwood brown cocoons adhered to the leaf substrate; adult parasitoids emerged on 09.iv.2004. • 56 (3♀, 3♂) (27♀, 23♂); 06-SRNP-5231, DHJPAR0012009; same data as for preceding except: 28.vi.2006; Anabelle Córdoba leg.; caterpillar collected in fourth instar; cocoons adhered to the leaf substrate, cocoon characteristics not reported; adult parasitoids emerged on 12.vii.2006. • 45 (3♀, 3♂) (34♀, 5♂); 06-SRNP-5232, DHJPAR0012012; same data as for preceding except: 28.vi.2006; Anabelle Córdoba leg.; caterpillar collected in fourth instar; adult parasitoids emerged on 11.vii.2006.

*Área de Conservación Guanacaste*, *Alajuela*, *Sector San Cristóbal*, *Puente Palma*: • 21 (3♀, 0♂) (18♀, 0♂); 03-SRNP-9723, DHJPAR0000274; rain forest; 460 m; 10.9163, -85.37869; 30.x.2003; Gloria Sihezar leg.; caterpillar collected in fourth instar; row of brown cordwood cocoons on each side of the cadaver; adult parasitoids emerged on 08.xi.2003. • 84 (3♀, 3♂) (74♀, 4♂); 04-SRNP-4137, DHJPAR0001466; same data as for preceding except: 19.viii.2004; Yessenia Mendoza leg.; caterpillar collected in third instar; two rows of stacked cordwood cocoons adhered to the leaf substrate; adult parasitoids on 02.ix.2004. • 59 (3♀, 3♂) (49♀, 4♂); 04-SRNP-4138, DHJPAR0001461; same data as for preceding except: 19.viii.2004; Yessenia Mendoza leg.; caterpillar collected in third instar; two rows of cordwood cocoons adhered to the leaf substrate; adult parasitoids on 02.ix.2004. • 104 (3♀, 3♂) (90♀, 8♂); 04-SRNP-4139, DHJPAR0000282; same data as for preceding except: 19.viii.2004; Yessenia Mendoza leg.; caterpillar collected in third instar; two rows of stacked cordwood adhered to the leaf substrate; adult parasitoids emerged on 02.ix.2004. • 54 (3♀, 3♂) (41♀, 7♂); 04-SRNP-4801, DHJPAR0001478; same data as for preceding except: 24.ix.2004; Osvaldo Espinoza leg.; caterpillar collected in third instar; two rows of cordwood cocoons adhered to the leaf substrate; adult parasitoids emerged on 13.x.2004. • 66 (3♀, 3♂) (51♀, 9♂); 04-SRNP-4804, DHJPAR0001469; same data as for preceding except: 24.ix.2004; Osvaldo Espinoza leg.; caterpillar collected in third instar; two rows of brown cordwood cocoons adhered to the leaf substrate; adult parasitoids emerged on 09.x.2004. • 63 (3♀, 3♂) (53♀, 4♂); 04-SRNP-4814, DHJPAR0000279; same data as for preceding except: 24.ix.2004; Osvaldo Espinoza leg.; caterpillar collected in third instar; cordwood cocoons on both sides of cadaver, cocoons adhered to the leaf substrate; adult parasitoids emerged on 09.x.2004. • 47 (3♀, 3♂) (37♀, 4♂); 05-SRNP-6986, DHJPAR0004772; same data as for preceding except: 06.xi.2005; Yessenia Mendoza leg.; two rows of cordwood cocoons adhered to the leaf substrate; adult parasitoids emerged on 20.xi.2005. • 41 (3♀, 3♂) (30♀, 5♂); 05-SRNP-6987, DHJPAR0004778; same data as for preceding except: 06.xi.2005; Yessenia Mendoza leg.; two rows of cordwood cocoons adhered to the leaf substrate; adult parasitoids emerged on 20.xi.2005.

*Área de Conservación Guanacaste*, *Alajuela*, *Sector San Cristóbal*, *Vado Río Cucaracho*: • 24 (3♀, 3♂) (15♀, 3♂); 99-SRNP-5745, DHJPAR0001520; rain forest; 640 m; 10.8702, -85.39153; 08.vi.1999; Gloria Sihezar leg.; caterpillar collected in fourth instar; gray cordwood cocoons adhered to the leaf substrate; adult parasitoids emerged on 12.vi.1999. • 52 (3♀, 3♂) (46♀, 0♂); 06-SRNP-5588, DHJPAR0012117; same data as for preceding except: 11.vii.2006; cocoons adhered to the leaf substrate; adult parasitoids emerged on 24.vii.2006. • 60 (3♀, 3♂)(36♀, 18♂); 06-SRNP-5589, DHJPAR0012115; 11.vii.2006; cocoon characteristics not reported; adult parasitoids emerged on 22.vii.2006. • 42 (3♀, 2♂) (37♀, 0♂); 06-SRNP-5590, DHJPAR0012109; same data as for preceding except: 11.vii.2006; caterpillar collected in third instar; two rows of cordwood cocoons with caterpillar in between; adult parasitoids emerged on 30.vii.2006. • 24 (3♀, 3♂) (3♀, 15♂); 06-SRNP-5591, DHJPAR0012097; same data as for preceding except: 11.vii.2006; caterpillar collected in third instar; larval caterpillar is still alive between the two rows of cordwood cocoons; adult parasitoids emerged on 28.vii.2006. • 60 (3♀, 3♂) (42♀, 12♂); 06-SRNP-5592, DHJPAR0012116; same data as for preceding except: 11.vii.2006; caterpillar collected in third instar; brown cocoons arranged in two rows of cordwood; adult parasitoids emerged on 31.vii.2006.

*Área de Conservación Guanacaste*, *Guanacaste*, *Sector Cacao*, *Puente Gongora*: • 47 (3♀, 2♂) (42♀, 0♂); 06-SRNP-46302, DHJPAR0012108; cloud forest; 540 m; 10.88489, -85.47203; 23.vii.2006; Harry Ramirez leg.; caterpillar collected in fifth instar; brown cordwood cocoons adhered to the leaf substrate, cocoons formed on 27.vii.2006; adult parasitoids emerged on 03.viii.2006.

*Área de Conservación Guanacaste*, *Guanacaste*, *Sector Pitilla*, *Coneja*: • 12 (3♀, 2♂) (7♀, 0♂); 08-SRNP-32254, DHJPAR0031042; rain forest; 415 m; 11.01525, -85.39766; 08.ix.2008; Manuel Rios leg.; caterpillar collected in fifth instar, cocoons already present and adhered to the leaf substrate; adult parasitoids emerged on 15.ix.2008. • 59 (3♀, 3♂) (59♀, 4♂); 08-SRNP-32255, DHJPAR0031039; same data as for preceding except: Calixto Moraga leg.; caterpillar collected in fourth instar, two rows of cordwood cocoons already present and adhered to the leaf substrate; adult parasitoids emerged on 16.ix.2008.

*Área de Conservación Guanacaste*, *Guanacaste*, *Sector Pitilla*, *Estación Quica*: • 104 (3♀, 3♂) (38♀, 60♂); 08-SRNP-71793, DHJPAR0031105; rain forest; 470 m; 10.99697, -85.39666; 21.vii.2008; Leonel Siezar leg.; caterpillar collected in fifth instar; two rows of brown cordwood cocoons already present and adhered to the leaf substrate; adult parasitoids emerged on 26.vii.2008. • 97 (3♀, 3♂) (33♀, 58♂); 08-SRNP-71803, DHJPAR0031096; same data as for preceding except: 25.vii.2008; caterpillar collected in fourth instar; brown cordwood cocoons adhered to the leaf substrate and formed on 03.viii.208; adult parasitoids emerged on 11.viii.2008. • 96 (3♀, 3♂) (74♀, 16♂); 08-SRNP-72135, DHJPAR0031033; same data as for preceding except: 25.viii.2008; double cordwood cocoons adhered to the leaf substrate and formed on 02.ix.2008; adult parasitoids emerged on 08.ix.2008. • 63 (3♀, 3♂) (27♀, 30♂); 08-SRNP-72136, DHJPAR0031027; same data as for preceding except: 25.viii.2008; Ronald Siezar leg.; double cordwood cocoons adhered to the leaf substrate and formed on 05.ix.2008; adult parasitoids emerged on 11.ix.2008. • 64 (3♀, 3♂) (54♀, 4♂); 08-SRNP-72138, DHJPAR0031026; same data as for preceding except: 25.viii.2008; Marta Acosta leg.; two rows of brown cordwood cocoons adhered to the leaf substrate and formed on 26.viii.2008; adult parasitoids emerged on 02.ix.2008. • 135 (3♀, 3♂) (109♀, 20♂); 08-SRNP-72383, DHJPAR0031030; same data as for preceding except: 13.ix.2008; Walter Siezar leg.; cocoons adhered to the leaf substrate and formed on 17.ix.2008; cocoon characteristics not reported; adult parasitoids emerged on 25.ix.2008. • 121 (3♀, 3♂) (96♀, 59♂); 08-SRNP-72384, DHJPAR0031041; same data as for preceding except: 13.ix.2008; two rows of cordwood cocoons adhered to the leaf substrate and formed on 17.ix.2008; adult parasitoids emerged on 23.ix.2008. • 106 (3♀, 3♂) (27♀, 73♂); 08-SRNP-72385, DHJPAR0031034; same data as for preceding except: 13.ix.2008; two rows of cordwood cocoons adhered to the leaf substrate and formed on 18.ix.2008; adult parasitoids emerged on 24.ix.2008. • 134 (3♀, 3♂) (65♀, 62♂); 08-SRNP-72386, DHJPAR0031021; same data as for preceding except: 13.ix.2008; Marta Acosta leg.; two rows of cordwood cocoons adhered to the leaf substrate and formed on 17.ix.2008; adult parasitoids emerged on 23.ix.2008. • 48 (3♀, 3♂) (39♀, 3♂); 09-SRNP-70451, DHJPAR0035403; same data as for preceding except: 19.v.2009; Calixto Moraga leg.; mass of very ordered cocoons on leaf below alive caterpillar; adult parasitoids emerged on 25.v.2009. • 56 (5♀, 5♂) (42♀, 4♂); 09-SRNP-70452, DHJPAR0035427; same data as for preceding except: 19.v.2009; Ricardo Calero leg.; row of cordwood cocoons on each side of alive caterpillar; adult parasitoids emerged on 24.v.2009.

*Área de Conservación Guanacaste*, *Guanacaste*, *Sector Pitilla*, *Medrano*: • 19 (3♀, 3♂) (13♀, 0♂); 08-SRNP-72115, DHJPAR0031020; rain forest; 380 m; 11.01602, -85.38053; 20.viii.2008; Leonel Siezar leg.; caterpillar collected in fourth instar; cocoons adhered to the leaf substrate and formed on 29.viii.2008; adult parasitoids emerged on 06.ix.2008. • 100 (3♀, 3♂) (89♀, 5♂); 08-SRNP-72116, DHJPAR0031035; same data as for preceding except: Marta Acosta leg.; two rows of cordwood cocoons adhered to the leaf substrate and formed on 02.ix.2008; adult parasitoids emerged on 09.ix.2008. • 68 (3♀, 3♂) (60♀, 2♂); 08-SRNP-72120, DHJPAR0031036; same data as for preceding except: Marta Acosta leg.; two rows of brown cordwood cocoons; adult parasitoids emerged on 05.ix.2008. • 48 (3♀, 3♂) (38♀, 4♂); 08-SRNP-72184, DHJPAR0031038; same data as for preceding except: 26.viii.2008; Walter Siezar leg.; two rows of cordwood cocoons adhered to the leaf substrate and formed on 02.ix.2008. • 88 (3♀, 3♂) (58♀, 24♂); 08-SRNP-72185, DHJPAR0031031; same data as for preceding except: 26.viii.2008; Walter Siezar leg.; two rows of cordwood cocoons adhered to the leaf substrate and formed on 02.ix.2008; adult parasitoids emerged on 08.ix.2008. • 85 (3♀, 3♂) (67♀, 12♂); 08-SRNP-72186, DHJPAR0031028; same data as for preceding except: 26.viii.2008; Walter Siezar leg.; two rows of cordwood cocoons adhered to the leaf substrate and formed on 02.ix.2008; adult parasitoids emerged on 07.ix.2008. • 27 (3♀, 3♂) (7♀, 14♂); 08-SRNP-72187, DHJPAR0031043; same data as for preceding except: 26.viii.2008; Walter Siezar leg.; two rows of cordwood cocoons adhered to the leaf substrate and formed on 06.ix.2008; adult parasitoids emerged on 10.ix.2008. • 78 (3♀, 3♂) (46♀, 26♂); 08-SRNP-72189, DHJPAR0031024; same data as for preceding except: 26.viii.2008; Walter Siezar leg.; double cordwood cocoons adhered to the leaf substrate and formed on 02.ix.2008; adult parasitoids emerged on 07.ix.2008. • 109 (3♀, 3♂) (95♀, 18♂); 08-SRNP-72190, DHJPAR0031025; same data as for preceding except: 26.viii.2008; Walter Siezar; two rows of cordwood cocoons adhered to the leaf substrate and formed on 02.ix.2008; adult parasitoids emerged on 08.ix.2008. • 75 (3♀, 3♂) (45♀, 24♂); 08-SRNP-72192, DHJPAR0031022; same data as for preceding except: 26.viii.2008; Walter Siezar; caterpillar collected in third instar; two rows of cordwood cocoons adhered to the leaf substrate and formed on 10.ix.2008; adult parasitoids emerged on 15.ix.2008. • 104 (3♀, 3♂) (28♀, 70♂); 08-SRNP-72194, DHJPAR0031032; same data as for preceding except: 26.viii.2008; Walter Siezar leg.; caterpillar collected in third instar; two rows of cordwood cocoons adhered to the leaf substrate and formed on 10.ix.2008; adult parasitoids emerged on 15.ix.2008.

#### Diagnosis.

**Figure 101.**
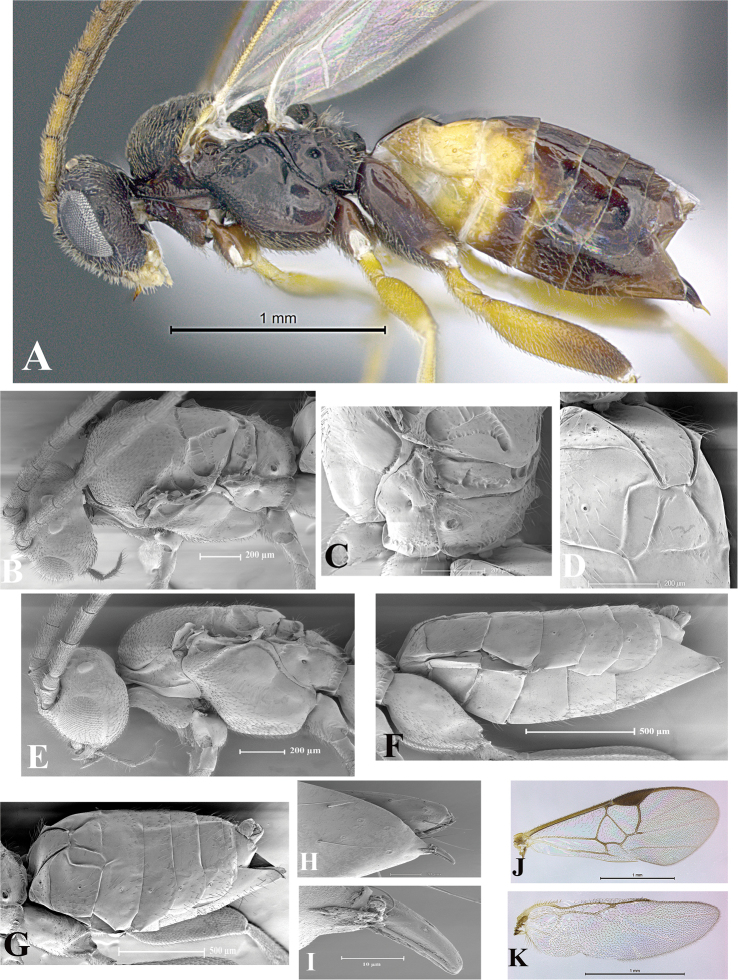
*Glyptapanteleshowelldalyi* sp. nov. female 99-SRNP-5745 DHJPAR0001520, 08-SRNP-72188 DHJPAR0031040 **A** Habitus **B, E** Head, mesosoma **B** Dorsolateral view **E** lateral view **C** Metanotum, propodeum, dorsolateral view **D**T1–2, dorsolateral view **F, G** Metasoma **F** lateral view **G** Dorsolateral view **H, I** Genitalia: hypopygium, ovipositor, ovipositor sheaths, lateral view **J, K** Wings **J** Fore **K** Hind.

**Figure 102.**
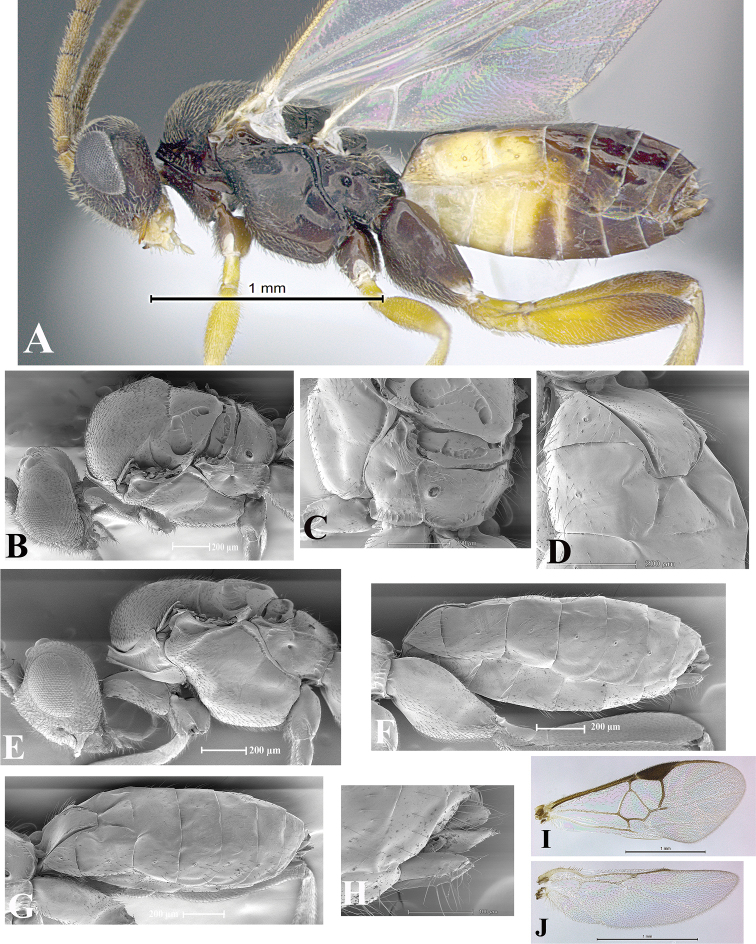
*Glyptapanteleshowelldalyi* sp. nov. male 99-SRNP-5745 DHJPAR0001520, 08-SRNP-72188 DHJPAR0031040 **A** Habitus **B, E** Head, mesosoma **B** Dorsolateral view **E** Lateral view **C** Metanotum, propodeum, laterodorsal view **D**T1–2, laterodorsal view **F, G** Metasoma **F** Lateral view **G** Dorsolateral view **H** Genitalia: parameres, lateral view **I, J** Wings **I** Fore **J** Hind.

Mesoscutum punctate throughout (Figs 101B, 102B), phragma of the scutellum partially exposed (Figs 101C, 102C), ntenna shorter than body, ventral margin of fore telotarsus slightly excavated, scutellar punctation indistinct throughout (Figs 101B, 102B), propodeal spiracle without distal carina (Figs 101C, 102C), petiole on T1 distally with lateral margins relatively straight, finely sculptured only laterally (Figs 101D, G, 102D, G), surface of metasternum flat or nearly so, precoxal groove deep with lineate sculpture (Figs 101A, E, 102A, E), fore wing with vein 1 cu-a curved, r vein curved, outer side of junction of r and 2RS veins not forming a stub (Figs 101J, 102I), dorsal outer depression on hind coxa present (Figs 101A, F, 102A, F), inner margin of eyes diverging slightly at antennal sockets, propodeum without median longitudinal carina (Figs 101C, 102C), lateral grooves delimiting the median area on T2 clearly defined and reaching the distal edge of T2 (Figs 101D, G, 102D, G).

#### Coloration

(Fig. 101A). General body coloration polished brown-black except labrum and mandibles yellow-brown; glossa, maxillary and labial palps and tegulae yellow; scape and pedicel yellow, but laterally brown; three most proximal antennal flagellomeres dorsally lighter (yellow-brown) than ventrally (dark brown), remaining flagellomeres dark brown on both sides. Eyes and ocelli silver. Fore and middle legs yellow except coxae and claws brown-black; hind legs yellow except coxae, apex of femora and most part of the tibiae brown-black, tarsomeres brown except proximal half of basitarsus. Petiole on T1 black, but middle yellow and sublateral areas yellow; T2 with median and very narrow adjacent areas black and lateral ends yellow; T3 black, but proximal corners yellow and distal corners with one oval yellow spot at each side; T3 and beyond brown; distally each tergum with a narrow transparent band. In lateral view, T1–2 completely yellow; T3 yellow, dorsally dark brown; T4 and beyond completely brown. S1–2 completely yellow; S3 yellow distally with a narrow brown band; S4 proximally with a narrow yellow band, distally with a wide brown band; penultimalte sternum and hypopygium completely brown.

#### Description.

**Head** (Fig. 101A, B, E). Head triangular with pubescence short and dense. Proximal three antennal flagellomeres longer than wide (0.21:0.06, 0.20:0.06, 0.20:0.06), distal antennal flagellomere longer than penultimate (0.13:0.06, 0.09:0.06), antenna shorter than body (2.63, 2.68); antennal scrobes-frons shallow. Face flat or nearly so, with dense fine punctations, interspaces wavy and longitudinal median carina present. Frons smooth. Temple wide, punctate and interspaces wavy. Inner margin of eyes diverging slightly at antennal sockets; in lateral view, eye anteriorly convex and posteriorly straight. POL shorter than OOL (0.10, 0.13). Malar suture present. Median area between lateral ocelli without depression. Vertex laterally rounded and dorsally wide.

**Mesosoma** (Fig. 101A–C, E). Mesosoma dorsoventrally convex. Distal 1/3 of mesoscutum with lateral margin slightly dented, punctation distinct throughout, interspaces wavy/lacunose. Scutellum triangular, apex sloped and fused with BS, scutellar punctation indistinct throughout, in profile scutellum flat and on same plane as mesoscutum, phragma of the scutellum partially exposed; BS only very partially overlapping the MPM; ATS demilune with short stubs delineating the area; dorsal ATS groove with semicircular/parallel carinae. Transscutal articulation with small and heterogeneous foveae, area just behind transscutal articulation smooth, shiny and depressed centrally. Metanotum with BM wider than PFM (clearly differentiated); MPM circular and bisected by a median longitudinal carina; AFM without setiferous lobes and not as well delineated as PFM; PFM thick and smooth; ATM proximally with semircular/undulate carina and distally smooth. Propodeum without median longitudinal carina, proximal half curved and relatively polished and distal half relatively polished; distal edge of propodeum with a flange at each side and without stubs; propodeal spiracle without distal carina; nucha surrounded by very short radiating carinae. Pronotum with a distinct dorsal furrow, dorsally with a well-defined smooth band; central area of pronotum smooth, but both dorsal and ventral furrows with short parallel carinae. Propleuron with a mix of rugae and fine punctation, dorsally with a carina. Metasternum flat or nearly so. Contour of mesopleuron straight/angulate or nearly so; precoxal groove deep with faintly lineate sculpture; epicnemial ridge elongated more fusiform (tapering at both ends).

**Legs.** Ventral margin of fore telotarsus slightly excavated and with a tiny curved seta, fore telotarsus almost same width throughout and longer than fourth tarsomere (0.12, 0.08). Hind coxa with punctation only on ventral surface and dorsal outer depression present. Inner spur of hind tibia longer than outer spur (0.26, 0.19), entire surface of hind tibia with dense strong spines clearly differentiated by color and length. Hind telotarsus longer than fourth tarsomere (0.16, 0.11).

**Wings** (Fig. 101J, K). Fore wing with r vein slightly curved; 2RS vein slightly convex to convex; r and 2RS veins forming a weak, even curve at their junction and outer side of junction not forming a stub; 2M vein slightly curved/swollen; distally fore wing [where spectral veins are] with microtrichiae more densely concentrated than the rest of the wing; anal cell 1/3 proximally lacking microtrichiae; subbasal cell with a small smooth area; vein 2CUa absent and 2CUb spectral; vein 2 cu-a absent; vein 2-1A present only proximally as tubular vein; tubular vein 1 cu-a curved and complete, but junction with 1-1A vein spectral. Hind wing with vannal lobe narrow, subdistally and subproximally straightened, and setae present only proximally.

**Metasoma** (Fig. 101A, D, F–I). Metasoma laterally compressed. Petiole on T1 finely sculptured only laterally, virtually parallel-sided over most of length, but narrowing over distal 1/3 (length 0.35, maximum width 0.21, minimum width 0.10), and with scattered pubescence on distal half only laterally. Lateral grooves delimiting the median area on T2 clearly defined and reaching the distal edge of T2 (length median area 0.18, length T2 0.18), edges of median area polished and lateral grooves deep, median area broader than long (length 0.18, maximum width 0.25, minimum width 0.08); T2 with scattered pubescence only distally. T3 longer than T2 (0.24, 0.18) and with scattered pubescence throughout. Pubescence on hypopygium dense.

**Cocoons** (Fig. 4AB). Brown oval cocoons with ordered silk fibers, but covered by a net. Two rows of cordwood cocoons on each side of living caterpillar and attached to cuticle.

#### Male

(Fig. 102A–J). Similar in coloration and shape to female.

#### Etymology.

Howell Vann Daly Jr. (30 Oct 1933-27 Aug 2018) was a professor emeritus at the department of Environmental Science, Policy, & Management, University of California, Berkeley, CA, USA. His career was focused on biosystematics of bees, using traditional and modern taxonomic procedures, including the use of computers in classification, data analysis, and management.

#### Distribution.

Parasitized caterpillars were collected in Costa Rica, ACG, Sector Cacao (Puente Gongora), Sector Pitilla (Coneja, Estación Quica, and Medrano), and Sector San Cristóbal (Corrales viejos, Estación San Cristobal, Finca San Gabriel, Potrero Argentina, Puente Palma, Quebrada Cementerio, Río Blanco Abajo, and Vado Río Cucaracho), during June 1999, October-November 2003, April, July-August, and September 2004, November 2005, June-July 2006, June 2007, July-September 2008, and May 2009 at 380 m, 415 m, 460 m, 470 m, 495 m, 500 m, 520 m, 540 m, 640 m, 645 m, and 700 m in rain and cloud forests.

#### Biology.

The lifestyle of this parasitoid species is gregarious.

#### Host.

*Dyopschromatophila* Walker (Noctuidae: Catocalinae) (Fig. 4AB) feeding on *Coussapoanymphaeifolia* and *Cecropiapeltata* (Urticaceae). Caterpillars were collected in third, fourth, and fifth instar.

### Glyptapanteles hugokonsi

Taxon classificationAnimaliaHymenopteraBraconidae

Arias-Penna, sp. nov.

http://zoobank.org/36C84B84-3238-47F1-A201-4BC135FF6BE6

Figs 103 , 104

#### Female.

Body length 2.37 mm, antenna length 2.70 mm, fore wing length 2.53 mm.

#### Type material.

**Holotype**: COSTA RICA • 1♀; 04-SRNP-2868, DHJPAR0001496; Área de Conservación Guanacaste, Alajuela, Sector San Cristóbal, Potrero Argentina; pastures; 520 m; 10.89021, -85.38803; 21.vi.2004; Carolina Cano leg.; caterpillar collected in fourth instar; brown or discolored cocoons stuffed in among the setae along the back of caterpillar, cocoons formed on 12.vii.2004; adult parasitoids emerged on 12.vii.2004; (CNC). **Paratypes.** • 94 (4♀, 5♂) (83♀, 2♂); 04-SRNP-2868, DHJPAR0001496; same data as for holotype; (CNC).

#### Other material.

**Reared material.** COSTA RICA: *Área de Conservación Guanacaste*, *Alajuela*, *Sector San Cristóbal*, *Potrero Argentina*: • 20 (4♀, 4♂) (14♀, 0♂); 04-SRNP-2869, DHJPAR0001472; pastures; 520 m; 10.89021, -85.38803; 21.vi.2004; Carolina Cano leg.; caterpillar collected in third instar; cocoons formed on 28.vi.2004; adult parasitoids emerged on 07.vii.2004.

*Área de Conservación Guanacaste*, *Alajuela*, *Sector Rincón Rain Forest*, *Flecha*: • 34 (3♀, 3♂) (28♀, 0♂); 09-SRNP-69395, DHJPAR0039964; 491 m; 10.94741, -85.31501; 25.vi.2009; Noé Castillo leg.; caterpillar collected in third instar; cocoons all over the back of the cadaver rotted of caterpillar, cocoons formed on 05.vii.2009; adult parasitoids emerged on 12.vii.2009.

*Área de Conservación Guanacaste*, *Alajuela*, *Sector Brasilia*, *Moga*: • 65 (3♀, 3♂) (57♀, 2♂); 11-SRNP-65725, DHJPAR0045133; rain forest; 320 m; 11.01227, -85.34929; 26.vii.2011; Duvalier Briceño leg.; caterpillar collected in fourth instar; cocoons formed on 06.viii.2011; adult parasitoids emerged on 09.viii.2011.

#### Malaise-trapped material.

COSTA RICA: *Área de Conservación Guanacaste*, *Alajuela*, *Sector San Cristóbal*, *Río Blanco Abajo*: • 1 (1♀, 0♂) (0♀, 0♂); 08-SRNP-3656, DHJPAR0027237; Malaise trap; rain forest; 500 m; 10.90037, -85.37254; 30.iv.2008; DH Janzen & W Hallwachs leg.

#### Diagnosis.

**Figure 103.**
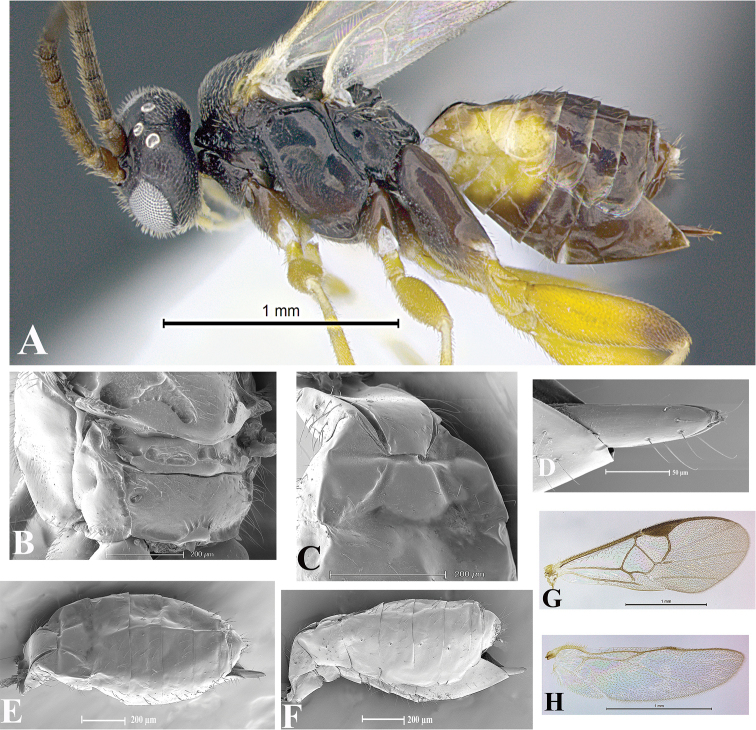
*Glyptapanteleshugokonsi* sp. nov. female 04-SRNP-2868 DHJPAR0001496 **A** Habitus **B** Scutellum, metanotum, propodeum, dorsolateral view **C**T1–3, dorsal view **D** Genitalia: hypopygium, ovipositor, ovipositor sheaths, lateral view **E, F** Metasoma **E** Dorsolateral view **F** Lateral view **G, H** Wings **G** Fore **H** Hind.

**Figure 104.**
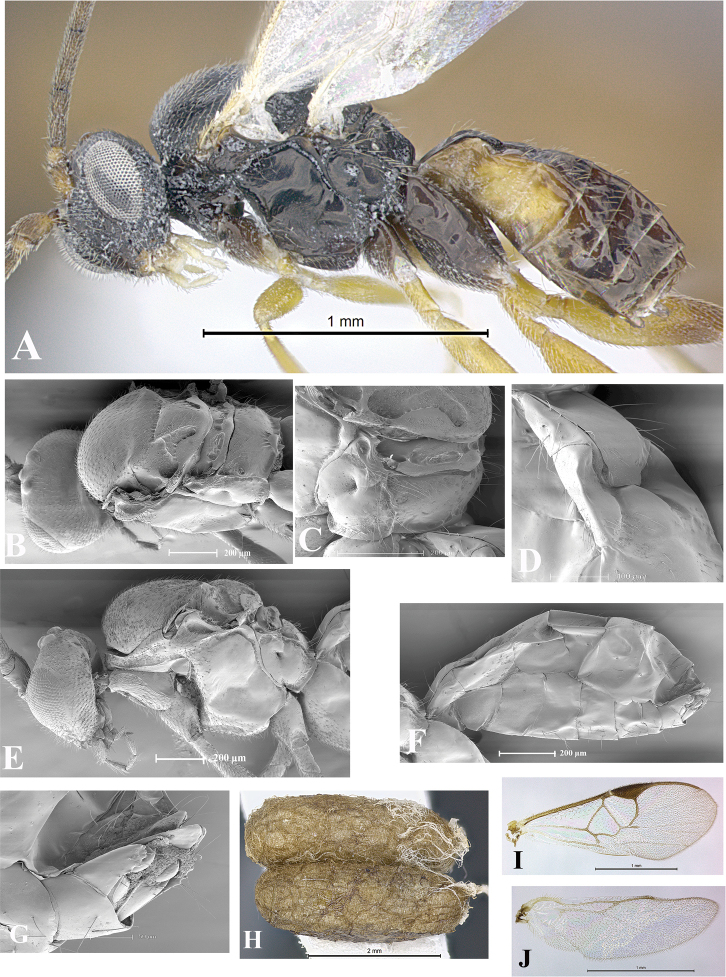
*Glyptapanteleshugokonsi* sp. nov. male 04-SRNP-2868 DHJPAR0001496 **A** Habitus **B, E** Head, mesosoma **B** Dorsolateral view **E** Lateral view **C** Metanotum, propodeum, laterodorsal view **D**T1–2, dorsolateral view **F** Metasoma, lateral view **D** Genitalia: parameres, lateral view **H** Cocoons **I, J** Wings **I** Fore **J** Hind.

Vertex in lateral view rounded (Fig. 104E), scutellum in profile flat and on same plane as mesoscutum (Fig. 104E), scutellar punctation indistinct throughout (Figs 103B, 104B), anteroventral contour of mesopleuron straight/angulate or nearly so (Figs 103A, 104A, E), distal antennal flagellomere subequal in length with penultimate, propodeal spiracle without distal carina (Figs 103B, 104B, C), scutellum in profile flat and on same plane as mesoscutum (Fig. 104E), fore wing with 2RS slightly convex, outer side of junction of r and 2RS veins not forming a stub (Figs 103G, 104I), and lateral grooves delimiting the median area on T2 distally losing definition (Figs 103C, 104D).

#### Coloration

(Fig. 103A). General body coloration dark brown except scape, pedicel, labrum, and mandibles yellow-brown; first four proximal antennal flagellomeres dorsally lighter (light brown) than ventrally (dark brown), remaining flagellomeres brown on both sides; glossa, maxillary and labial palps, and tegulae yellow. Fore and middle legs yellow except coxae light brown (inner side yellow), and claws brown; hind legs yellow except dark brown coxae, apex of femora, distal half of tibiae, and tarsomeres brown. Petiole on T1 reddish brown, contours darkened and sublateral areas yellow-brown; T2 with median and adjacent areas brown and lateral ends yellow-brown; T3 and beyond completely brown; distally each tergum with a narrow yellowish transparent band. In lateral view, T1–2 completely yellow; T3 yellow, dorsally brown; T4 and beyond completely brown. S1–2 completely yellow; S3 yellow, medially with a small brown area; S4 yellow, medially brown, distally with a wide brown band; penultimate sternum and hypopygium completely brown.

#### Description.

**Head** (Fig. 103A). Head rounded with pubescence long and dense. Proximal three antennal flagellomeres longer than wide (0.20:0.06, 0.20:0.06, 0.20:0.06), distal antennal flagellomere subequal in length with penultimate (0.11:0.05, 0.10:0.05), antenna longer than body (2.70, 2.37); antennal scrobes-frons shallow. Face flat or nearly so, with dense fine punctations, interspaces with microsculpture and longitudinal median carina present. Frons smooth. Temple wide, punctate and interspaces with microsculpture. Inner margin of eyes diverging slightly at antennal sockets; in lateral view, eye anteriorly convex and posteriorly straight. POL shorter than OOL (0.10, 0.12). Malar suture absent or difficult to see. Median area between lateral ocelli without depression. Vertex laterally rounded and dorsally wide.

**Mesosoma** (Fig. 103A, B). Mesosoma dorsoventrally convex. Mesoscutum proximally convex and distally flat with punctation distinct throughout, interspaces with microsculpture. Scutellum triangular, apex sloped and fused with BS, scutellar punctation indistinct throughout, in profile scutellum flat and on same plane as mesoscutum, phragma of the scutellum partially exposed; BS only very partially overlapping the MPM; ATS demilune with short stubs delineating the area; dorsal ATS groove smooth. Transscutal articulation with small and heterogeneous foveae, area just behind transscutal articulation with a smooth and shiny sloped transverse strip. Metanotum with BM wider than PFM (clearly differentiated); MPM circular and bisected by a median longitudinal carina; AFM without setiferous lobes and not as well delineated as PFM; PFM thick and smooth; ATM proximally with semircular/undulate carina and distally smooth. Propodeum relatively polished without median longitudinal carina, proximal half curved; distal edge of propodeum with a flange at each side and without stubs; propodeal spiracle without distal carina; nucha surrounded by very short radiating carinae. Pronotum with a distinct dorsal furrow, dorsally with a well-defined smooth band; central area of pronotum smooth, but both dorsal and ventral furrows with short parallel carinae. Propleuron with fine rugae and dorsally with a carina. Metasternum flat or nearly so. Contour of mesopleuron straight/angulate or nearly so; precoxal groove deep and with faintly transverse lineate sculpture; epicnemial ridge elongated more fusiform (tapering at both ends).

**Legs.** Ventral margin of fore telotarsus entire, but with a tiny curved seta, fore telotarsus almost same width throughout and longer than fourth tarsomere (0.12, 0.08). Hind coxa with punctation only on ventral surface and dorsal outer depression present. Inner spur of hind tibia longer than outer spur (0.21, 0.17), entire surface of hind tibia with dense strong spines clearly differentiated by color and length. Hind telotarsus longer than fourth tarsomere (0.14, 0.11).

**Wings** (Fig. 103G, H). Fore wing with r vein curved; 2RS vein slightly convex to convex; r and 2RS veins forming a weak, even curve at their junction and outer side of junction not forming a stub; 2M vein slightly curved/swollen; distally fore wing [where spectral veins are] with microtrichiae more densely concentrated than the rest of the wing; anal cell 1/3 proximally lacking microtrichiae; subbasal cell with a small smooth area; vein 2CUa absent and vein 2CUb spectral; vein 2 cu-a absent; vein 2-1A proximally tubular and distally spectral, although sometimes difficult to see; tubular vein 1 cu-a curved and complete, but junction with 1-1A vein spectral. Hind wing with vannal lobe narrow, subdistally and subproximally straightened, and setae present only proximally.

**Metasoma** (Fig. 103A, C–F). Metasoma laterally compressed. Petiole on T1 completely smooth and polished, with faint, satin-like sheen, parallel-sided in proximal half and then narrowing (length 0.30, maximum width 0.15, minimum width 0.07), and with scattered pubescence concentrated in the first distal third. Lateral grooves delimiting the median area on T2 distally losing definition (length median area 0.11, length T2 0.15), edges of median area polished and lateral grooves deep, median area broader than long (length 0.11, maximum width 0.15, minimum width 0.05); T2 with scattered pubescence only distally. T3 longer than T2 (0.19, 0.15) and with scattered pubescence throughout. Pubescence on hypopygium scattered.

**Cocoons** (Fig. 104H). White or brown oval cocoons with silk fibers evenly smooth. Cocoons in among the setae along the back of caterpillar.

#### Male

(Fig. 104A–J). Similar in coloration to female. The mesosoma is stouter than females.

#### Etymology.

Hugo L. Kons Jr. is a retired Lepidopterologist at the University of Wisconsin-Madison, WI, USA.

#### Distribution.

Parasitized caterpillars were collected in Costa Rica, ACG, Sector Brasilia (Moga), Sector Rincón Rain Forest (Flecha), and Sector San Cristóbal (Potrero Argentina), during June 2004 and 2009 at 320 m, 491 m, and 520 m in grassland and rain forest.

Adult parasitoid was collected in Costa Rica, ACG, Sector San Cristóbal (Río Blanco Abajo), during April 2008.

#### Biology.

The lifestyle of this parasitoid species is gregarious.

#### Host.

*Olceclosteraamoria* Druce (Apatelodidae) feeding on *Gmelinaarborea*, introduced species, (Verbenaceae), *Pleonotomavariabilis* and *Amphilophiumpaniculatum* (Bignoniaceae). Caterpillars were collected in third and fourth instar.

### Glyptapanteles iangauldi

Taxon classificationAnimaliaHymenopteraBraconidae

Arias-Penna, sp. nov.

http://zoobank.org/1CC9B1A5-CADB-4930-ACDC-EC2D2F1F6251

Figs 105 , 106

#### Female.

Body length 2.32 mm, antenna length 2.63 mm, fore wing length 2.42 mm.

#### Type material.

**Holotype**: COSTA RICA • 1♀; 06-SRNP-8750, DHJPAR0012681; Área de Conservación Guanacaste, Alajuela, Sector San Cristóbal, Puente Palma; rain forest; 460 m; 10.9163, -85.37869; 24.x.2006; Anabelle Córdoba leg.; caterpillar collected in fourth instar; cocoons adhered to the larval cuticle and formed on 02.xi.2006; adult parasitoids emerged on 07.xi.2006; (CNC). **Paratypes.** • 9 (4♀, 5♂) (0♀, 0♂); 06-SRNP-8750, DHJPAR0012681; same data as for holotype; (CNC).

#### Other material.

**Reared material. COSTA RICA**: *Área de Conservación Guanacaste*, *Alajuela*, *Sector San Cristóbal*, *Sendero Corredor*: • 51 (5♀, 4♂) (42♀, 0♂); 00-SRNP-11404, DHJPAR0000001; rain forest; 620 m; 10.87868, -85.38963; 08.vi.2000; Carolina Cano leg.; caterpillar collected in third instar; white separate cocoons packed in among the larval setae; adult parasitoids emerged on 28.vi.2000.

*Área de Conservación Guanacaste*, *Alajuela*, *Sector San Cristóbal*, *Río Areno*: • 88 (3♀, 3♂) (81♀, 1♂); 05-SRNP-456, DHJPAR0004241; rain forest; 460 m; 10.91407, -85.38174; 04.ii.2005; Yessenia Mendoza leg.; caterpillar collected in fourth instar; masses of brownish small separate cocoons adhered to back of caterpillar; adult parasitoids emerged on 26.ii.2005.

*Área de Conservación Guanacaste*, *Alajuela*, *Sector San Cristóbal*, *Sendero Huerta*: • 25 (3♀, 1♂) (21♀, 0♂); 06-SRNP-3984, DHJPAR0012016; rain forest; 527 m; 10.9305, -85.37223; 19.v.2006; Osvaldo Espinoza leg.; caterpillar collected in fifth instar; mass of vertical cocoons among the setae on the back of the caterpillar; adult parasitoids emerged on 11.vi.2006.

*Área de Conservación Guanacaste*, *Alajuela*, *Sector San Cristóbal*, *Vado Río Cucaracho*: • 198 (5♀, 5♂) (132♀, 54♂); 01-SRNP-971, DHJPAR0000022; rain forest; 640 m; 10.8702, -85.39153; 20.iii.2001; Carolina Cano leg.; caterpillar collected in fourth instar; cocoons densely packed among the setae of the caterpillar; adult parasitoids emerged on 01.iv.2001 and caterpillar still alive. • 93 (4♀ + 3♂) (76♀, 10♂); 04-SRNP-261, DHJPAR0000288; same data as for preceding except: 12.i.2004; Neyvin Hernandez; scattered cocoons adhered to cadaver; cocoon characteristics not reported; adult parasitoids emerged on 02.ii.2004.

*Área de Conservación Guanacaste*, *Alajuela*, *Sector San Cristóbal*, *Río Blanco Abajo*: • 164 (3♀, 3♂) (152♀, 6♂); 02-SRNP-714, DHJPAR0001480; rain forest; 500 m; 10.90037, -85.37254; 05.ii.2002; Tom Prescott leg.; caterpillar collected in fifth instar; cocoons adhered to the larval cuticle; adult parasitoids emerged on 16.ii.2002. • 130 (3♀, 3♂) (110♀, 14♂); 02-SRNP-884, DHJPAR0000264; same data as for preceding except: 08.ii.2002; caterpillar collected in fourth instar; brown single cocoons adhered among the seta on larva; adult parasitoids emerged on 20.ii.2002. • 32 (3♀, 3♂) (19♀, 7♂); 07-SRNP-5154, DHJPAR0020729; same data as for preceding except: 24.xii.2007; Elda Araya; caterpillar collected in fourth instar; cocoons brown cocoons adhered among the setae of caterpillar; adult parasitoids emerged on 11.i.2008.

*Área de Conservación Guanacaste*, *Alajuela*, *Sector San Cristóbal*, *Puente Palma*: • 133 (5♀, 5♂) (107♀, 16♂); 06-SRNP-9670, DHJPAR0012674; rain forest; 460 m; 10.9163, -85.37869; 28.xi.2006; Elda Araya leg.; caterpillar collected in third instar; solitary cocoons among the setae in the back of caterpillar cadaver densely packed upright; adult parasitoids emerged on 24.xii.2006. • 114 (5♀, 5♂) (81♀, 23♂); 06-SRNP-9671, DHJPAR0012670; same data as for preceding except: caterpillar collected in fourth instar; cocoons adhered to the larval cuticle; adult parasitoids emerged on 16.xii.2006. • 125 (5♀, 5♂) (99♀, 16♂); 09-SRNP-19, DHJPAR0034257; same data as for preceding except: 03.i.2009; caterpillar collected in fifth instar; cocoons adhered to the larval cuticle; cocoon characteristics not reported; adult parasitoids emerged on 09.i.2009.

*Área de Conservación Guanacaste*, *Alajuela*, *Sector Brasilia*, *Moga*: • 43 (4♀, 4♂) (35♀, 0♂); 11-SRNP-65131, DHJPAR0042961; rain forest; 320 m; 11.01227, -85.34929; 29.iii.2011; Minor Carmona leg.; caterpillar collected in fifth instar; cocoons adhered to the larval cuticle and formed on 07.iv.2011; adult parasitoids emerged on 11.iv.2011.

#### Diagnosis.

**Figure 105.**
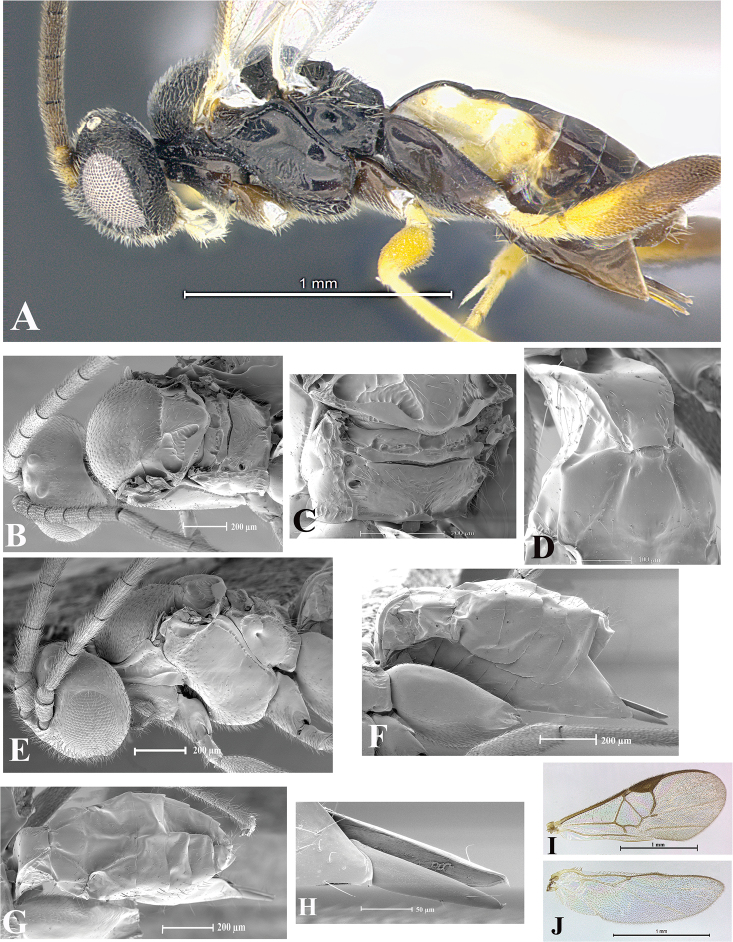
*Glyptapantelesiangauldi* sp. nov. female 01-SRNP-971 DHJPAR0000022, 06-SRNP-8750 DHJPAR0012681 **A** Habitus **B, E** Head, mesosoma **B** Dorsolateral view **E** Lateral view **C** Metanotum, propodeum, dorsal view **D**T1–2, dorsolateral view **F, G** Metasoma **F** Lateral view **G** Dorsolateral view **H** Genitalia: hypopygium, ovipositor, ovipositor sheaths, lateral view **I, J** Wings **I** Fore **J** Hind.

**Figure 106.**
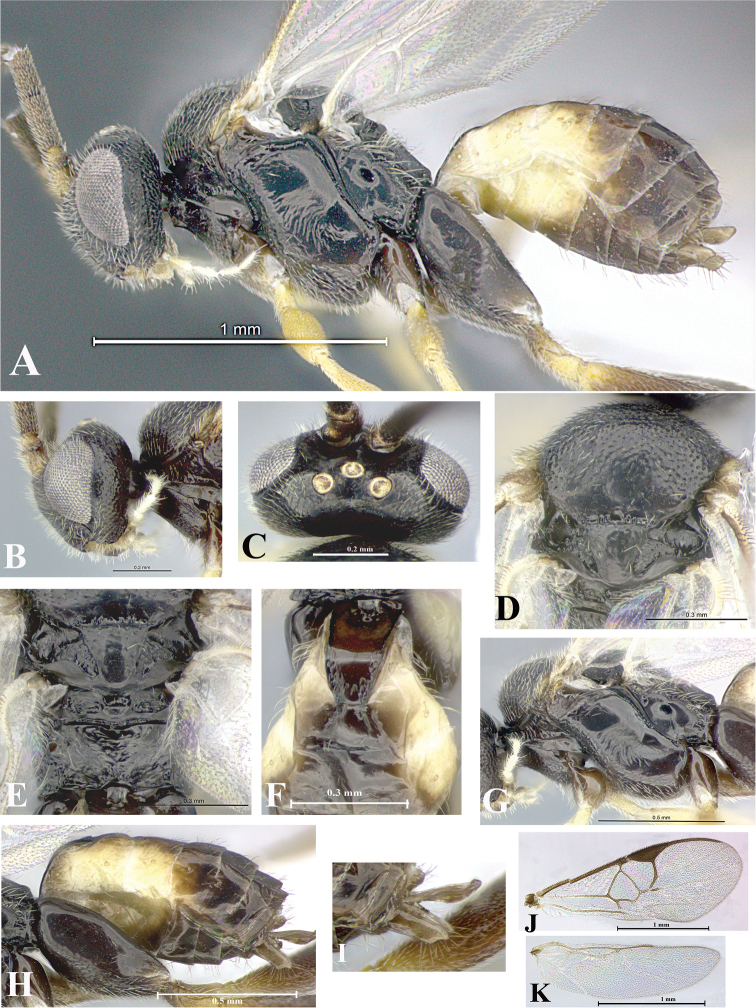
*Glyptapantelesiangauldi* sp. nov. male 01-SRNP-971 DHJPAR0000022, 06-SRNP-8750 DHJPAR0012681 **A** Habitus **B, C** Head **B** Lateral view **C** Dorsal view **D** Mesonotum, dorsal view **E** Scutellum, metanotum, propodeum, dorsal view **C**T1–2, dorsal view **G** Mesosoma, lateral view **H** Metasoma, lateral view **I** Genitalia: parameres, lateral view **J, K** Wings **J** Fore **K** Hind.

Medioanterior pit of metanotum without median longitudinal carina (Figs 105C, 106E), propodeum medially rhomboid-shaped with transverse rugae (Figs 105C, 106E), scutellum in profile convex and slightly higher than mesoscutum (Figs 105E, 106G), propodeal spiracle without distal carina (Figs 105C, 106E), phragma of the scutellum partially exposed (Figs 105C, 106E), nucha surrounded by long radiating carinae (Figs 105C, 106E), propodeum medially rhomboid-shaped with transverse rugae (Figs 105C, 106E), dorsal carina delimiting a dorsal furrow on propleuron present (Fig. 105E), petiole on T1 parallel-sided, but narrowing over distal 1/3 (Figs 105D, 106F), precoxal groove deep (Figs 105A, 106A, G), anteroventral contour of mesopleuron straight/angulate or nearly so (Figs 105A, 106G), edges of median area on T2 polished and followed by a deep groove (Figs 105D, 106F), and fore wing with r vein curved, outer side of junction of r and 2RS veins forming a distinct stub (Figs 105I, 106J).

#### Coloration

(Fig. 105A). General body coloration brown-black except labrum, mandible, scape and pedicel yellow-brown; glossa, maxillary and labial palps, and tegulae yellow. Eyes and median ocellus silver, and lateral ocelli reddish (in preserved specimen). Fore and middle legs yellow except coxae (inner side lighter than outer side) and claws brown; hind legs yellow except brown-black coxae, most of the femora brown, distal half of tibiae brown, and tarsomeres brown. Petiole on T1 dark yellow-brown, contours darkened and sublateral areas yellow; T2 with median and adjacent areas brown, and lateral ends yellow; T3 broadly brown except proximal corners yellow; T4 and beyond completely brown; distally each tergum with a narrow whitish transparent band. In lateral view, T1–2 completely yellow; T3 yellow, distally with a narrow brown band; T4 and beyond completely brown. S1–3 yellow, but medially brown; S4 yellow, but distally with a broad brown band; penultimate sternum and hypopygium completely brown.

#### Description.

**Head** (Fig. 105A, B, E). Head rounded with pubescence short and dense. Proximal three antennal flagellomeres longer than wide (0.19:0.07, 0.19:0.07, 0.19:0.07), distal antennal flagellomere longer than penultimate (0.11:0.05, 0.09:0.05), antenna longer than body (2.63, 2.32); antennal scrobes-frons shallow. Face with dense fine punctations, interspaces with microsculpture, distal half dented only laterally and longitudinal median carina present. Frons smooth. Temple wide, punctate and interspaces wavy. Inner margin of eyes diverging slightly at antennal sockets; in lateral view, eye anteriorly convex and posteriorly straight. POL shorter than OOL (0.09, 0.12). Malar suture present. Median area between lateral ocelli slightly depressed. Vertex laterally rounded and dorsally wide.

**Mesosoma** (Fig. 105A–C, E). Mesosoma dorsoventrally convex. Mesoscutum proximally convex and distally flat, punctation distinct proximally with polished area distally, interspaces wavy/lacunose. Scutellum triangular, apex sloped and fused with BS, scutellar punctation scattered throughout, in profile scutellum convex and slightly higher than mesoscutum, phragma of the scutellum partially exposed; BS only very partially overlapping the MPM; ATS demilune with short stubs delineating the area; dorsal ATS groove with carinae only proximally. Transscutal articulation with small and homogeneous foveae, area just behind transscutal articulation smooth, shiny and nearly at the same level as mesoscutum (flat). Metanotum with BM wider than PFM (clearly differentiated); MPM circular without median longitudinal carina; AFM with a small lobe and not as well delineated as PFM; PFM thick and smooth; ATM proximally with semircular/undulate carina and distally smooth. Propodeum medially rhomboid-shaped with rugae, proximal half curved with fine sculpture and distal half relatively polished; distal edge of propodeum with a flange at each side and without stubs; propodeal spiracle without distal carina; nucha surrounded by very short radiating carinae. Pronotum with a distinct dorsal furrow, dorsally with a well-defined smooth band; central area smooth, but both dorsal and ventral furrows with short parallel carinae. Propleuron with a mix of rugae and fine punctation, dorsally with a carina. Metasternum flat or nearly so. Contour of mesopleuron straight/angulate or nearly so; precoxal groove deep with faintly transverse lineate sculpture; epicnemial ridge convex, teardrop-shaped.

**Legs.** Ventral margin of fore telotarsus entire without seta, fore telotarsus almost same width throughout and longer than fourth tarsomere (0.10, 0.07). Hind coxa with punctation only on ventral surface and dorsal outer depression present. Inner spur of hind tibia longer than outer spur (0.26, 0.19), entire surface of hind tibia with dense strong spines clearly differentiated by color and length. Hind telotarsus shorter than fourth tarsomere (0.10, 0.13).

**Wings** (Fig. 105I, J). Fore wing with r vein slightly curved; 2RS vein straight; r and 2RS veins forming a weak, even curve at their junction and outer side of junction forming a slight stub; 2M vein slightly curved/swollen; distally fore wing [where spectral veins are] with microtrichiae more densely concentrated than the rest of the wing; anal cell 1/3 proximally lacking microtrichiae; subbasal cell with a small smooth area; vein 2CUa absent and vein 2CUb spectral; vein 2 cu-a absent; vein 2-1A proximally tubular, distally spectral, although sometimes difficult to see; tubular vein 1 cu-a curved, incomplete/broken and not reaching the edge of 1-1A vein. Hind wing with vannal lobe, subdistally and subproximally straightened, and setae present only proximally.

**Metasoma** (Fig. 105A, D, F–H). Metasoma laterally compressed. Petiole on T1 finely sculptured only distally, virtually parallel-sided over most of length, but narrowing over distal 1/3 (length 0.33, maximum width 0.16, minimum width 0.08), and with scattered pubescence concentrated in the first distal third. Lateral grooves delimiting the median area on T2 distally losing definition (length median area 0.11, length T2 0.15), edges of median area polished and lateral grooves deep, median area broader than long (length 0.11, maximum width 0.16, minimum width 0.08); T2 with scattered pubescence only distally. T3 longer than T2 (0.20, 0.15) and with scattered pubescence throughout. Pubescence on hypopygium scattered.

**Cocoons** (Fig. 4T). White or brown oval cocoons with silk fibers evenly smooth. Masses of separate cocoons adhered among the setae of caterpillar.

#### Male

(Fig. 106A–K). Similar in coloration to female. The mesosoma is slightly stouter than female.

#### Etymology.

Ian David Gauld (25 May 1947-12 January 2009) is a well-known British entomologist who dedicated his entire career to the evolutionary biology of Ichneumonids (Anomaloniae, Labeninae, Ophioninae, and Pimplinae). He spent the last two decades of his life focusing upon the Costa Rican fauna.

#### Distribution.

Parasitized caterpillars were collected in Costa Rica, ACG, Sector Brasilia (Moga) and Sector San Cristóbal (Río Areno, Río Blanco Abajo, Puente Palma, Sendero Corredor, Sendero Huerta, and Vado Río Cucaracho), during June 2000, March 2001, February 2002 and 2005, January 2004, May, October-November 2006, December 2007, January 2009, and March 2011 at 460 m, 500 m, 620 m, and 640 m in rain forest.

#### Biology.

The lifestyle of this parasitoid species is gregarious.

#### Host.

*Zanolaverago* Cramer (Apatelodidae) (Fig. 4T) feeding on *Iresinediffusa* (Amaranthaceae), *Philodendron* sp. (Araceae), *Psychotriaberteriana*, *Hameliapatens* and *Spermacoceocymifolia* (Rubiaceae), *Ingaoerstediana* and *I.samanensis* (Fabaceae), *Solanumcircinatum* (Solanaceae). Caterpillars were collected in third, fourth, and fifth instar.

### Glyptapanteles ianyarrowi

Taxon classificationAnimaliaHymenopteraBraconidae

Arias-Penna, sp. nov.

http://zoobank.org/921124B9-CAD7-48E6-BA26-A5510E592D83

Figs 107 , 108

#### Female.

Body length 2.22 mm, antenna length 2.63, fore wing length 2.53 mm.

#### Type material.

**Holotype**: COSTA RICA • 1♀; 08-SRNP-71961, DHJPAR0031131; Área de Conservación Guanacaste, Guanacaste, Sector Pitilla, Canita; rain forest; 480 m; 11.00006, -85.40195; 06.viii.2008; Oscar Siezar leg.; caterpillar collected in fourth instar; cocoons formed on 11.viii.2008 and adhered to the leaf substrate; adult parasitoids emerged on 15.viii.2008 and 17.viii.2008; *Mesochorus* (Ichneumonidae: Mesochorinae) was reported as hyperparasitoid; (CNC). **Paratypes.** • 25 (2♀, 3♂) (13♀, 7♂); 08-SRNP-71961, DHJPAR0031131; same data as for holotype; (CNC).

#### Other material.

**Reared material.** COSTA RICA: *Área de Conservación Guanacaste*, *Alajuela*, *Sector Rincón Rain Forest*, *Palmital*: • 19 (6♀, 5♂) (7♀, 1♂); 00-SRNP-14192, DHJPAR0000002; 420 m; 10.88264, -85.25164; 17.viii.2000; Freyci Vargas leg.; caterpillar collected in third instar; two rows of parallel side by side brown cordwood cocoons, with the caterpillar in the middle; adult parasitoids emerged on 15.viii.2008 and 01.ix.2000.

*Área de Conservación Guanacaste*, *Alajuela*, *Sector Rincón Rain Forest*, *Sendero Juntas*: • 1 (1♀, 0♂) (0♀, 0♂); 05-SRNP-41184, DHJPAR0002636; 400 m, 10.9066, -85.28784; 27.iv.2005; José Pérez leg.; caterpillar collected in fourth instar; semi-cordwood cocoons on each side of the larval cadaver, and lightly adhered to it and each other, cadaver fell off without its setae; adult parasitoids emerged on 11.v.2005.

*Área de Conservación Guanacaste*, *Alajuela*, *Sector Rincón Rain Forest*, *Sendero Llano*: 52 (3♀, 3♂) (30♀, 16♂), 06-SRNP-40409, DHJPAR0012018; 400 m, 10.90276, -85.28996; 29.i.2006; Minor Carmona leg.; caterpillar collected in fifth instar; two stacks of light brown cordwood on each side of the larva, cocoons adhered to the leaf substrate and formed on 31.i.2006; adult parasitoids emerged on 09.ii.2006.

*Área de Conservación Guanacaste*, *Alajuela*, *Sector Rincón Rain Forest*, *Finca Aurita*: • 54 (3♀, 3♂) (28♀, 20♂); 06-SRNP-42040, DHJPAR0012023; 460 m; 10.88409, -85.25728; 08.vi.2006; Minor Carmona leg.; caterpillar collected in third instar; brown cocoons in cordwood stack on each side of the cadaver, cocoons adhered to the leaf substrate and formed on 23.vi.2006; adult parasitoids emerged on 28.vi.2006.

*Área de Conservación Guanacaste*, *Alajuela*, *Sector Rincón Rain Forest*, *Quebrada Escondida*: • 48 (3♀, 3♂) (34♀, 8♂); 06-SRNP-42448, DHJPAR0012111; 420 m; 10.89928, -85.27486; 10.vii.2006; Minor Carmona leg.; caterpillar collected in second instar; two rows of cordwood on each side of caterpillar and adhered to the leaf substrate; adult parasitoids emerged on 06.viii.2006.

*Área de Conservación Guanacaste*, *Alajuela*, *Sector Rincón Rain Forest*, *Montanya Figueres*: • 16 (3♀, 3♂) (10♀, 0♂); 08-SRNP-40959, DHJPAR0030764; 460 m; 10.88367, -85.29081; 29.iv.2008; José Pérez leg.; caterpillar collected in fourth instar; cocoons adhered to the leaf substrate and formed on 10.v.2008; adult parasitoids emerged on 17.v.2008.

*Área de Conservación Guanacaste*, *Alajuela*, *Sector Rincón Rain Forest*, *Sendero Venado*: • 48 (3♀, 3♂) (27♀, 15♂); 11-SRNP-43583, DHJPAR0045226; 420 m; 10.89678, -85.27001; 01.viii.2011; José Pérez leg.; caterpillar collected in fourth instar; cocoons adhered to the leaf substrate and formed on 01.viii.2011; adult parasitoids emerged on 03.viii.2011.

*Área de Conservación Guanacaste*, *Alajuela*, *Sector Rincón Rain Forest*, *Jacobo*: • 37 (3♀, 3♂) (28♀, 3♂); 11-SRNP-80709, DHJPAR0045251; 461 m; 10.94076, -85.3177; 10.vi.2011; Edwin Apu leg.; caterpillar collected in fourth instar; cocoons adhered to the leaf substrate and formed on 16.vi.2011; adult parasitoids emerged on 23.vi.2011.

*Área de Conservación Guanacaste*, *Alajuela*, *Sector San Cristóbal*, *Potrero Argentina*: • 34 (3♀, 3♂) (22♀, 6♂); 06-SRNP-4048, DHJPAR0012011; pastures; 520 m; 10.89021, -85.38803; 22.v.2006; Gloria Sihezar leg.; caterpillar collected in third instar; two rows of beige cordwood cocoons on each side of larval cadaver, cocoons adhered to the leaf substrate; adult parasitoids emerged on 11.vi.2006.

*Área de Conservación Guanacaste*, *Guanacaste*, *Sector Pitilla*, *Sendero Lagun*: • 20 (3♀, 3♂) (9♀, 5♂); 10-SRNP-31764, DHJPAR0040444; rain forest; 680 m; 10.9888, -85.42336; 06.viii.2010; Manuel Ríos leg.; caterpillar collected in fifth instar; cocoons adhered to the leaf substrate and formed on 12.viii.2010; adult parasitoids emerged on 19.viii.2010.

*Área de Conservación Guanacaste*, *Guanacaste*, *Sector Pitilla*, *Estación Quica*: • 50 (3♀, 3♂) (36♀, 8♂); 09-SRNP-71313, DHJPAR0039963; rain forest; 470 m; 10.99697, -85.39666; 10.vii.2009; Ricardo Calero leg.; caterpillar collected in third instar; two parallel rows of cordwood of cocoons adhered to the leaf substrate and formed on 19.vii.2009; adult parasitoids emerged on 19.viii.2008. • 79 (3♀, 3♂) (32♀, 41♂); 09-SRNP-71411, DHJPAR0039965; same data as for preceding except: 20.vii.2009; batch of cordwood cocoons on each side of the cadaver adhered to the larval cuticle and formed on 02.viii.2009; adult parasitoids emerged on 09.viii.2009.

#### Diagnosis.

**Figure 107.**
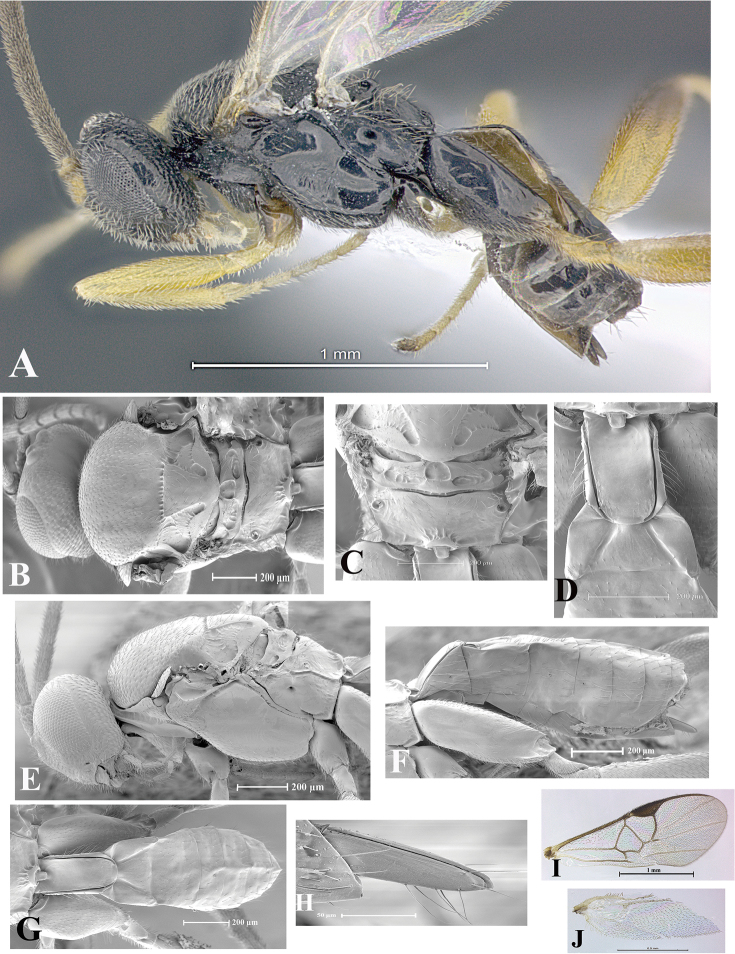
*Glyptapantelesianyarrowi* sp. nov. female 06-SRNP-42448 DHJPAR0012111, 08-SRNP-71961 DHJPAR0031131 **A** Habitus **B, E** Head, mesosoma **B** Dorsal view **E** Lateral view **C** Metanotum, propodeum, dorsal view **D**T1–2, dorsal view **F, G** Metasoma **F** Lateral view **G** Dorsal view **H** Genitalia: hypopygium, ovipositor, ovipositor sheaths, lateral view **I, J** Wings **I** Fore **J** Hind.

**Figure 108.**
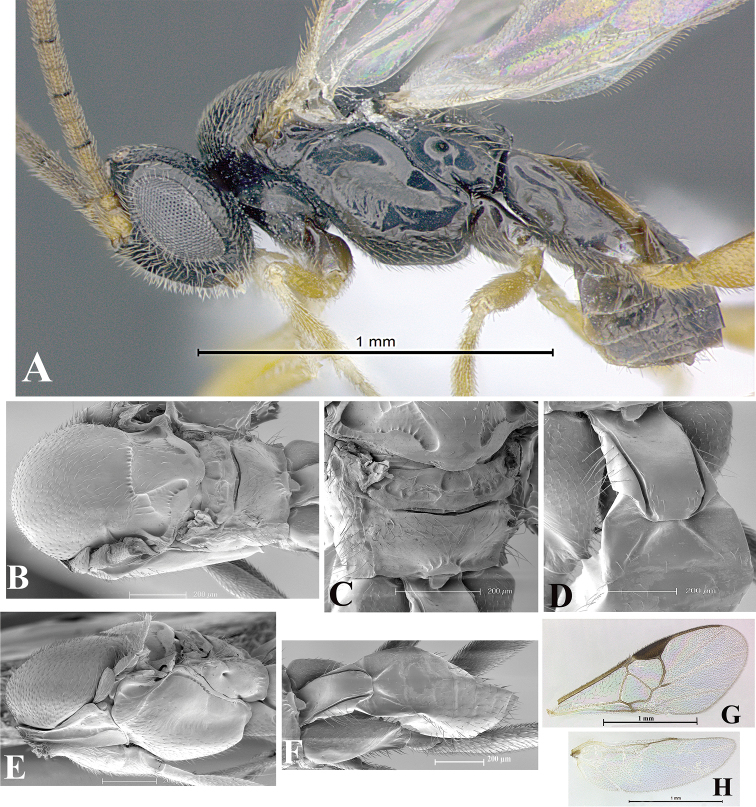
*Glyptapantelesianyarrowi* sp. nov. male 06-SRNP-42448 DHJPAR0012111, 08-SRNP-71961 DHJPAR0031131 **A** Habitus **B, E** Mesosoma **B** Dorsal view **E** Laterodorsal view **C** Metanotum, propodeum, dorsal view **D**T1–2, dorsal view **F** Metasoma, dorsolateral view **G, H** Wings **G** Fore **H** Hind.

Phragma of the scutellum widely visible (Figs 107B, C, 108B, C), longitudinal median carina on face present, inner margin of eyes diverging slightly at antennal sockets, scutellar punctation indistinct throughout (Figs 107B, C, 108B, C), petiole on T1 distally with lateral margins curved (convex, Figs 107D, 108G, D, F), propodeum without median longitudinal carina, propodeal spiracle without distal carina (Figs 107B, C, 108B, C), nucha surrounded by very short radiating carinae (Figs 107B, C, 108B, C), antenna longer than body, fore wing with 2RS vein straight, outer side of junction of r and 2RS veins not forming a stub (Fig. 107I, G), and lateral grooves delimiting the median area on T2 distally losing definition (Figs 107D, 108G, D, F).

#### Coloration

(Fig. 107A). General body coloration polished black except scape and pedicel yellow-brown; first proximal antennal flagellomeres lighter dorsally (light brown) than ventrally (dark brown), remaining flagellomeres brown on both sides; labrum and mandibles dark yellow-brown; maxillary and labial palps, and tegulae yellow. Eyes gray and ocelli reddish (in preserved specimen). Fore and middle legs yellow except fore coxae yellow-brown (proximal half darker than distal half), middle coxae black and claws brown; hind legs yellow except coxae black with brown apex, distal 1/3 of femora, distal half of tibiae and tarsomeres brown, although basitarsus proximally with a yellow-brown band. Petiole on T1 black and sublateral areas yellow-brown; T2 with median and adjacent areas black, adjacent area wide and forming together with median area a rectangle-shape, and lateral ends yellow-brown; T3 black with proximal corners yellow-brown; T4 and beyond completely black-brown; distally each tergum with a narrow yellowish transparent band. In lateral view, T1–2 completely yellow; T3 yellow, but dorsally brown; T4 and beyond completely brown-black. S1–3 yellow; S4-5 brown; hypopygium brown, but medially yellow-brown.

#### Description.

**Head** (Fig. 107A, B, E). Head rounded with pubescence short and dense. Proximal three antennal flagellomeres longer than wide (0.20:0.08, 0.19:0.08, 0.18:0.08), distal antennal flagellomere longer than penultimate (0.12:0.07, 0.09:0.07), antenna longer than body (2.63, 2.22); antennal scrobes-frons shallow. Face convex with dense fine punctations, interspaces with microsculpture and longitudinal median carina present. Frons smooth. Temple wide, punctate and interspaces wavy. Inner margin of eyes diverging slightly at antennal sockets; in lateral view, eye anteriorly convex and posteriorly straight. POL shorter than OOL (0.09, 0.13). Malar suture present. Median area between lateral ocelli slightly depressed. Vertex laterally rounded and dorsally wide.

**Mesosoma** (Fig. 107A–C, E). Mesosoma dorsoventrally convex. Distal 1/3 of mesoscutum with lateral margin slightly dented, proximally with distinctive punctation distally with a polished area, interspaces wavy/lacunose. Scutellum triangular, apex sloped and fused with BS, scutellar punctation indistinct throughout, in profile scutellum flat and on same plane as mesoscutum, phragma of the scutellum widely visible; BS not overlapping the MPM; ATS demilune with short stubs delineating the area; dorsal ATS groove with carinae only proximally. Transscutal articulation with small and homogeneous foveae, area just behind transscutal articulation with a smooth and shiny sloped transverse strip. Metanotum with BM wider than PFM (clearly differentiated); MPM circular without median longitudinal carina; AFM without setiferous lobes and not as well delineated as PFM; PFM thick and smooth; ATM proximally with semircular/undulate carina and distally smooth. Propodeum relatively polished without median longitudinal carina, proximal half curved; distal edge of propodeum with a flange at each side and without stubs; propodeal spiracle without distal carina; nucha surrounded by very short radiating carinae. Pronotum with a distinct dorsal furrow, dorsally with a well-defined smooth band; central area of pronotum smooth, but both dorsal and ventral furrows with short parallel carinae. Propleuron with a mix of rugae and fine punctation, dorsally without a carina. Metasternum flat or nearly so. Contour of mesopleuron straight/angulate or nearly so; precoxal groove deep with faintly transverse lineate sculpture; epicnemial ridge convex, teardrop-shaped.

**Legs.** Ventral margin of fore telotarsus slightly excavated and with a tiny curved seta, fore telotarsus almost same width throughout and longer than fourth tarsomere (0.11, 0.07). Hind coxa with punctation only on ventral surface and dorsal outer depression present. Inner spur of hind tibia longer than outer spur (0.22, 0.16), entire surface of hind tibia with dense strong spines clearly differentiated by color and length. Hind telotarsus as equal in length with fourth tarsomere (0.11, 0.11).

**Wings** (Fig. 107I, J). Fore wing with r vein slightly curved; 2RS vein straight; r and 2RS veins forming a weak, even curve at their junction and outer side of junction not forming a stub; 2M vein slightly curved/swollen; distally fore wing [where spectral veins are] with microtrichiae more densely concentrated than the rest of the wing; anal cell 1/3 proximally lacking microtrichiae; subbasal cell with a small smooth area; vein 2CUa absent and vein 2CUb spectral; vein 2 cu-a absent; vein 2-1A present only proximally as tubular vein; tubular vein 1 cu-a curved and complete, but junction with 1-1A vein spectral. Hind wing with vannal lobe narrow, subdistally and subproximally straightened, and setae present only proximally.

**Metasoma** (Fig. 107A, D, F–H). Metasoma laterally compressed. Petiole on T1 completely smooth and polished, with faint, satin-like sheen, virtually parallel-sided over most of length, but barely narrowing at apex (length 0.28, maximum width 0.17, minimum width 0.08), with scattered pubescence concentrated in the first distal third and apex truncate. Lateral grooves delimiting the median area on T2 distally losing definition (length median area 0.12, length T2 0.15), edges of median area polished and lateral grooves deep, median area broader than long (length 0.12, maximum width 0.17, minimum width 0.08); T2 with scattered pubescence only distally. T3 longer than T2 (0.20, 0.15) and with scattered pubescence throughout. Pubescence on hypopygium scattered.

**Cocoons.** Beige or brown oval cocoons with ordered silk fibers, but covered by a net. Two rows of cordwood on each side of caterpillar and adhered to the leaf substrate.

#### Male

(Fig. 108A–H). Similar in coloration and shape to female.

#### Etymology.

Ian Harly Hanes Yarrow (1912–1989) was a hymenopteran taxonomist at the British Museum of Natural History (today, the Natural History Museum, NHMUK, London, UK) who helped with early wasp identifications from ACG.

#### Distribution.

The parasitized caterpillars were collected in Costa Rica, ACG, Sector Pitilla (Canita, Estación Quica, and Sendero Lagun), Sector Rincón Rain Forest (Finca Aurita, Jacobo, Montanya Figueres, Palmital, Quebrada Escondida, Sendero Juntas, Sendero Llano, and Sendero Venado), and Sector San Cristóbal (Potrero Argentina), during August 2000, April 2005, January and May-July 2006, April and August 2008, July 2009, August 2010, and June and August 2011 at 400 m, 420 m, 460–480 m, 520 m, and 680 m in pasture and rain forest.

#### Biology.

The lifestyle of this parasitoid species is gregarious. *Mesochorus* (Ichneumonidae: Mesochorinae) was reported as hyperparasitoid.

#### Host.

*Napataflaviceps* Hampson (Erebidae, Arctiinae) feeding on *Cespedesiaspathulata* (Ochnaceae); *Episcepsishypoleuca* (Hampson) (Erebidae, Arctiinae) feeding on *Ochromapyramidale* (Malvaceae); *Hyaleucereamorosa* Schaus (Erebidae, Arctiinae) feeding on *Pouroumabicolor* (Urticaceae); *Eucereonaurantiaca* Draudt (Erebidae, Arctiinae) feeding on *Ficuscitrifolia* and *F.colubrinae* (Moraceae). Caterpillars were collected in second, third, fourth, and fifth instar.

### Glyptapanteles ilarisaaksjarvi

Taxon classificationAnimaliaHymenopteraBraconidae

Arias-Penna, sp. nov.

http://zoobank.org/91B2251E-34FD-4D5E-8424-5C33DFA8AA7F

Figs 109 , 110

#### Female.

Body length 2.27 mm, antenna length 2.22 mm, fore wing length 2.12 mm.

#### Type material.

**Holotype**: COSTA RICA • 1♀; 06-SRNP-56482, DHJPAR0012003; Área de Conservación Guanacaste, Guanacaste, Sector Mundo Nuevo, Vado Miramonte; dry-rain intergrade forest; 305 m; 10.77175, -85.43400; 11.vi.2006; José A. Sánchez leg.; caterpillar collected in fifth instar; larva was found in the field with two rows of cordwood cocoons next to it; adult parasitoids emerged on 18.vi.2006, 25.vi.2006 and 26.vi.2006; *Copidosomafloridanum* Ashmead (Chalcidoidea: Encyrtidae, Encyrtinae) was reported as other parasitoid; (CNC). **Paratypes.** • 5 (2♀, 3♂) (0♀, 0♂); 06-SRNP-56482, DHJPAR0012003; same data as for holotype; (CNC).

#### Other material.

**Reared material.** COSTA RICA: *Área de Conservación Guanacaste*, *Guanacaste*, *Sector Santa Rosa*, *Vado Cuajiniqui*: • 18 (3♀, 2♂) (12♀, 1♂); 92-SRNP-350, DHJPAR0001475; dry forest; 275 m; 10.94041, -85.68043; 05.ii.1992; gusaneros leg.; brown cocoons at right angles to caterpillar body forming two rows parallel cordwood to the caterpillar body, caterpillar stayed alive for three days next to the cocoons; cocoons formed on 15.ii.1992; adult parasitoids emerged on 23.ii.1992. • 33 (2♀, 2♂) (18♀, 11♂); 92-SRNP-348, DHJPAR0001460; same data as for preceding except: brown cocoons at right angles to caterpillar body in two rows parallel to the caterpillar body; date of cocoons not reported; adult parasitoids emerged on 18.ii.1992. • 8 (4♀, 0♂) (4♀, 0♂); 94-SRNP-5424, DHJPAR0000086; same data as for preceding except: 06.vii.1994; caterpillar collected in third instar; two rows of parallel cordwood cocoons adhered to the leaf substrate; date of cocoons not reported; adult parasitoids emerged on 22.vii.1994. • 7 (2♀, 2♂) (2♀, 1♂); 94-SRNP-5427, DHJPAR0001439; same data as for preceding except: 06.vii.1994; caterpillar collected in fifth instar; two rows of parallel cordwood cocoons adhered to the leaf substrate; date of cocoons not reported; adult parasitoids emerged on 17.vii.1994.

*Área de Conservación Guanacaste*, *Guanacaste*, *Sector Potrerillos*, *Río Azufrado*: • 51 (6♀, 3♂) (36♀, 6♂); 02-SRNP-32075, DHJPAR0000029; dry forest; 95 m; 10.81224, -85.54438; 01.x.2002; Guillermo Pereira leg.; caterpillar collected in fourth instar; two rows of parallel gray cordwood cocoons on each side of the larva and adhered to the leaf substrate; adult parasitoids emerged on 18.x.2002. • 7 (1♀, 2♂) (0♀, 4♂); 06-SRNP-13960, DHJPAR0005105; same data as for preceding except: 18.v.2006; two rows of beige cordwood cocoons on each side of the larva and adhered to the leaf substrate; adult parasitoids emerged on 03.vi.2006. • 75 (7♀, 6♂) (61♀, 1♂); 06-SRNP-13955, DHJPAR0005106, DHJPAR0012004; same data as for preceding except: 18.v.2006; Lucía Vargas leg.; caterpillar collected in fifth instar; cocoon characteristics not reported; adult parasitoids emerged on 30.v.2006. • 57 (3♀, 3♂) (47♀, 4♂); 08-SRNP-12256, DHJPAR0030795; same data as for preceding except: 11.iv.2008; Lucía Vargas leg.; caterpillar collected in fifth instar; two rows of cordwood cocoons on each side of larva cadaver, cocoons formed on 16.iv.2008 and adhered to the leaf substrate; adult parasitoids emerged on 22.iv.2008.

*Área de Conservación Guanacaste*, *Guanacaste*, *Sector Liberia*, *Liberia*: • 43 (3♀, 3♂) (31♀, 6♂); 10-SRNP-13639, DHJPAR0039427; dry forest; 140 m; 10.62972, -85.44162; 19.vi.2010; Guillermo Pereira leg.; caterpillar collected in fifth instar; cocoons formed on 21.vi.2010; adult parasitoids emerged on 27.vi.2010. • 42 (3♀, 3♂) (26♀, 10♂); 10-SRNP-13640, DHJPAR0039428; same data as for preceding except: cocoons formed on 20.vi.2010. • 37 (3♀, 3♂) (24♀, 7♂); 10-SRNP-13641, DHJPAR0041712; ; same data as for preceding except: caterpillar already with cocoons.

*Área de Conservación Guanacaste*, *Guanacaste*, *Sector Cacao*, *Sendero a Maritza*, *1 km NW estación Cacao*: • 17 (3♀, 2♂) (12♀, 0♂); 10-SRNP-35966, DHJPAR0041678; cloud forest; 1,150 m; 10.92691, -85.46822; 26.viii.2010; Manuel Pereira leg.; caterpillar collected in third instar; cocoons formed on 03.ix.2010; adult parasitoids emerged on 14.ix.2010.

*Área de Conservación Guanacaste*, *Guanacaste*, *Sector Cacao*, *Estación Cacao*: • 19 (3♀, 3♂) (16♂, 0♂); 10-SRNP-35502, DHJPAR0040382; cloud forest; 1,150 m; 10.92691, -85.46822; 10.vii.2010; Fredy Quesada leg.; caterpillar collected in third instar; cocoons formed on 19.vii.2010; adult parasitoids emerged on 26.vii.2010. • 47 (3♀, 3♂) (41♀, 0♂); 10-SRNP-35505, DHJPAR0040386; same data as for preceding except: Manuel Pereira leg.; caterpillar collected in fifth instar; two rows of cordwood cocoons adhered to the leaf substrate and formed on 15.vii.2010; adult parasitoids emerged on 23.vii.2010. • 43 (3♀, 2♂) (38♀, 0♂); 10-SRNP-35500, DHJPAR0040391; same data as for preceding except: 09.vii.2010; Manuel Pereira leg.; cordwood cocoons adhered to te leaf substrate; adult parasitoids emerged on 27.vii.2010. • 6 (2♀, 2♂) (2♂, 0♂; 10-SRNP-35504, DHJPAR0040393; same data as for preceding except: Manuel Pereira leg.; caterpillar collected in fifth instar; two rows of cordwood cocoons adhered to the leaf substrate and formed on 15.vii.2010; adult parasitoids emerged on 23.vii.2010. • 40 (3♀, 3♂), (31♀, 3♂); 10-SRNP-35517, DHJPAR0040395; same data as for preceding except: 09.vii.2010; Manuel Pereira leg.; caterpillar collected in fifth instar; two rows of cordwood cocoons adhered to the leaf substrate; adult parasitoids emerged on 25.vii.2010. • 27 (3♀, 3♂) (21♀, 0♂); 10-SRNP-35498, DHJPAR0040399; same data as for preceding except: 09.vii.2010; Manuel Pereira leg.; caterpillar collected in fifth instar; two rows of cordwood cocoons adhered to the leaf substrate; adult parasitoids emerged on 25.vii.2010. • 84 (3♀, 3♂) (73♀, 5♂); 10-SRNP-35509, DHJPAR0040409; same data as for preceding except: 12.vii.2010, Harry Ramirez leg.; caterpillar collected in fifth instar; two rows of cordwood cocoons adhered to the leaf substrate; adult parasitoids emerged on 29.vii.2010.

*Área de Conservación Guanacaste*, *Guanacaste*, *Sector Horizontes*, *Vado Río Tempisque*: • 37 (3♀, 3♂) (25♀, 6♂); 10-SRNP-13469, DHJPAR0041710; dry forest; 19.vi.2010; Guillermo Pereira leg.; caterpillar collected in fifth instar. • 97 (3♀, 3♂) (81♀, 10♂; 10-SRNP-13470, DHJPAR0045167; same data as for preceding except: caterpillar collected in fifth instar; large stacks of brown cordwood cocoons adhered to the leaf substrate.

*Área de Conservación Guanacaste*, *Guanacaste*, *Sector Pitilla*, *Estación Quica*: • 17 (3♀, 3♂) (10♀, 1♂); 10-SRNP-73408, DHJPAR0041644; rain forest; 470 m; 10.99697, -85.39666; 27.xii.2010; Ricardo Calero leg.; caterpillar collected in third instar; cocoons adhered to the leaf substrate and formed on 11.i.2011; adult parasitoids emerged on 20.i.2010. • 8 (2♀, 2♂) (4♀, 0♂); 11-SRNP-70040, DHJPAR0042065; same data as for preceding except: 03.i.2011; caterpillar collected in fourth instar; cocoons formed on 14.i.2011; adult parasitoids emerged on 21.i.2011. • 7 (2♀, 2♂) (3♀, 0♂); 11-SRNP-70063, DHJPAR0042066; same data as for preceding except: 04.i.2011; Dinia Martinez leg.; caterpillar collected in fourth instar; cocoons formed on 14.i.2011; adult parasitoids emerged on 21.i.2011. • 28 (3♀, 3♂) (22♀, 0♂); 11-SRNP-70137, DHJPAR0042508; same data as for preceding except: 06.i.2011; Dinia Martinez leg.; caterpillar collected in fourth instar; cocoons formed on 04.ii.2011; adult parasitoids emerged on 10.ii.2011.

#### Malaise-trapped material.

COSTA RICA: *Área de Conservación Guanacaste*, *Guanacaste*, *Sector El Hacha*, *Sendero Bejuquilla*: • 1 (1♀, 0♂) (0♀, 0♂); 98-SRNP-16170, DHJPAR0013352; Malaise trap; intergrade dry-rain forest; 280 m; 11.03004, -85.52699; 10.viii.1998; DH Janzen & W Hallwachs leg.

**Yellow pan-trapped material.** COSTA RICA: *Área de Conservación Guanacaste*, *Guanacaste*, *Sector Santa Rosa*, *Área Administrativa*: • 1 (1♀, 0♂) (0♀, 0♂); 08-SRNP-12187, DHJPAR0024753; yellow pan-trapped; dry forest; 295 m; 10.83764, -85.61871; 12.i.2008; Andy Deans leg.

#### Diagnosis.

**Figure 109.**
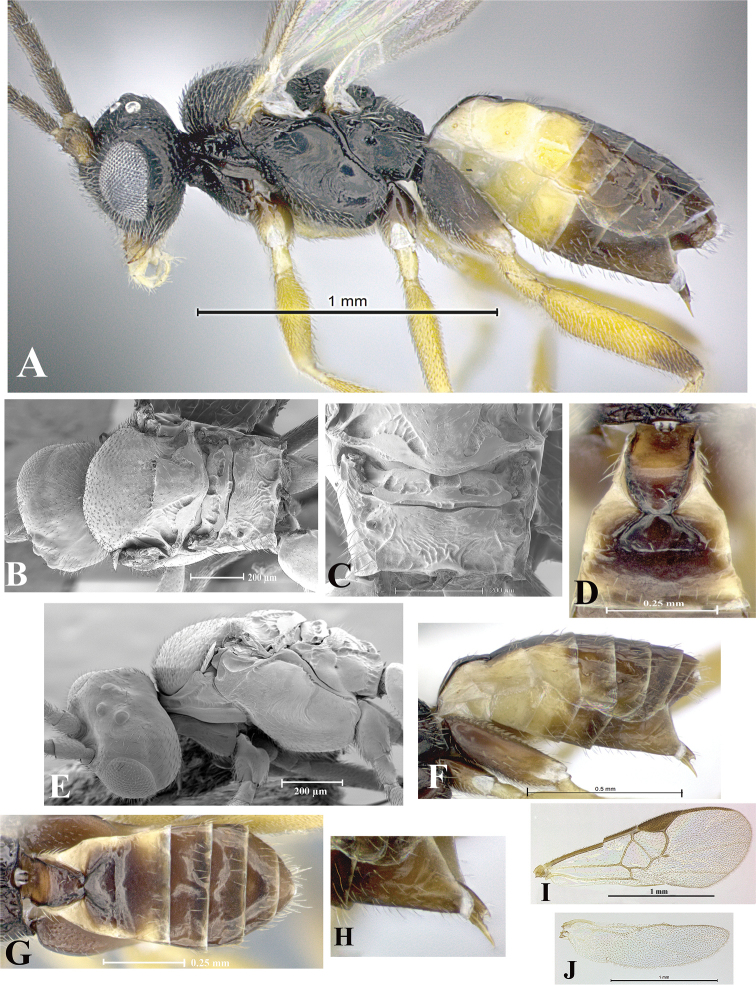
*Glyptapantelesilarisaaksjarvi* sp. nov. female 92-SRNP-348 DHJPAR0001460, 06-SRNP-56482 DHJPAR0012003 **A** Habitus **B, E** Head, mesosoma **B** Dorsal view **E** Lateral view **C** Metanotum, propodeum, dorsal view **D**T1–3, dorsal view **F, G** Metasoma **F** Lateral view **G** Dorsal view **H** Genitalia: hypopygium, ovipositor, ovipositor sheaths, lateral view **I, J** Wings **I** Fore **J** Hind.

**Figure 110.**
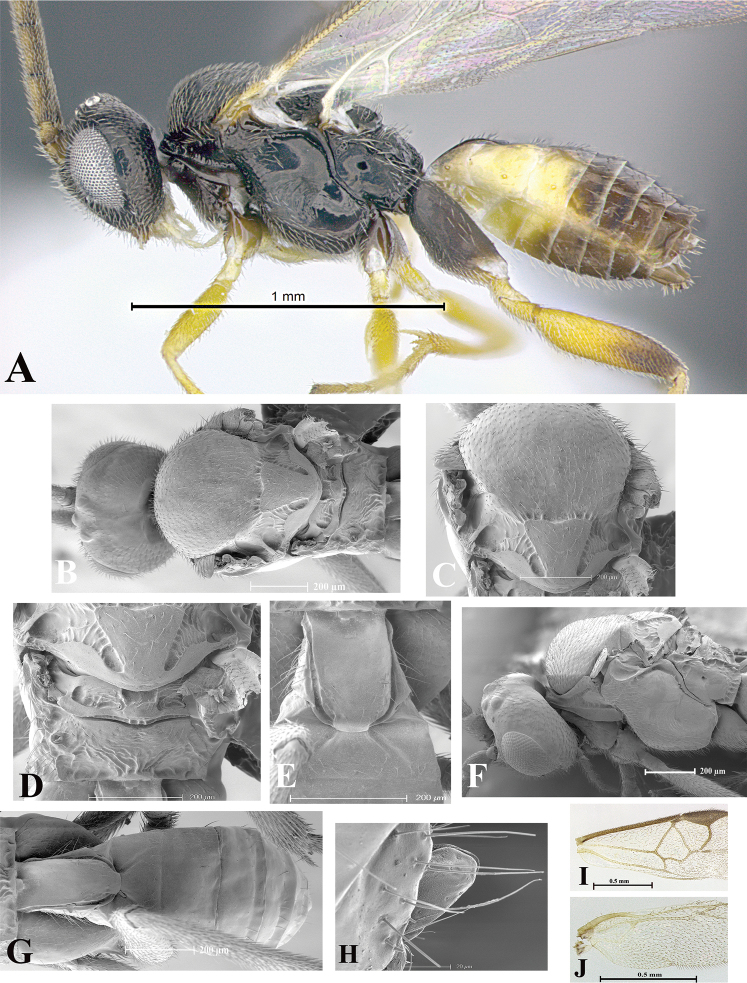
*Glyptapantelesilarisaaksjarvi* sp. nov. male 92-SRNP-348 DHJPAR0001460, 06-SRNP-56482 DHJPAR0012003 **A** Habitus **B, F** Head, mesosoma **B** Dorsal view **F** Lateral view **C** Mesonotum, dorsal view **D** Scutellum, metanotum, propodeum, dorsal view **E**T1–2, dorsal view **G** Metasoma, dorsal view **H** Genitalia: parameres, lateral view **I, J** Wings **I** Fore **J** Hind.

Nucha surrounded by long radiating carinae (Figs 109B, C, 110B, D), propodeum medially rhomboid-shaped with transverse rugae, but no trace of median longitudinal carina (Figs 109B, C, 110B, D), antenna shorter than body, fore wing with 2RS vein straight, outer side of junction of r and 2RS veins not forming a stub (Figs 109I, 110I), and lateral grooves delimiting the median area on T2 distally losing definition (Figs 109D, G, 110E, G).

#### Coloration

(Fig. 109A, D, F–J). General body coloration brown-black except labrum, mandibles, scape, and pedicel yellow-brown; first four proximal antennal flagellomeres dorsally lighter (light brown) than ventrally (dark brown), remaining flagellomeres dark brown on both sides; glossa, maxillary and labial palps, and tegulae yellow. Eyes and ocelli silver. Fore and middle legs yellow except coxae which outer side is light brown, but inner side is yellow-brown, and claws brown; hind legs yellow except black coxae, apex of femora, apex of tibiae, and tarsomeres brown. Petiole on T1 with two colorations: proximal 3/4 reddish brown and distal 1/4 brown, contours black, and sublateral areas yellow; T2 with median and adjacent areas dark brown, adjacent area narrow with limits well-defined, and lateral ends yellow; medially T3 with a brown area reaching the distal edge of T3, width of brown area coincides with the distal width of median and adjacent dark areas on T2, and lateral ends yellow; T4 and beyond completely brown; distally each tergum with a narrow yellowish transparent band. In lateral view, T1–3 completely yellow; T4 mostly brown only with a small ventral yellow area; T5 and beyond completely brown. S1–4 yellow; penultimate sternum and hypopygium brown.

#### Description.

**Head** (Fig. 109A, B, E). Head rounded with pubescence short and dense. Proximal three antennal flagellomeres longer than wide (0.17:0.08, 0.16:0.08, 0.16:0.08), distal antennal flagellomere longer than penultimate (0.11:0.05, 0.07:0.05), antenna shorter than body (2.22, 2.27); antennal scrobes-frons shallow. Face flat or nearly so, with dense and fine punctations, interspaces with microsculpture and longitudinal median carina absent. Frons smooth. Temple wide, punctate and interspaces wavy. Inner margin of eyes diverging slightly at antennal sockets; in lateral view, eye anteriorly convex, posteriorly straight. POL subequal in length with OOL (0.10, 0.11). Malar suture present. Median area between lateral ocelli without depression. Vertex lateral rounded and dorsally wide.

**Mesosoma** (Fig. 109A–C, E). Mesosoma dorsoventrally convex. Distal 1/3 of mesoscutum with lateral margin slightly dented, punctation distinct throughout, interspaces with microsculpture. Scutellum triangular, apex sloped and fused with BS, scutellar punctation indistinct throughout, in profile scutellum flat and on same plane as mesoscutum phragma of the scutellum partially exposed; BS only very partially overlapping the MPM; ATS demilune with quite a little complete parallel carinae; dorsal ATS groove with carinae only proximally. Transscutal articulation with small and heterogeneous foveae, area just behind transscutal articulation smooth, shiny and depressed centrally. Metanotum with BM wider than PFM (clearly differentiated); MPM circular and bisected by a median longitudinal carina; AFM with a small lobe and not as well delineated as PFM; PFM thick, smooth and with a distal flat flange; ATM proximally with semircular/undulate carina and distally smooth. Propodeum with transverse rugae, proximal half curved with fine sculpture and distal half rugose; distal edge of propodeum with a flange at each side and without stubs; propodeal spiracle distally framed by a short concave carina; nucha surrounded by long radiating carinae. Pronotum with a distinct dorsal furrow, and dorsally with a well-defined smooth band; central area of pronotum and both dorsal and ventral furrows smooth. Propleuron with a mix of rugae and fine punctation, dorsally with a carina. Metasternum flat or nearly so. Contour of mesopleuron straight/angulate or nearly so; precoxal groove deep with transverse lineate sculpture; epicnemial ridge elongated more fusiform (tapering at both ends).

**Legs.** Ventral margin of fore telotarsus entire, but with a tiny curved seta, fore telotarsus almost same width throughout and longer than fourth tarsomere (0.11, 0.07). Hind coxa with punctation only on ventral surface, dorsal outer depression present. Inner spur of hind tibia longer than outer spur (0.29, 0.25), entire surface of hind tibia with dense strong spines clearly differentiated by color and length. Hind telotarsus longer than fourth tarsomere (0.12, 0.09).

**Wings** (Fig. 109I, J). Fore wing with r vein curved; 2RS vein straight; r and 2RS veins forming a weak, even curve at their junction and outer side of junction not forming a stub; 2M vein slightly curved/swollen; distally fore wing [where spectral veins are] with microtrichiae more densely concentrated than the rest of the wing; anal cell 1/3 proximally lacking microtrichiae; subbasal cell with a small smooth area; vein 2CUa absent and 2CUb spectral; vein 2 cu-a absent; vein 2-1A present only proximally as tubular vein; tubular vein 1 cu-a curved, complete and touching the edge of 1-1A vein. Hind wing with vannal lobe very narrow, subdistally and subproximally straightened, and setae present only proximally.

**Metasoma** (Fig. 109A, D, F–H). Metasoma laterally compressed, petiole finely sculptured only laterally, virtually parallel-sided over most of length, but barely narrowing over distal 1/3, apex truncate (length 0.28, maximum width 0.14, minimum width 0.08), and with scattered pubescence concentrated in the first distal third. Lateral grooves delimiting the median area on T2 distally losing definition (length median area 0.10, length T2 0.13), edges of median area polished and lateral grooves deep, median area broader than long (length 0.10, maximum width 0.18, minimum width 0.07); T2 with scattered pubescence only distally. T3 longer than T2 (0.17, 0.13) and with scattered pubescence only distally. Pubescence on hypopygium dense.

**Cocoons** (Fig. 4V). Light brown, beige or gray oval cocoons with ordered silk fibers, but covered by a net. Two rows of parallel cordwood on each side of the larva and adhered to the leaf substrate.

#### Comments.

Distally, the propodeal spiracle is framed by a concave carina; lateral areas on the propodeum, at each side of nucha, with some carinae. Some specimens with the petiole reddish brown and contours black. Both sexes are slim.

#### Male

(Fig. 110A–J). Similar in coloration and shape to female.

#### Etymology.

Ilari Eerikki Sääksjärvi is director at the Biodiversity Unit, University of Turku, Finland. His research is focused on diversity, taxonomy, and systematics of tropical, especially Amazonian, ichneumonid parasitoid wasps.

#### Distribution.

The parasitized caterpillars were collected in Costa Rica, ACG, Sector Cacao (Estación Cacao and Sendero a Maritza), Sector Horizontes (Vado Río Tempisque), Sector Liberia (Liberia), Sector Pitilla (Estación Quica), Sector Mundo Nuevo (Vado Miramonte), Sector Potrerillos (Río Azufrado), and Sector Santa Rosa (Vado Cuajiniqui), during February 1992, July 1994, October 2002, April-June 2006, June-August 2010, and January-February and December 2011 at 95 m, 140 m, 275 m, 305 m, 470 m, and 1,150 m on dry, dry-rain intergrade, rain , and cloud forests.Adult parasitoids were collected in Costa Rica, ACG, Sector El Hacha (Sendero Bejuquilla) and Sector Santa Rosa (Área Administrativa) on August 1998 and January 2008 at 280 m and 295 m in intergrade dry-rain forest and dry forest.

#### Biology.

The lifestyle of this parasitoid species is gregarious. A case of multiparasitoidism was reported: *Copidosomafloridanum* Ashmead (Chalcidoidea: Encyrtidae, Encyrtinae).

#### Host.

*Agraphaoxygramma* (Geyer) (Noctuidae: Plusiinae) feeding on *Baccharistrinervis* (Asteraceae). *Argyrogrammabasigera* (Walker) (Noctuidae: Plusiinae) feeding on *Hydrocotyleumbellate* (Araliaceae) and *A.verruca* (F.) (Noctuidae: Plusiinae) (Fig. 4V) feeding on *Echinodorussubalatus* (Alismataceae). Soybean loope *Pseudoplusiaincludens* (Walker) (Noctuidae: Plusiinae) feeding on *Milleriaquinqueflora* (Asteraceae). *Condicacupienta* (Cramer) (Noctuidae: Amphipyrinae) feeding on *Mikaniacordifolia* and *M.micrantha* (Asteraceae) and *C.sutor* (Guenée) (Noctuidae: Amphipyrinae) feeding on *Eryngiumfoetidum* (Apiaceae), *Elephantopusmollis* and *Lepidaploacinera* (Asteraceae). Undetermined species of Noctuidae feeding on *Stachytarphetajamaicensis* (Verbenaceae). Caterpillars were collected in third, fourth, and fifth instar.

### Glyptapanteles jacklonginoi

Taxon classificationAnimaliaHymenopteraBraconidae

Arias-Penna, sp. nov.

http://zoobank.org/E8E74CF6-05CC-43AF-BF7D-4491F7026655

Figs 111 , 112

#### Female.

Body length 2.17 mm, antenna length 2.53 mm, fore wing length 2.22 mm.

#### Type material.

**Holotype**: COSTA RICA • 1♀; 98-SRNP-2542, DHJPAR0000108; Área de Conservación Guanacaste, Guanacaste, Sector Cacao, Estación Cacao; cloud forest; 1,150 m; 10.92691, -85.46822; 06.iii.1998; Michael Jacobson leg.; caterpillar collected in fifth instar already with cocoons on it; slate gray single cocoons, elongate ovoid, appear to be normally that dark gray color, look like were lightly adhered singly to cuticle and then fell off; adult parasitoids emerged on 09.iii.1998; (CNC). **Paratypes.** • 25 (4♀, 1♂) (20♀, 0♂); 98-SRNP-2542, DHJPAR0000108; same data as for holotype; (CNC).

#### Diagnosis.

**Figure 111.**
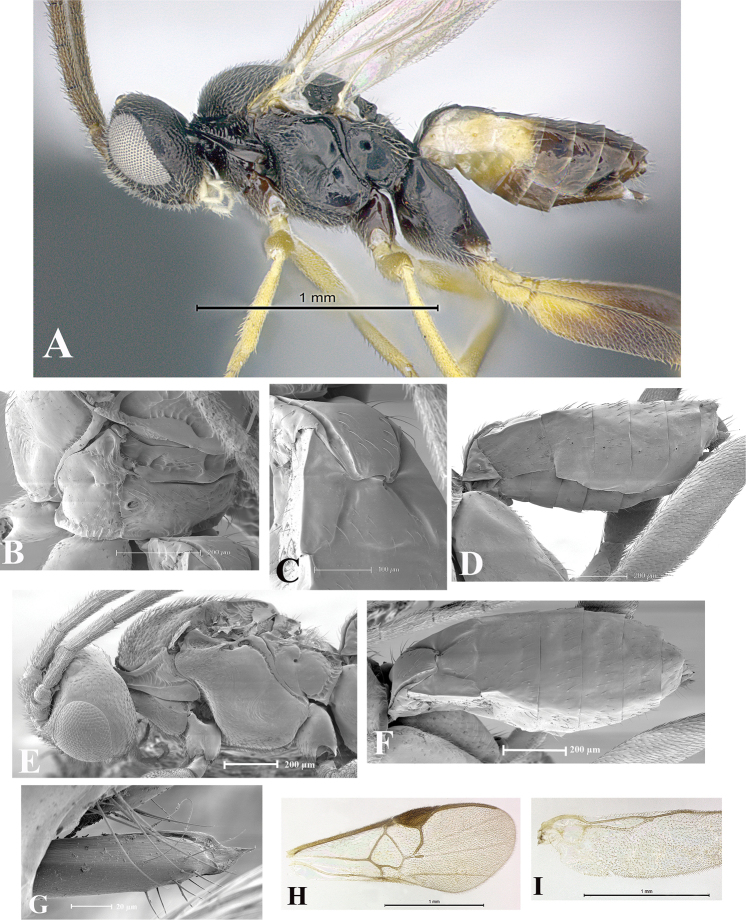
*Glyptapantelesjacklonginoi* sp. nov. female 98-SRNP-2542 DHJPAR0000108 **A** Habitus **B** Metanotum, propodeum, laterodorsal view **C**T1–2, dorsolateral view **D, F** Metasoma **D** Lateral view **F** Dorsal view **E** Head, mesosoma, lateral view **G** Genitalia: hypopygium, ovipositor, ovipositor sheaths, lateral view **H, I** Wings **H** Fore **I** Hind.

**Figure 112.**
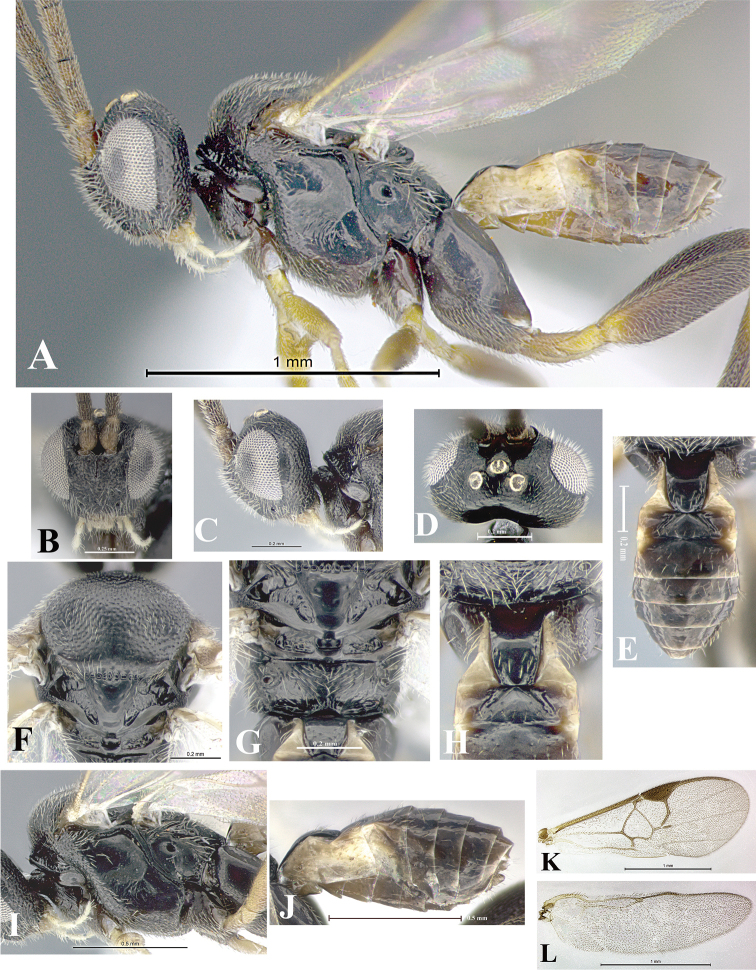
*Glyptapantelesjacklonginoi* sp. nov. male 98-SRNP-2542 DHJPAR0000108 **A** Habitus **B–D** Head **B** Frontal view **C** Lateral view **D** Dorsal view **E, J** Metasoma **E** Dorsal view **J** Lateral view **F** Mesonotum, dorsal view **G** Scutellum, metanotum, propodeum, dorsal view **H**T1–2, dorsal view **I** Mesosoma, lateral view **K, L** Wings **K** Fore **L** Hind.

Face convex (Fig. 112C), area just behind transscutal articulation nearly at the same level as mesoscutum (Fig. 112F), ventral margin of fore telotarsus slightly excavated and with a tiny curved seta, distal antennal flagellomere longer than penultimate, inner spur of hind tibia much longer than outer spur, median area on T2 broader than long (Figs 111C, F, 112E, H), propodeal spiracle distally framed by a short concave carina, propodeum without median longitudinal carina (Figs 111B, 112G), petiole on T1 distally with lateral margins relatively straight, finely sculptured only laterally (Figs 111C, F, 112H, E), surface of metasternum flat or nearly so, precoxal groove deep with lineate sculpture (Figs 111A, E, 112A, I), fore wing with vein 1 cu-a curved, r vein slightly curved, outer side of junction of r and 2RS veins not forming a stub (Figs 111F, 112K), dorsal outer depression on hind coxa present (Figs 111A, 112A), inner margin of eyes diverging slightly at antennal sockets (Fig. 112B), and lateral grooves delimiting the median area on T2 clearly defined and reaching the distal edge of T2 (Figs 111C, F, 112E, H).

#### Coloration

(Fig. 111A). General body coloration black-brown except labrum, mandibles and glossa yellow-brown; maxillary and labial palps, and tegulae yellow; scape and pedicel yellow-brown, but inner side brown; all antennal flagellomeres dark brown on both sides. Eyes silver and ocelli yellowish. Fore and middle legs yellow except brown coxae (inner side of fore coxae is lighter that outer side), and brown claws; hind legs yellow except coxae, most of the femora, distal half of tibiae and tarsomeres brown. Petiole on T1 black and sublateral areas yellow; T2 with median and adjacent areas dark brown, and lateral ends yellow; T3 mostly dark brown, but laterally yellow– T4 and beyond completely brown; distally each tergum with a narrow yellowish transparent band. In lateral view, T1-2 completely yellow; T3 yellow with a small dorsal brown area; T4 and beyond completely brown. S1–3 yellow; S4 and beyond brown.

#### Description.

**Head** (Fig. 111A, E). Head rounded with pubescence short and dense. Proximal three antennal flagellomeres longer than wide (0.18:0.05, 0.17:0.05, 0.19:0.05), distal antennal flagellomere longer than penultimate (0.11:0.05, 0.09:0.05), antenna longer than body (2.53, 2.17); antennal scrobes-frons shallow. Face convex, dense and finely punctate, interspaces with microsculpture and longitudinal median carina present. Frons smooth. Temple wide, punctate and interspaces wavy. Inner margin of eyes diverging slightly at antennal sockets; in lateral view, eye anteriorly convex and posteriorly straight. POL shorter than OOL (0.09, 0.12). Malar suture absent or difficult to see. Median area between lateral ocelli without depression. Vertex laterally rounded and dorsally wide.

**Mesosoma** (Fig. 111A, B, E). Mesosoma dorsoventrally convex. Mesoscutum proximally convex and distally flat, punctation distinct throughout, interspaces with microsculpture. Scutellum triangular, apex sloped and fused with BS, scutellar punctation scattered throughout, in profile scutellum flat and on same plane as mesoscutum, phragma of the scutellum partially exposed; BS only very partially overlapping the MPM; ATS demilune with quite a little complete parallel carinae; dorsal ATS groove with semicircular/parallel carinae. Transscutal articulation with small and homogeneous foveae, area just behind transscutal articulation smooth, shiny and nearly at the same level as mesoscutum (flat). Metanotum with BM wider than PFM (clearly differentiated); MPM circular without median longitudinal carina; AFM with a small lobe and not as well delineated as PFM; PFM thick, smooth and with a distal flat flange; ATM proximally with semircular/undulate carina and distally smooth. Propodeum with medium-sized punctation, without median longitudinal carina, proximal half weakly curved; distal edge of propodeum with a flange at each side and without stubs; propodeal spiracle distally framed by a short concave carina; nucha surrounded by very short radiating carinae. Pronotum virtually without trace of dorsal furrow, dorsally with a well-defined smooth band; central area of pronotum smooth, but both dorsal and ventral furrows with short parallel carinae. Propleuron with fine punctations throughout and dorsally with a carina. Metasternum flat or nearly so. Contour of mesopleuron straight/angulate or nearly so; precoxal groove deep with transverse lineate sculpture; epicnemial ridge elongated more fusiform (tapering at both ends).

**Legs.** Ventral margin of fore telotarsus slightly excavated and with a tiny curved seta, fore telotarsus almost same width throughout and longer than fourth tarsomere (0.12, 0.07). Hind coxa with punctation only on ventral surface and dorsal outer depression present. Inner spur of hind tibia longer than outer spur (0.22, 0.16), entire surface of hind tibia with dense strong spines clearly differentiated by color and length. Hind telotarsus as equal in length as fourth tarsomere (0.11, 0.10).

**Wings** (Fig. 111H, I). Fore wing with r vein curved; 2RS vein slightly convex to convex; r and 2RS veins forming a weak, even curve at their junction and outer side of junction not forming a stub; 2M vein slightly curved/swollen; distally fore wing [where spectral veins are] with microtrichiae more densely concentrated than the rest of the wing; anal cell 1/3 proximally lacking microtrichiae; subbasal cell with a small smooth area; vein 2CUa absent and vein 2CUb spectral; vein 2 cu-a absent; vein 2-1A present only proximally as tubular vein; tubular vein 1 cu-a curved, incomplete/broken and not reaching the edge of 1-1A vein. Hind wing with vannal lobe wide, subdistally evenly convex, subproximally straightened, and setae present only proximally.

**Metasoma** (Fig. 111A, C, D, F, G). Metasoma laterally compressed. Petiole on T1 finely sculptured only laterally, parallel-sided in proximal half and then narrowing (length 0.27, maximum width 0.15, minimum width 0.08), and with scattered pubescence concentrated in the first distal third. Lateral grooves delimiting the median area on T2 clearly defined and reaching the distal edge of T2 (length median area 0.14, length T2 0.14), edges of median area polished and lateral grooves deep, median area broader than long (length 0.14, maximum width 0.18, minimum width 0.07); T2 with scattered pubescence only distally. T3 longer than T2 (0.18, 0.14) and with scattered pubescence throughout. Pubescence on hypopygium scattered.

**Cocoons.** Gray oval cocoons with evenly smooth silk fibers. Cocoons lightly but individually adhered to caterpillar cuticle.

#### Comments.

Both sexes with slim bodies.

#### Male

(Fig. 112A–L). Coloration and shape similar to female.

#### Etymology.

John (Jack) T. Longino is a professor of Biology at the University of Utah, Salt Lake City, UT, USA. He is a specialist in neotropical myrmecology. His research is focused on understanding how species are distributed on tropical mountainsides, what ecological factors explain the elevational range limits of species, and how species might respond to climate change.

#### Distribution.

The parasitized caterpillar was collected in Costa Rica, ACG, Sector Cacao (Estación Cacao), during March 1998 at 1,150 m in cloud forest.

#### Biology.

The lifestyle of this parasitoid species is gregarious.

#### Host.

*Gonodontapulverea* Schaus (Erebidae: Calpinae), food plant was not reported. Caterpillar was collected in fifth instar.

### Glyptapanteles jamesrobertsoni

Taxon classificationAnimaliaHymenopteraBraconidae

Arias-Penna, sp. nov.

http://zoobank.org/12B7FEB1-7A1C-4E1C-8156-82A5F4C7696E

Figs 113 , 114

#### Female.

Body length 2.02 mm, antenna length 2.83 mm, fore wing length 2.53 mm.

#### Type material.

**Holotype**: COSTA RICA • 1♀; 07-SRNP-42572, DHJPAR0020736; Área de Conservación Guanacaste, Alajuela, Sector Rincón Rain Forest, Río Francia Arriba; 400 m; 10.89666, -85.29003; 06.x.2007; Minor Carmona leg.; caterpillar collected in fifth instar; white cocoons forming two rows cordwood adhered to the leaf substrate; adult parasitoids emerged on 08.x.2004; (CNC). **Paratypes.** • 31 (2♀, 3♂) (26♀, 0♂); 07-SRNP-42572, DHJPAR0020736; same data as for holotype; (CNC).

#### Other material.

**Reared material.** COSTA RICA: *Área de Conservación Guanacaste*, *Alajuela*, *Sector San Cristóbal*, *Sendero Perdido*: • 20 (4♀, 0♂) (16♀, 0♂); 04-SRNP-1228, DHJPAR0000283; rain forest; 620 m; 10.8794, -85.38607; 06.iii.2004; Elda Araya leg.; caterpillar collected in fifth instar; white parallel cordwood cocoons adhered to the leaf substrate; adult parasitoids emerged on 08.x.2004.

*Área de Conservación Guanacaste*, *Alajuela*, *Sector San Cristóbal*, *Quebrada Cementerio*: • 8 (3♀, 0♂) (5♀, 0♂); 04-SRNP-320, DHJPAR0000285; rain forest; 700 m; 10.87124, -85.38749; 15.i.2004; Gloria Sihezar leg.; caterpillar collected in fourth instar; white parallel cordwood cocoons adhered to the leaf substrate; adult parasitoids emerged on 04.ii.2004.

*Área de Conservación Guanacaste*, *Alajuela*, *Sector San Cristóbal*, *Quebrada San Francisco*: • 10 (3♀, 2♂) (5♀, 0♂); 05-SRNP-3214, DHJPAR0004239; rain forest; 690 m; 10.87247, -85.37933; 06.vi.2005; Osvaldo Espinoza leg.; caterpillar collected in third instar; white separate cocoons adhered to the leaf substrate; adult parasitoids emerged on 21.vi.2005.

*Área de Conservación Guanacaste*, *Alajuela*, *Sector San Cristóbal*, *Bosque Trampa Malaise*: • 21 (3♀, 3♂) (15♀, 0♂); 05-SRNP-6502, DHJPAR0004783; rain forest; 815 m; 10.86280, -85.38460; 17.x.2005; Yessenia Mendoza leg.; caterpillar collected in third instar; cordwood of cocoons stacked on each side of cadaver and adhered to the leaf substrate; adult parasitoids emerged on 02.xi.2005.

*Área de Conservación Guanacaste*, *Alajuela*, *Sector Rincón Rain Forest*, *Sendero Rincón*: • 14 (3♀, 1♂) (10♀, 0♂); 05-SRNP-43610, DHJPAR0004768; 430 m; 10.8962, -85.27769; 07.xii.2005; José Pérez leg.; caterpillar collected in third instar; adult parasitoids emerged on 13.xii.2005.

*Área de Conservación Guanacaste*, *Alajuela*, *Sector Rincón Rain Forest*, *Sendero Albergue Crater*: • 41 (3♀, 2♂) (36♀, 0♂); 10-SRNP-5531, DHJPAR0041769; 980 m; 10.84886, -85.3281; 23.ix.2010; Osvaldo Espinoza leg.; caterpillar collected in fifth instar; two rows of cordwood cocoons adhered to the leaf substrate; adult parasitoids emerged on 07.x.2010.

*Área de Conservación Guanacaste*, *Guanacaste*, *Sector Pitilla*, *Sendero Laguna*: • 27 (4♀, 4♂) (11♀, 8♂); 06-SRNP-65592, DHJPAR0012669; rain forest; 680 m; 10.9888, -85.42336; 16.xii.2006; Petrona Rios leg.; caterpillar collected in third instar; white small disordered cordwood cocoons on each side of cadaver, but only approximating, and not tightly glued to leaf, cocoons formed on 29.xii.2006; adult parasitoids emerged on 06.i.2007.

#### Malaise-trapped material.

COSTA RICA: *Área de Conservación Guanacaste*, *Alajuela*, *Sector San Cristóbal*, *Potrero Argentina*: • 1 (1♀, 0♂) (0♀, 0♂); 07-SRNP-67753, DHJPAR0027491; pastures; Malaise; 520 m; 10.89021, -85.38803; 09.viii.2007; DH Janzen & W Hallwachs leg.

*Área de Conservación Guanacaste*, *Alajuela*, *Sector San Cristóbal*, *Bosque Trampa Malaise*: • 1 (0♀, 1♂) (0♀, 0♂); 07-SRNP-67833, DHJPAR0027629; Malaise; rain forest; 815 m; 10.86280, -85.38460; 09.viii.2007; DH Janzen & W Hallwachs leg.

#### Diagnosis.

**Figure 113.**
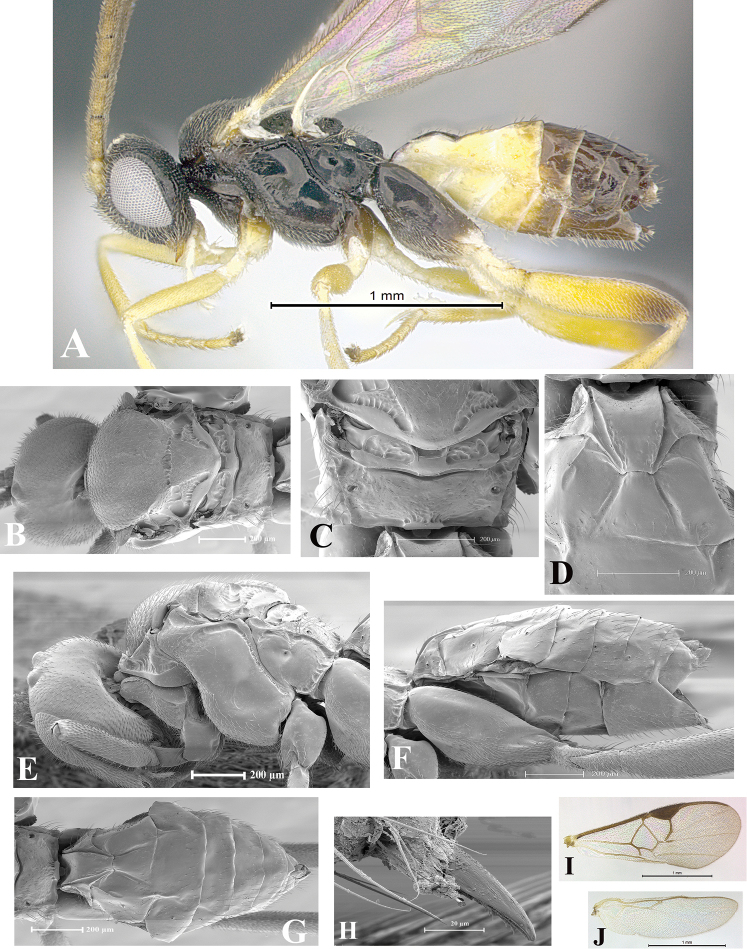
*Glyptapantelesjamesrobertsoni* sp. nov. female 06-SRNP-65592 DHJPAR0012669, 07-SRNP-42572 DHJPAR0020736 **A** Habitus **B, E** Head, mesosoma **B** Dorsal view **E** Lateral view **C** Metanotum, propodeum, dorsal view **D**T1–2, dorsal view **F, G** Metasoma **F** Lateral view **G** Dorsal view **H** Genitalia: hypopygium, ovipositor, ovipositor sheaths, lateral view **I, J** Wings **I** Fore **J** Hind.

**Figure 114.**
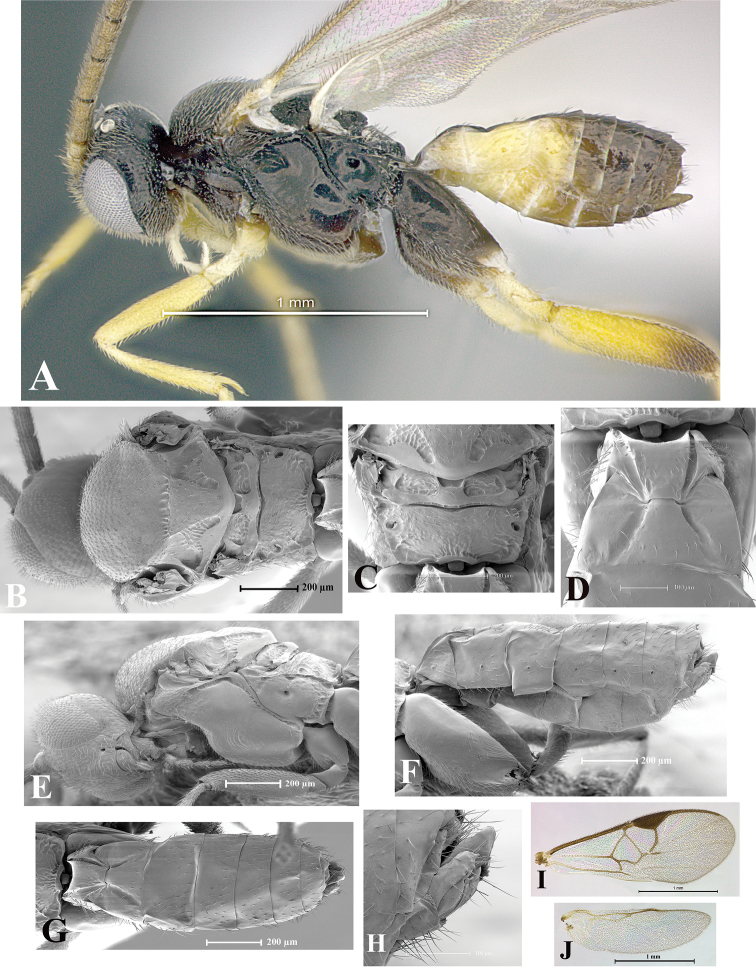
*Glyptapantelesjamesrobertsoni* sp. nov. male 06-SRNP-65592 DHJPAR0012669, 07-SRNP-42572 DHJPAR0020736 **A** Habitus **B, E** Head, mesosoma **B** Dorsal view **E** Lateral view **C** Scutellum, metanotum, propodeum, dorsal view **D**T1–2, dorsal view **F, G** Metasoma **F** Lateral view **G** Dorsal view **H** Genitalia: parameres, lateral view **I, J** Wings **I** Fore **J** Hind.

Face flat or nearly so, area just behind transscutal articulation with a sloped transverse strip (Figs 113B, 114B), ventral margin of fore telotarsus slightly excavated and with a tiny curved seta, distal antennal flagellomere longer than penultimate, inner spur of hind tibia much longer than outer spur, median area on T2 broader than long (Figs 113D, G, 114D, G), propodeal spiracle distally framed by a short concave carina, propodeum without median longitudinal carina (Figs 113C, 114C), petiole on T1 distally with lateral margins relatively straight, finely sculptured only distally (Figs 113D, G, 114D, G), surface of metasternum flat or nearly so, precoxal groove deep with lineate sculpture (Figs 113A, E, 114A, E), fore wing with vein 1 cu-a curved, r vein curved, outer side of junction of r and 2RS veins not forming a stub (Figs 113I, 114I), dorsal outer depression on hind coxa present (Figs 113A, F, 114A, F), inner margin of eyes diverging slightly at antennal sockets, and lateral grooves delimiting the median area on T2 clearly defined and reaching the distal edge of T2 (Figs 113D, G, 114D, G).

#### Coloration

(Fig. 113A). General body coloration brown-black except scape, pedicel, labrum, and mandibles yellow-brown; first three-four proximal antennal flagellomeres dorsally lighter (yellow-brown) than ventrally (brown), remaining flagellomeres brown on both sides; glossa, maxillary and labial palps, and tegulae yellow. Eyes and ocelli silver. Fore and middle legs yellow except yellow-brown/light brown coxae (fore coxae inner side yellow-brown) and brown claws; hind legs yellow except brown coxae, apex of femora brown, distal 1/3 of tibiae and tarsomeres brown. Petiole on T1 with two colorations: proximal half yellow and distal half black, and contours darkened; T2 with median and wide adjacent areas black, and sublateral ends yellow; T3 mostly black forming a triangle that looks like a continuum of pyramid of median area on T2, and lateral ends yellow; T4 and beyond completely brown; distally each tergum with a narrow yellowish transparent band. In lateral view, T1–3 and S1–3 completely yellow, remaining terga and sterna completely dark brown.

#### Description.

**Head** (Fig. 113A, B, E). Head rounded with short and dense pubescence. Proximal three antennal flagellomeres longer than wide (0.19:0.07, 0.19:0.07, 0.19:0.07), distal antennal flagellomere longer than penultimate (0.12:0.06, 0.09:0.06), antenna longer than body (2.83, 2.02); antennal scrobes-frons shallow. Face flat or nearly so, with dense fine punctations, interspaces smooth and longitudinal median carina present. Frons smooth. Temple wide, punctate and interspaces clearly smooth. Inner margin of eyes diverging slightly at antennal sockets; in lateral view, eye anteriorly convex and posteriorly straight, POL shorter than OOL (0.09, 0.11). Malar suture present. Median area between lateral ocelli slightly depressed. Vertex laterally rounded and dorsally wide.

**Mesosoma** (Fig. 113A–C, E). Mesosoma dorsoventrally convex. Distal 1/3 of mesoscutum with lateral margin slightly dented, punctation distinct proximally with polished area distally, interspaces with microsculpture. Scutellum triangular, apex sloped and fused with BS, scutellar punctation scattered throughout, in profile scutellum flat and on same plane as mesoscutum, phragma of the scutellum partially exposed; BS only very partially overlapping the MPM; ATS demilune with a little, complete parallel carinae; dorsal ATS groove with semicircular/parallel carinae. Transscutal articulation with small and heterogeneous foveae, area just behind transscutal articulation with a smooth and shiny sloped transverse strip. Metanotum with BM wider than PFM (clearly differentiated); MPM semicircular without median longitudinal carina; AFM without setiferous lobes and not as well delineated as PFM; PFM thick, smooth and with a distal flat flange; ATM proximally with semircular/undulate carina and distally smooth. Propodeum without median longitudinal carina, proximal half curved with medium-sized sculpture and distal half relatively polished and with a shallow dent at each side of nucha; distal edge of propodeum with a flange at each side and without stubs; propodeal spiracle distally framed by a short concave carina; nucha surrounded by very short radiating carinae. Pronotum with a distinct dorsal furrow, dorsally with a well-defined smooth band; central area of pronotum smooth, but both dorsal and ventral furrows with short parallel carinae. Propleuron with a mix of rugae and fine punctation, dorsally with a carina. Metasternum flat or nearly so. Contour of mesopleuron straight/angulate or nearly so; precoxal groove deep with faintly transverse lineate sculpture; epicnemial ridge elongated more fusiform (tapering at both ends).

**Legs.** Ventral margin of fore telotarsus slightly excavated and with a tiny curved seta, fore telotarsus almost same width throughout and longer than fourth tarsomere (0.12, 0.06). Hind coxa with punctation only on ventral surface and dorsal outer depression present. Inner spur of hind tibia longer than outer spur (0.21, 0.16), entire surface of hind tibia with dense strong spines clearly differentiated by color and length. Hind telotarsus as equal in length as fourth tarsomere (0.10, 0.10).

**Wings** (Fig. 113I, J). Fore wing with r vein slightly curved; 2RS vein straight; r and 2RS veins forming a weak, even curve at their junction and outer side of junction not forming a stub; 2M vein slightly curved/swollen; distally fore wing [where spectral veins are] with microtrichiae more densely concentrated than the rest of the wing; anal cell 1/3 proximally lacking microtrichiae; subbasal cell with a small smooth area; vein 2CUa absent and 2CUb spectral; vein 2 cu-a absent; vein 2-1A present only proximally as tubular vein; tubular vein 1 cu-a curved, incomplete/broken and not reaching the edge of 1-1A vein. Hind wing with vannal lobe narrow, subdistally evenly convex and subproximally straightened, and setae present only proximally.

**Metasoma** (Fig. 113A, D, F–H). Metasoma laterally compressed. Petiole on T1 finely sculptured only distally, virtually parallel-sided over most of length, but narrowing over distal 1/3 (length 0.31, maximum width 0.16, minimum width 0.08) and with scattered pubescence concentrated in the first distal third. Lateral grooves delimiting the median area on T2 clearly defined and reaching the distal edge of T2 (length median area 0.15, length T2 0.15), edges of median area polished and lateral grooves deep, median area broader than long (length 0.15, maximum width 0.20, minimum width 0.07), T2 with scattered pubescence only distally. T3 longer than T2 (0.21, 0.15) and with scattered pubescence only distally. Pubescence on hypopygium dense.

**Cocoons.** White oval cocoons with silk fibers that are messy/disordered/fluffy. Two rows of cordwood cocoons on each side of cadaver caterpillar and adhered to the leaf substrate.

#### Comments.

Both sexes with slim bodies.

#### Male

(Fig. 114A–J). Coloration and shape similar to female.

#### Etymology.

James Robertson is a coleopterologist interested in biodiversity, evolution, and ecology of Cucujoidea [Erotylidae (pleasing fungus beetles), Bothrideridae (ectoparasitic, cocoon-forming beetles), Cerylonidae (minute fungus beetles), Corylophidae (minute hooded beetles), and Discolomatidae (Mexican hat beetles)]. He is a postdoctoral associate at the University of Arizona, Tucson, AZ, USA.

#### Distribution.

Parasitized caterpillars were collected in Costa Rica, ACG, Sector Pitilla (Sendero Laguna), Sector Rincón Rain Forest (Río Francia Arriba, Sendero Albergue Crater, and Sendero Rincón), and Sector San Cristóbal (Bosque Trampa Malaise, Sendero Perdido, Quebrada Cementerio, and Quebrada San Francisco), during January and March 2004, June, October, and December 2005, December 2006, October 2007, and November 2010 at 400 m, 430 m, 620 m, 690 m, and 700 m in rain forest.

Adult parasitoids were collected in Costa Rica, ACG, Sector San Cristóbal (Bosque Trampa Malaise and Potrero Argentina), during August 2007 at 520 m and 815 m in pasture and rain forest.

#### Biology.

The lifestyle of this parasitoid species is gregarious.

#### Host.

*Antiblemma* sp. Hübner (Erebidae: Eulepidotinae) feeding on *Psychotriachagrensis*, *P.graciliflora*, and *Psychotriapanamensis* (Rubiaceae). Caterpillars were collected in third, fourth and fifth instar.

### Glyptapanteles jaquioconnorae

Taxon classificationAnimaliaHymenopteraBraconidae

Arias-Penna, sp. nov.

http://zoobank.org/DB74CD3E-1A46-465F-B16A-E000F7FF1113

Fig. 115

#### Female.

Body length 3.13 mm, antenna length 3.53 mm, fore wing length 3.33 mm.

#### Type material.

**Holotype**: ECUADOR • 1♀; EC-2997, YY-A025; Napo, Yanayacu Biological Station, Yanayacu Road; cloud forest; 2,100 m; -0.566667, -77.866667; 29.v.2005; CAPEA leg.; caterpillar instar not reported; adult parasitoid emerged on 07.vi.2005; (PUCE).

#### Diagnosis.

**Figure 115.**
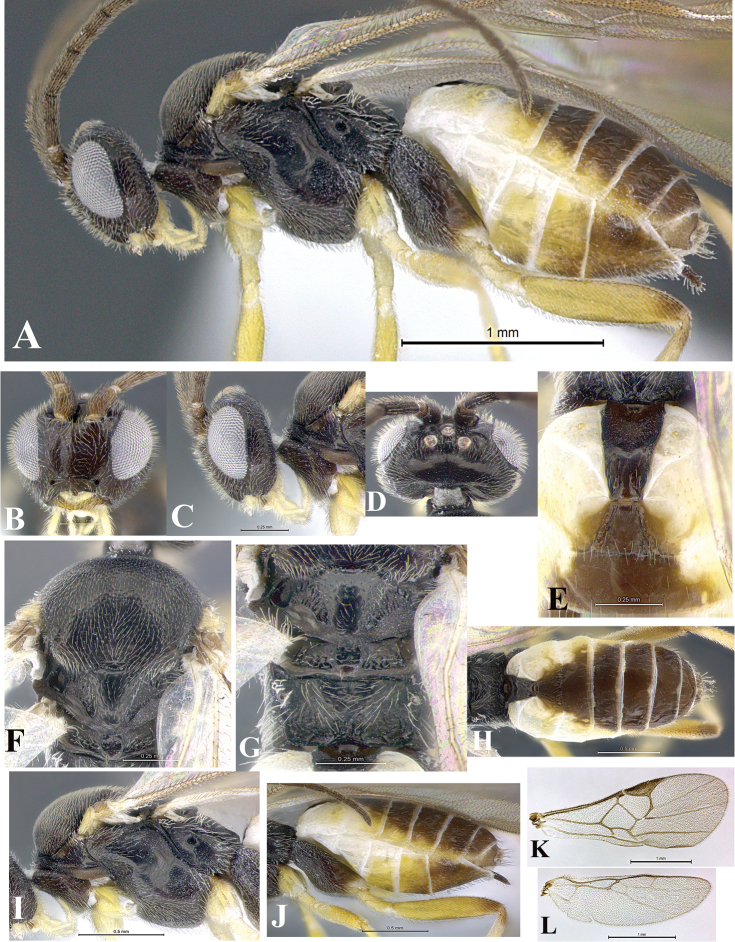
*Glyptapantelesjaquioconnorae* sp. nov. female EC-2997 YY-A025 **A** Habitus **B, D** Head **B** Frontal view **D** Dorsal view **C** Head, pronotum, propleuron, lateral view **E**T1–3, dorsal view **F** Mesonotum, dorsal view **G** Scutellum, metanotum, propodeum, dorsal view **H, J** Metasoma **H** Dorsal view **J** Lateral view **I** Mesosoma, lateral view **K, L** Wings **K** Fore **L** Hind.

Distal 1/4 of mesoscutum with a central dent (Fig. 115F), medioposterior band of scutellum only very partially overlapping the medioanterior pit of metanotum (Fig. 115F, G), median area on T2 slightly longer than broad, lateral grooves delimiting the median area clearly defined and reaching the distal edge of T2, median area distally with lateral margins relatively straight, edges of median area polished and followed by a deep groove (Fig. 115E, H), scutellum in profile flat, fore wing with vein 2-1A tubular throughout, r vein curved, outer side of junction of r and 2RS veins forming a distinct stub (Fig. 115K), dorsal carina delimiting a dorsal furrow on propleuron absent (Fig. 115C, I), anterior furrow of metanotum without setiferous lobes (Fig. 115F, G), axillary trough of scutellum with sculpture (Fig. 115F, G), propodeum without median longitudinal carina (Fig. 115G), and anteroventral contour of mesopleuron convex (Fig. 115A, I).

#### Coloration

(Fig. 115A–L). General body coloration brown-black although propleuron, dorsal and ventral furrows of pronotum, ventrally mesopleuron, distal corners of mesoscutum, lunules, and PFM with light brown/reddish tints; labrum, glossa, maxillary and labial palps, and tegulae yellow; mandibles, scape and pedicel yellow-brown. Eyes silver and ocelli brownish. Fore and middle legs dark yellow, although tarsomeres with a brownish tint; hind legs dark yellow except coxae dark brown with apex light brown, femora apically with a tiny brown dot, tibiae with 1/3 distal brown and proximally with a narrow brown band, and tarsomeres light brown, but basitarsus proximally with a narrow yellow band. Petiole on T1 brown, contours darkened and sublateral areas ivory/pale yellow; T2 with median area brown, contours darkened, median and wide adjacent areas brown, and lateral ends ivory/pale yellow; T3 brown with proximal corners ivory/pale yellow, and distal corners each one with a oval pale ivory/pale yellow spots; T4 and beyond completely brown; distally each tergum with a yellowish transparent band. In lateral view, T1–2 completely ivory/pale yellow; T3 and beyond yellow, but dorsally brown, extent of brown area increasing slightly from proximal to distal. S1–3 ivory/yellow; S4–5 yellow, but medially brown; hypopygium yellow-brown.

#### Description.

**Head** (Fig. 115A–D). Head rounded with pubescence long and dense. Proximal three antennal flagellomeres longer than wide (0.25:0.07, 0.26:0.07, 0.25:0.07), distal antennal flagellomere longer than penultimate (0.16:0.05, 0.11:0.05), antenna longer than body (3.53, 3.13); antennal scrobes-frons shallow. Face with fine and punctate-lacunose sculpture, lateral depression only middle, interspaces wavy and longitudinal median carina present. Frons smooth. Temple wide, punctate and interspaces clearly smooth. Inner margin of eyes diverging slightly at antennal sockets; in lateral view, eye anteriorly convex and posteriorly straight. POL shorter than OOL (0.09, 0.13). Malar suture absent or difficult to see. Median area between lateral ocelli slightly depressed. Vertex laterally rounded and dorsally wide.

**Mesosoma** (Fig. 115A, F, G, I). Mesosoma dorsoventrally convex. Mesoscutum 1/4 distal with a central dent, punctation distinct throughout, interspaces wavy/lacunose. Scutellum triangular, apex sloped and fused with BS, but not in the same plane, scutellar punctation scattered throughout, in profile scutellum flat and on same plane as mesoscutum, phragma of the scutellum completely concealed; BS only very partially overlapping the MPM; ATS demilune with complete undulate/reticulate carinae; dorsal ATS groove with carinae only proximally. Transscutal articulation with small and heterogeneous foveae, area just behind transscutal articulation sloped and with same kind of sculpture as mesoscutum. Metanotum with BM upward; MPM oval/circular with a short proximal carina; AFM without setiferous lobes and not as well delineated as PFM; PFM thick and smooth; ATM proximally with a groove with some sculpturing and distally smooth. Propodeum without median longitudinal carina, proximal half weakly curved with fine sculpture and distal half slightly rugose; distal edge of propodeum with a flange at each side and without stubs; propodeal spiracle without distal carina; nucha surrounded by very short radiating carinae. Pronotum with a distinct dorsal furrow, dorsally with a well-defined smooth band; central area of pronotum and dorsal furrow smooth, but ventral furrow with short parallel carinae. Propleuron with fine punctations throughout and dorsally without a carina. Metasternum convex. Contour of mesopleuron convex; precoxal groove deep, smooth and shiny; epicnemial ridge convex, teardrop-shaped.

**Legs.** Ventral margin of fore telotarsus entire without seta, fore telotarsus almost same width throughout and longer than fourth tarsomere (0.12, 0.09). Hind coxa finely punctate throughout, and dorsal outer depression present. Inner spur of hind tibia longer than outer spur (0.24, 0.20), entire surface of hind tibia with dense strong spines clearly differentiated by color and length. Hind telotarsus longer than fourth tarsomere (0.16, 0.13).

**Wings** (Fig. 115K, L). Fore wing with r vein slightly curved; 2RS vein straight; r and 2RS veins forming a weak, even curve at their junction and outer side of junction forming a slight stub; 2M vein straight; distally fore wing [where spectral veins are] with microtrichiae more densely concentrated than the rest of the wing; anal cell 1/3 proximally lacking microtrichiae; subbasal cell with microtrichiae virtually throughout; veins 2CUa and 2CUb completely spectral; vein 2 cu-a present as spectral vein, sometimes difficult to see; vein 2-1A tubular throughout; tubular vein 1 cu-a curved, incomplete/broken and not reaching the edge of 1-1A vein. Hind wing with vannal lobe very narrow, subdistally and subproximally evenly convex, and setae evenly scattered in the margin.

**Metasoma** (Fig. 115A, E, H, K). Metasoma cylindrical. Petiole on T1 finely sculptured only distally, parallel-sided in proximal half and then narrowing (length 0.40, maximum width 0.22, minimum width 0.12), and with scattered pubescence concentrated in the first distal third. Lateral grooves delimiting the median area on T2 clearly defined and reaching the distal edge of T2 (length median area 0.22, length T2 0.22), lateral grooves deep, median area longer than broad (length 0.22, maximum width 0.20, minimum width 0.08); T2 with pubescence only distally. T3 longer than T2 (0.27, 0.22) and with scattered pubescence only distally. Pubescence on hypopygium dense.

**Cocoons.** Unknown.

#### Comments.

Some of the first proximal antennal flagellomeres seem to have more of three dark bands (multi-rings). A whole specimen was used for DNA extraction.

#### Male.

Unknown.

#### Etymology.

Jaqueline (Jaqui) Megan O’Connor is from United Kingdom. As a graduate student at UIUC, IL, USA she worked on systematics and host use of *Parapanteles* and *Cotesia* from Ecuador and Costa Rica. Currently, she is a biology teacher at a school called Christ’s Hospital, Horsham, West Sussex, England, and dedicated to social change.

#### Distribution.

Parasitized caterpillar was collected in Ecuador, Napo, Yanayacu Biological Station (Yanayacu Road), during May 2005 at 2,100 m in cloud forest.

#### Biology.

The lifestyle of this parasitoid species is gregarious.

#### Host.

Undetermined species of Nymphalidae (Ithomiinae) feeding on undetermined species of Solanaceae. Caterpillar instar was not reported.

### Glyptapanteles jeremydewaardi

Taxon classificationAnimaliaHymenopteraBraconidae

Arias-Penna, sp. nov.

http://zoobank.org/90823A6B-7B97-4FAF-B59F-347C28CB587F

Figs 116 , 117

#### Female.

Body length 2.27 mm, antenna length 2.68 mm, fore wing length 2.63 mm.

#### Type material.

**Holotype**: COSTA RICA • 1♀; 06-SRNP-35622, DHJPAR0012114; Área de Conservación Guanacaste, Guanacaste, Sector Cacao, Estación Cacao; cloud forest; 1,150 m; 10.92691, -85.46822; 04.vii.2006; Manuel Pereira leg.; caterpillar collected in fourth instar; cordwood of cocoons on each side of the caterpillar adhered to the leaf substrate and formed on 10.vii.2006; adult parasitoids emerged on 17.vii.2006; (CNC). **Paratypes.** • 30 (2♀, 3♂) (23♀, 2♂); 06-SRNP-35622, DHJPAR0012114; same data as for holotype; (CNC).

#### Other material.

**Reared material.** COSTA RICA: *Área de Conservación Guanacaste*, *Guanacaste*, *Sector Cacao*, *Sendero Circular*: • 47 (5♀, 4♂) (25♀, 13♂); 02-SRNP-9910, DHJPAR0000033; cloud forest; 1,185 m; 10.92714, -85.46683; 01.vii.2002; Freddy Quesada leg.; caterpillar collected in fourth instar; white medium small cocoons, jumbled and lightly adhered to each other adhered to the leaf substrate, cocoons formed on 01.vii.2002; adult parasitoids emerged on 12.vii.2002.

*Área de Conservación Guanacaste*, *Guanacaste*, *Sector Cacao*, *Estación Cacao*: • 17 (2♀, 2♂) (11♀, 1♂); 05-SRNP-35658, DHJPAR0004237; cloud forest; 1,150 m; 10.92691, -85.46822; 27.vi.2005; Manuel Pereira leg.; caterpillar collected in fourth instar; thin white cylinders, not grouped together adhered to the leaf substrate and formed on 30.vi.2005; adult parasitoids emerged on 30.vi.2010.

*Área de Conservación Guanacaste*, *Guanacaste*, *Sector Pailas*, *Gemelos*: • 25 (3♀, 3♂) (13♀, 6♂); 09-SRNP-56293, DHJPAR0039960; dry forest; 1,276 m; 10.76928, -85.34662; 02.vi.2009; Mariano Pereira leg.; caterpillar collected in fifth instar; two rows of cordwood cocoons adhered to the leaf substrate and formed on 04.vi.2009; adult parasitoids emerged on 07.vii.2009, 08.vii.2009.

#### Diagnosis.

**Figure 116.**
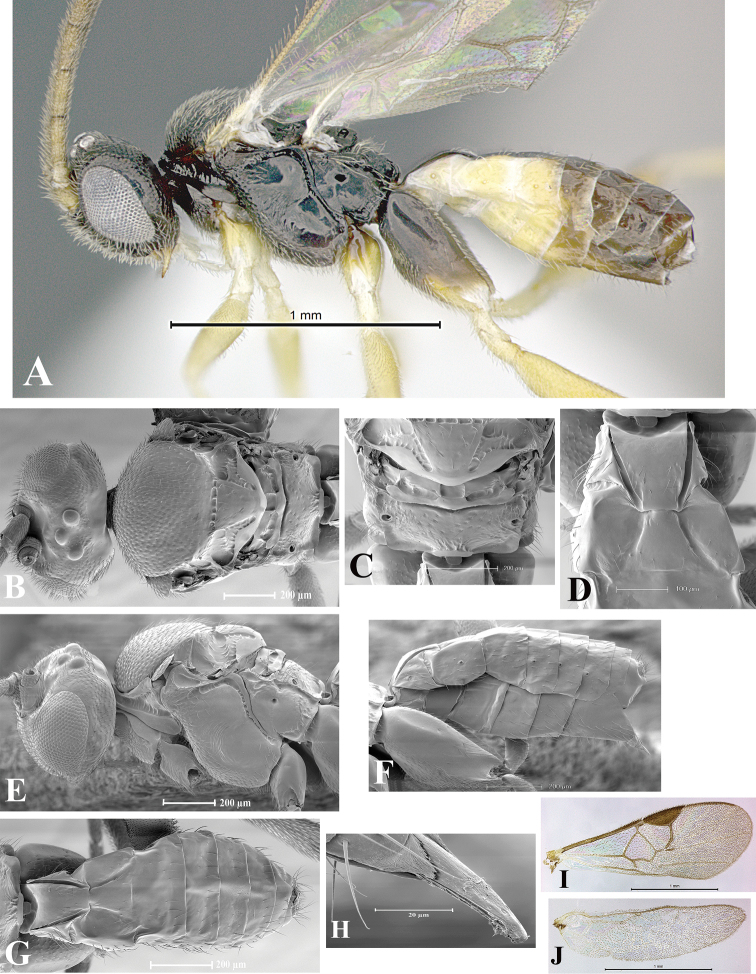
*Glyptapantelesjeremydewaardi* sp. nov. female 02-SRNP-9910 DHJPAR0000033, 06-SRNP-35622 DHJPAR0012114 **A** Habitus **B, E** Head, mesosoma **B** Dorsal view **E** Lateral view **C** Metanotum, propodeum, dorsal view **D**T1–2, dorsal view **F, G** Metasoma **F** Lateral view **G** Dorsal view **H** Genitalia: hypopygium, ovipositor, ovipositor sheaths, lateral view **I, J** Wings **I** Fore **J** Hind.

**Figure 117.**
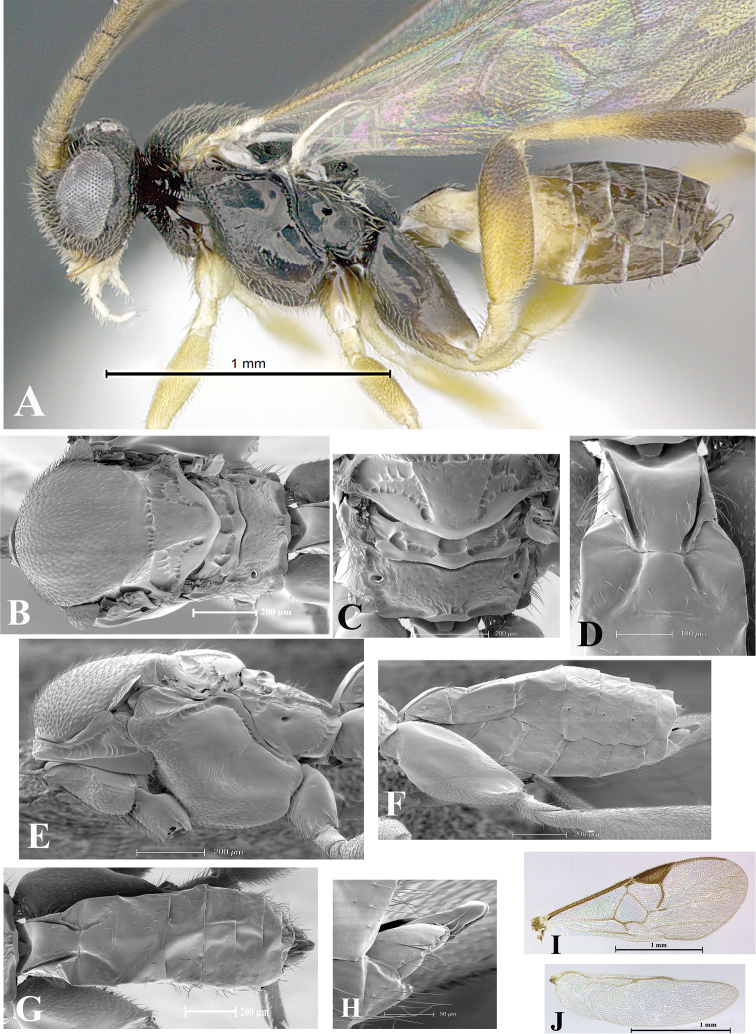
*Glyptapantelesjeremydewaardi* sp. nov. male 02-SRNP-9910 DHJPAR0000033, 06-SRNP-35622 DHJPAR0012114 **A** Habitus **B, E** Mesosoma **B** Dorsal view **E** Lateral view **C** Scutellum, metanotum, propodeum, dorsal view **D**T1–2, dorsal view **F, G** Metasoma **F** Lateral view **G** Dorsal view **H** Genitalia: parameres, lateral view **I, J** Wings **I** Fore **J** Hind.

Fore wing with r vein straight, outer side of junction of r and 2RS veins not forming a stub (Figs 116I, 117I), distal antennal flagellomere subequal in length with penultimate, petiole on T1 evenly narrowing distally, completely smooth and polished, with faint, satin-like sheen (Figs 116D, G, 117D), propodeum without median longitudinal carina (Figs 116C, 117C), and lateral grooves delimiting the median area on T2 clearly defined and reaching the distal edge of T2 (Figs 116D, 117D).

#### Coloration

(Fig. 116A). General body coloration dark brown, although ventrally body lighter than dorsally; scape, pedicel, labrum, and mandibles yellow-brown; first four-five proximal antennal flagellomeres dorsally lighter (yellow-brown) than ventrally (brown), remaining flagellomeres brown on both sides; glossa, maxillary and labial palps, and tegulae yellow. Eyes and ocelli silver. Fore and middle legs yellow except mid coxae which proximally with a small area light brown, and claws brown; hind legs yellow except dark brown coxae only distally yellow (yellow coloration is more extensive on the inner side), apex of femora and tibia, and tarsomeres light brown. Petiole on T1 brown with a middle yellow spot and contours brown; T2 with median and adjacent areas black, and lateral ends yellow; T3 mostly black and lateral ends yellow; T4 and beyond completely brown; distally each tergum with a narrow whitish transparent band. In lateral view, T1–3 yellow; T4 and beyond brown. S1–4 yellow; penultimate sternum proximal half yellow, distal half brown; hypopygium brown, but medially yellow-brown.

#### Description.

**Head** (Fig. 116A, B, E). Head rounded with pubescence short and dense. Proximal three antennal flagellomeres longer than wide (0.21:0.05, 0.19:0.05, 0.19:0.05), distal antennal flagellomere subequal in length with penultimate (0.12:0.05, 0.11:0.05), antenna longer than body (2.68, 2.27); antennal scrobes-frons shallow. Face flat or nearly so, with punctate-lacunose sculpture, interspaces smooth and longitudinal median carina present. Frons smooth. Temple wide, punctate and interspaces clearly smooth or wavy. Inner margin of eyes diverging slightly at antennal sockets; in lateral view, eye anteriorly convex and posteriorly straight. POL shorter than OOL (0.10, 0.12). Malar suture present. Median area between lateral ocelli slightly depressed. Vertex laterally rounded and dorsally wide.

**Mesosoma** (Fig. 116A–C, E). Mesosoma dorsoventrally convex. Mesoscutum proximally convex and distally flat, punctation distinct throughout, interspaces wavy/lacunose. Scutellum triangular, apex sloped and fused with BS, scutellar punctation scattered throughout, in profile scutellum slightly convex, but on same plane as mesoscutum, phragma of the scutellum partially exposed; BS only very partially overlapping the MPM; ATS demilune with short stubs delineating the area; dorsal ATS groove with carinae only proximally. Transscutal articulation with small and homogeneous foveae, area just behind transscutal articulation with a smooth and shiny sloped transverse strip. Metanotum with BM wider than PFM (clearly differentiated); MPM circular without median longitudinal carina; AFM without setiferous lobes and not as well delineated as PFM; PFM thick, smooth and with a distal flat flange; ATM proximally with a groove with some sculpturing and distally smooth. Propodeum without median longitudinal carina, proximal half curved with medium-sized sculpture and distal half rugose; distal edge of propodeum with a flange at each side and without stubs; propodeal spiracle distally framed by faintly concave/wavy carina; nucha surrounded by very short radiating carinae. Pronotum with a distinct dorsal furrow, dorsally with a well-defined smooth band; central area of pronotum smooth, but both dorsal and ventral furrows with short parallel carinae. Propleuron rugose and dorsally with a carina. Metasternum flat or nearly so. Contour of mesopleuron convex; precoxal groove deep with transverse lineate sculpture; epicnemial ridge elongated more fusiform.

**Legs.** Ventral margin of fore telotarsus slightly excavated and with a tiny curved seta, fore telotarsus almost same width throughout and longer than fourth tarsomere (0.15, 0.07). Hind coxa with punctation only on ventral surface and dorsal outer depression present. Inner spur of hind tibia longer than outer spur (0.21, 0.16), entire surface of hind tibia with dense strong spines clearly differentiated by color and length. Hind telotarsus longer than fourth tarsomere (0.15, 0.11).

**Wings** (Fig. 116I, J). Fore wing with r vein straight; 2RS vein straight; r and 2RS veins forming an angle at their junction and outer side of junction not forming a stub; 2M vein slightly curved/swollen; distally fore wing [where spectral veins are] with microtrichiae more densely concentrated than the rest of the wing; anal cell 1/3 proximally lacking microtrichiae; subbasal cell with a small smooth area; vein 2CUa absent and vein 2CUb spectral; vein 2 cu-a absent; vein 2-1A present only proximally as tubular vein; tubular vein 1 cu-a curved and complete, but junction with 1-1A vein spectral. Hind wing with vannal lobe narrow, subdistally and subproximally straightened, and setae present only proximally.

**Metasoma** (Fig. 116A, D, F–H). Metasoma laterally compressed. Petiole on T1 completely smooth and polished, with faint, satin-like sheen, evenly narrowing distally (length 0.25, maximum width 0.16, minimum width 0.08) and with scattered pubescence concentrated in the first distal third. Lateral grooves delimiting the median area on T2 clearly defined and reaching the distal edge of T2 (length median area 0.16, length T2 0.16), edges of median area polished and lateral grooves deep, median area as broad as long (length 0.16, maximum width 0.18, minimum width 0.06); T2 with scattered pubescence only distally. T3 longer than T2 (0.20, 0.16) and with scattered pubescence only distally. Pubescence on hypopygium dense.

**Cocoons** (Fig. 4Y). White oval cocoons with evenly smooth silk fibers. Two rows of cordwood cocoons on each side of the caterpillar and adhered to the leaf substrate.

#### Comments.

Both sexes with slim bodies.

#### Male

(Fig. 117A–J). Similar in coloration and shape to female.

#### Etymology.

Jeremy Ryan deWaard is an associate director, Collections at the Centre for Biodiversity Genomics, Biodiversity Institute of Ontario (BIO), University of Guelph, Ontario, Canada. He is interested in how barcoding might enhance biosurveillance programs.

#### Distribution.

Parasitized caterpillars were collected in Costa Rica, ACG, Sector Cacao (Estación Cacao and Sendero Circular) and Sector Pailas (Gemelos), during July 2002 and 2006, and June 2005 and 2009 at 1,150 m and 1,185 m in cloud forest.

#### Biology.

The lifestyle of this parasitoid species is gregarious.

#### Host.

*Antiblemma* sp. Hübner (Erebidae: Eulepidotinae) feeding on *Psychotriahorizontalis* (Rubiaceae). Caterpillars were collected in fourth and fifth instar.

### Glyptapanteles jerrypowelli

Taxon classificationAnimaliaHymenopteraBraconidae

Arias-Penna, sp. nov.

http://zoobank.org/49B2C6FF-E9F5-46C7-A8D1-C4B7CA1119C3

Figs 118 , 119

#### Female.

Body length 2.53 mm, antenna length 3.08 mm, fore wing length 2.85 mm.

#### Type material.

**Holotype**: ECUADOR • 1♀; EC-19802, YY-A050; Napo, Yanayacu Biological Station, Yanayacu Road; cloud forest; 2,100 m; -0.566667, -77.866667; 15.xii.2006; Lee Dyer leg.; caterpillar collected in second instar; cocoons formed on 06.i.2007; adult parasitoids emerged 15.i.2007; (PUCE). **Paratypes.** • 3 (1♀, 1♂) (1♀, 0♂); EC-19802, YY-A050; same data as for holotype; (PUCE).

#### Diagnosis.

**Figure 118.**
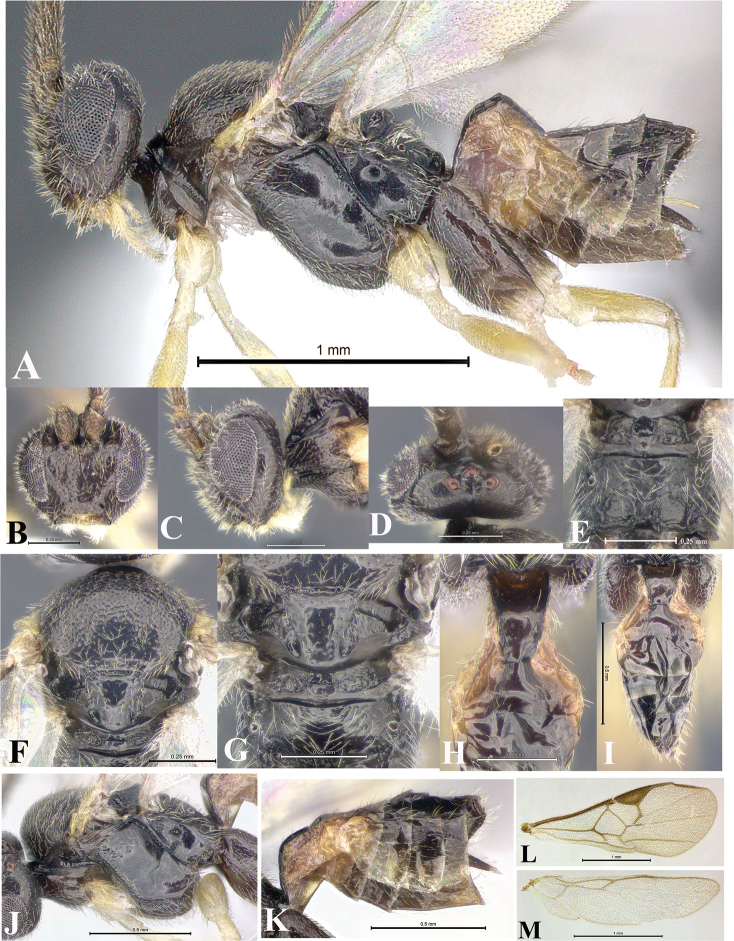
*Glyptapantelesjerrypowelli* sp. nov. female EC-19802 YY-A050 **A** Habitus **B, D** Head **B** Frontal view **D** Dorsal view **C** Head, pronotum, propleuron, lateral view **E** Metanotum, propodeum, dorsal view **F** Mesonotum, dorsal view **G** Scutellum, metanotum, dorsal view **H**T1–3, dorsal view **I, K** Metasoma **I** Dorsal view **K** Lateral view **J** Mesosoma, lateral view **L, M** Wings **L** Fore **M** Hind.

**Figure 119.**
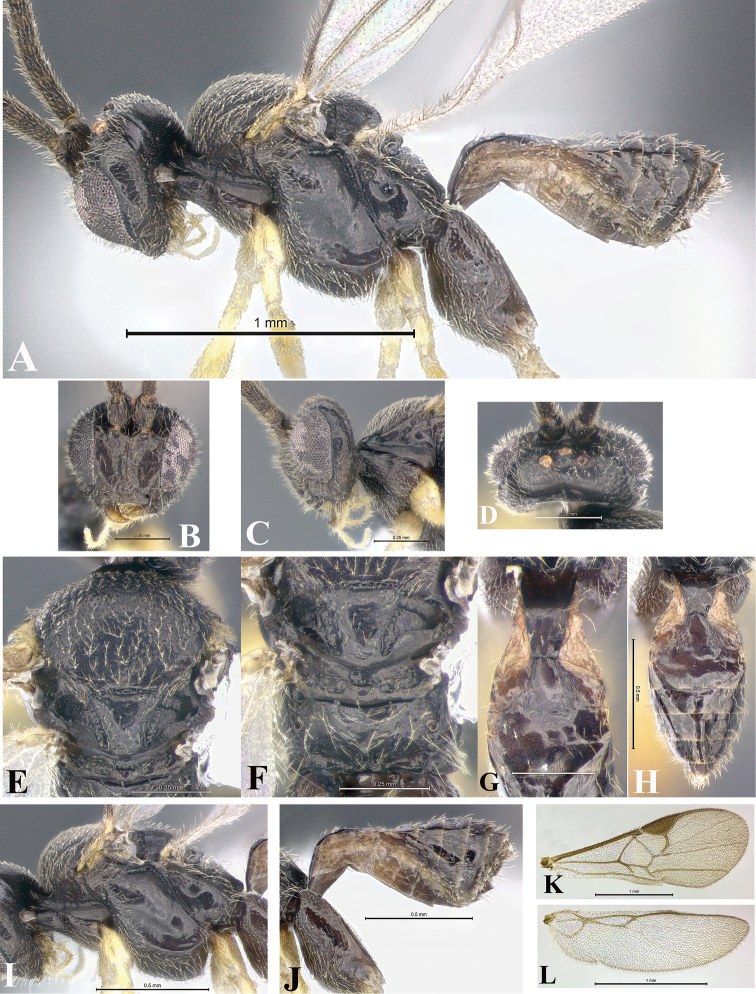
*Glyptapantelesjerrypowelli* sp. nov. male EC-19802 YY-A050 **A** Habitus **B, D** Head **B** Frontal view **D** Dorsal view **C** Head, pronotum, propleuron, lateral view **E** Mesonotum, dorsal view **F** Scutellum, metanotum, propodeum, dorsal view **G**T1–3, dorsal view **H, J** Metasoma **H** Dorsal view **J** Lateral view **I** Mesosoma, lateral view **K, L** Wings **K** Fore **L** Hind.

Median area on T2 as broad as long (Figs 118H, 119G), vertex in dorsal view quite wide (Figs 118D, 119D), edges of median area on T2 obscured by weak longitudinal stripes (Figs 118H, 119G) and lateral grooves delimiting the median area on T2 distally losing definition on T2 (Figs 118I, 119H), in lateral view, metasoma laterally compressed (Figs 118A, 119A), T3 longer than T2 (Figs 118I, 119H), inner margin of eyes diverging slightly at antennal sockets (Figs 118B, 119B), petiole on T1 evenly narrowing distally (wide base to a narrow apex, Figs 118H, 119G) and finely sculptured (Figs 118H, 119G), propodeum without a median longitudinal dent (Figs 118G, 119F), and fore wing with r vein straight, outer side of junction of r and 2RS veins forming a stub (Figs 118L, 119K).

#### Coloration

(Fig. 118A–M). General body coloration brown-to-reddish except all antennal flagellomeres dark brown on both sides; labrum and mandibles yellow-brown; glossa, maxillary and labial palps, and tegulae yellow. Eyes gray/black and ocelli brownish/reddish (in preserved specimen). Fore and middle legs dark yellow, tarsomeres with a brownish tint, and claws brown; hind legs dark yellow except dark brown coxae, femora apically with a tiny brown dot, tibiae with distal half brown and proximally with a narrow brown band, and tarsomeres brown. Petiole on T1 light brown, contours darkened and sublateral areas yellow-brown; T2 with median area and lateral ends brown; T3 and beyond completely brown; distally each tergum with a yellowish transparent band barely noticeable. In lateral view, T1–2 completely brown-reddish; T3 brown-reddish, but dorsally brown; T4 and beyond completely brown. S1–3 brown-reddish; S4 and beyond completely brown.

#### Description.

**Head** (Fig. 118A–D). Head rounded with pubescence long and dense. Proximal three antennal flagellomeres longer than wide (0.22:0.05, 0.21:0.05, 0.22:0.05), distal antennal flagellomere longer than penultimate (0.13:0.45, 0.11:0.45), antenna longer than body (3.08, 2.53); antennal scrobes-frons sloped and forming a shelf. Face convex, dense and finely punctate, interspaces smooth and longitudinal median carina present. Frons rugose. Temple wide, punctations barely noticeable and interspaces clearly smooth. Inner margin of eyes diverging slightly at antennal sockets; in lateral view, eye anteriorly convex and posteriorly straight. POL shorter than OOL (0.08, 0.13). Malar suture faint. Median area between lateral ocelli without depression. Vertex laterally rounded and dorsally quite wide.

**Mesosoma** (Fig. 118A, E–G, J). Mesosoma dorsoventrally convex. Mesoscutum proximally convex and distally flat, punctation distinct throughout, interspaces wavy/lacunose. Scutellum shield-shaped, apex sloped and fused with BS, but not in the same plane, scutellar punctation distinct peripherally and absent centrally, in profile scutellum flat and on same plane as mesoscutum, phragma of the scutellum partially exposed; BS only very partially overlapping the MPM; ATS demilune inner side with a row of foveae; dorsal ATS groove with carinae only proximally. Transscutal articulation with small and heterogeneous foveae, area just behind transscutal articulation nearly at the same level as mesoscutum (flat) and with same kind of sculpture as mesoscutum. Metanotum with BM upward; MPM semicircular without median longitudinal carina; AFM without setiferous lobes and not as well delineated as PFM; PFM thick, smooth and with lateral ends rounded; ATM proximally with sculpture distally without a well delimited smooth area. Propodeum without median longitudinal carina, proximal half weakly curved with medium-sized sculpture and distal half with a shallow dent at each side of nucha; distal edge of propodeum with a flange at each side and without stubs; propodeal spiracle without distal carina; nucha surrounded by long radiating carinae. Pronotum with a faint dorsal furrow, dorsally with a well-defined smooth band; central area of pronotum and dorsal furrow smooth, but ventral furrow with short parallel carinae. Propleuron finely sculptured only ventrally and dorsally without a carina. Metasternum convex. Contour of mesopleuron convex; precoxal groove smooth, shiny and shallow, but visible; epicnemial ridge elongated more fusiform (tapering at both ends).

**Legs.** Ventral margin of fore telotarsus entire without seta, fore telotarsus almost same width throughout and longer than fourth tarsomere (0.11, 0.08). Hind coxa finely punctate throughout, and dorsal outer depression present. Inner spur of hind tibia longer than outer spur (0.20, 0.16), entire surface of hind tibia with dense strong spines clearly differentiated by color and length. Hind telotarsus longer than fourth tarsomere (0.14, 0.11).

**Wings** (Fig. 118L, M). Fore wing with r vein straight; 2RS vein straight; r and 2RS veins forming a weak, even curve at their junction and outer side of junction forming a slight stub; 2M vein slightly curved/swollen; distally fore wing [where spectral veins are] with microtrichiae more densely concentrated than the rest of the wing; anal cell 1/3 proximally lacking microtrichiae; subbasal cell with microtrichiae virtually throughout; veins 2CUa and 2CUb completely spectral; vein 2 cu-a present as spectral vein, sometimes difficult to see; vein 2-1A proximally tubular and distally spectral, although sometimes difficult to see; tubular vein 1 cu-a straight, incomplete/broken and not reaching the edge of 1-1A vein. Hind wing with vannal lobe very narrow, subdistally and subproximally straightened, and setae evenly scattered in the margin.

**Metasoma** (Fig. 118A, H, I, K). Metasoma laterally compressed. Petiole on T1 finely sculptured distally, but only laterally, evenly narrowing distally (length 0.30, maximum width 0.17, minimum width 0.07), and with scattered pubescence on distal half only laterally. Lateral grooves delimiting the median area on T2 clearly defined and reaching the distal edge of T2 (length median area 0.17, length T2 0.17), edges of median area obscured by weak longitudinal stripes, median area as broad as long (length 0.17, maximum width 0.16, minimum width 0.07); T2 with pubescence in distal half. T3 longer than T2 (0.19, 0.17) and with scattered pubescence throughout. Pubescence on hypopygium dense.

**Cocoons.** Unknown.

#### Comments.

The mesoscutum is elongated; dorsally, the head is wide; the median area of vertex is not dinted and has a longitudinal groove. In the holotype, only the coxae and the trochanters are present. Both sexes with slim bodies.

#### Male

(Fig. 119A–L). Similar in coloration to females. Body size 2.22 mm. The median area on T2 darker in coloration than females.

#### Etymology.

Jerry A. Powell’s major fields are systematics, comparative biology, rearing programs, and faunal inventories. He has concentrated his efforts in New World Tortricinae (Tortricoidea) and Ethmiinae (Gelechioidea). He is director emeritus of the Essig (Edward O. Essig) Museum at the University of California, Berkeley, CA, USA.

#### Distribution.

Parasitized caterpillar was collected in Ecuador, Napo, Yanayacu Biological Station (Yanayacu Road), during December 2006 at 2,100 m in cloud forest.

#### Biology.

The lifestyle of this parasitoid species is gregarious.

#### Host.

Undetermined species of Nymphalidae (Ithomiinae) feeding on Schoenobibluscf.peruvianus (Thymeliaceae). Caterpillar was collected in second instar.

### Glyptapanteles jesusugaldei

Taxon classificationAnimaliaHymenopteraBraconidae

Arias-Penna, sp. nov.

http://zoobank.org/3CEBE94B-22AD-4AEF-973F-C306AAAC767E

Figs 120 , 121

#### Female.

Body length 2.07 mm, antenna length 2.17 mm, fore wing length 2.27 mm.

#### Type material.

**Holotype**: COSTA RICA • 1♀; 10-SRNP-56312, DHJPAR0040396; Área de Conservación Guanacaste, Guanacaste, Sector Mundo Nuevo, Vado Zanja Tapada; dry-rain intergrade forest; 550 m; 10.76480, -85.38445; 06.viii.2010; Mariano Pereira leg.; caterpillar collected in fifth instar; cocoons adhered to the leaf substrate and formed on 12.viii.2010; adult parasitoids emerged on 17.viii.2010; (CNC). **Paratypes.** • 9 (2♀, 2♂) (5♀, 0♂); 10-SRNP-56312, DHJPAR0040396; same data as for holotype; (CNC).

#### Other material.

**Reared material.** COSTA RICA: *Área de Conservación Guanacaste*, *Guanacaste*, *Sector Santa Rosa*, *Bosque Humedo*: • 12 (2♀, 2♂) (5♀, 3♂); 92-SRNP-5824, DHJPAR0000068; dry forest; 290 m; 10.85145, -85.60801; 05.xi.1992; gusaneros leg.; caterpillar collected in fifth instar; cocoons on leaf on both sides of caterpillar, rather than underneath body, widely spaced cocoons; adult parasitoids emerged on 16.xi.1992. • 17 (5♀, 1♂) (11♀, 0♂); 98-SRNP-12670, DHJPAR0000100; same data as for preceding except: 01.xi.1998; caterpillar collected in fourth instar; two rows of white parallel cordwood cocoons on each side of larva, not stacked and adhered to the leaf substrate; adult parasitoid emerged on 09.xi.1998. • 18 (2♀, 1♂) (14♀, 1♂); 98-SRNP-12679, DHJPAR0000101; same data as for preceding except: 01.xi.1998; two parallel rows of white cylinders on each side of larva, not stacked, each slightly separate from the other, cocoons adhered to the leaf substrate; adult parasitoids emerged on 14.xi.1998. • 5 (2♀, 1♂) (2♀, 0♂); 98-SRNP-12783, DHJPAR0000103; same data as for preceding except: 09.xi.1998; Manuel Pereira leg.; white neatly ordered cocoons side by side on each side of the larva, not stacked, cocoons adhered to the leaf substrate; adult parasitoids emerged on 19.xi.1998. • 7 (2♀, 2♂) (2♀, 1♂); 98-SRNP-12784, DHJPAR0000104; same data as for preceding except: 09.xi.1998; Manuel Pereira leg.; caterpillar collected in fourth instar; white cocoons irregular fluffy adhered to each other and adhered to the leaf substrate; adult parasitoids emerged on 21.xi.1998. • 6 (2♀, 2♂) (1♀, 1♂); 98-SRNP-12785, DHJPAR0000105; same data as for preceding except: 09.xi.1998; Manuel Pereira leg.; caterpillar collected in fourth instar; white single cocoons in two orderly rows of cordwood on each side of body, not stacked, side by side, cocoons adhered to the leaf substrate; adult parasitoids emerged on 21.xi.1998; specimens of *Mesochorus* (Ichneumonidae: Mesochorinae) were reported as hyperparasitoids.

*Área de Conservación Guanacaste*, *Guanacaste*, *Sector El Hacha*, *Sendero Bejuquilla*: • 19 (5♀, 5♂) (9♀, 0♂); 01-SRNP-11932, DHJPAR0000012; dry-rain intergrade forest; 280 m; 11.03004, -85.52699; 07.xi.2001; Lucia Ríos leg.; caterpillar collected in fourth instar; adult parasitoids emerged on 25.xi.2001. • 29 (5♀, 4♂) (20♀, 0♂); 01-SRNP-24006, DHJPAR0000017; same data as for preceding except: 18.xi.2001; grayish lightly cocoons adhered to the leaf forming two parallel rows of cordwood on both sides of the cadaver, cocoons at right angles to the cadaver; adult parasitoids emerged on 29.xi.2001.

#### Malaise-trapped material.

COSTA RICA: *Área de Conservación Guanacaste*, *Guanacaste*, *Sector El Hacha*, *Sendero Bejuquilla*: • 1 (0♀, 1♂) (0♀, 0♂); 99-SRNP-19244, DHJPAR0013631; dry-rain intergrade forest; Malaise trap; 280 m; 11.03004, -85.52699; 18.i.1999; DH Janzen & W Hallwachs leg.

#### Diagnosis.

**Figure 120.**
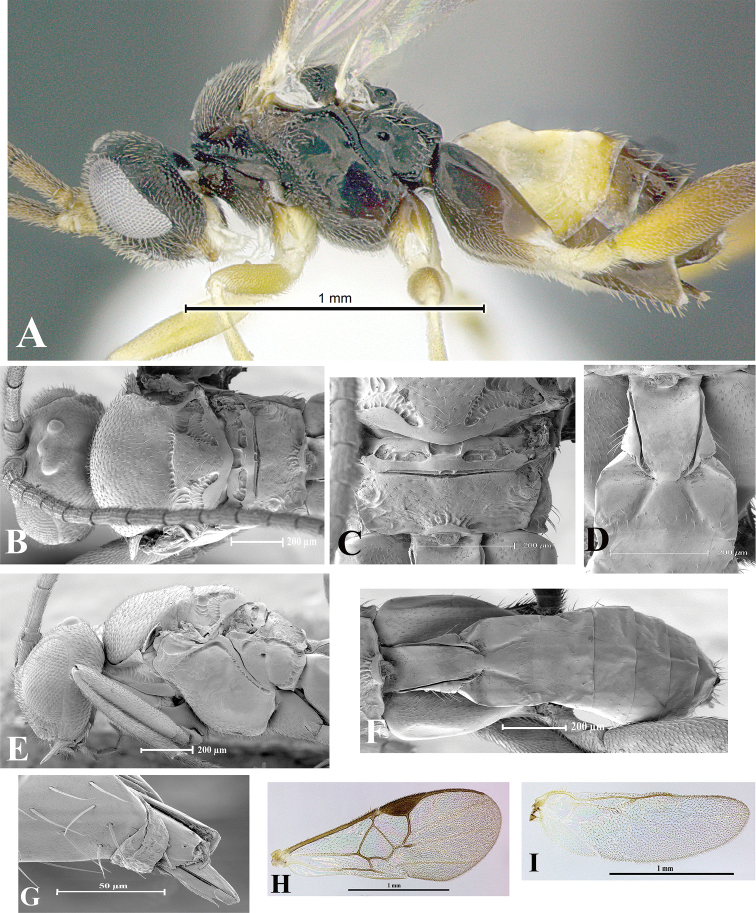
*Glyptapantelesjesusugaldei* sp. nov. female 98-SRNP-12679 DHJPAR0000101, 10-SRNP-56312 DHJPAR0040396 **A** Habitus **B, E** Head, mesosoma **B** Dorsal view **E** Lateral view **C** Metanotum, propodeum, dorsal view **D**T1–2, dorsal view **F** Metasoma, dorsal view **G** Genitalia: hypopygium, ovipositor, ovipositor sheaths, lateral view **H, I** Wings **H** Fore **I** Hind.

**Figure 121.**
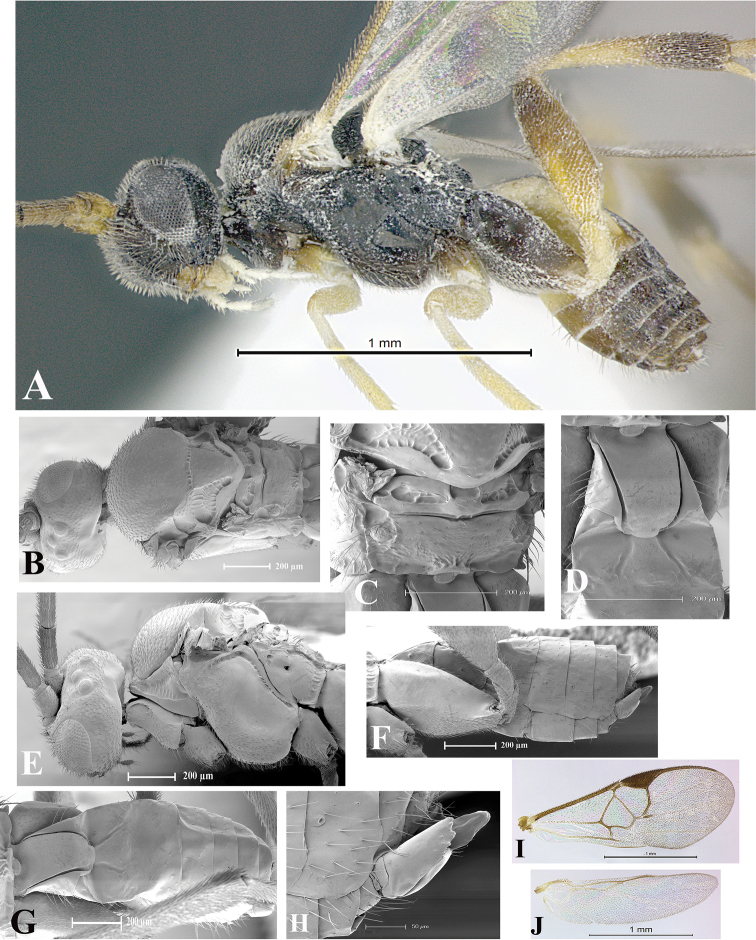
*Glyptapantelesjesusugaldei* sp. nov. male 98-SRNP-12679 DHJPAR0000101, 10-SRNP-56312 DHJPAR0040396 **A** Habitus **B, E** Head, mesosoma **B** Dorsal view **E** Lateral view **C** Scutellum, metanotum, propodeum, dorsal view **D**T1–2, dorsal view **F, G** Metasoma **F** Lateral view **G** Dorsal view **H** Genitalia: parameres, lateral view **I, J** Wings **I** Fore **J** Hind.

Antenna longer than body, malar suture present, longitudinal median carina on face absent, surface of metasternum flat or nearly so, fore wing with r vein curved, outer side of junction of r and 2RS veins not forming a stub (Figs 120H, 121I), distal antennal flagellomere longer than penultimate, petiole on T1 evenly narrowing distally, completely smooth, and polished, with faint, satin-like sheen (Figs 120D, F, 121D, G), propodeum without median longitudinal carina (Figs 120C, 121C), and lateral grooves delimiting the median area on T2 clearly defined and reaching the distal edge of T2 (Figs 120D, F, 121D, G).

#### Coloration

(Fig. 120A). General body coloration dark brown, although some areas on body are light brown/reddish as propleuron, both dorsal and ventral furrows of pronotum, epicnemial ridge, ventral edge of mesopleuron, distal corners of mesoscutum, scape, pedicel, labrum, and mandibles yellow-brown; first four-five proximal antennal flagellomeres dorsally lighter (yellow-brown) than ventrally (brown), remaining flagellomeres brown on both sides; glossa, maxillary and labial palps, and tegulae yellow. Eyes and ocelli silver. Fore and middle legs yellow except fore coxae which proximally with a small light brown area, middle coxae proximally brown, but distally yellow-brown, and claws brown; hind legs yellow except dark brown coxae, yellow-brown femora (intensity of yellow-brown coloration increasing from proximal to distal), distal 1/3 of tibia, and tarsomeres brown although basitarsus proximally yellow. Petiole on T1 brown, but proximally yellow, contours darkened and sublateral areas yellow; T2 with median and adjacent areas brown, and lateral ends yellow; T3 with a brown area which width coincides with the width of median area on T2, thus both T2 and T3 forming a large brown triangle and lateral ends yellow; T4 and beyond completely brown; distally each tergum with a narrow yellowish transparent band. In lateral view, T1–3 yellow; T4 and beyond brown. S1–4 yellow; penultimate sternum and hypopygium brown.

#### Description.

**Head** (Fig. 120A, B, E). Head rounded with pubescence short and dense. Proximal three antennal flagellomeres longer than wide (0.17:0.06, 0.17:0.06, 0.18:0.06), distal antennal flagellomere longer than penultimate (0.10:0.05, 0.08:0.05), antenna longer than body (2.17, 2.07); antennal scrobes-frons shallow. Face flat or nearly so, with dense fine punctations, interspaces wavy and longitudinal median carina absent. Frons punctate. Temple wide, punctate and interspaces wavy. Inner margin of eyes diverging slightly at antennal sockets; in lateral view, eye anteriorly convex and posteriorly straight. POL shorter than OOL (0.08, 0.11). Malar suture present. Median area between lateral ocelli without depression. Vertex laterally rounded and dorsally wide.

**Mesosoma** (Fig. 120A–C, E). Mesosoma dorsoventrally convex. Mesoscutum proximally convex and distally flat, punctation distinct throughout, interspaces wavy/lacunose. Scutellum triangular, apex sloped and fused with BS, scutellar punctation scattered throughout, in profile scutellum flat and on same plane as mesoscutum, phragma of the scutellum partially exposed; BS only very partially overlapping the MPM; ATS demilune with short stubs delineating the area; dorsal ATS groove with semicircular/parallel carinae. Transscutal articulation with small and heterogeneous foveae, area just behind transscutal articulation smooth, shiny and depressed centrally. Metanotum with BM wider than PFM (clearly differentiated); MPM circular without median longitudinal carina; AFM without setiferous lobes and not as well delineated as PFM; PFM thick, smooth and with a distal flat flange; ATM proximally with a groove with some sculpturing and distally smooth. Propodeum without median longitudinal carina, proximal half weakly curved with medium-sized sculpture and distal half slightly rugose; distal edge of propodeum with a flange at each side and without stubs; propodeal spiracle distally framed by a short concave carina; nucha surrounded by very short radiating carinae. Pronotum with a distinct dorsal furrow, dorsally with a well-defined smooth band; central area of pronotum smooth, but both dorsal and ventral furrows with short parallel carinae. Propleuron with a mix of rugae and fine punctation, dorsally with a carina. Metasternum flat or nearly so. Contour of mesopleuron straight/angulate or nearly so; precoxal groove deep, smooth and shiny; epicnemial ridge elongated and more fusiform.

**Legs.** Ventral margin of fore telotarsus slightly excavated and with a tiny curved seta, fore telotarsus almost same width throughout and longer than fourth tarsomere (0.12, 0.06). Hind coxa with punctation only on ventral surface, dorsal outer depression present. Inner spur of hind tibia longer than outer spur (0.20, 0.17), entire surface of hind tibia with dense strong spines clearly differentiated by color and length. Hind telotarsus as equal in length as fourth tarsomere (0.10, 0.09).

**Wings** (Fig. 120H, I). Fore wing with r vein slightly curved; 2RS vein slightly convex to convex; r and 2RS veins forming an angle at their junction and outer side of junction not forming a stub; 2M vein slightly curved/swollen; distally fore wing [where spectral veins are] with microtrichiae more densely concentrated than the rest of the wing; anal cell 1/3 proximally lacking microtrichiae; subbasal cell with a small smooth area; vein 2CUa absent and vein 2CUb spectral; vein 2 cu-a absent; vein 2-1A present only proximally as tubular vein; tubular vein 1 cu-a curved and complete, but junction with 1-1A vein spectral. Hind wing with vannal lobe narrow, subdistally and subproximally evenly convex, and setae present only proximally.

**Metasoma** (Fig. 120A, D, F, G). Metasoma laterally compressed. Petiole on T1 completely smooth and polished, with faint, satin-like sheen, evenly narrowing distally (length 0.28, maximum width 0.14, minimum width 0.07) and with scattered pubescence concentrated in the first distal third. Lateral grooves delimiting the median area on T2 clearly defined and reaching the distal edge of T2 (length median area 0.12, length T2 0.12), edges of median area polished and lateral grooves deep, median area broader than long (length 0.12, maximum width 0.17, minimum width 0.06); T2 with scattered pubescence only distally. T3 longer than T2 (0.18, 0.12) and with scattered pubescence only distally. Pubescence on hypopygium dense.

**Cocoons.** White oval cocoons with messy/disordered/fluffy silk fibers. Cocoons forming two rows of cordwood on each side of larva and adhered to the leaf substrate.

#### Comments.

Both sexes with slim bodies.

#### Male

(Fig. 121A–J). Coloration and shape similar to female.

#### Etymology.

Jesús Armando Ugalde Gómez works at the Instituto Nacional de Biodiversidad (INBio), Santo Domingo de Heredia, Costa Rica.

#### Distribution.

Parasitized caterpillars were collected in Costa Rica, ACG, Sector El Hacha (Sendero Bejuquilla), Sector Mundo Nuevo (Vado Zanja Tapada), and Sector Santa Rosa (Bosque Humedo), during November 1998 and 2001, and August 2010 at 280 m, 290 m, and 550 m in dry and dry-rain integrated forests.

Adult parasitoid was collected in Costa Rica, ACG, Sector El Hacha (Sendero Bejuquilla), during January 1999 at 280 m in dry-rain integrated forest.

#### Biology.

The lifestyle of this parasitoid species is gregarious. *Mesochorus* (Ichneumonidae: Mesochorinae) was reported as hyperparasitoid.

#### Host.

*Antiblemma* sp. Hübner (Erebidae: Eulepidotinae) feeding on *Psychotrianervosa* and *P.microdon* (Rubiaceae). Caterpillars were collected in fourth and fifth instar.

### Glyptapanteles jimmilleri

Taxon classificationAnimaliaHymenopteraBraconidae

Arias-Penna, sp. nov.

http://zoobank.org/D6CBBE65-CACE-4F7D-B0CD-F34BF0A192BC

Fig. 122

#### Female.

Body length 2.68 mm, antenna length 3.33 mm, fore wing length 3.03 mm.

#### Type material.

**Holotype**: ECUADOR • 1♀; EC-12886, YY-A037; Napo, Yanayacu Biological Station, Yanayacu Road; cloud forest; 2,100 m; -0.566667, -77.866667; 06.iii.2006; Rafael Granizo leg.; caterpillar collected in second instar; cocoons formed on 20.iii.2006; adult parasitoids emerged on 06.iv.2006; (PUCE). **Paratypes.** • 5 (3♀, 1♂) (1♀, 0♂); EC-12886, YY-A037; same data as for holotype; (PUCE).

#### Other material.

**Reared material.** ECUADOR: *Napo*, *Yanayacu Biological Station*, *Yanayacu Road/Birding Circuit*: • 10 (5♀, 2♂) (3♀, 0♂); EC-12715, YY-A047; cloud forest; 2,100 m; -0.566667, -77.866667; 01.iii.2006; Rafael Granizo leg.; caterpillar collected in second instar; cocoons formed on 10.iv.2006; adult parasitoids emerged on 17.iv.2006.

*Napo*, *Yanayacu Biological Station*, *Sendero Granja Integral Baeza*, *Baeza Sendero Granja*: • 4 (2♀, 1♂) (1♀, 0♂); EC-12795, YY-A095; cloud forest; 1,800 m; -0.5833, -77.8833; 02.iii.2006; Rafael Granizo leg.; caterpillar collected in fifth instar; cocoons formed on 20.v.2006; adult parasitoids emerged on 01.iv.2006.

*Napo*, *Yanayacu Biological Station Yanayacu Station*, *YanayacuForest*: • 3 (0♀, 2♂) (0♀, 1♂); EC-35157, YY-A068; cloud forest; 2,100 m; -0.6, -77.883333; 24.vii.2008; Earthwatch volunteers leg.; caterpillar collected in first instar; bud-like white floret cocoons formed on 10.ix.2008; adult parasitoids emerged on 06.x.2008.

*Napo*, *Yanayacu Biological Station*, *Sendero Macuculoma*, *MPassiflora Plot 1*: • 3 (1♀, 1♂) (1♀, 0♂); EC-39687, YY-A008; cloud forest; 2,000 m; -0.604806, -77.886417; 03.vii.2009; CAPEA leg.; Lepidoptera collected as eggs; cocoons formed on 13.viii.2009; adult parasitoids emerged on 01.ix.2009. • 1 (1♀, 0♂) (0♀, 0♂); EC-39707, YY-A176; same data as for preceding except: caterpillar instar not reported; adult parasitoids emerged on 17.xi.2009.

#### Diagnosis.

**Figure 122.**
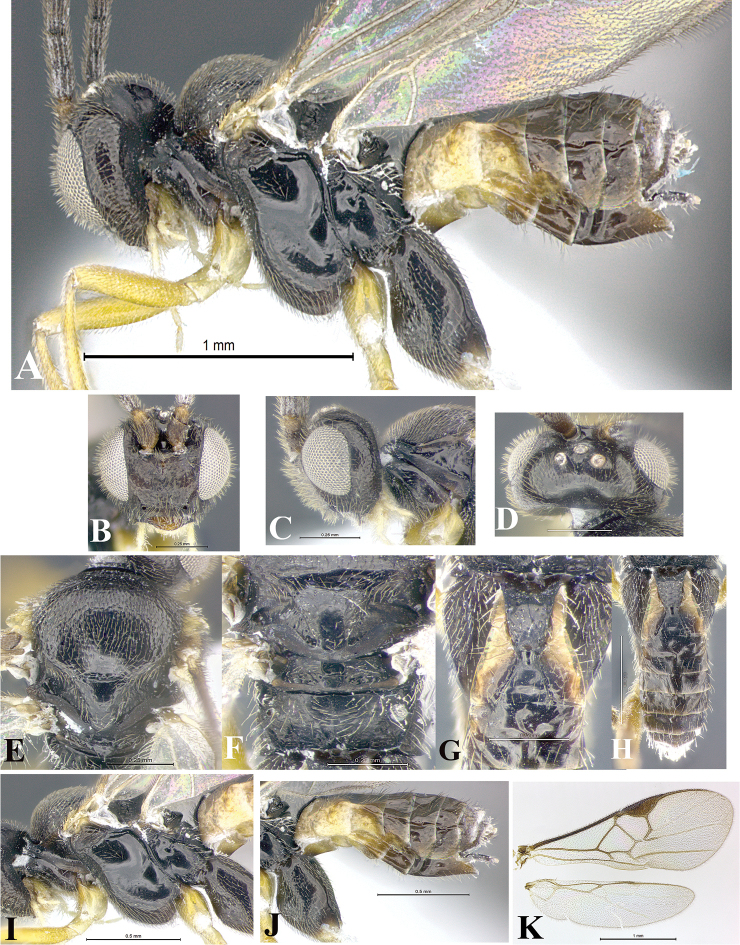
*Glyptapantelesjimmilleri* sp. nov. female EC-12795 YY-A095, EC-12886 YY-A037 **A** Habitus **B, D** Head **B** Frontal view **D** Dorsal view **C** Head, pronotum, propleuron, lateral view **E** Mesonotum, dorsal view **F** Scutellum, metanotum, propodeum, dorsal view **G**T1–3, dorsal view **H, J** Metasoma **H** Dorsal view **J** Lateral view **I** Mesosoma, lateral view **K** Fore and hind wings.

Petiole on T1 finely sculptured only distally (Fig. 122G, H), vertex in lateral view rounded (Fig. 122C), scutellar punctation indistinct throughout (Fig. 122E), phragma of the scutellum widely visible (Fig. 122F), median area on T2 as broad as long, edges of median area on T2 polished and followed by a deep groove, lateral grooves delimiting the median area on T2 clearly defined and reaching the distal edge of T2 (Fig. 122G), anterior furrow of metanotum with a small lobe, without setae (Fig. 122F), axillary trough of scutellum almost smooth (Fig. 122F), propodeum without median longitudinal carina (Fig. 122F), anteroventral contour of mesopleuron convex (Fig. 122A, I), and fore wing with r vein slightly curved, outer side of junction of r and 2RS veins forming a distinct stub (Fig. 122K).

#### Coloration

(Fig. 122A–J). General body coloration shiny black except apex of mandibles, labrum, apex of propleuron, dorsal and ventral furrows of pronotum, distal corners of mesoscutum, a distal narrow band through lunules and beyond, and half inner part of PFM with light brown/reddish tints; glossa, maxillary and labial palps, and tegulae yellow; pedicel yellow-brown; scape and all antennal flagellomeres (on both sides) dark brown. Eyes silver and ocelli yellowish. Fore and middle legs dark yellow except brown claws; hind legs dark yellow except black coxae, femora dorsally with a small brown spot, tibiae with 1/3 distal brown and proximally with a narrow brown band, and tarsomeres light brown, but basitarsus proximally with a narow yellow band. Petiole on T1 dark brown/black and sublateral areas light yellow-brown; T2 with median and adjacent areas brown, and lateral ends light yellow-brown; T3 brown with two oval yellow-brown spots at each proximal corner; T4 and beyond completely brown; distally each tergum with a narrow yellowish transparent band. In lateral view, T1–2 completely yellow; T3–4 yellow with a brown dorsal area which extent increasing from proximal to distal; T5 and beyond completely brown. S1–3 yellow; S4 yellow, distally brown; penultimate sternum and hypopygium completely brown.

#### Description.

**Head** (Fig. 122A–D). Head rounded with pubescence long and dense. Proximal three antennal flagellomeres longer than wide (0.23:0.09, 0.23:0.09, 0.24:0.09), distal antennal flagellomere longer than penultimate (0.13:0.05, 0.11:0.05), antenna longer than body (3.33, 2.68); antennal scrobes-frons shallow. Face convex, punctations barely noticeable, interspaces smooth and longitudinal median carina present. Frons rugose. Temple wide, punctations barely noticeable and interspaces clearly smooth. Inner margin of eyes diverging slightly at antennal sockets; in lateral view, eye anteriorly convex and posteriorly straight. POL shorter than OOL (0.09, 0.11). Malar suture present. Median area between lateral ocelli without depression. Vertex laterally rounded and dorsally wide.

**Mesosoma** (Fig. 122A, E, F, I). Mesosoma dorsoventrally convex. Distal 1/3 of mesoscutum with lateral margin slightly dented, punctation distinct throughout, interspaces smooth. Scutellum triangular, apex sloped and fused with BS, but not in the same plane, scutellar punctation indistinct throughout, in profile scutellum flat and on same plane as mesoscutum, phragma of the scutellum widely visible; BS only very partially overlapping the MPM; ATS demilune almost smooth; dorsal ATS groove smooth. Transscutal articulation with small and heterogeneous foveae, area just behind transscutal articulation depressed centrally and with same kind of sculpture as mesoscutum. Metanotum with BM wider than PFM (clearly differentiated); MPM circular without median longitudinal carina; AFM with a small lobe and not as well delineated as PFM; PFM thick, smooth and with lateral ends rounded; ATM proximally with sculpture distally without a well delimited smooth area. Propodeum with indistinct sculpture and without median longitudinal carina, proximal half straight or nearly so; distal edge of propodeum with a flange at each side and without stubs; propodeal spiracle without distal carina; nucha surrounded by very short radiating carinae. Pronotum with a distinct dorsal furrow, dorsally with a well-defined smooth band; central area of pronotum and both dorsal and ventral furrows smooth. Propleuron with fine punctations throughout and dorsally without a carina. Metasternum convex. Contour of mesopleuron convex; precoxal groove smooth, shiny and shallow, but visible; epicnemial ridge convex, teardrop-shaped.

**Legs.** Ventral margin of fore telotarsus entire without seta, fore telotarsus almost same width throughout and longer than fourth tarsomere (0.10, 0.07). Hind coxa with very finely punctate throughout and dorsal outer depression present. Inner spur of hind tibia longer than outer spur (0.23, 0.16), entire surface of hind tibia with dense strong spines clearly differentiated by color and length. Hind telotarsus as equal in length as fourth tarsomere (0.12, 0.12).

**Wings** (Fig. 122K). Fore wing with r vein slightly curved; 2RS vein slightly convex to convex; r and 2RS veins forming a weak, even curve at their junction and outer side of junction forming a distinct stub; 2M vein slightly curved/swollen; distally fore wing [where spectral veins are] with microtrichiae more densely concentrated than the rest of the wing; anal cell 1/3 proximally lacking microtrichiae; subbasal cell with microtrichiae virtually throughout; veins 2CUa and 2CUb completely spectral; vein 2 cu-a present as spectral vein, sometimes difficult to see; vein 2-1A proximally tubular and distally spectral, although sometimes difficult to see; tubular vein 1 cu-a curved, incomplete/broken and not reaching the edge of 1-1A vein. Hind wing with vannal lobe very narrow, subdistally and subproximally straightened, and setae evenly scattered in the margin.

**Metasoma** (Fig. 122A, G, H, J). Metasoma laterally compressed. Petiole on T1 finely sculptured only distally, virtually parallel-sided over most of length, but narrowing over distal 1/3 (length 0.35, maximum width 0.19, minimum width 0.08), and with scattered pubescence on distal half. Lateral grooves delimiting the median area on T2 clearly defined and reaching the distal edge of T2 (length median area 0.18, length T2 0.18), edges of median area polished, median area as broad as long (length 0.18, maximum width 0.18, minimum width 0.07); T2 with scarce pubescence throughout. T3 longer than T2 (0.21, 0.18) and with scattered pubescence throughout. Pubescence on hypopygium dense.

**Cocoons.** Bud-like white cocoons.

#### Comments.

The contours of the median area on T2 are weakly defined. The proximal edge of the mesopleuron is slightly inclined/sloped. The ATS demilune and groove are smooth; however, in some females, the ATS has a quite little stubs and the sculpture on ATM cover more area.

#### Male.

Coloration similar to females; however, the punctate on the mesoscutum tend to be more scattered distally and the coloration on fore and middle tarsomeres has a brown tinge. The males are darker and more polished than females.

#### Etymology.

James (Jim) Stuart Miller’s research addresses general issues in taxonomy, biodiversity, phylogeny, and historical ecology of Noctuoidea moths. He is a research associate at American Museum of Natural History, New York, NY, USA.

#### Distribution.

Parasitized caterpillars were collected in Ecuador, Napo, Yanayacu Biological Station (Sendero Granja Integral Baeza, Sendero Macuculoma, Yanayacu Road, Yanayacu Road/Birding Circuit, and YanayacuForest), during March 2006, and July 2008 and 2009 at 1,800 m, 2,000 m, and 2,100 m in cloud forest.

#### Biology.

The lifestyle of this parasitoid species is solitary/gregarious. First species of *Glyptapanteles* reported attacking egg of Lepidoptera: Notodontidae, Dioptinae.

#### Host.

*Josia* sp. Hübner and *Lyces* sp. Walker (Notodontidae: Dioptinae) feeding on *Passiflora* sp. (Passifloraceae) and *Lycesfornax* Druce (Notodontidae: Dioptinae) feeding on *Passifloraligularis* (Passifloraceae). Undetermined species of Notodontidae feeding on *Passiflora* sp. and *P.ligularis* (Passifloraceae). Caterpillars were collected as eggs, and larvae in first, second, and fifth instar.

### Glyptapanteles jjrodriguezae

Taxon classificationAnimaliaHymenopteraBraconidae

Arias-Penna, sp. nov.

http://zoobank.org/1F94E506-F45C-4E94-88C7-37DF8EEE79F3

Figs 123 , 124

#### Female.

Body length 2.07 mm, antenna length 1.81 mm, fore wing length 2.02 mm.

#### Type material.

**Holotype**: COSTA RICA • 1♀; 09-SRNP-70365, DHJPAR0035414; Área de Conservación Guanacaste, Guanacaste, Sector Pitilla, Manguera; rain forest; 470 m; 10.99590, -85.39842; 15.v.2009; Ricardo Calero leg.; caterpillar collected in fifth instar; cocoons adhered to the larval cuticle and already present in the caterpillar; adult parasitoids emerged on 21.v.2009; (CNC). **Paratypes.** • 73 (3♀, 4♂) (33♀, 33♂); 09-SRNP-70365, DHJPAR0035414; same data as for holotype; (CNC).

#### Other material.

**Reared material.** COSTA RICA: *Área de Conservación Guanacaste*, *Guanacaste*, *Sector Santa Rosa*, *Bosque Humedo*: • 11 (3♀, 1♂) (4♀, 0♂); 93-SRNP-7332, DHJPAR0000081; dry forest; 290 m; 10.85145, -85.60801; 27.x.1993; gusaneros leg.; caterpillar collected in fifth instar; brown cocoons neatly aligned in two rows of cordwood on each side of live larva, cocoons adhered to the leaf substrate; adult parasitoids emerged on 12.xi.1993. • 19 (3♀, 3♂) (9♀, 4♂); 93-SRNP-7333, DHJPAR0000082; same data as for preceding except: caterpillar instar not reported; neat brown cocoons forming two rows of cordwood on both sides of the larva, cocoons adhered to the leaf substrate and formed 31.x.1993; adult parasitoids emerged on 05.xi.1993. • 38 (3♀, 4♂) (31♀, 0♂); 94-SRNP-12, DHJPAR0000084; 02.i.1994; same data as for preceding except: caterpillar instar not reported; stacked up brown/gray cocoons adhered to the leaf substrate and formed on 07.i.1994; adult parasitoids emerged on 12.i.1994.

*Área de Conservación Guanacaste*, *Guanacaste*, *Sector Santa Rosa*, *Alacrán*: • 29 (3♀, 3♂) (18♀, 5♂); 92-SRNP-3413, DHJPAR0000065; dry forest; 260 m; 10.89249, -85.60336; 16.vii.1992; gusaneros leg.; brown cocoons in neat row of cordwood on each side of caterpillar, cocoons adhered to the leaf substrate and formed on 17.vii.1992; adult parasitoids emerged on 25.vii.1992.

*Área de Conservación Guanacaste*, *Guanacaste*, *Sector Pitilla*, *Manguera*: • 74 (4♀, 4♂) (33♀, 33♂); 09-SRNP-70365, DHJPAR0035414; rain forest; 470 m; 10.99590, -85.39842; 15.v.2009; Ricardo Calero leg.; caterpillar collected in fifth instar; cocoons adhered to the larval cuticle and already present in the caterpillar; adult parasitoids emerged on 21.v.2009.

*Área de Conservación Guanacaste*, *Alajuela*, *Sector San Cristobal*, *Río Blanco Abajo*: • 15 (3♀, 1♂) (11♀, 0♂); 08-SRNP-828, DHJPAR0020735; rain forest; 500 m; 10.90037, -85.37254; 22.ii.2008; Gloria Sihezar leg.; caterpillar collected in fifth instar; brown cordwood cocoons on each side of caterpillar, cocoons adhered to the leaf substrate; adult parasitoids emerged on 10.iii.2008. • 31 (3♀, 2♂) (26♀, 0♂); 08-SRNP-877, DHJPAR0020732; same data as for preceding except: caterpillar collected in fourth instar; cordwood cocoons on each side of cadaver adhered to the leaf substrate. • 20 (3♀, 2♂) (15♀, 0♂); 08-SRNP-878, DHJPAR0020889; same data as for preceding except: caterpillar collected in fourth instar; cordwood cocoons on each side of caterpillar cadaver, cocoons adhered to the leaf substrate; adult parasitoids emerged on 31.iii.2008. • 43 (3♀, 1♂) (39♀, 0♂); 08-SRNP-949, DHJPAR0020734; same data as for preceding except: Elda Araya; caterpillar collected in fourth instar; two rows of cordwood cocoons adhered to the leaf substrate; adult parasitoids emerged on 07.iii.2008.

*Área de Conservación Guanacaste*, *Guanacaste*, *Sector Mundo Nuevo*, *Vado Ficus*: • 29 (3♀, 3♂) (20♀, 3♂); 07-SRNP-56363, DHJPAR0030831; dry-rain intergrade forest; 375 m; 10. 77090, -85. 42455; 10.v.2007; José Alberto Sánchez leg.; caterpillar collected in fourth instar; jumbled cordwood of cocoons on each side of the cadaver, cocoons adhered to the leaf substrate and formed on 17.v.2007; adult parasitoids emerged on 21.v.2007. 45 (2♀, 2♂) (29♀, 12♂); 07-SRNP-56372, DHJPAR0030821; same data as for preceding except: caterpillar collected in second instar; single rwo of brown cordwood cocoons on each side of the caterpillar.

*Área de Conservación Guanacaste*, *Guanacaste*, *Sector Mundo Nuevo*, *Quebrada Tibio Perla*: • 7 (2♀, 3♂) (2♀, 0♂); 07-SRNP-56485, DHJPAR0031181; dry-rain intergrade forest; 330 m; 10.76261, -85.42979; 14.v.2007; Jose Cortez leg.; caterpillar collected in fifth instar; small brown cocoons forming irregular cordwood on each side of cadaver, cocoons adhered to the leaf substrate and formed on 17.v.2007; adult parasitoids emerged on 20.v.2007, 21.v.2007, 27.v.2007.

*Área de Conservación Guanacaste*, *Guanacaste*, *Sector Mundo Nuevo*, *Vado Huacas*: • 2 (0♀, 1♂) (0♀, 1♂); 07-SRNP-56495, DHJPAR0030800; dry-rain intergrade forest; 490 m; 10.75533, -85.39117; 14.v.2007; Jose Alberto Sánchez leg.; caterpillar collected in fourth instar; brown cordwood cocoons adhered to the leaf substrate and formed on 20.v.2007; adult parasitoids emerged on 27.v.2007, 30.v.2007; *Mesochorus* (Ichneumonidae: Mesochorinae) was reported as hyperparasitoid. • 18 (3♀, 3♂) (9♀, 3♂); 07-SRNP-56496, DHJPAR0030901; same data as for preceding except: brown cordwood cocoons on each side of larva; adult parasitoid emerged on 28.v.2007. • 31 (0♀, 5♂) (0♀, 22♂); 07-SRNP-56497, DHJPAR0030898; same data as for preceding except: caterpillar collected in fifth instar; irregularly cordwood cocoons on both sides of the larva, cocoons formed on 17.v.2007; adult parasitoids on 21.v.2007.

#### Diagnosis.

**Figure 123.**
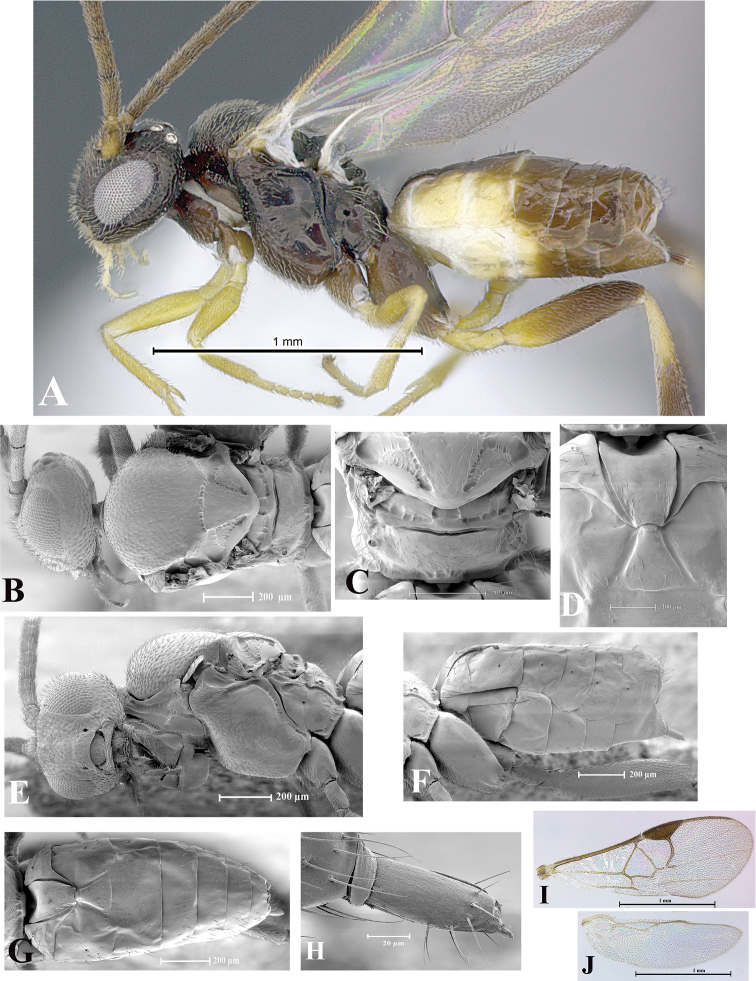
*Glyptapantelesjjrodriguezae* sp. nov. female 09-SRNP-70365 DHJPAR0035414 **A** Habitus **B, E** Head, mesosoma **B** Dorsal view **E** Lateral view **C** Metanotum, propodeum, dorsal view **D**T1–2, dorsal view **F, G** Metasoma **F** Lateral view **G** Dorsal view **H** Genitalia: hypopygium, ovipositor, ovipositor sheaths, lateral view **I, J** Wings **I** Fore **J** Hind.

**Figure 124.**
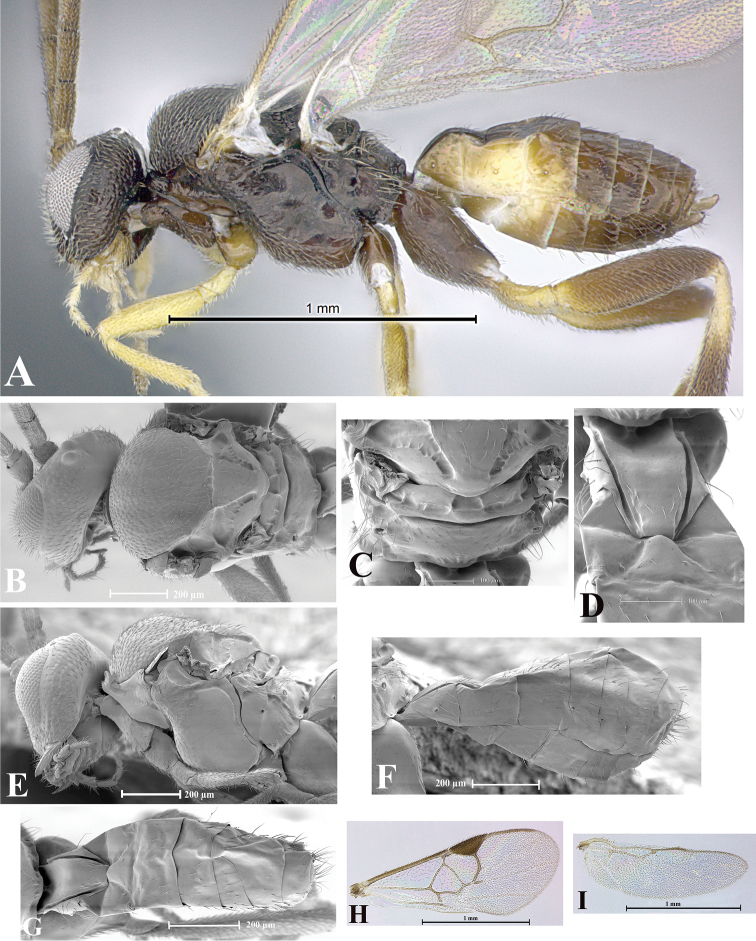
*Glyptapantelesjjrodriguezae* sp. nov. male 09-SRNP-70365 DHJPAR0035414 **A** Habitus **B, E** Head, mesosoma **B** Dorsal view **E** Lateral view **C** Scutellum, metanotum, propodeum, dorsal view **D**T1–2, dorsal view **F, G** Metasoma **F** Lateral view **G** Dorsal view **H, I** Wings **H** Fore **I** Hind.

Antenna shorter than body, malar suture absent or difficult to see (Fig. 123E), longitudinal median carina on face present, surface of metasternum flat or nearly so, fore wing with r vein curved, outer side of junction of r and 2RS veins not forming a stub (Figs 123I, 124H), distal antennal flagellomere longer than penultimate, petiole on T1 evenly narrowing distally, completely smooth and polished, with faint, satin-like sheen (Figs 123D, G, 124D, G), propodeum without median longitudinal carina (Figs 123C, 124C), and lateral grooves delimiting the median area on T2 clearly defined and reaching the distal edge of T2 (Figs 123D, G, 124D, G).

#### Coloration

(Fig. 123A). General body coloration dark brown although ventrally light brown, except scape, pedicel, labrum and mandibles yellow-brown; first five proximal antennal flagellomeres dorsally lighter (yellow-brown) than ventrally (brown), remaining flagellomeres brown on both sides; glossa, maxillary and labial palps, and tegulae yellow. Eyes and ocelli silver. Fore and middle legs yellow except brown coxae and claws; hind legs yellow except dark brown coxae, most of the femora yellow-brown (coloration intensity increasing from proximal to distal), most of tibia and tarsomeres brown, although basitarsus with a proximal yellow band. Petiole on T1 light brown, contours slightly darkened, and sublateral areas yellow; T2 with median area dark brown, adjacent area brown with smeared yellow boundaries, and lateral ends yellow; T3 and beyond completely brown; distally each tergum with a narrow whitish transparent band. In lateral view, T1–3 yellow; T4 and beyond brown. S1–3 yellow; S4 proximal half yellow, distal half brown; penultimate sternum and hypopygium brown.

#### Description.

**Head** (Fig. 123A, B, E). Head rounded with pubescence short and dense. Proximal three antennal flagellomeres longer than wide (0.13:0.05, 0.14:0.05, 0.14:0.05), distal antennal flagellomere longer than penultimate (0.10:0.05, 0.08:0.05), antenna shorter than body (1.81, 2.07); antennal scrobes-frons shallow. Face flat or nearly so, with dense fine punctations, interspaces with microsculpture and longitudinal median carina present. Frons punctate. Temple wide, punctate and interspaces wavy. Inner margin of eyes diverging slightly at antennal sockets; in lateral view, eye anteriorly convex and posteriorly straight. POL shorter than OOL (0.08, 0.10). Malar suture absent or difficult to see. Median area between lateral ocelli slightly depressed. Vertex laterally rounded and dorsally wide.

**Mesosoma** (Fig. 123A–C, E). Mesosoma dorsoventrally convex. Mesoscutum proximally convex and distally flat, punctation distinct throughout, interspaces with microsculpture. Scutellum triangular, apex sloped and fused with BS, scutellar punctation scattered throughout, in profile scutellum flat and on same plane as mesoscutum, phragma of the scutellum partially exposed; BS only very partially overlapping the MPM; ATS demilune with short stubs delineating the area; dorsal ATS groove with carinae only proximally. Transscutal articulation with small and heterogeneous foveae, area just behind transscutal articulation smooth, shiny and depressed centrally. Metanotum with BM wider than PFM (clearly differentiated); MPM semicircular without median longitudinal carina; AFM without setiferous lobes and not as well delineated as PFM; PFM thick, smooth and with a distal flat flange; ATM proximally with a groove with some sculpturing and distally smooth. Propodeum polished without median longitudinal carina, proximal half weakly curved; distal edge of propodeum without flange; propodeal spiracle without distal carina; nucha surrounded by very short radiating carinae. Pronotum with a distinct dorsal furrow, dorsally with a well-defined smooth band; central area of pronotum smooth, but both dorsal and ventral furrows with short parallel carinae. Propleuron with fine punctations throughout and dorsally with a carina. Metasternum flat or nearly so. Contour of mesopleuron convex; precoxal groove deep with faintly transverse lineate sculpture; epicnemial ridge elongated more fusiform (tapering at both ends).

**Legs.** Ventral margin of fore telotarsus slightly excavated and with a tiny curved seta, fore telotarsus almost same width throughout and longer than fourth tarsomere (0.10, 0.05). Hind coxa with punctation only on ventral surface and dorsal outer depression present. Inner spur of hind tibia longer than outer spur (0.19, 0.14), entire surface of hind tibia with dense strong spines clearly differentiated by color and length. Hind telotarsus as equal in length as fourth tarsomere (0.10, 0.09).

**Wings** (Fig. 123I, J). Fore wing with r vein slightly curved; 2RS vein straight; r and 2RS veins forming an angle at their junction and outer side of junction not forming a stub; 2M vein slightly curved/swollen; distally fore wing [where spectral veins are] with microtrichiae more densely concentrated than the rest of the wing; anal cell 1/3 proximally lacking microtrichiae; subbasal cell with a small smooth area; vein 2CUa absent and 2CUb spectral; vein 2 cu-a absent; vein 2-1A present only proximally as tubular vein; tubular vein 1 cu-a curved and complete, but junction with 1-1A vein spectral. Hind wing with vannal lobe narrow, subdistally and subproximally evenly convex, and setae present only proximally.

**Metasoma** (Fig. 123A, D, F–H). Metasoma laterally compressed. Petiole on T1 completely smooth and polished, with faint, satin-like sheen, evenly narrowing distally (length 0.26, maximum width 0.14, minimum width 0.07) and with scattered pubescence concentrated in the first distal third. Lateral grooves delimiting the median area on T2 clearly defined and reaching the distal edge of T2 (length median area 0.11, length T2 0.12), edges of median area polished and lateral grooves deep, median area broader than long (length 0.11, maximum width 0.16, minimum width 0.05), T2 with scattered pubescence only distally. T3 longer than T2 (0.17, 0.12) and with scattered pubescence throughout. Pubescence on hypopygium dense.

**Cocoons.** Brown or gray oval cocoons with ordered silk fibers, but covered by a net. Two rows of cordwood cocoons on each side of live larvae and adhered to the leaf substrate.

#### Comments.

Both sexes with slim bodies.

#### Male

(Fig. 124A–I). Coloration and shape similar to female.

#### Etymology.

Josephine Jose Rodriguez is an American entomologist who has been working in Microgastrinae (Braconidae) and in biodiversity studies. Currently, she is an assistant professor of biology at the University of Virginia’s College at Wise, VA, USA.

#### Distribution.

Parasitized caterpillars were collected in Costa Rica, ACG, Sector Mundo Nuevo (Quebrada Tibio Perla, Vado Ficus, and Vado Huacas), Sector San Cristobal (Río Blanco Abajo), Sector Santa Rosa (Alacrán, Bosque Humedo), and Sector Pitilla (Manguera), during July 1992; October 1993; January 1994; May 2007; February 2008; and May 2009 at 260, 290, 330, 375, 470, 490 and 500 m in dry forest, rain forest and dry-rain intergrade forest.

#### Biology.

The lifestyle of this parasitoid species is gregarious. *Mesochorus* (Ichneumonidae: Mesochorinae) was reported as hyperparasitoid.

#### Host.

*Nagaravitrea* (Guenée) (Noctuidae: Stictopterinae) feeding on *Clusiacylindrica* and *Garciniaintermedia* (Clusiaceae). Caterpillars were collected in second, fourth, and fifth instar.

### Glyptapanteles johnburnsi

Taxon classificationAnimaliaHymenopteraBraconidae

Arias-Penna, sp. nov.

http://zoobank.org/7344E22D-E283-48AA-AC84-0F1652901EC5

Figs 125 , 126

#### Female.

Body length 2.02 mm, antenna length 2.02 mm, fore wing length 1.97 mm.

#### Type material.

**Holotype**: COSTA RICA • 1♀; 92-SRNP-2477, DHJPAR0001441; Área de Conservación Guanacaste, Guanacaste, Sector Santa Rosa, Alacrán; dry forest; 260 m; 10.89249, -85.60336; 22.vi.1992; gusaneros leg.; caterpillar collected in fourth instar; cocoons adhered to the larval cuticle; adult parasitoids emerged on 27.vi.1992; (CNC). **Paratypes.** • 12 (3♀, 3♂) (6♀, 0♂); 92-SRNP-2477, DHJPAR0001441; same data as for holotype; (CNC).

#### Other material.

**Reared material.** COSTA RICA: *Área de Conservación Guanacaste*, *Guanacaste*, *Sector Santa Rosa*, *Bosque Humedo*: • 27 (3♀, 3♂) (14♀, 7♂); 89-SRNP-376, DHJPAR0000056; dry forest; 290 m; 10.85145, -85.60801; 26.vi.1989; gusaneros leg.; caterpillar collected in third instar; cocoons adhered to the larval cuticle; adult parasitoids emerged on 08-09.vii.1989, and the caterpillar still quite alive, but has not moved.

*Área de Conservación Guanacaste*, *Guanacaste*, *Sector Santa Rosa*, *Alacrán*: • 5 (2♀, 0♂) (3♀, 0♂); 91-SRNP-1861, DHJPAR0001507; dry forest; 260 m; 10.89249, -85.60336; 17.vii.1991; gusaneros leg.; caterpillar collected in fourth instar; cocoons adhered to the larval cuticle and formed on 20.vii.1991; adult parasitoids emerged on 25.vii.1991. • 29 (3♀, 3♂) (14♀, 9♂); 91-SRNP-2268.1, DHJPAR0001438; same data as for preceding except: 27.vii.1991; cocoons formed on 28.vii.1991; adult parasitoids emerged on 02.viii.1991. • 5 (1♀, 1♂) (1♀, 2♂); 91-SRNP-2309, DHJPAR0001493; same data as for preceding except: 27.vii.1991; caterpillar found with wasp cocoons on its back; date of cocoons not reported; adult parasitoids emerged on 01.viii.1991. • 19 (3♀, 3♂) (4♀, 9♂); 91-SRNP-2322, DHJPAR0001517; same data as for preceding except: 27.vii.1991; oval white cocoons formed on 01.viii.1991; adult parasitoids emerged on 07.viii.1991. • 43 (3♀, 3♂) (19♀, 18♂); 92-SRNP-2139, DHJPAR0001510; same data as for preceding except: 19.vi.1992; caterpillar already with cocoons on back; adult parasitoids emerged on 22.vi.1992 and caterpillar still alive when wasps eclosed. • 26 (3♀, 3♂) (20♀ + 0♂); 92-SRNP-2142, DHJPAR0001487; same data as for preceding except: 19.vi.1992; caterpillar already with cocoons on back; adult parasitoids emerged on 21.vi.1992 and caterpillar still alive when wasps eclosed. • 20 (3♀, 3♂) (4♀, 10♂); 92-SRNP-2142.1, DHJPAR0001495; same data as for preceding except: 19.vi.1992; caterpillar already with cocoons adhered to the larval cuticle; adult parasitoids emerged on 22.vi.1992. • 16 (4♀, 3♂) (0♀, 9♂); 92-SRNP-2158, DHJPAR0000064; 19.vi.1992; cocoons adhered to the larval cuticle and formed on 23.vi.1992; adult parasitoids emerged on 29.vi.1992. • 35 (3♀, 3♂) (8♀, 21♂); 92-SRNP-2423, DHJPAR0001504; same data as for preceding except: 18.vi.1992; cocoons adhered to the larval cuticle and formed on 23.vi.1992; adult parasitoids emerged on 26.vi.1992. • 20 (3♀, 2♂) (15♀, 0♂); 93-SRNP-4903, DHJPAR0001508; same data as for preceding except: 17.viii.1993; oval cocoons adhered to the larval cuticle; date of cocoons not reported; adult parasitoids emerged on 01.ix.1993.

*Área de Conservación Guanacaste*, *Guanacaste*, *Sector Santa Rosa*, *Casetilla*: • 9 (3♀, 3♂) (3♀, 0♂); 93-SRNP-5341, DHJPAR0000075; 250 m; 10.87652, -85.58605; 02.ix.1993; gusaneros leg.; caterpillar collected in fourth instar; cocoons adhered to the larval cuticle; adult parasitoids emerged on 09.ix.1993.

*Área de Conservación Guanacaste*, *Guanacaste*, *Sector Santa Elena*, *Mancha*: • 3 (2♀, 0♂) (1♀, 0♂); 03-SRNP-12417, DHJPAR0000035; 330 m; 10.85273, -85.67419; 30.v.2003; José Cortez leg.; caterpillar collected in fourth instar; cocoons adhered to the larval cuticle and formed 31.v.2003; adult parasitoids emerged on 06.vi.2003.

#### Diagnosis.

**Figure 125.**
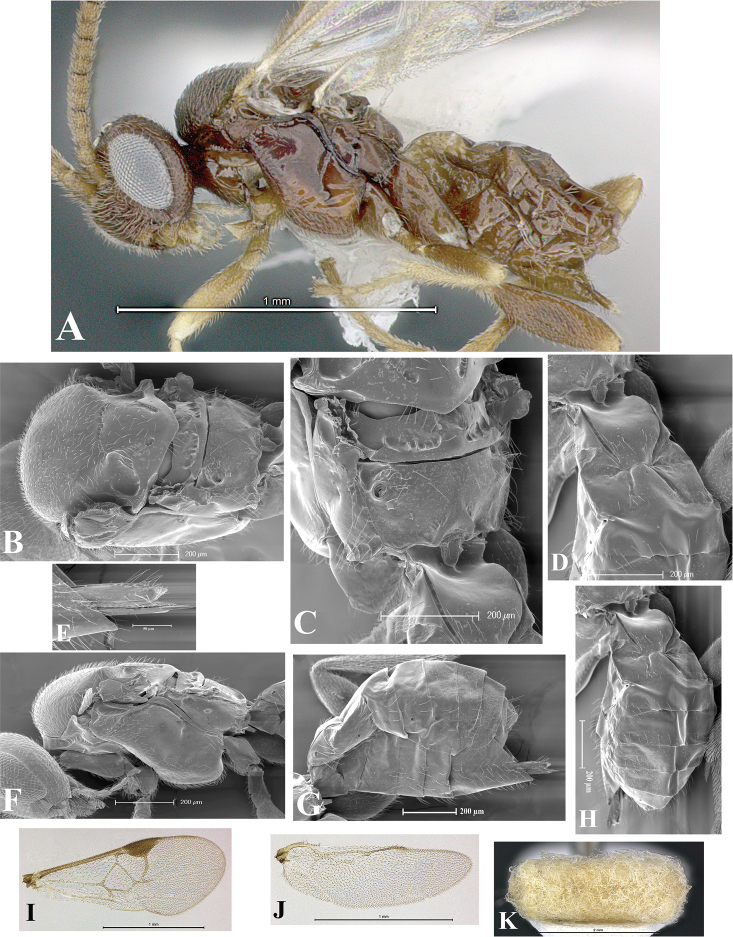
*Glyptapantelesjohnburnsi* sp. nov. female 92-SRNP-2477 DHJPAR0001441 **A** Habitus **B, F** Mesosoma **B** Dorsal view **F** Lateral view **C** Metanotum, propodeum, dorsolateral view **D**T1–3, dorsolateral view **E** Genitalia: hypopygium, ovipositor, ovipositor sheaths, lateral view **G, H** Metasoma **G** Lateral view **H** Dorsolateral view **I, J** Wings **I** Fore **J** Hind **K** Cocoon.

**Figure 126.**
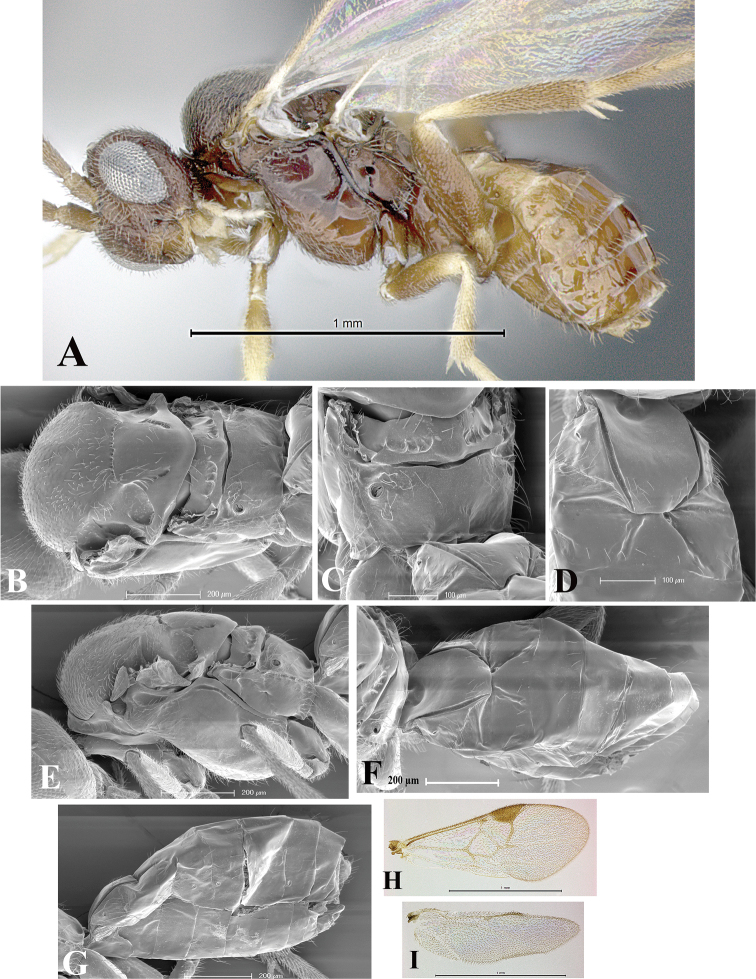
*Glyptapantelesjohnburnsi* sp. nov. male 92-SRNP-2477 DHJPAR0001441 **A** Habitus **B, E** Mesosoma **B** Dorsal view **E** Lateral view **C** Metanotum, propodeum, dorsolateral view **D**T1–2, dorsal view **F, G** Metasoma **F** Dorsal view **G** Lateral view **H, I** Wings **H** Fore **I** Hind.

Nucha without distinct short radiating carinae (Figs 125B, C, 126B, C), proximal half of propodeum straight or nearly so (Figs 125B, C, 126C), antenna as same length as body length, mesoscutum distinctly punctate throughout (Figs 125B, 126B), axillary trough of metanotum proximally with semircular/undulate carina, distally smooth (Figs 125C, 126C), inner margin of eyes diverging slightly at antennal sockets, petiole on T1 virtually parallel-sided, but narrowing over distal 1/3, completely smooth and polished, with faint, satin-like sheen (Figs 125D, 126D), propodeum without median longitudinal carina (Figs 125C, 126C), lateral grooves delimiting the median area on T2 clearly defined and reaching the distal edge of T2 (Figs 125H, 126F), and fore wing with outer side of junction of r and 2RS veins not forming a stub (Figs 125I, 126H).

#### Coloration

(Fig. 125A). General body coloration light brown except scape, pedicel, all antennal flagellomeres (on both sides) and mesosternum yellow-brown; glossa, maxillary and labial palps yellow. Eyes and ocelli silver. Fore and middle legs yellow except coxae, trochanters, trochantellus and proximal half of fore femora brown and middle femora completely brown; hind legs brown except junction between tibiae and femora. Petiole on T1 light brown, although edges remarkably darkened and sublateral areas yellow-brown; T2 with median area light-brown, contours darkened and lateral ends light-brown; T3 and beyond completely light-brown; distally each tergum with a narrow whitish transparent band. In lateral view, all sterna and all terga light brown.

#### Description.

**Head** (Fig. 125A). Head rounded with pubescence long and dense. Proximal three antennal flagellomeres longer than wide (0.13:0.06, 0.14:0.06, 0.13:0.06), distal antennal flagellomere longer than penultimate (0.09:0.05, 0.07:0.05), antenna as same as body length (2.02, 2.02); antennal scrobes-frons shallow. Face flat or nearly so, with scattered and finely punctate, interspaces with microsculpture and longitudinal median carina present. Frons smooth. Temple wide, punctate and interspaces with microsculpture. Inner margin of eyes diverging slightly at antennal sockets; in lateral view, eye anteriorly convex and posteriorly straight. POL shorter than OOL (0.09, 0.11). Malar suture present. Median area between lateral ocelli without depression. Vertex laterally rounded and dorsally wide.

**Mesosoma** (Fig. 125B, C, F). Mesosoma dorsoventrally convex. Mesoscutum with punctation distinct throughout, interspaces wavy/lacunose, distal half with a central dent. Scutellum triangular, apex sloped and fused with BS, scutellar punctation distinct throughout, in profile scutellum flat and on same plane as mesoscutum, phragma of the scutellum partially exposed; BS only very partially overlapping the MPM; ATS demilune with a little and complete parallel carinae; dorsal ATS groove smooth. Transscutal articulation with small and heterogeneous foveae, area just behind transscutal articulation with same kind of sculpture as mesoscutum and nearly at the same level as mesoscutum (flat) or depressed centrally. Metanotum with BM wider than PFM (clearly differentiated); MPM circular without median longitudinal carina; AFM without setiferous lobes and not as well delineated as PFM; PFM thick and smooth; ATM proximally with semircular/undulate carina and distally smooth. Propodeum relatively polished without median longitudinal carina, proximal half straight or nearly so; distal edge of propodeum without flange; propodeal spiracle without distal carina; nucha without distinct short radiating carinae. Pronotum with a distinct dorsal furrow, dorsally with a well-defined smooth band; central area of pronotum and dorsal furrow smooth, but ventral furrow with short parallel carinae. Propleuron finely sculptured only ventrally and dorsally without a carina. Metasternum flat or nearly so. Contour of mesopleuron straight/angulate or nearly so; precoxal groove smooth, shiny and shallow, but visible; epicnemial ridge elongated more fusiform (tapering at both ends).

**Legs.** Ventral margin of fore telotarsus entire without seta, fore telotarsus almost same width throughout, fore telotarsus longer than fourth tarsomere (0.08, 0.04). Dorsal half of hind coxa with scattered punctation, ventral half with dense punctation and dorsal outer depression absent. Inner spur of hind tibia longer than outer spur (0.13, 0.10), entire surface of hind tibia with dense strong spines clearly differentiated by color and length. Hind telotarsus longer than fourth tarsomere (0.10, 0.07).

**Wings** (Fig. 125I, J). Fore wing with r vein slightly curved; 2RS vein straight; r and 2RS veins forming a weak, even curve at their junction and outer side of junction not forming a stub; 2M vein slightly curved/swollen; distally fore wing [where spectral veins are] with microtrichiae more densely concentrated than the rest of the wing; anal cell 1/3 proximally lacking microtrichiae, subbasal cell with a small smooth area; vein 2CUa absent and 2CUb vein spectral; vein 2 cu-a absent; vein 2-1A absent; tubular vein 1 cu-a curved, incomplete/broken and not reaching the edge of 1-1A vein. Hind wing with vannal lobe wide, subdistally straightened and subproximally evenly convex, and setae present only proximally.

**Metasoma** (Fig. 125A, D, E, G, H). Metasoma laterally compressed. Petiole on T1 completely smooth and polished, with faint, satin-like sheen, virtually parallel-sided over most of length, but narrowing over distal 1/3, apex truncate (length 0.28, maximum width 0.17, minimum width 0.05), and with scattered pubescence concentrated in the first distal third. Lateral grooves delimiting the median area on T2 clearly defined and reaching the distal edge of T2 (length median area 0.10, length T2 0.10), edges of median area polished and lateral grooves deep, median area broader than long (length 0.10, maximum width 0.14, minimum width 0.05); T2–3 with a distinctive row of pubescence only at the distal margin. T3 longer than T2 (0.16, 0.10). Pubescence on hypopygium dense.

**Cocoons** (Figs 4H, M, 125K). Beige or white oval cocoons and drum-shaped cocoons with silk fibers evenly smooth. Oval cocoons are somewhat separate from one another and individually adhered to the larval cuticle. Drum-shaped cocoon never eclose because no pupa is inside.

#### Comments.

The shape of the pronotum is convex, thus junction between the distal edge of ventral furrow of the pronotum and the mesopleuron forming a deep hollow. The central area in pronotum is narrow.

#### Male

(Fig. 125A–I). Metasoma coloration lighter than in females.

#### Etymology.

John M. Burns is emeritus curator of Lepidoptera at the Smithsonian Institution, Washington, DC, USA. He is mainly interested in problems at and around the species level, in the process of speciation, and in evolution. He works mostly on a family of distinctive butterflies called skippers (Hesperiidae).

#### Distribution.

Parasitized caterpillars were collected in Costa Rica, ACG, Sector Santa Elena (Mancha) and Sector Santa Rosa (Alacrán, Bosque Humedo, and Casetilla), during June 1989 and 1992, July 1991, August and October 1993, and May 2003 at 250 m, 260 m, 290 m, and 330 m in dry forest.

#### Biology.

The lifestyle of this parasitoid species is gregarious.

#### Host.

*Eunicamalvina* Bates (Fig. 4H, M), *E.caresa* Hewitson and *Eunica* sp. Hübner (Nymphalidae: Biblidinae) feeding on *Mabeaoccidentalis* (Euphorbiaceae). Caterpillars were collected in third and fourth instar. After emerging the parasitoids, caterpillar still quite alive, but has not moved.

### Glyptapanteles johnheratyi

Taxon classificationAnimaliaHymenopteraBraconidae

Arias-Penna, sp. nov.

http://zoobank.org/85D4ED06-AD67-4373-A6CE-C08E7D43CF9B

Figs 127 , 128

#### Female.

Body length 2.27 mm, antenna length 2.30 mm, fore wing length 2.22 mm.

#### Type material.

**Holotype**: COSTA RICA • 1♀; 03-SRNP-9865, DHJPAR0001490; Área de Conservación Guanacaste, Alajuela, Sector San Cristóbal, Cementerio Viejo; rain forest; 570 m; 10.88111, -85.38889; 04.xi.2003; Gloria Sihezar leg.; caterpillar collected in fourt instar; two clusters/masses of small beige cocoons disorganized orientation within the mass, adhered to the leaf, not to the setae of the caterpillar; adult parasitoids emerged on 23.xi.2003; (CNC). **Paratypes.** • 28 (2♀, 2♂) (24♀, 0♂); 03-SRNP-9865, DHJPAR0001490; same data as for holotype; (CNC).

#### Other material.

**Reared material.** COSTA RICA: *Área de Conservación Guanacaste*, *Alajuela*, *Sector San Cristobal*, *Cementerio Viejo*: • 31 (4♀, 3♂) (24♀, 0♂); 03-SRNP-9864, DHJPAR0000273; rain forest; 570 m; 10.88111, -85.38889; 04.xi.2003; Gloria Sihezar leg.; caterpillar collected in fourth instar; two rows of brown cordwood cocoons adjacent, but not sloppily overlapped, cocoons could have been below the caterpillar or to one side, no space for the caterpillar between them; adult parasitoids emerged on 13.xi.2003.

#### Diagnosis.

**Figure 127.**
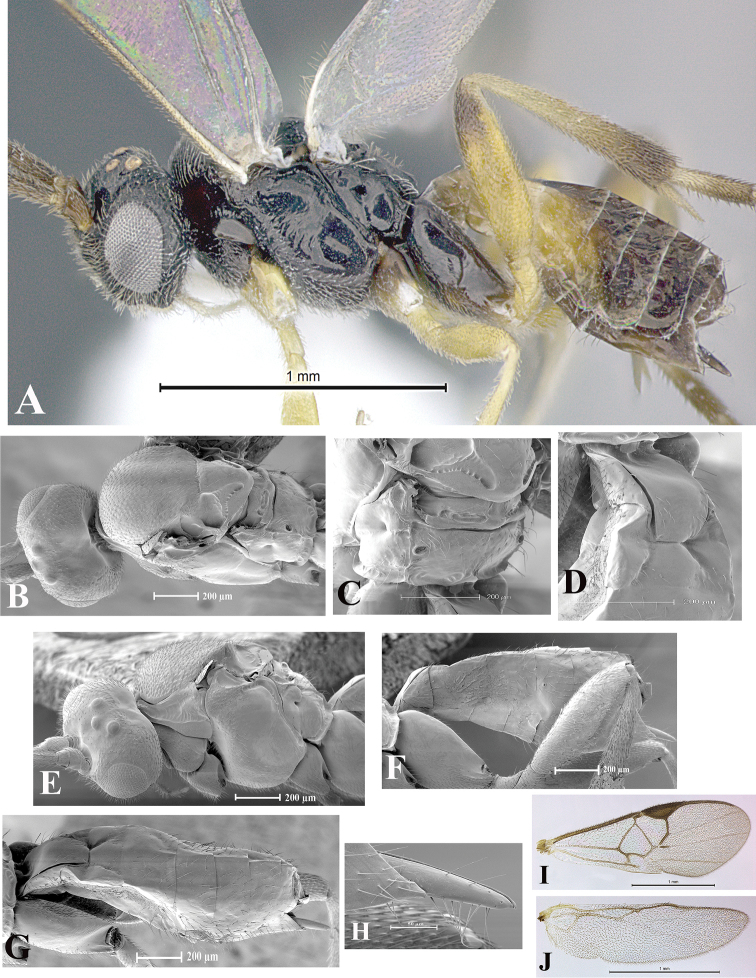
*Glyptapantelesjohnheratyi* sp. nov. female 03-SRNP-9864 DHJPAR0000273, 03-SRNP-9865 DHJPAR0001490 **A** Habitus **B, E** Head, mesosoma **B** Dorsal view **E** Lateral view **C** Metanotum, propodeum, dorsolateral view **D**T1–2, dorsolateral view **F, G** Metasoma **F** Lateral view **G** Dorsolateral view **H** Genitalia: hypopygium, ovipositor, ovipositor sheaths, lateral view **I, J** Wings **I** Fore **J** Hind.

**Figure 128.**
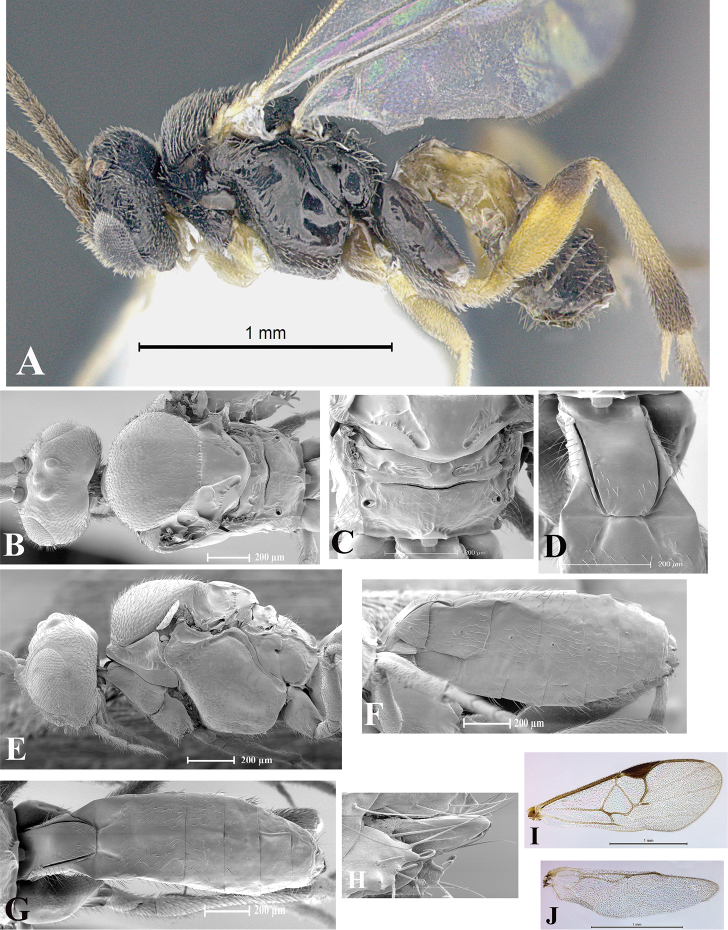
*Glyptapantelesjohnheratyi* sp. nov. male 03-SRNP-9864 DHJPAR0000273, 03-SRNP-9865 DHJPAR0001490 **A** Habitus **B, E** Head, mesosoma **B** Dorsal view **E** Lateral view **C** Scutellum, metanotum, propodeum, dorsal view **D**T1–2, dorsal view **F, G** Metasoma **F** Lateral view **G** Dorsal view **H** Genitalia: parameres, lateral view **I, J** Wings **I** Fore **J** Hind.

Dorsal outer depression on hind coxa present (Figs 127A, 128A), fore telotarsus as equal as fourth tarsomere, antenna slightly longer than body, distal antennal flagellomere longer than penultimate, vertex in dorsal view wide (Figs 127B, 128B), scutellar punctation indistinct throughout (Figs 127B, 128B), shape of proximal half of propodeum weakly curved in dorsal view (Figs 127C, 128C), longitudinal median carina on face present, lateral grooves delimiting the median area on T2 distally losing definition, edges of median area on T2 polished and followed by a deep groove (Figs 127D, 128D), propodeum without median longitudinal carina (Figs 127C, 128C), anteroventral contour of mesopleuron convex (Figs 127A, E, 128A, E), and fore wing with r vein curved, outer side of junction of r and 2RS veins forming a distinct stub (Figs 127I, 128I).

#### Coloration

(Fig. 127A). General body coloration brown-black except pedicel, labrum, mandibles and tegulae yellow-brown; scape and all antennal flagellomeres (on both sides) brown; glossa, maxillary and labial palps yellow. Eyes silver and ocelli reddish (in preserved scpecimen). Fore legs yellow except brown claws; middle legs yellow except coxae yellow-brown and brown claws; hind legs yellow except dark brown coxae, apex of femora brown, most of tibiae brown (coloration intensity increasing from proximal to distal), and tarsomeres brown although basitarsus proximally with a yellow band. Petiole on T1 black with a small yellow/yellow-brown spot in the middle, contours slightly darkened and sublateral areas yellow-brown; T2 with median area dark brown, adjacent area brown, but boundaries smeared with the yellow coloration of lateral ends; T3 and beyond completely brown; distally each tergum with a narrow whitish transparent band. In lateral view, T1–3 yellow-brown; T4 yellow-brown, but dorsally brown; T5 and beyond brown. S1–3 yellow-brown; S4 and beyond brown.

#### Description.

**Head** (Fig. 127A, B, E). Head triangular with short and dense pubescence. Proximal three antennal flagellomeres longer than wide (0.17:0.06, 0.16:0.06, 0.17:0.06), distal antennal flagellomere longer than penultimate (0.10:0.06, 0.08:0.06), antenna longer than body (2.30, 2.27); antennal scrobes-frons shallow. Face flat or nearly so, with dense and fine punctations, interspaces wavy and longitudinal median carina present. Frons smooth. Temple wide, punctate and interspaces clearly smooth. Inner margin of eyes diverging slightly at antennal sockets; in lateral view, eye anteriorly convex and posteriorly straight. POL shorter than OOL (0.09, 0.13). Malar suture present. Median area between lateral ocelli slightly depressed. Vertex laterally rounded and dorsally wide.

**Mesosoma** (Fig. 127A–C, E). Mesosoma dorsoventrally convex. Distal 1/3 of mesoscutum with lateral margin slightly dented, punctation distinct throughout, interspaces wavy/lacunose. Scutellum triangular, apex sloped and fused with BS, scutellar punctation indistinct throughout, in profile scutellum flat and on same plane as mesoscutum, phragma of the scutellum partially exposed; BS only very partially overlapping the MPM; ATS demilune with a little and incomplete parallel carinae only proximally; dorsal ATS groove smooth. Transscutal articulation with small and homogeneous foveae; area just behind transscutal articulation with a smooth and shiny sloped transverse strip. Metanotum with BM wider than PFM (clearly differentiated); MPM circular without median longitudinal carina; AFM without setiferous lobes and not as well delineated as PFM; PFM thick, smooth and with a distal flat flange; ATM proximally with a groove with some sculpturing and distally smooth. Propodeum relatively polished without median longitudinal carina, proximal half weakly curved; distal edge of propodeum with a flange at each side and without stubs; propodeal spiracle without distal carina; nucha surrounded by very short radiating carinae. Pronotum with a distinct dorsal furrow, dorsally with a well-defined smooth band; central area of pronotum smooth, but both dorsal and ventral furrows with short parallel carinae. Propleuron with a mix of rugae and fine punctation, dorsally with a carina. Metasternum flat or nearly so. Contour of mesopleuron convex; precoxal groove deep, smooth and shiny; epicnemial ridge elongated more fusiform (tapering at both ends).

**Legs.** Ventral margin of fore telotarsus entire, but with a tiny curved seta, fore telotarsus almost same width throughout and as equal in length as fourth tarsomere (0.10, 0.09). Hind coxa with punctation only on ventral surface and dorsal outer depression present. Inner spur of hind tibia longer than outer spur (0.21, 0.16), entire surface of hind tibia with dense strong spines clearly differentiated by color and length. Hind telotarsus as equal in length as fourth tarsomere (0.11, 0.11).

**Wings** (Fig. 127I, J). Fore wing with r vein slightly curved; 2RS vein slightly convex to convex; r and 2RS veins forming an angle at their junction and outer side of junction forming a slight stub; 2M vein slightly curved/swollen; distally fore wing [where spectral veins are] with microtrichiae more densely concentrated than the rest of the wing; anal cell 1/3 proximally lacking microtrichiae; subbasal cell with microtrichiae virtually throughout; vein 2CUa absent and vein 2CUb spectral; vein 2 cu-a absent; vein 2-1A present only proximally as tubular vein; tubular vein 1 cu-a curved and complete, but junction with 1-1A vein spectral. Hind wing with vannal lobe narrow, subdistally evenly convex and subproximally straightened, and setae present only proximally.

**Metasoma** (Fig. 127A, D, F–H). Metasoma laterally compressed. Petiole on T1 completely smooth and polished, with faint and satin-like sheen, virtually parallel-sided over most of length, but narrowing over distal 1/3 (length 0.30, maximum width 0.13, minimum width 0.07) and with scattered pubescence concentrated in the first distal third. Lateral grooves delimiting the median area on T2 distally losing definition (length median area 0.10, length T2 0.12), edges of median area polished and lateral grooves deep, median area broader than long (length 0.10, maximum width 0.15, minimum width 0.07); T2 with scattered pubescence only distally. T3 longer than T2 (0.23, 0.12) and with scattered pubescence throughout. Pubescence on hypopygium dense.

**Cocoons.** Light brown or beige oval cocoons with evenly smooth silk fibers. Two rows of brown cordwood cocoons with no space for the caterpillar between them and adhered to the leaf substrate.

#### Male

(Fig. 128A–J). Similar in coloration and shape to female.

#### Etymology.

John M. Heraty is a professor at the University of California, Riverside (UCR), CA, USA. His research is focused on the systematics, phylogeny, and biogeography of the Chalcidoidea (Hymenoptera).

#### Distribution.

Parasitized caterpillars were collected in Costa Rica, ACG, Sector San Cristóbal (Cementerio Viejo), during November 2003 at 570 m in rain forest.

#### Biology.

The lifestyle of this parasitoid species is gregarious.

#### Host.

*Scaptiusvinasia* (Schaus) (Erebidae: Arctiinae) feeding on *Eugeniabasilaris* (Myrtaceae). Caterpillars were collected in forth instar.

### Glyptapanteles johnlasallei

Taxon classificationAnimaliaHymenopteraBraconidae

Arias-Penna, sp. nov.

http://zoobank.org/96A36190-DE1F-4833-8B64-D286FAA9072A

Figs 129 , 130

#### Female.

Body length 2.77 mm, antenna length 3.33 mm, fore wing length 2.92 mm.

#### Type material.

**Holotype**: COSTA RICA • 1♀; 08-SRNP-65265, DHJPAR0030873; Área de Conservación Guanacaste, Alajuela, Sector Brasilia, Piedrona; rain forest; 340 m; 11.01618, -85.35902; 17.iii.2008; Duvalier Briceño leg.; caterpillar collected in second instar; cocoon adhered to the leaf substrate and formed on 24.iii.2008; adult parasitoid emerged on 27.iii.2008; (CNC). **Paratypes**. • 1 (1♀, 0♂) (0♀, 0♂); 08-SRNP-65261, DHJPAR0030867; same data as for holotype except: adult parasitoid emerged on 31.iii.2008; (CNC). • 1 (0♀, 1♂) (0♀, 0♂); 08-SRNP-65270, DHJPAR0030779; same data as for holotype except: small brown cocoon adhered to the leaf substrate and formed on 29.iii.2008; adult parasitoid emerged on 04.iv.2008; (CNC). • 1 (1♀, 0♂) (0♀, 0♂); 08-SRNP-65271, DHJPAR0030766; same data as for holotype except: adult parasitoid emerged on 31.iii.2008; (CNC).

#### Other material.

**Reared material.** COSTA RICA: *Área de Conservación Guanacaste*, *Guanacaste*, *Sector Pitilla*, *Estación Pitilla*: • 1 (0♀, 1♂) (0♀, 0♂); 05-SRNP-31758, DHJPAR0002318; rain forest; 675 m; 10.98931, -85.42581; 07.v.2005; gusaneros leg.; caterpillar collected in third instar; a single beige cocoon adhered to larva and leaf substrate; adult parasitoid emerged on 11.v.2005.

*Área de Conservación Guanacaste*, *Guanacaste*, *Sector Pitilla*, *Loaiciga*: • 1 (1♀, 0♂) (0♀, 0♂); 07-SRNP-32825, DHJPAR0020273; rain forest; 445 m; 11.01983, -85.41342; 18.vii.2007; Petrona Rios leg.; caterpillar collected in third instar; cocoon adhered to the leaf substrate and formed on 4.vii.2007; caterpillar still alive; adult parasitoid emerged on 31.vii.2007.

*Área de Conservación Guanacaste*, *Guanacaste*, *Sector Pitilla*, *Colocho*: • 1 (0♀, 0♂) (1♀, 0♂); 07-SRNP-31412, DHJPAR0012895; rain forest; 375 m; 11.02367, -85.41884; 21.ii.2007; Petrona Rios leg.; caterpillar collected in third instar; a single beige relatively smooth cocoon adhered to the leaf substrate; adult parasitoid emerged on 11.iii.2007. • 1 (0♀, 0♂) (0♀, 1♂ in pieces); 07-SRNP-32626, DHJPAR0020264; same data as for preceding except: 12.vi.2007; caterpillar collected in third instar; cocoon adhered to the leaf substrate and formed on 15.vi.2007; cocoon characteristics not reported; adult parasitoid emerged on 27.vi.2007.

*Área de Conservación Guanacaste*, *Guanacaste*, *Sector Pitilla*, *Quebradona*: • 1 (1♀, 0♂) (0♀, 0♂); 09-SRNP-70346, DHJPAR0035517; rain forest; 475 m; 10.99102, -85.39539; 09.v.2009; Ronald Siezar leg.; caterpillar collected in third instar; small brown hard cocoon adhered to the leaf substrate and formed on 12.v.2009; adult parasitoid emerged on 25.v.2009. • 1 (0♀, 0♂) (1♀, 0♂); 10-SRNP-72979, DHJPAR0042012; same data as for preceding except: 25.ix.2010; Ricardo Calero leg.; caterpillar collected in fourth instar; cocoon adhered to the leaf substrate and formed on 01.x.2010; cocoon characteristics not reported; adult parasitoid emerged on 10.x.2010.

*Área de Conservación Guanacaste*, *Guanacaste*, *Sector Pitilla*, *Cano*: • 1 (0♀, 0♂) (1♀, 0♂); 11-SRNP-70915, DHJPAR0043109; rain forest; 490 m; 10.9954, -85.39980; 26.iv.2011; Ricardo Calero leg.; caterpillar collected in third instar; cocoon adhered to the leaf substrate and formed on 05.v.2011; adult parasitoid emerged on 17.v.2011.

*Área de Conservación Guanacaste*, *Guanacaste*, *Sector Del Oro*, *Guacimos*: • 1 (0♀, 0♂) (1♀, 0♂); 08-SRNP-20609, DHJPAR0020722; dry-rain intergrade forest; 380 m; 11.01454, -85.47492; 15.ii.2008; Elieth Cantillano leg.; caterpillar collected in second instar; cocoon adhered to the leaf substrate and formed on 19.ii.2008; adult parasitoid emerged on 02.iii.2008.

*Área de Conservación Guanacaste*, *Guanacaste*, *Sector Del Oro*, *Quebrada Lajosa*: • 1 (0♀, 0♂) (0♀, 1♂); 09-SRNP-21378, DHJPAR0041633; dry-rain intergrade forest; 400 m; 11.03306, -85.42876; 09.vi.2009; Elieth Cantillano leg.; caterpillar collected in third instar; cocoon adhered to the leaf substrate; adult parasitoid emerged on 19.vi.2008.

*Área de Conservación Guanacaste*, *Alajuela*, *Sector Brasilia*, *Moga*: • 1 (0♀, 0♂) (0♀, 1♂ in pieces); 08-SRNP-65661, DHJPAR0031115; rain forest; 320 m; 11.01227, -85.34929; 01.vii.2008; Duvalier Briceño leg.; caterpillar collected in second instar; cocoon adhered to the leaf substrate and formed on 03.vii.2008; adult parasitoid emerged on 10.vii.2008.

#### Diagnosis.

**Figure 129.**
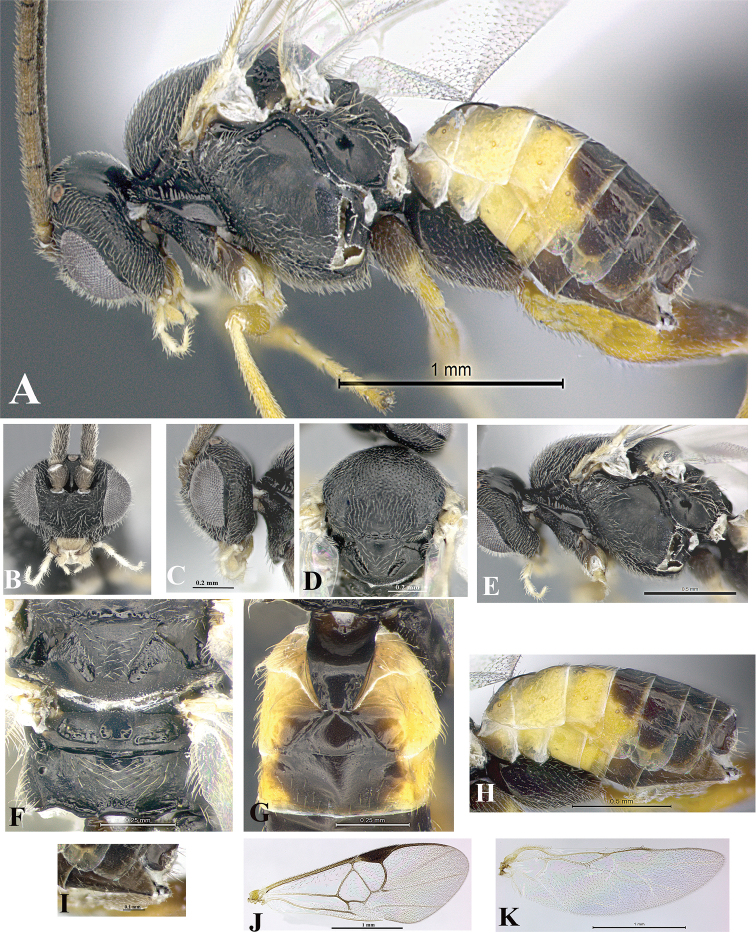
*Glyptapantelesjohnlasallei* sp. nov. female 07-SRNP-32561 DHJPAR0020272, 08-SRNP-65265 DHJPAR0030873 **A** Habitus **B, C** Head **B** Dorsal view **E** Lateral view **D** Mesonotum, dorsal view **E** Mesosoma, lateral view **F** Scutellum, metanotum, propodeum, dorsal view **G**T1–3, dorsal view **H** Metasoma, lateral view **I** Genitalia: hypopygium, ovipositor, ovipositor sheaths, lateral view **J, K** Wings **J** Fore **K** Hind.

**Figure 130.**
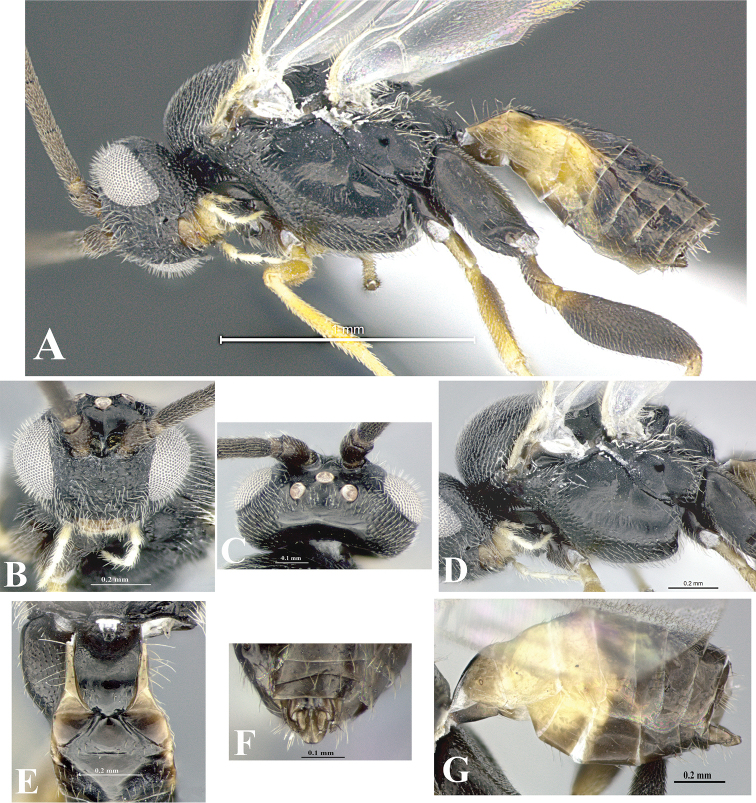
*Glyptapantelesjohnlasallei* sp. nov. male 05-SRNP-31758 DHJPAR0002318, 08-SRNP-65270 DHJPAR0030779 **A** Habitus **B, C** Head **B** Frontal view **C** Dorsal view **D** Mesosoma, lateral view **E**T1–2, dorsal view **F** Genitalia: parameres, ventral view **G** Metasoma, lateral view.

Edges of median area on T2 polished and followed by a deep groove (Figs 129G, 130E), scutellar punctation only on distal half (Fig. 129D, F), in lateral view, metasoma laterally compressed (Figs 129A, H, 130A, 130G), inner margin of eyes straight throughout (Figs 129B, 130B), petiole on T1 finely sculptured only laterally (Figs 129G, 130E), propodeum without median longitudinal carina (Fig. 129F), lateral grooves delimiting the median area on T2 clearly defined and reaching the distal edge of T2 (Figs 129G, 130E), and fore wing with outer side of junction of r and 2RS veins not forming a stub (Fig. 129J).

#### Coloration

(Fig. 129A–K). General body coloration black except first five proximal antennal flagellomeres dorsally lighter (light brown) than ventrally (dark brown), remaining flagellomeres dark brown on both sides; scape and pedicel yellow-brown and with lateral light brown areas; labrum and mandibles yellow-brown; glossa, maxillary and labial palps, and tegulae yellow. Eyes gray and ocelli reddish (in preserved specimen). Fore and middle legs yellow black except coxae and brown claws; hind legs yellow except black coxae, distal half of the femora brown and proximal half orange-yellow, most of tibiae and tarsomeres brown although basitarsus with a narrow yellow band proximally. Petiole on T1 black and sublateral areas yellow; T2 with median area black, adjacent area dark brown (both areas forming a rectangle-shaped) and lateral ends yellow; T3 mostly brown with lateral ends brown; T4 and beyond completely brown; distally each tergum with a yellowish transparent band. In lateral view, T1–3 completely yellow; T4 yellow, dorsally brown; T5 and beyond brown. S1–4 completely yellow; penultimate sternum and hypopygium completely brown.

#### Description.

**Head** (Fig. 129A–C). Head rounded with pubescence long and dense. Proximal three antennal flagellomeres longer than wide (0.24:0.08, 0.23:0.08, 0.23:0.08), distal antennal flagellomere longer than penultimate (0.14:0.06, 0.11:0.06), antenna longer than body (3.33, 2.77); antennal scrobes-frons shallow. Face with fine punctate, with depression only laterally, interspaces with microsculpture and longitudinal median carina present. Frons smooth. Temple wide, punctate and interspaces wavy. Inner margin of eyes straight throughout; in lateral view, eye anteriorly convex and posteriorly straight. POL shorter than OOL (0.09, 0.12). Malar suture present. Median area between lateral ocelli without depression. Vertex laterally rounded and dorsally wide.

**Mesosoma** (Fig. 129A, D–F). Mesosoma dorsoventrally convex. Mesoscutum proximally convex and distally flat, punctation distinct throughout, interspaces with microsculpture. Scutellum triangular, apex sloped and fused with BS, scutellar punctation present only distal half, in profile scutellum flat and on same plane as mesoscutum, phragma of the scutellum partially exposed; BS not overlapping the MPM; ATS demilune inner side with a row of foveae; dorsal ATS groove with carinae only proximally. Transscutal articulation with small and heterogeneous foveae, area just behind transscutal articulation depressed centrally, smooth and shiny. Metanotum with BM wider than PFM (clearly differentiated); MPM circular without median longitudinal carina; AFM without setiferous lobes and not as well delineated as PFM; PFM thick, smooth and with a distal flat flange; ATM proximally with a groove with some sculpturing and distally smooth. Propodeum without median longitudinal carina, proximal half curved with medium-sized sculpture and distal half relatively polished; distal edge of propodeum with a flange at each side and without stubs; propodeal spiracle without distal carina; nucha surrounded by very short radiating carinae. Pronotum with a distinct dorsal furrow, dorsally with a well-defined smooth band; central area of pronotum smooth, but both dorsal and ventral furrows with short parallel carinae. Propleuron with fine punctations throughout and dorsally without a carina. Metasternum flat or nearly so. Contour of mesopleuron straight/angulate or nearly so; precoxal groove deep with faintly transverse lineate sculpture; epicnemial ridge convex, teardrop-shaped.

**Legs.** Ventral margin of fore telotarsus slightly excavated and with a tiny curved seta, fore telotarsus almost same width throughout and longer than fourth tarsomere (0.12, 0.06). Hind coxa with punctation only on ventral surface and dorsal outer depression present. Inner spur of hind tibia longer than outer spur (0.31, 0.21), entire surface of hind tibia with dense strong spines clearly differentiated by color and length. Hind telotarsus as equal in length as fourth tarsomere (0.13, 0.12).

**Wings** (Fig. 129J, K). Fore wing with r vein curved; 2RS vein slightly concave; r and 2RS veins forming a weak, even curve at their junction and outer side of junction not forming a stub; 2M vein slightly curved/swollen; distally fore wing [where spectral veins are] with microtrichiae more densely concentrated than the rest of the wing; anal cell 1/3 proximally lacking microtrichiae; subbasal cell with a small smooth area; vein 2CUa absent and vein 2CUb spectral; vein 2 cu-a absent; vein 2-1A present only proximally as tubular vein; tubular vein 1 cu-a straight, incomplete/broken and not reaching the edge of 1-1A vein. Hind wing with vannal lobe narrow, subdistally and subproximally straightened, and setae present only proximally.

**Metasoma** (Fig. 129A, G–I). Metasoma laterally compressed. Petiole finely sculptured only laterally, parallel-sided in proximal half and then narrowing (length 0.41, maximum width 0.22, minimum width 0.10) and with scattered pubescence concentrated in the first distal third. Lateral grooves delimiting the median area on T2 clearly defined and reaching the distal edge of T2 (length median area 0.16, length T2 0.16), edges of median area polished and lateral grooves deep, median area broader than long (length 0.16, maximum width 0.32, minimum width 0.10), T2 with scattered pubescence only distally. T3 longer than T2 (0.24, 0.16) and with scattered pubescence throughout. Pubescence on hypopygium dense.

**Cocoon.** Beige or brown oval cocoon with ordered silk fibers, but covered by a net. Cocoon adhered to the leaf substrate.

#### Comments.

In some specimens, the petiole, the median area on T2 and the terga are polished black. In other specimens, laterally the metasoma coloration is yellow-brown instead of yellow. The maximum width of median area on T2 is wider in comparison with other species. Both sexes with stout bodies.

#### Male

(Fig. 130A–G). The coloration on the femora is nearly completely black. Dorsally, the petiole is black, the sublateral areas are yellow-brown/light brown and the median area on T2 as well as the remaining terga are dark brown. The S1–4 are yellow, although medially they are brown, and the remaining sterna brown.

#### Etymology.

John La Salle (25 February 1951-27 May 2018) was an entomologist interested in the systematics of parasitoid Hymenoptera using new technology for the generation and delivery of insect knowledge. He worked with the CSIRO (The Commonwealth Scientific and Industrial Research Organisation) and played a key role in establishing the “Atlas of Living Australia”.

#### Distribution.

Parasitized caterpillars were collected in Costa Rica, ACG, Sector Brasilia (Moga and Piedrona), Sector Del Oro (Guacimos and Quebrada Lajosa), and Sector Pitilla (Cano, Colocho, Estación Pitilla, Loaiciga, and Quebradona), during May 2005, February and June-July 2007, February-March and July 2008, May-June 2009, October 2010, and April 2011 at 320 m, 340 m, 375 m, 380 m, 400 m, 445 m, 475 m, 490 m, and 675 m in rain and dry-rain intergrade forests.

#### Biology.

The lifestyle of this parasitoid species is solitary.

#### Host.

*Sericochroa* sp. Felder (Notodontidae: Heterocampinae) feeding on *Vochysiaferruginea* and *V.guatemalensis* (Vochysiaceae). Caterpillars were collected in second, third, and fourth instar.

### Glyptapanteles johnnoyesi

Taxon classificationAnimaliaHymenopteraBraconidae

Arias-Penna, sp. nov.

http://zoobank.org/B6B30BF3-8599-405B-8E47-76BF4B2C4B6E

Figs 131 , 132

#### Female.

Body length 2.68 mm, antenna length 3.33 mm, fore wing length 2.68 mm.

#### Type material.

**Holotype**: COSTA RICA • 1♀; 06-SRNP-32352, DHJPAR0012013; Área de Conservación Guanacaste, Guanacaste, Sector Pitilla, Sendero Mismo; rain forest; 680 m; 10.98758, -85.41967; 18.vi.2006; Manuel Rios leg.; caterpillar collected in third instar; cocoon adhered to larva and the leaf substrate; adult parasitoid emerged on 26.vi.2006; (CNC). **Paratype**. • 1 (0♀, 1♂) (0♀, 0♂); 06-SRNP-32383, DHJPAR0012021; same data as for holotype except: adult parasitoid emerged on 29.vi.2006; (CNC).

#### Other material.

**Reared material.** COSTA RICA: *Área de Conservación Guanacaste*, *Guanacaste*, *Sector Pitilla*, *Sendero Cuestona*: • 1 (0♀, 0♂) (1♀, 0♂); 05-SRNP-31619, DHJPAR0002894; rain forest; 640 m; 10.99455, -85.41461; 21.iv.2005; Petrona Rios leg.; caterpillar collected in fourth instar; cocoon adhered to larva and the leaf substrate; adult parasitoid emerged on 08.v.2005. • 1 (1♀, 0♂) (0♀, 0♂); 06-SRNP-32559, DHJPAR0012014; same data as for preceding except: 25.vi.2006; caterpillar collected in third instar; single gray beige cocoon glued to midrib of leaf, cocoon formed on 29.vi.2006; adult parasitoid emerged on 10.vii.2006.

*Área de Conservación Guanacaste*, *Guanacaste*, *Sector Pitilla*, *Estación Pitilla*: • 1 (0♀, 1♂) (0♀, 0♂); 06-SRNP-32635, DHJPAR0012098; rain forest; 675 m; 10.98931, -85.42581; 30.vi.2006; Calixto Moraga leg.; caterpillar collected in second instar; a single beige cocoon adhered to the leaf substrate, cocoon formed on 06.vii.2006; adult parasitoid emerged on 15.vii.2006.

*Área de Conservación Guanacaste*, *Guanacaste*, *Sector Pitilla*, *Medrano*: • 1 (0♀, 0♂) (0♀, 1♂); 11-SRNP-70805, DHJPAR0043003; rain forest; 380 m; 11.01602, -85.38053; 01.iv.2011; Ricardo Calero leg.; caterpillar collected in fourth instar; cocoon adhered to the larval cuticle and formed on 02.iv.2011; adult parasitoid emerged on 24.iv.2011.

*Área de Conservación Guanacaste*, *Guanacaste*, *Sector Pitilla*, *Manguera*: • 1 (0♀, 0♂) (1♀, 0♂); 11-SRNP-71277, DHJPAR0045272; rain forest; 470 m; 10.99590, -85.39842; 10.vi.2011; Ricardo Calero leg.; caterpillar collected in third instar; cocoon adhered to the larval cuticle and formed on 14.vi.2011; adult parasitoid emerged on 20.vi.2011. • 1 (0♀, 0♂) (0♀, 1♂); 11-SRNP-71279, DHJPAR0045278; same data as for preceding.

*Área de Conservación Guanacaste*, *Alajuela*, *Sector Rincon Rain Forest*, *San Lucas*: • 1 (0♀, 0♂) (1♀, 0♂); 09-SRNP-41059, DHJPAR0035362; rain forest; 320 m; 10.91847, -85.30338; 13.v.2009; José Pérez leg.; caterpillar collected in fourth instar; a single dark cocoon adhered to the leaf substrate and formed on 18.v.2009; adult parasitoid emerged on 25.v.2009.

*Área de Conservación Guanacaste*, *Alajuela*, *Sector Brasilia*, *Brumas*: • 1 (0♀, 0♂) (0♀, 1♂); 11-SRNP-65787, DHJPAR0045359; rain forest; 10.vii.2011; Duvalier Briceño leg.; caterpillar collected in second instar; cocoon adhered to the leaf substrate and formed on 05.viii.2011; adult parasitoid emerged on 15.viii.2011.

#### Coloration

**Figure 131.**
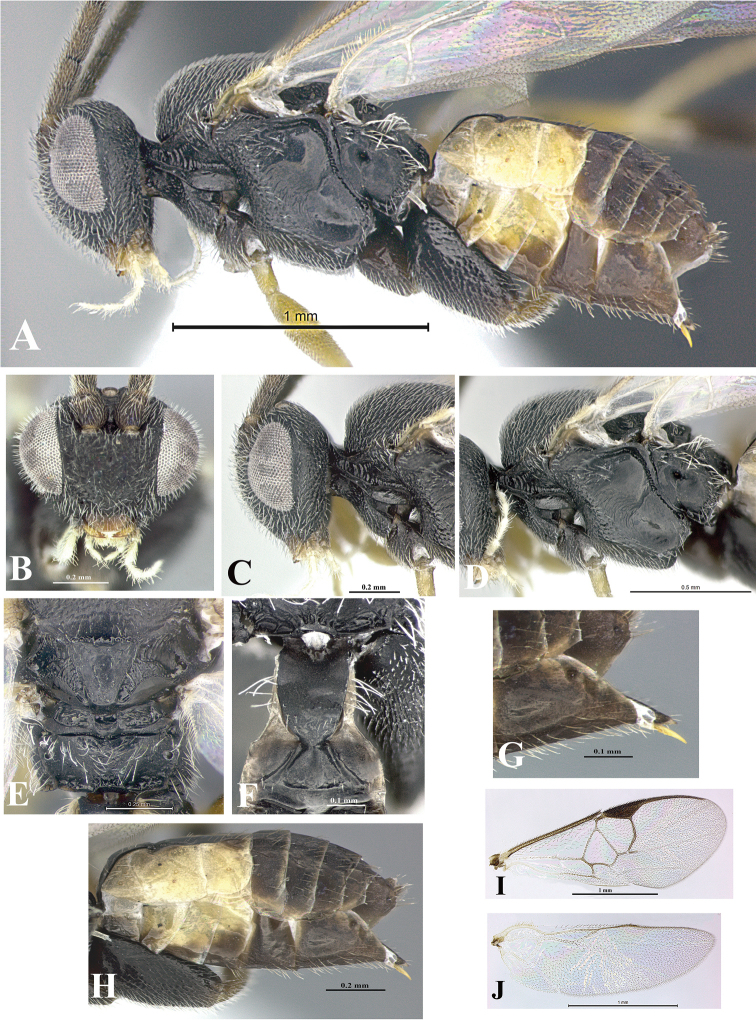
*Glyptapantelesjohnnoyesi* sp. nov. female 06-SRNP-32352 DHJPAR0012013, 06-SRNP-32559 DHJPAR0012014, 11-SRNP-70940 DHJPAR0043020 **A** Habitus **B, C** Head, frontal view **C** Head, pronotum, propleuron, lateral view **D** Mesosoma, lateral view **E** Scutellum, metanotum, propodeum, dorsal view **F**T1–2, dorsal view **G** Genitalia: hypopygium, ovipositor, ovipositor sheaths, lateral view **H** Metasoma, lateral view **I, J** Wings **I** Fore **J** Hind.

(Fig. 131A–J). General body coloration black except labrum and mandibles yellow-brown; scape and all antennal flagellomeres (on both sides) brown; pedicel brown, but distally yellow-brown; glossa, maxillary and labial palps, and tegulae yellow. Eyes silver and ocelli reddish (in preserved specimen). Fore and middle legs yellow except coxae, middle femora with a ventral brown strip, and claws brown; hind legs yellow except black coxae, femora completely dark brown, and most of tibia and tarsomeres brown, although basitarsus proximally with a band yellow. Petiole on T1 black and sublateral areas light yellow-brown; T2 with median area dark brown, adjacent area brown which boundaries blurred with the yellow of lateral ends; T3 and beyond completely brown; distally each tergum with a narrow yellowish band. In lateral view, T1–3 yellow; T4 and beyond brown. S1–3 yellow; S4 yellow, but medially brown; penultimate sternum and hypopygium brown.

#### Description.

**Head** (Fig. 131A–C). Head rhomboid with pubescence long and dense. Proximal three antennal flagellomeres longer than wide (0.26:0.07, 0.24:0.07, 0.24:0.07), distal antennal flagellomere subequal in length with penultimate (0.13;0.05, 0.12:0.05), antenna longer than body (3.33, 2.68); antennal scrobes-frons shallow. Face flat or nearly so, with dense fine punctations, interspaces with microsculpture, and longitudinal median carina present. Frons smooth. Temple wide, punctate and interspaces with microsculpture. Inner margin of eyes straight throughout; in lateral view, eye anteriorly convex and posteriorly straight. POL shorter than OOL (0.09, 0.30). Malar suture absent or difficult to see. Median area between lateral ocelli slightly depressed. Vertex laterally rounded and dorsally wide.

**Mesosoma** (Fig. 131A, D, E). Mesosoma dorsoventrally convex. Distal 1/3 of mesoscutum with lateral margin slightly dented, punctation distinct throughout, interspaces with microsculpture. Scutellum triangular, apex sloped and fused with BS, scutellar punctation scattered throughout, in profile scutellum flat and on same plane as mesoscutum, phragma of the scutellum completely concealed; BS only very partially overlapping the MPM; ATS demilune with short stubs delineating the area; dorsal ATS groove with semicircular/parallel carinae. Transscutal articulation with small and heterogeneous foveae, area just behind transscutal articulation with a smooth and shiny sloped transverse strip. Metanotum with BM wider than PFM (clearly differentiated); MPM semicircular without median longitudinal carina; AFM without setiferous lobes and not as well delineated as PFM; PFM thick, smooth and with a distal flat flange; ATM proximally with sculpture distally without a well delimited smooth area. Propodeum with medium-sized punctation, without median longitudinal carina and proximal half curved; distal edge of propodeum with a flange at each side and without stubs; propodeal spiracle without distal carina; nucha surrounded by very short radiating carinae. Pronotum with a distinct dorsal furrow, dorsally with a well-defined smooth band; central area of pronotum smooth, but both dorsal and ventral furrows with short parallel carinae. Propleuron with fine punctations throughout and dorsally with a carina. Metasternum flat or nearly so. Contour of mesopleuron straight/angulate or nearly so; precoxal groove deep with transverse lineate sculpture; epicnemial ridge elongated more fusiform (tapering at both ends).

**Legs.** Ventral margin of fore telotarsus slightly excavated and with a tiny curved seta, fore telotarsus almost same width throughout and longer than fourth tarsomere (0.12, 0.07). Hind coxa with punctation only on ventral surface and dorsal outer depression present. Inner spur of hind tibia longer than outer spur (0.26, 0.17), entire surface of hind tibia with dense strong spines clearly differentiated by color and length. Hind telotarsus as equal in length as fourth tarsomere (0.11, 0.10).

**Wings** (Fig. 131I, J). Fore wing with r vein straight; 2RS vein straight; r and 2RS veins forming an angle at their junction and outer side of junction not forming a stub; 2M vein slightly curved/swollen; distally fore wing [where spectral veins are] with microtrichiae more densely concentrated than the rest of the wing; anal cell 1/3 proximally lacking microtrichiae; subbasal cell with a small smooth area; vein 2CUa absent and vein 2CUb spectral; vein 2 cu-a absent; vein 2-1A present only proximally as tubular vein; tubular vein 1 cu-a curved, incomplete/broken and not reaching the edge of 1-1A vein. Hind wing with vannal lobe narrow, subdistally and subproximally straightened, and setae present only proximally.

**Metasoma** (Fig. 131A, F–H). Metasoma laterally compressed. Petiole on T1 completely smooth and polished, with faint, satin-like sheen, parallel-sided in proximal half and then narrowing (length 0.33, maximum width 0.20, minimum width 0.10), and with scattered pubescence concentrated in the first distal third. Lateral grooves delimiting the median area on T2 clearly defined and reaching the distal edge of T2 (length median area 0.16, length T2 0.16), edges of median area polished and lateral grooves deep, median area broader than long (length 0.16, maximum width 0.25, minimum width 0.08); T2 with scattered pubescence only distally. T3 longer than T2 (0.21, 0.16) and with scattered pubescence throughout. Pubescence on hypopygium dense.

**Cocoon** (Fig. 4W). Gray or beige oval cocoon with silk fibers messy/disordered/fluffy. Cocoon adhered to larva and the leaf substrate.

#### Comments.

In some specimens (e.g., 06-SRNP-32559) the sterna are completely dark brown-black (sterna are shrunken); the petiole on T1 is black, the median and the adjacent areas on T2 are black, but lateral ends brown.

#### Male

**Figure 132.**
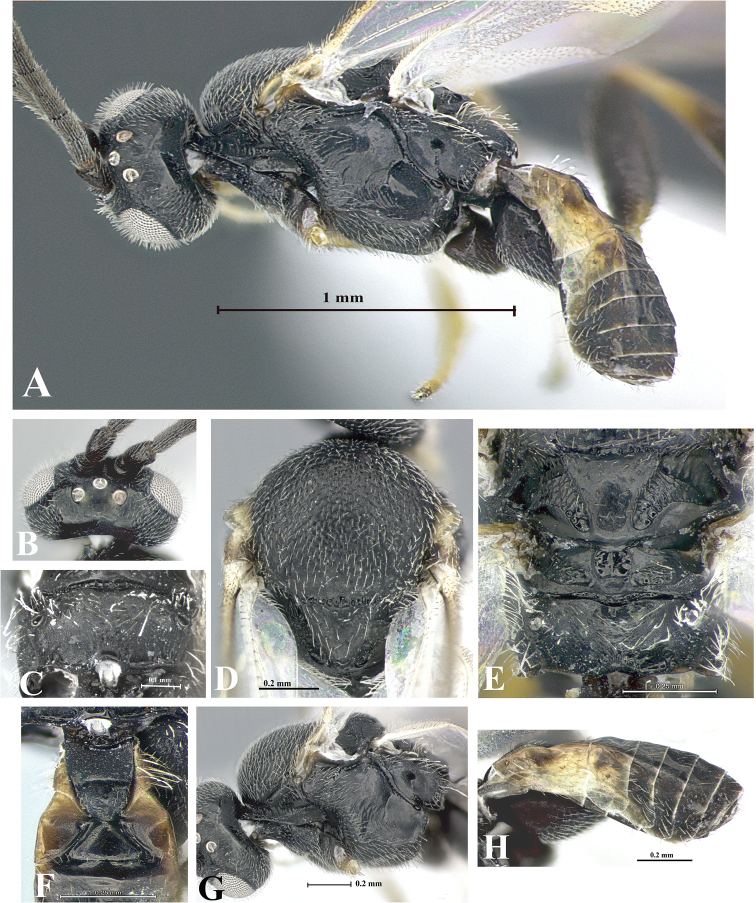
*Glyptapantelesjohnnoyesi* sp. nov. male 06-SRNP-32635 DHJPAR0012098 **A** Habitus **B** Head, dorsal view **C** Propodeum, dorsal view D Mesonotum, dorsal view **E** Scutellum, metanotum, propodeum, dorsal view **F**T1–2, dorsal view **G** Mesosoma, lateral view **H** Metasoma, lateral view.

(Fig. 132A–H). Similar to female, however some specimens (e.g., 06-SRNP-32635) have the sterna completely dark brown-black (possibly because of the shranked sterna); in lateral view, the coloration from T1 to T3 is yellow-brown, but dorsally is brown; the petiole is black, the median and the adjacent areas on T2 are black, but the lateral ends are brown; the coloration on middle femora is completely dark yellow-brown or brown.

#### Etymology.

John Stuart Noyes is a retired British entomologist worked at the Natural History Museum, London, UK. He is well known for his outstanding research work on the biosystematics of Chalcidoidea. Currently, he is revising the 1,500 plus species of Encyrtidae found in Costa Rica.

#### Distribution.

Parasitized caterpillars were collected in Costa Rica, ACG, Sector Brasilia (Brumas), Sector Pitilla (Estación Pitilla, Manguera, Sendero Cuestona, Sendero Mismo, and Medrano), and Sector Rincon Rain Forest (San Lucas), during April 2005, June 2006, May 2009, and April and June-July 2011 at 320 m, 380 m, 470 m, 640 m, 675 m, and 680 m in rain forest.

#### Biology.

The lifestyle of this parasitoid species is solitary.

#### Host.

*Deinopasigniplena* Walker (Erebidae: Calpinae) (Fig. 4W) feeding on *Swartziacostaricensis* (Fabaceae) and *D.biligula* Guenée feeding on *Pterocarpushayesii* (Fabaceae). Caterpillars were collected in second, third, and fourth instar.

### Glyptapanteles johnstiremani

Taxon classificationAnimaliaHymenopteraBraconidae

Arias-Penna, sp. nov.

http://zoobank.org/B0BB9C98-AD6A-469E-A0CA-1678DB3A3AB2

Figs 133 , 134

#### Female.

Body length 2.88 mm, antenna [incomplete], fore wing length 3.68 mm.

#### Type material.

**Holotype**: ECUADOR • 1♀; EC-5396, YY-A127; Napo, Yanayacu Biological Station, tanque de agua, Papallacta, Finca ganadera, cerca de Guango, Plot 41; cloud forest; 2,876 m; -0.366667, -78.1; 15.vii.2005; Toni Walters leg.; caterpillar collected in first instar; cocoon formed on 15.viii.2005; adult parasitoid emerged on 27.viii.2005; (PUCE). **Paratypes.** • 1 (0♀, 1♂) (0♀, 0♂); EC-4324, YY-A190; Napo, Yanayacu Biological Station, Yanayacu Road, Plot 8; cloud forest; 2,112 m; -0.6, -77.883333; 07.vi.2005; Genoveva Rodriguez-Castañeda leg.; caterpillar collected in second instar; cocoon formed on 26.vi.2005; adult parasitoid emerged on 07.vii.2005; (PUCE). • 1 (0♀, 1♂) (0♀, 0♂); EC-9101, YY-A209; Napo, Yanayacu Biological Station, Río Chalpi Grande, Plot 112; cloud forest; 2,768 m; -0.366667, -78.083333; 24.x.2005; Rafael Granizo leg.; caterpillar collected in late instar or prepupal; adult parasitoid emerged on 11.xi.2005; (PUCE).

#### Diagnosis.

**Figure 133.**
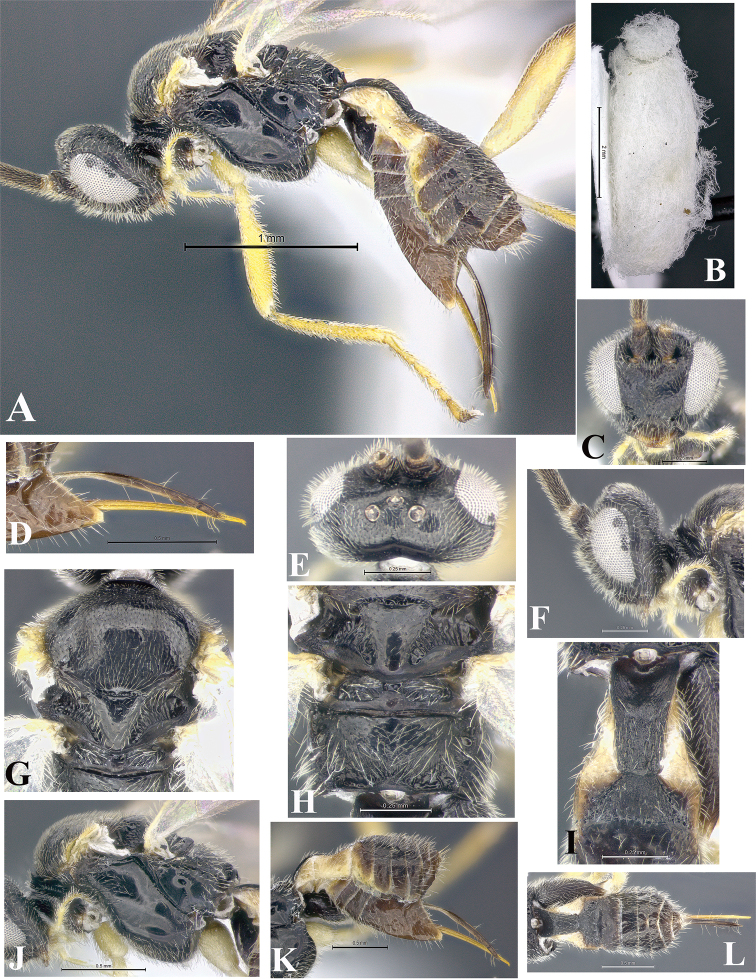
*Glyptapantelesjohnstiremani* sp. nov. female EC-5396 YY-A127 **A** Habitus **B** Cocoon **C, E** Head **C** Frontal view **E** Dorsal view **D** Genitalia: hypopygium, ovipositor, ovipositor sheaths, lateral view **F** Head, pronotum, propleuron, lateral view **G** Mesonotum, dorsal view **H** Scutellum, metanotum, propodeum, dorsal view **I**T1–2, dorsal view **J** Mesosoma, lateral view **K, L** Metasoma **K** Dorsal view **L** Lateral view.

**Figure 134.**
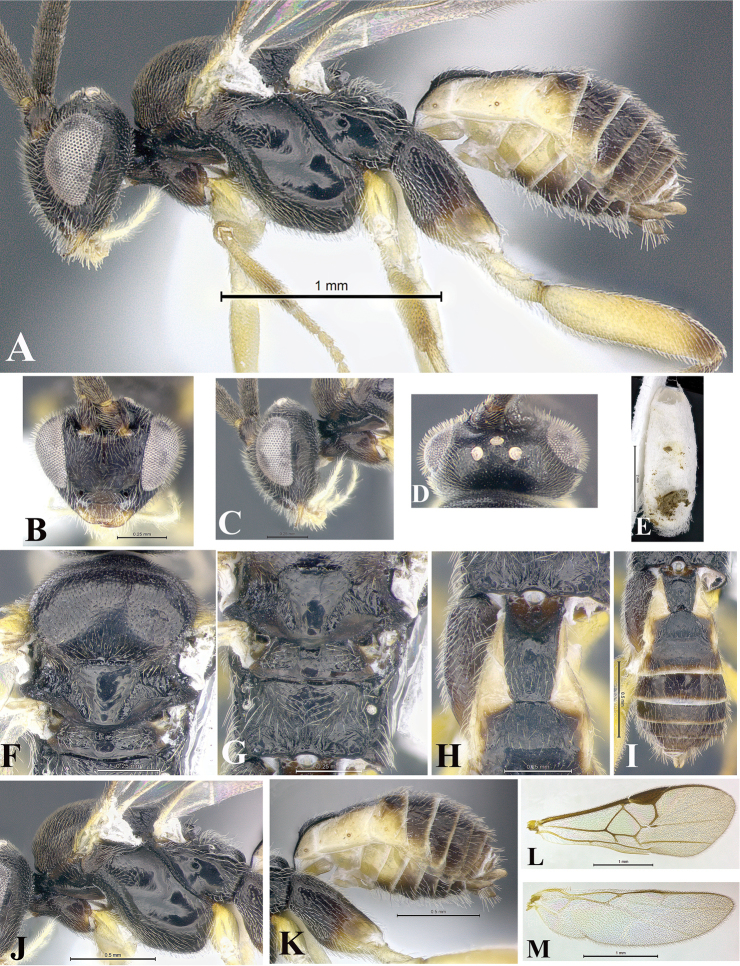
*Glyptapantelesjohnstiremani* sp. nov. male EC-4324 YY-A190 **A** Habitus **B, D** Head **B** Frontal view **D** Dorsal view **C** Head, pronotum, propleuron, lateral view **E** Cocoon **F** Mesonotum, dorsal view **G** Scutellum, metanotum, propodeum, dorsal view **H**T1–2, dorsal view **I, K** Metasoma **I** Dorsal view **K** Lateral view **J** Mesosoma, lateral view **L, M** Wings **L** Fore **M** Hind.

Distal 1/3 of mesoscutum with lateral margin slightly dented (Figs 133G, 134F), medioposterior band of scutellum mostly overlapping the medioanterior pit of metanotum (Figs 133G, H, 134F, G), median area on T2 broader than long, distally with lateral margins relatively straight, lateral grooves delimiting the median area clearly defined and reaching the distal edge of T2, edges of median area polished and followed by a deep groove (Figs 133I, 134H, I), scutellum in profile flat (Figs 133A, 134J), fore wing with vein 2-1A tubular throughout, r vein curved, outer side of junction of r and 2RS veins forming a distinct stub (Fig. 134L), dorsal carina delimiting a dorsal furrow on propleuron absent (Fig. 134C, J), anterior furrow of metanotum without setiferous lobes (Figs 133G, H, 134F, G), axillary trough of scutellum with sculpture (Figs 133G, H, 134F, G), propodeum without median longitudinal carina (Figs 133H, 134G), and anteroventral contour of mesopleuron convex (Figs 133A, J, 134A, J).

#### Coloration

(Fig. 133A–L). General body coloration polished black except pedicel brown with distal yellow-brown ring; scape and all antennal flagellomeres (on both sides) brown; labrum and mandibles yellow-brown; glossa, maxillary and labial palps, and tegulae yellow; labrum, mandibles, apex of propleuron, dorsal furrow of pronotum, and epicnemial ridge with brown-red/reddish tints. Eyes and ocelli silver. Fore and middle legs yellow although femora and tibiae with a narrow dorsal brown strip from top to bottom, middle coxae proximally with a brown spot, brown claws, and tarsomeres with brown tints. Hind leg yellow except black coxae although distally with brown-red/reddish tints, femora apically with a small brown spot and with a narrow dorsal brown strip from top to bottom, tibiae and tarsomeres brown. Petiole on T1 black and sublateral areas yellow-brown; T2 with median area black, adjacent area brown, adjacent area very narrow with a silhouette well-defined, and lateral ends yellow-brown; T3 and beyond completely brown; distally each tergum with a narrow whitish translucent band. In lateral view, T1–2 yellow; T3–4 yellow, but dorsally brown; T5 and beyond brown. S1–2 yellow; S3 and beyond brown.

#### Description.

**Head** (Fig. 133A, C, E, F). Head triangular with pubescence long and dense. Proximal first antennal flagellomere longer than wide (0.24:0.08), distal antennal flagellomere longer than penultimate (0.14:0.06, 0.09:0.06); antennal scrobes-frons shallow. Face flat or nearly so, punctations barely noticeable, interspaces smooth and longitudinal median carina present. Frons smooth. Temple wide, punctations barely noticeable and interspaces clearly smooth. Inner margin of eyes diverging slightly at antennal sockets; in lateral view, eye anteriorly convex and posteriorly straight. POL shorter than OOL (0.10, 0.13). Malar suture present. Median area between lateral ocelli slightly depressed. Vertex laterally rounded and dorsally wide.

**Mesosoma** (Fig. 133A, G, H, J). Mesosoma dorsoventrally convex. Distal 1/3 of mesoscutum with lateral margin slightly dented, punctation distinct throughout, interspaces smooth. Scutellum triangular, apex sloped and fused with BS, but not in the same plane, scutellar punctation scattered throughout, in profile scutellum flat and on same plane as mesoscutum, phragma of the scutellum completely concealed; BS mostly overlapping the MPM; ATS demilune entirely covered by parallel carinae; dorsal ATS groove with carinae only proximally. Transscutal articulation with small and heterogeneous foveae, area just behind transscutal articulation nearly at the same level as mesoscutum (flat) and with same kind of sculpture as mesoscutum. Metanotum with BM convex; MPM semicircular and bisected by a median longitudinal carina; AFM without setiferous lobes and not as well delineated as PFM; PFM thick, smooth and with lateral ends rounded; ATM proximally with a groove with some sculpturing and distally smooth. Propodeum without median longitudinal carina, proximal half curved with medium-sized sculpture, distal half slightly rugose; distal edge of propodeum with a flange at each side and short stubs; propodeal spiracle distally framed by faintly concave/wavy carina; nucha surrounded by long radiating carinae. Pronotum with a distinct dorsal furrow, dorsally with a well-defined smooth band; central area of pronotum and dorsal furrow smooth, but ventral furrow with short parallel carinae. Propleuron with fine punctations throughout and dorsally without a carina. Metasternum convex. Contour of mesopleuron convex; precoxal groove indistinct, smooth and shiny; epicnemial ridge widen.

**Legs.** Ventral margin of fore telotarsus entire without seta, fore telotarsus almost same width throughout and longer than fourth tarsomere (0.12, 0.08). Hind coxa finely punctate throughout, and dorsal outer depression absent. Inner spur of hind tibia longer than outer spur (0.25, 0.22), entire surface of hind tibia with dense strong spines clearly differentiated by color and length. Hind telotarsus as equal in length as fourth tarsomere (0.15, 0.15).

**Wings** (Fig. 134L, M). Fore wing with r vein slightly curved; 2RS vein straight; r and 2RS veins forming a weak, even curve at their junction and outer side of junction forming a distinct stub; 2M vein straight; distally fore wing [where spectral veins are] with microtrichiae more densely concentrated than the rest of the wing; anal cell 1/3 proximally lacking microtrichiae; subbasal cell with microtrichiae virtually throughout; veins 2CUa and 2CUb completely spectral; vein 2 cu-a present as spectral vein, sometimes difficult to see; vein 2-1A tubular throughout; tubular vein 1 cu-a straight and complete, but junction with 1-1A vein spectral. Vannal lobe in hind wing very narrow, subdistally and subproximally straightened, and setae absent proximally, but scattered distally.

**Metasoma** (Fig. 133A, I, K, L). Metasoma laterally compressed. Petiole on T1 with a mix of fine rugae and punctate sculpture over most of the surface, virtually parallel-sided over most of length, but barely narrowing over distal 1/3, apex truncate (length 0.41, maximum width 0.24, minimum width 0.13), and with scattered pubescence on distal half. Lateral grooves delimiting the median area on T2 clearly defined and reaching the distal edge of T2 (length median area 0.18, length T2 0.18), edges of median area polished and lateral groove deep, median area broader than long (length 0.18, maximum width 0.28, minimum width 0.12); T2 with scattered pubescence throughout. T3 longer than T2 (0.24, 0.18) and with scattered pubescence throughout. Pubescence on hypopygium dense.

**Cocoon** (Fig. 133B). White oval cocoon with messy/disordered/fluffy silk fibers, although sometimes evenly smooth.

#### Comments.

The median area on T2 with noticeable rugae throughout; the pubescence are all over the entire surface of T2; the hind telotarsus and fourth tarsomere are missing in holotype. The females have long ovipositor as *Sathon*.

#### Male

(Fig. 134A–M). Similar in coloration to female.

#### Etymology.

John O. Stireman III’s research is focused on tritrophic interactions, speciation, adaptive radiation, insect biodiversity, insect community structure, biology/systematics of Tachinidae (Diptera), plant-insect interactions, parasitoid biology and behavior. Currently, he is on the faculty at Wright State University, Dayton, OH, USA.

#### Distribution.

Parasitized caterpillars were collected in Ecuador, Napo, Yanayacu Biological Station (Yanayacu Road, Papallacta, and Río Chalpi Grande), during June, August, and October 2005 at 2,112 m, 2,768 m, and 2,876 m in cloud forest.

#### Biology.

The lifestyle of this parasitoid species is solitary.

#### Host.

Undetermined species of Pyralidae feeding on *Urtica* sp. (Urticaceae) and undetermined species of Apiaceae. Undetermined species of Lepidoptera feeding on undetermined species of Urticaceae. Caterpillars were collected in first, second and late instar or prepupal.

### Glyptapanteles josesimbanai

Taxon classificationAnimaliaHymenopteraBraconidae

Arias-Penna, sp. nov.

http://zoobank.org/03A73E6A-0F42-4666-A5B5-AE6214677103

Fig. 135

#### Male.

Body length 3.18, antenna length 4.14 mm, fore wing length 3.78 mm.

#### Type material.

**Holotype**: ECUADOR • 1♀; EC-579, YY-A146; Napo, Yanayacu Biological Station, Yanayacu Road; cloud forest; 2,100 m; -0.566667, -77.866667; 18.vii.2004; Lee Dyer leg.; caterpillar collected in third instar; (PUCE).

#### Diagnosis.

**Figure 135.**
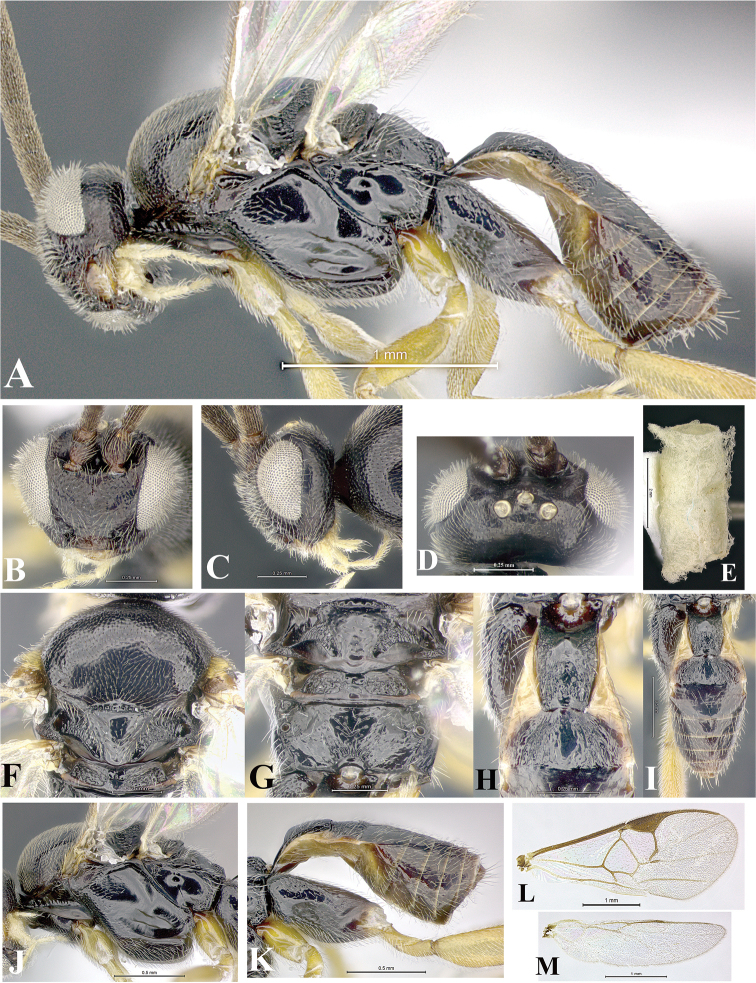
*Glyptapantelesjosesimbanai* sp. nov. male EC-579 YY-A146 **A** Habitus **B, D** Head **B** Frontal view **D** Dorsal view **C** Head, pronotum, propleuron, lateral view **E** Cocoon **F** Mesonotum, dorsal view **G** Scutellum, metanotum, propodeum, dorsal view **H**T1–2, dorsal view **I, K** Metasoma **I** Dorsal view **K** Lateral view **J** Mesosoma, lateral view **L, M** Wings **L** Fore **M** Hind.

Petiole on T1 virtually parallel-sided, but narrowing over distal 1/3 (Fig. 135H, I), distal edge on T2 straight (Fig. 135H, I), lateral grooves delimiting the median area on T2 clearly defined and reaching the distal edge of T2 (Fig. 135H), edges of median area on T2 obscured by strong longitudinal stripes (Fig. 135H, I), T3 longer than T2 (Fig. 135I), distal antennal flagellomere longer than penultimate, mesoscutum punctation distinct throughout (Fig. 135F), in lateral view, metasoma curved (Fig. 135A, K), dorsal outer depression on hind coxa present (Fig. 135A, K), and fore wing with r vein curved, outer side of junction of r and 2RS veins forming a stub (Fig. 135L).

#### Coloration

(Fig. 135A–M). General body coloration polished black except pedicel brown-red/reddish; scape brown-red/reddish, but distally with a brown ring; all antennal flagellomeres dark brown on both sides; clypeus brown/reddish; mandibles proximally reddish and distally yellow-brown; contours of labrum yellow-brown, but remaining area brown-red/reddish; glossa, maxillary and labial palps, and tegulae yellow; lunules and BS with a distal narrow brown-red/reddish as well as PFM and BM; both dorsal and ventral furrows of pronotum, epicnemial ridge, and ventral edge of mesopleuron with brown-red/reddish tints. Eyes and ocelli silver. Fore and middle legs yellow except brown claws, and middle telotarsus light brown; hind legs yellow except black coxae with apex yellow, femora with a small brown area in the apex, tibiae with 1/3 distal and proximally with a small brown band, and tarsomeres brown. Petiole on T1 black and sublateral areas yellow; T2 with median and adjacent areas brown, adjacent area with contours well-defined, and lateral ends yellow-brown; T3 completely brown except two notches each one at the proximal corners of T3; T4 and beyond brown; distally each tergum with a narrow whitish translucent band. In lateral view, T1–2 yellow; T3 yellow, dorsally brown-reddish; T4 and beyond brown. S1–4 yellow; penultimate sternum and hypopygium brown-reddish.

#### Description.

**Head** (Fig. 135A–D). Head rounded with pubescence long and dense. Proximal three antennal flagellomeres longer than wide (0.29:0.12, 0.29:0.12, 0.29:0.12), distal antennal flagellomere longer than penultimate (0.20:0.07, 0.15:0.07), antenna longer than body (4.14, 3.18); antennal scrobes-frons sloped and forming a shelf. Face distal half dented only laterally, punctations barely noticeable, interspaces smooth and longitudinal median carina present. Frons smooth. Temple wide, punctations barely noticeable and interspaces clearly smooth. Inner margin of eyes diverging slightly at antennal sockets; in lateral view, eye anteriorly convex and posteriorly straight. POL shorter than OOL (0.11, 0.16). Malar suture present. Median area between lateral ocelli without depression. Vertex laterally rounded and dorsally wide.

**Mesosoma** (Fig. 135A, F, G, J). Mesosoma dorsoventrally convex. Distal 1/3 of mesoscutum with lateral margin slightly dented, punctation distinct throughout, interspaces smooth. Scutellum triangular, apex sloped and fused with BS, but not in the same plane, scutellar punctation scattered throughout, in profile scutellum flat and on same plane as mesoscutum, phragma of the scutellum partially exposed; BS only very partially overlapping the MPM; ATS demilune with complete undulate/reticulate carinae; dorsal ATS groove with semicircular/parallel carinae. Transscutal articulation with small and heterogeneous foveae, area just behind transscutal articulation nearly at the same level as mesoscutum (flat) and with same kind of sculpture as mesoscutum. Metanotum with BM upward; MPM oval/circular with a short proximal carina; AFM with a small lobe and not as well delineated as PFM; PFM thick, smooth and with lateral ends rounded; ATM proximally sculptured and distally without a well delimited smooth area. Propodeum with a median longitudinal dent, but no trace of median longitudinal carina, propodeum relatively polished although with some medium-sized sculpture, proximal half weakly curved; distal edge of propodeum with a flange at each side and without stubs; propodeal spiracle distally framed by a short concave carina; nucha surrounded by very short radiating carinae. Pronotum with a distinct dorsal furrow, dorsally with a well-defined smooth band; central area of pronotum and dorsal furrow smooth, but ventral furrow with short parallel carinae. Propleuron finely sculptured only ventrally and dorsally without a carina. Metasternum convex. Contour of mesopleuron convex; precoxal groove smooth, shiny and shallow, but visible; epicnemial ridge convex, teardrop-shaped.

**Legs.** Ventral margin of fore telotarsus entire without seta, fore telotarsus almost same width throughout and longer than fourth tarsomere (0.15, 0.12). Hind coxa with dorsal half sparsely punctate, ventral half densely punctate, and dorsal outer depression present. Inner spur of hind tibia longer than outer spur (0.33, 0.26), entire surface of hind tibia with dense strong spines clearly differentiated by color and length. Hind telotarsus longer than fourth tarsomere (0.18, 0.15).

**Wings** (Fig. 135L, M). Fore wing with r vein slightly curved; 2RS vein slightly convex to convex; r and 2RS veins forming a weak, even curve at their junction and outer side of junction forming a distinct stub; 2M vein slightly curved/swollen; distally fore wing [where spectral veins are] with microtrichiae more densely concentrated than the rest of the wing; anal cell 1/3 proximally lacking microtrichiae; subbasal cell with a small smooth area; vein 2CUa absent and vein 2CUb spectral; vein 2 cu-a absent; vein 2-1A proximally tubular and distally spectral, although sometimes difficult to see; tubular vein 1 cu-a curved and complete, but junction with 1-1A vein spectral. Hind wing with vannal lobe very narrow, subdistally and subproximally straightened, and setae evenly scattered in the margin.

**Metasoma** (Fig. 135A, H, I, K). Metasoma curved. Petiole on T1 with a mix of fine rugae and punctate sculpture over most of the surface, virtually parallel-sided over most of length, but barely narrowing over distal 1/3, apex truncate (length 0.51, maximum width 0.22, minimum width 0.18) and with scattered pubescence on distal half. Lateral grooves delimiting the median area on T2 clearly defined and reaching the distal edge of T2 (length median area 0.24, length T2 0.24), edges of median area obscured by strong longitudinal stripes, median area broader than long (length 0.24, maximum width 0.40, minimum width 0.15); T2 with scattered pubescence throughout. T3 longer than T2 (0.30, 0.24) and with scattered pubescence throughout.

**Cocoon** (Fig. 135E). Beige oval cocoon with messy/disordered/fluffy silk fibers; body of cocoon with disorganized and tangled silk.

#### Comments.

The petiole on T1 medially with lateral margins slightly curved (convex, Fig. 135H); the propleuron looks slim (Fig. 135A).

#### Female.

Unknown.

#### Etymology.

José Arturo Simbaña is one of the gusaneros at Yanayacu Biological Station and thus responsible for collecting much of the Ecuadorean material for this study.

#### Distribution.

Parasitized caterpillar was collected in Ecuador, Napo, Yanayacu Biological Station (Yanayacu Road), during July 2004 at 2,100 m in cloud forest.

#### Biology.

The lifestyle of this parasitoid species is solitary.

#### Host.

Undetermined species of Lepidoptera feeding on *Rubus* sp. (Rosaceae). Caterpillar was collected in third instar.

### Glyptapanteles juanvargasi

Taxon classificationAnimaliaHymenopteraBraconidae

Arias-Penna, sp. nov.

http://zoobank.org/22F5EEAE-8170-49F8-8291-8A5DE582EDF9

Fig. 136

#### Male.

Body length 2.73 mm, antenna length 4.06 mm, fore wing length 3.63 mm.

#### Type material.

**Holotype**: ECUADOR • 1♀; EC-30777, YY-A173; Napo, Yanayacu Biological Station, Río Pumayacu, Quebrada Pumayacu; cloud forest; 2,000 m; -0.604722, -77.880833; 26.iv.2008; CAPEA leg.; caterpillar collected in third instar; cocoon formed on 30.iv.2008; adult parasitoid emerged on 14.vi.2008; (PUCE).

#### Diagnosis.

**Figure 136.**
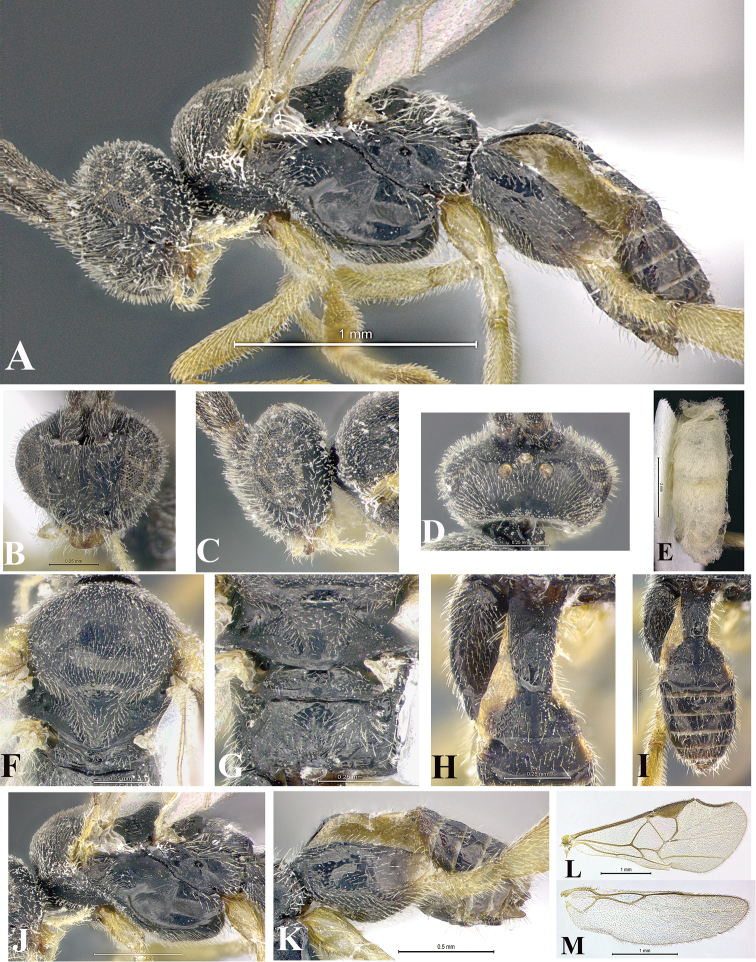
*Glyptapantelesjuanvargasi* sp. nov. male EC-30777 YY-A173 **A** Habitus **B, D** Head **B** Frontal view **D** Dorsal view **C** Head, pronotum, propleuron, lateral view **E** Cocoon **F** Mesonotum, dorsal view **G** Scutellum, metanotum, propodeum, dorsal view **H**T1–3, dorsal view **I, K** Metasoma **I** Dorsal view **K** Lateral view **J** Mesosoma, lateral view **L, M** Wings **L** Fore **M** Hind.

Edges of median area on T2 obscured by weak longitudinal stripes (Fig. 136H, I), scutellar punctation scattered throughout (Fig. 136F, G), in lateral view, metasoma curved (Fig. 136A, K), inner margin of eyes straight throughout (Fig. 136B), oetiole on T1 with rugae all over except antero-median depression (Fig. 136H, I), propodeum with a median longitudinal dent (Fig. 136G), lateral grooves delimiting the median area on T2 clearly defined and reaching the distal edge of T2 (Fig. 136H, I), and fore wing with outer side of junction of r and 2RS veins not forming a stub (Fig. 136L).

#### Coloration

(Fig. 136A–M). General body coloration polished black except scape, pedicel, and all antennal flagellomeres (on both sides) dark brown; labrum and mandibles yellow-brown; glossa, maxillary and labial palps, and tegulae yellow; apex and dorsal furrow of pronotum with brown-red/reddish tints. Eyes black and ocelli reddish (in preserved specimen). Fore and middle legs yellow except brown claws; hind legs yellow except black coxae with apex yellow, femora with a small brown area in the apex, tibia with 1/3 distal and proximally with a small band brown, and tarsomeres brown. Petiole on T1 black and sublateral areas yellow; T2 with median and adjacent areas brown, and lateral ends yellow-brown; T3 completely brown except a small area in the proximal half of lateral ends; T4 and beyond brown; distally each tergum with a narrow yellowish translucent band. In lateral view, T1–2 yellow; T3 yellow, dorsally with an extended brown area; T4 and beyond brown. S1–4 yellow and remaining sterna brown.

#### Description.

**Head** (Fig. 136A–D). Head rhomboid with pubescence long and dense. Proximal three antennal flagellomeres longer than wide (0.31:0.09, 0.31:0.09, 0.31:0.09), distal antennal flagellomere longer than penultimate (0.20:0.06, 0.15:0.07), antenna longer than body (4.06, 2.73); antennal scrobes-frons sloped and forming a shelf. Face with depression only laterally, punctations barely noticeable, interspaces smooth and longitudinal median carina present. Frons smooth. Temple wide, punctations barely noticeable and interspaces clearly smooth. Inner margin of eyes straight throughout; in lateral view, eye anteriorly convex and posteriorly straight. POL subequal in length with OOL (0.11, 0.12). Malar suture absent or difficult to see. Median area between lateral ocelli without depression. Vertex laterally rounded and dorsally wide.

**Mesosoma** (Fig. 136A, F, G, J). Mesosoma dorsoventrally convex. Distal 1/3 of mesoscutum with lateral margin slightly dented, punctation distinct throughout, interspaces smooth. Scutellum triangular, apex sloped and fused with BS, but not in the same plane, scutellar punctation scattered throughout, in profile scutellum flat and on same plane as mesoscutum, phragma of the scutellum partially exposed; BS only very partially overlapping the MPM; ATS demilune with complete undulate/reticulate carinae; dorsal ATS groove with carinae only proximally. Transscutal articulation with small and heterogeneous foveae, area just behind transscutal articulation nearly at the same level as mesoscutum (flat) and with same kind of sculpture as mesoscutum. Metanotum with BM convex, MPM oval/circular with a short proximal carina; AFM with a small lobe and not as well delineated as PFM; PFM thick, smooth and with lateral ends rounded; ATM proximally with sculpture distally without a well delimited smooth area. Propodeum with medium-sized sculpture and with a median longitudinal dent, but no trace of median longitudinal carina, proximal half weakly curved; distal edge of propodeum with a flange at each side and without stubs; propodeal spiracle distally framed by a short concave carina; nucha surrounded by long radiating carinae. Pronotum with a distinct dorsal furrow, dorsally with a well-defined smooth band; central area of pronotum and both dorsal and ventral furrows smooth. Propleuron with fine punctations throughout and dorsally without a carina. Metasternum convex. Contour of mesopleuron convex; precoxal groove deep, smooth and shiny; epicnemial ridge convex, teardrop-shaped.

**Legs.** Ventral margin of fore telotarsus entire without seta, fore telotarsus proximally narrow and distally wide and longer than fourth tarsomere (0.16, 0.07). Hind coxa with dorsal half sparsely punctate, ventral half densely punctate, and dorsal outer depression present. Inner spur of hind tibia longer than outer spur (0.30, 0.23), entire surface of hind tibia with dense strong spines clearly differentiated by color and length. Hind telotarsus as equal in length as fourth tarsomere (0.15, 0.15).

**Wings** (Fig. 136L, M). Fore wing with r vein curved; 2RS vein straight; r and 2RS veins forming a weak, even curve at their junction and outer side of junction not forming a stub; 2M vein straight; distally fore wing [where spectral veins are] with microtrichiae more densely concentrated than the rest of the wing; anal cell 1/3 proximally lacking microtrichiae; subbasal cell with microtrichiae virtually throughout; veins 2CUa and 2CUb completely spectral; vein 2 cu-a present as spectral vein, sometimes difficult to see; vein 2-1A proximally tubular and distally spectral, although sometimes difficult to see; tubular vein 1 cu-a straight and complete, but junction with 1-1A vein spectral. Hind wing with vannal lobe very narrow, subdistally and subproximally straightened, and setae evenly scattered in the margin.

**Metasoma** (Fig. 136A, H, I, K). Metasoma curved. Petiole on T1 with rugae all over except antero-median depression, virtually parallel-sided over most of length, but barely narrowing over distal 1/3, apex truncate (length 0.47, maximum width 0.19, minimum width 0.14), and with scattered pubescence on distal half. Lateral grooves delimiting the median area on T2 clearly defined and reaching the distal edge of T2 (length median area 0.18, length T2 0.18), edges of median area obscured by weak longitudinal stripes, median area broader than long (length 0.18, maximum width 0.29, minimum width 0.13); T2 scarce pubescence throughout. T3 longer than T2 (0.21, 0.18) and with scattered pubescence throughout.

**Cocoon** (Figs 4B, 136E). Beige oval cocoon with messy/disordered/fluffy silk fibers and body of cocoon with disorganized and tangled silk.

#### Comments.

Body with dense pubescence.

#### Female.

Unknown.

#### Etymology.

Juan Manuel Vargas Rojas is a Colombian entomologist. His research is focused mainly on Hymenoptera (Bethylidae) and Hemiptera (Cicadellidae) and he works at the Instituto Colombiano Agropecuario (ICA), Bogotá, Colombia.

#### Distribution.

Parasitized caterpillar was collected in Ecuador, Napo, Yanayacu Biological Station (Río Pumayacu and Quebrada Pumayacu), during April 2008 at 2,000 m in cloud forest.

#### Biology.

The lifestyle of this parasitoid species is solitary.

#### Host.

Undetermined species of Pyralidae feeding on *Boehmeria* sp. (Urticaceae). Caterpillar was collected in third instar.

### Glyptapanteles jumamuturii

Taxon classificationAnimaliaHymenopteraBraconidae

Arias-Penna, sp. nov.

http://zoobank.org/E9EC311A-8CE1-4502-AC83-6A427B215ED4

Fig. 137

#### Female.

Body length 2.73 mm, antenna length 3.13 mm, fore wing length 3.68 mm.

#### Type material.

**Holotype**: ECUADOR • 1♀; EC-28553, YY-A071; Napo, Yanayacu Biological Station, Yanayacu Road; cloud forest; 2,100 m; -0.6, -77.866667; 26.xi.2007; Rafael Granizo leg.; caterpillar collected in fourth instar; cocoons formed on 26.xi.2007; adult parasitoids emerged on 11.xii.2007; (PUCE). **Paratypes.** • 24 (5♀, 6♂) (10♀, 3♂); EC-28553, YY-A071; same data as for holotype; (PUCE).

#### Diagnosis.

**Figure 137.**
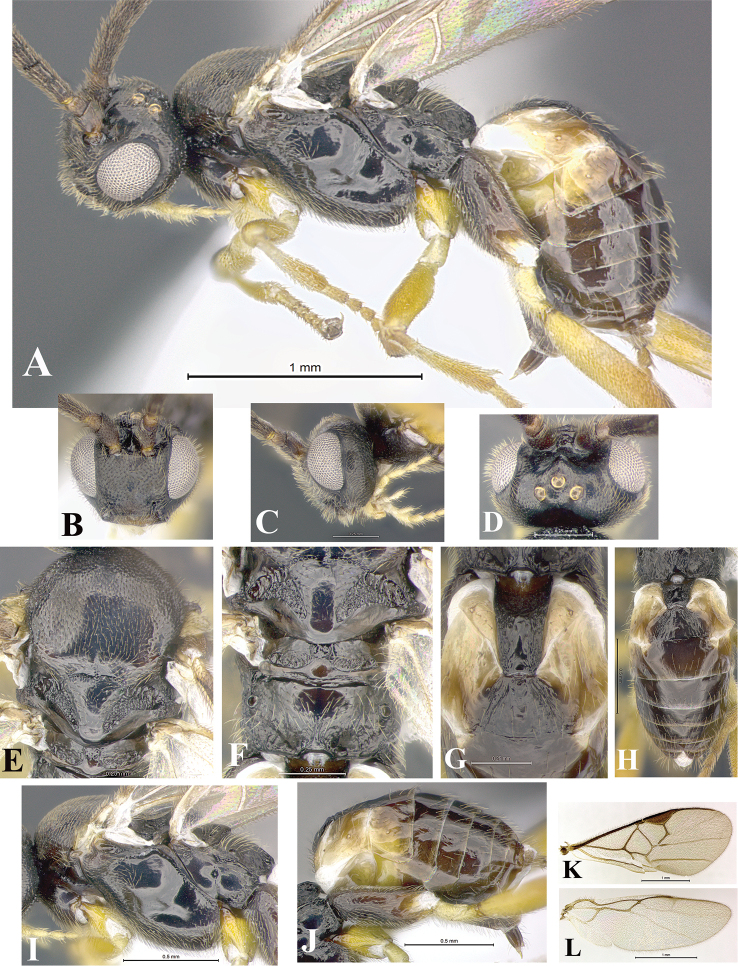
*Glyptapantelesjumamuturii* sp. nov. female EC-28553 YY-A071 **A** Habitus **B–D** Head **B** Frontal view **C** Lateral view **D** Dorsal view **E** Mesonotum, dorsal view **F** Scutellum, metanotum, propodeum, dorsal view **G**T1–2, dorsal view **H, J** Metasoma **H** Dorsal view **J** Lateral view **I** Mesosoma, lateral view **K, L** Wings **K** Fore **L** Hind.

Petiole on T1 with a mix of sculptures: finely rugulate and punctate (Fig. 137G, H), lateral grooves delimiting the median area on T2 distally losing definition on T2 (Fig. 137G, H), and fore wing with r vein straight, outer side of junction of r and 2RS veins forming a stub (Fig. 137K).

#### Coloration

(Fig. 137A–L). General body coloration satin black except scape and all antennal flagellomeres dark brown on both sides; pedicel brown-red/reddish; labrum and mandibles yellow-brown; glossa, maxillary and labial palps, and tegulae yellow; propleuron, dorsal and ventral furrows of pronotum, epicnemial ridge, distal corner of mesoscutum that expand forward in one longitudinal band at each side and goes until distal 1/3 of mesoscutum, lunules, BS, AFM, PFM, and medially propodeum with brown-red/reddish tints. Eyes silver and ocelli reddish (in preserved specimen). Fore and middle legs yellow except brown claws and tarsomeres with brown tints; hind legs yellow except black coxae with apex yellow/yellow-brown, femora with a small brown area in the apex, distal half of tibiae brown with a small distal brown band, and tarsomeres brown. Petiole on T1 proximally brown-red/reddish, distally black, contours darkened and sublateral areas yellow; T2 with median and adjacent areas brown, and lateral ends yellow-brown; T3 completely brown except a small area in the proximal half of lateral corners; T4 and beyond brown; distally each tergum with a narrow whitish translucent band. In lateral view, T1–3 dorsally yellow-brown, ventrally yellow; T4 and beyond brown. S1–3 yellow; S4 and beyond brown.

#### Description.

**Head** (Fig. 137A–D). Head rhomboid with pubescence long and dense. Proximal three antennal flagellomeres longer than wide (0.23:0.08, 0.21:0.08, 0.21:0.08), distal antennal flagellomere longer than penultimate (0.13:0.07, 0.11:0.07), antenna longer than body (3.13, 2.73); antennal scrobes-frons sloped and forming a shelf. Face flat or nearly so, with dense fine punctation, interspaces smooth and longitudinal median carina present. Frons smooth. Temple wide, punctate and interspaces clearly smooth. Inner margin of eyes diverging slightly at antennal sockets; in lateral view, eye anteriorly convex and posteriorly straight. POL shorter than OOL (0.10, 0.15). Malar suture present. Median area between lateral ocelli slightly depressed. Vertex laterally rounded and dorsally wide.

**Mesosoma** (Fig. 137A, E, F, I). Mesosoma dorsoventrally convex. Mesoscutum proximally convex and distally flat, punctation distinct throughout, interspaces smooth. Scutellum triangular, apex sloped and fused with BS, scutellar punctation scattered throughout, in profile scutellum flat and on same plane as mesoscutum, phragma of the scutellum partially exposed; BS only very partially overlapping the MPM; ATS demilune with complete undulate/reticulate carinae, dorsal ATS groove with semicircular/parallel carinae. Transscutal articulation with small and heterogeneous foveae, area just behind transscutal articulation sloped and with same kind of sculpture as mesoscutum. Metanotum with BM wider than PFM (clearly differentiated); MPM semicircular without median longitudinal carina; AFM with a small lobe and not as well delineated as PFM; PFM thick, smooth and with lateral ends rounded; ATM proximally with semircular/undulate carina and distally smooth. Propodeum relatively polished and without median longitudinal carina, proximal half weakly curved; distal edge of propodeum with a flange at each side and without stubs; propodeal spiracle without distal carina; nucha surrounded by long radiating carinae. Pronotum with a distinct dorsal furrow, dorsally with a well-defined smooth band; central area of pronotum smoot, but both dorsal and ventral furrows with short parallel carinae. Propleuron finely sculptured only ventrally and dorsally without a carina. Metasternum convex. Contour of mesopleuron convex; precoxal groove smooth, shiny and shallow, but visible; epicnemial ridge convex, teardrop-shaped.

**Legs.** Ventral margin of fore telotarsus entire without seta, fore telotarsus proximally narrow and distally wide, and longer than fourth tarsomere (0.12, 0.05). Hind coxa finely punctate throughout, and dorsal outer depression present. Inner spur of hind tibia much longer than outer spur (0.21, 0.18), entire surface of hind tibia with dense strong spines clearly differentiated by color and length. Hind telotarsus longer than fourth tarsomere (0.15, 0.12).

**Wings** (Fig. 137K, L). Fore wing with r vein straight; 2RS vein slightly convex to convex; r and 2RS veins forming a weak, even curve at their junction and outer side of junction forming a distinct stub; 2M vein slightly curved/swollen; distally fore wing [where spectral veins are] with microtrichiae more densely concentrated than the rest of the wing; anal cell 1/3 proximally lacking microtrichiae; subbasal cell with microtrichiae virtually throughout; veins 2CUa and 2CUb completely spectral; vein 2 cu-a present as spectral vein, sometimes difficult to see; vein 2-1A proximally tubular and distally spectral, although sometimes difficult to see; vein1 cu-a straight. Hind wing with vannal lobe very narrow, subdistally and subproximally straightened, and setae present proximally, but absent distally.

**Metasoma** (Fig. 137A, G, H, J). Metasoma curved. Petiole on T1 with a mix of sculptures finely rugulate and punctate over most of the surface, virtually parallel-sided over most of length, but barely narrowing over distal 1/3, apex truncate (length 0.40, maximum width 0.22, minimum width 0.12), and with scattered pubescence on distal half. Lateral grooves delimiting the median area on T2 clearly defined and reaching the distal edge of T2 (length median area 0.22, length T2 0.22), edges of median area obscured by weak longitudinal stripes, median area broader than long (length 0.22, maximum width 0.32, minimum width 0.12); T2 with scarce pubescence throughout. T3 longer than T2 (0.26, 0.22) and with scattered pubescence throughout. Pubescence on hypopygium dense.

**Cocoons.** Unknown.

#### Comments.

The body is distinctively curved.

#### Male.

Similar in coloration to female.

#### Etymology.

Ephantus Juma Muturi is a Kenyan-born entomologist. His research is focused on vector biology, primarily mosquito-microbe interactions and the development of ecofriendly strategies for mosquito control. Currently, he is a research entomologist at the Crop Bioprotection Research Unit, National Center for Agricultural Utilization Research, United States Department of Agriculture, Agricultural Research Service, Peoria, IL, USA.

#### Distribution.

Parasitized caterpillar was collected in Ecuador, Napo, Yanayacu Biological Station (Yanayacu Road), during November 2007 at 2,100 m in cloud forest.

#### Biology.

The lifestyle of this parasitoid species is gregarious.

#### Host.

Undetermined species of Pyralidae feeding on *Oreopanax* sp. (Araliaceae). Caterpillar was collected in fourth instar.

### Glyptapanteles keithwillmotti

Taxon classificationAnimaliaHymenopteraBraconidae

Arias-Penna, sp. nov.

http://zoobank.org/D4DDEAC3-1E1A-4699-A6E6-E150EF2BF5C7

Fig. 138

#### Female.

Body length 2.73 mm, antenna length 3.23 mm, fore wing length 3.43 mm.

#### Type material.

**Holotype**: ECUADOR • 1♀; EC-36095, YY-A107; Napo, Yanayacu Biological Station, Yanayacu Road; cloud forest; 2,100 m; -0.566667, -77.866667; 20.xi.2008; CAPEA leg.; caterpillar collected in third instar; cocoons formed on 05.xii.2008; adult parasitoids emerged on 10.i.2009; (PUCE). **Paratypes.** • 5 (2♀, 1♂) (2♀, 0♂); EC-36095, YY-A107; same data as for holotype; (PUCE).

#### Other material.

**Reared material.** ECUADOR: *Napo*, *Yanayacu Biological Station*, *Río Aliso*, *Isla del Río Aliso*: • 17 (5♀, 0♂) (12♀, 0♂); EC-29410, YY-A109; cloud forest; 2,100 m; -0.633333, -77.9; 23.i.2008; CAPEA leg.; caterpillar collected in third instar; cocoons formed on 19.ii.2008; adult parasitoids emerged on 10.iii.2008.

*Napo*, *Yanayacu Biological Station*, *Sendero Macuculoma*, *Plot 443*: • 15 (6♀, 6♂) (1♀, 2♂); EC-42168B, YY-A006; cloud forest; 2,014 m; -0.604806, -77.886417; 11.ix.2009; Luis Salagaje leg.; caterpillar collected in third instar; cocoons formed on 08.x.2009; adult parasitoids emerged on17.x.2009.

#### Diagnosis.

**Figure 138.**
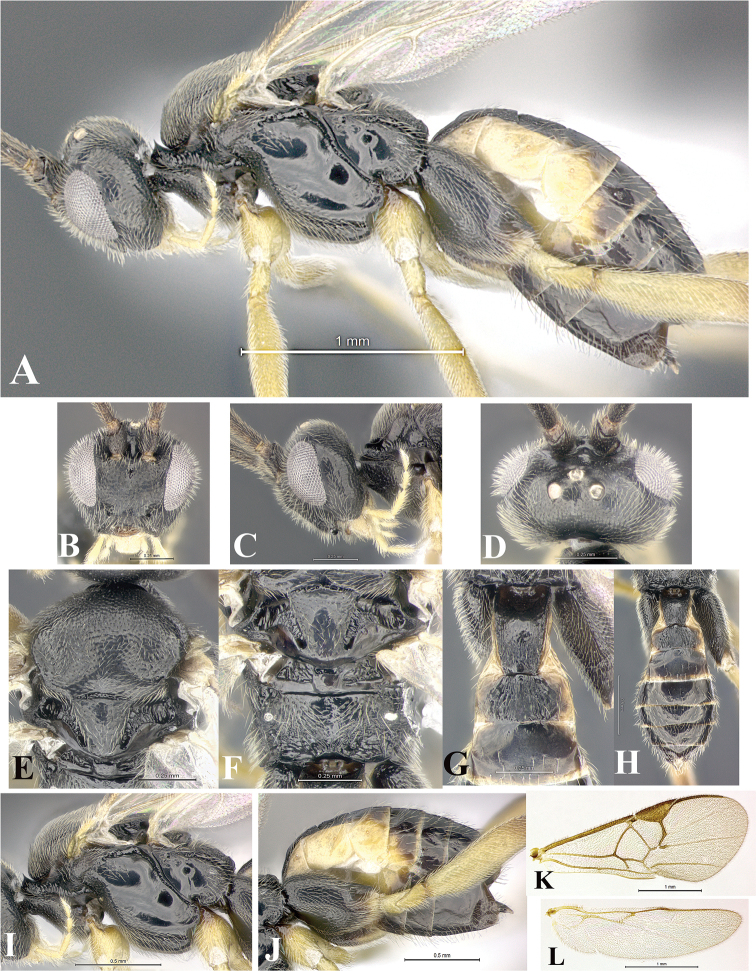
*Glyptapanteleskeithwillmotti* sp. nov. female EC-29410 YY-A109, EC-36095 YY-A107 **A** Habitus **B–D** Head **B** Frontal view **C** Lateral view **D** Dorsal view **E** Mesonotum, dorsal view **F** Scutellum, metanotum, propodeum, dorsal view **G**T1–2, dorsal view **H, J** Metasoma **H** Dorsal view **J** Lateral view **I** Mesosoma, lateral view **K, L** Wings **K** Fore **L** Hind.

Petiole on T1 with rugae (Fig. 138G, H), lateral grooves delimiting the median area on T2 distally losing definition on T2 (Fig. 138G, H), and fore wing with r vein straight, outer side of junction of r and 2RS veins forming a stub (Fig. 138K).

#### Coloration

(Fig. 138A–L). General body coloration polished black except distal half of scape, labrum, mandibles, lunules, BS, PFM and BS with brown-red/reddish tints; pedicel dark brown; first four-five proximal antennal flagellomeres dorsally lighter (light brown) than ventrally (dark brown), remaining flagellomeres dark brown on both sides; glossa, maxillary and labial palps, and tegulae yellow. Eyes and ocelli silver. Fore and middle legs yellow except brown claws; hind legs yellow except black coxae with apex yellow, femora with brown apex, tibiae brown although both proximal and distal apexes are darkered, and tarsomeres brown. Petiole on T1 black and sublateral areas yellow; T2 with median and adjacent areas brown, and lateral ends yellow-brown; T3 completely brown, but distally with a yellow-brown band; T4 and beyond brown; distally each tergum with a narrow yellowish translucent band. In lateral view, T1–2 yellow; T3–4 yellow, but dorsally yellow-brown, the extent of that dark area increasing from proximal to distal; T5 and beyond brown. S1–2 yellow; S3 yellow, but medially brown; S4 and beyond brown.

#### Description.

**Head** (Fig. 138A–D). Head elongate with pubescence long and dense. Proximal three antennal flagellomeres longer than wide (0.24:0.07, 0.26:0.07, 0.24:0.07), distal antennal flagellomere longer than penultimate (0.13:0.05, 0.10:0.05), antenna longer than body (3.23, 2.73); antennal scrobes-frons sloped and forming a shelf. Face flat or nearly so, punctations barely noticeable, interspaces smooth, and longitudinal median carina present. Frons smooth. Temple wide, punctations barely noticeable, and interspaces clearly smooth. Inner margin of eyes diverging slightly at antennal sockets; in lateral view, eye anteriorly convex and posteriorly straight. POL shorter than OOL (0.09, 0.13). Malar suture absent or difficult to see. Median area between lateral ocelli without depression. Vertex laterally rounded and dorsally wide.

**Mesosoma** (Fig. 138A, E, F, I). Mesosoma dorsoventrally convex. Mesoscutum proximally convex and distally flat, punctation distinct throughout, interspaces smooth. Scutellum triangular, apex sloped and fused with BS, but not in the same plane, scutellar punctation scattered throughout, in profile scutellum flat and on same plane as mesoscutum, phragma of the scutellum completely concealed; BS mostly overlapping the MPM, rarely overlapping mostly the MPM; ATS demilune with a little and complete parallel carinae; dorsal ATS groove with semicircular/parallel carinae. Transscutal articulation with small and heterogeneous foveae, area just behind transscutal articulation with same kind of sculpture as mesoscutum and with a sloped transverse strip. Metanotum with BM wider than PFM (clearly differentiated); MPM oval/circular with a short proximal carina; AFM without setiferous lobes and not as well delineated as PFM; PFM thick and smooth; ATM proximally with sculpture distally without a well delimited smooth area. Propodeum with a median longitudinal dent, but no trace of median longitudinal carina, proximal half weakly curved with medium-sized sculpture and distal half slightly rugose; distal edge of propodeum with a flange at each side and without stubs; propodeal spiracle distally framed by faintly concave/wavy carina; nucha surrounded by long radiating carinae. Pronotum with a distinct dorsal furrow, dorsally with a well-defined smooth band, central area of pronotum and dorsal furrow smooth, but ventral furrow with short parallel carinae. Propleuron with fine punctations throughout and dorsally without a carina. Metasternum convex. Contour of mesopleuron convex; precoxal groove deep, smooth and shiny; epicnemial ridge convex, teardrop-shaped.

**Legs.** Ventral margin of fore telotarsus slightly excavated and with a tiny curved seta, fore telotarsus almost same width throughout and longer than fourth tarsomere (0.12, 0.09). Hind coxa finely punctate throughout, and dorsal outer depression absent. Inner spur of hind tibia longer than outer spur (0.25, 0.21), entire surface of hind tibia with dense strong spines clearly differentiated by color and length. Hind telotarsus as equal in length as fourth tarsomere (0.15, 0.14).

**Wings** (Fig. 138K, L). Fore wing with r vein straight; 2RS vein straight; r and 2RS veins forming a weak, even curve at their junction and outer side of junction forming a slight stub; 2M vein slightly curved/swollen; distally fore wing [where spectral veins are] with microtrichiae more densely concentrated than the rest of the wing; anal cell 1/3 proximally lacking microtrichiae; subbasal cell with microtrichiae virtually throughout; veins 2CUa and 2CUb completely spectral; vein 2 cu-a present as spectral vein, sometimes difficult to see; vein 2-1A proximally tubular and distally spectral, although sometimes difficult to see; tubular vein 1 cu-a curved, incomplete/broken and not reaching the edge of 1-1A vein. Hind wing with vannal lobe narrow, subdistally and subproximally straightened, and setae evenly scattered in the margin.

**Metasoma** (Fig. 138A, G, H, J). Metasoma cylindrical. Petiole on T1 with rugae all over except antero-median depression, virtually parallel-sided over most of length, but barely narrowing over distal 1/3, apex truncate (length 0.40, maximum width 0.20, minimum width 0.12), and with scattered pubescence on distal half. Lateral grooves delimiting the median area on T2 clearly defined and reaching the distal edge of T2 (length median area 0.18, length T2 0.18), edges of median area obscured by strong longitudinal stripes, median area broader than long (length 0.18, maximum width 0.27, minimum width 0.12); T2 with scattered pubescence throughout. T3 longer than T2 (0.27, 0.18) and with scattered pubescence throughout. Pubescence on hypopygium dense.

**Cocoons.** Unknown.

#### Comments.

In some females, T3 with lateral ends lighter than remaining area; the middle coxae dorsally with a small brown spot. Laterally, the body is distinctively curved.

#### Male.

Similar in coloration to female, except that metasoma is more elongated and cylindrical.

#### Etymology.

Keith Willmott’s interests lie in studying butterfly diversity, understanding its spatial and temporal patterns, investigating the evolution and maintenance of diversity, and applying results to biodiversity conservation. He works at the Florida Museum of Natural History, University of Florida, Gainesville, FL, USA.

#### Distribution.

Parasitized caterpillars were collected in Ecuador, Napo, Yanayacu Biological Station (Sendero Macuculoma, Río Aliso, and Yanayacu Road), during January and November 2008 and September 2009 at 2,014 m and 2,100 m in cloud forest.

#### Biology.

The lifestyle of this parasitoid species is gregarious.

#### Host.

Undetermined species of Noctuidae feeding on *Dendrophorbiumlloense* (Asteraceae) and *Salviatortuosa* (Lamiaceae). Caterpillars were collected in third instar.

### Glyptapanteles kevinjohnsoni

Taxon classificationAnimaliaHymenopteraBraconidae

Arias-Penna, sp. nov.

http://zoobank.org/9C8EB398-4443-43F9-8067-CE088A4FFFBB

Fig. 139

#### Female.

Body length 2.83 mm, antenna length 3.28 mm, fore wing length 3.43 mm.

#### Type material.

**Holotype**: ECUADOR • 1♀; EC-38518, YY-A004; Napo, Yanayacu Biological Station, Yanayacu Road; cloud forest; 2,100 m; -0.566667, -77.866667; 30.iv.2009; CAPEA leg.; caterpillar collected in second instar; cocoons formed on 05.vi.2009; adult parasitoids emerged on 26.vi.2009; (PUCE). **Paratypes.** • 73 (9♀, 5♂) (59♀, 0♂); EC-38518, YY-A004; same data as for holotype; (PUCE).

#### Other material.

**Reared material.** ECUADOR: *Napo*, *Yanayacu Biological Station*, *Yanayacu Road*: • 83 (6♀, 6♂) (62♀, 9♂); EC-2807, YY-A083; cloud forest; 2,100 m; - 0.566667, -77.866667; 22.v.2005; CAPEA leg.; adult parasitoids emerged on 12.vii.2005.

#### Diagnosis.

**Figure 139.**
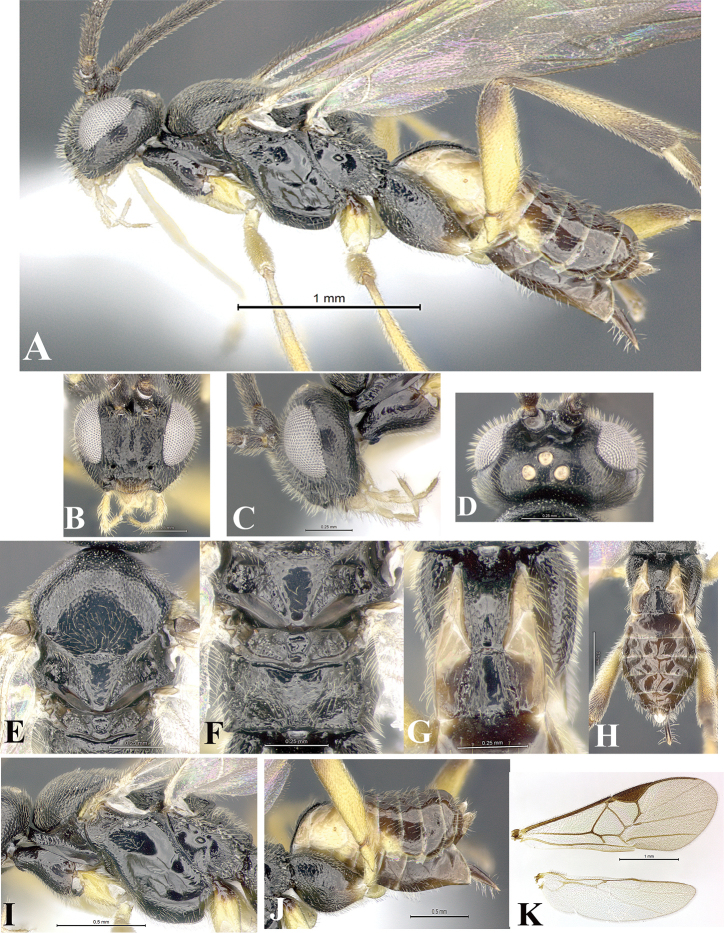
*Glyptapanteleskevinjohnsoni* sp. nov. female EC-38518 YY-A004 **A** Habitus **B, D** Head **B** Frontal view **D** Dorsal view **C** Head, propleuron, lateral view **E** Mesonotum, dorsal view **F** Scutellum, metanotum, propodeum, dorsal view **G**T1–2, dorsal view **H, J** Metasoma **H** Dorsal view **J** Lateral view **I** Mesosoma, lateral view **K** Fore and hind wings.

Propleuron finely sculptured only ventrally (Fig. 139A, C), longitudinal median carina on face absent (Fig. 139B), surface of metasternum convex, edges of median area on T2 obscured by weak longitudinal stripes (Fig. 139G, H), dorsal outer depression on hind coxa absent (Fig. 139A, J), and fore wing with r vein slightly curved or curved, outer side of junction of r and 2RS veins forming a slight or distinct stub (Fig. 139K).

#### Coloration

(Fig. 139A–K). General body coloration polished black except scape and all antennal flagellomeres (on both sides) dark brown; pedicel, labrum and mandibles brown-red/reddish; glossa, maxillary and labial palps, and tegulae yellow; lunules, BS, PFM and BS with brown-red/reddish tints. Eyes silver and ocelli yellowish. Fore and middle legs yellow except brown claws and tarsomeres with brown tints; hind legs yellow except black coxae with apex yellow, femora with brown apex, distal half of tibiae brown and distally with a small brown band, and tarsomeres brown. Petiole on T1 black, contours darkened and sublateral areas yellow; T2 with median and wide adjacent areas brown, and lateral ends yellow-brown; T3 brown except a small yellow-brown area in proximal corners; T4 and beyond brown; distally each tergum with a narrow whitish translucent band. In lateral view, T1–2 yellow; T3–4 yellow, but dorsally yellow-brown, the extent of yellow-brown area increasing from proximal to distal; T5 and beyond completely brown. S1–2 yellow; S3 proximal half yellow, distal half brown; S4 and beyond brown.

#### Description.

**Head** (Fig. 139A–D). Head elongate with pubescence long and dense. Proximal three antennal flagellomeres longer than wide (0.26:0.09, 0.28:0.09, 0.25:0.09), distal antennal flagellomere longer than penultimate (0.13:0.05, 0.10:0.07), antenna longer than body (3.28, 2.83); antennal scrobes-frons sloped and forming a shelf. Face fine and punctate-lacunose, interspaces wavy and with lateral depression only middle, and longitudinal median carina absent. Frons smooth. Temple wide, punctations barely noticeable and interspaces clearly smooth. Inner margin of eyes diverging slightly at antennal sockets; in lateral view, eye anteriorly convex and posteriorly straight. POL shorter than OOL (0.08, 0.13). Malar suture absent or difficult to see. Median area between lateral ocelli without depression. Vertex laterally rounded and dorsally wide.

**Mesosoma** (Fig. 139A, E, F, I). Mesosoma dorsoventrally convex. Mesoscutum proximally convex and distally flat, punctation distinct throughout, interspaces smooth. Scutellum long and slender, apex sloped and fused with BS, but not in the same plane, scutellar punctation scattered throughout, in profile scutellum flat and on same plane as mesoscutum, phragma of the scutellum partially exposed; BS not overlapping the MPM; ATS demilune only inner side with sculpture; dorsal ATS groove with carinae only proximally. Transscutal articulation with small and heterogeneous foveae, area just behind transscutal articulation nearly at the same level as mesoscutum (flat) and with same kind of sculpture as mesoscutum. Metanotum with BM wider than PFM (clearly differentiated); MPM circular with some sculpturing inside; AFM without setiferous lobes and not as well delineated as PFM; PFM thick, smooth and with lateral ends rounded; ATM proximally with sculpture distally without a well delimited smooth area. Propodeum with a median longitudinal dent, but no trace of median longitudinal carina, proximal half weakly curved with medium-sized sculpture and distal half slightly rugose; distal edge of propodeum with a flange at each side and without stubs; propodeal spiracle distally framed by faintly concave/wavy carina; nucha surrounded by long radiating carinae. Pronotum with a distinct dorsal furrow, dorsally with a well-defined smooth band; central area of pronotum and dorsal furrow smooth, but ventral furrow with short parallel carinae. Propleuron finely sculptured only ventrally and dorsally with a carina. Metasternum convex. Contour of mesopleuron convex; precoxal groove smooth, shiny and shallow, but visible; epicnemial ridge convex, teardrop-shaped.

**Legs.** Ventral margin of fore telotarsus entire without seta, fore telotarsus almost same width throughout and longer than fourth tarsomere (0.14, 0.12). Hind coxa finely punctate throughout, and dorsal outer depression absent. Inner spur of hind tibia longer than outer spur (0.30, 0.21), entire surface of hind tibia with dense strong spines clearly differentiated by color and length. Hind telotarsus as equal in length as fourth tarsomere (0.18, 0.17).

**Wings** (Fig. 139K). Fore wing with r vein slightly curved; 2RS vein straight; r and 2RS veins forming a weak, even curve at their junction and outer side of junction forming a slight stub; 2M vein slightly curved/swollen; distally fore wing [where spectral veins are] with microtrichiae more densely concentrated than the rest of the wing; anal cell 1/3 proximally lacking microtrichiae; veins 2CUa and 2CUb completely spectral; vein 2 cu-a present as spectral vein, sometimes difficult to see; vein 2-1A proximally tubular and distally spectral, although sometimes difficult to see; tubular vein 1 cu-a straight, incomplete/broken and not reaching the edge of 1-1A vein. Hind wing with vannal lobe narrow, subdistally and subproximally straightened, and setae evenly scattered in the margin.

**Metasoma** (Fig. 139A, G, H, J). Metasoma laterally compressed. Petiole on T1 with a mix of fine rugae and punctate sculpture over most of the surface, virtually parallel-sided over most of length, but barely narrowing over distal 1/3, apex truncate (length 0.41, maximum width 0.19, minimum width 0.11), and with scattered pubescence concentrated in the first distal third. Lateral grooves delimiting the median area on T2 clearly defined and reaching the distal edge of T2 (length median area 0.21, length T2 0.21), edges of median area obscured by weak longitudinal stripes, median area broader than long (length 0.21, maximum width 0.23, minimum width 0.09); T2 with scattered pubescence throughout. T3 longer than T2 (0.24, 0.21) and with pubescence more notorious in distal half. Pubescence on hypopygium dense.

**Cocoons.** Unknown.

#### Comments.

In some specimens of the same sample, the coloration of sterna is a little different, distally all are brown with a longitudinal yellow band; the middle coxae proximally with a dorsal brown spot. In some specimens (e.g., EC-2807), the pronotum and the propleuron with brown-red/reddish tints. The malar space in this species is wide. The area between antennal scrobes is dented. The shape of the body is very elongated.

#### Male.

Similar in coloration to females. The same color variation found in females are also present in males.

#### Etymology.

Kevin P. Johnson is an American biologist. His major fields of interest are avian and insect systematics, host-parasite coevolution, island biogeography, population genetics, and behavioral ecology. He works at the Illinois Natural History Survey, Champaign, IL, USA.

#### Distribution.

Parasitized caterpillars were collected in Ecuador, Napo, Yanayacu Biological Station (Yanayacu Road), during May 2005 and April 2009 at 2,100 m in cloud forest.

#### Biology.

The lifestyle of this parasitoid species is gregarious.

#### Host.

Undetermined species of Erebidae (Arctiinae) feeding on *Rubus* sp. (Rosaceae). Caterpillar was collected in second instar.

### Glyptapanteles kyleparksi

Taxon classificationAnimaliaHymenopteraBraconidae

Arias-Penna, sp. nov.

http://zoobank.org/F37C106A-916B-4646-95A5-6AB258693F9C

Fig. 140

#### Female.

Body length 2.48 mm, antenna length 2.58 mm, fore wing length 3.03 mm.

#### Type material.

**Holotype**: ECUADOR • 1♀; EC-5125, YY-A074; Napo, Yanayacu Biological Station, YanayacuForest, Plot 31; cloud forest; 2,359 m; -0.6, -77.9; 06.vii.2005; Genoveva Rodriguez-Castañeda leg.; caterpillar collected in second instar; cocoons with “frill” around hosts body, sticking out perpendicular to larval cuticle and formed on 15.viii.2005; adult parasitoids emerged on 30.viii.2005; (PUCE). **Paratypes.** • 20 (5♀, 3♂) (12♀, 0♂); EC-5125, YY-A074; same data as for holotype; (PUCE).

#### Diagnosis.

**Figure 140.**
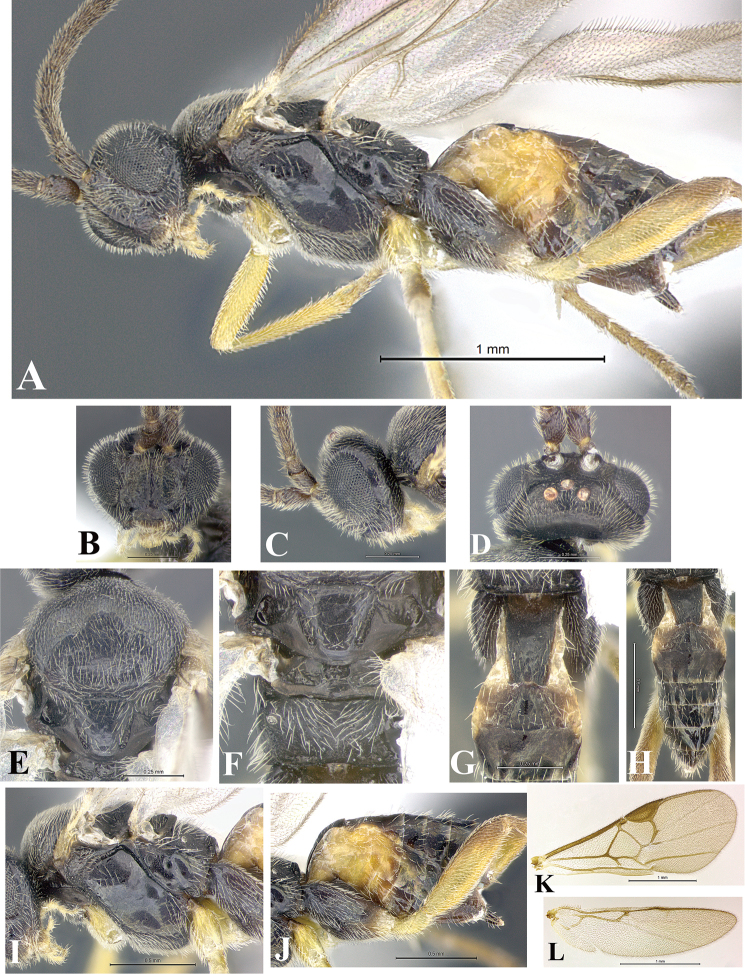
*Glyptapanteleskyleparksi* sp. nov. female EC-5125 YY-A074 **A** Habitus **B–D** Head **B** Frontal view **C** Lateral view **D** Dorsal view **E** Mesonotum, dorsal view **F** Scutellum, metanotum, propodeum, dorsal view **G**T1–2, dorsal view **H, J** Metasoma **H** Dorsal view **J** Lateral view **I** Mesosoma, lateral view **K, L** Wings **K** Fore **L** Hind.

Medioanterior pit of metanotum circular without median longitudinal carina and very partially covered by medioposterior band of scutellum (Fig. 140F), transscutal articulation with tiny homogeneous foveae without carina (Fig. 140E), inner margin of eyes diverging slightly at antennal sockets (Fig. 140B), median area on T2 broader than long, edges of median area on T2 obscured by weak longitudinal stripes (Fig. 140G, H), ventral margin of fore telotarsus entire without seta, anteroventral contour of mesopleuron straight/angulate (Fig. 140A, I), propleuron with fine punctations throughout, longitudinal median carina on face present (Fig. 140B), surface of metasternum convex, dorsal outer depression on hind coxa absent (Fig. 140A, J), and fore wing with r vein curved, outer side of junction of r and 2RS veins forming a stub (Fig. 140K).

#### Coloration

(Fig. 140A–L). General body coloration brown except scape and pedicel with apex yellow-brown; first four-five proximal antennal flagellomeres dorsally lighter (light brown) than ventrally (dark brown), remaining flagellomeres dark brown on both sides; labrum and mandibles yellow-brown; glossa, maxillary and labial palps, and tegulae yellow; pronotum, lunules, BS, AFM, and PFM with brown-red/reddish tints. Eyes gray and ocelli reddish (in preserved specimen). Fore and middle legs yellow except brown claws; hind legs yellow except black coxae with apex yellow/yellow-brown (coloration that is more extended ventrally), femora with a small brown area in the apex, distal half of tibiae brown and distally with a small brown band, and tarsomeres brown. Petiole on T1 with two colorations: distally brown and proximally brown-red/reddish, and sublateral areas yellow; T2 with median and adjacent areas brown, and lateral ends yellow-brown; T3 completely brown except a small area in the proximal half of lateral ends; T4 and beyond brown; distally each tergum with a narrow yellowish translucent band. In lateral view, T1–3 completely yellow; T4 yellow, but distally with a brown band; T5 and beyond completely brown. S1–2 yellow; S3–4 proximal half yellow, distal half brown; penultimate sternum and hypopygium completely brown.

#### Description.

**Head** (Fig. 140A–D). Head rectangular with pubescence long and dense. Proximal three antennal flagellomeres longer than wide (0.22:0.06, 0.20:0.06, 0.20:0.06), distal antennal flagellomere longer than penultimate (0.13:0.05, 0.10:0.05), antenna longer than body (2.58, 2.48); antennal scrobes-frons sloped and forming a shelf. Face finely punctate-lacunose, interspaces wavy, distal half dented only laterally, and longitudinal median carina present. Frons smooth. Temple wide, punctate and interspaces clearly smooth. Inner margin of eyes diverging slightly at antennal sockets; in lateral view, eye anteriorly convex and posteriorly straight. POL shorter than OOL (0.07, 0.12). Malar suture absent or difficult to see. Median area between lateral ocelli slightly depressed. Vertex laterally pointed or nearly so and dorsally wide.

**Mesosoma** (Fig. 140A, E, F, I). Mesosoma dorsoventrally convex. Mesoscutum distal half with a central dent, punctation distinct throughout, interspaces smooth. Scutellum triangular, apex sloped and fused with BS, but not in the same plane, scutellar punctation distinct peripherally, absent centrally; in profile scutellum flat and on same plane as mesoscutum, phragma of the scutellum partially exposed; BS only very partially overlapping the MPM; ATS demilune inner side with a row of foveae; dorsal ATS groove smooth. Transscutal articulation with small and homogeneous foveae, area just behind transscutal articulation nearly at the same level as mesoscutum (flat) and with same kind of sculpture as mesoscutum. Metanotum with BM wider than PFM (clearly differentiated); MPM circular without median longitudinal carina; AFM with a small lobe and not as well delineated as PFM; PFM thick, smooth and with lateral ends rounded; ATM proximally with sculpture distally without a well delimited smooth area. Propodeum finely sculptured without median longitudinal carina, proximal half weakly curved with fine sculpture and distal half with a shallow dent at each side of nucha; distal edge of propodeum with a flange at each side and without stubs; propodeal spiracle without distal carina; nucha surrounded by very short radiating carinae. Pronotum with a distinct dorsal furrow, dorsally with a well-defined smooth band; central area of pronotum and dorsal furrow smooth, but ventral furrow with short parallel carinae. Propleuron with fine punctations throughout and dorsally without a carina. Metasternum convex. Contour of mesopleuron straight/angulate or nearly so; precoxal groove smooth, shiny and shallow, but visible; epicnemial ridge convex, teardrop- shaped.

**Legs.** Ventral margin of fore telotarsus entire without seta, fore telotarsus proximally narrow and distally wide, and longer than fourth tarsomere (0.13, 0.06). Hind coxa finely punctate throughout, and dorsal outer depression absent. Inner spur of hind tibia longer than outer spur (0.21, 0.17), entire surface of hind tibia with dense strong spines clearly differentiated by color and length. Hind telotarsus as equal in length as fourth tarsomere (0.12, 0.13).

**Wings** (Fig. 140K, L). Fore wing with r vein curved; 2RS vein straight; r and 2RS veins forming a weak, even curve at their junction and outer side of junction forming a distinct stub; 2M vein slightly curved/swollen; distally fore wing [where spectral veins are] with microtrichiae more densely concentrated than the rest of the wing; anal cell 1/3 proximally lacking microtrichiae; subbasal cell with microtrichiae virtually throughout; veins 2CUa and 2CUb completely spectral; vein 2 cu-a present as spectral vein, sometimes difficult to see; vein 2-1A proximally tubular and distally spectral, although sometimes difficult to see; tubular vein 1 cu-a straight, incomplete/broken and not reaching the edge of 1-1A vein. Hind wing with vannal lobe very narrow, subdistally and subproximally straightened, and setae present proximally, but absent distally.

**Metasoma** (Fig. 140A, E, F, I). Metasoma laterally compressed. Petiole on T1, distal half with faint rugae only laterally, with virtually parallel-sided over most of length, but barely narrowing over distal 1/3, apex truncate (length 0.38, maximum width 0.19, minimum width 0.14), and with scattered pubescence on distal half. Lateral grooves delimiting the median area on T2 clearly defined and reaching the distal edge of T2 (length median area 0.16, length T2 0.16), edges of median area obscured by weak longitudinal stripes, median area broader than long (length 0.16, maximum width 0.20, minimum width 0.12); T2 with scattered pubescence throughout. T3 longer than T2 (0.21, 0.16) and with pubescence more notorious in distal half. Pubescence on hypopygium dense.

**Cocoons.** With “frill” around hosts body and attached to larval cuticle.

#### Male.

Similar coloration to female.

#### Etymology.

Kyle Parks is an American entomologist. As a graduate student at the UIUC, IL, USA, he was interested in Microgastrinae, mainly the genera *Parapanteles* and *Clarkinella*. Mason.

#### Distribution.

Parasitized caterpillar was collected in Ecuador, Napo, Yanayacu Biological Station (YanayacuForest), during July 2005 at 2,359 m in cloud forest.

#### Biology.

The lifestyle of this parasitoid species is gregarious.

#### Host.

Undetermined species of Nymphalidae, food plant was not reported. Caterpillar was collected in second instar.

### Glyptapanteles linghsiuae

Taxon classificationAnimaliaHymenopteraBraconidae

Arias-Penna, sp. nov.

http://zoobank.org/9EF1DDCB-1EEE-4849-B14A-E26E019FC506

Fig. 141

#### Female.

Body length 2.58 mm, antenna length 3.13 mm [only 13 antennal flagellomeres in other female 3.48 mm], fore wing length 3.64 mm.

#### Type material.

**Holotype**: ECUADOR • 1♀; EC-4711, YY-A079; Napo, Yanayacu Biological Station, YanayacuForest; 2,100 m; -0.6, -77.883333; 13.vi.2005; CAPEA leg.; caterpillar collected in late instar or pre-pupa; adult parasitoids emerged on 26.vi.2005; (PUCE). **Paratypes.** • 29 (6♀, 1♂) (22♀, 0♂); EC-4711, YY-A079; same data as for holotype; (PUCE).

#### Diagnosis.

**Figure 141.**
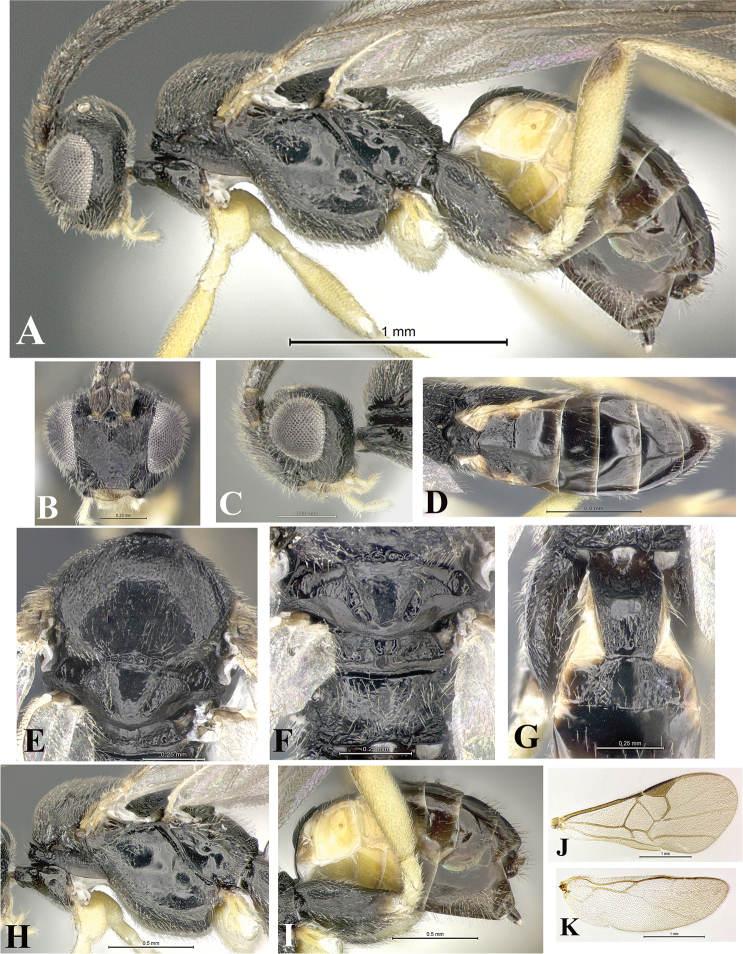
*Glyptapanteleslinghsiuae* sp. nov. female EC-4711 YY-A079 **A** Habitus **B, C** Head **B** Frontal view **C** Lateral view **D, I** Metasoma **D** Dorsal view **I** Lateral view **E** Mesonotum, dorsal view **F** Scutellum, metanotum, propodeum, dorsal view **G**T1–2, dorsal view **H** Mesosoma, lateral view **J, K** Wings **J** Fore **K** Hind.

Medioanterior pit of metanotum circular and bisected by a median longitudinal carina (Fig. 141F), edges of median area on T2 obscured by coarse sculpture (Fig. 141G), scutellar punctation scattered throughout (Fig. 141E, F), in lateral view, metasoma curved (Fig. 141A, I), dorsal outer depression on hind coxa absent (Fig. 141A, I), and fore wing with r vein slightly curved or curved, outer side of junction of r and 2RS veins forming a slight or distinct stub (Fig. 141J).

#### Coloration

(Fig. 141A–K). General body coloration polished black except labrum, mandibles, glossa, maxillary and labial palps, and tegulae yellow; distally both scape and pedicel with yellow-brown ring; all antennal flagellomeres dark brown on both sides. Eyes gray-purple and ocelli yellowish (in preserved specimen). Fore and middle legs yellow except brown claws and tarsomeres with a light brown tints; hind legs yellow except black coxae with apex yellow, femora with a small brown spot in the apex, distal half of tibiae brown, and tarsomeres brown. Petiole on T1 black and sublateral areas yellow; T2 with median and adjacent areas brown, adjacent area with contours well-defined, both areas together forming a rectangle-shaped area, and lateral ends yellow with one elongate brown spot at each side; T3 completely brown except dorsal corner with a small pale spot; T4 and beyond brown; distally each tergum with a narrrow yellow-brown translucent band. In lateral view, T1–2 completely yellow; T3 yellow, dorsally yellow-brown; T4 and beyond brown. S1–4 yellow; penultimate sternum brown-red/reddish; hypopygium brown.

#### Description.

**Head** (Fig. 141A–C). Head rectangular with pubescence long and dense. Proximal three antennal flagellomeres longer than wide (0.25:0.09, 0.25:0.09, 0.25:0.09), distal antennal flagellomere longer than penultimate (0.15:0.09, 0.12:0.09), antenna longer than body; antennal scrobes-frons shallow. Face flat or nearly so, punctations barely noticeable, interspaces smooth and longitudinal median carina present. Frons smooth. Temple wide, punctations barely noticeable and interspaces wavy. Inner margin of eyes diverging slightly at antennal sockets; in lateral view, eye anteriorly convex and posteriorly straight. POL shorter than OOL (0.10, 0.14). Malar suture present. Median area between lateral ocelli without depression. Vertex laterally rounded and dorsally wide.

**Mesosoma** (Fig. 141A, E, F, H). Mesosoma dorsoventrally convex. Mesoscutum proximally convex and distally flat, punctation distinct throughout, interspaces smooth. Scutellum long and slender, apex sloped and fused with BS, but not in the same plane, scutellar punctation scattered throughout, in profile scutellum flat and on same plane as mesoscutum, phragma of the scutellum partially exposed; BS only very partially overlapping the MPM; ATS demilune with complete and undulate/reticulate carinae; dorsal ATS groove smooth. Transscutal articulation with small and heterogeneous foveae, area just behind transscutal articulation nearly at the same level as mesoscutum (flat) and with same kind of sculpture as mesoscutum. Metanotum with BM convex; MPM circular and bisected by a median longitudinal carina; AFM without setiferous lobes and not as well delineated as PFM; PFM thick and smooth; ATM with little, incomplete and parallel carinae proximally. Propodeum with a median longitudinal dent, but no trace of median longitudinal carina, proximal half weakly curved with medium-sized sculpture and distal half with fine sculpture and with medium-sized punctation; distal edge of propodeum with a flange at each side and short stubs; propodeal spiracle without distal carina; nucha surrounded by very short radiating carinae. Pronotum with a distinct dorsal furrow, dorsally with a well-defined smooth band; central area of pronotum smooth, but both dorsal and ventral furrows with short parallel carinae. Propleuron finely sculptured only ventrally and dorsally without a carina. Metasternum convex. Contour of mesopleuron convex; precoxal groove smooth, shiny and shallow, but visible; epicnemial ridge convex, teardrop-shaped.

**Legs.** Ventral margin of fore telotarsus entire without seta, fore telotarsus almost same width throughout and longer than fourth tarsomere (0.14, 0.07). Hind coxa finely punctate throughout, and dorsal outer depression absent. Inner spur of hind tibia longer than outer spur (0.24, 0.18), entire surface of hind tibia with dense strong spines clearly differentiated by color and length. Hind telotarsus longer than fourth tarsomere (0.15, 0.13).

**Wings** (Fig. 141J, K). Fore wing with r vein slightly curved; 2RS vein straight; r and 2RS veins forming a weak, even curve at their junction and outer side of junction forming a slight stub; 2M vein slightly curved/swollen; distally fore wing [where spectral veins are] with microtrichiae more densely concentrated than the rest of the wing; anal cell 1/3 proximally lacking microtrichiae; subbasal cell with microtrichiae virtually throughout; veins 2CUa and 2CUb completely spectral; vein 2 cu-a present as spectral vein, sometimes difficult to see; vein 2-1A proximally tubular and distally spectral, although sometimes difficult to see; tubular vein 1 cu-a straight, incomplete/broken and not reaching the edge of 1-1A vein. Hind wing with vannal lobe narrow, subdistally and subproximally straightened, and setae evenly scattered in the margin.

**Metasoma** (Fig. 141A, D, G, I). Metasoma curved. Petiole on T1, laterally with a mix of rugae and coarse sculpture, virtually parallel-sided over most of length, but barely narrowing at apex, apex truncate (length 0.45, maximum width 0.26, minimum width 0.17), and with scattered pubescence on distal half. Lateral grooves delimiting the median area on T2 clearly defined and reaching the distal edge of T2 (length median area 0.19, length T2 0.19), edges of median area obscured by coarse sculpture, median area broader than long (length 0.19, maximum width 0.33, minimum width 0.15); T2 with scarce pubescence throughout. T3 longer than T2 (0.26, 0.19) and with scattered pubescence throughout. Pubescence on hypopygium dense.

**Cocoons.** Unknown.

#### Comments.

The metasoma is distinctively curved. Both sexes with slim bodies.

#### Male.

Similar in coloration to female and with large genitalia.

#### Etymology.

Ling-Hsiu Liao is a Taiwanese entomologist. As a graduate student at UIUC, IL, USA, she studied the plant-insect interactions and detoxification processes in honey bees.

#### Distribution.

Parasitized caterpillar was collected in Ecuador, Napo, Yanayacu Biological Station (YanayacuForest), during June 2005 at 2,100 m in cloud forest.

#### Biology.

The lifestyle of this parasitoid species is gregarious.

#### Host.

*Hypanartia* sp. Hübner (Nymphalidae: Nymphalinae) feeding on *Boehmeria* sp. (Urticaceae). Caterpillar was collected in late instar or pre-pupa.

### Glyptapanteles lubomasneri

Taxon classificationAnimaliaHymenopteraBraconidae

Arias-Penna, sp. nov.

http://zoobank.org/5F6029A3-0CDD-4AB2-AB9F-FA8FC3EDE6D0

Figs 142 , 143

#### Female.

Body length 2.07 mm, antenna length 2.53 mm, fore wing length 2.53 mm.

#### Type material.

**Holotype**: COSTA RICA • 1♀; 06-SRNP-31462, DHJPAR0005112; Área de Conservación Guanacaste, Guanacaste, Sector Pitilla, Pasmompa; rain forest; 440 m; 11.01926, -85.40997; 05.iii.2007; Manuel Rios leg.; caterpillar collected in second instar; white bud-like cocoons and adhered to the larval cuticle, cocoons formed on 13.iv.2006; adult parasitoid emerged on 18.iv.2006; (CNC). **Paratypes**. • 4 (1♀, 0♂) (3♀, 0♂); 06-SRNP-31462, DHJPAR0005112; same data as for holotype; (CNC).

#### Other material.

**Reared material.** COSTA RICA: *Área de Conservación Guanacaste*, *Guanacaste*, *Sector Pitilla*, *Sendero Memos*: • 3 (2♀, 0♂) (1♀, 0♂); 05-SRNP-31506, DHJPAR0004242; rain forest; 740 m; 10.98171, -85.42785; 14.iv.2005; Manuel Rios leg.; caterpillar collected in third instar; elongate white bud-like cocoons lightly adhered to each other, cocoons adhered to the leaf substrate, cocoons formed on 24.iv.2005; adult parasitoid emerged on 29.iv.2005.

*Área de Conservación Guanacaste*, *Alajuela*, *Sector San Cristóbal*, *Tajo Ángeles*: • 3 (2♀, 0♂) (0♀, 1♂); 11-SRNP-1210, DHJPAR0042897; rain forest; 540 m; 10.86472, -85.41531; 19.iii.2011; Carolina Cano leg.; caterpillar collected in third instar; cocoons in litter or soil; adult parasitoids emerged on 03.iv.2011. • 8 (2♀, 0♂) (6♀, 0♂); 11-SRNP-1211, DHJPAR0042908; same data for preceding except: adult parasitoid emerged on 04.iv.2011.

#### Diagnosis.

**Figure 142.**
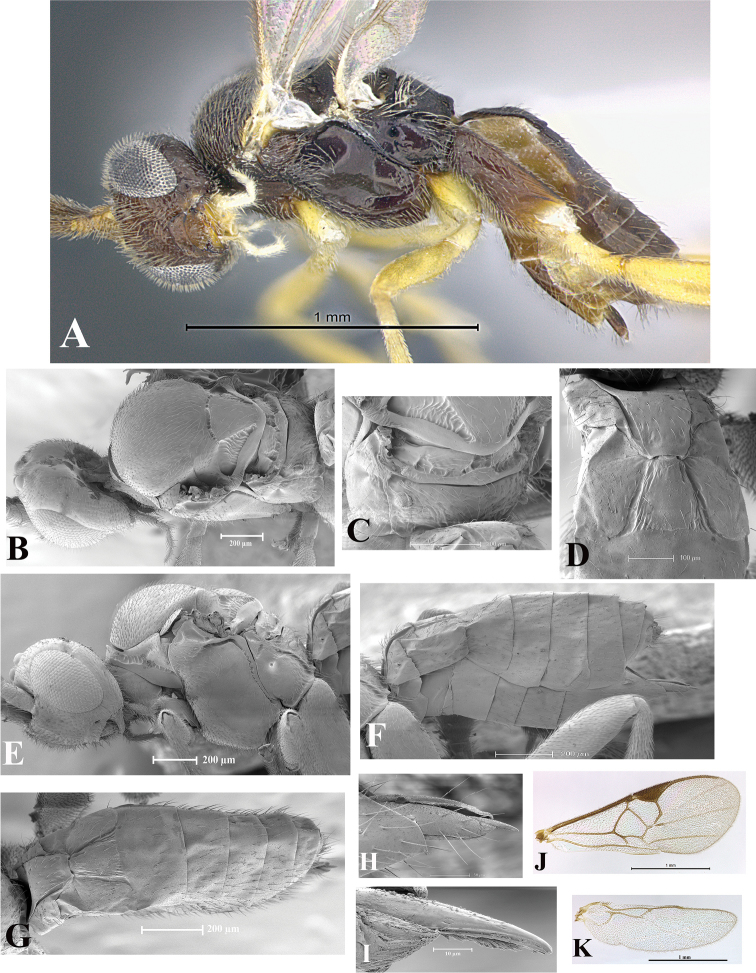
*Glyptapanteleslubomasneri* sp. nov. female 07-SRNP-547 DHJPAR0012892 **A** Habitus **B, E** Head, mesosoma **B** Dorsolareral view **E** Lateral view **C** Metanotum, propodeum, dorsolateral view **D**T1–2, dorsal view **F, G** Metasoma **F** Lateral view **G** Dorsal view **H, I** Genitalia **H** Hypopygium, ovipositor, ovipositor sheaths, lateral view **I** Detail **J, K** Wings **J** Fore **K** Hind.

**Figure 143.**
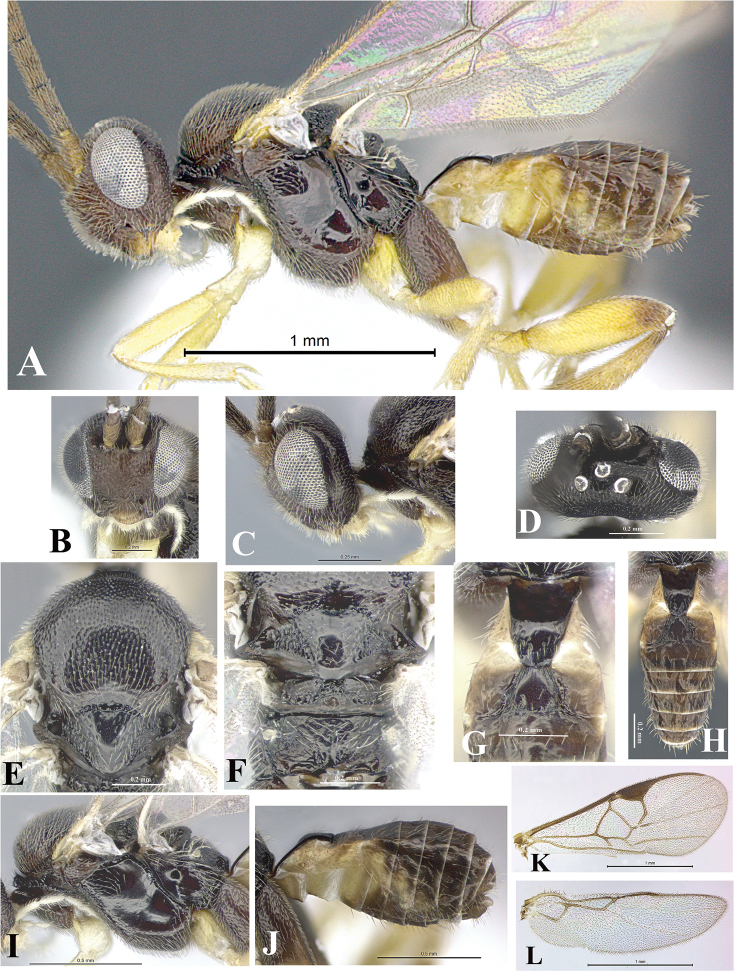
*Glyptapanteleslubomasneri* sp. nov. male 07-SRNP-547 DHJPAR0012892 **A** Habitus **B–D** Head **B** Frontal view **C** Lateral view **D** Dorsal view **E** Mesonotum, dorsal view **F** Scutellum, metanotum, propodeum, dorsal view **G**T1–2, dorsal view **H, J** Metasoma **H** Dorsal view **J** Lateral view **I** Mesosoma, lateral view **K, L** Wings **K** Fore **L** Hind.

Fore telotarsus almost same width throughout, ventral margin without seta, medioposterior band of scutellum only very partially overlapping the medioanterior pit of metanotum (Figs 142C, 143F), phragma of the scutellum partially exposed (Figs 142B, 143F), fore wing with vein 2 cu-a absent, with r vein straight, outer side of junction of r and 2RS veins forming a stub (Figs 142J, 143K), median area on T2 broader than long, lateral grooves delimiting the median area on T2 distally losing definition on T2 (Fig. 143G), and edges of median area on T2 obscured by weak longitudinal stripes (Figs 142D, 143G), vertex in dorsal view wide (Fig. 143D), in lateral view, metasoma laterally compressed (Figs 142A, 143J), T3 longer than T2 (Figs 142G, 143H), inner margin of eyes diverging slightly at antennal sockets (Fig. 143B), petiole on T1 evenly narrowing distally and finely sculptured (Fig. 143G, H), and propodeum without a median longitudinal dent (Figs 142C, 143F).

#### Coloration

(Fig. 142A). General body coloration dark brown except scape and pedicel yellow-brown; all antennal flagellomeres dark brown on both sides; labrum and mandibles yellow-brown; glossa, maxillary and labial palps yellow; tegulae light brown. Eyes silver and ocelli reddish (in preserved specimen). Fore and middle legs yellow except brown claws; hind legs yellow except light brown coxae (brown coloration is more extensive on the inner side), distal brown spot on the femora, both ends of tibia and tarsomeres brown, although proximally basitarsus with a small yellow band. Petiole on T1 dark brown and sublateral areas yellow; T2 with median area brown, adjacent area yellow-brown which boundaries with lateral ends are blurred, and yellow lateral ends with two elongate brown spots each one at distal corners; T3 almost completely brown, but proximally with a tiny yellow area; T4 and beyond completely brown; distally each tergum with a narrow yellowish transparent band. In lateral view, T1–2 completely yellow; T3 proximal half yellow, distal half brown; T4 and beyond completely brown. S1–3 yellow; S4 and beyond light brown/brown.

#### Description.

**Head** (Fig. 142A, B, E). Head rounded with pubescence long and dense. Proximal three antennal flagellomeres longer than wide (0.20:0.06, 0.29:0.06, 0.19:0.06), distal antennal flagellomere longer than penultimate (0.13:0.05, 0.10:0.05), antenna longer than body (2.53, 2.07); antennal scrobes-frons shallow. Face dense fine punctations, interspaces smooth, depression only laterally, and longitudinal median carina present. Frons smooth. Temple wide, punctate and interspaces clearly smooth. Inner margin of eyes diverging slightly at antennal sockets; in lateral view, eye anteriorly convex and posteriorly straight. POL shorter than OOL (0.08, 0.13). Malar suture present. Median area between lateral ocelli without depression. Vertex laterally rounded and dorsally wide.

**Mesosoma** (Fig. 142A–C, E). Mesosoma dorsoventrally convex. Mesoscutum proximally convex and distally flat, punctation distinct throughout, interspaces wavy/lacunose. Scutellum triangular, apex sloped and fused with BS, scutellar punctation scattered throughout, in profile scutellum flat and on same plane as mesoscutum, phragma of the scutellum partially exposed; BS only very partially overlapping the MPM; ATS demilune with quite a little complete parallel carinae; dorsal ATS groove with carinae only proximally. Transscutal articulation with small and heterogeneous foveae, area just behind transscutal articulation with a smooth and shiny sloped transverse strip. Metanotum with BM wider than PFM (clearly differentiated); MPM circular and bisected by a median longitudinal carina; AFM with a small lobe and not as well delineated as PFM; PFM thick and smooth; ATM proximally with a groove with some sculpturing and distally smooth. Propodeum without median longitudinal carina, proximal half straight or nearly so with medium-sized sculpture and distal half relatively polished; distal edge of propodeum with a flange at each side and without stubs; propodeal spiracle without distal carina; nucha surrounded by very short radiating carinae. Pronotum with a distinct dorsal furrow, dorsally with a well-defined smooth band; central area of pronotum smooth, but both dorsal and ventral furrows with short parallel carinae. Propleuron with fine punctations throughout and dorsally without a carina. Metasternum flat or nearly so. Contour of mesopleuron straight/angulate or nearly so; precoxal groove deep, smooth and shiny; epicnemial ridge elongated more fusiform (tapering at both ends).

**Legs.** Ventral margin of fore telotarsus entire without seta, fore telotarsus almost same width throughout and longer than fourth tarsomere (0.11, 0.06). Dorsal half of hind coxa with scattered punctation and ventral half with dense punctation, and dorsal outer depression present. Inner spur of hind tibia longer than outer spur (0.21, 0.16). Entire surface of hind tibia with dense strong spines clearly differentiated by color and length. Hind telotarsus as equal in length as fourth tarsomere (0.10, 0.09).

**Wings** (Fig. 142J, K). Fore wing with r vein straight; 2RS vein straight; r and 2RS veins forming an angle at their junction and outer side of junction forming a slight stub; 2M vein slightly curved/swollen; distally fore wing [where spectral veins are] with microtrichiae more densely concentrated than the rest of the wing; anal cell 1/3 proximally lacking microtrichiae; subbasal cell with a small smooth area; veins 2CUa and 2CUb completely spectral; vein 2 cu-a absent; vein 2-1A proximally tubular and distally spectral, although sometimes difficult to see; tubular vein 1 cu-a curved and complete, but junction with 1-1A vein spectral. Hind wing with vannal lobe very narrow, subdistally and subproximally straightened, and setae evenly scattered in the margin.

**Metasoma** (Fig. 142A, D, F–I). Metasoma laterally compressed. Petiole on T1 finely sculptured only distally, evenly narrowing distally (length 0.31, maximum width 0.15, minimum width 0.08) and with scattered pubescence concentrated in the first distal third. Lateral grooves delimiting the median area on T2 clearly defined and reaching the distal edge of T2 (length median area 0.14, length T2 0.14), edges of median area obscured by weak longitudinal stripes, median area broader than long (length 0.14, maximum width 0.17, minimum width 0.07); T2 with scattered pubescence only distally. T3 longer than T2 (0.20, 0.14) and with scattered pubescence only distally. Pubescence on hypopygium dense.

**Cocoons.** White bud-like cocoon with body ridge-shaped and evenly smooth silk fibers. Cocoons lightly adhered to each other and adhered to the larval cuticle, in litter or soil.

#### Comments.

The coloration of the adjacent area on T2 is lighter. In some females, the sterna coloration differs a little: S1 yellow, but medially is brown, S4 proximal half yellow and distal half brown.

#### Male

(Fig. 143A–L). Similar coloration and shape to females.

#### Etymology.

Lubomir (Lubo) Masner is a Czech-born entomologist. He is the world specialist in the systematics of proctotrupoid parasitioid wasps. Currently, he is an honorary research associate at Agriculture and Agri-Food Canada, Ottawa, Ontario, Canada.

#### Distribution.

The parasitized caterpillars were collected in Costa Rica, ACG, Sector Pitilla (Pasmompa and Sendero Memos) and Sector San Cristóbal (Tajo Ángeles), during April 2005, March 2007, and March 2011 at 440 m, 540 m, and 740 m in rain forest.

#### Biology.

The lifestyle of this parasitoid species is gregarious.

#### Host.

*Ithomiahippocrenis* Bates (Nymphalidae: Ithomiinae) feeding on *Witheringiasolanacea* (Solanaceae) and *Mechanitisisthmia* (Nymphalidae: Ithomiinae) feeding on *Solanumhayesii* (Solanaceae). Caterpillars were collected in second and third instar.

### Glyptapanteles luchosalagajei

Taxon classificationAnimaliaHymenopteraBraconidae

Arias-Penna, sp. nov.

http://zoobank.org/6798913D-E334-42BC-B374-CB15E4173791

Fig. 144

#### Female.

Body length 2.58 mm, antenna length 2.73 mm, fore wing length 3.28 mm.

#### Type material.

**Holotype**: ECUADOR • 1♀; EC-6135, YY-A027; Napo, Yanayacu Biological Station, Yanayacu Road; cloud forest; 2,100 m; -0.566667, -77.866667; 19.viii.2005; Earthwatch volunteers leg.; adult parasitoids emerged on 29.ix.2005; (PUCE). **Paratypes.** • 57 (6♀, 2♂) (49♀, 0♂); EC-6135, YY-A027; same data as for holotype; (PUCE).

#### Other material.

**Reared material.** ECUADOR: *Napo*, *Yanayacu Biological Station*, *Yanayacu Road*: • 6 (2♀, 3♂) (1♀, 0♂); EC-2783, YY-A024; cloud forest; 2,100 m; -0.566667, -77.866667; 22.v.2005; Harold Greeney leg.; adult parasitoid emerged on 22.vii.2005. • 15 (5♀, 1♂ broken) (9♀, 0♂); EC-2868, YY-A023; same data as for preceding except: 24.v.2005; CAPEA leg.; adult parasitoids emerged on 18.vi.2005. • 16 (5♀, 3♂) (8♀, 0♂); EC-7033, YY-A031; same data as for preceding except: 05.ix.2005; Earthwatch volunteers leg.; caterpillar collected in fourth instar; cocoons formed on 26.ix.2005; adult parasitoids emerged on 13.x.2005. • 33 (5♀, 5♂) (23♀, 10♂); EC-36044, YY-A003; same data as for preceding except: 13.xi.2008; CAPEA leg.; caterpillar collected in second instar; cocoons formed on 12.xii.2008; adult parasitoids emerged on 23.xii.2008.

*Napo*, *Yanayacu Biological Station*, *Cascada San Rafael*, *Plot 5*: • 1 (1♀, 0♂) (0♀, 0♂); EC-4233, YY-A026; cloud forest; 1,275 m; -0.1, -77.583333 (+50m W); 03.vi.2005; Grant Gentry leg.; cocoons formed on 06.vi.2005; adult parasitoids emerged on 18.vi.2005; *Mesochorus* sp. (Ichneumonidae: Mesochorinae) was reported as hyperparasitoid.

#### Diagnosis.

**Figure 144.**
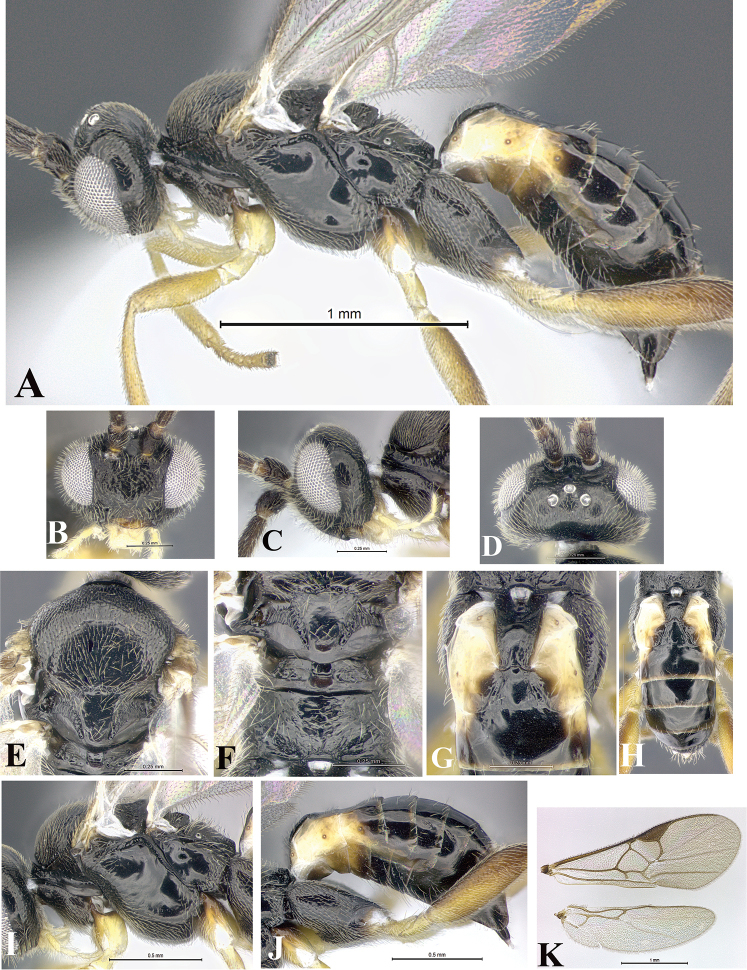
*Glyptapantelesluchosalagajei* sp. nov. female EC-6135 YY-A027, EC-7033 YY-A031 **A** Habitus **B, D** Head **B** Frontal view **D** Dorsal view **C** Head, propleuron, lateral view **E** Mesonotum, dorsal view **F** Scutellum, metanotum, propodeum, dorsal view **G**T1–3, dorsal view **H, J** Metasoma **H** Dorsal view **J** Lateral view **I** Mesosoma, lateral view **K** Fore and hind wings.

In lateral view, metasoma curved (Fig. 144A, J), hind coxa medium-size punctate throughout (Fig. 144A, J), antenna longer than body, scutellar punctation distinct peripherally, absent centrally (Fig. 144E, F), edges of median area on T2 with little sculpture (Fig. 144G, H), petiole on T1 parallel-sided in proximal half and then narrowing (Fig. 144G), dorsal outer depression on hind coxa present (Fig. 144A, J), and fore wing with r vein slightly curved, outer side of junction of r and 2RS veins forming a slight stub (Fig. 144K).

#### Coloration

(Fig. 144A–K). General body coloration polished black except pedicel brown-red/reddish; first four-five proximal antennal flagellomeres dorsally lighter (light brown) than ventrally (dark brown), remaining flagellomeres dark brown on both sides; scape dark brown; labrum and mandibles yellow-brown; glossa, maxillary and labial palps, and tegulae yellow; distal corners of mesoscutum, dorsal half of lunules, BM and lateral ends of metanotum with brown-red/reddish tints. Eyes and ocelli silver. Fore and middle legs yellow except coxae proximally with a dark area, claws brown and middle femora with a narrow dorsal brown strip from top to bottom; hind legs yellow except black coxae, femora distally with a small brown spot additionally with a narrow dorsal brown strip from top to bottom, distal half of tibiae brown and proximally with a brown ring, and tarsomeres brown. Petiole on T1 black and sublateral areas yellow; T2 with median and adjacent areas brown, adjacent area with contours well-defined and together with adjacent area forming a rectangle-shaped area, and lateral ends yellow-brown; T3 completely brown except proximally corners with a small pale spot; T4 and beyond brown; distally each tergum with a narrow yellow-translucent band. In lateral view, T1–2 yellow; T3 yellow, but dorsally black; T4 and beyond brown. S1–3 proximal half yellow, distal half brown; S4 and beyond black.

#### Description.

**Head** (Fig. 144A–D). Head rounded with pubescence long and dense. Proximal three antennal flagellomeres longer than wide (0.19:0.09, 0.20:0.09, 0.21:0.09), distal antennal flagellomere longer than penultimate (0.12:0.06, 0.10:0.06), antenna longer than body (2.73, 2.58); antennal scrobes-frons shallow. Face flat or nearly so, punctate-lacunose, interspaces wavy and longitudinal median carina absent. Frons smooth. Temple wide, punctate-lacunose and interspaces wavy. Inner margin of eyes diverging slightly at antennal sockets; in lateral view, eye anteriorly convex and posteriorly straight. POL shorter than OOL (0.09, 0.14). Malar suture present. Median area between lateral ocelli slightly depressed. Vertex laterally pointed or nearly so and dorsally wide.

**Mesosoma** (Fig. 143A, E, F, I). Mesosoma dorsoventrally convex. Mesoscutum proximally convex and distally flat, punctation distinct throughout, interspaces smooth. Scutellum long and slender, apex sloped and fused with BS, but not in the same plane, scutellar punctation distinct peripherally and absent centrally, in profile scutellum flat and on same plane as mesoscutum, phragma of the scutellum partially exposed; BS only very partially overlapping the MPM; ATS demilune entirely covered by parallel carinae; dorsal ATS groove with carinae only proximally. Transscutal articulation with small and heterogeneous foveae, area just behind transscutal articulation nearly at the same level as mesoscutum (flat) and with same kind of sculpture as mesoscutum. Metanotum with BM convex; MPM semicircular without median longitudinal carina; AFM with a small lobe and not as well delineated as PFM; PFM thick and smooth; ATM proximally with a groove with some sculpturing and distally smooth. Propodeum with medium-sized punctation and with a median longitudinal dent, but no trace of median longitudinal carina, proximal half curved; distal edge of propodeum with a flange at each side and without stubs; propodeal spiracle without distal carina; nucha surrounded by very short radiating carinae. Pronotum with a distinct dorsal furrow, dorsally with a well-defined smooth band, central area of pronotum smooth, but both dorsal and ventral furrows with short parallel carinae. Propleuron with fine punctations throughout and dorsally without a carina. Metasternum convex. Contour of mesopleuron convex; precoxal groove smooth, shiny and shallow, but visible; epicnemial ridge elongated more fusiform (tapering at both ends).

**Legs.** Ventral margin of fore telotarsus entire without seta, fore telotarsus almost same width throughout and longer than fourth tarsomere (0.15, 0.09). Hind coxa with medium-size punctate throughout and dorsal outer depression present. Inner spur of hind tibia longer than outer spur (0.23, 0.17), entire surface of hind tibia with dense strong spines clearly differentiated by color and length. Hind telotarsus as equal in length as fourth tarsomere (0.15, 0.14).

**Wings** (Fig. 144K). Fore wing with r vein slightly curved; 2RS vein straight; r and 2RS veins forming a weak, even curve at their junction and outer side of junction forming a slight stub; 2M vein slightly curved/swollen; distally fore wing [where spectral veins are] with microtrichiae more densely concentrated than the rest of the wing; anal cell 1/3 proximally lacking microtrichiae; subbasal cell with microtrichiae virtually throughout; veins 2CUa and 2CUb completely spectral; vein 2 cu-a present as spectral vein, sometimes difficult to see; vein 2-1A proximally tubular and distally spectral, although sometimes difficult to see; tubular vein 1 cu-a straight, incomplete/broken and not reaching the edge of 1-1A vein. Hind wing with vannal lobe very narrow, subdistally and subproximally straightened, and setae evenly scattered in the margin.

**Metasoma** (Fig. 144A, G, H, J). Metasoma curved. Petiole on T1 finely sculptured only laterally, parallel-sided in proximal half and then narrowing (length 0.36, maximum width 0.20, minimum width 0.12), and with scattered pubescence concentrated in the first distal third. Lateral grooves delimiting the median area on T2 clearly defined and reaching the distal edge of T2 (length median area 0.19, length T2 0.19), edges of median area with little sculpture, median area broader than long (length 0.19, maximum width 0.25, minimum width 0.11); T2 with pubescence in distal half. T3 longer than T2 (0.24, 0.19) and with a distinctive row of pubescence only at the distal margin. Pubescence on hypopygium dense.

**Cocoons.** Unknown.

#### Comments.

The body is elongate and cylindrical. The metasoma is distinctively curved. The antenna is short and curled. In some females, the coloration of the hind femora and the hind tibiae is almost brown.

#### Male.

Similar in coloration to female. In some males, the body coloration is brown-red/reddish instead of black.

#### Etymology.

Luis (Lucho) Alberto Salagaje is one of the gusaneros who has assisted with caterpillar rearing at Yanayacu Biological Station.

#### Distribution.

Parasitized caterpillars were collected in Ecuador, Napo, Yanayacu Biological Station (Cascada San Rafael and Yanayacu Road), during May-June and September 2005, and November 2008 at 1,275 m and 2,100 m in cloud forest.

#### Biology.

The lifestyle of this parasitoid species is gregarious. *Mesochorus* sp. (Ichneumonidae: Mesochorinae) was reported as hyperparasitoid.

#### Host.

*Hypanartia* sp. Hübner (Nymphalidae: Nymphalinae) feeding on *Miriocarpa* sp. and undetermined species of Urticaceae. *Pseudautomerisyourii* Lemaire (Saturniidae: Hemileucinae) feeding on undetermined species of Melastomataceae. Undetermined species of Saturniidae feeding on *Boehmeriacaudate* (Urticaceae) and undetermined species of Nymphalidae feeding on *Boehmeriacaudate* and *Miriocarpa* sp. (Urticaceae). Caterpillars were collected at second and fourth instar.

### Glyptapanteles malleyneae

Taxon classificationAnimaliaHymenopteraBraconidae

Arias-Penna, sp. nov.

http://zoobank.org/25723078-831F-4DE0-987B-ECACB3123CF0

Fig. 145

#### Male.

Body length 3.88 mm, antenna length 3.68 mm, fore wing length 3.18 mm.

#### Type material.

**Holotype**: ECUADOR • 1♀; EC-1732, YY-A022; Napo, Yanayacu Biological Station, Sendero Pumayacu; cloud forest; 2,000 m; -0.6, -77.883333; 04.ii.2005; Lee Dyer leg.; cocoons formed on 26.ii.2005; adult parasitoids emerged on 12.iii.2005; (PUCE). **Paratypes.** • 5 (0♀, 3♂) (0♀, 2♂); EC-1732, YY-A022; same data as for holotype; (PUCE).

#### Diagnosis.

**Figure 145.**
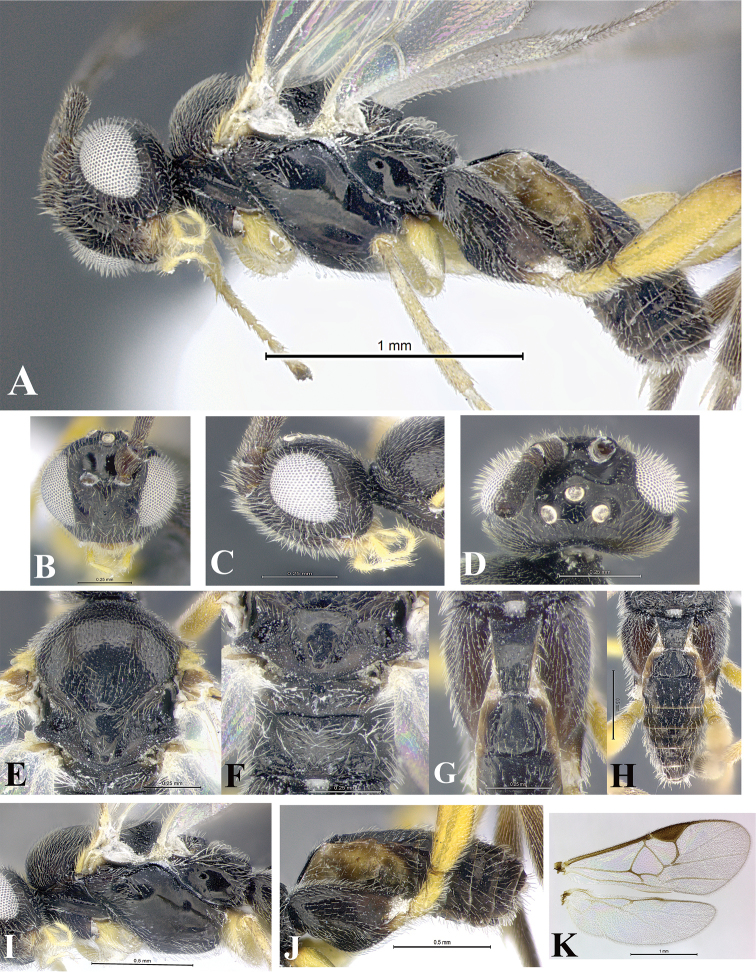
*Glyptapantelesmalleyneae* sp. nov. male EC-1732 YY-A022 **A** Habitus **B–D** Head **B** Frontal view **C** Lateral view **D** Dorsal view **E** Mesonotum, dorsal view **F** Scutellum, metanotum, propodeum, dorsal view **G**T1–2, dorsal view **H, J** Metasoma **H** Dorsal view **J** Lateral view **I** Mesosoma, lateral view **K** Fore and hind wings.

In lateral view, metasoma laterally compressed (Fig. 145A, J), hind coxa very finely punctate throughout (Fig. 145A, J), antenna shorter than body, scutellar punctation scattered throughout (Fig. 145E, F), edges of median area on T2 with little sculpture (Fig. 145G, H), petiole on T1 parallel-sided in proximal half and then narrowing (Fig. 145G, H), dorsal outer depression on hind coxa present (Fig. 145A, J), and fore wing with r vein curved, outer side of junction of r and 2RS veins forming a slight stub (Fig. 145K).

#### Coloration

(Fig. 145A–K). General body coloration polished black except scape, pedicel, and all antennal flagellomeres (on both sides) dark brown; mandibles yellow-brown; glossa, maxillary and labial palps, and tegulae yellow; clypeus, labrum, dorsal and ventral furrows of pronotum, epicnemial ridge, ventral edge of mesopleuron, lunules, BS, and PFM with brown-red/reddish tint. Eyes and ocelli silver. Fore and middle legs yellow except brown claws; hind legs yellow except black coxae only distally yellow (the yellow area is more extensive on the inner side), femora distally with a tiny brown spot, distal half of tibiae brown and proximally with a brown band, and tarsomeres brown. Petiole on T1 black and sublateral areas yellow; T2 with median and adjacent areas brown, adjacent area with contours well-defined, and narrow lateral ends yellow-brown; T3 and beyond completely brown; distally each tergum with a narrow yellow translucent band. In lateral view, T1–2 yellow-brown; T3 yellow-brown, but dorsally brown; T4 and beyond brown. S1–3 yellow-brown; S4 and beyond brown.

#### Description.

**Head** (Fig. 145A–D). Head rounded with pubescence long and dense. Proximal three antennal flagellomeres longer than wide (0.25:0.08, 0.25:0.08, 0.24:0.08), distal antennal flagellomere longer than penultimate (0.15:0.06, 0.12:0.06), antenna shorter than body (3.68, 3.88); antennal scrobes-frons shallow. Face with distal half dented only laterally, punctations barely noticeable, interspaces smooth and longitudinal median carina absent. Frons smooth. Temple wide, punctations barely noticeable and interspaces clearly smooth. Inner margin of eyes diverging slightly at antennal sockets; in lateral view, eye anteriorly convex and posteriorly straight. POL shorter than OOL (0.09, 0.11). Malar suture present. Median area between lateral ocelli slightly depressed. Vertex laterally pointed or nearly so and dorsally wide.

**Mesosoma** (Fig. 145A, E, F, I). Mesosoma dorsoventrally convex. Mesoscutum proximally convex and distally flat, punctation distinct throughout, interspaces wavy/lacunose. Scutellum long and slender, apex sloped and fused with BS, but not in the same plane, scutellar punctation scattered throughout, in profile scutellum flat and on same plane as mesoscutum, phragma of the scutellum partially exposed; BS only very partially overlapping the MPM; ATS demilune with complete undulate/reticulate carinae; dorsal ATS groove with carinae only proximally. Transscutal articulation with small and homogeneous foveae, area just behind transscutal articulation nearly at the same level as mesoscutum (flat) and with same kind of sculpture as mesoscutum. Metanotum with BM convex; MPM circular and bisected by a median longitudinal carina; AFM without setiferous lobes and not as well delineated as PFM; PFM thick and smooth; ATM proximally with a groove with some sculpturing and distally smooth. Propodeum with medium-sized punctation and without median longitudinal carina, proximal half weakly curved; distal edge of propodeum with a flange at each side and without stubs; propodeal spiracle without distal carina; nucha surrounded by very short radiating carinae. Pronotum with a distinct dorsal furrow, dorsally with a well-defined smooth band; central area of pronotum and dorsal furrow smooth, but ventral furrow with short parallel carinae. Propleuron with fine punctations throughout and dorsally without a carina. Metasternum convex. Contour of mesopleuron convex; precoxal groove smooth, shiny and shallow, but visible; epicnemial ridge convex, teardrop-shaped.

**Legs.** Ventral margin of fore telotarsus entire without seta, fore telotarsus almost same width throughout and longer than fourth tarsomere (0.12, 0.07). Hind coxa finely punctate throughout, and dorsal outer depression present. Inner spur of hind tibia longer than outer spur (0.25, 0.17), entire surface of hind tibia with dense strong spines clearly differentiated by color and length. Hind telotarsus longer than fourth tarsomere (0.15, 0.13).

**Wings** (Fig. 145K). Fore wing with r vein slightly curved; 2RS vein slightly concave, r and 2RS veins forming a weak, even curve at their junction and outer side of junction forming a slight stub; 2M vein slightly curved/swollen; distally fore wing [where spectral veins are] with microtrichiae more densely concentrated than the rest of the wing; anal cell 1/3 proximally lacking microtrichiae; subbasal cell with microtrichiae virtually throughout; vein 2CUa absent and vein 2CUb spectral; vein 2 cu-a absent; vein 2-1A proximally tubular and distally spectral, although sometimes difficult to see; tubular vein 1 cu-a curved, incomplete/broken and not reaching the edge of 1-1A vein. Hind wing with vannal lobe very narrow, subdistally and subproximally straightened, and setae evenly scattered in the margin.

**Metasoma** (Fig. 145A, G, H, J). Metasoma laterally compressed. Petiole on T1 with sculpture on distal half, virtually parallel-sided over most of length, but narrowing over distal 1/3, apex truncate (length 0.33, maximum width 0.17, minimum width 0.10), and with scattered pubescence on distal half. Lateral grooves delimiting the median area on T2 clearly defined and reaching the distal edge of T2 (length median area 0.18, length T2 0.18), edges of median area with little sculpture and lateral grooves deep, median area broader than long (length 0.18, maximum width 0.20, minimum width 0.10); T2 scarce pubescence throughout. T3 longer than T2 (0.22, 0.18) and with scattered pubescence throughout.

**Cocoons.** Unknown.

#### Comments.

The body is slim and covered by dense pubescence.

#### Female.

Unknown.

#### Etymology.

Marianne (M) Alleyne is a Dutch-born entomologist interested in the physiological mechanisms involved in determining host range of an insect parasitoid. Her studies have mostly focused on the immune system and the metabolic pathways of the host in response to parasitization. Currently, she is a research scientist at UIUC, IL, USA.

#### Distribution.

Parasitized caterpillar was collected in Ecuador, Napo, Yanayacu Biological Station (Sendero Pumayacu), during February 2005 at 2,000 m in cloud forest.

#### Biology.

The lifestyle of this parasitoid species is gregarious.

#### Host.

Undetermined species of Pyralidae feeding on undetermined species of Melastomataceae. Caterpillar instar was not reported.

### Glyptapanteles malloryvanwyngaardenae

Taxon classificationAnimaliaHymenopteraBraconidae

Arias-Penna, sp. nov.

http://zoobank.org/3B54B14F-36E9-4ACE-B5CF-187E799F43FA

Figs 146 , 147

#### Female.

Body length 2.47 mm, antenna length 2.78 mm, fore wing length 2.58 mm.

#### Type material.

**Holotype**: COSTA RICA • 1♀; 07-SRNP-60525, DHJPAR0020563; Área de Conservación Guanacaste, Guanacaste, Sector Mundo Nuevo, Vado Zanja Tapada; dry-rain intergrade forest; 550 m; 10.76480, -85.38445; 10.xi.2007; José Alberto Sánchez leg.; caterpillar collected in fourth instar; brown cordwood cocoons adhered to the leaf substrate, cocoons formed on 11.xi.2007; adult parasitoid emerged on 17.xi.2007; (CNC). **Paratypes.** • 9 (2♀, 0♂) (6♀, 1♂); 07-SRNP-60525, DHJPAR0020563; same data as for holotype; (CNC).

#### Other material.

**Reared material.** COSTA RICA: *Área de Conservación Guanacaste*, *Guanacaste*, *Sector Mundo Nuevo*, *Portón Rivas*: • 3 (1♀, 1♂) (0♀, 1♂); 05-SRNP-59661, DHJPAR0004227; dry-rain intergrade forest; 570 m; 10.75864, -85.37269; 30.viii.2005; Mariano Pereira leg.; caterpillar collected in fourth instar; large number of small white cocoons that were apparently adhered to the leaf substrate, cocoons formed on 09.ix.2005; adult parasitoid emerged on 10.ix.2005.

#### Diagnosis.

**Figure 146.**
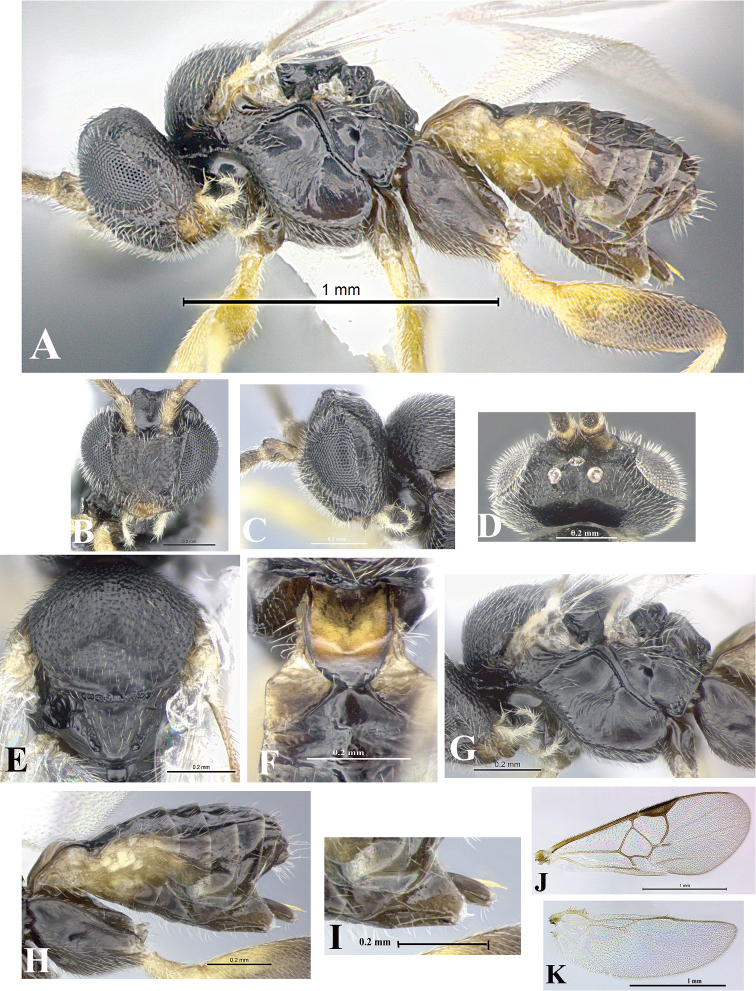
*Glyptapantelesmalloryvanwyngaardenae* sp. nov. female 05-SRNP-59661 DHJPAR0004227, 07-SRNP-60525 DHJPAR0020563 **A** Habitus **B–D** Head **B** Frontal view **C** Lateral view **D** Dorsal view **E** Mesonotum, dorsal view **F**T1–2, dorsal view **G** Mesosoma, lateral view **H** Metasoma, lateral view **I** Genitalia: hypopygium, ovipositor, ovipositor sheaths, lateral view **J, K** Wings **J** Fore **K** Hind.

**Figure 147.**
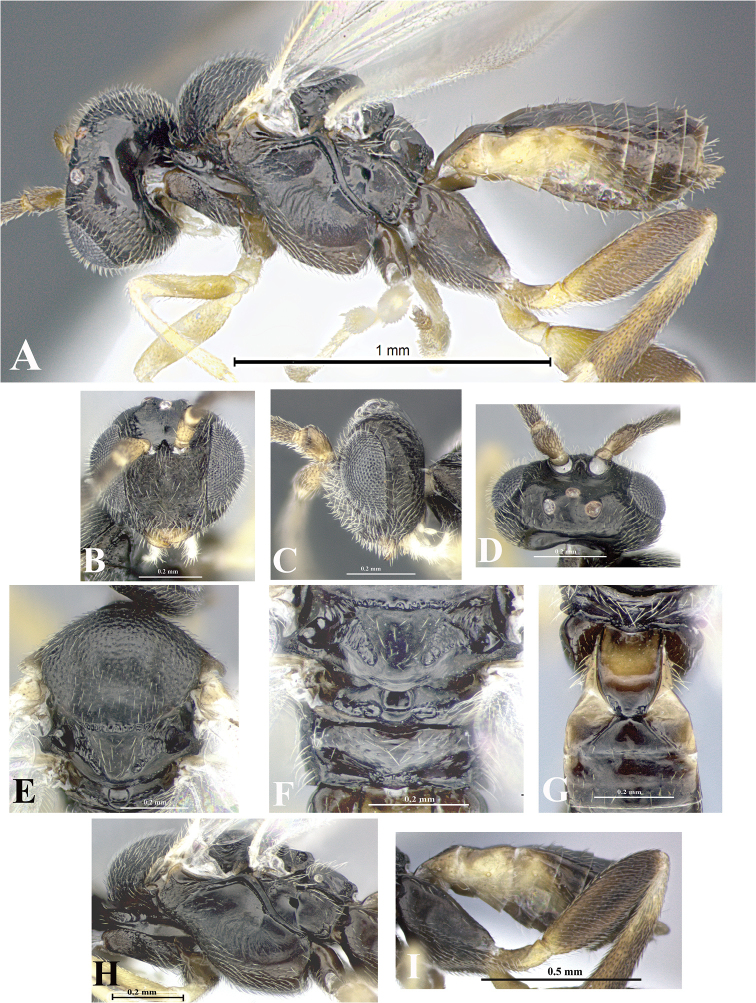
*Glyptapantelesmalloryvanwyngaardenae* sp. nov. male 05-SRNP-59661 DHJPAR0004227 **A** Habitus **B–D** Head **B** Frontal view **C** Lateral view **D** Dorsal view **E** Mesonotum, dorsal view **F** Scutellum, metanotum, propodeum, dorsal view **G**T1–3, dorsal view **H** Mesosoma, lateral view **I** Metasoma, lateral view.

Vertex in lateral view pointed or nearly so (Figs 146C, 147C), scutellum in profile slightly convex, but on same plane as mesoscutum (Figs 146G, 147H), scutellar punctation distinct throughout (Figs 146E, 147E, F), anteroventral contour of mesopleuron straight/angulate or nearly so (Figs 146A, G, 147A, H), distal antennal flagellomere subequal in length with penultimate, propodeal spiracle without distal carina (Fig. 147F), fore wing with 2RS slightly convex, outer side of junction of r and 2RS veins not forming a stub (Fig. 146J), and lateral grooves delimiting the median area on T2 distally losing definition (Fig. 146F, G).

#### Coloration

(Fig. 146A–K). General body coloration brown-black except scape and pedicel yellow; first three-four proximal antennal flagellomeres dorsally lighter (yellow-brown) than ventrally (brown), remaining flagellomeres brown on both sides; labrum and mandible yellow/yellow-brown; glossa, maxillary and labial palps, and tegulae yellow. Eyes gray and ocelli silver. Fore and middle legs yellow except brown coxae and brown claws; hind legs yellow except brown-black coxae, femora distally and ventrally brown, distal half of tibiae brown and tarsomeres brown, although basitarsus proximally with a narrow yellow band. Petiole on T1 with two colorations: proximal 3/4 reddish/yellow-brown and distal 1/4 brown, contours darkened and sublateral areas yellow with some brown tint; T2 with median and adjacent areas brown, and lateral ends yellow; T3 broadly brown except lateral ends yellow, thus brown coloration from T2–3 looks like a large pyramid-shaped; T4 mostly brown, but corners proximally yellow; T5 and beyond completely brown; distally each tergum with a narrow whitish translucent band. In lateral view, T1–2 yellow; T3 yellow, but dorsodistal corner brown; T4 and beyond brown. S1–2 yellow; S3 yellow, but medially with a longitudinal brown band; S4 and beyond brown.

#### Description.

**Head** (Fig. 146A–D). Head rhomboid with pubescence long and dense. Proximal three antennal flagellomeres longer than wide (0.21:0.07, 0.19:0.07, 0.20:0.07), distal antennal flagellomere subequal in length with penultimate (0.11:0.06, 0.10:0.06), antenna longer than body (2.78, 2.47); antennal scrobes-frons shallow. Face flat or nearly so, with dense fine punctations, interspaces with microsculpture and longitudinal median carina present. Frons smooth. Temple wide, punctate and interspaces wavy. Inner margin of eyes diverging slightly at antennal sockets; in lateral view, eye anteriorly convex and posteriorly straight. POL shorter than OOL (0.09, 0.12). Malar suture present. Median area between lateral ocelli slightly depressed. Vertex laterally pointed or nearly so and dorsally wide.

**Mesosoma** (Fig. 146A, E). Mesosoma dorsoventrally convex. Mesoscutum 1/4 distal with a central dent, punctation distinct throughout, interspaces with microsculpture. Scutellum triangular, apex sloped and fused with BS, scutellar punctation distinct throughout, in profile scutellum slightly convex, but on same plane as mesoscutum, phragma of the scutellum partially exposed; BS only very partially overlapping the MPM; ATS demilune with a little, complete and parallel carinae; dorsal ATS groove with semicircular/parallel carinae. Transscutal articulation with small and heterogeneous foveae, area just behind transscutal articulation smooth, shiny and nearly at the same level as mesoscutum (flat). Metanotum with BM wider than PFM (clearly differentiated); MPM circular without median longitudinal carina; AFM without setiferous lobes and not as well delineated as PFM; PFM thick, smooth and with lateral ends rounded; ATM proximally with a groove with some sculpturing and distally smooth. Propodeum without median longitudinal carina, proximal half curved with medium-sized sculpture and distal half relatively polished; distal edge of propodeum with a flange at each side and without stubs; propodeal spiracle without distal carina; nucha surrounded by very short radiating carinae. Pronotum with a distinct dorsal furrow, dorsally with a well-defined smooth band; central area of pronotum smooth, but both dorsal and ventral furrows with short parallel carinae. Propleuron with fine punctations throughout and dorsally with a carina. Metasternum flat or nearly so. Contour of mesopleuron straight/angulate or nearly so; precoxal groove deep with faintly transverse lineate sculpture; epicnemial ridge elongated more fusiform (tapering at both ends).

**Legs.** Ventral margin of fore telotarsus slightly excavated and with a tiny curved seta, fore telotarsus almost same width throughout and longer than fourth tarsomere (0.12, 0.06). Hind coxa with punctation only on ventral surface and dorsal outer depression present. Inner spur of hind tibia longer than outer spur (0.23, 0.16), entire surface of hind tibia with dense strong spines clearly differentiated by color and length. Hind telotarsus as equal in length as fourth tarsomere (0.10, 0.10).

**Wings** (Fig. 146J, K). Fore wing with r vein curved; 2RS vein slightly convex to convex; r and 2RS veins forming a weak, even curve at their junction and outer side of junction not forming a stub; 2M vein slightly curved/swollen; distally fore wing [where spectral veins are] with microtrichiae more densely concentrated than the rest of the wing; anal cell 1/3 proximally lacking microtrichiae; subbasal cell with a small smooth area, vein 2CUa absent and vein 2CUb spectral; vein 2 cu-a absent; vein 2-1A present only proximally as tubular vein; tubular vein 1 cu-a straight, incomplete/broken and not reaching the edge of 1-1A vein. Hind wing with vannal lobe narrow, subdistally straightened and subproximally straightened, and setae present only proximally.

**Metasoma** (Fig. 146A, H, I). Metasoma laterally compressed. Petiole on T1 finely sculptured only laterally, virtually parallel-sided over most of length, but narrowing over distal 1/3 (length 0.32, maximum width 0.18, minimum width 0.10), and with scattered pubescence concentrated in the first distal third. Lateral grooves delimiting the median area on T2 distally losing definition (length median area 0.10, length T2 0.15), edges of median area polished and lateral grooves deep, median area broader than long (length 0.10, maximum width 0.22, minimum width 0.08); T2 with scattered pubescence only distally. T3 longer than T2 (0.20, 0.15) and with scattered pubescence throughout. Pubescence on hypopygium dense.

**Cocoons.** White or brown oval cocoons with ordered silk fibers but covered by a net. Cordwood cocoons adhered to the leaf substrate.

#### Comments.

In some females, the coloration on S1–2 is completely light yellow-brown, S3 and beyond, including the hypopygium are completely brown. The median longitudinal carina on the face is short. The body is short and stout.

#### Male

(Fig. 147A–I). Coloration and body shape similar to female. The fore telotarsus with ventral margin even instead of excavated and seta right instead of curved.

#### Etymology.

Mallory Van Wyngaarden worked from 2010 to 2013 as a data manager in the Barcode of Life Data Systems. Currently, she works at the Institute of Infection, Immunity and Inflammation, University of Glasgow, United Kingdom.

#### Distribution.

The parasitized caterpillars were collected in Costa Rica, ACG, Sector Mundo Nuevo (Portón Rivas and Vado Zanja Tapada), during August 2005 and November 2007 at 550 m and 570 m in dry-rain intergrade forest.

#### Biology.

The lifestyle of this parasitoid species is gregarious.

#### Host.

*Rifargiaelgiva* Schaus (Notodontidae: Heterocampinae) feeding on *Styraxargenteus* (Styracaceae). Caterpillars were collected in fourth instar.

### Glyptapanteles mamiae

Taxon classificationAnimaliaHymenopteraBraconidae

Arias-Penna, sp. nov.

http://zoobank.org/A690CB56-EA9C-47A2-AEF9-F474255D3C34

Fig. 148

#### Female.

Body length 3.13 mm, antenna length 3.28 mm, fore wing length 3.18 mm.

#### Type material.

**Holotype**: ECUADOR • 1♀; EC-10806, YY-A029; Napo, Yanayacu Biological Station, Río Chalpi, Plot 144; cloud forest; 2,847 m; -0.366667, -78.083333; 16.xii.2005; Wilmer Simbaña leg.; caterpillar collected in third instar; cocoons formed on 22.i.2006; adult parasitoids emerged on 30.i.2006; (PUCE). **Paratype.** • 1 (0♀, 1♂) (0♀, 0♂); EC-10806, YY-A029; same data as for holotype; (PUCE).

#### Other material.

**Reared material.** ECUADOR: *Napo*, *Yanayacu Biological Station*, *Río Chalpi*, *Plot 144*: • 7 (3♀, 2♂) (1♀, 1♂); EC-10803, YY-A028, YY-A036; cloud forest; 2,847 m; -0.366667, -78.083333; 16.xii.2005; Wilmer Simbaña leg.; caterpillar collected in third instar; cocoons formed on 19.i.2006; adult parasitoids emerged on 01.ii.2006. • 7 (3♀, 3♂) (0♀, 1♂); EC-10804, YY-A034; same data as for preceding except: cocoons formed on 22.i.2006; adult parasitoids emerged on 29.i.2006. • 1 (0♀, 1♂) (0♀, 0♂); EC-10805, YY-A150; same data as for preceding except: cocoons formed on 22.i.2006; adult parasitoids emerged on 30.i.2006. • 25 (6♀, 6♂) (13♀, 0♂); EC-10808, YY-A039; same data as for preceding except: cocoons formed on 22.i.2006; adult parasitoids emerged on 30.i.2006. • 18 (6♀, 6♂) (6♀, 0♂); EC-10809, YY-A048; same data as for preceding except: cocoons formed on 22.i.2006; adult parasitoids emerged on 28.i.2006. • 13 (5♀, 5♂) (3♀, 0♂); EC-10810, YY-A035; same data as for preceding except: cocoons formed on 22.i.2006; adult parasitoids emerged on 26.i.2006.

*Napo*, *Yanayacu Biological Station*, *Río Chalpi Grande*, *Plot 211*: • 3 (1♀, 1♂) (0♀, 1♂); EC-14121, YY-A088; cloud forest; 2,777 m; -0.35, -78.083333; 24.iv.2006; Rafael Granizo leg.; caterpillar collected in late instar or prepupa; cocoons formed on 26.iv.2006; adult parasitoids emerged on 11.v.2006.

*Napo*, *Yanayacu Biological Station*, *Sendero Macuculoma*, *Plot 415*: • 18 (6♀, 2♂) (10♀, 0♂); EC-36447, YY-A070; cloud forest; 2,120 m; -0.601111, -77.883889; 05.i.2009; Wilmer Simbaña leg.; caterpillar collected in second instar; cocoons formed on 30.i.2009; adult parasitoids emerged on 16.ii.2009.

#### Diagnosis.

**Figure 148.**
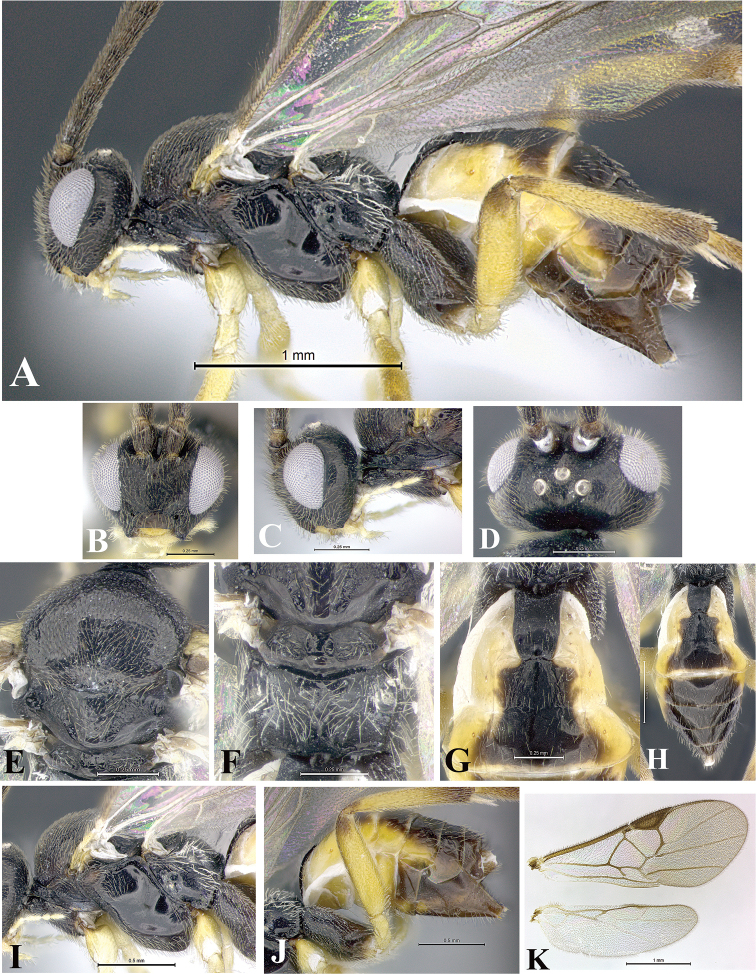
*Glyptapantelesmamiae* female sp. nov. EC-10806 YY-A029 **A** Habitus **B, D** Head **B** Frontal view **D** Dorsal view **C** Head, pronotum lateral view **E** Mesonotum, dorsal view **F** Scutellum, metanotum, propodeum, dorsal view **G**T1–3, dorsal view **H, J** Metasoma **H** Dorsal view **J** Lateral view **I** Mesosoma, lateral view **K** Fore and hind wings.

In lateral view, metasoma curved (Fig. 148A, J), hind coxa very finely punctate throughout and dorsal outer depression present (Fig. 148A, J), propodeum without a transverse discontinuous carina (Fig. 148F), petiole on T1 virtually parallel-sided, but narrowing over distal 1/3 (Fig. 148G, H), scutellar punctation scattered throughout (Fig. 148E), edges of median area on T2 obscured by weak longitudinal stripes (Fig. 148G, H), and fore wing with r vein slightly curved, outer side of junction of r and 2RS veins forming a distinct stub (Fig. 148K).

#### Coloration

(Fig. 148A–K). General body coloration polished black except scape brown with apex yellow-brown; pedicel and all antennal flagellomeres (on both sides) dark brown; labrum, mandibles, glossa, maxillary and labial palps, and tegulae yellow; clypeus, propleuron, both dorsal and ventral furrows of pronotum, epicnemial ridge, ventral edge of mesopleuron, metasternum, distal corners of mesoscutum, BM, and PFM with brown-red/reddish tint. Eyes and ocelli silver. Fore legs yellow except brown claws; middle legs yellow except femora, tibiae and tarsomeres with a narrow dorsal brown strip from top to bottom; hind legs yellow except black coxae with yellow/brown-red apex (brown-red coloration extended in the inner side), femora with a tiny brown spot in the apex and with a narrow dorsal brown strip from top to bottom, apex of tibiae brown and proximally with a brown band, and tarsomeres brown. Petiole on T1 black and sublateral areas yellow; T2 with median and adjacent areas black, adjacent area with contours well-defined, and lateral ends yellow; T3 mostly brown, proximal width of dark area coincides with the width of median and adjacent areas on T2, although distally dark area of T3 wider than proximal, and lateral ends yellow; T4 and beyond brown; distally each tergum with a narrow yellow translucent band. In lateral view, T1–2 yellow; T3–5 yellow, but dorsally brown, remaining tergites completely brown. S1–3 yellow; S4 yellow, but medially brown; penultimate sternum and hypopygium brown, but medially with some parts yellow-brown.

#### Description.

**Head** (Fig. 148A–D). Head rhomboid with pubescence long and dense. Proximal three antennal flagellomeres longer than wide (0.22:0.08, 0.25:0.08, 0.23:0.08), distal antennal flagellomere longer than penultimate (0.13:0.06, 0.11:0.06), antenna longer than body (3.28, 3.13); antennal scrobes-frons shallow. Face with distal half dented only laterally with dense fine punctations, interspaces smooth and longitudinal median carina present. Frons smooth. Temple wide, punctate and interspaces clearly smooth. Inner margin of eyes diverging slightly at antennal sockets; in lateral view, eye anteriorly convex and posteriorly straight. POL shorter than OOL (0.11, 0.13). Malar suture absent or difficult to see. Median area between lateral ocelli slightly depressed. Vertex laterally pointed or nearly so and dorsally wide.

**Mesosoma** (Fig. 148A, E, F, I). Mesosoma dorsoventrally convex. Mesoscutum 1/4 distal with a central dent, punctation distinct throughout, interspaces smooth. Scutellum triangular, apex sloped and fused with BS, but not in the same plane, scutellar punctation scattered throughout, in profile scutellum flat and on same plane as mesoscutum, phragma of the scutellum partially exposed; BS only very partially overlapping the MPM; ATS demilune inner side with a row of foveae; dorsal ATS groove with carinae only proximally. Transscutal articulation with large and heterogeneous foveae, area just behind transscutal articulation depressed centrally and with same kind of sculpture as mesoscutum. Metanotum with BM convex; MPM oval/circular with a short proximal carina; AFM without setiferous lobes and not as well delineated as PFM; PFM thick and smooth; ATM proximally with a groove with some sculpturing and distally smooth. Propodeum relatively polished with a median longitudinal dent, but no trace of median longitudinal carina, proximal half curved; distal edge of propodeum with a flange at each side and without stubs; propodeal spiracle without distal carina; nucha surrounded by very short radiating carinae. Pronotum with a distinct dorsal furrow, dorsally with a well-defined smooth band; central area of pronotum smooth, but both dorsal and ventral furrows with short parallel carinae. Propleuron finely sculptured only ventrally and dorsally without a carina. Metasternum convex. Contour of mesopleuron convex; precoxal groove smooth, shiny and shallow, but visible; epicnemial ridge elongated more fusiform (tapering at both ends).

**Legs.** Ventral margin of fore telotarsus entire without seta, fore telotarsus almost same width throughout and longer than fourth tarsomere (0.15, 0.07). Hind coxa finely punctate throughout, and dorsal outer depression present. Inner spur of hind tibia longer than outer spur (0.25, 0.18), entire surface of hind tibia with dense strong spines clearly differentiated by color and length. Hind telotarsus longer than fourth tarsomere (0.18, 0.13).

**Wings** (Fig. 148K). Fore wing with r vein slightly curved; 2RS vein slightly convex to convex; r and 2RS veins forming a weak, even curve at their junction and outer side of junction forming a distinct stub; 2M vein slightly curved/swollen; distally fore wing [where spectral veins are] with microtrichiae more densely concentrated than the rest of the wing; anal cell 1/3 proximally lacking microtrichiae; subbasal cell with microtrichiae virtually throughout; veins 2CUa and 2CUb completely spectral; vein 2 cu-a present as spectral vein, sometimes difficult to see; vein 2-1A proximally tubular and distally spectral, although sometimes difficult to see; tubular vein 1 cu-a vein straight and complete, but junction with 1-1A vein spectral. Hind wing with vannal lobe very narrow, subdistally and subproximally straightened, and setae absent proximally, but scattered distally.

**Metasoma** (Fig. 148A, G, H, J). Metasoma curved. Petiole on T1 finely sculptured distally, but only laterally, virtually parallel-sided over most of length, but barely narrowing at apex, apex truncate (length 0.38, maximum width 0.23, minimum width 0.14), and with scattered pubescence concentrated in the first distal third. Lateral grooves delimiting the median area on T2 clearly defined and reaching the distal edge of T2 (length median area 0.21, length T2 0.21), edges of median area obscured by weak longitudinal stripes, median area broader than long (length 0.21, maximum width 0.30, minimum width 0.13); T2 scarce pubescence throughout. T3 longer than T2 (0.29, 0.21) and with scattered pubescence throughout. Pubescence on hypopygium dense.

**Cocoons.** White or beige oval cocoons with messy/disordered/fluffy silk fibers and body of cocoon with disorganized and tangled silk.

#### Comments.

The limit between mesopleuron and metasternum with a dented area.

#### Male.

Coloration similar to females although darker. In some males, the femora, the tibiae and the tarsomeres of all legs with a narrow dorsal strip from top to bottom.

#### Etymology.

Maminirina (Mami) Randrianandrasana is a Malagasy entomologist. As graduate student at UIUC, IL, USA, she worked in ecology of wild silkworms, mainly *Antherinasuraka* (Boisduval) (Saturniidae), using its natural history for conservation in Madagascar.

#### Distribution.

Parasitized caterpillars were collected in Ecuador, ACG, Yanayacu Biological Station (Río Chalpi and Sendero Macuculoma), during December 2005, April 2006, and January 2009 at 2,120, 2,777 m, and 2,847 m in cloud forest.

#### Biology.

The lifestyle of this parasitoid species is solitary/gregarious.

#### Host.

Undetermined species of Erebidae (Arctiinae) feeding on *Miconia* sp. (Melastomataceae) and *Chusqueascandens* (Poaceae). Caterpillars were collected in second, third and late instar or prepupa.

### Glyptapanteles marcelotavaresi

Taxon classificationAnimaliaHymenopteraBraconidae

Arias-Penna, sp. nov.

http://zoobank.org/C028D9C0-BCDC-44A8-ADD9-38C89550F0B0

Fig. 149

#### Female.

Body length 2.58 mm, antenna length 3.13 mm, fore wing length 3.03 mm.

#### Type material.

**Holotype**: ECUADOR • 1♀; EC-39782, YY-A010; Napo, Yanayacu Biological Station, Sierra Azul; 2,250 m; -0.666667, -77.947778; 06.vii.2009; CAPEA leg.; caterpillar collected in third instar; cocoons formed on 15.vii.2009; adult parasitoids emerged on 04.viii.2009; (PUCE). **Paratypes.** • 3 (1♀, 1♂) (1♀, 0♂); EC-39782, YY-A010; same data as for holotype; (PUCE).

#### Diagnosis.

**Figure 149.**
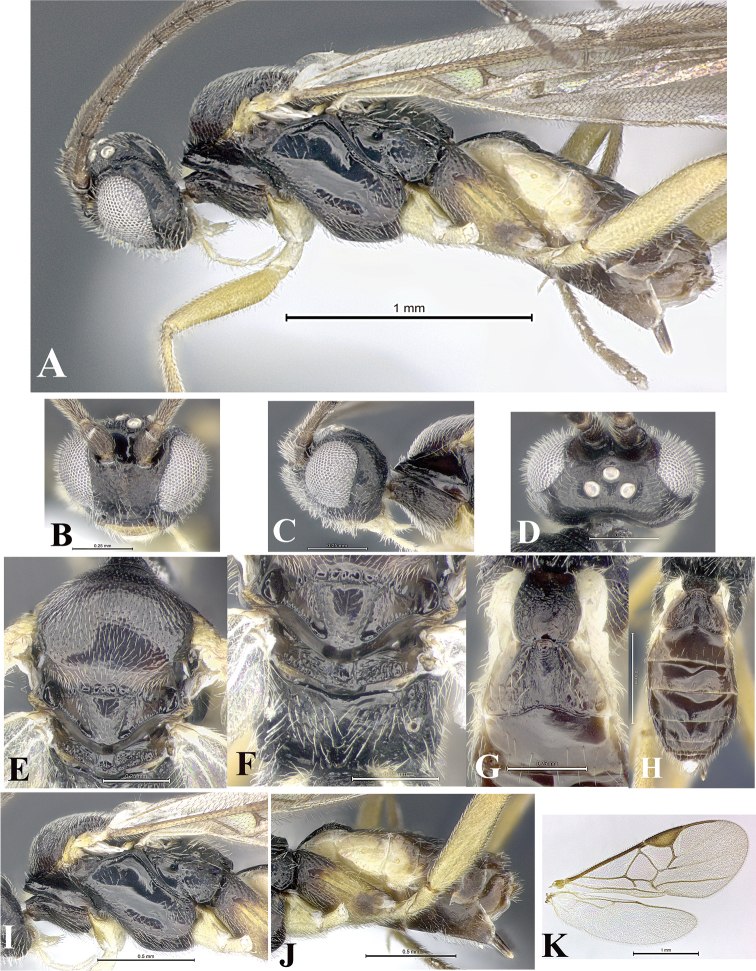
*Glyptapantelesmarcelotavaresi* sp. nov. female EC-39782 YY-A010 **A** Habitus **B, D** Head **B** Frontal view **D** Dorsal view **C** Head, pronotum, propleuron, lateral view **E** Mesonotum, dorsal view **F** Scutellum, metanotum, propodeum, dorsal view **G**T1–3, dorsal view **H, J** Metasoma **H** Dorsal view **J** Lateral view **I** Mesosoma, lateral view **K** Fore and hind wings.

Petiole on T1, proximal half straight and distal half convex (Fig. 149G), distal edge on T2 slightly convex (Fig. 149G, H), lateral grooves delimiting the median area on T2 distally losing definition (Fig. 149G), edges of median area on T2 obscured by strong longitudinal stripes (Fig. 149G, H), T3 as long as T2 (Fig. 149H), distal antennal flagellomere subequal in length with penultimate, mesoscutum punctation proximally distinct, but distally absent/dispersed (Fig. 149E), in lateral view, metasoma laterally compressed (Fig. 149A, J), dorsal outer depression on hind coxa present (Fig. 149A, J), and fore wing with r vein slightly curved, outer side of junction of r and 2RS veins forming a distinct stub (Fig. 149K).

#### Coloration

(Fig. 149A–K). General body coloration polished black except scape proximally yellow-brown/reddish and distally brown; pedicel distally yellow-brown/reddish and proximally brown; all antennal flagellomeres dorsally lighter (light brown) than ventrally (dark brown); labrum, mandible, and tegulae dark yellow; dorsal area of low face just down between antennal scrobes, clypeus, propleuron, both ventral and dorsal furrows of pronotum, epicnemial ridge, ventral edge of mesopleuron, metasternum, distal corners of mesoscutum, dorsally ATS grove, ATS demilune, lunules, BS, and PFM with brown-red/reddish tints; glossa, maxillary and labial palps yellow. Eyes and ocelli silver. Fore and middle legs dark yellow, except tibiae and tarsomeres with a narrow dorsal strip from top to bottom; hind legs dark yellow except coxae proximally brown/brown-reddish and distally with a brown spot, femora distally with a tiny brown spot, tibiae and tarsomeres brown. Petiole on T1 brown with contours darkened, and sublateral areas yellow; T2 with median area brown and lateral areas light brown; T3 and beyond completely brown; distally each tergum with a very narrow yellow translucent band. In lateral view, T1–3 yellow; T4 and beyond brown. S1–2 yellow; S3–4 yellow, but medially brown; penultimate sternum and hypopygium brown.

#### Description.

**Head** (Fig. 149A–D). Head rounded with pubescence long and dense. Proximal three antennal flagellomeres longer than wide (0.20:0.06, 0.20:0.06, 0.20:0.06), distal antennal flagellomere subequal in length with penultimate (0.13:0.05, 0.12:0.06), antenna longer than body (3.13, 2.58); antennal scrobes-frons sloped and forming a shelf. Face convex, punctate-lacunose, interspaces wavy and longitudinal median carina present. Frons smooth. Temple wide, punctations barely noticeable and interspaces clearly smooth. Inner margin of eyes diverging slightly at antennal sockets; in lateral view, eye anteriorly convex and posteriorly straight. POL shorter than OOL (0.07, 0.10). Malar suture absent or difficult to see. Median area between lateral ocelli slightly depressed. Vertex laterally rounded and dorsally wide.

**Mesosoma** (Fig. 149A, E, F, I). Mesosoma dorsoventrally convex. Mesoscutum proximally convex and distally flat, punctation distinct proximally, but absent/dispersed distally, interspaces smooth. Scutellum triangular, apex sloped and fused with BS, but not in the same plane, scutellar punctation scattered throughout, in profile scutellum convex and slightly higher than mesoscutum, phragma of the scutellum partially exposed; BS only very partially overlapping the MPM; ATS demilune with short stubs delineating the area; dorsal ATS groove with semicircular/parallel carinae. Transscutal articulation with large and heterogeneous foveae, area just behind transscutal articulation sloped and with same kind of sculpture as mesoscutum. Metanotum with BM convex; MPM circular without median longitudinal carina; AFM with a small lobe and not as well delineated as PFM; PFM thick, smooth and with lateral ends rounded; ATM proximally sculptured and distally without a well delimited smooth area; propodeum without median longitudinal carina, proximal half curved with medium-sized sculpture and distal half relatively polished and with a shallow dent at each side of nucha; distal edge of propodeum with a flange at each side and without stubs, propodeal spiracle without distal carina; nucha surrounded by long radiating carinae. Pronotum virtually without trace of dorsal furrow, dorsally without a smooth band; central area of pronotum and ventral furrow smooth. Propleuron finely sculptured only ventrally and dorsally without a carina. Metasternum convex. Contour of mesopleuron straight/angulate or nearly so; precoxal groove smooth, shiny and shallow, but visible; epicnemial ridge convex, teardrop-shaped.

**Legs.** Ventral margin of fore telotarsus entire without seta, fore telotarsus almost same width throughout and longer than fourth tarsomere (0.15, 0.07). Medially hind coxa smooth, dorsally with scattered punctation and ventrally with dense punctation, dorsal outer depression present. Inner spur of hind tibia longer than outer spur (0.22, 0.16), entire surface of hind tibia with dense strong spines clearly differentiated by color and length. Hind telotarsus as equal in length as fourth tarsomere (0.14, 0.13).

**Wings** (Fig. 149K). Fore wing with r vein slightly curved; 2RS vein straight; r and 2RS veins forming an angle at their junction and outer side of junction forming a distinct stub; 2M vein straight; distally fore wing [where spectral veins are] with microtrichiae more densely concentrated than the rest of the wing; anal cell 1/3 proximally lacking microtrichiae; subbasal cell with microtrichiae virtually throughout; veins 2CUa and 2CUb completely spectral; vein 2 cu-a present as spectral vein, sometimes difficult to see; vein 2-1A proximally tubular and distally spectral, although sometimes difficult to see; tubular vein 1 cu-a straight, incomplete/broken and not reaching the edge of 1-1A vein. Hind wing with vannal lobe very narrow, subdistally and subproximally straightened, and setae evenly scattered in the margin.

**Metasoma** (Fig. 149A, G, H, J). Metasoma laterally compressed. Petiole on T1 with rugae all over the surface, virtually parallel-sided over most of length, but barely narrowing at apex, apex truncate (length 0.35, maximum width 0.19, minimum width 0.14), and with scattered pubescence concentrated in the first distal third. Lateral grooves delimiting the median area on T2 distally losing definition (length median area 0.14, length T2 0.19), edges of median area obscured by strong longitudinal stripes, median area broader than long (length 0.14, maximum width 0.25, minimum width 0.10); T2 with scattered pubescence only distally. T3 as long as T2 (0.18, 0.19) and with pubescence more notorious in distal half. Pubescence on hypopygium dense.

**Cocoons.** Unknown.

#### Comments.

Contours of the petiole with proximal half straight and distal half convex (Fig. 149G, H); the distal edge on T2 slightly convex (Fig. 149G, H); the median area is similar to *Austrocotesia* Austin & Dangerfield; the limit between the mesopleuron and the metasternum is flattened/dented; in lateral view, the eyes cover a large area of the head (Fig. 149C); the body is slim and elongated.

#### Male.

Similar in coloration to female.

#### Etymology.

Marcelo Teixeira Tavares works on the taxonomy, systematics, biology, and ecology of parasitoid wasps (Hymenoptera), with an emphasis on Chalcididae. Currently, he works at the Universidade Federal do Espirito Santo (UFES), Vitória, Brazil.

#### Distribution.

Parasitized caterpillar was collected in Ecuador, Napo, Yanayacu Biological Station (Sierra Azul) during July 2009 at 2,250 m in cloud forest.

#### Biology.

The lifestyle of this parasitoid species is gregarious.

#### Host.

Undetermined species of Erebidae (Arctiinae) feeding on *Monninasubspeciosa* (Polygalaceae). Caterpillar was collected in third instar.

### Glyptapanteles marcepsteini

Taxon classificationAnimaliaHymenopteraBraconidae

Arias-Penna, sp. nov.

http://zoobank.org/94F80DBF-BB3A-4C5E-BB64-4DE2746C4F43

Fig. 150

#### Female.

Body length 2.68 mm, antenna length 3.48 mm, fore wing length 3.43 mm.

#### Type material.

**Holotype**: ECUADOR • 1♀; EC-42101, YY-A123; Napo, Yanayacu Biological Station, Stream trail, Plot 442; cloud forest; 2,444 m; -0.6015, -77.886444; 09.ix.2009; Luis Salagaje leg.; caterpillar collected in second instar; cocoon formed on 16.ix.2009; adult parasitoid emerged on 08.x.2009; (PUCE).

#### Diagnosis.

**Figure 150.**
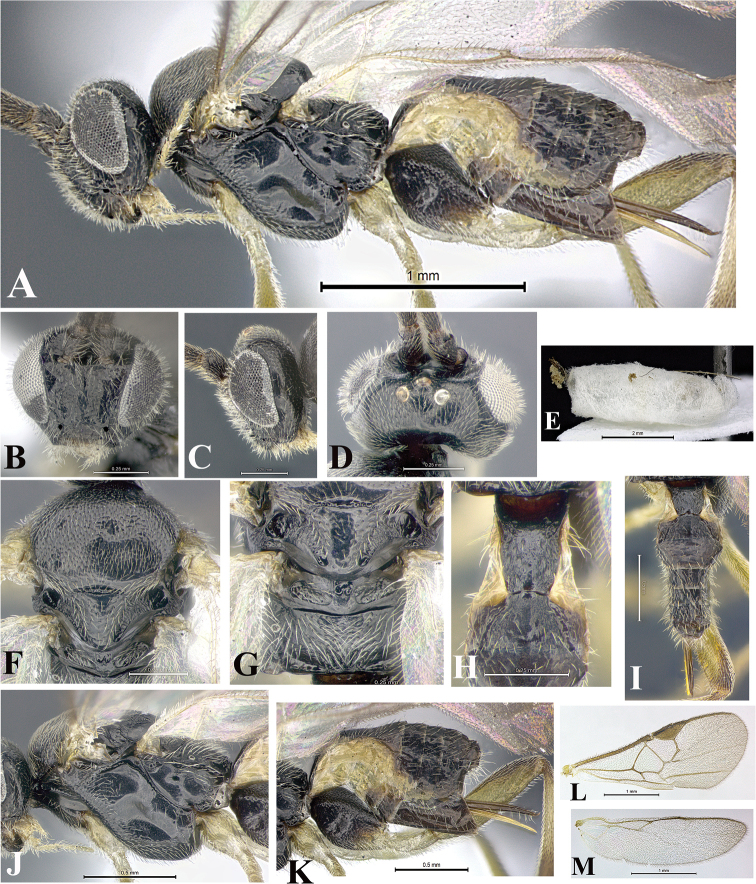
*Glyptapantelesmarcepsteini* sp. nov. female EC-42101 YY-A123 **A** Habitus **B–D** Head **B** Frontal view **C** Lateral view **D** Dorsal view **E** Cocoon **F** Mesonotum, dorsal view **G** Scutellum, metanotum, propodeum, dorsal view **H**T1–2, dorsal view **I, K** Metasoma **I** Dorsal view **K** Lateral view **J** Mesosoma, lateral view **L, M** Wings **L** Fore **M** Hind.

Malar suture absent or difficult to see (Fig. 150B), median area between lateral ocelli slightly depressed (Fig. 150D), propodeum with a median longitudinal dent, but no trace of median longitudinal carina (Fig. 150G), scutellar punctation distinct throughout (Fig. 150F, G), axillary trough of metanotum proximally with sculpture, but dorsally without a well delimited smooth area (Fig. 150F, G), anterior furrow of metanotum with a small lobe without setae (Fig. 150G), petiole on T1 parallel-sided in proximal half, then narrowing (Fig. 150H), edges of median area on T2 obscured by weak longitudinal stripes (Fig. 150H, I), dorsal outer depression on hind coxa present, and fore wing with r vein curved, outer side of junction of r and 2RS veins forming a distinct stub (Fig. 150L).

#### Coloration

(Fig. 150A–M). General body coloration black except scape and all antennal flagellomeres (on both sides) brown; pedicel brown, but distally brown-red/reddish; labrum, mandibles, propleuron apically with a small spot, and dorsal furrow of pronotum with brown-red/reddish tints; glossa, maxillary and labial palps, and tegulae light yellow-brown. Eyes silver and ocelli reddish/silver (in preserved specimen). Fore and middle legs light yellow-brown although coloration from tibiae to tarsomeres more intense, and claws brown, both femora and tibiae of middle legs with a lighter narrow dorsal brown strip from top to bottom; hind legs yellow-brown except black coxae distally yellow, femora distally with a small brown spot, additionally with a narrow, dorsal, light brown strip from top to bottom, distal half of tibiae brown and proximally with a brown ring, and tarsomeres brown. Petiole on T1 dark brown and sublateral areas yellow-brown; T2 with median and adjacent areas dark brown, and narrow lateral ends yellow-brown; T3 and beyond complete dark brown; distally each tergum with a narrow whitish transparent band. In lateral view, T1–2 yellow; T3–4 yellow, but dorsally brown, the extent of brown area increasing from proximal to distal; T5 and beyond completely brown. S1–3 yellow; S4–5 brown, but distally with a narrow yellow band; hypopygium brown.

#### Description.

**Head** (Fig. 150A–D). Head rounded with pubescence long and dense. Proximal three antennal flagellomeres longer than wide (0.25:0.08, 0.25:0.08, 0.25:0.08), distal antennal flagellomere longer than penultimate (0.14:0.07, 0.10:0.07), antenna longer than body (3.48, 2.68); antennal scrobes-frons sloped and forming a shelf. Face with depression only laterally, dense fine punctations, interspaces smooth and longitudinal median carina present. Frons smooth. Temple wide, punctations barely noticeable and interspaces clearly smooth. Inner margin of eyes diverging slightly at antennal sockets; in lateral view, eye anteriorly convex and posteriorly straight. POL shorter than OOL (0.10, 0.20). Malar suture absent or difficult to see. Median area between lateral ocelli slightly depressed. Vertex laterally rounded and dorsally wide.

**Mesosoma** (Fig. 150A, F, G, J). Mesosoma dorsoventrally convex. Distal 1/3 of mesoscutum with lateral margin slightly dented, punctation distinct throughout, interspaces smooth. Scutellum long and slender, apex sloped and fused with BS, but not in the same plane, scutellar punctation distinct throughout, in profile scutellum flat and on same plane as mesoscutum, phragma of the scutellum partially exposed; BS only very partially overlapping the MPM; ATS demilune inner side with a row of foveae; dorsal ATS groove with carinae only proximally. Transscutal articulation with small and homogeneous foveae, area just behind transscutal articulation with a sloped transverse strip and with same kind of sculpture as mesoscutum. Metanotum with BM wider than PFM (clearly differentiated); MPM oval/circular with a short proximal carina; AFM with a small lobe and not as well delineated as PFM; PFM thick, smooth and with lateral ends rounded; ATM proximally with sculpture distally without a well delimited smooth area. Propodeum with a median longitudinal dent, but no trace of median longitudinal carina, proximal half curved with medium-sized sculpture and distal half with medium-sized punctation; distal edge of propodeum with a flange at each side and without stubs; propodeal spiracle without distal carina; nucha surrounded by long radiating carinae. Pronotum with a distinct dorsal furrow, dorsally with a well-defined smooth band; central area of pronotum and dorsal furrow smooth, but ventral furrow with short parallel carinae. Propleuron with fine punctations throughout and dorsally without a carina. Metasternum convex. Contour of mesopleuron convex; precoxal groove deep, smooth and shiny; epicnemial ridge elongated more fusiform (tapering at both ends).

**Legs.** Ventral margin of fore telotarsus slightly excavated and with a tiny curved seta, fore telotarsus proximally narrow and distally wide, and longer than fourth tarsomere (0.15, 0.10). Hind coxa with medium-size punctate throughout and dorsal outer depression present. Inner spur of hind tibia longer than outer spur (0.27, 0.23), entire surface of hind tibia with dense strong spines clearly differentiated by color and length. Hind telotarsus longer than fourth tarsomere (0.20, 0.14).

**Wings** (Fig. 150L, M). Fore wing with r vein slightly curved; 2RS vein straight; r and 2RS veins forming an angle at their junction and outer side of junction forming a distinct stub; 2M vein straight; distally fore wing [where spectral veins are] with microtrichiae more densely concentrated than the rest of the wing; anal cell 1/3 proximally lacking microtrichiae; subbasal cell with microtrichiae virtually throughout; veins 2CUa and 2CUb completely spectral; vein 2 cu-a present as spectral vein, sometimes difficult to see; vein 2-1A proximally tubular and distally spectral, although sometimes difficult to see; tubular vein 1 cu- a straight, incomplete/broken and not reaching the edge of 1-1A vein. Hind wing with vannal lobe very narrow, subdistally and subproximally straightened, and setae absent proximally but scattered distally.

**Metasoma** (Fig. 150A, H, I, K). Metasoma laterally compressed. Petiole on T1 with a mix of fine rugae and punctate sculpture over most of the surface, parallel-sided in proximal half and then narrowing (length 0.38, maximum width 0.20, minimum width 0.12), and with scattered pubescence on distal half. Lateral grooves delimiting the median area on T2 clearly defined and reaching the distal edge of T2 (length median area 0.18, length T2 0.18), edges of median area obscured by weak longitudinal stripes, median area broader than long (length 0.18, maximum width 0.24, minimum width 0.10); T2 scarce pubescence throughout. T3 longer than T2 (0.23, 0.18) and with scattered pubescence throughout. Pubescence on hypopygium dense.

**Cocoon** (Figs 4C, 150E). White oval cocoon with evenly smooth silk fibers.

#### Comments.

The antenna is curled; the ovipositor is large as in *Sathon* and with pubescence distally (Fig. 150A, K).

#### Male.

Unknown.

#### Etymology.

Marc E. Epstein is the Senior Insect Biosystematist for Lepidoptera at the California Department of Food & Agriculture, Plant Pest Diagnostics Branch, Sacramento, CA, USA. He is a specialist in Limacodidae and related families (Zygaenoidea).

#### Distribution.

Parasitized caterpillar was collected in Ecuador, Napo, Yanayacu Biological Station (Stream trail), during September 2009 at 2,444 m in cloud forest.

#### Biology.

The lifestyle of this parasitoid species is solitary.

#### Host.

Undetermined species of Pyralidae feeding on Diplaziumcostalevar.robustum (Dryopteridaceae). Caterpillar was collected in second instar.

### Glyptapanteles marcpolleti

Taxon classificationAnimaliaHymenopteraBraconidae

Arias-Penna, sp. nov.

http://zoobank.org/5111CB61-0EB5-4284-A3ED-5904EF420AAC

Fig. 151

#### Male.

Body length 2.53 mm, antenna length 3.58 mm, fore wing length 3.0 mm.

#### Type material.

**Holotype**: ECUADOR• 1♀; EC-37478, YY-A167; Napo, Yanayacu Biological Station, Yanayacu Road; cloud forest; 2,100 m; -0.566667, -77.866667; 10.iii.2009; CAPEA leg.; caterpillar collected in fourth instar; cocoon formed on 19.iii.2009; adult parasitoid emerged on 18.iv.2009; *Mesochorus* (Ichneumonidae: Mesochorinae) was reported as hyperparasitoid; (PUCE).

#### Diagnosis.

**Figure 151.**
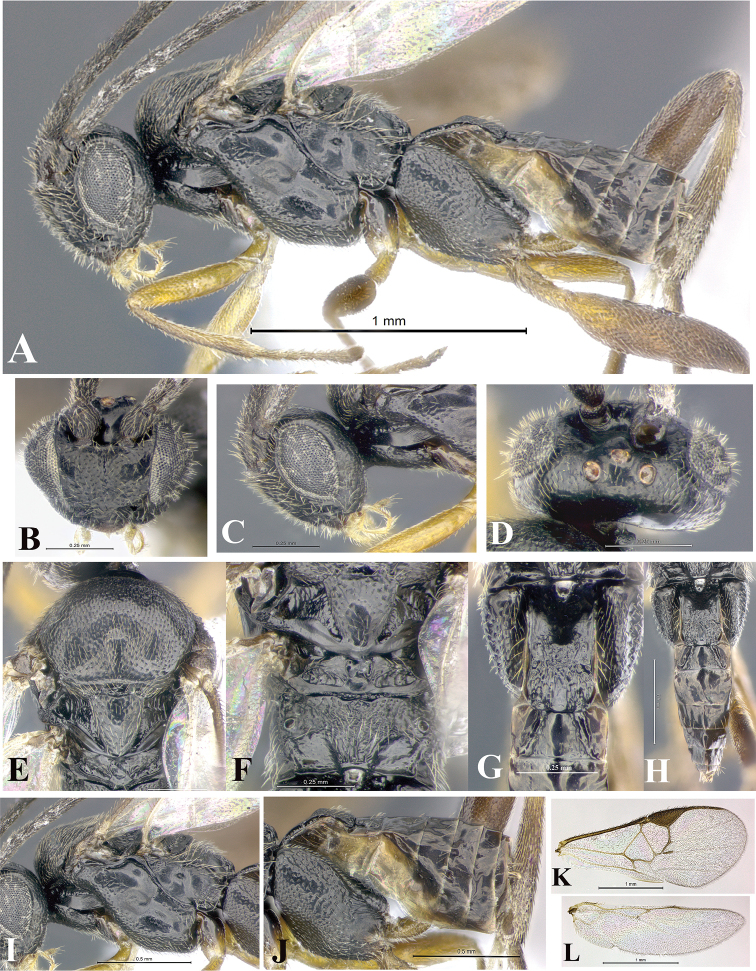
*Glyptapantelesmarcpolleti* sp. nov. male EC-37478 YY-A167 **A** Habitus **B, D** Head **B** Frontal view **D** Dorsal view **C** Head, propleuron, lateral view **E** Mesonotum, dorsal view **F** Scutellum, metanotum, propodeum, dorsal view **G**T1–2, dorsal view **H, J** Metasoma **H** Dorsal view **J** Lateral view **I** Mesosoma, lateral view **K, L** Wings **K** Fore **L** Hind.

Hind coxa with medium-size punctate throughout (Fig. 151A, J), hind telotarsus as equal in length as fourth tarsomere, distal antennal flagellomere subequal in length with penultimate, phragma of the scutellum widely visible (Fig. 151F), propodeum medially rhomboid-shaped with transverse rugae, but no trace of median longitudinal carina (Fig. 151F), anteroventral contour of mesopleuron convex (Fig. 151A, I), edges of median area on T2 polished and followed by a deep groove (Fig. 151G, H), and fore wing with r vein curved, outer side of junction of r and 2RS veins forming a distinct stub (Fig. 151K).

#### Coloration

(Fig. 151A–L). General body coloration polished black except scape, pedicel and all antennal flagellomeres (on both sides) brown; mandibles brown-reddish; glossa maxillary and labial palps light yellow-brown; tegulae brown. Eyes gray and ocelli reddish (in preserved specimen). Fore and middle legs light yellow-brown, except coxae medially brown with surrounded areas light brown, femora, tibiae and tarsomeres with a narrow dorsal brown strip from top to bottom, and claws brown; hind legs light yellow-brown except black coxae, femora brown, but proximally light yellow-brown, tibiae and tarsomeres brown. Petiole on T1 black and sublateral areas yellow-brown; T2 with median area black and lateral ends brown; T3 and beyond completely brown; distally each tergum with a very narrow whitish translucent band. In lateral view, T1–2 completely yellow-brown; T3–4 yellow-brown, but dorsally brown; T5 and beyond completely brown. S1–4 yellow-brown; penultimate sternum and hypopygium brown.

#### Description.

**Head** (Fig. 151A–D). Head rhomboid with pubescence long and dense. Proximal three antennal flagellomeres longer than wide (0.20:0.06, 0.25:0.06, 0.22:0.06), distal antennal flagellomere subequal in length with penultimate (0.13:0.05, 0.12:0.05), antenna longer than body (3.58, 2.53); antennal scrobes-frons sloped and forming a shelf. Distal half of face dented only laterally, punctate-lacunose, interspaces wavy and longitudinal median carina present. Frons smooth. Temple wide, punctate-lacunose and interspaces wavy. Inner margin of eyes diverging slightly at antennal sockets; in lateral view, eye anteriorly convex and posteriorly straight. POL shorter than OOL (0.10, 0.12). Malar suture present. Median area between lateral ocelli slightly depressed. Vertex laterally pointed or nearly so and dorsally wide.

**Mesosoma** (Fig. 151A, E, F, I). Mesosoma dorsoventrally convex. Distal 1/3 of mesoscutum with lateral margin slightly dented, punctation distinct throughout, interspaces smooth. Scutellum shield-shaped, apex sloped and fused with BS, but not in the same plane, scutellar punctation distinct throughout, in profile scutellum slightly convex, but on same plane as mesoscutum, phragma of the scutellum widely visible; BS only very partially overlapping the MPM; ATS demilune with short stubs delineating the area; dorsal ATS groove with semicircular/parallel carinae. Transscutal articulation with small and heterogeneous foveae, area just behind transscutal articulation with a smooth and shiny sloped transverse strip. Metanotum with BM convex; MPM circular without median longitudinal carina; AFM with a small lobe and not as well delineated as PFM; PFM thick, smooth and with lateral ends rounded; ATM proximally with a groove with some sculpturing and distally smooth. Propodeum medially romboid-shaped with rugae, proximal half curved rather coarse sculpture and distal half relatively polished and with a shallow dent at each side of nucha; distal edge of propodeum with a flange at each side and without stubs; propodeal spiracle distally framed by a short transverse carina; nucha surrounded by long radiating carinae. Pronotum with a distinct dorsal furrow, dorsally with a well-defined smooth band; central area of pronotum smooth, but both dorsal and ventral furrows with short parallel carinae. Propleuron with fine punctations throughout and dorsally without a carina. Metasternum convex. Contour of mesopleuron convex; precoxal groove smooth, shiny and shallow, but visible; epicnemial ridge convex, teardrop-shaped.

**Legs.** Ventral margin of fore telotarsus apex excavated, but without seta, fore telotarsus almost same width throughout and longer than fourth tarsomere (0.12, 0.07). Hind coxa with medium-size punctate throughout and dorsal outer depression present. Inner spur of hind tibia longer than outer spur (0.27, 0.19), entire surface of hind tibia with dense strong spines clearly differentiated by color and length; hind telotarsus as equal in length as fourth tarsomere (0.15, 0.14).

**Wings** (Fig. 151K, L). Fore wing with r vein slightly curved; 2RS vein slightly convex to convex; r and 2RS veins forming a weak, even curve at their junction and outer side of junction forming a distinct stub; 2M vein slightly curved/swollen; distally fore wing [where spectral veins are] with microtrichiae more densely concentrated than the rest of the wing; anal cell 1/3 proximally lacking microtrichiae; subbasal cell with microtrichiae virtually throughout; vein 2CUa absent and vein 2CUb spectral; vein 2 cu-a absent; vein 2-1A present only proximally as tubular vein; tubular vein 1 cu-a curved, incomplete/broken and not reaching the edge of 1-1A vein. Hind wing with vannal lobe very narrow, subdistally and subproximally straightened, and setae evenly scattered in the margin.

**Metasoma** (Fig. 151A, G, H, J). Metasoma laterally compressed. Petiole on T1with a mix of fine rugae and coarse sculpture over most of the surface, virtually parallel-sided over most of length, but barely narrowing at apex, apex truncate (length 0.37, maximum width 0.20, minimum width 0.15), and with scattered pubescence on distal half. Lateral grooves delimiting the median area on T2 clearly defined and reaching the distal edge of T2 (length median area 0.15, length T2 0.15), edges of median area polished and lateral grooves deep, median area broader than long (length 0.15, maximum width 0.20, minimum width 0.11); T2 with scattered pubescence throughout. T3 longer than T2 (0.20, 0.15) and with scattered pubescence throughout.

**Cocoon.** Unknown.

#### Comments.

The propodeum with a transverse discontinuous carina present only laterally; proximally the propodeum with coarse sculpture, in contrast distally is polished and each lateral side has a deep dent (Fig. 151F). This species looks like *Parapanteles* because of petiole shape (parallel-sided almost throughout) and the propodeum (each side of distal half with a distinctive dent). A whole specimen was used for DNA extraction.

#### Female.

Unknown.

#### Etymology.

Marc A. A. Pollet’s research is focused on biodiversity, sampling methodologies, ecology, systematics, taxonomy, phylogeny, and conservation of the long-legged flies Dolichopodidae (Diptera) in the Palaearctic and the Neotropics. Currently, he is a research manager at the Research Institute for Nature and Forest (INBO), Brussels, Belgium.

#### Distribution.

Parasitized caterpillar was collected in Ecuador, Napo, Yanayacu Biological Station (Yanayacu Road), during March 2009 at 2,100 m in cloud forest.

#### Biology.

The lifestyle of this parasitoid species is gregarious. *Mesochorus* (Ichneumonidae: Mesochorinae) was reported as hyperparasitoid.

#### Host.

Undetermined species of Apatelodidae feeding on *Miconia* sp. (Melastomataceae). Caterpillar was collected in fourth instar.

### Glyptapanteles marjorietownesae

Taxon classificationAnimaliaHymenopteraBraconidae

Arias-Penna, sp. nov.

http://zoobank.org/8F029B5B-1A1A-43B8-9FBB-C0DBBBDF7980

Figs 152 , 153

#### Female.

Body length 2.02 mm, antenna length 2.22 mm, fore wing length 2.17 mm.

#### Type material.

**Holotype**: COSTA RICA • 1♀; 05-SRNP-59772, DHJPAR0004223; Área de Conservación Guanacaste, Guanacaste, Sector Mundo Nuevo, Sendero Mora; dry-rain intergrade forest; 480 m; 10.76828, -85.42567; 04.ix.2005; José Alberto Sánchez leg.; caterpillar collected in fifth instar; cordwood cocoons on each side of larval cadaver and adhered to the leaf substrate, cocoons formed on 05.ix.2005; adult parasitoid emerged on 12.ix.2005; (CNC). **Paratypes.** • 60 (4♀, 4♂) (46♀, 6♂); 05-SRNP-59772, DHJPAR0004223; same data as for holotype; (CNC).

#### Other material.

**Reared material.** COSTA RICA: *Área de Conservación Guanacaste*, *Guanacaste*, *Sector Mundo Nuevo; Sendero Mora*: • 65 (0♀, 3♂) (0♀, 62♂); 05-SRNP-65601, DHJPAR0004780; dry-rain intergrade forest; 480 m; 10.76828, -85.42567; 11.xi.2005; Mariano Pereira leg.; caterpillar collected in fifth instar and already with cocoons; two rows of cordwood cocoons stacked on each side of the cadaver, cocoons adhered to the leaf substrate; adult parasitoid emerged on 18.xi.2005.

#### Diagnosis.

**Figure 152.**
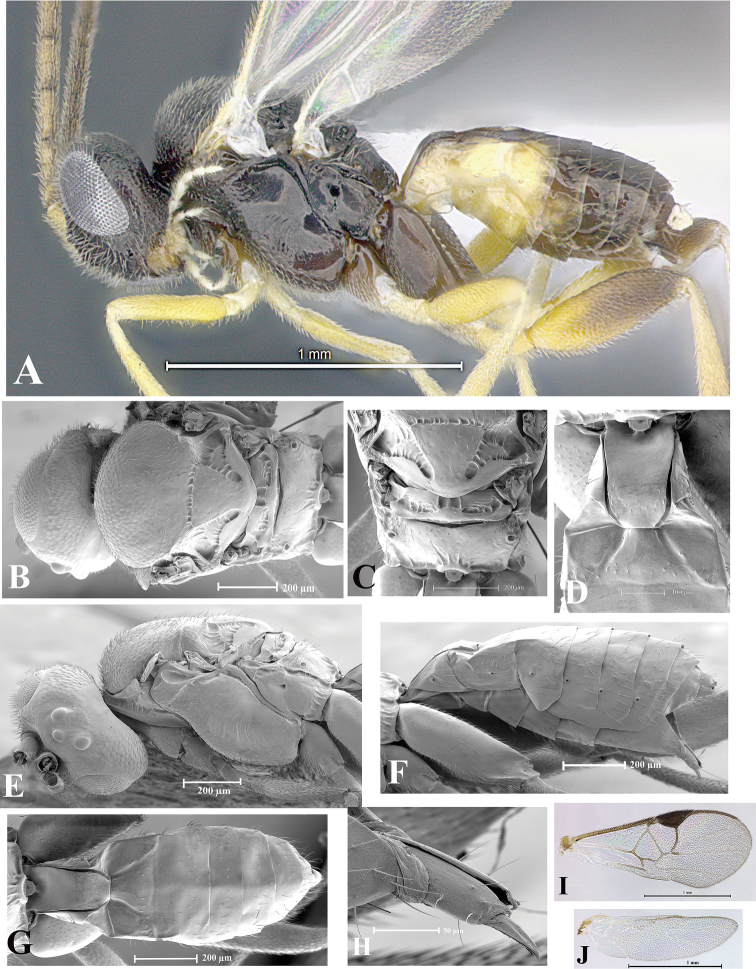
*Glyptapantelesmarjorietownesae* sp. nov. female 05-SRNP-59772 DHJPAR0004223 **A** Habitus **B, E** Head, mesosoma **B** Dorsal view **E** Lateral view **C** Scutellum, metanotum, propodeum, dorsal view **D**T1–2, dorsal view **F, G** Metasoma **F** Lateral view **G** Dorsal view **H** Genitalia: hypopygium, ovipositor, ovipositor sheaths, lateral view **I, J** Wings **I** Fore **J** Hind.

**Figure 153.**
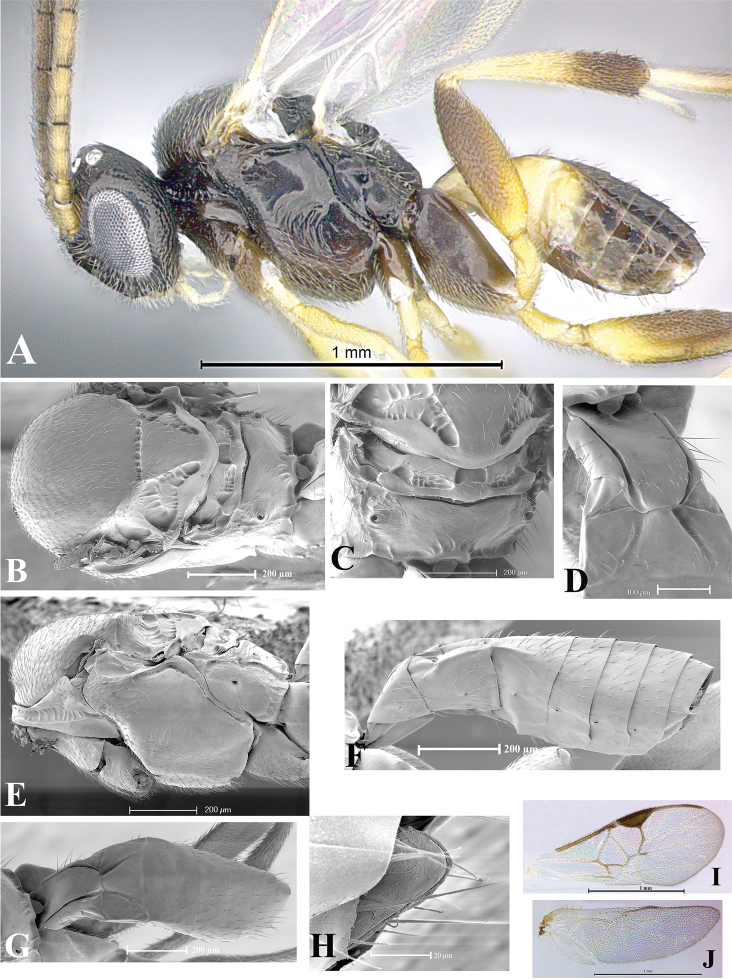
*Glyptapantelesmarjorietownesae* sp. nov. male 05-SRNP-59772 DHJPAR0004223 **A** Habitus **B, E** Mesosoma **B** Dorsal view **E** Lateral view **C** Scutellum, metanotum, propodeum, dorsal view **D**T1–2, dorsolateral view **F, G** Metasoma **F** Lateral view **G** Dorsolateral view **H** Genitalia: parameres, lateral view **I, J** Wings **I** Fore **J** Hind.

Vertex in lateral view pointed or nearly so (Fig. 153A), anterior furrow of metanotum with a small lobe, without setae, and not as well delineated as posterior furrow of metanotum (Figs 152C, 153C), mesoscutum proximally distinctly punctate, distally with a polished area (Figs 152B, 153B), fore wing with vein 2-1A absent, outer side of junction of r and 2RS veins not forming a stub (Figs 152I, 153I), median area between lateral ocelli without depression (Fig. 152E), distal antennal flagellomere longer than penultimate, petiole on T1 parallel-sided in proximal half, then narrowing, completely smooth and polished, with faint, satin-like sheen (Figs 152D, G, 153D, G), inner margin of eyes diverging slightly at antennal sockets, propodeum without median longitudinal carina (Figs 152C, 153C), and lateral grooves delimiting the median area on T2 clearly defined and reaching the distal edge of T2 (Figs 152D, G, 153D, G).

#### Coloration

(Fig. 152A). General body coloration brown-black except scape, pedicel, labrum, mandibles, glossa, and tegulae dark yellow; first three-four proximal antennal flagellomeres dorsally lighter (yellow-brown) than ventrally (brown), remaining flagellomeres brown on both sides; maxillary and labial palps light yellow. Eyes and ocelli silver. Fore and middle legs yellow except light brown coxae and brown claws; hind legs yellow except coxae completely light brown, distal 3/4 of femora from yellow-brown to brown (intensity of coloration increasing from proximal to distal), distal 1/3 of tibia brown, and tarsomeres brown although proximal half of three most proximal tarsomeres yellow. Petiole on T1 with coloration intensifying from proximal to distal, thus, proximal 1/3 yellow, middle third reddish/yellow-brown and distal 1/3 brown, contours darkened, and sublateral areas yellow-brown; T2 with median area brown and lateral ends yellow-brown/light brown; T3 mostly dark brown and lateral ends yellow-brown; T4 and beyond completely brown; distally each tergum with a narrow yellowish transparent band. In lateral view, T1–3 completely yellow; T4 yellow, but dorsally brown; T5 and beyond brown. S1–3 yellow; S4 proximal half yellow, distal half brown; penultimate sternum and hypopygium brown.

#### Description.

**Head** (Fig. 152A, B, E). Head rounded with pubescence long and dense. Proximal three antennal flagellomeres longer than wide (0.18:0.05, 0.17:0.05, 0.17:0.05), distal antennal flagellomere longer than penultimate (0.11:0.05, 0.08:0.05), antenna longer than body (2.22, 2.02); antennal scrobes-frons shallow. Face with depression only laterally with dense fine punctations, interspaces with microsculpture and longitudinal median carina present. Frons smooth. Temple wide, punctate and interspaces wavy. Inner margin of eyes diverging slightly at antennal sockets; in lateral view, eye anteriorly convex and posteriorly straight. POL shorter than OOL (0.06, 0.11). Malar suture absent or difficult to see. Median area between lateral ocelli without depression. Vertex laterally pointed or nearly so and dorsally wide.

**Mesosoma** (Fig. 152A, C–E). Mesosoma dorsoventrally convex. Mesoscutum proximally convex and distally flat, punctation distinct proximally with polished area distally, interspaces with microsculpture. Scutellum triangular, apex sloped and fused with BS, scutellar punctation distinct peripherally, but absent centrally, in profile scutellum flat and on same plane as mesoscutum, phragma of the scutellum partially exposed; BS only very partially overlapping the MPM; ATS demilune with short stubs delineating the area; dorsal ATS groove with semicircular/parallel carinae. Transscutal articulation with small and heterogeneous foveae, area just behind transscutal articulation smooth, shiny and nearly at the same level as mesoscutum (flat). Metanotum with BM wider than PFM (clearly differentiated); MPM semicircular without median longitudinal carina; AFM with a small lobe and not as well delineated as PFM; PFM thick, smooth and with a distal flat flange; ATM proximally with a well-defined row of foveae and distally smooth. Propodeum relatively polished without median longitudinal carina, proximal half curved; distal edge of propodeum with a flange at each side and without stubs; propodeal spiracle without distal carina; nucha surrounded by very short radiating carinae. Pronotum with a distinct dorsal furrow, dorsally with a well-defined smooth band; central area of pronotum smooth, but both dorsal and ventral furrows with short parallel carinae. Propleuron with a mix of rugae and fine punctation, dorsally with a carina. Metasternum flat or nearly so. Contour of mesopleuron straight/angulate or nearly so; precoxal groove deep with faintly transverse lineate sculpture; epicnemial ridge convex, teardrop-shaped.

**Legs.** Ventral margin of fore telotarsus slightly excavated and with a tiny curved seta, fore telotarsus almost same width throughout and longer than fourth tarsomere (0.11, 0.06). Hind coxa with punctation only on ventral surface and dorsal outer depression present. Inner spur of hind tibia longer than outer spur (0.17, 0.15), entire surface of hind tibia with dense strong spines clearly differentiated by color and length. Hind telotarsus as equal in length as fourth tarsomere (0.10, 0.10).

**Wings** (Fig. 152I, J). Fore wing with r vein slightly curved; 2RS vein straight; r and 2RS veins forming a weak, even curve at their junction and outer side of junction not forming a stub; 2M vein slightly curved/swollen; distally fore wing [where spectral veins are] with microtrichiae more densely concentrated than the rest of the wing; anal cell 1/3 proximally lacking microtrichiae; subbasal cell with a small smooth area; vein 2CUa absent and vein 2CUb spectral; vein 2 cu-a absent; vein 2-1A absent; tubular vein 1 cu-a straight, incomplete/broken and not reaching the edge of 1-1A vein. Hind wing with vannal lobe narrow, subdistally and subproximally straightened, and setae present proximally, but absent distally.

**Metasoma** (Fig. 152A, D, F–H). Metasoma laterally compressed. Petiole on T1 completely smooth and polished, with faint, satin-like sheen, parallel-sided in proximal half and then narrowing (length 0.26, maximum width 0.14, minimum width 0.07), and with scattered pubescence concentrated in the first distal third. Lateral grooves delimiting the median area on T2 clearly defined and reaching the distal edge of T2 (length median area 0.14, length T2 0.14), edges of median area polished and lateral grooves deep, median area broader than long (length 0.14, maximum width 0.18, minimum width 0.05); T2 with scattered pubescence only distally. T3 longer than T2 (0.19, 0.14) and with scattered pubescence throughout. Pubescence on hypopygium dense.

**Cocoons.** Brown oval cocoons with evenly smooth silk fibers. Cocoons forming two rows of cordwood stacked on each side of the cadaver caterpillar and adhered to the leaf substrate.

#### Comments.

Both sexes with slim body.

#### Male

(Fig. 153A–J). In some specimens S1-4 yellow, but medial with brown tint. In some specimens, the body coloration (e.g., 05-SRNP-65601) is darker than females.

#### Etymology.

Marjorie Chapman Townes (228 March 1909-8 October 2006) together with her husband, Henry Townes, established the American Entomological Institute (AEI), Gainsville, FL, USA, as a not-for-profit organization to manage the huge, world-class Hymenoptera collection and library. The Townes were appreciated internationally as a team who contributed significantly to our understanding of the taxonomy of Hymenoptera, especially of the family Ichneumonidae.

#### Distribution.

The parasitized caterpillars were collected in Costa Rica, ACG, Sector Mundo Nuevo (Sendero Mora), during September and November 2005 at 480 m in dry-rain intergrade forest.

#### Biology.

The lifestyle of this parasitoid species is gregarious.

#### Host.

*Azetaceramina* Hübner (Noctuidae: Catocalinae) feeding on *Acosmiumpanamense* (Fabaceae) and undetermined species of plant. Caterpillars were collected in fifth instar.

### Glyptapanteles markshawi

Taxon classificationAnimaliaHymenopteraBraconidae

Arias-Penna, sp. nov.

http://zoobank.org/54F91E21-4E07-4A45-856C-9D5C82B9D790

Figs 154 , 155

#### Female.

Body length 2.22 mm, antenna length 2.53 mm, fore wing length 2.47 mm.

#### Type material.

**Holotype**: COSTA RICA • 1♀; 07-SRNP-24093, DHJPAR0020471; Área de Conservación Guanacaste, Guanacaste, Sector Del Oro, Uncaria; dry-rain intergrade forest; 370 m; 11.01752, -85.47411; 14.x.200; Roster Moraga leg.; caterpillar collected in fifth instar; two parallel rows of cordwood cocoons on each side of the cadaver adhered to the leaf substrate and formed on 16.x.2007; adult parasitoids emerged on 23.x.2007; (CNC). **Paratypes.** • 7 (1♀, 1♂) (2♀, 3♂); 07-SRNP-24093, DHJPAR0020471; same data as for holotype; (CNC).

#### Other material.

**Reared material.** COSTA RICA: *Área de Conservación Guanacaste*, *Guanacaste*, *Sector Mundo Nuevo*, *Sendero Puertas*: • 7 (2♀, 1♂) (3♀, 1♂); 05-SRNP-22188, DHJPAR0002897; dry-rain intergrade forest; 400 m; 11.01087, -85.48817; 16.vi.2005; Roster Moraga leg.; caterpillar collected in fifth instar; light gray cocoons formed on 23.vi.2005; adult parasitoids emerged on 28.vi.2005.

*Área de Conservación Guanacaste*, *Guanacaste*, *Sector Del Oro*, *Quebrada Romero*: • 6 (2♀, 1♂) (3♀, 0♂); 06-SRNP-20030, DHJPAR0012022; dry-rain intergrade forest; 490 m; 11.00519, -85.47398; 03.i.2006; Roster Moraga leg.; caterpillar collected in fourth instar; scattered white cocoons formed on 11.i.2006; adult parasitoids emerged on 20.i.2006.

#### Diagnosis.

**Figure 154.**
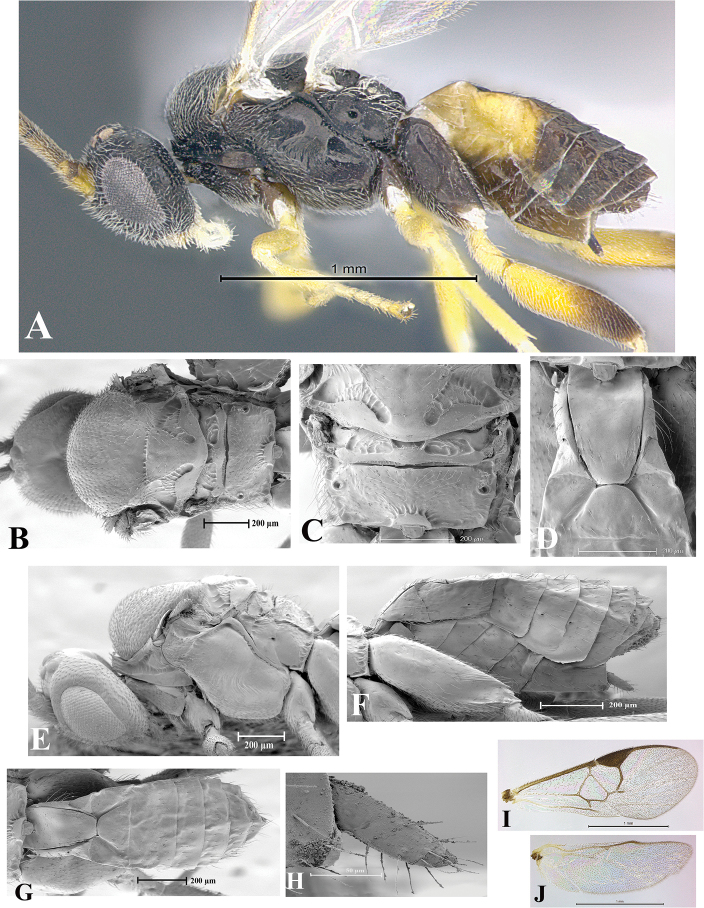
*Glyptapantelesmarkshawi* sp. nov. female 07-SRNP-24093 DHJPAR0020471 **A** Habitus **B, E** Head, mesosoma **B** Dorsal view **F** Lateral view **C** Scutellum, metanotum, propodeum, dorsal view **D**T1–2, dorsal view **F, G** Metasoma **F** Lateral view **G** Dorsal view **H** Genitalia: hypopygium, ovipositor, ovipositor sheaths, lateral view **I, J** Wings **I** Fore **J** Hind.

**Figure 155.**
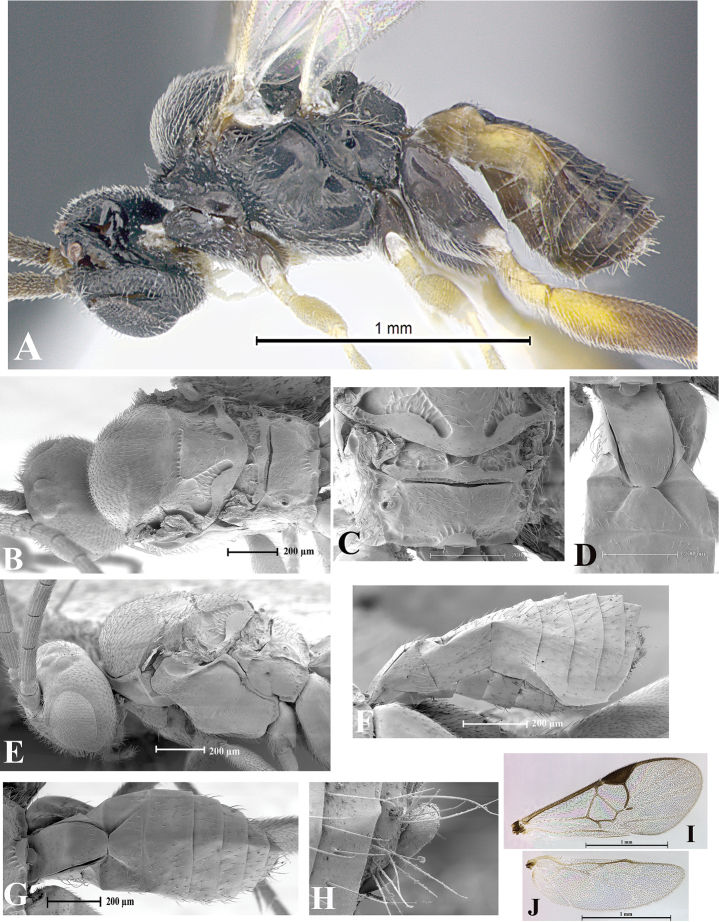
*Glyptapantelesmarkshawi* sp. nov. male 07-SRNP-24093 DHJPAR0020471 **A** Habitus **B, E** Head, mesosoma **B** Dorsal view **F** Lateral view **C** Scutellum, metanotum, propodeum, dorsal view **D**T1–2, dorsal view **F, G** Metasoma **F** Lateral view **G** Dorsal view **H** Genitalia: parameres, lateral view **I, J** Wings **I** Fore **J** Hind.

Medioposterior band of scutellum mostly overlapping the medioanterior pit of metanotum (Figs 154B, C, 155B, C), fore wing with vein 2-1A present only proximally as tubular vein, 2RS vein straight, outer side of junction of r and 2RS veins not forming a stub (Figs 154I, 155I), medioanterior pit of metanotum semicircular without median longitudinal carina (Figs 154B, C, 155B, C), anteroventral contour of mesopleuron convex (Figs 154A, E, 155A, E), petiole on T1 distally with lateral margins relatively straight (Figs 154D, G, 155D, G), propodeum without median longitudinal carina, propodeal spiracle without distal carina (Figs 154B, C, 155B, C), nucha surrounded by very short radiating carinae (Figs 154B, C, 155B, C), antenna longer than body, and lateral grooves delimiting the median area on T2 distally losing definition (Figs 154D, G, 155D, G).

#### Coloration

(Fig. 154A). General body coloration black except scape, pedicel, labrum, and mandibles dark yellow; first four-five proximal antennal flagellomeres dorsally lighter (light brown) than ventrally (dark brown), remaining flagellomeres dark brown on both sides; glossa, maxillary and labial palps light yellow. Eyes gray and ocelli yellowish. Fore and middle legs yellow except light brown coxae and brown claws; hind legs yellow except coxae completely brown-black, distal half of femora and tibiae brown, and tarsomeres completely brown except basitarsus with proximal half yellow. Petiole on T1 with coloration that intensifies from proximal to distal, proximally yellow-brown, medially light brown and distally dark brown, contours darkened and sublateral areas yellow-brown; T2 with median and adjacent areas brown, adjacent area wide and together with median area forming a rectangle-shaped area, and narrow lateral ends yellow; T3 light brown/brown proximally with a small yellow/yellow-brown area on corners; T4 and beyond completely dark brown; distally each tergum with a narrow whitish transparent band. In lateral view, T1–3 yellow; T4 and beyond brown. S1–2 completely yellow; S3 yellow, but distally with a longitudinal narrow brown band; S4 and beyond brown.

#### Description.

**Head** (Fig. 154A, B, E). Head rhomboid with pubescence long and dense. Proximal three antennal flagellomeres longer than wide (0.17:0.08, 0.17:0.08, 0.17:0.08), distal antennal flagellomere longer than penultimate (0.11:0.07, 0.08:0.07), antenna longer than body (2.53, 2.22); antennal scrobes-frons shallow. Face with depression only laterally, dense fine punctations, interspaces with microsculpture and longitudinal median carina present. Frons smooth. Temple wide, punctate and interspaces wavy. Inner margin of eyes diverging slightly at antennal sockets; in lateral view, eye anteriorly convex and posteriorly straight. POL shorter than OOL (0.08, 0.12). Malar suture present. Median area between lateral ocelli slightly depressed. Vertex laterally rounded and dorsally wide.

**Mesosoma** (Fig. 154A–C, E). Mesosoma dorsoventrally convex. Mesoscutum proximally convex and distally flat, punctation distinct proximally with polished area distally, interspaces with microsculpture. Scutellum triangular, apex sloped and fused with BS, scutellar punctation distinct peripherally and absent centrally, in profile scutellum flat and on same plane as mesoscutum, phragma of the scutellum partially exposed; BS mostly overlapping the MPM; ATS demilune with quite a little complete parallel carinae; dorsal ATS groove with carinae only proximally. Transscutal articulation with small and homogeneous foveae, area just behind transscutal articulation smooth, shiny and depressed centrally. Metanotum with BM wider than PFM (clearly differentiated); MPM circular/semicircular without median longitudinal carina; AFM without setiferous lobes and not as well delineated as PFM; PFM thick, smooth and with a distal flat flange; ATM proximally with sculpture distally without a well delimited smooth area. Propodeum relatively polished without median longitudinal carina, proximal half curved; distal edge of propodeum with a flange at each side and without stubs; propodeal spiracle without distal carina; nucha surrounded by very short radiating carinae. Pronotum with a distinct dorsal furrow, dorsally with a well-defined smooth band; central area of pronotum smooth, but both dorsal and ventral furrows with short parallel carinae. Propleuron with fine punctations throughout and dorsally with a carina. Metasternum flat or nearly so. Contour of mesopleuron convex; precoxal groove deep with transverse lineate sculpture; epicnemial ridge elongated more fusiform (tapering at both ends).

**Legs.** Ventral margin of fore telotarsus slightly excavated and with a tiny curved seta, fore telotarsus almost same width throughout and longer than fourth tarsomere (0.10, 0.05). Hind coxa with punctation only on ventral surface and dorsal outer depression present. Inner spur of hind tibia longer than outer spur (0.21, 0.15), entire surface of hind tibia with dense strong spines clearly differentiated by color and length. Hind telotarsus as equal in length as fourth tarsomere (0.11, 0.10).

**Wings** (Fig. 154I, J). Fore wing with r vein slightly curved; 2RS vein straight; r and 2RS veins forming a weak, even curve at their junction and outer side of junction not forming a stub; 2M vein slightly curved/swollen; distally fore wing [where spectral veins are] with microtrichiae more densely concentrated than the rest of the wing; anal cell 1/3 proximally lacking microtrichiae; subbasal cell with a small smooth area; vein 2CUa absent and vein 2CUb spectral; vein 2 cu-a absent; vein 2-1A present only proximally as tubular vein; tubular vein 1 cu-a curved, incomplete/broken and not reaching the edge of 1-1A vein. Hind wing with vannal lobe narrow, subdistally and subproximally straightened, and setae present proximally, but absent distally.

**Metasoma** (Fig. 154A, D, F–H). Metasoma laterally compressed. Petiole on T1 finely sculptured only laterally, parallel-sided in proximal half and then narrowing (length 0.32, maximum width 0.18, minimum width 0.10), and with scattered pubescence concentrated in the first distal third. Lateral grooves delimiting the median area on T2 distally losing definition (length median area 0.10, length T2 0.13), edges of median area polished and lateral grooves deep, median area broader than long (length 0.10, maximum width 0.23, minimum width 0.10); T2 with scattered pubescence only distally. T3 longer than T2 (0.17, 0.13) and with scattered pubescence throughout. Pubescence on hypopygium dense.

**Cocoons.** Light gray or white oval cocoons with ordered silk fibers, but covered by a net. Cocoons forming two rows of cordwood on each side of the caterpillar adhered to the leaf substrate.

#### Male

(Fig. 155A–J). Body shape similar to female; however, male without tricolored petiole, instead it is completely light brown although the contours are darkened.

#### Etymology.

Mark R. Shaw works on the natural history and systematics of Lepidoptera and parasitioid wasps at the National Museums of Scotland, U.K.

#### Distribution.

The parasitized caterpillars were collected in Costa Rica, ACG, Sector Del Oro (Quebrada Romero and Uncaria) and Sector Mundo Nuevo (Sendero Puertas), during June 2005, January 2006, and October 2007 at 370 m, 400 m, and 490 m in dry-rain intergrade forest.

#### Biology.

The lifestyle of this parasitoid species is gregarious.

#### Host.

*Ethmiascythropa* Walsingham (Depressariidae: Ethmiinae) feeding on *Bourreriacostaricensis* and *B.oxyphylla* (Boraginaceae). Caterpillars were collected in fourth and fifth instar.

### Glyptapanteles marshawheelerae

Taxon classificationAnimaliaHymenopteraBraconidae

Arias-Penna, sp. nov.

http://zoobank.org/6769DF01-06EA-4059-ACB4-7B7A0FD63388

Fig. 156

#### Female.

Body length 3.28 mm, antenna length 3.33 mm, fore wing length 3.53 mm.

#### Type material.

**Holotype**: ECUADOR • 1♀; EC-1491, YY-A021; Napo, Yanayacu Biological Station, YanayacuForest; cloud forest; 2,100 m; -0.6, -77.883333; 24.i.2005; Lee Dyer leg.; cocoons away from host in running trail; adult parasitoids emerged on 21.iii.2005; (PUCE). **Paratypes.** • 17 (4♀, 5♂) (4♀, 4♂); EC-1491, YY-A021; same data as for holotype; (PUCE).

#### Diagnosis.

**Figure 156.**
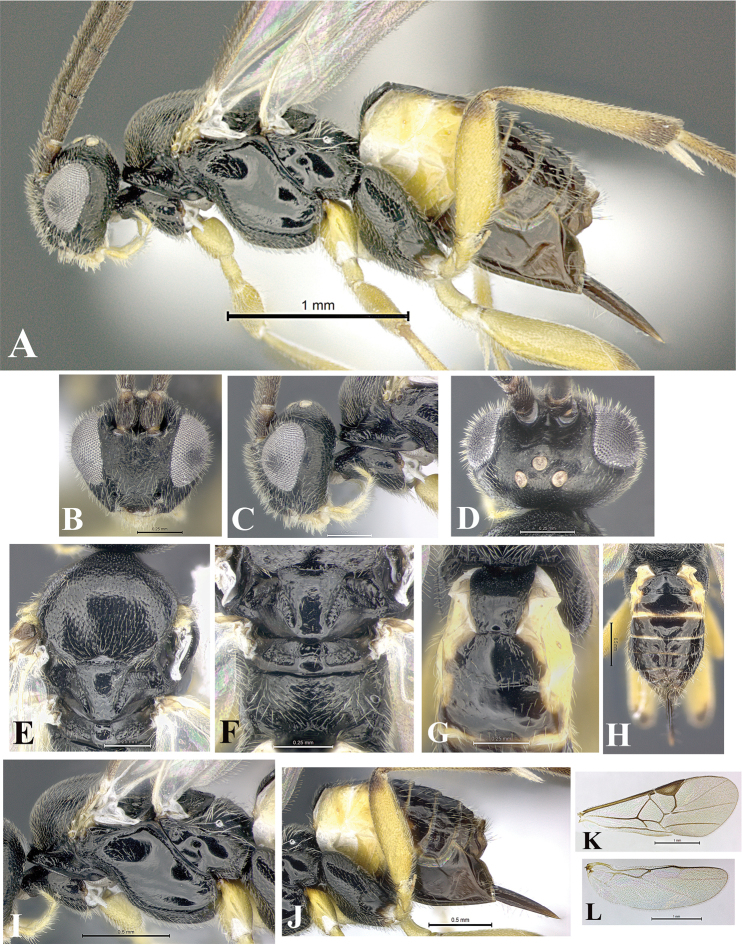
*Glyptapantelesmarshawheelerae* sp. nov. female EC-1491 YY-A021 **A** Habitus **B, D** Head **B** Frontal view **D** Dorsal view **C** Head, pronotum, propleuron, lateral view **E** Mesonotum, dorsal view **F** Scutellum, metanotum, propodeum, dorsal view **G**T1–3, dorsal view **H, J** Metasoma **H** Dorsal view **J** Lateral view **I** Mesosoma, lateral view **K, L** Wings **K** Fore **L** Hind.

Shape of proximal half of propodeum more strongly curved in dorsal view (Fig. 156F), longitudinal median carina on face absent (Fig. 156B), lateral grooves delimiting the median area on T2 distally losing definition, edges of median area on T2 polished and followed by a deep groove (Fig. 156G, H), propodeum without median longitudinal carina (Fig. 156F), anteroventral contour of mesopleuron convex (Fig. 156A, I), and fore wing with r vein curved, outer side of junction of r and 2RS veins forming a distinct stub (Fig. 156K).

#### Coloration

(Fig. 156A–L). General body coloration polished black except scape brown distally with a yellow-brown ring; labrum, maxillary and labial palps, and tegulae yellow; pedicel and all antennal flagellomeres (on both sides) brown; labrum and mandibles brown-red/reddish. Eyes and ocelli silver. Fore and middle legs yellow except brown claw and tarsomeres with brown tints; hind legs yellow except coxae black, but distally yellow, femora distally with a small brown spot, distal 1/3 of tibiae brown, additionally tibiae proximally with a brown band, and tarsomeres brown. Petiole on T1 black and sublateral areas yellow; T2 with median area black, adjacent area brown with a silhouette well-defined, and lateral ends yellow; T3 mostly brown with proximal half of lateral ends yellow, proximally width of dark area coincides with the width of median and adjacent areas on T2, but distally dark area reaching the edge of T3; T4 and beyond completely brown; distally each tergum with an narrow yellow translucent band. In lateral view, T1–2 completely yellow; T3–4 yellow, but dorsally brown; T5 and beyond brown. S1–4 yellow; penultimate sternum and hypopygium brown.

#### Description.

**Head** (Fig. 156A–D). Head rounded with pubescence long and dense. Proximal three antennal flagellomeres longer than wide (0.28:0.08, 0.29:0.08, 0.28:0.08), distal antennal flagellomere longer than penultimate (0.15:0.07, 0.12:0.07), antenna longer than body (3.33, 3.28); antennal scrobes-frons sloped and forming a shelf. Face flat or nearly so, with dense fine punctations, interspaces smooth and longitudinal median carina absent. Frons smooth. Temple wide, punctate and interspaces clearly smooth. Inner margin of eyes diverging slightly at antennal sockets; in lateral view, eye anteriorly convex and posteriorly straight. POL shorter than OOL (0.09, 0.14). Malar suture present. Median area between lateral ocelli slightly depressed. Vertex laterally pointed or nearly so and dorsally wide.

**Mesosoma** (Fig. 156A, E, F, I). Mesosoma dorsoventrally convex. Distal 1/3 of mesoscutum with lateral margin slightly dented, punctation distinct throughout, interspaces smooth. Scutellum long and slender, apex sloped and fused with BS, but not in the same plane, scutellar punctation distinct peripherally and absent centrally, in profile scutellum flat and on same plane as mesoscutum, phragma of the scutellum partially exposed; BS only very partially overlapping the MPM; ATS demilune inner side with a row of foveae; dorsal ATS groove with carinae only proximally. Transscutal articulation with small and heterogeneous foveae, area just behind transscutal articulation sloped, smooth and shiny. Metanotum with BM wider than PFM (clearly differentiated); MPM circular without median longitudinal carina; AFM without setiferous lobes and not as well delineated as PFM; PFM thick, smooth and with lateral ends rounded; ATM proximally with sculpture distally without a well delimited smooth area. Propodeum without median longitudinal carina, proximal half curved with fine sculpture and distal half relatively polished; distal edge of propodeum with a flange at each side and short stubs; propodeal spiracle without distal carina; nucha surrounded by very short radiating carinae. Pronotum with a distinct dorsal furrow, dorsally with a well-defined smooth band; central area of pronotum smooth, but both dorsal and ventral furrows with short parallel carinae. Propleuron finely sculptured only ventrally and dorsally without a carina. Metasternum convex. Contour of mesopleuron convex; precoxal groove smooth, shiny and shallow, but visible; epicnemial ridge elongated more fusiform (tapering at both ends).

**Legs.** Ventral margin of fore telotarsus slightly excavated and with a tiny curved seta, fore telotarsus almost same width throughout and longer than fourth tarsomere (0.12, 0.09). Hind coxa finely punctate throughout, and dorsal outer depression present. Inner spur of hind tibia longer than outer spur (0.31, 0.25), entire surface of hind tibia with dense strong spines clearly differentiated by color and length. Hind telotarsus as equal in length as fourth tarsomere (0.17, 0.16).

**Wings** (Fig. 156K, L). Fore wing with r vein slightly curved; 2RS vein straight; r and 2RS veins forming a weak, even curve at their junction and outer side of junction forming a slight stub; 2M vein slightly curved/swollen; distally fore wing [where spectral veins are] with microtrichiae more densely concentrated than the rest of the wing; anal cell 1/3 proximally lacking microtrichiae; subbasal cell with microtrichiae virtually throughout; veins 2CUa and 2CUb completely spectral; vein 2 cu-a present as spectral vein, sometimes difficult to see; vein 2-1A proximally tubular and distally spectral, although sometimes difficult to see; tubular vein 1 cu-a curved, incomplete/broken and not reaching the edge of 1-1A vein. Hind wing with vannal lobe very narrow, subdistally and subproximally straightened, and setae absent proximally, but scattered distally.

**Metasoma** (Fig. 156A, G, H, J). Metasoma laterally compressed. Petiole on T1 finely sculptured only laterally, virtually parallel-sided over most of length, but barely narrowing at apex, apex truncate (length 0.41, maximum width 0.27, minimum width 0.17), and with scattered pubescence concentrated in the first distal third. Lateral grooves delimiting the median area on T2 distally losing definition (length median area 0.12, length T2 0.21), edges of median area polished, median area broader than long (length 0.12, maximum width 0.30, minimum width 0.16); T2 with scarce pubescence throughout. T3 longer than T2 (0.26, 0.21) and with scattered pubescence throughout. Pubescence on hypopygium dense.

**Cocoons.** Unknown.

#### Comments.

The ovipositor is long as in *Sathon* (Fig. 156A, J).

#### Male.

Coloration similar to female.

#### Etymology.

Marsha Wheeler was interested in molecular analyses of endocrine and nutritional factors that affect division of labor and health in honey bees (*ApisMellifera* Linnaeus) as a graduate student at UIUC, IL, USA.

#### Distribution.

Parasitized caterpillar was collected in Ecuador, Napo, Yanayacu Biological Station (YanayacuForest), during January 2005 at 2,100 m in cloud forest.

#### Biology.

The lifestyle of this parasitoid species is gregarious.

#### Host.

Undetermined species of Lepidoptera feeding on *Vismia* sp. (Clusiaceae). Caterpillar instar was not reported.

### Glyptapanteles mayberenbaumae

Taxon classificationAnimaliaHymenopteraBraconidae

Arias-Penna, sp. nov.

http://zoobank.org/C762D794-4036-4BA4-86E3-BFEBEAB7E512

Fig. 157

#### Female.

Body length 2.73 mm, antenna length 3.48 mm, fore wing length 3.33 mm.

#### Type material.

**Holotype**: ECUADOR • 1♀; EC-40241, YY-A000; Napo, Yanayacu Biological Station, Yanayacu Road; cloud forest; 2,100 m; -0.566667, -77.866667; 05.viii.2009; CAPEA leg.; caterpillar collected in fourth instar; cocoons formed on 26.viii.2009; adult parasitoids emerged on 31.viii.2009; (PUCE). **Paratypes.** • 86 (9♀, 4♂) (73♀, 0♂); EC-40241, YY-A000; same data as for holotype; (PUCE).

#### Diagnosis.

**Figure 157.**
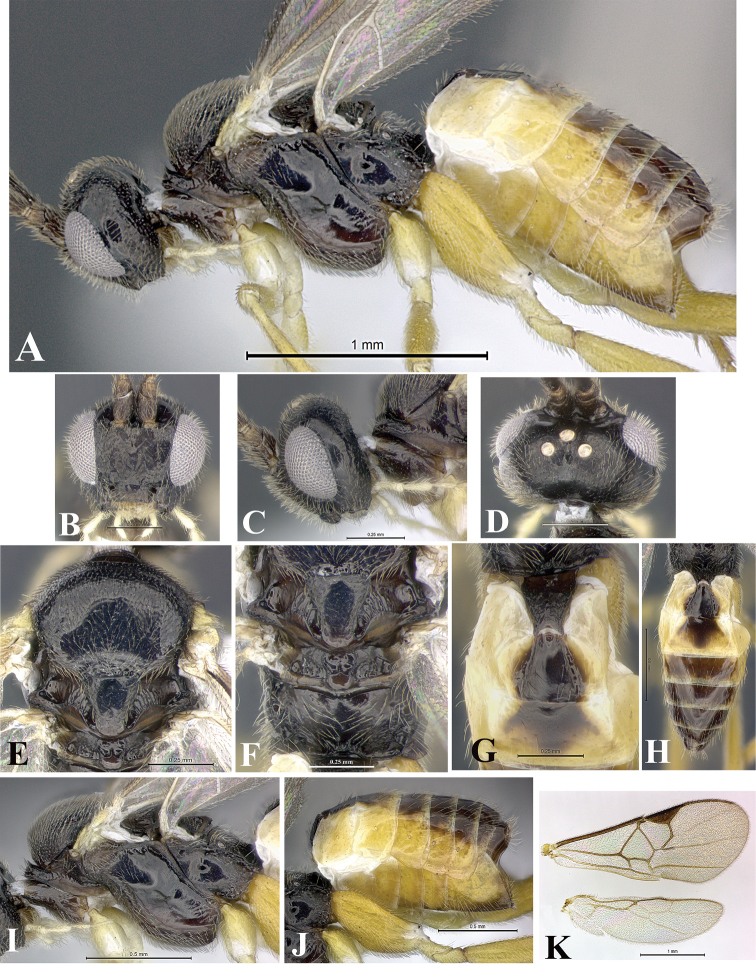
*Glyptapantelesmayberenbaumae* sp. nov. female EC-40241 YY-A000 **A** Habitus **B, D** Head **B** Frontal view **D** Dorsal view **C** Head, pronotum, propleuron lateral view **E** Mesonotum, dorsal view **F** Scutellum, metanotum, propodeum, dorsal view **G**T1–3, dorsal view **H, J** Metasoma **H** Dorsal view **J** Lateral view **I** Mesosoma, lateral view **K** Fore and hind wings.

Medioanterior pit of metanotum circular or oval with a short proximal carina (Fig. 157E, F), vertex in dorsal view wide (Fig. 157D), scutellar punctation indistinct throughout (Fig. 157E, F), dorsal furrow of pronotum without a smooth band (Fig. 157A, C, I), dorsal carina delimiting a dorsal furrow on propleuron present (Fig. 157A, C, I), anterior furrow of metanotum without setiferous lobes (Fig. 157F), axillary trough of scutellum with sculpture (Fig. 157E, F), lateral grooves delimiting the median area on T2 clearly defined and reaching the distal edge of T2, edges of median area polished and followed by a deep groove (Fig. 157G, H), propodeum without median longitudinal carina (Fig. 157F), anteroventral contour of mesopleuron convex (Fig. 157A, I), and fore wing with r vein curved, outer side of junction of r and 2RS veins forming a stub (Fig. 157K).

#### Coloration

(Fig. 156A–K). General body coloration polished black except pedicel brown distally with a yellow-brown ring; scape and all antennal flagellomeres (on both sides) brown; labrum, mandibles and glossa yellow-brown; maxillary and labial palps, and tegulae yellow; propleuron, both dorsal and ventral furrows of pronotum, epicnemial ridge, ventral edge of mesopleuron, distal edges of mesoscutum, dorsal ATS groove, lunules, BS, PFM, BM and lateral ends of metapleuron with brown-red/reddish tints. Eyes and ocelli silver. Fore, middle and hind legs yellow; however, coloration in tibiae and tarsomeres with light yellow-brown tints. Petiole on T1 with two colorations, proximal half brown-red/reddish and distal half brown, contours darkened and sublateral areas yellow; T2 with median and adjacent areas brown, adjacent area narrow reaching very close to the contours of median area, and lateral ends yellow; T3 medially with a brown area which proximal width coincides with the width of median and adjacent areas on T2, distally the brown area forming like three projections not reaching the distal edge of T3, remaining area of T3 yellow; T4 and beyond completely brown, coloration darkened from proximal to distal; distally each tergum with a narrow yellow translucent band. In lateral view, T1–2 yellow; T3 and beyond yellow, but dorsally brown, the extent of brown area remains almost constant from proximal to distal. All sterna yellow, but hypopygium medially brown.

#### Description.

**Head** (Fig. 157A–D). Head rounded with pubescence long and dense. Proximal three antennal flagellomeres longer than wide (0.23:0.06, 0.24:0.06, 0.23:0.06), distal antennal flagellomere longer than penultimate (0.15:0.05, 0.12:0.05), antenna longer than body (3.48, 2.73); antennal scrobes-frons sloped and forming a shelf. Face flat or nearly so, with dense fine punctations, interspaces smooth and longitudinal median carina present. Frons smooth. Temple narrow, punctations barely noticeable and interspaces clearly smooth; inner margin of eyes diverging slightly at antennal sockets; in lateral view, eye anteriorly convex and posteriorly straight. POL shorter than OOL (0.09, 0.13). Malar suture present. Median area between lateral ocelli without depression. Vertex laterally rounded and dorsally wide.

**Mesosoma** (Fig. 157A, E, F, I). Mesosoma dorsoventrally convex. Mesoscutum 1/4 distal with a central dent, punctation distinct throughout, interspaces smooth. Scutellum long and slender, apex sloped and fused with BS, but not in the same plane, scutellar punctation indistinct throughout, in profile scutellum flat and on same plane as mesoscutum, phragma of the scutellum partially exposed; BS only very partially overlapping the MPM; ATS demilune with complete undulate/reticulate carinae; dorsal ATS groove smooth. Transscutal articulation with small and heterogeneous foveae, area just behind transscutal articulation depressed centrally and with same kind of sculpture as mesoscutum. Metanotum with BM convex; MPM oval/circular with a short proximal carina; AFM without setiferous lobes and not as well delineated as PFM; PFM thick and smooth; ATM proximally with sculpture distally without a well delimited smooth area. Propodeum with indistinct sculpture, without median longitudinal carina, proximal half weakly curved; distal edge of propodeum with a flange at each side and without stubs; propodeal spiracle distally framed by a short concave carina; nucha surrounded by very short radiating carinae. Pronotum with dorsal furrow distinctive only proximally, dorsally without a smooth band; central area of pronotum smooth, and ventral furrow with short parallel carinae. Propleuron finely sculptured only ventrally and dorsally with a carina. Metasternum convex. Contour of mesopleuron convex; precoxal groove smooth, shiny and shallow, but visible; epicnemial ridge convex, teardrop-shaped.

**Legs.** Ventral margin of fore telotarsus slightly excavated and with a tiny curved seta, fore telotarsus almost same width throughout and longer than fourth tarsomere (0.13, 0.09). Hind coxa finely punctate throughout, and dorsal outer depression absent. Inner spur of hind tibia longer than outer spur (0.21, 0.18), entire surface of hind tibia with dense strong spines clearly differentiated by color and length. Hind telotarsus longer than fourth tarsomere (0.17, 0.14).

**Wings** (Fig. 157K). Fore wing with r vein slightly curved; 2RS vein straight; r and 2RS veins forming a weak, even curve at their junction and outer side of junction forming a slight stub; 2M vein straight; distally fore wing [where spectral veins are] with microtrichiae more densely concentrated than the rest of the wing; anal cell 1/3 proximally lacking microtrichiae; subbasal cell with microtrichiae virtually throughout; vein 2 cu-a present as spectral vein, sometimes difficult to see; vein 2-1A proximally tubular and distally spectral, although sometimes difficult to see; tubular vein 1 cu-a straight and complete, but junction with 1-1A vein spectral. Hind wing with vannal lobe very narrow, subdistally and subproximally evenly convex, and setae evenly scattered in the margin.

**Metasoma** (Fig. 157A, G, H, J). Metasoma cylindrical. Petiole on T1 finely sculptured distal, but only laterally, parallel-sided in proximal half and then narrowing (length 0.34, maximum width 0.20, minimum width 0.11), and with scattered pubescence concentrated in the first distal third. Lateral grooves delimiting the median area on T2 clearly defined and reaching the distal edge of T2 (length median area 0.22, length T2 0.22), lateral grooves deep, median area as broad as long (length 0.22, maximum width 0.22, minimum width 0.10); T2 with scarce pubescence throughout. T3 longer than T2 (0.26, 0.22) and with scattered pubescence throughout. Pubescence on hypopygium dense.

**Cocoons.** Unknown.

#### Comments.

In some females, the S4 and beyond medially are brown.

#### Male.

Similar in coloration to female.

#### Etymology.

May Roberta Berenbaum is a renowned American entomologist known for elucidating chemical mechanisms underlying interactions between insects and their food plants, including detoxification of natural and synthetic chemicals, and for applying ecological principles in developing sustainable management practices for natural and agricultural communities. Currently, she is a professor and head of the Department of Entomology at UIUC, IL, USA, and also Editor-in-Chief of Proceedings of the National Academy of Sciences of the USA.

#### Distribution.

Parasitized caterpillar was collected in Ecuador, Napo, Yanayacu Biological Station (Yanayacu Road), during August 2009 at 2,100 m in cloud forest.

#### Biology.

The lifestyle of this parasitoid species is gregarious.

#### Host.

Undetermined species of Noctuidae feeding on *Burmeisteraborgensis* (Campanulaceae). Caterpillar was collected in fourth instar.

### Glyptapanteles meganmiltonae

Taxon classificationAnimaliaHymenopteraBraconidae

Arias-Penna, sp. nov.

http://zoobank.org/39A0BDEB-DCEA-40D0-A05B-0807D73F3842

Figs 158 , 159

#### Female.

Body length 2.17 mm, antenna length 2.37 mm, fore wing length 2.58 mm.

#### Type material.

**Holotype**: COSTA RICA • 1♀; 06-SRNP-9041, DHJPAR0012683; Área de Conservación Guanacaste, Alajuela, Sector San Cristóbal, Finca San Gabriel; rain forest; 645 m; 10.87766, -85.39343; 05.xi.2006; Elda Araya leg.; caterpillar collected in third instar; cocoons adhered to the leaf substrate; adult parasitoids emerged on 16.xi.2006; (CNC). **Paratypes.** • 21 (4♀, 4♂) (0♀, 13♂); 06-SRNP-9041, DHJPAR0012683; same data as for holotype; (CNC).

#### Other material.

**Reared material.** COSTA RICA: *Área de Conservación Guanacaste*, *Guanacaste*, *Sector Del Oro*, *Quebrada Raíz*: • 1 (1♀, 0♂) (0♂, 0♀); 04-SRNP-55920, DHJPAR0004238; dry-rain intergrade forest; 280 m; 11.02865, -85.48669; 15.xi.2004; Lucia Ríos leg.; caterpillar collected in fourth instar; elongate ridged white bud-like cocoons adhered to the leaf substrate and formed on 20.xi.2004; adult parasitoids emerged on 27.xi.2004.

*Área de Conservación Guanacaste*, *Alajuela*, *Sector San Cristóbal*, *Finca San Gabriel*: • 16 (5♀, 2♂) (9♀, 0♂); 09-SRNP-6146, DHJPAR0038065; rain forest; 645 m; 10.87766, -85.39343; 20.xi.2009; Elda Araya leg.; caterpillar collected in fifth instar; medium fluffy white cocoons adhered together and adhered to the leaf substrate; adult parasitoids emerged on 01.xii.20069.

*Área de Conservación Guanacaste*, *Guanacaste*, *Sector Mundo Nuevo*, *Vado Miramonte*: • 16 (3♀, 1♂) (12♀, 0♂); 10-SRNP-57267, DHJPAR0041715; dry-rain intergrade forest; 305 m; 10.77175, -85.43400; 14.xi.2010; José Cortéz leg.; caterpillar collected in fifth instar; cocoons formed on 19.xi.2010; adult parasitoids emerged on 30.xi.2010. • 12 (0♀, 3♂) (0♀, 9♂); 10-SRNP-57355, DHJPAR0041650; same data as for preceding except: 15.xii.2010; caterpillar collected in fourth instar; cocoons formed on 20.xii.2010; adult parasitoid emerged on 28.xii.2010.

*Área de Conservación Guanacaste*, *Alajuela*, *Sector Rincón Rain Forest*, *Camino Albergue Oscar*: • 5 (2♀, 1♂) (2♀, 0♂); 10-SRNP-6685, DHJPAR0041625; 560 m; 10.87741, -85.32363; 10.xi.2010; Carolina Cano leg.; caterpillar collected in fifth instar; cocoons adhered to the leaf substrate and formed on 16.xi.2010; adult parasitoids emerged on 23.xi.2010. • 11 (1♀, 3♂) (0♀, 4♂); 10-SRNP-6687, DHJPAR0041834; same data as for preceding except: multiple white cocoons adhered in host cocoon; date of cocoons not reported; adult parasitoid emerged on 22.xi.2010. • 11 (3♀, 3♂) (3♀, 2♂); 10-SRNP-6688, DHJPAR0041620; same data as for preceding except: multiple white cocoons adhered in host cocoon; date of cocoons not reported. • 12 (3♀, 1♂) (8♀, 0♂); 10-SRNP-6689, DHJPAR0041839; same data as for preceding except: adult parasitoids emerged on 26.xi.2010.

*Área de Conservación Guanacaste*, *Alajuela*, *Sector Rincón Rain Forest*, *San Lucas*: • 1 (0♀, 0♂) (0♀, 1♂); 11-SRNP-41426, DHJPAR0043167; 320 m; 10.91847, -85.30338; 24.iii.2011; Anabelle Córdoba leg.; caterpillar collected in fourth instar; cocoons adhered to the leaf substrate; adult parasitoids emerged on 08.iv.2011. • 8 (2♀, 1♂) (5♀, 0♂); 11-SRNP-41427, DHJPAR0042884; same data as for preceding.

#### Diagnosis.

**Figure 158.**
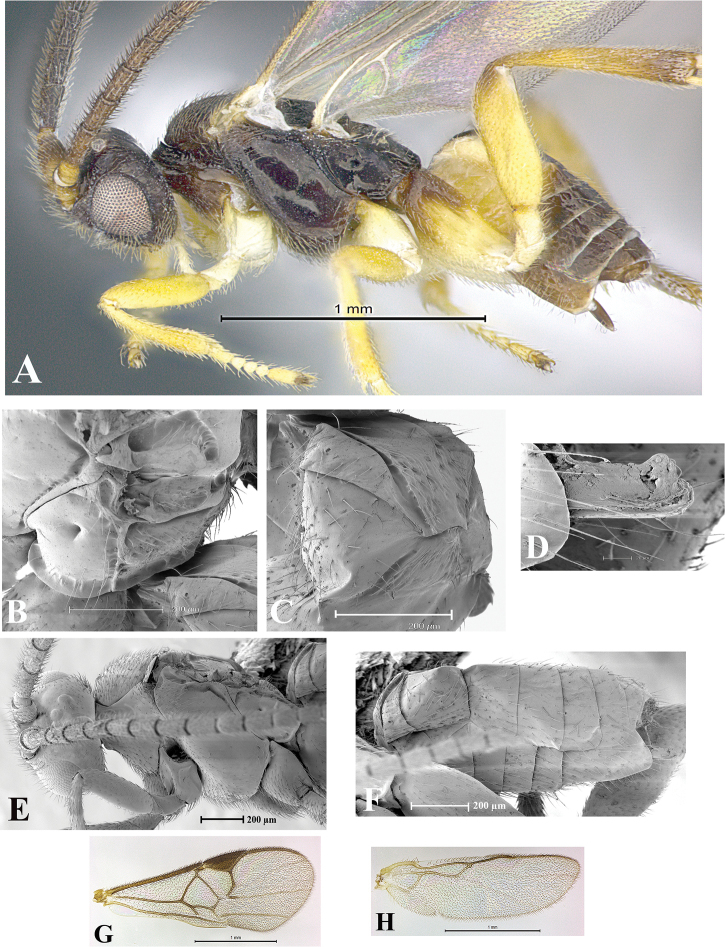
*Glyptapantelesmeganmiltonae* sp. nov. female 06-SRNP-9041 DHJPAR0012683 **A** Habitus **B** Scutellum, metanotum, propodeum, laterodorsal view **C**T1–2, dorsolateral view **D** Genitalia: hypopygium, ovipositor, ovipositor sheaths, lateral view **E** Head, mesosoma, lateral view **F** Metasoma, lateral view **G, H** Wings **G** Fore **H** Hind.

**Figure 159.**
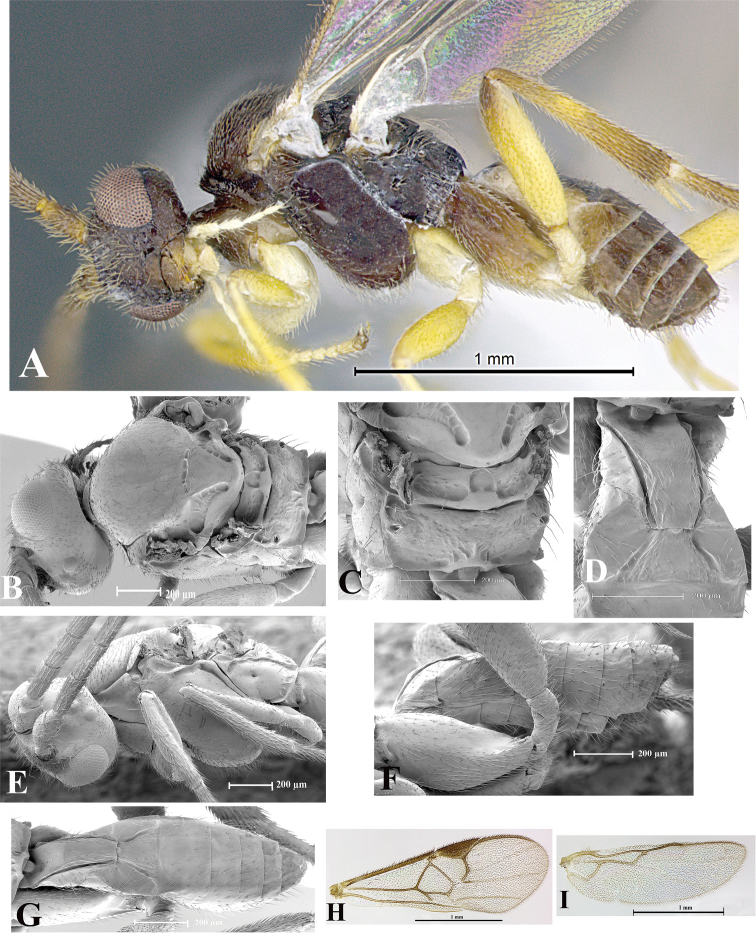
*Glyptapantelesmeganmiltonae* sp. nov. male 06-SRNP-9041 DHJPAR0012683 **A** Habitus **B, E** Head, mesosoma **B** Dorsal view **F** Lateral view **C** Scutellum, metanotum, propodeum, dorsal view **D**T1–2, dorsal view **F, G** Metasoma **F** Lateral view **G** Dorsal view **H, I** Wings **H** Fore **I** Hind.

Fore telotarsus basally narrow, apically wide (Fig. 158A), ventral margin with a tiny curved seta, medioposterior band of scutellum not overlapping the medioanterior pit of metanotum (Fig. 159B, C), phragma of the scutellum widely visible (Figs 158B, 159C), fore wing with vein 2 cu-a absent, r vein straight, outer side of junction of r and 2RS veins forming a stub (Figs 158G, 159H), median area on T2 broader than long (Fig. 159D), edges of median area on T2 obscured by weak longitudinal stripes (Figs 158C, 159D), and lateral grooves delimiting the median area on T2 distally losing definition on T2, vertex in dorsal view wide (Fig. 159E), in lateral view, metasoma laterally compressed (Figs 158F, 159F), T3 longer than T2 (Fig. 159G), inner margin of eyes diverging slightly at antennal sockets, petiole on T1 evenly narrowing distally (wide base to a narrow apex) and finely sculptured (Fig. 159D, G), and propodeum without a median longitudinal dent (Fig. 159C).

#### Coloration

(Fig. 158A). General body coloration dark brown except scape and pedicel yellow-brown with inner sides brown; first four-five proximal antennal flagellomeres dorsally lighter (light brown) than ventrally (dark brown), remaining flagellomeres dark brown on both sides; clypeus and labrum light brown; mandibles and tegulae dark yellow; glossa, maxillary and labial palps yellow; propleuron and both dorsal and ventral furrows of pronotum lighter than mesosoma coloration. Eyes and ocelli reddish (in preserved specimen). Fore and middle legs yellow except claws brown; hind legs yellow except coxae proximally with a edge light brown forming a irregular shape, a tiny brown spot in femora, tibiae distally light brown, and tarsomeres yellow-brown. Petiole on T1 with two colorations, proximal 1/3 reddish/yellow-brown and distal 2/3 brown, contours darkened and sublateral areas yellow; T2 with median and adjacent areas brown, and lateral ends yellow; T3 almost completely brown, but with a tiny yellow area in proximal corner; T4 and beyond completely brown; distally each tergum with a narrow yellowish transparent band. In lateral view, T1–2 completely yellow; T3 yellow with a small dorsodistal brown area; T4 and beyond brown. S1–4 yellow; penultimate sternum and hypopygium light brown/brown.

#### Description.

**Head** (Fig. 158A, E). Head rhomboid with pubescence long and dense. Proximal three antennal flagellomeres longer than wide (0.22:0.07, 0.18:0.07, 0.18:0.07), distal antennal flagellomere longer than penultimate (0.11:0.07, 0.08:0.07), antenna longer than body (2.37, 2.17); antennal scrobes-frons sloped and forming a shelf. Face flat or nearly so, with dense fine punctations, interspaces with microsculpture and longitudinal median carina present. Frons smooth. Temple wide, punctate and interspaces clearly smooth. Inner margin of eyes diverging slightly at antennal sockets; in lateral view, eye anteriorly convex and posteriorly straight. POL shorter than OOL (0.10, 0.12). Malar suture present. Median area between lateral ocelli without depression. Vertex laterally pointed or nearly so and dorsally wide.

**Mesosoma** (Fig. 158A, B, E). Mesosoma dorsoventrally convex. Mesoscutum proximally convex and distally flat, punctation distinct throughout, interspaces smooth. Scutellum long and slender, apex sloped and fused with BS, scutellar punctation indistinct throughout, in profile scutellum flat and on same plane as mesoscutum, phragma of the scutellum widely visible; BS not overlapping the MPM; ATS demilune inner side with a row of foveae; dorsal ATS groove with semicircular/parallel carinae. Transscutal articulation wih large and heterogeneous foveae, area just behind transscutal articulation smooth, shiny and depressed centrally. Metanotum with BM wider than PFM (clearly differentiated); MPM proximally circle and distally straight with a short distal carina; AFM with a small lobe and not as well delineated as PFM; PFM slim and smooth; ATM proximally with a groove with some sculpturing and distally smooth. Propodeum finely sculptured, without median longitudinal carina, proximal half curved; distal edge of propodeum with a flange at each side and without stubs; propodeal spiracle without distal carina; nucha surrounded by very short radiating carinae. Pronotum with a distinct dorsal furrow, dorsally with a well-defined smooth band; central area of pronotum and both dorsal and ventral furrows smooth. Propleuron with fine punctations throughout and dorsally without a carina. Metasternum flat or nearly so. Contour of mesopleuron convex; precoxal groove smooth, shiny and shallow, but visible; epicnemial ridge elongated more fusiform (tapering at both ends).

**Legs.** Ventral margin of fore telotarsus entire, but with a tiny curved seta, fore telotarsus proximally narrow and distally wide, and longer than fourth tarsomere (0.10, 0.05). Hind coxa with dorsal half sparsely punctate, ventral half densely punctate, and dorsal outer depression present. Inner spur of hind tibia longer than outer spur (0.21, 0.16), entire surface of hind tibia with dense strong spines clearly differentiated by color and length. Hind telotarsus as equal in length as fourth tarsomere (0.12, 0.11).

**Wings** (Fig. 158G, H). Fore wing with r vein straight; 2RS vein straight; r and 2RS veins forming an angle at their junction and outer side of junction forming a slight stub; 2M vein slightly curved/swollen; distally fore wing [where spectral veins are] with microtrichiae almost homogeneously distributed as the rest of the wing; anal cell 1/3 proximally lacking microtrichiae; subbasal cell with microtrichiae virtually throughout; veins 2CUa and 2CUb completely spectral; vein 2 cu-a absent; vein 2-1A proximally tubular and distally spectral, although sometimes difficult to see; tubular vein 1 cu-a straight, incomplete/broken and not reaching the edge of 1-1A vein. Hind wing with vannal lobe very narrow, subdistally and subproximally straightened, and setae evenly scattered in the margin.

**Metasoma** (Fig. 158A, C, D, F). Metasoma laterally compressed. Petiole on T1 finely sculptured throughout, evenly narrowing distally (length 0.30, maximum width 0.15, minimum width 0.09) and with scattered pubescence on distal half. Lateral grooves delimiting the median area on T2 clearly defined and reaching the distal edge of T2 (length median area 0.15, length T2 0.15), edges of median area obscured by weak longitudinal stripes, median area broader than long (length 0.15, maximum width 0.23, minimum width 0.07); T2 with scattered pubescence only distally. T3 longer than T2 (0.20, 0.15) and with scattered pubescence throughout. Pubescence on hypopygium dense.

**Cocoons.** White bud-like cocoons with medium fluffy silk fibers. Cocoons adhered to the leaf substrate or in host cocoon

#### Comments.

The antennal scrobes (frons) form a shelf-shaped, so this area is strongly sloped; the scape is very swollen with inner sides curved. Some specimens body with lighter pale brown coloration, although the color pattern can de distinguished, maybe they emerged early. In other females, the epicnemial ridge is lighter than mesosoma coloration. The penultimate sternum and the hypopygium are yellow-brown. Some females (e.g., 04-SRNP-55920) exhibit coxae completely dark brown and the hypopygium brown, but medially yellow-brown.

#### Male

(Fig. 159A–I). In some specimens (e.g., 10-SRNP-57355), the mesoscutum is reddish/dark yellow-brown, coloration taking the place of notauli; the coloration on these specimens is more reddish than yellow; in some specimens the hind coxa is completely light brown.

#### Etymology.

Megan Milton currently is a data and communication lead at Barcode of Life Data Systems (BOLD).

#### Distribution.

The parasitized caterpillars were collected in Costa Rica, ACG, Sector Mundo Nuevo (Vado Miramonte), Sector Rincón Rain Forest (Camino Albergue Oscar and San Lucas), Sector San Cristóbal (Finca San Gabriel), and Sector Del Oro (Quebrada Raíz), during November 2004, 2006, and 2009; November-December 2010; and March 2011 at 280 m, 305 m, 320 m, 560 m, and 645 m in dry-rain intergrade forest and rain forest.

#### Biology.

The lifestyle of this parasitoid species is solitary/gregarious.

#### Host.

*Herpetogramma* sp. Lederer (Crambidae: Spilomelinae) feeding on *Achyranthesaspera*, *A.indica* and *Alternantherapubiflora* (Amaranthaceae). Caterpillars were collected in third, fourth and fifth instar.

### Glyptapanteles mehrdadhajibabaei

Taxon classificationAnimaliaHymenopteraBraconidae

Arias-Penna, sp. nov.

http://zoobank.org/DDE8EFB0-8F3D-4FA4-828C-6713EF4981EE

Figs 160 , 161

#### Female.

Body length 2.02 mm, antenna length 2.37 mm, fore wing length 2.22 mm.

#### Type material.

**Holotype**: COSTA RICA • 1♀; 06-SRNP-3399, DHJPAR0005109; Área de Conservación Guanacaste, Alajuela, Sector San Cristóbal, Sendero Carmona; rain forest; 670 m; 10.87621, -85.38632; 24.iv.2006; Elda Araya leg.; caterpillar collected in fourth instar; cordwood cocoons adhered to the leaf substrate; adult parasitoids emerged on 04.v.2006; (CNC). **Paratypes.** • 20 (3♀, 3♂) (14♀, 0♂); 06-SRNP-3399, DHJPAR0005109; same data as for holotype; (CNC).

#### Diagnosis.

**Figure 160.**
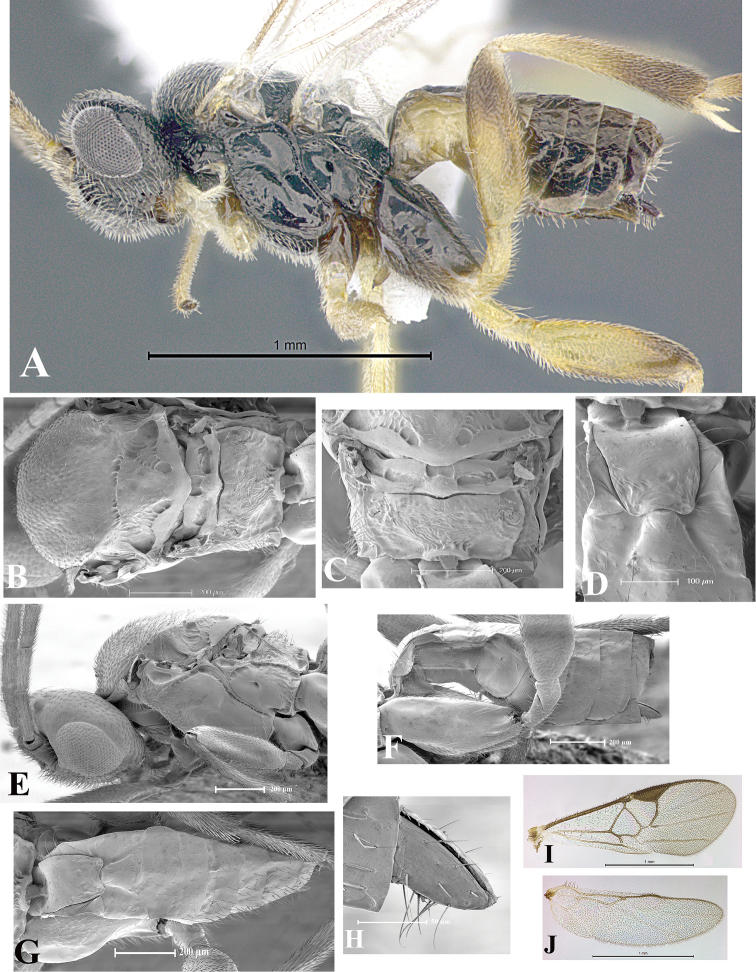
*Glyptapantelesmehrdadhajibabaei* sp. nov. female 06-SRNP-3399 DHJPAR0005109 **A** Habitus **B** Mesosoma, dorsal view **C** Scutellum, metanotum, propodeum, dorsal view **D**T1–2, dorsolateral view **E** Head, mesosoma, lateral view **F, G** Metasoma **F** Lateral view **G** Dorsal view **H** Genitalia: hypopygium, ovipositor, ovipositor sheaths, lateral view **I, J** Wings **I** Fore **J** Hind.

**Figure 161.**
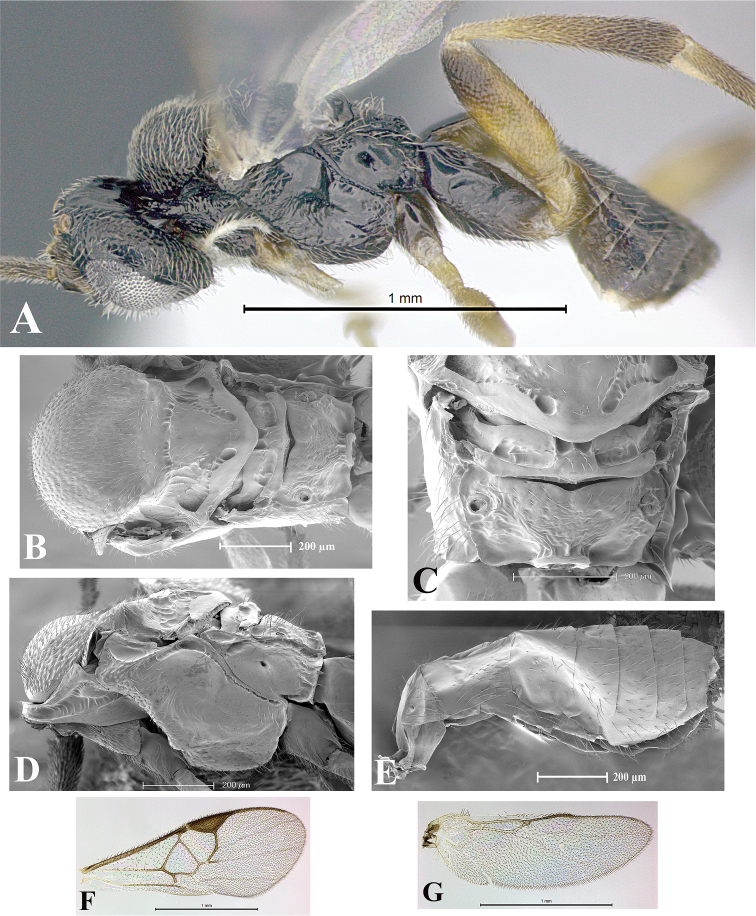
*Glyptapantelesmehrdadhajibabaei* sp. nov. male 06-SRNP-3399 DHJPAR0005109 **A** Habitus **B, D** mesosoma **B** Dorsal view **D** Lateral view **C** Scutellum, metanotum, propodeum, dorsal view **E** Metasoma, lateral view **F, G** Wings **F** Fore **G** Hind.

Inner spur of hind tibia slightly longer than outer spur, median area on T2 as broad as long (Fig. 160D, G), propodeal spiracle distally framed by a short concave carina (Figs 160C, 161C), petiole on T1 distally with lateral margins relatively straight, finely sculptured only distally (Fig. 160D, G), surface of metasternum flat or nearly so, precoxal groove deep with lineate sculpture (Figs 160A, 161A, D), fore wing with vein 1 cu-a curved, r vein curved, outer side of junction of r and 2RS veins not forming a stub (Figs 160I, 161F), dorsal outer depression on hind coxa present (Figs 160A, F, 161A), inner margin of eyes diverging slightly at antennal sockets, propodeum with transverse rugae (Figs 160C, 161C), and lateral grooves delimiting the median area on T2 clearly defined and reaching the distal edge of T2 (Fig. 160D, G).

#### Coloration

(Fig. 160A). General body coloration shiny black except brown scape, but proximally yellow-brown; first four-five proximal antennal flagellomeres lighter (light brown) dorsally than ventrally (dark brown), remaining flagellomeres dark brown on both sides; pedicel, labrum, and mandibles yellow-brown/reddish; glossa, maxillary and labial palps, and tegulae yellow. Eyes gray and ocelli reddish (in preserved specimen). Fore and middle legs yellow except brown claws; hind legs yellow except black coxae, distal 1/3 of femora brown, distal half of tibiae brown, and tarsomeres brown although basitarsus proximally with a yellow-brown ring. Petiole on T1 1/3 proximal reddish and 2/3 distal black, contours darkened and sublateral areas yellow-brown; T2 with median and adjacent areas brown, and lateral ends yellow-brown; T3 and beyond completely brown; distally each tergum with a narrow yellowish transparent band. In lateral view, T1–3 yellow-brown; T4 and beyond brown. S1–3 yellow-brown; S4 and beyond proximally yellow-brown, distally brown.

#### Description.

**Head** (Fig. 160A, E). Head rhomboid with pubescence long and dense. Proximal three antennal flagellomeres longer than wide (0.19:0.06, 0.18:0.06, 0.18:0.06), distal antennal flagellomere longer than penultimate (0.11:0.04, 0.08:0.04), antenna longer than body (2.37, 2.02); antennal scrobes-frons sloped and forming a shelf. Face with lateral depression, punctate-lacunose, interspaces wavy and longitudinal median carina present. Frons smooth. Temple wide, punctate-lacunose and interspaces wavy. Inner margin of eyes diverging slightly at antennal sockets; in lateral view, eye anteriorly convex and posteriorly straight. POL shorter than OOL (0.09, 0.13). Malar suture absent or difficult to see. Median area between lateral ocelli without depression. Vertex laterally rounded and dorsally wide.

**Mesosoma** (Fig. 160A–C, E). Mesosoma dorsoventrally convex. Distal 1/3 of mesoscutum with lateral margin slightly dented, proximally with distinctive punctation, but distally with a polished area, interspaces wavy/lacunose. Scutellum triangular, apex sloped and fused with BS, scutellar punctation scattered throughout, in profile scutellum flat and on same plane as mesoscutum, phragma of the scutellum partially exposed; BS only very partially overlapping the MPM; ATS demilune with quite a little, complete and parallel carinae; dorsal ATS groove with semicircular/parallel carinae. Transscutal articulation with small and heterogeneous foveae, area just behind transscutal articulation smooth, shiny and nearly at the same level as mesoscutum (flat). Metanotum with BM wider than PFM (clearly differentiated); MPM semicircular without median longitudinal carina; AFM with a small lobe and not as well delineated as PFM; PFM thick, smooth and with lateral ends rounded; ATM proximally with a groove with some sculpturing and distally smooth. Propodeum with transverse rugae, proximal half weakly curved with medium-sized sculpture and distal half with a shallow dent at each side of nucha or rugose; distal edge of propodeum with a flange at each side and without stubs; propodeal spiracle distally framed by a short concave carina; nucha surrounded by very short radiating carinae. Pronotum with a distinct dorsal furrow, dorsally with a well-defined smooth band; central area of pronotum smooth, but both dorsal and ventral furrows with short parallel carinae. Propleuron with fine rugae and dorsally without a carina. Metasternum flat or nearly so. Contour of mesopleuron straight/angulate or nearly so; precoxal groove deep with faintly transverse lineate sculpture; epicnemial ridge elongated more fusiform (tapering at both ends).

**Legs.** Ventral margin of fore telotarsus slightly excavated and with a tiny curved seta, fore telotarsus almost same width throughout and longer than fourth tarsomere (0.11, 0.08). Hind coxa with punctation only on ventral surface, dorsal outer depression present. Inner spur of hind tibia longer than outer spur (0.18, 0.16), entire surface of hind tibia with dense strong spines clearly differentiated by color and length. Hind telotarsus as equal in length as fourth tarsomere (0.11, 0.10).

**Wings** (Fig. 160I, J). Fore wing with r vein slightly curved; 2RS vein straight; r and 2RS veins forming an angle at their junction and outer side of junction not forming a stub; 2M vein slightly curved/swollen; distally fore wing [where spectral veins are] with microtrichiae more densely concentrated than the rest of the wing; anal cell 1/3 proximally lacking microtrichiae; subbasal cell with microtrichiae virtually throughout; veins 2CUa and 2CUb completely spectral; vein 2 cu-a absent; vein 2-1A proximally tubular and distally spectral, although sometimes difficult to see; tubular vein 1 cu-a curved, incomplete/broken and not reaching the edge of 1-1A vein. Hind wing with vannal lobe very narrow, subdistally and subproximally straightened, and setae evenly scattered in the margin.

**Metasoma** (Fig. 160A, D, F–H). Metasoma laterally compressed. Petiole on T1 finely sculptured only distally, parallel-sided in proximal half and then narrowing (length 0.31, maximum width 0.17, minimum width 0.10) and with scattered pubescence concentrated in the first distal third. Lateral grooves delimiting the median area on T2 clearly defined and reaching the distal edge of T2 (length median area 0.13, length T2 0.13), edges of median area polished and lateral grooves deep, median area as broad as long (length 0.13, maximum width 0.13, minimum width 0.09); T2 with scattered pubescence only distally. T3 longer than T2 (0.22, 0.13) and with scattered pubescence throughout. Pubescence on hypopygium dense.

**Cocoons** (Fig. 4Z). Light brown oval cocoons with ordered silk fibers, but covered by a net. Cordwood cocoons adhered to the leaf substrate.

#### Comments.

Both sexes with slim body.

#### Male

(Fig. 161A–G). Similar in coloration and shape to female.

#### Etymology.

Mehrdad Hajibabaei is an expert in molecular evolutionary biology and bioinformatics. He has been one of the pioneers in the use of high-throughput genomics technologies, such as microarrays and Next-Generation Sequencing (NGS) for the assessment of biodiversity in samples as varied as natural health products to bulk environmental samples. He is an Assistant Professor at Biodiversity Institute of Ontario (BIO), University of Guelph, Ontario, Canada.

#### Distribution.

The parasitized caterpillar was collected in Costa Rica, ACG, Sector San Cristóbal (Sendero Carmona), during April 2006 at 670 m in rain forest.

#### Biology.

The lifestyle of this parasitoid species is gregarious.

#### Host.

*Carathisseptentrionalis* Becker (Erebidae: Arctiinae) (Fig. 4Z) feeding on *Nectandramartinicensis* (Lauraceae). Caterpillar was collected in fourth instar.

### Glyptapanteles michelleduennesae

Taxon classificationAnimaliaHymenopteraBraconidae

Arias-Penna, sp. nov.

http://zoobank.org/03BFB6CB-BCC2-4090-A0F2-015F5601E5E0

Fig. 162

#### Female.

Body length 2.48 mm, antenna length 2.78 mm, fore wing length 3.03 mm.

#### Type material.

**Holotype**: ECUADOR • 1♀; EC-36028, YY-A061; Napo, Yanayacu Biological Station, Yanayacu Road; cloud forest; 2,100 m; -0.566667, -77.866667; 13.xi.2008; CAPEA leg.; caterpillar collected in third instar; cocoons formed on 17.xii.2008; adult parasitoids emerged on 29.xii.2008; (PUCE). **Paratypes.** • 7 (2♀, 4♂) (1♀, 0♂); EC-36028, YY-A061; same data as for holotype; (PUCE).

#### Diagnosis.

**Figure 162.**
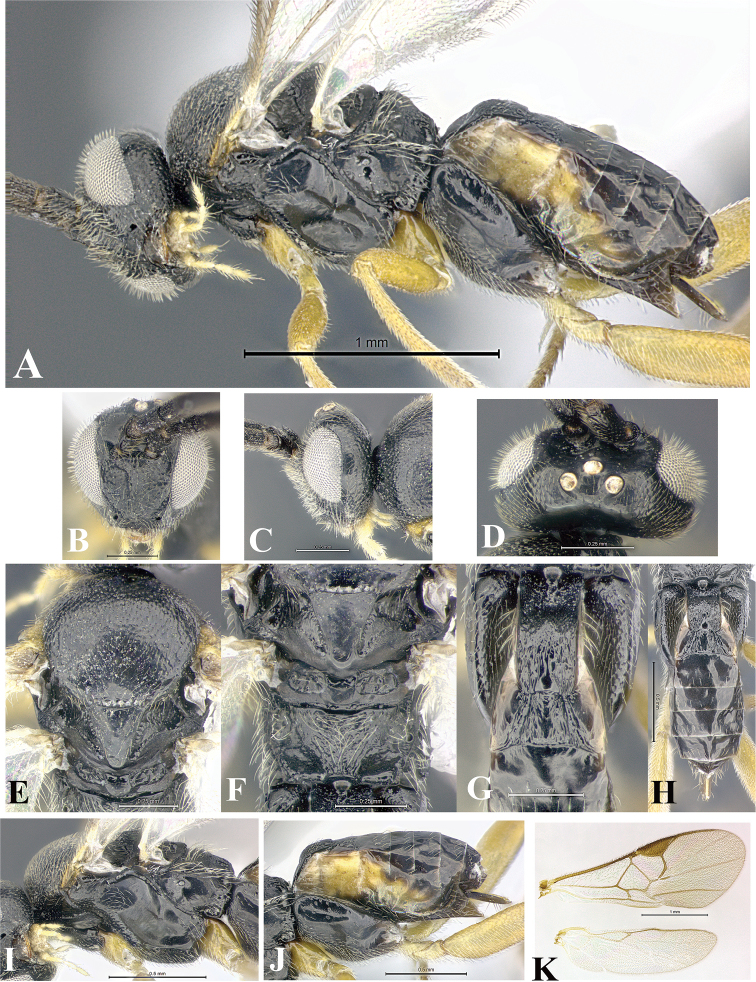
*Glyptapantelesmichelleduennesae* sp. nov. female EC-36028 YY-A061 **A** Habitus **B–D** Head **B** Frontal view **C** Lateral view **D** Dorsal view **E** Mesonotum, dorsal view **F** Scutellum, metanotum, propodeum, dorsal view **G**T1–2, dorsal view **H, J** Metasoma **H** Dorsal view **J** Lateral view **I** Mesosoma, lateral view **K** Fore and hind wings.

In lateral view, metasoma cylindrical (Fig. 162A, J), hind coxa punctate only on ventral surface (Fig. 162A, J), propodeum with a transverse discontinuous carina only present laterally (Fig. 162F), petiole on T1 virtually parallel-sided, but narrowing at apex (Fig. 162G, H), scutellar punctation indistinct throughout (Fig. 162E, F), edges of median area on T2 obscured by weak longitudinal stripes (Fig. 162G), dorsal outer depression on hind coxa present (Fig. 162A, J), and fore wing with r vein curved, outer side of junction of r and 2RS veins forming a slight stub (Fig. 162K).

#### Coloration

(Fig. 162A–K). General body coloration polished black except mandibles brown/reddish; glossa, maxillary and labial palps, and tegulae yellow; pedicel distally yellow-reddish; scape and all antennal flagellomeres (on both sides) dark brown/black. Eyes silver and ocelli yellowish. Fore and middle legs dark yellow, and claws brown, although tibiae and tarsomeres with brown tints; hind legs dark yellow except black coxae, femora dorsally with a tiny brown spot, tibiae distally brown and proximally with a narrow brown band, and tarsomeres brown. Petiole on T1 black and sublateral areas yellow-brown; T2 with median area black, wide adjacent area dark brown/black, limits of adjacent area with lateral ends not clearly defined; T3 and beyond completely dark brown/black; distally each tergum with a narrow whitish transparent band. In lateral view, T1-–2 yellow; T3 and beyond yellow, but dorsally brown, extent of brown area increasing from proximal to distal, so distal terga completely brown. S1–3 yellow; S4 and beyond brown.

#### Description.

**Head** (Fig. 162A–D). Head rounded pubescence long and dense. Proximal three antennal flagellomeres longer than wide (0.22:0.08, 0.22:0.08, 0.23:0.08), distal antennal flagellomere longer than penultimate (0.14:0.06, 0.10:0.06), antenna longer than body (2.78, 2.48); antennal scrobes-frons sloped and forming a shelf. Face with dense fine punctations, distal half dented only laterally, interspaces with microsculpture and longitudinal median carina present. Frons smooth. Temple wide, punctate-lacunose and interspaces wavy. Inner margin of eyes diverging slightly at antennal sockets; in lateral view, eye anteriorly convex and posteriorly straight. POL shorter than OOL (0.08, 0.15). Malar suture present. Median area between lateral ocelli slightly depressed. Vertex laterally pointed or nearly so and dorsally wide.

**Mesosoma** (Fig. 162A, E, F, I). Mesosoma dorsoventrally convex. Mesoscutum proximally convex and distally flat, punctation distinct throughout, interspaces wavy/lacunose. Scutellum triangular, apex sloped and fused with BS, but not in the same plane, scutellar punctation indistinct throughout, in profile scutellum flat and on same plane as mesoscutum, phragma of the scutellum completely concealed; BS mostly overlapping the MPM; ATS demilune inner side with a row of foveae; dorsal ATS groove with semicircular/parallel carinae. Transscutal articulation with small and heterogeneous foveae, area just behind transscutal articulation sloped and with same kind of sculpture as mesoscutum. Metanotum with BM wider than PFM (clearly differentiated); MPM semicircular and bisected by a median longitudinal carina; AFM without setiferous lobes and not as well delineated as PFM; PFM thick, smooth and with lateral ends rounded; ATM proximally with a groove with some sculpturing and distally smooth. Propodeum with a median longitudinal dent, but no trace of median longitudinal carina, proximal half curved with medium-sized sculpture and distal half with a shallow dent at each side of nucha; distal edge of propodeum with a flange at each side and without stubs; propodeal spiracle distally framed by a short transverse carina; nucha surrounded by long radiating carinae. Pronotum with a distinct dorsal furrow, dorsally with a defined smooth band only proximally; central area of pronotum and dorsal furrow smooth, but ventral furrow with short parallel carinae. Propleuron finely sculptured only ventrally and dorsally without a carina. Metasternum flat or nearly so. Contour of mesopleuron straight/angulate or nearly so; precoxal groove smooth, shiny and shallow, but visible; epicnemial ridge convex, teardrop-shaped.

**Legs.** Ventral margin of fore telotarsus slightly excavated and with a tiny curved seta, fore telotarsus almost same width throughout and longer than fourth tarsomere (0.15, 0.08). Hind coxa with punctation only on ventral surface, dorsal outer depression present. Inner spur of hind tibia longer than outer spur (0.35, 0.21), entire surface of hind tibia with dense strong spines clearly differentiated by color and length. Hind telotarsus as equal in length as fourth tarsomere (0.13, 0.13).

**Wings** (Fig. 162K). Fore wing with r vein slightly curved; 2RS vein straight; r and 2RS veins forming a weak, even curve at their junction and outer side of junction forming a slight stub; 2M vein slightly curved/swollen; distally fore wing [where spectral veins are] with microtrichiae more densely concentrated than the rest of the wing; anal cell 1/3 proximally lacking microtrichiae; subbasal cell with a small smooth area; veins 2CUa and 2CUb completely spectral; vein 2 cu-a present as spectral vein, sometimes difficult to see; vein 2-1A proximally tubular and distally spectral, although sometimes difficult to see; tubular vein 1 cu-a straight and complete, but junction with 1-1A vein spectral. Hind wing with vannal lobe narrow, subdistally and subproximally straightened, and setae absent proximally, but scattered distally.

**Metasoma** (Fig. 162A, G, H, J). Metasoma cylindrical. Petiole on T1 with a mix of fine rugae and punctate sculpture over most of the surface, parallel-sided over most of length, but barely narrowing at apex, apex truncate (length 0.35, maximum width 0.20, minimum width 0.18), and with scattered pubescence concentrated in the first distal third. Lateral grooves delimiting the median area on T2 clearly defined and reaching the distal edge of T2 (length median area 0.15, length T2 0.15), edges of median area obscured by weak longitudinal stripes, median area broader than long (length 0.15, maximum width 0.23, minimum width 0.12); T2 with scattered pubescence only distally. T3 longer than T2 (0.22, 0.15) and with pubescence more notorious in distal half. Pubescence on hypopygium scattered.

**Cocoons.** Unknown.

#### Comments.

The antenna is curled. The median ocellus is very close to lateral ocelli (Fig. 162D, diameter of median ocellus 0.07 mm, the distance between median and lateral ocellus is 0.02 mm); the propodeum with a transverse discontinuous carina only present laterally; distally the propodeum forming a wall (Fig. 162F); the pronotum is very deep, at different plane than mesopleuron (Fig. 162A, I); the body is stout. This species looks like *Parapanteles* because of petiole shape (parallel sides, Fig. 162G) and distal half of propodeum (each lateral side with a deep dent, Fig. 162F).

#### Male.

Similar in coloration to females.

#### Etymology.

Michelle (Poly Nator) Audrey Duennes’ research at UIUC, IL, USA, was focused on the phylogenetic relationships among a New World bumble bee species complex. Currently, she is assistant professor at St. Vincent College, Latrobe, PA, USA

#### Distribution.

Parasitized caterpillar was collected in Ecuador, Napo, Yanayacu Biological Station (Yanayacu Road), during November 2008 at 2,100 m in cloud forest.

#### Biology.

The lifestyle of this parasitoid species is gregarious.

#### Host.

Undetermined species of Pantheidae feeding on *Rubus* sp. (Rosaceae). Caterpillar was collected in third instar.

### Glyptapanteles mikegatesi

Taxon classificationAnimaliaHymenopteraBraconidae

Arias-Penna, sp. nov.

http://zoobank.org/13BD99CF-1AE0-4378-8ACF-6B8BCEAD0182

Figs 163 , 164

#### Female.

Body length 2.02 mm, antenna length 2.27 mm, fore wing length 2.02 mm.

#### Type material.

**Holotype**: COSTA RICA • 1♀; 06-SRNP-45871, DHJPAR0012107; Área de Conservación Guanacaste, Guanacaste, Sector Cacao, Cuesta Caimito; cloud forest; 640 m; 10.89080, -85.47192; 06.vii.2006; Yendry Ruiz leg.; caterpillar collected in fourth instar; small dark cocoons adhered to the larval cuticle and formed on 10.vii.2006; adult parasitoids emerged on 16.vii.2006; (CNC). **Paratypes.** • 41 (5♀, 5♂) (24♀, 7♂); 06-SRNP-45871, DHJPAR0012107; same data as for holotype; (CNC).

#### Other material.

**Reared material.** COSTA RICA: *Área de Conservación Guanacaste*, *Guanacaste*, *Sector Cacao*, *Quebrada Otilio*: • 2 (1♀, 0♂) (1♀, 0♂); 05-SRNP-45887, DHJPAR0004770; cloud forest; 550 m; 10.88996, -85.47966; 15.vi.2005; Dunia Garcia leg.; caterpillar collected in fifth instar; cocoons adhered to the leaf substrate; adult parasitoids emerged on 28.vi.2005.

#### Diagnosis.

**Figure 163.**
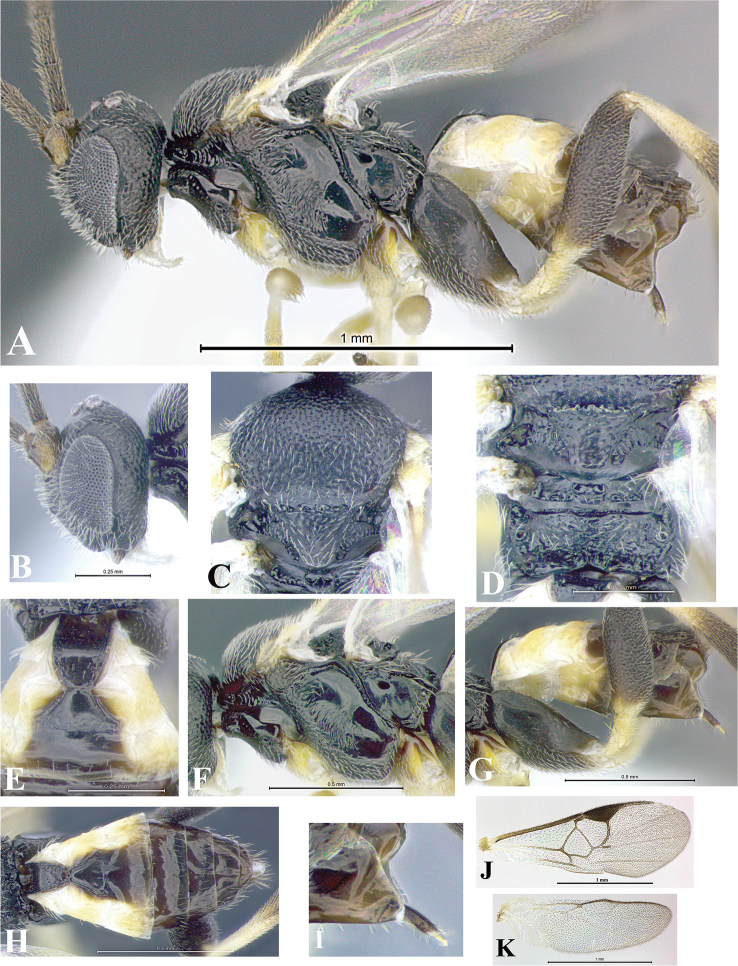
*Glyptapantelesmikegatesi* sp. nov. female 06-SRNP-45871 DHJPAR0012107 **A** Habitus **B** Head, lateral view **C** Mesonotum, dorsal view **D** Scutellum, metanotum, propodeum, dorsal view **E**T1–2, dorsal view **F** Mesosoma, lateral view **G, H** Metasoma **G** Lateral view **H** Dorsal view **I** Genitalia: hypopygium, ovipositor,ovipositor sheaths, lateral view **J, K** Wings **J** Fore **K** Hind.

**Figure 164.**
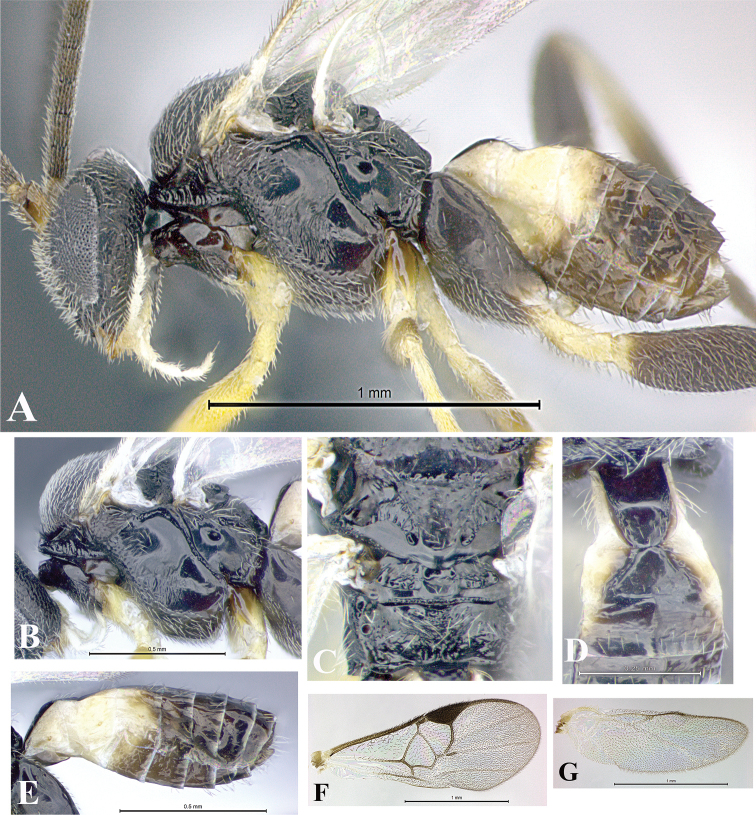
*Glyptapantelesmikegatesi* sp. nov. male 06-SRNP-45871 DHJPAR0012107 **A** Habitus **B** Mesosoma, lateral view **C** Scutellum, metanotum, propodeum, dorsal view **D**T1–2, dorsal view E Metasoma, lateral view **F, G** Wings **F** Fore **G** Hind.

Fore telotarsus proximally narrow, distally wide, medioposterior band of scutellum mostly overlapping the medioanterior pit of metanotum (Figs 163D, 164C), petiole on T1 distally with lateral margins curved (convex), finely sculptured only laterally (Figs 163E, H, 164D), surface of metasternum flat or nearly so, precoxal groove deep with lineate sculpture (Figs 163A, F, 164A, B), fore wing with vein 1 cu-a curved, r vein curved, outer side of junction of r and 2RS veins not forming a stub (Figs 163J, 164F), dorsal outer depression on hind coxa present (Figs 163A, G, 164A), inner margin of eyes diverging slightly at antennal sockets, propodeum without median longitudinal carina (Figs 163D, 164C), and lateral grooves delimiting the median area on T2 clearly defined and reaching the distal edge of T2 (Figs 163E, H, 164D).

#### Coloration

(Fig. 163A–K). General body coloration polished black except scape and pedicel yellow-brown with lateral brown band; last seven-eight distal antennal flagellomeres lighter (light brown) than remaining flagellomeres (dark brown); labrum and mandible yellow-brown; glossa, maxillary and labial palps, and tegulae yellow; both dorsal and ventral furrows of pronotum lighter than mesosoma coloration. Eyes gray and ocelli yellowish. Fore and middle legs yellow except middle coxae proximally with a small brown area, and claws brown; hind legs black except trochanters, trochantellus, distal 1/3 of tibiae yellow, and basitarsus proximally with a narrow yellow band. Petiole on T1 black, contours darkened and sublateral areas yellow; T2 with median and adjacent areas brown, adjacent area very narrow, and lateral ends yellow; T3 with a brown area, which width coinciding with the width of median area plus adjacent area on T2, thus brown coloration from T2-–3 looks like a large pyramid-shaped, and lateral ends yellow; T4 and beyond completely brown; distally each tergum with a narrow yellowish transparent band. In lateral view, T1–3 yellow; T4 and beyond brown. S1–3 yellow; S4 proximal half yellow, distal half brown; penultimate sternum and hypopygium brown.

#### Description.

**Head** (Fig. 163A, B). Head rhomboid with pubescence long and dense. Proximal three antennal flagellomeres longer than wide (0.16:0.05, 0.16:0.05, 0.17:0.05), distal antennal flagellomere longer than penultimate (0.10:0.05, 0.08:0.05), antenna longer than body (2.27, 20.02); antennal scrobes-frons sloped and forming a shelf. Face flat or nearly so, punctate-lacunose, interspaces wavy and longitudinal median carina present. Frons smooth. Temple wide, punctate-lacunose and interspaces wavy. Inner margin of eyes diverging slightly at antennal sockets; in lateral view, eye anteriorly convex and posteriorly straight. POL shorter than OOL (0.09, 0.11). Malar suture present. Median area between lateral ocelli slightly depressed. Vertex laterally pointed or nearly so and dorsally wide.

**Mesosoma** (Fig. 163A, C, D, F). Mesosoma dorsoventrally convex. Mesoscutum proximally convex and distally flat, punctation distinct throughout, interspaces wavy/lacunose. Scutellum triangular, apex sloped and fused with BS, scutellar punctation distinct throughout, in profile scutellum flat and on same plane as mesoscutum, phragma of the scutellum partially exposed; BS mostly overlapping the MPM; ATS demilune with quite a little, complete and parallel carinae; dorsal ATS groove with semicircular/parallel carinae. Transscutal articulation with small and heterogeneous foveae, area just behind transscutal articulation with a smooth and shiny sloped transverse strip. Metanotum with BM wider than PFM (clearly differentiated); MPM semicircular and bisected by a median longitudinal carina; AFM with a small lobe and not as well delineated as PFM; PFM thick, smooth and with lateral ends rounded; ATM proximally with a groove with some sculpturing and distally smooth. Propodeum without median longitudinal carina, proximal half curved with medium-sized sculpture and distal half slightly rugose; distal edge of propodeum with a flange at each side and short stubs; propodeal spiracle distally framed by a short concave carina; nucha surrounded by very short radiating carinae. Pronotum with a distinct dorsal furrow, dorsally with a well-defined smooth band; central area of pronotum smooth, but both dorsal and ventral furrows with short parallel carinae. Propleuron with fine punctations throughout and dorsally with a carina. Metasternum flat or nearly so. Contour of mesopleuron straight/angulate or nearly so; precoxal groove deep with transverse lineate sculpture; epicnemial ridge elongated more fusiform (tapering at both ends).

**Legs.** Ventral margin of fore telotarsus slightly excavated and with a tiny curved seta, fore telotarsus proximally narrow and distally wide, and longer than fourth tarsomere (0.11, 0.06). Hind coxa with punctation only on ventral surface, dorsal outer depression present. Inner spur of hind tibia longer than outer spur (0.20, 0.14), entire surface of hind tibia with dense strong spines clearly differentiated by color and length. Hind telotarsus as equal in length as fourth tarsomere (0.10, 0.09).

**Wings** (Fig. 163J, K). Fore wing with r vein curved; 2RS vein straight; r and 2RS veins forming a weak, even curve at their junction and outer side of junction not forming a stub; 2M vein slightly curved/swollen; distally fore wing [where spectral veins are] with microtrichiae more densely concentrated than the rest of the wing; anal cell 1/3 proximally lacking microtrichiae; subbasal cell with a small smooth area; vein 2CUa absent and vein 2CUb spectral; vein 2 cu-a absent; vein 2-1A proximally tubular and distally spectral, although sometimes difficult to see; tubular vein 1 cu-a curved and complete, but junction with 1-1A vein spectral. Hind wing with vannal lobe very narrow, subdistally and subproximally straightened, and setae evenly scattered in the margin.

**Metasoma** (Fig. 163A, E, G–I). Metasoma laterally compressed. Petiole on T1 finely sculptured only laterally, virtually parallel-sided over most of length, but narrowing over distal 1/3 (length 0.25, maximum width 0.14, minimum width 0.10), and with scattered pubescence on distal half only laterally. Lateral grooves delimiting the median area on T2 clearly defined and reaching the distal edge of T2 (length median area 0.12, length T2 0.12), edges of median area polished and lateral grooves deep, median area broader than long (length 0.12 mm, maximum width 0.18, minimum width 0.07); T2 with scattered pubescence only distally. T3 longer than T2 (0.17, 0.12) and with scattered pubescence only distally. Pubescence on hypopygium scattered.

**Cocoons** (Fig. 4F). Brown oval cocoons with evenly smooth silk fibers. Cocoons adhered to the larval cuticle or to the leaf substrate.

#### Male

(Fig. 164A–G). Similar in coloration and shape to female.

#### Etymology.

Michael (Mike) Williams Gates is interested in morphology, natural history, phylogeny, systematics, and taxonomy of Chalcidoidea (Hymenoptera). He works at the Smithsonian Institution, Washington, DC., USA.

#### Distribution.

The parasitized caterpillars were collected in Costa Rica, ACG, Sector Cacao (Cuesta Caimito and Quebrada Otilio), during June 2005 and July 2006 at 550 m and 640 m in cloud forest.

#### Biology.

The lifestyle of this parasitoid species is gregarious.

#### Host.

*Pero* sp. Herrich-Schäffer (Geometridae: Ennominae) (Fig. 4F) feeding on *Cyathulaachyranthoides* (Amaranthaceae) and undetermined species of plant. Caterpillars were collected in fourth and fifth instar.

### Glyptapanteles mikepoguei

Taxon classificationAnimaliaHymenopteraBraconidae

Arias-Penna, sp. nov.

http://zoobank.org/7638E2C3-A3CE-4C15-9337-E6CCAF358D09

Fig. 165

#### Female.

Body length 2.78 mm, antenna length 3.03 mm, fore wing length 3.13 mm.

#### Type material.

**Holotype**: ECUADOR • 1♀; EC-31293, YY-A059; Napo, Yanayacu Biological Station, Road to San Rafael Waterfall; cloud forest; 1,288 m; -0.083333, -77.583333; 19.v.2008; CAPEA leg.; caterpillar collected in first instar; cocoons formed on 07.vii.2008; adult parasitoids emerged on 24.vii.2008; (PUCE). **Paratypes.** • 16 (5♀, 4♂) (7♀, 0♂); EC-31293, YY-A059; same data as for holotype; (PUCE).

#### Diagnosis.

**Figure 165.**
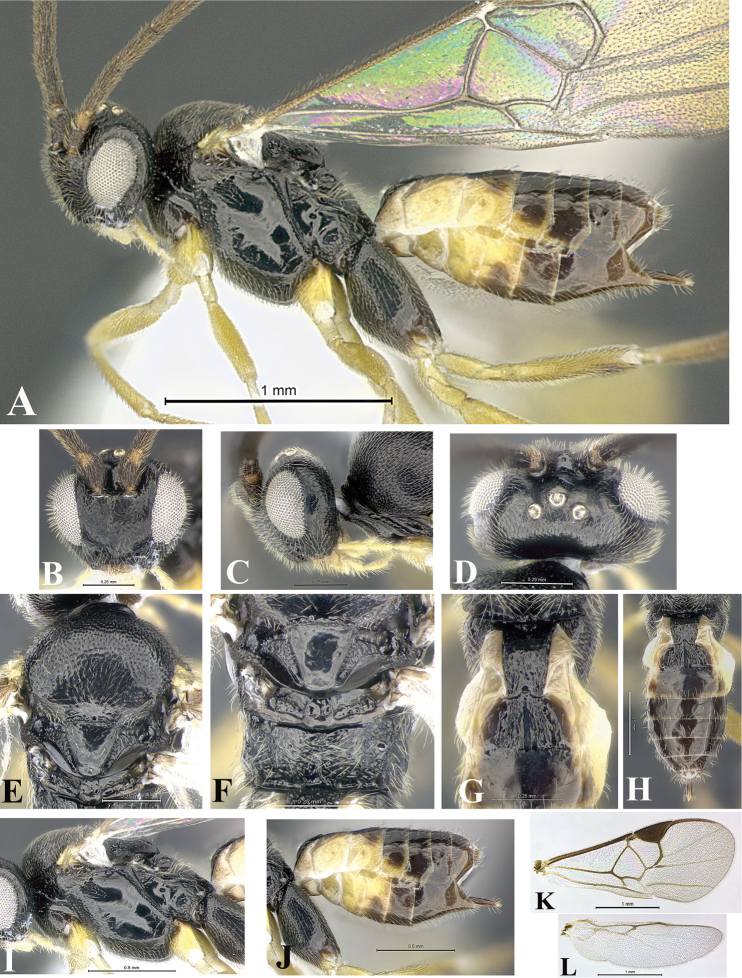
*Glyptapantelesmikepoguei* sp. nov. female EC-31293 YY-A059 **A** Habitus **B–D** Head **B** Frontal view **C** Lateral view **D** Dorsal view **E** Mesonotum, dorsal view **F** Scutellum, metanotum, propodeum, dorsal view **G**T1–3, dorsal view **H, J** Metasoma **H** Dorsal view **J** Lateral view **I** Mesosoma, lateral view **K, L** Wings **K** Fore **L** Hind.

Ventral margin of fore telotarsus slightly excavated and with a tiny curved seta, anteroventral contour of mesopleuron convex (Fig. 165A, I), propleuron with fine punctations throughout (Fig. 165A, I), longitudinal median carina on face present (Fig. 165B), surface of metasternum convex, edges of median area on T2 obscured by weak longitudinal stripes (Fig. 165G, H), dorsal outer depression on hind coxa absent (Fig. 165A, J), and fore wing with r vein slightly curved, outer side of junction of r and 2RS veins forming a stub (Fig. 165K).

#### Coloration

(Fig. 165A–L). General body coloration polished black except pedicel yellow-brown; scape and all antennal flagellomeres brown on both sides; labrum and mandibles yellow-brown; glossa maxillary and labial palps, and tegulae yellow; clypeus, dorsal furrow of pronotum, dorsal ATS groove, lunules, BS, PFM, BM, and lateral ends of metanotum with a slightly brown-red/reddish tints. Eyes and ocelli silver. Fore and middle legs yellow except brown claws, and middle coxae proximally with a brown spot; hind legs yellow except black coxae, distal 1/3 of femora brown, additionally with a narrow dorsal brown strip from top to bottom; tibia brown with 1/3 distal yellow, and tarsomeres brown, although basitarsus proximally with a yellow band. Petiole on T1 black and sublateral areas yellow; T2 with median and adjacent areas brown, silhouette of adjacent area well-defined, and lateral ends yellow; T3 mostly brown, proximally dark area coincides with the width of median and adjacent area onT2, and proximal half of lateral ends yellow; T4 and beyond completely brown; distally each tergum with a narrow yellow translucent band. In lateral view, T1–3 completely yellow; T4 yellow, but dorsally brown; T5 and beyond brown. S1–3 yellow; S4 yellow, but medially brown; penultimate sternum and hypopygium brown.

#### Description.

**Head** (Fig. 165A–D). Head rounded with pubescence long and dense. Proximal three antennal flagellomeres longer than wide (0.22:0.06, 0.25:0.06, 0.23:0.06), distal antennal flagellomere longer than penultimate (0.13:0.06, 0.10:0.06), antenna longer than body (3.03, 2.78); antennal scrobes-frons sloped and forming a shelf. Face flat or nearly so, punctate-lacunose, interspaces wavy and longitudinal median carina present. Frons smooth. Temple wide, punctate-lacunose and interspaces wavy. Inner margin of eyes diverging slightly at antennal sockets; in lateral view, eye anteriorly convex and posteriorly straight. POL shorter than OOL (0.11, 0.13). Malar suture present. Median area between lateral ocelli slightly depressed. Vertex laterally rounded and dorsally wide.

**Mesosoma** (Fig. 165A, E, F, I). Mesosoma dorsoventrally convex. Mesoscutum proximally convex and distally flat, punctation distinct throughout, interspaces wavy/lacunose. Scutellum long and slender, apex sloped and fused with BS, but not in the same plane, scutellar punctation scattered throughout, in profile scutellum flat and on same plane as mesoscutum, phragma of the scutellum partially exposed; BS only very partially overlapping the MPM; ATS demilune with complete and undulate/reticulate carinae; dorsal ATS groove with carinae only proximally. Transscutal articulation with small and heterogeneous foveae, area just behind transscutal articulation with a sloped transverse strip and with same kind of sculpture as mesoscutum. Metanotum with BM wider than PFM (clearly differentiated); MPM oval/circular with a short proximal carina; AFM with a small lobe and not as well delineated as PFM; PFM thick and smooth; ATM proximally with a groove with some sculpturing and distally smooth. Propodeum with medium-sized punctation and with a median longitudinal dent, but no trace of median longitudinal carina, proximal half weakly curved; distal edge of propodeum with a flange at each side and without stubs; propodeal spiracle distally framed by faintly concave/wavy carina; nucha surrounded by very short radiating carinae. Pronotum with a distinct dorsal furrow, dorsally with a well-defined smooth band; central area of pronotum and dorsal furrow smooth, but ventral furrow with short parallel carinae. Propleuron with fine punctations throughout and dorsally without a carina. Metasternum convex. Contour of mesopleuron convex; precoxal groove smooth, shiny and shallow, but visible; epicnemial ridge convex, teardrop-shaped.

**Legs.** Ventral margin of fore telotarsus slightly excavated and with a tiny curved seta, fore telotarsus almost same width throughout and longer than fourth tarsomere (0.12, 0.07). Hind coxa with medium-size punctate throughout, dorsal outer depression absent. Inner spur of hind tibia longer than outer spur (0.21, 0.15), entire surface of hind tibia with dense strong spines clearly differentiated by color and length. Hind telotarsus longer than fourth tarsomere (0.15, 0.12).

**Wings** (Fig. 165K, L). Fore wing with r vein straight; 2RS vein straight; r and 2RS veins forming an angle at their junction and outer side of junction forming a slight stub; 2M vein slightly curved/swollen; distally fore wing [where spectral veins are] with microtrichiae more densely concentrated than the rest of the wing; anal cell 1/3 proximally lacking microtrichiae; subbasal cell with microtrichiae virtually throughout; veins 2CUa and 2CUb completely spectral; vein 2 cu-a present as spectral vein, sometimes difficult to see; vein 2-1A proximally tubular and distally spectral, although sometimes difficult to see; tubular vein 1 cu-a straight, incomplete/broken and not reaching the edge of 1-1A vein. Hind wing with vannal lobe very narrow, subdistally and subproximally straightened, and setae evenly scattered in the margin.

**Metasoma** (Fig. 165A, G, H, J). Metasoma laterally compressed. Petiole on T1 with sculpture on distal half, virtually parallel-sided over most of length, but barely narrowing over distal 1/3, apex truncate (length 0.32, maximum width 0.18, minimum width 0.13), and with scattered pubescence concentrated in the first distal third. Lateral grooves delimiting the median area on T2 clearly defined and reaching the distal edge of T2 (length median area 0.19, length T2 0.19), edges of median area obscured by weak longitudinal stripes, median area broader than long (length 0.19, maximum width 0.25, minimum width 0.12); T2 scarce pubescence throughout. T3 longer than T2 (0.24, 0.19) and with pubescence more notorious in distal half. Pubescence on hypopygium dense.

**Cocoon.** Unknown.

#### Comments.

Females with body slender and elongate.

#### Male.

Similar in coloration to female, although the metasoma is slender and cylindrical.

#### Etymology.

Michael (Mike) G. Pogue is a lepidopterist working at the National Museum of Natural History (NMNH), Smithsonian Institution (IS), Washington, D.C., USA.

#### Distribution.

Parasitized caterpillar was collected in Ecuador, Napo, Yanayacu Biological Station (Road to San Rafael Waterfall), during May 2008 at 1,288 m in cloud forest.

#### Biology.

The lifestyle of this parasitoid species is gregarious.

#### Host.

Undetermined species of Erebidae (Arctiinae) feeding on *Saurauia* sp. (Actinidiaceae). Caterpillar was collected in first instar.

### Glyptapanteles mikeschauffi

Taxon classificationAnimaliaHymenopteraBraconidae

Arias-Penna, sp. nov.

http://zoobank.org/51619F82-F0F2-4B10-9065-2C552B61CC16

Figs 166 , 167

#### Female.

Body length 2.02 mm, antenna length 2.12 mm, fore wing length 2.17 mm.

#### Type material.

**Holotype**: COSTA RICA • 1♀; 06-SRNP-4537 DHJPAR0012006; Área de Conservación Guanacaste, Alajuela, Sector San Cristóbal, Vado Río Cucaracho; rain forest; 640 m; 10.8702, -85.39153; 11.vi.2006; Anabelle Córdoba leg.; caterpillar collected in fourth instar; large number of separate cocoons adhered among the setae of the caterpillar; adult parasitoids emerged on 30.vi.2006; (CNC). **Paratypes.** • 73 (4♀, 3♂) (66♀, 0♂); 06-SRNP-4537 DHJPAR0012006; same data as for holotype; (CNC).

#### Other material.

**Reared material.** COSTA RICA: *Área de Conservación Guanacaste*, *Alajuela*, *Sector San Cristóbal*, *Puente Palma*: • 23 (5♀, 3♂) (15♀, 0♂); 06-SRNP-4814, DHJPAR0012007; rain forest; 460 m; 10. 9163, -85.37869; 17.vi.2006; Carolina Cano leg.; caterpillar collected in fourth instar; cocoons adhered to the larval cuticle; adult parasitoids emerged on 27.vi.2006. • 7 (3♀, 1♂) (3♀, 0♂); 08-SRNP-6889, DHJPAR0030705; same data as for preceding except: 12.ix.2008; brown separate cocoons among the setae, standing on end; adult parasitoid emerged on 26.xii.2008.

#### Diagnosis.

**Figure 166.**
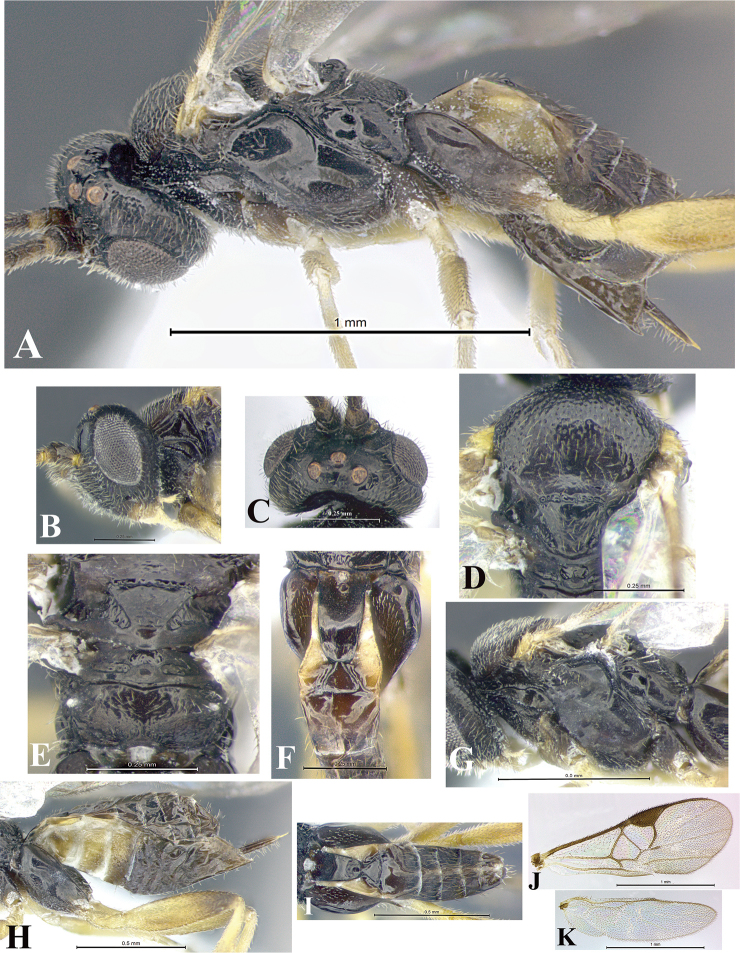
*Glyptapantelesmikeschauffi* sp. nov. female 06-SRNP-4814 DHJPAR0012007 **A** Habitus **B, C** Head B lateral view **C** Dorsal view **D** Mesonotum, dorsal view **E** Scutellum, metanotum, propodeum, dorsal view **F**T1–4, dorsal view **G** Mesosoma, lateral view **H, I** Metasoma **H** Lateral view **I** Dorsal view **J, K** Wings **J** Fore **K** Hind.

**Figure 167.**
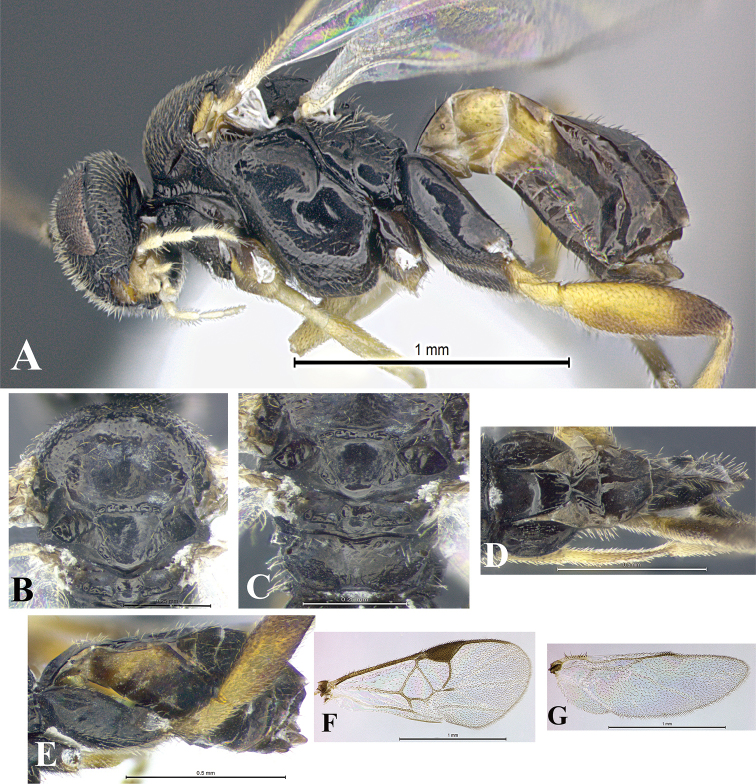
*Glyptapantelesmikeschauffi* sp. nov. male 06-SRNP-4814 DHJPAR0012007, 08-SRNP-6889 DHJPAR0030705 **A** Habitus **B** Mesonotum, dorsal view **C** Scutellum, metanotum, propodeum, dorsal view **D, E** Metasoma **D** Dorsal view **E** Lateral view **F, G** Wings **F** Fore **G** Hind.

Surface of metasternum convex, fore wing with r vein curved, outer side of junction of r and 2RS veins not forming a stub (Figs 166J, 167F), distal antennal flagellomere longer than penultimate, petiole on T1 evenly narrowing distally, completely smooth and polished, with faint, satin-like sheen (Figs 166F, I, 167D), propodeum without median longitudinal carina (Figs 166E, 167C), and lateral grooves delimiting the median area on T2 clearly defined and reaching the distal edge of T2 (Figs 166F, I, 167D).

#### Coloration

(Fig. 166A–K). General body coloration black except pedicel brown distally with a ring yellow; first five-six proximal antennal flagellomeres dorsally lighter (light brown) than ventrally (dark brown), remaining flagellomeres dark brown on both sides; labrum, mandible, and glossa yellow-brown; maxillary and labial palps, and tegulae yellow. Eyes black and ocelli silver/reddish (in preserved specimen). Fore and middle legs yellow except coxae brown with inner side almost completely yellow, and claws brown; hind legs yellow except black coxae, femora with apex brown, distal 1/3 of tibiae brown, and tarsomeres brown although proximally basitarsus with a narrow yellow band. Petiole on T1 dark brown and sublateral areas yellow-brown; T2 with median area brown, and adjacent area and lateral ends yellow-brown; T3 broadly brown with a small yellow-brown area on the corner proximal; T4 and beyond completely brown; distally each tergum with a narrow whitish transparent band. In lateral view, T1–3 yellow-brown; T4 and beyond brown. S1–4 yellow-brown; penultimate sternum completely brown; hypopygium brown, although medially lighter.

#### Description.

**Head** (Fig. 166A–C). Head rectangle with pubescence long and dense. Proximal three antennal flagellomeres length longer than wide (0.16:0.05, 0.18:0.05, 0.17:0.05), distal antennal flagellomere longer than penultimate (0.11:0.05, 0.08:0.05), antenna longer than body (2.12, 2.02); antennal scrobes-frons sloped and forming a shelf. Face flat or nearly so, punctate-lacunose, interspaces wavy and longitudinal median carina present. Frons smooth. Temple wide, punctate-lacunose and interspaces wavy. Inner margin of eyes diverging slightly at antennal sockets; in lateral view, eye with both sides convex. POL shorter than OOL (0.09, 0.11). Malar suture present. Median area between lateral ocelli slightly depressed. Vertex laterally pointed or nearly so and dorsally wide.

**Mesosoma** (Fig. 166A, D, E, G). Mesosoma dorsoventrally convex. Mesoscutum proximally convex and distally flat, punctation distinct proximally with polished area distally, interspaces smooth. Scutellum triangular, apex sloped and fused with BS, scutellar punctation indistinct throughout, in profile scutellum flat and on same plane as mesoscutum, phragma of the scutellum partially exposed; BS only very partially overlapping the MPM; ATS demilune with a little, complete and parallel carinae; dorsal ATS groove with semicircular/parallel carinae. Transscutal articulation with small and heterogeneous foveae, area just behind transscutal articulation smooth, shiny and nearly at the same level as mesoscutum (flat). Metanotum with BM wider than PFM (clearly differentiated); MPM circular and bisected by a median longitudinal carina; AFM without setiferous lobes and not as well delineated as PFM; PFM thick, smooth and with lateral ends rounded; ATM proximally with a groove with some sculpturing and distally with rugae. Propodeum without median longitudinal carina and with indistinct sculpture, proximal half weakly curved and distal half with a shallow dent at each side of nucha; distal edge of propodeum with a flange at each side and without stubs; propodeal spiracle without distal carina; nucha surrounded by very short radiating carinae. Pronotum with a distinct dorsal furrow, dorsally with a well-defined smooth band; central area of pronotum smooth, but both dorsal and ventral furrows with short and parallel carinae. Propleuron with fine punctations throughout and dorsally without a carina. Metasternum convex. Contour of mesopleuron straight/angulate or nearly so; precoxal groove smooth, shiny and distinct; epicnemial ridge elongated more fusiform (tapering at both ends).

**Legs.** Ventral margin of fore telotarsus entire without seta, fore telotarsus almost same width throughout and longer than fourth tarsomere (0.11, 0.06). Hind coxa with punctation only on ventral surface, dorsal outer depression present. Inner spur of hind tibia longer than outer spur (0.18, 0.14), entire surface of hind tibia with dense strong spines clearly differentiated by color and length. Hind telotarsus as equal in length as fourth tarsomere (0.11, 0.11).

**Wings** (Fig. 166J, K). Fore wing with r vein slightly curved; 2RS vein straight; r and 2RS veins forming a weak, even curve at their junction and outer side of junction not forming a stub; 2M vein slightly curved/swollen; distally fore wing [where spectral veins are] with microtrichiae more densely concentrated than the rest of the wing; anal cell 1/3 proximally lacking microtrichiae; subbasal cell with a small smooth area; vein 2CUa absent and 2CUb spectral; vein 2 cu-a absent; vein 2-1A proximally tubular and distally spectral, although sometimes difficult to see; tubular vein 1 cu-a curved and complete, but junction with 1-1A vein spectral. Hind wing with vannal lobe very narrow, subdistally evenly convex and subproximally straightened, and setae evenly scattered in the margin.

**Metasoma** (Fig. 166A, F, H, I). Metasoma laterally compressed. Petiole on T1 completely smooth and polished, with faint, satin-like sheen, evenly narrowing distally (length 0.27, maximum width 0.13, minimum width 0.09) and with scattered pubescence concentrated in the first distal third. Lateral grooves delimiting the median area on T2 clearly defined and reaching the distal edge of T2 (length median area 0.11, length T2 0.11), edges of median area polished and lateral grooves deep, median area broader than long (length 0.11, maximum width 0.13, minimum width 0.07); T2 with scattered pubescence only distally. T3 longer than T2 (0.16, 0.11) and with scattered pubescence throughout. Pubescence on hypopygium scattered.

**Cocoons.** Light brown oval cocoons with evenly smooth silk fibers. Cocoons adhered among the setae of the caterpillar

#### Comments.

The ovipositor sheath is long as in *Sathon*, the pubescence is distributed along the surface, but not concentrated at the apex (Fig. 166A, H).

#### Male

(Fig. 167A–G). Similar in coloration and shape to female.

#### Etymology.

Michael (Mike) E. Schauff’s research has focused upon the biology and systematics of chalcidoid wasps, including Eulophidae. He has been based at the USDA Systematic Entomology Laboratory at the American National Museum of Natural History, New York, NY, USA.

#### Distribution.

The parasitized caterpillars were collected in Costa Rica, ACG, Sector San Cristóbal (Puente Palma and Vado Río Cucaracho), during June 2006 and September 2008 at 460 and 640 m in rain forest.

#### Biology.

The lifestyle of this parasitoid species is gregarious.

#### Host.

*Bertholdiaalbipuncta* Schaus (Erebidae: Arctiinae) feeding on *Drymoniamacrophylla* (Gesneriaceae) and *B.specularis* (Herrich-Schäffer) (Erebidae: Arctiinae) feeding on *Sabiceavillosa* (Rubiaceae). Caterpillars were collected in fourth instar.

### Glyptapanteles mikesharkeyi

Taxon classificationAnimaliaHymenopteraBraconidae

Arias-Penna, sp. nov.

http://zoobank.org/385842C8-378B-4326-A62B-13729E57E29B

Figs 168 , 169

#### Female.

Body length 3.23 mm, antenna length 4.04 mm, fore wing length 3.28 mm.

#### Type material.

**Holotype**: COSTA RICA • 1♀; 98-SRNP-16039, DHJPAR0012637; Área de Conservación Guanacaste, Guanacaste, Sector El Hacha, Sendero Bejuquilla; dry-rain intergrade forest; Malaise; 280 m; 11.03004, -85.52699; 07.xii.1998; DH Janzen & W Hallwachs leg.; (CNC). **Paratypes.** • 1 (0♀, 0♂) (1♀, 0♂); 98-SRNP-16038, DHJPAR0012635; same data as for holotype except: 28.xii.1998; (CNC). • 1 (0♀, 0♂) (1♀, 0♂); 98-SRNP-16040, DHJPAR0012638; same data as for holotype; (CNC). • 1 (0♀, 0♂) (1♀, 0♂); 99-SRNP-18933, DHJPAR0012630; same data as for holotype except: 05.vii.1999; (CNC). •1 (0♀, 0♂) (1♀, 0♂); 99-SRNP-18934, DHJPAR0012632; same data as for holotype except: 19.vii.1999, (CNC). •1 (1♀, 0♂) (0♀, 0♂); 99-SRNP-18936, DHJPAR0012636; same data as for holotype except: 18.i.1999; (CNC). • 1 (0♀, 1♂) (0♀, 0♂); 99-SRNP-19246, DHJPAR0013633; same data as for holotype except: 17.v.1999; (CNC).

#### Other material.

**Malaise-trapped material**. COSTA RICA: *Área de Conservación Guanacaste*, *Alajuela*, *Sector Rincón Rain Forest*, *Vado Río Francia*: • 1 (0♀, 0♂) (1♀, 0♂); 07-SRNP-66822, DHJPAR0025360; Malaise; 400 m; 10.90093, -85.28915; 26.xi.2007; DH Janzen & W Hallwachs leg. • 1 (0♀, 1♂) (0♀, 0♂); 07-SRNP-66823, DHJPAR0025361; same data as for preceding. • 1 (1♀, 0♂) (0♀, 0♂); 07-SRNP-66972, DHJPAR0025510; same data as for preceding except: 29.vi.2007. • 1 (0♀, 0♂) (0♀, 1♂); 07-SRNP-67587, DHJPAR0026117; same data as for preceding except: 02.xi.2007.

*Área de Conservación Guanacaste*, *Alajuela*, *Sector San Cristóbal*, *Bosque Trampa Malaise*: • 1 (1♀, 0♂) (0♀, 0♂); 08-SRNP-3950, DHJPAR0027652; rain forest; 815 m; 10.86280, -85.38460; 30.i.2008; DH Janzen & W Hallwachs leg.

*Área de Conservación Guanacaste*, *Alajuela*, *Sector San Cristóbal*, *Estación San Gerardo*: • 1 (1♀, 0♂) (0♀, 0♂); 07-SRNP-67296, DHJPAR0025834; rain forest; Malaise; 575 m; 10.88009, -85.38887; 04.vii.2007; DH Janzen & W Hallwachs leg. • 1 (0♀, 0♂) (0♀, 1♂); 07-SRNP-67321, DHJPAR0025859; same data as for preceding xcept: 05.vi.2007. • 1 (0♀, 1♂) (0♀, 0♂); 07-SRNP-67326, DHJPAR0025864; same data as for preceding except: 10.vi.2007. • 1 (0♀, 0♂) (0♀, 1♂); 07-SRNP-67329, DHJPAR0025867; same data as for preceding except: 10.vi.2007.

*Área de Conservación Guanacaste*, *Alajuela*, *Sector San Cristóbal*, *Potrero Argentina*: • 1 (0♀, 0♂) (1♀, 0♂); 07-SRNP-67016, DHJPAR0025554; pastures; Malaise; 520 m; 10.89021, -85.38803; 14.x.2007; DH Janzen & W Hallwachs leg. • 1 (0♀, 1♂) (0♀, 0♂); 07-SRNP-67021, DHJPAR0025559; same data as for preceding except: 08.ix.2007. • 1 (1♀, 0♂) (0♀, 0♂); 07-SRNP-67032, DHJPAR0025570; same data as for preceding except: 04.vii.2007. • 1 (1♀, 0♂) (0♀, 0♂); 07-SRNP-67037, DHJPAR0025575; same data as for preceding except: 04.vii.2007. • 1 (0♀, 0♂) (0♀, 1♂); 07-SRNP-67047, DHJPAR0025585; same data as for preceding except: 04.vii.2007. • 1 (0♀, 0♂) (0♀, 1♂); 07-SRNP-67094, DHJPAR0025632; same data as for preceding except: 02.ix.2007. • 1 (1♀, 0♂) (0♀, 0♂); 07-SRNP-67101, DHJPAR0025639; same data as for preceding except: 02.ix.2007. • 1 (0♀, 0♂) (0♀, 1♂); 07-SRNP-67118, DHJPAR0025656; same data as for preceding except: 28.xii.2007. • 1 (0♀, 0♂) (1♀, 0♂); 07-SRNP-67170, DHJPAR0025708; same data as for preceding except: 22.vii.2007. • 1 (0♀, 0♂) (1♀, 0♂); 07-SRNP-67171, DHJPAR0025709; same data as for preceding except: 22.vii.2007. • 1 (0♀, 0♂) (0♀, 1♂); 07-SRNP-67223, DHJPAR0025761; same data as for preceding except: 16.vi.2007. • 1 (0♀, 1♂) (0♀, 0♂); 07-SRNP-67232, DHJPAR0025770; same data as for preceding except: 16.vi.2007. • 1 (0♀, 1♂) (0♀, 0♂); 07-SRNP-67249, DHJPAR0025787; same data as for preceding except: 26.ix.2007. • 1 (0♀, 0♂) (1♀, 0♂); 07-SRNP-67250, DHJPAR0025788; same data as for preceding except: 26.ix.2007. • 1 (0♀, 0♂) (0♀, 1♂); 07-SRNP-67729, DHJPAR0027467; same data as for preceding except: 03.viii.2007. • 1 (0♀, 0♂) (0♀, 1♂); 07-SRNP-67730, DHJPAR0027468; same data as for preceding except: 03.viii.2007. • 1 (0♀, 0♂) (1♀, 0♂); 07-SRNP-67735, DHJPAR0027473; same data as for preceding except: 03.viii.2007. • 1 (0♀, 0♂) (1♀, 0♂); 07-SRNP-67791, DHJPAR0027529; same data as for preceding except: 07.xi.2007.

*Área de Conservación Guanacaste*, *Alajuela*, *Sector San Cristóbal*, *Río Blanco Abajo*: • 1 (0♀, 0♂) (1♀, 0♂); 07-SRNP-66222, DHJPAR0024760; rain forest; Malaise; 500 m; 10.90037, -85.37254; 08.viii.2007; DH Janzen & W Hallwachs leg. • 1 (0♀, 0♂) (1♀, 0♂); 07-SRNP-66223, DHJPAR0024761; same data as for preceding. • 1 (1♀, 0♂) (0♀, 0♂); 07-SRNP-66231, DHJPAR0024769; same data as for preceding. • 1 (1♀, 0♂) (0♀, 0♂); 07-SRNP-66272, DHJPAR0024810; same data as for preceding except: 26.ix.2007. • 1 (1♀, 0♂) (0♀, 0♂); 07-SRNP-66275, DHJPAR0024813; same data as for preceding except: 26.ix.2007. • 1 (0♀, 0♂) (1♀, 0♂); 07-SRNP-66276, DHJPAR0024814; same data as for preceding except: 26.ix.2007. • 1 (0♀, 0♂) (1♀, 0♂); 07-SRNP-66314, DHJPAR0024852; same data as for preceding except: 15.viii.2007. • 1 (0♀, 0♂) (0♀, 1♂); 07-SRNP-66358, DHJPAR0024896; same data as for preceding except: 02.x.2007. • 1 (0♀, 0♂) (1♀, 0♂); 07-SRNP-66373, DHJPAR0024911; same data as for preceding except: 25.xi.2007. • 1 (0♀, 0♂) (1♀, 0♂); 07-SRNP-66374, DHJPAR0024912; same data as for preceding except: 10.vii.2007. • 1 (0♀, 0♂) (0♀, 1♂); 07-SRNP-66396, DHJPAR0024934; same data as for preceding except: 21.vii.2007. • 1 (0♀, 1♂) (0♀, 0♂); 07-SRNP-66423, DHJPAR0024961; same data as for preceding except: 28.vii.2007. • 1 (0♀, 0♂) (1♀, 0♂); 07-SRNP-66462, DHJPAR0025000; same data as for preceding except: 22.vii.2007. • 1 (0♀, 0♂) (0♀, 1♂); 07-SRNP-66472, DHJPAR0025010; same data as for preceding except: 27.viii.2007. • 1 (0♀, 0♂) (0♀, 1♂); 07-SRNP-66480, DHJPAR0025018; same data as for preceding except: 27.viii.2007. • 1 (0♀, 0♂) (1♀, 0♂); 07-SRNP-66539, DHJPAR0025077; same data as for preceding except: 05.vi.2007. • 1 (0♀, 0♂) (1♀, 0♂); 07-SRNP-66567, DHJPAR0025105; same data as for preceding except: 05.vi.2007. • 1 (0♀, 0♂) (1♀, 0♂); 07-SRNP-66573, DHJPAR0025111; same data as for preceding except: 04.vii.2007. • 1 (0♀, 1♂) (0♀, 0♂); 07-SRNP-66579, DHJPAR0025117; same data as for preceding except: 04.vii.2007. • 1 (0♀, 0♂) (0♀, 1♂); 07-SRNP-66594, DHJPAR0025132; same data as for preceding except: 04.vii.2007. • 1 (0♀, 0♂) (1♀, 0♂); 07-SRNP-66598, DHJPAR0025136; same data as for preceding except: 04.vii.2007. • 1 (0♀, 0♂) (1♀, 0♂); 07-SRNP-66606, DHJPAR0025144; same data as for preceding except: 04.vii.2007. • 1 (0♀, 0♂) (0♀, 1♂); 07-SRNP-66625, DHJPAR0025163; same data as for preceding except: • 21.viii.2007. • 1 (0♀, 0♂) (0♀, 1♂); 07-SRNP-66634, DHJPAR0025172; same data as for preceding except: 04.vii.2007. • 1 (1♀, 0♂) (0♀, 0♂); 07-SRNP-66667, DHJPAR0025205; same data as for preceding except: 14.x.2007. • 1 (0♀, 0♂) (1♀, 0♂); 07-SRNP-66669, DHJPAR0025207; same data as for preceding except: 14.x.2007. • 1 (0♀, 0♂) (1♀, 0♂); 07-SRNP-66679, DHJPAR0025217; same data as for preceding except: 22.vii.2007. • 1 (0♀, 0♂) (1♀, 0♂); 07-SRNP-66684, DHJPAR0025222; same data as for preceding except: 22.vii.2007. • 1 (0♀, 1♂) (0♀, 0♂); 07-SRNP-66687, DHJPAR0025225; same data as for preceding except: 22.vii.2007. • 1 (0♀, 0♂) (0♀, 1♂); 07-SRNP-66688, DHJPAR0025226; same data as for preceding except: 22.vii.2007. • 1 (1♀, 0♂) (0♀, 0♂); 07-SRNP-66703, DHJPAR0025241; same data as for preceding except: 26.x.2007. 1 (0♀, 0♂) (1♀, 0♂); 07-SRNP-66709, DHJPAR0025247; same data as for preceding except: 26.x.2007. • 1 (1♀, 0♂) (0♀, 0♂); [07-SRNP-66710, DHJPAR0025248]; same data as for preceding except: 26.x.2007, metasoma missing. • 1 (0♀, 0♂) (1♀, 0♂); 07-SRNP-66712, DHJPAR0025250; same data as for preceding except: 25.xii.2007. • 1 (0♀, 0♂) (1♀, 0♂); 07-SRNP-66715, DHJPAR0025253; same data as for preceding except: 07.xii.2007. • 1 (0♀, 0♂) (1♀, 0♂); 07-SRNP-66724, DHJPAR0025262; same data as for preceding except: 14.x.2007. • 1 (0♀, 0♂) (0♀, 1♂); 07-SRNP-66729, DHJPAR0025267; same data as for preceding except: 10.vi.2007. • 1 (0♀, 0♂) (1♀, 0♂); 07-SRNP-66748, DHJPAR0025286; same data as for preceding except: 02.ix.2007. • 1 (0♀, 0♂) (1♀, 0♂); 07-SRNP-66753, DHJPAR0025291; same data as for preceding except: 02.ix.2007. • 1 (0♀, 0♂) (1♀, 0♂); 07-SRNP-66762, DHJPAR0025300; same data as for preceding except: 02.ix.2007. • 1 (0♀, 0♂) (1♀, 0♂); 07-SRNP-66769, DHJPAR0025307; same data as for preceding except: 02.ix.2007. • 1 (0♀, 0♂) (1♀, 0♂); 07-SRNP-66775, DHJPAR0025313; same data as for preceding except: 16.vi.2007. • 1 (0♀, 0♂) (0♀, 1♂); 07-SRNP-66778, DHJPAR0025316; same data as for preceding except: 16.vi.2007. • 1 (0♀, 0♂) (0♀, 1♂); 07-SRNP-66797, DHJPAR0025335; same data as for preceding except: 16.vi.2007. • 1 (0♀, 0♂) (0♀, 1♂); 07-SRNP-67597, DHJPAR0026292; same data as for preceding except: 03.viii.2007. • 1 (0♀, 0♂) (1♀, 0♂); 07-SRNP-67598, DHJPAR0026293; same data as for preceding except: 03.viii.2007. • 1 (0♀, 0♂) (0♀, 1♂); 07-SRNP-67625, DHJPAR0026320; same data as for preceding except: 03.viii.2007. • 1 (0♀, 0♂) (1♀, 0♂); 07-SRNP-67637, DHJPAR0026332; same data as for preceding except: 09.viii.2007. • 1 (0♀, 0♂) (1♀, 0♂); 07-SRNP-67642, DHJPAR0026337; same data as for preceding except: 09.viii.2007. • 1 (0♀, 0♂) (0♀, 1♂); 07-SRNP-67652, DHJPAR0026347; same data as for preceding except: 09.viii.2007. • 1 (0♀, 0♂) (0♀, 1♂); 07-SRNP-67678, DHJPAR0026373; same data as for preceding except: 09.viii.2007. • 1 (0♀, 1♂) (0♀, 0♂); 07-SRNP-67679, DHJPAR0026374; same data as for preceding except: 09.viii.2007. • 1 (0♀, 0♂) (0♀, 1♂); 07-SRNP-67685, DHJPAR0026380; same data as for preceding except: 01.xi.2007. • 1 (0♀, 0♂) (0♀, 1♂); 07-SRNP-67688, DHJPAR0026383; same data as for preceding except: 01.xi.2007. • 1 (0♀, 0♂) (1♀, 0♂); 07-SRNP-67704, DHJPAR0026399; same data as for preceding except: 07.xi.2007. • 1 (0♀, 0♂) (1♀, 0♂); 07-SRNP-67710, DHJPAR0026405; same data as for preceding except: 07.xi.2007. • 1 (0♀, 0♂) (1♀, 0♂); 07-SRNP-67711, DHJPAR0026406; same data as for preceding except: 07.xi.2007. • 1 (0♀, 0♂) (1♀, 0♂); 07-SRNP-67715, DHJPAR0026410; same data as for preceding except: 13.xi.2007. • 1 (0♀, 0♂) (1♀, 0♂); 07-SRNP-67717, DHJPAR0026412; same data as for preceding except: 13.xi.2007. • 1 (0♀, 0♂) (1♀, 0♂); 08-SRNP-2845, DHJPAR0026426; same data as for preceding except: 06.i.2008. • 1 (0♀, 0♂) (0♀, 1♂); 08-SRNP-2889, DHJPAR0026470; same data as for preceding except: 05.ii.2008. • 1 (0♀, 0♂) (1♀, 0♂); 08-SRNP-2894, DHJPAR0026475; same data as for preceding except: 05.ii.2008. • 1 (0♀, 0♂) (1♀, 0♂); 08-SRNP-2928, DHJPAR0026509; same data as for preceding except: 11.ii.2008. • 1 (0♀, 0♂) (1♀, 0♂); 08-SRNP-2931, DHJPAR0026512; same data as for preceding except: 11.ii.2008. • 1 (0♀, 0♂) (0♀, 1♂); 08-SRNP-2937, DHJPAR0026518; same data as for preceding except: 11.ii.2008. • 1 (0♀, 0♂) (0♀, 1♂); 08-SRNP-2952, DHJPAR0026533; same data as for preceding except: 17.ii.2008. • 1 (0♀, 0♂) (1♀, 0♂); 08-SRNP-2967, DHJPAR0026548; same data as for preceding except: 17.ii.2008. • 1 (0♀, 0♂) (0♀, 1♂); 08-SRNP-2973, DHJPAR0026554; same data as for preceding except: 23.ii.2008. • 1 (0♀, 0♂) (1♀, 0♂); 08-SRNP-2992, DHJPAR0026573; same data as for preceding except: 23.ii.2008. • 1 (0♀, 0♂) (0♀, 1♂); 08-SRNP-3176, DHJPAR0026757; same data as for preceding except: 24.iii.2008. • 1 (0♀, 0♂) (1♀, 0♂); 08-SRNP-3185, DHJPAR0026766; same data as for preceding except: 24.iii.2008. • 1 (0♀, 0♂) (1♀, 0♂); 08-SRNP-3189, DHJPAR0026770; same data as for preceding except: 24.iii.2008. • 1 (0♀, 0♂) (0♀, 1♂); 08-SRNP-3243, DHJPAR0026824; same data as for preceding except: 30.iii.2008. • 1 (0♀, 0♂) (1♀, 0♂); 08-SRNP-3371, DHJPAR0026952; same data as for preceding except: 05.iv.2008. • 1 (0♀, 0♂) (0♀, 1♂); 08-SRNP-3372, DHJPAR0026953; same data as for preceding except: 11.iv.2008. • 1 (0♀, 0♂) (1♀, 0♂); 08-SRNP-3398, DHJPAR0026979; same data as for preceding except: 11.iv.2008. • 1 (0♀, 0♂) (1♀, 0♂); 08-SRNP-3403, DHJPAR0026984; same data as for preceding except: 11.iv.2008. • 1 (0♀, 0♂) (1♀, 0♂); 08-SRNP-3415, DHJPAR0026996; same data as for preceding except: 11.iv.2008. • 1 (0♀, 0♂) (1♀, 0♂); 08-SRNP-3471, DHJPAR0027052; same data as for preceding except: 11.iv.2008. • 1 (0♀, 0♂) (0♀, 1♂); 08-SRNP-3483, DHJPAR0027064; same data as for preceding except: 17.iv.2008. • 1 (0♀, 0♂) (1♀, 0♂); 08-SRNP-3484, DHJPAR0027065; same data as for preceding except: 17.iv.2008. • 1 (0♀, 0♂) (1♀, 0♂); 08-SRNP-3487, DHJPAR0027068; same data as for preceding except: 17.iv.2008. • 1 (0♀, 0♂) (0♀, 1♂); 08-SRNP-3524, DHJPAR0027105; same data as for preceding except: 17.iv.2008. • 1 (0♀, 0♂) (1♀, 0♂); 08-SRNP-3555, DHJPAR0027136; same data as for preceding except: 23.iv.2008. • 1 (0♀, 0♂) (0♀, 1♂); 08-SRNP-3562, DHJPAR0027143; same data as for preceding except: 23.iv.2008. • 1 (0♀, 0♂) (0♀, 1♂); 08-SRNP-3566, DHJPAR0027147; same data as for preceding except: 23.iv.2008. • 1 (0♀, 1♂) (0♀, 0♂); 08-SRNP-3571, DHJPAR0027152; same data as for preceding except: 23.iv.2008. • 1 (0♀, 0♂) (0♀, 1♂); 08-SRNP-3577, DHJPAR0027158; same data as for preceding except: 23.iv.2008. • 1 (0♀, 0♂) (0♀, 1♂); 08-SRNP-3594, DHJPAR0027175; same data as for preceding except: 23.iv.2008. • 1 (0♀, 0♂) (0♀, 1♂); 08-SRNP-3655, DHJPAR0027236; same data as for preceding except: 30.iv.2008. • 1 (0♀, 0♂) (0♀, 1♂); 08-SRNP-3667, DHJPAR0027248; same data as for preceding except: 30.iv.2008. • 1 (0♀, 0♂) (1♀, 0♂); 08-SRNP-3676, DHJPAR0027257; same data as for preceding except: 30.iv.2008. • 1 (0♀, 0♂) (1♀, 0♂); 08-SRNP-3679, DHJPAR0027260; same data as for preceding except: 30.iv.2008. • 1 (0♀, 0♂) (0♀, 1♂); 08-SRNP-3688, DHJPAR0027269; same data as for preceding except: 30.iv.2008. • 1 (0♀, 0♂) (1♀, 0♂); 08-SRNP-3764, DHJPAR0027345; same data as for preceding except: 12.v.2008. • 1 (0♀, 0♂) (0♀, 1♂); 08-SRNP-3783, DHJPAR0027364; same data as for preceding except: 12.v.2008. • 1 (0♀, 1♂) (0♀, 0♂); 08-SRNP-3801, DHJPAR0027382; same data as for preceding except: 12.v.2008. • 1 (0♀, 0♂) (1♀, 0♂); 08-SRNP-3837, DHJPAR0027418; same data as for preceding except: 18.v.2008. • 1 (0♀, 0♂) (0♀, 1♂); 08-SRNP-3881, DHJPAR0027462; same data as for preceding except: 19.v.2008.

#### Diagnosis.

**Figure 168.**
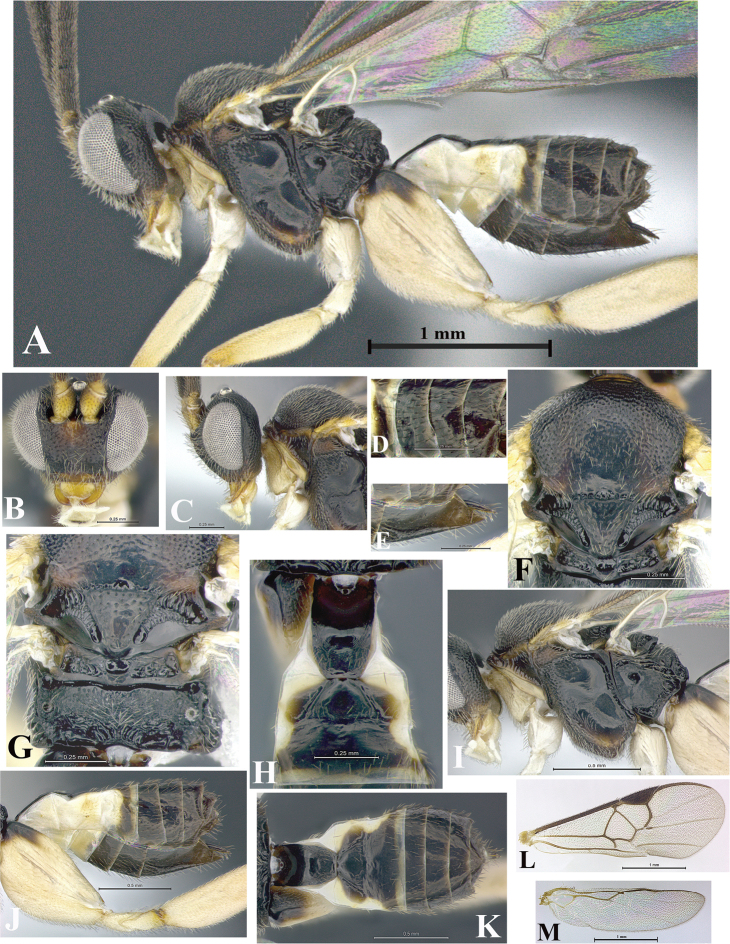
*Glyptapantelesmikesharkeyi* sp. nov. female 98-SRNP-16039 DHJPAR0012637, 07-SRNP-67296 DHJPAR0025834 **A** Habitus **B** Head, frontal view **C** Head, pronotum, propleuron, lateral view **D** Metasomal glands **E** Genitalia: hypopygium, ovipositor, ovipositor sheaths, lateral view **F** Mesonotum, dorsal view **G** Scutellum, metanotum, propodeum, dorsal view **H**T1–3, dorsal view **I** Mesosoma, lateral view **J, K** Metasoma **J** Lateral view **K** Dorsal view **L, M** Wings **L** Fore **M** Hind.

**Figure 169.**
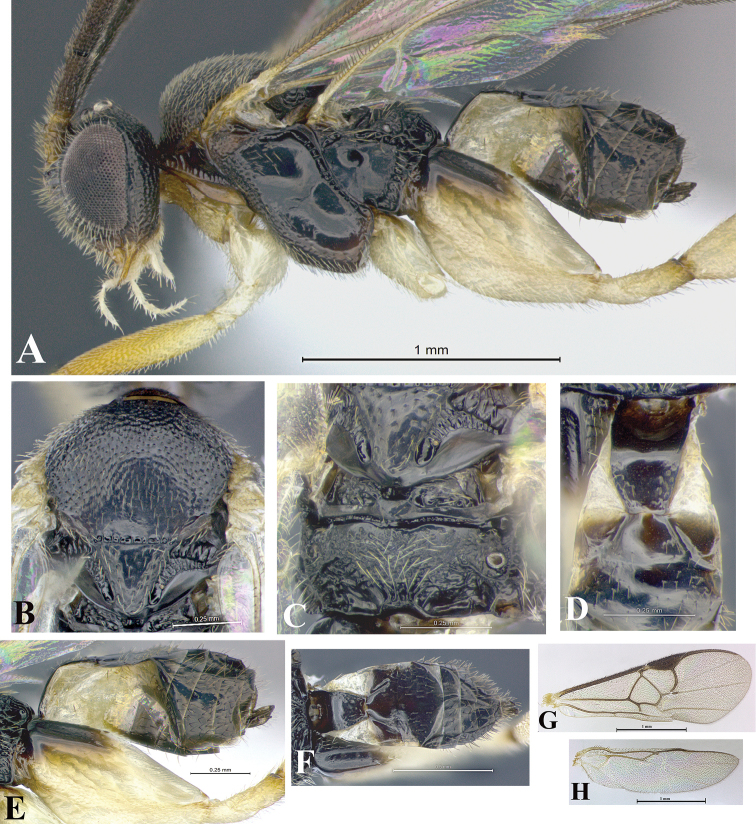
*Glyptapantelesmikesharkeyi* sp. nov. male 08-SRNP-3571 DHJPAR0027152, 08-SRNP-3801 DHJPAR0027382 **A** Habitus **B** Mesonotum, dorsal view **C** Scutellum, metanotum, propodeum, dorsal view **D**T1–2, dorsal view **E, F** Metasoma **E** Lateral view **F** Dorsal view **G, H** Wings **G** Fore **H** Hind.

Ventral margin of fore telotarsus entire without seta, distal antennal flagellomere subequal in length with penultimate, inner spur of hind tibia much longer than outer spur, median area on T2 broader than long (Figs 168H, K, 169D, F), propodeal spiracle distally framed by a short concave carina (Figs 168G, 169C), petiole on T1 distally with lateral margins relatively straight, finely sculptured on 3/4 proximal (Figs 168H, K, 169D, F), surface of metasternum flat or nearly so, precoxal groove deep with lineate sculpture (Figs 168A, I, 169A), fore wing with vein 1 cu-a curved, r vein curved, outer side of junction of r and 2RS veins not forming a stub (Figs 168L, 169G), dorsal outer depression on hind coxa present (Figs 168A, J, 169A, E), inner margin of eyes diverging slightly at antennal sockets (Fig. 168B), propodeum with transverse rugae, but no trace of median longitudinal carina (Figs 168G, 169C), and lateral grooves delimiting the median area on T2 clearly defined and reaching the distal edge of T2 (Figs 168H, K, 169D, F).

#### Coloration

(Fig. 168A–M). General body coloration satin black except yellow scape and pedicel, both with lateral brown band; all antennal flagellomeres dark brown on both sides; gena, clypeus, labrum, mandible, distal 1/3 of low face, some small areas of metasternum, a small ventral band of mesopleuron, dorsal furrow of pronotum, epicnemial ridge, distal corners of mesoscutum, lateral ends of metapleuron, and narrow band taking the place of notauli with yellow/reddish tints; maxillary and labial palps, tegulae, propleuron, and ventral furrow of pronotum yellow. Eyes and ocelli silver. Fore and middle legs yellow (intensity of yellow coloration increasing from proximal to distal), and claws brown; hind legs pale yellow except coxae with a tiny dorso-proximal brown area, femora distally with a tiny brown dot, tibiae and tarsomeres dark brown. Petiole on T1 black and sublateral areas ivory/pale yellow; T2 with median area black, adjacent area yellow-brown and lateral ends ivory/pale yellow; T3 broadly black, shape of area coinciding with the width of median and adjacent areas on T2, 1/3 proximal of lateral ends ivory/pale yellow, and distally T3 with a wide ivory/pale yellow band; T4 and beyond completely brown; distally each tergum with a narrow yellow translucent band. In lateral view, T1–2 completely ivory/pale yellow; T3 proximal half ivory/pale yellow, distal half brown; T4 and beyond brown. S1–3 yellow; S4 and beyond brown.

#### Description.

**Head** (Fig. 168A–C). Head rhomboid with pubescence long and dense. Proximal three antennal flagellomeres longer than wide (0.27:0.10, 0.30:0.10, 0.29:0.10), distal antennal flagellomere subequal in length with penultimate (0.16:0.10, 0.15:0.10), antenna longer than body (4.04, 3.23); antennal scrobes-frons shallow. Face flat or nearly so, with dense fine punctations, interspaces smooth and longitudinal median carina present. Frons smooth. Temple wide, punctate-lacunose and interspaces wavy. Inner margin of eyes diverging slightly at antennal sockets; in lateral view, eye anteriorly convex and posteriorly straight. POL shorter than OOL (0.10, 0.12). Malar suture present. Median area between lateral ocelli slightly depressed. Vertex laterally pointed or nearly so and dorsally wide.

**Mesosoma** (Fig. 168A, F, G, I). Mesosoma dorsoventrally convex. Mesoscutum proximally convex and distally flat, punctation distinct proximally with polished area distally, interspaces wavy/lacunose. Scutellum triangular, apex sloped and fused with BS, scutellar punctation distinct throughout, in profile scutellum slightly convex, but on same plane as mesoscutum, phragma of the scutellum partially exposed; BS only very partially overlapping the MPM; ATS demilune with quite a little, complete and parallel carinae; dorsal ATS groove with semicircular/parallel carinae. Transscutal articulation with small and homogeneous foveae, area just behind transscutal articulation depressed centrally and with same kind of sculpture as mesoscutum. Metanotum with BM wider than PFM (clearly differentiated); MPM circular without median longitudinal carina; AFM with a small lobe and not as well delineated as PFM; PFM thick, smooth and with lateral ends rounded; ATM proximally with a groove with some sculpturing and distally smooth. Propodeum with transverse rugae, proximal half curved with medium-sized sculpture and distal half rugose; distal edge of propodeum with a flange at each side and without stubs; propodeal spiracle distally framed by a short concave carina; nucha surrounded by very short radiating carinae. Pronotum with a distinct dorsal furrow, dorsally with a well-defined smooth band; central area of pronotum smooth, but both dorsal and ventral furrows with short parallel carinae. Propleuron with a mix of rugae and fine punctation, dorsally with a carina present. Metasternum flat or nearly so. Contour of mesopleuron straight/angulate or nearly so; precoxal groove deep with transverse lineate sculpture; epicnemial ridge elongated more fusiform (tapering at both ends).

**Legs.** Ventral margin of fore telotarsus entire without seta, fore telotarsus almost same width throughout and longer than fourth tarsomere (0.13, 0.08). Hind coxa with punctation only on ventral surface, dorsal outer depression present. Inner spur of hind tibia longer than outer spur (0.40, 0.25), entire surface of hind tibia with dense strong spines clearly differentiated by color and length. Hind telotarsus as equal in length as fourth tarsomere (0.13, 0.14).

**Wings** (Fig. 168L, M). Fore wing with r vein curved; 2RS vein straight; r and 2RS veins forming a weak, even curve at their junction and outer side of junction not forming a stub; 2M vein slightly curved/swollen; distally fore wing [where spectral veins are] with microtrichiae more densely concentrated than the rest of the wing; anal cell 1/3 proximally lacking microtrichiae; subbasal cell with microtrichiae virtually throughout; vein 2CUa absent and vein 2CUb spectral; vein 2 cu-a absent; vein 2-1A proximally tubular and distally spectral, although sometimes difficult to see; tubular vein 1 cu-a curved, incomplete/broken and not reaching the edge of 1-1A vein. Hind wing with vannal lobe very narrow, subdistally and subproximally straightened, and setae evenly scattered in the margin.

**Metasoma** (Fig. 168A, D, E, H, J, K). Metasoma laterally compressed. Petiole finely sculptured on 3/4 proximal, virtually parallel-sided over most of length, but narrowing over distal 1/3 (length 0.45, maximum width 0.23, minimum width 0.12), and with scattered pubescence concentrated in the first distal third. Lateral grooves delimiting the median area on T2 clearly defined and reaching the distal edge of T2 (length median area 0.17, length T2 0.17), edges of median area polished and lateral grooves deep, median area broader than long (length 0.17, maximum width 0.27, minimum width 0.12); T2 with scattered pubescence only distally. T3 longer than T2 (0.28, 0.17) and with scattered pubescence throughout. Pubescence on hypopygium dense.

**Cocoons**. Unknown.

#### Comments.

The extent of the yellow spot in the hind coxae varies in size, the hind coxae are long (Fig. 168A), the distal half of propodeum is flat, and the spiracles propodeal are framed by a concave distal carina.

#### Male

(Fig. 169A–H). The mesosoma is stouter than females.

#### Etymology.

Michael (Mike) Joseph Sharkey is a Canadian braconologist who was a research scientist at the Canadian National Collection in Ottawa and later a professor at the University of Kentucky. Currently, he is the CEO and curator of the Hymenoptera Institute in Redlands CA, USA.

#### Distribution.

Adult parasitoids were collected in Costa Rica, ACG, Sector El Hacha (Sendero Bejuquilla), Sector Rincón Rain Forest (Vado Río Francia), and Sector San Cristóbal (Bosque Trampa Malaise, Estación San Gerardo, Potrero Argentina and Río Blanco Abajo), during December 1998, January and May-July 1999, June-December 2007, and January-May and September 2008 at 280 m, 400 m, 500 m, 520 m, 575 m, and 815 m in dry-rain intergrade and rain forests.

#### Biology.

Unknown.

#### Host.

Unknown.

### Glyptapanteles montywoodi

Taxon classificationAnimaliaHymenopteraBraconidae

Arias-Penna, sp. nov.

http://zoobank.org/EA825A32-F308-4883-A4FA-AEBD4348F98C

Fig. 170

#### Male.

Body length 2.68 mm, antenna length 3.78 mm, fore wing length 3.18 mm.

#### Type material.

**Holotype**: ECUADOR • 1♀; EC-26912, YY-A228; Napo, Yanayacu Biological Station, Sendero Macuculoma, Plot 370; cloud forest; 2,091 m; -0.6, -77.883333; 10.x.2007, Rafael Granizo leg.; caterpillar collected in second instar; cocoon formed on 18.x.2007; adult parasitoid emerged on 22.xi.2007; (PUCE).

#### Diagnosis.

**Figure 170.**
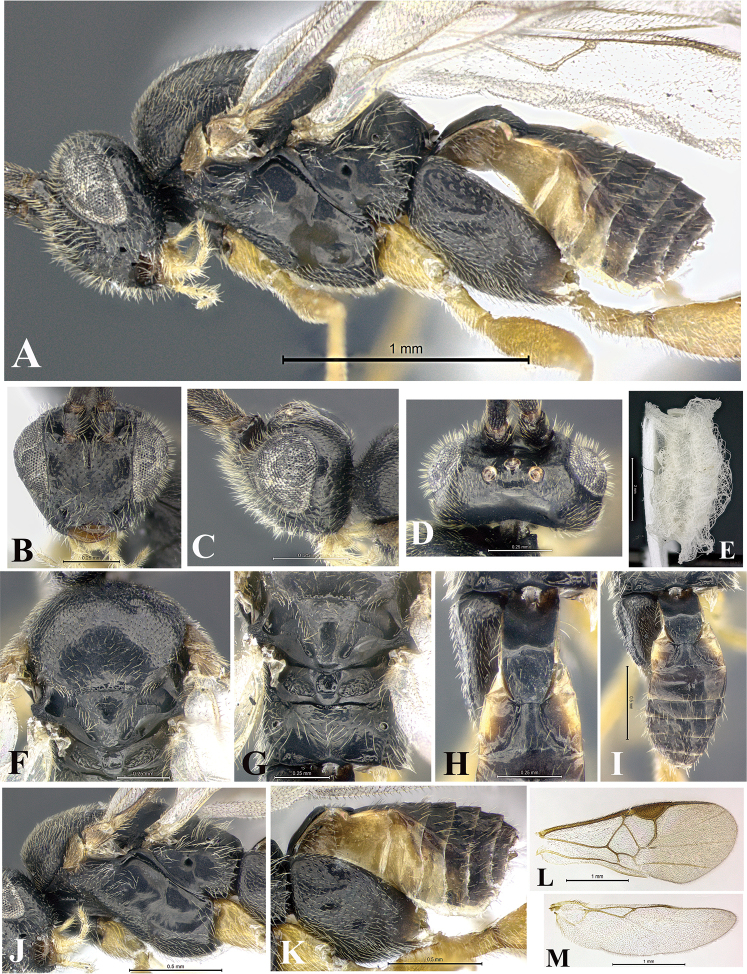
*Glyptapantelesmontywoodi* sp. nov. male EC-26912 YY-A228 **A** Habitus **B–D** Head **B** Frontal view **C** Lateral view **D** Dorsal view **E** Cocoon **F** Mesonotum, dorsal view **G** Scutellum, metanotum, propodeum, dorsal view **H**T1–2, dorsal view **I, K** Metasoma **I** Dorsal view **K** Lateral view **J** Mesosoma, lateral view **L, M** Wings **L** Fore **M** Hind.

Scutellum in profile slightly convex (Fig. 170A, J), fore wing with vein 2-1A proximally tubular, distally spectral, r vein slightly curved or curved, outer side of junction of r and 2RS veins forming a slight or distinct stub (Fig. 170L), median area on T2 distally with lateral margins curved (concave), edges of median area on T2 polished and followed by a deep groove, lateral grooves delimiting the median area clearly defined and reaching the distal edge of T2 (Fig. 170H, I), dorsal carina delimiting a dorsal furrow on propleuron absent, anterior furrow of metanotum without setiferous lobes (Fig. 170F, H), axillary trough of scutellum with sculpture (Fig. 170F, H), propodeum without median longitudinal carina (Fig. 170G), and anteroventral contour of mesopleuron convex (Fig. 170A, J).

#### Coloration

(Fig. 170A–M). General body coloration shiny black except apex of mandibles, labrum, and tegulae light brown-reddish; apex of pedicel light brown; scape and all antennal flagellomeres (on both sides) dark brown/black; glossa, maxillary and labial palps light yellow-brown. Eyes silver and ocelli yellow-brown. Fore and middle legs light yellow-brown and claws brown; hind legs light yellow-brown except black coxae distally brown-reddish, femora distally brown, tibiae completely brown, and tarsomeres brown. Petiole on T1 dark brown/black with contours darkened and sublateral areas light yellow-brown; T2 with median and adjacent areas brown, and lateral ends yellow; T3 and beyond completely brown; distally each tergum with a narrow yellow transparent band. In lateral view, T1-2 completely yellow-brown; T3–4 ventrally yellow, dorsally brown, extent of brown area wider in T4 than T3; T5 and beyond brown. S1 yellow-brown; S3–4 yellow; penultimate sternum and hypopygium brown.

#### Description.

**Head** (Fig. 170A–D). Head triangular with pubescence long and dense. Proximal three antennal flagellomeres longer than wide (0.23:0.09, 0.25:0.09, 0.25:0.09), distal antennal flagellomere longer than penultimate (0.15:0.06, 0.12:0.06), antenna longer than body (3.78, 2.68); antennal scrobes-frons sloped and forming a shelf. Face with dense fine punctations, distal half dented only laterally, interspaces smooth and longitudinal median carina present. Frons smooth. Temple wide, punctate and interspaces clearly smooth. Inner margin of eyes diverging slightly at antennal sockets; in lateral view, eye anteriorly convex and posteriorly straight. POL subequal in length with OOL (0.11, 0.11). Malar suture present. Median area between lateral ocelli without depression. Vertex laterally pointed or nearly so and dorsally wide.

**Mesosoma** (Fig. 170A, F, G, J). Mesosoma dorsoventrally convex. Mesoscutum proximally convex and distally flat, punctation distinct throughout, interspaces smooth. Scutellum triangular, apex sloped and fused with BS, but not in the same plane, scutellar punctation scattered throughout, in profile scutellum slightly convex, but on same plane as mesoscutum, phragma of the scutellum partially exposed; BS only very partially overlapping the MPM; ATS demilune with short stubs delineating the area; dorsal ATS groove with carinae only proximally. Transscutal articulation with small and homogeneous foveae, area just behind transscutal articulation sloped and with same kind of sculpture as mesoscutum. Metanotum with BM convex; MPM circular without median longitudinal carina; AFM without setiferous lobes and not as well delineated as PFM; PFM thick, smooth and with lateral ends rounded; ATM proximally with a groove with some sculpturing and distally smooth. Propodeum without median longitudinal carina, proximal half curved with medium-sized sculpture and distal half relatively polished and with a shallow dent at each side of nucha; distal edge of propodeum with a flange at each side and without stubs; propodeal spiracle without distal carina; nucha surrounded by very short radiating carinae. Pronotum with a distinct dorsal furrow, dorsally with a well-defined smooth band; central area of pronotum and dorsal furrow smooth, but ventral furrow with short parallel carinae. Propleuron with fine punctations throughout and dorsally without a carina. Metasternum flat or nearly so. Contour of mesopleuron convex; precoxal groove smooth, shiny and shallow, but visible; epicnemial ridge convex, teardrop-shaped.

**Legs.** Ventral margin of fore telotarsus entire without seta, fore telotarsus almost same width throughout and longer than fourth tarsomere (0.12, 0.06). Hind coxa with dorsal half sparsely punctate, ventral half densely punctate, and dorsal outer depression present. Inner spur of hind tibia longer than outer spur (0.41, 0.31), entire surface of hind tibia with dense strong spines clearly differentiated by color and length.

**Wings** (Fig. 170L, M). Fore wing with r vein slightly curved; 2RS vein straight; r and 2RS veins forming a weak, even curve at their junction and outer side of junction forming a slight stub; 2M vein slightly curved/swollen; distally fore wing [where spectral veins are] with microtrichiae more densely concentrated than the rest of the wing; anal cell 1/3 proximally lacking microtrichiae; subbasal cell with microtrichiae virtually throughout; veins 2CUa and 2CUb completely spectral; vein 2 cu-a absent; vein 2-1A proximally tubular and distally spectral, although sometimes difficult to see; tubular vein 1 cu-a curved, incomplete/broken and not reaching the edge of 1-1A vein. Hind wing with vannal lobe very narrow, subdistally and subproximally straightened, and setae evenly scattered in the margin.

**Metasoma** (Fig. 170A, H, I, K). Metasoma laterally compressed. Petiole on T1 completely smooth and polished, with faint, satin-like sheen, virtually parallel-sided over most of length with round apex (length 0.39, maximum width 0.19, minimum width 0.13), and with scattered pubescence concentrated in the first distal third. Lateral grooves delimiting the median area on T2 clearly defined and reaching the distal edge of T2 (length median area 0.15, length T2 0.15), lateral grooves deep, median area broader than long (length 0.15, maximum width 0.20, minimum width 0.08); T2 with scattered pubescence throughout. T3 longer than T2 (0.23, 0.15) and with scattered pubescence throughout.

**Cocoon** (Figs 4D, 170E). White lace-shaped cocoon with evenly smooth silk fibers.

#### Comments.

The ocelli are very close to each other (Fig. 170D, diameter of ocelli 0.06 mm, distance between median ocellus and lateral ocellus 0.03 mm), the margin of median area on T2 curved rather than straight (Fig. 170H), the propodeum proximally is curved and distally drops and looks like a slightly inclined wall (Fig. 170G), the limit between the mesopleuron and the metasternum has a dented area, the hind coxa is stout (Fig. 170K), and the mesosoma is broad and stout (Fig. 170F, J). The holotype has hind tarsomeres missing.

#### Female.

Unknown.

#### Etymology.

D. Monty Wood is a Canadian specialist on Tachinidae for which he is the world’s leading expert. He also has contributed to the knowledge of Simuliidae (black flies). He is an honorary research associate at Canadian National Collection (CNC) of Insect, Arachnids and Nematodes, Ottawa, Ontario, Canada. Monty divides his time between the CNC and Instituto Nacional de Biodiversidad (INBio), Santo Domingo de Heredia, Costa Rica.

#### Distribution.

Parasitized caterpillar was collected in Ecuador Napo, Yanayacu Biological Station (Sendero Macuculoma), during October 2007 at 2,091 m in cloud forest.

#### Biology.

The lifestyle of this parasitoid species is solitary.

#### Host.

Undetermined species of Erebidae (Arctiinae) feeding on *Chusqueascandens* (Poaceae). Caterpillar was collected in second instar.

### Glyptapanteles nataliaivanovae

Taxon classificationAnimaliaHymenopteraBraconidae

Arias-Penna, sp. nov.

http://zoobank.org/BC956E61-79D3-4C65-A00F-7E847F357B61

Figs 171 , 172

#### Female.

Body length 2.53 mm, antenna length 3.33 mm, fore wing length 2.58 mm.

#### Type material.

**Holotype**: COSTA RICA • 1♀; 98-SRNP-16065.1, DHJPAR0012754; Área de Conservación Guanacaste, Guanacaste, Sector El Hacha, Sendero Bejuquilla; intergrade dry-rain forest; Malaise; 280 m; 11.03004, -85.52699; 07.xii.1998; DH Janzen & W Hallwachs leg.; (CNC). **Paratypes.** • 1 (0♀, 0♂) (0♀, 1♂); 98-SRNP-16164, DHJPAR0013624; same data as for holotype except: 23.xi.1998; (CNC). • 1 (0♀, 0♂) (0♀, 1♂); 98-SRNP-16167, DHJPAR0013626; same data as for holotype except: 14.xii.1998; (CNC). • 1 (0♀, 1♂) (0♀, 0♂); 98-SRNP-16169, DHJPAR0013628; same data as for holotype except: 14.xii.1998; (CNC). • 1 (0♀, 0♂) (0♀, 1♂); 98-SRNP-16163, DHJPAR0013623; same data as for holotype except: 28.xii.1998; (CNC). • 1 (0♀, 0♂) (0♀, 1♂); 98-SRNP-16166, DHJPAR0013625; same data as for holotype except: 28.xii.1998; (CNC). • 1 (0♀, 0♂) (0♀, 1♂); 98-SRNP-16168, DHJPAR0013627; same data as for holotype except: 28.xii.1998; (CNC). • 1 (0♀, 0♂) (0♀, 1♂); 99-SRNP-19242, DHJPAR0013629; same data as for holotype except: 18.i.1999; (CNC). • 1 (0♀, 0♂) (0♀, 1♂); 99-SRNP-19243, DHJPAR0013630; same data as for holotype except: 18.i.1999; (CNC). • 1 (0♀, 0♂) (0♀, 1♂); 99-SRNP-19245, DHJPAR0013632; same data as for holotype except: 18.i.1999; (CNC).

#### Diagnosis.

**Figure 171.**
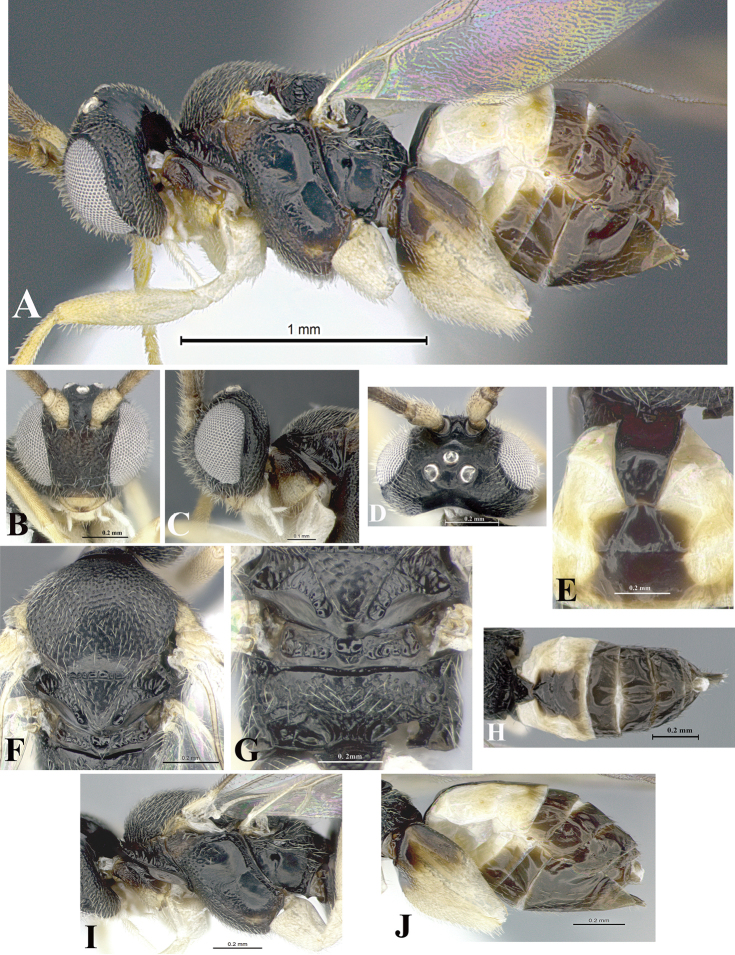
*Glyptapantelesnataliaivanovae* sp. nov. female 98-SRNP-16065.1 DHJPAR0012754 **A** Habitus **B, D** Head **B** Frontal view **D** Dorsal view **C** Head, pronotum, propleuron, lateral view **E**T1–3, dorsal view **F** Mesonotum, dorsal view **G** Scutellum, metanotum, propodeum, dorsal view **H, J** Metasoma **H** Dorsal view **J** Lateral view **I** Mesosoma, lateral view.

**Figure 172.**
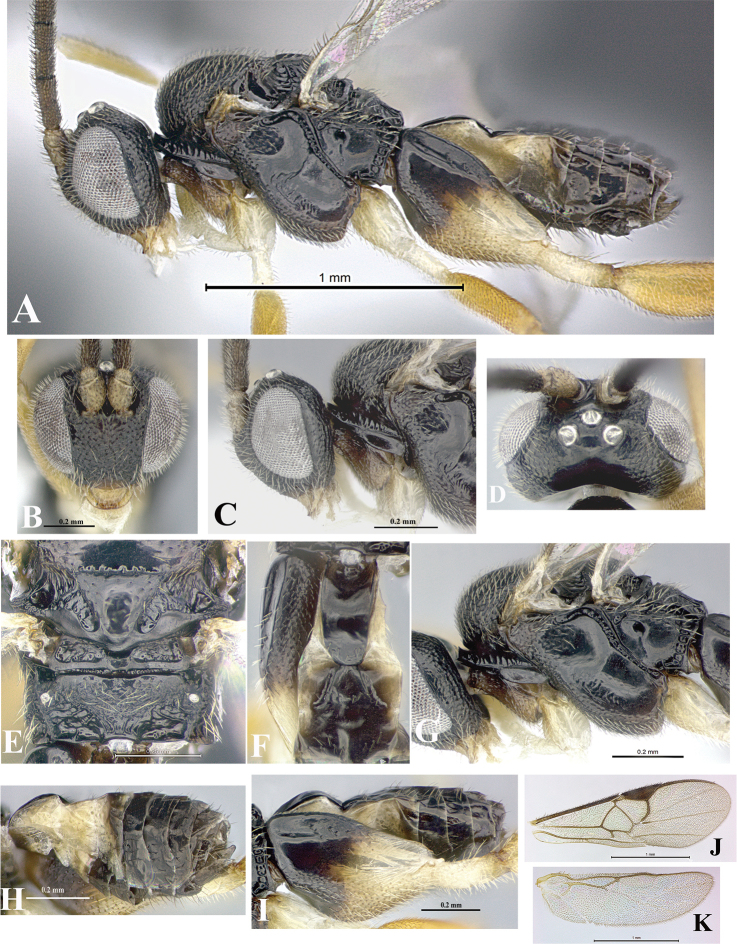
*Glyptapantelesnataliaivanovae* sp. nov. male 98-SRNP-16169 DHJPAR0013628, 07-SRNP-67011 DHJPAR0025549 **A** Habitus **B, D** Head **B** Frontal view **D** Dorsal view **C** Head, pronotum, propleuron, lateral view **E** Scutellum, metanotum, propodeum, dorsal view **F**T1–2, dorsal view **G** Mesosoma, lateral view **H** Metasoma, lateral view **I** Hind coxa, lateral view **J, K** Wings **J** Fore **K** Hind.

Petiole on T1 parallel-sided in proximal half, then narrowing, finely sculptured (Figs 171E, H, 172F), propodeum without median longitudinal carina (Figs 171G, 172E), fore wing with vein 1 cu-a straight, r vein curved, outer side of junction of r and 2RS veins not forming a stub (Fig. 172J), dorsal outer depression on hind coxa present (Figs 171A, J, 172A, I), inner margin of eyes diverging slightly at antennal sockets (Figs 171B, 172B), and lateral grooves delimiting the median area on T2 clearly defined and reaching the distal edge of T2 (Figs 171E, H, 172F).

#### Coloration

(Fig. 171A–J). General body coloration brown-black except scape yellow with a lateral brown band; pedicel, labrum, mandible and glossa yellow; first four proximal antennal flagellomeres dorsally lighter (light brown) than ventrally (dark brown), remaining flagellomeres dark brown on both sides; maxillary and labial palps, and tegulae ivory/pale yellow; propleuron proximal half yellow-brown/reddish and distal half yellow; central area of distal half on low face, ventral furrow of pronotum, distally dorsal furrow of pronotum, epicnemial ridge, and a small dot in the ventral edge of mesopleuron with yellow-brown/reddish tints. Eyes and ocelli silver. Fore and middle legs ivory/pale yellow and claws brown; hind legs yellow except coxae which distal half with unevenly brown/yellow-brown blotches, trochanter and trochantellus ivory/pale yellow, distally femora with a tiny brown dot, and distally tibiae and tarsomeres light brown. Petiole on T1 black and sublateral areas ivory/pale yellow; T2 with median and adjacent areas brown, adjacent area with contours well-defined, and lateral ends ivory/pale yellow; T3 broadly brown, proximally shape of the dark area coinciding with the width of median and adjacent areas on T2, but distally shape slightly wider, lateral ends with proximal half ivory/pale yellow and distal half with two bands: a brown one followed by a yellow-brown band; T4 and beyond completely brown; distally each tergum with a narrow yellowish transparent area. In lateral view, T1–2 completely ivory/pale yellow; T3 ivory/pale yellow, but dorsally brown; T4 and beyond completely brown. S1–3 yellow; S4 and beyond brown.

#### Description.

**Head** (Fig. 171A–D). Head rounded with pubescence long and dense. Proximal three antennal flagellomeres length longer than wide (0.21:0.07, 0.22:0.07, 0.23:0.07), distal antennal flagellomere longer than penultimate (0.13:0.06, 0.10:0.06), antenna longer than body (3.33, 2.53); antennal scrobes-frons shallow. Face flat or nearly so, punctate-lacunose, interspaces wavy and longitudinal median carina present. Frons smooth. Temple wide, punctate-lacunose and interspaces wavy. Inner margin of eyes diverging slightly at antennal sockets; in lateral view, eye anteriorly convex and posteriorly straight. POL shorter than OOL (0.08, 0.10). Malar suture present. Median area between lateral ocelli slightly depressed. Vertex laterally pointed or nearly so and dorsally wide.

**Mesosoma** (Fig. 171A, F, G, I). Mesosoma dorsoventrally convex. Mesoscutum proximally convex and distally flat, punctation distinct proximally with polished area distally, interspaces wavy/lacunose. Scutellum triangular, apex sloped and fused with BS, scutellar punctation distinct throughout, in profile scutellum flat and on same plane as mesoscutum, phragma of the scutellum partially exposed; BS only very partially overlapping the MPM; ATS demilune with quite a little, complete and parallel carinae; dorsal ATS groove with semicircular/parallel carinae. Transscutal articulation with small and heterogeneous foveae, area just behind transscutal articulation smooth, shiny and depressed centrally. Metanotum with BM wider than PFM (clearly differentiated); MPM circular and bisected by a median longitudinal carina; AFM with a small lobe and not as well delineated as PFM; PFM thick, smooth and with lateral ends rounded; ATM proximally with a groove with some sculpturing and distally smooth. Propodeum without median longitudinal carina, proximal half curved with medium-sized sculpture and distal half rugose; distal edge of propodeum with a flange at each side and without stubs; propodeal spiracle distally framed by a short concave carina; nucha surrounded by very short radiating carinae. Pronotum with a distinct dorsal furrow, dorsally with a narrow band; central area of pronotum and both dorsal and ventral furrows with sculpture. Propleuron with fine rugae and dorsally with a carina. Metasternum flat or nearly so. Contour of mesopleuron convex; precoxal groove deep with transverse lineate sculpture; epicnemial ridge elongated more fusiform (tapering at both ends).

**Legs.** Ventral margin of fore telotarsus entire without seta, fore telotarsus almost same width throughout and longer than fourth tarsomere (0.10, 0.07). Hind coxa with punctation only on ventral surface, dorsal outer depression present. Inner spur of hind tibia longer than outer spur (0.26, 0.18), entire surface of hind tibia with dense strong spines clearly differentiated by color and length. Hind telotarsus longer than fourth tarsomere (0.13, 0.11).

**Wings** (Fig. 172J, K). Fore wing with r vein curved; 2RS vein straight; r and 2RS veins forming a weak, even curve at their junction and outer side of junction not forming a stub; 2M vein straight; distally fore wing [where spectral veins are] with microtrichiae more densely concentrated than the rest of the wing; anal cell 1/3 proximally lacking microtrichiae; subbasal cell with microtrichiae virtually throughout; veins 2CUa and 2CUb completely spectral; vein 2 cu-a present as spectral vein, sometimes difficult to see; vein 2-1A proximally tubular and distally spectral, although sometimes difficult to see; tubular vein 1 cu-a straight, incomplete/broken and not reaching the edge of 1-1A vein. Hind wing with vannal lobe very narrow, subdistally and subproximally straightened, and setae evenly scattered in the margin.

**Metasoma** (Fig. 171A, E, H, J). Metasoma laterally compressed. Petiole on T1 finely sculptured only laterally, parallel-sided in proximal half and then narrowing (length 0.30, maximum width 0.18, minimum width 0.10), and with scattered pubescence concentrated in the first distal third. Lateral grooves delimiting the median area on T2 clearly defined and reaching the distal edge of T2 (length median area 0.15, length T2 0.15), edges of median area polished and lateral grooves deep, median area broader than long (length 0.15, maximum width 0.17, minimum width 0.08); T2 with scattered pubescence only distally. T3 longer than T2 (0.25, 0.15) and with scattered pubescence only distally. Pubescence on hypopygium dense.

**Cocoon.** Unknown.

#### Comments.

The fore tarsomeres are missing; the hind coxae are stout (Fig. 171A).

#### Male

(Fig. 172A–K). The fore and middle legs have yellow trochanters, the trochantellus are ivory/pale yellow, proximally the coxae is darker than distally and the claws are brown; the hind legs are yellow except the distal half of the coxae which is black, the trochanter and the trochantellus are ivory/pale yellow, the femora distally have a tiny brown dot, the coloration of tibiae and tarsi is yellow/light yellow-brown.

#### Etymology.

Natalia Ivanova is (since 2004) the lead DNA scientist at Biodiversity Institute of Ontario (BIO), University of Guelph, Ontario, Canada.

#### Distribution.

Adult parasitoids were collected in Costa Rica, Sector El Hacha (Sendero Bejuquilla), during November-December 1998 and January 1999 at 280 m and 400 m in intergrade dry-rain and rain forests.

#### Biology.

Unknown.

#### Host.

Unknown.

### Glyptapanteles nealweberi

Taxon classificationAnimaliaHymenopteraBraconidae

Arias-Penna, sp. nov.

http://zoobank.org/910DDC24-826B-472E-87F6-A91D3BD6390D

Figs 173 , 174

#### Female.

Body length 2.73 mm, antenna length 3.38 mm, fore wing length 3.03 mm.

#### Type material.

**Holotype**: COSTA RICA • 1♀; 07-SRNP-67067, DHJPAR0025605; Área de Conservación Guanacaste, Alajuela, Sector San Cristóbal, Potrero Argentina; pastures; Malaise; 520 m; 10.89021, -85.38803; 02.x.2007; DH Janzen & W Hallwachs leg.; (CNC). **Paratypes**. • 1 (1♀, 0♂) (0♀, 0♂); 07-SRNP-67066, DHJPAR0025604; same data as for holotype except: 15.viii.2007; (CNC). • 1 (0♀, 1♂) (0♀, 0♂); 07-SRNP-67116, DHJPAR0025654; same data as for holotype except: 28.xii.2007; (CNC). • 1 (0♀, 0♂) (0♀, 1♂); 07-SRNP-67758, DHJPAR0027496; same data as for holotype except: 09.viii.2007; (CNC). • 1 (0♀, 1♂) (0♀, 0♂); 07-SRNP-67765, DHJPAR0027503; same data as for holotype except: 09.viii.2007; (CNC).

#### Other material.

**Reared material.** COSTA RICA: *Área de Conservación Guanacaste*, *Guanacaste*, *Sector Pitilla*, *Coneja*: • 1 (1♀, 0♂) (0♀, 0♂); 09-SRNP-73650, DHJPAR0038202; rain forest; 415 m; 11.01525, -85.39766; 07.xi.2009; Ricardo Calero leg.; caterpillar collected in second instar; cocoon adhered to the leaf substrate and formed on 19.xi.2009; adult parasitoid emerged on 26.xi.2009.

*Área de Conservación Guanacaste*, *Guanacaste*, *Sector Pitilla*, *Sendero Naciente*: • 1 (0♀, 0♂) (1♀, 0♂); 11-SRNP-30888, DHJPAR0043084; rain forest; 700 m; 10.98705, -85.42816; 01.iv.2011; Manuel Rios leg.; caterpillar collected in second instar; cocoon adhered to the leaf substrate and formed on 08.iv.2011; adult parasitoid emerged on 20.iv.2011.

*Área de Conservación Guanacaste*, *Alajuela*, *Sector Rincón Rain Forest*, *Quebrada Escondida*: • 1 (0♀, 0♂) (1♀, 0♂); 10-SRNP-40104, DHJPAR0039041; 420 m; 10.89928, -85.27486; 14.i.2010; José Pérez leg.; caterpillar collected in fifth instar; very small very dark cylinder cocoon adhered to the leaf substrate and formed on 18.i.2010; adult parasitoid emerged on 13.iii.2010.

*Área de Conservación Guanacaste*, *Alajuela*, *Sector Rincón Rain Forest*, *Conguera*: • 1 (0♀, 0♂) (1♀, 0♂); 10-SRNP-44571, DHJPAR0041885; 420 m; 10.91589, -85.26631; 30.xi.2010; Pablo Umaña Calderon leg.; caterpillar collected in third instar; cocoon adhered to the leaf substrate and formed on 06.xii.2010; adult parasitoid emerged on 13.xii.2010. • 1 (0♀, 0♂) (1♀, 0♂); 10-SRNP-44572, DHJPAR0041930; same data as for preceding except: single white cocoon; adult parasitoid emerged on 18.xii.2010. • 1 (0♀, 0♂) (0♀, 1♂); 10-SRNP-44609, DHJPAR0041925; same data as for precedig except: Jorge Hernández; single white cocoon.

#### Malaise-trapped material.

COSTA RICA: *Área de Conservación Guanacaste*, *Guanacaste*, *Sector El Hacha*, *Sendero Bejuquilla*: • 1♀ (1♀, 0♂) (0♀, 0♂); 98-SRNP-16035, DHJPAR0012622; intergrade dry-rain forest; Malaise; 280 m; 11.03004, -85.52699; 16.xi.1998; DH Janzen & W Hallwachs leg.

*Área de Conservación Guanacaste*, *Alajuela*, *Sector San Cristóbal*, *Potrero Argentina*: • 1♀; 07-SRNP-67049, DHJPAR0025587; pastures; Malaise; 520 m; 10.89021, -85.38803; 10.vii.2007; DH Janzen & W Hallwachs leg.; whole specimen was used for DNA extraction.

*Área de Conservación Guanacaste*, *Alajuela*, *Sector San Cristóbal*, *Río Blanco Abajo*: • 1♀ (1♀, 0♂) (0♀, 0♂); 07-SRNP-66364, DHJPAR0024902; Malaise; rain forest; 500 m; 10.90037, -85.37254; 02.x.2007; DH Janzen & W Hallwachs leg. • 1♀ (0♀, 0♂) (1♀, 0♂); 07-SRNP-66391, DHJPAR0024929; same data as for preceding except: 21.viii.2007. • 1 (0♀, 0♂) (1♀, 0♂); 07-SRNP-66677, DHJPAR0025215; same data as for preceding except: 22.vii.2007. • 1 (0♀, 0♂) (0♀, 1♂); 07-SRNP-67716, DHJPAR0026411; same data as for preceding except: 13.xi.2007. • 1 (0♀, 1♂) (0♀, 0♂); 08-SRNP-3127, DHJPAR0026708; same data as for preceding except: 18.iii.2008.

*Área de Conservación Guanacaste*, *Alajuela*, *Sector San Cristóbal*, *Bosque trampa Malaise*: • 1 (0♀, 1♂) (0♀, 0♂); 07-SRNP-67363, DHJPAR0025901; rain forest; 815 m; 10.86280, -85.38460; 22.vii.2007; DH Janzen & W Hallwachs leg. • 1 (0♀, 0♂) (0♀, 1♂); 07-SRNP-67373, DHJPAR0025911; same data as for preceding.

*Área de Conservación Guanacaste*, *Alajuela*, *Sector Rincón Rain Forest*, *Vado Río Francia*: • 1 (0♀, 1♂) (0♀, 0♂); 07-SRNP-66846, DHJPAR0025384; Malaise; 400 m; 10.90093, -85.28915; 01.xii.2007; DH Janzen & W Hallwachs leg.

#### Diagnosis.

**Figure 173.**
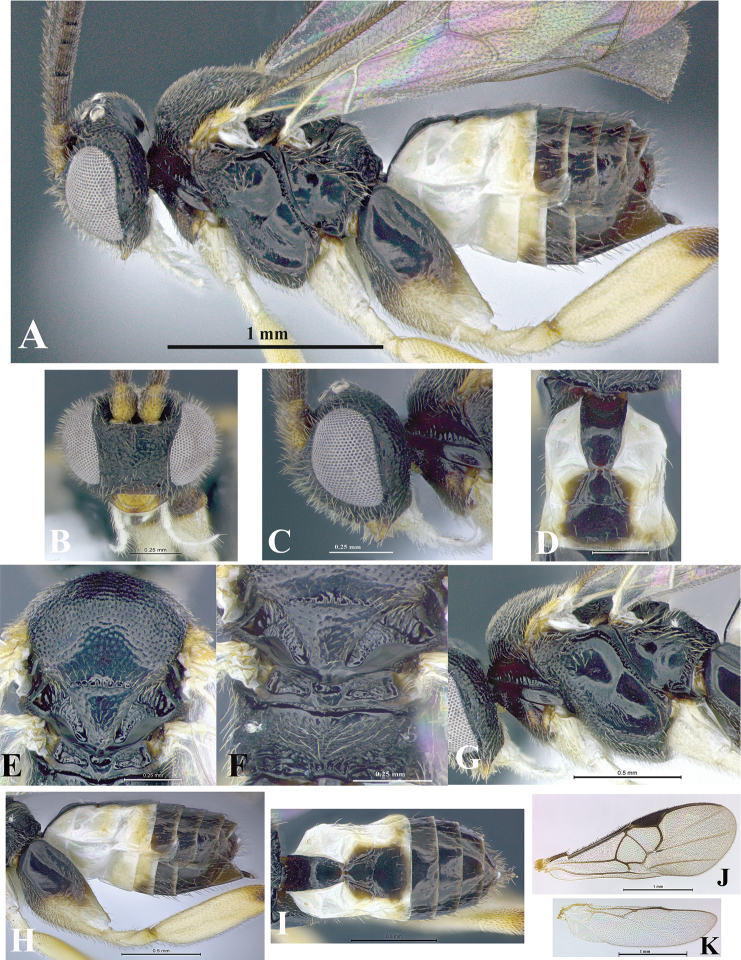
*Glyptapantelesnealweberi* sp. nov. female 07-SRNP-67066 DHJPAR0025604, 07-SRNP-66364 DHJPAR0024902 **A** Habitus **B** Head, frontal view **C** Head, pronotum, propleuron, lateral view **D**T1–3, dorsal view **E** Mesonotum, dorsal view **F** Scutellum, metanotum, propodeum, dorsal view **G** Mesosoma, lateral view **H, I** Metasoma **H** Lateral view **I** Dorsal view **J, K** Wings **J** Fore **K** Hind.

**Figure 174.**
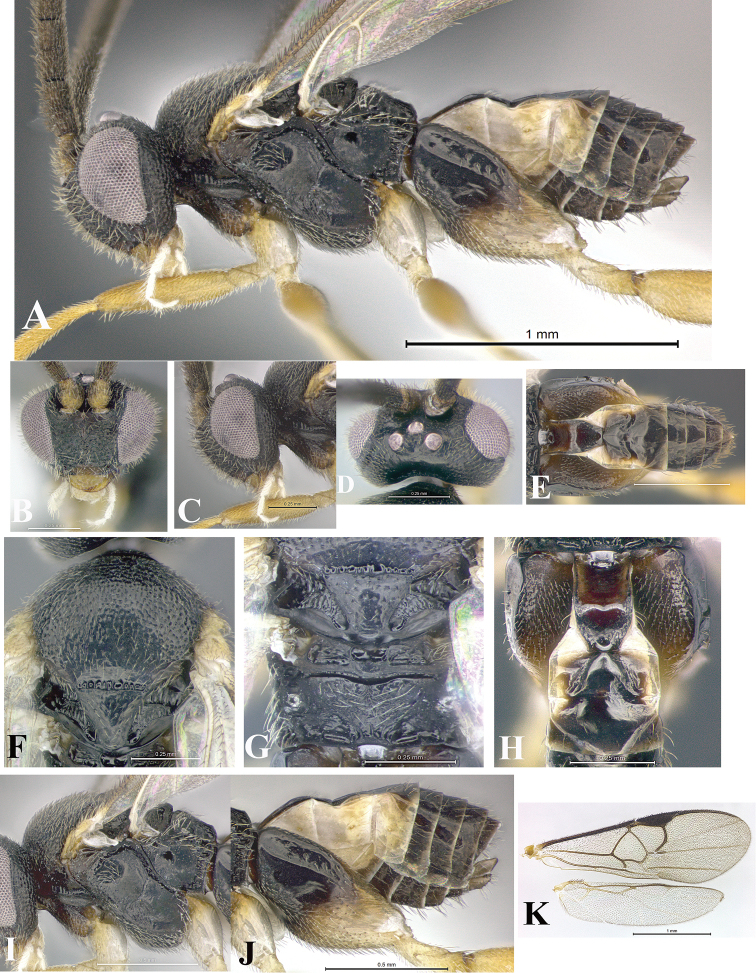
*Glyptapantelesnealweberi* sp. nov. male 07-SRNP-67116 DHJPAR0025654, 07-SRNP-66846 DHJPAR0025384 **A** Habitus **B, D** Head **B** Frontal view **D** Dorsal view **C** Head, pronotum, propleuron, lateral view **E, J** Metasoma **E** Dorsal view **J** Lateral view **F** Mesonotum, dorsal view **G** Scutellum, metanotum, propodeum, dorsal view **H**T1–3, dorsal view **I** Mesosoma, lateral view **K** Fore and hind wings.

Ventral margin of fore telotarsus slightly excavated and with a tiny curved seta, medioanterior pit of metanotum semicircular and bisected by a median longitudinal carina (Figs 173F, 174G), propleuron with fine rugae, dorsal carina delimiting a dorsal furrow present (Figs 173A, C, G, 174C, I), antenna longer than body, anterior furrow of metanotum with a small lobe, without setae (Figs 173F, 174G), distal antennal flagellomere longer than penultimate, surface of metasternum convex, precoxal groove deep with lineate sculpture (Figs 173A, G, 174A, I), fore wing with vein 1 cu-a curved, r vein curved (Figs 173J, 174K), dorsal outer depression on hind coxa present (Figs 173H, 174J), inner margin of eyes diverging slightly at antennal sockets (Figs 173B, 174B), petiole on T1 finely sculptured only laterally (Figs 173D, I, 174H), and lateral grooves delimiting the median area on T2 clearly defined and reaching the distal edge of T2 (Figs 173D, I, 174H).

#### Coloration

(Fig. 173A–K). General body coloration shiny black except scape and pedicel yellow, both with a lateral brown band; clypeus brown; labrum, mandible, glossa, and tegulae yellow; all antennal flagellomeres dark brown on both sides; maxillary and labial palps ivory/pale yellow; apex of propleuron, dorsal furrow of pronotum, epicnemial ridge, a narrow ventral band on mesopleuron, distal-lateral corners of mesoscutum, apex of scutellum, and lateral ends of metanotum with yellow-brown/reddish tints. Eyes and ocelli silver. Fore and middle legs ivory/pale yellow except most of the femora, tibiae and tarsomeres dark yellow, and claws brown; hind legs ivory/pale yellow except distal half of coxae with evenly black coloration, distally femora with a tiny brown dot, tibiae and tarsomeres reddish/orange. Petiole on T1 polished black, contours darkened and sublateral areas ivory/pale yellow; T2 with median area black, wide adjacent area yellow-brown, median and adjacent areas together forming a rectangle-shaped, and lateral ends ivory/pale yellow; T3 broadly brown, proximally brown area coinciding with the distal width of median plus adjacent areas on T2, proximal half of lateral ends ivory/pale yellow and distal half yellow-brown; T4 and beyond completely brown; distally each tergum with a wide yellow-brown transparent band. In lateral view, T1–3 ivory/pale yellow; T4 and beyond brown. S1–3 ivory/pale yellow; S4 yellow, but medially with a brown area; penultimate sternum and hypopygium completely brown.

#### Description.

**Head** (Fig. 173A–C). Head rounded with pubescence long and dense. Proximal three antennal flagellomeres longer than wide (0.23:0.08, 0.26:0.08, 0.25:0.08), distal antennal flagellomere longer than penultimate (0.14:0.06, 0.11:0.06), antenna longer than body (3.38, 2.73); antennal scrobes-frons shallow. Face flat or nearly so, punctate-lacunose, interspaces wavy and longitudinal median carina present. Frons smooth. Temple wide, punctate-lacunose and interspaces wavy. Inner margin of eyes diverging slightly at antennal sockets; in lateral view, eye anteriorly convex and posteriorly straight. POL shorter than OOL (0.09, 0.12). Malar suture present. Median area between lateral ocelli slightly depressed. Vertex laterally pointed or nearly so and dorsally wide.

**Mesosoma** (Fig. 173A, E–G). Mesosoma dorsoventrally convex. Mesoscutum proximally convex and distally flat, punctation distinct proximally with polished area distally, interspaces wavy/lacunose. Scutellum triangular, apex sloped and fused with BS, scutellar punctation distinct throughout, in profile scutellum slightly convex, but on same plane as mesoscutum, phragma of the scutellum partially exposed; BS only very partially overlapping the MPM; ATS demilune with quite a little, complete and parallel carinae; dorsal ATS groove with semicircular/parallel carinae. Transscutal articulation with small and heterogeneous foveae, area just behind transscutal articulation nearly at the same level as mesoscutum (flat), smooth and shiny. Metanotum with BM wider than PFM (clearly differentiated); MPM semicircular and bisected by a median longitudinal carina; AFM with a small lobe and not as well delineated as PFM; PFM thick, smooth and with lateral ends rounded; ATM proximally with a groove with some sculpturing and distally smooth. Propodeum without median longitudinal carina, proximal half curved with medium-sized sculpture and distal half rugose; distal edge of propodeum with a flange at each side and without stubs; propodeal spiracle distally framed by a short concave carina; nucha surrounded by very short radiating carinae. Pronotum with a distinct dorsal furrow, dorsally with a well-defined smooth band; central area of pronotum and both dorsal and ventral furrows with sculpture. Propleuron with fine rugae and dorsally without a carina. Metasternum convex. Contour of mesopleuron convex, precoxal groove deep with transverse lineate sculpture; epicnemial ridge elongated more fusiform (tapering at both ends).

**Legs.** Ventral margin of fore telotarsus slightly excavated and with a tiny curved seta, fore telotarsus almost same width throughout and longer than fourth tarsomere (0.12, 0.07). Hind coxa with punctation only on ventral surface, dorsal outer depression present. Inner spur of hind tibia longer than outer spur (0.34, 0.21), entire surface of hind tibia with dense strong spines clearly differentiated by color and length. Hind telotarsus as equal in length as fourth tarsomere (0.15, 0.15).

**Wings** (Fig. 173J, K). Fore wing with r vein curved; 2RS vein straight; r and 2RS veins forming a weak, even curve at their junction and outer side of junction not forming a stub; 2M vein slightly curved/swollen; distally fore wing [where spectral veins are] with microtrichiae more densely concentrated than the rest of the wing; anal cell 1/3 proximally lacking microtrichiae; subbasal cell with microtrichiae virtually throughout; vein 2CUa absent and vein 2CUb spectral; vein 2 cu-a absent; vein 2-1A proximally tubular and distally spectral, although sometimes difficult to see; tubular vein 1 cu-a curved, incomplete/broken and not reaching the edge of 1-1A vein. Hind wing with vannal lobe narrow, subdistally and subproximally straightened, and setae present only proximally.

**Metasoma** (Fig. 173A, D, H, I). Metasoma laterally compressed. Petiole on T1 finely sculptured only laterally, virtually parallel-sided over most of length, but narrowing over distal 1/3 (length 0.42, maximum width 0.20, minimum width 0.10) and with scattered pubescence concentrated in the first distal third. Lateral grooves delimiting the median area on T2 clearly defined and reaching the distal edge of T2 (length median area 0.16, length T2 0.16), edges of median area polished and lateral grooves deep, median area broader than long (length 0.16, maximum width 0.22, minimum width 0.08); T2 with scattered pubescence only distally. T3 longer than T2 (0.21, 0.16) and with scattered pubescence only distally. Pubescence on hypopygium dense.

**Cocoon.** Beige or white oval cocoon with evenly smooth silk fibers. Cocoon adhered to the leaf substrate.

#### Male

(Fig. 174A–K). The sterna of metasoma are darker than female.

#### Etymology.

Neal A. Weber’s interests are focused on ants (Myrmicinae, Attini). He works at the University of North Dakota, Grand Forks, ND, USA.

#### Distribution.

The parasitized caterpillars were collected in Costa Rica, ACG, Sector Rincón Rain Forest (Conguera, Quebrada Escondida), Sector Pitilla (Coneja, Sendero Naciente), and Sector San Cristóbal (Potrero Argentina), during November 2009; January and October-November 2010; and April 2011 at 415, 420 and 700 m in rain forest.

Adult parasitoids were collected in Costa Rica, ACG, Sector El Hacha (Sendero Bejuquilla), Sector Rincón Rain Forest (Vado Río Francia), and Sector San Cristóbal (Bosque trampa Malaise, Río Blanco Abajo, and Potrero Argentina), during November 1998, July-August and October-December 2007, and March 2008 at 280 m, 400 m, 500 m, 520 m, and 815 m in pasture, intergrade dry-rain, and rain forests.

#### Biology.

The lifestyle of this parasitoid species is solitary.

#### Host.

*Rejectaria* sp. Guenée (Erebidae: Herminiinae) feeding on *Alsophilafirma*, *Cyatheamultiflora*, *C.trichiata* (Cyatheaceae) and *Serpocaulonmaritimum* (Polypodiaceae). *Scopiferaantelia* Druce (Erebidae: Herminiinae) feeding on *Cyatheamultiflora* and *C.trichiata* (Cyatheaceae). Caterpillars were collected in second, third and fifth instar.

### Glyptapanteles ninazitaniae

Taxon classificationAnimaliaHymenopteraBraconidae

Arias-Penna, sp. nov.

http://zoobank.org/00B7231F-9E68-4072-9618-0ECF6B906854

Figs 175 , 176

#### Female.

Body length 3.78 mm, antenna length 4.55 mm, fore wing length 3.33 mm.

#### Type material.

**Holotype**: COSTA RICA •1♀; 00-SRNP-23990, DHJPAR0013364;

Área de Conservación Guanacaste, Guanacaste, Sector Santa Rosa, Bosque Humedo; dry forest; Malaise; 290 m; 10.85145, -85.60801; 07.ii.2000; DH Janzen & W Hallwachs leg.; (CNC). **Paratypes**. • 1 (0♀, 1♂) (0♀, 0♂); 00-SRNP-23972, DHJPAR0013603; same data as for holotype except: 17.i.2000; (CNC). • 1 (0♀, 0♂) (1♀, 0♂); 00-SRNP-23975, DHJPAR0013363; same data as for holotype; (CNC).

#### Other material.

**Malaise-trapped material.** COSTA RICA: *Área de Conservación Guanacaste*, *Guanacaste*, *Sector El Hacha*, *Sendero Bejuquilla*: • 1 (0♀, 1♂) (0♀, 0♂); 99-SRNP-19251, DHJPAR0013638; dry-rain intergrade forest; Malaise; 280 m; 11.03004, -85.52699; 05.vii.1999; DH Janzen & W Hallwachs leg.

*Área de Conservación Guanacaste*, *Alajuela*, *Sector San Cristóbal*, *Río Blanco Abajo*: • 1 (0♀, 0♂) (0♀, 1♂); 08-SRNP-3559, DHJPAR0027140; rain forest; Malaise; 500 m; 10.90037, -85.37254; 23.iv.2008; DH Janzen & W Hallwachs leg.

#### Diagnosis.

**Figure 175.**
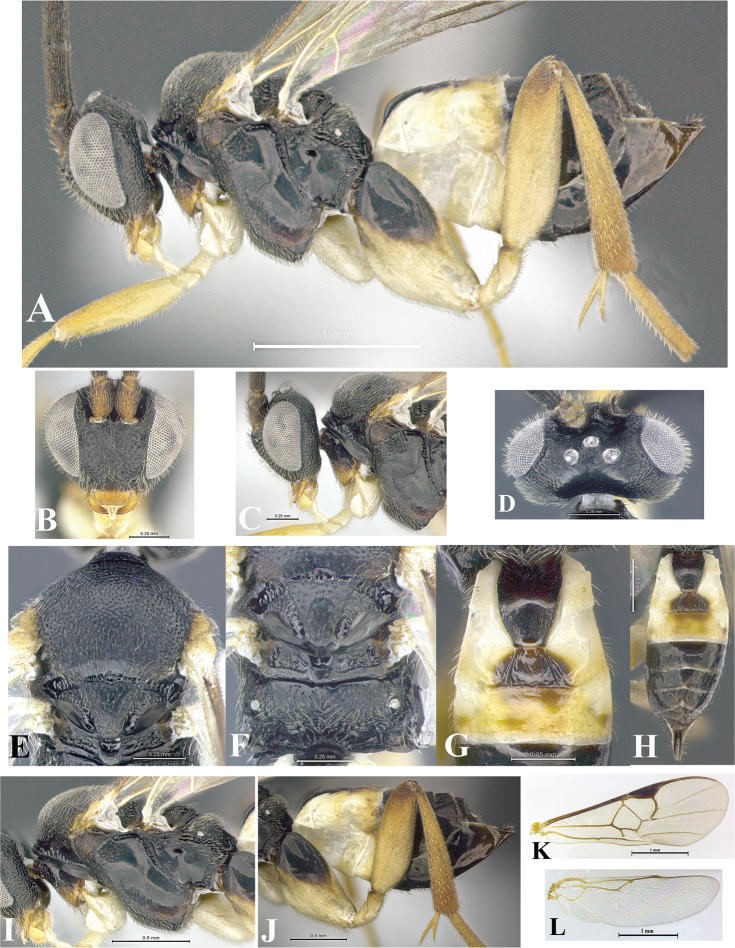
*Glyptapantelesninazitaniae* sp. nov. female 00-SRNP-23990 DHJPAR0013364, 99-SRNP-18929 DHJPAR0012623 **A** Habitus **B, D** Head **B** Frontal view **D** Dorsal view **C** Head, pronotum, propleuron, lateral view **E** Mesonotum, dorsal view **F** Scutellum, metanotum, propodeum, dorsal view **G**T1–3, dorsal view **H, J** Metasoma **H** Dorsal view **J** Lateral view **I** Mesosoma, lateral view **K, L** Wings **K** Fore **L** Hind.

**Figure 176.**
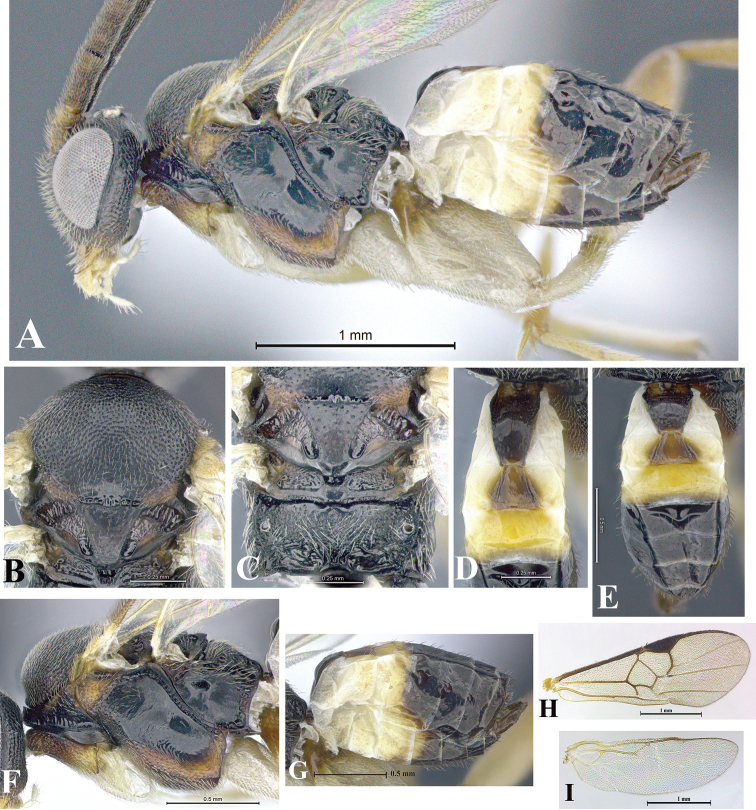
*Glyptapantelesninazitaniae* sp. nov. male 99-SRNP-19251 DHJPAR0013638, 08-SRNP-2976 DHJPAR0026557 **A** Habitus **B** Mesonotum, dorsal view **C** Scutellum, metanotum, propodeum, dorsal view **D**T1–3, dorsal view **E, G** Metasoma **E** Dorsal view **G** Lateral view **F** Mesosoma, lateral view **H, I** Wings **H** Fore **I** Hind.

Fore wing with vein 2 cu-a present as spectral vein, sometimes difficult to see, vein 1 cu-a straight, r vein slightly curved or curved, outer side of junction of r and 2RS veins not forming a stub (Figs 175K, 176H), dorsal groove on axillary trough of scutellum with parallel carinae (Figs 175F, 176C), propodeum with a median longitudinal dent (Figs 175F, 176C), mesoscutum proximally distinctly punctate, distally with a polished area (Figs 175E, 176B), temple punctate-lacunose, petiole virtually parallel-sided over most of length, but narrowing over distal 1/3, finely sculptured only laterally (Figs 175G, H, 176D, E), dorsal outer depression on hind coxa present (Fig. 175A, J), inner margin of eyes diverging slightly at antennal sockets (Fig. 175B), and lateral grooves delimiting the median area on T2 clearly defined and reaching the distal edge of T2 (Figs 175G, H, 176D, E).

#### Coloration

(Fig. 175A–L). General body coloration satin black except scape and pedicel yellow-brown with lateral brown band; last four-five distal antennal flagellomeres completely yellow, remaining flagellomeres dark brown on both sides; labrum, mandible, glossa, and tegulae yellow-brown; both ends of propleuron, some small areas of metasternum, a small ventral band of mesopleuron, epicnemial ridge, distal corners of mesoscutum, lateral ends of metapleuron, and distally lunules yellow-brown/reddish; maxillary and labial palps ivory/pale yellow. Eyes and ocelli silver. Fore and middle legs dark yellow, except coxae and trochanter ivory/pale yellow, and claws brown; hind legs pale yellow except coxae proximally with an elongate black spot, femora distally with a tiny brown dot, tibiae and tarsomeres dark yellow. Petiole on T1 dark brown, contours darkened and sublateral areas ivory/pale yellow; T2 with median area black although medially reddish/brown, wide adjacent area yellow-brown, and lateral ends ivory/pale yellow with some tints yellow-brown; T3 yellow with two small brown spots medially; T4 and beyond completely brown; distally each tergum with a narrow ivory/pale yellow transparent band. In lateral view, T1–2 completely ivory/pale yellow; T3 proximal half ivory/pale yellow, distal half with some yellow-brown tints; T4 and beyond completely brown. S1–3 completely yellow; S4 yellow, but medially brown; penultimate sternum and hypopygium brown.

#### Description.

**Head** (Fig. 175A–D). Head rhomboid with pubescence long and dense. Proximal three antennal flagellomeres longer than wide (0.33:0.10, 0.32:0.10, 0.32:0.10), distal antennal flagellomere longer than penultimate (0.25:0.11, 0.19:0.11), antenna longer than body (4.55, 3.78); antennal scrobes-frons shallow. Face flat or nearly so, punctate-lacunose, interspaces wavy and longitudinal median carina present. Frons smooth. Temple wide, punctate-lacunose and interspaces wavy. Inner margin of eyes diverging slightly at antennal sockets; in lateral view, eye anteriorly convex and posteriorly straight. POL shorter than OOL (0.10, 0.12). Malar suture present. Median area between lateral ocelli slightly depressed. Vertex laterally pointed or nearly so and dorsally wide.

**Mesosoma** (Fig. 175A, E, F, I). Mesosoma dorsoventrally convex. Mesoscutum proximally convex and distally flat, punctation distinct proximally with polished area distally, interspaces wavy/lacunose. Scutellum triangular, apex sloped and fused with BS, scutellar punctation distinct throughout, in profile scutellum flat and on same plane as mesoscutum, phragma of the scutellum partially exposed; BS mostly overlapping the MPM; ATS demilune with quite a little, complete and parallel carinae; dorsal ATS groove with semicircular/parallel carinae. Transscutal articulation with small and heterogeneous foveae, area just behind transscutal articulation with a smooth and shiny sloped transverse strip. Metanotum with BM wider than PFM (clearly differentiated); MPM semicircular without median longitudinal carina; AFM with a small lobe and not as well delineated as PFM; PFM thick, smooth and with a distal flat flange; ATM proximally with a groove with some sculpturing and distally with rugae. Propodeum with a median longitudinal dent, but no trace of median longitudinal carina, proximal half curved with rather coarse sculpture and distal half rugose; distal edge of propodeum with a flange at each side and without stubs; propodeal spiracle distally framed by a short concave carina; nucha surrounded by very short radiating carinae. Pronotum with a distinct dorsal furrow, dorsally with a well-defined smooth band; central area of pronotum smooth, but both dorsal and ventral furrows with short parallel carinae. Propleuron with a mix of rugae and fine punctation, dorsally with a carina. Metasternum flat or nearly so. Contour of mesopleuron convex; precoxal groove deep with transverse lineate sculpture; epicnemial ridge elongated more fusiform (tapering at both ends).

**Legs.** Ventral margin of fore telotarsus entire without seta, fore telotarsus almost same width throughout. Hind coxa with punctation only on ventral surface, dorsal outer depression present. Inner spur of hind tibia longer than outer spur (0.41, 0.15), entire surface of hind tibia with dense strong spines clearly differentiated by color and length.

**Wings** (Fig. 175K, L). Fore wing with r vein curved; 2RS vein straight; r and 2RS veins forming a weak, even curve at their junction and outer side of junction not forming a stub; 2M vein slightly curved/swollen; distally fore wing [where spectral veins are] with microtrichiae more densely concentrated than the rest of the wing; subbasal cell with a small smooth area; veins 2CUa and 2CUb completely spectral; vein 2 cu-a present as spectral vein, sometimes difficult to see; vein 2-1A proximally tubular and distally spectral, although sometimes difficult to see; tubular vein 1 cu-a straight, incomplete/broken and not reaching the edge of 1-1A vein. Hind wing with vannal lobe narrow, subdistally and subproximally straightened, and setae evenly scattered in the margin.

**Metasoma** (Fig. 175A, G, H, J). Metasoma laterally compressed. Petiole on T1 finely sculptured only laterally, virtually parallel-sided over most of length, but narrowing over distal 1/3 (length 0.45, maximum width 0.23, minimum width 0.12) and with scattered pubescence concentrated in the first distal third. Lateral grooves delimiting the median area on T2 clearly defined and reaching the distal edge of T2 (length median area 0.20, length T2 0.20), edges of median area polished and lateral grooves deep, median area broader than long (length 0.20, maximum width 0.24, minimum width 0.10); T2 with scattered pubescence only distally. T3 longer than T2 (0.28, 0.20) and with scattered pubescence throughout. Pubescence on hypopygium dense.

**Cocoons**. Unknown.

#### Comments.

Specimen with just one fore and one hind leg although the tarsomeres are missing.

#### Male

(Fig. 176A–I). The coloration of antennal flagellomeres is evenly throughout; the entire propleuron is reddish/yellow-brown with some areas darker than others; the mesopleuron with a more distinctive ventral reddish/yellow-brown band; the gena, the clypeus, the mesosternum, the ATS, the dorsal ATS groove and the lunules with reddish/yellow-brown tints; the median area on T2 is yellow-brown with contours darkened and the T3 is completely yellow.

#### Etymology.

Nina Michelle Zitani has worked in the systematics and biology of *Meteorus* (Braconidae: Meteorinae). She works at the University of Western Ontario, Canada.

#### Distribution.

The adult parasitoids were collected in Costa Rica, ACG, Sector El Hacha (Sendero Bejuquilla), Sector San Cristóbal (Río Blanco Abajo) and Sector Santa Rosa (Bosque Humedo), during July 1999, January-February 2000, and April 2008 at 280 m, 290 m, and 500 m in dry, dry-rain intergrade, and rain forests.

#### Biology.

Unknown.

#### Host.

Unknown.

### Glyptapanteles pachopinasi

Taxon classificationAnimaliaHymenopteraBraconidae

Arias-Penna, sp. nov.

http://zoobank.org/4277B423-86B7-4877-82C8-DDA64E9D7598

Fig. 177

#### Male.

Body length 3.58 mm, antenna length 4.45 mm, fore wing length 3.53 mm.

#### Type material.

**Holotype**: ECUADOR • 1♀; EC-12001, YY-A202; Napo, Yanayacu Biological Station, Río Arenillas, Plot 174; cloud forest; 1,973 m; -0.566667, -77.866667; 06.ii.2006; María de los Angeles Simbaña leg.; caterpillar collected in third instar; cocoon formed on 28.ii.2006; adult parasitoid emerged on 21.iii.2006; (PUCE).

#### Diagnosis.

**Figure 177.**
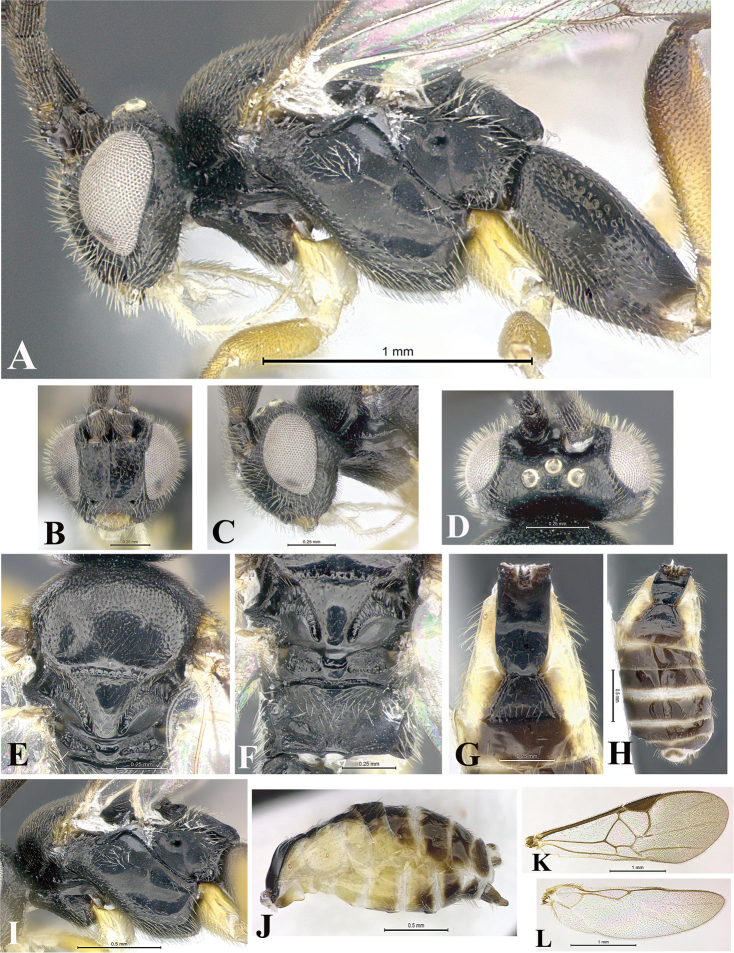
*Glyptapantelespachopinasi* sp. nov. male EC-12001 YY-A202 **A** Habitus **B–D** Head **B** Frontal view **C** Lateral view **D** Dorsal view **E** Mesonotum, dorsal view **F** Scutellum, metanotum, propodeum, dorsal view **G**T1–2, dorsal view **H, J** Metasoma **H** Dorsal view **J** Lateral view **I** Mesosoma, lateral view **K** Fore and hind wings.

Medioanterior pit of metanotum semicircular without median longitudinal carina (Fig. 177E, F), vertex in dorsal view narrow (Fig. 177D), scutellar punctation scattered throughout (Fig. 177E, F), dorsal furrow of pronotum with a well-defined smooth band (Fig. 177A, C, I), dorsal carina delimiting a dorsal furrow on propleuron present (Fig. 157C), anterior furrow of metanotum without setiferous lobes (Fig. 177E, F), axillary trough of scutellum with sculpture (Fig. 177E, F), lateral grooves delimiting the median area on T2 clearly defined and reaching the distal edge of T2, edges of median area polished and followed by a deep groove (Fig. 177G, H), propodeum without median longitudinal carina (Fig. 177F), anteroventral contour of mesopleuron convex (Fig. 177A, I), and fore wing with r vein curved, outer side of junction of r and 2RS veins forming a slight stub (Fig. 177K).

#### Coloration

(Fig. 177A–L). General body coloration satin black except apex labrum and maxillary yellow-brown; clypeus brown/reddish; glossa, maxillary and labial palps, and tegulae light brown; pedicel with apex yellow-reddish; scape and all antennal flagellomeres (on both sides) dark brown. Eyes and ocelli silver. Fore and middle legs dark yellow except claws brown; hind legs yellow-brown except coxae black distally brown/reddish, femora dorso-distal brown, tibiae brown, and tarsomeres brown. Petiole on T1 with two colorations: proximal half brown-reddish and distal half black, contours darkened mainly in proximal half, and sublateral areas yellow; T2 with median area brown, adjacent area very narrow, thus contours of median area looks darker, and lateral ends yellow; T3 with a brown area, proximally that dark area coinciding with width of median area of T2 and distally almost reaching the edge of T3; T4 and beyond completely brown; distally each tergum with a narrow yellow transparent band. In lateral view, T1–3 completely yellow; T4–5 yellow, but dorsally brown; T5 and beyond completely brown. S1–3 yellow; S4–5 yellow and medially with a brown area; remaining sterna brown.

#### Description.

**Head** (Fig. 177A–D). Head rounded with pubescence long and dense. Proximal three antennal flagellomeres longer than wide (0.29:0.09, 0.30:0.09, 0.29:0.09), distal antennal flagellomere longer than penultimate (0.18:0.08, 0.15:0.08), antenna longer than body (4.45, 3.58); antennal scrobes-frons sloped and forming a shelf. Face flat or nearly so, punctate-lacunose, interspaces wavy and longitudinal median carina present. Frons smooth. Temple wide, punctate-lacunose and interspaces wavy. Inner margin of eyes diverging slightly at antennal sockets; in lateral view, eye anteriorly convex and posteriorly straight. POL shorter than OOL (0.12, 0.14). Malar suture present. Median area between lateral ocelli slightly depressed. Vertex laterally pointed or nearly so and dorsally narrow.

**Mesosoma** (Fig. 177A, E, F, I). Mesosoma dorsoventrally convex. Mesoscutum with narrow grooves laterally, punctation distinct proximally with polished area distally, interspaces smooth. Scutellum triangular, apex sloped and fused with BS, but not in the same plane, scutellar punctation scattered throughout, in profile scutellum flat and on same plane as mesoscutum, phragma of the scutellum completely concealed; BS mostly overlapping the MPM; ATS demilune inner side with a row of foveae; dorsal ATS groove with carinae only proximally. Transscutal articulation with small and homogeneous foveae, area just behind transscutal articulation smooth, shiny and sloped. Metanotum with BM convex; MPM semicircular without median longitudinal carina; AFM without setiferous lobes and not as well delineated as PFM; PFM thick, smooth and with lateral ends rounded; ATM proximally with a groove with some sculpturing and distally smooth. Propodeum without median longitudinal carina, proximal half curved with medium-sized sculpture and distal half relatively polished; distal edge of propodeum with a flange at each side and without stubs; propodeal spiracle distally framed by a short concave carina; nucha surrounded by very short radiating carinae. Pronotum with a distinct dorsal furrow, dorsally with a well-defined smooth band; central area of pronotum and dorsal furrow smooth, but ventral furrow with short parallel carinae. Propleuron with fine punctations throughout and dorsally with a carina. Metasternum convex. Contour of mesopleuron convex; precoxal groove smooth, shiny and shallow, but visible; epicnemial ridge elongated more fusiform (tapering at both ends).

**Legs.** Ventral margin of fore telotarsus entire without seta, fore telotarsus almost same width throughout and longer than fourth tarsomere (0.15, 0.08). Half dorsal hind coxa with scattered punctation and half ventral with dense punctation, dorsal outer depression present. Inner spur of hind tibia longer than outer spur (0.44, 0.35), entire surface of hind tibia with dense strong spines clearly differentiated by color and length. Hind telotarsus longer than fourth tarsomere (0.20, 0.16).

**Wings** (Fig. 177K, L). Fore wing with r vein slightly curved; 2RS vein straight; r and 2RS veins forming a weak, even curve at their junction and outer side of junction forming a slight stub; 2M vein slightly curved/swollen; distally fore wing [where spectral veins are] with microtrichiae more densely concentrated than the rest of the wing; anal cell 1/3 proximally lacking microtrichiae; subbasal cell proximal half smooth; veins 2CUa and 2CUb completely spectral; vein 2 cu-a present as spectral vein, sometimes difficult to see; vein 2-1A tubular throughout; tubular vein 1 cu-a straight and complete, but junction with 1-1A vein spectral. Hind wing with vannal lobe, subdistally and subproximally straightened, and setae evenly scattered in the margin.

**Metasoma** (Fig. 177G, H, J). Metasoma cylindrical. Petiole on T1 finely sculptured distally, but only laterally, virtually parallel-sided over most of length, but narrowing over distal 1/3 (length 0.52, maximum width 0.23, minimum width 0.15), and with scattered pubescence concentrated in the first distal third. Lateral grooves delimiting the median area on T2 clearly defined and reaching the distal edge of T2 (length median area 0.17, length T2 0.17), edges of median area polished and lateral grooves deep, median area broader than long (length 0.17, maximum width 0.28, minimum width 0.12); T2 with scattered pubescence only distally. T3 longer than T2 (0.22, 0.17) and with scattered pubescence only distally.

**Cocoons.** Unknown.

#### Comments.

The ocelli are very close to each other (Fig. 177D, the median ocellus diameter is 0.07 mm, the distance between the median ocellus and the posterior ocellus is 0.03 mm), the limit between the mesopleuron and the metasternum has a dent, and the hind coxa is stout (Fig. 177A).

#### Female.

Unknown.

#### Etymology.

Francisco (Pacho) Piñas Rubio is an Ecuadorian lepidopterologist who has dedicated his research to documenting the diversity of this insect group in Ecuador.

#### Distribution.

Parasitized caterpillar was collected in Ecuador, Napo, Yanayacu Biological Station (Río Arenillas), during February 2006 at 1,973 m in cloud forest.

#### Biology.

The lifestyle of this parasitoid species is solitary.

#### Host.

Undetermined species of Noctuidae feeding on *Acalypha* sp. (Euphorbiaceae). Caterpillar was collected in third instar.

### Glyptapanteles pamitchellae

Taxon classificationAnimaliaHymenopteraBraconidae

Arias-Penna, sp. nov.

http://zoobank.org/A944FFAA-694E-433A-A5CF-BFACB08EFD08

Figs 178 , 179

#### Female.

Body length 2.17 mm, antenna length 2.58 mm, fore wing length 2.17 mm.

#### Type material.

**Holotype**: COSTA RICA •1♀; 00-SRNP-24057, DHJPAR0024693; Área de Conservación Guanacaste, Guanacaste, Sector Santa Rosa, Bosque Humedo; dry forest; Malaise; 290 m; 10.85145, -85.60801; 08.v.2000; DH Janzen & W Hallwachs leg.; (CNC). **Paratypes.** • 1 (0♀, 0♂) (0♀, 1♂); 00-SRNP-24012, DHJPAR0013610; same data as for holotype except: 01.v.2000; (CNC). • 1 (0♀, 0♂) (1♀, 0♂); 00-SRNP-24013, DHJPAR0013361; same data as for holotype except: 17.v.2000; (CNC). • 1 (0♀, 0♂) (1♀, 0♂); 00-SRNP-24016, DHJPAR0013362; same data as for holotype except: 15.v.2000; (CNC). • 1 (0♀, 0♂) (1♀, 0♂); 00-SRNP-24017, DHJPAR0013358; same data as for holotype except: 15.v.2000; (CNC).

#### Other material.

**Malaise-trapped material.** COSTA RICA: *Área de Conservación Guanacaste*, *Guanacaste*, *Sector El Hacha*, *Sendero Bejuquilla*: • 1 (1♀, 0♂) (0♀, 0♂); 98-SRNP-16034, DHJPAR0012621; dry-rain intergrade forest; Malaise; 280 m; 11.03004, -85.52699; 07.ix.1998; DH Janzen & W Hallwachs leg. • 1 (0♀, 0♂) (1♀, 0♂); 99-SRNP-18932, DHJPAR0012626; same data as for preceding except: 10.v.1999. • 1 (0♀, 1♂) (0♀, 0♂); 99-SRNP-19262, DHJPAR0013649; same data as for preceding except: 08.ii.1999. • 1 (0♀, 0♂) (1♀, 0♂); 99-SRNP-19266, DHJPAR0013653; same data as for preceding except: 01.ii.1999.

*Área de Conservación Guanacaste*, *Guanacaste*, *Sector Santa Rosa*, *Estación San Gerardo*: • 1 (1♀, 0♂) (0♀, 0♂); 08-SRNP-2835, DHJPAR0026279; Malaise; rain forest; 575 m; 10.88009, -85.38887; 15.iv.2008; DH Janzen & W Hallwachs leg.

*Área de Conservación Guanacaste*, *Guanacaste*, *Sector Santa Rosa*, *Río Blanco Abajo*: • 1 (0♀, 1♂) (0♀, 0♂); 08-SRNP-3240, DHJPAR0026821; Malaise; rain forest; 500 m; 10.90037, -85.37254; 30.iii.2008; DH Janzen & W Hallwachs leg.

*Área de Conservación Guanacaste*, *Guanacaste*, *Sector Santa Rosa*, *Bosque Humedo*: • 1 (0♀, 0♂) (0♀, 1♂); 98-SRNP-16107, DHJPAR0013570; dry forest; Malaise; 290 m; 10.85145, -85.60801; 09.iii.1998; DH Janzen & W Hallwachs leg. • 1 (0♀, 0♂) (0♀, 1♂); 98-SRNP-16108, DHJPAR0013349; same data as for preceding. • 1 (0♀, 0♂) (0♀, 1♂); 98-SRNP-16110, DHJPAR0013571; same data as for preceding except: 23.iii.1998. • 1 (0♀, 0♂) (0♀, 1♂); 98-SRNP-16111, DHJPAR0013572; same data as for preceding. • 1 (0♀, 1♂) (0♀, 0♂); 98-SRNP-16114, DHJPAR0013573; same data as for preceding • 1 (0♀, 0♂) (0♀, 1♂); 98-SRNP-16116, DHJPAR0013574; same data as for preceding except: 23.ii.1998. • 1 (0♀, 1♂) (0♀, 0♂); 98-SRNP-16118, DHJPAR0013348; same data as for preceding except: 16.ii.1998. • 1 (0♀, 0♂) (0♀, 1♂); 98-SRNP-16119, DHJPAR0013575; same data as for preceding. • 1 (1♀, 0♂) (0♀, 0♂); 98-SRNP-16121, DHJPAR0013375; same data as for preceding except: 16.iii.1998. • 1 (0♀, 0♂) (0♀, 1♂); 98-SRNP-16122, DHJPAR0013576; same data as for preceding except: 23.ii.1998. • 1 (0♀, 0♂) (0♀, 1♂); 98-SRNP-16123, DHJPAR0013577; same data as for preceding except: 23.ii.1998. • 1 (0♀, 0♂) (0♀, 1♂); 98-SRNP-16126, DHJPAR0013578; same data as for preceding except: 23.ii.1998. • 1 (1♀, 0♂) (0♀, 0♂); 98-SRNP-16128, DHJPAR0013369; same data as for preceding except: 12.i.1998. • 1 (0♀, 0♂) (0♀, 1♂); 98-SRNP-16176, DHJPAR0024666; same data as for preceding except: 26.i.1998. • 1 (0♀, 0♂) (0♀, 1♂); 98-SRNP-16180, DHJPAR0024670; same data as for preceding except: 09.ii.1998. • 1 (0♀, 0♂) (0♀, 1♂); 98-SRNP-16182, DHJPAR0024672; same data as for preceding except: 09.ii.1998. • 1 (0♀, 0♂) (0♀, 1♂); 98-SRNP-16188, DHJPAR0024678; same data as for preceding except: 09.ii.1998. • 1 (0♀, 0♂) (1♀, 0♂); 98-SRNP-16190, DHJPAR0024680; same data as for preceding except: 09.ii.1998. • 1 (0♀, 0♂) (0♀, 1♂); 99-SRNP-19077, DHJPAR0013580; same data as for preceding except: 03.v.1999. • 1 (0♀, 0♂) (0♀, 1♂); 99-SRNP-19084, DHJPAR0013582; same data as for preceding except: 24.v.1999. • 1 (0♀, 0♂) (0♀, 1♂); 99-SRNP-19087, DHJPAR0013584; same data as for preceding except: 03.v.1999. • 1 (0♀, 0♂) (0♀, 1♂); 99-SRNP-19088, DHJPAR0013585; same data as for preceding except: 03.v.1999. • 1 (0♀, 0♂) (0♀, 1♂); 99-SRNP-19092, DHJPAR0013586; same data as for preceding except: 24.v.1999. • 1 (0♀, 0♂) (0♀, 1♂); 99-SRNP-19097, DHJPAR0013588; same data as for preceding except: 24.v.1999. • 1 (0♀, 0♂) (0♀, 1♂); 99-SRNP-19098, DHJPAR0013589; same data as for preceding except: 10.v.1999. • 1 (0♀, 1♂) (0♀, 0♂); 99-SRNP-19099, DHJPAR0013590; same data as for preceding except: 24.v.1999. • 1 (0♀, 0♂) (1♀, 0♂); 99-SRNP-19101, DHJPAR0013368; same data as for preceding except: 26.iv.1999. • 1 (0♀, 0♂) (0♀, 1♂); 99-SRNP-19107, DHJPAR0013592; same data as for preceding except: 03.v.1999. • 1 (0♀, 0♂) (0♀, 1♂); 99-SRNP-19272, DHJPAR0024686; same data as for preceding except: 17.v.1999. • 1 (0♀, 0♂) (0♀, 1♂); 00-SRNP-23953, DHJPAR0013593; same data as for preceding except: 29.v.2000. • 1 (0♀, 0♂) (0♀, 1♂); 00-SRNP-23954, DHJPAR0013594; same data as for preceding except: 10.iv.2000. • 1 (0♀, 0♂) (0♀, 1♂); 00-SRNP-23956, DHJPAR0013595; same data as for preceding except: 28.ii.2000. • 1 (0♀, 0♂) (0♀, 1♂); 00-SRNP-23958, DHJPAR0013597; same data as for preceding except: 29.v.2000. • 1 (0♀, 0♂) (0♀, 1♂); 00-SRNP-23959, DHJPAR0013598; same data as for preceding except: 29.v.2000. • 1 (1♀, 0♂) (0♀, 0♂); 00-SRNP-23960, DHJPAR0013370; same data as for preceding except: 10.iv.2000. • 1 (0♀, 0♂) (1♀, 0♂); 00-SRNP-23966, DHJPAR0013372; same data as for preceding except: 31.i.2000. • 1 (0♀, 0♂) (0♀, 1♂); 00-SRNP-23970, DHJPAR0013601; same data as for preceding except: 21.ii.2000. • 1 (0♀, 0♂) (0♀, 1♂); 00-SRNP-23971, DHJPAR0013602; same data as for preceding except: 24.iv.2000. • 1 (0♀, 0♂) (0♀, 1♂); 00-SRNP-23978, DHJPAR0013604; same data as for preceding except: 10.iv.2000. • 1 (0♀, 0♂) (1♀, 0♂); 00-SRNP-23981, DHJPAR0013365; same data as for preceding except: 21.ii.2000. • 1 (0♀, 0♂) (0♀, 1♂); 00-SRNP-23986, DHJPAR0013606; same data as for preceding except: 21.ii.2000. • 1 (0♀, 0♂) (1♀, 0♂); 00-SRNP-23987, DHJPAR0013367; same data as for preceding except: 07.ii.2000. • 1 (0♀, 0♂) (0♀, 1♂); 00-SRNP-23992, DHJPAR0013415; same data as for preceding except: 07.ii.2000. • 1 (0♀, 0♂) (0♀, 1♂); 00-SRNP-23995, DHJPAR0013413; same data as for preceding except: 28.ii.2000. • 1 (0♀, 1♂) (0♀, 0♂); 00-SRNP-23996, DHJPAR0013417; same data as for preceding except: 24.i.2000. • 1 (0♀, 0♂) (0♀, 1♂); 00-SRNP-24010, DHJPAR0013418; same data as for preceding except: 27.iii.2000. • 1 (0♀, 0♂) (0♀, 1♂); 00-SRNP-24018, DHJPAR0013412; same data as for preceding except: 05.vi.2000. • 1 (0♀, 0♂) (1♀, 0♂); 00-SRNP-24022, DHJPAR0013360; same data as for preceding except: 06.iii.2000. • 1 (0♀, 0♂) (0♀, 1♂); 00-SRNP-24023, DHJPAR0013611; same data as for preceding except: 17.iv.2000. • 1 (0♀, 0♂) (0♀, 1♂); 00-SRNP-24024, DHJPAR0013612; same data as for preceding except: 17.iv.2000. • 1 (0♀, 0♂) (0♀, 1♂); 00-SRNP-24027, DHJPAR0013406; same data as for preceding except: 21.ii.2000. • 1 (0♀, 0♂) (0♀, 1♂); 00-SRNP-24028, DHJPAR0013613; same data as for preceding except: 21.ii.2000. • 1 (0♀, 0♂) (0♀, 1♂); 00-SRNP-24029, DHJPAR0013409; same data as for preceding except: 06.iii.2000. • 1 (0♀, 0♂) (0♀, 1♂); 00-SRNP-24030, DHJPAR0013410; same data as for preceding except: 06.iii.2000. • 1 (1♀, 0♂) (0♀, 0♂); 00-SRNP-24031, DHJPAR0013353; same data as for preceding except: 28.ii.2000. • 1 (0♀, 0♂) (0♀, 1♂); 00-SRNP-24033, DHJPAR0013614; same data as for preceding except: 21.ii.2000. • 1 (1♀, 0♂) (0♀, 0♂); 00-SRNP-24034, DHJPAR0013366; same data as for preceding except: 21.ii.2000. • 1 (0♀, 0♂) (1♀, 0♂); 00-SRNP-24035, DHJPAR0013355; same data as for preceding except: 21.ii.2000. • 1 (0♀, 0♂) (1♀, 0♂); 00-SRNP-24038, DHJPAR0013356; same data as for preceding except: 28.ii.2000. • 1 (0♀, 0♂) (0♀, 1♂); 00-SRNP-24052, DHJPAR0024688; same data as for preceding except: 13.iii.2000. • 1 (0♀, 0♂) (0♀, 1♂); 00-SRNP-24053, DHJPAR0024689; same data as for preceding except: 13.iii.2000. • 1 (0♀, 0♂) (1♀, 0♂); 00-SRNP-24061, DHJPAR0024697; same data as for preceding except: 24.i.2000. • 1 (0♀, 0♂) (0♀, 1♂); 00-SRNP-24062, DHJPAR0024698; same data as for preceding except: 24.i.2000. • 1 (0♀, 0♂) (0♀, 1♂); 00-SRNP-24066, DHJPAR0024702; same data as for preceding except: 20.iii.2000. • 1 (1♀, 0♂) (0♀, 0♂); 00-SRNP-24067, DHJPAR0024703; same data as for preceding except: 02.i.2000. • 1 (0♀, 0♂) (0♀, 1♂); 07-SRNP-15055, DHJPAR0013615; same data as for preceding except: 05.vi.2007; AR Deans & J Rodriguez. • 1 (0♀, 0♂) (0♀, 1♂); 07-SRNP-15056, DHJPAR0013616; same data as for preceding except: 05.vi.2007; AR Deans & J Rodriguez leg. • 1 (0♀, 0♂) (1♀, 0♂); 07-SRNP-15059, DHJPAR0013354; same data as for preceding except: 05.vi.2007; AR Deans & J Rodriguez leg.

*Área de Conservación Guanacaste*, *Guanacaste*, *Sector Santa Rosa*, *Bosque San Emilio*: • 1 (1♀, 0♂) (0♀, 0♂); 98-SRNP-16066, DHJPAR0013391; dry forest; Malaise; 300 m; 10.84389, -85.61384; 26.x.1998; DH Janzen & W Hallwachs leg. • 1 (1♀, 0♂) (0♀, 0♂); 98-SRNP-16074, DHJPAR0013388; same data as for preceding except: 14.ix.1998. • 1 (0♀, 1♂) (0♀, 0♂); 98-SRNP-16078, DHJPAR0013530; same data as for preceding except: 31.viii.1998. • 1 (0♀, 0♂) (0♀, 1♂); 98-SRNP-16080, DHJPAR0013531; same data as for preceding except: 24.viii.1998. • 1 (0♀, 0♂) (1♀, 0♂); 98-SRNP-16082, DHJPAR0013532; same data as for preceding except: 31.viii.1998. • 1 (0♀, 0♂) (1♀, 0♂); 98-SRNP-16083, DHJPAR0013389; same data as for preceding except: 14.ix.1998. • 1 (0♀, 0♂) (0♀, 1♂); 98-SRNP-16091, DHJPAR0013533; same data as for preceding except: 28.ix.1998. • 1 (0♀, 0♂) (0♀, 1♂); 98-SRNP-16096, DHJPAR0013534; same data as for preceding except: 07.ix.1998. • 1 (0♀, 0♂) (0♀, 1♂); 98-SRNP-16102, DHJPAR0013535; same data as for preceding except: 31.viii.1998. • 1 (0♀, 0♂) (0♀, 1♂); 99-SRNP-18971, DHJPAR0013538; same data as for preceding except: 08.ii.1999. • 1 (0♀, 0♂) (1♀, 0♂); 99-SRNP-18973, DHJPAR0013539; same data as for preceding except: 11.i.1999. • 1 (0♀, 1♂) (0♀, 0♂); 99-SRNP-19001, DHJPAR0013382; same data as for preceding except: 19.x.1999. • 1 (0♀, 0♂) (0♀, 1♂); 99-SRNP-19047, DHJPAR0013548; same data as for preceding except: 18.i.1999. • 1 (0♀, 0♂) (0♀, 1♂); 99-SRNP-19068, DHJPAR0013553; same data as for preceding except: 22.iii.1999. • 1 (0♀, 0♂) (0♀, 1♂); 07-SRNP-15070, DHJPAR0013662; same data as for preceding except: 01.v.2007.

#### Diagnosis.

**Figure 178.**
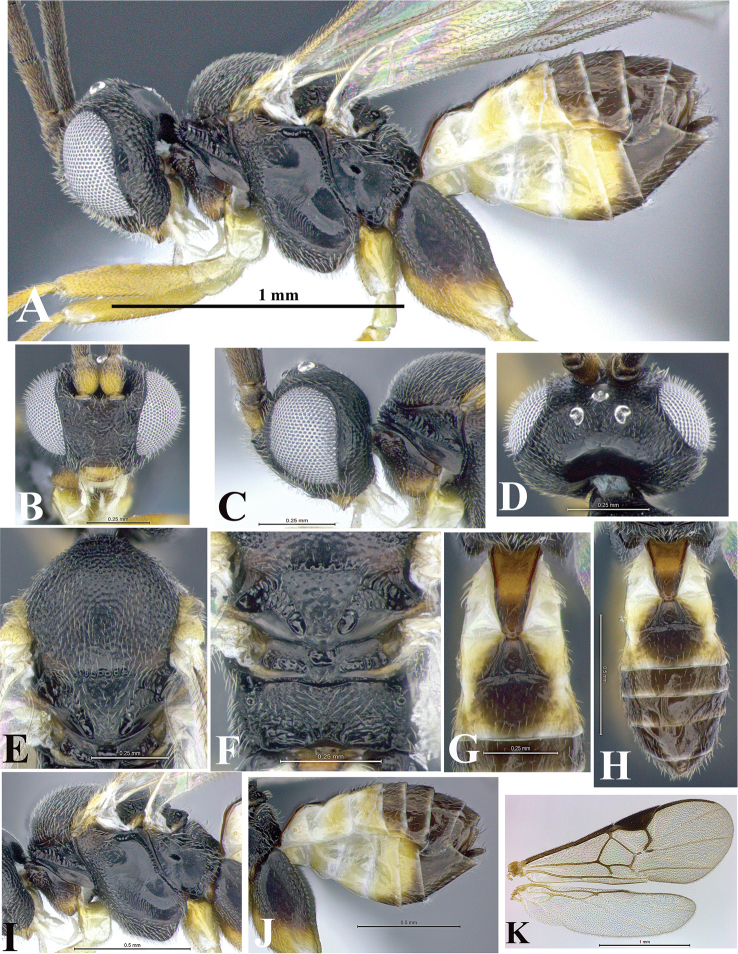
*Glyptapantelespamitchellae* sp. nov. female 00-SRNP-24057 DHJPAR0024693, 08-SRNP-2835 DHJPAR0026279 **A** Habitus **B, D** Head **B** Frontal view **D** Dorsal view **C** Head, pronotum, propleuron, lateral view **E** Mesonotum, dorsal view **F** Scutellum, metanotum, propodeum, dorsal view **G**T1–3, dorsal view **H, J** Metasoma **H** Dorsal view **J** Lateral view **I** Mesosoma, lateral view **K** Fore and hind wings.

**Figure 179.**
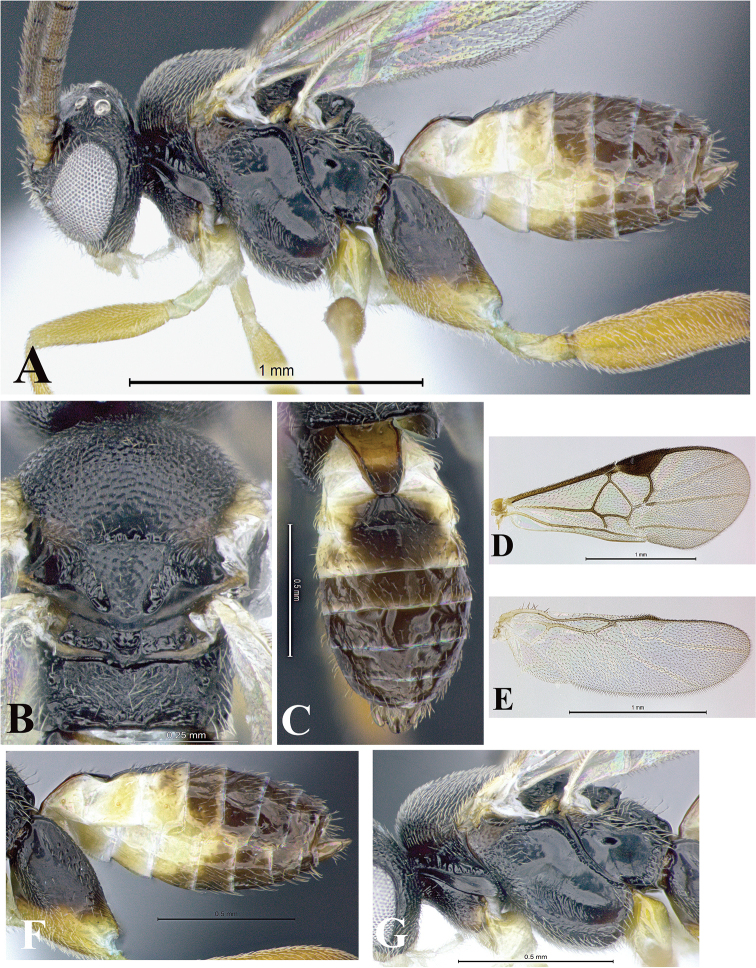
*Glyptapantelespamitchellae* sp. nov. male 00-SRNP-23996 DHJPAR0013417, 00-SRNP-24052 DHJPAR0024688 **A** Habitus **B** Mesosoma, dorsal view **C, F** Metasoma **C** Dorsal view **F** Lateral view **D, E** Wings **D** Fore **E** Hind **G** Mesosoma, lateral view.

Ventral margin of fore telotarsus entire without seta, medioanterior pit of metanotum circular without median longitudinal carina (Figs 178F, 179B), propleuron with fine rugae, dorsal carina delimiting a dorsal furrow present (Figs 178A, I, 179A, G), antenna longer than body, anterior furrow of metanotum with a small lobe, without setae (Figs 178F, 179B), distal antennal flagellomere longer than penultimate, surface of metasternum convex, precoxal groove deep with lineate sculpture (Figs 178A, I, 179A, G), fore wing with vein 1 cu-a curved, r vein curved (Figs 178K, 179D), dorsal outer depression on hind coxa present (178A, J, 179A, F), inner margin of eyes diverging slightly at antennal sockets (Fig. 178B), petiole on T1 finely sculptured only distally (Figs 178G, H, 179C), and lateral grooves delimiting the median area on T2 clearly defined and reaching the distal edge of T2 (Figs 178G, H, 179C).

#### Coloration

(Fig. 178A–K). General body coloration black except scape yellow with lateral brown band; all antennal flagellomeres dark brown on both sides; labrum, mandible, and tegulae dark yellow; maxillary and labial palps ivory/pale yellow; clypeus, both ends of propleuron, ventral furrow of pronotum, adjacent area of mesoscutum, epicnemial ridge, narrow band taking the place of notauli, distal corners of mesoscutum, lunules, lateral ends of metapleuron, and PFM with yellow-brown/reddish tints. Eyes and ocelli silver. Fore and middle legs dark yellow except coxae trochanters and trochantellus ivory/pale yellow, and claws brown; hind legs pale dark yellow except coxae 2/3 proximal black, femora distally with a tiny brown dot, tibiae distally brown, and tarsomeres brown. Petiole on T1 with two coloration: 3/4 proximally dark yellow-brown, 1/4 distal brown, contours yellow-brown/reddish, and sublateral areas ivory/pale yellow; T2 with median area brown, but proximally with a small yellow-brown/reddish spot, narrow adjacent area yellow-brown, and lateral ends ivory/pale yellow; T3 broadly with a oval-shaped area, proximally width of that dark area coinciding with the width of median and adjacent areas on T2, and lateral ends proximal half of ivory/pale yellow and distal half yellow-brown; T4 and beyond completely brown; distally each tergum with a narrow yellowish transparent band. In lateral view, T1–3 ivory/pale yellow; T4 and beyond brown. S1–3 ivory/pale yellow; S4–5 yellow, but medially brown; hypopygium completely brown.

#### Description.

**Head** (Fig. 178A–D). Head rectangular with pubescence long and dense. Proximal three antennal flagellomeres longer than wide (0.19:0.06, 0.19:0.06, 0.19:0.06), distal antennal flagellomere longer than penultimate (0.11:0.06, 0.09:0.06), antenna longer than body (2.58, 2.17); antennal scrobes-frons shallow. Face flat or nearly so, punctate-lacunose, interspaces wavy and longitudinal median carina present. Frons smooth. Temple wide, punctate-lacunose and interspaces wavy. Inner margin of eyes diverging slightly at antennal sockets; in lateral view, eye anteriorly convex and posteriorly straight. POL subequal in length with OOL (0.09, 0.10). Malar suture present. Median area between lateral ocelli slightly depressed. Vertex laterally pointed or nearly so and dorsally wide.

**Mesosoma** (Fig. 178A, E, F, I). Mesosoma dorsoventrally convex. Mesoscutum proximally convex and distally flat, punctation distinct proximally with polished area distally, interspaces wavy/lacunose. Scutellum triangular, apex sloped and fused with BS, scutellar punctation distinct throughout, in profile scutellum slightly convex, but on same plane as mesoscutum, phragma of the scutellum partially exposed; BS only very partially overlapping the MPM; ATS demilune with quite a little, complete and parallel carinae; dorsal ATS groove with semicircular/parallel carinae. Transscutal articulation with small and heterogeneous foveae, area just behind transscutal articulation smooth, shiny and depressed centrally. Metanotum with BM wider than PFM (clearly differentiated); MPM circular without median longitudinal carina; AFM with a small lobe and not as well delineated as PFM; PFM thick, smooth and with lateral ends rounded; ATM proximally with a groove with some sculpturing and distally smooth. Propodeum with transverse rugae, proximal half curved with medium-sized sculpture and distal half rugose; distal edge of propodeum with a flange at each side and without stubs; propodeal spiracle distally framed by a short concave carina; nucha surrounded by very short radiating carinae. Pronotum with a distinct dorsal furrow, dorsally with a well-defined smooth band; central area of pronotum smooth, but both dorsal and ventral furrows with short parallel carinae. Propleuron with fine rugae and dorsally with a carina. Metasternum convex. Contour of mesopleuron convex; precoxal groove deep transverse lineate sculpture; epicnemial ridge elongated more fusiform (tapering at both ends).

**Legs.** Ventral margin of fore telotarsus entire without seta, fore telotarsus almost same width throughout and longer than fourth tarsomere (0.10, 0.05). Hind coxa medially smooth, dorsally with scattered punctation and ventrally with dense punctation, dorsal outer depression present. Inner spur of hind tibia longer than outer spur (0.23, 0.18), entire surface of hind tibia with dense strong spines clearly differentiated by color and length. Hind telotarsus as equal in length as fourth tarsomere (0.11, 0.10).

**Wings** (Fig. 178K). Fore wing with r vein slightly curved; 2RS vein straight; r and 2RS veins forming a weak, even curve at their junction and outer side of junction not forming a stub; 2M vein slightly curved/swollen; distally fore wing [where spectral veins are] with microtrichiae more densely concentrated than the rest of the wing; anal cell 1/3 proximally lacking microtrichiae; subbasal cell with a small smooth area; veins 2CUa and 2CUb completely spectral; vein 2 cu-a present as spectral vein, sometimes difficult to see; vein 2-1A proximally tubular and distally spectral, although sometimes difficult to see; tubular vein 1 cu-a curved, incomplete/broken and not reaching the edge of 1-1A vein. Hind wing with vannal lobe very narrow, subdistally and subproximally straightened, and setae evenly scattered in the margin.

**Metasoma** (Fig. 178A, G, H, J). Metasoma laterally compressed. Petiole on T1 finely sculptured only distally, petiole evenly narrowing distally (length 0.30, maximum width 0.15, minimum width 0.06) and with scattered pubescence concentrated in the first distal third. Lateral grooves delimiting the median area on T2 clearly defined and reaching the distal edge of T2 (length median area 0.11, length T2 0.11), edges of median area polished and lateral grooves deep, median area broader than long (length 0.11, maximum width 0.22, minimum width 0.05); T2 with scattered pubescence only distally. T3 longer than T2 (0.18, 0.11) and with scattered pubescence throughout. Pubescence on hypopygium dense.

**Cocoons.** Unknown.

#### Comments.

The inner side of ATS proximally with a large elongate fovea at each side (Fig. 178F).

#### Male

(Fig. 179A–G). The body shape and the coloration are similar to female.

#### Etymology.

Pamela A. Mitchell (RIP) was Ian Gauld’s wife who helped with many Hymenoptera identifications from Costa Rica. She was a real entomological partner with Ian Gauld, as well as sorting an enormous number of Malaise-trap samples from ACG and elsewhere.

#### Distribution.

The adult parasitoids were collected in Costa Rica, ACG, Sector El Hacha (Sendero Bejuquilla) and Sector Santa Rosa (Bosque Humedo, Bosque San Emilio, Estación San Gerardo, and Río Blanco Abajo), during January–March and August–October 1988, January–May and October 1999, January–June 2000, May–June 2007, and Mach–April 2008 at 290 m, 300 m, 500 m, and 575 m in dry, dry-rain intergrade, and rain forests.

#### Biology.

Unknown.

#### Host.

Unknown.

### Glyptapanteles paulhansoni

Taxon classificationAnimaliaHymenopteraBraconidae

Arias-Penna, sp. nov.

http://zoobank.org/1F90E310-6425-431F-AE45-653383BA3BD9

Figs 180 , 181

#### Female.

Body length 2.17 mm, antenna length 2.97 mm, fore wing length 2.47 mm.

#### Type material.

**Holotype**: COSTA RICA • 1♀; 05-SRNP-34533, DHJPAR0004769; Área de Conservación Guanacaste, Guanacaste, Sector Pitilla, Pasmompa; rain forest; 440 m; 11.01926, -85.40997; 25.x.2005; Calixto Moraga leg.; caterpillar collected in fifth instar; single row of brown coordwod cocoons lined up at right angles to the twig at the end, with the caterpillar on the twig defending, cocoons formed on 02.xi.2005 and adhered to the larval cuticle; adult parasitoids emerged on 12.xi.2005; (CNC). **Paratypes.** • 13 (3♀, 0♂) (10♀, 0♂); 05-SRNP-34533, DHJPAR0004769; same data as for holotype; (CNC).

#### Other material.

**Reared material.** COSTA RICA: *Área de Conservación Guanacaste*, *Guanacaste*, *Sector Pitilla*, *Pasmompa*: • 12 (4♀, 3♂) (5♀, 0♂); 05-SRNP-34905, DHJPAR0004779; rain forest; 440 m; 11.01926, -85.40997; 21.xi.2005; Calixto Moraga leg.; caterpillar collected in fifth instar; brown cocoons adhered on end to the midrib at strong angle, adhered to the leaf substrate and formed on 20.xi.2005; adult parasitoids emerged on 30.xi.2005.

*Área de Conservación Guanacaste*, *Guanacaste*, *Sector Pitilla*, *Sendero Montecele*: • 9 (4♀, 1♂) (4♀, 0♂); 10-SRNP-30507, DHJPAR0038960; rain forest; 680 m; 10.97337, -85.42088; 16.ii.2010; Manuel Rios leg.; caterpillar collected in fifth instar and still alive taking care of cocoons; cocoons adhered to the leaf substrate; adult parasitoids emerged on 21.ii.2010.

*Área de Conservación Guanacaste*, *Guanacaste*, *Sector Pitilla*, *Colocho*: • 17 (3♀, 1♂), (12♀, 1♂); 11-SRNP-31305, DHJPAR0042953; rain forest; 375 m; 11.02367, -85.41884; 07.v.2011; Calixto Moraga leg.; caterpillar collected in fourth instar; cocoons adhered to the larval cuticle and formed on 14.v.2011; adult parasitoids emerged on 22.v.2011.

*Área de Conservación Guanacaste*, *Alajuela*, *Sector Brasilia*, *Piedrona*: • 40 (4♀, 3♂) (22♀, 11♂); 07-SRNP-65627, DHJPAR0020470; rain forest; 340 m; 11.01618, -85.35902; 18.ix.2007; Duvalier Briceño leg.; caterpillar collected in fifth instar; brown cocoons adhered to the leaf substrate and formed on 20.ix.2007; adult parasitoids emerged on 27.ix.2007.

*Área de Conservación Guanacaste*, *Alajuela*, *Sector Brasilia*, *Brumas*: • 12 (3♀, 2♂) (7♀, 0♂); 11-SRNP-65650, DHJPAR0045135; rain forest; 360 m; 11.01825, -85.37199; 10.vii.2011; Minor Carmona leg.; caterpillar collected in fifth instar; cocoons in host cocoon and formed on 18.vii.2011; adult parasitoids emerged on 25.vii.2011.

#### Diagnosis.

**Figure 180.**
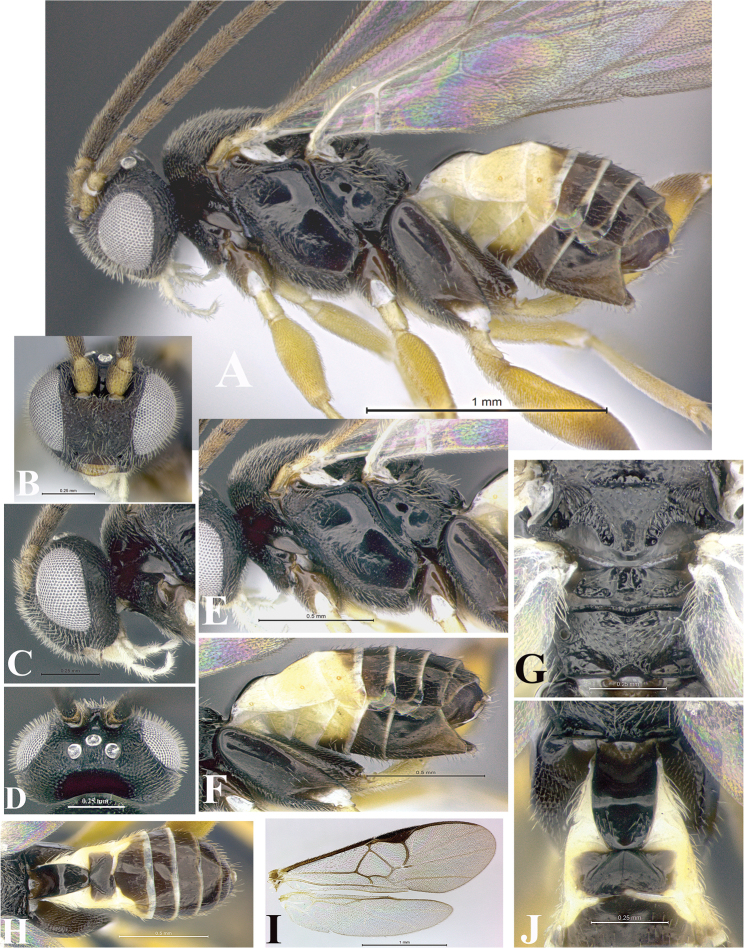
*Glyptapantelespaulhansoni* sp. nov. female 10-SRNP-30507 DHJPAR0038960 **A** Habitus **B, D** Head **B** Frontal view **D** Dorsal view **C** Head, pronotum, propleuron, lateral view **E** Mesosoma, lateral view **F, H** Metasoma **F** Lateral view **H** Dorsal view **G** Scutellum, metanotum, propodeum, dorsal view **I** Fore and hind wings **J**T1–3, dorsal view.

**Figure 181.**
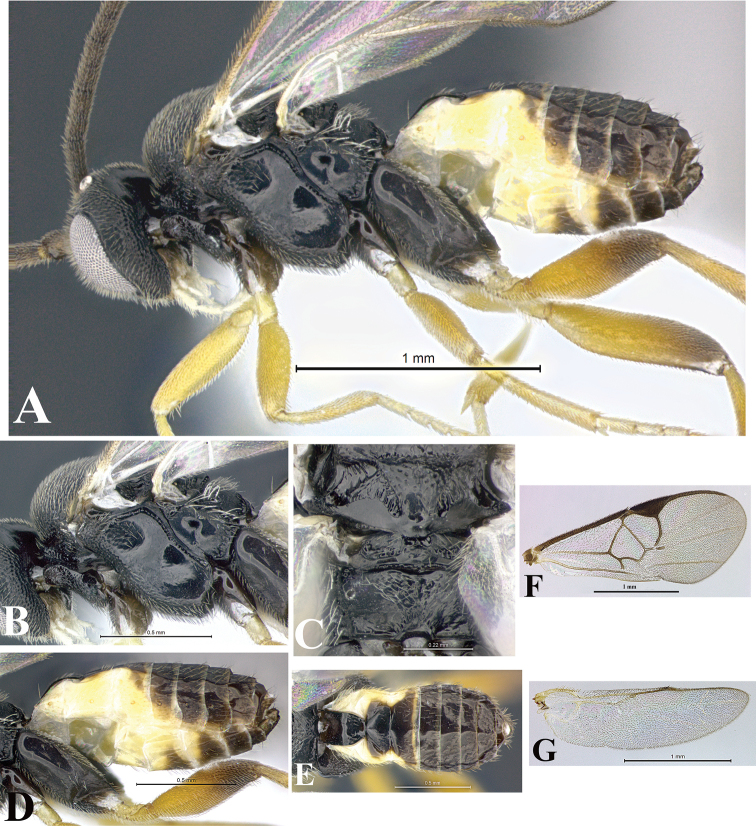
*Glyptapantelespaulhansoni* sp. nov. male 10-SRNP-30507 DHJPAR0038960 **A** Habitus **B** Mesosoma, lateral view **C** Scutellum, metanotum, propodeum, dorsal view **D, E** Metasoma **D** Lateral view **E** Dorsal view **F, G** Wings **F** Fore **G** Hind.

Medioanterior pit of metanotum circular and bisected by a median longitudinal carina (Figs 180G, 181C), anteroventral contour of mesopleuron convex (Figs 180A, E, 181A, B), petiole on T1 distally with lateral margins relatively straight (Figs 180H, J, 181E), propodeum without median longitudinal carina, propodeal spiracle without distal carina (Figs 180G, 181C), nucha surrounded by very short radiating carinae (Figs 180G, 181C), antenna longer than body, fore wing with 2RS vein straight, outer side of junction of r and 2RS veins not forming a stub (Figs 180I, 181F), and lateral grooves delimiting the median area on T2 distally losing definition (Figs 180H, J, 181E).

#### Coloration

(Fig. 180A–J). General body coloration satin black except scape and pedicel yellow-brown; all antennal flagellomeres dorsally lighter (light brown) than ventrally (dark brown); labrum, mandibles and tegulae yellow-brown; glossa, maxillary and labial palps ivory/pale yellow. Eyes and ocelli silver. Fore and middle legs yellow-brown except light brown coxae and claws brown; hind legs yellow-brown except brown coxae, femora distally brown, tibia 1/3 distal and tarsomeres brown, although proximally basitarsus with a small yellow band. Petiole on T1 dark brown and sublateral areas yellow; T2 with median and adjacent areas yellow-brown, both forming a rectangle-shaped area, and lateral ends yellow; T3 almost completely brown, proximally dark area coincides with width of dark median plus adjacent areas on T2, but distally dark area covering all the width of T3, thus only proximal half of lateral ends yellow, and distally T3 with an ivory/pale yellow band; T4 and beyond completely brown; distally each tergum with an ivory/pale yellow transparent band. In lateral view, T1–2 yellow; T3 yellow with a small brown area on distal corner; T4 and beyond brown. S1–3 yellow; S4 yellow, but medially brown; penultimate sternum and hypopygium brown.

#### Description.

**Head** (Fig. 180A–D). Head rounded with pubescence long and dense. Proximal three antennal flagellomeres longer than wide (0.20:0.06, 0.21:0.06, 0.19:0.06), distal antennal flagellomere longer than penultimate (0.12:0.04, 0.10:0.04), antenna longer than body (2.97, 2.17); antennal scrobes-frons sloped and forming a shelf. Face convex, punctate-lacunose, interspaces wavy and longitudinal median carina present. Frons smooth. Temple wide, punctate-lacunose and interspaces wavy. Inner margin of eyes diverging slightly at antennal sockets; in lateral view, eye anteriorly convex and posteriorly straight. POL short than OOL (0.09, 0.12). Malar suture present. Median area between lateral ocelli slightly depressed. Vertex laterally rounded and dorsally wide.

**Mesosoma** (Fig. 180A, E, G). Mesosoma dorsoventrally convex. Mesoscutum proximally convex and distally flat, punctation distinct throughout, interspaces wavy/lacunose. Scutellum triangular, apex sloped and fused with BS, scutellar punctation scattered throughout, in profile scutellum flat and on same plane as mesoscutum, phragma of the scutellum partially exposed; BS only very partially overlapping the MPM; ATS demilune with quite a little, complete and parallel carinae; dorsal ATS groove with semicircular/parallel carinae. Transscutal articulation with small and heterogeneous foveae, area just behind transscutal articulation smooth, shiny and depressed centrally. Metanotum with BM wider than PFM (clearly differentiated); MPM circular and bisected by a median longitudinal carina; AFM without setiferous lobes and not as well delineated as PFM; PFM thick, smooth and with lateral ends rounded; ATM proximally with a groove with some sculpturing and distally smooth. Propodeum without median longitudinal carina, proximal half curved with medium-sized sculpture and distal half slightly rugose; distal edge of propodeum with a flange at each side and without stubs; propodeal spiracle without distal carina; nucha surrounded by very short radiating carinae. Pronotum with a distinct dorsal furrow, dorsally with a well-defined smooth band; central area of pronotum smooth, but both dorsal and ventral furrows with short parallel carinae. Propleuron with a mix of rugae and fine punctation, dorsally with a carina. Metasternum flat or nearly so. Contour of mesopleuron convex; precoxal groove deep with transverse lineate sculpture; epicnemial ridge convex, teardrop-shaped.

**Legs.** Ventral margin of fore telotarsus entire without seta, fore telotarsus almost same width throughout and longer than fourth tarsomere (0.10, 0.07). Hind coxa with punctation only on ventral surface, dorsal outer depression present. Inner spur of hind tibia longer than outer spur (0.25, 0.16), entire surface of hind tibia with dense strong spines clearly differentiated by color and length. Hind telotarsus as equal in length as fourth tarsomere (0.11, 0.11).

**Wings** (Fig. 180I). Fore wing with r vein curved; 2RS vein straight; r and 2RS veins forming a weak, even curve at their junction and outer side of junction not forming a stub; 2M vein slightly curved/swollen; distally fore wing [where spectral veins are] with microtrichiae more densely concentrated than the rest of the wing; anal cell 1/3 proximally lacking microtrichiae; subbasal cell with microtrichiae virtually throughout; vein 2CUa absent and vein 2CUb spectral; vein 2 cu-a present as spectral vein, sometimes difficult to see; vein 2-1A proximally tubular and distally spectral, although sometimes difficult to see; tubular vein 1 cu-a curved, incomplete/broken and not reaching the edge of 1-1A vein. Hind wing with vannal lobe very narrow, subdistally and subproximally straightened, and setae evenly scattered in the margin.

**Metasoma** (Fig. 180A, F, H, J). Metasoma laterally compressed. Petiole on T1 finely sculptured only distally, virtually parallel-sided over most of length, but narrowing over distal 1/3 (length 0.35, maximum width 0.16, minimum width 0.10), and with scattered pubescence concentrated in the first distal third. Lateral grooves delimiting the median area on T2 distally losing definition (length median area 0.10, length T2 0.14), edges of median area polished and lateral grooves deep, median area broader than long (length 0.10, maximum width 0.27, minimum width 0.08); T2 with scattered pubescence only distally. T3 longer than T2 (0.18, 0.14) and with scattered pubescence throughout. Pubescence on hypopygium dense.

**Cocoons.** Beige or light brown oval cocoons with silk fibers messy/disordered/fluffy. Single row of coordwod cocoons adhered to the leaf substrate, to the larval cuticle or in host cocoon.

#### Comments.

In some females (e.g., 11-SRNP-6560), the petiole with three colors: proximally yellow, medially reddish/yellow-brown and distally brown, and contours of the whole petiole darkened.

#### Male

(Fig. 181A–G). As is the case with some females, the coloration on the petiole varies in some males too (e.g., 11-SRNP-6560, 05-SRNP-34905). Thus, the petiole has three colors: proximally yellow, medially reddish/yellow-brown and distally brown, all surrounded by brown and contours of petiole darkened. In other males, the petiole has two colors: proximal 3/4 are reddish/yellow-brown and distal 1/4 is brown; and the lateral ends of the metanotum are lighter than remaining area.

#### Etymology.

Paul E. Hanson is interested in systematics and host associations in parasitoids. He works at the Universidad de Costa Rica San Pedro, San José, Costa Rica.

#### Distribution.

Parasitized caterpillars were collected in Costa Rica, ACG, Sector Brasilia (Brumas and Piedrona) and Sector Pitilla (Colocho, Pasmompa, and Sendero Montecele), during October–November 2005, September 2007, February 2010, and May and July 2011 at 340 m, 440 m, and 680 m in rain forest.

#### Biology.

The lifestyle of this parasitoid species is gregarious.

#### Host.

*Yidalptaauragalis* Guenée (Noctuidae: Catocalinae) feeding on *Securidacasylvestris* and *S.diversifolia* (Polygalaceae). Caterpillars were collected in fourth and fifth instar.

### Glyptapanteles paulheberti

Taxon classificationAnimaliaHymenopteraBraconidae

Arias-Penna, sp. nov.

http://zoobank.org/C630E728-A839-4AAD-A61C-A75FC46F66B1

Figs 182 , 183

#### Female.

Body length 2.02 mm, antenna length 2.78 mm, fore wing length 2.58 mm.

#### Type material.

**Holotype**: COSTA RICA • 1♀; 06-SRNP-35282, DHJPAR0012113; Área de Conservación Guanacaste, Guanacaste, Sector Cacao, SenderoDerrumbe; cloud forest; 1,220 m; 10.92918, 85.46426; 06.vi.2006; Dunia Garcia leg.; caterpillar collected in third instar; white bud-like cocoons in litter or soil and formed on 19.vi.2006; adult parasitoid emerged on 27.vi.2006; (CNC). **Paratypes.** • 14 (2♀, 2♂) (9♀, 1♂); 06-SRNP-35282, DHJPAR0012113; same data as for holotype; (CNC).

#### Other material.

**Reared material.** COSTA RICA: *Área de Conservación Guanacaste*, *Guanacaste*, *Sector Cacao*, *SenderoDerrumbe*: • 9 (3♀, 2♂) (4♀, 0♂); 06-SRNP-35283, DHJPAR0012110; cloud forest; 1,220 m; 10.92918, 85.46426; 06.vi.2006; Dunia Garcia leg.; caterpillar collected in third instar; white bud-like cocoons in litter or soil and formed on 19.vi.2006; adult parasitoids emerged on 27.vi.2006.

*Área de Conservación Guanacaste*, *Guanacaste*, *Sector Cacao*, *Sendero a Maritza*, *1 km NW Estación Cacao*: • 9 (3♀, 2♂) (4♀, 0♂); 10-SRNP-35968, DHJPAR0041645; cloud forest; 1,150 m; 10.92691, 85.46822; 26.viii.2010; Dunia Garcia leg.; caterpillar collected in third instar; cocoons formed on 07.ix.2010; adult parasitoids emerged on 15.ix.2010, 17.ix.2010.

#### Diagnosis.

**Figure 182.**
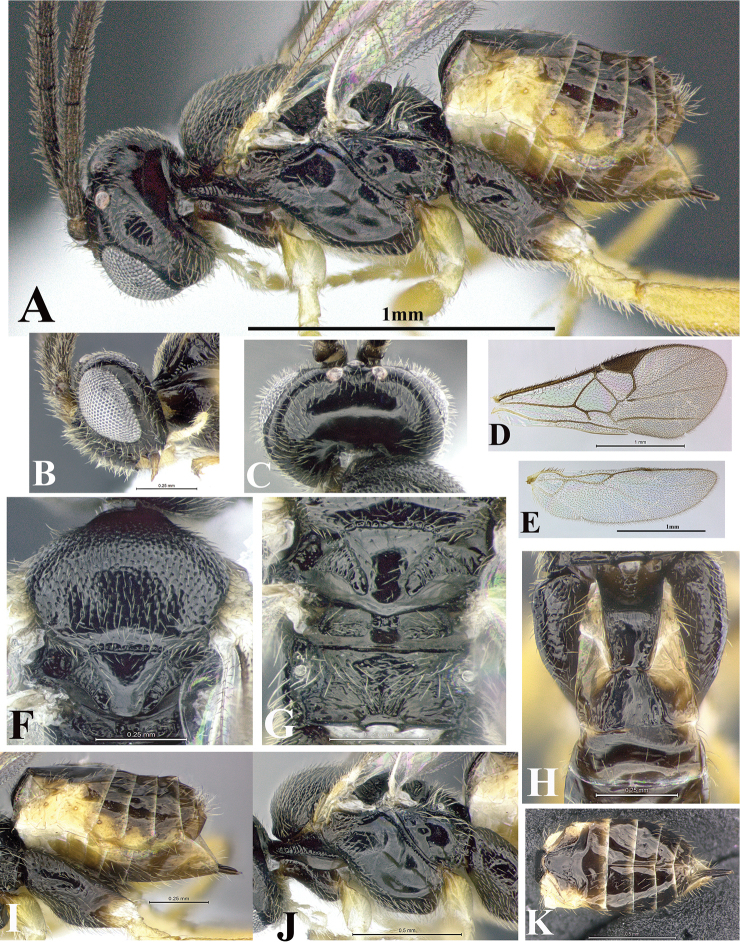
*Glyptapantelespaulheberti* sp. nov. female 06-SRNP-35282 DHJPAR0012113 **A** Habitus **B, C** Head **B** Lateral view **C** Dorsal view **D, E** Wings **D** Fore **E** Hind **F** Mesonotum, dorsal view **G** Scutellum, metanotum, propodeum, dorsal view **H**T1–3, dorsal view **I, K** Metasoma **I** Lateral view **K** Dorsal view **J** Mesosoma, lateral view.

**Figure 183.**
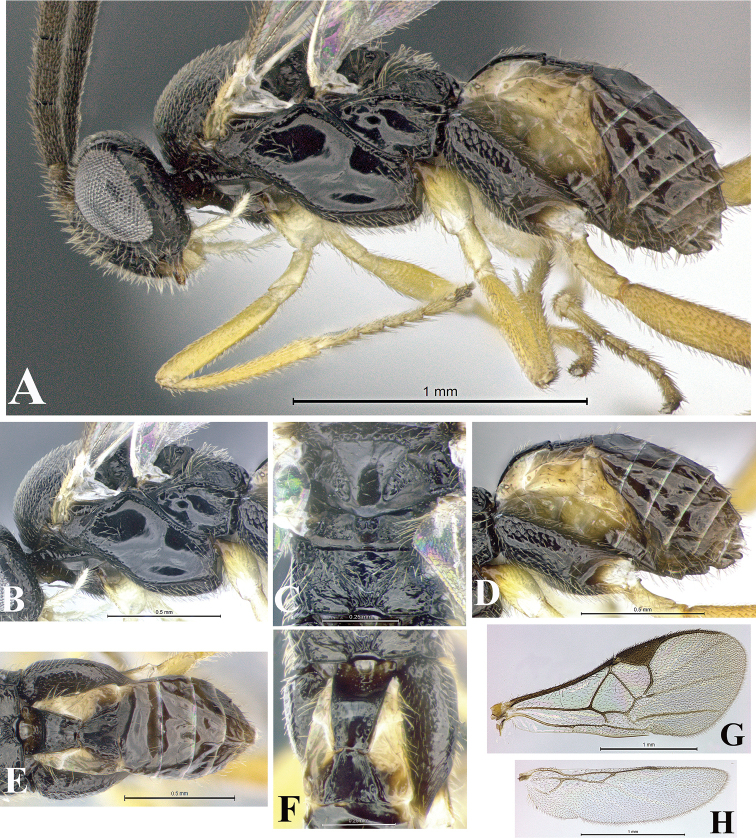
*Glyptapantelespaulheberti* sp. nov. male 06-SRNP-35282 DHJPAR0012113 **A** Habitus **B** Mesosoma, lateral view **C** Scutellum, metanotum, propodeum, dorsal view **D, E** Metasoma **D** Lateral view **F** Dorsal view **F**T1–2, dorsal view **G, H** Wings **G** Fore **H** Hind.

Distal antennal flagellomere subequal in length with penultimate, median area between lateral ocelli slightly depressed (Fig. 182C), in dorsal view, proximal half of propodeum more strongly curved (Figs 182G, 183C), petiole on T1 evenly narrowing distally (Figs 182H, 183F), dorsal outer depression on hind coxa present (Figs 182A, I, 183A, D), edges of median area on T2 obscured by little sculpture, and fore wing with r vein curved, outer side of junction of r and 2RS veins forming a slight stub (Figs 182D, 183D).

#### Coloration

(Fig. 182A–K). General body coloration shiny black except scape brown, but 2/3 proximal yellow; pedicel brown, but distally yellow; all antennal flagellomeres dark brown on both sides; labrum, mandible, and tegulae yellow; maxillary and labial palps ivory/pale yellow; both ends of propleuron, epicnemial ridge, and both dorsal and ventral furrows of pronotum lighter than mesosoma coloration. Eyes and ocelli silver. Fore and middle legs yellow except brown claws; hind legs yellow except black coxae only distally yellow, distal half of tibiae and tarsomeres dark brown, but basitarsus proximally with a yellow ring. Petiole on T1 brown and sublateral areas yellow; T2 with median and adjacent areas yellow-brown, and lateral ends yellow; T3 broadly brown, shape of dark area coinciding with the width of median and adjacent areas on T2, 1/3 proximal of lateral ends yellow, and T3 distally with a wide yellow-brown band; T4 and beyond completely brown; distally each tergum with a narrow yellow-brown transparent band. In lateral view, T1–2 completely yellow; T3 and beyond yellow, but dorsally brown, extent of brown area remaining constant from proximal to distal. S1–2 yellow; S3–4 yellow, but medially brown; penultimate sternum and hypopygium yellow-brown/brown.

#### Description.

**Head** (Fig. 182A–C). Head rounded with pubescence long and dense. Proximal three antennal flagellomeres longer than wide (0.19:0.06, 0.21:0.06, 0.20:0.06), distal antennal flagellomere subequal in length with penultimate (0.10:0.04, 0.09:0.04), antenna longer than body (2.78, 2.02); antennal scrobes-frons sloped and forming a shelf. Face convex, punctate-lacunose, interspaces wavy and longitudinal median carina present. Frons punctate. Temple wide, punctate and interspaces clearly smooth. Inner margin of eyes diverging slightly at antennal sockets; in lateral view, eye anteriorly convex and posteriorly straight. POL shorter than OOL (0.08, 0.12). Malar suture present. Median area between lateral ocelli slightly depressed. Vertex lateral rounded and dorsally wide.

**Mesosoma** (Fig. 182A, F, G, J). Mesosoma dorsoventrally convex. Mesoscutum proximally convex and distally flat, punctation distinct throughout, interspaces smooth. Scutellum triangular, apex sloped and fused with BS, scutellar punctation indistinct throughout, in profile scutellum flat and on same plane as mesoscutum, phragma of the scutellum partially exposed; BS only very partially overlapping the MPM; ATS demilune with quite a little, complete and parallel carinae; dorsal ATS groove smooth. Transscutal articulation with small and heterogeneous foveae, area just behind transscutal articulation with a smooth and shiny sloped transverse strip. Metanotum with BM wider than PFM (clearly differentiated); MPM semicircular and bisected by a median longitudinal carina; AFM with a small lobe and not as well delineated as PFM; PFM thick and smooth; ATM proximally with a groove with some sculpturing and distally smooth. Propodeum without median longitudinal carina, proximal half curved with medium-sized sculpture and distal half with a shallow dent at each side of nucha; distal edge of propodeum with a flange at each side and without stubs; propodeal spiracle without distal carina; nucha surrounded by long radiating carinae. Pronotum with a distinct dorsal furrow, dorsally with a well-defined smooth band; central area of pronotum smooth, but both dorsal and ventral furrows with short parallel carinae. Propleuron with fine punctations throughout and dorsally without a carina. Metasternum flat or nearly so. Contour of mesopleuron straight/angulate or nearly so; precoxal groove smooth, shiny and distinct; epicnemial ridge convex, teardrop-shaped.

**Legs.** Ventral margin of fore telotarsus excavated with conspicuous curved seta over this excavation, fore telotarsus almost same width throughout and longer than fourth tarsomere (0.11, 0.06). Hind coxa with dorsal half sparsely punctate, ventral half densely punctate, and dorsal outer depression present. Inner spur of hind tibia longer than outer spur (0.18, 0.15), entire surface of hind tibia with dense strong spines clearly differentiated by color and length. Hind telotarsus as equal in length as fourth tarsomere (0.11, 0.10).

**Wings** (Fig. 182D, E). Fore wing with r vein slightly curved; 2RS vein slightly convex to convex; r and 2RS veins forming a weak, even curve at their junction and outer side of junction forming a slight stub; 2M vein slightly curved/swollen; distally fore wing [where spectral veins are] with microtrichiae more densely concentrated than the rest of the wing; anal cell 1/3 proximally lacking microtrichiae; subbasal cell with microtrichiae virtually throughout; veins 2CUa and 2CUb completely spectral; vein 2 cu-a absent; vein 2-1A proximally tubular and distally spectral, although sometimes difficult to see; tubular vein 1 cu-a straight, incomplete/broken and not reaching the edge of 1-1A vein. Hind wing with vannal lobe very narrow, subdistally and subproximally evenly convex, and setae evenly scattered in the margin.

**Metasoma** (Fig. 182A, H, I, K). Metasoma laterally compressed. Petiole on T1 finely sculptured only laterally, evenly narrowing distally (length 0.33, maximum width 0.14, minimum width 0.10) and with scattered pubescence concentrated in the first distal third. Lateral grooves delimiting the median area on T2 clearly defined and reaching the distal edge of T2 (length median area 0.17, length T2 0.17), edges of median area with little sculpture, median area longer than broad (length 0.17, maximum width 0.15, minimum width 0.09); T2 with scarce pubescence throughout. T3 as long as T2 (0.18, 0.17) and with scattered pubescence throughout. Pubescence on hypopygium dense.

**Cocoons.** White bud-like cocoons with ridge-shaped body and evenly smooth silk fibers. Cocoons in litter or soil.

#### Comments.

In some females, the coloration on sterna varies: S1–3 yellow, S4 and beyond yellow, but medially brown, extent of that brown area increasing from proximal to distal.

#### Male

(Fig. 183A–H). Similar in coloration to female. In profile, the body looks more curved than female.

#### Etymology.

Paul D. N. Hebert is a Canadian biologist, director of the Biodiversity Institute of Ontario (BIO), University of Guelph, Ontario, Canada. He is best known as the “father of DNA barcoding”.

#### Distribution.

The parasitized caterpillars were collected in Costa Rica, ACG, Sector Cacao (SenderoDerrumbe and Sendero a Maritza), during June 2006 and August 2010 at 1,150 m and 1,220 m in cloud forest.

#### Biology.

The lifestyle of this parasitoid species is gregarious.

#### Host.

*Disphragisproba* Schaus (Notodontidae: Heterocampinae) feeding on *Ocotealeucoxylon* and *Nectandrasalicifolia* (Lauraceae). Caterpillars were collected in third instar.

### Glyptapanteles paulhurdi

Taxon classificationAnimaliaHymenopteraBraconidae

Arias-Penna, sp. nov.

http://zoobank.org/92480897-46FB-4421-9DBA-B4AE29F66F09

Figs 184 , 185

#### Female.

Body length 2.63 mm, antenna length 3.33 mm, fore wing length 3.00 mm.

#### Type material.

**Holotype**: COSTA RICA • 1♀; 08-SRNP-49, DHJPAR0020737; Área de Conservación Guanacaste, Alajuela, Sector San Cristóbal, Bosque Trampa Malaise; rain forest; 815 m; 10.86280, -85.38460; 06.i.2008; Carolina Cano leg.; caterpillar collected in fifth instar; white bud-like cocoons in litter or soil and formed on 06.i.2008; adult parasitoids emerged on 21.i.2008; (CNC). **Paratypes.** • 12 (6♀, 2♂) (4♀, 0♂); 08-SRNP-49, DHJPAR0020737; same data as for holotype; (CNC).

#### Diagnosis.

**Figure 184.**
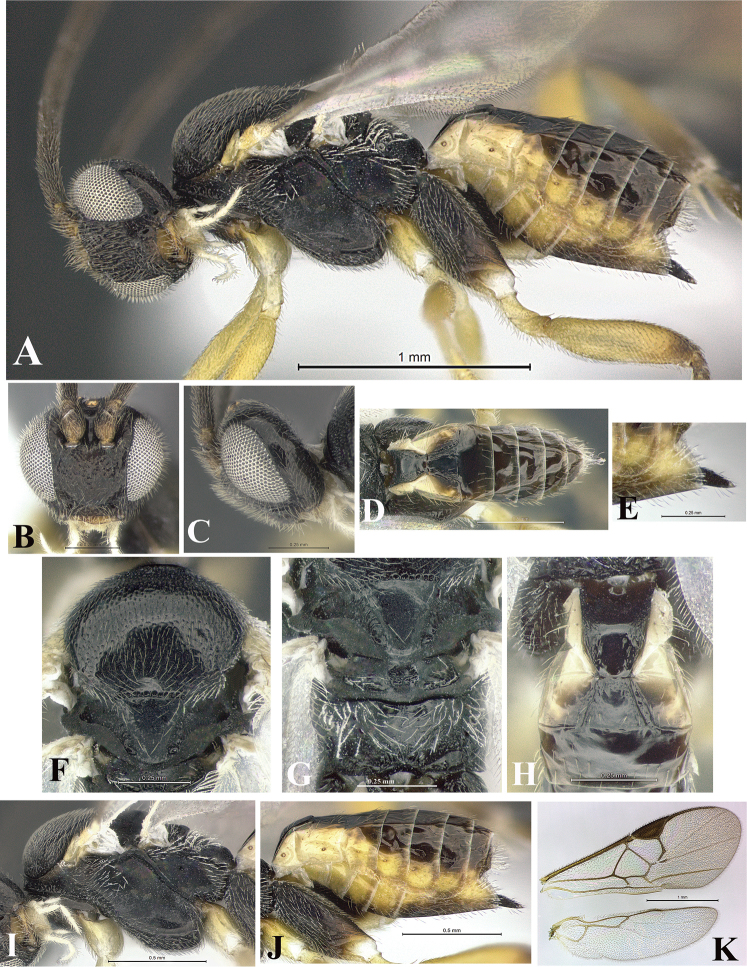
*Glyptapantelespaulhurdi* sp. nov. female 08-SRNP-49 DHJPAR0020737 **A** Habitus **B, C** Head **B** Frontal view **C** Lateral view **D, J** Metasoma **D** Dorsal view **J** Lateral view **E** Genitalia: hypopygium, ovipositor, ovipositor sheaths, lateral view **F** Mesonotum, dorsal view **G** Scutellum, metanotum, propodeum, dorsal view **H**T1–3, dorsal view **I** Mesosoma, lateral view **K** Fore and hind wings.

**Figure 185.**
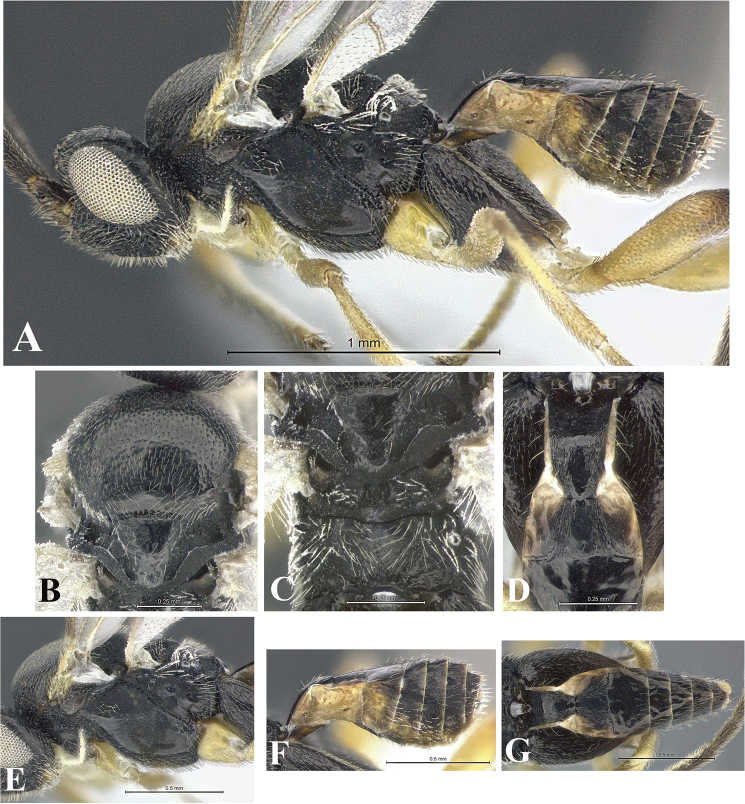
*Glyptapantelespaulhurdi* sp. nov. male 08-SRNP-49 DHJPAR0020737 **A** Habitus **B** Mesonotum, dorsal view **C** Scutellum, metanotum, propodeum, dorsal view **D**T1–3, dorsal view **E** Mesosoma, lateral view **F, G** Metasoma **F** Lateral view **G** Dorsal view.

Vertex in lateral view pointed or nearly so (Fig. 184C), dorsal carina delimiting a dorsal furrow on propleuron present, inner margin of eyes diverging slightly at antennal sockets (Fig. 184B), fore wing with vein 2-1A proximally tubular, distally spectral, r vein slightly curved, outer side of junction of r and 2RS veins forming a distinct stub (Fig. 184K), median area on T2 as broad as long, edges of median area on T2 obscured by weak longitudinal stripes (Figs 184H, 185D), antenna longer than body, scutellum in profile flat and on same plane as mesoscutum, in dorsal view, proximal half of propodeum weakly curved (Figs 184G, 185C), petiole on T1 evenly narrowing distally (Figs 184H, 185D), and dorsal outer depression on hind coxa present (Figs 184A, J, 185A).

#### Coloration

(Fig. 184A–K). General body coloration black except scape yellow-brown distally with a ring brown; pedicel proximally yellow-brown and distally brown; labrum, mandible, glossa, propleuron distally, and dorsal furrow of pronotum yellow-brown; all antennal flagellomeres dark brown on both sides; maxillary and labial palps, and tegulae yellow; clypeus, low face (just below antennal scrobes) with a small spot, propleuron proximally and also mostly ventrally, ventral furrow of pronotum, lunules, and lateral ends of metanotum with yellow-brown/reddish tints. Eyes and ocelli silver. Fore and middle legs yellow except brown claws; hind legs yellow except coxae distally yellow, femora distally with a tiny brown dot, both ends of tibiae brown, and tarsomeres brown although basitarsus proximally with a narrow yellow band. Petiole on T1 dark brown and sublateral areas yellow-brown; T2 with median area brown and lateral ends with two colorations: most of the area brown and some irregular spots yellow-brown; T3 and beyond brown; distally each tergum with a narrow yellowish transparent band. In lateral view, T1–2 yellow; T3 and beyond yellow, but dorsally brown, extent of brown area remaining constant throughout. S1–3 yellow; S4–5 yellow, but distally with a narrow longitudinal brown band; hypopygium yellow, but medially brown.

#### Description.

**Head** (Fig. 184A–C). Head rounded with pubescence long and dense. Proximal three antennal flagellomeres longer than wide (0.24:0.08, 0.25:0.08, 0.25:0.08), distal antennal flagellomere longer than penultimate (0.13:0.05, 0.10:0.05), antenna longer than body (3.33, 2.63); antennal scrobes-frons sloped and forming a shelf. Face flat or nearly so, with dense fine punctations, interspaces smooth and longitudinal median carina present. Frons smooth. Temple wide, punctate and interspaces clearly smooth. Inner margin of eyes diverging slightly at antennal sockets; in lateral view, eye anteriorly convex and posteriorly straight. POL shorter than OOL (0.09, 0.12). Malar suture present. Median area between lateral ocelli slightly depressed. Vertex laterally pointed or nearly so and dorsally wide.

**Mesosoma** (Fig. 184A, F, G, I). Mesosoma dorsoventrally convex. Mesoscutum proximally convex and distally flat, punctation distinct throughout, interspaces smooth. Scutellum triangular, apex sloped and fused with BS, scutellar punctation indistinct throughout, in profile scutellum flat and on same plane as mesoscutum, phragma of the scutellum partially exposed; BS only very partially overlapping the MPM; ATS demilune with a little, complete and parallel carinae, dorsal ATS groove with semicircular/parallel carinae. Transscutal articulation with small and homogeneous foveae, area just behind transscutal articulation with a smooth and shiny sloped transverse strip. Metanotum with BM as same width as PFM (not clearly differentiated); MPM circular with some sculpture inside; AFM with a small lobe and not as well delineated as PFM; PFM thick, smooth and with lateral ends rounded; ATM proximally with a groove with some sculpturing and distally smooth. Propodeum with a shallow dent, proximal half weakly curved with medium-sized sculpture and distal half slightly rugose; distal edge of propodeum with a flange at each side and without stubs; propodeal spiracle without distal carina; nucha surrounded by long radiating carinae. Pronotum with a distinct dorsal furrow, dorsally with a well-defined smooth band; central area of pronotum smooth, but both dorsal and ventral furrows with short parallel carinae. Propleuron with fine punctations throughout and dorsally with a carina. Metasternum flat or nearly so. Contour of mesopleuron convex; precoxal groove deep, smooth and shiny; epicnemial ridge convex, teardrop-shaped.

**Legs.** Ventral margin of fore telotarsus slightly excavated and with a tiny curved seta, fore telotarsus almost same width throughout and longer than fourth tarsomere (0.14, 0.08). Hind coxa with dorsal half sparsely punctate, ventral half densely punctate, and dorsal outer depression present. Inner spur of hind tibia longer than outer spur (0.26, 0.21), entire surface of hind tibia with dense strong spines clearly differentiated by color and length. Hind telotarsus longer than fourth tarsomere (0.16, 0.12).

**Wings** (Fig. 184K). Fore wing with r vein slightly curved; 2RS vein straight; r and 2RS veins forming an angle at their junction and outer side of junction forming a slight stub; 2M vein slightly curved/swollen; distally fore wing [where spectral veins are] with microtrichiae more densely concentrated than the rest of the wing; anal cell 1/3 proximally lacking microtrichiae; subbasal cell with microtrichiae virtually throughout; veins 2CUa and 2CUb completely spectral; vein 2 cu-a present as spectral vein, sometimes difficult to see; vein 2-1A proximally tubular and distally spectral, although sometimes difficult to see; tubular vein 1 cu-a curved and complete, but junction with 1-1A vein spectral. Hind wing with vannal lobe very narrow, subdistally and subproximally straightened, and setae evenly scattered in the margin.

**Metasoma** (Fig. 184A, D, E, H, J). Metasoma laterally compressed. Petiole on T1 finely sculptured only laterally, evenly narrowing distally (length 0.36, maximum width 0.21, minimum width 0.10) and with scattered pubescence concentrated in the first distal third. Lateral grooves delimiting the median area on T2 clearly defined and reaching the distal edge of T2 (length median area 0.17, length T2 0.17), edges of median area obscured by weak longitudinal stripes, median area as broad as long (length 0.17, maximum width 0.18, minimum width 0.09); T2 scarce pubescence throughout. T3 longer than T2 (0.22, 0.17) and with scattered pubescence only distally. Pubescence on hypopygium dense.

**Cocoons.** White bud-like cocoon with ridge-shaped body and evenly smooth silk fibers. Cocoons in litter or soil.

#### Comments.

In some females, the scape is mostly brown with some yellow areas.

#### Male

(Fig. 185A–G). The mesosoma is wider and stouter than female.

#### Etymology.

Paul David Hurd Jr. (2 April 1921-12 March 1982) was an authority on the taxonomy and biology of bees (superfamily Apoidea). He was curator at the National Museum of Natural History, Smithsonian Institution, Washington DC., USA.

#### Distribution.

The parasitized caterpillar was collected in Costa Rica, ACG, Sector San Cristóbal (Bosque Trampa Malaise), during January 2008 at 815 m in rain forest.

#### Biology.

The lifestyle of this parasitoid species is gregarious.

#### Host.

*Rosemaattenuata* (Dognin) (Notodontidae: Phalerinae) feeding on *Ingaoerstediana* (Fabaceae). Caterpillar was collected in fifth instar.

### Glyptapanteles petermarzi

Taxon classificationAnimaliaHymenopteraBraconidae

Arias-Penna, sp. nov.

http://zoobank.org/A5282B0D-7168-4473-903B-4E8B52B685CC

Fig. 186

#### Female.

Body length 2.38 mm, antenna length 3.08 mm, fore wing length 3.03 mm.

#### Type material.

**Holotype**: ECUADOR • 1♀; EC-12684, YY-A091; Napo, Yanayacu Biological Station, Río Cosanga, Plot 188; cloud forest; 2,145 m; -0.595917, -77.880017; 01.iii.2006; María de los Angeles Simbaña leg.; caterpillar collected in second instar; cocoons formed on 07.iv.2006; adult parasitoids emerged on 24.iv.2006; (PUCE). **Paratypes.** • 11 (4♀, 3♂) (4♀, 0♂); EC-12684, YY-A091; same data as for holotype; (PUCE).

#### Diagnosis.

**Figure 186.**
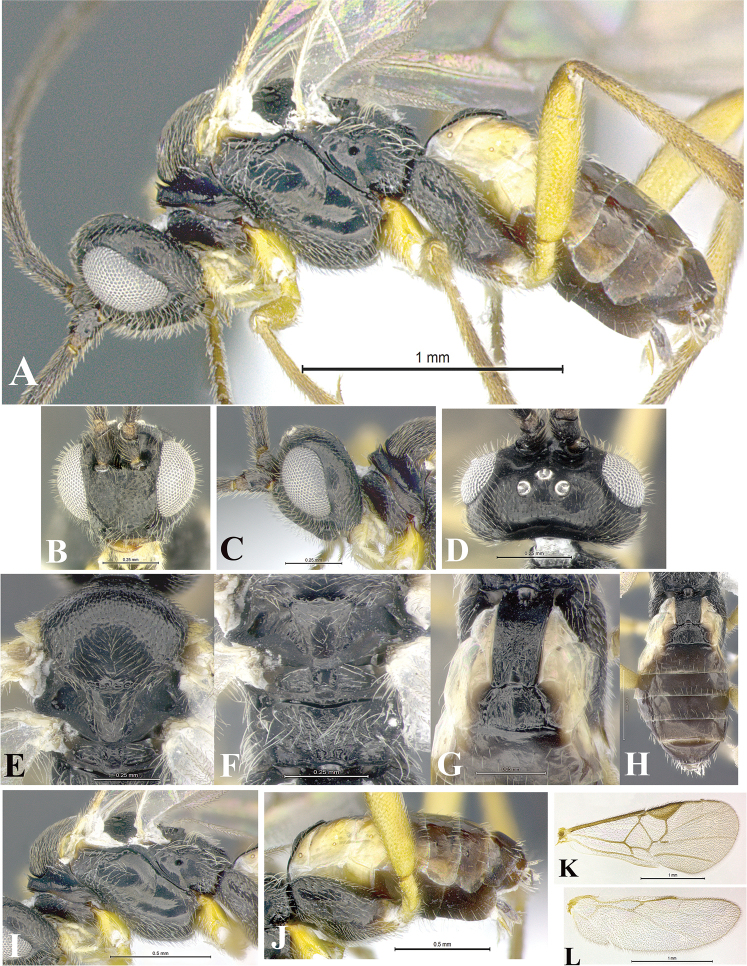
*Glyptapantelespetermarzi* sp. nov. female EC-12684 YY-A091 **A** Habitus **B, D** Head **B** Frontal view **D** Dorsal view **C** Head, pronotum, propleuron, lateral view **E** Mesonotum, dorsal view **F** Scutellum, metanotum, propodeum, dorsal view **G**T1–2, dorsal view **H, J** Metasoma **H** Dorsal view **J** Lateral view **I** Mesosoma, lateral view **K, L** Wings **K** Fore **L** Hind.

Petiole on T1 with lateral margin straight throughout (Fig. 186G, H), fore telotarsus proximally narrow, distally wide, dorsal furrow of pronotum without a smooth band (Fig. 186A, I), petiole with a mix of fine rugae and coarse sculpture over most of the surface (Fig. 186G, H), propodeum rather coarse sculpture, with transverse rugae (Fig. 186F), fore wing with vein 1 cu-a straight, r vein curved, outer side of junction of r and 2RS veins not forming a stub (Fig. 186K), dorsal outer depression on hind coxa present (Fig. 186A, J), inner margin of eyes diverging slightly at antennal sockets (Fig. 186B), and lateral grooves delimiting the median area on T2 clearly defined and reaching the distal edge of T2 (Fig. 186G, H).

#### Coloration

(Fig. 186A–L). General body coloration black except labrum, mandibles, lateral ends of metapleuron, ventral furrow of pronotum, and metapleuron with brown/reddish tints; glossa, maxillary and labial palps, and tegulae yellow; pedicel yellow-reddish; scape and all antennal flagellomeres (on both sides) dark brown/black. Eyes and ocelli silver. Fore and middle legs dark yellow except claws brown, although femora, tibiae and tarsomeres with a narrow dorsal strip brown from top to bottom; hind legs dark yellow except coxae black distally brown/reddish, (coloration brown/reddish cover more area in the inner side), femora dorsally with a tiny brown spot, additionally with a narrow dorsal brown strip from top to bottom, tibiae with both ends brown, and tarsomeres brown. Petiole on T1 black and sublateral areas yellow; T2 with median area black, very narrow adjacent area dark brown, thus contours of median area looks darker, and lateral ends yellow; T3 light brown except lateral ends proximally yellow-brown; T4 and beyond light brown; distally each tergum with a narrow yellowish transparent band. In lateral view, T1–2 yellow; T3 yellow, but dorsally brown; T4 and beyond brown-reddish. S1–2 yellow; S3 yellow, but medially brown; S4 and beyond brown.

#### Description.

**Head** (Fig. 186A–D). Head rounded with pubescence long and dense. Proximal three antennal flagellomeres longer than wide (0.22:0.05, 0.21:0.05, 0.22:0.05), distal antennal flagellomere longer than penultimate (0.13:0.05, 0.11:0.05), antenna longer than body (3.08, 2.38); antennal scrobes-frons shallow. Face convex, punctations barely noticeable, interspaces wavy and longitudinal median carina present. Frons smooth. Temple wide, punctate-lacunose and interspaces wavy. Inner margin of eyes diverging slightly at antennal sockets; in lateral view, eye anteriorly convex and posteriorly straight. POL shorter than OOL (0.08, 0.12). Malar suture absent or difficult to see. Median area between lateral ocelli without depression. Vertex laterally rounded and dorsally wide.

**Mesosoma** (Fig. 186A, E, F, I). Mesosoma dorsoventrally convex. Mesoscutum 1/4 distal with a central dent, punctation distinct proximally, but absent/dispersed distally, interspaces wavy/lacunose. Scutellum long and slender, apex sloped and fused with BS, but not in the same plane, scutellar punctation scattered throughout, in profile scutellum flat and on same plane as mesoscutum, phragma of the scutellum partially exposed; BS only very partially overlapping the MPM; ATS demilune with complete undulate/reticulate carinae; dorsal ATS groove smooth. Transscutal articulation with small and heterogeneous foveae, area just behind transscutal articulation depressed centrally and with same kind of sculpture as mesoscutum. Metanotum with BM convex; MPM circular without median longitudinal carina; AFM with a small lobe and not as well delineated as PFM; PFM thick, smooth and with lateral ends rounded; ATM proximally with a groove with some sculpturing and distally smooth. Propodeum rather coarse sculpture, with transverse rugae, proximal half curved; distal edge of propodeum with a flange at each side and without stubs; propodeal spiracle distally framed by a short transverse carina; nucha surrounded by long radiating carinae. Pronotum virtually without trace of dorsal furrow, dorsally without a smooth band; short parallel carinae only in ventral furrow. Propleuron with fine punctations throughout and dorsally without a carina. Metasternum convex. Contour of mesopleuron convex; precoxal groove smooth, shiny and shallow, but visible; epicnemial ridge convex, teardrop-shaped.

**Legs.** Ventral margin of fore telotarsus excavated with conspicuous curved seta over this excavation, fore telotarsus proximally narrow and distally wide, and longer than fourth tarsomere (0.18, 0.07). Hind coxa with dorsal half sparsely punctate, ventral half densely punctate, and dorsal outer depression present. Inner spur of hind tibia longer than outer spur (0.23, 0.20), entire surface of hind tibia with dense strong spines clearly differentiated by color and length. Hind telotarsus longer than fourth tarsomere (0.17, 0.14).

**Wings** (Fig. 186K, L). Fore wing with r vein slightly curved; 2RS vein slightly convex to convex; r and 2RS veins forming a weak, even curve at their junction and outer side of junction not forming a stub; 2M vein slightly curved/swollen; distally fore wing [where spectral veins are] with microtrichiae more densely concentrated than the rest of the wing; anal cell 1/3 proximally lacking microtrichiae; subbasal cell with microtrichiae virtually throughout; vein 2CUa absent and vein 2CUb spectral; vein 2 cu-a absent; vein 2-1A proximally tubular and distally spectral, although sometimes difficult to see; tubular vein 1 cu-a straight, incomplete/broken and not reaching the edge of 1-1A vein. Hind wing with vannal lobe narrow, subdistally and subproximally straightened, and setae evenly scattered in the margin.

**Metasoma** (Fig. 186A, G, H, J). Metasoma cylindrical. Petiole on T1 with a mix of fine rugae and coarse sculpture over most of the surface, virtually parallel-sided over most of length, but barely narrowing at apex, apex truncate (length 0.36, maximum width 0.18, minimum width 0.16), and with scattered pubescence concentrated in the first distal third. Lateral grooves delimiting the median area on T2 clearly defined and reaching the distal edge of T2 (length median area 0.14, length T2 0.14), edges of median area obscured by coarse sculpture, median area broader than long (length 0.14, maximum width 0.22, minimum width 0.12); T2 with scattered pubescence only distally. T3 longer than T2 (0.20, 0.14) and with scattered pubescence throughout. Pubescence on hypopygium dense.

**Cocoons.** Unknown.

#### Comments.

The specimens are slender/elongate and cylindrical, the body is distinctively curved (Fig. 186A), and the limit between the mesopleuron and the metasternum is truncate throughout.

#### Male.

Similar in coloration to females.

#### Etymology.

Peter Marz is a German journalist and husband of DCAP.

#### Distribution.

Parasitized caterpillar was collected in Ecuador, Napo, Yanayacu Biological Station (Río Cosanga), during March 2006 at 2,145 m in cloud forest.

#### Biology.

The lifestyle of this parasitoid species is gregarious.

#### Host.

Undetermined species of Geometridae, food plant was not reported. Caterpillar was collected in second instar.

### Glyptapanteles phildevriesi

Taxon classificationAnimaliaHymenopteraBraconidae

Arias-Penna, sp. nov.

http://zoobank.org/C244B56D-2DF3-41FC-9F26-0C7565A64E36

Fig. 187

#### Female.

Body length 2.53 mm, antenna length 2.68 mm, fore wing length 2.83 mm.

#### Type material.

**Holotype**: ECUADOR • 1♀; EC-12877, YY-A038; Napo, Yanayacu Biological Station, Yanayacu Road; cloud forest; 2,100 m; -0.566667, -77.866667; 05.iii.2006; Rafael Granizo leg.; caterpillar collected in third instar; cocoons formed on 30.i.2009; adult parasitoids emerged on 19.iv.2006; (PUCE). **Paratypes.** • 13 (4♀, 2♂) (7♀, 0♂); EC-12877, YY-A038; same data as for holotype; (PUCE).

#### Diagnosis.

**Figure 187.**
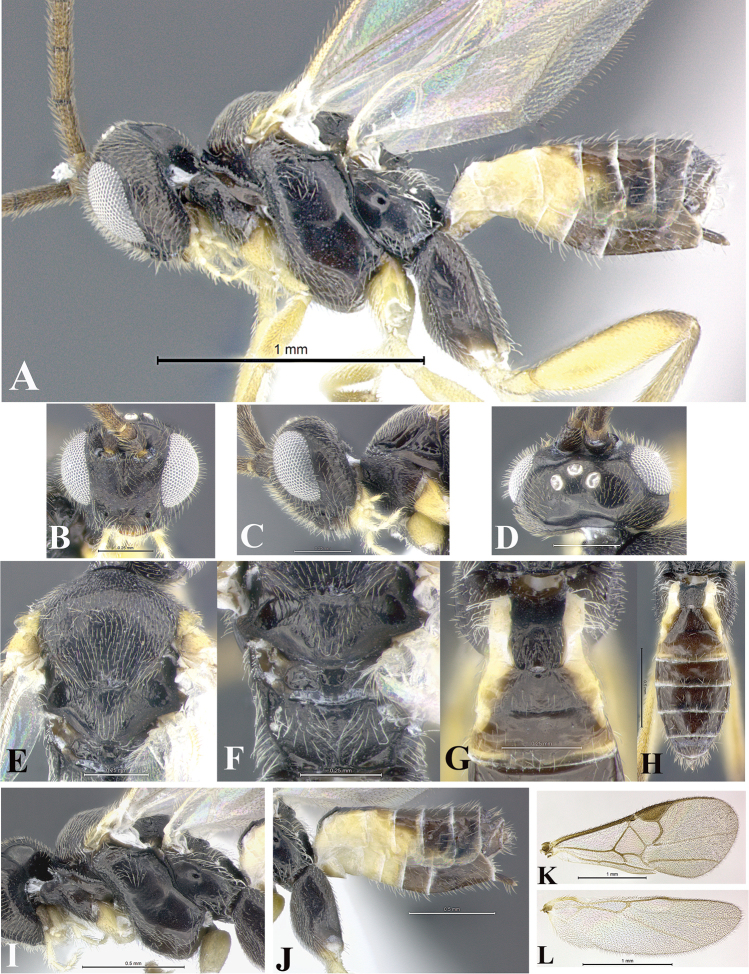
*Glyptapantelesphildevriesi* sp. nov. female EC-12877 YY-A038 **A** Habitus **B, D** Head **B** Frontal view **D** Dorsal view **C** Head, pronotum, propleuron, lateral view **E** Mesonotum, dorsal view **F** Scutellum, metanotum, propodeum, dorsal view **G**T1–3, dorsal view **H, J** Metasoma **H** Dorsal view **J** Lateral view **I** Mesosoma, lateral view **K, L** Wings **K** Fore **L** Hind.

Petiole on T1 with rugae, lateral margin in proximal half straight and distal half curved (convex, Fig. 187G, H), propodeum with a median longitudinal dent, but no trace of median longitudinal carina (Fig. 187F), hind coxa medially smooth, dorsally sparsely punctate, ventrally densely punctate, dorsal outer depression present (Fig. 187A, J), mesoscutum punctation proximally distinct, but distally absent/dispersed (Fig. 187E), precoxal groove shallow, but visible (Fig. 187A, I), fore wing with vein 1 cu-a curved, r vein s curved, outer side of junction of r and 2RS veins not forming a stub (Fig. 187K), inner margin of eyes diverging slightly at antennal sockets (Fig. 187B), and lateral grooves delimiting the median area on T2 clearly defined and reaching the distal edge of T2 (Fig. 187G, H).

#### Coloration

(Fig. 187A–L). General body coloration black except apex of mandibles, labrum, and pedicel yellow-brown; scape brown; all antennal flagellomeres dorsally lighter (light brown) than ventrally (dark brown); propleuron, both dorsal and ventral furrows of pronotum, epicnemial ridge, ventral edge of mesopleuron, metasternum, mesoscutum, lunules, BS, PFM and BM with some brown/reddish tints; glossa, maxillary and labial palps, and tegulae yellow. Eyes and ocelli silver. Fore and middle legs dark yellow except brown claws, although dorsally coxae with a brown smear area, additionally femora, tibiae and tarsomeres with a dorsal narrow light brown strip from top to bottom; hind legs yellow except brown-reddish coxae apically yellow, femora dorsally with a small brown spot, tibiae distally brown and proximally with a narrow brown band, and tarsomeres light brown. Petiole on T1 brown, contours darkened and sublateral areas yellow; T2 with median and adjacent areas light brown, adjacent area very narrow, and lateral ends yellow; T3 mostly brown although distal half lighter than proximal half, additionally proximally corners yellow; T4 and beyond completely brown; distally each tergum with a narrow yellowish transparent band. In lateral view, T1–2 yellow; T3 yellow, but dorsally brown; T4 and beyond completely brown. S1–4 yellow; penultimate sternum and hypopygium brown.

#### Description.

**Head** (Fig. 187A–D). Head rounded with pubescence long and dense. Proximal three antennal flagellomeres longer than wide (0.21:0.07, 0.20:0.07, 0.20:0.07), distal antennal flagellomere longer than penultimate (0.11:0.05, 0.08:0.05), antenna longer than body (2.68, 2.53); antennal scrobes-frons sloped and forming a shelf. Face with distal half dented only laterally, fine and punctate-lacunose, interspaces wavy and longitudinal median carina present. Frons smooth. Temple wide, punctate-lacunose and interspaces wavy. Inner margin of eyes diverging slightly at antennal sockets; in lateral view, eye anteriorly convex and posteriorly straight. POL shorter than OOL (0.08, 0.11). Malar suture faint. Median area between lateral ocelli without depression. Vertex laterally rounded and dorsally wide.

**Mesosoma** (Fig. 187A, E, F, I). Mesosoma dorsoventrally convex. Mesoscutum proximally convex and distally flat, punctation distinct proximally, but absent/dispersed distally, interspaces smooth. Scutellum triangular, apex sloped and fused with BS, but not in the same plane, scutellar punctation distinct throughout, in profile scutellum flat and on same plane as mesoscutum, phragma of the scutellum partially exposed; BS only very partially overlapping the MPM; ATS demilune with short stubs delineating the area; dorsal ATS groove with semicircular/parallel carinae. Transscutal articulation with small and heterogeneous foveae, area just behind transscutal articulation nearly at the same level as mesoscutum (flat) and with same kind of sculpture as mesoscutum. Metanotum with BM convex; MPM circular without median longitudinal carina; AFM without setiferous lobes and not as well delineated as PFM; PFM thick, smooth and with lateral ends rounded; ATM proximally with a groove and some sculpture, and distally smooth. Propodeum with a median longitudinal dent, but no trace of median longitudinal carina, proximal half weakly curved with medium-sized sculpture and distal half relatively polished and with a shallow dent at each side of nucha; distal edge of propodeum with a flange at each side and without stubs; propodeal spiracle distally framed by a short transverse carina; nucha surrounded by very short radiating carinae. Pronotum with a distinct dorsal furrow, dorsally with a well-defined smooth band; central area of pronotum and dorsal furrow smooth, but ventral furrow with short parallel carinae. Propleuron finely sculptured only ventrally and dorsally without a carina. Metasternum convex. Contour of mesopleuron convex; precoxal groove smooth, shiny and shallow, but visible; epicnemial ridge convex, teardrop-shaped.

**Legs.** Ventral margin of fore telotarsus excavated, but without seta, fore telotarsus almost same width throughout and longer than fourth tarsomere (0.12, 0.06). Hind coxa dorsally with scattered punctation, medially smooth and ventrally with dense punctation, dorsal outer depression present. Inner spur of hind tibia longer than outer spur (0.26, 0.20), entire surface of hind tibia with dense strong spines clearly differentiated by color and length. Hind telotarsus longer than fourth tarsomere (0.14, 0.12).

**Wings** (Fig. 187K, L). Fore wing with r vein slightly curved; 2RS vein straight; r and 2RS veins forming a weak, even curve at their junction and outer side of junction not forming a stub; 2M vein slightly curved/swollen; distally fore wing [where spectral veins are] with microtrichiae more densely concentrated than the rest of the wing; anal cell 1/3 proximally lacking microtrichiae; subbasal cell with microtrichiae virtually throughout; veins 2CUa and 2CUb completely spectral; vein 2 cu-a present as spectral vein, sometimes difficult to see; vein 2-1A proximally tubular and distally spectral, although sometimes difficult to see; tubular vein 1 cu-a curved and complete, but junction with 1-1A vein spectral. Hind wing with vannal lobe very narrow, subdistally and subproximally straightened, and setae evenly scattered in the margin.

**Metasoma** (Fig. 187A, G, H, J). Metasoma laterally compressed. Petiole on T1 with sculpture on distal half, virtually parallel-sided over most of length, but barely narrowing at apex, apex truncate (length 0.30, maximum width 0.17, minimum width 0.15), and with scattered pubescence concentrated in the first distal third. Lateral grooves delimiting the median area on T2 clearly defined and reaching the distal edge of T2 (length median area 0.15, length T2 0.15), edges of median area polished and lateral grooves deep, median area broader than long (length 0.15, maximum width 0.25, minimum width 0.11); T2 with scattered pubescence only distally. T3 longer than T2 (0.19, 0.15) and with scattered pubescence throughout. Pubescence on hypopygium dense.

**Cocoons.** Unknown.

#### Comments.

The ocelli are very close to each other (Fig. 187D, the diameter of lateral ocellus is 0.06 mm, the median ocellus is separated by 0.02 mm from the posterior ocellus), the petiole on T1 with lateral margins slightly straight in the proximal half, but in the distal half is curved (convex, Fig. 187G). As well as *G.suniae*, the petiole shape resembles the petiole of *Venanushelavai* Mason (Mason 1981, Fig. 77b). In some females, the coloration on T3 is brown proximally corners yellow, the limit between the mesopleuron and the metasternum is dented, and the body is slender.

#### Male.

Similar in coloration to female.

#### Etymology.

Philip (Phil) James DeVries is a tropical field ecologist interested in evolutionary patterns of species diversity, caterpillar-ant symbioses, insect wing shapes and flight behavior, and habitat partitioning. He works at the University of New Orleans, LA, USA.

#### Distribution.

Parasitized caterpillar was collected in Ecuador, Napo, Yanayacu Biological Station (Yanayacu Road), during March 2006 at 2,100 m in cloud forest.

#### Biology.

The lifestyle of this parasitoid species is gregarious.

#### Host.

*Daedalmadinias* Hewitson (Nymphalidae: Satyrinae) feeding on *Chusqueascandens* (Poaceae). Caterpillar was collected in third instar.

### Glyptapanteles philwardi

Taxon classificationAnimaliaHymenopteraBraconidae

Arias-Penna, sp. nov.

http://zoobank.org/ED7757A8-529F-4507-B132-DAF54E819589

Figs 188 , 189

#### Female.

Body length 1.81 mm, antenna length 1.81 mm, fore wing length 1.91 mm.

#### Type material.

**Holotype**: COSTA RICA • 1♀; 10-SRNP-13014, DHJPAR0040387; Área de Conservación Guanacaste, Guanacaste, Sector Santa Rosa, Área Administrativa; dry forest; 295 m; 10.83764, -85.61871; 01.viii.2010; Daniel H Janzen leg.; caterpillar collected in fifth instar and cocoons already formed; cocoons adhered to the larval cuticle; adult parasitoids emerged on 03.viii.2010; (CNC). **Paratypes.** • 72 (4♀, 5♂) (29♀, 34♂); 10-SRNP-13014, DHJPAR0040387; same data as for holotype; (CNC).

#### Other material.

**Malaise-trapped material.** COSTA RICA: *Área de Conservación Guanacaste*, *Guanacaste*, *Sector Santa Rosa*, *Bosque San Emilio*: • 1 (0♀, 0♂) (1♀, 0♂); 99-SRNP-18957, DHJPAR0013383; dry forest; Malasie; 300 m; 10.84389, -85.61384; 26.iv.1999; DH Janzen & W Hallwachs leg.• 1 (1♀, 0♂) (0♀, 0♂); 99-SRNP-19006, DHJPAR0013386; same data as for preceding except: 10.v.1999. • 1 (1♀, 0♂) (0♀, 0♂); 99-SRNP-19017, DHJPAR0013380; same data as for preceding except: 17.v.1999. • 1 (1♀, 0♂) (0♀, 0♂); 00-SRNP-23929, DHJPAR0013387; same data as for preceding except: 15.v.1999.

#### Diagnosis.

**Figure 188.**
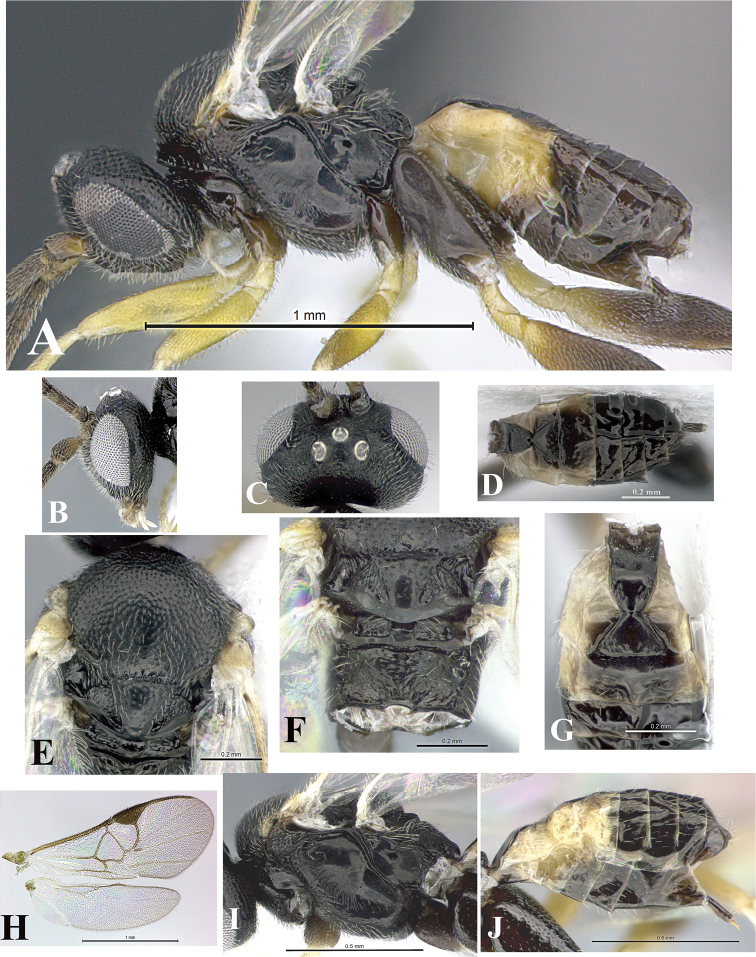
*Glyptapantelesphilwardi* sp. nov. female 99-SRNP-19017 DHJPAR0013380, 00-SRNP-23929 DHJPAR0013387, 10-SRNP-13014 DHJPAR0040387 **A** Habitus **B, C** Head **B** Lateral view **C** Dorsal view **D, J** Metasoma **D** Dorsal view **J** Lateral view **E** Mesonotum, dorsal view **F** Scutellum, metanotum, propodeum, dorsal view **G**T1–3, dorsal view **H** Fore and hind wings **I** Mesosoma, lateral view.

**Figure 189.**
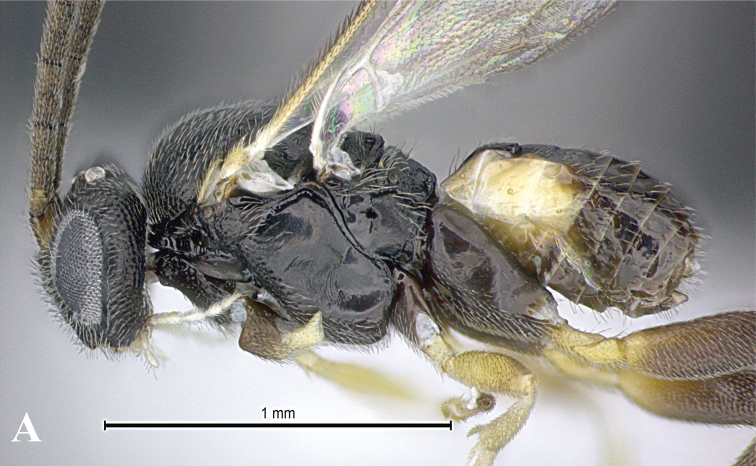
Habitus of *Glyptapantelesphilwardi* sp. nov. male 10-SRNP-13014 DHJPAR0040387 **A** Habitus.

Antenna as same length as body, anterior furrow of metanotum without setiferous lobes (Fig. 188F), distal antennal flagellomere subequal in length with penultimate, dorsal carina delimiting a dorsal furrow on propleuron present (Fig. 188A, I), surface of metasternum convex, precoxal groove deep with lineate sculpture (Figs 188A, I, 189A), fore wing with vein 1 cu-a curved, r vein curved (Fig. 188H), dorsal outer depression on hind coxa present (Figs 188A, 189A), inner margin of eyes diverging slightly at antennal sockets, petiole on T1 finely sculptured only laterally (Fig. 188G), and lateral grooves delimiting the median area on T2 clearly defined and reaching the distal edge of T2 (Fig. 188G).

#### Coloration

(Fig. 188A–J). General body coloration black except scape and pedicel yellow-brown with lateral brown band; all antennal flagellomeres brown on both sides; mandibles yellow-brown; labrum yellow; glossa, maxillary and labial palps, and tegulae ivory/pale yellow; metasternum and ventral furrow of pronotum lighter than mesosoma coloration. Eyes and ocelli silver. Fore and middle legs yellow-brown except light brown coxae, and claws brown; hind legs dark brown-black except trochanters and trochantellus yellow, femora dark although proximally yellow, medially yellow-brown and distally dark brown, tibia 1/3 proximal yellow and basitarsus proximally with a narrow yellow band. Petiole on T1 dark brown and sublateral areas yellow-brown; T2 with median and adjacent areas brown both forming a rectangle-shaped area, and lateral ends yellow; T3 almost completely brown, proximally dark area coinciding with width of dark median plus adjacent areas on T2, lateral ends yellow, additionally T3 distally with an ivory/pale yellow band; T4 and beyond completely brown; distally each tergum with an ivory/pale yellow transparent band. In lateral view, T1–3 yellow; T4 and beyond brown. S1–3 yellow; S4 and beyond brown.

#### Description.

**Head** (Fig. 188A–C). Head rounded with pubescence long and dense. Proximal three antennal flagellomeres longer than wide (0.15:0.05, 0.15:0.05, 0.14:0.05), distal antennal flagellomere subequal in length with penultimate (0.08:0.04, 0.07:0.04), antenna as same length as body (1.81, 1.81); antennal scrobes-frons shallow. Face with depression only laterally, punctate-lacunose, interspaces wavy and longitudinal median carina present. Frons smooth. Temple wide, punctate-lacunose and interspaces wavy. Inner margin of eyes diverging slightly at antennal sockets; in lateral view, eye anteriorly convex and posteriorly straight. POL shorter than OOL (0.08, 0.11). Malar suture absent or difficult to see. Median area between lateral ocelli slightly depressed. Vertex laterally pointed or nearly so and dorsally wide.

**Mesosoma** (Fig. 188A, E, F, I). Mesosoma dorsoventrally convex. Mesoscutum proximally convex and distally flat, punctation distinct proximally with polished area distally. Scutellum triangular, apex sloped and fused with BS, scutellar punctation scattered throughout, interspaces wavy/lacunose, in profile scutellum convex and slightly higher than mesoscutum, phragma of the scutellum partially exposed; BS only very partially overlapping the MPM; ATS demilune with short stubs delineating the area; dorsal ATS groove with semicircular/parallel carinae. Transscutal articulation with small and heterogeneous foveae, area just behind transscutal articulation with a smooth and shiny sloped transverse strip. Metanotum with BM wider than PFM (clearly differentiated); MPM circular without median longitudinal carina; AFM without setiferous lobes and not as well delineated as PFM; PFM thick, smooth and with lateral ends rounded; ATM proximally with a groove with some sculpturing and distally smooth. Propodeum without median longitudinal carina, proximal half curved with medium-sized sculpture and distal half relatively polished; distal edge of propodeum with a flange at each side and without stubs; propodeal spiracle without distal carina; nucha surrounded by very short radiating carinae. Pronotum with a distinct dorsal furrow, dorsally with a well-defined smooth band; central area of pronotum smooth, but both dorsal and ventral furrows with short parallel carinae. Propleuron with fine punctations throughout and dorsally with a carina. Metasternum convex. Contour of mesopleuron straight/angulate or nearly so; precoxal groove deep with transverse lineate sculpture; epicnemial ridge convex, teardrop-shaped.

**Legs.** Ventral margin of fore telotarsus excavated with conspicuous curved seta over this excavation, fore telotarsus almost same width throughout and longer than fourth tarsomere (0.10, 0.06). Hind coxa with punctation only on ventral surface, dorsal outer depression present. Inner spur of hind tibia longer than outer spur (0.17, 0.14), entire surface of hind tibia with dense strong spines clearly differentiated by color and length. Hind telotarsus longer than fourth tarsomere (0.11, 0.08).

**Wings** (Fig. 188H). Fore wing with r vein curved; 2RS vein straight; r and 2RS veins forming a weak, even curve at their junction and outer side of junction not forming a stub; 2M vein slightly curved/swollen; distally fore wing [where spectral veins are] with microtrichiae more densely concentrated than the rest of the wing; anal cell 1/3 proximally lacking microtrichiae; subbasal cell with a small smooth area; vein 2CUa absent and 2CUb spectral; vein 2 cu-a absent; vein 2-1A present only proximally as tubular vein; tubular vein 1 cu-a curved and complete, but junction with 1-1A vein spectral. Hind wing with vannal lobe narrow, subdistally and subproximally straightened, and setae present proximally, but absent distally.

**Metasoma** (Fig. 188A, D, G, J). Metasoma laterally compressed. Petiole on T1 finely sculptured only laterally, virtually parallel-sided over most of length, but narrowing over distal 1/3 (length 0.24, maximum width 0.12, minimum width 0.06), and with scattered pubescence concentrated in the first distal third. Lateral grooves delimiting the median area on T2 clearly defined and reaching the distal edge of T2 (length median area 0.10, length T2 0.10), edges of median area polished and lateral grooves deep, median area broader than long (length 0.10, maximum width 0.19, minimum width 0.05); T2 with scattered pubescence only distally. T3 longer than T2 (0.12, 0.10) and with scattered pubescence only distally. Pubescence on hypopygium scattered.

**Cocoons.** Unknown.

#### Male

(Fig. 189A). The metasoma looks wider and stouter than female.

#### Etymology.

Philip (Phil) S. Ward is a myrmecologist interested in systematics, biogeography, and evolution of ants, ant-plant mutualisms, phylogeny, and speciation. He works at the University of California, Davis, CA, USA.

#### Distribution.

The parasitized caterpillar was collected in Costa Rica, ACG, Sector Santa Rosa (Área Administrativa), during August 2010 at 295 m in dry forest.

Adult parasitoids were collected in Costa Rica, ACG, Sector Santa Rosa (Bosque San Emilio) during April, May 1999 at 300 m in dry forest.

#### Biology.

The lifestyle of this parasitoid species is gregarious.

#### Host.

Undetermined species of Geometridae feeding on *Pisoniaaculeata* (Nyctaginaceae). Caterpillar was collected in fifth instar.

### Glyptapanteles rafamanitioi

Taxon classificationAnimaliaHymenopteraBraconidae

Arias-Penna, sp. nov.

http://zoobank.org/5C742399-98CB-419F-B0B6-5574719CA965

Fig. 190

#### Female.

Body length 2.92 mm, antenna length 3.83 mm, fore wing length 3.78 mm.

#### Type material.

**Holotype**: ECUADOR • 1♀; EC-43493, YY-A138; Napo, Yanayacu Biological Station, Sendero Culo del Mundo, Plot 450; cloud forest; 2,352 m; -0.590833, -77.896389; 18.xi.2009; Wilmer Simbaña leg.; caterpillar collected in second instar; cocoon formed on 08.xii.2009; adult parasitoid emerged on 08.i.2010; (PUCE).

#### Diagnosis.

**Figure 190.**
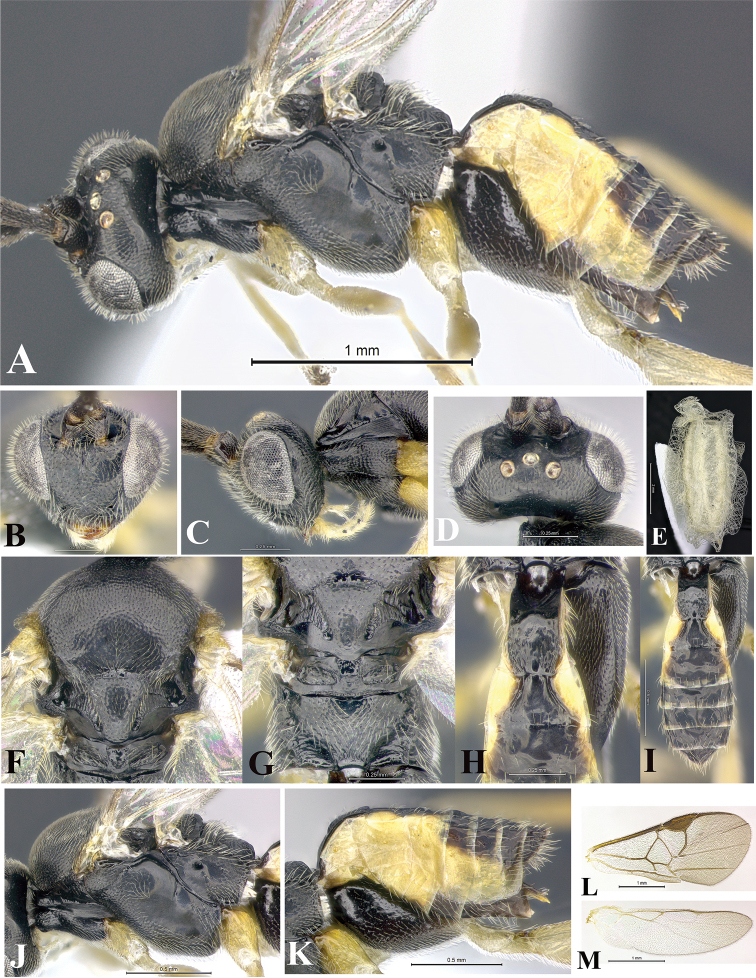
*Glyptapantelesrafamanitioi* sp. nov. female EC-43493 YY-A138 **A** Habitus **B, D** Head **B** Frontal view **D** Dorsal view **C** Head, pronotum, propleuron, lateral view **E** Cocoon **F** Mesonotum, dorsal view **G** Scutellum, metanotum, propodeum, dorsal view **H**T1–3, dorsal view **I, K** Metasoma **I** Dorsal view **K** Lateral view **J** Mesosoma, lateral view **L, M** Wings **L** Fore **M** Hind.

Vertex in lateral view pointed or nearly so (Fig. 190C), dorsal groove on axillary trough of scutellum with carinae only proximally (Fig. 190F, G), distal antennal flagellomere longer than penultimate, mesoscutum punctation proximally distinct, but distally absent/dispersed (Fig. 190F), temple punctate, propodeum without median longitudinal carina (Fig. 190G), petiole on T1 virtually parallel-sided over most of length, but narrowing over distal 1/3, finely sculptured (Fig. 190H, I), fore wing with vein 1 cu-a straight, r vein curved, outer side of junction of r and 2RS veins not forming a stub (Fig. 190L), dorsal outer depression on hind coxa present, inner margin of eyes diverging slightly at antennal sockets (Fig. 190B), and lateral grooves delimiting the median area on T2 clearly defined and reaching the distal edge of T2 (Fig. 190H, I).

#### Coloration

(Fig. 190A–M). General body coloration polished black except scape and all antennal flagellomeres (on both sides) brown; pedicel brown, but distally with a brown-reddish ring; apex of mandibles and glossa brown-reddish; maxillary and labial palps light yellow-brown; tegulae brown. Eyes gray/silver and ocelli reddish (in preserved specimen). Fore and middle legs dark yellow except claws brown; hind legs dark yellow, except black coxae, femora distally with a brown dot, 2/3 distal of tibiae and tarsomeres brown, although basitarsus proximally with a yellow band. Petiole on T1 black and sublateral areas yellow; T2 with median area black, adjacent area very narrow with silhouette well-defined, and lateral ends yellow; T3 mostly brown and lateral ends narrow and yellow/yellow-brown; T4 and beyond completely brown; distally each tergum with a wide yellow-translucent band. In lateral view, T1–3 completely yellow; T3 and beyond yellow, but dorsally brown, extent of brown area remaining almost constant, although the most distal tergum completely brown. S1–3 yellow; S4 yellow, but ventrally brown; penultimate sternum and hypopygium brown.

#### Description.

**Head** (Fig. 190A–D). Head triangular with pubescence long and dense. Proximal three antennal flagellomeres longer than wide (0.27:0.08, 0.27:0.08, 0.28:0.08), distal antennal flagellomere longer than penultimate (0.15:0.08, 0.12:0.05), antenna longer than body (3.83, 2.92); antennal scrobes-frons shallow. Face with dense fine punctations, medially with lateral depression, interspaces smooth and longitudinal median carina present. Frons smooth. Temple wide, punctate and interspaces clearly smooth. Inner margin of eyes diverging slightly at antennal sockets; in lateral view, eye anteriorly convex and posteriorly straight. POL subequal in length with OOL (0.12, 0.12). Malar suture present. Median area between lateral ocelli slightly depressed. Vertex laterally pointed or nearly so and dorsally wide.

**Mesosoma** (Fig. 190A, F, G, J). Mesosoma dorsoventrally convex. Distal 1/3 of mesoscutum with lateral margin slightly dented, punctation proximally distinct, but distally absent/dispersed, interspaces wavy/lacunose. Scutellum triangular, apex sloped and fused with BS, but not in the same plane, scutellar punctation distinct throughout, in profile scutellum flat and on same plane as mesoscutum, phragma of the scutellum partially exposed; BS only very partially overlapping the MPM; ATS demilune with short stubs delineating the area; dorsal ATS groove with carinae only proximally. Transscutal articulation with small and heterogeneous foveae, area just behind transscutal articulation nearly at the same level as mesoscutum (flat) and with same kind of sculpture as mesoscutum. Metanotum with BM convex; AFM with a small lobe and not as well delineated as PFM; PFM thick, smooth and with lateral ends rounded; ATM proximally with a groove with some sculpturing and distally smooth. Propodeum without median longitudinal carina, proximal half curved with medium-sized sculpture and distal half relatively polished and with a shallow dent at each side of nucha; distal edge of propodeum with a flange at each side and without stubs; propodeal spiracle distally framed by a short transverse carina; nucha surrounded by very short radiating carinae. Pronotum with a distinct dorsal furrow, dorsally with a well-defined smooth band; central area of pronotum and dorsal furrow smooth, but ventral furrow with short parallel carinae. Propleuron finely sculptured only ventrally and dorsally without a carina. Metasternum convex. Contour of mesopleuron convex; precoxal groove smooth, shiny and shallow, but visible; epicnemial ridge convex, teardrop-shaped.

**Legs.** Ventral margin of fore telotarsus slightly excavated and with a tiny curved seta, fore telotarsus almost same width throughout and longer than fourth tarsomere (0.15, 0.08). Hind coxa with dorsal half sparsely punctate, ventral half densely punctate, and dorsal outer depression present. Inner spur of hind tibia longer than outer spur (0.46, 0.30), entire surface of hind tibia with dense strong spines clearly differentiated by color and length. Hind telotarsus as equal in length as fourth tarsomere (0.15, 0.15).

**Wings** (Fig. 190L, M). Fore wing with r vein curved; 2RS vein straight; r and 2RS veins forming a weak, even curve at their junction and outer side of junction not forming a stub; 2M vein slightly curved/swollen; distally fore wing [where spectral veins are] with microtrichiae more densely concentrated than the rest of the wing; anal cell 1/3 proximally lacking microtrichiae; subbasal cell with microtrichiae virtually throughout; veins 2CUa and 2CUb completely spectral; vein 2 cu-a present as spectral vein, sometimes difficult to see; vein 2-1A proximally tubular and distally spectral, although sometimes difficult to see; tubular vein 1 cu-a straight, incomplete/broken and not reaching the edge of 1-1A vein. Hind wing with vannal lobe narrow, subdistally and subproximally straightened, and setae evenly scattered in the margin.

**Metasoma** (Fig. 190A, H, I, K). Metasoma laterally compressed. Petiole on T1 finely sculptured distal, but only laterally, virtually parallel-sided over most of length, but barely narrowing at apex, apex truncate (length 0.45, maximum width 0.20, minimum width 0.17), and with scattered pubescence concentrated in the first distal third. Lateral grooves delimiting the median area on T2 clearly defined and reaching the distal edge of T2 (length median area 0.18, length T2 0.18), edges of median area polished and lateral grooves deep, median area broader than long (length 0.18, maximum width 0.30, minimum width 0.12); T2 with scattered pubescence only distally. T3 longer than T2 (0.25, 0.18) and with pubescence more notorious in distal half. Pubescence on hypopygium dense.

**Cocoon** (Fig. 190E). White lace-shaped cocoon with evenly smooth silk fibers.

#### Comments.

The edges of the median area on T2 are not straight throughout, thus 1/3 proximally are straight, but afterwards are curved (Fig. 190H), the propodeal spiracle distally are framed with a short carina touching the pleural carina, the ventral furrow of the pronotum with parallel costate (Fig. 190C), most of the metepimeron with punctate sculpture, and the body is stout (Fig. 190A).

#### Male.

Unknown

#### Etymology.

Rafael (Rafa) Bolivar Manitio García is one of the Ecuadorian gusaneros who assisted with caterpillar rearing at Yanayacu Biological Station, Ecuador.

#### Distribution.

Parasitized caterpillar was collected in Ecuador, Napo, Yanayacu Biological Station (Sendero Culo del Mundo), during November 2009 at 2,352 m in cloud forest.

#### Biology.

The lifestyle of this parasitoid species is solitary.

#### Host.

Undetermined species of Noctuidae feeding on *Chusqueascandens* (Poaceae). Caterpillar was collected in second instar.

### Glyptapanteles robbinthorpi

Taxon classificationAnimaliaHymenopteraBraconidae

Arias-Penna, sp. nov.

http://zoobank.org/9617FE68-0117-4C2A-BC5A-9BF074A17CCB

Figs 191 , 192

#### Female.

Body length 2.32 mm, antenna length 2.53 mm, fore wing length 2.58 mm.

#### Type material.

**Holotype**: COSTA RICA • 1♀; 07-SRNP-4145, DHJPAR0020730; Área de Conservación Guanacaste, Alajuela, Sector San Cristóbal, Finca San Gabriel; rain forest; 645 m; 10.87766, -85.39343; 22.x.2007; Carolina Cano leg.; caterpillar collected in fifth instar; cocoons adhered to the larval cuticle and formed on 24.x.2007; adult parasitoids emerged on 03.xi.2007; (CNC). **Paratypes.** • 84 (4♀, 3♂) (75♀, 2♂); 07-SRNP-4145, DHJPAR0020730; same data as for holotype; (CNC).

#### Other material.

**Reared material.** COSTA RICA: *Área de Conservación Guanacaste*, *Alajuela*, *Sector San Cristóbal*, *Sendero Huerta*: • 60 (5♀, 5♂) (50♀, 0♂); 07-SRNP-3768, DHJPAR0020716; rain forest; 527 m; 10.9305, -85.37223; 15.ix.2007; Gloria Sihezar leg.; caterpillar collected in third instar; cordwood cocoons adhered to the leaf substrate; adult parasitoid emerged on 04.x.2007.

#### Diagnosis.

**Figure 191.**
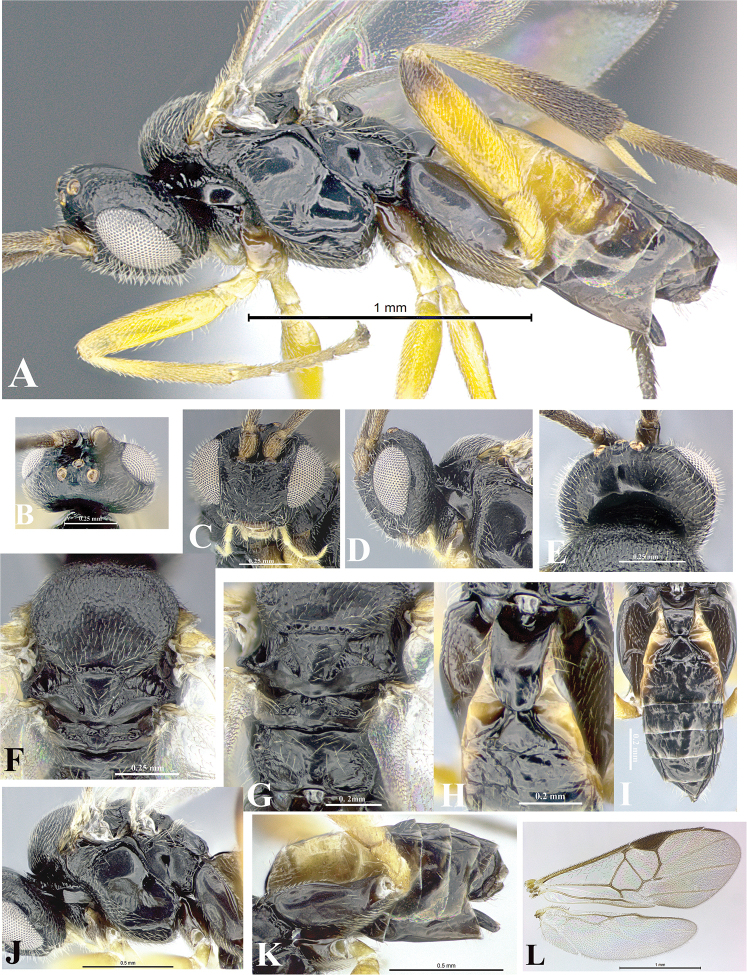
*Glyptapantelesrobbinthorpi* sp. nov. female 07-SRNP-4145 DHJPAR0020730 **A** Habitus **B, C, E** Head **B, E** Dorsal view **C** Frontal view **D** Head, pronotum, propleuron, lateral view **F** Mesonotum, dorsal view **G** Scutellum, metanotum, propodeum, dorsal view **H**T1–3, dorsal view **I, K** Metasoma **I** Dorsal view **K** Lateral view **J** Mesosoma, lateral view **L** Fore and hind wings.

**Figure 192.**
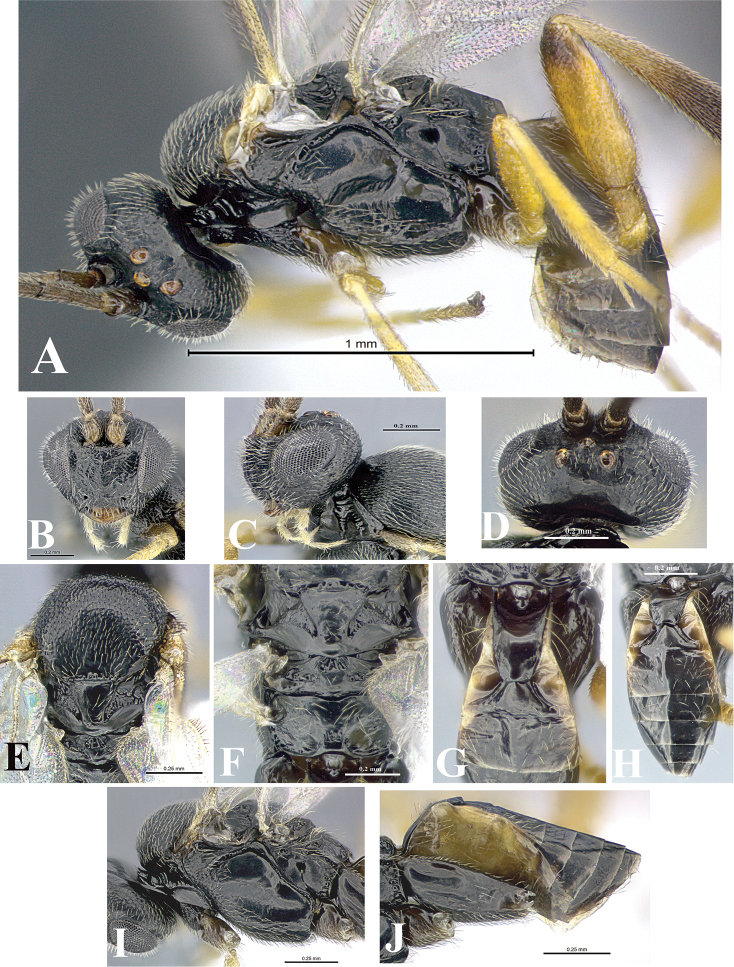
*Glyptapantelesrobbinthorpi* sp. nov. male 07-SRNP-4145 DHJPAR0020730 **A** Habitus **B–D** Head **B** Frontal view **C** Lateral view **D** Dorsal view **E** Mesonotum, dorsal view **F** Scutellum, metanotum, propodeum, dorsal view **G**T1–3, dorsal view **H, J** Metasoma **H** Dorsal view **J** Lateral view **I** Mesosoma, lateral view.

In lateral view metasoma curved (Figs 191K, 192A), fore wing with 1 cu-a vein curved, incomplete/broken, not reaching the edge of 1-1A vein, r vein curved, outer side of junction of r and 2RS veins forming a stub (Fig. 191L), inner margin of eyes diverging slightly at antennal sockets (Figs 191C, 192B), scutellum in profile convex and slightly higher than mesoscutum (Figs 191J, 192I), precoxal groove with faintly lineate sculpture (Figs 191J, 192I), dorsal carina delimiting a dorsal furrow on propleuron absent, petiole on T1 parallel-sided in proximal half then narrowing (Figs 191H, 192G), precoxal groove deep (Figs 191J, 192I), anteroventral contour of mesopleuron straight/angulate or nearly so (Figs 191J, 192I), and dges of median area on T2 polished and followed by a deep groove (Figs 191H, 192G).

#### Coloration

(Fig. 191A–L). General body coloration shiny, satin black except pedicel yellow-brown; scape yellow-brown distally with a ring brown; three-four most proximal antennal flagellomeres dorsally lighter (light brown) than ventrally (dark brown), remaining flagellomeres dark brown on both sides; labrum, mandible, glossa, and tegulae yellow-brown; maxillary and labial palps ivory/pale yellow. Eyes silver and ocelli reddish (in preserved specimen). Fore and middle legs yellow except coxae brown (inner side almost completely yellow) and claws brown; hind legs yellow except coxae black (inner side yellow-brown), femora with apex brown, distal half of tibiae and tarsomeres brown, although basitarsus proximally with a narrow yellow band. Petiole on T1 black with a reddish tint in the middle, contours darkened and sublateral areas yellow-brown; T2 with median and wide adjacent areas brown, and lateral ends yellow-brown; T3 broadly brown and narrow lateral ends yellow-brown; T4 and beyond brown; distally each tergum with a narrow whitish transparent band. In lateral view, T1–3 yellow-brown; T4 yellow, but dorsally brown; T5 and beyond brown. S1–3 yellow-brown; S4 proximal half yellow, distal half brown; penultimate sternum and hypopygium brown.

#### Description.

**Head** (Fig. 191A–D). Head rounded with pubescence long and dense. Proximal three antennal flagellomeres longer than wide (0.19:0.05, 0.19:0.05, 0.19:0.05), distal antennal flagellomere longer than penultimate (0.11:0.05, 0.09:0.05), antenna longer than body (2.53, 2.32); antennal scrobes-frons shallow. Face with slightly rugose sculpture, with depression only laterally, interspaces wavy and longitudinal median carina present. Frons smooth. Temple wide, punctate-lacunose and interspaces wavy. Inner margin of eyes diverging slightly at antennal sockets; in lateral view, eye anteriorly convex and posteriorly straight. POL shorter than OOL (0.09, 013). Malar suture absent or difficult to see. Median area between lateral ocelli slightly depressed. Vertex laterally rounded and dorsally wide.

**Mesosoma** (Fig. 191A, F, G, J). Mesosoma dorsoventrally convex. Mesoscutum proximally convex and distally flat, punctation distinct throughout, interspaces wavy/lacunose. Scutellum triangular, apex sloped and fused with BS, scutellar punctation scattered throughout, in profile scutellum convex and slightly higher than mesoscutum, phragma of the scutellum partially exposed; BS mostly overlapping the MPM; ATS demilune with short stubs delineating the area; dorsal ATS groove with carinae only proximally. Transscutal articulation with small and heterogeneous foveae, area just behind transscutal articulation sloped, smooth and shiny. Metanotum with BM wider than PFM (clearly differentiated); MPM circular with some sculpture inside; AFM without setiferous lobes and not as well delineated as PFM; PFM thick, smooth and with lateral ends rounded; ATM proximally with a well-defined row of foveae and distally smooth. Propodeum relatively polished without median longitudinal carina, proximal half curved; distal edge of propodeum with a flange at each side and without stubs; propodeal spiracle distally framed by faintly concave/wavy carina; nucha surrounded by very short radiating carinae. Pronotum with a distinct dorsal furrow, dorsally with a well-defined smooth band; central area of pronotum smooth, but both dorsal and ventral furrows with short parallel carinae. Propleuron with a mix of rugae and fine punctation, dorsally without a carina. Metasternum flat or nearly so. Contour of mesopleuron straight/angulate or nearly so; precoxal groove deep and with faintly lineate sculpture; epicnemial ridge convex, teardrop-shaped.

**Legs.** Ventral margin of fore telotarsus excavated with conspicuous curved seta over this excavation, fore telotarsus almost same width throughout and longer than fourth tarsomere (0.13, 0.07). Hind coxa with dorsal half sparsely punctate, ventral half densely punctate, and dorsal outer depression present. Inner spur of hind tibia longer than outer spur (0.25, 0.20), entire surface of hind tibia with dense strong spines clearly differentiated by color and length. Hind telotarsus as equal in length as fourth tarsomere (0.11, 0.10).

**Wings** (Fig. 191L). Fore wing with r vein slightly curved; 2RS vein straight; r and 2RS veins forming an angle at their junction and outer side of junction forming a slight stub; 2M vein straight; distally fore wing [where spectral veins are] with microtrichiae more densely concentrated than the rest of the wing; anal cell 1/3 proximally lacking microtrichiae; subbasal cell with a small smooth area; vein 2CUa absent and vein 2CUb spectral; vein 2 cu-a absent; vein 2-1A present only proximally as tubular vein; tubular vein 1 cu-a curved, incomplete/broken and not reaching the edge of 1-1A vein. Hind wing with vannal lobe narrow, subdistally and subproximally straightened, and setae present proximally, but absent distally.

**Metasoma** (Fig. 191A, H, I, K). Metasoma curved. Petiole on T1 completely smooth and polished, with faint, satin-like sheen, parallel-sided in proximal half and then narrowing (length 0.32, maximum width 0.17, minimum width 0.08), and with scattered pubescence concentrated in the first distal third. Lateral grooves delimiting the median area on T2 distally losing definition (length median area 0.11, length T2 0.14), edges of median area polished and lateral grooves deep, median area broader than long (length 0.11, maximum width 0.27, minimum width 0.06); T2 with scattered pubescence only distally. T3 longer than T2 (0.21, 0.14) and with scattered pubescence throughout. Pubescence on hypopygium scattered.

**Cocoons.** Light brown oval cocoons with silk fibers ordered, but covered by a net. Cordwood cocoons adhered to the leaf substrate.

#### Comments.

The body is elongated, cylindrical, and curved (Fig. 191A).

#### Male

(Fig. 192A–J). The body shape is similar to female.

#### Etymology.

Robbin W. Thorp is a professor emeritus at the University of California, Davis (UC), CA, USA. His research has been focused on bee biology: pollination ecology, foraging behavior, management of bee populations, and the systematics and ecology of bees.

#### Distribution.

The parasitized caterpillars were collected in Costa Rica, ACG, Sector San Cristóbal (Finca San Gabriel and Sendero Huerta), during September–October 2007 at 527m and 645 m in rain forest.

#### Biology.

The lifestyle of this parasitoid species is gregarious.

#### Host.

*Letismycerina* (Cramer) (Erebidae: Erebiinae) feeding on *Ingaoerstediana* and *I.punctata* (Fabaceae). Caterpillars were collected in third and fifth instar.

### Glyptapanteles ronaldzunigai

Taxon classificationAnimaliaHymenopteraBraconidae

Arias-Penna, sp. nov.

http://zoobank.org/C456F004-2739-4B04-B683-624CF92EB6FD

Figs 193 , 194

#### Female.

Body length 1.86 mm, antenna length 2.07 mm, fore wing length 2.22 mm.

#### Type material.

**Holotype**: COSTA RICA • 1♀; 08-SRNP-55003, DHJPAR0020565; Área de Conservación Guanacaste, Guanacaste, Sector Mundo Nuevo, Vado Ficus; dry-rain intergrade forest; 375 m; 10.77090, -85.42455; 04.i.2008; Mariano Pereira leg.; caterpillar collected in fourth instar; very small dark cocoons adhered lightly to cuticle, jumbled, cocoons formed on 14.i.2008; adult parasitoids emerged on 18.i.2008; (CNC). **Paratypes.** • 22 (2♀, 2♂) (12♀, 6♂); 08-SRNP-55003, DHJPAR0020565; same data as for holotype; (CNC).

#### Diagnosis.

**Figure 193.**
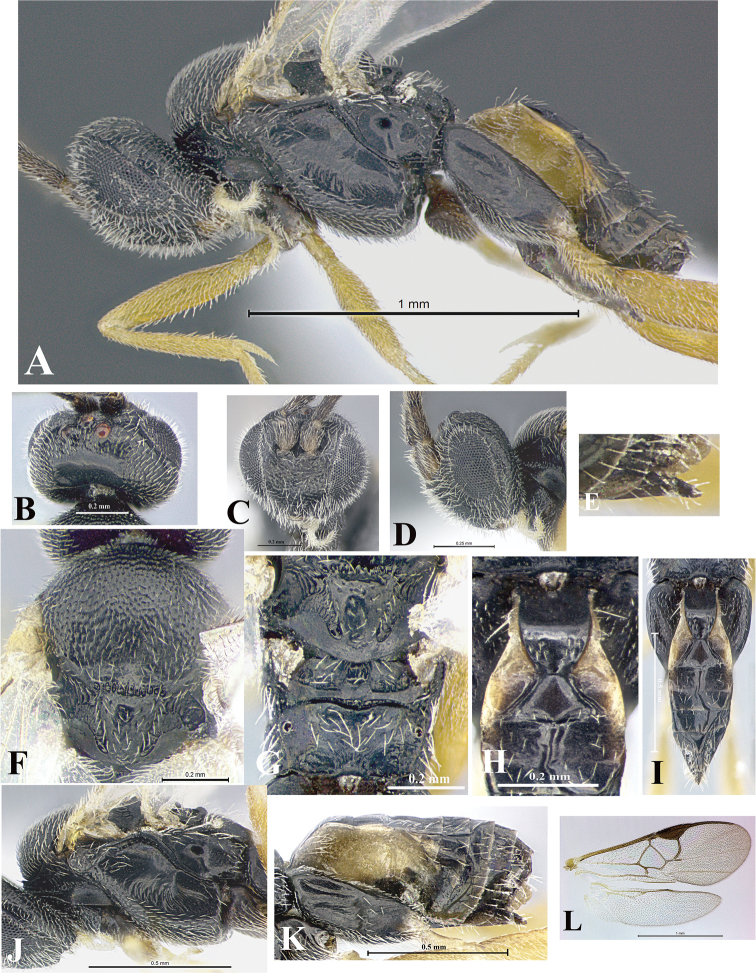
*Glyptapantelesronaldzunigai* sp. nov. female 08-SRNP-55003 DHJPAR0020565 **A** Habitus **B–D** Head **B** Dorsal view **C** Frontal view **D** Lateral view **E** Genitalia: hypopygium, ovipositor, ovipositor sheaths, lateral view **F** Mesonotum, dorsal view **G** Scutellum, metanotum, propodeum, dorsal view **H**T1–3, dorsal view **I, K** Metasoma **I** Dorsal view **K** Lateral view **J** Mesosoma, lateral view **L** Fore and hind wings.

**Figure 194.**
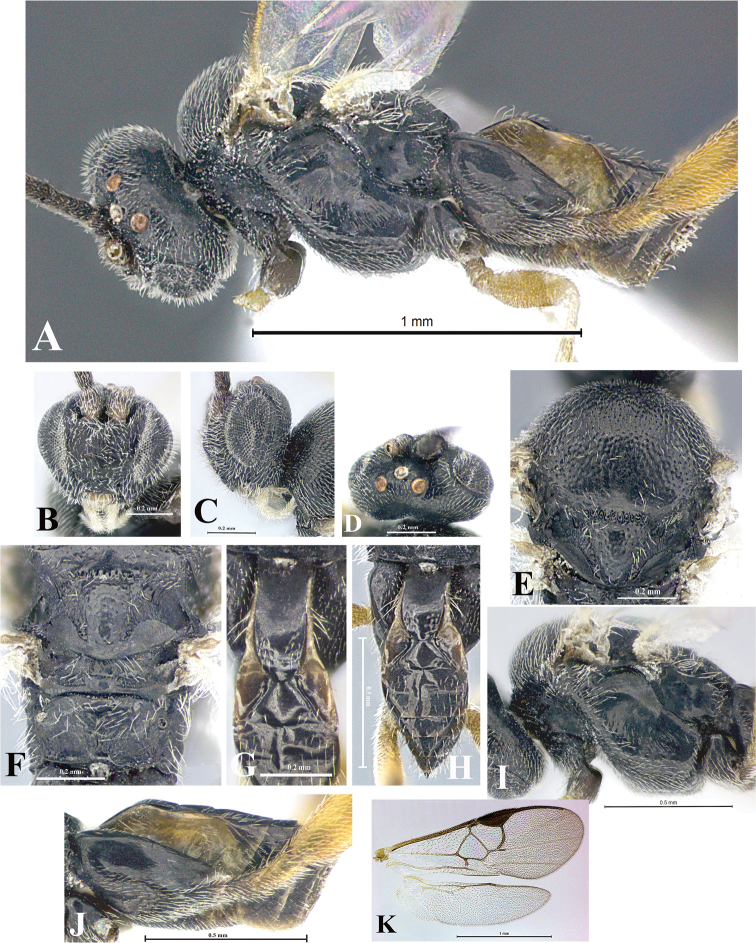
*Glyptapantelesronaldzunigai* sp. nov. male 08-SRNP-55003 DHJPAR0020565 **A** Habitus **B–D** Head **B** Frontal view **C** Lateral view **D** Dorsal view **E** Mesonotum, dorsal view **F** Scutellum, metanotum, propodeum, dorsal view **G**T1–3, dorsal view **H, J** Metasoma **H** Dorsal view **J** Lateral view **I** Mesosoma, lateral view **K** Fore and hind wings.

Dorsal carina delimiting a dorsal furrow on propleuron absent (Figs 193J, 194I), surface of metasternum convex, precoxal groove deep with lineate sculpture (Figs 193A, J, 194A, I), fore wing with vein 1 cu-a curved, r vein curved (Figs 193L, 194K), dorsal outer depression on hind coxa present (Figs 193A, K, 194A, J), inner margin of eyes diverging slightly at antennal sockets (Figs 193C, 194B), petiole on T1 finely sculptured (Figs 193H, 194G, H), and lateral grooves delimiting the median area on T2 clearly defined and reaching the distal edge of T2 (Figs 193H, I, 194G, H).

#### Coloration

(Fig. 193A–L). General body coloration black except yellow-brown scape distally brown; brown pedicel distally yellow-brown; all antennal flagellomeres dark brown on both sides; labrum, mandibles, and tegulae yellow-brown; glossa, maxillary and labial palps ivory/pale yellow. Eyes black and ocelli silver/reddish (in preserved specimen). Fore and middle legs yellow except brown coxae and claws; hind legs yellow-brown except black coxae distally yellow-brown, femora distally brown, distal half of tibiae, and tarsomeres brown, although proximally basitarsus with a small yellow band. Petiole on T1 dark brown, contours darkened and sublateral areas yellow-brown; T2 with median and adjacent areas brown, together forming a rectangle well-defined, and lateral ends yellow; T3 almost completely brown; T4 and beyond completely brown; distally each tergum with a whitish transparent band. In lateral view, T1–3 yellow; T4 and beyond brown. S1–3 yellow; S4 and beyond brown.

#### Description.

**Head** (Fig. 193A–D). Head rounded with pubescence long and dense. Proximal three antennal flagellomeres longer than wide (0.17:0.06, 0.17:0.06, 0.16:0.06), distal antennal flagellomere longer than penultimate (0.10:0.05, 0.08:0.05), antenna longer than body (2.07, 1.86); antennal scrobes-frons shallow. Face flat or nearly so, with dense and finely punctate, interspaces smooth and longitudinal median carina present. Frons smooth. Temple wide, punctate-lacunose and interspaces wavy. Inner margin of eyes diverging slightly at antennal sockets; in lateral view, eye anteriorly convex and posteriorly straight. POL shorter than OOL (0.09, 0.13). Malar suture absent or difficult to see. Median area between lateral ocelli slightly depressed. Vertex laterally rounded and dorsally wide.

**Mesosoma** (Fig. 193A, F, G, J). Mesosoma dorsoventrally convex. Mesoscutum 1/4 distal with a central dent, punctation distinct throughout, interspaces smooth. Scutellum triangular, apex sloped and fused with BS, scutellar punctation distinct throughout, in profile scutellum slightly convex, but on same plane as mesoscutum, phragma of the scutellum partially exposed; BS only very partially overlapping the MPM; ATS demilune with complete undulate/reticulate carinae; dorsal ATS groove with carinae only proximally. Transscutal articulation with small and heterogeneous foveae; area just behind transscutal articulation depressed centrally, smooth and shiny. Metanotum with BM wider than PFM (clearly differentiated); MPM circular and bisected by a median longitudinal carina; AFM without setiferous lobes and not as well delineated as PFM; PFM thick, smooth and with lateral ends rounded; ATM proximally with a groove with some sculpturing and distally smooth. Propodeum without median longitudinal carina, proximal half curved with medium-sized sculpture and distal half slightly rugose; distal edge of propodeum with a flange at each side and without stubs; propodeal spiracle without distal carina; nucha surrounded by very short radiating carinae. Pronotum with a distinct dorsal furrow, dorsally with a well-defined smooth band; central area of pronotum smooth, but both dorsal and ventral furrows with short parallel carinae. Propleuron rugose and dorsally without a carina. Metasternum convex. Contour of mesopleuron convex; precoxal groove deep and with transverse lineate sculpture; epicnemial ridge convex, teardrop-shaped.

**Legs.** Ventral margin of fore telotarsus excavated with conspicuous curved seta over this excavation, fore telotarsus almost same width throughout and longer than fourth tarsomere (0.11, 0.05). Hind coxa with punctation only on ventral surface, dorsal outer depression present. Inner spur of hind tibia longer than outer spur (0.21, 0.16), entire surface of hind tibia with dense strong spines clearly differentiated by color and length. Hind telotarsus longer than fourth tarsomere (0.12, 0.10).

**Wings** (Fig. 193L). Fore wing with r vein curved; 2RS vein straight; r and 2RS veins forming a weak, even curve at their junction and outer side of junction not forming a stub; 2M vein straight, distally fore wing [where spectral veins are] with microtrichiae more densely concentrated than the rest of the wing; anal cell 1/3 proximally lacking microtrichiae; subbasal cell with a small smooth area; vein 2CUa absent and vein 2CUb spectral; vein 2 cu-a absent; vein 2-1A present only proximally as tubular vein; tubular vein 1 cu-a curved and complete, but junction with 1-1A vein spectral. Hind wing with vannal lobe wide, subdistally concave and subproximally straightened, and setae evenly scattered in the margin.

**Metasoma** (Fig. 193A, E, H, I, K). Metasoma laterally compressed. Petiole on T1 finely sculptured only laterally, virtually parallel-sided over most of length, but narrowing over distal 1/3 (length 0.29, maximum width 0.14, minimum width 0.07), and with scattered pubescence concentrated in the first distal third. Lateral grooves delimiting the median area on T2 clearly defined and reaching the distal edge of T2 (length median area 0.11, length T2 0.11), edges of median area polished and lateral grooves deep, median area broader than long (length 0.11, maximum width 0.17, minimum width 0.06); T2 with scattered pubescence only distally. T3 longer than T2 (0.19, 0.11) and with scattered pubescence throughout. Pubescence on hypopygium scattered.

**Cocoons.** Oval cocoons with silk fibers evenly smooth. Untidy cocoons adhered lightly to cuticle.

**Comment.** Specimens short and stout (Fig. 193A).

#### Male

(Fig. 194A–K). Similar in coloration and shape to female.

#### Etymology.

Ronald Zuñiga is a specialist in Hymenoptera who works at the Instituto Nacional de Biodiversidad (INBio), Santo Domingo de Heredia, Costa Rica.

#### Distribution.

The parasitized caterpillar was collected in Costa Rica, ACG, Sector Mundo Nuevo (Vado Ficus), during January 2008 at 375 m in dry-rain intergrade forest.

#### Biology.

The lifestyle of this parasitoid species is gregarious.

#### Host.

*Macarianundinata* Guenée (Geometridae: Ennominae) feeding on *Daleacarthagenensis* (Fabaceae). Caterpillar was collected in fourth instar.

### Glyptapanteles roysnellingi

Taxon classificationAnimaliaHymenopteraBraconidae

Arias-Penna, sp. nov.

http://zoobank.org/25F6B245-5815-4A0A-8726-D2AE7FF891BA

Figs 195 , 196

#### Female.

Body length 2.22 mm, antenna length 2.46 mm, fore wing length 2.27 mm.

#### Type material.

**Holotype**: COSTA RICA • 1♀; 08-SRNP-58202, DHJPAR0034197; Área de Conservación Guanacaste, Guanacaste, Sector Nuevo Mundo, Camino Pozo Dos; dry-rain intergrade forest; 728 m; 10.77111, -85.3607; 09.xi.2008; Daniel M. Acuña leg.; caterpillar collected in fifth instar; cocoons adhered to the larval cuticle and formed on 11.xi.2008; adult parasitoids emerged on 12.xi.2008; (CNC). **Paratypes.** • 18 (3♀, 4♂) (0♀, 11♂); 08-SRNP-58202, DHJPAR0034197; same data as for holotype; (CNC).

#### Diagnosis.

**Figure 195.**
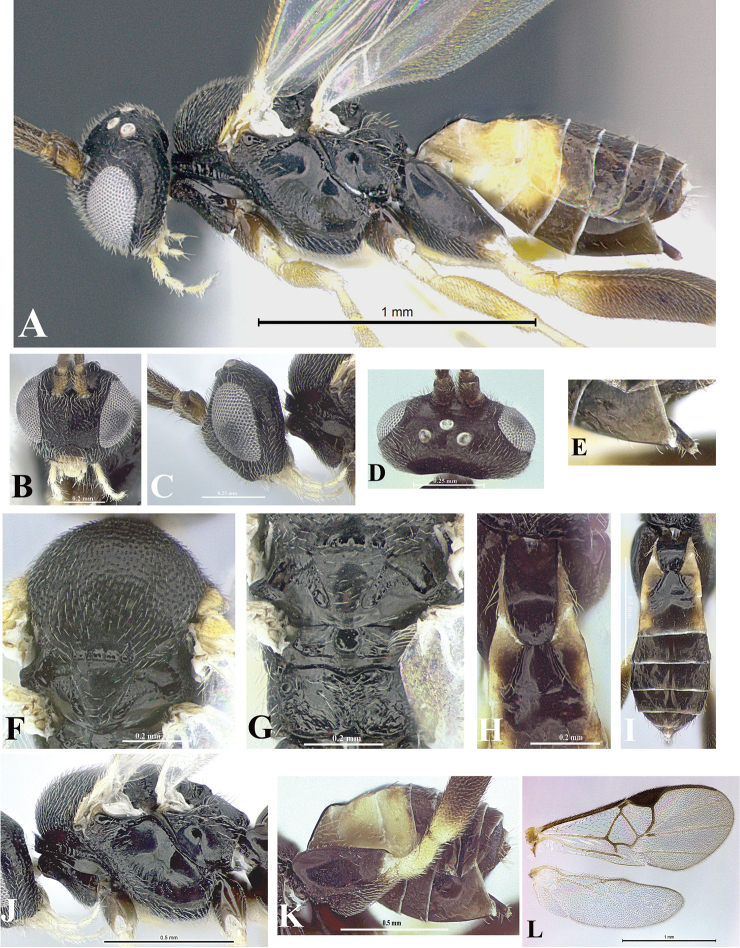
*Glyptapantelesroysnellingi* sp. nov. female 08-SRNP-58202 DHJPAR0034197 **A** Habitus **B, D** Head **B** Frontal view **D** Dorsal view **C** Head, pronotum, propleuron, lateral view **E** Genitalia: hypopygium, ovipositor, ovipositor sheaths, lateral view **F** Mesonotum, dorsal view **G** Scutellum, metanotum, propodeum, dorsal view **H**T1–3, dorsal view **I, K** Metasoma **I** Dorsal view **K** Lateral view **J** Mesosoma, lateral view **L** Fore and hind wings.

**Figure 196.**
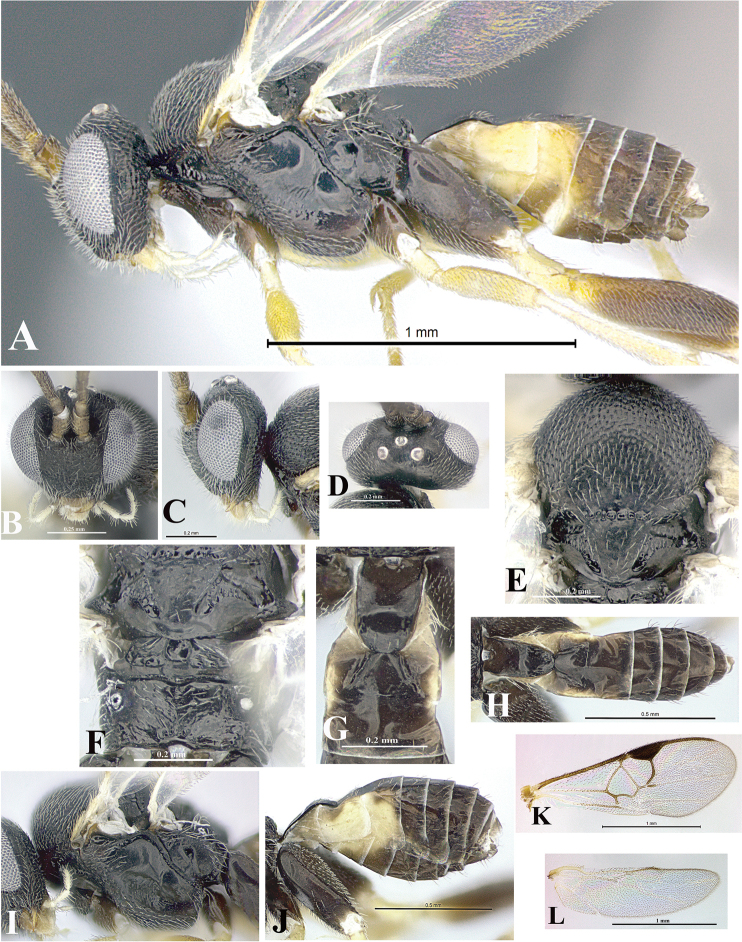
*Glyptapantelesroysnellingi* sp. nov. male 08-SRNP-58202 DHJPAR0034197 **A** Habitus **B, D** Head **B** Frontal view **D** Dorsal view **C** Head, pronotum, propleuron, lateral view **E** Mesonotum, dorsal view **F** Scutellum, metanotum, propodeum, dorsal view **G**T1–3, dorsal view **H, J** Metasoma **H** Dorsal view **J** Lateral view **I** Mesosoma, lateral view **K, L** Wings **K** Fore **L** Hind.

Ventral margin of fore telotarsus excavated with conspicuous curved seta over this excavation, almost same width throughout, mesoscutum distinctly punctate throughout (Figs 195F, 196E), fore wing with vein 2-1A present only proximally as tubular vein, vein 1 cu-a curved, r vein curved, outer side of junction of r and 2RS veins not forming a stub (Figs 195L, 196K), medioposterior band of scutellum only very partially overlapping the medioanterior pit of metanotum (Figs 195G, 196F), petiole on T1 finely sculptured nly laterally, distally with lateral margins convex (Figs 195H, I, 196G, H), surface of metasternum flat or nearly so, precoxal groove deep with lineate sculpture (Figs 195A, J, 196A, I), dorsal outer depression on hind coxa present (Figs 195A, K, 196A, J), inner margin of eyes diverging slightly at antennal sockets (Figs 195B, 196B), propodeum without median longitudinal carina (Figs 195G, 196F), and lateral grooves delimiting the median area on T2 clearly defined and reaching the distal edge of T2 (Figs 195H, I, 196G, H).

#### Coloration

(Fig. 195A–L). General body coloration black except scape and pedicel yellow-brown and outer edges both with a lateral brown band; first five-six proximal antennal flagellomeres dorsally lighter (light brown) than ventrally (dark brown), remaining flagellomeres dark brown; labrum, mandibles, and tegulae yellow-brown; glossa, maxillary and labial palps ivory/pale yellow. Eyes and ocelli silver. Fore and middle legs yellow except light brown coxae and claws brown; hind legs yellow except black coxae only distally yellow-brown (coloration is more extensive on the inner side), femora yellow-brown/brown only distally yellow, 2/3 distal of tibiae and tarsomeres brown, although proximally basitarsus with a small yellow band. Petiole on T1 brown, but proximal half lighter than distal half, contours darkened and sublateral areas yellow-brown; T2 with median and adjacent areas brown together forming a rectangle-shaped area, and lateral ends yellow-brown; T3 medially brown forming a dark area coinciding with the width of median and adjacent areas of T2, and lateral ends yellow-brown; T4 and beyond brown; distally each tergum with a narrow hyaline band. In lateral view, T1–3 yellow; T4 and beyond brown. S1–3 yellow; S4 yellow, but medially brown; penultimate sternum and hypopygium brown.

#### Description.

**Head** (Fig. 195A–D). Head rounded with pubescence long and dense. Proximal three antennal flagellomeres longer than wide (0.15:0.07, 0.16:0.07, 0.16:0.070), distal antennal flagellomere longer than penultimate (0.11:0.04, 0.08:0.04), antenna longer than body (2.46, 2.22); antennal scrobes-frons shallow. Face convex with dense fine punctations, interspaces smooth and longitudinal median carina present. Frons smooth. Temple wide, punctate and interspaces clearly smooth. Inner margin of eyes diverging slightly at antennal sockets; in lateral view, eye anteriorly convex and posteriorly straight. POL shorter than OOL (0.95, 0.11). Malar suture present. Median area between lateral ocelli slightly depressed. Vertex laterally pointed or nearly so and dorsally wide.

**Mesosoma** (Fig. 195A, F, G, J). Mesosoma dorsoventrally convex. Mesoscutum distal half with a central dent, punctation distinct throughout, interspaces smooth. Scutellum triangular, apex sloped and fused with BS, scutellar punctation distinct peripherally, absent centrally, in profile scutellum flat and on same plane as mesoscutum, phragma of the scutellum partially exposed; BS only very partially overlapping the MPM; ATS demilune with short stubs delineating the area; dorsal ATS groove smooth. Transscutal articulation with large and heterogeneous foveae, area just behind transscutal articulation smooth, shiny and depressed centrally. Metanotum with BM wider than PFM (clearly differentiated); MPM circular without median longitudinal carina; AFM without setiferous lobes and not as well delineated as PFM; PFM thick, smooth and with lateral ends rounded; ATM proximally with a groove with some sculpturing and distally smooth. Propodeum without median longitudinal carina, proximal half curved with medium-sized sculpture and distal half with a shallow dent at each side of nucha; distal edge of propodeum with a flange at each side and without stubs; propodeal spiracle distally framed by faintly concave/wavy carina; nucha surrounded by very short radiating carinae. Pronotum with a distinct dorsal furrow, dorsally with a well-defined smooth band; central area of pronotum smooth, but both dorsal and ventral furrows with short parallel carinae. Propleuron with fine rugae and dorsally with a carina. Metasternum flat or nearly so. Contour of mesopleuron straight/angulate or nearly so; precoxal groove deep with transverse lineate sculpture; epicnemial ridge convex, teardrop-shaped.

**Legs.** Ventral margin of fore telotarsus excavated with conspicuous curved seta over this excavation, fore telotarsus almost same width throughout and longer than fourth tarsomere (0.11, 0.06). Hind coxa with punctation only on ventral surface, dorsal outer depression present. Inner spur of hind tibia longer than outer spur (0.21, 0.12), entire surface of hind tibia with dense strong spines clearly differentiated by color and length. Hind telotarsus longer than fourth tarsomere (0.12, 0.10).

**Wings** (Fig. 195L). Fore wing with r vein slightly curved; 2RS vein straight; r and 2RS veins forming a weak, even curve at their junction and outer side of junction not forming a stub; 2M vein slightly curved/swollen; distally fore wing [where spectral veins are] with microtrichiae more densely concentrated than the rest of the wing; anal cell 1/3 proximally lacking microtrichiae; subbasal cell with a small smooth area; vein 2CUa absent and vein 2CUb spectral; vein 2 cu-a absent; vein 2-1A present only proximally as tubular vein; tubular vein 1 cu-a curved, incomplete/broken and not reaching the edge of 1-1A vein. Hind wing with vannal lobe narrow, subdistally and subproximally straightened, and setae evenly scattered in the margin.

**Metasoma** (Fig. 195A, E, H, I, K). Metasoma laterally compressed. Petiole on T1 finely sculptured only laterally, virtually parallel-sided over most of length, but narrowing over distal 1/3 (length 0.28, maximum width 0.15, minimum width 0.09), and with scattered pubescence concentrated in the first distal third. Lateral grooves delimiting the median area on T2 clearly defined and reaching the distal edge of T2 (length median area 0.12, length T2 0.12), edges of median area polished and lateral grooves deep, median area broader than long (length 0.12, maximum width 0.17, minimum width 0.06); T2 with scattered pubescence only distally. T3 longer than T2 (0.19, 0.12) and with scattered pubescence only distally. Pubescence on hypopygium scattered.

**Cocoons.** Characteristics unknown. Cocoons adhered to the larval cuticle.

#### Comments.

The body is elongate and slim (Fig. 195A).

#### Male

(Fig. 196A–L). Similar in coloration and shape to female.

#### Etymology.

Roy R. Snelling (30 September 1934-21 April 2008) was an internationally renowned American entomologist who studied Hymenoptera, mainly ants, wasps, and bees. He dedicated his professional life to making insect biodiversity better known and appreciated.

#### Distribution.

The parasitized caterpillar was collected in Costa Rica, ACG, Sector Nuevo Mundo (Camino Pozo Dos), during November 2008 at 728 m in dry-rain intergrade forest.

#### Biology.

The lifestyle of this parasitoid species is gregarious.

#### Host.

Undetermined species of Geometridae feeding on *Bunchosiapolystachia* (Malpighiaceae). Caterpillar was collected in fifth instar.

### Glyptapanteles scottmilleri

Taxon classificationAnimaliaHymenopteraBraconidae

Arias-Penna, sp. nov.

http://zoobank.org/0747896E-35B4-42B8-AA7E-A997F915C3A1

Figs 197 , 198

#### Female.

Body length 2.12 mm, antenna length 2.48 mm, fore wing length 2.15 mm.

#### Type material.

**Holotype**: COSTA RICA • 1♀; 10-SRNP-72490, DHJPAR0040428; Área de Conservación Guanacaste, Guanacaste, Sector Pitilla, Estación Quica; rain forest; 470 m; 10.99697, -85.39666; 29.vii.2010; Ricardo Calero leg.; caterpillar collected in fifth instar; cocoons adhered to the leaf substrate and formed on 31.vii.2010; adult parasitoids emerged on 06.viii.2010; (CNC). **Paratypes.** • 6 (1♀, 2♂) (3♀, 0♂); 10-SRNP-72490, DHJPAR0040428; same data as for holotype; (CNC).

#### Diagnosis.

**Figure 197.**
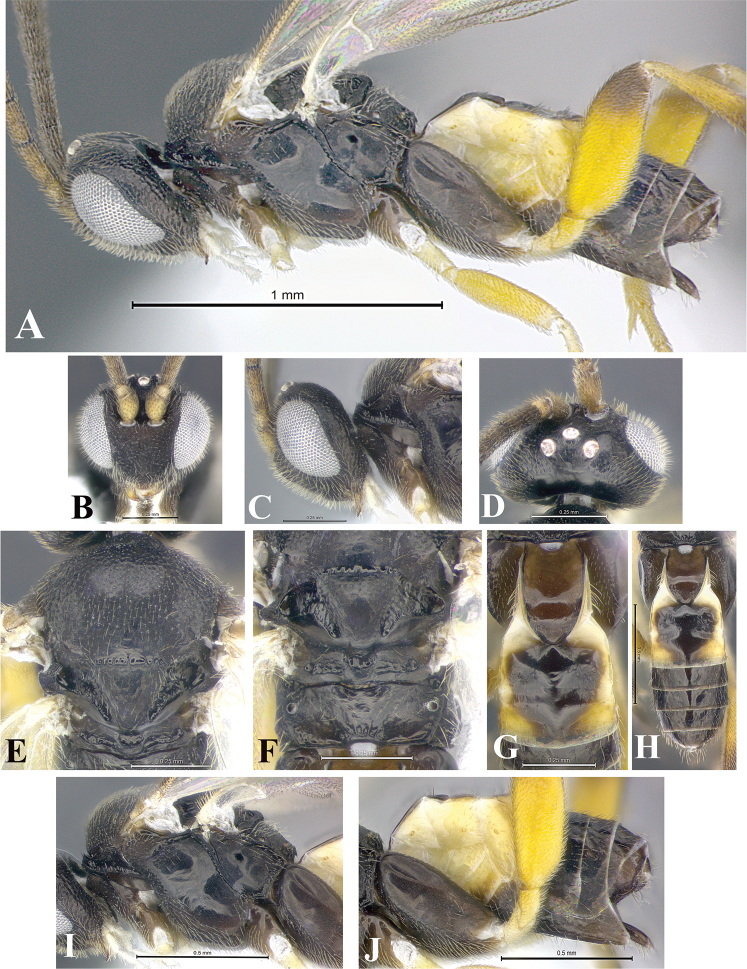
*Glyptapantelesscottmilleri* sp. nov. female 10-SRNP-72490 DHJPAR0040428 **A** Habitus **B, D** Head **B** Frontal view **D** Dorsal view **C** Head, pronotum, propleuron, lateral view **E** Mesonotum, dorsal view **F** Scutellum, metanotum, propodeum, dorsal view **G**T1–3, dorsal view **H, J** Metasoma **H** Dorsal view **J** Lateral view **I** Mesosoma, lateral view.

**Figure 198.**
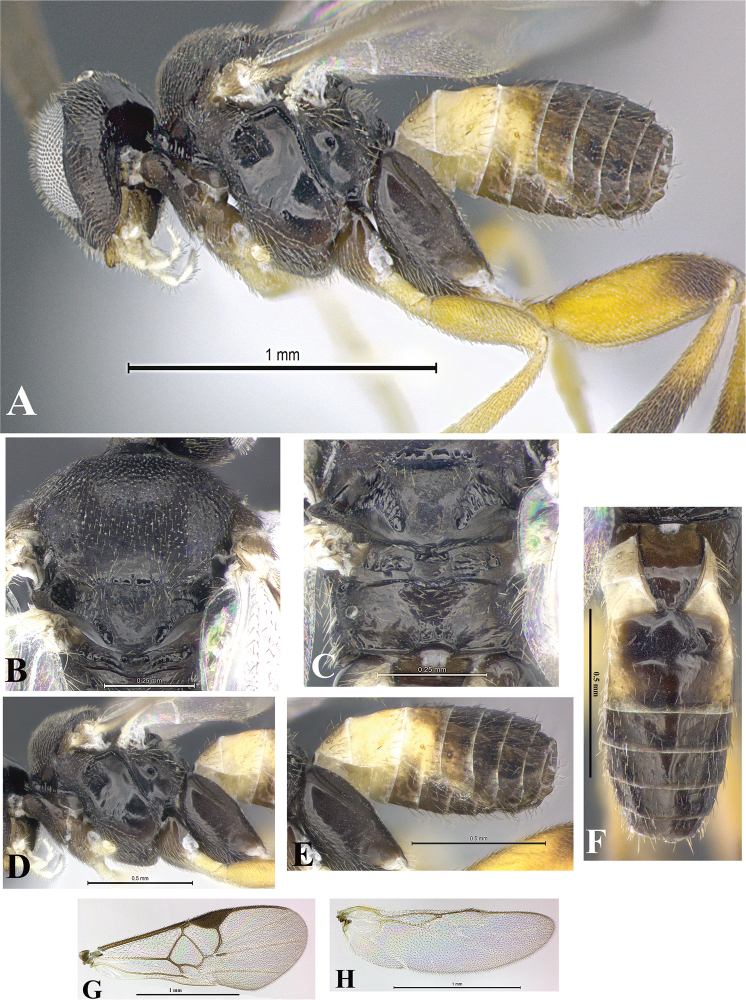
*Glyptapantelesscottmilleri* sp. nov. male 10-SRNP-72490 DHJPAR0040428 **A** Habitus **B** Mesonotum, dorsal view **C** Scutellum, metanotum, propodeum, dorsal view **D** Mesosoma, lateral view **E, F** Metasoma **E** Lateral view **F** Dorsal view **G, H** Wings **G** Fore **H** Hind.

Propleuron with fine punctation throughout, dorsal carina delimiting a dorsal furrow present (Figs 197C, I, 198A, D), distal antennal flagellomere longer than penultimate, mesoscutum proximally distinctly punctate, distally with a polished area (Figs 197E, 198B), median area on T2 broader than long, lateral grooves delimiting the median area distally losing definition (Figs 197G, H, 198F), propodeal spiracle distally framed by a short concave carina (Figs 197F, 198C), scutellum in profile convex and slightly higher than mesoscutum, and fore wing with 2RS convex, outer side of junction of r and 2RS veins not forming a stub (Fig. 198G).

#### Coloration

(Fig. 197A–J). General body coloration black except scape and pedicel yellow; first three proximal antennal flagellomeres dorsally lighter (light brown) than ventrally (dark brown), remaining flagellomeres dark brown on both sides; glossa, maxillary and labial palps pale yellow/ivory; tegulae light brown. Eyes and ocelli silver. Fore and middle legs yellow except light brown coxae and brown claws; hind legs brown except trochanters, trochantellus, proximal 2/3 of femora, distal 1/3 of tibiae, both tibial spurs and proximally basitarsus with a narrow yellow band. Petiole on T1 reddish/yellow-brown, contours darkened and sublateral areas yellow; T2 with median (sometimes hard to see without correct position of light) and adjacent areas brown both forming a rectangle-shaped area, and lateral ends yellow; T3 medially brown forming an inverted triangle which width coinciding proximally with dark area on T2, remaining area yellow; T4 and beyond brown; distally each tergum with a narrow hyaline band. In lateral view, T1–2 yellow; T3 yellow, but dorsodistal corners brown; T4 and beyond brown. S1–3 yellow; S4 proximal half yellow, distal half brown; penultimate sternum and hypopygium brown.

#### Description.

**Head** (Fig. 197A–D). Head rounded with pubescence long and dense. Proximal three antennal flagellomeres longer than wide (0.17:0.06, 0.18:0.06, 0.17:0.06), distal antennal flagellomere longer than penultimate (0.12:0.05, 0.09:0.05), antenna longer than body (2.48, 2.12); antennal scrobes-frons shallow. Face flat or nearly so, with dense fine punctations, interspaces smooth and longitudinal median carina present. Frons smooth. Temple wide, punctate and interspaces clearly smooth. Inner margin of eyes diverging slightly at antennal sockets; in lateral view, eye anteriorly convex and posteriorly straight. POL subequal in length with OOL (0.09, 0.10). Malar suture present. Median area between lateral ocelli slightly depressed. Vertex laterally pointed or nearly so and dorsally wide.

**Mesosoma** (Fig. 197A, E, F, I). Mesosoma dorsoventrally convex. Mesoscutum proximally convex and distally flat, punctation distinct proximally with polished area distally, interspaces smooth. Scutellum triangular, apex sloped and fused with BS, scutellar punctation distinct throughout, in profile scutellum convex and slightly higher than mesoscutum, phragma of the scutellum partially exposed; BS only very partially overlapping the MPM; ATS demilune with short stubs delineating the area; dorsal ATS groove smooth. Transscutal articulation with small and heterogeneous foveae, area just behind transscutal articulation nearly at the same level as mesoscutum (flat), smooth and shiny. Metanotum with BM upward; MPM circular without median longitudinal carina; AFM with a small lobe and not as well delineated as PFM; PFM thick, smooth and with lateral ends rounded; ATM proximally with a groove with some sculpturing and distally smooth. Propodeum relatively polished without median longitudinal carina, proximal half weakly curved; distal edge of propodeum with a flange at each side and without stubs; propodeal spiracle distally framed by a short concave carina; nucha surrounded by very short radiating carinae. Pronotum with a distinct dorsal furrow, dorsally with a well-defined smooth band; central area of pronotum smooth, but both dorsal and ventral furrows with short parallel carinae. Propleuron with fine punctations throughout and dorsally with a carina. Metasternum convex. Contour of mesopleuron convex; precoxal groove deep with faintly lineate sculpture; epicnemial ridge convex, teardrop-shaped.

**Legs.** Ventral margin of fore telotarsus slightly excavated and with a tiny curved seta, fore telotarsus almost same width throughout and longer than fourth tarsomere (0.10, 0.05). Hind coxa with punctation only on dorsal surface, dorsal outer depression present. Inner spur of hind tibia longer than outer spur (0.20, 0.15), entire surface of hind tibia with dense strong spines clearly differentiated by color and length. Hind telotarsus as equal in length as fourth tarsomere (0.10, 0.09).

**Wings** (Fig. 198G, H). Fore wing with r vein slightly curved; 2RS vein slightly convex to convex; r and 2RS veins forming a weak, even curve at their junction and outer side of junction not forming a stub; 2M vein slightly curved/swollen; distally fore wing [where spectral veins are] with microtrichiae more densely concentrated than the rest of the wing; anal cell 1/3 proximally lacking microtrichiae; subbasal cell with microtrichiae virtually throughout; vein 2CUa absent and vein 2CUb spectral; vein 2 cu-a absent; vein 2-1A present only proximally as tubular vein; tubular vein 1 cu-a curved, incomplete/broken and not reaching the edge of 1-1A vein. Hind wing with vannal lobe narrow, subdistally straightened and subproximally concave, and setae evenly scattered in the margin.

**Metasoma** (Fig. 197A, G, H, J). Metasoma laterally compressed. Petiole on T1 completely smooth and polished with faint, satin-like sheen, virtually parallel-sided over most of length, but narrowing over distal 1/3 (length 0.30, maximum width 0.19, minimum width 0.07), and with scattered pubescence concentrated in the first distal third. Lateral grooves delimiting the median area on T2 distally losing definition (length median area 0.10, length T2 0.12), edges of median area polished and lateral grooves deep, median area broader than long (length 0.10, maximum width 0.21, minimum width 0.05); T2 with scattered pubescence only distally. T3 longer than T2 (0.19, 0.12) and with scattered pubescence throughout. Pubescence on hypopygium dense.

**Cocoons.** White or beige oval cocoons with ordered silk fibers, but covered by a net. Cocoons adhered to the leaf substrate.

#### Comments.

In some females, the labrum and the mandibles are yellow-brown.

#### Male

**s** (Fig. 198A–H). Coloration similar to females; however, some specimens, as well as some females, have the mandibles yellow-brown. The coloration on T3 is a somewhat different, the inverted brown triangle is not so evident.

#### Etymology.

Scott E. Miller is very interested in tropical biology, and the role of biodiversity information in understanding and managing the related issues of tropical deforestation, climate change, invasive species, loss of biological diversity, and the resultant biological, economic, and political consequences. Currently, he is the curator of Lepidoptera at Smithsonian Institute, National Museum of Natural History, Washington, D.C., USA.

#### Distribution.

The parasitized caterpillar was collected in Costa Rica, ACG, Sector Pitilla (Estación Quica), during July 2010 at 470 m in rain forest.

#### Biology.

The lifestyle of this parasitoid species is gregarious.

#### Host.

*Metalectra* sp. Hübner (Noctuidae: Boletobiinae) feeding on epiphytic microplants. Caterpillar was collected in fifth instar.

### Glyptapanteles scottshawi

Taxon classificationAnimaliaHymenopteraBraconidae

Arias-Penna, sp. nov.

http://zoobank.org/4E74D955-F643-46CB-A3D5-A2CFF3B23C21

Figs 199 , 200

#### Female.

Body length 2.48 mm, antenna length 3.23 mm, fore wing length 2.77 mm.

#### Type material.

**Holotype** COSTA RICA • 1♀; 07-SRNP-66369, DHJPAR0024907; Área de Conservación Guanacaste, Alajuela, Sector San Cristóbal, Río Blanco Abajo; rain forest; Malaise; 500 m; 10.90037, -85.37254; 25.xi.2007; DH Janzen & W Hallwachs leg.; (CNC). **Paratypes**. • 1 (0♀, 1♂) (0♀, 0♂); 07-SRNP-67700, DHJPAR0026395; same data as for holotype except: 07.xi.2007; (CNC). • 1 (0♀, 0♂) (0♀, 1♂); 07-SRNP-66816, DHJPAR0025354; same data as for holotype except: 28.vii.2007; (CNC). • 1 (0♀, 0♂) (0♀, 1♂); 07-SRNP-67611, DHJPAR0026306; same data as for holotype except: 03.viii.2007; (CNC). • 1 (0♀, 1♂) (0♀, 0♂); 07-SRNP-67613, DHJPAR0026308; same data as for holotype except: 03.viii.2007; (CNC). • 1 (0♀, 0♂) (0♀, 1♂); 07-SRNP-67671, DHJPAR0026366; same data as for holotype except: 09.viii.2007; (CNC). • 1 (0♀, 0♂) (1♀, 0♂); 07-SRNP-67683, DHJPAR0026378; same data as for holotype except: 09.viii.2007; (CNC).

#### Other material.

**Malaise-trapped material.** COSTA RICA: *Área de Conservación Guanacaste*, *Alajuela*, *Sector San Cristóbal*, *Estación San Gerardo*: • 1 (0♀, 1♂) (0♀, 0♂); 07-SRNP-67284, DHJPAR0025822; rain forest; Malaise; 575 m; 10.88009, -85.38887; 04.vii.2007; DH Janzen & W Hallwachs leg.

*Área de Conservación Guanacaste*, *Alajuela*, *Sector San Cristóbal*, *Río Blanco Abajo*: • 1 (0♀, 0♂) (0♀, 1♂); 07-SRNP-66237, DHJPAR0024775; rain forest; Malaise; 500 m; 10.90037, -85.37254; 08.viii.2007; DH Janzen & W Hallwachs leg. • 1 (0♀, 0♂) (0♀, 1♂); 07-SRNP-66267, DHJPAR0024805; same data as for preceding except: 16.vi.2007. • 1 (0♀, 1♂) (0♀, 0♂); 07-SRNP-66320, DHJPAR0024858; same data as for preceding except: 27.viii.2007. • 1 (0♀, 1♂) (0♀, 0♂); 07-SRNP-66441, DHJPAR0024979; same data as for preceding except: 28.vii.2007. • 1 (0♀, 0♂) (0♀, 1♂); 07-SRNP-66442, DHJPAR0024980; same as for preceding data except: 28.vii.2007. • 1 (0♀, 0♂) (0♀, 1♂); 07-SRNP-66485, DHJPAR0025023; same data as for preceding except: 16.vii.2007. • 1 (1♀, 0♂) (0♀, 0♂); 07-SRNP-66495, DHJPAR0025033; same data as for preceding except: 16.vii.2007. • 1 (0♀, 0♂) (0♀, 1♂); 07-SRNP-66548, DHJPAR0025086; same data as for preceding except: 05.vi.2007. • 1 (0♀, 0♂) (0♀, 1♂); 07-SRNP-66656, DHJPAR0025194; same data as for preceding except: 04.vii.2007. • 1 (0♀, 1♂) (0♀, 0♂); 07-SRNP-66676, DHJPAR0025214; same data as for preceding except: 22.vii.2007. • 1 (0♀, 0♂) (0♀, 1♂); 07-SRNP-66690, DHJPAR0025228; same data as for preceding except: 22.vii.2007. • 1 (0♀, 0♂) (0♀, 1♂); 07-SRNP-66774, DHJPAR0025312; same data as for preceding except: 16.vi.2007. • 1 (0♀, 0♂) (0♀, 1♂); 07-SRNP-66789, DHJPAR0025327; same data as for preceding except: 16.vi.2007. • 1 (0♀, 0♂) (0♀, 1♂); 08-SRNP-2857, DHJPAR0026438; same data as for preceding except: 18.i.2008. • 1 (0♀, 1♂) (0♀, 0♂); 08-SRNP-2884, DHJPAR0026465; same data as for preceding except: 30.i.2008. • 1 (0♀, 0♂) (0♀, 1♂); 08-SRNP-2922, DHJPAR0026503; same data as for preceding except: 11.ii.2008. • 1 (1♀, 0♂) (0♀, 0♂); 08-SRNP-2950, DHJPAR0026531; same data as for preceding except: 17.ii.2008. • 1 (0♀, 0♂) (0♀, 1♂); 08-SRNP-2951, DHJPAR0026532; same data as for preceding except: 17.ii.2008. • 1 (0♀, 0♂) (0♀, 1♂); 08-SRNP-2954, DHJPAR0026535; same data as for preceding except: 17.ii.2008. • 1 (0♀, 0♂) (1♀, 0♂); 08-SRNP-2963, DHJPAR0026544; same data as for preceding except: 17.ii.2008. • 1 (0♀, 1♂) (0♀, 0♂); 08-SRNP-2969, DHJPAR0026550; same data as for preceding except: 23.ii.2008. • 1 (0♀, 0♂) (0♀, 1♂); 08-SRNP-2978, DHJPAR0026559; same data as for preceding except: 23.ii.2008. • 1 (0♀, 0♂) (0♀, 1♂); 08-SRNP-2982, DHJPAR0026563; same data as for preceding except: 23.ii.2008. • 1 (0♀, 0♂) (0♀, 1♂); 08-SRNP-2986, DHJPAR0026567; same data as for preceding except: 23.ii.2008. • 1 (0♀, 0♂) (0♀, 1♂); 08-SRNP-2989, DHJPAR0026570; same data as for preceding except: 23.ii.2008. • 1 (0♀, 0♂) (0♀, 1♂); 08-SRNP-3004, DHJPAR0026585; same data as for preceding except: 29.ii.2008. • 1 (0♀, 1♂) (0♀, 0♂); 08-SRNP-3009, DHJPAR0026590; same data as for preceding except: 29.ii.2008. • 1 (0♀, 0♂) (0♀, 1♂); 08-SRNP-3016, DHJPAR0026597; same data as for preceding except: 29.ii.2008. • 1 (0♀, 0♂) (0♀, 1♂); 08-SRNP-3024, DHJPAR0026605; same data as for preceding except: 29.ii.2008. • 1 (0♀, 0♂) (0♀, 1♂); 08-SRNP-3046, DHJPAR0026627; same data as for preceding except: 06.iii.2008. • 1 (0♀, 0♂) (0♀, 1♂); 08-SRNP-3060, DHJPAR0026641; same data as for preceding except: 06.iii.2008. • 1 (0♀, 0♂) (0♀, 1♂); 08-SRNP-3061, DHJPAR0026642; same data as for preceding except: 06.iii.2008. • 1 (0♀, 0♂) (0♀, 1♂); 08-SRNP-3075, DHJPAR0026656; same data as for preceding except: 12.iii.2008. • 1 (0♀, 1♂) (0♀, 0♂); 08-SRNP-3092, DHJPAR0026673; same data as for preceding except: 12.iii.2008. • 1 (0♀, 1♂) (0♀, 0♂); 08-SRNP-3097, DHJPAR0026678; same data as for preceding except: 12.iii.2008. • 1 (0♀, 0♂) (0♀, 1♂); 08-SRNP-3106, DHJPAR0026687; same data as for preceding except: 12.iii.2008. • 1 (0♀, 1♂) (0♀, 0♂); 08-SRNP-3111, DHJPAR0026692; same data as for preceding except: 12.iii.2008. • 1 (1♀, 0♂) (0♀, 0♂); 08-SRNP-3168, DHJPAR0026749; same data as for preceding except: 24.iii.2008. • 1 (0♀, 1♂) (0♀, 0♂); 08-SRNP-3216, DHJPAR0026797; same data as for preceding except: 24.iii.2008. • 1 (0♀, 0♂) (0♀, 1♂); 08-SRNP-3223, DHJPAR0026804; same data as for preceding except: 24.iii.2008. • 1 (0♀, 0♂) (0♀, 1♂); 08-SRNP-3233, DHJPAR0026814; same data as for preceding except: 30.iii.2008. • 1 (0♀, 0♂) (0♀, 1♂); 08-SRNP-3248, DHJPAR0026829; same data as for preceding except: 30.iii.2008. • 1 (0♀, 0♂) (0♀, 1♂); 08-SRNP-3252, DHJPAR0026833; same data as for preceding except: 30.iii.2008. • 1 (0♀, 0♂) (0♀, 1♂); 08-SRNP-3305, DHJPAR0026886; same data as for preceding except: 05.iv.2008. • 1 (0♀, 0♂) (1♀, 0♂); 08-SRNP-3313, DHJPAR0026894; same data as for preceding except: 05.iv.2008. • 1 (1♀, 0♂) (0♀, 0♂); 08-SRNP-3316, DHJPAR0026897; same data as for preceding except: 05.iv.2008. • 1 (0♀, 0♂) (0♀, 1♂); 08-SRNP-3334, DHJPAR0026915; same data as for preceding except: 05.iv.2008. • 1 (0♀, 0♂) (0♀, 1♂); 08-SRNP-3413, DHJPAR0026994; same data as for preceding except: 11.iv.2008. • 1 (0♀, 0♂) (1♀, 0♂); 08-SRNP-3441, DHJPAR0027022; same data as for preceding except: 11.iv.2008. • 1 (0♀, 0♂) (1♀, 0♂); 08-SRNP-3445, DHJPAR0027026; same data as for preceding except: 11.iv.2008. • 1 (0♀, 0♂) (0♀, 1♂); 08-SRNP-3474, DHJPAR0027055; same data as for preceding except: 17.iv.2008. • 1 (0♀, 0♂) (0♀, 1♂); 08-SRNP-3476, DHJPAR0027057; same data as for preceding except: 17.iv.2008. • 1 (0♀, 0♂) (0♀, 1♂); 08-SRNP-3481, DHJPAR0027062; same data as for preceding except: 17.iv.2008. • 1 (0♀, 1♂) (0♀, 0♂); 08-SRNP-3538, DHJPAR0027119; same data as for preceding except: 23.iv.2008. • 1 (0♀, 0♂) (0♀, 1♂); 08-SRNP-3545, DHJPAR0027126; same data as for preceding except: 23.iv.2008. • 1 (0♀, 0♂) (0♀, 1♂); 08-SRNP-3563, DHJPAR0027144; same data as for preceding except: 23.iv.2008. • 1 (0♀, 1♂) (0♀, 0♂); 08-SRNP-3578, DHJPAR0027159; same data as for preceding except: 23.iv.2008. • 1 (0♀, 0♂) (0♀, 1♂); 08-SRNP-3580, DHJPAR0027161; same data as for preceding except: 23.iv.2008. • 1 (0♀, 0♂) (0♀, 1♂); 08-SRNP-3586, DHJPAR0027167; same data as for preceding except: 23.iv.2008. • 1 (0♀, 0♂) (0♀, 1♂); 08-SRNP-3603, DHJPAR0027184; same data as for preceding except: 23.iv.2008. • 1 (0♀, 0♂) (1♀, 0♂); 08-SRNP-3613, DHJPAR0027194; same data as for preceding except: 23.iv.2008. • 1 (0♀, 0♂) (0♀, 1♂); 08-SRNP-3615, DHJPAR0027196; same data as for preceding except: 23.iv.2008. • 1 (0♀, 0♂) (0♀, 1♂); 08-SRNP-3629, DHJPAR0027210; same data as for preceding except: 23.iv.2008. • 1 (0♀, 0♂) (0♀, 1♂); 08-SRNP-3652, DHJPAR0027233; same data as for preceding except: 30.iv.2008. • 1 (0♀, 1♂) (0♀, 0♂); 08-SRNP-3707, DHJPAR0027288; same data as for preceding except: 06.v.2008. • 1 (0♀, 0♂) (0♀, 1♂); 08-SRNP-3710, DHJPAR0027291; same data as for preceding except: 06.v.2008. • 1 (0♀, 0♂) (0♀, 1♂); 08-SRNP-3737, DHJPAR0027318; same data as for preceding except: 06.v.2008. • 1 (0♀, 1♂) (0♀, 0♂); 08-SRNP-3762, DHJPAR0027343; same data as for preceding except: 06.v.2008. • 1 (0♀, 0♂) (0♀, 1♂); 08-SRNP-3769, DHJPAR0027350; same data as for preceding except: 12.v.2008. • 1 (0♀, 0♂) (0♀, 1♂); 08-SRNP-3797, DHJPAR0027378; same data as for preceding except: 12.v.2008. • 1 (0♀, 0♂) (0♀, 1♂); 08-SRNP-3803, DHJPAR0027384; same data as for preceding except: 12.v.2008. • 1 (0♀, 0♂) (0♀, 1♂); 08-SRNP-3832, DHJPAR0027413; same data as for preceding except: 18.v.2008. • 1 (0♀, 0♂) (0♀, 1♂); 08-SRNP-3833, DHJPAR0027414; same data as for preceding except: 18.v.2008. • 1 (0♀, 0♂) (0♀, 1♂); 08-SRNP-3857, DHJPAR0027438; same data as for preceding except: 18.v.2008. • 1 (0♀, 0♂) (0♀, 1♂); 08-SRNP-3877, DHJPAR0027458; same data as for preceding except: 18.v.2008.

*Área de Conservación Guanacaste*, *Alajuela*, *Sector San Cristóbal*, *Potrero Argentina*: • 1 (0♀, 1♂) (0♀, 0♂); 07-SRNP-67027, DHJPAR0025565; pastures; Malaise; 520 m; 10.89021, -85.38803; 04.vii.2007; DH Janzen & W Hallwachs leg. • 1 (0♀, 0♂) (0♀, 1♂); 07-SRNP-67059, DHJPAR0025597; same data as for preceding except: 21.viii.2007. • 1 (0♀, 1♂) (0♀, 0♂); 07-SRNP-67069, DHJPAR0025607; same data as for preceding except: 02.x.2007. • 1 (0♀, 0♂) (0♀, 1♂); 07-SRNP-67112, DHJPAR0025650; same data as for preceding except: 10.vi.2007. • 1 (0♀, 1♂) (0♀, 0♂), 07-SRNP-67146, DHJPAR0025684; same data as for preceding except: 16.vii.2007. • 1 (1♀, 0♂) (0♀, 0♂); 07-SRNP-67200, DHJPAR0025738; same data as for preceding except: 04.vii.2007. • 1 (0♀, 1♂) (0♀, 0♂); 07-SRNP-67214, DHJPAR0025752; same data as for preceding except: 16.vi.2007. • 1 (0♀, 0♂) (0♀, 1♂); 07-SRNP-67221, DHJPAR0025759; same data as for preceding except: 16.vi.2007. • 1 (0♀, 0♂) (0♀, 1♂); 07-SRNP-67224, DHJPAR0025762; same data as for preceding except: 16.vi.2007. • 1 (0♀, 0♂) (0♀, 1♂); 07-SRNP-67225, DHJPAR0025763; same data as for preceding except: 16.vi.2007. • 1 (1♀, 0♂) (0♀, 0♂); 07-SRNP-67228, DHJPAR0025766; same data as for preceding except: 16.vi.2007. • 1 (0♀, 0♂) (0♀, 1♂); 07-SRNP-67255, DHJPAR0025793; same data as for preceding except: 10.vi.2007. • 1 (0♀, 0♂) (0♀, 1♂); 07-SRNP-67259, DHJPAR0025797; same data as for preceding except: 22.vi.2007. • 1 (0♀, 1♂) (0♀, 0♂); 07-SRNP-67749, DHJPAR0027487; same data as for preceding except: 03.viii.2007. • 1 (0♀, 1♂) (0♀, 0♂); 08-SRNP-3903, DHJPAR0027557; same data as for preceding except: 11.ii.2008. • 1 (0♀, 0♂) (0♀, 1♂); 08-SRNP-3914, DHJPAR0027568; same data as for preceding except: 17.ii.2008. • 1 (0♀, 0♂) (0♀, 1♂); 08-SRNP-3915, DHJPAR0027569; same data as for preceding except: 17.ii.2008. • 1 (0♀, 0♂) (0♀, 1♂); 08-SRNP-3916, DHJPAR0027570; same data as for preceding except: 17.ii.2008. • 1 (0♀, 0♂) (0♀, 1♂); 08-SRNP-3917, DHJPAR0027571; same data as for preceding except: 23.ii.2008. • 1 (0♀, 0♂) (0♀, 1♂); 08-SRNP-3920, DHJPAR0027574; same data as for preceding except: 23.ii.2008. • 1 (0♀, 1♂) (0♀, 0♂); 08-SRNP-3921, DHJPAR0027575; same data as for preceding except: 23.ii.2008. • 1 (0♀, 1♂) (0♀, 0♂); 08-SRNP-3922, DHJPAR0027576; same data as for preceding except: 23.ii.2008. • 1 (0♀, 1♂) (0♀, 0♂); 08-SRNP-3932, DHJPAR0027586; same data as for preceding except: 06.iii.2008. • 1 (0♀, 0♂) (0♀, 1♂); 08-SRNP-3933, DHJPAR0027587; same data as for preceding except: 06.iii.2008.

*Área de Conservación Guanacaste*, *Alajuela*, *Sector Rincón Rain Forest*, *Vado Río Francia*: • 1 (0♀, 0♂) (0♀, 1♂); 07-SRNP-66861, DHJPAR0025399; Malaise; 400 m; 10.90093, -85.28915; 11.vi.2007; DH Janzen & W Hallwachs leg. • 1 (0♀, 1♂) (0♀, 0♂); 07-SRNP-66864, DHJPAR0025402; same data as for preceding. • 1 (0♀, 0♂) (0♀, 1♂); 07-SRNP-66866, DHJPAR0025404; same data as for preceding. • 1 (0♀, 0♂) (0♀, 1♂); 07-SRNP-66895, DHJPAR0025433; same data as for preceding except: 21.ix.2007. • 1 (0♀, 0♂) (0♀, 1♂); 07-SRNP-66922, DHJPAR0025460; same data as for preceding except: 20.xi.2007. • 1 (0♀, 0♂) (0♀, 1♂); 07-SRNP-66925, DHJPAR0025463; same data as for preceding except: 20.xi.2007. • 1 (1♀, 0♂) (0♀, 0♂); 07-SRNP-66974, DHJPAR0025512; same data as for preceding except: 29.vi.2007. • 1 (0♀, 0♂) (0♀, 1♂); 07-SRNP-66977, DHJPAR0025515; same data as for preceding except: 29.vi.2007. • 1 (1♀, 0♂) (0♀, 0♂); 07-SRNP-66978, DHJPAR0025516; same data as for preceding except: 29.vi.2007. • 1 (0♀, 0♂) (0♀, 1♂); 07-SRNP-66981, DHJPAR0025519; same data as for preceding except: 29.vi.2007. • 1 (1♀, 0♂) (0♀, 0♂); 07-SRNP-66996, DHJPAR0025534; same data as for preceding except: 29.vi.2007. • 1 (0♀, 1♂) (0♀, 0♂); 07-SRNP-67009, DHJPAR0025547; same data as for preceding except: 05.vii.2007. • 1 (0♀, 1♂) (0♀, 0♂); 07-SRNP-67572, DHJPAR0026102; same data as for preceding except: 04.viii.2007. • 1 (0♀, 0♂) (0♀, 1♂); 07-SRNP-67574, DHJPAR0026104; same data as for preceding except: 27.x.2007. • 1 (1♀, 0♂) (0♀, 0♂); 08-SRNP-41728, DHJPAR0026171; same data as for preceding except: 18.ii.2008. • 1 (0♀, 0♂) (0♀, 1♂); 08-SRNP-41729, DHJPAR0026172; same data as for preceding except: 18.ii.2008. • 1 (0♀, 1♂) (0♀, 0♂); 08-SRNP-41739, DHJPAR0026182; same data as for preceding except: 01.iii.2008. • 1 (0♀, 0♂) (0♀, 1♂); 08-SRNP-41741, DHJPAR0026184; same data as for preceding except: 01.iii.2008. • 1 (0♀, 1♂) (0♀, 0♂); 08-SRNP-41787, DHJPAR0026230; same data as for preceding except: 31.iii.2008.

#### Diagnosis.

**Figure 199.**
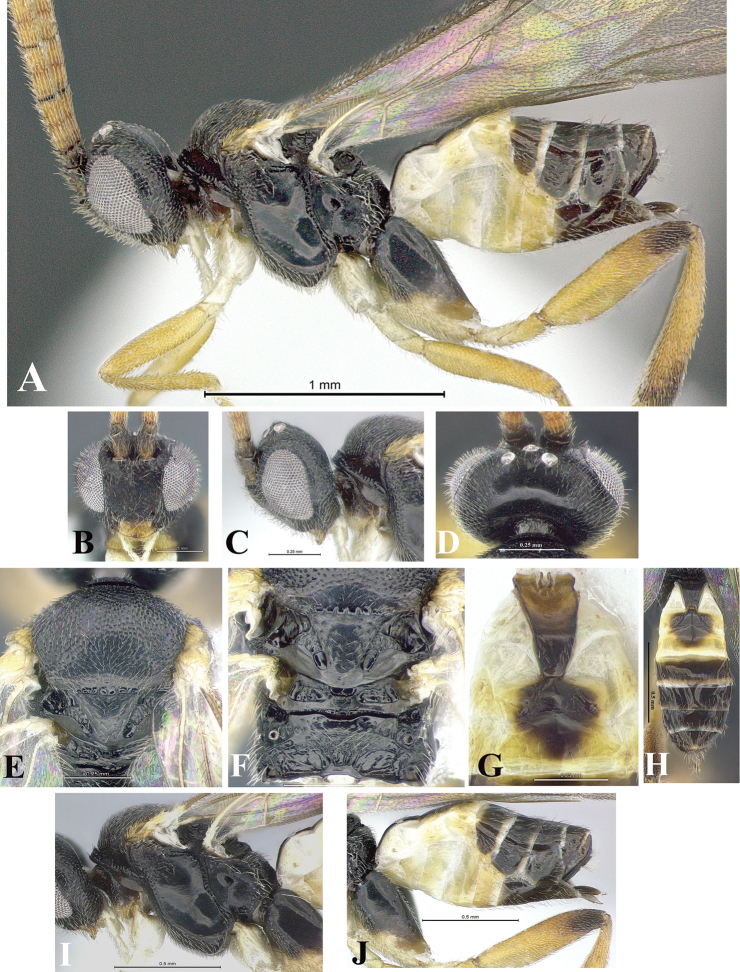
*Glyptapantelesscottshawi* sp. nov. female 07-SRNP-66369 DHJPAR0024907, 08-SRNP-2950 DHJPAR0026531 **A** Habitus **B, D** Head **B** Frontal view **D** Dorsal view **C** Head, pronotum, propleuron, lateral view **E** Mesonotum, dorsal view **F** Scutellum, metanotum, propodeum, dorsal view **G**T1–3, dorsal view **H, J** Metasoma **H** Dorsal view **J** Lateral view **I** Mesosoma, lateral view.

**Figure 200.**
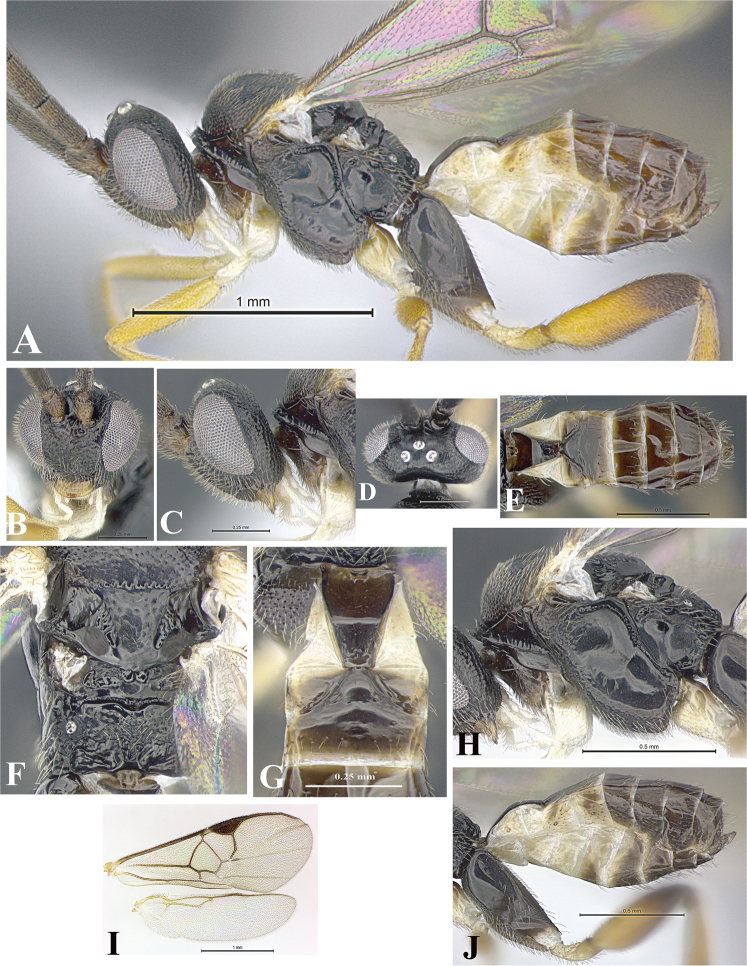
*Glyptapantelesscottshawi* sp. nov. male 07-SRNP-67749 DHJPAR0027487, 08-SRNP-3917 DHJPAR0027571 **A** Habitus **B, D** Head **B** Frontal view **D** Dorsal view **C** Head, pronotum, propleuron, lateral view **E, J** Metasoma **E** Dorsal view **J** Lateral view **F** Scutellum, metanotum, propodeum, dorsal view **G**T1–3, dorsal view **H** Mesosoma, lateral view **I** Fore and hind wings.

Medioposterior band of scutellum mostly overlapping the medioanterior pit of metanotum (Figs 199F, 220F), pronotum with a distinct dorsal furrow (Figs 199C, I, 200H), petiole on T1 evenly narrowing distally, finely sculptured only laterally (Figs 199G, H, 200G), antenna longer than body, distal antennal flagellomere longer than penultimate, precoxal groove deep, smooth, and shiny (Figs 199A, I, 200A, H), fore wing with vein 1 cu-a curved, r vein curved (Fig. 200I), dorsal outer depression on hind coxa present (Figs 199A, J, 200A, J), inner margin of eyes diverging slightly at antennal sockets (Figs 199B, 200B), and lateral grooves delimiting the median area on T2 clearly defined and reaching the distal edge of T2 (Figs 199G, H, 200G).

#### Coloration

(Fig. 199A–J). General body coloration black except scape proximally yellow-brown/reddish and distally brown; pedicel distally yellow-brown/reddish and proximally brown; first seven-eight proximal antennal flagellomeres completely yellow (the yellow coloration is darker on the first four antennal flagellomeres), remaining flagellomeres completely brown; tegulae yellow; labrum, mandible, propleuron distally, and dorsal furrow of pronotum yellow-brown/reddish; glossa, maxillary and labial palps pale yellow/ivory. Eyes and ocelli silver. Fore and middle legs dark yellow, except coxae and trochanters pale yellow/ivory; hind legs dark yellow except proximal half of coxae black, femora distally and tibiae brown, and tarsomeres dark yellow. Petiole on T1 with two colorations: proximal 1/4 yellow-brown/reddish and distal 3/4 brown, contours darkened and sublateral areas ivory/pale yellow; T2 with median and adjacent areas brown, and lateral ends ivory/pale yellow; T3 medially brown, proximally dark area coinciding with the width of dark area of median and adjacent areas on T2, but distally T3 narrowing, remaining area ivory/pale yellow; T4 and beyond completely brown; distally each tergum with a narrow ivory/pale yellow transparent band. In lateral view, T1–3 ivory/pale yellow; T4 and beyond brown. S1–5 ivory/yellow; hypopygium brown.

#### Description.

**Head** (Fig. 199A–D). Head rounded with pubescence long and dense. Proximal three antennal flagellomeres longer than wide (0.25:0.08, 0.24:0.08, 0.24:0.08), distal antennal flagellomere longer than penultimate (0.14:0.06, 0.11:0.07), antenna longer than body (3.23, 2.48); antennal scrobes-frons shallow. Face flat or nearly so, with dense fine punctations, interspaces smooth and longitudinal median carina present. Frons smooth. Temple wide, punctate and interspaces wavy. Inner margin of eyes diverging slightly at antennal sockets; in lateral view, eye anteriorly convex and posteriorly straight. POL shorter than OOL (0.08, 0.12). Malar suture present. Median area between lateral ocelli slightly depressed. Vertex laterally pointed or nearly so and dorsally wide.

**Mesosoma** (Fig. 199A, E, F, I). Mesosoma dorsoventrally convex. Mesoscutum proximally convex and distally flat, punctation distinct throughout, interspaces smooth. Scutellum triangular, apex sloped and fused with BS, scutellar punctation scattered throughout, in profile scutellum flat and on same plane as mesoscutum, phragma of the scutellum partially exposed; BS mostly overlapping the MPM; ATS demilune with complete undulate/reticulate carinae; dorsal ATS groove with semicircular/parallel carinae. Transscutal articulation with small and heterogeneous foveae, area just behind transscutal articulation nearly at the same level as mesoscutum (flat) and with same kind of sculpture as mesoscutum. Metanotum with BM upward; MPM semicircular and bisected by a median longitudinal carina; AFM with a small lobe and not as well delineated as PFM; PFM thick, smooth and with lateral ends rounded; ATM with undulate carinae throughout. Propodeum without median longitudinal carina, proximal half curved with medium-sized sculpture and distal half rugose; distal edge of propodeum with a flange at each side and without stubs; propodeal spiracle distally framed by a short concave carina; nucha surrounded by very short radiating carinae. Pronotum with a distinct dorsal furrow, dorsally with a well-defined smooth band; central area of pronotum and dorsal furrow smooth, but ventral furrow with short parallel carinae. Propleuron with fine rugae and dorsally without a carina. Metasternum convex. Contour of mesopleuron convex; precoxal groove deep, smooth and shiny; epicnemial ridge convex, teardrop-shaped.

**Legs.** Ventral margin of fore telotarsus entire without seta, fore telotarsus proximally narrow and distally wide, and longer than fourth tarsomere (0.11, 0.07). Hind coxa with punctation only on ventral surface, dorsal outer depression present. Inner spur of hind tibia longer than outer spur (0.26, 0.20), entire surface of hind tibia with dense strong spines clearly differentiated by color and length. Hind telotarsus as equal in length as fourth tarsomere (0.12, 0.11).

**Wings** (Fig. 200I). Fore wing with r vein slightly curved; 2RS vein straight; r and 2RS veins forming a weak, even curve at their junction and outer side of junction not forming a stub; 2M vein slightly curved/swollen; distally fore wing [where spectral veins are] with microtrichiae more densely concentrated than the rest of the wing; anal cell 1/3 proximally lacking microtrichiae; subbasal cell with a small smooth area, vein 2CUa absent and vein 2CUb spectral; vein 2 cu-a absent; vein 2-1A proximally tubular and distally spectral, although sometimes difficult to see; tubular vein 1 cu-a curved and complete, but junction with 1-1A vein spectral. Hind wing with vannal lobe very narrow, subdistally and subproximally straightened, and setae evenly scattered in the margin.

**Metasoma** (Fig. 199A, G, H, J). Metasoma laterally compressed. Petiole on T1 finely sculptured only laterally, evenly narrowing distally (length 0.34, maximum width 0.17, minimum width 0.07) and with scattered pubescence concentrated in the first distal third. Lateral grooves delimiting the median area on T2 clearly defined and reaching the distal edge of T2 (length median area 0.14, length T2 0.14), edges of median area polished and lateral grooves deep, median area broader than long (length 0.14, maximum width 0.20, minimum width 0.05); T2 with scattered pubescence only distally. T3 longer than T2 (0.19, 0.14) and with scattered pubescence throughout. Pubescence on hypopygium dense.

**Cocoons.** Unknown.

#### Comments.

In some females, the propleuron distally yellow; both the dorsal and the ventral furrows of pronotum, the area of the mesoscutum just above the dorsal furrow of pronotum and the epicnemial ridge are lighter than mesosoma coloration (light brown, reddish or yellow-brown); proximal half of the petiole yellow-brown/reddish and distal half brown; the shape of dark area on T3 can be slightly different. In other specimens, the coloration of hind legs is yellow-brown instead of dark yellow. In other females, the penultimate sternum with two colorations: proximal half yellow and distal half brown.

#### Male

(Fig. 200A–J). All the antennal flagellomeres have the same color; in some preserved males, the eyes are black and the ocelli reddish; in other preserved males, the coloration on metosoma varies thus: the petiole and the T3 are brown; the S3–4 are yellow, but with some brown tints, the S5 and beyond are completely brown; in other males, the hind legs are light yellow-brown.

#### Etymology.

Scott R. Shaw is a Professor and Insect Museum Curator at the University of Wyoming, Laramie, WY, USA. His research is focused in systematics of Braconidae, mainly Meteorinae, Rogadinae, and Euphorinae.

#### Distribution.

The adult parasitoids were collected in Costa Rica, ACG, Sector Rincón Rain Forest (Vado Río Francia) and Sector San Cristóbal (Estación San Gerardo, Potrero Argentina, and Río Blanco Abajo), during June–November 2007 and January–May 2008 at 400 m, 520 m, 550 m, and 575 m in pasture and rain forest.

#### Biology.

Unknown.

#### Host.

Unknown.

### Glyptapanteles shelbystedenfeldae

Taxon classificationAnimaliaHymenopteraBraconidae

Arias-Penna, sp. nov.

http://zoobank.org/BE4E864D-2A14-4C41-AA4A-166F178E9927

Figs 201 , 202

#### Male.

Body length 2.17 mm, antenna length 3.08 mm, fore wing length 3.68 mm.

#### Type material.

**Holotype**: COSTA RICA • 1♀; 00-SRNP-24000, DHJPAR0013608; Área de Conservación Guanacaste, Guanacaste, Sector Santa Rosa, Bosque Humedo; dry forest; Malasie; 290 m; 10.85145, -85.60801; 24.iv.2000; DH Janzen & W Hallwachs leg.; (CNC). **Paratypes**. • 1 (0♀, 0♂) (0♀, 1♂); 00-SRNP-23957, DHJPAR0013596; same data as for holotype except: 10.iv.2000; (CNC).

#### Other material.

**Malaise-trapped material.** COSTA RICA: *Área de Conservación Guanacaste*, *Guanacaste*, *Sector Santa Rosa*, *Bosque Humedo*: • 1 (1♀, 0♂) (0♀, 0♂); 98-SRNP-16106, DHJPAR0013373; Malaise; dry forest; 290 m; 10.85145, -85.60801; 09.iii.1998; DH Janzen & W Hallwachs leg. • 1 (0♀, 1♂) (0♀, 0♂); 98-SRNP-16130, DHJPAR0013579; same data as for preceding except: 30.iii.1998. • 1 (0♀, 0♂) (0♀, 1♂); 99-SRNP-19079, DHJPAR0013581; same data as for preceding except: 03.v.1999. • 1 (0♀, 1♂) (0♀, 0♂); 99-SRNP-19086, DHJPAR0013583; same data as for preceding except: 03.v.1999. • 1 (0♀, 1♂) (0♀, 0♂); 99-SRNP-19096, DHJPAR0013587; same data as for preceding except: 10.v.1999. • 1 (0♀, 1♂) (0♀, 0♂); 00-SRNP-24025, DHJPAR0013408; same data as for preceding except: 06.iii.2000. • 1 (0♀, 0♂) (0♀, 1♂); 00-SRNP-24006, DHJPAR0013414; same data as for preceding except: 27.iii.2000. • 1 (0♀, 1♂) (0♀, 0♂); 00-SRNP-23961, DHJPAR0013599; same data as for preceding except: 22.v.2000. • 1 (0♀, 1♂) (0♀, 1♂); 00-SRNP-23988, DHJPAR0013607; same data as for preceding except: 22.v.2000. • 1 (0♀, 1♂) (0♀, 0♂); 07-SRNP-15057, DHJPAR0013617; same data as for preceding except: 05.vi.2007; AR Deans & J Rodriguez leg.

*Área de Conservación Guanacaste*, *Guanacaste*, *Sector Santa Rosa*, *Bosque San Emilio*: • 1 (0♀, 1♂) (0♀, 0♂); 00-SRNP-23869, DHJPAR0013555; Malaise; dry forest; 300 m; 10.84389; -85.61384; 22.v.2000; DH Janzen & W Hallwachs leg.

#### Diagnosis.

**Figure 201.**
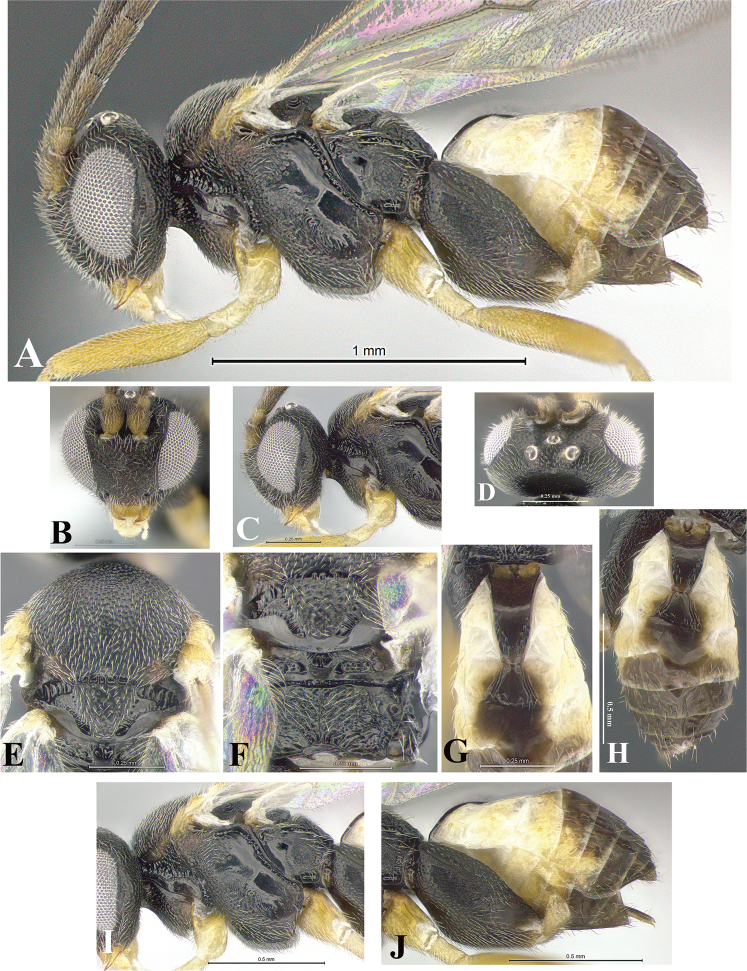
*Glyptapantelesshelbystedenfeldae* sp. nov. female 98-SRNP-16106 DHJPAR0013373 **A** Habitus **B, D** Head **B** Frontal view **D** Dorsal view **C** Head, pronotum, propleuron, lateral view **E** Mesonotum, dorsal view **F** Scutellum, metanotum, propodeum, dorsal view **G**T1–3, dorsal view **H, J** Metasoma **H** Dorsal view **J** Lateral view **I** Mesosoma, lateral view.

**Figure 202.**
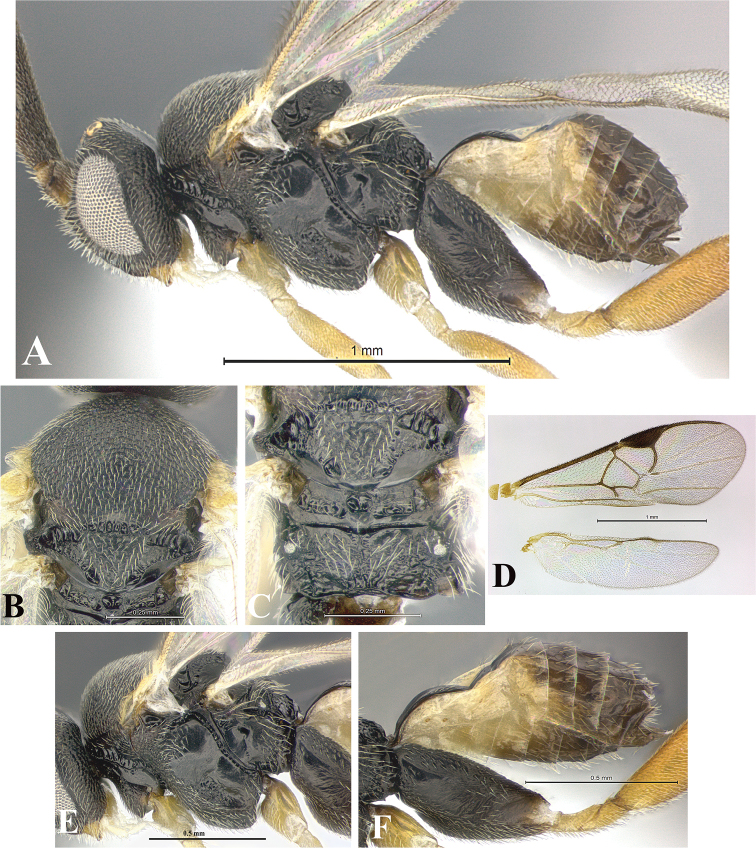
*Glyptapantelesshelbystedenfeldae* sp. nov. male 00-SRNP-23869 DHJPAR0013555, 00-SRNP-24020 DHJPAR0013403 **A** Habitus **B** Mesonotum, dorsal view **C** Scutellum, metanotum, propodeum, dorsal view **D** Fore and hind wings **E** Mesosoma, lateral view **F** Metasoma, lateral view.

Precoxal groove with transverse lineate sculpture (Figs 201A, I, 202E), medioanterior pit of metanotum circular and bisected by a median longitudinal carina (Figs 201F, 202C), inner margin of eyes diverging slightly at antennal sockets (Fig. 201B), scutellar punctation distinct throughout (Figs 201E, F, 202B, C), fore wing with 1 cu-a vein complete, but junction with 1-1A vein spectral, outer side of junction of r and 2RS veins not forming a stub (Fig. 202D), propodeum with a clearly visible median longitudinal carina (Figs 201F, 202C), and lateral grooves delimiting the median area on T2 clearly defined and reaching the distal edge of T2 (Figs 201G, H).

#### Coloration

(Fig. 202A–F). General body coloration black except scape yellow-brown/reddish distally with a brown band; pedicel yellow-brown/reddish; first four-five proximal antennal flagellomeres dorsally lighter (light brown) than ventrally (dark brown), remaining flagellomeres dark brown on both sides; labrum, mandible, and tegulae yellow; propleuron distally with a tiny dot, dorsal furrow of pronotum, and epicnemial ridge with yellow-brown/reddish tints; glossa, maxillary and labial palps pale yellow/ivory. Eyes and ocelli silver. Fore and middle legs dark yellow except brown claws; hind legs dark yellow except black coxae, femora distally brown, and tibiae with both ends brown. Petiole on T1 light brown, contours darkened and sublateral areas ivory/pale yellow; T2 with median and adjacent areas brown, and lateral ends ivory/pale yellow; T3 brown, but 1/3 proximal of lateral ends ivory/pale yellow; T4 and beyond completely brown; distally each tergum with a narrow yellowish translucent band. In lateral view, T1–3 ivory/pale yellow; T4 and beyond yellow with brown tints. S1–2 ivory/pale yellow; S3 yellow, but medially brown; S4 and beyond brown.

#### Description.

**Head** (Fig. 202A). Head rounded with pubescence long and dense. Proximal three antennal flagellomeres longer than wide (0.19:0.07, 0.20:0.07, 0.19:0.07), distal antennal flagellomere longer than penultimate (0.13:0.05, 0.10:0.05), antenna longer than body (3.08, 2.17); antennal scrobes-frons shallow. Face convex with dense fine punctations, interspaces wavy and longitudinal median carina present. Frons smooth. Temple wide, punctate-lacunose and interspaces wavy. Inner margin of eyes diverging slightly at antennal sockets; in lateral view, eye anteriorly convex and posteriorly straight. POL subequal in length with OOL (0.09, 0.10). Malar suture present. Median area between lateral ocelli slightly depressed. Vertex laterally pointed or nearly so and dorsally wide.

**Mesosoma** (Fig. 202A–C, E). Mesosoma dorsoventrally convex. Mesoscutum proximally convex and distally flat, punctation distinct throughout, interspaces wavy/lacunose. Scutellum triangular, apex sloped and fused with BS, scutellar punctation distinct throughout, in profile scutellum flat and on same plane as mesoscutum, phragma of the scutellum partially exposed; BS not overlapping the MPM; ATS demilune with a little, complete and parallel carinae; dorsal ATS groove with semicircular/parallel carinae. Transscutal articulation with small and heterogeneous foveae, area just behind transscutal articulation smooth, shiny and depressed centrally. Metanotum with BM wider than PFM (clearly differentiated); MPM circular and bisected by a median longitudinal carina; AFM with a small lobe and not as well delineated as PFM; PFM thick, smooth and with lateral ends rounded; ATM proximally with a groove with some sculpturing and distally smooth. Propodeum with a clearly visible median longitudinal carina, proximal half curved rather coarse sculpture and distal half rugose; distal edge of propodeum with a flange at each side and without stubs; propodeal spiracle distally framed by a short concave carina; nucha surrounded by very short radiating carinae. Pronotum with a distinct dorsal furrow, dorsally with a well-defined smooth band; both dorsal and ventral furrows and central area of pronotum with sculpture. Propleuron rugose and dorsally with a carina. Metasternum convex. Contour of mesopleuron convex; precoxal groove deep with transverse lineate sculpture; epicnemial ridge convex, teardrop-shaped.

**Legs** (Fig. 202A, F). Ventral margin of fore telotarsus entire without seta, fore telotarsus almost same width throughout and longer than fourth tarsomere (0.10, 0.06). Medially hind coxa smooth, dorsally with scattered punctation and ventrally with dense punctation, dorsal outer depression present. Inner spur of hind tibia longer than outer spur (0.23, 0.17), entire surface of hind tibia with dense strong spines clearly differentiated by color and length. Hind telotarsus as equal in length as fourth tarsomere (0.11, 0.11).

**Wings** (Fig. 202D). Fore wing with r vein curved; 2RS vein slightly concave; r and 2RS veins forming a weak, even curve at their junction and outer side of junction not forming a stub; 2M vein slightly curved/swollen; distally fore wing [where spectral veins are] with microtrichiae more densely concentrated than the rest of the wing; anal cell 1/3 proximally lacking microtrichiae; subbasal cell with a small smooth area; vein 2CUa absent and vein 2CUb spectral; vein 2 cu-a absent; vein 2-1A proximally tubular and distally spectral, although sometimes difficult to see; tubular vein 1 cu-a curved and complete, but junction with 1-1A vein spectral. Hind wing with vannal lobe narrow, subdistally and subproximally straightened, and setae evenly scattered in the margin.

**Metasoma** (Fig. 202A, F). Metasoma laterally compressed. Petiole on T1 finely sculptured only distally, evenly narrowing distally (length 0.31, maximum width 0.15, minimum width 0.04) and with scattered pubescence concentrated in the first distal third. Lateral grooves delimiting the median area on T2 clearly defined and reaching the distal edge of T2 (length median area 0.11, length T2 0.11), edges of median area polished and lateral grooves deep, median area broader than long (length 0.11, maximum width 0.14, minimum width 0.04); T2 with scattered pubescence only distally. T3 longer than T2 (0.19, 0.11) and with scattered pubescence throughout. Pubescence on hypopygium dense.

**Cocoons.** Unknown.

#### Comments.

The sculpture on body are dense (Fig. 201A, F), the hind coxae is stout, and both sexes with stout and short bodies.

#### Female

(Fig. 201A–J). The metasoma is missing in the only preserved female; however, there is a photographic record. In female, the distal corners of mesoscutum are yellow-brown/reddish.

#### Etymology.

Shelby E. Stedenfeld as graduate student at the Kentucky University, Lexington, KY, USA, was interested in the taxonomy and systematics of Ichneumonidae and their roles in biological control.

#### Distribution.

The adult parasitoids were collected in Costa Rica, ACG, Sector Santa Rosa (Bosque Humedo and Bosque San Emilio), during May 1999, March-May 2000, and June 2007 at 290 m in dry forest.

#### Biology.

Unknown.

#### Host.

Unknown.

### Glyptapanteles sondrawardae

Taxon classificationAnimaliaHymenopteraBraconidae

Arias-Penna, sp. nov.

http://zoobank.org/F18AD254-CC52-4DFD-96E3-6EC31CD9AEF8

Fig. 203

#### Female.

Body length 2.73 mm, antenna length 3.38 mm, fore wing length 2.68 mm.

#### Type material.

**Holotype**: COSTA RICA • 1♀; 07-SRNP-66795, DHJPAR0025333; Área de Conservación Guanacaste, Alajuela, Sector San Cristóbal, Río Blanco Abajo; rain forest; Malaise; 500 m; 10.90037, -85.37254; 16.vi.2007; DH Janzen & W Hallwachs leg.; (CNC). **Paratypes.** • 1 (0♀, 0♂) (0♀, 1♂); 07-SRNP-66529, DHJPAR0025067; same data as for holotype except: 22.vi.2007; (CNC). • 1 (0♀, 0♂) (0♀, 1♂); 07-SRNP-66552, DHJPAR0025090; same data as for holotype except: 05.vi.2007; (CNC). • 1 (0♀, 1♂) (0♀, 0♂); 07-SRNP-66559, DHJPAR0025097; same data as for holotype except: 05.vi.2007; (CNC).

#### Other material.

**Malaise-trapped material.** COSTA RICA: *Área de Conservación Guanacaste*, *Alajuela*, *Sector San Cristóbal*, *Río Blanco Abajo*: • 1 (0♀, 0♂) (0♀, 1♂); 07-SRNP-66255, DHJPAR0024793; rain forest; Malaise; 500 m; 10.90037, -85.37254; 08.x.2007; DH Janzen & W Hallwachs leg. • 1 (0♀, 0♂) (0♀, 1♂); 07-SRNP-66256, DHJPAR0024794; same data as for preceding. • 1 (0♀, 0♂) (0♀, 1♂); 07-SRNP-66332, DHJPAR0024870; same data as for preceding except: 27.viii.2007. • 1 (0♀, 0♂) (0♀, 1♂); 07-SRNP-66427, DHJPAR0024965; same data as for preceding except: 28.vii.2007. • 1 (0♀, 0♂) (0♀, 1♂); 07-SRNP-66492, DHJPAR0025030; same data as for preceding except: 16.vii.2007. • 1 (0♀, 1♂) (0♀, 0♂); 07-SRNP-66493, DHJPAR0025031; same data as for preceding except: 16.vii.2007. • 1 (0♀, 0♂) (0♀, 1♂); 07-SRNP-66586, DHJPAR0025124; same data as for preceding except: 04.vii.2007. • 1 (0♀, 0♂) (0♀, 1♂); 07-SRNP-66657, DHJPAR0025195; same data as for preceding except: 04.vii.2007. • 1 (0♀, 0♂) (0♀, 1♂); 07-SRNP-66672, DHJPAR0025210; same data as for preceding except: 22.vii.2007. • 1 (0♀, 0♂) (0♀, 1♂); 08-SRNP-2934, DHJPAR0026515; same data as for preceding except: 11.ii.2008. • 1 (0♀, 0♂) (0♀, 1♂); 08-SRNP-2958, DHJPAR0026539; same data as for preceding except: 17.ii.2008. • 1 (0♀, 0♂) (0♀, 1♂); 08-SRNP-3012, DHJPAR0026593; same data as for preceding except: 29.ii.2008. • 1 (0♀, 0♂) (0♀, 1♂); 08-SRNP-3246, DHJPAR0026827; same data as for preceding except: 30.iii.2008. • 1 (1♀, 0♂) (0♀, 0♂); 08-SRNP-3337, DHJPAR0026918; same data as for preceding except: 05.iv.2008. • 1 (0♀, 1♂) (0♀, 0♂); 08-SRNP-3388, DHJPAR0026969; same data as for preceding except: 11.iv.2008. • 1 (0♀, 1♂) (0♀, 0♂); 08-SRNP-3489, DHJPAR0027070; same data as for preceding except: 17.iv.2008. • 1 (0♀, 0♂) (0♀, 1♂); 08-SRNP-3537, DHJPAR0027118; same data as for preceding except: 23.iv.2008. • 1 (0♀, 0♂) (0♀, 1♂); 08-SRNP-3597, DHJPAR0027178; same data as for preceding except: 23.iv.2008. • 1 (1♀, 0♂) (0♀, 0♂); 08-SRNP-3598, DHJPAR0027179; same data as for preceding except: 23.iv.2008. • 1 (0♀, 0♂) (0♀, 1♂); 08-SRNP-3686, DHJPAR0027267; same data as for preceding except: 30.iv.2008. • 1 (0♀, 0♂) (0♀, 1♂); 08-SRNP-3700, DHJPAR0027281; same data as for preceding except: 06.v.2008. • 1 (0♀, 0♂) (0♀, 1♂); 08-SRNP-3741, DHJPAR0027322; same data as for preceding except: 06.v.2008. • 1 (0♀, 1♂) (0♀, 0♂); 08-SRNP-3760, DHJPAR0027341; same data as for preceding except: 06.v.2008. • 1 (0♀, 1♂) (0♀, 0♂); 08-SRNP-3863, DHJPAR0027444; same data as for preceding except: 18.v.2008.

*Área de Conservación Guanacaste*, *Alajuela*, *Sector San Cristóbal*, *Potrero Argentina*: • 1 (0♀, 1♂) (0♀, 0♂); 07-SRNP-67035, DHJPAR0025573; pastures; Malaise; 520 m; 10.89021, -85.38803; 04.vii.2007; DH Janzen & W Hallwachs leg.; specimen used for DNA extraction. • 1 (0♀, 1♂) (0♀, 0♂); 07-SRNP-67034, DHJPAR0025572; same data as for preceding. • 1 (0♀, 1♂) (0♀, 0♂); 07-SRNP-67036, DHJPAR0025574; same data as for preceding. • 1 (0♀, 0♂) (0♀, 1♂); 07-SRNP-67041, DHJPAR0025579; same data as for preceding. • 1 (0♀, 0♂) (0♀, 1♂); 07-SRNP-67044, DHJPAR0025582; same data as for preceding. • 1 (0♀, 1♂) (0♀, 0♂); 07-SRNP-67053, DHJPAR0025591; same data as for preceding except: 21.viii.2007. • 1 (0♀, 0♂) (0♀, 1♂); 07-SRNP-67063, DHJPAR0025601; same data as for preceding except: 15.viii.2007. • 1 (0♀, 1♂) (0♀, 0♂); 07-SRNP-67133, DHJPAR0025671; same data as for preceding except: 20.ix.2007. • 1 (0♀, 0♂) (0♀, 1♂); 07-SRNP-67134, DHJPAR0025672; same data as for preceding except: 20.ix.2007. • 1 (0♀, 0♂) (0♀, 1♂); 07-SRNP-67135, DHJPAR0025673; same data as for preceding except: 20.ix.2007. • 1 (0♀, 0♂) (0♀, 1♂); 07-SRNP-67136, DHJPAR0025674; same data as for preceding except: 20.ix.2007. • 1 (0♀, 0♂) (0♀, 1♂); 07-SRNP-67180, DHJPAR0025718; same data as for preceding except: 14.ix.2007. • 1 (0♀, 0♂) (0♀, 1♂); 07-SRNP-67190, DHJPAR0025728; same data as for preceding except: 14.ix.2007. • 1 (0♀, 0♂) (0♀, 1♂); 07-SRNP-67197, DHJPAR0025735; same data as for preceding. • 1 (0♀, 1♂) (0♀, 0♂); 07-SRNP-67209, DHJPAR0025747; same data as for preceding. • 1 (0♀, 0♂) (0♀, 1♂); 07-SRNP-67220, DHJPAR0025758; same data as for preceding except: 16.vi.2007. • 1 (0♀, 0♂) (0♀, 1♂); 07-SRNP-67252, DHJPAR0025790; same data as for preceding except: 26.ix.2007. • 1 (1♀, 0♂) (0♀, 0♂); 07-SRNP-67254, DHJPAR0025792; same data as for preceding except: 10.vi.2007. • 1 (0♀, 0♂) (0♀, 1♂); 07-SRNP-67262, DHJPAR0025800; same data as for preceding except: 22.vi.2007. • 1 (0♀, 0♂) (0♀, 1♂); 07-SRNP-67273, DHJPAR0025811; same data as for preceding except: 21.viii.2007. • 1 (0♀, 0♂) (0♀, 1♂); 07-SRNP-67736, DHJPAR0027474; same data as for preceding except: 03.viii.2007. • 1 (0♀, 0♂) (0♀, 1♂); 07-SRNP-67739, DHJPAR0027477; same data as for preceding except: 03.viii.2007. • 1 (0♀, 0♂) (0♀, 1♂); 07-SRNP-67747, DHJPAR0027485; same data as for preceding except: 03.viii.2007. • 1 (0♀, 0♂) (0♀, 1♂); 07-SRNP-67752, DHJPAR0027490; same data as for preceding except: 09.viii.2007. • 1 (0♀, 0♂) (0♀, 1♂); 07-SRNP-67759, DHJPAR0027497; same data as for preceding except: 09.viii.2007. • 1 (0♀, 0♂) (0♀, 1♂); 07-SRNP-67767, DHJPAR0027505; same data as for preceding except: 09.viii.2007.

*Área de Conservación Guanacaste*, *Alajuela*, *Sector San Cristóbal*, *Estación San Gerardo*: • 1 (0♀, 0♂) (0♀, 1♂); 07-SRNP-67294, DHJPAR0025832; rain forest; Malaise; 575 m; 10.88009, -85.38887; 04.vii.2007; DH Janzen & W Hallwachs leg. • 1 (0♀, 0♂) (0♀, 1♂), 07-SRNP-67324, DHJPAR0025862; same data as for preceding except: 16.vii.2007. • 1 (0♀, 0♂) (0♀, 1♂); 07-SRNP-67332, DHJPAR0025870; same data as for preceding except: 15.viii.2007. • 1 (0♀, 0♂) (0♀, 1♂); 07-SRNP-67337, DHJPAR0025875; same data as for preceding except: 15.viii.2007. • 1 (0♀, 1♂) (0♀, 0♂); 07-SRNP-67338, DHJPAR0025876; same data as for preceding except: 15.viii.2007. • 1 (0♀, 0♂) (0♀, 1♂); 07-SRNP-67339, DHJPAR0025877; same data as for preceding except: 15.viii.2007. • 1 (0♀, 1♂) (0♀, 0♂); 07-SRNP-67340, DHJPAR0025878; same data as for preceding except: 15.viii.2007. • 1 (0♀, 0♂) (0♀, 1♂); 07-SRNP-67341, DHJPAR0025879; same data as for preceding except: 15.viii.2007. • 1 (0♀, 0♂) (0♀, 1♂); 07-SRNP-67342, DHJPAR0025880; same data as for preceding except: 15.viii.2007. • 1 (0♀, 0♂) (0♀, 1♂); 07-SRNP-67343, DHJPAR0025881; same data as for preceding except: 15.viii.2007. • 1 (0♀, 1♂) (0♀, 0♂); 07-SRNP-67344, DHJPAR0025882; same data as for preceding except: 15.viii.2007. • 1 (0♀, 1♂) (0♀, 0♂); 07-SRNP-67346, DHJPAR0025884; same data as for preceding except: 15.viii.2007. • 1 (0♀, 1♂) (0♀, 0♂); 07-SRNP-67347, DHJPAR0025885; same data as for preceding except: 15.viii.2007. • 1 (0♀, 0♂) (0♀, 1♂); 07-SRNP-67351, DHJPAR0025889; same data as for preceding except: 15.viii.2007. • 1 (0♀, 0♂) (0♀, 1♂); 07-SRNP-67352, DHJPAR0025890; same data as for preceding except: 15.viii.2007.

*Área de Conservación Guanacaste*, *Alajuela*, *Sector San Cristóbal*, *Bosque Trampa Malaise*: • 1 (0♀, 0♂) (0♀, 1♂), 07-SRNP-67353, DHJPAR0025891; rain forest, 815 m, 10.86280, -85.38460, 22.vii.2007, DH Janzen & W Hallwachs leg. • 1 (0♀, 0♂) (0♀, 1♂); 07-SRNP-67356, DHJPAR0025894; same data as for preceding. • 1 (1♀, 0♂) (0♀, 0♂); 07-SRNP-67358, DHJPAR0025896; same data as for preceding. • 1 (0♀, 1♂) (0♀, 0♂); 07-SRNP-67359, DHJPAR0025897; same data as for preceding. • 1 (0♀, 0♂) (0♀, 1♂); 07-SRNP-67365, DHJPAR0025903; same data as for preceding. • 1 (0♀, 0♂) (0♀, 1♂); 07-SRNP-67367, DHJPAR0025905; same data as for preceding. • 1 (0♀, 1♂) (0♀, 0♂); 07-SRNP-67368, DHJPAR0025906; same data as for preceding. • 1 (0♀, 0♂) (0♀, 1♂); 07-SRNP-67374, DHJPAR0025912; same data as for preceding. • 1 (0♀, 0♂) (0♀, 1♂); 07-SRNP-67375, DHJPAR0025913; same data as for preceding. • 1 (0♀, 0♂) (0♀, 1♂); 07-SRNP-67376, DHJPAR0025914; same data as for preceding. • 1 (0♀, 0♂) (0♀, 1♂); 07-SRNP-67377, DHJPAR0025915; same data as for preceding except: 28.vii.2007. • 1 (0♀, 0♂) (0♀, 1♂); 07-SRNP-67378, DHJPAR0025916; same data as for preceding except: 28.vii.2007. • 1 (0♀, 0♂) (0♀, 1♂); 07-SRNP-67380, DHJPAR0025918; same data as for preceding except: 28.vii.2007. • 1 (0♀, 0♂) (0♀, 1♂); 07-SRNP-67381, DHJPAR0025919; same data as for preceding except: 28.vii.2007. • 1 (0♀, 0♂) (0♀, 1♂); 07-SRNP-67382, DHJPAR0025920; same data as for preceding except: 28.vii.2007. • 1 (0♀, 0♂) (0♀, 1♂); 07-SRNP-67389, DHJPAR0025927; same data as for preceding except: 28.vii.2007. • 1 (0♀, 0♂) (0♀, 1♂); 07-SRNP-67391, DHJPAR0025929; same data as for preceding except: 16.vi.2007. • 1 (0♀, 0♂) (0♀, 1♂); 07-SRNP-67392, DHJPAR0025930; same data as for preceding except: 16.vi.2007. • 1 (0♀, 0♂) (0♀, 1♂); 07-SRNP-67396, DHJPAR0025934; same data as for preceding except: 10.vii.2007. • 1 (0♀, 0♂) (0♀, 1♂); 07-SRNP-67397, DHJPAR0025935; same data as for preceding except: 10.vii.2007. • 1 (0♀, 0♂) (0♀, 1♂); 07-SRNP-67399, DHJPAR0025937; same data as for preceding except: 28.vii.2007. • 1 (0♀, 0♂) (0♀, 1♂); 07-SRNP-67400, DHJPAR0025938; same data as for preceding except: 27.viii.2007. • 1 (0♀, 0♂) (0♀, 1♂); 07-SRNP-67405, DHJPAR0025943; same data as for preceding except: 27.viii.2007. • 1 (0♀, 0♂) (0♀, 1♂); 07-SRNP-67406, DHJPAR0025944; same data as for preceding except: 27.viii.2007. • 1 (0♀, 1♂) (0♀, 0♂); 07-SRNP-67408, DHJPAR0025946; same data as for preceding except: 27.viii.2007. • 1 (0♀, 0♂) (0♀, 1♂); 07-SRNP-67411, DHJPAR0025949; same data as for preceding except: 27.viii.2007. • 1 (0♀, 0♂) (0♀, 1♂); 07-SRNP-67412, DHJPAR0025950; same data as for preceding except: 27.viii.2007. • 1 (0♀, 0♂) (0♀, 1♂); 07-SRNP-67417, DHJPAR0025955; same data as for preceding except: 27.viii.2007. • 1 (1♀, 0♂) (0♀, 0♂); 07-SRNP-67431, DHJPAR0025969; same data as for preceding except: 04.vii.2007. • 1 (0♀, 0♂) (0♀, 1♂); 07-SRNP-67432, DHJPAR0025970; same data as for preceding except: 04.vii.2007. • 1 (0♀, 1♂) (0♀, 0♂); 07-SRNP-67436, DHJPAR0025974; same data as for preceding except: 08.ix.2007. • 1 (0♀, 0♂) (0♀, 1♂); 07-SRNP-67440, DHJPAR0025978; same data as for preceding except: 08.ix.2007. • 1 (0♀, 0♂) (0♀, 1♂); 07-SRNP-67453, DHJPAR0025991; same data as for preceding except: 16.vi.2007. • 1 (0♀, 0♂) (0♀, 1♂); 07-SRNP-67458, DHJPAR0025996; same data as for preceding except: 16.vi.2007. • 1 (0♀, 0♂) (0♀, 1♂); 07-SRNP-67459, DHJPAR0025997; same data as for preceding except: 16.vi.2007. • 1 (0♀, 0♂) (0♀, 1♂); 07-SRNP-67462, DHJPAR0026000; same data as for preceding except: 16.vi.2007. • 1 (0♀, 0♂) (0♀, 1♂); 07-SRNP-67465, DHJPAR0026003; same data as for preceding except: 16.vi.2007. • 1 (0♀, 0♂) (0♀, 1♂); 07-SRNP-67467, DHJPAR0026005; same data as for preceding except: 14.ix.2007. • 1 (0♀, 0♂) (0♀, 1♂); 07-SRNP-67475, DHJPAR0026013; same data as for preceding except: 02.ix.2007. • 1 (0♀, 0♂) (0♀, 1♂); 07-SRNP-67476, DHJPAR0026014; same data as for preceding except: 02.ix.2007. • 1 (0♀, 0♂) (0♀, 1♂); 07-SRNP-67479, DHJPAR0026017; same data as for preceding except: 02.ix.2007. • 1 (0♀, 0♂) (0♀, 1♂); 07-SRNP-67481, DHJPAR0026019; same data as for preceding except: 02.ix.2007. • 1 (0♀, 0♂) (0♀, 1♂); 07-SRNP-67486, DHJPAR0026024; same data as for preceding except: 02.ix.2007. • 1 (0♀, 0♂) (0♀, 1♂); 07-SRNP-67490, DHJPAR0026028; same data as for preceding except: 02.ix.2007. • 1 (0♀, 0♂) (0♀, 1♂); 07-SRNP-67494, DHJPAR0026032; same data as for preceding except: 05.vi.2007. • 1 (0♀, 0♂) (0♀, 1♂); 07-SRNP-67498, DHJPAR0026036; same data as for preceding except: 19.xii.2007. • 1 (0♀, 0♂) (0♀, 1♂); 07-SRNP-67499, DHJPAR0026037; same data as for preceding except: 10.vii.2007. • 1 (0♀, 0♂) (0♀, 1♂); 07-SRNP-67504, DHJPAR0026042; same data as for preceding except: 10.vii.2007. • 1 (0♀, 0♂) (0♀, 1♂); 07-SRNP-67505, DHJPAR0026043; same data as for preceding except: 10.vii.2007. • 1 (0♀, 0♂) (0♀, 1♂); 07-SRNP-67508, DHJPAR0026046; same data as for preceding except: 26.ix.2007. • 1 (0♀, 0♂) (0♀, 1♂); 07-SRNP-67510, DHJPAR0026048; same data as for preceding except: 26.ix.2007. • 1 (0♀, 0♂) (0♀, 1♂); 07-SRNP-67511, DHJPAR0026049; same data as for preceding except: 26.ix.2007. • 1 (0♀, 0♂) (0♀, 1♂); 07-SRNP-67512, DHJPAR0026050; same data as for preceding except: 21.viii.2007. • 1 (0♀, 0♂) (0♀, 1♂); 07-SRNP-67513, DHJPAR0026051; same data as for preceding except: 21.viii.2007. • 1 (0♀, 0♂) (0♀, 1♂); 07-SRNP-67514, DHJPAR0026052; same data as for preceding except: 21.viii.2007. • 1 (0♀, 0♂) (0♀, 1♂); 07-SRNP-67515, DHJPAR0026053; same data as for preceding except: 21.viii.2007. • 1 (0♀, 0♂) (0♀, 1♂); 07-SRNP-67520, DHJPAR0026058; same data as for preceding except: 21.viii.2007. • 1 (0♀, 0♂) (0♀, 1♂); 07-SRNP-67523, DHJPAR0026061; same data as for preceding except: 21.viii.2007. • 1 (0♀, 0♂) (0♀, 1♂); 07-SRNP-67535, DHJPAR0026073; same data as for preceding except: 26.x.2007. • 1 (0♀, 0♂) (0♀, 1♂); 07-SRNP-67536, DHJPAR0026074; same data as for preceding except: 26.x.2007. • 1 (0♀, 0♂) (0♀, 1♂); 07-SRNP-67540, DHJPAR0026078; same data as for preceding except: 26.x.2007. • 1 (0♀, 0♂) (0♀, 1♂); 07-SRNP-67542, DHJPAR0026080; same data as for preceding except: 10.vi.2007. • 1 (0♀, 0♂) (0♀, 1♂); 07-SRNP-67544, DHJPAR0026082; same data as for preceding except: 10.vi.2007. • 1 (0♀, 0♂) (0♀, 1♂); 07-SRNP-67548, DHJPAR0026086; same data as for preceding except: 10.vi.2007. • 1 (0♀, 0♂) (0♀, 1♂); 07-SRNP-67549, DHJPAR0026087; same data as for preceding except: 10.vi.2007. • 1 (0♀, 0♂) (0♀, 1♂); 07-SRNP-67809, DHJPAR0027605; same data as for preceding except: 03.viii.2007. • 1 (1♀, 0♂) (0♀, 0♂); 07-SRNP-67822, DHJPAR0027618; same data as for preceding except: 09.viii.2007. • 1 (0♀, 0♂) (0♀, 1♂); 07-SRNP-67829, DHJPAR0027625; same data as for preceding except: 09.viii.2007. • 1 (0♀, 0♂) (0♀, 1♂); 07-SRNP-67830, DHJPAR0027626; same data as for preceding except: 09.viii.2007. • 1 (0♀, 0♂) (0♀, 1♂); 07-SRNP-67832, DHJPAR0027628; same data as for preceding except: 09.viii.2007. • 1 (0♀, 0♂) (0♀, 1♂); 07-SRNP-67835, DHJPAR0027631; same data as for preceding except: 09.viii.2007. • 1 (1♀, 0♂) (0♀, 0♂); 08-SRNP-3943, DHJPAR0027645; same data as for preceding except: 12.i.2008. • 1 (0♀, 0♂) (0♀, 1♂); 08-SRNP-3986, DHJPAR0027688; same data as for preceding except: 06.v.2008. • 1 (0♀, 0♂) (0♀, 1♂); 08-SRNP-4001, DHJPAR0027703; same data as for preceding except: 21.v.2008. • 1 (0♀, 0♂) (0♀, 1♂); 08-SRNP-4002, DHJPAR0027704; same data as for preceding except: 21.v.2008.

*Área de Conservación Guanacaste*, *Guanacaste*, *Sector Cacao*, *Sendero Cima*: • 1 (0♀, 1♂) (0♀, 0♂); 99-SRNP-19238, DHJPAR0013475; cloud forest; Malaise; 1,460 m; 10.93328, -85.45729; 25.v.1999; DH Janzen & W Hallwachs leg.

*Área de Conservación Guanacaste*, *Alajuela*, *Sector Rincón Rain Forest*, *Vado Río Francia*: • 1 (0♀, 1♂) (0♀, 0♂); 07-SRNP-66867, DHJPAR0025405; Malaise; 400 m; 10.90093, -85.28915; 15.ix.2007; DH Janzen & W Hallwachs leg. • 1 (0♀, 0♂) (0♀, 1♂); 07-SRNP-67029, DHJPAR0025567; same data as for preceding except: 04.vii.2007.

#### Diagnosis.

**Figure 203.**
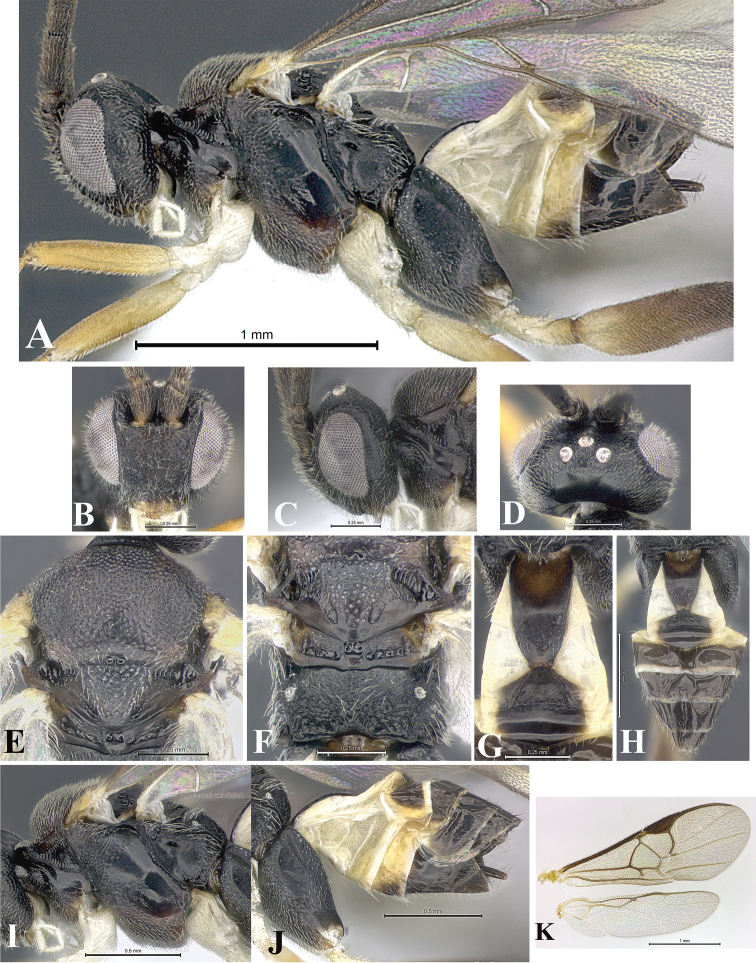
*Glyptapantelessondrawardae* sp. nov. female 07-SRNP-66795 DHJPAR0025333 **A** Habitus **B, D** Head **B** Frontal view **D** Dorsal view **C** Head, pronotum, propleuron, lateral view **E** Mesonotum, dorsal view **F** Scutellum, metanotum, propodeum, dorsal view **G**T1–3, dorsal view **H, J** Metasoma **H** Dorsal view **J** Lateral view **I** Mesosoma, lateral view **D** Fore and hind wings, male 07-SRNP-67035 DHJPAR0025573.

Propleuron with a mix of rugae and fine punctation, dorsal carina delimiting a dorsal furrow present (Fig. 203A, C, I), antenna longer than body, anterior furrow of metanotum with a small lobe, without setae (Fig. 203F), distal antennal flagellomere longer than penultimate, surface of metasternum convex, precoxal groove deep with lineate sculpture (Fig. 203A, I), fore wing with vein 1 cu-a curved, r vein slightly curved or curved (Fig. 203K), dorsal outer depression on hind coxa present (Fig. 203A, J), inner margin of eyes diverging slightly at antennal sockets (Fig. 203B), petiole on T1 finely sculptured (Fig. 203G, H), and lateral grooves delimiting the median area on T2 clearly defined and reaching the distal edge of T2 (Fig. 203G, H).

#### Coloration

(Fig. 203A–K). General body coloration black except scape brown, but proximally with a yellow ring; pedicel brown, but distally yellow; first five proximal antennal flagellomeres dorsally lighter (light brown) than ventrally (dark brown), remaining flagellomeres dark brown on both sides; labrum, mandible, and tegulae yellow; medially low face just below antennal socket (toruli), propleuron distally, dorsal furrow of pronotum, latero-ventral part of mesopleuron, mesosternum, mesoscutum with two longitudinal bands taking the place of notauli, edges postero-distal of mesoscutum, lunules, and lateral ends of metanotum with yellow-brown/reddish tints; glossa, maxillary and labial palps pale yellow/ivory. Eyes and ocelli silver. Fore and middle legs dark yellow, except coxae and trochanters pale yellow/ivory; hind legs with black coxae, trochanters and trochantellus dark yellow, femora proximally yellow, medially yellow-brown to brown and distally brown, tibial spurs yellow, tibiae yellow-brown with both ends brown, and tarsomeres yellow-brown. Petiole on T1 with two colorations: distal 3/4 dark brown and proximal 1/4 yellow-brown/reddish, contours darkened, and sublateral areas ivory/pale yellow; T2 with median and adjacent areas brown, and lateral ends ivory/pale yellow; T3 medially brown forming a dark area which proximally coinciding with the width of median and adjacent areas on T2; however, this dark area not reaching distal edge of T3, instead there is a pale-yellow/ivory band, and lateral ends ivory/pale yellow; T4 and beyond completely brown; distally each tergum with a narrow ivory/pale yellow transparent band. In lateral view, T1–2 completely ivory/pale yellow; T3 yellow, but dorsally brown; T4 and beyond completely brown. S1–4 ivory/yellow; penultimate sternum yellow, but medially with a brown area; hypopygium brown.

#### Description.

**Head** (Fig. 203A–D) Head rounded with pubescence short and dense. Proximal three antennal flagellomeres longer than wide (0.24:0.09, 0.25:0.09, 0.25:0.09), distal antennal flagellomere longer than penultimate (0.17:0.08, 0.11:0.09), antenna longer than body (3.38, 2.73); antennal scrobes-frons shallow. Face convex, punctate-lacunose, interspaces wavy and longitudinal median carina present. Frons smooth. Temple wide, punctate-lacunose and interspaces wavy. Inner margin of eyes diverging slightly at antennal sockets; in lateral view, eye anteriorly convex and posteriorly straight. POL shorter than OOL (0.09, 0.13). Malar suture present. Median area between lateral ocelli slightly depressed. Vertex laterally pointed or nearly so and dorsally wide.

**Mesosoma** (Fig. 203A, E, F, I). Mesosoma dorsoventrally convex. Distal 1/3 of mesoscutum with lateral margin slightly dented, punctation distinct throughout, interspaces wavy/lacunose. Scutellum triangular, apex sloped and fused with BS, phragma of the scutellum partially exposed, scutellar punctation distinct throughout, in profile scutellum flat and on same plane as mesoscutum; BS only very partially overlapping the MPM; ATS demilune with quite a little, complete and parallel carinae; dorsal ATS groove with semicircular/parallel carinae. Transscutal articulation with small and heterogeneous foveae, area just behind transscutal articulation smooth, shiny and depressed centrally. Metanotum with BM wider than PFM (clearly differentiated); MPM circular without median longitudinal carina; AFM with a small lobe and not as well delineated as PFM; PFM thick, smooth and with lateral ends rounded; ATM proximally with a well-defined row of foveae and distally smooth. Propodeum without median longitudinal carina, proximal half curved with medium-sized sculpture and distal half rugose; distal edge of propodeum with a flange at each side and without stubs; propodeal spiracle distally framed by a short concave carina; nucha surrounded by very short radiating carinae. Pronotum with a distinct dorsal furrow, dorsally with a well-defined smooth band; central area of pronotum smooth, but both dorsal and ventral furrows with short parallel carinae. Propleuron with a mix of rugae and fine punctation, dorsally with a carina. Metasternum convex. Contour of mesopleuron convex; precoxal groove deep with faintly lineate sculpture; epicnemial ridge convex, teardrop-shaped.

**Legs.** Ventral margin of fore telotarsus entire without seta, fore telotarsus almost same width throughout and longer than fourth tarsomere (0.12, 0.07). Medially hind coxa smooth, dorsally with scattered punctation and ventrally with dense punctation, dorsal outer depression present. Inner spur of hind tibia longer than outer spur (0.29, 0.21), entire surface of hind tibia with dense strong spines clearly differentiated by color and length. Hind telotarsus longer than fourth tarsomere (0.16, 0.14).

**Wings** (Fig. 203K). Fore wing with r vein slightly curved; 2RS vein straight; r and 2RS veins forming a weak, even curve at their junction and outer side of junction not forming a stub; 2M vein slightly curved/swollen; distally fore wing [where spectral veins are] with microtrichiae more densely concentrated than the rest of the wing; anal cell 1/3 proximally lacking microtrichiae; subbasal cell with microtrichiae virtually throughout; veins 2CUa and 2CUb completely spectral; vein 2 cu-a absent; vein 2-1A proximally tubular and distally spectral, although sometimes difficult to see; tubular vein 1 cu-a curved and complete, but junction with 1-1A vein spectral. Hind wing with vannal lobe narrow, subdistally concave and subproximally straightened, and setae evenly scattered in the margin.

**Metasoma** (Fig. 203A, G, H, K). Metasoma laterally compressed. Petiole on T1 finely sculptured only laterally, evenly narrowing distally (length 0.41, maximum width 0.23, minimum width 0.09) and with scattered pubescence concentrated in the first distal third. Lateral grooves delimiting the median area on T2 clearly defined and reaching the distal edge of T2 (length median area 0.14, length T2 0.14), edges of median area polished and lateral grooves deep, median area broader than long (length 0.14, maximum width 0.20, minimum width 0.07); T2 with scattered pubescence only distally. T3 longer than T2 (0.22, 0.14) and with scattered pubescence throughout. Pubescence on hypopygium dense.

**Cocoons.** Unknown.

#### Comments.

Additionally, to the described coloration, some females have mesoscutum with three parallel yellow-brown/reddish bands which merging in the 1/4 distal; the apex of the scutellum and the epicnemial furrow are yellow-brown/reddish (e.g., 07-SRNP-67822, 08-SRNP-3943). One antenna broken off on the holotype.

#### Male.

Similar in coloration to female. As well as females, some males with three distinctive parallel yellow-brown/reddish bands in the mesoscutum. In some specimens, the petiole is completely black. In some cases, the coloration in the mesoscutum and the mesopleuron is lighter than in other specimens. The clypeus and the middle area of low face, just above the clypeus, the PFM and the BS are orange/reddish; most of the mesoscutum is orange/reddish, but distally with a dark middle area.

#### Etymology.

Sondra L. Ward research has contributed to the understanding of the ichneumonid fauna from Costa Rica.

#### Distribution.

The adult parasitoids were collected in Costa Rica, ACG, Sector Cacao (Sendero Cima), Sector Rincón Rain Forest (Vado Río Francia), and Sector San Cristóbal (Bosque Trampa Malaise, Estación San Gerardo, Potrero Argentina, and Río Blanco Abajo), during May 1999, June-October and December 2007, and January-May 2008 at 400 m, 500 m, 520 m, 575 m, 815 m, and 1,460 m in rain and cloud forests.

#### Biology.

Unknown.

#### Host.

Unknown.

### Glyptapanteles stephaniecluttsae

Taxon classificationAnimaliaHymenopteraBraconidae

Arias-Penna, sp. nov.

http://zoobank.org/7E0080CB-6ED7-4060-ACAC-4E5F7E277114

Figs 204 , 205

#### Female.

Body length 2.17 mm, antenna length 2.73 mm [distal flagellomere is missing], fore wing length 2.48 mm.

#### Type material.

**Holotype**: COSTA RICA •1♀; 07-SRNP-2903, DHJPAR0020275; Área de Conservación Guanacaste, Guanacaste, Sector San Cristóbal, Sendero Vivero; rain forest; 730 m; 10.86739, -85.38744; 22.vi.2007; Elda Araya leg.; caterpillar collected in fourth instar; dark brown hard cocoons adhered to the leaf substrate; (CNC). **Paratypes.** • 5 (0♀, 1♂) (4♀, 0♂); 07-SRNP-2903, DHJPAR0020275; same data as for holotype; (CNC).

#### Diagnosis.

**Figure 204.**
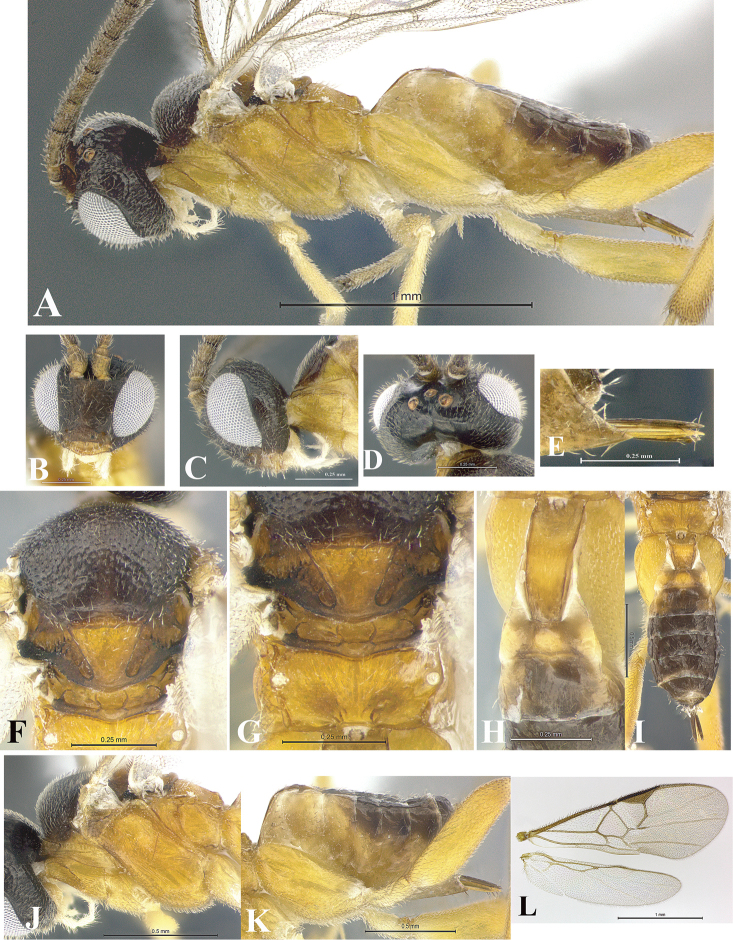
*Glyptapantelesstephaniecluttsae* sp. nov. female 07-SRNP-2903 DHJPAR0020275 **A** Habitus **B, D** Head **B** Frontal view **D** Dorsal view **C** Head, pronotum, propleuron, lateral view **E** Genitalia: hypopygium, ovipositor, ovipositor sheaths, lateral view **F** Mesonotum, dorsal view **G** Scutellum, metanotum, propodeum, dorsal view **H**T1–3, dorsal view **I, K** Metasoma **I** Dorsal view **K** Lateral view **J** Mesosoma, lateral view **L** Fore and hind wings.

**Figure 205.**
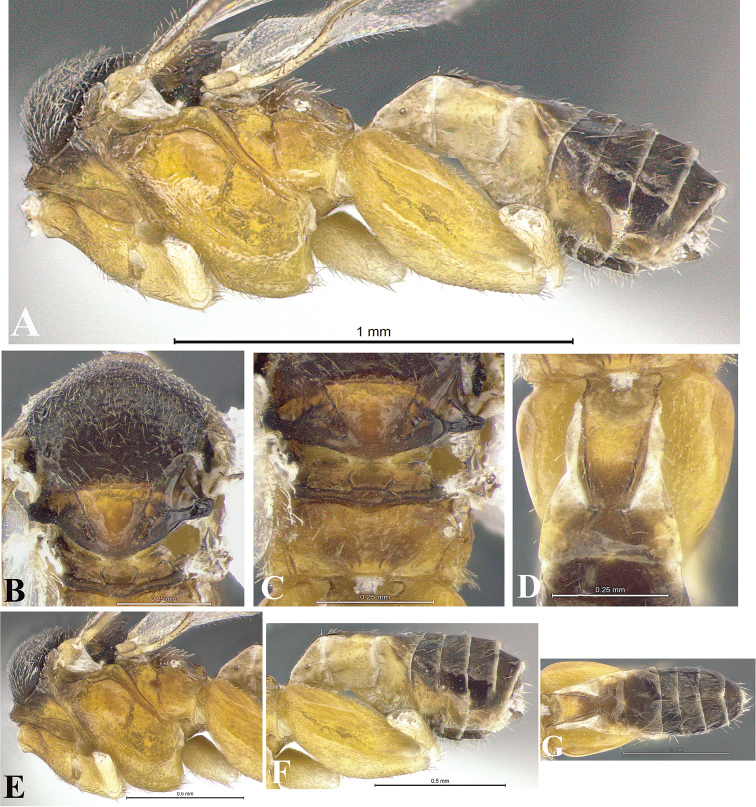
*Glyptapantelesstephaniecluttsae* sp. nov. male 07-SRNP-2903 DHJPAR0020275 **A** Habitus **B** Mesonotum, dorsal view **C** Scutellum, metanotum, propodeum, dorsal view **D**T1–2, dorsal view **E** Mesosoma, lateral view **F, G** Metasoma **F** Lateral view **G** Dorsal view.

Lateral grooves delimiting the median area on T2 distally losing definition, edges of median area on T2 polished and followed by a deep groove (Figs 204H, 205D), axillary trough of metanotum completely smooth (Figs 204G, 205C), precoxal groove shallow, but visible (Figs 204J, 205E), anteroventral contour of mesopleuron straight/angulate or nearly so (Figs 204J, 205E), and fore wing with r vein curved, outer side of junction of r and 2RS veins forming a distinct stub (Fig. 204L).

#### Coloration

(Fig. 204A–L). General body coloration yellow except dark brown-black head; scape and pedicel yellow-brown; all antennal flagellomeres dorsally lighter (light brown) that ventrally (dark brown); tegulae yellow; mesoscutum brown; lunules and area between lunules, ATS groove, PFM dark brown-black; glossa, maxillary and labial palps pale yellow/ivory. Eyes silver and ocelli yellow. Fore and middle legs dark yellow except brown claws; hind legs dark yellow except tibiae distally light brown and tarsomeres brown. Petiole on T1 yellow, contours yellow-brown, and sublateral areas ivory/pale yellow; T2 with median area yellow, adjacent area yellow-brown, and lateral ends yellow; T3 yellow-brown with brown tints; T4 and beyond completely brown; distally each tergum with a narrow hyaline band. In lateral view, T1–3 dark yellow; T4 and beyond yellow, but dorsally yellow-brown. S1–5 yellow; hypopygium yellow-brown.

#### Description.

**Head** (Fig. 204A–D). Head rounded with pubescence long and dense. Proximal three antennal flagellomeres longer than wide (0.19:0.06, 0.20:0.06, 0.20:0.06), antenna longer than body (2.73 [distal antennal flagellomere missing], 2.17); antennal scrobes-frons shallow. Face convex, punctate-lacunose, interspaces wavy and longitudinal median carina present. Frons smooth. Temple wide, punctate-lacunose and interspaces wavy. Inner margin of eyes diverging slightly at antennal sockets; in lateral view, eye anteriorly convex and posteriorly straight. POL shorter than OOL (0.08, 0.11). Malar suture present. Median area between lateral ocelli without depression. Vertex laterally pointed or nearly so and dorsally wide.

**Mesosoma** (Fig. 204A, F, G, J). Mesosoma dorsoventrally convex. Distal 1/3 of mesoscutum with lateral margin slightly dented, punctation proximally distinct, but distally with a polished area, interspaces wavy/lacunose. Scutellum triangular, apex sloped and fused with BS, scutellar punctation indistinct throughout, in profile scutellum flat and on same plane as mesoscutum; phragma of the scutellum partially exposed; BS not overlapping the MPM; ATS demilune with a little, incomplete and parallel carinae only proximally; dorsal ATS groove with carinae only proximally. Transscutal articulation with small and heterogeneous foveae, area just behind transscutal articulation with a smooth and shiny sloped transverse strip. Metanotum with BM upward; MPM circular without median longitudinal carina; AFM with a small lobe and not as well delineated as PFM; PFM thick, smooth and with a proximal flat flange; ATM completely smooth. Propodeum with indistinct sculpture and without median longitudinal carina, proximal half weakly curved; distal edge of propodeum with a flange at each side and without stubs; propodeal spiracle without distal carina; nucha surrounded by very short radiating carinae. Pronotum with a distinct dorsal furrow, dorsally with a well-defined smooth band; central area of pronotum and dorsal furrow smooth, but ventral furrow with short parallel carinae. Propleuron with a mix of rugae and fine punctation, dorsally with a carina. Metasternum flat or nearly so. Contour of mesopleuron straight/angulate or nearly so; precoxal groove smooth, shiny and shallow, but visible; epicnemial ridge convex, teardrop-shaped.

**Legs** (Fig. 204A). Ventral margin of fore telotarsus entire without seta, fore telotarsus almost same width throughout and longer than fourth tarsomere (0.11, 0.08). Hind coxa with punctation only on ventral surface, dorsal outer depression present. Inner spur of hind tibia longer than outer spur (0.25, 0.17), entire surface of hind tibia with dense strong spines clearly differentiated by color and length. Hind telotarsus longer than fourth tarsomere (0.14, 0.11).

**Wings** (Fig. 204L). Fore wing with r vein slightly curved; 2RS vein straight; r and 2RS veins forming a weak, even curve at their junction and outer side of junction forming a slight stub; 2M vein straight; distally fore wing [where spectral veins are] with microtrichiae more densely concentrated than the rest of the wing; anal cell 1/3 proximally lacking microtrichiae; subbasal cell with microtrichiae virtually throughout; veins 2CUa and 2CUb completely spectral; vein 2 cu-a present as spectral vein, sometimes difficult to see; vein 2-1A proximally tubular and distally spectral, although sometimes difficult to see; tubular vein 1 cu-a curved and complete, but junction with 1-1A vein spectral. Hind wing with vannal lobe narrow, subdistally and subproximally straightened, and setae present proximally, but absent distally.

**Metasoma** (Fig. 204A, E, H, I, K). Metasoma laterally compressed. Petiole on T1 completely smooth and polished, with faint, satin-like sheen, with parallel-sided in proximal half and then narrowing (length 0.35, maximum width 0.15, minimum width 0.09), and with scattered pubescence concentrated in the first distal third. Lateral grooves delimiting the median area on T2 distally losing definition (length median area 0.06, length T2 0.10), edges of median area polished and lateral grooves deep, median area broader than long (length 0.06, maximum width 0.12, minimum width 0.08); T2 with scattered pubescence only distally. T3 longer than T2 (0.20, 0.10) and with scattered pubescence throughout. Pubescence on hypopygium dense.

**Cocoons.** Dark brown oval cocoons adhered to the leaf substrate.

#### Male

(Fig. 205A–G). Similar in coloration to female. The head and the legs are missing.

#### Etymology.

Stephanie A. Clutts is a research analyst at the University of Kentucky, Lexington, KY, USA. Her primary responsibilities include obtaining and analyzing molecular data related to the revision of Agathidinae wasps (Braconidae) and maintaining and distributing samples pertaining to the TIGER project (Thailand Inventory Group for Entomological Research).

#### Distribution.

The parasitized caterpillar was collected in Costa Rica, ACG, Sector San Cristóbal (Sendero Vivero), during June 2007 at 730 m in rain forest.

#### Biology.

The lifestyle of this parasitoid species is gregarious.

#### Host.

*Bertholdiaalbipuncta* Schaus (Erebidae: Arctiinae) feeding on *Guazumaulmifolia* (Malvaceae). Caterpillar was collected in fourth instar.

### Glyptapanteles stephaniekirkae

Taxon classificationAnimaliaHymenopteraBraconidae

Arias-Penna, sp. nov.

http://zoobank.org/FD1C87C8-93C9-45C4-B49A-A086DB55AEB9

Figs 206 , 207

#### Female.

Body length 2.98 mm, antenna length 3.78 mm, fore wing length 2.88 mm.

#### Type material.

**Holotype**: COSTA RICA • 1♀; 07-SRNP-67233, DHJPAR0025771; Área de Conservación Guanacaste, Alajuela, Sector San Cristóbal, Potrero Argentina; pastures; Malaise; 520 m; 10.89021, -85.38803; 08.x.2007; DH Janzen & W Hallwachs leg.; (CNC). **Paratypes**. • 1 (0♀, 0♂) (0♀, 1♂); 07-SRNP-67114, DHJPAR0025652; same data as for holotype except: 28.xii.2007; (CNC). • 1 (0♀, 0♂) (0♀, 1♂); 07-SRNP-67119, DHJPAR0025657; same data as for holotype except: 28.xii.2007; (CNC). • 1 (0♀, 0♂) (0♀, 1♂); 07-SRNP-67128, DHJPAR0025666; same data as for holotype except: 28.xii.2007; (CNC). • 1 (0♀, 0♂) (0♀, 1♂); 07-SRNP-67129, DHJPAR0025667; same data as for holotype except: 28.xii.2007; (CNC).

#### Other material.

**Malaise-trapped material.** COSTA RICA: *Área de Conservación Guanacaste*, *Alajuela*, *Sector San Cristóbal*, *Bosque Trampa Malaise*: • 1 (1♀, 0♂) (0♀, 0♂); 07-SRNP-67444, DHJPAR0025982; 815 m; 10.86280, -85.38460; 02.x.2007; DH Janzen & W Hallwachs leg.

*Área de Conservación Guanacaste*, *Alajuela*, *Sector San Cristóbal*, *Río Blanco Abajo*: • 1 (0♀, 0♂) (0♀, 1♂); 07-SRNP-66226, DHJPAR0024764; rain forest; Malaise; 500 m; 10.90037, -85.37254; 08.viii.2007; DH Janzen & W Hallwachs leg. • 1 (0♀, 0♂) (0♀, 1♂); 07-SRNP-66306, DHJPAR0024844; same data as for preceding except: 15.viii.2007. • 1 (0♀, 0♂) (0♀, 1♂); 07-SRNP-66335, DHJPAR0024873; same data as for preceding except: 27.viii.2007. • 1 (0♀, 1♂) (0♀, 0♂); 07-SRNP-66363, DHJPAR0024901; same data as for preceding except: 02.x.2007. • 1 (0♀, 0♂) (0♀, 1♂); 07-SRNP-66395, DHJPAR0024933; same data as for preceding except: 21.viii.2007. • 1 (0♀, 0♂) (1♀, 0♂); 07-SRNP-66432, DHJPAR0024970; same data as for preceding except: 28.vii.2007. • 1 (1♀, 0♂) (0♀, 0♂); 07-SRNP-66457, DHJPAR0024995; same data as for preceding except: 19.xi.2007. • 1 (0♀, 0♂) (0♀, 1♂); 07-SRNP-66477, DHJPAR0025015; same data as for preceding except: 27.viii.2007. • 1 (0♀, 0♂) (0♀, 1♂); 07-SRNP-66499, DHJPAR0025037; same data as for preceding except: 16.vii.2007. • 1 (0♀, 0♂) (0♀, 1♂), 07-SRNP-66501, DHJPAR0025039; same data as for preceding except: 16.vii.2007. • 1 (0♀, 0♂) (0♀, 1♂); 07-SRNP-66508, DHJPAR0025046; same data as for preceding except: 16.vii.2007. • 1 (0♀, 0♂) (0♀, 1♂); 07-SRNP-66515, DHJPAR0025053; same data as for preceding except: 28.vii.2007. • 1 (0♀, 0♂) (0♀, 1♂); 07-SRNP-66519, DHJPAR0025057; same data as for preceding except: 28.vii.2007. • 1 (0♀, 0♂) (0♀, 1♂); 07-SRNP-66526, DHJPAR0025064; same data as for preceding except: 22.vi.2007. • 1 (0♀, 0♂) (0♀, 1♂); 07-SRNP-66535, DHJPAR0025073; same data as for preceding except: 22.vi.2007. • 1 (0♀, 0♂) (0♀, 1♂); 07-SRNP-66576, DHJPAR0025114; same data as for preceding except: 04.vii.2007. • 1 (0♀, 0♂) (0♀, 1♂); 07-SRNP-66581, DHJPAR0025119; same data as for preceding except: 04.vii.2007. • 1 (0♀, 0♂) (0♀, 1♂); 07-SRNP-66603, DHJPAR0025141; same data as for preceding except: 04.vii.2007. • 1 (0♀, 0♂) (0♀, 1♂); 07-SRNP-66604, DHJPAR0025142; same data as for preceding except: 04.vii.2007. • 1 (0♀, 0♂) (0♀, 1♂); 07-SRNP-66635, DHJPAR0025173; same data as for preceding except: 04.vii.2007. • 1 (0♀, 0♂) (0♀, 1♂); 07-SRNP-66646, DHJPAR0025184; same data as for preceding except: 04.vii.2007. • 1 (0♀, 0♂) (0♀, 1♂); 07-SRNP-66647, DHJPAR0025185; same data as for preceding except: 04.vii.2007. • 1 (0♀, 1♂) (0♀, 0♂); 07-SRNP-66695, DHJPAR0025233; same data as for preceding except: 22.vii.2007. • 1 (0♀, 0♂) (0♀, 1♂); 07-SRNP-66699, DHJPAR0025237; same data as for preceding except: 22.vii.2007. • 1 (0♀, 0♂) (0♀, 1♂); 07-SRNP-66701, DHJPAR0025239; same data as for preceding except: 22.vii.2007. • 1 (0♀, 0♂) (0♀, 1♂); 07-SRNP-66749, DHJPAR0025287; same data as for preceding except: 02.ix.2007. • 1 (0♀, 0♂) (0♀, 1♂); 07-SRNP-66782, DHJPAR0025320; same data as for preceding except: 16.vi.2007. • 1 (0♀, 0♂) (0♀, 1♂); 07-SRNP-66785, DHJPAR0025323; same data as for preceding except: 16.vi.2007. • 1 (0♀, 0♂) (0♀, 1♂); 07-SRNP-66802, DHJPAR0025340; same data as for preceding except: 16.vi.2007. • 1 (0♀, 0♂) (0♀, 1♂); 07-SRNP-67590, DHJPAR0026285; same data as for preceding except: 03.viii.2007. • 1 (0♀, 0♂) (1♀, 0♂); 07-SRNP-67596, DHJPAR0026291; same data as for preceding except: 03.viii.2007. • 1 (0♀, 0♂) (0♀, 1♂); 07-SRNP-67677, DHJPAR0026372; same data as for preceding except: 09.viii.2007. • 1 (0♀, 0♂) (0♀, 1♂); 07-SRNP-67681, DHJPAR0026376; same data as for preceding except: 09.viii.2007. • 1 (0♀, 0♂) (0♀, 1♂); 07-SRNP-67693, DHJPAR0026388; same data as for preceding except: 01.xi.2007. • 1 (1♀, 0♂) (0♀, 0♂); 08-SRNP-2877, DHJPAR0026458; same data as for preceding except: 30.i.2008. • 1 (0♀, 0♂) (0♀, 1♂); 08-SRNP-2898, DHJPAR0026479; same data as for preceding except: 05.ii.2008. • 1 (0♀, 0♂) (0♀, 1♂); 08-SRNP-2983, DHJPAR0026564; same data as for preceding except: 23.ii.2008. • 1 (0♀, 0♂) (0♀, 1♂); 08-SRNP-3010, DHJPAR0026591; same data as for preceding except: 29.ii.2008. • 1 (0♀, 0♂) (0♀, 1♂); 08-SRNP-3239, DHJPAR0026820; same data as for preceding except: 30.iii.2008. • 1 (0♀, 0♂) (0♀, 1♂); 08-SRNP-3335, DHJPAR0026916; same data as for preceding except: 05.iv.2008. • 1 (0♀, 1♂) (0♀, 0♂); 08-SRNP-3378, DHJPAR0026959; same data as for preceding except: 11.iv.2008. • 1 (0♀, 0♂) (0♀, 1♂); 08-SRNP-3408, DHJPAR0026989; same data as for preceding except: 11.iv.2008. • 1 (0♀, 0♂) (0♀, 1♂); 08-SRNP-3431, DHJPAR0027012; same data as for preceding except: 11.iv.2008. • 1 (0♀, 1♂) (0♀, 0♂); 08-SRNP-3460, DHJPAR0027041; same data as for preceding except: 11.iv.2008. • 1 (0♀, 0♂) (0♀, 1♂); 08-SRNP-3488, DHJPAR0027069; same data as for preceding except: 17.iv.2008. • 1 (0♀, 0♂) (0♀, 1♂); 08-SRNP-3582, DHJPAR0027163; same data as for preceding except: 23.iv.2008. • 1 (0♀, 0♂) (0♀, 1♂); 08-SRNP-3645, DHJPAR0027226; same data as for preceding except: 23.iv.2008. • 1 (1♀, 0♂) (0♀, 0♂); 08-SRNP-3729, DHJPAR0027310; same data as for preceding except: 06.v.2008. • 1 (1♀, 0♂) (0♀, 0♂); 08-SRNP-3790, DHJPAR0027371; same data as for preceding except: 12.v.2008. • 1 (1♀, 0♂) (0♀, 0♂); 08-SRNP-3799, DHJPAR0027380; same data as for preceding except: 12.v.2008. • 1 (0♀, 0♂) (0♀, 1♂); 08-SRNP-3800, DHJPAR0027381; same data as for preceding except: 12.v.2008.

*Área de Conservación Guanacaste*, *Alajuela*, *Sector San Cristóbal*, *Potrero Argentina*: • 1 (0♀, 0♂) (0♀, 1♂); 07-SRNP-67019, DHJPAR0025557, pastures; Malaise; 520 m; 10.89021, -85.38803; 25.xii.2007; DH Janzen & W Hallwachs leg. • 1 (0♀, 0♂) (0♀, 1♂); 07-SRNP-67028, DHJPAR0025566; same data as for preceding except: 04.vii.2007. • 1 (1♀, 0♂) (0♀, 0♂); 07-SRNP-67040, DHJPAR0025578; same data as for preceding except: 04.vii.2007. • 1 (0♀, 0♂) (0♀, 1♂); 07-SRNP-67064, DHJPAR0025602; same data as for preceding except: 15.viii.2007. • 1 (0♀, 0♂) (0♀, 1♂); 07-SRNP-67076, DHJPAR0025614; same data as for preceding except: 02.x.2007. • 1 (0♀, 0♂) (0♀, 1♂); 07-SRNP-67092, DHJPAR0025630; same data as for preceding except: 02.ix.2007. • 1 (0♀, 0♂) (0♀, 1♂); 07-SRNP-67111, DHJPAR0025649; same data as for preceding except: 10.vi.2007. • 1 (0♀, 0♂) (0♀, 1♂); 07-SRNP-67206, DHJPAR0025744; same data as for preceding except: 04.vii.2007. • 1 (0♀, 1♂) (0♀, 0♂); 07-SRNP-67271, DHJPAR0025809; same data as for preceding except: 21.viii.2007. • 1 (0♀, 0♂) (0♀, 1♂); 07-SRNP-67237, DHJPAR0025775; same data as for preceding except: 10.viii.2007. • 1 (0♀, 0♂) (0♀, 1♂); 07-SRNP-67734, DHJPAR0027472; same data as for preceding except: 03.viii.2007. • 1 (0♀, 1♂) (0♀, 0♂); 07-SRNP-67742, DHJPAR0027480; same data as for preceding except: 03.viii.2007. • 1 (0♀, 0♂) (0♀, 1♂); 07-SRNP-67748, DHJPAR0027486; same data as for preceding except: 03.viii.2007. • 1 (0♀, 1♂) (0♀, 0♂); 07-SRNP-67751, DHJPAR0027489; same data as for preceding except: 03.viii.2007. • 1 (0♀, 0♂) (0♀, 1♂); 07-SRNP-67762, DHJPAR0027500; same data as for preceding except: 09.viii.2007. • 1 (0♀, 0♂) (0♀, 1♂); 07-SRNP-67794, DHJPAR0027532; same data as for preceding except: 07.xi.2007. • 1 (0♀, 0♂) (0♀, 1♂); 07-SRNP-67222, DHJPAR0025760; same data as for preceding except: 16.vi.2007. • 1 (0♀, 0♂) (0♀, 1♂); 08-SRNP-3884, DHJPAR0027538; same data as for preceding except: 06.i.2008. • 1 (0♀, 0♂) (0♀, 1♂); 08-SRNP-3928, DHJPAR0027582; same data as for preceding except: 06.iii.2008. • 1 (0♀, 0♂) (0♀, 1♂); 08-SRNP-3930, DHJPAR0027584; same data as for preceding except: 06.iii.2008.

*Área de Conservación Guanacaste*, *Alajuela*, *Sector Rincón Rain Forest*, *Vado Río Francia*: • 1 (0♀, 0♂) (0♀, 1♂); 07-SRNP-66908, DHJPAR0025446; Malaise; 400 m; 10.90093, -85.28915; 17.vii.2007; DH Janzen & W Hallwachs leg. • 1 (0♀, 1♂) (0♀, 0♂); 07-SRNP-66936, DHJPAR0025474; same data as for preceding except: 06.vi.2007. • 1 (0♀, 1♂) (0♀, 0♂); 07-SRNP-67000, DHJPAR0025538; same data as for preceding except: 16.viii.2007. • 1 (0♀, 0♂) (0♀, 1♂); 07-SRNP-67005, DHJPAR0025543; same data as for preceding except: 05.vii.2007. • 1 (0♀, 0♂) (0♀, 1♂); 07-SRNP-67568, DHJPAR0026098; same data as for preceding except: 04.viii.2007. • 1 (0♀, 0♂) (0♀, 1♂); 07-SRNP-67576, DHJPAR0026106; same data as for preceding except: 27.x.2007. • 1 (0♀, 1♂) (0♀, 0♂); 07-SRNP-67675, DHJPAR0026370; same data as for preceding except: 04.viii.2007.

#### Diagnosis.

**Figure 206.**
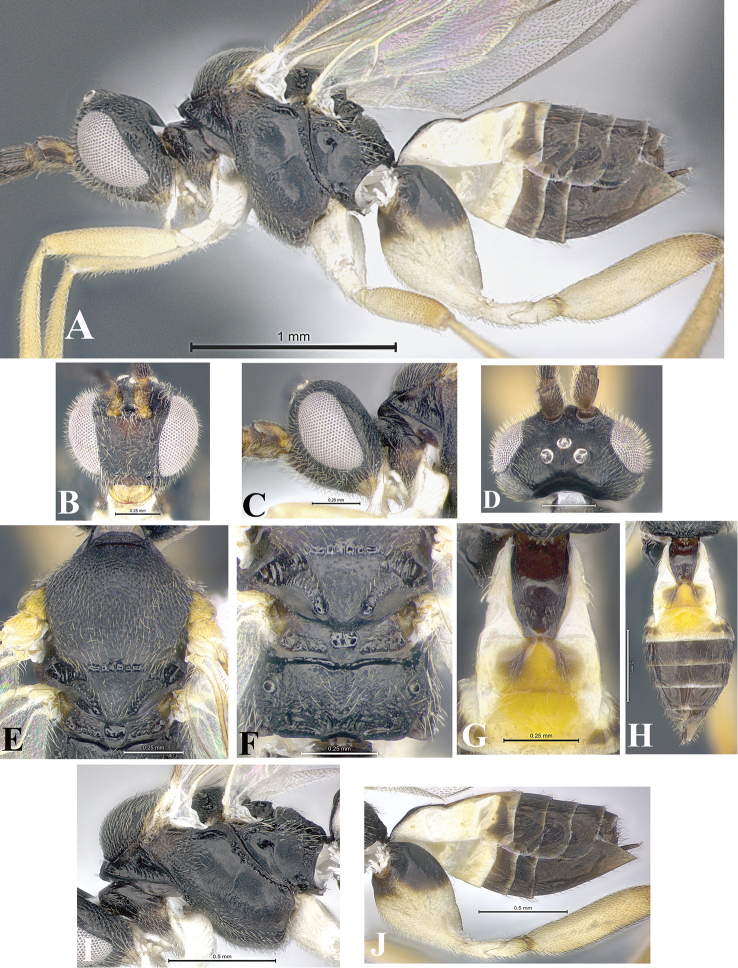
*Glyptapantelesstephaniekirkae* sp. nov. female 07-SRNP-67233 DHJPAR0025771 **A** Habitus **B, D** Head **B** Frontal view **D** Dorsal view **C** Head, pronotum, propleuron, lateral view **E** Mesonotum, dorsal view **F** Scutellum, metanotum, propodeum, dorsal view **G**T1–3, dorsal view **H, J** Metasoma **H** Dorsal view **J** Lateral view **I** Mesosoma, lateral view.

**Figure 207.**
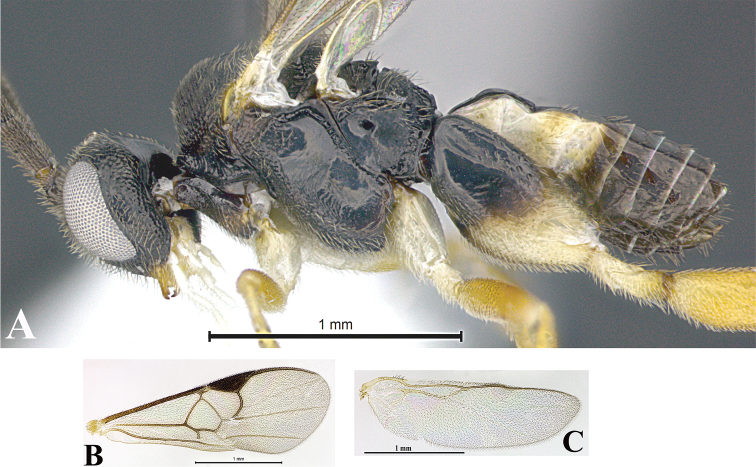
*Glyptapantelesstephaniekirkae* sp. nov. male 08-SRNP-3460 DHJPAR0027041, 07-SRNP-66621 DHJPAR0025159 **A** Habitus, **B, C** Wings **B** Fore **C** Hind.

Petiole on T1 with lateral margins straight throughout, with rugae (Fig. 206G, H), propodeum medially rhomboid-shaped with transverse rugae, but no trace of median longitudinal carina (Fig. 206F), hind coxa punctate only ventrally, dorsal outer depression present (Figs 206A, J, 207A), mesoscutum proximally distinctly punctate, distally with a polished area (Fig. 206E), precoxal groove shallow, but visible (Figs 206A, I, 207A), fore wing with vein 1 cu-a curved, r vein curved, outer side of junction of r and 2RS veins not forming a stub (Fig. 207B), inner margin of eyes diverging slightly at antennal sockets (Fig. 206B), and lateral grooves delimiting the median area on T2 clearly defined and reaching the distal edge of T2 (Fig. 206G, H).

#### Coloration

(Fig. 206A–D). General body coloration satin black except scape yellow-brown with brown areas; brown pedicel distally with a yellow-brown ring; all antennal flagellomeres dark brown on both sides; labrum, mandible, and tegulae yellow; apex of propleuron, both dorsal and ventral furrows of pronotum, epicnemial ridge, and mesopleuron distally with a small area yellow-brown/reddish; glossa, maxillary and labial palps pale yellow/ivory. Eyes and ocelli silver. Fore and middle legs dark yellow, except coxae and trochanters pale yellow/ivory, and claws brown; hind legs pale yellow/ivory except 1/3 proximal of black coxae, femora distally brown, and tibiae and tarsomeres dark yellow/orange. Petiole on T1 with two colorations: distal 3/4 brown and proximal 1/4 lighter yellow-brown/reddish, contours darkened and sublateral areas ivory/pale yellow; T2 with median area dark yellow, adjacent area brown, and lateral ends ivory/pale yellow; T3 medially dark yellow, proximally yellow area coinciding with the width of median and adjacent areas on T2, but distally widen, laterally distal ends with brown coloration; T4 and beyond completely brown; distally each tergum with a wide ivory/pale yellow transparent band. In lateral view, T1–3 ivory/pale yellow; T4 light brown; T5 and beyond brown. S1–3 ivory/yellow; S4 and beyond brown.

#### Description.

**Head** (Fig. 206A–D). Head rounded with pubescence long and dense. Proximal three antennal flagellomeres longer than wide (0.28:0.08, 0.28:0.08, 0.27:0.08), distal antennal flagellomere longer than penultimate (0.15:0.07, 0.12:0.07), antenna longer than body (3.78, 2.98); antennal scrobes-frons sloped and forming a shelf. Face flat or nearly so, punctate-lacunose, interspaces wavy and longitudinal median carina present. Frons smooth. Temple wide, punctate-lacunose and interspaces wavy. Inner margin of eyes diverging slightly at antennal sockets; in lateral view, eye anteriorly convex and posteriorly straight. POL shorter than OOL (0.09, 0.12). Malar suture present. Median area between lateral ocelli slightly depressed. Vertex laterally pointed or nearly so and dorsally wide.

**Mesosoma** (Fig. 206A, E, F, I). Mesosoma dorsoventrally convex. Distal 1/3 of mesoscutum with lateral margin slightly dented, punctation proximally distinct, but distally with a polished area, interspaces wavy/lacunose. Scutellum triangular, apex sloped and fused with BS, scutellar punctation distinct throughout, in profile scutellum flat and on same plane as mesoscutum, but not in the same plane, phragma of the scutellum completely concealed; BS only very partially overlapping the MPM; ATS demilune with a little, complete and parallel carinae; dorsal ATS groove with semicircular/parallel carinae. Transscutal articulation with small and heterogeneous foveae, area just behind transscutal articulation smooth, shiny and sloped. Metanotum with BM upward; MPM circular without median longitudinal carina; AFM without setiferous lobes and not as well delineated as PFM; PFM thick and smooth; ATM proximally with a groove with some sculpturing and distally smooth. Propodeum with transverse rugae, proximal half curved with medium-sized sculpture and distal half rugose; distal edge of propodeum with a flange at each side and without stubs; propodeal spiracle distally framed by a short concave carina; nucha surrounded by very short radiating carinae. Pronotum with a distinct dorsal furrow, dorsally with a well-defined smooth band; central area of pronotum smooth, but both dorsal and ventral furrows with short parallel carinae. Propleuron with fine rugae and dorsally with a carina. Metasternum convex. Contour of mesopleuron straight/angulate or nearly so; precoxal groove shallow, but visible and with transverse lineate sculpture; epicnemial ridge convex, teardrop-shaped.

**Legs** (Fig. 206A, J). Ventral margin of fore telotarsus entire without seta, fore telotarsus almost same width throughout and longer than fourth tarsomere (0.12, 0.10). Hind coxa with punctation only on ventral surface, dorsal outer depression present. Inner spur of hind tibia longer than outer spur (0.32, 0.20), entire surface of hind tibia with dense strong spines clearly differentiated by color and length. Hind telotarsus longer than fourth tarsomere (0.15, 0.13).

**Wings** (Fig. 207B, C). Fore wing with r vein curve; 2RS vein straight; r and 2RS veins forming a weak, even curve at their junction and outer side of junction not forming a stub; 2M vein slightly curved/swollen; distally fore wing [where spectral veins are] with microtrichiae more densely concentrated than the rest of the wing; anal cell 1/3 proximally lacking microtrichiae; subbasal cell with microtrichiae virtually throughout; veins 2CUa and 2CUb completely spectral; vein 2 cu-a absent; vein 2-1A proximally tubular and distally spectral, although sometimes difficult to see; tubular vein 1 cu-a curved, incomplete/broken and not reaching the edge of 1-1A vein. Hind wing with vannal lobe narrow, subdistally evenly convex and subproximally straightened, and setae evenly scattered in the margin.

**Metasoma** (Fig. 206A, G, H, J). Metasoma laterally compressed. Petiole on T1, distal half with faint rugae only laterally, virtually parallel-sided over most of length, but narrowing over distal 1/3 (length 0.36, maximum width 0.18, minimum width 0.10), and with scattered pubescence concentrated in the first distal third. Lateral grooves delimiting the median area on T2 clearly defined and reaching the distal edge of T2 (length median area 0.18, length T2 0.18), edges of median area polished and lateral grooves deep, median area broader than long (length 0.18, maximum width 0.21, minimum width 0.07); T2 with scattered pubescence only distally. T3 longer than T2 (0.24, 0.18) and with scattered pubescence only distally. Pubescence on hypopygium dense.

**Cocoons.** Unknown.

#### Comments.

The pale coloration on T2 and T3 are distinct.

#### Male

(Fig. 207A–C). Some males exhibit the hind legs darker than females and the brown coloration in the hind coxae covers more area. In all males, the coloration on T2 and T3 is darker than in females, yellow coloration is replaced by light brown; however, in some males the junction between T2 and T3 exhibits yellow-brown/reddish coloration.

#### Etymology.

Stephanie Kirk is a research technician at the Biodiversity Institute of Ontario (BIO) in Guelph, Canada.

#### Distribution.

Adult parasitoids were collected in Costa Rica, ACG, Sector Rincón Rain Forest (Vado Río Francia) and Sector San Cristóbal (Bosque Trampa Malaise, Potrero Argentina, and Río Blanco Abajo), during June-December 2007 and January-May 2008 at 400 m, 500 m, 520 m, and 815 m in pasture and rain forest.

#### Biology.

Unknown.

#### Host.

Unknown.

### Glyptapanteles sujeevanratnasinghami

Taxon classificationAnimaliaHymenopteraBraconidae

Arias-Penna, sp. nov.

http://zoobank.org/82BE301F-4B50-4C32-A9E5-994A9EBC3F1F

Fig. 208

#### Female.

Body length 2.37 mm, antenna length 3.23 mm, fore wing length 2.48 mm.

#### Type material.

**Holotype**: COSTA RICA • 1♀; 07-SRNP-67768, DHJPAR0027506; Área de Conservación Guanacaste, Alajuela, Sector San Cristóbal, Potrero Argentina; pastures; Malaise; 520 m; 10.89021, -85.38803; 09.viii.2007; DH Janzen & W Hallwachs leg.; (CNC). **Paratypes.** • 1 (0♀, 1♂) (0♀, 0♂); 07-SRNP-67051, DHJPAR0025589; same data as for holotype except: 21.viii.2007; (CNC). • 1 (1♀, 0♂) (0♀, 0♂); 07-SRNP-67156, DHJPAR0025694; same data as for holotype except: 15.viii.2007; (CNC). • 1 (0♀, 0♂) (1♀, 0♂); 07-SRNP-67236, DHJPAR0025774; same data as for holotype, except: 10.viii.2007; (CNC). • 1 (0♀, 1♂) (0♀, 0♂); 07-SRNP-67268, DHJPAR0025806; same data as for holotype except: 21.viii.2007; (CNC). • 1 (0♀, 1♂) (0♀, 0♂); 07-SRNP-67790, DHJPAR0027528; same data as for holotype except: 07.xi.2007; (CNC). • 1 (1♀, 0♂) (0♀, 0♂); 08-SRNP-3891, DHJPAR0027545; same data as for holotype except: 18.i.2008; (CNC). • 1 (0♀, 0♂) (1♀, 0♂); 08-SRNP-3889, DHJPAR0027543; same data as for holotype except: 12.i.2008; (CNC).

#### Other material.

**Reared material.** COSTA RICA: *Área de Conservación Guanacaste*, *Guanacaste*, *Sector Pitilla*, *Sendero Mismo*: • 1 (0♀, 0♂) (1♀, 0♂); 11-SRNP-31020, DHJPAR0043089; rain forest; 680 m; 10.98758, -85.41967; 16.iv.2011; Petrona Rios leg.; caterpillar collected in third instar; cocoon in larval mummy formed on 21.iv.2011; adult parasitoid emerged on 29.iv.2011. • 1 (0♀, 0♂) (1♀, 0♂); 11-SRNP-31035, DHJPAR0043100; same data as for preceding except: 19.iv.2011; cocoon adhered to the leaf substrate.

*Área de Conservación Guanacaste*, *Guanacaste*, *Sector Pitilla*, *Sendero Memo*: • 1 (0♀, 0♂) (1♀, 0♂); 11-SRNP-31023, DHJPAR0043082; rain forest; 740 m; 10.98171, -85.42785; 16.iv.2011; Freddy Quesada leg.; caterpillar collected in fourth instar; cocoons adhered to the leaf substrate and formed on 21.iv.2011; adult parasitoid emerged on 04.v.2011.

#### Malaise-trapped material.

COSTA RICA: *Área de Conservación Guanacaste*, *Guanacaste*, *Sector Cacao*, *Sendero Cima*: • 1 (1♀, 0♂) (0♀, 0♂); 99-SRNP-19182, DHJPAR0013441; cloud forest; Malaise; 1460 m; 10.93328, -85.45729; 18.x.1999; DH Janzen & W Hallwachs leg.

*Área de Conservación Guanacaste*, *Alajuela*, *Sector Rincón Rain Forest*, *Vado Río Francia*: • 1 (1♀, 0♂) (0♀, 0♂); 07-SRNP-66912, DHJPAR0025450; Malaise; 400 m; 10.90093, -85.28915; 17.vii.2007; DH Janzen & W Hallwachs leg. • 1 (0♀, 1♂) (0♀, 0♂); 08-SRNP-41764, DHJPAR0026207; same data as for preceding except: 25.iii.2008. • 1 (0♀, 0♂) (0♀, 1♂); 08-SRNP-41743, DHJPAR0026186; same data as for preceding except: 08.iii.2008. • 1 (0♀, 0♂) (0♀, 1♂); 08-SRNP-41727, DHJPAR0026170; same data as for preceding except: 18.ii.2008. • 1 (0♀, 1♂) (0♀, 0♂); 08-SRNP-41714, DHJPAR0026157; same data as for preceding except: 12.ii.2008.

*Área de Conservación Guanacaste*, *Alajuela*, *Sector San Cristóbal*, *Río Blanco Abajo*: • 1 (0♀, 1♂) (0♀, 0♂); 07-SRNP-66577, DHJPAR0025115; rain forest; Malaise; 500 m; 10.90037, -85.37254; 04.vii.2007; DH Janzen & W Hallwachs leg. • 1 (1♀, 0♂) (0♀, 0♂); 08-SRNP-3080, DHJPAR0026661; same data as for preceding except: 12.iii.2008. • 1 (0♀, 0♂) (0♀, 1♂); 08-SRNP-3126, DHJPAR0026707; same data as for preceding except: 18.iii.2008. • 1 (0♀, 0♂) (1♀, 0♂); 08-SRNP-3197, DHJPAR0026778; same data as for preceding except: 24.iii.2008. • 1 (0♀, 1♂) (0♀, 0♂); 08-SRNP-3540, DHJPAR0027121; same data as for preceding except: 23.iv.2008. • 1 (1♀, 0♂) (0♀, 0♂); 08-SRNP-3572, DHJPAR0027153; same data as for preceding except: 23.iv.2008.

*Área de Conservación Guanacaste*, *Alajuela*, *Sector San Cristóbal*, *Bosque Trampa Malaise*: • 1 (1♀, 0♂) (0♀, 0♂); 07-SRNP-67393, DHJPAR0025931; rain forest; 815 m; 10.86280, -85.38460; 16.vi.2007; DH Janzen & W Hallwachs leg. • 1 (0♀, 0♂) (0♀, 1♂); 07-SRNP-67449, DHJPAR0025987; same data as for preceding except: 22.vi.2007. • 1 (0♀, 1♂) (0♀, 0♂); 07-SRNP-67500, DHJPAR0026038; same data as for preceding except: 10.vii.2007. • 1 (0♀, 1♂) (0♀, 0♂); 07-SRNP-67802, DHJPAR0027598; same data as for preceding except: 03.viii.2007. • 1 (0♀, 0♂) (0♀, 1♂); 07-SRNP-67840, DHJPAR0027636; same data as for preceding except: 01.xi.2007.

*Área de Conservación Guanacaste*, *Alajuela*, *Sector San Cristóbal*, *Estación San Gerardo*: • 1 (0♀, 1♂) (0♀, 0♂); 07-SRNP-67345, DHJPAR0025883; rain forest; 575 m; 10.88009, -85.38887; 15.viii.2007; DH Janzen & W Hallwachs leg.

#### Diagnosis.

**Figure 208.**
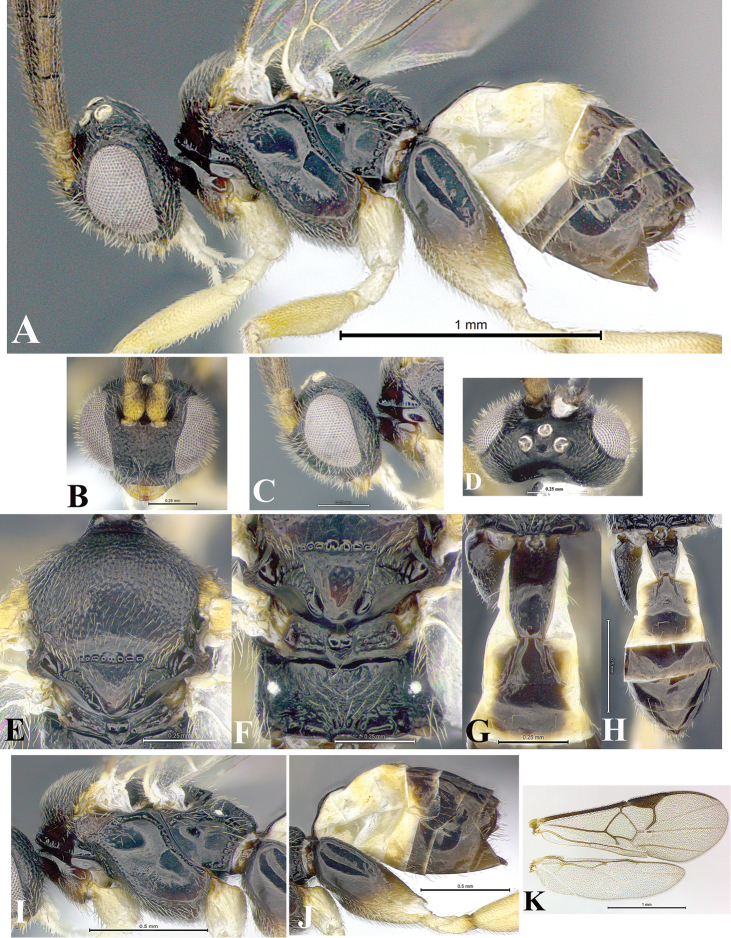
*Glyptapantelessujeevanratnasinghami* sp. nov. female 07-SRNP-67156 DHJPAR0025694, 07-SRNP-67768 DHJPAR0027506 **A** Habitus **B, D** Head **B** Frontal view **D** Dorsal view **C** Head, pronotum, propleuron, lateral view **E** Mesonotum, dorsal view **F** Scutellum, metanotum, propodeum, dorsal view **G**T1–3, dorsal view **H, J** Metasoma **H** Dorsal view **J** Lateral view **I** Mesosoma, lateral view **K** Fore and hind wings, male 07-SRNP-67795 DHJPAR0027533.

Hind coxa punctate only ventrally (Fig. 208A, J), hind telotarsus longer than fourth tarsomere, distal antennal flagellomere longer than penultimate, phragma of the scutellum completely concealed (Fig. 208F) propodeum medially rhomboid-shaped with transverse rugae, but no trace of median longitudinal carina (Fig. 208F), anteroventral contour of mesopleuron convex (Fig. 208A, I), edges of median area on T2 polished and followed by a deep groove (Fig. 208G, H), and fore wing with r vein curved, outer side of junction of r and 2RS veins forming a slight stub (Fig. 208K).

#### Coloration

(Fig. 208A–K). General body coloration satin black except scape and pedicel yellow, but inner and outer sides brown; first three-four proximal antennal flagellomeres dorsally lighter (light brown) than ventrally (dark brown), remaining flagellomeres dark brown on both sides; tegulae, labrum, and mandible yellow; middle area of clypeus, middle area of face just below antennal sockets, propleuron distally, mesosternum, dorsal furrow of pronotum, area of mesoscutum just above of dorsal furrow of pronotum, epicnemial ridge, dorso-ventrally edge of mesopleuron, distal corners of mesoscutum, apex of scutellum, and lateral ends of metanotum with yellow-brown/reddish tints; glossa, maxillary and labial palps pale yellow/ivory. Eyes and ocelli silver. Fore and middle legs dark yellow, except coxae and trochanters pale yellow/ivory, and claws brown; hind legs dark yellow except proximal half of coxae black and distal half yellow-brown, femora distally brown, and tibiae (with both ends darker) and tarsomeres dark yellow/orange. Petiole on T1 brown, contours darkened and sublateral areas ivory/pale yellow; T2 with median and wide adjacent areas brown, both forming a rectangle-shape area, and lateral ends ivory/pale yellow; T3 medially brown, shape of that dark area coinciding in both proximally and distally extremes with the width of median plus adjacent areas on T2; however, dark area not reaching distal edge of T3, rather T3 distally with a wide yellow band, and lateral ends on T3 ivory/pale yellow; T4 and beyond completely brown; distally each tergum with a narrow ivory/pale yellow transparent band. In lateral view, T1–3 ivory/pale yellow; T4 yellow-brown; T5 and beyond brown. S1–3 ivory/yellow; S4 yellow, but medially with a brown spot; penultimate sternum and hypopygium brown.

#### Description.

**Head** (Fig. 208A–D). Head rhomboid with pubescence long and dense. Proximal three antennal flagellomeres longer than wide (0.23:0.08, 0.24:0.08, 0.24:0.08), distal antennal flagellomere longer than penultimate (0.15:0.06, 0.11:0.06), antenna longer than body (3.23, 2.37); antennal scrobes-frons shallow. Face flat or nearly so, punctate-lacunose, interspaces wavy and longitudinal median carina present. Frons smooth. Temple wide, punctate-lacunose and interspaces wavy. Inner margin of eyes diverging slightly at antennal sockets; in lateral view, eye anteriorly convex and posteriorly straight. POL shorter than OOL (0.09, 0.11). Malar suture present. Median area between lateral ocelli slightly depressed. Vertex laterally pointed or nearly so and dorsally wide.

**Mesosoma** (Fig. 208A, E, F, I). Mesosoma dorsoventrally convex. Distal 1/3 of mesoscutum with lateral margin slightly dented, punctation distinct throughout, interspaces wavy/lacunose. Scutellum triangular, apex sloped and fused with BS, scutellar punctation distinct throughout, in profile scutellum flat and on same plane as mesoscutum, but not in the same plane, phragma of the scutellum completely concealed; BS only very partially overlapping the MPM; ATS demilune with a little, complete and parallel carinae, dorsal ATS groove with semicircular/parallel carinae. Transscutal articulation with small and homogeneous foveae, area just behind transscutal articulation sloped, smooth and shiny. Metanotum with BM upward; MPM circular without median longitudinal carina; AFM without setiferous lobes and not as well delineated as PFM; PFM thick, smooth and with lateral ends rounded; ATM proximally with a groove with some sculpturing and distally smooth. Propodeum with transverse rugae, proximal half curved with medium-sized sculpture and distal half rugose; distal edge of propodeum with a flange at each side and without stubs; propodeal spiracle distally framed by a short concave carina; nucha surrounded by very short radiating carinae. Pronotum with a distinct dorsal furrow, dorsally with a well-defined smooth band; central area of pronotum and dorsal furrow smooth, but ventral furrow with short parallel carinae. Propleuron with fine punctations throughout and dorsally with a carina. Metasternum flat or nearly so. Contour of mesopleuron convex; precoxal groove deep with transverse lineate sculpture; epicnemial ridge convex, teardrop-shaped.

**Legs** (Fig. 208A, J). Ventral margin of fore telotarsus slightly excavated and with a tiny curved seta, fore telotarsus almost same width throughout and longer than fourth tarsomere (0.12, 0.07). Hind coxa with punctation only on ventral surface, dorsal outer depression present. Inner spur of hind tibia longer than outer spur (0.29, 0.20), entire surface of hind tibia with dense strong spines clearly differentiated by color and length. Hind telotarsus longer than fourth tarsomere (0.15, 0.12).

**Wings** (Fig. 208K). Fore wing with r vein slightly curved; 2RS vein straight; r and 2RS veins forming a weak, even curve at their junction and outer side of junction forming a slight stub; 2M vein slightly curved/swollen; distally fore wing [where spectral veins are] with microtrichiae more densely concentrated than the rest of the wing; anal cell 1/3 proximally lacking microtrichiae; subbasal cell with a small smooth area; vein 2CUa absent and vein 2CUb spectral; vein 2 cu-a absent; vein 2-1A proximally tubular and distally spectral, although sometimes difficult to see; tubular vein 1 cu-a curved, incomplete/broken and not reaching the edge of 1-1A vein. Hind wing with vannal lobe very narrow, subdistally evenly convex and subproximally straightened, and setae evenly scattered in the margin.

**Metasoma** (Fig. 208A, G, H, J). Metasoma laterally compressed. Petiole on T1 finely sculptured only laterally, petiole parallel-sided in proximal half and then narrowing (length 0.35, maximum width 0.16, minimum width 0.08) and with scarse pubescence concentrated in the first distal third. Lateral grooves delimiting the median area on T2 clearly defined and reaching the distal edge of T2 (length median area 0.16, length T2 0.16), edges of median area polished and lateral grooves deep, median area broader than long (length 0.16, maximum width 0.19, minimum width 0.08); T2 with scattered pubescence only distally. T3 longer than T2 (0.20, 0.16) and with scattered pubescence only distally. Pubescence on hypopygium scattered.

**Cocoons.** Beige or white oval cocoon with silk fibers evenly smooth. Cocoon adhered to the leaf substrate.

#### Comments.

In some females, the whole propleuron is yellow-brown and the T3 distally lacking of a yellow band.

#### Male.

Similar in shape and color to female; although the coloration on some body parts varies: the dark area on T3 extending to the distal edge. In some males (e.g., 08-SRNP-3540), the coloration of hind coxae is different: the coxae are black, but distally yellow-brown. Also, the metepimeron has some faintly transverse lineate sculpture. In other males, the hind coxae with two colorations: proximal half brown and distal half yellow-brown (e.g., 07-SRNP-66577). Other males have the entire propleuron yellow and the coloration of the mesopleuron is more distinctive than in females (e.g., 07-SRNP-67500). Some males with femora distally and tibiae proximally brown.

#### Etymology.

Sujeevan Ratnasingham is (since 2010) the chair of Informatics, International Barcode of Life project (iBOLD).

#### Distribution.

Parasitized caterpillars were collected in Costa Rica, ACG, Sector Pitilla (Sendero Memo and Sendero Mismo), during April 2011 at 680 m and 740 m in rain forest.

Adult parasitoids were collected in Costa Rica, ACG, Sector Cacao (Sendero Cima), Sector Rincón Rain Forest (Vado Río Francia), and Sector San Cristóbal (Bosque Trampa Malaise, Estación San Gerardo, Río Blanco Abajo, and Potrero Argentina), during October 1999; June-August and November 2007; and January-April 2008 at 400, 500, 520, 575, 815 and 1,460 m in pastures, rain forest and cloud forest.

#### Biology.

The lifestyle of this parasitoid species is solitary.

#### Host.

*Psaliodes* sp. Guenée (Geometridae: Larentiinae) feeding on *Cyatheamultiflora* (Cyatheaceae). Caterpillars were collected in third and fourth instar.

### Glyptapanteles suniae

Taxon classificationAnimaliaHymenopteraBraconidae

Arias-Penna, sp. nov.

http://zoobank.org/6F8BE86A-F440-4E25-9A1D-939880A0BC88

Fig. 209

#### Female.

Body length 2.78 mm, antenna length 3.33 mm, fore wing length 3.18 mm.

#### Type material.

**Holotype**: ECUADOR • 1♀; EC-12773, YY-A041; Napo, Yanayacu Biological Station, Camino a Loreto; 1,200 m; -0.7, -77.733333; 02.iii.2006; Rafael Granizo leg.; caterpillar collected in third instar; cocoons formed on 17.iii.2006; adult parasitoids emerged on 12.iv.2006; (PUCE). **Paratypes.** • 18 (5♀, 5♂) (6♀, 2♂); EC-12773, YY-A041; same data as for holotype; (PUCE).

#### Diagnosis.

**Figure 209.**
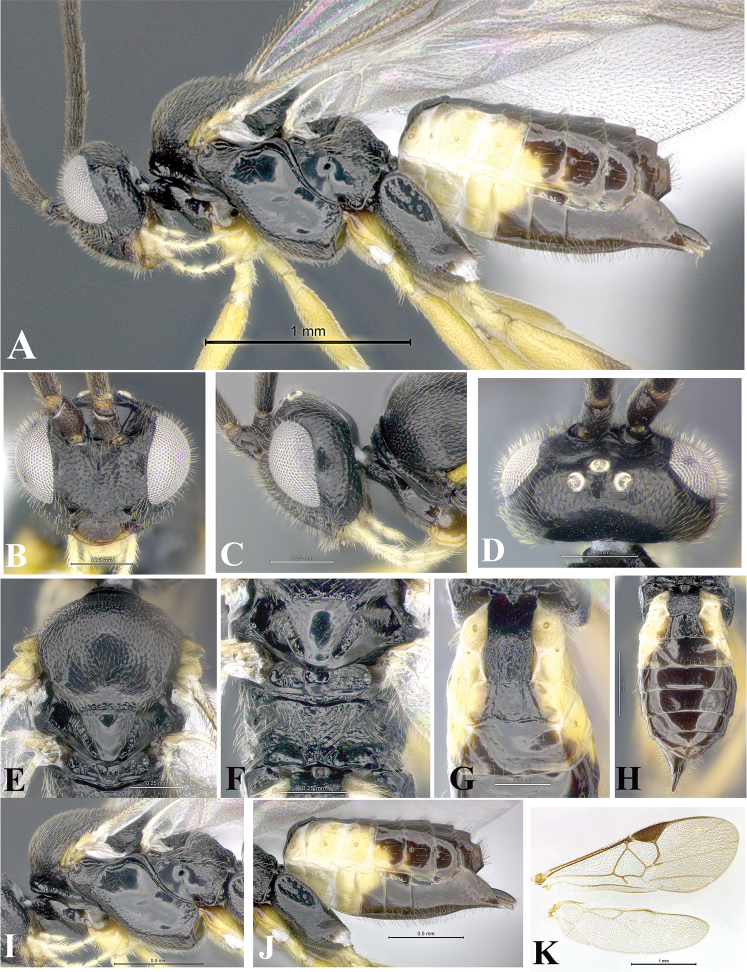
*Glyptapantelessuniae* female sp. nov. EC-12773 YY-A041 **A** Habitus **B, D** Head **B** Frontal view **D** Dorsal view **C** Head, propleuron, lateral view **E** Mesonotum, dorsal view **F** Scutellum, metanotum, propodeum, dorsal view **G**T1–3, dorsal view **H, J** Metasoma **H** Dorsal view **J** Lateral view **I** Mesosoma, lateral view **K** Fore and hind wings.

Petiole on T1 with lateral margin relatively straight in proximal half, but distal half curved (convex, Fig. 209G, H), fore telotarsus almost same width throughout, dorsal furrow of pronotum with a well-defined smooth band (Fig. 209A, I), petiole on T1 with a mix of fine rugae and coarse sculpture over most of the surface (Fig. 209G, H), propodeum medially rhomboid-shaped with transverse rugae, but no trace of median longitudinal carina (Fig. 209F), fore wing with vein 1 cu-a straight, r vein slightly curved or curved, outer side of junction of r and 2RS veins not forming a stub (Fig. 209K), dorsal outer depression on hind coxa present (Fig. 209A, J), inner margin of eyes diverging slightly at antennal sockets (Fig. 209B), and lateral grooves delimiting the median area on T2 clearly defined and reaching the distal edge of T2 (Fig. 209G, H).

#### Coloration

(Fig. 209A–K). General body coloration polished black except pedicel distal half yellow-brown and proximal half brown; scape brown; first five-six proximal antennal flagellomeres dorsally lighter (light brown) than ventrally (dark brown), remaining flagellomeres dark brown on both sides; mandibles distally yellow-brown; glossa, maxillary and labial palps, and tegulae yellow; clypeus, labrum, propleuron, both dorsal and ventral furrows of pronotum, epicnemial ridge, ventral edge of mesopleuron and lateral edges of metasternum with brown-red/reddish tints. Eyes and ocelli silver. Fore and middle legs yellow except coxae proximal with a brown smear area and brown claws; hind legs yellow except black coxae distally brown-red/reddish, femora distally brown, distal half of tibiae brown, and tarsomeres brown. Petiole on T1 black and sublateral areas yellow; T2 with median and adjacent areas brown, median area with contours darkened, adjacent area very narrow, and lateral ends yellow; T3 mostly brown, proximally this dark area coinciding with the width of median and adjacent areas on T2, and proximal half of lateral ends yellow; T4 and beyond completely brown; distally each tergum with a narrow translucent band. In lateral view, T1–3 yellow; T4 yellow, but dorsally brown; T5 and beyond brown. S1–3 yellow; S4 brown with a dorsal yellow area; penultimate sternum and hypopygium brown.

#### Description.

**Head** (Fig. 209A–D). Head rounded with pubescence long and dense. Proximal three antennal flagellomeres longer than wide (0.22:0.08, 0.25:0.08, 0.24:0.08), distal antennal flagellomere longer than penultimate (0.16:0.06, 0.11:0.06), antenna longer than body (3.33, 2.78); antennal scrobes-frons shallow. Face flat or nearly so, finely punctate-lacunose, interspaces wavy and longitudinal median carina present. Frons smooth. Temple wide, punctate-lacunose and interspaces wavy. Inner margin of eyes diverging slightly at antennal sockets; in lateral view, eye anteriorly convex and posteriorly straight. POL shorter than OOL (0.10, 0.15). Malar suture present. Median area between lateral ocelli slightly depressed. Vertex laterally pointed or nearly so and dorsally wide.

**Mesosoma** (Fig. 209A, E, F, I). Mesosoma dorsoventrally convex. Mesoscutum proximally convex and distally flat, punctation distinct proximally, but absent/dispersed distally. Scutellum triangular, apex sloped and fused with BS, but not in the same plane, scutellar punctation distinct peripherally and absent centrally, in profile scutellum flat and on same plane as mesoscutum, phragma of the scutellum partially exposed; BS only very partially overlapping the MPM; ATS demilune inner side with a row of foveae; dorsal ATS groove smooth. Transscutal articulation with small and heterogeneous foveae, area just behind transscutal articulation sloped, smooth and shiny. Metanotum with BM wider than PFM (clearly differentiated); MPM semicircular without median longitudinal carina; AFM without setiferous lobes and not as well delineated as PFM; PFM thick, smooth and with lateral ends rounded; ATM proximally with a groove with some sculpturing and distally smooth. Propodeum medially rhomboid-shaped with rugae, proximal half curved with medium-sized sculpture and distal half with a shallow dent at each side of nucha; distal edge of propodeum with a flange at each side and without stubs; propodeal spiracle distally framed by a short transverse carina; nucha surrounded by very short radiating carinae. Pronotum with a distinct dorsal furrow, dorsally with a well-defined smooth band; central area of pronotum and dorsal furrow smooth, but ventral furrow with short parallel carinae. Propleuron finely sculptured only ventrally and dorsally without a carina. Metasternum convex. Contour of mesopleuron convex; precoxal groove smooth, shiny and shallow, but visible; epicnemial ridge widen.

**Legs** (Fig. 209A, J). Ventral margin of fore telotarsus slightly excavated and with a tiny curved seta, fore telotarsus almost same width throughout and longer than fourth tarsomere (0.15, 0.10). Hind coxa with dorsal half sparsely punctate, ventral half densely punctate, and dorsal outer depression present. Inner spur of hind tibia longer than outer spur (0.25, 0.20), entire surface of hind tibia with dense strong spines clearly differentiated by color and length. Hind telotarsus as equal in length as fourth tarsomere (0.15, 0.15).

**Wings** (Fig. 209K). Fore wing with r vein curved; 2RS vein straight; r and 2RS veins forming a weak, even curve at their junction and outer side of junction not forming a stub; 2M vein slightly curved/swollen; distally fore wing [where spectral veins are] with microtrichiae more densely concentrated than the rest of the wing; anal cell 1/3 proximally lacking microtrichiae; subbasal cell proximal half smooth; vein 2CUa absent and vein 2CUb spectral; vein 2 cu-a absent; vein 2-1A proximally tubular and distally spectral, although sometimes difficult to see; tubular vein 1 cu-a straight, incomplete/broken and not reaching the edge of 1-1A vein. V Hind wing with vannal lobe narrow, subdistally and subproximally straightened, and setae evenly scattered in the margin.

**Metasoma** (Fig. 209A, G, H, J). Metasoma cylindrical. Petiole on T1 with a mix of fine rugae and coarse sculpture over most of the surface, virtually parallel-sided over most of length, but barely narrowing at apex, apex truncate (length 0.37 mm, maximum width 0.22, minimum width 0.18), and with scattered pubescence concentrated in the first distal third. Lateral grooves delimiting the median area on T2 clearly defined and reaching the distal edge of T2 (length median area 0.16, length T2 0.16), edges of median area polished and lateral grooves deep, median area broader than long (length 0.16, maximum width 0.24, minimum width 0.15); T2 with scattered pubescence only distally. T3 longer than T2 (0.23, 0.16) and with scattered pubescence throughout. Pubescence on hypopygium dense.

**Cocoons.** Unknown.

#### Comments.

The specimens are slender and elongated (Fig. 209A), petiole on T1 with lateral margins relatively straight in proximal half, but distal half curved (convex, Fig. 209G). As well as G. *phildevriesi*, that shape resembles the petiole of *Venanushelavai* Mason (Mason 1981, Fig. 77b). The propodeum distally forming a distinctive wall (Fig. 209F) and the propodeal spiracle distally framed by a concave carina.

#### Male.

Similar in coloration to female.

#### Etymology.

Sindhu (Suni) Krishnankutty is an Indian entomologist who, as a graduate student at UIUC, IL, USA, worked on the taxonomy and systematics of the endemic leafhoppers from Madagascar. Currently, she is a researcher at Plant Protection and Quarantine program, Animal and Plant Health Inspection Service, United States Department of Agriculture, Buzzards Bay, MA, USA.

#### Distribution.

Parasitized caterpillar was collected in Ecuador, Napo, Yanayacu Biological Station (Camino a Loreto), during March 2006 at 1,200 m in cloud forest.

#### Biology.

The lifestyle of this parasitoid species is gregarious.

#### Host.

Undetermined species of Erebidae (Arctiinae), food plant was not reported. Caterpillar was collected in third instar.

### Glyptapanteles sureshnaiki

Taxon classificationAnimaliaHymenopteraBraconidae

Arias-Penna, sp. nov.

http://zoobank.org/5D6CBA75-D3A3-4910-B68E-D57969CE6970

Fig. 210

#### Female.

Body length 3.63 mm, antenna length 4.70, fore wing length 3.53 mm.

#### Type material.

**Holotype**: COSTA RICA • 1♀; 08-SRNP-2927, DHJPAR0026508; Área de Conservación Guanacaste, Alajuela, Sector San Cristóbal, Río Blanco Abajo; rain forest; Malaise; 500 m; 10.90037, -85.37254; 11.ii.2008; DH Janzen & W Hallwachs leg.; (CNC). **Paratypes**. • 1 (0♀, 0♂) (0♀, 1♂); 08-SRNP-2876, DHJPAR0026457; same data as for holotype except: 30.i.2008; (CNC). • 1 (0♀, 1♂) (0♀, 0♂); 08-SRNP-2913, DHJPAR0026494; same data as for holotype except: 05.ii.2008; (CNC). • 1 (0♀, 1♂) (0♀, 0♂); 08-SRNP-2993, DHJPAR0026574; same data as for holotype except: 23.ii.2008; (CNC). • 1 (1♀, 0♂) (0♀, 0♂); 08-SRNP-3017, DHJPAR0026598; same data as for holotype except: 29.ii.2008; (CNC). • 1 (0♀, 0♂) (0♀, 1♂); 08-SRNP-3032, DHJPAR0026613; same data as for holotype except: 06.iii.2008; (CNC). • 1 (0♀, 0♂) (0♀, 1♂); 08-SRNP-3073, DHJPAR0026654; same data as for holotype except: 12.iii.2008; (CNC). • 1 (0♀, 0♂) (0♀, 1♂); 08-SRNP-3162, DHJPAR0026743; same data as for holotype except: 18.iii.2008; (CNC). • 1 (0♀, 0♂) (0♀, 1♂); 08-SRNP-3183, DHJPAR0026764; same data as for holotype except: 24.iii.2008; (CNC). • 1 (0♀, 0♂) (0♀, 1♂); 08-SRNP-3238, DHJPAR0026819; same data as for holotype except: 30.iii.2008; (CNC). • 1 (0♀, 1♂) (0♀, 0♂); 08-SRNP-3247, DHJPAR0026828; same data as for holotype except: 30.iii.2008; (CNC). • 1 (0♀, 1♂) (0♀, 0♂); 08-SRNP-3260, DHJPAR0026841; same data as for holotype except: 30.iii.2008; (CNC).

#### Other material.

**Malaise-trapped material.** COSTA RICA: *Área de Conservación Guanacaste*, *Alajuela*, *Sector San Cristóbal*, *Bosque Trampa Malaise*: • 1 (0♀, 0♂) (0♀, 1♂); 07-SRNP-67452, DHJPAR0025990; rain forest; 815 m; 10.86280, -85.38460; 04.vii.2007; DH Janzen & W Hallwachs leg. • 1 (0♀, 1♂) (0♀, 0♂); 07-SRNP-67801, DHJPAR0027597; same data as for preceding except: 03.viii.2007.

*Área de Conservación Guanacaste*, *Alajuela*, *Sector San Cristóbal*, *Río Blanco Abajo*: • 1 (0♀, 1♂) (0♀, 0♂); 07-SRNP-66291, DHJPAR0024829; rain forest; Malaise; 500 m; 10.90037, -85.37254; 26.ix.2007; DH Janzen & W Hallwachs leg. • 1 (1♀, 0♂) (0♀, 0♂); 07-SRNP-66315, DHJPAR0024853; same data as for preceding except: 08.viii.2007. • 1 (0♀, 0♂) (0♀, 1♂); 07-SRNP-66362, DHJPAR0024900; same data as for preceding except: 02.x.2007. • 1 (0♀, 0♂) (0♀, 1♂); 07-SRNP-66532, DHJPAR0025070; same data as for preceding except: 22.vi.2007. • 1 (0♀, 1♂) (0♀, 0♂); 07-SRNP-66668, DHJPAR0025206; same data as for preceding except: 14.x.2007. • 1 (0♀, 0♂) (0♀, 1♂); 07-SRNP-66670, DHJPAR0025208; same data as for preceding except: 22.vii.2007. • 1 (0♀, 0♂) (0♀, 1♂); 07-SRNP-66675, DHJPAR0025213; same data as for preceding except: 22.vii.2007. • 1 (1♀, 0♂) (0♀, 0♂); 07-SRNP-66681, DHJPAR0025219; same data as for preceding except: 22.vii.2007. • 1 (0♀, 1♂) (0♀, 0♂); 07-SRNP-66683, DHJPAR0025221; same data as for preceding except: 22.vii.2007. • 1 (1♀, 0♂) (0♀, 0♂); 07-SRNP-66700, DHJPAR0025238; 22.vii.2007. • 1 (0♀, 1♂) (0♀, 0♂); 07-SRNP-66714, DHJPAR0025252; same data as for preceding except: 07.xii.2007. • 1 (1♀, 0♂) (0♀, 0♂); 07-SRNP-66720, DHJPAR0025258; same data as for preceding except: 14.x.2007. • 1 (0♀, 1♂) (0♀, 0♂); 07-SRNP-66788, DHJPAR0025326; same data as for preceding except: 16.vi.2007. • 1 (0♀, 0♂) (0♀, 1♂); 07-SRNP-66798, DHJPAR0025336; same data as for preceding except: 16.vi.2007. • 1 (0♀, 1♂) (0♀, 0♂); 07-SRNP-67599, DHJPAR0026294; same data as for preceding except: 03.viii.2007. • 1 (0♀, 0♂) (1♀, 0♂); 07-SRNP-67616, DHJPAR0026311; same data as for preceding except: 03.viii.2007. • 1 (1♀, 0♂) (0♀, 0♂); 07-SRNP-67617, DHJPAR0026312; same data as for preceding except: 03.viii.2007. • 1 (0♀, 0♂) (0♀, 1♂); 07-SRNP-67623, DHJPAR0026318; same data as for preceding except: 03.viii.2007. • 1 (0♀, 1♂) (0♀, 0♂); 08-SRNP-3308, DHJPAR0026889; same data as for preceding except: 05.iv.2008. • 1 (0♀, 0♂) (0♀, 1♂); 08-SRNP-3314, DHJPAR0026895; same data as for preceding except: 05.iv.2008. • 1 (0♀, 0♂) (0♀, 1♂); 08-SRNP-3352, DHJPAR0026933; same data as for preceding except: 05.iv.2008. • 1 (0♀, 1♂) (0♀, 0♂); 08-SRNP-3357, DHJPAR0026938; same data as for preceding except: 05.iv.2008. • 1 (0♀, 0♂) (0♀, 1♂); 08-SRNP-3370, DHJPAR0026951; same data as for preceding except: 05.iv.2008. • 1 (0♀, 0♂) (0♀, 1♂); 08-SRNP-3382, DHJPAR0026963; same data as for preceding except: 11.iv.2008. • 1 (1♀, 0♂) (0♀, 0♂); 08-SRNP-3386, DHJPAR0026967; same data as for preceding except: 11.iv.2008. • 1 (0♀, 1♂) (0♀, 0♂); 08-SRNP-3399, DHJPAR0026980; same data as for preceding except: 11.iv.2008. • 1 (0♀, 1♂) (0♀, 0♂); 08-SRNP-3436, DHJPAR0027017; same data as for preceding except: 11.iv.2008. • 1 (0♀, 0♂) (0♀, 1♂); 08-SRNP-3561, DHJPAR0027142; same data as for preceding except: 23.iv.2008. • 1 (0♀, 0♂) (1♀, 0♂); 08-SRNP-3579, DHJPAR0027160; same data as for preceding except: 23.iv.2008. • 1 (0♀, 1♂) (0♀, 0♂); 08-SRNP-3658, DHJPAR0027239; same data as for preceding except: 30.iv.2008. • 1 (0♀, 1♂) (0♀, 0♂); 08-SRNP-3685, DHJPAR0027266; same data as for preceding except: 30.iv.2008. • 1 (0♀, 0♂) (0♀, 1♂); 08-SRNP-3701, DHJPAR0027282; same data as for preceding except: 06.v.2008. • 1 (0♀, 1♂) (0♀, 0♂); 08-SRNP-3711, DHJPAR0027292; same data as for preceding except: 06.v.2008.

*Área de Conservación Guanacaste*, *Alajuela*, *Sector San Cristóbal*, *Potrero Argentina*: • 1 (0♀, 1♂) (0♀, 0♂); 07-SRNP-67015, DHJPAR0025553; pastures; Malaise; 520 m; 10.89021, -85.38803; 19.xii.2007; DH Janzen & W Hallwachs leg. • 1 (1♀, 0♂) (0♀, 0♂); 07-SRNP-67056, DHJPAR0025594; same data as for preceding except: 21.viii.2007. • 1 (1♀, 0♂) (0♀, 0♂); 07-SRNP-67074, DHJPAR0025612; same data as for preceding except: 02.x.2007. • 1 (0♀, 0♂) (0♀, 1♂); 07-SRNP-67120, DHJPAR0025658; same data as for preceding except: 28.xii.2007. • 1 (0♀, 0♂) (0♀, 1♂); 07-SRNP-67127, DHJPAR0025665; same data as for preceding except: 28.xii.2007. • 1 (1♀, 0♂) (0♀, 0♂); 07-SRNP-67143, DHJPAR0025681; same data as for preceding except: 16.vii.2007. • 1 (0♀, 0♂) (0♀, 1♂); 07-SRNP-67256, DHJPAR0025794; same data as for preceding except: 19.xi.2007. • 1 (0♀, 1♂) (0♀, 0♂); 07-SRNP-67272, DHJPAR0025810; same data as for preceding except: 19.xi.2007. • 1 (0♀, 1♂) (0♀, 0♂); 08-SRNP-3886, DHJPAR0027540; same data as for preceding except: 12.i.2008. • 1 (0♀, 0♂) (0♀, 1♂); 08-SRNP-3898, DHJPAR0027552; same data as for preceding except: 24.i.2008.

*Área de Conservación Guanacaste*, *Alajuela*, *Sector Rincón Rain Forest*, *Vado Río Francia*: • 1 (0♀, 1♂) (0♀, 0♂); 08-SRNP-41740, DHJPAR0026183; Malaise; 400 m; 10.90093, -85.28915; 01.iii.2008; DH Janzen & W Hallwachs leg. • 1 (0♀, 0♂) (0♀, 1♂); 08-SRNP-41786, DHJPAR0026229; same data as for preceding except: 31.iii.2008.

#### Diagnosis.

**Figure 210.**
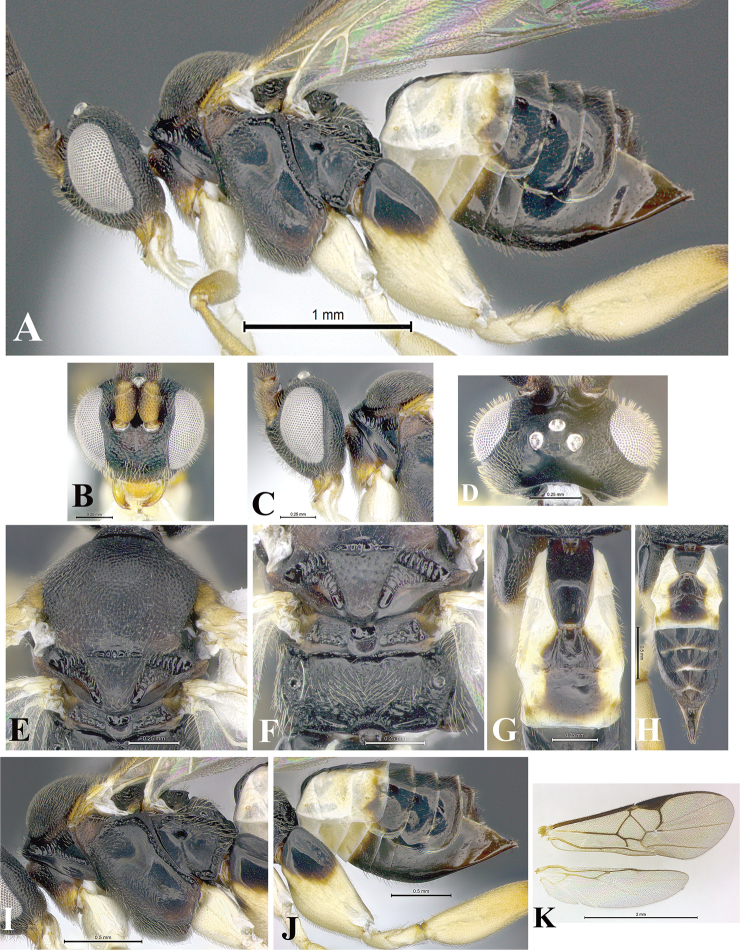
*Glyptapantelessureshnaiki* sp. nov. female 08-SRNP-2927 DHJPAR0026508 **A** Habitus **B, D** Head **B** Frontal view **D** Dorsal view **C** Head, pronotum, propleuron, lateral view **E** Mesonotum, dorsal view **F** Scutellum, metanotum, propodeum, dorsal view **G**T1–3, dorsal view **H, J** Metasoma **H** Dorsal view **J** Lateral view **I** Mesosoma, lateral view **K** Fore and hind wings, male 08-SRNP-3416 DHJPAR0026997.

Nucha surrounded by very short radiating carinae (Fig. 210F). Propodeum with a median longitudinal dent, but no trace of median longitudinal carina (Fig. 210F). Dorsal carina delimiting a dorsal furrow on propleuron present (Fig. 210C). Petiole on T1 parallel-sided in proximal half then narrowing (Fig. 210G). Precoxal groove deep (Fig. 210A, I). Anteroventral contour of mesopleuron straight/angulate or nearly so (Fig. 210A, I). Edges of median area on T2 polished and followed by a deep groove (Fig. 210G). Fore wing with r vein curved, outer side of junction of r and 2RS veins forming a distinct stub (Fig. 210K).

#### Coloration

(Fig. 210A–K). General body coloration black except scape and pedicel yellow, but inner and outer sides brown; last six-seven distal antennal flagellomeres completely yellow, remaining flagellomeres brown on both sides; labrum, mandible, and tegulae yellow; face medially just below antennal socket (toruli), both ends of propleuron, dorsal furrow of pronotum, epicnemial ridge, latero-ventral part of mesopleuron, mesosternum, mesoscutum distal corner, lunules, and lateral ends of metanotum with yellow/yellow-brown/reddish tints; glossa, maxillary and labial palps pale yellow/ivory. Eyes and ocelli silver. Fore and middle legs dark yellow, except coxae and trochanters pale yellow/ivory, and claws brown; hind legs dark yellow/orange except coxae proximally with a rounded black spot, remaining area pale yellow/ivory, trochanters and trochantellus pale yellow/ivory, femora pale yellow/ivory, but distally with a tiny brown dot, tibiae, tibial spurs and tarsomeres dark yellow/orange. Petiole on T1 dark brown and sublateral areas ivory/pale yellow; T2 with median and adjacent areas brown, contours of adjacent area well-defined, and lateral ends ivory/pale yellow; T3 medially brown, proximally dark area coinciding with the width of median and adjacent areas on T2; however, this dark area not touching the distal edge instead there is a broad and yellow-brown band followed by a pale-yellow/ivory area, lateral ends ivory/pale yellow; T4 and beyond completely dark brown/black; distally each tergum with a narrow ivory/pale yellow transparent band. In lateral view, T1– ivory/pale yellow; T3 proximal half ivory/pale, distal half brown; T4 and beyond dark brown/black. S1–3 ivory/pale yellow; S4 proximal half ivory/pale, distal half brown; penultimate sternum and hypopygium dark brown/black.

#### Description.

**Head** (Fig. 210A–D). Head rounded with pubescence long and dense. Proximal three antennal flagellomeres longer than wide (0.31:0.11, 0.35:0.11, 0.32:0.11), distal antennal flagellomere longer than penultimate (0.25:0.11, 0.17:0.11), antenna longer than body (4.70, 3.63); antennal scrobes-frons shallow. Face flat or nearly so, punctate-lacunose, interspaces wavy and longitudinal median carina present. Frons smooth. Temple wide, punctate-lacunose and interspaces wavy. Inner margin of eyes diverging slightly at antennal sockets; in lateral view, eye anteriorly convex and posteriorly straight. POL shorter than OOL (0.10, 0.14). Malar suture present. Median area between lateral ocelli slightly depressed. Vertex laterally pointed or nearly so and dorsally wide.

**Mesosoma** (Fig. 210A, E, F, I). Mesosoma dorsoventrally convex. Mesoscutum proximally convex and distally flat, punctation distinct throughout, interspaces wavy/lacunose. Scutellum triangular, apex sloped and fused with BS, but not in the same plane, scutellar punctation scattered throughout, in profile scutellum slightly convex, but on same plane as mesoscutum, phragma of the scutellum completely concealed; BS only very partially overlapping the MPM; ATS demilune with short stubs delineating the area; dorsal ATS groove with semicircular/parallel carinae. Transscutal articulation with small and heterogeneous foveae, area just behind transscutal articulation sloped, smooth and shiny. Metanotum with BM upward; MPM semicircular without median longitudinal carina; AFM without setiferous lobes and not as well delineated as PFM; PFM thick, smooth and with lateral ends rounded; ATM proximally with a groove with some sculpturing and distally with rugae. Propodeum with a median longitudinal dent, but no trace of median longitudinal carina, proximal half curved with medium-sized sculpture and distal half rugose; distal edge of propodeum with a flange at each side and without stubs; propodeal spiracle distally framed by a short concave carina; nucha surrounded by long radiating carinae. Pronotum with a distinct dorsal furrow, dorsally with a well-defined smooth band; central area of pronotum smooth, but both dorsal and ventral furrows with short parallel carinae. Propleuron with fine rugae and dorsally with a carina. Metasternum flat or nearly so. Contour of mesopleuron straight/angulate or nearly so; precoxal groove deep with transverse lineate sculpture; epicnemial ridge convex, teardrop-shaped.

**Legs** (Fig. 210A, J). Ventral margin of fore telotarsus entire without seta, fore telotarsus almost same width throughout and longer than fourth tarsomere (0.18, 0.10). Hind coxa with punctation only on ventral surface, dorsal outer depression present. Inner spur of hind tibia longer than outer spur (0.40, 0.17), entire surface of hind tibia with dense strong spines clearly differentiated by color and length. Hind telotarsus longer than fourth tarsomere (0.20, 0.13).

**Wings** (Fig. 210K). Fore wing with r vein curved; 2RS vein straight; r and 2RS veins forming a weak, even curve at their junction and outer side of junction forming a slight stub; 2M vein slightly curved/swollen; distally fore wing [where spectral veins are] with microtrichiae more densely concentrated than the rest of the wing; anal cell 1/3 proximally lacking microtrichiae, subbasal cell with microtrichiae virtually throughout; veins 2CUa and 2CUb completely spectral; vein 2 cu-a present as spectral vein, sometimes difficult to see; vein 2-1A proximally tubular and distally spectral, although sometimes difficult to see; tubular vein 1 cu-a curved, incomplete/broken and not reaching the edge of 1-1A vein. Hind wing with vannal lobe narrow, subdistally and subproximally straightened, and setae present only proximally.

**Metasoma** (Fig. 210A, G, H, J). Metasoma laterally compressed. Petiole on T1 finely sculptured distal, but only laterally, petiole parallel-sided in proximal half and then narrowing (length 0.46, maximum width 0.24, minimum width 0.14), and with scattered pubescence concentrated in the first distal third. Lateral grooves delimiting the median area on T2 clearly defined and reaching the distal edge of T2 (length median area 0.20, length T2 0.20), edges of median area polished and lateral grooves deep, median area as broad as long (length 0.20, maximum width 0.20, minimum width 0.12); T2 with scattered pubescence only distally. T3 longer than T2 (0.30, 0.20) and with scattered pubescence throughout. Pubescence on hypopygium dense.

**Cocoons.** Unknown.

#### Male.

Similar in shape and coloration to female; however, some males with the petiole, the median area on T2 and the T3 with yellow-brown/reddish tints. In other males, the distal band on T3 is brown; all the antennal flagellomeres have the same coloration throughout.

#### Etymology.

Suresh Naik is a research associate, DNA analyst at the Biodiversity Institute of Ontario (BIO), Ontario, Canada.

#### Distribution.

Adult parasitoids were collected in Costa Rica, ACG, Sector Rincón Rain Forest (Vado Río Francia) and Sector San Cristóbal (Bosque Trampa Malaise, Potrero Argentina, and Río Blanco Abajo), during April and June–December 2007; and January–May 2008 at 400, 500, 520 and 815 m in pastures and rain forest.

#### Biology.

Unknown.

#### Host.

Unknown.

### Glyptapanteles suzannegreenae

Taxon classificationAnimaliaHymenopteraBraconidae

Arias-Penna, sp. nov.

http://zoobank.org/F8989C45-E86A-436C-ABD3-C755D4B80860

Fig. 211

#### Female.

Body length 3.18 mm, antenna length 3.98 mm, fore wing length 3.83 mm.

#### Type material.

**Holotype**: ECUADOR • 1♀; EC-7074, YY-A217; Napo, Yanayacu Biological Station, Comedor Susanita (Loreto), Plot 93; cloud forest; 1,009 m; -0.7, -77.733333; 10.ix.2005; Drew Townsend leg.; caterpillar collected in second instar; cocoon formed on 07.x.2009; adult parasitoid emerged on 23.x.2005; (PUCE).

#### Diagnosis.

**Figure 211.**
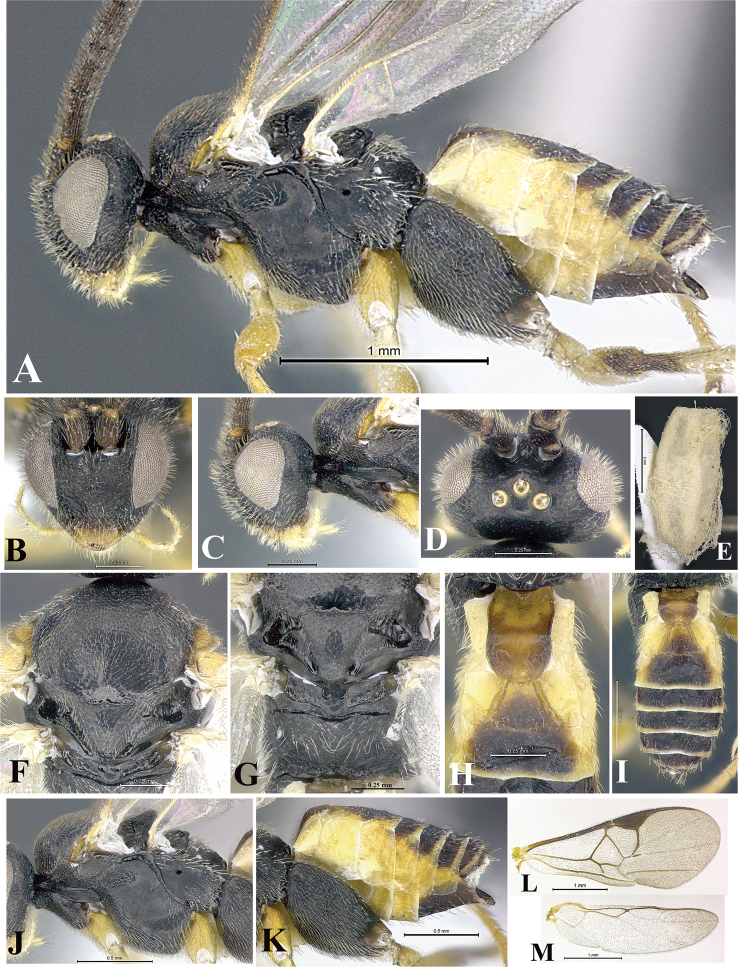
*Glyptapantelessuzannegreenae* sp. nov. female EC-7074 YY-A217 **A** Habitus **B, D** Head **B** Frontal view **D** Dorsal view **C** Head, pronotum, propleuron, lateral view **E** Cocoon **F** Mesonotum, dorsal view **G** Scutellum, metanotum, propodeum, dorsal view **H**T1–3, dorsal view **I, K** Metasoma **I** Dorsal view **K** Lateral view **J** Mesosoma, lateral view **L, M** Wings **L** Fore **M** Hind.

Face punctate-lacunose (Fig. 211B), distal antennal flagellomere longer than penultimate, scutellum in profile slightly convex, but on same plane as mesoscutum (Fig. 211J), petiole on T1 distally with lateral margins convex (Fig. 211H), mesoscutum punctation proximally distinct, but distally absent/dispersed (Fig. 211F), dorsal furrow of pronotum with a well-defined smooth band (Fig. 211C, J), precoxal groove deep, smooth and shiny (Fig. 211A, J), dorsal carina delimiting a dorsal furrow on propleuron absent (Fig. 211C), petiole on T1 parallel-sided but narrowing at apex (Fig. 211H), anteroventral contour of mesopleuron straight/angulate or nearly so (Fig. 211A, J), edges of median area on T2 polished and followed by a deep groove (Fig. 211H, I), and fore wing with r vein curved, outer side of junction of r and 2RS veins forming a slight stub (Fig. 211L).

#### Coloration

(Fig. 211A–M). General body coloration satin black except pedicel brown-reddish distally with a brown ring; scape yellow-brown; all antennal flagellomeres brown on both sides; labrum and mandibles yellow-brown; maxillary and labial palps, and tegulae yellow; clypeus brown-red/reddish. Eyes and ocelli silver. Fore middle and legs dark yellow except brown claws; hind legs dark yellow except black coxae distally yellow, femora distally brown, distal 1/3 of tibiae brown and proximally with a brown band, and tarsomeres brown, although basitarsus proximally with a yellow ring. Petiole on T1 dark yellow, however, laterally distal half with light yellow-brown tints, contours brown, and sublateral areas light yellow; T2 with median area with two colorations: 1/3 proximal yellow-brown and 2/3 distal brown, and lateral ends yellow; T3 mostly brown, but distally with a wide yellow band, and lateral ends yellow; T4 and beyond completely dark brown; distally each tergum with a wide yellow transparent band. In lateral view, T1–3 yellow; T3 and beyond yellow, but dorsally brown, the extent of that brown area remaining relatively constant from proximal to distal. S1–5 yellow; hypopygium brown.

#### Description.

**Head** (Fig. 211A–D). Head triangular with pubescence long and dense. Proximal three antennal flagellomeres longer than wide (0.25:0.09, 0.27:0.09, 0.26:0.09), distal antennal flagellomere longer than penultimate (0.17:0.07, 0.14:0.07), antenna longer than body (3.98, 3.18); antennal scrobes-frons shallow. Face convex, punctate-lacunose, interspaces wavy and longitudinal median carina present. Frons smooth. Temple wide, punctate-lacunose and interspaces wavy. Inner margin of eyes diverging slightly at antennal sockets; in lateral view, eye anteriorly convex and posteriorly straight. POL shorter than OOL (0.10, 0.13). Malar suture present. Median area between lateral ocelli without depression. Vertex laterally pointed or nearly so and dorsally wide.

**Mesosoma** (Fig. 211A, F, G, J). Mesosoma dorsoventrally convex. Distal 1/3 of mesoscutum with lateral margin slightly dented, punctation proximally distinct, but distally absent/dispersed, interspaces smooth. Scutellum triangular, apex sloped and fused with BS, but not in the same plane, scutellar punctation distinct throughout, in profile scutellum slightly convex, but on same plane as mesoscutum, phragma of the scutellum completely concealed; BS mostly overlapping the MPM; ATS demilune almost smooth; dorsal ATS groove with carinae only proximally. Transscutal articulation with small and heterogeneous foveae, area just behind transscutal articulation depressed centrally and with same kind of sculpture as mesoscutum. Metanotum with BM convex; MPM semicircular without median longitudinal carina; AFM without setiferous lobes and not as well delineated as PFM; PFM thick, smooth and with lateral ends rounded; ATM proximally with a groove with some sculpturing and distally smooth. Propodeum without median longitudinal carina, proximal half curved with medium-sized sculpture and distal half relatively polished and with a shallow dent at each side of nucha; distal edge of propodeum with a flange at each side and without stubs; propodeal spiracle without distal carina; nucha surrounded by very short radiating carinae. Pronotum with a distinct dorsal furrow, dorsally with a well-defined smooth band; central area of pronotum smooth, but both dorsal and ventral furrows with short parallel carinae. Propleuron finely sculptured only ventrally and dorsally without a carina. Metasternum convex. Contour of mesopleuron straight/angulate or nearly so; precoxal groove deep, smooth and shiny; epicnemial ridge widen.

**Legs** (Fig. 211A). Ventral margin of fore telotarsus slightly excavated and with a tiny curved seta, fore telotarsus almost same width throughout and longer than fourth tarsomere (0.15, 0.08). Hind coxa finely punctate throughout, and dorsal outer depression present. Inner spur of hind tibia longer than outer spur (0.49, 0.34), entire surface of hind tibia with dense strong spines clearly differentiated by color and length.

**Wings** (Fig. 211L, M). Fore wing with r vein slightly curved; 2RS vein straight; r and 2RS veins forming a weak, even curve at their junction and outer side of junction forming a slight stub; 2M vein slightly curved/swollen; distally fore wing [where spectral veins are] with microtrichiae more densely concentrated than the rest of the wing; anal cell 1/3 proximally lacking microtrichiae; subbasal cell with microtrichiae virtually throughout; veins 2CUa and 2CUb completely spectral; vein 2 cu-a present as spectral vein, sometimes difficult to see; vein 2-1A proximally tubular and distally spectral, although sometimes difficult to see; tubular vein 1 cu-a straight, incomplete/broken and not reaching the edge of 1-1A vein. Hind wing with vannal lobe narrow, subdistally and subproximally straightened, and setae evenly scattered in the margin.

**Metasoma** (Fig. 211A, H, I, K). Metasoma laterally compressed. Petiole on T1 finely sculptured only laterally, virtually parallel-sided over most of length, but barely narrowing at apex, apex truncate (length 0.42, maximum width 0.26, minimum width 0.20), and with scattered pubescence on distal half only laterally. Lateral grooves delimiting the median area on T2 clearly defined and reaching the distal edge of T2 (length median area 0.20, length T2 0.20), lateral grooves deep, median area broader than long (length 0.20, maximum width 0.28, minimum width 0.13); T2 with scattered pubescence only distally. T3 longer than T2 (0.26, 0.20) and with pubescence more notorious in distal half. Pubescence on hypopygium dense.

**Cocoons** (Fig. 211E). White or beige oval cocoon with ordered silk fibers, but covered by a net.

#### Comments.

The body is stout as well as the hind coxae (Fig. 211A), the limit between the mesopleuron and the metasternum with a flattened area, the petiole on T1 with lateral margins sinuous and distally curved (convex, Fig. 211H), and the hind telotarsus and fourth tarsomere are missing in the holotype.

#### Male.

Unknown.

#### Etymology.

Suzanne Rab Green’s research is focused upon Arctiinae, the tiger moths. Her major fields are general systematics, biodiversity, and biogeography. She is a curatorial assistant at American Museum of Natural History, New York, NY, USA.

#### Distribution.

Parasitized caterpillar was collected in Ecuador, Napo, Yanayacu Biological Station (Comedor Susanita –Loreto), during September 2005 at 1,009 m in cloud forest.

#### Biology.

The lifestyle of this parasitoid species is solitary.

#### Host.

Undetermined species of Pyralidae feeding on *Miconia* sp. (Melastomataceae). Caterpillar was collected in second instar.

### Glyptapanteles sydneycameronae

Taxon classificationAnimaliaHymenopteraBraconidae

Arias-Penna sp. nov.

http://zoobank.org/C9FC60AF-56DB-4BDD-902D-1DA396D503B8

Fig. 212

#### Female.

Body length 2.63 mm, antenna length 3.03 mm, fore wing length 2.92 mm.

#### Type material.

**Holotype**: COSTA RICA • 1♀; 09-SRNP-41351, DHJPAR0035467; Área de Conservación Guanacaste, Guanacaste, Sector Rincón Rain Forest, Sendero Alajuela; 405 m; 10.90528, -85.27882; 18.vi.2009; Anabelle Córdoba leg.; caterpillar collected in fourth instar; adult parasitoids emerged on 23.vi.2009, 29.vi.2009. *Mesochorus* (Ichneumonidae: Mesochorinae) was reported as hyperparasitoid; (CNC). **Paratypes.** • 61 (4♀, 2♂) (54♀, 1♂); 09-SRNP-41351, DHJPAR0035467; same data as for holotype; (CNC).

#### Other material.

**Reared material.** COSTA RICA: *Área de Conservación Guanacaste*, *Guanacaste*, *Sector Pitilla*, *Estación Pitilla*: • 19 (5♀, 5♂) (5♀, 4♂); 11-SRNP-31462, DHJPAR0045147; rain forest; 675 m; 10.98931, -85.42581; 22.v.2011; Ricardo Calero leg.; caterpillar collected in first instar; cocoons adhered to the larval cuticle and formed on 12.vi.2011; adult parasitoids emerged on 21.vi.2011.

*Área de Conservación Guanacaste*, *Guanacaste*, *Sector Pitilla*, *Sendero Carica*: • 212 (5♀, 5♂) (186♀, 16♂); 11-SRNP-31634, DHJPAR0045222; rain forest; 660 m; 10.99284, -85.42936; 14.vi.2011; Calixto Moraga leg.; caterpillar collected in fifth instar; cocoons adhered to the larval cuticle and formed on 22.vi.2011; adult parasitoids emerged on 29.vi.2011.

*Área de Conservación Guanacaste*, *Guanacaste*, *Sector Pitilla*, *Sendero Orosilito*: • 64 (5♀, 4♂) (55♀, 0♂); 11-SRNP-31539, DHJPAR0045239; rain forest; 900 m; 10.98332, -85.43623; 03.vi.2011; Freddy Quesada leg.; caterpillar collected in second instar; cocoons adhered to the leaf substrate; adult parasitoids emerged on 15.vii.2011.

#### Diagnosis.

**Figure 212.**
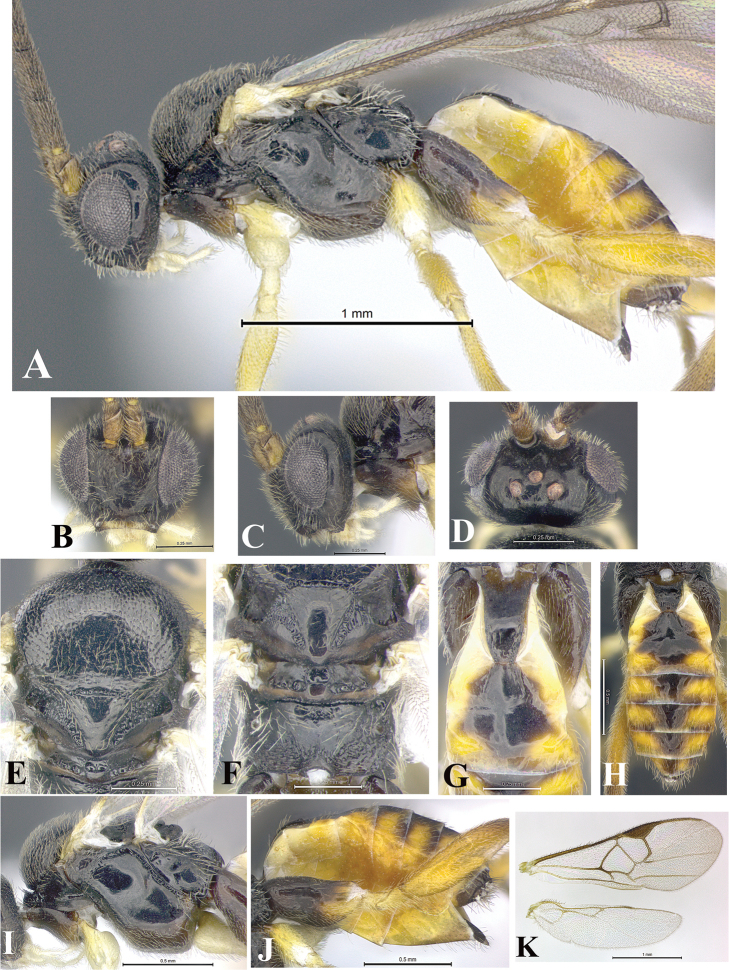
*Glyptapantelessydneycameronae* sp. nov. female 09-SRNP-41351 DHJPAR0035467 **A** Habitus **B, D** Head **B** Frontal view **D** Dorsal view **C** Head, pronotum, propleuron, lateral view **E** Mesonotum, dorsal view **F** Scutellum, metanotum, propodeum, dorsal view **G**T1–3, dorsal view **H, J** Metasoma **H** Dorsal view **J** Lateral view **I** Mesosoma, lateral view **K** Fore and hind wings.

Edges of median area on T2 polished and followed by a deep groove (Fig. 212G) and lateral grooves delimiting the median area on T2 distally losing definition on T2 (Fig. 212G), in lateral view, metasoma laterally compressed (Fig. 212A), T3 longer than T2 (Fig. 212H), inner margin of eyes diverging slightly at antennal sockets (Fig. 212B), petiole on T1 evenly narrowing distally (wide base to a narrow apex, Fig. 212G) and finely sculptured (Fig. 212G, H), propodeum without a median longitudinal dent (Fig. 212F), and fore wing with r vein straight, outer side of junction of r and 2RS veins forming a stub (Fig. 212K).

#### Coloration

(Fig. 212A–K). General body coloration black except scape and pedicel light brown; all antennal flagellomeres dorsally lighter (light brown) than ventrally (dark brown); glossa and tegulae yellow; mandible, clypeus, middle part of face just below antennal socket (toruli), propleuron (distally yellow), dorsal furrow of pronotum, epicnemial ridge, latero-ventral part of mesopleuron, mesosternum, distal corners of mesoscutum, lunules, BS, lateral ends of metanotum, PFM, BM with yellow-brown/reddish tints; maxillary and labial palps pale yellow/ivory. Eyes gray and ocelli reddish (in preserved specimen). Fore and middle legs dark yellow, except coxae and trochanters pale yellow/ivory, and brown claws; hind legs dark yellow except light brown coxae distally yellow, femora distally with a tiny brown dot, tibiae and tarsomeres brown. Petiole on T1 dark brown, contours darkened and sublateral areas ivory/pale yellow; T2 with median and adjacent areas brown, adjacent area with contours well-defined, and lateral ends dark yellow; T3 medially brown, proximally dark area coinciding with the width of median and adjacent areas on T2, but distally narrow, and not touching the distal edge, lateral ends yellow; T4 and beyond yellow, but medially with a dark brown area wider proximally than distally; distally each tergum with a narrow whitish translucent band. In lateral view, T1–2 yellow; T3 and beyond yellow, but distally with a narrow brown band. S1–5 yellow; hypopygium yellow, but medially light brown.

#### Description.

**Head** (Fig. 212A–D). Head rounded with pubescence long and dense. Proximal three antennal flagellomeres longer than wide (0.22:0.65, 0.23:0.65, 0.22:0.65), distal antennal flagellomere longer than penultimate (0.14:0.06, 0.11:0.06), antenna longer than body (3.03, 2.63); antennal scrobes-frons sloped and forming a shelf. Face flat or nearly so, scattered and finely punctate, interspaces smooth and longitudinal median carina present. Frons smooth. Temple wide, punctate and interspaces clearly smooth. Inner margin of eyes diverging slightly at antennal sockets; in lateral view, eye anteriorly convex and posteriorly straight. POL shorter than OOL (0.10, 0.13). Malar suture present. Median area between lateral ocelli slightly depressed. Vertex laterally pointed or nearly so and dorsally wide.

**Mesosoma** (Fig. 212A, E, F, I). Mesosoma dorsoventrally convex. Mesoscutum proximally convex and distally flat, punctation distinct throughout, interspaces smooth. Scutellum triangular, apex sloped and fused with BS, but not in the same plane, scutellar punctation indistinct throughout, in profile scutellum flat and on same plane as mesoscutum, phragma of the scutellum partially exposed; BS mostly overlapping the MPM; ATS demilune with complete undulate/reticulate carinae; dorsal ATS groove with carinae only proximally. Transscutal articulation with small and heterogeneous foveae, area just behind transscutal articulation nearly at the same level as mesoscutum (flat) and with same kind of sculpture as mesoscutum. Metanotum with BM wider than PFM (clearly differentiated); MPM semicircular without median longitudinal carina; AFM without setiferous lobes and not as well delineated as PFM; PFM thick and smooth; ATM proximally with a groove with some sculpturing and distally smooth. Propodeum without median longitudinal carina, proximal half weakly curved relatively polished and distal half slightly rugose; distal edge of propodeum with a flange at each side and without stubs; propodeal spiracle without distal carina; nucha without distinct short radiating carinae. Pronotum with a distinct dorsal furrow, dorsally with a well-defined smooth band; central area of pronotum smooth, but both dorsal and ventral furrows with short parallel carinae. Propleuron finely sculptured only ventrally and dorsally without a carina. Metasternum convex. Contour of mesopleuron convex; precoxal groove smooth, shiny and shallow, but visible; epicnemial ridge convex, teardrop-shaped.

**Legs** (Fig. 212A, J). Ventral margin of fore telotarsus entire without seta, fore telotarsus almost same width throughout and longer than fourth tarsomere (0.13, 0.06). Hind coxa with punctation only on ventral surface, dorsal outer depression absent. Inner spur of hind tibia longer than outer spur (0.22, 0.18), entire surface of hind tibia with dense strong spines clearly differentiated by color and length. Hind telotarsus as equal in length as fourth tarsomere (0.11, 0.12).

**Wings** (Fig. 212K). Fore wing with r vein straight; 2RS vein slightly convex to convex; r and 2RS veins forming a weak, even curve at their junction and outer side of junction forming a slight stub; 2M vein slightly curved/swollen; distally fore wing [where spectral veins are] with microtrichiae more densely concentrated than the rest of the wing; anal cell 1/3 proximally lacking microtrichiae; subbasal cell with microtrichiae virtually throughout; veins 2CUa and 2CUb completely spectral; vein 2 cu-a present as spectral vein, sometimes difficult to see; vein 2-1A proximally tubular and distally spectral, although sometimes difficult to see; tubular vein 1 cu-a curved and complete, but junction with 1-1A vein spectral. Hind wing with vannal lobe very narrow, subdistally straightened and subproximally evenly convex, and setae evenly scattered in the margin.

**Metasoma** (Fig. 212A, G, H, J). Metasoma laterally compressed. Petiole on T1 finely sculptured distal, but only laterally, evenly narrowing distally (length 0.40, maximum width 0.22, minimum width 0.09) and with scattered pubescence concentrated in the first distal third. Lateral grooves delimiting the median area on T2 clearly defined and reaching the distal edge of T2 (length median area 0.18, length T2 0.18), edges of median area polished and lateral grooves deep, median area broader than long (length 0.18, maximum width 0.20, minimum width 0.10); T2 with scattered pubescence only distally. T3 longer than T2 (0.21, 0.18) and with scattered pubescence only distally. Pubescence on hypopygium dense.

**Cocoons.** Oval cocoons with evenly smooth silk fibers. Cocoons adhered to the larval cuticle.

#### Male.

Coloration similar to that of females, although on metasoma the T4 and beyond terga are darker that the others and without the medial dark brown area present in females.

#### Etymology.

Sydney Anne Cameron is interested in social insect behavior, evolution, ecology, and phylogenetic theory of bees. Currently, she is a professor at UIUC, IL, USA.

#### Distribution.

Parasitized caterpillars were collected in Costa Rica, ACG, Sector Pitilla (Estación Pitilla, Sendero Carica, and Sendero Orosilito) and Sector Rincón Rain Forest (Sendero Alajuela), during June 2009 and May–June 2011 at 405 m, 660 m, 675 m, and 900 m in rain forest.

#### Biology.

The lifestyle of this parasitoid species is gregarious. *Mesochorus* (Ichneumonidae: Mesochorinae) was reported as hyperparasitoid.

#### Host.

*Pachygonidiadrucei* (Rothschild & Jordan), *Enyoocypete* (L.) and *Aleuroncarinata* (Walker) (Sphingidae: Macroglossinae) feeding on *Doliocarpusmultiflorus* (Dilleniaceae). Caterpillars were collected in first, second, fourth and fifth instar.

### Glyptapanteles taniaariasae

Taxon classificationAnimaliaHymenopteraBraconidae

Arias-Penna, sp. nov.

http://zoobank.org/218C99F4-B99C-4C9F-B4BB-A0687B5373FA

Fig. 213

#### Female.

Body length 2.92 mm, antenna length 3.88 mm, fore wing length 3.63 mm.

#### Type material.

**Holotype**: ECUADOR • 1♀; EC-34481, YY-A234; Napo, Yanayacu Biological Station, Yanayacu Road; cloud forest; 2,100 m; -0.566667, -77.866667; 20.vii.2008; Earthwatch volunteers leg.; caterpillar collected in second instar; white solitary cocoon formed on 04.viii.2008; adult parasitoid emerged on 23.xii.2008; (PUCE).

#### Diagnosis.

**Figure 213.**
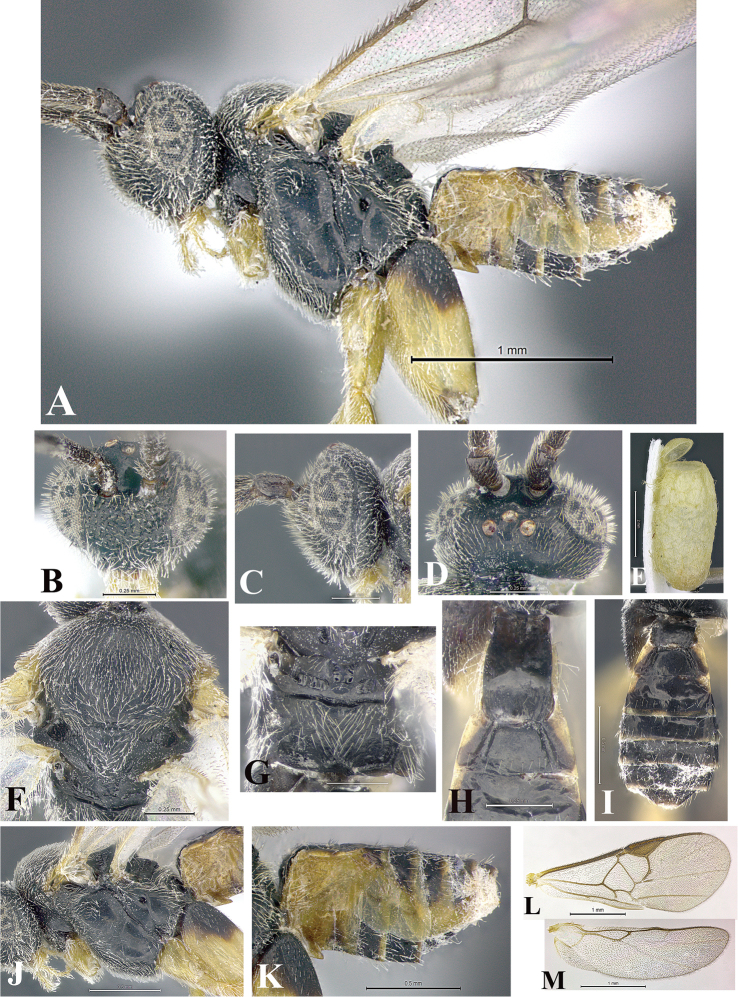
*Glyptapantelestaniaariasae* sp. nov. male EC-34481 YY-A234 **A** Habitus **B–D** Head **B** Frontal view **C** Lateral view **D** Dorsal view **E** Cocoon **F** Mesonotum, dorsal view **G** Metanotum, propodeum, dorsal view **H**T1–2, dorsal view **I, K** Metasoma **I** Dorsal view **K** Lateral view **J** Mesosoma, lateral view **L, M** Wings **L** Fore **M** Hind.

Face with dense fine punctations (Fig. 213B), distal antennal flagellomere subequal in length with penultimate, scutellum in profile convex and slightly higher than mesoscutum (Fig. 213A), petiole on T1 distally with lateral margins convex, petiole parallel-sided but barely narrowing at apex (Fig. 213H), mesoscutum punctation proximally distinct, but distally absent/dispersed (Fig. 213F), dorsal furrow of pronotum with a well-defined smooth band, precoxal groove deep, smooth and shiny (Fig. 213A, J), dorsal carina delimiting a dorsal furrow on propleuron absent. (Fig. 213H), anteroventral contour of mesopleuron straight/angulate or nearly so (Fig. 213A, J), edges of median area on T2 polished and followed by a deep groove (Fig. 213H), and fore wing with r vein curved, outer side of junction of r and 2RS veins forming a stub (Fig. 213L).

#### Coloration

(Fig. 213A–M). General body coloration satin black except apex of labrum, mandibles and pedicel brown-red/reddish; scape and all antennal flagellomeres (on both sides) dark brown; glossa, maxillary and labial palps, and tegulae light yellow-brown. Eyes gray/black and ocelli brownish/reddish (in preserved specimen). Fore and middle legs dark yellow/ light yellow-brown except tibiae and tarsomeres of middle legs with brown tints and claws brown; hind legs dark yellow/light yellow-brown except coxae dorsally with a brown spot, femora distally with a tiny brown area, tibiae distal half brown (coloration intensity increasing from proximal to distal) and proximally with a narrow brown band, and tarsomeres brown, although basitarsus proximally with a yellow band. Petiole on T1 black and sublateral areas yellow-brown; T2 with median and adjacent areas black, adjacent area with contours well-defined, and lateral ends yellow-brown; T3 mostly black except proximal corners of lateral ends; T4 and beyond completely brown; distally each tergum with a narrow yellow translucent band. In lateral view, T1–3 yellow-brown; T3 and beyond yellow-brown, but dorsally brown, extent of brown area remaining relatively constant from proximal to distal. S1–3 yellow-brown, but medially brown; S4 and beyond completely brown.

#### Description.

**Head** (Fig. 213A–D). Head rounded with pubescence long and dense. Proximal three antennal flagellomeres longer than wide (0.24:0.09, 0.30:0.09, 0.25:0.09), distal antennal flagellomere subequal in length with penultimate (0.15:0.07, 0.15:0.07), antenna longer than body (3.88, 2.92); antennal scrobes-frons sloped and forming a shelf. Face flat or nearly so, dense fine punctations, interspaces smooth and longitudinal median carina present. Frons smooth. Temple wide, punctate and interspaces clearly smooth. Inner margin of eyes diverging slightly at antennal sockets; in lateral view, eye anteriorly convex and posteriorly straight. POL shorter than OOL (0.10, 0.17). Malar suture present. Median area between lateral ocelli slightly depressed. Vertex laterally pointed or nearly so and dorsally wide.

**Mesosoma** (Fig. 213A, F, G, J). Mesosoma dorsoventrally convex. Distal 1/3 of mesoscutum with lateral margin slightly dented, punctation proximally distinct, but distally absent/dispersed, interspaces with microsculpture. Scutellum triangular, apex sloped and fused with BS, but not in the same plane, scutellar punctation scattered throughout, in profile scutellum convex and slightly higher than mesoscutum, phragma of the scutellum partially exposed; BS only very partially overlapping the MPM; ATS demilune with short stubs delineating the area; dorsal ATS groove with carinae only proximally. Transscutal articulation with small and heterogeneous foveae, area just behind transscutal articulation depressed centrally, smooth and shiny. Metanotum with BM wider than PFM (clearly differentiated); MPM circular without median longitudinal carina; AFM without setiferous lobes and not as well delineated as PFM; PFM thick, smooth and with lateral ends rounded; ATM proximally with a well-defined row of foveae and distally smooth. Propodeum without median longitudinal carina, proximal half curved with medium-sized sculpture and distal half relatively polished and with a shallow dent at each side of nucha; distal edge of propodeum with a flange at each side and without stubs; propodeal spiracle distally framed by a short concave carina; nucha surrounded by long radiating carinae. Pronotum with a distinct dorsal furrow, dorsally with a well-defined smooth band; central area of pronotum and dorsal furrow smooth, but ventral furrow with short parallel carinae. Propleuron with a mix of rugae and fine punctation, dorsally without a carina. Metasternum convex. Contour of mesopleuron straight/angulate or nearly so; precoxal groove deep, smooth and shiny; epicnemial ridge widen.

**Legs** (Fig. 213A). Ventral margin of fore telotarsus excavated with conspicuous curved seta over this excavation, fore telotarsus almost same width throughout and longer than fourth tarsomere (0.17, 0.10). Hind coxa with dorsal half sparsely punctate, ventral half densely punctate, and dorsal outer depression present. Inner spur of hind tibia longer than outer spur (0.46, 0.31), entire surface of hind tibia with dense strong spines clearly differentiated by color and length. Hind telotarsus longer than fourth tarsomere (0.18, 0.16).

**Wings** (Fig. 213L, M). Fore wing with r vein curved; 2RS vein straight; r and 2RS veins forming a weak, even curve at their junction and outer side of junction forming a distinct stub; 2M vein slightly curved/swollen; distally fore wing [where spectral veins are] with microtrichiae more densely concentrated than the rest of the wing; anal cell completely covered by microtrichiae; subbasal cell with microtrichiae virtually throughout; veins 2CUa and 2CUb completely spectral;vein 2 cu-a present as spectral vein, sometimes difficult to see; vein 2-1A proximally tubular and distally spectral, although sometimes difficult to see; tubular vein 1 cu-a curved, incomplete/broken and not reaching the edge of 1-1A vein. Hind wing with vannal lobe very narrow, subdistally and subproximally straightened, and setae evenly scattered in the margin.

**Metasoma** (Fig. 213A, H, I, K). Metasoma laterally compressed. Petiole on T1 completely smooth and polished, with faint, satin-like sheen, virtually parallel-sided over most of length, but barely narrowing at apex, apex truncate (length 0.37, maximum width 0.25, minimum with 0.20), and with scattered pubescence concentrated in the first distal third. Lateral grooves delimiting the median area on T2 clearly defined and reaching the distal edge of T2 (length median area 0.17, length T2 0.17), lateral grooves deep, median area broader than long (length 0.17, maximum width 0.25, minimum width 0.13); T2 with a distinctive row of pubescence only at the distal margin. T3 longer than T2 (0.26, 0.17) and with a distinctive row of pubescence only at the distal margin. Pubescence on hypopygium dense.

**Cocoons** (Fig. 213E). White or beige oval cocoon with ordered silk fibers, but covered by a net.

#### Comments.

The limit between the mesopleuron and the metasternum with a flattened area, the petiole on T1 distally slightly wider and lateral margins more curved (convex) than proximally (Fig. 213H), the body is stout, short and covered with dense pubescence, and the hind coxae is stout (Fig. 213A).

#### Male.

Unknown.

#### Etymology.

Tania Milena Arias-Penna is a Colombian entomologist. Her research has been focused on taxonomy and systematics of ants (Ponerinae) and parasitoid wasps (Platygastroidea). She is DCAP’s sister.

#### Distribution.

Parasitized caterpillar was collected in Ecuador, Napo, Yanayacu Biological Station (Yanayacu Road), during July 2008 at 2,100 m in cloud forest.

#### Biology.

The lifestyle of this parasitoid species is solitary.

#### Host.

*Pantherodesunciaria* Guenée (Geometridae: Ennominae) feeding on *Boehmeriabullata* (Urticaceae). Caterpillar was collected in second instar.

### Glyptapanteles tanyadapkeyae

Taxon classificationAnimaliaHymenopteraBraconidae

Arias-Penna, sp. nov.

http://zoobank.org/E830D252-1FFF-4D97-A9EC-69553A136A3D

Fig. 214

#### Male.

Body length 2.37 mm, antenna length 3.03 mm. fore wing length 2.58 mm.

#### Type material.

**Holotype**: COSTA RICA • 1♀; 08-SRNP-31475, DHJPAR0031101; Área de Conservación Guanacaste, Guanacaste, Sector Pitilla, Pasmompa; rain forest; 440 m; 11.01926, -85.40997; 24.vi.2008; Manuel Ríos leg.; caterpillar collected in fourth instar; cocoons adhered to the larval cuticle and formed on 26.vi.2008; adult parasitoids emerged on 04.vii.2008; (CNC). **Paratypes.** • 16 (0♀, 4♂), (0♀, 12♂); 08-SRNP-31475, DHJPAR0031101; same data as for holotype; (CNC).

#### Diagnosis.

**Figure 214.**
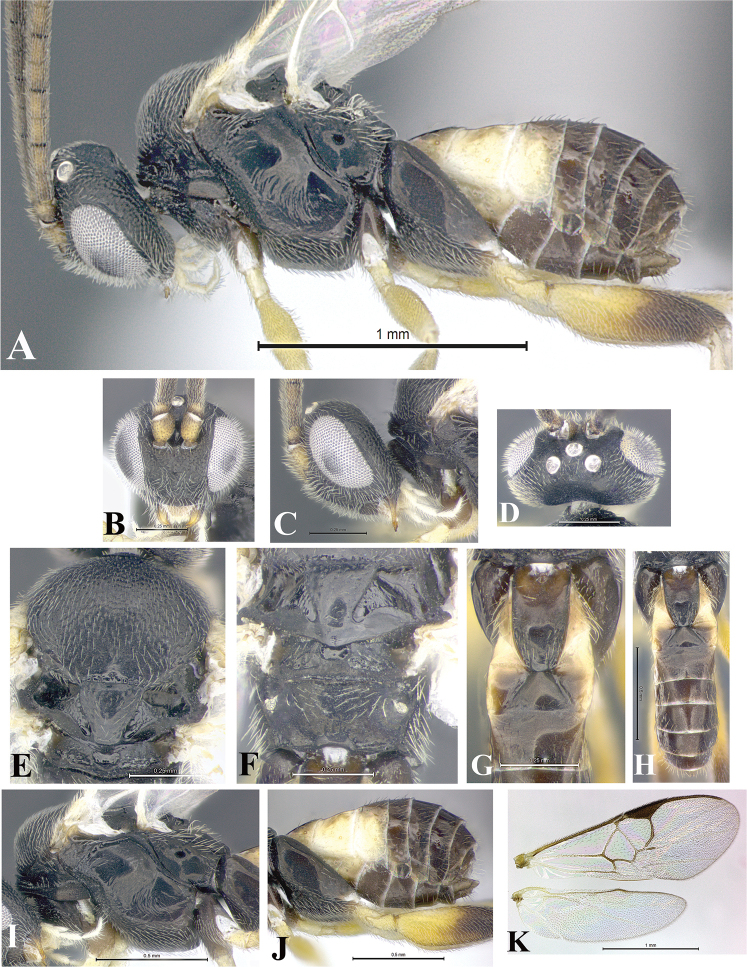
*Glyptapantelestanyadapkeyae* sp. nov. male 08-SRNP-31435 DHJPAR0031101 **A** Habitus **B, D** Head **B** Frontal view **D** Dorsal view **C** Head, pronotum, propleuron, lateral view **E** Mesonotum, dorsal view **F** Scutellum, metanotum, propodeum, dorsal view **G**T1–3, dorsal view **H, J** Metasoma **H** Dorsal view **J** Lateral view **I** Mesosoma, lateral view **K** Fore and hind wings.

Medioposterior band of scutellum only very partially overlapping the medioanterior pit of metanotum (Fig. 214E, F), fore wing with vein 2-1A absent, 2RS vein straight, outer side of junction of r and 2RS veins not forming a stub (Fig. 214K), medioanterior pit of metanotum semicircular without median longitudinal carina (Fig. 214E, F), anteroventral contour of mesopleuron convex (Fig. 214A, I), petiole on T1 distally with lateral margins relatively straight (Fig. 214G, H), propodeum without median longitudinal carina, propodeal spiracle without distal carina (Fig. 214F), nucha surrounded by very short radiating carinae (Fig. 214F), antenna longer than body, and lateral grooves delimiting the median area on T2 distally losing definition (Fig. 214G, H).

#### Coloration

(Fig. 214A–K). General body coloration black except yellow scape with inner sides brown; yellow pedicel proximally with a brown ring; labrum, mandible, and glossa yellow; first four-five proximal antennal flagellomeres dorsally lighter (light brown) than ventrally (dark brown), remaining flagellomeres dark brown on both sides; maxillary and labial palps, and tegulae ivory/pale yellow. Eyes and ocelli silver. Fore and middle legs yellow except fore coxae light brown, middle coxae brown, and claws brown; hind legs yellow except coxae completely brown/black, distal 1/3 of femora brown, distal half of tibiae brown, and tarsomeres brown except proximal 1/3 of basitarsus yellow. Petiole on T1 with proximal half light brown, distal half dark brown, contours darkened, and sublateral areas pale yellow-brown; T2 with median and adjacent areas brown, adjacent area wide with smears limits, lateral ends very narrow and pale yellow-brown; T3 and beyond completely brown; distally each tergum with a narrow whitish transparent band. In lateral view, T1–2 completely ivory/pale yellow; T3 ivory/pale yellow, but dorsally with a tiny brown area; T4 and beyond brown. S1–3 yellow, but medially brown; S4 and beyond completely brown.

#### Description.

**Head** (Fig. 214A–D). Head rounded with pubescence long and dense. Proximal three antennal flagellomeres longer than wide (0.20:0.05, 0.20:0.05, 0.20:0.05), distal antennal flagellomere longer than penultimate (0.12:0.05, 0.09:0.05), antenna longer than body (3.03, 2.37); antennal scrobes-frons shallow. Face flat or nearly so, with dense fine punctations, interspaces smooth and longitudinal median carina present. Frons smooth. Temple wide, punctate-lacunose and interspaces wavy. Inner margin of eyes diverging slightly at antennal sockets; in lateral view, eye anteriorly convex and posteriorly straight. POL shorter than OOL (0.10, 0.12). Malar suture present. Median area between lateral ocelli slightly depressed. Vertex laterally rounded and dorsally wide.

**Mesosoma** (Fig. 214A, E, F, I). Mesosoma dorsoventrally convex. Distal 1/3 of mesoscutum with lateral margin slightly dented, punctation proximally distinct, but distally with a polished area, interspaces wavy/lacunose. Scutellum triangular, apex sloped and fused with BS, scutellar punctation distinct throughout, in profile scutellum slightly convex, but on same plane as mesoscutum, phragma of the scutellum partially exposed; BS only very partially overlapping the MPM; ATS demilune almost smooth; dorsal ATS groove smooth. Transscutal articulation with small and heterogeneous foveae, area just behind transscutal articulation depressed centrally, smooth and shiny. Metanotum with BM upward; MPM semicircular without median longitudinal carina; AFM without setiferous lobes and not as well delineated as PFM; PFM thick, smooth and with lateral ends rounded; ATM proximally with a groove with some sculpturing and distally smooth. Propodeum without median longitudinal carina, proximal half weakly curved with medium-sized sculpture and distal half relatively polished; distal edge of propodeum with a flange at each side and without stubs; propodeal spiracle without distal carina; nucha surrounded by very short radiating carinae. Pronotum with a distinct dorsal furrow, dorsally with a well-defined smooth band; central area of pronotum smooth, but both dorsal and ventral furrows with short parallel carinae. Propleuron with fine punctations throughout and dorsally without a carina. Metasternum convex. Contour of mesopleuron convex; precoxal groove deep with transverse lineate sculpture; epicnemial ridge convex, teardrop-shaped.

**Legs** (Fig. 214A, J). Ventral margin of fore telotarsus entire without seta, fore telotarsus almost same width throughout and longer than fourth tarsomere (0.11, 0.06). Hind coxa with punctation only on ventral surface, dorsal outer depression present. Inner spur of hind tibia longer than outer spur (0.24, 0.17), entire surface of hind tibia with dense strong spines clearly differentiated by color and length. Hind telotarsus longer than fourth tarsomere (0.12, 0.10).

**Wings** (Fig. 214K). Fore wing with r vein curved; 2RS vein straight; r and 2RS veins forming a weak, even curve at their junction and outer side of junction not forming a stub; 2M vein slightly curved/swollen; distally fore wing [where spectral veins are] with microtrichiae more densely concentrated than the rest of the wing; anal cell 1/3 proximally lacking microtrichiae; subbasal cell with a small smooth area; vein 2CUa absent and vein 2CUb spectral, vein 2 cu-a absent; vein 2-1A absent; tubular vein 1 cu-a curved and complete, but junction with 1-1A vein spectral. Hind wing with vannal lobe wide, subdistally and subproximally straightened, and setae absent proximally, but scattered distally.

**Metasoma** (Fig. 214A, G, H, J). Metasoma laterally compressed. Petiole on T1 distally finely sculptured, but only laterally, virtually parallel-sided over most of length, but narrowing over distal 1/3 (length 0.31, maximum width 0.18, minimum width 0.09), and with scattered pubescence concentrated in the first distal third. Lateral grooves delimiting the median area on T2 distally losing definition (length median area 0.10, length T2 0.13), edges of median area polished and lateral grooves deep, median area broader than long (length 0.10, maximum width 0.20, minimum width 0.07); T2 with scattered pubescence only distally. T3 longer than T2 (0.17, 0.13) and with scattered pubescence throughout.

**Cocoons** (Fig. 4J). Brown oval cocoons with silk fibers evenly smooth. Cocoons adhered to the larval cuticle.

#### Female.

Unknown.

#### Etymology.

Tanya Heckmann Dapkey’s interests lie in ecological monitoring and restoration. She works at the University of Pennsylvania, Philadelphia, PA, USA, on The Barcode of Life Initiative.

#### Distribution.

The parasitized caterpillar was collected in Costa Rica, ACG, Sector Pitilla (Pasmompa), during June 2008 at 440 m in rain forest.

#### Biology.

The lifestyle of this parasitoid species is gregarious.

#### Host.

*Perochapela* Poole (Geometridae: Ennominae) feeding on *Anemopaegmaorbiculatum* (Bignoniaceae). Caterpillar was collected in fourth instar.

### Glyptapanteles thibautdelsinnei

Taxon classificationAnimaliaHymenopteraBraconidae

Arias-Penna, sp. nov.

http://zoobank.org/6B94055A-7D27-49B3-8D0E-B1E45285BCBD

Fig. 215

#### Male.

Body length 2.78 mm, antenna length 3.23 mm, fore wing length 3.05 mm.

#### Type material.

**Holotype**: ECUADOR • 1♀; EC-43790, YY-A188; Napo, Yanayacu Biological Station, Sendero Baboo; cloud forest; 2,051 m; -0.583333, -77.897778; 26.xi.2009; CAPEA leg.; caterpillar collected in fourth instar; cocoons formed on 07.xii.2009; adult parasitoids emerged on 21.xii.2009; (PUCE).

#### Diagnosis.

**Figure 215.**
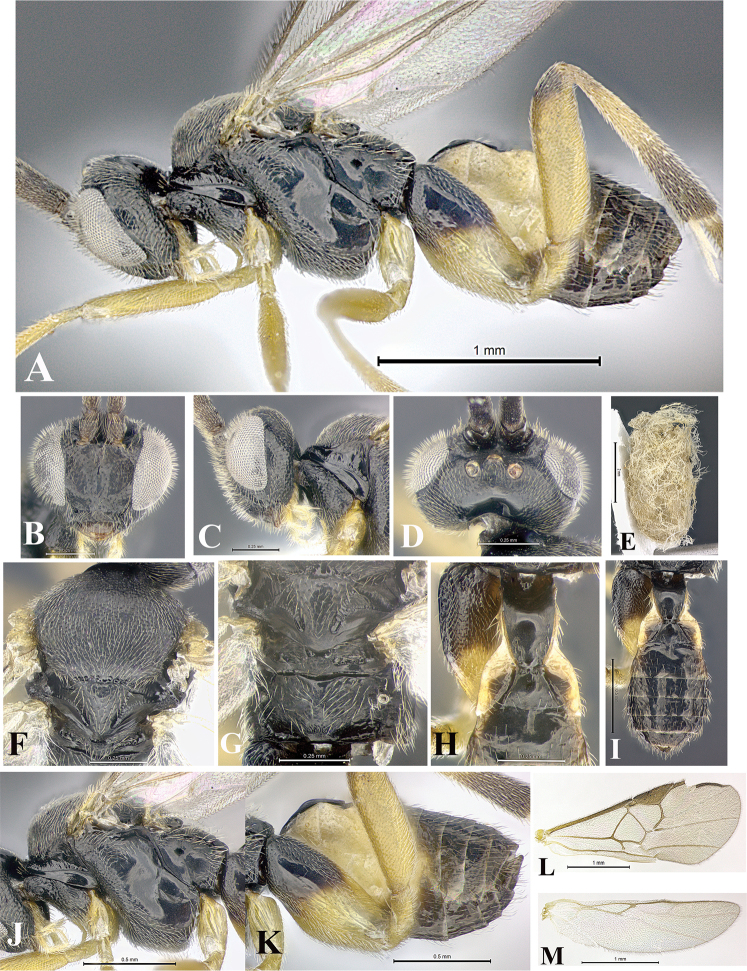
*Glyptapantelesthibautdelsinnei* sp. nov. male EC-43790 YY-A188 **A** Habitus **B, D** Head **B** Frontal view **D** Dorsal view **C** Head, pronotum, propleuron, lateral view **E** Cocoon **F** Mesonotum, dorsal view **G** Scutellum, metanotum, propodeum, dorsal view **H**T1–2, dorsal view **I, K** Metasoma **I** Dorsal view **K** Lateral view **J** Mesosoma, lateral view **L, M** Wings **L** Fore **M** Hind.

Petiole on T1 distally with lateral margins relatively straight, parallel-sided in proximal half then narrowing (Fig. 215H, I), mesoscutum punctation distinct throughout (Fig. 215F), dorsal furrow of pronotum without a smooth band (Fig. 215C, J), precoxal groove deep, smooth and shiny (Fig. 215J), dorsal carina delimiting a dorsal furrow on propleuron absent (Fig. 215C), anteroventral contour of mesopleuron straight/angulate or nearly so (Fig. 215J), edges of median area on T2 polished and followed by a deep groove (Fig. 215H), and fore wing with r vein curved, outer side of junction of r and 2RS veins forming a distinct stub (Fig. 215L).

#### Coloration

(Fig. 215A–M). General body coloration polished black except yellow-brown scape dorsally with a brown ring; pedicel yellow-brown; first five-six proximal antennal flagellomeres dorsally lighter (light brown) than ventrally (dark brown), remaining flagellomeres dark brown on both sides; labrum and mandible brown-red; glossa, maxillary and labial palps, and tegulae light yellow-brown. Eyes silver and ocelli reddish (in preserved specimen). Fore and middle legs light yellow-brown, except brown claws; hind legs light yellow-brown except coxae proximally 1/3 brown/black, femora distally with a tiny brown spot, tibiae distal half brown and proximally with a brown band, and tarsomeres brown. Petiole on T1 brown, although proximally with some brown-red tints, contours darkened and sublateral areas yellow; T2 with median and adjacent areas brown, adjacent area with contours well-defined, both dark areas forming a rectangle-shaped area, and lateral ends yellow-brown; T3 brown, corners of lateral ends proximally with brown-reddish tints; T4 and beyond completely brown; distally each tergum with a very narrow whitish translucent band. In lateral view, T1–2 yellow; T3–4 yellow, but dorsally brown, extent of brown area larger on T4 than T3; T5 and beyond brown. S1–2 yellow; S3–4 yellow, but medially brown, remaining sterna brown.

#### Description.

**Head** (Fig. 215A–D). Head rounded with pubescence long and dense. Proximal three antennal flagellomeres longer than wide (0.27:0.08, 0.29:0.08, 0.28:0.08), distal antennal flagellomere longer than penultimate (0.18:0.06, 0.15:0.06), antenna longer than body (3.23, 2.78); antennal scrobes-frons shallow. Face with punctations barely noticeable, distal half dented only laterally, interspaces smooth and longitudinal median carina present. Frons smooth. Temple wide, punctate-lacunose and interspaces wavy. Inner margin of eyes diverging slightly at antennal sockets; in lateral view, eye anteriorly convex and posteriorly straight. POL subequal in length with OOL (0.10, 0.11). Malar suture faint. Median area between lateral ocelli slightly depressed. Vertex laterally pointed or nearly so and dorsally wide.

**Mesosoma** (Fig. 215A, F, G, J). Mesosoma dorsoventrally convex. Mesoscutum with narrow grooves/dents taking the place of notauli, punctation distinct throughout, interspaces wavy/lacunose. Scutellum triangular, apex sloped and fused with BS, but not in the same plane, scutellar punctation distinct throughout, in profile scutellum flat and on same plane as mesoscutum, phragma of the scutellum partially exposed; BS mostly overlapping the MPM; ATS demilune with short stubs delineating the area; dorsal ATS groove with semicircular/parallel carinae. Transscutal articulation with small and heterogeneous foveae, area just behind transscutal articulation sloped and with same kind of sculpture as mesoscutum. Metanotum with BM convex; MPM semicircular without median longitudinal carina; AFM with a small lobe and not as well delineated as PFM; PFM thick, smooth and with lateral ends rounded; ATM proximally with a groove with some sculpturing and distally smooth; propodeum without median longitudinal carina; proximal half straight or nearly so and with medium-sized sculpture and distal half relatively polished; distal edge of propodeum with a flange at each side and without stubs; propodeal spiracle without distal carina; nucha surrounded by very short radiating carinae. Pronotum virtually without trace of dorsal furrow, dorsally without a smooth band; short parallel carinae only in ventral furrow. Propleuron with a mix of rugae and fine punctation, dorsally without a carina. Metasternum flat or nearly so. Contour of mesopleuron straight/angulate or nearly so; precoxal groove deep, smooth and shiny; epicnemial ridge widen.

**Legs** (Fig. 215A, J, K). Ventral margin of fore telotarsus entire without seta, fore telotarsus almost same width throughout and longer than fourth tarsomere (0.15, 0.07). Medially hind coxa smooth, dorsally with scattered punctation and ventrally with dense punctation, dorsal outer depression present. Inner spur of hind tibia longer than outer spur (0.31, 0.21), entire surface of hind tibia with dense strong spines clearly differentiated by color and length. Hind telotarsus as equal in length as fourth tarsomere (0.15, 0.14).

**Wings** (Fig. 215L, M). Fore wing with r vein curved; 2RS vein slightly convex to convex; r and 2RS veins forming a weak, even curve at their junction and outer side of junction forming a slight stub; 2M vein slightly curved/swollen; distally fore wing [where spectral veins are] with microtrichiae more densely concentrated than the rest of the wing; anal cell 1/3 proximally lacking microtrichiae; subbasal cell with microtrichiae virtually throughout; veins 2CUa and 2CUb completely spectral; vein 2 cu-a present as spectral vein, sometimes difficult to see; vein 2-1A proximally tubular and distally spectral, although sometimes difficult to see; tubular vein 1 cu-a curved, incomplete/broken and not reaching the edge of 1-1A vein. Hind wing with vannal lobe very narrow, subdistally and subproximally straightened, and setae present proximally, but absent distally.

**Metasoma** (Fig. 215A, H, I, K). Metasoma laterally compressed. Petiole on T1 finely sculptured laterodistally, parallel-sided in proximal half and then narrowing (length 0.36, maximum width 0.18, minimum width 0.09) and with scattered pubescence concentrated in the first distal third. Lateral grooves delimiting the median area on T2 clearly defined and reaching the distal edge of T2 (length median area 0.15, length T2 0.15), edges of median area polished and lateral grooves deep, median area broader than long (length 0.15, maximum width 0.20, minimum width 0.07); T2 with scattered pubescence throughout. T3 longer than T2 (0.22, 0.15) and with scattered pubescence throughout.

**Cocoons** (Fig. 215E). White or beige oval cocoon with silk fibers messy/disordered/fluffy.

#### Comments.

The BM is convex and punctate (Fig. 215G), the limit between the mesopleuron and the metasternum forming a flattened area, and the body is elongate and slim (Fig. 215A).

#### Female.

Unknown.

#### Etymology.

Thibaut Dominique Delsinne is a French ecologist. His major fields include insect taxonomy, species diversity, ecology, and evolution. Currently, he is working as entomologist at the Société d’Histoire Naturelle Alcide d’Orbigny, France.

#### Distribution.

Parasitized caterpillar was collected in Ecuador, Napo, Yanayacu Biological Station (Sendero Baboo), during November 2009 at 2,051 m in cloud forest.

#### Biology.

The lifestyle of this parasitoid species is solitary.

#### Host.

Undetermined species of Geometriidae feeding on *Chusqueascandens* (Poaceae). Caterpillar was collected in fourth instar.

### Glyptapanteles thomaspapei

Taxon classificationAnimaliaHymenopteraBraconidae

Arias-Penna, sp. nov.

http://zoobank.org/14DB45C1-C2FC-402D-998D-E182A01DF24D

Fig. 216

#### Female.

Body length 2.48 mm [with only three first metasomal segments, remaining missing], antenna length 3.68 mm, fore wing length 3.58 mm.

#### Type material.

**Holotype**: ECUADOR • 1♀; EC-38570, YY-A186; Napo, Yanayacu Biological Station, Sendero Culo del Mundo, Plot 430; cloud forest; 2,414 m; -0.590833, -77.896389; 07.v.2009; Mattias Lanas leg.; caterpillar collected in third instar; cocoon formed on 09.v.2009; adult parasitoid emerged on 30.v.2009; [metasoma with only three terga present]; (PUCE).

#### Diagnosis.

**Figure 216.**
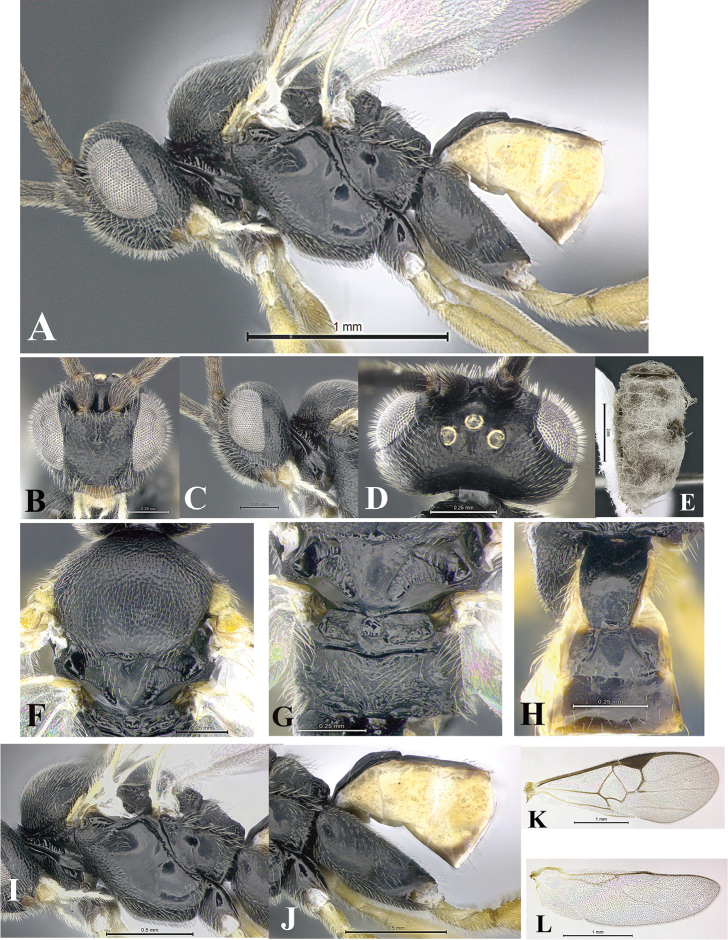
*Glyptapantelesthomaspapei* sp. nov. female EC-38570 YY-A186 **A** Habitus **B, D** Head **B** Frontal view **D** Dorsal view **C** Head, pronotum, propleuron, lateral view **E** Cocoon **F** Mesonotum, dorsal view **G** Scutellum, metanotum, propodeum, dorsal view **H**T1–2, dorsal view **I** Mesosoma, lateral view **J** Metasoma, lateral view **K, L** Wings **K** Fore **L** Hind.

Inner margin of eyes diverging slightly at antennal sockets (Fig. 216B), medioanterior pit of metanotum circular with a short proximal carina (Fig. 216G), mesoscutum punctation proximally distinct, but distally absent/dispersed (Fig. 216F), phragma of the scutellum widely visible (Fig. 216G), petiole on T1virtually parallel-sided over most of length but narrowing over distal 1/3 and finely sculptured (Fig. 216H), propodeum without a median longitudinal dent (Fig. 216G), lateral grooves delimiting the median area on T2 distally losing definition on T2 (Fig. 216H), and fore wing with r vein straight, outer side of junction of r and 2RS veins forming a stub (Fig. 216K).

#### Coloration

(Fig. 216A–L). General body coloration polished satin black except yellow-brown scape; pedicel distally brown; first three-four proximal antennal flagellomeres dorsally lighter (light brown) than ventrally (dark brown), remaining flagellomeres dark brown on both sides; labrum and mandible yellow-brown; glossa and tegulae dark yellow; maxillary and labial palps light yellow. Eyes and ocelli silver. Fore and middle legs dark yellow, except brown-red/black coxae, and brown claws; hind legs dark yellow except black coxae, femora distally brown, distal half of tibiae and tarsomeres brown, although basitarsus proximally with a yellow band. Petiole on T1 brown, contours darkened and sublateral areas yellow; T2 with median and adjacent areas brown and both areas forming a rectangle-shaped area, adjacent area with contours well-defined, and lateral ends yellow-brown; T3 brown and proximal half of lateral ends yellow; T4 and beyond missing. In lateral view, T1–3 yellow; remaining terga missing. S1–2 yellow; S3 yellow, but medially with a spot brown; remaining sterna missing.

#### Description.

**Head** (Fig. 216A–D). Head rounded with pubescence long and dense. Proximal three antennal flagellomeres longer than wide (0.27:0.07, 0.29:0.07, 0.28:0.07), distal antennal flagellomere longer than penultimate (0.15:0.06, 0.13:0.06), antennal scrobes-frons sloped and forming a shelf. Face flat or nearly so, punctations barely noticeable, interspaces smooth and longitudinal median carina present. Frons smooth. Temple narrow, punctate-lacunose and interspaces wavy. Inner margin of eyes diverging slightly at antennal sockets; in lateral view, eye anteriorly convex and posteriorly straight. POL shorter than OOL (0.10, 0.14). Malar suture absent or difficult to see. Median area between lateral ocelli slightly depressed. Vertex laterally rounded and dorsally wide.

**Mesosoma** (Fig. 216A, F, G, I). Mesosoma dorsoventrally convex. Mesoscutum proximally convex and distally flat, punctation distinct proximally, but absent/dispersed distally, interspaces wavy/lacunose. Scutellum triangular, apex sloped and fused with BS, but not in the same plane, scutellar punctation distinct peripherally and absent centrally, in lateral view, scutellum slightly convex, but on same plane as mesoscutum, phragma of the scutellum widely visible; BS not overlapping the MPM; ATS demilune with a little complete, but faint parallel carinae; dorsal ATS groove with semicircular/parallel carinae. Transscutal articulation with small and heterogeneous foveae, area just behind transscutal articulation sloped, smooth and shiny. Metanotum with BM wider than PFM (clearly differentiated); MPM oval/circular with a short proximal carina; AFM without setiferous lobes and not as well delineated as PFM; PFM thick, smooth and distally fused with ATM; ATM proximally with a groove with some sculpturing and distally smooth. Propodeum rather coarse sculpture and without median longitudinal carina, proximal half curved; distal edge of propodeum with a flange at each side and without stubs; propodeal spiracle distally framed by a short concave carina; nucha surrounded by very short radiating carinae. Pronotum with a distinct dorsal furrow, dorsally with a well-defined smooth band; central area of pronotum smooth, but both dorsal and ventral furrows with short parallel carinae. Propleuron with a mix of rugae and fine punctation, dorsally with a carina. Metasternum flat or nearly so. Contour of mesopleuron straight/angulate or nearly so; precoxal groove smooth, shiny and shallow, but visible; epicnemial ridge convex, teardrop-shaped.

**Legs** (Fig. 216A). Ventral margin of fore telotarsus excavated with conspicuous curved seta over this excavation, fore telotarsus almost same width throughout and longer than fourth tarsomere (0.20, 0.09). Medially hind coxa smooth, dorsally with scattered punctation and ventrally with dense punctation, dorsal outer depression present. Inner spur of hind tibia longer than outer spur (0.32, 0.25), entire surface of hind tibia with dense strong spines clearly differentiated by color and length. Hind telotarsus as equal in length as fourth tarsomere (0.16, 0.15).

**Wings** (Fig. 216K, L). Fore wing with r vein straight; 2RS vein straight; r and 2RS veins forming a weak, even curve at their junction and outer side of junction forming a slight stub; 2M vein slightly curved/swollen; distally fore wing [where spectral veins are] with microtrichiae more densely concentrated than the rest of the wing; anal cell 1/3 proximally lacking microtrichiae; subbasal cell with a small smooth area; vein 2CUa absent and vein 2CUb spectral, vein 2 cu-a absent; vein 2-1A present only proximally as tubular vein; tubular vein 1 cu-a complete and junction with 1-1A vein spectral. Hind wing with vannal lobe narrow, subdistally and subproximally straightened, and setae evenly scattered in the margin.

**Metasoma** (Fig. 216A, H, J). Metasoma laterally compressed. Petiole on T1 finely sculptured only laterally, virtually parallel-sided over most of length, but narrowing over distal 1/3 (length 0.39, maximum width 0.21, minimum width 0.12), and with scarse pubescence concentrated in the first distal third. Lateral grooves delimiting the median area on T2 clearly defined and reaching the distal edge of T2 (length median area 0.20, length T2 0.20), edges of median area polished and lateral grooves deep, median area broader than long (length 0.20, maximum width 0.25, minimum width 0.09); T2 with scattered pubescence only distally. T3 longer than T2 (0.24, 0.20) and with scattered pubescence throughout.

**Cocoons** (Figs 4E, 216E). White oval cocoon with dark spots throughout. Silk fibers of cocoon are ordered, but covered by a net.

#### Comments.

The metasoma has only tree most proximal terga (T1–3), the remaining terga are missing (Fig. 216A), the propodeal spiracle distally are framed by a concave carina, the propodeum distally with a diagonal carina at each side (Fig. 216G), and the lateral margins of the median area on T2 are slightly curved (concave, Fig. 216H) resembling the median area on T2 of *G.bourquini* (Blanchard) and *G.ecuadorius* (Whitfield et al. 2002a).

#### Etymology.

Thomas Pape is a Danish dipterologist whose interests are systematics, taxonomy, phylogeny, biogeography, and evolution of the Calyptratae (Diptera). Currently, he is the head of Biosystematics at Zoological Museum, Natural History Museum of Denmark, and also the president of the International Commission on Zoological Nomenclature (ICZN).

#### Distribution.

Parasitized caterpillar was collected in Ecuador, Napo, Yanayacu Biological Station (Sendero Culo del Mundo), during May 2009 at 2,414 m in cloud forest.

#### Biology.

The lifestyle of this parasitoid species is solitary.

#### Host.

Undetermined species of Noctuidae feeding on *Munnoziapinnatipartita* (Asteraceae). Caterpillar was collected in third instar.

### Glyptapanteles toluagunbiadeae

Taxon classificationAnimaliaHymenopteraBraconidae

Arias-Penna, sp. nov.

http://zoobank.org/F23DA5C8-3DF7-4F56-A8C2-F40EE3BB6703

Fig. 217

#### Female.

Body length 2.17 mm, antenna length 2.63 mm, fore wing length 2.78 mm.

#### Type material.

**Holotype**: ECUADOR • 1♀; EC-14572, YY-A094; Napo, Yanayacu Biological Station, Km 33 vía Tena, Plot 222; -0.683333, -77.8; 13.v.2006; Grant Gentry leg.; caterpillar collected in late instar or pre-pupa; cocoons formed on 13.v.2006 and adhered to the larval cuticle; adult parasitoids emerged on 17.v.2006; (PUCE). **Paratypes.** • 9 (4♀, 3♂) (2♀, 0♂); EC-14572, YY-A094; same data as for holotype; (PUCE).

#### Diagnosis.

**Figure 217.**
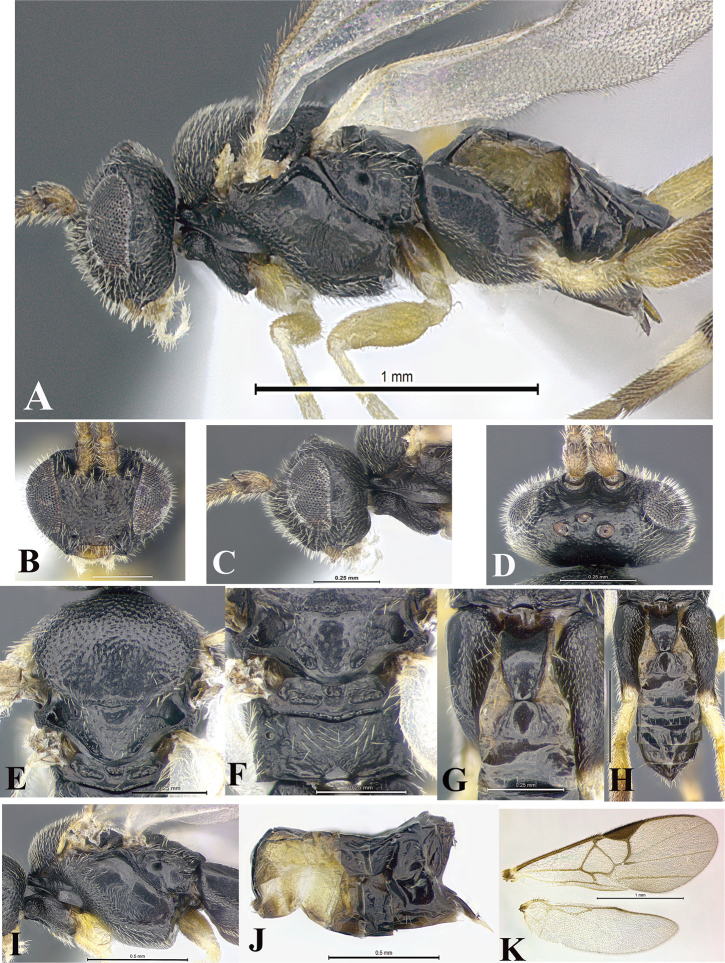
*Glyptapantelestoluagunbiadeae* sp. nov. female EC-14572 YY-A094 **A** Habitus **B, D** Head **B** Frontal view **D** Dorsal view **C** Head, propleuron, lateral view **E** Mesonotum, dorsal view **F** Scutellum, metanotum, propodeum, dorsal view **G**T1–3, dorsal view **H, J** Metasoma **H** Dorsal view **J** Lateral view **I** Mesosoma, lateral view **K** Fore and hind wings.

Precoxal groove with transverse lineate sculpture (Fig. 217A, I), scutellar punctation distinct peripherally, absent centrally (Fig. 217F), vertex in dorsal view quite wide, mesoscutum punctation distinct proximally but absent distally (Fig. 217E), T3 longer than T2 (Fig. 217H), propodeum with a median longitudinal dent, petiole on T1 finely sculptured (Fig. 217G, H), lateral grooves delimiting the median area on T2 distally losing definition on T2 (Fig. 217G), and fore wing with r vein straight, outer side of junction of r and 2RS veins forming a stub.

#### Coloration

(Fig. 217A–K). General body coloration polished black except scape proximally yellow-brown/reddish and distally brown; pedicel yellow-brown; first four-five proximal antennal flagellomeres dorsally lighter (light brown) than ventrally (dark brown), remaining flagellomeres dark brown on both sides; labrum, mandible, and tegulae light yellow-brown; glossa, maxillary and labial palps yellow. Eyes gray/black and ocelli reddish (in preserved specimen). Fore and middle legs dark yellow, except brown claws, telotarsus and penultimate tarsomeres of middle legs with brown tints; hind legs dark yellow except black coxae, distal 3/4 of femora brown, distal half of tibiae brown, and tarsomeres brown although basitarsus proximally with a yellow band. Petiole on T1 brown and sublateral areas yellow-brown; T2 with median area and lateral ends brown; T3 and beyond completely brown; distally each tergum with a very narrow whitish translucent band. In lateral view, T1–3 yellow-brown; T4 and beyond brown. S1–3 yellow, but medially brown; S4 and beyond completely brown.

#### Description.

**Head** (Fig. 217A–D). Head rectangle with pubescence long and dense. Proximal three antennal flagellomeres longer than wide (0.19:0.06, 0.19:0.06, 0.19:0.06), distal antennal flagellomere longer than penultimate (0.13:0.05, 0.08:0.05), antenna longer than body (2.63, 2.17); antennal scrobes-frons shallow. Face flat or nearly so, punctate-lacunose, interspaces wavy and longitudinal median carina present. Frons smooth. Temple quite wide, punctate-lacunose and interspaces wavy. Inner margin of eyes diverging slightly at antennal sockets; in lateral view, eye anteriorly convex and posteriorly straight. POL subequal in length with OOL (0.09, 0.10). Malar suture present. Median area between lateral ocelli slightly depressed. Vertex laterally rounded and dorsally quite wide.

**Mesosoma** (Fig. 217A, E, F, I). Mesosoma dorsoventrally convex. Distal 1/3 of mesoscutum with lateral margin slightly dented, punctation proximally distinct, but distally absent/dispersed, interspaces wavy/lacunose. Scutellum short and broad, apex sloped and fused with BS, but not in the same plane, scutellar punctation distinct peripherally and absent centrally, in profile scutellum flat and on same plane as mesoscutum, phragma of the scutellum partially exposed; BS mostly overlapping the MPM; ATS demilune with short stubs delineating the area; dorsal ATS groove with semicircular/parallel carinae. Transscutal articulation with small and homogeneous foveae, area just behind transscutal articulation with a smooth and shiny sloped transverse strip. Metanotum with BM convex; MPM circular and bisected by a median longitudinal carina; AFM without setiferous lobes and not as well delineated as PFM; PFM thick, smooth and with lateral ends rounded; ATM proximally with a groove with some sculpturing and distally smooth. Propodeum with a median longitudinal dent, but no trace of median longitudinal carina, proximal half straight or nearly so and with fine sculpture, and distal half with a mix of coarse sculpture and rugae; distal edge of propodeum with a flange at each side and without stubs; propodeal spiracle distally framed by a short concave carina; nucha surrounded by long radiating carinae. Pronotum with a distinct dorsal furrow, dorsally with a well-defined smooth band; central area of pronotum and dorsal furrow smooth, but ventral furrow with short parallel carinae. Propleuron with fine rugae and dorsally with a carina. Metasternum flat or nearly so. Contour of mesopleuron convex; precoxal groove deep with transverse lineate sculpture; epicnemial ridge convex, teardrop-shaped.

**Legs** (Fig. 217A). Ventral margin of fore telotarsus slightly excavated and with a tiny curved seta, fore telotarsus almost same width throughout and longer than fourth tarsomere (0.12, 0.09). Hind coxa with punctation only on ventral surface, dorsal outer depression present. Inner spur of hind tibia longer than outer spur (0.25, 0.15), entire surface of hind tibia with dense strong spines clearly differentiated by color and length. Hind telotarsus as equal in length as fourth tarsomere (0.11, 0.10).

**Wings** (Fig. 217K). Fore wing with r vein straight; 2RS vein straight; r and 2RS veins forming an angle at their junction and outer side of junction forming a slight stub; 2M vein slightly curved/swollen; distally fore wing [where spectral veins are] with microtrichiae more densely concentrated than the rest of the wing; anal cell 1/3 proximally lacking microtrichiae; subbasal cell with microtrichiae virtually throughout; vein 2CUa absent and vein 2CUb spectral; vein 2 cu-a absent; vein 2-1A present only proximally as spectral vein; tubular vein 1 cu-a straight, incomplete/broken and not reaching the edge of 1-1A vein. Hind wing with vannal lobe narrow, subdistally and subproximally straightened, and setae evenly scattered in the margin.

**Metasoma** (Fig. 217A, G, H, J). Metasoma laterally compressed. Petiole on T1 completely smooth and polished, with faint, satin-like sheen, parallel-sided in proximal half and then narrowing (length 0.31, maximum width 0.14, minimum width 0.08), and with scattered pubescence concentrated in the first distal third. Lateral grooves delimiting the median area on T2 clearly defined and reaching the distal edge of T2 (length median area 0.11, length T2 0.11), edges of median area polished and lateral grooves deep, median area broader than long (length 0.11, maximum width 0.13, minimum width 0.08); T2 with scattered pubescence only distally. T3 longer than T2 (0.18, 0.10) and with pubescence more notorious in distal half. Pubescence on hypopygium scattered.

**Cocoons.** Adhered to the larval cuticle, but characteristic unknown.

#### Comments.

In lateral view, the head is wide and looks globose (Fig. 217C) and the body is short and stout (Fig. 217A).

#### Male.

Coloration darker than female; the hind femora is completely brown.

#### Etymology.

Tolulope (Tolu) Adebimpe Agunbiade is a Nigerian-born entomologist who, as a graduate student at UIUC, IL, USA, studied the population genetics of the insects that attack cowpeas, also known as black-eyed peas. Currently, she is a lecturer at the University of Florida’s Entomology and Nematology Department in Gainesville, Florida, USA.

#### Distribution.

Parasitized caterpillar was collected in Ecuador, Napo, Yanayacu Biological Station (Km 33 vía Tena), during May 2006.

#### Biology.

The lifestyle of this parasitoid species is gregarious.

#### Host.

Undetermined species of Noctuidae feeding on *Miconia* sp. (Melastomataceae). Caterpillar was collected in late instar or pre-pupa.

### Glyptapanteles tomwallai

Taxon classificationAnimaliaHymenopteraBraconidae

Arias-Penna, sp. nov.

http://zoobank.org/14950B2B-14EF-4BF7-8711-55D5D0764CCE

Fig. 218

#### Female.

Body length 2.83 mm, antenna length 3.03 mm, fore wing length 3.13 mm.

#### Type material.

**Holotype**: ECUADOR • 1♀; EC-38743, YY-A007; Napo, Yanayacu Biological Station, Yanayacu Road; cloud forest; 2,100 m; -0.566667, -77.866667; 16.v.2009; CAPEA leg.; caterpillar collected in third instar; cocoons formed on 05.vi.2009; adult parasitoids emerged on 26.vi.2009; (PUCE). **Paratypes.** • 83 (9♀, 4♂) (70♀, 0♂); EC-38743, YY-A007; same data as for holotype; (PUCE).

#### Other material.

**Reared material.** ECUADOR: *Napo*, *Yanayacu Biological Station*, *Yanayacu Road*: • 18 (5♀, 4♂) (9♀, 0♂); EC-2734/2735, YY-A076; cloud forest; 2,100 m; -0.566667, -77.866667; 13.v.2005; Harold Greeney leg.; cocoons formed on 17.v.2005. • 62 (5♀, 5♂) (47♀, 5♂); EC-38747, YY-A103; same data as for preceding except: CAPEA leg.; caterpillar collected in third instar; cocoons formed on 15.vi.2009; cocoon characteristics not reported; adult parasitoids emerged on 01.vii.2009.

#### Diagnosis.

**Figure 218.**
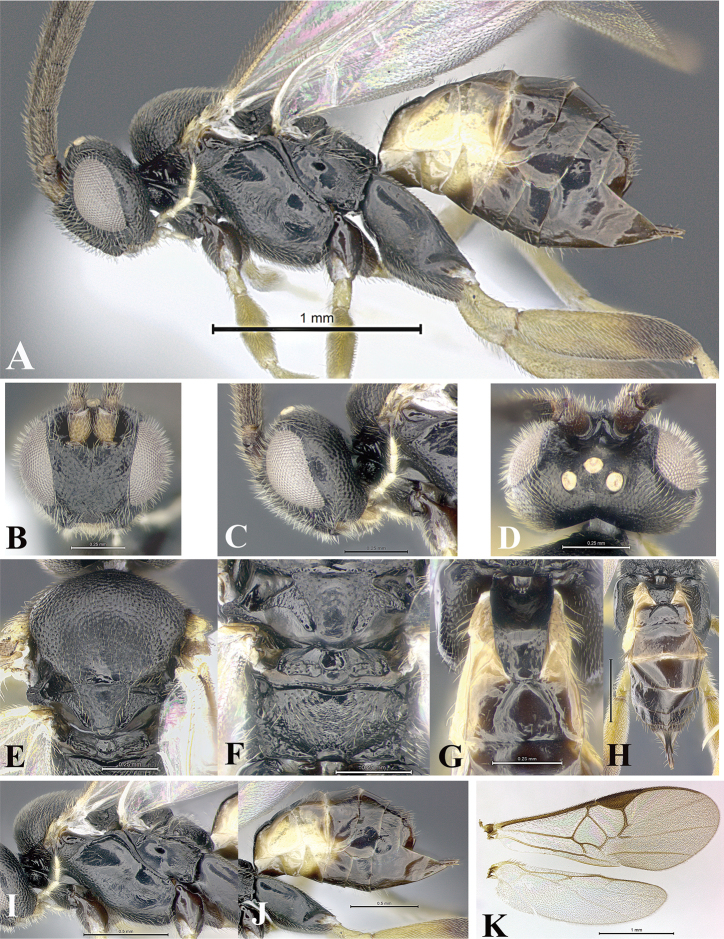
*Glyptapantelestomwallai* sp. nov. female EC-38743 YY-A007, EC-38747 YY-A103 **A** Habitus **B, D** Head **B** Frontal view **D** Dorsal view **C** Head, propleuron, lateral view **E** Mesonotum, dorsal view **F** Scutellum, metanotum, propodeum, dorsal view **G**T1–2, dorsal view **H, J** Metasoma **H** Dorsal view **J** Lateral view **I** Mesosoma, lateral view **K** Fore and hind wings.

Area just behind transscutal articulation with a sloped transverse strip (Fig. 218E), dorsal furrow of pronotum with a defined smooth band only proximally (Fig. 218A), entire surface of hind tibia with numerous strong spines, propodeal spiracle distally framed by faintly concave/wavy carina (Fig. 218F), phragma of the scutellum widely visible (Fig. 218F), nucha surrounded by long radiating carinae (Fig. 218F), propodeum without median longitudinal carina (Fig. 218F), dorsal carina delimiting a dorsal furrow on propleuron present (Fig. 218C), petiole on T1 parallel-sided, but narrowing over distal 1/3 (Fig. 218G), precoxal groove deep (Fig. 218A, I), anteroventral contour of mesopleuron straight/angulate or nearly so (Fig. 218A, I), edges of median area on T2 polished and followed by a deep groove (Fig. 218G), and fore wing with r vein curved, outer side of junction of r and 2RS veins forming a distinct stub (Fig. 218K).

#### Coloration

(Fig. 218A–K). General body coloration satin black except scape yellow-brown; pedicel distal half yellow-brown and proximal half brown; all antennal flagellomeres brown on both sides; labrum and mandible yellow-brown; tegulae light brown; glossa, maxillary and labial palps yellow. Eyes reddish (in preserved specimen) and ocelli silver. Fore and middle legs dark yellow except brown-red/reddish coxae and brown claws; hind legs dark yellow except black coxae, femora distally brown, tibiae 1/3 distal and tarsomeres brown although proximal half of basitarsus yellow. Petiole on T1 brown with some reddish tints, contours darkened and sublateral areas yellow; T2 with median and adjacent areas brown, adjacent area with contours well-defined, both dark areas forming a rectangle-shape area, narrow lateral ends yellow-brown; T3 mostly brown except lateral ends proximally with yellow corners; T4 and beyond brown; distally each tergum with a narrow yellowish translucent band. In lateral view, T1–2 yellow; T3 yellow, but dorsally with a small brown area; T4 and beyond brown. S1–2 yellow; S3 yellow, but medially brown; S4 and beyond brown.

#### Description.

**Head** (Fig. 218A–D). Head rounded with pubescence long and dense. Proximal three antennal flagellomeres longer than wide (0.20:0.08, 0.22:0.08, 0.23:0.08), distal antennal flagellomere longer than penultimate (0.15:0.06, 0.11:0.06), antenna longer than body (3.03, 2.83); antennal scrobes-frons sloped and forming a shelf. Face flat or nearly so, punctate-lacunose, interspaces wavy and longitudinal median carina present. Frons smooth. Temple wide, punctate-lacunose and interspaces wavy. Inner margin of eyes diverging slightly at antennal sockets; in lateral view, eye anteriorly convex and posteriorly straight. POL shorter than OOL (0.11, 0.15). Malar suture present. Median area between lateral ocelli slightly depressed. Vertex laterally pointed or nearly so and dorsally wide.

**Mesosoma** (Fig. 218A, E, F, I). Mesosoma dorsoventrally convex. Distal 1/3 of mesoscutum with lateral margin slightly dented, punctation proximally distinct, but distally absent/dispersed, interspaces wavy/lacunose. Scutellum triangular, apex sloped and fused with BS, but not in the same plane, scutellar punctation scattered throughout, in profile scutellum slightly convex, but on same plane as mesoscutum, phragma of the scutellum widely visible; BS only very partially overlapping the MPM; ATS demilune with short stubs delineating the area; dorsal ATS groove smooth. Transscutal articulation with small and heterogeneous foveae, area just behind transscutal articulation with a sloped transverse strip, smooth and shiny. Metanotum with BM convex; MPM circular without median longitudinal carina; AFM without setiferous lobes and not as well delineated as PFM; PFM thick, smooth and with lateral ends rounded; ATM proximally with a groove with some sculpturing and distally smooth. Propodeum with a mix of faint rugae and fine sculpture and without median longitudinal carina, proximal half curved; distal edge of propodeum with a flange at each side and without stubs; propodeal spiracle distally framed by faintly concave/wavy carina; nucha surrounded by very short radiating carinae. Pronotum with a distinct dorsal furrow, dorsally with a defined smooth band only proximally; central area of pronotum smooth, but both dorsal and ventral furrows with short parallel carinae. Propleuron with a mix of rugae and fine punctation, dorsally with a carina. Metasternum convex. Contour of mesopleuron straight/angulate or nearly so; precoxal groove deep with transverse lineate sculpture; epicnemial ridge widen.

**Legs** (Fig. 218A). Ventral margin of fore telotarsus entire without seta, fore telotarsus almost same width throughout and longer than fourth tarsomere (0.11, 0.08). Hind coxa with punctation only on ventral surface, dorsal outer depression present. Inner spur of hind tibia longer than outer spur (0.27, 0.20), entire surface of hind tibia with dense strong spines clearly differentiated by color and length. Hind telotarsus as equal in length as fourth tarsomere (0.13, 0.14).

**Wings** (Fig. 218K). Fore wing with r vein curved; 2RS vein straight; r and 2RS veins forming a weak, even curve at their junction and outer side of junction forming a slight stub; 2M vein slightly curved/swollen; distally fore wing [where spectral veins are] with microtrichiae more densely concentrated than the rest of the wing; anal cell 1/3 proximally lacking microtrichiae; subbasal cell with microtrichiae virtually throughout; vein 2CUa absent and vein 2CUb spectral; vein 2 cu-a absent; vein 2-1A proximally tubular and distally spectral, although sometimes difficult to see; tubular vein 1 cu-a curved and complete, but junction with 1-1A vein spectral. Hind wing with vannal lobe wide, subdistally and subproximally straightened, and setae present proximally, but absent distally.

**Metasoma** (Fig. 218A, G, H, J). Metasoma laterally compressed. Petiole on T1 finely sculptured distal, but only laterally, virtually parallel-sided over most of length, but narrowing over distal 1/3 (length 0.39, maximum width 0.20, minimum width 0.07), and with scattered pubescence concentrated in the first distal third. Lateral grooves delimiting the median area on T2 clearly defined and reaching the distal edge of T2 (length median area 0.17, length T2 0.17), edges of median area polished and lateral grooves deep, median area broader than long (length 0.17, maximum width 0.23, minimum width 0.09); T2 with scattered pubescence throughout. T3 longer than T2 (0.25, 0.17) and with pubescence more notorious in distal half. Pubescence on hypopygium scattered.

**Cocoons.** Unknown.

#### Comments.

In general, the female body is slender and cylindrical. In some females, the coloration on S1–2 is yellow, but the remaining sterna completely brown. In other females, only the three distal sterna (S4–6) are completely brown. The proximal edge of ATS demilune is carinate.

#### Male.

The male body is slender and cylindrical like the female. Male is similar in coloration except that the hind femora has two colorations: proximal 3/4 dark brown-red and distal 1/4 brown; the coloration on S1–3 is yellow-brown, but the remaining sterna are completely brown; the external genitalia is small.

#### Etymology.

Thomas (Tom) R. Walla is an American entomologist whose speciality is in tropical ecology, tropical butterflies, and patterns of species diversity. He is a professor at Mesa State College, Grand Junction, CO, USA.

#### Distribution.

Parasitized caterpillars were collected in Ecuador, Napo, Yanayacu Biological Station (Yanayacu Road), during May 2005 and May 2009 at 2,100 m in cloud forest.

#### Biology.

The lifestyle of this parasitoid species is gregarious.

#### Host.

Undetermined species of Apatelodidae feeding on *Dendrophorbiumlloense* (Asteraceae). Undetermined species of Erebidae (Arctiinae) feeding on *Baccharislatifolia* (Asteraceae). Caterpillars were collected in third instar.

### Glyptapanteles victoriapookae

Taxon classificationAnimaliaHymenopteraBraconidae

Arias-Penna, sp. nov.

http://zoobank.org/710AAB03-4B93-4121-89B4-8796C5AF990F

Fig. 219

#### Female.

Body length 2.42 mm, antenna length 2.28 mm, fore wing length 2.15 mm.

#### Type material.

**Holotype**: COSTA RICA • 1♀; 08-SRNP-57260, DHJPAR0031106; Área de Conservación Guanacaste, Guanacaste, Sector Mundo Nuevo, Vado Agria; dry-rain intergrade forest; 560 m; 10.75876, -85.37543; 03.viii.2008; José Cortez leg.; caterpillar collected in fourth instar; cocoons adhered to the leaf substrate and formed on 04.viii.2008; adult parasitoids emerged on 10.viii.2008; (CNC). **Paratypes.** • 10 (2♀, 3♂) (4♀, 1♂); 08-SRNP-57260, DHJPAR0031106; same data as for holotype; (CNC).

#### Other material.

**Reared material.** COSTA RICA: *Área de Conservación Guanacaste*, *Guanacaste*, *Sector Mundo Nuevo*, *Vado Huacas*: • 8 (2♀, 3♂) (1♀, 2♂); 08-SRNP-56887, DHJPAR0031100; dry-rain intergrade forest; 490 m; 10.75533, -85.39117; 22.vi.2008; Dinier Guadamuz leg.; caterpillar collected in fourth instar; very small brown cocoons forming two rows of cordwood, strong glue to leaf, cocoons formed on 26.vii.2008; adult parasitoids emerged on 31.vii.2008.

#### Diagnosis.

**Figure 219.**
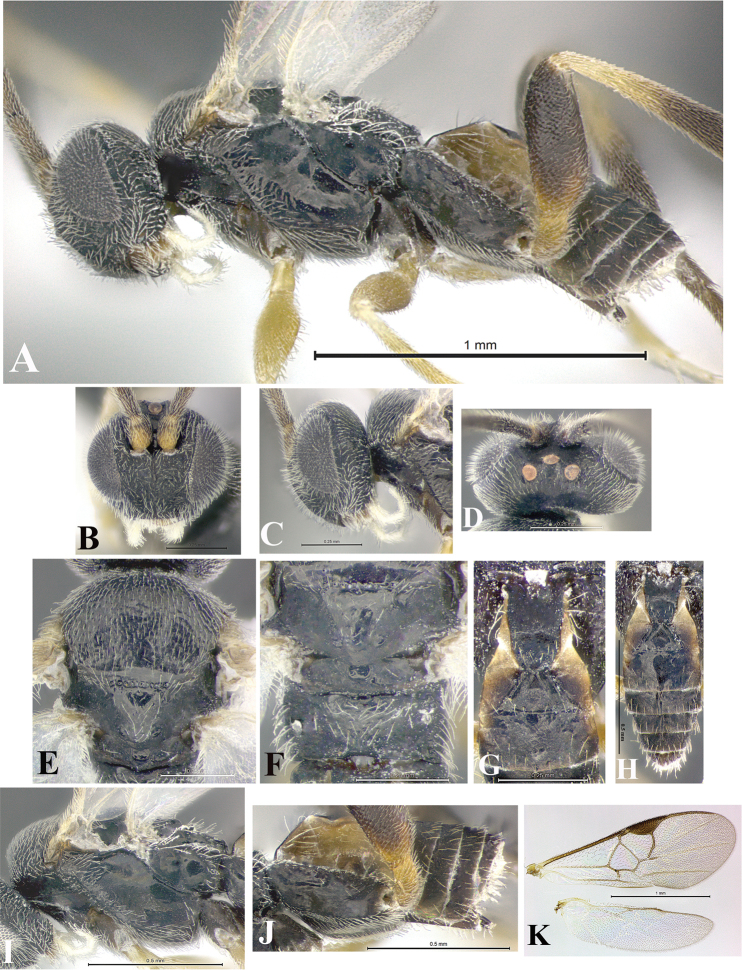
*Glyptapantelesvictoriapookae* sp. nov. female 08-SRNP-57260 DHJPAR0031106 **A** Habitus **B, D** Head **B** Frontal view **D** Dorsal view **C** Head, pronotum, propleuron, lateral view **E** Mesonotum, dorsal view **F** Scutellum, metanotum, propodeum, dorsal view **G**T1–3, dorsal view **H, J** Metasoma **H** Dorsal view **J** Lateral view **I** Mesosoma, lateral view **K** Fore and hind wings.

Vertex laterally pointed or nearly so (Fig. 219C), contour of mesopleuron angulate or nearly so (Fig. 219A, I), area just behind transscutal articulation with a sloped transverse strip (Fig. 219F), antenna shorter than body, distal antennal flagellomere longer than penultimate, longitudinal median carina on face present (Fig. 219B), surface of metasternum flat or nearly so, fore wing with r vein curved, outer side of junction of r and 2RS veins not forming a stub (Fig. 219K), petiole on T1 completely smooth and polished, with faint, satin-like sheen, evenly narrowing distally (Fig. 219G, H), propodeum without median longitudinal carina (Fig. 219F), and lateral grooves delimiting the median area on T2 clearly defined and reaching the distal edge of T2 (Fig. 219G, H).

#### Coloration

(Fig. 219A–K). General body coloration shiny black except scape and pedicel yellow both with inner side brown; all antennal flagellomeres dark brown on both sides; labrum, mandibles, and glossa yellow-brown; maxillary and labial palps pale yellow/ivory; tegulae yellow. Eyes silver and ocelli reddish (in preserved specimen). Fore and middle legs yellow except brown coxae and brown claws; hind legs dark brown/black except trochanter, trochantelli, femora proximally, distal 1/3 of tibiae and basitarsus proximally with a ring yellow/yellow-brown. Petiole on T1 black and sublateral areas yellow-brown; T2 with median and adjacent areas black, and lateral ends brown; T3 mostly black, lateral ends narrow with proximal corners yellow-brown; T4 and beyond completely brown; distally each tergum with a narrow whitish transparent band. In lateral view, T1–2 yellow-brown; T3 yellow-brown, but corners distally brown; T4 and beyond brown. S1–3 yellow-brown; S4 and beyond brown.

#### Description.

**Head** (Fig. 219A–D). Head rounded with pubescence long and dense. Proximal three antennal flagellomeres longer than wide (0.17:0.07, 0.18:0.07, 0.16:0.07), distal antennal flagellomere subequal in length with penultimate (0.09:0.05, 0.09:0.05), antenna shorter than body (2.28, 2.42); antennal scrobes-frons sloped and forming a shelf. Face distal half dented laterally, punctate-lacunose, interspaces wavy and longitudinal median carina present. Frons smooth. Temple wide, punctate-lacunose and interspaces wavy. Inner margin of eyes diverging slightly at antennal sockets; in lateral view, eye anteriorly convex and posteriorly straight. POL shorter than OOL (0.09, 0.11). Malar suture present. Median area between lateral ocelli slightly depressed. Vertex laterally pointed or nearly so and dorsally wide.

**Mesosoma** (Fig. 219A, E, F, I). Mesosoma dorsoventrally convex. Mesoscutum proximally convex and distally flat, punctation distinct throughout, interspaces wavy/lacunose. Scutellum triangular, apex sloped and fused with BS Scutellar punctation scattered throughout, in profile scutellum flat and on same plane as mesoscutum, but not in the same plane, phragma of the scutellum partially exposed; BS only very partially overlapping the MPM; ATS demilune only inner side with sculpture; dorsal ATS groove with semicircular/parallel carinae. Transscutal articulation with small and heterogeneous foveae, area just behind transscutal articulation with a smooth and shiny sloped transverse strip. Metanotum with BM wider than PFM (clearly differentiated); MPM semicircular without median longitudinal carina; AFM without setiferous lobes and not as well delineated as PFM; PFM thick, smooth and with lateral ends rounded; ATM proximally with a groove with some sculpturing and distally smooth. Propodeum without median longitudinal carina, proximal half weakly curved with fine sculpture and distal half relatively polished; distal edge of propodeum with a flange at each side and without stubs; propodeal spiracle without distal carina; nucha surrounded by very short radiating carinae. Pronotum with a distinct dorsal furrow, dorsally with a well-defined smooth band; central area of pronotum and dorsal furrow smooth, but ventral furrow with short parallel carinae. Propleuron with fine punctations throughout and dorsally without a carina. Metasternum flat or nearly so. Contour of mesopleuron straight/angulate or nearly so; precoxal groove deep, smooth and shiny; epicnemial ridge convex, teardrop-shape.

**Legs** (Fig. 219A). Ventral margin of fore telotarsus entire without seta, fore telotarsus proximally narrow and distally wide, and longer than fourth tarsomere (0.13, 0.06). Hind coxa with punctation only on ventral surface, dorsal outer depression present. Inner spur of hind tibia longer than outer spur (0.18, 0.13), entire surface of hind tibia with dense strong spines clearly differentiated by color and length. Hind telotarsus as equal in length as fourth tarsomere (0.10, 0.10).

**Wings** (Fig. 219K). Fore wing with r vein slightly curved; 2RS vein straight; r and 2RS veins forming a weak, even curve at their junction and outer side of junction not forming a stub; 2M vein slightly curved/swollen; distally fore wing [where spectral veins are] with microtrichiae more densely concentrated than the rest of the wing; anal cell 1/3 proximally lacking microtrichiae; subbasal cell with a small smooth area; vein 2CUa absent and vein 2CUb spectral; vein 2 cu-a absent; vein 2-1A present only proximally as tubular vein; tubular vein 1 cu-a curved, incomplete/broken and not reaching the edge of 1-1A vein. Hind wing with vannal lobe narrow, subdistally evenly convex and subproximally straightened, and setae evenly scattered in the margin.

**Metasoma** (Fig. 219A, G, H, J). Metasoma laterally compressed. Petiole on T1 completely smooth and polished, with faint, satin-like sheen, virtually parallel-sided over most of length, but narrowing over distal 1/3 (length 0.25, maximum width 0.15, minimum width 0.06), and with scattered pubescence concentrated in the first distal third. Lateral grooves delimiting the median area on T2 clearly defined and reaching the distal edge of T2 (length median area 0.12, length T2 0.12), edges of median area polished and lateral grooves deep, median area broader than long (length 0.12, maximum width 0.18, minimum width 0.05), T2 with scattered pubescence only distally. T3 longer than T2 (0.17, 0.12) and with scattered pubescence throughout. Pubescence on hypopygium dense.

**Cocoons.** Light brown oval cocoons with evenly smooth silk fibers. Two rows of cordwood cocoons strongly adhered to the leaf substrate.

#### Comments.

In some specimens, the body coloration is light brown; all the sterna are light brown except that a dorsal area from S1 to S4 is yellow; the middle femora is yellow-brown; the body is slim and elongated.

#### Male.

Coloration similar to females; however, there are some variations: body coloration is darker than female, the middle femora is brown, the sublateral areas on T1–2 are dark reddish/dark yellow-brown. In some males, the coloration on the petiole is different: completely yellow-brown/reddish with darkened contours; in others, even in the same sample, the proximal half of the petiole is light brown and the distal half is dark brown, and the petiole has darkened contours.

#### Etymology.

Victoria G. Pook as a graduate student at the University of Kentucky, Lexington, KY, USA, was interested in systematics and venom composition in Ichneumonidae (*Megarhyssa*: Rhyssinae).

#### Distribution.

The parasitized caterpillars were collected in Costa Rica, ACG, Sector Mundo Nuevo (Vado Agria and Vado Huacas), during June and August 2008 at 490 m and 560 m in dry-rain intergrade forest.

#### Biology.

The lifestyle of this parasitoid species is gregarious.

#### Host.

*Paecteslunodes* Guenée (Euteliidae: Euteliinae) feeding on *Ocoteaveraguensis* (Lauraceae). Caterpillars were collected in fourth instar.

### Glyptapanteles wilmersimbanai

Taxon classificationAnimaliaHymenopteraBraconidae

Arias-Penna, sp. nov.

http://zoobank.org/0ACEE544-D97F-4E2F-A4DE-BCB2D36B244A

Fig. 220

#### Female.

Body length 2.73 mm, antenna length 3.03 mm, fore wing length3.13 mm.

#### Type material.

**Holotype**: ECUADOR • 1♀; EC-38749, YY-A098; Napo, Yanayacu Biological Station, Yanayacu Road; cloud forest; 2,100 m; -0.566667, -77.866667; 16.v.2009; CAPEA leg.; caterpillar collected in third instar; cocoons formed on 05.vi.2009; adult parasitoids emerged on 20.vi.2009; (PUCE). **Paratypes.** • 63 (4♀, 5♂) (45♀, 9♂); EC-38749, YY-A098; same data as for holotype; (PUCE).

#### Diagnosis.

**Figure 220.**
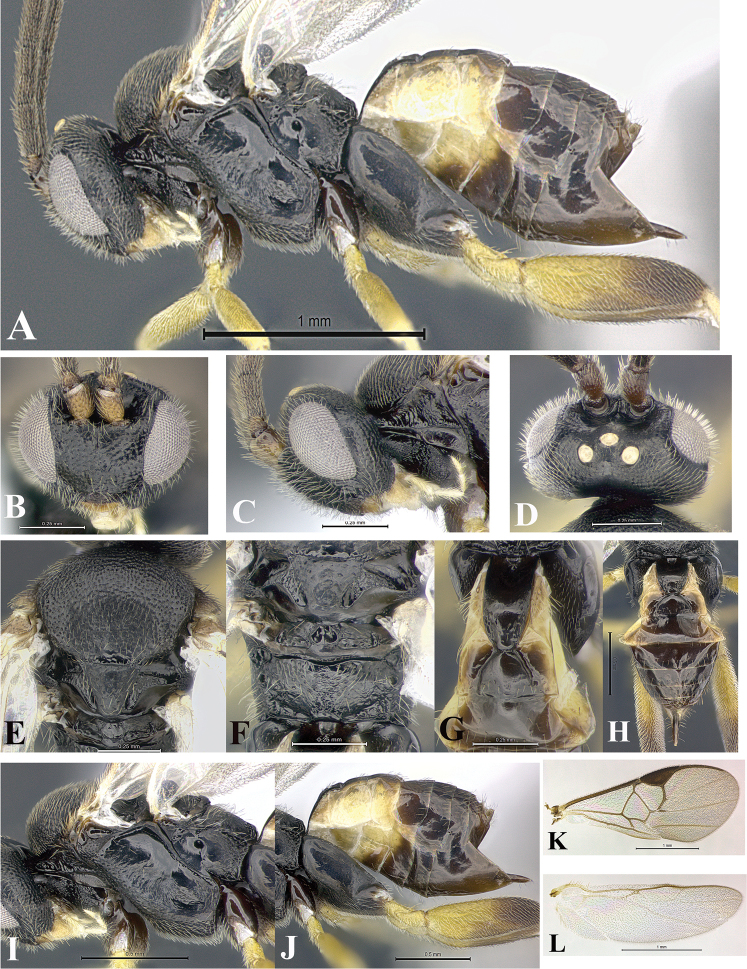
*Glyptapanteleswilmersimbanai* sp. nov. female EC-38749 YY-A098 **A** Habitus **B, D** Head **B** Frontal view **D** Dorsal view **C** Head, propleuron, lateral view **E** Mesonotum, dorsal view **F** Scutellum, metanotum, propodeum, dorsal view **G**T1–3, dorsal view **H, J** Metasoma **H** Dorsal view **J** Lateral view **I** Mesosoma, lateral view **K, L** Wings **K** Fore **L** Hind.

Area just behind transscutal articulation nearly at the same level as mesoscutum (flat, Fig. 220E), dorsal furrow of pronotum with a well-defined smooth band throughout (Fig. 220C), surface of hind tibia with strong spines only on distal half, propodeal spiracle distally framed by faintly concave/wavy carina (Fig. 220F), phragma of the scutellum widely visible (Fig. 220F), nucha surrounded by long radiating carinae (Fig. 220F), propodeum without median longitudinal carina (Fig. 220F), dorsal carina delimiting a dorsal furrow on propleuron present (Fig. 220C, I), petiole on T1 parallel-sided, but narrowing over distal 1/3 (Fig. 220G), precoxal groove deep (Fig. 220A, I), anteroventral contour of mesopleuron straight/angulate or nearly so (Fig. 220A, I), edges of median area on T2 polished and followed by a deep groove (Fig. 218G), and fore wing with r vein curved, outer side of junction of r and 2RS veins forming a distinct stub (Fig. 220K).

#### Coloration

(Fig. 220A–L). General body coloration satin black except scape yellow-brown, although distally brown; pedicel distal half yellow-brown and proximal half brown; all antennal flagellomeres brown on both sides; labrum and mandible yellow-brown; tegulae light brown; glossa, maxillary and labial palps yellow; both dorsal and ventral furrows of pronotum, epicnemial ridge, lunules, lateral ends of metanotum, and PFM with some brown-red tints. Eyes reddish (in preserved specimen) and ocelli silver. Fore and middle legs dark yellow except brown-red/reddish coxae and brown claws; hind legs dark yellow except black coxae, femora 1/3 distal brown, distal 1/3 of tibiae and tarsomeres brown, although basitarsus proximal half yellow. Petiole on T1 brown-red/reddish, contours darkened and sublateral areas yellow-brown; T2 with median and adjacent areas brown-red/reddish, these two dark areas forming a rectangle-shaped area, and lateral ends yellow-brown; T3 mostly light brown and lateral ends yellow-brown; T4 and beyond completely brown; distally each tergum with a very narrow yellowish translucent band. In lateral view, T1–3 completely yellow; T4 yellow, but dorsally brown; T5 and beyond brown. S1–2 yellow; S3–4 yellow, but medially brown, extent of brown area larger on S4 than S3; penultimate sternum and hypopygium completely brown.

#### Description.

**Head** (Fig. 220A–D). Head rounded with pubescence long and dense. Proximal three antennal flagellomeres longer than wide (0.21:0.07, 0.22:0.07, 0.23:0.07), distal antennal flagellomere longer than penultimate (0.15:0.07, 0.12:0.07), antenna longer than body (3.03, 2.73); antennal scrobes-frons sloped and forming a shelf. Face flat or nearly so, punctations barely noticeable, interspaces smooth and longitudinal median carina present. Frons smooth. Temple wide, punctate-lacunose and interspaces wavy. Inner margin of eyes diverging slightly at antennal sockets; in lateral view, eye anteriorly convex and posteriorly straight. POL shorter than OOL (0.10, 0.13). Malar suture present. Median area between lateral ocelli slightly depressed. Vertex laterally pointed or nearly so and dorsally wide.

**Mesosoma** (Fig. 220A, E, F, I). Mesosoma dorsoventrally convex. Mesoscutum 1/4 distal with a central dent, punctation distinct proximally, but absent/dispersed distally, interspaces wavy/lacunose. Scutellum triangular, apex sloped and fused with BS, but not in the same plane, scutellar punctation scattered throughout, in profile scutellum slightly convex, but on same plane as mesoscutum, phragma of the scutellum widely visible; BS only very partially overlapping the MPM; ATS demilune with faint wavy rugae; dorsal ATS groove smooth. Transscutal articulation with small and heterogeneous foveae, area just behind transscutal articulation nearly at the same level as mesoscutum (flat) and with same kind of sculpture as mesoscutum. Metanotum with BM convex; MPM circular without median longitudinal carina; AFM without setiferous lobes and not as well delineated as PFM; PFM thick, smooth and with lateral ends rounded; ATM proximally with a groove with some sculpturing and distally smooth. Propodeum without median longitudinal carina, proximal half straight or nearly so with fine sculpture and distal half with faint rugae; distal edge of propodeum with a flange at each side and without stubs; propodeal spiracle distally framed by faintly concave/wavy carina; nucha surrounded by very short radiating carinae. Pronotum with a distinct dorsal furrow, dorsally with a well-defined smooth band; central area of pronotum smooth, but both dorsal and ventral furrows with short parallel carinae. Propleuron with fine rugae and dorsally with a carina. Metasternum convex. Contour of mesopleuron straight/angulate or nearly so; precoxal groove deep with faintly transverse lineate sculpture; epicnemial ridge widen.

**Legs** (Fig. 220A, J). Ventral margin of fore telotarsus entire without seta, fore telotarsus almost same width throughout and longer than fourth tarsomere (0.11, 0.07). Hind coxa with punctation only on ventral surface, dorsal outer depression present. Inner spur of hind tibia longer than outer spur (0.29, 0.20), hind tibia with strong spines only on distal half.

**Wings** (Fig. 220K, L). Fore wing with 2RS vein straight; r and 2RS veins forming a weak, even curve at their junction and outer side of junction forming a slight stub; 2M vein slightly curved/swollen; distally fore wing [where spectral veins are] with microtrichiae more densely concentrated than the rest of the wing; anal cell 1/3 proximally lacking microtrichiae; subbasal cell with microtrichiae virtually throughout; vein 2CUa absent and vein 2CUb spectral; vein 2 cu-a absent, vein 2-1A proximally tubular and distally spectral, although sometimes difficult to see; tubular vein 1 cu-a curved, incomplete/broken and not reaching the edge of 1-1A vein. Hind wing with vannal lobe narrow, subdistally and subproximally straightened, and setae present proximally, but absent distally.

**Metasoma** (Fig. 220A, G, H, J). Metasoma laterally compressed. Petiole on T1 distal half with faint rugae only laterally, virtually parallel-sided over most of length, but narrowing over distal 1/3 (length 0.38, maximum width 0.17, minimum width 0.08), and with scattered pubescence concentrated in the first distal third. Lateral grooves delimiting the median area on T2 clearly defined and reaching the distal edge of T2 (length median area 0.17, length T2 0.17), edges of median area polished and lateral grooves deep, median area broader than long (length 0.17, maximum width 0.23, minimum width 0.08); T2 with pubescence in distal half. T3 longer than T2 (0.22, 0.17) and with scattered pubescence throughout. Pubescence on hypopygium scattered.

**Cocoon.** Unknown.

#### Comments.

The median area on T2 with the lateral margins curved (convex, Fig. 220G). In some females, the black body coloration is very intense which makes the reddish tints imperceptible at first sight and the ventral furrow of the pronotum has faint parallel rugae.

#### Male.

Coloration similar to female. However, the coloration on hind legs differ a little: trochanter and trochantellus are yellow, but with brown tints, the femora almost completely brown, but proximally is yellow, and distal half of tibiae is brown; and the external genitalia is large and gradually narrows towards the apex.

#### Etymology.

Wilmer Rosendo Simbaña is an Ecuadorian gusanero who has assisted with caterpillar rearing at Yanayacu Biological Station, Ecuador.

#### Distribution.

Parasitized caterpillar was collected in Ecuador, Napo, Yanayacu Biological Station (Yanayacu Road), during May 2009 at 2,100 m in cloud forest.

#### Biology.

The lifestyle of this parasitoid species is gregarious.

#### Host.

Undetermined species of Apatelodidae feeding on *Dendrophorbiumlloense* (Asteraceae). Caterpillar was collected in third instar.

### Glyptapanteles wonyoungchoi

Taxon classificationAnimaliaHymenopteraBraconidae

Arias-Penna, sp. nov.

http://zoobank.org/7915A631-8155-4530-A9C5-8CE9691408FE

Fig. 221

#### Female.

Body length 1.91 mm, antenna length 2.27 mm, fore wing length 2.07 mm.

#### Type material.

**Holotype** COSTA RICA • 1♀; 08-SRNP-32128, DHJPAR0031023; Área de Conservación Guanacaste, Guanacaste, Sector Pitilla, Colocho; rain forest; 375 m; 11.02367, -85.41884; 25.viii.2008; Calixto Moraga leg.; caterpillar collected in fifth instar; cocoons adhered to the leaf substrate and formed on 02.ix.2008; adult parasitoids emerged on 05.ix.2008; (CNC). **Paratypes.** • 7 (1♀, 1♂) (5♀, 0♂); 08-SRNP-32128, DHJPAR0031023; same data as for holotype; (CNC).

#### Other material.

**Reared material**. COSTA RICA: *Área de Conservación Guanacaste*, *Alajuela*, *Sector Rincón Rain Forest*, *Estación llanura*: • 7 (3♀, 1♂) (3♀, 0♂); 09-SRNP-44894, DHJPAR0039971; rain forest; 135 m; 10.93332, -85.25331; 04.vii.2009; Mercedes Moraga leg.; caterpillar collected in fifth instar; cocoons adhered to the leaf substrate and formed on 07.vii.2009; adult parasitoids emerged on 14.viii.2009. • 15 (4♀, 4♂) (0♀, 4♂); 11-SRNP-75522, DHJPAR0045124; same data as for preceding except: 31.vii.2011; Duvalier Briceño leg.; caterpillar collected in fourth instar; cocoons formed on 05.viii.2011; adult parasitoids emerged on 15.viii.2011.

*Área de Conservación Guanacaste*, *Alajuela*, *Sector Rincón Rain Forest*, *Jacobo*: • 19 (5♀, 2♂) (12♀, 0♂); 10-SRNP-81702, DHJPAR0041651; rain forest; 461 m; 10.94076, -85.3177; 26.xii.2010; Edwin Apu leg.; caterpillar collected in fourth instar; two rows of cordwood cocoons adhered to the leaf substrate and formed on 28.xii.2010; adult parasitoids emerged on 07.i.2011.

#### Diagnosis.

**Figure 221.**
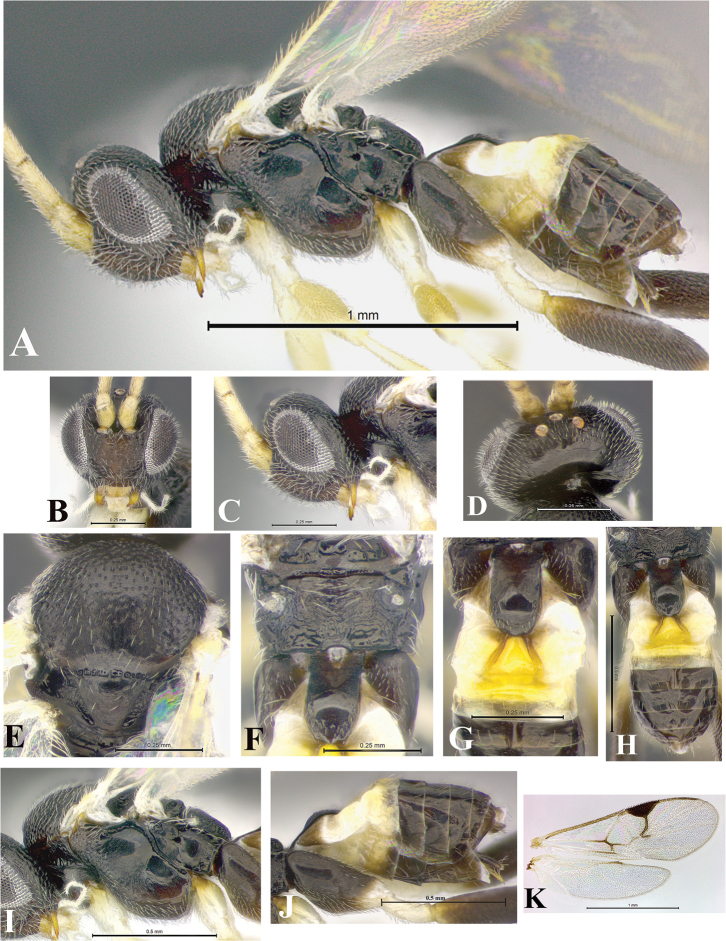
*Glyptapanteleswonyoungchoi* sp. nov. female 08-SRNP-32128 DHJPAR0031023 **A** Habitus **B, D** Head **B** Frontal view **D** Dorsal view **C** Head, pronotum, propleuron, lateral view **E** Mesonotum, dorsal view **F** Metanotum, propodeum, T1, dorsal view **G**T1–2, dorsal view **H, J** Metasoma **H** Dorsal view **J** Lateral view **I** Mesosoma, lateral view **K** Fore and hind wings, male 11-SRNP-75522 DHJPAR0045124.

Ventral margin of fore telotarsus apex excavated, but without seta, mesoscutum punctation distinct proximally ranging to satiny distally (Fig. 221E), fore wing with vein 2-1A absent, vein 1 cu-a curved, r vein curved, outer side of junction of r and 2RS veins not forming a stub (Fig. 221K), fore telotarsus almost same width throughout, medioposterior band of scutellum only very partially overlapping the medioanterior pit of metanotum (Fig. 221E), petiole on T1 distally with lateral margins curved (convex), finely sculptured on distal half (Fig. 221F, G, H), surface of metasternum flat or nearly so, precoxal groove deep with lineate sculpture (Fig. 221A, I), dorsal outer depression on hind coxa present (Fig. 221A, J), inner margin of eyes diverging slightly at antennal sockets (Fig. 221B), propodeum without median longitudinal carina (Fig. 221F), and lateral grooves delimiting the median area on T2 clearly defined and reaching the distal edge of T2 (Fig. 221G, H).

#### Coloration

(Fig. 221A–K). General body coloration black except scape and pedicel yellow; antenna tricolored: first four proximal antennal flagellomeres completely yellow, following five-seven flagellomeres totally yellow-brown and remaining flagellomeres brown on both sides; labrum and mandible yellow; glossa, maxillary and labial palps, and tegulae pale yellow/ivory. Eyes gray/silver and ocelli reddish (in preserved specimen); entire middle part of face, dorsal furrow of pronotum, epicnemial ridge, mesopleuron ventrally, and distal corners of mesoscutum with yellow-brown/reddish tints. Fore and middle legs yellow, except brown claws; hind legs dark brown/black except coxae distally, trochanter, trochanteli, tibial spurs, distal 1/3 of tibiae, and proximal 1/3 of basitarsus yellow. Petiole on T1 brown, but proximal 1/4 yellow-brown/reddish, contours darkened and sublateral areas with two colorations: proximal half light brown and distal half ivory/pale yellow-brown; T2 with median area and lateral ends completely yellow, although contour of median area dark light brown; T3 yellow; T4 and beyond brown; distally each tergum with a narrow yellowish translucent band. In lateral view, T1 yellow-brown; T2–3 yellow; T4 and beyond brown. S1–3 yellow; S4 proximal half yellow, distal half brown; penultimate sternum and hypopygium brown.

#### Description.

**Head** (Fig. 221A–D). Head rounded with pubescence long and dense. Proximal three antennal flagellomeres longer than wide (0.16:0.06, 0.17:0.06, 0.16:0.06), distal antennal flagellomere longer than penultimate (0.11:0.03, 0.09:0.03), antenna longer than body (2.27, 1.91); antennal scrobes-frons shallow. Face flat or nearly so, dense fine punctations, interspaces smooth and longitudinal median carina present. Frons smooth. Temple wide, punctate-lacunose and interspaces wavy. Inner margin of eyes diverging slightly at antennal sockets; in lateral view, eye anteriorly convex and posteriorly straight. POL shorter than OOL (0.08, 012). Malar suture present. Median area between lateral ocelli slightly depressed. Vertex laterally rounded and dorsally wide.

**Mesosoma** (Fig. 221A, E, F, I). Mesosoma dorsoventrally convex. Distal 1/3 of mesoscutum with lateral margin slightly dented, punctation distinct proximally ranging to satiny distally, interspaces with microsculpture. Scutellum triangular, apex sloped and fused with BS, but not in the same plane, scutellar punctation indistinct throughout, in profile scutellum flat and on same plane as mesoscutum, phragma of the scutellum partially exposed; BS only very partially overlapping the MPM; ATS demilune with a little, complete parallel carinae; dorsal ATS groove with carinae only proximally. Transscutal articulation with small and heterogeneous foveae, area just behind transscutal articulation with a smooth and shiny sloped transverse strip. Metanotum with BM wider than PFM (clearly differentiated); MPM circular without median longitudinal carina; AFM without setiferous lobes and not as well delineated as PFM; PFM thick, smooth and with lateral ends rounded; ATM proximally with a well-defined row of foveae and distally smooth. Propodeum without median longitudinal carina, proximal half weakly curved with fine sculpture and distal half rugose; distal edge of propodeum with a flange at each side and without stubs; propodeal spiracle distally framed by a short concave carina; nucha surrounded by very short radiating carinae. Pronotum with a distinct dorsal furrow, dorsally with a well-defined smooth band; central area of pronotum smooth, but both dorsal and ventral furrows with short parallel carinae. Propleuron with a mix of rugae and fine punctation, dorsally with a carina. Metasternum flat or nearly so. Contour of mesopleuron convex; precoxal groove deep with transverse lineate sculpture; epicnemial ridge convex, teardrop-shaped.

**Legs** (Fig. 221A). Ventral margin of fore telotarsus apex excavated, but without seta, fore telotarsus almost same width throughout and longer than fourth tarsomere (0.10, 0.06). Hind coxa with punctation only on ventral surface, dorsal outer depression present. Inner spur of hind tibia longer than outer spur (0.16, 0.11), entire surface of hind tibia with dense strong spines clearly differentiated by color and length. Hind telotarsus as equal in length as fourth tarsomere (0.10, 0.09).

**Wings** (Fig. 221K). Fore wing with r vein curved; 2RS vein straight; r and 2RS veins forming a weak, even curve at their junction and outer side of junction not forming a stub; 2M vein straight; distally fore wing [where spectral veins are] with microtrichiae more densely concentrated than the rest of the wing; anal cell 1/3 proximally lacking microtrichiae; subbasal cell with a small smooth area; vein 2CUa absent and vein 2CUb spectral; vein 2 cu-a absent; vein 2-1A absent; tubular vein 1 cu-a curved and complete, but junction with 1-1A vein spectral. Hind wing with vannal lobe narrow, subdistally and subproximally straightened, and setae evenly scattered in the margin.

**Metasoma** (Fig. 221A, G, H, J). Metasoma laterally compressed. Petiole on T1 finely sculptured on distal half, virtually parallel-sided over most of length, but narrowing over distal 1/3 (length 0.27, maximum width 0.12, minimum width 0.09), and with scattered pubescence concentrated in the first distal third. Lateral grooves delimiting the median area on T2 clearly defined and reaching the distal edge of T2 (length median area 0.11, length T2 0.11), edges of median area polished and lateral grooves deep, median area broader than long (length 0.11, maximum width 0.19, minimum width 0.05), T2 with scattered pubescence only distally. T3 longer than T2 (0.15, 0.11) and with pubescence more notorious in distal half. Pubescence on hypopygium dense.

**Cocoons.** Oval cocoons with ordered silk fibers, covered by a net. Two rows of cordwood cocoons adhered to the leaf substrate.

#### Comments.

In some specimens their bodies are nearly colorless; however, the lighter areas present in so called ‘normal specimens’ can also be distinguished in these albinos; the petiole distally looks convex; the lateral grooves delimiting the median area on T2 are almost reaching the distal edge of T2; however, the brown coloration is not throughout the edge (Fig. 221G, H). Some females are darker than others and the yellow coloration on T2–3 is replaced by yellow-brown; and the body is slim and elongated.

#### Male.

Similar in coloration to females except than in males, indeed in the same gregarious sample, the petiole is completely brown, the median area on T2 is brown with lateral ends yellow-brown, and the T3 is light brown and lateral ends with some yellow-brown spot.

#### Etymology.

Won-Young Choi was a Korean entomologist, who worked at the National Institute of Biological Resources (NIBR), Incheon, Korea. As a graduate student at UIUC, IL, USA, he contributed especially to the knowledge of taxonomy of *Diolcogaster* (Microgastrinae) from ACG.

#### Distribution.

The parasitized caterpillars were collected in Costa Rica, ACG, Sector Rincón Rain Forest (Estación llanura and Jacobo) and Sector Pitilla (Colocho), during August 2008, July 2009, December 2010, and July 2011 at 135 m, 375 m, and 461 m in rain forest.

#### Biology.

The lifestyle of this parasitoid species is gregarious.

#### Host.

*Antiblemmaceras* Druce (Erebidae: Eulepidotinae) feeding on *Conostegiaxalapensis* (Melastomataceae). Caterpillars were collected in fourth and fifth instar.

### Glyptapanteles yalizhangae

Taxon classificationAnimaliaHymenopteraBraconidae

Arias-Penna, sp. nov.

http://zoobank.org/F4088FA8-1FC4-4816-9EA5-88DB858C85DF

Fig. 222

#### Female.

Body length 3.13 mm, antenna length 3.43 mm, fore wing length 3.38 mm.

#### Type material.

**Holotype**: ECUADOR • 1♀; EC-38911, YY-A001; Napo, Yanayacu Biological Station, Sendero de las Lágrimas; cloud forest; 2,075 m; -0.598333, -77.882778; 25.v.2009; CAPEA leg.; caterpillar collected in third instar; cocoons formed on 15.vii.2009; adult parasitoids emerged on 03.viii.2009; (PUCE). **Paratypes.** • 49 (10♀, 5♂) (34♀, 0♂); EC-38911, YY-A001; same data as for holotype; (PUCE).

#### Other material.

**Reared material.** ECUADOR: *Napo*, *Yanayacu Biological Station*, *Sendero de los Sapos*, *Plot 441*: • 46 (9♀, 5♂) (31♀, 0♂); EC-41813, YY-A002; cloud forest; 2,004 m; -0.553333, -77.875556; 22.viii.2009; Luis Salagaje leg.; caterpillar collected in fourth instar; cocoons formed on 16.ix.2009; adult parasitoids emerged on 08.x.2009.

#### Diagnosis.

**Figure 222.**
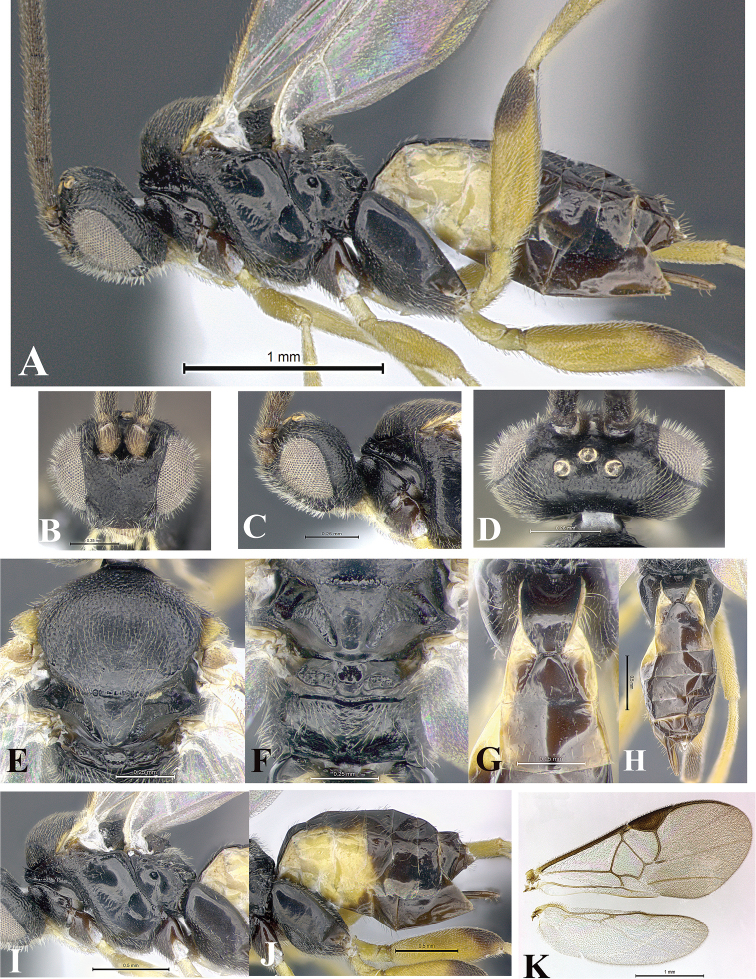
*Glyptapantelesyalizhangae* sp. nov. female EC-38911 YY-A001 **A** Habitus **B, D** Head **B** Frontal view **D** Dorsal view **C** Head, propleuron, lateral view **E** Mesonotum, dorsal view **F** Scutellum, metanotum, propodeum, dorsal view **G**T1–3, dorsal view **H, J** Metasoma **H** Dorsal view **J** Lateral view **I** Mesosoma, lateral view **K** Fore and hind wings.

Fore wing with vein 2 cu-a absent, vein 1 cu-a straight, r vein curved, outer side of junction of r and 2RS veins not forming a stub (Fig. 222K), dorsal groove on axillary trough of scutellum smooth (Fig. 222E, F), propodeum with a shallow median longitudinal dent with rugae (Fig. 222F), mesoscutum punctation proximally distinct, but distally absent/dispersed (Fig. 222E), temple punctate-lacunose, petiole virtually parallel-sided over most of length, but narrowing over distal 1/3, finely sculptured (Fig. 222G, H), dorsal outer depression on hind coxa present (Fig. 222A, J), inner margin of eyes diverging slightly at antennal sockets (Fig. 222B), and lateral grooves delimiting the median area on T2 clearly defined and reaching the distal edge of T2 (Fig. 222G, H).

#### Coloration

(Fig. 222A–K). General body coloration polished satin black except labrum, mandibles, glossa, and tegulae yellow-brown; yellow-brown scape distally brown; pedicel proximally brown and distally yellow-brown; first four-five proximal antennal flagellomeres dorsally lighter (light brown) than ventrally (dark brown), remaining flagellomeres dark brown on both sides; maxillary and labial palps yellow; clypeus, pronotum, propleuron, epicnemial ridge, lunules, BS, PFM, BM, and MPM with brown-red/reddish tints. Eyes silver and ocelli yellowish. Fore and middle legs dark yellow except brown-red/reddish coxae, and brown claws; hind legs dark yellow except black coxae, femora dorso-distally with an elongate brown spot, tibiae distally brown, and tarsomeres brown although basitarsus proximal half yellow. Petiole on T1 brown-red/reddish, contours darkened and sublateral areas yellow; T2 with median and adjacent areas brown-red/reddish, adjacent area with contours well-defined, both dark areas forming a rectangle-shaped area, and lateral ends yellow; T3 mostly brown-red/brown with corners proximally yellow; T4 and beyond brown-red/brown; distally each tergum with a narrow whitish transparent band. In lateral view, T1–3 yellow; T4 brown, but proximoventrally with a small yellow area; T5 and beyond brown. S1–3 yellow; S4 yellow, but medially brown; penultimate sternum and hypopygium brown.

#### Description.

**Head** (Fig. 222A–D). Head rounded with pubescence long and dense. Proximal three antennal flagellomeres longer than wide (0.23:0.09, 0.25:0.09, 0.27:0.09), distal antennal flagellomere longer than penultimate (0.15:0.07, 0.13:0.07), antenna longer than body (3.43, 3.13); antennal scrobes-frons shallow. Face flat or nearly so, finely punctate-lacunose, interspaces wavy and longitudinal median carina present. Frons smooth. Temple wide, punctate-lacunose and interspaces wavy. Inner margin of eyes diverging slightly at antennal sockets; in lateral view, eye anteriorly convex and posteriorly straight. POL shorter than OOL (0.11, 0.15). Malar suture faint. Median area between lateral ocelli without depression. Vertex laterally pointed or nearly so and dorsally wide.

**Mesosoma** (Fig. 222A, E, F, I). Mesosoma dorsoventrally convex. Mesoscutum distal half with a central dent, punctation distinct proximally, but absent/dispersed distally, interspaces wavy/lacunose. Scutellum triangular, apex sloped and fused with BS, but not in the same plane, scutellar punctation indistinct throughout, in profile scutellum slightly convex, but on same plane as mesoscutum, phragma of the scutellum widely visible; BS only very partially overlapping the MPM; ATS demilune with faint wavy rugae; dorsal ATS groove smooth. Transscutal articulation with small and heterogeneous foveae, area just behind transscutal articulation smooth, shiny and depressed centrally. Metanotum with BM convex; MPM circular without median longitudinal carina; AFM without setiferous lobes and not as well delineated as PFM; PFM thick, smooth and with lateral ends rounded; ATM proximally with a groove with some sculpturing and distally smooth. Propodeum with a shallow dent, proximal half weakly curved with fine sculpture and distal half relatively polished; distal edge of propodeum with a flange at each side and without stubs; propodeal spiracle without distal carina; nucha surrounded by very short radiating carinae. Pronotum with a distinct dorsal furrow, dorsally with a well-defined smooth band; central area of pronotum smooth, but both dorsal and ventral furrows with short parallel carinae. Propleuron with a mix of rugae and fine punctation, dorsally with a carina. Metasternum convex. Contour of mesopleuron convex; precoxal groove deep with faintly transverse lineate sculpture; epicnemial ridge convex, teardrop-shaped.

**Legs** (Fig. 222A). Ventral margin of fore telotarsus entire without seta, fore telotarsus almost same width throughout and longer than fourth tarsomere (0.12, 0.08). Hind coxa with punctation only on ventral surface, dorsal outer depression present. Inner spur of hind tibia longer than outer spur (0.31, 0.23), entire surface of hind tibia with dense strong spines clearly differentiated by color and length. Hind telotarsus as equal in length as fourth tarsomere (0.16, 0.15).

**Wings** (Fig. 222K). Fore wing with r vein curved; 2RS vein slightly concave; r and 2RS veins forming a weak, even curve at their junction and outer side of junction not forming a stub; 2M vein slightly curved/swollen; anal cell 1/3 proximally lacking microtrichiae; subbasal cell with a small smooth area; vein 2CUa absent and vein 2CUb spectral; vein 2 cu-a absent; vein 2-1A proximally tubular and distally spectral, although sometimes difficult to see; tubular vein 1 cu-a straight and complete, but junction with 1-1A vein spectral. Hind wing with vannal lobe narrow, subdistally and subproximally straightened, and setae present only proximally.

**Metasoma** (Fig. 222A, G, H, J). Metasoma laterally compressed. Petiole on T1 finely sculptured distal, but only laterally, virtually parallel-sided over most of length, but narrowing over distal 1/3 (length 0.38, maximum width 0.20, minimum width 0.08), and with scattered pubescence concentrated in the first distal third. Lateral grooves delimiting the median area on T2 clearly defined and reaching the distal edge of T2 (length median area 0.18, length T2 0.18), edges of median area polished and lateral grooves deep, median area broader than long (length 0.18, maximum width 0.21, minimum width 0.08); T2 with pubescence in distal half. T3 longer than T2 (0.27, 0.18) and with scattered pubescence throughout. Pubescence on hypopygium scattered.

**Cocoons.** Unknown.

#### Comments.

In some females, both the mesopleuron ventrally and the metasternum with brown-red/reddish tints, ATS demilune proximally is carinate (Fig. 222F). the propodeal spiracle distally is framed by a faint carina, and the limit between the mesopleuron and the metasternum is flattened.

#### Male.

Coloration similar to female except that the hind femora is almost completely brown, the trochanter and the trochantellus with brown tints; the external genitalia is large, but the apex is truncate diagonally; and the specimens with body slender and cylindrical.

#### Etymology.

Yali Zhang as an undergraduate student at UIUC, IL, USA, held a research opportunities grant through National Science Foundation, Research Experiences for Undergraduates. She assisted in sorting and identifying Microgastrinae genera in the Whitfield Lab and coauthoring papers on *Rhygoplitis* Mason and *Wilkinsonellus*. Also, she studied Curriculum and Instruction/Education at University of Illinois, Chicago, IL, USA.

#### Distribution.

Parasitized caterpillars were collected in Ecuador, Napo, Yanayacu Biological Station (Sendero de las Lágrimas and Sendero de los Sapos), during May and August 2009 at 2,004 m and 2,075 m in cloud forest.

#### Biology.

The lifestyle of this parasitoid species is gregarious.

#### Host.

*Zanola* sp. Walker (Apatelodidae) feeding on *Psammisiapauciflora* (Ericaceae) and undetermined species of Asteraceae. Caterpillars were collected in third and fourth instar.

### Glyptapanteles yanayacuensis

Taxon classificationAnimaliaHymenopteraBraconidae

Arias-Penna, sp. nov.

http://zoobank.org/29BA2826-29C7-4B82-ADE9-AAB3F66A9E9B

Fig. 223

#### Female.

Body length 2.83 mm, antenna length 3.53 mm, fore wing length 3.33 mm.

#### Type material.

**Holotype**: ECUADOR • 1♀; EC-41685, YY-A154; Napo, Yanayacu Biological Station, Stream trail, Plot 439; cloud forest; 2,114 m; -0.596944, -77.888333; 18.viii.2009; Lee Dyer leg.; caterpillar collected in third instar; cocoon formed on 28.viii.2009; adult parasitoid emerged on 24.ix.2009; (PUCE).

#### Diagnosis.

**Figure 223.**
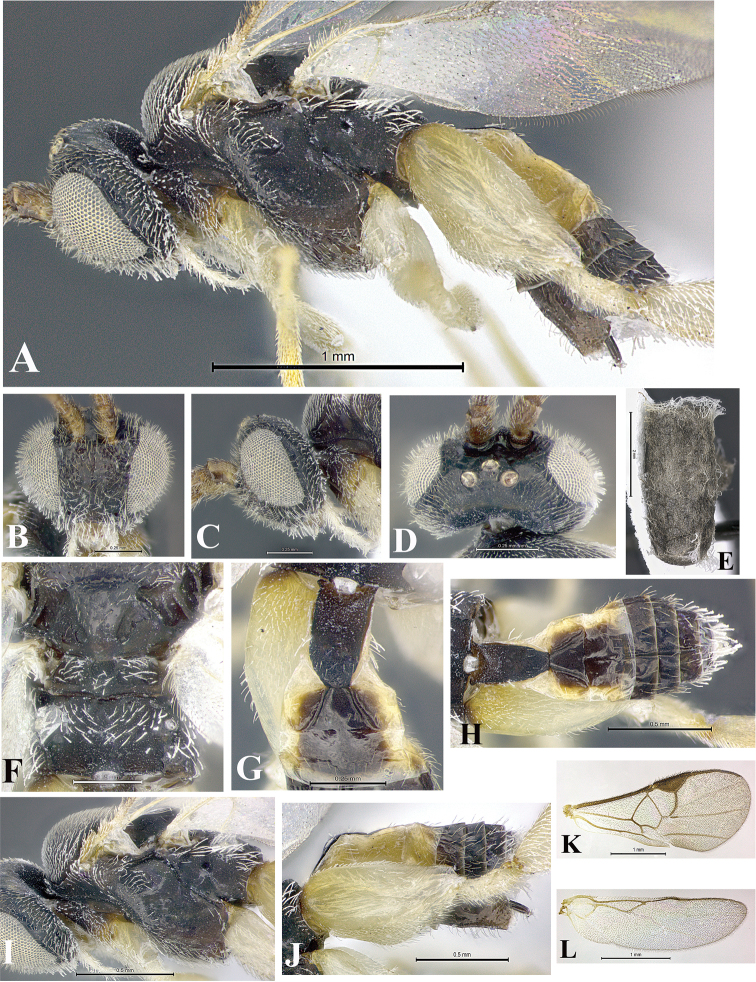
*Glyptapantelesyanayacuensis* sp. nov. female EC-41685 YY-A154 **A** Habitus **B, D** Head **B** Frontal view **D** Dorsal view **C** Head, pronotum, propleuron, lateral view **E** Cocoon **F** Scutellum, metanotum, propodeum, dorsal view **G**T1–3, dorsal view **H, J** Metasoma **H** Dorsal view **J** Lateral view **I** Mesosoma, lateral view **K, L** Wings **L** Fore **M** Hind.

Petiole on T1 completely smooth and polished, with faint, satin-like sheen (Fig. 223G, H), vertex in lateral view pointed or nearly so (Fig. 223C), scutellar punctation scattered throughout (Fig. 223F), phragma of the scutellum partially exposed (Fig. 223F), median area on T2 broader than long, lateral grooves delimiting the median area clearly defined and reaching the distal edge of T2, edges of median area polished and followed by a deep groove (Fig. 223G, H), anterior furrow of metanotum with a small lobe, without setae (Fig. 223F), axillary trough of scutellum almost smooth (Fig. 223F), propodeum without median longitudinal carina (Fig. 223F), anteroventral contour of mesopleuron convex (Fig. 223A, I), and fore wing with r vein curved, outer side of junction of r and 2RS veins forming a slight stub (Fig. 223K).

#### Coloration

(Fig. 223A–L). General body coloration black except labrum, mandibles, glossa, and tegulae yellow-brown; scape and pedicel yellow-brown, but laterally brown; first four-five proximal antennal flagellomeres dorsally lighter (light brown) than ventrally (dark brown), remaining flagellomeres dark brown on both sides; maxillary and labial palps pale yellow/ivorish; apex of propleuron yellow; clypeus, area just below antennal scrobes, pronotum, epicnemial ridge, ventral edge of mesopleuron, distal corners of mesoscutum, lunules, BS, PFM, and lateral ends of metapleuron with brown-red/reddish tints. Eyes silver and ocelli yellowish. Fore and middle legs light yellow except brown claws; hind legs light yellow except femora distally with tiny a dorsal brown spot, distal 1/3 of tibiae brown and proximally with a tiny brown area, and tarsomeres brown although basitarsus proximal half yellow. Petiole on T1 brown-red/reddish, contours darkened and sublateral areas yellow; T2 with median and adjacent areas brown-red/reddish, adjacent area with contours well-defined, both dark areas forming a rectangle-shaped area, and lateral ends yellow; T3 mostly brown, that dark area coinciding with the width of dark area formed by both median and adjacent areas on T2, and lateral ends narrow and yellow; T4 and beyond completely brown; distally each tergum with a narrow whitish transparent band. In lateral view, T1–3 yellow; T4 and beyond brown. S1–3 yellow; S4 yellow, but medially brown; penultimate sternum and hypopygium brown.

#### Description.

**Head** (Fig. 223A–D). Head rounded with pubescence long and dense. Proximal three antennal flagellomeres longer than wide (0.26:0.07, 0.28:0.07, 0.28:0.07), distal antennal flagellomere longer than penultimate (0.15:0.06, 0.12:0.06), antenna longer than body (3.53, 2.83); antennal scrobes-frons sloped and forming a shelf. Distal half of face dented laterally, finely punctate-lacunose, interspaces wavy and longitudinal median carina present. Frons smooth. Temple wide, punctate and interspaces clearly smooth. Inner margin of eyes diverging slightly at antennal sockets; in lateral view, eye anteriorly convex and posteriorly straight. POL shorter than OOL (0.10, 0.13). Malar suture present. Median area between lateral ocelli slightly depressed. Vertex laterally pointed or nearly so and dorsally wide.

**Mesosoma** (Fig. 223A, F, I). Mesosoma dorsoventrally convex. Mesoscutum poximally convex and distally flat, punctation distinct proximally, but absent/dispersed distally, interspaces wavy/lacunose. Scutellum triangular, apex sloped and fused with BS, but not in the same plane, scutellar punctation scattered throughout, in profile scutellum flat and on same plane as mesoscutum, phragma of the scutellum partially exposed; BS only very partially overlapping the MPM; ATS demilune almost smooth; dorsal ATS groove smooth. Transscutal articulation with small and homogeneous foveae, area just behind transscutal articulation nearly at the same level as mesoscutum (flat), smooth and shiny. Metanotum with BM wider than PFM (clearly differentiated); MPM circular without median longitudinal carina; AFM with a small lobe and not as well delineated as PFM; PFM thick, smooth and with lateral ends rounded; ATM proximally with a groove with some sculpturing and distally smooth. Propodeum without median longitudinal carina, proximal half weakly curved with fine sculpture and distal half relatively polished and with a shallow dent at each side of nucha; distal edge of propodeum with a flange at each side and without stubs; propodeal spiracle distally framed by faintly concave/wavy carina; nucha surrounded by very short radiating carinae. Pronotum with a faint dorsal furrow, dorsally with a well-defined smooth band; central area of pronotum and dorsal furrow smooth, but ventral furrow with short parallel carinae. Propleuron with fine punctations throughout and dorsally without a carina. Metasternum convex. Contour of mesopleuron convex; precoxal groove deep, smooth and shiny; epicnemial ridge convex, teardrop-shaped.

**Legs** (Fig. 223A). Ventral margin of fore telotarsus entire without seta, fore telotarsus almost same width throughout and longer than fourth tarsomere (0.13, 0.08). Hind coxa with punctation only on dorsal surface, dorsal outer depression present. Inner spur of hind tibia longer than outer spur (0.31, 0.23), entire surface of hind tibia with dense strong spines clearly differentiated by color and length. Hind telotarsus as equal in length as fourth tarsomere (0.15, 0.14).

**Wings** (Fig. 223K, L). Fore wing with r vein curved; 2RS vein straight; r and 2RS veins forming a weak, even curve at their junction and outer side of junction forming a slight stub; 2M vein slightly curved/swollen; distally fore wing [where spectral veins are] with microtrichiae more densely concentrated than the rest of the wing; anal cell 1/3 proximally lacking microtrichiae; subbasal cell with microtrichiae virtually throughout; vein 2CUa absent and vein 2CUb spectral; vein 2 cu-a absent; vein 2-1A proximally tubular and distally spectral, although sometimes difficult to see; tubular vein 1 cu-a curved, incomplete/broken and not reaching the edge of 1-1A vein. Hind wing with vannal lobe narrow, subdistally and subproximally straightened, and setae evenly scattered in the margin.

**Metasoma** (Fig. 223A, G, H, J). Metasoma laterally compressed. Petiole on T1 completely smooth and polished, with faint, satin-like sheen, parallel-sided in proximal half and then narrowing (length 0.42, maximum width 0.18, minimum width 0.08), and with scattered pubescence concentrated in the first distal third. Lateral grooves delimiting the median area on T2 clearly defined and reaching the distal edge of T2 (length median area 0.16, length T2 0.16), edges of median area polished and lateral grooves deep, median area broader than long (length 0.16, maximum width 0.20, minimum width 0.08); T2 with pubescence in distal half. T3 longer than T2 (0.24, 0.16) and with pubescence more notorious in distal half. Pubescence on hypopygium dense.

**Cocoons** (Fig. 223E). Gray or black oval cocoon with ordered silk fibers, but covered by a net.

#### Comments.

The coloration of the hind coxae is completely yellow (Fig. 223A, J), the body is stout, but elongated and covered by dense pubescence that may impede to see the sculpture on the body (Fig. 223A), and the limit between the mesopleuron and the metasternum is flattened.

#### Male.

Unknown.

#### Etymology.

Yanayacu Biological Station and Center for Creative Studies is an area of 100 hectares in the cloud forest of Ecuador, and the core site for much of our inventory work in the eastern Andes.

#### Distribution.

Parasitized caterpillar was collected in Ecuador, Napo, Yanayacu Biological Station (Stream trail), during August 2009 at 2,114 m in cloud forest.

#### Biology.

The lifestyle of this parasitoid species is solitary.

#### Host.

Undetermined species of Noctuidae feeding on Diplaziumcostalevar.robustum (Dryopteridaceae). Caterpillar was collected in third instar.

## Conclusions

The elevated number of new species described here is a reflection of the poorly known taxonomic state of *Glyptapanteles* (and other Microgastrinae genera) in the Neotropics, as well as its extreme diversity that remains to be explored. The information compiled here provides a working framework to investigate this diverse group and to improve our taxonomic knowledge, as the diversity within this enormous genus had barely been scratched. If the number of species formally described has increased so enormously from only two Neotropical countries, it is anticipated that the number will increase greatly when faunas of diverse countries such as Mexico, Panama, Colombia, Peru, Bolivia, and Brazil are incorporated. A Neotropical revision of this genus is a daunting task that will require replicating the rearing efforts of the ACG and Ecuador projects, with ecological interactions and the participation of many specialists in the not too distant future.
